# Whole genome sequencing analysis of SARS-CoV-2 from Malaysia: From alpha to Omicron

**DOI:** 10.3389/fmed.2022.1001022

**Published:** 2022-09-23

**Authors:** Choo Yee Yu, Sie Yeng Wong, Nancy Woan Charn Liew, Narcisse Joseph, Zunita Zakaria, Isa Nurulfiza, Hui Jen Soe, Rachna Kairon, Syafinaz Amin-Nordin, Hui Yee Chee

**Affiliations:** ^1^Laboratory of Vaccine and Biomolecules, Institute of Bioscience, Universiti Putra Malaysia, Serdang, Malaysia; ^2^Department of Medical Microbiology, Faculty of Medicine and Health Sciences, Universiti Putra Malaysia, Serdang, Malaysia; ^3^Institute of Bioscience, Universiti Putra Malaysia, Serdang, Malaysia; ^4^Department of Veterinary Pathology and Microbiology, Faculty of Veterinary Medicine, Universiti Putra Malaysia, Serdang, Malaysia; ^5^Department of Cell and Molecular Biology, Faculty of Biotechnology and Biomolecular Sciences, Universiti Putra Malaysia, Serdang, Malaysia; ^6^Synapse Sdn. Bhd., Petaling Jaya, Malaysia

**Keywords:** COVID-19, Malaysia variant, SARS-CoV-2 genome, VOC, clade replacement, epidemiology, genetic diversity\keywordbelowspace-30pt

## Abstract

Countries around the world are gearing for the transition of the coronavirus disease 2019 (COVID-19) from pandemic to endemic phase but the emergence of new severe acute respiratory syndrome coronavirus 2 (SARS-CoV-2) variants could lead to a prolonged pandemic. SARS-CoV-2 has continued to evolve as it optimizes its adaptation to the human host and the successive waves of COVID-19 have been linked to the explosion of particular variant of concern. As the genetic diversity and epidemiological landscape of SARS-CoV-2 differ from country to country, this study aims to provide insights into the variants that are circulating in Malaysia. Whole genome sequencing was performed for 204 SARS-CoV-2 from COVID-19 cases and an additional 18,667 SARS-CoV-2 genome sequences were retrieved from the GISAID EpiCoV database for clade, lineage and genetic variation analyses. Complete genome sequences with high coverage were then used for phylogeny investigation and the resulting phylogenetic tree was constructed from 8,716 sequences. We found that the different waves of COVID-19 in Malaysia were dominated by different clades with the L and O clade for first and second wave, respectively, whereas the progressive replacement by G, GH, and GK of the GRA clade were observed in the subsequence waves. Continuous monitoring of the genetic diversity of SARS-CoV-2 is important to identify the emergence and dominance of new variant in different locality so that the appropriate countermeasures can be taken to effectively contain the spread of SARS-CoV-2.

## Introduction

Almost 3 years after its emergence in China, the causative agent of coronavirus disease 2019 (COVID-19) that has claimed over 6 million lives and resulted in more than 500 million cases (1) is very unlikely to be eliminated; instead, severe acute respiratory syndrome coronavirus 2 (SARS-CoV-2) is expected to circulate endemically around the globe (2). Nevertheless, the anticipated shift to endemicity continues to be threaten by the emergence of new variants as evidenced by the waves of COVID-19 infections that have swept across various countries and geographical regions (3). Next generation sequencing (NGS) technology has been imperative in unraveling the novel virus genome and has continued to power SARS-CoV-2 sequencing projects worldwide ever since (4, 5). Presently, an enormous collection of more 12 million whole genome sequences (WGS) of SARS-CoV-2 is available in the publicly accessible Global Initiative on Sharing All Influenza Data (GISAID) EpiCoV database (6). This unprecedented rate of genome generation allowed the evolution and transmission of SARS-CoV-2 to be tracked as viruses circulating in different regions start to diversify and form distinct lineages through the accumulation of mutations during replication of the viral genome and subsequent spread among susceptible individuals (7–11).

To facilitate the tracking of SARS-CoV-2 genetic lineages at local and global levels, several nomenclature systems are currently in use including the World Health Organization (WHO) label (12), GISAID (13), NextStrain (14), and Phylogenetic Assignment of Named Global Outbreak LINeages (Pango lineages) (15). WHO uses letters of the Greek Alphabet as a naming scheme and only variants of concern (VOCs) and variants of interest (VOIs) are given a WHO label. A VOI is a variant with genetic changes that are predicted or known to affect transmission, disease severity, immune, diagnostic or therapeutic escape and causes increased proportion of cases over time or multiple COVID-19 clusters in multiple countries. A VOC not only meets the definition of a VOI but also displays evidence of an increase in transmissibility, an increase in disease severity and/or a decrease in effectiveness of available diagnostics, vaccines or therapeutics. Previously circulating VOCs included Alpha, Beta, Gamma, and Delta with Omicron being the current dominant VOC that is circulating globally and accounts for more than 98% of SARS-CoV-2 sequences that were shared on GISAID after February 2022 (16). Although no SARS-CoV-2 variants are designated as currently circulating VOI at the time of writing, previously circulating VOIs included Epsilon, Zeta, Eta, Theta, Iota, Kappa, Lambda, and Mu (16).

The GISAID nomenclature system, which is based on shared marker mutations, currently has eleven clades with L and S clades forming early in the pandemic before L is split into V and G. Splitting from base clade G resulted in clades GR, GH, GV, and GK. GR evolved into GRY and later also GRA that is presently the predominant clade. All unclassified sequences are grouped into the O clade. Variants in clades GH/GV/GK/GR/GRY/GRA share the common D614G signature mutation in the spike protein that increases infectivity as the mutation enhances binding to the angiotensin converting enzyme 2 (ACE2) receptor and increases viral entry into the host cell (17). In addition to D614G, the E484A mutation in the GRA clade is associated with substantial antibody neutralization resistance and contributes toward a stronger vaccine-breakthrough capability of the Omicron variant (18). The Pango nomenclature is a dynamic nomenclature that integrates genetic and geographical information to generate genetic lineages with epidemiological relevance. Pango lineage names consist of an alphabetical prefix and a numerical suffix such as the Alpha, Beta, Delta, and Omicron variants corresponded to Pango lineages B.1.1.7, B.1.351, B.1.617.2, and B.1.1.529 (including descendent lineages), respectively. More than 1,800 distinct lineages are included in the Pango nomenclature as of June 2022.^1^ In comparison to GISAID that focuses on broader phylogenetic clades, the finer scale of the Pango nomenclature can provide more detailed outbreak cluster information and assists in tracking the movement of emerging lineages between and within countries.

The potential epidemiological consequences of novel mutations provide grounds for the continuous genomic surveillance so that public health measures can be tailored at a regional or national level. Malaysia is a multiethnic country in the Southeast Asia with an estimated population of 32.7 million people in 2021 (19). On 25 January 2020, three Chinese nationals from Wuhan who had entered Malaysia through the state of Johor became the earliest confirmed COVID-19 cases in this country, setting off the first COVID-19 wave that ended on 15 February 2020 with only a total of 22 confirmed cases (20). Whereas the first wave was mostly cases that have a history of travel to China or a contact history with people who had been to China, the second wave was triggered by a COVID-19 outbreak (Tabligh cluster) that occurred during a 4-day religious gathering that was attended by 16,000 people (20). As 1,500 of the attendees were foreign nationals from dozens of countries including Brunei, Indonesia, Philippines, Vietnam, Cambodia, Myanmar, and Thailand, the gathering resulted in both national and international spread of the virus (21). The second COVID-19 wave that started in March 2020 lasted for 4 months before the third wave began in September 2020 following the formation of a major cluster in the state of Sabah (20). The Benteng LD cluster originated from two undocumented migrants from Philippines who spread the virus to other detainees in the Lahad Datu district police headquarters’ lockup due to close proximity (22). Over at the peninsular, formation of multiple COVID-19 clusters was detected in the northern region including the PUI Sivaganga (45 cases), Tawar (92 cases), Sungai (101 cases), and Tembok (3,169 cases) clusters. The third wave peaked in January 2021 before declining through the months of February and March 2021. This was followed by another two phases of surge and decline in COVID-19 cases (23). The fourth wave, spanning from April 2021 to January 2022, and the fifth wave, spanning from February to May 2022. The fifth wave saw the highest daily COVID-19 infection of 33,406 cases in Malaysia, a record that was set on which was 5 March 2022, and surpassed the previous record of 24,599 cases reported on 26 August, 2021 during the fourth wave (24). As of 31 May 2022, over 4.5 million of COVID-19 cases with 35, 676 deaths had been reported in Malaysia (24).

Virus evolution, accompanied by changes in population immunity, risk mitigation behaviors and government intervention policies in respond to COVID-19, will undoubtedly continue to influence transmission and severity of the disease. Given that a new highly infectious and/or virulent variant can nullify the current success achieved through efficacious vaccines and public health interventions, the continuous characterization of circulating SARS-CoV-2 strains will allow the early detection of mutations that could provide an early indication for an upcoming wave. The genetic epidemiology of SARS-CoV-2 in Malaysia has been investigated and described previously (21, 25–28); however, these studies were mainly confined to small dataset and covered only the first three epidemic waves. We hypothesized that new SARS-CoV-2 lineages were mainly responsible for the fourth and fifth COVID-19 waves instead of the lineages that have been detected during the first three waves. Therefore, the objectives of this study are to contribute to the genomic surveillance effort in Malaysia through SARS-CoV-2 genome sequencing and to present an updated, comprehensive overview of the COVID-19 epidemiology in this country by analyzing available genomic sequences originating from Malaysia in the GISAID EpiCoV database as of 31 May 2022. In addition, we also present the genetic diversity of Malaysia variants and provide insights into the lineages that have been driving the clade replacement events in Malaysia.

## Materials and methods

### Collection of samples

This study was approved by the Ethics Committee for Research Involving Human Subjects Universiti Putra Malaysia (JKEUPM 2020-289). A total of 204 real time RT-PCR-confirmed COVID-19 positive samples with cycle threshold values below 30 were received from seven states in Malaysia for whole viral genome sequencing. Most of the samples originated from Selangor (*n* = 101) followed by Kuala Lumpur (*n* = 57), Penang (*n* = 16), Johor (*n* = 13), Negeri Sembilan (*n* = 12), Pahang (*n* = 3), and Kelantan (*n* = 2). These samples were collected in May 2021 (*n* = 84), February 2022 (*n* = 55), March 2022 (*n* = 46), and April 2022 (*n* = 19).

### Epidemiology of coronavirus disease 2019 in Malaysia

The COVID-19 epidemiological information were retrieved from the official Malaysia government website for COVID-19 data called COVIDNOW^2^ and the dataset in CSV format was made available by the Ministry of Health (MOH) *via* its official GitHub account.^3^ The official data on the COVID-19 epidemic in MOH GitHub account were compiled from various sources that include the Crisis Preparedness and Response Centre (CPRC), CPRC hospital system, the National Public Health Laboratory and MySejahtera (a locally developed contract tracing mobile application). Information pertaining to the number cases, deaths, hospitalizations, intensive care unit (ICU) admission and ventilation usage as well as vaccination status as of 31 May 2022 were used for analysis in this study.

### Whole genome sequencing of SARS-CoV-2

The cDNA synthesis, SARS-CoV-2 sequence enrichment, library amplification, and indexing were performed using the Enhanced QIAseq DIRECT SARS-CoV-2 kit (Qiagen, Germany) in accordance to the manufacturer’s instruction. The libraries then were quantified using Qubit DNA High Sensitivity Kit (Thermo Fisher Scientific, USA) and quality of the libraries was assessed using a 2100 Bioanalyzer (Agilent Technologies, USA) with High Sensitivity DNA chips (Agilent Technologies, USA). The resulting libraries were pooled, normalized and quantified Qubit DNA High Sensitivity Kit before paired-end sequencing was performed on a MiSeq system (Illumina, USA) with a MiSeq 600 cycle V3 kit (Illumina, USA). Sequencing data was then processed using a protocol adapted from a previous study (29). Briefly, raw reads generated from the sequencing process were trimmed with Trimmomatic (30) to remove adaptor sequences and poor-quality reads. An average base quality of Q30 was used for trimming. The trimmed reads were then mapped against the human reference genome GRCh38/hg38 and SARS-CoV-2 Wuhan-Hu-1 reference genome sequence (GenBank accession no. NC_045512.2) using HISAT2 tool (31). Reads that mapped to GRCh38/hg38 were discarded and the remaining reads were mapped to the Wuhan-Hu-1 genome. The aligned consensus sequence was then called using samtools (32) and bcftools (33). Genome sequences obtained in this study, which ranged from 29,565 to 29,877 bp and covered 98.9–99.9% of the SARS-CoV-2 genome, have been deposited into the GISAID database (see Supplementary Table 1).

### Analysis of SARS-CoV-2 sequences from Malaysia

In addition to the viral genomes that were sequenced in this study, the genomic sequences of SARS-CoV-2 that have been deposited in the GISAID EpiCoV database were also retrieved. During the retrieval process, the dataset was restricted to Malaysia with a submission date that was no later than 31 May 2022. Sequences that lacked a collection date (*n* = 4) were removed. This resulted in a dataset of 18,871 SARS-CoV-2 genomic sequences (see Supplementary Table 2). The genomic sequences were further categorized into GISAID clades and Pango lineages for the detection of VOCs and VOIs. For phylogenetic analysis, additional filter options in the GISAID EpiCoV database were used to identify complete genomes with high coverage. GISAID considers genomes with length greater than 29,000 nucleotides as complete and assigns the high coverage label when there is less than 1% of undefined bases, less than 0.05% unique amino acid mutations and without insertion or deletion unless verified by the submitter. The 8,897 complete genomes with high coverage (see Supplementary Table 3) were then used as input in the Nextstrain v3.0.3 SARS-CoV-2 workflow (14) to construct a phylogenetic tree. Briefly, the default filtering criteria were used to filter the input sequences and metadata before the genomes were aligned to the reference sequence using nextalign. A phylogenetic tree was then constructed using IQTree (34) before TreeTime (35) was used to reroot, resolve polytomies, prune sequences, infer internal node dates, and label internal nodes of the resulting tree. The workflow also inferred nucleotide changes at internal nodes and translated them into amino acid changes as well as labeled clades based on pre-defined mutations. The outputs of the workflow were JSON files that serve as the inputs to the web-based Auspice visualization tool,^4^ allowing the final phylogenetic tree comprising 8,716 genomes, geographic transmission and genetic diversity to be viewed and explored.

## Results

### Epidemiology of coronavirus disease 2019 in Malaysia

**FIGURE 1:**
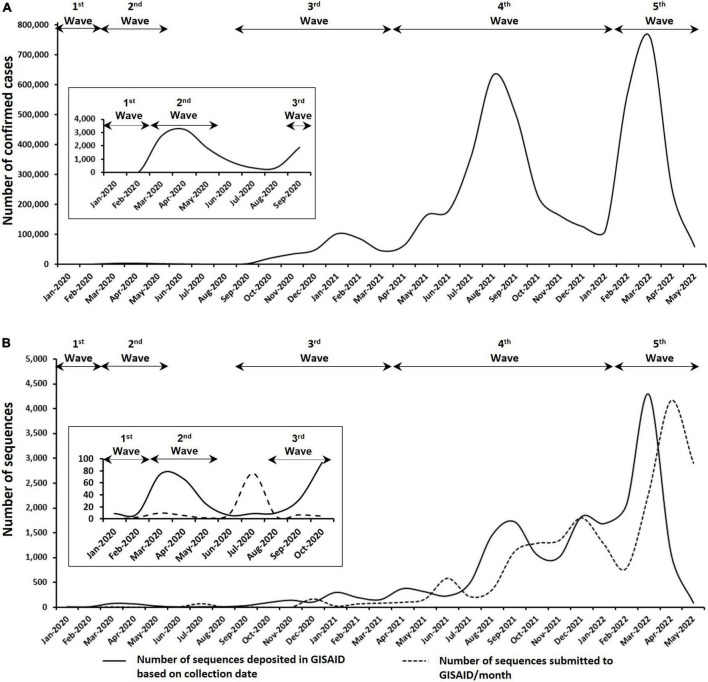
Epidemiology of COVID-19 in Malaysia as of 31 May 2022. **(A)** Number of confirmed COVID-19 cases in Malaysia. **(B)** Number of SARS-CoV-2 genomic sequences submitted to GISAID based on the collection date of samples and submission date. Insets are enlarged figure of the first few months of the COVID-19 pandemic in Malaysia.

As shown in Figure 1, Malaysia has experienced a total of five epidemic waves of COVID-19 since the identification of the first COVID-19 cases on 25 January 2020 (36). A total of 4,506,510 confirmed cases of COVID-19 has been reported in Malaysia as of 31 May 2022 and only 0.8% were imported cases. With an estimated population size of 32,655,400 (19), the total number of local cases translates into 13.8% of the Malaysian population that has succumbed to the SARS-CoV-2 infection although the percentage may be lower if reinfection were to be taken into account. The total number of cases escalated with each successive waves, starting from 25 cases to 8,614 cases, 336,160 cases, 2,525,258 cases, and 1,635,752 cases for first, second, third, fourth and fifth waves, respectively (Figure 1 upper panel). A total of 18,875 SARS-CoV-2 genomic sequences from Malaysia has been deposited in the GISAID database up to the end of May 2022, representing only 0.42% of the total number of cases reported in the same duration (Figure 1 lower panel). A breakdown of the number of available genomic sequences based on the date of sample collection revealed the under-representation of these sequences in Malaysia as the epidemic progressed. Based on the total number of cases reported for each wave, the corresponding number of genomes that were sequenced represented 72.0, 2.0, 0.3, 0.4, and 0.5% for first, second, third, fourth and fifth waves, respectively.

### Analysis of SARS-CoV-2 sequences from Malaysia

**FIGURE 2:**
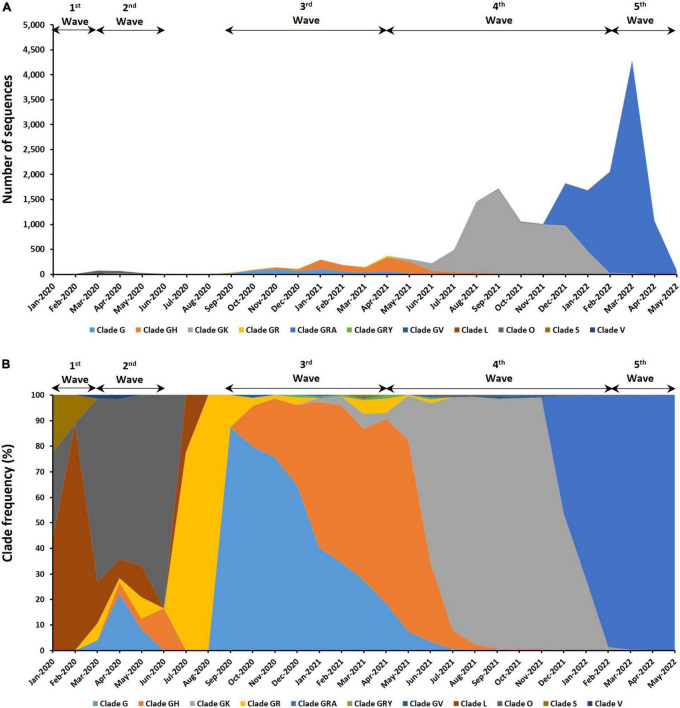
Distribution of clades among the 18,871 genomic sequences in Malaysia. **(A)** Number of sequences based on clades. **(B)** Distribution of clade frequency.

The distribution of 18,871 genomic sequences retrieved from the GISAID EpiCoV database in accordance with GISAID clades is shown in Figure 2. Eleven different clades were found in the Malaysian SARS-CoV-2 isolates with the most abundant clade being GRA (*n* = 9,510), followed by GK (*n* = 7,268), GH (*n* = 1,116), G (*n* = 649), O (*n* = 167), GR (*n* = 80), L (*n* = 34), GV (*n* = 30), GRY (*n* = 11), S (*n* = 4), and V (*n* = 2). Frequencies of these clades also differed at different time points over the course of the epidemic in Malaysia. The clade L (63.16%) and clade O (68.02%) dominated the first and second waves, respectively. Clades G (51.47%) and GH (46.69%) were predominant during the third wave whereas the fourth and fifth waves were dominated by clades GRA (99.33%) and GK (71.07%), respectively. The other clades (S, V, GR, GV, and GRY) were found at relatively low frequencies and some were only present for a short period of time.

**FIGURE 3:**
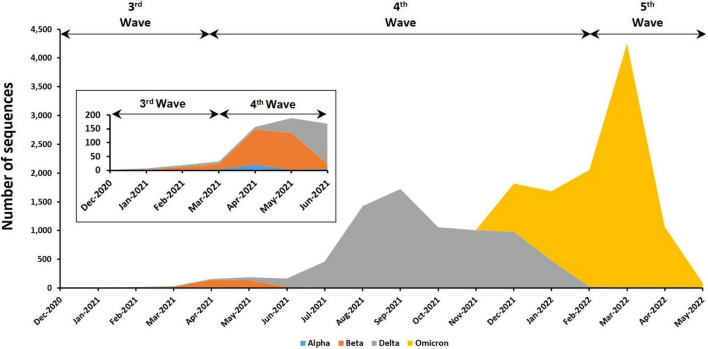
Distribution of VOCs among the 17,193 genomic sequences in Malaysia. Insets are enlarged figure of the first few months of the emergence of VOC in Malaysia.

Among the 18,871 genomic sequences, 91.1% fell into one of the four VOCs that have been detected in this country (Figure 3). The most abundant VOC is Omicron (55.06%), followed by Delta (42.83%), Beta (1.91%), and Alpha (0.2%). No Gamma variant has been detected in Malaysia. The earliest VOCs, which were Alpha and Beta, were reported in Malaysia during the third wave in December 2020. The Alpha and Beta variants were the dominant VOCs before they were replaced by Delta and Omicron variants in the fourth and fifth waves, respectively. Other than VOCs, previously circulating VOIs such as Kappa (*n* = 4), Eta (*n* = 4), and Theta variants (*n* = 10) were also found in Malaysia. However, these variants were present in relatively low frequencies and only for a short period of time at the beginning of fourth epidemic wave (around April to June 2021).

**FIGURE 4:**
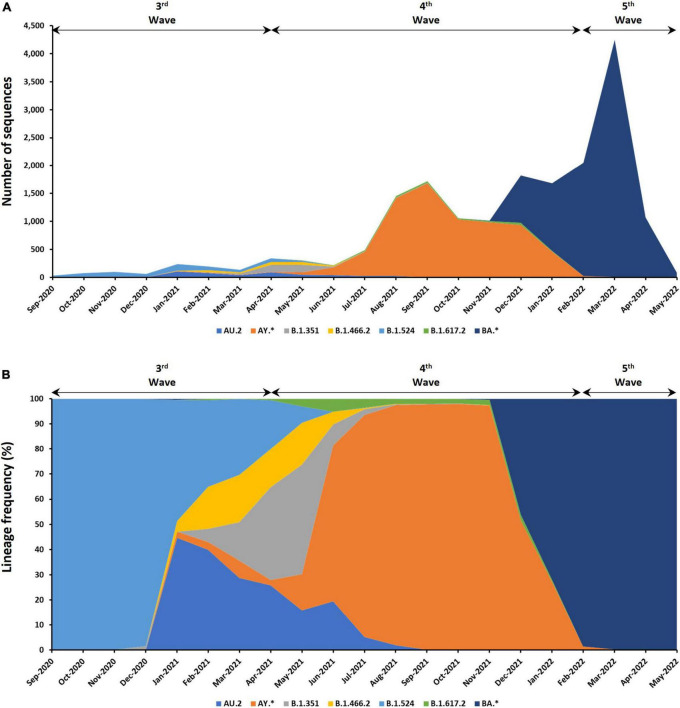
Distribution of lineages among the 9,468 genomic sequences in Malaysia. **(A)** Number of sequences based on lineages. **(B)** Distribution of lineage frequency. Only lineages with more than a total of 100 sequences are presented. *Indicates all descendent lineages.

The SARS-CoV-2 genomic sequences in Malaysia can be further assigned into 137 different Pango lineages (Figure 4 and see Supplementary Table 4). Prominent lineages detected in Malaysia include BA (50.17%), AY (37.96%), B.1.524 (3.05%), AU.2 (2.40%), B.1.351 (1.73%), and B.1.617.2 (1.06%) lineages while the rest were found at frequencies below 1%. Among the BA lineages of B.1.1.529 (Omicron), BA.2 (42.34%) dominated over BA.1.1 (27.26%), BA.2.3 (15.84%), BA.2.23 (4.45%), BA.2.10 (3.89%), and BA.1 (2.58%). For the AY lineage, AY.59 (34.96%) was predominant followed by AY.23 (34.18%), AY.79 (21.86%), and AY.76 (4.10%).

**FIGURE 5:**
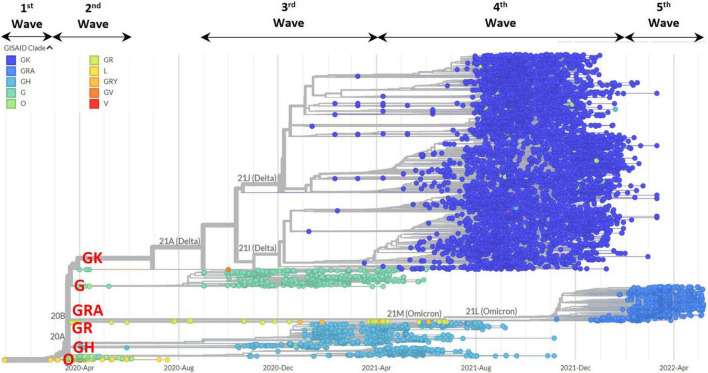
Phylogenetic tree analysis of 8,717 genomic sequences in Malaysia based on clade distributions and collection date. The clades are represented by different color codes. The major clades in Malaysia (GRA, GK, GH, G, O, and GR) are highlighted in red.

The phylogenetic tree in Figure 5 shows the 8,716 complete genomes that were sampled in Malaysia from February 2020 to May 2022. The tree branched out in accordance to the clades and clearly showed the replacement of clades over time in Malaysia over the five epidemic waves. Highest diversity was observed in the *N* gene at codon position 203 (R203M/I/V/K) followed by the *S* gene at codon positions 19 (T19R/G/I) and 681 (P681R/L/Y/H) with Shannon entropy values of 0.81, 0.802, and 0.716, respectively (see Supplementary Figure 1 and Supplementary Table 5).

## Discussion

In the span of 2 years since COVID-19 was declared a pandemic in March 2020, Malaysia has endured multiple waves of infections and deaths that were driven mostly by the emergence of new SARS-CoV-2 variants. Despite the availability of efficacious vaccines and the implementation of various public health interventions in this country, the number of confirmed cases during the peak period increased substantially with each successive wave. The most recent COVID-19 wave of infection in Malaysia reached a record high of 759,183 cases per month in March 2022, which is almost a 20% increment over that of the previous wave. New SARS-CoV-2 variants emerge naturally when mismatches are incorporated during the replication of viral genome but the fate of these variants is largely determined by the interplay of natural selection and chance events that differs across communities and countries (37).

In this study, a total of 204 SARS-CoV-2 genomes from Malaysia were sequenced in which 84 and 120 of the sequences were from the fourth and fifth waves, respectively. These sequences were analyzed together with the publicly available sequences in order to understand its evolutionary patterns and emerging variants in Malaysia. The increase in the number of SARS-CoV-2 genomes being sequenced and submitted to GISAID EpiCoV database from Malaysia can be seen starting from September 2021 due to the SARS-CoV-2 genomic surveillance effort by the Ministry of Science, Technology and Innovation (MOSTI), Malaysia (2). Overall, the percentage of cases being sequenced in Malaysia (0.42%) was comparable to neighboring countries such as Thailand (0.60%), Indonesia (0.47%), and Philippines (0.56%) (3). Active SARS-CoV-2 genomic surveillance and data sharing remained important for timely monitoring of emerging variants and containment effort (4, 5). The analysis of 18,871 genomic sequences of SARS-CoV-2 isolated in Malaysia from 24 January 2020 to 31 May 2022 revealed the dominance of B lineages (99.98%) with successive replacements by new lineages contributing to the resurgence of COVID-19 as opposed to the rekindling of persistent lineages. The present study also showed a shift in the dominant clade starting from L (January 2020) to O (March 2020), GR (July 2020), G (September 2020), GH (January 2021), GK (June 2021), and finally GRA from January 2022 onward.

The first known confirmed infections in Malaysia could be traced back to three samples collected on 24 January 2020 and the corresponding whole genome sequences were made available on 23 March 2020. In line with the detection of imported cases from China, the three isolates belonged to clade L (lineage B) that consists of early strains isolated from the Wuhan outbreak in December 2019. Imported cases accounted for 92% of the 25 cases detected in Malaysia during the first wave. The majority of the sequences belonged to clade L (lineage B, *n* = 11; B.12, *n* = 1) followed by clade S (lineage A, *n* = 3), and clade O (*n* = 3). The S clade (L84S in ORF8), which was initially prevalent in Americas, Asia, and Oceania during the early phase of the pandemic (38), failed to establish a prevalence in Malaysia. Only four S clade genomes from Malaysia were found in the GISAID database and the clade was lastly detected in March 2021 in which the isolate was assigned to the international lineage A.23.1.

A shift was then observed from L to the O clade during the second wave as 68% of the sequenced genomes belonged to the O clade followed by clades L (12%), G (12%), GR (5%), GH (3%), and V (1%). Notable clusters that were formed during the second wave included the Tabligh cluster that was linked to the religious gathering and the immigration depo cluster that resulted in 3,375 and 653 confirmed cases, respectively (39). Genome sequences of SARS-CoV-2 isolated from cases linked to the Tabligh and the immigration depo clusters were shown to be phylogenetically related and aligned with B.6 and its descendent lineages (25). The B.6 lineage is a variant that was predominantly seen in India and substantial local transmission resulted in two of its sub-lineages (B.6.1 and B.6.2) becoming predominant in Malaysia. While B.6.1 spreads to Brunei and India, the circulation of B.6.2 was limited to Malaysia. Overall, B.6 and three of its sub-lineages (B.6.1, B.6.2, and B.6.6) accounted for 66% of the genomes that were isolated during the second wave (*n* = 171) while European lineage B.1 and lineage B accounted for 15 and 9%, respectively. Although B.1 and B.1.1 were the first lineages to be detected in Malaysia with the D614G mutation that has been associated with higher viral load (40) and increased infectivity (41, 42), these G614 lineages did not appear to spread faster than the D614 lineages that were co-circulating during the second wave. Following the closure of Malaysia’s international borders on 18 March 2020 and the concurrent implementation of a restrictive, nationwide movement control order (MCO) (43), the number of monthly cases began to dwindle from 3,236 in April 2020 to 337 in July 2020.

The genome of SARS-CoV-2 encodes for structural (nucleocapsid, membrane, envelope, and spike), non-structural and accessory proteins (44). However, mutations in the spike proteins draw significant attention due to the potential capability of the mutation-carrying variants to escape *S* gene-targeting diagnostic assays (45) as well as therapeutic and vaccine-induced antibodies (46). The appearance of similar mutations in different lineages is suggestive of convergent evolution as the virus adapts to the changing immune profile of its human host (47). Whilst the D614G mutation was only found in 10% of the global sequences prior to 1 March 2020, the G614 form spreads rapidly throughout the world and became dominant with a prevalence of almost 100% by June 2020 (47, 48). This global phenomenon was also reflected in Malaysia as the dominant clade started to shift from O to GR in July 2020. Lineage B.1.1.354 (clade GR), which was first detected in India and subsequently spread to at least 25 countries, was introduced into Malaysia and detected in samples that were collected during the inter-wave period of July and August 2020. Genome sequences from multiple COVID-19 clusters in the northern region of Malaysia, including PUI Sivaganga, Sala, Sungai, Tembok, and Tawar clusters, belonged to the B.1.1.354 lineage (25). This lineage carries two characteristic mutations namely D614G and D138Y in the spike protein domain. The D138Y mutation is located in the center of the N-terminal domain (NTD) supersite and hence, contributes toward resistant to neutralization by NTD-targeting antibodies (49). The enforcement of targeted enhanced MCO in the affected localities appeared to have contained the spread of this lineage in Malaysia, as B.1.1.354 was not detected in subsequent genomic sequences from January 2021 onward.

The rise in the number of cases during the third wave coincided with the rise in the number of isolates assigned to the B.1.524 lineage (clade G). The first B.1.524 sequence in Asia could be traced back to a sample collected on 22 August, 2020 in Philippines and incidentally, the index cases of the Benteng LD cluster in Sabah that led the third wave were two undocumented migrants from Philippines. The cluster resulted in 1,146 cases and extensive traveling for political campaigning between Sabah and other states in the peninsular has been cited as a major cause for the surge in COVID-19 cases in multiple states (26). B.1.524 dominated the early part of the third wave and were linked to several other prominent clusters with more than 1,000 cases such as Teratai, Damanlela construction, and Perigi clusters (25). In addition to D614G, B.1.524 also carries the A701V mutation that sits adjacent to the furin cleavage site at the S1–S2 boundary. A701V, which is also found in Beta and Iota variants, was recently found to be an important fusion inducer that increases SARS-CoV-2 transmissibility by enhancing spike processing and fusogenicity (50).

The emergence of B.1.466.2 (clade GH) and more importantly, its sub-lineage AU.2, around the same time caused the wave to reach its peak in January 2021. Although the parental lineage B.1.466.2 was found predominantly in Indonesia, AU.2 became a domestic lineage and was mostly detected in Sarawak. B.1.466.2 appeared to have circulated in Sarawak before spreading to the peninsular based on the sample collection date of B.1.466.2 genomes in Malaysia. Globally, the transmission of B.1.466.2 was more extensive than AU.2 as the lineage has been detected in at least 27 countries whereas the latter has been found in five countries. Characteristic mutations in the S gene of B.1.466.2 include N439K, D614G, and P681R and sub-lineage AU.2 carries an additional G1251V. The receptor-binding motif (RBM) mutation N439K has been shown to increase ACE2 affinity and enhance immune evasion while maintaining virulence and fitness of the virus (51). Amino acid substitution at position 681 from P to R or H increases SARS-CoV-2 virulence by augmenting S1/S2 cleavage (52). P681R is also notably found in Delta variant whilst P681H is reported in Alpha and Omicron variants. Although G1251V lies outside of the receptor-binding domain (RBD), the mutation is reported to cause alteration in the structure of the S protein in a way that may impact infectivity of the virus (53). Onward transmission of B.1.524 and AU.2 in Malaysia could have been impeded by the enforcement of the second MCO that took place from January 2021 to March 2021 (54) as genomic surveillance indicated a downward trend in the number of B.1.524 and AU.2 isolates.

While the peak of the third wave was mostly driven by B.1.524 and AU.2, several VOCs and VOIs began to emerge at around the same time: Alpha (B.1.1.7), Beta (B.1.351), and Zeta (P.2) in December 2020 followed by Delta (several sub-lineages of B.1.617.2) and Eta (B.1.525) in January 2021. Two other VOIs were detected later namely Theta (P.3) and Kappa (B.1.617.1) in March and April of 2021, respectively. Compared to VOCs, the very few genome sequences of VOIs that were detected in Malaysia indicated that they only circulated briefly and did not contribute substantially to the surge of the COVID-19 wave. Following the emergence of the Alpha variant in the United Kingdom in September 2020, several studies have found that it is associated with significantly increased viral transmission (55, 56). The increased transmissibility is conferred by several RBD mutations including N501Y, 69/70 deletion and P681H. Although 69/70 deletion alone is not associated with increased transmissibility, its occurrence with N501Y that augments transmissibility by 70–80% is highly suggestive of epistasis (57, 58). The N501Y and 69/70 deletion were shown to confer fitness advantages for the replication of SARS-CoV-2 in the upper respiratory tract and lead to increased virus shedding (58). In October 2020, the Beta variant emerged independently in South Africa and led to a surge in new cases (59). Both Alpha and Beta variants have spread to more than 100 countries ever since. Although Alpha and Beta variants were first detected around the same time in Malaysia, Beta genomes had a higher representation as compared to Alpha genomes and the Beta variant also circulated longer than Alpha variant. Other than N501Y, Beta variant also carries immune evasion mutations E484K and K417N. Whereas the Alpha variant is susceptible to the neutralizing activity of most monoclonal antibodies as well as convalescent and vaccine (mRNA-1273 and BNT162b2) sera, the Beta variant has been found to be more resistant (47). The higher neutralization antibody that is required to protect against infection by variants with the E484K mutation often results in reinfection (60, 61).

Nevertheless, the number of sequenced genomes in Malaysia that belonged to Alpha and Beta variants pale in comparison to that of the Delta variant (clade GK) as the country experienced its first massive surge in COVID-19 cases from April to August 2021. The third MCO was enforced for the month of June 2021 due to the rapid increase in the number of cases and hospitalization but it failed to stem the tide of the fourth wave. The increased transmissibility of the Delta variant that propelled it into a dominant global VOC is possible driven by higher infectious viral load, longer duration of infectious viral shedding, a higher rate of reinfection due to immune evasion (47). Specifically, the RBD mutation L452R in Delta variant has been shown to reduce neutralizing activity by several monoclonal antibodies (46), convalescent plasma (62) and vaccine sera (63). When the peak number of daily new cases reached 24,599 on 26 August 2021, only 6.3% of the Malaysian population has been completely vaccinated, following the deployment of the National COVID-19 Immunization Program on 24 February 2021 (64). A total of 2,525,258 cases and 30,706 deaths were recorded from April 2021 to January 2022. Of the 491,743 COVID-19-related hospital admission, 50.6% were admitted to intensive care units and 27.6% required ventilator support. During the fourth wave, unvaccinated individuals accounted for the majority of the cases (54.2%) and death (63.7%). Genomic surveillance revealed that B.1.617.2 and 53 of its descendent lineages were detected in Malaysia. AY.23 was found to be the predominant lineage, followed by domestic lineages AY.59 and AY.79 with 2,448, 2,504, and 1,566 sequences being detected, respectively. These three sub-lineages established sustained transmission from January 2021 to March 2022, longer than any other sub-lineages of B.1.617.2 that were detected in Malaysia.

The global dominance of Delta variant eventually came to an end with the emergence of Omicron variant (B.1.1.529; clade GRA) in late 2021. Unlike other VOCs, the Omicron carries more than 30 mutations in S gene including E484A, K417N, T478K, N501Y, and P681H that have been associated with increased transmissibility, higher binding affinity to ACE2, and higher antibody escape (47). In Malaysia, the Omicron variant, specifically the sub-lineage BA.1.1, was first detected in November 2021 before a further 38 other sub-lineages of B.1.1.529 were detected in the following months. After its first detection in Malaysia, Omicron rapidly displaced Delta as the dominant variant and caused a sharp increase in the number of COVID-19 cases. Whereas the fourth wave took 8 months to reach the peak number of monthly cases (632,982 cases/month) following its first detection in this country, the fifth wave only took 5 months and resulted in a higher peak (759,183 cases/month) than that of the fourth wave. Despite the higher peak number of monthly cases, the drawn-out fourth wave resulted in a greater number of cases and deaths (2,525,258 cases; 30,706 deaths) as compared to that of the fifth wave (1,635,752 cases; 3,698 deaths). On the day that the country was hit with the highest daily COVID-19 infection of 33,406 cases, 78.9% of the Malaysian population has been completely vaccinated (see text footnote 2). Contrary to the outcomes of the fourth wave, significant reduction in the number of COVID-19-related hospital (*n* = 148,040) and ICU (*n* = 22,797) admissions as well as in the number of COVID-19 patients requiring ventilator support (*n* = 13, 404) were seen during the fifth wave. However, the majority of the cases (84.6%) and deaths (67.3%) were vaccinated individuals. Studies on vaccine effectiveness have shown reduced neutralizing activity of vaccine (ChAdOx1-S, mRNA-1273, and BNT162b2) sera against Omicron BA.1 and sera from convalescent individuals infected with the Alpha, Beta, or Delta VOC also have low neutralizing activities against BA.1 (47).

Genomic surveillance indicated that the parental lineage of the Omicron variant (B.1.1.529) was not the cause behind the fifth wave in Malaysia and B.1.1.529 has not been detected in this country to date. Instead, the fifth wave was fueled by its descendent lineages specifically BA.2 (*n* = 4,008), which is predominant in United Kingdom, and two other sub-lineages that were predominant in United States of America namely BA.1.1 (*n* = 2,581) and BA.2.3 (*n* = 1,500). Although BA.1.1.was the predominant lineage at the beginning of the fifth wave, it was rapidly replaced by BA.2. The high representation of BA.2 genomes during the fifth wave may be attributed to its high transmissibility and immune-evasive properties as BA.2 has been found to be associated with an increased susceptibility of infection for unvaccinated, fully vaccinated and booster-vaccinated individuals as compared to BA.1 (65). Other prominent sub-lineages that contributed to the Omicron surge include BA.2.23 (*n* = 421), BA.1 (*n* = 244), and BA.2.32 (*n* = 81). BA.2, BA.2.3, BA.2.10, BA.2.23, and BA.2.32 were continued to be detected as of May 2022, providing evidence that at least 5 sub-lineages of Omicron are still actively circulating at the time of writing.

In conclusion, the epidemiological landscape of COVID-19 in Malaysia is characterized by major clade replacement events that are linked to the emergence of new SARS-CoV-2 variants. Whilst distribution of the clades showed greater variation between continents in the first half of the pandemic era (38), a similar trend began to appear globally following the emergence of clade GK. Clade GK, which the Delta variant belonged to, was able to establish dominance over all other clades before it was gradually replaced by Omicron variant of the clade GRA. New sub-lineages of the Omicron variant have continued to emerge as the variant circulates around the world at the time of writing. The epidemiological landscape of COVID-19 in Malaysia as described in this study is based on available sequenced genomes in the GISAID EpiCoV database as of 31 May 2022. We acknowledge that the total genomic sequences that were available and analyzed is a limitation in this study as the sequences represented less than 0.5% of the total COVID-19 cases recorded in Malaysia. Nevertheless, the current findings could still provide valuable insights into the diversity and evolution of SARS-CoV-2 variants in Malaysia as well as the driving factors behind the multiple waves of COVID-19 in this country. Given that the GISAID EpiCoV database continues to expand on a daily basis, future studies undertaking a similar analysis will be needed and may uncover a different COVID-19 epidemiological landscape in Malaysia. In depth characterization of SARS-CoV-2 mutations in Malaysia variants could also be considered in future studies in order to elucidate Malaysia-specific mutation pattern and/or signature. As new variants of SARS-CoV-2 with increased transmissibility, resistance to neutralization and/or disease severity can lead to a significant loss of human lives, overwhelm healthcare infrastructure during a surge and cause profound societal and economic disruption, continuous genomic surveillance at a nation-scale is warranted for the early anticipation and initiation of public health measures to contain further outbreaks.

## Data availability statement

The datasets presented in this study can be found in online repositories. The names of the repository/repositories and accession number(s) can be found in the article/Supplementary material.

## Ethics statement

The studies involving human participants were reviewed and approved by the Universiti Putra Malaysia. Written informed consent from the participants’ legal guardian/next of kin was not required to participate in this study in accordance with the national legislation and the institutional requirements.

## Author contributions

HC, SA-N, CY, NJ, ZZ, and IN: conceptualization. CY, SW, and NL: methodology. CY, SW, and HC: formal analysis. CY, SW, NL, NJ, ZZ, IN, HS, RK, SA-N, and HC: draft preparation and review. All authors contributed to the article and approved the final submitted version.

## Footnotes

1
https://cov-lineages.org/lineage_list.html

2
https://covidnow.moh.gov.my/

3
https://github.com/MoH-Malaysia/covid19-public

4
https://auspice.us/

## References

[1] World Health Organization. *WHO Coronavirus (COVID-19) Dashboard.* Geneva: WHO (2022).

[2] Antia R Halloran ME. Transition to endemicity: understanding COVID-19. *Immunity.* (2021) 54:2172–6. 10.1016/j.immuni.2021.09.019 34626549 PMC8461290

[3] Al-Tawfiq JA Hoang V-T Le Bui N Chu D-T Memish ZA. The Emergence of the Omicron (B.1.1.529) SARS-CoV-2 variant: what is the impact on the continued pandemic? *J Epidemiol Glob Health.* (2022) 12:143–6. 10.1007/s44197-022-00032-w 35089588 PMC8795715

[4] Chen Z Azman AS Chen X Zou J Tian Y Sun R Global landscape of SARS-CoV-2 genomic surveillance and data sharing. *Nat Genet.* (2022) 54:499–507. 10.1038/s41588-022-01033-y 35347305 PMC9005350

[5] Fahmi M Kitagawa H Yasui G Kubota Y Ito M. The functional classification of ORF8 in SARS-CoV-2 replication. immune evasion, and viral pathogenesis inferred through phylogenetic profiling. *Evol Bioinformatics.* (2021) 17:11769343211003079. 10.1177/11769343211003079 33795929 PMC7970180

[6] Khare S Gurry C Freitas L Schultz MB Bach G Diallo A GISAID’s role in pandemic response. *China CDC Wkly.* (2021) 3:1049–51. 10.46234/ccdcw2021.255 34934514 PMC8668406

[7] Motayo BO Oluwasemowo OO Olusola BA Akinduti PA Arege OT Obafemi YD Evolution and genetic diversity of SARS-CoV-2 in Africa using whole genome sequences. *Int J Infect Dis.* (2021) 103:282–7. 10.1016/j.ijid.2020.11.190 33259879 PMC7698667

[8] Rockett RJ Arnott A Lam C Sadsad R Timms V Gray K-A Revealing COVID-19 transmission in Australia by SARS-CoV-2 genome sequencing and agent-based modeling. *Nat Med.* (2020) 26:1398–404. 10.1038/s41591-020-1000-7 32647358

[9] Kumar S Bansal K. Cross-sectional genomic perspective of epidemic waves of SARS-CoV-2: a pan India study. *Virus Res.* (2022) 308:198642. 10.1016/j.virusres.2021.198642 34822953 PMC8606321

[10] Bansal K Kumar S. Mutational cascade of SARS-CoV-2 leading to evolution and emergence of omicron variant. *Virus Res.* (2022) 315:198765. 10.1016/j.virusres.2022.198765 35367284 PMC8968180

[11] Ahammad I Hossain MU Rahman A Chowdhury ZM Bhattacharjee A Das KC Wave-wise comparative genomic study for revealing the complete scenario and dynamic nature of COVID-19 pandemic in Bangladesh. *PLoS One.* (2021) 16:e0258019. 10.1371/journal.pone.0258019 34587212 PMC8480844

[12] World Health Organization. *WHO Announces Simple, Easy-To-Say Labels for SARS-CoV-2 Variants of Interest and Concern.* (2021). Available online at: https://www.who.int/news/item/31-05-2021-who-announces-simple-easy-to-say-labels-for-sars-cov-2-variants-of-interest-and-concern (accessed May 15, 2022).

[13] Elbe S Buckland-Merrett G. Data, disease and diplomacy: GISAID’s innovative contribution to global health. *Glob Chall.* (2017) 1:33–46. 10.1002/gch2.1018 31565258 PMC6607375

[14] Hadfield J Megill C Bell SM Huddleston J Potter B Callender C Nextstrain: real-time tracking of pathogen evolution. *Bioinformatics.* (2018) 34:4121–3. 10.1093/bioinformatics/bty407 29790939 PMC6247931

[15] Rambaut A Holmes EC O’Toole A Hill V McCrone JT Ruis C A dynamic nomenclature proposal for SARS-CoV-2 lineages to assist genomic epidemiology. *Nat Microbiol.* (2020) 5:1403–7. 10.1038/s41564-020-0770-5 32669681 PMC7610519

[16] World Health Organization. *Tracking SARS-CoV-2 Variants.* (2022). Available online at: https://www.who.int/activities/tracking-SARS-CoV-2-variants (accessed May 15, 2022). 37184162

[17] Groves DC Rowland-Jones SL Angyal A. The D614G mutations in the SARS-CoV-2 spike protein: implications for viral infectivity, disease severity and vaccine design. *Biochem Biophys Res Commun.* (2021) 538:104–7. 10.1016/j.bbrc.2020.10.109 33199022 PMC7643658

[18] Chen J Wang R Gilby NB Wei GW. Omicron Variant (B.1.1.529): infectivity. Vaccine breakthrough, and antibody resistance. *J Chem Inf Model.* (2022) 62:412–22. 10.1021/acs.jcim.1c01451 34989238 PMC8751645

[19] Department of Statistics Malaysia. *Current Population Estimates, Malaysia, 2021.* Malaysia: Department of Statistics Malaysia (2021).

[20] Amir Y. *Timeline: How the COVID-19 Pandemic has Unfolded in Malaysia since January 2020.* (2021). Available online at: https://www.channelnewsasia.com/asia/timeline-how-covid-19-pandemic-has-unfolded-malaysia-january-2020-2082081 (accessed May 15, 2022).

[21] Che Mat NF Edinur HA Abdul Razab MKA Safuan S. A single mass gathering resulted in massive transmission of COVID-19 infections in Malaysia with further international spread. *J Travel Med.* (2020) 27:taaa059. 10.1093/jtm/taaa059 32307549 PMC7188142

[22] Kaos JJ. *Covid-19: 50 More Cases Linked to Benteng Lahad Datu Cluster, all Prisoners, says Health DG.* (2020). Available online at: https://www.nst.com.my/news/nation/2020/03/575560/how-sri-petaling-tabligh-became-southeast-asias-covid-19-hotspot (accessed May 15, 2022).

[23] Krishnan DB. *Nation Doing Better Against Omicron Wave Compared to Delta Wave, says KJ.* (2022). Available online at: https://www.nst.com.my/news/nation/2022/02/769748/nation-doing-better-against-omicron-wave-compared-delta-wave-says-kj (accessed May 15, 2022).

[24] Ministry Of Health Malaysia. *COVIDNOW in Malaysia.* (2022). Available online at: https://covidnow.moh.gov.my/ (accessed May 15, 2022).

[25] Suppiah J Kamel KA Mohd-Zawawi Z Afizan MA Yahya H Md-Hanif SA Phylogenomic analysis of SARS-CoV-2 from third wave clusters in Malaysia reveals dominant local lineage B.1.524 and persistent spike mutation A701V. *Trop Biomed.* (2021) 38:289–93. 10.47665/tb.38.3.070 34362872

[26] Tan KK Tan JY Wong JE Teoh BT Tiong V Abd-Jamil J Emergence of B.1.524(G) SARS-CoV-2 in Malaysia during the third COVID-19 epidemic wave. *Sci Rep.* (2021) 11:22105. 10.1038/s41598-021-01223-4 34764315 PMC8586159

[27] Mohamad Noordin N Tan JL Chong CK Chem YK Tajudin N Abu Bakar RS Genomic diversity of SARS-CoV-2 in Malaysia. *PeerJ.* (2021) 9:e12449. 10.7717/peerj.12449 34760404 PMC8571957

[28] Zainulabid UA Mat Yassim AS Hussain M Aslam A Soffian SN Mohd Ibrahim MS Whole genome sequence analysis showing unique SARS-CoV-2 lineages of B.1.524 and AU.2 in Malaysia. *PLoS One.* (2022) 17:e0263678. 10.1371/journal.pone.0263678 35213571 PMC8880882

[29] Bhoyar RC Jain A Sehgal P Divakar MK Sharma D Imran M High throughput detection and genetic epidemiology of SARS-CoV-2 using COVIDSeq next-generation sequencing. *PLoS One.* (2021) 16:e0247115. 10.1371/journal.pone.0247115 33596239 PMC7888613

[30] Bolger AM Lohse M Usadel B. Trimmomatic: a flexible trimmer for Illumina sequence data. *Bioinformatics.* (2014) 30:2114–20. 10.1093/bioinformatics/btu170 24695404 PMC4103590

[31] Kim D Paggi JM Park C Bennett C Salzberg SL. Graph-based genome alignment and genotyping with HISAT2 and HISAT-genotype. *Nat Biotechnol.* (2019) 37:907–15. 10.1038/s41587-019-0201-4 31375807 PMC7605509

[32] Li H Handsaker B Wysoker A Fennell T Ruan J Homer N The sequence alignment/map format and SAMtools. *Bioinformatics.* (2009) 25:2078–9. 10.1093/bioinformatics/btp352 19505943 PMC2723002

[33] Li H. A statistical framework for SNP calling, mutation discovery, association mapping and population genetical parameter estimation from sequencing data. *Bioinformatics.* (2011) 27:2987–93. 10.1093/bioinformatics/btr509 21903627 PMC3198575

[34] Nguyen LT Schmidt HA von Haeseler A Minh BQ. IQ-TREE: a fast and effective stochastic algorithm for estimating maximum-likelihood phylogenies. *Mol Biol Evol.* (2015) 32:268–74. 10.1093/molbev/msu300 25371430 PMC4271533

[35] Sagulenko P Puller V Neher RA. TreeTime: maximum-likelihood phylodynamic analysis. *Virus Evol.* (2018) 4:vex042. 10.1093/ve/vex042 29340210 PMC5758920

[36] Hashim JH Adman MA Hashim Z Mohd Radi MF Kwan SC. COVID-19 Epidemic in Malaysia: epidemic progression, challenges, and response. *Front Public Health.* (2021) 9:560592. 10.3389/fpubh.2021.560592 34026696 PMC8138565

[37] Lauring AS Hodcroft EB. Genetic variants of SARS-CoV-2—What do they mean? *JAMA.* (2021) 325:529–31. 10.1001/jama.2020.27124 33404586

[38] Mercatelli D Giorgi FM. Geographic and genomic distribution of SARS-CoV-2 mutations. *Front Microbiol.* (2020) 11:1800. 10.3389/fmicb.2020.01800 32793182 PMC7387429

[39] Danial M Arulappen AL Ch’ng ASH Looi I. Mitigation of COVID-19 clusters in Malaysia. *J Glob Health.* (2020) 10:0203105. 10.7189/jogh.10.0203105 33403108 PMC7750020

[40] Volz E Hill V McCrone JT Price A Jorgensen D O’Toole A Evaluating the effects of SARS-CoV-2 spike mutation D614G on transmissibility and pathogenicity. *Cell.* (2021) 184:64–75.e11. 10.1101/2020.07.31.20166082 33275900 PMC7674007

[41] Hou YJ Chiba S Halfmann P Ehre C Kuroda M Dinnon KH III SARS-CoV-2 D614G variant exhibits efficient replication ex vivo and transmission in vivo. *Science.* (2020) 370:1464–8. 10.1126/science.abe8499 33184236 PMC7775736

[42] Plante JA Liu Y Liu J Xia H Johnson BA Lokugamage KG Spike mutation D614G alters SARS-CoV-2 fitness. *Nature.* (2021) 592:116–21. 10.1038/s41586-020-2895-3 33106671 PMC8158177

[43] Tang KHD. Movement control as an effective measure against Covid-19 spread in Malaysia: an overview. *Z Gesundh Wiss.* (2022) 30:583–6. 10.1007/s10389-020-01316-w 32837842 PMC7293423

[44] Fahmi M Kharisma VD Ansori ANM Ito M. Retrieval and investigation of data on SARS-CoV-2 and COVID-19 using bioinformatics approach. In: Rezaei N editor. *Coronavirus Disease - COVID-19.* Cham: Springer International Publishing (2021). p. 839–57. 10.1007/978-3-030-63761-3_47 33973215

[45] Yu CY Chan KG Yean CY Ang GY. Nucleic acid-based diagnostic tests for the detection SARS-CoV-2: an update. *Diagnostics.* (2021) 11:53. 10.3390/diagnostics11010053 33401392 PMC7823986

[46] Liu Z VanBlargan LA Bloyet L-M Rothlauf PW Chen RE Stumpf S Identification of SARS-CoV-2 spike mutations that attenuate monoclonal and serum antibody neutralization. *Cell Host Microbe.* (2021) 29:477–88.e4. 10.1016/j.chom.2021.01.014 33535027 PMC7839837

[47] Chen KK Huang DT Huang LM. SARS-CoV-2 variants - evolution, spike protein, and vaccines. *Biomed J.* (2022). 10.1016/j.bj.2022.04.006 [Epub ahead of print]. 35526825 PMC9072773

[48] Korber B Fischer WM Gnanakaran S Yoon H Theiler J Abfalterer W Tracking changes in SARS-CoV-2 spike: evidence that D614G increases infectivity of the COVID-19 virus. *Cell.* (2020) 182:812–27. 10.1016/j.cell.2020.06.043 32697968 PMC7332439

[49] Wang P Casner RG Nair MS Wang M Yu J Cerutti G Increased resistance of SARS-CoV-2 variant P.1 to antibody neutralization. *Cell Host Microbe.* (2021) 29:747–51. 10.1016/j.chom.2021.04.007 33887205 PMC8053237

[50] Escalera A Gonzalez-Reiche AS Aslam S Mena I Laporte M Pearl RL Mutations in SARS-CoV-2 variants of concern link to increased spike cleavage and virus transmission. *Cell Host Microbe.* (2022) 30:373–87.e7. 10.1016/j.chom.2022.01.006 35150638 PMC8776496

[51] Thomson EC Rosen LE Shepherd JG Spreafico R da Silva Filipe A Wojcechowskyj JA Circulating SARS-CoV-2 spike N439K variants maintain fitness while evading antibody-mediated immunity. *Cell.* (2021) 184:1171–87. 33621484 10.1016/j.cell.2021.01.037 PMC7843029

[52] Johnson BA Xie X Bailey AL Kalveram B Lokugamage KG Muruato A Loss of furin cleavage site attenuates SARS-CoV-2 pathogenesis. *Nature.* (2021) 591:293–9. 10.1038/s41586-021-03237-4 33494095 PMC8175039

[53] Rehman S Mahmood T Aziz E Batool R. Identification of novel mutations in SARS-COV-2 isolates from Turkey. *Arch Virol.* (2020) 165:2937–44. 10.1007/s00705-020-04830-0 33025199 PMC7538173

[54] Zamri ASSM Singh S Ghazali SM Herng LC Dass SC Aris T Effectiveness of the movement control measures during the third wave of COVID-19 in Malaysia. *Epidemiol Health.* (2021) 43:e2021073–80. 10.4178/epih.e2021073 34607399 PMC8891114

[55] Volz E Mishra S Chand M Barrett JC Johnson R Geidelberg L Assessing transmissibility of SARS-CoV-2 lineage B.1.1.7 in England. *Nature.* (2021) 593:266–9. 10.1038/s41586-021-03470-x 33767447

[56] Lyngse FP Molbak K Skov RL Christiansen LE Mortensen LH Albertsen M Increased transmissibility of SARS-CoV-2 lineage B.1.1.7 by age and viral load. *Nat Commun.* (2021) 12:7251. 10.1101/2021.04.16.21255459 PMC8669007 34903718

[57] Leung K Shum MH Leung GM Lam TT Wu JT. Early transmissibility assessment of the N501Y mutant strains of SARS-CoV-2 in the United Kingdom, October to November 2020. *Euro Surveill.* (2021) 26:2002106. 10.2807/1560-7917.ES.2020.26.1.2002106 33413740 PMC7791602

[58] Liu Y Liu J Plante KS Plante JA Xie X Zhang X The N501Y spike substitution enhances SARS-CoV-2 infection and transmission. *Nature.* (2022) 602:294–9. 10.1038/s41586-021-04245-0 34818667 PMC8900207

[59] Tegally H Wilkinson E Giovanetti M Iranzadeh A Fonseca V Giandhari J Detection of a SARS-CoV-2 variant of concern in South Africa. *Nature.* (2021) 592:438–43. 10.1038/s41586-021-03402-9 33690265

[60] Wise J. Covid-19: the E484K mutation and the risks it poses. *BMJ.* (2021) 372:n359. 10.1136/bmj.n359 33547053

[61] Jangra S Ye C Rathnasinghe R Stadlbauer D Alshammary H Amoako AA SARS-CoV-2 spike E484K mutation reduces antibody neutralisation. *Lancet Microbe.* (2021) 2:e283–4. 10.1016/S2666-5247(21)00068-9 33846703 PMC8026167

[62] Li Q Wu J Nie J Zhang L Hao H Liu S The impact of mutations in SARS-CoV-2 spike on viral infectivity and antigenicity. *Cell.* (2020) 182:1284–94.e9. 10.1016/j.cell.2020.07.012 32730807 PMC7366990

[63] Liu C Ginn HM Dejnirattisai W Supasa P Wang B Tuekprakhon A Reduced neutralization of SARS-CoV-2 B.1.617 by vaccine and convalescent serum. *Cell.* (2021) 184:4220–36. 10.1016/j.cell.2021.06.020 34242578 PMC8218332

[64] Suah JL Tok PSK Ong SM Husin M Tng BH Sivasampu S PICK-ing Malaysia’s epidemic apart: effectiveness of a diverse COVID-19 vaccine portfolio. *Vaccines.* (2021) 9:1381. 10.3390/vaccines9121381 34960126 PMC8706086

[65] Lyngse FP Kirkeby CT Denwood M Christiansen LE Mølbak K Møller CH Transmission of SARS-CoV-2 Omicron VOC subvariants BA.1 and BA.2: evidence from Danish Households. *medRxiv* [Preprint]. (2022). 10.1101/2022.01.28.22270044

