# Supplementary material for: Whole genome sequencing analysis of SARS-CoV-2 from Malaysia: From alpha to Omicron
Source: Front Med (Lausanne). 2022 Sep 23;9:1001022. doi: 10.3389/fmed.2022.1001022 (PMC9537942; doi:10.3389/fmed.2022.1001022)
Supplement: Supplementary file 3 [file Table_3.pdf]

Supplementary Table 3

Lineages in Malaysia	Total number of sequences
A	3
A.23.1	1
AU.2	452
AU.3	2
AY.100	9
AY.102	1
AY.103	3
AY.106	2
AY.109	2
AY.111	7
AY.112	5
AY.112.2	3
AY.114	16
AY.119	2
AY.120	10
AY.121	2
AY.122	16
AY.124	1
AY.124.1.1	1
AY.125	1
AY.126	1
AY.127	14
AY.127.2	1
AY.129	3
AY.131	1
AY.132	1
AY.19	18
AY.23	2448
AY.23.1	16
AY.24	98
AY.26	2
AY.29.1	1
AY.32	1
AY.33	1
AY.36	4
AY.39	4
AY.4	7
AY.4.2	3
AY.4.2.1	1
AY.4.2.2	1
AY.4.5	1
AY.4.7	1
AY.43	10
AY.44	1
AY.45	3

AY.46	1
AY.5	33
AY.54	3
AY.59	2504
AY.61	1
AY.75	20
AY.76	294
AY.79	1566
AY.84	3
AY.85	10
AY.86	3
AY.95	2
B	28
B.1	53
B.1.1	7
B.1.260	1
B.1.36	2
B.1.36.8	1
B.1.36.16	83
B.1.36.19	2
B.1.178	1
B.1.250	1
B.1.351	327
B.1.351.3	2
B.1.351.5	1
B.1.459	1
B.1.466	1
B.1.466.2	186
B.1.468	2
B.1.470	41
B.1.524	575
B.1.525	4
B.1.617.1	4
B.1.617.2	200
B.1.1.1	1
B.1.1.7	34
B.1.1.63	6
B.1.1.312	1
B.1.1.354	20
B.1.1.398	1
B.1.177.77	1
B.1.428.3	3
B.12	2
B.28	1
B.3	1
B.56	1
B.6	53
B.6.1	34

B.6.2	16
B.6.6	11
BA.1	244
BA.1.1	2581
BA.1.1.1	9
BA.1.1.13	1
BA.1.1.14	4
BA.1.1.15	5
BA.1.1.18	1
BA.1.1.7	6
BA.1.10	1
BA.1.13	2
BA.1.13.1	14
BA.1.14	2
BA.1.15	22
BA.1.15.1	6
BA.1.16	2
BA.1.17	15
BA.1.17.2	51
BA.1.18	39
BA.1.20	1
BA.1.21	1
BA.2	4008
BA.2.1	3
BA.2.10	368
BA.2.10.1	23
BA.2.12	5
BA.2.17	1
BA.2.18	2
BA.2.23	421
BA.2.24	1
BA.2.27	5
BA.2.3	1500
BA.2.3.2	2
BA.2.3.3	2
BA.2.31	3
BA.2.32	81
BA.2.4	5
BA.2.5	10
BA.2.9	15
BA.3	6
C.36	3
P.2	1
P.3	10
