# Supplementary material for: Whole genome sequencing analysis of SARS-CoV-2 from Malaysia: From alpha to Omicron
Source: Front Med (Lausanne). 2022 Sep 23;9:1001022. doi: 10.3389/fmed.2022.1001022 (PMC9537942; doi:10.3389/fmed.2022.1001022)
Supplement: Supplementary file 5 [file Image_1.pdf]

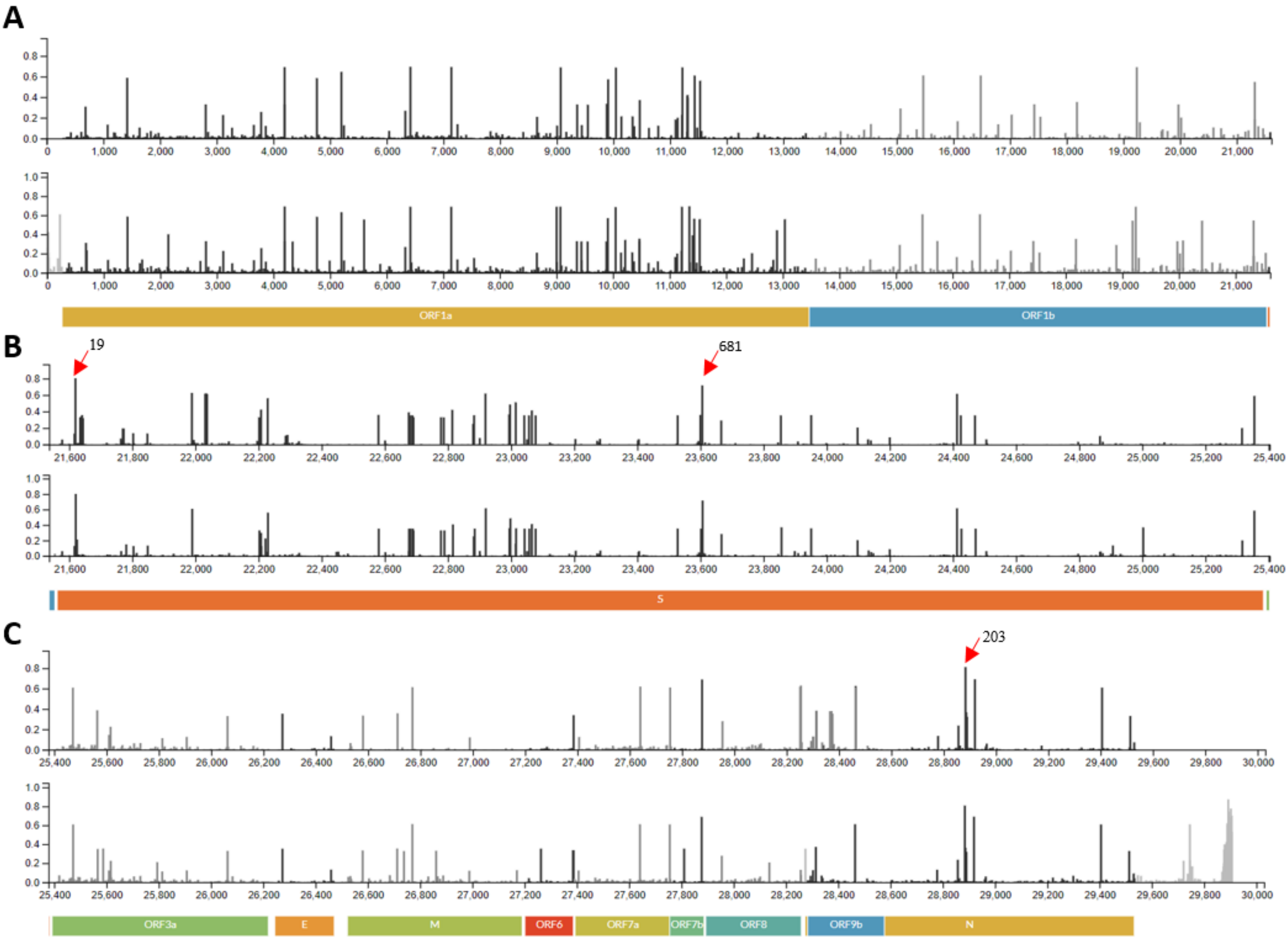

Supplementary Figure 1. Genomic diversity chart for (A) *ORF1a* and *ORF1b* genes, (B) *S* gene and (C) *ORF3a*, *E*, *M*, *ORF6*, *ORF7a*, *ORF7b*, *ORF8*, *ORF9b* and *N* genes. Horizontal axis represents the nucleotide position across genome and vertical axis represents entropy or variability at the genomic location. Upper and lower panels show the variations at the amino acid and nucleotide levels, respectively, for the 8,716 genomic sequences in Malaysia.
