# Supplementary material for: Whole genome sequencing analysis of SARS-CoV-2 from Malaysia: From alpha to Omicron
Source: Front Med (Lausanne). 2022 Sep 23;9:1001022. doi: 10.3389/fmed.2022.1001022 (PMC9537942; doi:10.3389/fmed.2022.1001022)
Supplement: Supplementary file 6 [file Data_Sheet_1.PDF]

We gratefully acknowledge the following Authors from the Originating laboratories responsible for obtaining the specimens, as well as the Submitting laboratories where the genome data were generated and shared via GISAID, on which this research is based.

All Submitters of data may be contacted directly via www.gisaid.org

Authors are sorted alphabetically.

Accession ID	Originating Laboratory	Submitting Laboratory	Authors
EPI_ISL_12322072, EPI_ISL_12322074, EPI_ISL_12322076	AMPANG HOSPITAL	Institute for Medical Research, Infectious Disease Research Centre, National Institutes of Health, Ministry of Health Malaysia	Ahmad FA; Ahmad Fazilah NA; Anasir MI; Azizan MA; Kamel K; Mohamad Sukri MZ; Norhisham SN; Ramly N; Robert F; Rosli NR; Suppiah J; Thayan R
EPI_ISL_12899665	BANDAR BOTANIK HEALTH CLINIC	Institute for Medical Research, Infectious Disease Research Centre, National Institutes of Health, Ministry of Health Malaysia	Ahmad FA; Ahmad Fazilah NA; Anasir MI; Azizan MA; Kamel K; Mohamad Sukri MZ; Norhisham SN; Ramly N; Robert F; Rosli NR; Suppiah J; Thayan R
EPI_ISL_12983187	BP Healthcare Group	Institute for Medical Research, Infectious Disease Research Centre, National Institutes of Health, Ministry of Health Malaysia	Anasir MI; G.Adypatti NM; Jamaluddin MS; Kalyanasundram J; Kamel K; MatRahim N; Nawi MH; Suib FA; Suppiah J; Thayan R
EPI_ISL_12099572, EPI_ISL_12099573, EPI_ISL_12099574, EPI_ISL_12099575, EPI_ISL_12099576, EPI_ISL_12099577, EPI_ISL_12099578, EPI_ISL_12099579, EPI_ISL_12099580, EPI_ISL_12099581, EPI_ISL_12099582, EPI_ISL_12099583, EPI_ISL_12099584, EPI_ISL_12099585, EPI_ISL_12099586, EPI_ISL_12099587, EPI_ISL_12099588, EPI_ISL_12099589, EPI_ISL_12099590, EPI_ISL_12099591, EPI_ISL_12099592, EPI_ISL_12099593, EPI_ISL_12099594, EPI_ISL_12099596, EPI_ISL_12099597, EPI_ISL_12099598, EPI_ISL_12099720, EPI_ISL_12099721, EPI_ISL_12099749			
see above	Bintulu Hospital PCR Lab, Bintulu	Institute of Health and Community Medicine	Chan Chia Jui; Chien Su Lin; Chua Hock Hin; David Perera; Ooi Mong How; Tonnii Sia Loong Loong
EPI_ISL_12099630, EPI_ISL_12099631, EPI_ISL_12099632, EPI_ISL_12099633, EPI_ISL_12099726, EPI_ISL_12509995, EPI_ISL_12509996, EPI_ISL_12509997, EPI_ISL_12509998, EPI_ISL_12509999, EPI_ISL_12510283, EPI_ISL_12510284, EPI_ISL_12510286, EPI_ISL_12510287, EPI_ISL_12510288, EPI_ISL_12510289, EPI_ISL_12510291			
see above	Bintulu Medical Centre (Bintulu)	Institute of Health and Community Medicine	Chan Chia Jui; Chua Hock Hin; David Perera; Ooi Mong How; Tonnii Sia Loong Loong; Wong Jyn Shan
EPI_ISL_12510156, EPI_ISL_12510157, EPI_ISL_12510184, EPI_ISL_12510185, EPI_ISL_12510186, EPI_ISL_12510187, EPI_ISL_12510188, EPI_ISL_12510189, EPI_ISL_12510190, EPI_ISL_12510191, EPI_ISL_12510192, EPI_ISL_12510193, EPI_ISL_12510194, EPI_ISL_12510195, EPI_ISL_12510196, EPI_ISL_12510197, EPI_ISL_12510198, EPI_ISL_12510412, EPI_ISL_12510413, EPI_ISL_12510414, EPI_ISL_12510415, EPI_ISL_12510416, EPI_ISL_12510417, EPI_ISL_12510418, EPI_ISL_12510419, EPI_ISL_12510420, EPI_ISL_12510421, EPI_ISL_12510422, EPI_ISL_12510423, EPI_ISL_12510424, EPI_ISL_12510425, EPI_ISL_12510426, EPI_ISL_12510427, EPI_ISL_12510428, EPI_ISL_12510429, EPI_ISL_12510430, EPI_ISL_12510431, EPI_ISL_12510432, EPI_ISL_12510433, EPI_ISL_12510434, EPI_ISL_12510435			
see above	Borneo Medical Centre (Kuching)	Institute of Health and Community Medicine	Chan Chia Jui; David Perera; Ooi Mong How; Wong Jyn Shan
EPI_ISL_12899696	CHERAS DISTRICT HEALTH OFFICE	Institute for Medical Research, Infectious Disease Research Centre, National Institutes of Health, Ministry of Health Malaysia	Ahmad FA; Ahmad Fazilah NA; Anasir MI; Azizan MA; Kamel K; Mohamad Sukri MZ; Norhisham SN; Ramly N; Robert F; Rosli NR; Suppiah J; Thayan R
EPI_ISL_12704568	Department of Medical Microbiology and Parasitology, Hospital Universiti Sains Malaysia	Molecular Research Laboratory	Abdul Haris bin Muhammad; Ahmad Sukari Bin Halim; Alexander Chong Shu Chien; Asraihan Che Abdul Malik; Azian Harun; Chan Yean Yean; Chua Wei Chuan; Farahana binti Mohamed; Kirnpal Kaur Banga Singh; Lau Nyok Sean; Lee Lih Huey; Lim Shu Yong; Maizun binti Mohd Zain; Mera Edora Binti Abdul Manap; Mohd Iman hafiz Bin Ibrahim; Mohd Nadzri Abu Yazid; Mohd Zulkifli Salleh; Muhamad Khairul Amirin Bin Zulkifli; Muhammad Azamuddeen bin Mohammad Nasir; Muhammad Fazli bin Khalid; Muhammad Nashrul Farhan Samsudin; Muhammad Zarul Hanifah Bin Md Zoqratt; Nik Zuraina binti Nik Mohd Noor; Noor Hafizan binti Mat Salleh; Nor Amizara Binti Azami; Nor Suhana Binti Mohd Satar; Nur Syuhada Binti Abdul Rahim; Nur-Leem Binti Murshid; Nurfadhlina Musa; Qasim Ayub; Rosline Hassan; Sadequr Rahman; Siti Nur Aisyah Binti Mohamad Sham; Syahida binti Omar; Wan Mohd Zahiruddin Wan Mohammad; Wardah Yusof; Zaini bin Hussin; Zakuan Zainy Deris
EPI_ISL_12721612	Department of Medical Microbiology, Hospital Pengajar Universiti Putra Malaysia	Department of Medical Microbiology, Hospital Pengajar Universiti Putra Malaysia	Afiqah Adzmi; Amiza Azmi; Azmiza Syawani Jasni; Hui Yee Chee; Leslie Thian Lung Than; Muadz Mohtar; Muhammad Mohd Isa; Narcisse MS Joseph; Niazlin Mohd Taib; Noor Hazirah Noor Azhari; Norlaila; Nur Raihana Ithnin; Nurul Huda Mohamed Rashidi; Nurul Nadiah Ismail; Rosni Ibrahim; Rukman Awang Hamat; Sallehhudin; Siti Norbaya Masri; Siti Zulaikha Zakariah; Syafinaz Amin Nordin; Tengku Zetty Maztura Tengku Jamaluddin; Zamberi Sekawi
EPI_ISL_12534906, EPI_ISL_12534907, EPI_ISL_12534908, EPI_ISL_12534909	FORENSIC HOSPITAL SULTANAH BAHIYAH	Tropical Infectious Diseases Research & Education Centre (TIDREC), Universiti Malaya	AsmaAnati CheMatSeri; Che-Norainon Yaacob; Jia-Yi Tan; Jo-Ern Wong; Kim-Kee Tan; Mulya-Mustika-Sari Zulkifli; Noor Syahida Azizan; Nur-Hidayana Mahfodz; Sazaly AbuBakar; Siti-Sarah Nor'e; Wei-Wei Aw
EPI_ISL_12510357, EPI_ISL_12510358, EPI_ISL_12510359, EPI_ISL_12510360, EPI_ISL_12510361, EPI_ISL_12510362	Gribbles Diagnostic Lab @Timberland Medical Centre, Kuching	Institute of Health and Community Medicine	Chan Chia Jui; Chua Hock Hin; David Perera; Liew Chin Chin J; Ooi Mong How; Tonnii Sia Loong Loong; Wong Jyn Shan
EPI_ISL_12534899	HOSPITAL KULIM, Asia / Malaysia / Kedah	Tropical Infectious Diseases Research & Education Centre (TIDREC), Universiti Malaya	AsmaAnati CheMatSeri; Che-Norainon Yaacob; Jia-Yi Tan; Jo-Ern Wong; Kim-Kee Tan; Mulya-Mustika-Sari Zulkifli; Noor Syahida Azizan; Nur-Hidayana Mahfodz; Sazaly AbuBakar; Siti-Sarah Nor'e; Wei-Wei Aw
EPI_ISL_12628131	HOSPITAL MELAKA	UKM Medical Molecular Biology Institute (UMBI)	Khairun Nur Abd Ghafar; Mira Farzana Mohamad Mokhtar; Muhiddin Ishak; Nor Azila Muhammad Azami; Nur Alyaa Afifah Md Shahri; Nurul Syakima Ab Mutalib; Rahman Jamal; Ryia Illani Mohd Yunos; Siti Nur Hasanah Mohd Yusuf; Zahirrah Begam Mohamed Rasheed
EPI_ISL_12771257, EPI_ISL_12771300, EPI_ISL_12771318	HOSPITAL PULAU PINANG	iPROMISE, UiTM	Ariza Adnan; Fadzilah Mohd Nor; Lim Wai Feng; Mohd Asif Mohd Sukri; Mohd Nur Fakhruzzaman Noorizhab; Mohd Zaki Salleh; Sazzli Shahlan Kassim; Siti Farah Alwani Mohd Nawi; Siti Hamimah Sheikh Abdul Kadir; Teh Lay Kek; Wang Seok Mui
EPI_ISL_12534816, EPI_ISL_12534817, EPI_ISL_12534818, EPI_ISL_12534819, EPI_ISL_12534820, EPI_ISL_12534821	HOSPITAL RAJA PERMAISURI BAINUN	Tropical Infectious Diseases Research & Education Centre (TIDREC), Universiti Malaya	AsmaAnati CheMatSeri; Che-Norainon Yaacob; Jia-Yi Tan; Jo-Ern Wong; Kim-Kee Tan; Mulya-Mustika-Sari Zulkifli; Noor Syahida Azizan; Nur-Hidayana Mahfodz; Sazaly AbuBakar; Siti-Sarah Nor'e; Wei-Wei Aw
EPI_ISL_12534759, EPI_ISL_12534760, EPI_ISL_12534761, EPI_ISL_12534762, EPI_ISL_12534763	HOSPITAL SULTANAH NUR ZAHIRAH	Tropical Infectious Diseases Research & Education Centre (TIDREC), Universiti Malaya	AsmaAnati CheMatSeri; Che-Norainon Yaacob; Jia-Yi Tan; Jo-Ern Wong; Kim-Kee Tan; Mulya-Mustika-Sari Zulkifli; Noor Syahida Azizan; Nur-Hidayana Mahfodz; Sazaly AbuBakar; Siti-Sarah Nor'e; Wei-Wei Aw
EPI_ISL_12213457, EPI_ISL_12213466, EPI_ISL_12213467, EPI_ISL_12213468	HSAJB	UKM Medical Molecular Biology Institute (UMBI)	Khairun Nur Abd Ghafar; Mira Farzana Mohamad Mokhtar; Muhiddin Ishak; Nor Azila Muhammad Azami; Nur Alyaa Afifah Md Shahri; Nurul Syakima Ab Mutalib; Rahman Jamal; Ryia Illani Mohd Yunos; Siti Nur Hasanah Mohd Yusuf; Zahirrah Begam Mohamed Rasheed
EPI_ISL_12213446, EPI_ISL_12213447, EPI_ISL_12213448, EPI_ISL_12213449	HTJ	UKM Medical Molecular Biology Institute (UMBI)	Khairun Nur Abd Ghafar; Mira Farzana Mohamad Mokhtar; Muhiddin Ishak; Nor Azila Muhammad Azami; Nur Alyaa Afifah Md Shahri; Nurul Syakima Ab Mutalib; Rahman Jamal; Ryia Illani Mohd Yunos; Siti Nur Hasanah Mohd Yusuf; Zahirrah Begam Mohamed Rasheed
EPI_ISL_12055351	HTJS	Institut Biologi Molekul Perubatan UKM (UMBI)	Khairun Nur Abd Ghafar; Mira Farzana Mohamad Mokhtar; Muhiddin Ishak; Nor Azila Muhammad Azami; Nur Alyaa Afifah Md Shahri; Nurul Syakima Ab Mutalib; Rahman Jamal; Ryia Illani Mohd Yunos; Siti Nur Hasanah Mohd Yusuf; Zahirrah Begam Mohamed Rasheed
EPI_ISL_12704561, EPI_ISL_12704562	Hospital Raja Perempuan Zainab II, Kota Bharu	Molecular Research Laboratory	Abdul Haris bin Muhammad; Ahmad Sukari Bin Halim; Alexander Chong Shu Chien; Asraihan Che Abdul Malik; Azian Harun; Chan Yean Yean; Chua Wei Chuan; Farahana binti Mohamed; Kirnpal Kaur Banga Singh; Lau Nyok Sean; Lee Lih Huey; Lim Shu Yong; Maizun binti Mohd Zain; Mera Edora Binti Abdul Manap; Mohd Iman hafiz Bin Ibrahim; Mohd Nadzri Abu Yazid; Mohd Zulkifli Salleh; Muhamad Khairul Amirin Bin Zulkifli; Muhammad Azamuddeen bin Mohammad Nasir; Muhammad Fazli bin Khalid; Muhammad Nashrul Farhan Samsudin; Muhammad Zarul Hanifah Bin Md Zoqratt; Nik Zuraina binti Nik Mohd Noor; Noor Hafizan binti Mat Salleh; Nor Amizara Binti Azami; Nor Suhana Binti Mohd Satar; Nur Syuhada Binti Abdul Rahim; Nur-Leem Binti Murshid; Nurfadhlina Musa; Qasim Ayub; Rosline Hassan; Sadequr Rahman; Siti Nur Aisyah Binti Mohamad Sham; Syahida binti Omar; Wan Mohd Zahiruddin Wan Mohammad; Wardah Yusof; Zaini bin Hussin; Zakuan Zainy Deris
EPI_ISL_12771337	Hospital Seberang Jaya	iPROMISE, UiTM	Ariza Adnan; Fadzilah Mohd Nor; Lim Wai Feng; Mohd Asif Mohd Sukri; Mohd Nur Fakhruzzaman Noorizhab; Mohd Zaki Salleh; Sazzli Shahlan Kassim; Siti Farah Alwani Mohd Nawi; Siti Hamimah Sheikh Abdul Kadir; Teh Lay Kek; Wang Seok Mui
EPI_ISL_12213465	JABATAN PATOLOGI HSAJB	UKM Medical Molecular Biology Institute (UMBI)	Khairun Nur Abd Ghafar; Mira Farzana Mohamad Mokhtar; Muhiddin Ishak; Nor Azila Muhammad Azami; Nur Alyaa Afifah Md Shahri; Nurul Syakima Ab Mutalib; Rahman Jamal; Ryia Illani Mohd Yunos; Siti Nur Hasanah Mohd Yusuf; Zahirrah Begam Mohamed Rasheed
EPI_ISL_12534910	Jabatan Forensik Hospital Sultanah Bahiyah	Tropical Infectious Diseases Research & Education Centre (TIDREC), Universiti Malaya	AsmaAnati CheMatSeri; Che-Norainon Yaacob; Jia-Yi Tan; Jo-Ern Wong; Kim-Kee Tan; Mulya-Mustika-Sari Zulkifli; Noor Syahida Azizan; Nur-Hidayana Mahfodz; Sazaly AbuBakar; Siti-Sarah Nor'e; Wei-Wei Aw
EPI_ISL_12084320, EPI_ISL_12899676	KEPONG DISTRICT HEALTH OFFICE	Institute for Medical Research, Infectious Disease Research Centre,	Ahmad FA; Ahmad Fazilah NA; Anasir MI; Azizan MA; G. Adypatti NM; Jamaluddin MS; Kamel K; Mohamad Sukri MZ; Norhisham SN; Ramly N; Robert F; Rosli NR; Suppiah J; Thayan R

		National Institutes of Health, Ministry of Health Malaysia	
EPI_ISL_12935248	Kota Bharu Public Health Laboratory	Molecular Research Laboratory	Abdul Haris bin Muhammad; Ahmad Sukari Bin Halim; Alexander Chong Shu Chien; Asraihan Che Abdul Malik; Azian Harun; Chan Yean Yean; Chua Wei Chuan; Farahana binti Mohamed; Kirnpal Kaur Banga Singh; Lau Nyok Sean; Lee Lih Huey; Lim Shu Yong; Maizun binti Mohd Zain; Mera Edora Binti Abdul Manap; Mohd Iman hafiz Bin Ibrahim; Mohd Nadzri Abu Yazid; Mohd Zulkifli Salleh; Muhamad Khairul Amirin Bin Zulkifli; Muhammad Azamuddeen bin Mohammad Nasir; Muhammad Fazli bin Khalid; Muhammad Nashrul Farhan Samsudin; Muhammad Zarul Hanifah Bin Md Zoqratt; Nik Zuraina binti Nik Mohd Noor; Noor Hafizan binti Mat Salleh; Nor Amizara Binti Azami; Nor Suhana Binti Mohd Satar; Nur Syuhada Binti Abdul Rahim; Nur-Leem Binti Murshid; Nurfadhlina Musa; Qasim Ayub; Rosline Hassan; Sadequr Rahman; Siti Nur Aisyah Binti Mohamad Sham; Syahida binti Omar; Wan Mohd Zahiruddin Wan Mohammad; Wardah Yusof; Zaini bin Hussin; Zakuan Zainy Deris
EPI_ISL_12101243, EPI_ISL_12101244, EPI_ISL_12101245, EPI_ISL_12101246, EPI_ISL_12101247, EPI_ISL_12101248, EPI_ISL_12101249, EPI_ISL_12101250, EPI_ISL_12101251, EPI_ISL_12101252, EPI_ISL_12101253, EPI_ISL_12101254, EPI_ISL_12101255, EPI_ISL_12101256, EPI_ISL_12101257, EPI_ISL_12101258, EPI_ISL_12148386, EPI_ISL_12148387, EPI_ISL_12148388, EPI_ISL_12148389, EPI_ISL_12148390, EPI_ISL_12148391, EPI_ISL_12148392, EPI_ISL_12148393, EPI_ISL_12148394, EPI_ISL_12148395, EPI_ISL_12148396, EPI_ISL_12148397, EPI_ISL_12148398, EPI_ISL_12148399, EPI_ISL_12148400, EPI_ISL_12148401, EPI_ISL_12148402, EPI_ISL_12148403, EPI_ISL_12148404, EPI_ISL_12148405, EPI_ISL_12148406, EPI_ISL_12148407, EPI_ISL_12148408, EPI_ISL_12150075, EPI_ISL_12172622, EPI_ISL_12172623, EPI_ISL_12172624, EPI_ISL_12172625, EPI_ISL_12172626, EPI_ISL_12172627, EPI_ISL_12172628, EPI_ISL_12172629, EPI_ISL_12175742, EPI_ISL_12175743, EPI_ISL_12175744, EPI_ISL_12175745, EPI_ISL_12175746, EPI_ISL_12175747, EPI_ISL_12175748, EPI_ISL_12175749, EPI_ISL_12175750, EPI_ISL_12175751, EPI_ISL_12175752, EPI_ISL_12175753, EPI_ISL_12175754, EPI_ISL_12175755, EPI_ISL_12175756, EPI_ISL_12175757, EPI_ISL_12175758, EPI_ISL_12175759, EPI_ISL_12175760, EPI_ISL_12175761, EPI_ISL_12175762, EPI_ISL_12175763, EPI_ISL_12175764, EPI_ISL_12175765, EPI_ISL_12175766, EPI_ISL_12175767, EPI_ISL_12175768, EPI_ISL_12175769, EPI_ISL_12175770, EPI_ISL_12175771, EPI_ISL_12175772, EPI_ISL_12175773, EPI_ISL_12175774, EPI_ISL_12175775, EPI_ISL_12175776, EPI_ISL_12175777, EPI_ISL_12175778, EPI_ISL_12175779, EPI_ISL_12175780, EPI_ISL_12175781, EPI_ISL_12175782, EPI_ISL_12175783, EPI_ISL_12175784, EPI_ISL_12175785, EPI_ISL_12175786, EPI_ISL_12175787, EPI_ISL_12175788, EPI_ISL_12175789, EPI_ISL_12175790, EPI_ISL_12175791, EPI_ISL_12175792, EPI_ISL_12175793, EPI_ISL_12175794, EPI_ISL_12175795, EPI_ISL_12175796, EPI_ISL_12175797, EPI_ISL_12175798, EPI_ISL_12175799, EPI_ISL_12175800, EPI_ISL_12175801, EPI_ISL_12175802, EPI_ISL_12175803, EPI_ISL_12175804, EPI_ISL_12175805, EPI_ISL_12175806, EPI_ISL_12175807, EPI_ISL_12175808, EPI_ISL_12175809, EPI_ISL_12175810, EPI_ISL_12175811, EPI_ISL_12175812, EPI_ISL_12175813, EPI_ISL_12175814, EPI_ISL_12175815, EPI_ISL_12175816, EPI_ISL_12175817, EPI_ISL_12175818, EPI_ISL_12175819, EPI_ISL_12175820, EPI_ISL_12175821, EPI_ISL_12175822, EPI_ISL_12175823, EPI_ISL_12175824, EPI_ISL_12175825, EPI_ISL_12175826, EPI_ISL_12175827, EPI_ISL_12175828, EPI_ISL_12175829, EPI_ISL_12175830, EPI_ISL_12175831, EPI_ISL_12175832, EPI_ISL_12175833, EPI_ISL_12175834, EPI_ISL_12175835, EPI_ISL_12175836, EPI_ISL_12175837, EPI_ISL_12175838, EPI_ISL_12175839, EPI_ISL_12175840, EPI_ISL_12175841, EPI_ISL_12175842, EPI_ISL_12175843, EPI_ISL_12175844, EPI_ISL_12175845, EPI_ISL_12175846, EPI_ISL_12175847, EPI_ISL_12175848, EPI_ISL_12175849, EPI_ISL_12175850, EPI_ISL_12175851, EPI_ISL_12175852, EPI_ISL_12175853, EPI_ISL_12175854, EPI_ISL_12175855, EPI_ISL_12175856, EPI_ISL_12175857, EPI_ISL_12175858, EPI_ISL_12175859, EPI_ISL_12175860, EPI_ISL_12175861, EPI_ISL_12175862, EPI_ISL_12175863, EPI_ISL_12175864, EPI_ISL_12175865, EPI_ISL_12175866, EPI_ISL_12175867, EPI_ISL_12175868, EPI_ISL_12175869, EPI_ISL_12175870, EPI_ISL_12175871, EPI_ISL_12175872, EPI_ISL_12175873			
see above	Kuala Lumpur International Airport	Institute for Medical Research, Infectious Disease Research Centre, National Institutes of Health, Ministry of Health Malaysia	Anasir MI; G.Adypatti NM; Jamaluddin MS; Kalyanasundram J; Kamel K; MatRahim N; Nawi MH; Suib FA; Suppiah J; Thayan R
EPI_ISL_12084330, EPI_ISL_12096708, EPI_ISL_12096709, EPI_ISL_12096710, EPI_ISL_12096711, EPI_ISL_12096712, EPI_ISL_12096713, EPI_ISL_12096714, EPI_ISL_12096715, EPI_ISL_12096716, EPI_ISL_12096717, EPI_ISL_12096718, EPI_ISL_12096719, EPI_ISL_12096720, EPI_ISL_12096721			
see above	Kuala Lumpur International Airport (KLIA) Health Office	Institute for Medical Research, Infectious Disease Research Centre, National Institutes of Health, Ministry of Health Malaysia	Ahmad FA; Ahmad Fazilah NA; Anasir MI; Azizan MA; G. Adypatti NM; Jamaluddin MS; Kamel K; Mohamad Sukri MZ; Norhisham SN; Ramly N; Robert F; Rosli NR; Suppiah J; Thayan R
EPI_ISL_12213458, EPI_ISL_12213462, EPI_ISL_12213464	MAKMAL KESIHATAN AWAM JOHOR BAHRU	UKM Medical Molecular Biology Institute (UMBI)	Khairun Nur Abd Ghafar; Mira Farzana Mohamad Mokhtar; Muhiddin Ishak; Nor Azila Muhammad Azami; Nur Alyaa Afifah Md Shahri; Nurul Syakima Ab Mutalib; Rahman Jamal; Ryia Illani Mohd Yunos; Siti Nur Hasanah Mohd Yusuf; Zahirrah Begam Mohamed Rasheed
EPI_ISL_12628105	MAKMAL KESIHATAN JOHOR	UKM Medical Molecular Biology Institute (UMBI)	Khairun Nur Abd Ghafar; Mira Farzana Mohamad Mokhtar; Muhiddin Ishak; Nor Azila Muhammad Azami; Nur Alyaa Afifah Md Shahri; Nurul Syakima Ab Mutalib; Rahman Jamal; Ryia Illani Mohd Yunos; Siti Nur Hasanah Mohd Yusuf; Zahirrah Begam Mohamed Rasheed
EPI_ISL_12534896, EPI_ISL_12534900, EPI_ISL_12534901, EPI_ISL_12534902	MAKMAL SEROLOGI, UNIT MIKROBIOLOGI, HSB	Tropical Infectious Diseases Research & Education Centre (TIDREC), Universiti Malaya	AsmaAnati CheMatSeri; Che-Norainon Yaacob; Jia-Yi Tan; Jo-Ern Wong; Kim-Kee Tan; Mulya-Mustika-Sari Zulkifli; Noor Syahida Azizan; Nur-Hidayana Mahfodz; Sazaly AbuBakar; Siti-Sarah Nor'e; Wei-Wei Aw
EPI_ISL_12510334, EPI_ISL_12510335	Miri Hospital Molecular Diagnostic Lab, Miri	Institute of Health and Community Medicine	Chan Chia Jui; Chua Hock Hin; David Perera; Ooi Mong How; Tonnii Sia Loong Loong
EPI_ISL_12899716	NATIONAL INSTITUTES OF HEALTH (NIH)	Institute for Medical Research, Infectious Disease Research Centre, National Institutes of Health, Ministry of Health Malaysia	Ahmad FA; Ahmad Fazilah NA; Anasir MI; Azizan MA; Kamel K; Mohamad Sukri MZ; Norhisham SN; Ramly N; Robert F; Rosli NR; Suppiah J; Thayan R
EPI_ISL_12289541, EPI_ISL_12289542, EPI_ISL_12289543, EPI_ISL_12289544, EPI_ISL_12289545, EPI_ISL_12289546, EPI_ISL_12289547, EPI_ISL_12289548, EPI_ISL_12289549, EPI_ISL_12321840, EPI_ISL_12321841, EPI_ISL_12321842, EPI_ISL_12321843, EPI_ISL_12321844, EPI_ISL_12321845, EPI_ISL_12321846, EPI_ISL_12321847, EPI_ISL_12321848, EPI_ISL_12321849, EPI_ISL_12321850, EPI_ISL_12321851, EPI_ISL_12321852, EPI_ISL_12321853, EPI_ISL_12321854, EPI_ISL_12321855, EPI_ISL_12321856, EPI_ISL_12321857, EPI_ISL_12321858, EPI_ISL_12321859, EPI_ISL_12321860, EPI_ISL_12321861, EPI_ISL_12321862, EPI_ISL_12321863, EPI_ISL_12321864, EPI_ISL_12321865, EPI_ISL_12321866, EPI_ISL_12321867, EPI_ISL_12321887, EPI_ISL_12321888, EPI_ISL_12321889, EPI_ISL_12321890, EPI_ISL_12321891, EPI_ISL_12321892, EPI_ISL_12321893, EPI_ISL_12321894, EPI_ISL_12321895, EPI_ISL_12321896, EPI_ISL_12321897, EPI_ISL_12321898, EPI_ISL_12321899, EPI_ISL_12665230, EPI_ISL_12665231, EPI_ISL_12749683			
see above	NATIONAL PUBLIC HEALTH LABORATORY	Institute for Medical Research, Infectious Disease Research Centre, National Institutes of Health, Ministry of Health Malaysia	Ahmad FA; Ahmad Fazilah NA; Anasir MI; Azizan MA; Kamel K; Mohamad Sukri MZ; Norhisham SN; Ramly N; Robert F; Rosli NR; Suppiah J; Thayan R
EPI_ISL_12099756, EPI_ISL_12099764, EPI_ISL_12252831, EPI_ISL_12252832, EPI_ISL_12252833, EPI_ISL_12252834, EPI_ISL_12252835, EPI_ISL_12252836, EPI_ISL_12252837, EPI_ISL_12252838			
see above	National Public Health Laboratory	National Public Health Laboratory	Kamal Hisham Bin Kamarul Zaman; Lim Cheau Ju; Muhamad Syamim Bin Roslan; Noriah Binti Mohd Yusof; Nur Hazliza Binti Salleh; Rehan Shuhada Binti Abu Bakar; Selvanesan A/L Sengol; Syahida Omar; Yu Kie A/P Chem
EPI_ISL_13026074, EPI_ISL_13026075, EPI_ISL_13026076, EPI_ISL_13026077, EPI_ISL_13026078, EPI_ISL_13026079	Neogenix Laboratoire Sdn Bhd	Universiti Putra Malaysia (UPM)	Chee Sian Kuan; Choo Yee Yu; Hui Yee Chee; Nancy Woan Charn Liew; Narcisse Joseph; Nurulfiza Mat Isa; Sie Yeng Wong; Su Mei Yew; Syafinaz Amin-Nordin; Zunita Zakaria
EPI_ISL_12510045, EPI_ISL_12510046, EPI_ISL_12510047, EPI_ISL_12510048, EPI_ISL_12510049, EPI_ISL_12510050, EPI_ISL_12510051, EPI_ISL_12510052, EPI_ISL_12510053, EPI_ISL_12510054, EPI_ISL_12510055, EPI_ISL_12510056			
see above	Normah Medical Specialist Centre, Kuching	Institute of Health and Community Medicine	Chan Chia Jui; Chua Hock Hin; David Perera; Ikwannuddin Y; Ooi Mong How; Tonnii Sia Loong Loong
EPI_ISL_12084321	PELABUHAN KLANG HEALTH CLINIC	Institute for Medical Research, Infectious Disease Research Centre, National Institutes of Health, Ministry of Health Malaysia	Ahmad FA; Ahmad Fazilah NA; Anasir MI; Azizan MA; G. Adypatti NM; Jamaluddin MS; Kamel K; Mohamad Sukri MZ; Norhisham SN; Ramly N; Robert F; Rosli NR; Suppiah J; Thayan R
EPI_ISL_12213459, EPI_ISL_12213460, EPI_ISL_12213461, EPI_ISL_12628107	PKD JOHOR BAHRU	UKM Medical Molecular Biology Institute (UMBI)	Khairun Nur Abd Ghafar; Mira Farzana Mohamad Mokhtar; Muhiddin Ishak; Nor Azila Muhammad Azami; Nur Alyaa Afifah Md Shahri; Nurul Syakima Ab Mutalib; Rahman Jamal; Ryia Illani Mohd Yunos; Siti Nur Hasanah Mohd Yusuf; Zahirrah Begam Mohamed Rasheed
EPI_ISL_12628114, EPI_ISL_12628115, EPI_ISL_12628118, EPI_ISL_12628123	PKD KULAI	UKM Medical Molecular Biology Institute (UMBI)	Khairun Nur Abd Ghafar; Mira Farzana Mohamad Mokhtar; Muhiddin Ishak; Nor Azila Muhammad Azami; Nur Alyaa Afifah Md Shahri; Nurul Syakima Ab Mutalib; Rahman Jamal; Ryia Illani Mohd Yunos; Siti Nur Hasanah Mohd Yusuf; Zahirrah Begam Mohamed Rasheed
EPI_ISL_12628116	PKD MUAR	UKM Medical Molecular Biology Institute (UMBI)	Khairun Nur Abd Ghafar; Mira Farzana Mohamad Mokhtar; Muhiddin Ishak; Nor Azila Muhammad Azami; Nur Alyaa Afifah Md Shahri; Nurul Syakima Ab Mutalib; Rahman Jamal; Ryia Illani Mohd Yunos; Siti Nur Hasanah Mohd Yusuf; Zahirrah Begam Mohamed Rasheed
EPI_ISL_12899715	PORT KLANG HEALTH CLINIC	Institute for Medical Research, Infectious Disease Research Centre, National Institutes of Health, Ministry of Health Malaysia	Ahmad FA; Ahmad Fazilah NA; Anasir MI; Azizan MA; Kamel K; Mohamad Sukri MZ; Norhisham SN; Ramly N; Robert F; Rosli NR; Suppiah J; Thayan R
EPI_ISL_12899685	PUTRAJAYA DISTRICT HEALTH OFFICE	Institute for Medical Research, Infectious Disease Research Centre, National Institutes of Health, Ministry of Health Malaysia	Ahmad FA; Ahmad Fazilah NA; Anasir MI; Azizan MA; Kamel K; Mohamad Sukri MZ; Norhisham SN; Ramly N; Robert F; Rosli NR; Suppiah J; Thayan R
EPI_ISL_12289476, EPI_ISL_12321869, EPI_ISL_12400591	QUEEN ELIZABETH HOSPITAL	Institute for Medical Research, Infectious Disease Research Centre, National Institutes of Health, Ministry of Health Malaysia	Ahmad FA; Ahmad Fazilah NA; Anasir MI; Azizan MA; Kamel K; Mohamad Sukri MZ; Norhisham SN; Ramly N; Robert F; Rosli NR; Suppiah J; Thayan R
EPI_ISL_12289478	SELAYANG HOSPITAL	Institute for Medical Research, Infectious Disease Research Centre, National Institutes of Health, Ministry of Health Malaysia	Ahmad FA; Ahmad Fazilah NA; Anasir MI; Azizan MA; Kamel K; Mohamad Sukri MZ; Norhisham SN; Ramly N; Robert F; Rosli NR; Suppiah J; Thayan R
EPI_ISL_12510172, EPI_ISL_12510174, EPI_ISL_12510175, EPI_ISL_12510176, EPI_ISL_12510177	Sarawak General Hospital (Kuching)	Institute of Health and Community Medicine	Chan Chia Jui; Chua Hock Hin; David Perera; Ooi Mong How
EPI_ISL_12099568, EPI_ISL_12099695, EPI_ISL_12099699, EPI_ISL_12099700, EPI_ISL_12099701, EPI_ISL_12099703, EPI_ISL_12099704, EPI_ISL_12099705, EPI_ISL_12099706, EPI_ISL_12099707, EPI_ISL_12099708, EPI_ISL_12099709, EPI_ISL_12099710, EPI_ISL_12099711, EPI_ISL_12099712, EPI_ISL_12099750			
see above	Sibu Hospital, PCR lab (Sibu, Sarawak)	Institute of Health and Community Medicine	Chan Chia Jui; Chua Hock Hin; David Perera; Ooi Mong How; Tonnii Sia Loong Loong
EPI_ISL_13026088, EPI_ISL_13026089, EPI_ISL_13026090, EPI_ISL_13026091, EPI_ISL_13026092, EPI_ISL_13026093, EPI_ISL_13026094, EPI_ISL_13026095			

see above	Synapse Sdn. Bhd.	Universiti Putra Malaysia (UPM)	Choo Yee Yu; Hui Jen Soe; Hui Yee Chee; Nancy Woan Charn Liew; Narcisse Joseph; Nurulfiza Mat Isa; Rachna Kairon; Sie Yeng Wong; Syafinaz Amin-Nordin; Zunita Zakaria
EPI_ISL_12899711, EPI_ISL_12899712	TELOK DATOK HEALTH CLINIC	Institute for Medical Research, Infectious Disease Research Centre, National Institutes of Health, Ministry of Health Malaysia	Ahmad FA; Ahmad Fazilah NA; Anasir MI; Azizan MA; Kamel K; Mohamad Sukri MZ; Norhisham SN; Ramly N; Robert F; Rosli NR; Suppiah J; Thayan R
EPI_ISL_12899718, EPI_ISL_12899719	TELOK PANGLIMA GARANG HEALTH CLINIC	Institute for Medical Research, Infectious Disease Research Centre, National Institutes of Health, Ministry of Health Malaysia	Ahmad FA; Ahmad Fazilah NA; Anasir MI; Azizan MA; Kamel K; Mohamad Sukri MZ; Norhisham SN; Ramly N; Robert F; Rosli NR; Suppiah J; Thayan R
EPI_ISL_13047118	TOK UBAN HEALTH CLINIC	MOLECULAR RESEARCH LABORATORY	Abdul Haris bin Muhammad; Ahmad Sukari Bin Halim; Alexander Chong Shu Chien; Azian Harun; Chan Yean Yean; Chua Wei Chuan; Farahana binti Mohamed; Kirnpal Kaur Banga Singh; Lau Nyok Sean; Lee Lih Huey; Lim Shu Yong; Maizun binti Mohd Zain; Mohd Nadzri Abu Yazid; Mohd Zulkifli Salleh; Muhammad Azamuddeen bin Mohammad Nasir; Muhammad Fazli bin Khalid; Muhammad Nashrul Farhan Samsudin; Muhammad Zarul Hanifah Bin Md Zoqratt; Nik Zuraina binti Nik Mohd Noor; Noor Hafizan binti Mat Salleh; Nurfadhlina Musa; Qasim Ayub; Rosline Hassan; Sadequr Rahman; Syahida binti Omar; Wan Mohd Zahiruddin Wan Mohammad; Wardah Yusof; Zaini bin Hussin; Zakuan Zainy Deris
EPI_ISL_12321873, EPI_ISL_12400589	TUNKU AZIZAH HOSPITAL	Institute for Medical Research, Infectious Disease Research Centre, National Institutes of Health, Ministry of Health Malaysia	Ahmad FA; Ahmad Fazilah NA; Anasir MI; Azizan MA; Kamel K; Mohamad Sukri MZ; Norhisham SN; Ramly N; Robert F; Rosli NR; Suppiah J; Thayan R
EPI_ISL_12560259	UMMC	Department of Medical Microbiology, Faculty of Medicine, University of Malaya; University of Malaya Medical Centre	I-Ching SAM; Jolene Yin Ling FU; Omar Khalilur Rahman; Yee Von Lee; Yoke Fun Chan

We gratefully acknowledge the following Authors from the Originating laboratories responsible for obtaining the specimens, as well as the Submitting laboratories where the genome data were generated and shared via GISAID, on which this research is based.

All Submitters of data may be contacted directly via www.gisaid.org

Authors are sorted alphabetically.

Accession ID	Originating Laboratory	Submitting Laboratory	Authors
EPI_ISL_12321877, EPI_ISL_12321878, EPI_ISL_12321879, EPI_ISL_12321880, EPI_ISL_12321881, EPI_ISL_12321901, EPI_ISL_12899670			
see above	AMPANG HOSPITAL	Institute for Medical Research, Infectious Disease Research Centre, National Institutes of Health, Ministry of Health Malaysia	Ahmad FA; Ahmad Fazilah NA; Anasir MI; Azizan MA; Kamel K; Mohamad Sukri MZ; Norhisham SN; Ramly N; Robert F; Rosli NR; Suppiah J; Thayan R
EPI_ISL_12321885	BANTING HOSPITAL	Institute for Medical Research, Infectious Disease Research Centre, National Institutes of Health, Ministry of Health Malaysia	Ahmad FA; Ahmad Fazilah NA; Anasir MI; Azizan MA; Kamel K; Mohamad Sukri MZ; Norhisham SN; Ramly N; Robert F; Rosli NR; Suppiah J; Thayan R
EPI_ISL_12235865, EPI_ISL_12235866, EPI_ISL_12235867, EPI_ISL_12235868, EPI_ISL_12235869, EPI_ISL_12235870, EPI_ISL_12235871, EPI_ISL_12235872, EPI_ISL_12235873, EPI_ISL_12235874, EPI_ISL_12235875, EPI_ISL_12235876, EPI_ISL_12235877, EPI_ISL_12235878, EPI_ISL_12235879, EPI_ISL_12235880, EPI_ISL_12235881, EPI_ISL_12235882, EPI_ISL_12235883, EPI_ISL_12235884, EPI_ISL_12235885, EPI_ISL_12235886, EPI_ISL_12235887, EPI_ISL_12235888, EPI_ISL_12235889, EPI_ISL_12235890, EPI_ISL_12235891, EPI_ISL_12235892, EPI_ISL_12235893, EPI_ISL_12235894, EPI_ISL_12972566, EPI_ISL_12972567, EPI_ISL_12972568, EPI_ISL_12972569, EPI_ISL_12972570, EPI_ISL_12972571, EPI_ISL_12972572, EPI_ISL_12972573, EPI_ISL_12972583, EPI_ISL_12973064, EPI_ISL_12973065, EPI_ISL_12973066, EPI_ISL_12973067, EPI_ISL_12973068, EPI_ISL_12973069, EPI_ISL_12973070, EPI_ISL_12973071, EPI_ISL_12973072, EPI_ISL_12973073, EPI_ISL_12973074, EPI_ISL_12973075, EPI_ISL_12973076, EPI_ISL_12973077, EPI_ISL_12973078, EPI_ISL_12973079, EPI_ISL_12973080, EPI_ISL_12973081, EPI_ISL_12973082, EPI_ISL_12973083, EPI_ISL_12973084, EPI_ISL_12973085, EPI_ISL_12973086, EPI_ISL_12973087, EPI_ISL_12973088, EPI_ISL_12973089, EPI_ISL_12973090, EPI_ISL_12973091, EPI_ISL_12973092, EPI_ISL_12973093, EPI_ISL_12973094, EPI_ISL_12973095, EPI_ISL_12973096, EPI_ISL_12973097, EPI_ISL_12973098, EPI_ISL_12973099, EPI_ISL_12973100, EPI_ISL_12973101, EPI_ISL_12973102, EPI_ISL_12973103, EPI_ISL_12973104, EPI_ISL_12973105, EPI_ISL_12973106, EPI_ISL_12973107, EPI_ISL_12973108, EPI_ISL_12973109, EPI_ISL_12973110, EPI_ISL_12973111, EPI_ISL_12973112, EPI_ISL_12983188, EPI_ISL_12983189, EPI_ISL_12983190, EPI_ISL_12983191, EPI_ISL_12983192, EPI_ISL_12983193, EPI_ISL_12983194, EPI_ISL_12983195, EPI_ISL_12983196, EPI_ISL_12983197, EPI_ISL_12983198, EPI_ISL_12983199, EPI_ISL_12983200			
see above	BP Healthcare Group	Institute for Medical Research, Infectious Disease Research Centre, National Institutes of Health, Ministry of Health Malaysia	Anasir MI; G.Adypatti NM; Jamaluddin MS; Kalyanasundram J; Kamel K; MatRahim N; Nawi MH; Suib FA; Suppiah J; Thayan R
EPI_ISL_12099567, EPI_ISL_12099569, EPI_ISL_12099595, EPI_ISL_12099599, EPI_ISL_12099600, EPI_ISL_12099601, EPI_ISL_12099602, EPI_ISL_12099603, EPI_ISL_12099604, EPI_ISL_12099605, EPI_ISL_12099725, EPI_ISL_12510070, EPI_ISL_12510071			
see above	Bintulu Hospital PCR Lab, Bintulu	Institute of Health and Community Medicine	Chan Chia Jui; Chien Su Lin; Chua Hock Hin; David Perera; Ooi Mong How; Tonnii Sia Loong Loong
EPI_ISL_12510295, EPI_ISL_12510296, EPI_ISL_12510298, EPI_ISL_12510300, EPI_ISL_12510301, EPI_ISL_12510302, EPI_ISL_12510303, EPI_ISL_12510304, EPI_ISL_12510305, EPI_ISL_12510306, EPI_ISL_12510307, EPI_ISL_12510310, EPI_ISL_12510313			
see above	Bintulu Medical Centre (Bintulu)	Institute of Health and Community Medicine	Chan Chia Jui; David Perera; Ooi Mong How; Wong Jyn Shan
EPI_ISL_12510199, EPI_ISL_12510200, EPI_ISL_12510202, EPI_ISL_12510203, EPI_ISL_12510204, EPI_ISL_12510239, EPI_ISL_12510240, EPI_ISL_12510241, EPI_ISL_12510242, EPI_ISL_12510243, EPI_ISL_12510245, EPI_ISL_12510246, EPI_ISL_12510248, EPI_ISL_12510252			
see above	Borneo Medical Centre (Kuching)	Institute of Health and Community Medicine	Chan Chia Jui; David Perera; Ooi Mong How; Wong Jyn Shan
EPI_ISL_12534911, EPI_ISL_12534922, EPI_ISL_12534923, EPI_ISL_12534924	FORENSIC HOSPITAL SULTANAH BAHIYAH	Tropical Infectious Diseases Research & Education Centre (TIDREC), Universiti Malaya	AsmaAnati CheMatSeri; Che-Norainon Yaacob; Jia-Yi Tan; Jo-Ern Wong; Kim-Kee Tan; Mulya-Mustika-Sari Zulkifli; Noor Syahida Azizan; Nur-Hidayana Mahfodz; Sazaly AbuBakar; Siti-Sarah Nor’e; Wei-Wei Aw
EPI_ISL_12510057, EPI_ISL_12510076, EPI_ISL_12510077, EPI_ISL_12510078, EPI_ISL_12510079, EPI_ISL_12510081, EPI_ISL_12510082, EPI_ISL_12510083, EPI_ISL_12510084, EPI_ISL_12510085, EPI_ISL_12510086, EPI_ISL_12510087, EPI_ISL_12510088, EPI_ISL_12510089, EPI_ISL_12510090, EPI_ISL_12510091, EPI_ISL_12510092, EPI_ISL_12510093, EPI_ISL_12510094, EPI_ISL_12510095, EPI_ISL_12510096, EPI_ISL_12510097, EPI_ISL_12510098, EPI_ISL_12510099, EPI_ISL_12510363, EPI_ISL_12510364, EPI_ISL_12510366, EPI_ISL_12510367, EPI_ISL_12510368, EPI_ISL_12510369, EPI_ISL_12510370, EPI_ISL_12510371, EPI_ISL_12510372, EPI_ISL_12510373, EPI_ISL_12510374, EPI_ISL_12510375, EPI_ISL_12510376, EPI_ISL_12510377, EPI_ISL_12510378, EPI_ISL_12510379, EPI_ISL_12510380, EPI_ISL_12510381, EPI_ISL_12510382, EPI_ISL_12510383, EPI_ISL_12510384, EPI_ISL_12510385, EPI_ISL_12510386, EPI_ISL_12510388, EPI_ISL_12510389, EPI_ISL_12510390, EPI_ISL_12510391			
see above	Gribbles Diagnostic Lab @Timberland Medical Centre, Kuching	Institute of Health and Community Medicine	Chan Chia Jui; Chua Hock Hin; David Perera; Liew Chin Chin J; Ooi Mong How; Tonnii Sia Loong Loong; Wong Jyn Shan
EPI_ISL_12628099, EPI_ISL_12628132, EPI_ISL_12628136, EPI_ISL_12628137, EPI_ISL_12628138	HOSPITAL MELAKA	UKM Medical Molecular Biology Institute (UMBI)	Khairun Nur Abd Ghafar; Mira Farzana Mohamad Mokhtar; Muhiddin Ishak; Nor Azila Muhammad Azami; Nur Alyaa Afifah Md Shahri; Nurul Syakima Ab Mutalib; Rahman Jamal; Ryia Illani Mohd Yunos; Siti Nur Hasanah Mohd Yusuf; Zahirrah Begam Mohamed Rasheed
EPI_ISL_12771319, EPI_ISL_12771321	HOSPITAL PULAU PINANG	iPROMISE, UiTM	Ariza Adnan; Fadzilah Mohd Nor; Lim Wai Feng; Mohd Asif Mohd Sukri; Mohd Nur Fakhruzzaman Noorizhab; Mohd Zaki Salleh; Sazzli Shahlan Kassim; Siti Farah Alwani Mohd Nawi; Siti Hamimah Sheikh Abdul Kadir; Teh Lay Kek; Wang Seok Mui
EPI_ISL_13047113	HOSPITAL SULTAN ISMAIL PETRA, KUALA KRAI, KELANTAN	MOLECULAR RESEARCH LABORATORY	Abdul Haris bin Muhammad; Ahmad Sukari Bin Halim; Alexander Chong Shu Chien; Azian Harun; Chan Yean Yean; Chua Wei Chuan; Farahana binti Mohamed; Kirnpal Kaur Banga Singh; Lau Nyok Sean; Lee Lih Huey; Lim Shu Yong; Maizun binti Mohd Zain; Mohd Nadzri Abu Yazid; Mohd Zulkifli Salleh; Muhammad Azamuddeen bin Mohammad Nasir; Muhammad Fazli bin Khalid; Muhammad Nashrul Farhan Samsudin; Muhammad Zarul Hanifah Bin Md Zoqratt; Nik Zuraina binti Nik Mohd Noor; Noor Hafizan binti Mat Salleh; Nurfadhlina Musa; Qasim Ayub; Rosline Hassan; Sadequr Rahman; Syahida binti Omar; Wan Mohd Zahiruddin Wan Mohammad; Wardah Yusof; Zaini bin Hussin; Zakuan Zainy Deris
EPI_ISL_12771258, EPI_ISL_12771259, EPI_ISL_12771260, EPI_ISL_12771261, EPI_ISL_12771262, EPI_ISL_12771263, EPI_ISL_12771322, EPI_ISL_12771334			
see above	HRPB	iPROMISE, UiTM	Ariza Adnan; Fadzilah Mohd Nor; Lim Wai Feng; Mohd Asif Mohd Sukri; Mohd Nur Fakhruzzaman Noorizhab; Mohd Zaki Salleh; Sazzli Shahlan Kassim; Siti Farah Alwani Mohd Nawi; Siti Hamimah Sheikh Abdul Kadir; Teh Lay Kek; Wang Seok Mui
EPI_ISL_12213469, EPI_ISL_12213470, EPI_ISL_12213471, EPI_ISL_12213472, EPI_ISL_12628098, EPI_ISL_12628130	HSAJB	UKM Medical Molecular Biology Institute (UMBI)	Khairun Nur Abd Ghafar; Mira Farzana Mohamad Mokhtar; Muhiddin Ishak; Nor Azila Muhammad Azami; Nur Alyaa Afifah Md Shahri; Nurul Syakima Ab Mutalib; Rahman Jamal; Ryia Illani Mohd Yunos; Siti Nur Hasanah Mohd Yusuf; Zahirrah Begam Mohamed Rasheed
EPI_ISL_12771320	Hospital Kepala Batas	iPROMISE, UiTM	Ariza Adnan; Fadzilah Mohd Nor; Lim Wai Feng; Mohd Asif Mohd Sukri; Mohd Nur Fakhruzzaman Noorizhab; Mohd Zaki Salleh; Sazzli Shahlan Kassim; Siti Farah Alwani Mohd Nawi; Siti Hamimah Sheikh Abdul Kadir; Teh Lay Kek; Wang Seok Mui
EPI_ISL_12980332, EPI_ISL_12980333, EPI_ISL_12980336	Hospital Kuala Penyu	Makmal Kesihatan Awam Kota Kinabalu	Erfiana Shamsaddin; Joel Judson Jaimin; Kitty Christhoper Hollip; Maznin Nisah Wajil; Mohd. Nazrin Shah Bin Jamlee; Muhamad Shah Arip; Rashidah Mohamad; Rufina Mohd Yassin; Tan Yee Chee
EPI_ISL_12832820	Hospital Labuan	Biotechnology Research Institute, Universiti Malaysia Sabah	Cahyo Budiman; Eric Chong Tzyy Jiann; Lee Ping Chin; Nurul Elyani Mohamad; Sylvia Jerome Daim; Yew Chee Wei
EPI_ISL_12708538, EPI_ISL_12708539, EPI_ISL_12708540, EPI_ISL_12708541, EPI_ISL_12708542, EPI_ISL_12708543, EPI_ISL_12708544, EPI_ISL_12708545, EPI_ISL_12708546, EPI_ISL_12708547, EPI_ISL_12708551, EPI_ISL_12935247			
see above	Hospital Raja Perempuan Zainab II, Kota Bharu	Molecular Research Laboratory	Abdul Haris bin Muhammad; Ahmad Sukari Bin Halim; Alexander Chong Shu Chien; Asraihan Che Abdul Malik; Azian Harun; Chan Yean Yean; Chua Wei Chuan; Farahana binti Mohamed; Kirnpal Kaur Banga Singh; Lau Nyok Sean; Lee Lih Huey; Lim Shu Yong; Maizun binti Mohd Zain; Mera Edora Binti Abdul Manap; Mohd Iman hafiz Bin Ibrahim; Mohd Nadzri Abu Yazid; Mohd Zulkifli Salleh; Muhamad Khairul Amirin Bin Zulkifli; Muhammad Azamuddeen bin Mohammad Nasir; Muhammad Fazli bin Khalid; Muhammad Nashrul Farhan Samsudin; Muhammad Zarul Hanifah Bin Md Zoqratt; Nik Zuraina binti Nik Mohd Noor; Noor Hafizan binti Mat Salleh; Nor Amizara Binti Azami; Nor Suhana Binti Mohd Satar; Nur Syuhada Binti Abdul Rahim; Nur-Leem Binti Murshid; Nurfadhlina Musa; Qasim Ayub; Rosline Hassan; Sadequr Rahman; Siti Nur Aisyah Binti Mohamad Sham; Syahida binti Omar; Wan Mohd Zahiruddin Wan Mohammad; Wardah Yusof; Zaini bin Hussin; Zakuan Zainy Deris
EPI_ISL_12980331	Hospital Sipitang	Makmal Kesihatan Awam Kota Kinabalu	Erfiana Shamsaddin; Joel Judson Jaimin; Kitty Christhoper Hollip; Maznin Nisah Wajil; Mohd. Nazrin Shah Bin Jamlee; Muhamad Shah Arip; Rashidah Mohamad; Rufina Mohd Yassin; Tan Yee Chee
EPI_ISL_12277175, EPI_ISL_12277176	Hospital Sungai Buloh	Malaysia Genome and Vaccine Institute	Azrin Ahmad; Enizza Kasim; Irni Suhayu Sapian; Mohd Faizal Abu Bakar; Mohd Ghows Mohd Azzam.; Mohd Noor Mat Isa; Nor Azfa Johari; Nurhezreen Md Iqbal; Shamsidar Sopie; Siti Noraini Othman; Yusuf Muhammad Noor
EPI_ISL_12534921	Hospital sultanah bahiyah	Tropical Infectious Diseases Research & Education Centre (TIDREC), Universiti Malaya	AsmaAnati CheMatSeri; Che-Norainon Yaacob; Jia-Yi Tan; Jo-Ern Wong; Kim-Kee Tan; Mulya-Mustika-Sari Zulkifli; Noor Syahida Azizan; Nur-Hidayana Mahfodz; Sazaly AbuBakar; Siti-Sarah Nor’e; Wei-Wei Aw
EPI_ISL_12771317	JABATAN PERUBATAN FORENSIK HPP	iPROMISE, UiTM	Ariza Adnan; Fadzilah Mohd Nor; Lim Wai Feng; Mohd Asif Mohd Sukri; Mohd Nur Fakhruzzaman Noorizhab; Mohd Zaki Salleh; Sazzli Shahlan Kassim; Siti Farah Alwani Mohd Nawi; Siti Hamimah Sheikh Abdul Kadir; Teh Lay Kek; Wang Seok Mui
EPI_ISL_12771201	Jabatan perubatan HTAA	iPROMISE, UiTM	Ariza Adnan; Fadzilah Mohd Nor; Lim Wai Feng; Mohd Asif Mohd Sukri; Mohd Nur Fakhruzzaman Noorizhab; Mohd Zaki Salleh; Sazzli Shahlan Kassim; Siti Farah Alwani Mohd Nawi; Siti Hamimah Sheikh Abdul Kadir; Teh Lay Kek; Wang Seok Mui
EPI_ISL_12899697	KUALA LANGAT DISTRICT HEALTH	Institute for Medical Research, Infectious Disease Research Centre,	Ahmad FA; Ahmad Fazilah NA; Anasir MI; Azizan MA; Kamel K; Mohamad Sukri MZ; Norhisham SN; Ramly N; Robert F; Rosli NR; Suppiah J; Thayan R

	OFFICE	National Institutes of Health, Ministry of Health Malaysia	
EPI_ISL_12899693, EPI_ISL_12899694	KUALA LUMPUR GENERAL HOSPITAL	Institute for Medical Research, Infectious Disease Research Centre, National Institutes of Health, Ministry of Health Malaysia	Ahmad FA; Ahmad Fazilah NA; Anasir MI; Azizan MA; Kamel K; Mohamad Sukri MZ; Norhisham SN; Ramly N; Robert F; Rosli NR; Suppiah J; Thayan R
EPI_ISL_11976278, EPI_ISL_12665232, EPI_ISL_12665233	KUALA LUMPUR HOSPITAL	Institute for Medical Research, Infectious Disease Research Centre, National Institutes of Health, Ministry of Health Malaysia	Ahmad FA; Ahmad Fazilah NA; Anasir MI; Azizan MA; Kamel K; Mohamad Sukri MZ; Norhisham SN; Ramly N; Robert F; Rosli NR; Suppiah J; Thayan R
EPI_ISL_12769961	KUALA LUMPUR INTERNATIONAL AIRPORT (KLIA) HEALTH OFFICE	Institute for Medical Research, Infectious Disease Research Centre, National Institutes of Health	Ahmad FA; Ahmad Fazilah NA; Anasir MI; Azizan MA; Kamel K; Mohamad Sukri MZ; Norhisham SN; Ramly N; Robert F; Rosli NR; Suppiah J; Thayan R
EPI_ISL_12665252, EPI_ISL_12665253, EPI_ISL_12665254, EPI_ISL_12665255, EPI_ISL_12665256, EPI_ISL_12665257, EPI_ISL_12665258, EPI_ISL_12665259, EPI_ISL_12665260, EPI_ISL_12665261, EPI_ISL_12665262, EPI_ISL_12665263, EPI_ISL_12665264, EPI_ISL_12665265, EPI_ISL_12665266, EPI_ISL_12665267, EPI_ISL_12665268, EPI_ISL_12665269, EPI_ISL_12665270, EPI_ISL_12665271, EPI_ISL_12665272, EPI_ISL_12665273, EPI_ISL_12665274, EPI_ISL_12665279, EPI_ISL_12665280, EPI_ISL_12665281, EPI_ISL_12749681, EPI_ISL_12749682			
see above	KUALA LUMPUR INTERNATIONAL AIRPORT (KLIA) HEALTH OFFICE	Institute for Medical Research, Infectious Disease Research Centre, National Institutes of Health, Ministry of Health Malaysia	Ahmad FA; Ahmad Fazilah NA; Anasir MI; Azizan MA; Kamel K; Mohamad Sukri MZ; Norhisham SN; Ramly N; Robert F; Rosli NR; Suppiah J; Thayan R
EPI_ISL_12980334, EPI_ISL_12980335	Klinik Kesihatan Luyang	Makmal Kesihatan Awam Kota Kinabalu	Erfiana Shamsaddin; Joel Judson Jaimin; Kitty Christhoper Hollip; Maznin Nisah Wajil; Mohd. Nazrin Shah Bin Jamlee; Muhamad Shah Arip; Rashidah Mohamad; Rufina Mohd Yassin; Tan Yee Chee
EPI_ISL_12918962, EPI_ISL_12918963, EPI_ISL_12919028, EPI_ISL_12919029, EPI_ISL_12919030, EPI_ISL_12919031, EPI_ISL_12919032, EPI_ISL_12919033, EPI_ISL_12919034, EPI_ISL_12919035, EPI_ISL_12919036, EPI_ISL_12919037, EPI_ISL_12919038, EPI_ISL_12919039, EPI_ISL_12919040, EPI_ISL_12919041, EPI_ISL_12919042, EPI_ISL_12919043, EPI_ISL_12919044, EPI_ISL_12919045, EPI_ISL_12919046, EPI_ISL_12919047, EPI_ISL_12919048, EPI_ISL_12919049, EPI_ISL_12919050, EPI_ISL_12919051, EPI_ISL_12919052, EPI_ISL_12919053, EPI_ISL_12919054, EPI_ISL_12919055, EPI_ISL_12919084, EPI_ISL_12919252, EPI_ISL_12919254, EPI_ISL_12942742			
see above	Kuala Lumpur International Airport	Institute for Medical Research, Infectious Disease Research Centre, National Institutes of Health, Ministry of Health Malaysia	Anasir MI; G.Adypatti NM; Jamaluddin MS; Kalyanasundram J; Kamel K; MatRahim N; Nawi MH; Suib FA; Suppiah J; Thayan R
EPI_ISL_12628103, EPI_ISL_12628104, EPI_ISL_12628106, EPI_ISL_12628109, EPI_ISL_12628117	MAKMAL KESIHATAN JOHOR	UKM Medical Molecular Biology Institute (UMBI)	Khairun Nur Abd Ghafar; Mira Farzana Mohamad Mokhtar; Muhiddin Ishak; Nor Azila Muhammad Azami; Nur Alyaa Afifah Md Shahri; Nurul Syakima Ab Mutalib; Rahman Jamal; Ryia Illani Mohd Yunos; Siti Nur Hasanah Mohd Yusuf; Zahirrah Begam Mohamed Rasheed
EPI_ISL_12534903, EPI_ISL_12534904, EPI_ISL_12534912, EPI_ISL_12534913, EPI_ISL_12534914, EPI_ISL_12534916, EPI_ISL_12534917, EPI_ISL_12534918, EPI_ISL_12534919, EPI_ISL_12534920			
see above	MAKMAL SEROLOGI, UNIT MIKROBIOLOGI, HSB	Tropical Infectious Diseases Research & Education Centre (TIDREC), Universiti Malaya	AsmaAnati CheMatSeri; Che-Norainon Yaacob; Jia-Yi Tan; Jo-Ern Wong; Kim-Kee Tan; Mulya-Mustika-Sari Zulkifli; Noor Syahida Azizan; Nur-Hidayana Mahfodz; Sazaly AbuBakar; Siti-Sarah Nor'e; Wei-Wei Aw
EPI_ISL_12534915	MAKMAL SEROLOGI, UNITMIKROBIOLGI, HSB	Tropical Infectious Diseases Research & Education Centre (TIDREC), Universiti Malaya	AsmaAnati CheMatSeri; Che-Norainon Yaacob; Jia-Yi Tan; Jo-Ern Wong; Kim-Kee Tan; Mulya-Mustika-Sari Zulkifli; Noor Syahida Azizan; Nur-Hidayana Mahfodz; Sazaly AbuBakar; Siti-Sarah Nor'e; Wei-Wei Aw
EPI_ISL_12510337, EPI_ISL_12510338, EPI_ISL_12510339, EPI_ISL_12510340, EPI_ISL_12510341, EPI_ISL_12510342, EPI_ISL_12510343, EPI_ISL_12510344, EPI_ISL_12510345, EPI_ISL_12510346, EPI_ISL_12510347, EPI_ISL_12510348			
see above	Miri Hospital Molecular Diagnostic Lab, Miri	Institute of Health and Community Medicine	Chan Chia Jui; Chua Hock Hin; David Perera; Ooi Mong How; Tonnii Sia Loong Loong
EPI_ISL_12099757, EPI_ISL_12099758, EPI_ISL_12099759, EPI_ISL_12099760, EPI_ISL_12099761, EPI_ISL_12429733, EPI_ISL_12429734, EPI_ISL_12429735, EPI_ISL_12429736, EPI_ISL_12429737, EPI_ISL_12429738			
see above	National Public Health Laboratory	National Public Health Laboratory	Kamal Hisham Bin Kamarul Zaman; Lim Cheau Ju; Maznin Nisah Binti Wajil; Muhd Hashim Chew; Noriah Binti Mohd Yusof; Rehan Shuhada Binti Abu Bakar; Selvanesan A/L Sengol; Syahida Omar; Yu Kie A/P Chem
EPI_ISL_12510058, EPI_ISL_12510059, EPI_ISL_12510060, EPI_ISL_12510061, EPI_ISL_12510062, EPI_ISL_12510063, EPI_ISL_12510064, EPI_ISL_12510065, EPI_ISL_12510066, EPI_ISL_12510067, EPI_ISL_12510068, EPI_ISL_12510069, EPI_ISL_12510072, EPI_ISL_12510073, EPI_ISL_12510075			
see above	Normah Medical Specialist Centre, Kuching	Institute of Health and Community Medicine	Chan Chia Jui; Chua Hock Hin; David Perera; Ikwannuddin Y; Ooi Mong How; Tonnii Sia Loong Loong
EPI_ISL_12665234, EPI_ISL_12665235, EPI_ISL_12665236	PAHANG STATE HEALTH DEPARTMENT	Institute for Medical Research, Infectious Disease Research Centre, National Institutes of Health, Ministry of Health Malaysia	Ahmad FA; Ahmad Fazilah NA; Anasir MI; Azizan MA; Kamel K; Mohamad Sukri MZ; Norhisham SN; Ramly N; Robert F; Rosli NR; Suppiah J; Thayan R
EPI_ISL_12628102, EPI_ISL_12628108, EPI_ISL_12628110, EPI_ISL_12628111, EPI_ISL_12628112, EPI_ISL_12628113, EPI_ISL_12628119, EPI_ISL_12628120, EPI_ISL_12628121, EPI_ISL_12628122, EPI_ISL_12628129			
see above	PKD JOHOR BAHRU	UKM Medical Molecular Biology Institute (UMBI)	Khairun Nur Abd Ghafar; Mira Farzana Mohamad Mokhtar; Muhiddin Ishak; Nor Azila Muhammad Azami; Nur Alyaa Afifah Md Shahri; Nurul Syakima Ab Mutalib; Rahman Jamal; Ryia Illani Mohd Yunos; Siti Nur Hasanah Mohd Yusuf; Zahirrah Begam Mohamed Rasheed
EPI_ISL_12628150, EPI_ISL_12628151, EPI_ISL_12628152	PKD KULAI	UKM Medical Molecular Biology Institute (UMBI)	Khairun Nur Abd Ghafar; Mira Farzana Mohamad Mokhtar; Muhiddin Ishak; Nor Azila Muhammad Azami; Nur Alyaa Afifah Md Shahri; Nurul Syakima Ab Mutalib; Rahman Jamal; Ryia Illani Mohd Yunos; Siti Nur Hasanah Mohd Yusuf; Zahirrah Begam Mohamed Rasheed
EPI_ISL_12628153	PKD MUAR	UKM Medical Molecular Biology Institute (UMBI)	Khairun Nur Abd Ghafar; Mira Farzana Mohamad Mokhtar; Muhiddin Ishak; Nor Azila Muhammad Azami; Nur Alyaa Afifah Md Shahri; Nurul Syakima Ab Mutalib; Rahman Jamal; Ryia Illani Mohd Yunos; Siti Nur Hasanah Mohd Yusuf; Zahirrah Begam Mohamed Rasheed
EPI_ISL_12008431	PKD SEREMBAN	UKM Medical Molecular Biology Institute (UMBI)	Khairun Nur Abd Ghafar; Mira Farzana Mohamad Mokhtar; Muhiddin Ishak; Nor Azila Muhammad Azami; Nur Alyaa Afifah Md Shahri; Nurul Syakima Ab Mutalib; Rahman Jamal; Ryia Illani Mohd Yunos; Siti Nur Hasanah Mohd Yusuf; Zahirrah Begam Mohamed Rasheed
EPI_ISL_12322024	PUTRAJAYA DISRICT HEALTH OFFICE	Institute for Medical Research, Infectious Disease Research Centre, National Institutes of Health, Ministry of Health Malaysia	Ahmad FA; Ahmad Fazilah NA; Anasir MI; Azizan MA; Kamel K; Mohamad Sukri MZ; Norhisham SN; Ramly N; Robert F; Rosli NR; Suppiah J; Thayan R
EPI_ISL_12899686, EPI_ISL_12899687	PUTRAJAYA DISTRICT HEALTH OFFICE	Institute for Medical Research, Infectious Disease Research Centre, National Institutes of Health, Ministry of Health Malaysia	Ahmad FA; Ahmad Fazilah NA; Anasir MI; Azizan MA; Kamel K; Mohamad Sukri MZ; Norhisham SN; Ramly N; Robert F; Rosli NR; Suppiah J; Thayan R
EPI_ISL_12980337, EPI_ISL_12980338	Pejabat Kesihatan Daerah Tuaran	Makmal Kesihatan Awam Kota Kinabalu	Erfiana Shamsaddin; Joel Judson Jaimin; Kitty Christhoper Hollip; Maznin Nisah Wajil; Mohd. Nazrin Shah Bin Jamlee; Muhamad Shah Arip; Rashidah Mohamad; Rufina Mohd Yassin; Tan Yee Chee
EPI_ISL_12646711	Public Health Laboratory Ipoh	Public Health Laboratory Ipoh Disease Section	A.Nisha; MH.Chew; N.Azmi; NM.Diyana; R.Rajes; R.Sabrina
EPI_ISL_12631123	Public Health Laboratory, Ipoh	Public Health Laboratory Ipoh Disease Section	A. Nisha; MH.Chew; R. Sabrina; R.Rajes; S. Humairah
EPI_ISL_12510205, EPI_ISL_12510206, EPI_ISL_12510207, EPI_ISL_12510208, EPI_ISL_12510209, EPI_ISL_12510210, EPI_ISL_12510211, EPI_ISL_12510212, EPI_ISL_12510213, EPI_ISL_12510214, EPI_ISL_12510215, EPI_ISL_12510216, EPI_ISL_12510217, EPI_ISL_12510218, EPI_ISL_12510219, EPI_ISL_12510220, EPI_ISL_12510221, EPI_ISL_12510222, EPI_ISL_12510223, EPI_ISL_12510224, EPI_ISL_12510225, EPI_ISL_12510226, EPI_ISL_12510227, EPI_ISL_12510228, EPI_ISL_12510229, EPI_ISL_12510230			
see above	Rejang Medical Centre (Sibu)	Institute of Health and Community Medicine	Chan Chia Jui; David Perera; Ooi Mong How; Wong Jyn Shan
EPI_ISL_12665237, EPI_ISL_12665238, EPI_ISL_12665239, EPI_ISL_12665240, EPI_ISL_12665241	SULTANAH AMINAH HOSPITAL	Institute for Medical Research, Infectious Disease Research Centre, National Institutes of Health, Ministry of Health Malaysia	Ahmad FA; Ahmad Fazilah NA; Anasir MI; Azizan MA; Kamel K; Mohamad Sukri MZ; Norhisham SN; Ramly N; Robert F; Rosli NR; Suppiah J; Thayan R
EPI_ISL_12665243, EPI_ISL_12665244, EPI_ISL_12665245, EPI_ISL_12665246, EPI_ISL_12665247	SULTANAH NUR ZAHIRAH HOSPITAL	Institute for Medical Research, Infectious Disease Research Centre, National Institutes of Health, Ministry of Health Malaysia	Ahmad FA; Ahmad Fazilah NA; Anasir MI; Azizan MA; Kamel K; Mohamad Sukri MZ; Norhisham SN; Ramly N; Robert F; Rosli NR; Suppiah J; Thayan R

EPI_ISL_12099680, EPI_ISL_12099681, EPI_ISL_12099682, EPI_ISL_12510178, EPI_ISL_12510179, EPI_ISL_12510180, EPI_ISL_12510181			
see above	Sarawak General Hospital (Kuching)	Institute of Health and Community Medicine	Chan Chia Jui; Chua Hock Hin; David Perera; Ooi Mong How; Tonnii Sia Loong Loong
EPI_ISL_12099713, EPI_ISL_12099714, EPI_ISL_12099715, EPI_ISL_12099716, EPI_ISL_12099717, EPI_ISL_12099718, EPI_ISL_12099719, EPI_ISL_12510312, EPI_ISL_12510319, EPI_ISL_12510321, EPI_ISL_12510325, EPI_ISL_12510329, EPI_ISL_12510336, EPI_ISL_12510365, EPI_ISL_12510387			
see above	Sibu Hospital, PCR lab (Sibu, Sarawak)	Institute of Health and Community Medicine	Chan Chia Jui; Chua Hock Hin; David Perera; Ooi Mong How; Tonnii Sia Loong Loong
EPI_ISL_11900871, EPI_ISL_11900872, EPI_ISL_11900873, EPI_ISL_11900874, EPI_ISL_11900875, EPI_ISL_11900876, EPI_ISL_11900877, EPI_ISL_11900878, EPI_ISL_11900879, EPI_ISL_11900880, EPI_ISL_11900881, EPI_ISL_11900882, EPI_ISL_11900883, EPI_ISL_11900884, EPI_ISL_11900885, EPI_ISL_11900886, EPI_ISL_11900887, EPI_ISL_11900888, EPI_ISL_11900889			
see above	Synapse Sdn. Bhd.	Universiti Putra Malaysia (UPM)	Choo Yee Yu; Hui Jen Soe; Hui Yee Chee; Nancy Woan Charn Liew; Narcisse Joseph; Nurulfiza Mat Isa; Rachna Kairon; Sie Yeng Wong; Syafinaz Amin-Nordin; Zunita Zakaria
EPI_ISL_12899677	TENGKU AMPAUN RAHIMAH HOSPITAL	Institute for Medical Research, Infectious Disease Research Centre, National Institutes of Health, Ministry of Health Malaysia	Ahmad FA; Ahmad Fazilah NA; Anasir MI; Azizan MA; Kamel K; Mohamad Sukri MZ; Norhisham SN; Ramly N; Robert F; Rosli NR; Suppiah J; Thayan R
EPI_ISL_12321882, EPI_ISL_12321883, EPI_ISL_12321884, EPI_ISL_12321886, EPI_ISL_12321902, EPI_ISL_12899684	TENGKU AMPUAN RAHIMAH HOSPITAL	Institute for Medical Research, Infectious Disease Research Centre, National Institutes of Health, Ministry of Health Malaysia	Ahmad FA; Ahmad Fazilah NA; Anasir MI; Azizan MA; Kamel K; Mohamad Sukri MZ; Norhisham SN; Ramly N; Robert F; Rosli NR; Suppiah J; Thayan R
EPI_ISL_12321903	TUNKU AZIZAH HOSPITAL	Institute for Medical Research, Infectious Disease Research Centre, National Institutes of Health, Ministry of Health Malaysia	Ahmad FA; Ahmad Fazilah NA; Anasir MI; Azizan MA; Kamel K; Mohamad Sukri MZ; Norhisham SN; Ramly N; Robert F; Rosli NR; Suppiah J; Thayan R
EPI_ISL_12560261, EPI_ISL_12560262, EPI_ISL_12560263, EPI_ISL_12560264, EPI_ISL_12560265, EPI_ISL_12560266, EPI_ISL_12560267			
see above	UMMC	Department of Medical Microbiology, Faculty of Medicine, University of Malaya; University of Malaya Medical Centre	I-Ching SAM; Jolene Yin Ling FU; Omar Khalilur Rahman; Yee Von Lee; Yoke Fun Chan

We gratefully acknowledge the following Authors from the Originating laboratories responsible for obtaining the specimens, as well as the Submitting laboratories where the genome data were generated and shared via GISAID, on which this research is based.

All Submitters of data may be contacted directly via www.gisaid.org

Authors are sorted alphabetically.

Acknowledgement EPI_SET Identifier: EPI_SET_20220603dp

Accession ID	Originating Laboratory	Submitting Laboratory	Authors
EPI_ISL_12321900, EPI_ISL_12749684, EPI_ISL_12899671	AMPANG HOSPITAL	Institute for Medical Research, Infectious Disease Research Centre, National Institutes of Health, Ministry of Health Malaysia	Ahmad FA; Ahmad Fazilah NA; Anasir MI; Azizan MA; Kamel K; Mohamad Sukri MZ; Norhisham SN; Ramly N; Robert F; Rosli NR; Suppiah J; Thayan R
EPI_ISL_12972574, EPI_ISL_12972575, EPI_ISL_12972576, EPI_ISL_12972577, EPI_ISL_12972578, EPI_ISL_12972579, EPI_ISL_12972580, EPI_ISL_12972581, EPI_ISL_12972582, EPI_ISL_12983201, EPI_ISL_12983202, EPI_ISL_12983203, EPI_ISL_12983204, EPI_ISL_12983205			
see above	BP Healthcare Group	Institute for Medical Research, Infectious Disease Research Centre, National Institutes of Health, Ministry of Health Malaysia	Anasir MI; G.Adypatti NM; Jamaluddin MS; Kalyanasundram J; Kamel K; MatRahim N; Nawi MH; Suib FA; Suppiah J; Thayan R
EPI_ISL_12510074, EPI_ISL_12510080, EPI_ISL_12510101, EPI_ISL_12510131, EPI_ISL_12510134, EPI_ISL_12510142, EPI_ISL_12510436, EPI_ISL_12510437, EPI_ISL_12510438, EPI_ISL_12510439			
see above	Bintulu Hospital PCR Lab, Bintulu	Institute of Health and Community Medicine	Chan Chia Jui; Chien Su Lin; Chua Hock Hin; David Perera; Ooi Mong How; Tonnii Sia Loong Loong
EPI_ISL_12510309, EPI_ISL_12510314, EPI_ISL_12510315, EPI_ISL_12510316, EPI_ISL_12510317, EPI_ISL_12510318, EPI_ISL_12510320, EPI_ISL_12510322, EPI_ISL_12510323, EPI_ISL_12510324, EPI_ISL_12510326			
see above	Bintulu Medical Centre (Bintulu)	Institute of Health and Community Medicine	Chan Chia Jui; David Perera; Ooi Mong How; Wong Jyn Shan
EPI_ISL_12510255, EPI_ISL_12510256, EPI_ISL_12510257, EPI_ISL_12510259	Borneo Medical Centre (Kuching)	Institute of Health and Community Medicine	Chan Chia Jui; David Perera; Ooi Mong How; Wong Jyn Shan
EPI_ISL_12510100, EPI_ISL_12510102, EPI_ISL_12510103, EPI_ISL_12510104, EPI_ISL_12510105, EPI_ISL_12510106, EPI_ISL_12510107, EPI_ISL_12510108, EPI_ISL_12510109, EPI_ISL_12510110, EPI_ISL_12510251, EPI_ISL_12510253, EPI_ISL_12510254, EPI_ISL_12510258, EPI_ISL_12510260, EPI_ISL_12510261, EPI_ISL_12510262, EPI_ISL_12510270, EPI_ISL_12510280, EPI_ISL_12510281, EPI_ISL_12510282, EPI_ISL_12510285, EPI_ISL_12510290, EPI_ISL_12510292, EPI_ISL_12510293, EPI_ISL_12510294, EPI_ISL_12510297, EPI_ISL_12510299, EPI_ISL_12510308, EPI_ISL_12510311			
see above	Gribbles Diagnostic Lab @Timberland Medical Centre, Kuching	Institute of Health and Community Medicine	Chan Chia Jui; Chua Hock Hin; David Perera; Liew Chin Chin J; Ooi Mong How; Tonnii Sia Loong Loong; Wong Jyn Shan
EPI_ISL_12628133, EPI_ISL_12628139	HOSPITAL MELAKA	UKM Medical Molecular Biology Institute (UMBI)	Khairun Nur Abd Ghafar; Mira Farzana Mohamad Mokhtar; Muhiddin Ishak; Nor Azila Muhammad Azami; Nur Alyaa Afifah Md Shahri; Nurul Syakima Ab Mutalib; Rahman Jamal; Ryia Illani Mohd Yunos; Siti Nur Hasanah Mohd Yusuf; Zahirrah Begam Mohamed Rasheed
EPI_ISL_13047121, EPI_ISL_13047122, EPI_ISL_13047123	HOSPITAL RAJA PEREMPUAN ZAINAB II, KOTA BHARU	MOLECULAR RESEARCH LABORATORY	Abdul Haris bin Muhammad; Ahmad Sukari Bin Halim; Alexander Chong Shu Chien; Azian Harun; Chan Yean Yean; Chua Wei Chuan; Farahana binti Mohamed; Kirnpal Kaur Banga Singh; Lau Nyok Sean; Lee Lih Huey; Lim Shu Yong; Maizun binti Mohd Zain; Mohd Nadzri Abu Yazid; Mohd Zulkifli Salleh; Muhammad Azamuddeen bin Mohammad Nasir; Muhammad Fazli bin Khalid; Muhammad Nashrul Farhan Samsudin; Muhammad Zarul Hanifah Bin Md Zoqratt; Nik Zuraina binti Nik Mohd Noor; Noor Hafizan binti Mat Salleh; Nurfadhlina Musa; Qasim Ayub; Rosline Hassan; Sadequr Rahman; Syahida binti Omar; Wan Mohd Zahiruddin Wan Mohammad; Wardah Yusof; Zaini bin Hussin; Zakuan Zainy Deris
EPI_ISL_13047114	HOSPITAL SULTAN ISMAIL PETRA, KUALA KRAI, KELANTAN	MOLECULAR RESEARCH LABORATORY	Abdul Haris bin Muhammad; Ahmad Sukari Bin Halim; Alexander Chong Shu Chien; Azian Harun; Chan Yean Yean; Chua Wei Chuan; Farahana binti Mohamed; Kirnpal Kaur Banga Singh; Lau Nyok Sean; Lee Lih Huey; Lim Shu Yong; Maizun binti Mohd Zain; Mohd Nadzri Abu Yazid; Mohd Zulkifli Salleh; Muhammad Azamuddeen bin Mohammad Nasir; Muhammad Fazli bin Khalid; Muhammad Nashrul Farhan Samsudin; Muhammad Zarul Hanifah Bin Md Zoqratt; Nik Zuraina binti Nik Mohd Noor; Noor Hafizan binti Mat Salleh; Nurfadhlina Musa; Qasim Ayub; Rosline Hassan; Sadequr Rahman; Syahida binti Omar; Wan Mohd Zahiruddin Wan Mohammad; Wardah Yusof; Zaini bin Hussin; Zakuan Zainy Deris
EPI_ISL_12771323, EPI_ISL_12771324, EPI_ISL_12771325, EPI_ISL_12771326, EPI_ISL_12771327, EPI_ISL_12771328, EPI_ISL_12771329, EPI_ISL_12771330, EPI_ISL_12771331, EPI_ISL_12771332, EPI_ISL_12771333			
see above	HRPB	iPROMISE, UiTM	Ariza Adnan; Fadzilah Mohd Nor; Lim Wai Feng; Mohd Asif Mohd Sukri; Mohd Nur Fakhruzzaman Noorizhab; Mohd Zaki Salleh; Sazzli Shahlan Kassim; Siti Farah Alwani Mohd Nawi; Siti Hamimah Sheikh Abdul Kadir; Teh Lay Kek; Wang Seok Mui
EPI_ISL_12771336	Hospital Kepala Batas	iPROMISE, UiTM	Ariza Adnan; Fadzilah Mohd Nor; Lim Wai Feng; Mohd Asif Mohd Sukri; Mohd Nur Fakhruzzaman Noorizhab; Mohd Zaki Salleh; Sazzli Shahlan Kassim; Siti Farah Alwani Mohd Nawi; Siti Hamimah Sheikh Abdul Kadir; Teh Lay Kek; Wang Seok Mui
EPI_ISL_12980343	Hospital Kuala Penyu	Makmal Kesihatan Awam Kota Kinabalu	Erfiana Shamsaddin; Joel Judson Jaimin; Kitty Christhoper Hollip; Maznin Nisah Wajil; Mohd. Nazrin Shah Bin Jamlee; Muhamad Shah Arip; Rashidah Mohamad; Rufina Mohd Yassin; Tan Yee Chee
EPI_ISL_12708548, EPI_ISL_12708549, EPI_ISL_12708550	Hospital Raja Perempuan Zainab II, Kota Bharu	Molecular Research Laboratory	Abdul Haris bin Muhammad; Ahmad Sukari Bin Halim; Alexander Chong Shu Chien; Asraihan Che Abdul Malik; Azian Harun; Chan Yean Yean; Chua Wei Chuan; Farahana binti Mohamed; Kirnpal Kaur Banga Singh; Lau Nyok Sean; Lee Lih Huey; Lim Shu Yong; Maizun binti Mohd Zain; Mera Edora Binti Abdul Manap; Mohd Iman hafiz Bin Ibrahim; Mohd Nadzri Abu Yazid; Mohd Zulkifli Salleh; Muhamad Khairul Amirin Bin Zulkifli; Muhammad Azamuddeen bin Mohammad Nasir; Muhammad Fazli bin Khalid; Muhammad Nashrul Farhan Samsudin; Muhammad Zarul Hanifah Bin Md Zoqratt; Nik Zuraina binti Nik Mohd Noor; Noor Hafizan binti Mat Salleh; Nor Amizara Binti Azami; Nor Suhana Binti Mohd Satar; Nur Syuhada Binti Abdul Rahim; Nur-Leem Binti Murshid; Nurfadhlina Musa; Qasim Ayub; Rosline Hassan; Sadequr Rahman; Siti Nur Aisyah Binti Mohamad Sham; Syahida binti Omar; Wan Mohd Zahiruddin Wan Mohammad; Wardah Yusof; Zaini bin Hussin; Zakuan Zainy Deris
EPI_ISL_13047105	KETEREH HEALTH CLINIC	MOLECULAR RESEARCH LABORATORY	Abdul Haris bin Muhammad; Ahmad Sukari Bin Halim; Alexander Chong Shu Chien; Azian Harun; Chan Yean Yean; Chua Wei Chuan; Farahana binti Mohamed; Kirnpal Kaur Banga Singh; Lau Nyok Sean; Lee Lih Huey; Lim Shu Yong; Maizun binti Mohd Zain; Mohd Nadzri Abu Yazid; Mohd Zulkifli Salleh; Muhammad Azamuddeen bin Mohammad Nasir; Muhammad Fazli bin Khalid; Muhammad Nashrul Farhan Samsudin; Muhammad Zarul Hanifah Bin Md Zoqratt; Nik Zuraina binti Nik Mohd Noor; Noor Hafizan binti Mat Salleh; Nurfadhlina Musa; Qasim Ayub; Rosline Hassan; Sadequr Rahman; Syahida binti Omar; Wan Mohd Zahiruddin Wan Mohammad; Wardah Yusof; Zaini bin Hussin; Zakuan Zainy Deris
EPI_ISL_13047095, EPI_ISL_13047096, EPI_ISL_13047097, EPI_ISL_13047098, EPI_ISL_13047099, EPI_ISL_13047100, EPI_ISL_13047101, EPI_ISL_13047102, EPI_ISL_13047103, EPI_ISL_13047104, EPI_ISL_13047117			
see above	KOTA BHARU DISTRICT HEALTH OFFICE	MOLECULAR RESEARCH LABORATORY	Abdul Haris bin Muhammad; Ahmad Sukari Bin Halim; Alexander Chong Shu Chien; Azian Harun; Chan Yean Yean; Chua Wei Chuan; Farahana binti Mohamed; Kirnpal Kaur Banga Singh; Lau Nyok Sean; Lee Lih Huey; Lim Shu Yong; Maizun binti Mohd Zain; Mohd Nadzri Abu Yazid; Mohd Zulkifli Salleh; Muhammad Azamuddeen bin Mohammad Nasir; Muhammad Fazli bin Khalid; Muhammad Nashrul Farhan Samsudin; Muhammad Zarul Hanifah Bin Md Zoqratt; Nik Zuraina binti Nik Mohd Noor; Noor Hafizan binti Mat Salleh; Nurfadhlina Musa; Qasim Ayub; Rosline Hassan; Sadequr Rahman; Syahida binti Omar; Wan Mohd Zahiruddin Wan Mohammad; Wardah Yusof; Zaini bin Hussin; Zakuan Zainy Deris
EPI_ISL_12980349	Klinik Kesihatan Ibu Dan Anak Harington	Makmal Kesihatan Awam Kota Kinabalu	Erfiana Shamsaddin; Joel Judson Jaimin; Kitty Christhoper Hollip; Maznin Nisah Wajil; Mohd. Nazrin Shah Bin Jamlee; Muhamad Shah Arip; Rashidah Mohamad; Rufina Mohd Yassin; Tan Yee Chee
EPI_ISL_12980339, EPI_ISL_12980340, EPI_ISL_12980341, EPI_ISL_12980342, EPI_ISL_12980345, EPI_ISL_12980346, EPI_ISL_12980347, EPI_ISL_12980348, EPI_ISL_12980350			
see above	Klinik Kesihatan Luyang	Makmal Kesihatan Awam Kota Kinabalu	Erfiana Shamsaddin; Joel Judson Jaimin; Kitty Christhoper Hollip; Maznin Nisah Wajil; Mohd. Nazrin Shah Bin Jamlee; Muhamad Shah Arip; Rashidah Mohamad; Rufina Mohd Yassin; Tan Yee Chee
EPI_ISL_12980344	Klinik Kesihatan Penampang	Makmal Kesihatan Awam Kota Kinabalu	Erfiana Shamsaddin; Joel Judson Jaimin; Kitty Christhoper Hollip; Maznin Nisah Wajil; Mohd. Nazrin Shah Bin Jamlee; Muhamad Shah Arip; Rashidah Mohamad; Rufina Mohd Yassin; Tan Yee Chee
EPI_ISL_12708558	Kota Bharu Public Health Laboratory	Molecular Research Laboratory	Abdul Haris bin Muhammad; Ahmad Sukari Bin Halim; Alexander Chong Shu Chien; Asraihan Che Abdul Malik; Azian Harun; Chan Yean Yean; Chua Wei Chuan; Farahana binti Mohamed; Kirnpal Kaur Banga Singh; Lau Nyok Sean; Lee Lih Huey; Lim Shu Yong; Maizun binti Mohd Zain; Mera Edora Binti Abdul Manap; Mohd Iman hafiz Bin Ibrahim; Mohd Nadzri Abu Yazid; Mohd Zulkifli Salleh; Muhamad Khairul Amirin Bin Zulkifli; Muhammad Azamuddeen bin Mohammad Nasir; Muhammad Fazli bin Khalid; Muhammad Nashrul Farhan Samsudin; Muhammad Zarul Hanifah Bin Md Zoqratt; Nik Zuraina binti Nik Mohd Noor; Noor Hafizan binti Mat Salleh; Nor Amizara Binti Azami; Nor Suhana Binti Mohd Satar; Nur Syuhada Binti Abdul Rahim; Nur-Leem Binti Murshid; Nurfadhlina Musa; Qasim Ayub; Rosline Hassan; Sadequr Rahman; Siti Nur Aisyah Binti Mohamad Sham; Syahida binti Omar; Wan Mohd Zahiruddin Wan Mohammad; Wardah Yusof; Zaini bin Hussin; Zakuan Zainy Deris
EPI_ISL_12401871, EPI_ISL_12911832, EPI_ISL_12911833, EPI_ISL_12911834, EPI_ISL_12911835, EPI_ISL_12911836, EPI_ISL_12911837, EPI_ISL_12911838, EPI_ISL_12911839, EPI_ISL_12911840, EPI_ISL_12911841, EPI_ISL_12911842, EPI_ISL_12911843, EPI_ISL_12911844, EPI_ISL_12911845, EPI_ISL_12911846, EPI_ISL_12911847, EPI_ISL_12911848, EPI_ISL_12911849, EPI_ISL_12911850, EPI_ISL_12911851, EPI_ISL_12911852, EPI_ISL_12911853, EPI_ISL_12911854, EPI_ISL_12911855, EPI_ISL_12911856, EPI_ISL_12911857, EPI_ISL_12911858, EPI_ISL_12911859, EPI_ISL_12911860, EPI_ISL_12911861, EPI_ISL_12911862, EPI_ISL_12911863, EPI_ISL_12911864, EPI_ISL_12911865, EPI_ISL_12911866, EPI_ISL_12911867, EPI_ISL_12911868, EPI_ISL_12911869, EPI_ISL_12911870, EPI_ISL_12911871, EPI_ISL_12911872, EPI_ISL_12911873, EPI_ISL_12911874, EPI_ISL_12911875, EPI_ISL_12911876, EPI_ISL_12911877, EPI_ISL_12911878, EPI_ISL_12911879, EPI_ISL_12911880, EPI_ISL_12911881, EPI_ISL_12911882, EPI_ISL_12911883, EPI_ISL_12911884, EPI_ISL_12911885, EPI_ISL_12911886, EPI_ISL_12911887, EPI_ISL_12911888, EPI_ISL_12911889, EPI_ISL_12918939, EPI_ISL_12918940, EPI_ISL_12918941, EPI_ISL_12918942, EPI_ISL_12918943, EPI_ISL_12918944, EPI_ISL_12918945, EPI_ISL_12918946, EPI_ISL_12918947, EPI_ISL_12918948, EPI_ISL_12918949, EPI_ISL_12918950, EPI_ISL_12918951, EPI_ISL_12918952, EPI_ISL_12918953, EPI_ISL_12918954, EPI_ISL_12918955, EPI_ISL_12918956, EPI_ISL_12918957, EPI_ISL_12918958, EPI_ISL_12918959, EPI_ISL_12918960, EPI_ISL_12918961, EPI_ISL_12918964, EPI_ISL_12918965, EPI_ISL_12918966, EPI_ISL_12918967, EPI_ISL_12918968, EPI_ISL_12918969, EPI_ISL_12918970, EPI_ISL_12918971, EPI_ISL_12918972, EPI_ISL_12918973, EPI_ISL_12918974, EPI_ISL_12918975, EPI_ISL_12918976, EPI_ISL_12918977, EPI_ISL_12918978, EPI_ISL_12918979, EPI_ISL_12918980, EPI_ISL_12918981, EPI_ISL_12918982, EPI_ISL_12918983, EPI_ISL_12918984, EPI_ISL_12918985, EPI_ISL_12918986, EPI_ISL_12918987, EPI_ISL_12918988, EPI_ISL_12918989, EPI_ISL_12918990, EPI_ISL_12918991, EPI_ISL_12918992, EPI_ISL_12918993, EPI_ISL_12918994, EPI_ISL_12918995, EPI_ISL_12918996, EPI_ISL_12918997, EPI_ISL_12918998, EPI_ISL_12918999, EPI_ISL_12919000, EPI_ISL_12919001, EPI_ISL_12919002, EPI_ISL_12919003, EPI_ISL_12919004, EPI_ISL_12919005, EPI_ISL_12919006, EPI_ISL_12919007, EPI_ISL_12919008, EPI_ISL_12919009, EPI_ISL_12919010, EPI_ISL_12919011, EPI_ISL_12919012, EPI_ISL_12919013, EPI_ISL_12919014, EPI_ISL_12919015, EPI_ISL_12919016, EPI_ISL_12919017, EPI_ISL_12919018, EPI_ISL_12919019, EPI_ISL_12919020, EPI_ISL_12919021, EPI_ISL_12919022, EPI_ISL_12919023, EPI_ISL_12919024, EPI_ISL_12919025, EPI_ISL_12919026, EPI_ISL_12919027, EPI_ISL_12919056, EPI_ISL_12919057, EPI_ISL_12919058, EPI_ISL_12919059, EPI_ISL_12919060, EPI_ISL_12919061, EPI_ISL_12919062, EPI_ISL_12919063, EPI_ISL_12919064, EPI_ISL_12919065, EPI_ISL_12919066, EPI_ISL_12919067, EPI_ISL_12919068, EPI_ISL_12919069, EPI_ISL_12919070, EPI_ISL_12919071, EPI_ISL_12919072, EPI_ISL_12919073, EPI_ISL_12919074, EPI_ISL_12919075, EPI_ISL_12919076, EPI_ISL_12919077, EPI_ISL_12919078, EPI_ISL_12919079, EPI_ISL_12919080, EPI_ISL_12919081, EPI_ISL_12919082, EPI_ISL_12919083, EPI_ISL_12919085, EPI_ISL_12919086, EPI_ISL_12919087, EPI_ISL_12919088, EPI_ISL_12919089, EPI_ISL_12919090, EPI_ISL_12919091, EPI_ISL_12919092, EPI_ISL_12919093, EPI_ISL_12919094, EPI_ISL_12919095, EPI_ISL_12919096, EPI_ISL_12919097, EPI_ISL_12919098, EPI_ISL_12919099, EPI_ISL_12919100, EPI_ISL_12919101, EPI_ISL_12919102, EPI_ISL_12919103, EPI_ISL_12919104, EPI_ISL_12919105, EPI_ISL_12919106, EPI_ISL_12919107, EPI_ISL_12919108, EPI_ISL_12919109, EPI_ISL_12919110, EPI_ISL_12919111, EPI_ISL_12919112, EPI_ISL_12919113, EPI_ISL_12919114, EPI_ISL_12919115, EPI_ISL_12919116, EPI_ISL_12919117, EPI_ISL_12919118, EPI_ISL_12919253, EPI_ISL_12919255, EPI_ISL_12919256, EPI_ISL_12919257, EPI_ISL_12919258, EPI_ISL_12942660, EPI_ISL_12942661, EPI_ISL_12942662, EPI_ISL_12942663, EPI_ISL_12942664, EPI_ISL_12942665, EPI_ISL_12942666, EPI_ISL_12942667, EPI_ISL_12942668, EPI_ISL_12942669, EPI_ISL_12942670, EPI_ISL_12942671, EPI_ISL_12942672, EPI_ISL_12942673, EPI_ISL_12942699, EPI_ISL_12942700, EPI_ISL_12942701, EPI_ISL_12942702, EPI_ISL_12942703, EPI_ISL_12942704, EPI_ISL_12942705, EPI_ISL_12942706, EPI_ISL_12942707, EPI_ISL_12942708, EPI_ISL_12942709, EPI_ISL_12942710, EPI_ISL_12942711, EPI_ISL_12942712, EPI_ISL_12942713, EPI_ISL_12942714, EPI_ISL_12942715, EPI_ISL_12942716, EPI_ISL_12942717, EPI_ISL_12942718, EPI_ISL_12942719, EPI_ISL_12942720, EPI_ISL_12942721, EPI_ISL_12942722, EPI_ISL_12942723, EPI_ISL_12942724, EPI_ISL_12942725, EPI_ISL_12942743, EPI_ISL_12942744, EPI_ISL_12942745, EPI_ISL_12942746, EPI_ISL_12942747, EPI_ISL_12942748, EPI_ISL_12942749, EPI_ISL_12942750, EPI_ISL_12942751, EPI_ISL_12970791, EPI_ISL_12991256, EPI_ISL_12999583, EPI_ISL_12999584, EPI_ISL_12999585, EPI_ISL_12999586, EPI_ISL_12999587, EPI_ISL_12999588, EPI_ISL_12999589, EPI_ISL_12999590, EPI_ISL_12999591, EPI_ISL_12999592, EPI_ISL_12999593, EPI_ISL_12999594, EPI_ISL_12999595, EPI_ISL_12999596, EPI_ISL_12999597, EPI_ISL_12999598, EPI_ISL_12999599, EPI_ISL_12999600, EPI_ISL_12999601, EPI_ISL_12999602, EPI_ISL_12999603, EPI_ISL_12999604, EPI_ISL_12999605, EPI_ISL_12999606, EPI_ISL_12999607, EPI_ISL_12999608, EPI_ISL_12999610, EPI_ISL_12999611, EPI_ISL_12999612, EPI_ISL_12999613, EPI_ISL_12999614, EPI_ISL_12999615, EPI_ISL_13018613, EPI_ISL_13018616, EPI_ISL_13040816, EPI_ISL_13040817, EPI_ISL_13040818, EPI_ISL_13040819, EPI_ISL_13040820, EPI_ISL_13040821, EPI_ISL_13040822, EPI_ISL_13040823, EPI_ISL_13040824, EPI_ISL_13040825			
see above	Kuala Lumpur International Airport	Institute for Medical Research, Infectious Disease Research Centre, National Institutes of Health, Ministry of	Anasir MI; G.Adypatti NM; Jamaluddin MS; Kalyanasundram J; Kamel K; MatRahim N; Nawi MH; Suib FA; Suppiah J; Thayan R

		Health Malaysia	
EPI_ISL_12628134, EPI_ISL_12628135, EPI_ISL_12628147, EPI_ISL_12628148	MAKMAL KESIHATAN JOHOR	UKM Medical Molecular Biology Institute (UMBI)	Khairun Nur Abd Ghafar; Mira Farzana Mohamad Mokhtar; Muhiddin Ishak; Nor Azila Muhammad Azami; Nur Alyaa Afifah Md Shahri; Nurul Syakima Ab Mutalib; Rahman Jamal; Ryia Illani Mohd Yunos; Siti Nur Hasanah Mohd Yusuf; Zahirrah Begam Mohamed Rasheed
EPI_ISL_12429739, EPI_ISL_12429740, EPI_ISL_12429741, EPI_ISL_12429742, EPI_ISL_12429743, EPI_ISL_12429744, EPI_ISL_12728843, EPI_ISL_12728847, EPI_ISL_12728848, EPI_ISL_12728849			
see above	National Public Health Laboratory	National Public Health Laboratory	Kamal Hisham Bin Kamarul Zaman; Maznin Nisah Binti Wajil; Mohd Asri Bin Yamin; Muhd Hashim Chew; Noriah Binti Mohd Yusof; Rehan Shuhada Binti Abu Bakar; Selvanesan A/L Sengol; Yu Kie A/P Chem
EPI_ISL_12628149	PKD KULAI	UKM Medical Molecular Biology Institute (UMBI)	Khairun Nur Abd Ghafar; Mira Farzana Mohamad Mokhtar; Muhiddin Ishak; Nor Azila Muhammad Azami; Nur Alyaa Afifah Md Shahri; Nurul Syakima Ab Mutalib; Rahman Jamal; Ryia Illani Mohd Yunos; Siti Nur Hasanah Mohd Yusuf; Zahirrah Begam Mohamed Rasheed
EPI_ISL_12628140, EPI_ISL_12628141	PKD MUAR	UKM Medical Molecular Biology Institute (UMBI)	Khairun Nur Abd Ghafar; Mira Farzana Mohamad Mokhtar; Muhiddin Ishak; Nor Azila Muhammad Azami; Nur Alyaa Afifah Md Shahri; Nurul Syakima Ab Mutalib; Rahman Jamal; Ryia Illani Mohd Yunos; Siti Nur Hasanah Mohd Yusuf; Zahirrah Begam Mohamed Rasheed
EPI_ISL_12708557	Pengkalan Chepa Health Clinic	Molecular Research Laboratory	Abdul Haris bin Muhammad; Ahmad Sukari Bin Halim; Alexander Chong Shu Chien; Asraihan Che Abdul Malik; Azian Harun; Chan Yean Yean; Chua Wei Chuan; Farahana binti Mohamed; Kirnpal Kaur Banga Singh; Lau Nyok Sean; Lee Lih Huey; Lim Shu Yong; Maizun binti Mohd Zain; Mera Edora Binti Abdul Manap; Mohd Iman hafiz Bin Ibrahim; Mohd Nadzri Abu Yazid; Mohd Zulkifli Salleh; Muhamad Khairul Amirin Bin Zulkifli; Muhammad Azamuddeen bin Mohammad Nasir; Muhammad Fazli bin Khalid; Muhammad Nashrul Farhan Samsudin; Muhammad Zarul Hanifah Bin Md Zoqratt; Nik Zuraina binti Nik Mohd Noor; Noor Hafizan binti Mat Salleh; Nor Amizara Binti Azami; Nor Suhana Binti Mohd Satar; Nur Syuhada Binti Abdul Rahim; Nur-Leem Binti Murshid; Nurfadhlina Musa; Qasim Ayub; Rosline Hassan; Sadequr Rahman; Siti Nur Aisyah Binti Mohamad Sham; Syahida binti Omar; Wan Mohd Zahiruddin Wan Mohammad; Wardah Yusof; Zaini bin Hussin; Zakuan Zainy Deris
EPI_ISL_12510231, EPI_ISL_12510232, EPI_ISL_12510233, EPI_ISL_12510234, EPI_ISL_12510235, EPI_ISL_12510236, EPI_ISL_12510237, EPI_ISL_12510238			
see above	Rejang Medical Centre (Sibu)	Institute of Health and Community Medicine	Chan Chia Jui; David Perera; Ooi Mong How; Wong Jyn Shan
EPI_ISL_12665242	SULTANAH AMINAH HOSPITAL	Institute for Medical Research, Infectious Disease Research Centre, National Institutes of Health, Ministry of Health Malaysia	Ahmad FA; Ahmad Fazilah NA; Anasir MI; Azizan MA; Kamel K; Mohamad Sukri MZ; Norhisham SN; Ramly N; Robert F; Rosli NR; Suppiah J; Thayan R
EPI_ISL_12510182, EPI_ISL_12510183, EPI_ISL_12510327, EPI_ISL_12510328, EPI_ISL_12510330, EPI_ISL_12510331, EPI_ISL_12510332			
see above	Sarawak General Hospital (Kuching)	Institute of Health and Community Medicine	Chan Chia Jui; Chua Hock Hin; David Perera; Ooi Mong How
EPI_ISL_12510158, EPI_ISL_12510159, EPI_ISL_12510160, EPI_ISL_12510161, EPI_ISL_12510162, EPI_ISL_12510163, EPI_ISL_12510164, EPI_ISL_12510165, EPI_ISL_12510166, EPI_ISL_12510168, EPI_ISL_12510173, EPI_ISL_12510201, EPI_ISL_12510244, EPI_ISL_12510247, EPI_ISL_12510249, EPI_ISL_12510250			
see above	Sibu Hospital, PCR lab (Sibu, Sarawak)	Institute of Health and Community Medicine	Chan Chia Jui; Chua Hock Hin; David Perera; Ooi Mong How; Tonnii Sia Loong Loong
EPI_ISL_12771335	Unit Forensik, Hospital Kepala Batas	iPROMISE, UiTM	Ariza Adnan; Fadzilah Mohd Nor; Lim Wai Feng; Mohd Asif Mohd Sukri; Mohd Nur Fakhruzzaman Noorizhab; Mohd Zaki Salleh; Sazzli Shahlan Kassim; Siti Farah Alwani Mohd Nawi; Siti Hamimah Sheikh Abdul Kadir; Teh Lay Kek; Wang Seok Mui

We gratefully acknowledge the following Authors from the Originating laboratories responsible for obtaining the specimens, as well as the Submitting laboratories where the genome data were generated and shared via GISAID, on which this research is based.

All Submitters of data may be contacted directly via www.gisaid.org

Authors are sorted alphabetically.

Accession ID	Originating Laboratory	Submitting Laboratory	Authors
EPI_ISL_12899668, EPI_ISL_12899669	AMPANG HOSPITAL	Institute for Medical Research, Infectious Disease Research Centre, National Institutes of Health, Ministry of Health Malaysia	Ahmad FA; Ahmad Fazilah NA; Anasir MI; Azizan MA; Kamel K; Mohamad Sukri MZ; Norhisham SN; Ramly N; Robert F; Rosli NR; Suppiah J; Thayan R
EPI_ISL_12973113, EPI_ISL_12973114, EPI_ISL_12973115, EPI_ISL_12973116, EPI_ISL_12973117, EPI_ISL_12973118, EPI_ISL_12973119, EPI_ISL_12973120, EPI_ISL_12973121, EPI_ISL_12973122, EPI_ISL_12973123, EPI_ISL_12973124, EPI_ISL_12973125, EPI_ISL_12973126, EPI_ISL_12973127, EPI_ISL_12973128, EPI_ISL_12973129, EPI_ISL_12983178, EPI_ISL_12983179, EPI_ISL_12983180, EPI_ISL_12983181, EPI_ISL_12983182, EPI_ISL_12983183, EPI_ISL_12983184, EPI_ISL_12983185, EPI_ISL_12983186, EPI_ISL_12983206, EPI_ISL_12983207, EPI_ISL_12983208, EPI_ISL_12983209, EPI_ISL_12983210, EPI_ISL_12983211, EPI_ISL_12983212, EPI_ISL_12983213, EPI_ISL_12983214, EPI_ISL_12983215, EPI_ISL_12983216, EPI_ISL_12983217, EPI_ISL_12983218, EPI_ISL_12983219, EPI_ISL_12983220, EPI_ISL_12983221, EPI_ISL_12983222, EPI_ISL_12983223, EPI_ISL_12983224, EPI_ISL_12983225, EPI_ISL_12983226, EPI_ISL_12983227, EPI_ISL_12983228, EPI_ISL_12983229, EPI_ISL_12983230, EPI_ISL_12983231, EPI_ISL_12983232, EPI_ISL_12983233, EPI_ISL_12983234, EPI_ISL_12983235, EPI_ISL_12983236, EPI_ISL_12983237, EPI_ISL_12983238, EPI_ISL_12983239, EPI_ISL_12983240, EPI_ISL_12983241, EPI_ISL_12983242, EPI_ISL_12983243, EPI_ISL_12983244, EPI_ISL_12983245, EPI_ISL_12983246, EPI_ISL_12983247, EPI_ISL_12983248, EPI_ISL_12983249, EPI_ISL_12983250, EPI_ISL_12983251, EPI_ISL_12983252, EPI_ISL_12983253, EPI_ISL_12983254, EPI_ISL_12983255, EPI_ISL_12983256, EPI_ISL_12983257, EPI_ISL_12983258, EPI_ISL_12983259, EPI_ISL_12983260, EPI_ISL_12983261, EPI_ISL_12983262, EPI_ISL_12983263, EPI_ISL_12983264, EPI_ISL_12983265, EPI_ISL_12983266, EPI_ISL_12983267, EPI_ISL_12983268, EPI_ISL_12983269, EPI_ISL_12983270, EPI_ISL_12983271, EPI_ISL_12983272			
see above	BP Healthcare Group	Institute for Medical Research, Infectious Disease Research Centre, National Institutes of Health, Ministry of Health Malaysia	Anasir MI; G.Adypatti NM; Jamaluddin MS; Kalyanasundram J; Kamel K; MatRahim N; Nawi MH; Suib FA; Suppiah J; Thayan R
EPI_ISL_12510440	Bintulu Hospital PCR Lab, Bintulu	Institute of Health and Community Medicine	Chan Chia Jui; Chien Su Lin; Chua Hock Hin; David Perera; Ooi Mong How; Tonnii Sia Loong Loong
EPI_ISL_13047115	HOSPITAL SULTAN ISMAIL PETRA, KUALA KRAI, KELANTAN	MOLECULAR RESEARCH LABORATORY	Abdul Haris bin Muhammad; Ahmad Sukari Bin Halim; Alexander Chong Shu Chien; Azian Harun; Chan Yean Yean; Chua Wei Chuan; Farahana binti Mohamed; Kirnpal Kaur Banga Singh; Lau Nyok Sean; Lee Lih Huey; Lim Shu Yong; Maizun binti Mohd Zain; Mohd Nadzri Abu Yazid; Mohd Zulkifli Salleh; Muhammad Azamuddeen bin Mohammad Nasir; Muhammad Fazli bin Khalid; Muhammad Nashrul Farhan Samsudin; Muhammad Zarul Hanifah Bin Md Zoqratt; Nik Zuraina binti Nik Mohd Noor; Noor Hafizan binti Mat Salleh; Nurfadhlina Musa; Qasim Ayub; Rosline Hassan; Sadequr Rahman; Syahida binti Omar; Wan Mohd Zahiruddin Wan Mohammad; Wardah Yusof; Zaini bin Hussin; Zakuan Zainy Deris
EPI_ISL_12980354, EPI_ISL_13068879	Hospital Kuala Penyu	Makmal Kesihatan Awam Kota Kinabalu	Erfiana Shamsaddin; Joel Judson Jaimin; Kitty Christhoper Hollip; Maznin Nisah Wajil; Mohd. Nazrin Shah Bin Jamlee; Muhamad Shah Arip; Rashidah Mohamad; Rufina Mohd Yassin; Tan Yee Chee
EPI_ISL_12980355	Klinik Kesihatan Ibu Dan Anak Harington	Makmal Kesihatan Awam Kota Kinabalu	Erfiana Shamsaddin; Joel Judson Jaimin; Kitty Christhoper Hollip; Maznin Nisah Wajil; Mohd. Nazrin Shah Bin Jamlee; Muhamad Shah Arip; Rashidah Mohamad; Rufina Mohd Yassin; Tan Yee Chee
EPI_ISL_12980353, EPI_ISL_12980356, EPI_ISL_12980357, EPI_ISL_12980358, EPI_ISL_12980359, EPI_ISL_12980360, EPI_ISL_12980361, EPI_ISL_12980362			
see above	Klinik Kesihatan Luyang	Makmal Kesihatan Awam Kota Kinabalu	Erfiana Shamsaddin; Joel Judson Jaimin; Kitty Christhoper Hollip; Maznin Nisah Wajil; Mohd. Nazrin Shah Bin Jamlee; Muhamad Shah Arip; Rashidah Mohamad; Rufina Mohd Yassin; Tan Yee Chee
EPI_ISL_12942674, EPI_ISL_12942675, EPI_ISL_12942676, EPI_ISL_12942677, EPI_ISL_12942678, EPI_ISL_12942679, EPI_ISL_12942680, EPI_ISL_12942681, EPI_ISL_12942682, EPI_ISL_12942683, EPI_ISL_12942684, EPI_ISL_12942686, EPI_ISL_12942687, EPI_ISL_12942688, EPI_ISL_12942689, EPI_ISL_12942690, EPI_ISL_12942691, EPI_ISL_12942692, EPI_ISL_12942693, EPI_ISL_12942694, EPI_ISL_12942695, EPI_ISL_12942696, EPI_ISL_12942697, EPI_ISL_12942698, EPI_ISL_12942726, EPI_ISL_12942727, EPI_ISL_12942728, EPI_ISL_12942729, EPI_ISL_12942730, EPI_ISL_12942731, EPI_ISL_12942732, EPI_ISL_12942733, EPI_ISL_12942734, EPI_ISL_12942735, EPI_ISL_12942736, EPI_ISL_12942737, EPI_ISL_12942738, EPI_ISL_12942739, EPI_ISL_12942740, EPI_ISL_12942741, EPI_ISL_12999582, EPI_ISL_13018615			
see above	Kuala Lumpur International Airport	Institute for Medical Research, Infectious Disease Research Centre, National Institutes of Health, Ministry of Health Malaysia	Anasir MI; G.Adypatti NM; Jamaluddin MS; Kalyanasundram J; Kamel K; MatRahim N; Nawi MH; Suib FA; Suppiah J; Thayan R
EPI_ISL_12728842, EPI_ISL_12728844, EPI_ISL_12728845, EPI_ISL_12728846, EPI_ISL_13040368, EPI_ISL_13040369, EPI_ISL_13040370, EPI_ISL_13040371, EPI_ISL_13040372, EPI_ISL_13040373, EPI_ISL_13040374, EPI_ISL_13040375, EPI_ISL_13040376, EPI_ISL_13040377, EPI_ISL_13040378, EPI_ISL_13040379, EPI_ISL_13040380, EPI_ISL_13040381, EPI_ISL_13040382, EPI_ISL_13040383, EPI_ISL_13040384, EPI_ISL_13040385, EPI_ISL_13040386, EPI_ISL_13040387, EPI_ISL_13040388, EPI_ISL_13040389, EPI_ISL_13040390, EPI_ISL_13040391			
see above	National Public Health Laboratory	National Public Health Laboratory	Kamal Hisham Bin Kamarul Zaman; Mohd Asri Bin Yamin; Muhamad Syamim Bin Roslan; Muhammad Shahir Bin Ali; Noriah Binti Mohd Yusof; Nur Hazliza Binti Salleh; Rehan Shuhada Binti Abu Bakar; Selvanesan A/L Sengol; Yu Kie A/P Chem
EPI_ISL_12980351, EPI_ISL_12980352	PEJABAT KESIHATAN DAERAH KOTA KINABALU	Makmal Kesihatan Awam Kota Kinabalu	Erfiana Shamsaddin; Joel Judson Jaimin; Kitty Christhoper Hollip; Maznin Nisah Wajil; Mohd. Nazrin Shah Bin Jamlee; Muhamad Shah Arip; Rashidah Mohamad; Rufina Mohd Yassin; Tan Yee Chee
EPI_ISL_12899695	SULTANAH AMINAH HOSPITAL	Institute for Medical Research, Infectious Disease Research Centre, National Institutes of Health, Ministry of Health Malaysia	Ahmad FA; Ahmad Fazilah NA; Anasir MI; Azizan MA; Kamel K; Mohamad Sukri MZ; Norhisham SN; Ramly N; Robert F; Rosli NR; Suppiah J; Thayan R
EPI_ISL_12510333, EPI_ISL_12510441	Sarawak General Hospital (Kuching)	Institute of Health and Community Medicine	Chan Chia Jui; Chua Hock Hin; David Perera; Ooi Mong How
EPI_ISL_12899678	TENGKU AMPAUN RAHIMAH HOSPITAL	Institute for Medical Research, Infectious Disease Research Centre, National Institutes of Health, Ministry of Health Malaysia	Ahmad FA; Ahmad Fazilah NA; Anasir MI; Azizan MA; Kamel K; Mohamad Sukri MZ; Norhisham SN; Ramly N; Robert F; Rosli NR; Suppiah J; Thayan R
EPI_ISL_12899679, EPI_ISL_12899680, EPI_ISL_12899681, EPI_ISL_12899682, EPI_ISL_12899683	TENGKU AMPUAN RAHIMAH HOSPITAL	Institute for Medical Research, Infectious Disease Research Centre, National Institutes of Health, Ministry of Health Malaysia	Ahmad FA; Ahmad Fazilah NA; Anasir MI; Azizan MA; Kamel K; Mohamad Sukri MZ; Norhisham SN; Ramly N; Robert F; Rosli NR; Suppiah J; Thayan R

We gratefully acknowledge the following Authors from the Originating laboratories responsible for obtaining the specimens, as well as the Submitting laboratories where the genome data were generated and shared via GISAID, on which this research is based.

All Submitters of data may be contacted directly via www.gisaid.org

Authors are sorted alphabetically.

Accession ID	Originating Laboratory	Submitting Laboratory	Authors
EPI_ISL_1363119, EPI_ISL_1363120, EPI_ISL_1363121, EPI_ISL_1363122, EPI_ISL_1363123, EPI_ISL_2367482, EPI_ISL_2367483, EPI_ISL_2367484, EPI_ISL_2367485, EPI_ISL_2367486, EPI_ISL_2367487, EPI_ISL_2367488, EPI_ISL_2367534, EPI_ISL_2379985, EPI_ISL_2379986, EPI_ISL_2380011, EPI_ISL_2380012, EPI_ISL_2380018, EPI_ISL_2380019, EPI_ISL_2380020, EPI_ISL_2380023, EPI_ISL_2380024, EPI_ISL_2380026, EPI_ISL_2380027, EPI_ISL_2380028, EPI_ISL_2380029, EPI_ISL_2380030, EPI_ISL_2380031, EPI_ISL_2380032, EPI_ISL_2380033			
see above	Borneo Medical Centre	Institute of Health and Community Medicine	Chan Chia Jui; Chua Hock Hin; David Perera; Ooi Mong How; Tonnii Sia Loong Loong; Wong Jyn Shan; Wong Kiing Aik
EPI_ISL_2367511, EPI_ISL_2367512, EPI_ISL_2367513, EPI_ISL_2367520, EPI_ISL_2367521, EPI_ISL_2367522, EPI_ISL_2367523, EPI_ISL_2367524, EPI_ISL_2367525, EPI_ISL_2367526, EPI_ISL_2367527, EPI_ISL_2367528, EPI_ISL_2367529, EPI_ISL_2367530, EPI_ISL_2367531, EPI_ISL_2367532, EPI_ISL_2367533, EPI_ISL_2367535, EPI_ISL_2367536, EPI_ISL_2367537, EPI_ISL_2367538, EPI_ISL_2379957, EPI_ISL_2379958, EPI_ISL_2379959, EPI_ISL_2379960			
see above	Clinical Research Centre Hospital Sibu	Institute of Health and Community Medicine	Chan Chia Jui; Chua Hock Hin; David Perera; Ooi Mong How; Tonnii Sia Loong Loong; Wong Jyn Shan; Wong Kiing Aik
EPI_ISL_2988870, EPI_ISL_4880389, EPI_ISL_4880390, EPI_ISL_4880391, EPI_ISL_4880392, EPI_ISL_4880393, EPI_ISL_4880394, EPI_ISL_4880395, EPI_ISL_4880396, EPI_ISL_4880397, EPI_ISL_8564940, EPI_ISL_8564941, EPI_ISL_8564942			
see above	Department of Medical Microbiology and Parasitology, Hospital Universiti Sains Malaysia	Molecular Research Laboratory	Abdul Haris bin Muhammad; Ahmad Adebayo Irekeola; Ahmad Sukari Bin Halim; Alexander Chong Shu Chien; Aswini Leela; Azian Harun; Chan Yean Yean; Chua Wei Chuan; Chua Wei Lian; Engku Nur Syafirah bt Engku Abd Rahman; Farahana binti Mohamed Alexander Chong Shu Chien; Kamarul Imran Musa; Kirnpal Kaur Banga Singh; Lau Nyok Sean; Lee Lih Huey; Lee Yeong Yeh; Lim Shu Yong; Maizun binti Mohd Zain; Muhammad Azamuddeen bin Mohammad Nasir; Muhammad Fazli bin Khalid; Muhammad Nashrul Farhan Samsudin; Muhammad Zarul Hanifah Bin Md Zoqratt; Najib Majdi Bin Yaacob; Naveed Ahmed; Nazmi Liana Binti Azmi; Noor Hafizan binti Mat Salleh; Nurfadhlina Musa; Qasim Ayub; Rosline Hassan; Sadequr Rahman; Syahida binti Omar; Wan Mohd Zahiruddin Wan Mohammad; Wardah Yusof; Wilhelm Eng Wei Han; Zaini bin Hussin; Zakuan Zainy Deris
EPI_ISL_2151584, EPI_ISL_2554730, EPI_ISL_5782323, EPI_ISL_5782328	Department of Medical Microbiology, Hospital Pengajar Universiti Putra Malaysia	Department of Medical Microbiology, Hospital Pengajar Universiti Putra Malaysia	Afiqah Adzmi; Amiza Azmi; Azmiza Syawani Jasni; Boo Sook Yee; Chee Hui Yee; Chin Siang Tean; Hui-Yee Chee; Jia-Yong Lam; Leslie Than Thian Lung; Mohd Syis Zulkipli; Muadz Mohtar; Muhammad Mohd Isa; Narcisse Joseph; Narcisse MS Joseph; Niazlin Mohd Taib; Noor Hazirah Noor Azhari; Norlaila; Nur Raihana Ithnin; Nurul Huda Mohamed Rashidi; Nurul Nadiah Ismail; Rosni Ibrahim; Sallehhudin; Siti Norbaya Masri; Siti Zulaikha Zakariah; Suppiah J; Syafinaz Amin Nordin; Syafinaz Amin-Nordin; Tengku Zetty Maztura Tengku Jamaluddin; Thayan R; Yien-Ping Wong; Zamberi Sekawi
EPI_ISL_2342566, EPI_ISL_2342567, EPI_ISL_2342568, EPI_ISL_2342569, EPI_ISL_2342570, EPI_ISL_2342571, EPI_ISL_2342572, EPI_ISL_2342573, EPI_ISL_2342574, EPI_ISL_2342575, EPI_ISL_2342576, EPI_ISL_2342577, EPI_ISL_2342578, EPI_ISL_2342579, EPI_ISL_2342580, EPI_ISL_2342581, EPI_ISL_2342582, EPI_ISL_2342583, EPI_ISL_2342584, EPI_ISL_3162191, EPI_ISL_3162192, EPI_ISL_3162193, EPI_ISL_3162194, EPI_ISL_3162195			
see above	Department of Medical Microbiology, Hospital Pengajar Universiti Putra Malaysia	Malaysia Genome Institute	Avisha Richards; Azrin Ahmad; Enizza Kasim; Hui-Yee Chee; Irni Suhayu Sapian; Mohd Anuar Jonet; Mohd Faizal Abu Bakar; Mohd Noor Mat Isa; Muhammad MI; Narcisse Joseph; Nor Azfa Johari; Nor Zahrin Hasran; Nurhezreen Md Iqbal; Shamsidar Sopie; Siti Noraini Othman; Syafinaz Amin-Nordin; Yusuf Muhammad Noor
EPI_ISL_2769400, EPI_ISL_2769401, EPI_ISL_2769402, EPI_ISL_2769403, EPI_ISL_2769404, EPI_ISL_2769405, EPI_ISL_2769406, EPI_ISL_2769407, EPI_ISL_2769408, EPI_ISL_2769409, EPI_ISL_2769410, EPI_ISL_2769411, EPI_ISL_2769412, EPI_ISL_2769413, EPI_ISL_2769414, EPI_ISL_2769415, EPI_ISL_2769416, EPI_ISL_2769417, EPI_ISL_2769418, EPI_ISL_2769419, EPI_ISL_2769420, EPI_ISL_2769421, EPI_ISL_2769422, EPI_ISL_2769423, EPI_ISL_2769424, EPI_ISL_2769425, EPI_ISL_2769426, EPI_ISL_2769427, EPI_ISL_2769428, EPI_ISL_2769429, EPI_ISL_2769430, EPI_ISL_2769431, EPI_ISL_2769432, EPI_ISL_2769433, EPI_ISL_2769434, EPI_ISL_2769435, EPI_ISL_2769436, EPI_ISL_2769437, EPI_ISL_2769438, EPI_ISL_2784328, EPI_ISL_2784329			
see above	Department of Medical Microbiology, University Malaya Medical Centre	Department of Medical Microbiology, Faculty of Medicine, University of Malaya	Adeeba KAMARULZAMAN; Chee Kuan WONG; Fadhil Hadi JAMALUDDIN; I-Ching SAM; Sasheela PONNAMPALAVANAR; Sharifah Faridah SYED OMAR; University Malaya Medical Centre COVID Team; Vijayan MUNUSAMY; Yoke Fun Chan; Yoong Min CHONG
EPI_ISL_1196864	Hospital Alor Gajah	Institute for Medical Research, Infectious Disease Research Centre, National Institutes of Health, Ministry of Health Malaysia	Kamel K; Mohd Zawawi Z; Ramly N; Robert F; Suppiah J; Thayan R
EPI_ISL_1196660, EPI_ISL_1196705, EPI_ISL_1196706, EPI_ISL_1196768, EPI_ISL_1196770, EPI_ISL_1196771	Hospital Melaka	Institute for Medical Research, Infectious Disease Research Centre, National Institutes of Health, Ministry of Health Malaysia	Kamel K; Mohd Zawawi Z; Ramly N; Robert F; Suppiah J; Thayan R
EPI_ISL_1196794, EPI_ISL_1196796, EPI_ISL_1196797	Hospital Pulau Pinang	Institute for Medical Research, Infectious Disease Research Centre, National Institutes of Health, Ministry of Health Malaysia	Kamel K; Mohd Zawawi Z; Ramly N; Robert F; Suppiah J; Thayan R
EPI_ISL_718280, EPI_ISL_728253, EPI_ISL_877228, EPI_ISL_912397, EPI_ISL_912399, EPI_ISL_912400, EPI_ISL_934424, EPI_ISL_936491, EPI_ISL_944094, EPI_ISL_944095, EPI_ISL_944096, EPI_ISL_944099, EPI_ISL_944100, EPI_ISL_944101, EPI_ISL_944102, EPI_ISL_944103, EPI_ISL_944104, EPI_ISL_962203, EPI_ISL_962204, EPI_ISL_962205, EPI_ISL_962206, EPI_ISL_962207, EPI_ISL_962523, EPI_ISL_962524, EPI_ISL_962525, EPI_ISL_962526, EPI_ISL_962527, EPI_ISL_968089, EPI_ISL_1055263, EPI_ISL_1055363, EPI_ISL_1055364, EPI_ISL_1055365, EPI_ISL_1068849, EPI_ISL_1068850, EPI_ISL_1068851, EPI_ISL_1068927, EPI_ISL_1068928, EPI_ISL_1068929, EPI_ISL_1081327, EPI_ISL_1081333, EPI_ISL_1081351, EPI_ISL_1081352, EPI_ISL_1114717, EPI_ISL_1114718, EPI_ISL_1114719, EPI_ISL_1114720, EPI_ISL_1114721, EPI_ISL_1114722, EPI_ISL_1114723, EPI_ISL_1114724, EPI_ISL_1114734, EPI_ISL_1114744, EPI_ISL_1114764, EPI_ISL_1114765, EPI_ISL_1138598, EPI_ISL_1138750, EPI_ISL_1186028, EPI_ISL_1204503, EPI_ISL_1424064, EPI_ISL_1424065, EPI_ISL_1714798			
see above	Institute for Medical Research, Infectious Disease Research Centre, National Institutes of Health, Ministry of Health Malaysia	Institute for Medical Research, Infectious Disease Research Centre, National Institutes of Health, Ministry of Health Malaysia	Azizan MA; Kamel K; Moh Zawawi Z; Mohd Zawawi Z; Mohd-Zawawi Z; Ramly N; Robert F; Suppiah J; Thayan R
EPI_ISL_2367539, EPI_ISL_2367540, EPI_ISL_2367541, EPI_ISL_2367542, EPI_ISL_2367543, EPI_ISL_2367544, EPI_ISL_2367545, EPI_ISL_2367546, EPI_ISL_2367547, EPI_ISL_2367548, EPI_ISL_2367549, EPI_ISL_2367550, EPI_ISL_2367551, EPI_ISL_2367552, EPI_ISL_2367553, EPI_ISL_2367554, EPI_ISL_2367555, EPI_ISL_2367556, EPI_ISL_2367557, EPI_ISL_2367558, EPI_ISL_2367559, EPI_ISL_2367560, EPI_ISL_2379901, EPI_ISL_2379902, EPI_ISL_2379903, EPI_ISL_2379904, EPI_ISL_2379905, EPI_ISL_2379906, EPI_ISL_2379907, EPI_ISL_2379908, EPI_ISL_2379909, EPI_ISL_2379910			
see above	Institute of Health and Community Medicine	Institute of Health and Community Medicine	Chan Chia Jui; Chua Hock Hin; David Perera; Ooi Mong How; Tonnii Sia Loong Loong; Wong Jyn Shan; Wong Kiing Aik
EPI_ISL_8254288	Jabatan Forensik Hospital Sultanah Bahiyah	Department of Medical Microbiology, Faculty of Medicine, University of Malaya; University of Malaya Medical Centre	I-Ching SAM; Jolene Yin Ling FU; Omar Khalilur Rahman; Yoke Fun Chan
EPI_ISL_8147372, EPI_ISL_8147373, EPI_ISL_8147375, EPI_ISL_8147376, EPI_ISL_8147377, EPI_ISL_8147379	Klinik Kesihatan Sikamat	Tropical Infectious Diseases Research & Education Centre (TIDREC), Universiti Malaya	AsmaAnati CheMatSeri; Che-Norainon Yaacob; Jia-Yi Tan; Jo-Ern Wong; Kim-Kee Tan; Mulya-Mustika-Sari Zulkifli; Noor Syahida Azizan; Nur-Hidayana Mahfodz; Sazaly AbuBakar; Siti-Sarah Nor'e
EPI_ISL_4880370, EPI_ISL_4880371, EPI_ISL_4880372, EPI_ISL_4880373, EPI_ISL_4880401, EPI_ISL_4886612, EPI_ISL_7972344, EPI_ISL_7972378, EPI_ISL_7972379			
see above	Kota Bharu Public Health Laboratory	Molecular Research Laboratory	Abdul Haris bin Muhammad; Ahmad Sukari Bin Halim; Alexander Chong Shu Chien; Aswini Leela; Azian Harun; Chan Yean Yean; Chua Wei Chuan; Chua Wei Lian; Engku Nur Syafirah bt Engku Abd Rahman; Kamarul Imran Musa; Kirnpal Kaur Banga Singh; Lau Nyok Sean; Lee Lih Huey; Lee Yeong Yeh; Lim Shu Yong; Maizun binti Mohd Zain; Muhammad Fazli bin Khalid; Muhammad Nashrul Farhan Samsudin; Muhammad Zarul Hanifah Bin Md Zoqratt; Najib Majdi Bin Yaacob; Nazmi Liana Binti Azmi; Noor Hafizan binti Mat Salleh; Nurfadhlina Musa; Qasim Ayub; Rosline Hassan; Sadequr Rahman; Syahida binti Omar; Wan Mohd Zahiruddin Wan Mohammad; Wilhelm Eng Wei Han; Zaini bin Hussin; Zakuan Zainy Deris
EPI_ISL_1059900, EPI_ISL_1059901, EPI_ISL_1435802, EPI_ISL_1435804, EPI_ISL_1435806, EPI_ISL_1435808, EPI_ISL_1435810, EPI_ISL_1435812, EPI_ISL_1435814, EPI_ISL_1435816, EPI_ISL_1435817, EPI_ISL_1435820, EPI_ISL_2342588, EPI_ISL_2342589, EPI_ISL_2342590, EPI_ISL_2342591, EPI_ISL_2342592, EPI_ISL_2342593, EPI_ISL_2342594, EPI_ISL_2342595, EPI_ISL_2342596, EPI_ISL_2342597, EPI_ISL_2342598, EPI_ISL_2342599, EPI_ISL_2342600, EPI_ISL_3356252, EPI_ISL_3356253, EPI_ISL_3356254, EPI_ISL_3356255, EPI_ISL_3356256			
see above	Malaysia Genome Institute	Malaysia Genome Institute	Azrin Ahmad; Enizza Kasim; Irni Suhayu Sapian; Mohd Faizal Abu Bakar; Mohd Noor Mat Isa; Nor Azfa Johari.; Nurhezreen Md Iqbal; Shamsidar Sopie; Siti Noraini Othman; Yusuf Muhammad Noor
EPI_ISL_1318203, EPI_ISL_1318204, EPI_ISL_1318205, EPI_ISL_1318206, EPI_ISL_1318208, EPI_ISL_1318209, EPI_ISL_1318210, EPI_ISL_1318211, EPI_ISL_1318212, EPI_ISL_1318214, EPI_ISL_1318216, EPI_ISL_1318217, EPI_ISL_1318218, EPI_ISL_1318219, EPI_ISL_1334365, EPI_ISL_2367471, EPI_ISL_2367472, EPI_ISL_2367481, EPI_ISL_2367489, EPI_ISL_2367490, EPI_ISL_2367491, EPI_ISL_2367492, EPI_ISL_2367493, EPI_ISL_2367494, EPI_ISL_2367495, EPI_ISL_2367496, EPI_ISL_2367497, EPI_ISL_2367498, EPI_ISL_2367499, EPI_ISL_2367500, EPI_ISL_2367501, EPI_ISL_2367502, EPI_ISL_2367503, EPI_ISL_2367504, EPI_ISL_2367505, EPI_ISL_2367506, EPI_ISL_2367507, EPI_ISL_2367508, EPI_ISL_2367509, EPI_ISL_2367510, EPI_ISL_2367514, EPI_ISL_2367515, EPI_ISL_2367516, EPI_ISL_2367517, EPI_ISL_2367518, EPI_ISL_2367519, EPI_ISL_2379928, EPI_ISL_2379935, EPI_ISL_2379936, EPI_ISL_2379937, EPI_ISL_2379938, EPI_ISL_2379939, EPI_ISL_2379940, EPI_ISL_2379941, EPI_ISL_2379942, EPI_ISL_2379943, EPI_ISL_2379944, EPI_ISL_2379945, EPI_ISL_2379946, EPI_ISL_2379947, EPI_ISL_2379948, EPI_ISL_2379949, EPI_ISL_2379950, EPI_ISL_2379951, EPI_ISL_2379952, EPI_ISL_2379953, EPI_ISL_2379954, EPI_ISL_2379955, EPI_ISL_2379961, EPI_ISL_2379962, EPI_ISL_2379963, EPI_ISL_2379964, EPI_ISL_2379973, EPI_ISL_2379974, EPI_ISL_2379975, EPI_ISL_2379976, EPI_ISL_2379977, EPI_ISL_2379991, EPI_ISL_2380000, EPI_ISL_2380001, EPI_ISL_2380003, EPI_ISL_2380004, EPI_ISL_2380005, EPI_ISL_2380006, EPI_ISL_2380007, EPI_ISL_2380008, EPI_ISL_2380009, EPI_ISL_2380010			
see above	Ministry of Health Hospitals	Institute of Health and Community Medicine	Chan Chia Jui; Chua Hock Hin; David Perera; Ooi Mong How; Tonnii Sia Loong Loong; Wong Jyn Shan; Wong Kiing Aik
EPI_ISL_8147367, EPI_ISL_8147368, EPI_ISL_8147369, EPI_ISL_8147370, EPI_ISL_8147371, EPI_ISL_8147374, EPI_ISL_8147378, EPI_ISL_8147380			
see above	Pejabat Kesihatan Daerah Hulu Langat	Tropical Infectious Diseases Research & Education Centre (TIDREC), Universiti Malaya	AsmaAnati CheMatSeri; Che-Norainon Yaacob; Jia-Yi Tan; Jo-Ern Wong; Kim-Kee Tan; Mulya-Mustika-Sari Zulkifli; Noor Syahida Azizan; Nur-Hidayana Mahfodz; Sazaly AbuBakar; Siti-Sarah Nor'e
EPI_ISL_11939146	Pejabat Kesihatan Daerah Hulu Perak	iPROMISE, UiTM	Ariza Adnan; Fadzilah Mohd Nor; Lim Wai Feng; Mohd Asif Mohd Sukri; Mohd Nur Fakhruzzaman Noorizhab; Mohd Zaki Salleh; Sazzli Shahlan Kassim; Siti Farah Alwani Mohd Nawi; Siti Hamimah Sheikh Abdul Kadir; Teh Lay Kek; Wang Seok Mui
EPI_ISL_8170428	Pejabat Kesihatan Daerah Seremban	Tropical Infectious Diseases Research & Education Centre (TIDREC), Universiti Malaya	AsmaAnati CheMatSeri; Che-Norainon Yaacob; Jia-Yi Tan; Jo-Ern Wong; Kim-Kee Tan; Mulya-Mustika-Sari Zulkifli; Noor Syahida Azizan; Nur-Hidayana Mahfodz; Sazaly AbuBakar; Siti-Sarah Nor'e
EPI_ISL_11939036	Pejabat Kesihatan Pintu Masuk Antarabangsa Pulau Pinang	iPROMISE, UiTM	Ariza Adnan; Fadzilah Mohd Nor; Lim Wai Feng; Mohd Asif Mohd Sukri; Mohd Nur Fakhruzzaman Noorizhab; Mohd Zaki Salleh; Sazzli Shahlan Kassim; Siti Farah Alwani Mohd Nawi; Siti Hamimah Sheikh Abdul Kadir; Teh Lay Kek; Wang Seok Mui

EPI_ISL_1068996, EPI_ISL_1068997, EPI_ISL_1068998, EPI_ISL_1068999, EPI_ISL_1069076, EPI_ISL_1069078	UCSI University	Institute for Medical Research, Infectious Disease Research Centre, National Institutes of Health, Ministry of Health Malaysia	Azizan MA; Kamel K; Sekaran SD; Suppiah J; Thayan R
EPI_ISL_8254275	UMMC	Department of Medical Microbiology, Faculty of Medicine, University of Malaya; University of Malaya Medical Centre	I-Ching SAM; Jolene Yin Ling FU; Omar Khalilur Rahman; Yoke Fun Chan
EPI_ISL_877229, EPI_ISL_877230, EPI_ISL_877231, EPI_ISL_877232, EPI_ISL_877233, EPI_ISL_877234, EPI_ISL_877235, EPI_ISL_877236, EPI_ISL_877329, EPI_ISL_877330			
see above	University College Sedaya International (UCSI University)	Institute for Medical Research, Infectious Disease Research Centre, National Institutes of Health, Ministry of Health Malaysia	Azizan MA; Kamel K; Sekaran SD; Suppiah J; Thayan R

We gratefully acknowledge the following Authors from the Originating laboratories responsible for obtaining the specimens, as well as the Submitting laboratories where the genome data were generated and shared via GISAID, on which this research is based.

All Submitters of data may be contacted directly via www.gisaid.org

Authors are sorted alphabetically.

Acknowledgement EPI_SET Identifier: EPI_SET_20220603wf

Accession ID	Originating Laboratory	Submitting Laboratory	Authors
EPI_ISL_6967822, EPI_ISL_6967830, EPI_ISL_6967834, EPI_ISL_6967840, EPI_ISL_6967845, EPI_ISL_6967851	Bintulu Medical Centre (Bintulu)	Institute of Health and Community Medicine	Chan Chia Jui; Chua Hock Hin; David Perera; Ooi Mong How; Tonnii Sia Loong Loong; Wong Jyn Shan
EPI_ISL_2379984, EPI_ISL_2379987, EPI_ISL_2379988, EPI_ISL_2380013, EPI_ISL_2380014, EPI_ISL_2380015, EPI_ISL_2380016, EPI_ISL_2380017, EPI_ISL_2380021, EPI_ISL_2380022, EPI_ISL_2380025, EPI_ISL_2535604, EPI_ISL_2535607, EPI_ISL_2535608, EPI_ISL_2535619, EPI_ISL_2535620, EPI_ISL_2535630, EPI_ISL_2535631, EPI_ISL_2535641, EPI_ISL_2535642, EPI_ISL_2535652, EPI_ISL_2535653, EPI_ISL_2535660, EPI_ISL_2535661, EPI_ISL_2535672, EPI_ISL_2535673, EPI_ISL_2535683, EPI_ISL_2535731, EPI_ISL_2535743, EPI_ISL_2535744, EPI_ISL_2535745			
see above	Borneo Medical Centre	Institute of Health and Community Medicine	Chan Chia Jui; Chua Hock Hin; David Perera; Ooi Mong How; Tonnii Sia Loong Loong; Wong Jyn Shan; Wong Kiing Aik
EPI_ISL_6967854, EPI_ISL_6970420, EPI_ISL_6970421, EPI_ISL_6970422, EPI_ISL_6970423, EPI_ISL_6970424, EPI_ISL_6970425, EPI_ISL_6970426, EPI_ISL_6970427, EPI_ISL_6970428			
see above	Borneo Medical Centre (Kuching)	Institute of Health and Community Medicine	Chan Chia Jui; Chua Hock Hin; David Perera; Ooi Mong How; Tonnii Sia Loong Loong; Wong Jyn Shan
EPI_ISL_2535703, EPI_ISL_2535704, EPI_ISL_2535705, EPI_ISL_2535706, EPI_ISL_2535707, EPI_ISL_2535708, EPI_ISL_2535709, EPI_ISL_2535710, EPI_ISL_2535711, EPI_ISL_2535712, EPI_ISL_2535713, EPI_ISL_2535714, EPI_ISL_2535715, EPI_ISL_2535716, EPI_ISL_2535717, EPI_ISL_2535718, EPI_ISL_2535719, EPI_ISL_2535720, EPI_ISL_2535721, EPI_ISL_2535722, EPI_ISL_2535723, EPI_ISL_2535724, EPI_ISL_2535725, EPI_ISL_2535726, EPI_ISL_2535727, EPI_ISL_2535728, EPI_ISL_2535729, EPI_ISL_2535730			
see above	Clinical Research Centre Hospital Sibu	Institute of Health and Community Medicine	Chan Chia Jui; Chua Hock Hin; David Perera; Ooi Mong How; Tonnii Sia Loong Loong; Wong Jyn Shan; Wong Kiing Aik
EPI_ISL_2342546, EPI_ISL_2342547, EPI_ISL_2342552, EPI_ISL_2342553, EPI_ISL_2342554, EPI_ISL_2342555, EPI_ISL_2342556, EPI_ISL_2342557, EPI_ISL_2342558, EPI_ISL_2342559, EPI_ISL_2342560, EPI_ISL_2342561			
see above	DNA Laboratories Sdn Bhd	Malaysia Genome Institute	Azrin Ahmad; Enizza Kasim; Irni Suhayu Sapian; Mohd Faizal Abu Bakar; Mohd Noor Mat Isa; Nor Azfa Johari.; Nurhezreen Md Iqbal; Shamsidar Sopie; Siti Noraini Othman; Wong Yong Wee; Yusuf Muhammad Noor
EPI_ISL_2554731	Department of Medical Microbiology, Hospital Pengajar Universiti Putra Malaysia	Department of Medical Microbiology, Hospital Pengajar Universiti Putra Malaysia	Boo Sook Yee; Chin Siang Tean; Hui-Yee Chee; Jia-Yong Lam; Mohd Syis Zulkipli; Narcisse Joseph; Syafinaz Amin-Nordin; Yien-Ping Wong
EPI_ISL_3162196, EPI_ISL_3162197, EPI_ISL_3162198, EPI_ISL_3162199, EPI_ISL_3162200, EPI_ISL_3162201, EPI_ISL_3162202, EPI_ISL_3162203, EPI_ISL_3162204, EPI_ISL_3162205, EPI_ISL_3162206, EPI_ISL_3162207, EPI_ISL_3162208, EPI_ISL_3162209, EPI_ISL_3162210, EPI_ISL_3162211, EPI_ISL_3162212, EPI_ISL_3162213, EPI_ISL_3162214, EPI_ISL_3162215, EPI_ISL_3162216, EPI_ISL_3162217			
see above	Department of Medical Microbiology, Hospital Pengajar Universiti Putra Malaysia	Malaysia Genome Institute	Avisha Richards; Azrin Ahmad; Enizza Kasim; Hui-Yee Chee; Irni Suhayu Sapian; Mohd Anuar Jonet; Mohd Faizal Abu Bakar; Mohd Noor Mat Isa; Muhammad MI; Narcisse Joseph; Nor Azfa Johari; Nor Zahrin Hasran; Nurhezreen Md Iqbal; Shamsidar Sopie; Siti Noraini Othman; Syafinaz Amin-Nordin; Yusuf Muhammad Noor
EPI_ISL_3246352, EPI_ISL_3246353, EPI_ISL_3246360, EPI_ISL_3246416, EPI_ISL_3246417, EPI_ISL_3246418, EPI_ISL_4056169			
see above	Department of Medical Microbiology, University Malaya Medical Centre	Department of Medical Microbiology, Faculty of Medicine, University of Malaya	I-Ching SAM; Izzati Kausar; Jolene Yin Ling FU; Yoke Fun Chan; Yoong Min CHONG
EPI_ISL_5417597	HOSPITAL PAKAR SULTANAH FATIMAH	UKM Medical Molecular Biology Institute (UMBI)	Mira Farzana binti Mohamad Mokhtar
EPI_ISL_8745651	Hospital Kudat	Malaysia Genome and Vaccine Institute	Azrin Ahmad; Enizza Kasim; Irni Suhayu Sapian; Mohd Faizal Abu Bakar; Mohd Ghows Mohd Azzam.; Mohd Noor Mat Isa; Nor Azfa Johari; Nurhezreen Md Iqbal; Shamsidar Sopie; Siti Noraini Othman; Yusuf Muhammad Noor
EPI_ISL_1673337	Hospital Pakar Sultanah Fatimah	Institute for Medical Research, Infectious Disease Research Centre, National Institutes of Health, Ministry of Health Malaysia	Kamel K; Mohd Zawawi Z; Suppiah J; Thayan R
EPI_ISL_1186114, EPI_ISL_1405917	Hospital Queen Elizabeth	Institute for Medical Research, Infectious Disease Research Centre, National Institutes of Health, Ministry of Health Malaysia	Azizan MA; Kamel K; Mohd Zawawi Z; Ramly N; Robert F; Suppiah J; Thayan R
EPI_ISL_1673288	Hospital Segamat Johor	Institute for Medical Research, Infectious Disease Research Centre, National Institutes of Health, Ministry of Health Malaysia	Kamel K; Mohd Zawawi Z; Suppiah J; Thayan R
EPI_ISL_1673280	Hospital Sultan Ismail	Institute for Medical Research, Infectious Disease Research Centre, National Institutes of Health, Ministry of Health Malaysia	Kamel K; Mohd Zawawi Z; Suppiah J; Thayan R
EPI_ISL_1673283	Hospital Sultanah Aminah	Institute for Medical Research, Infectious Disease Research Centre, National Institutes of Health, Ministry of Health Malaysia	Kamel K; Mohd Zawawi Z; Suppiah J; Thayan R
EPI_ISL_1673284	Hospital Sultanah Nora Ismail	Institute for Medical Research, Infectious Disease Research Centre, National Institutes of Health, Ministry of Health Malaysia	Kamel K; Mohd Zawawi Z; Suppiah J; Thayan R
EPI_ISL_1673309	Hospital Temenggong Seri Maharaja Tun Ibrahim	Institute for Medical Research, Infectious Disease Research Centre, National Institutes of Health, Ministry of Health Malaysia	Kamel K; Mohd Zawawi Z; Suppiah J; Thayan R
EPI_ISL_4101580	ICU Dahlia Hospital Melaka	UKM Medical Molecular Biology Institute (UMBI)	Nur Alyaa Afifah Md Shahri
EPI_ISL_1055037, EPI_ISL_1055261, EPI_ISL_1055262, EPI_ISL_1055264, EPI_ISL_1055361, EPI_ISL_1055362, EPI_ISL_1081350, EPI_ISL_1196866, EPI_ISL_1196867, EPI_ISL_1263458, EPI_ISL_1263460, EPI_ISL_1263540, EPI_ISL_1264762, EPI_ISL_1265161, EPI_ISL_1405966, EPI_ISL_1406078, EPI_ISL_1406146, EPI_ISL_1406148, EPI_ISL_1406153, EPI_ISL_1406178, EPI_ISL_1406185, EPI_ISL_1406187, EPI_ISL_1406192, EPI_ISL_1406194, EPI_ISL_1406197, EPI_ISL_1406199, EPI_ISL_1406201, EPI_ISL_1406204, EPI_ISL_1406242, EPI_ISL_1406244, EPI_ISL_1406247, EPI_ISL_1406250, EPI_ISL_1406274, EPI_ISL_1406277, EPI_ISL_1406281, EPI_ISL_1406286, EPI_ISL_1406292, EPI_ISL_1406294, EPI_ISL_1406295, EPI_ISL_1424130, EPI_ISL_1424143, EPI_ISL_1424466, EPI_ISL_1424467, EPI_ISL_1424468, EPI_ISL_1424470, EPI_ISL_1424471, EPI_ISL_1424510, EPI_ISL_1424513, EPI_ISL_1424518, EPI_ISL_1673307, EPI_ISL_1673332, EPI_ISL_1673336, EPI_ISL_1673338, EPI_ISL_1673341, EPI_ISL_1673344, EPI_ISL_1673683, EPI_ISL_1673735, EPI_ISL_1696746, EPI_ISL_1696747, EPI_ISL_1696748, EPI_ISL_1696749, EPI_ISL_1696750, EPI_ISL_1696751, EPI_ISL_1696752, EPI_ISL_1696753, EPI_ISL_1696754, EPI_ISL_1696755, EPI_ISL_1696756, EPI_ISL_1696757, EPI_ISL_1696758, EPI_ISL_1714796, EPI_ISL_1714797			
see above	Institute for Medical Research, Infectious Disease Research Centre, National Institutes of Health, Ministry of Health Malaysia	Institute for Medical Research, Infectious Disease Research Centre, National Institutes of Health, Ministry of Health Malaysia	Azizan MA; Kamel K; Moh Zawawi Z; Mohd Zawawi Z; Ramly MN; Ramly N; Robert F; Suppiah J; Thayan R
EPI_ISL_2367561, EPI_ISL_2367562, EPI_ISL_2379894, EPI_ISL_2379895, EPI_ISL_2379896, EPI_ISL_2379897, EPI_ISL_2379898, EPI_ISL_2379899, EPI_ISL_2379900, EPI_ISL_2379911, EPI_ISL_2379912, EPI_ISL_2379913, EPI_ISL_2379914, EPI_ISL_2379915, EPI_ISL_2379916, EPI_ISL_2379917, EPI_ISL_2379918, EPI_ISL_2379919, EPI_ISL_2379920, EPI_ISL_2379921, EPI_ISL_2379922, EPI_ISL_2379923, EPI_ISL_2379924, EPI_ISL_2379925, EPI_ISL_2379926, EPI_ISL_2379927, EPI_ISL_2379929, EPI_ISL_2379930, EPI_ISL_2379931, EPI_ISL_2379932, EPI_ISL_2379933, EPI_ISL_2379934, EPI_ISL_2608374, EPI_ISL_2608375, EPI_ISL_2608376			
see above	Institute of Health and Community Medicine	Institute of Health and Community Medicine	Chan Chia Jui; Chua Hock Hin; David Perera; Ooi Mong How; Tonnii Sia Loong Loong; Wong Jyn Shan; Wong Kiing Aik
EPI_ISL_13047124	KOTA BHARU PUBLIC HEALTH LABORATORY	MOLECULAR RESEARCH LABORATORY	Abdul Haris bin Muhammad; Ahmad Sukari Bin Halim; Alexander Chong Shu Chien; Aswini Leela; Azian Harun; Chan Yean Yean; Chua Wei Chuan; Chua Wei Lian; Engku Nur Syafirah bt Engku Abd Rahman; Kamarul Imran Musa; Kirnpal Kaur Banga Singh; Lau Nyok Sean; Lee Lih Huey; Lee Yeong Yeh; Lim Shu Yong; Maizun binti Mohd Zain; Muhammad Fazli bin Khalid; Muhammad Nashrul Farhan Samsudin; Muhammad Zarul Hanifah Bin Md Zoqratt; Najib Majdi Bin Yaacob; Nazmi Liana Binti Azmi; Noor Hafizan binti Mat Salleh; Nurfadhlina Musa; Qasim Ayub; Rosline Hassan; Sadequr Rahman; Syahida binti Omar; Wan Mohd Zahiruddin Wan Mohammad; Wilhelm Eng Wei Han; Zaini bin Hussin; Zakuan Zainy Deris
EPI_ISL_7972315, EPI_ISL_7972316, EPI_ISL_7972317, EPI_ISL_7972318, EPI_ISL_7972319, EPI_ISL_7972320, EPI_ISL_7972321, EPI_ISL_7972322, EPI_ISL_7972323, EPI_ISL_7972324, EPI_ISL_7972325, EPI_ISL_7972326, EPI_ISL_7972327, EPI_ISL_7972328, EPI_ISL_7972329, EPI_ISL_7972330, EPI_ISL_7972331, EPI_ISL_7982611, EPI_ISL_7982612			
see above	Kota Bharu Public Health Laboratory	Molecular Research Laboratory	Abdul Haris bin Muhammad; Ahmad Sukari Bin Halim; Alexander Chong Shu Chien; Aswini Leela; Azian Harun; Chan Yean Yean; Chua Wei Chuan; Chua Wei Lian; Engku Nur Syafirah bt Engku Abd Rahman; Kamarul Imran Musa; Kirnpal Kaur Banga Singh; Lau Nyok Sean; Lee Lih Huey; Lee Yeong Yeh; Lim Shu Yong; Maizun binti Mohd Zain; Muhammad Fazli bin Khalid; Muhammad Nashrul Farhan Samsudin; Muhammad Zarul Hanifah Bin Md Zoqratt; Najib Majdi Bin Yaacob; Nazmi Liana Binti Azmi; Noor Hafizan binti Mat Salleh; Nurfadhlina Musa; Qasim Ayub; Rosline Hassan; Sadequr Rahman; Syahida binti Omar; Wan Mohd Zahiruddin Wan Mohammad; Wilhelm Eng Wei Han; Zaini bin Hussin; Zakuan Zainy Deris
EPI_ISL_3266067	Likas Hospital	Institute for Medical Research, Infectious Disease Research Centre, National Institutes of Health, Ministry of Health Malaysia	Anasir MI; Azizan MA; Kamel K; Mohd Zawawi Z; Ramly N; Robert F; Suppiah J; Thayan R
EPI_ISL_3356257, EPI_ISL_3356258, EPI_ISL_3356259, EPI_ISL_3356260, EPI_ISL_3356261, EPI_ISL_3356262, EPI_ISL_3356263, EPI_ISL_3356264, EPI_ISL_3356265, EPI_ISL_3356266, EPI_ISL_3356267, EPI_ISL_3356268, EPI_ISL_3356269, EPI_ISL_3356270, EPI_ISL_3356271, EPI_ISL_3356272, EPI_ISL_3356273			
see above	Malaysia Genome Institute	Malaysia Genome Institute	Azrin Ahmad; Enizza Kasim; Irni Suhayu Sapian; Mohd Faizal Abu Bakar; Mohd Noor Mat Isa; Nor Azfa Johari.; Nurhezreen Md Iqbal; Shamsidar Sopie; Siti Noraini Othman; Yusuf Muhammad Noor
EPI_ISL_1318207, EPI_ISL_1318213, EPI_ISL_1318215, EPI_ISL_2379956, EPI_ISL_2379965, EPI_ISL_2379966, EPI_ISL_2379967, EPI_ISL_2379968, EPI_ISL_2379969, EPI_ISL_2379970, EPI_ISL_2379971, EPI_ISL_2379972, EPI_ISL_2379978, EPI_ISL_2379979, EPI_ISL_2379980, EPI_ISL_2379981, EPI_ISL_2379982, EPI_ISL_2379983, EPI_ISL_2379989, EPI_ISL_2379990, EPI_ISL_2379992, EPI_ISL_2379993, EPI_ISL_2379994, EPI_ISL_2379995, EPI_ISL_2379996, EPI_ISL_2379997, EPI_ISL_2379998, EPI_ISL_2379999, EPI_ISL_2380002, EPI_ISL_2535602, EPI_ISL_2535603, EPI_ISL_2535613, EPI_ISL_2535614, EPI_ISL_2535615, EPI_ISL_2535616, EPI_ISL_2535625, EPI_ISL_2535626, EPI_ISL_2535627, EPI_ISL_2535628, EPI_ISL_2535636, EPI_ISL_2535637, EPI_ISL_2535638, EPI_ISL_2535639, EPI_ISL_2535640, EPI_ISL_2535647, EPI_ISL_2535648, EPI_ISL_2535649, EPI_ISL_2535650, EPI_ISL_2535667, EPI_ISL_2535668, EPI_ISL_2535669, EPI_ISL_2535679, EPI_ISL_2535680, EPI_ISL_2535773, EPI_ISL_2535774, EPI_ISL_2608377, EPI_ISL_2608378			
see above	Ministry of Health Hospitals	Institute of Health and Community Medicine	Chan Chia Jui; Chua Hock Hin; David Perera; Ooi Mong How; Tonnii Sia Loong Loong; Wong Jyn Shan; Wong Kiing Aik
EPI_ISL_6967863, EPI_ISL_6967865	Rejang Medical Centre (Sibu)	Institute of Health and Community Medicine	Chan Chia Jui; Chua Hock Hin; David Perera; Ooi Mong How; Tonnii Sia Loong Loong; Wong Jyn Shan
EPI_ISL_5051748, EPI_ISL_5051766, EPI_ISL_5052016, EPI_ISL_5052017, EPI_ISL_5052018, EPI_ISL_5052019, EPI_ISL_6970349, EPI_ISL_6970354, EPI_ISL_6970358, EPI_ISL_6970362, EPI_ISL_6970370, EPI_ISL_6970373, EPI_ISL_6970431			
see above	Sarawak General Hospital (Kuching)	Institute of Health and Community Medicine	Chan Chia Jui; Chua Hock Hin; David Perera; Ooi Mong How; Tonnii Sia Loong Loong; Wong Jyn Shan
EPI_ISL_2815376	Serdang Hospital	Institute for Medical Research, Infectious Disease Research Centre, National Institutes of Health, Ministry of Health Malaysia	Azizan MA; Kamel K; Mohd Zawawi Z; Ramly N; Robert F; Suppiah J; Thayan R

We gratefully acknowledge the following Authors from the Originating laboratories responsible for obtaining the specimens, as well as the Submitting laboratories where the genome data were generated and shared via GISAID, on which this research is based.

All Submitters of data may be contacted directly via www.gisaid.org

Authors are sorted alphabetically.

Accession ID	Originating Laboratory	Submitting Laboratory	Authors
EPI_ISL_1973325, EPI_ISL_1973326, EPI_ISL_1973327, EPI_ISL_1973328, EPI_ISL_2020238, EPI_ISL_2020239, EPI_ISL_2020240			
see above	Bintulu Hospital	Institute for Medical Research, Infectious Disease Research Centre, National Institutes of Health, Ministry of Health Malaysia	Azizan MA; Kamel K; Mohd Zawawi Z; Ramly N; Robert F; Suppiah J; Thayan R
EPI_ISL_2535632, EPI_ISL_2535695, EPI_ISL_2535696, EPI_ISL_2535697, EPI_ISL_2535698, EPI_ISL_2535699, EPI_ISL_2535700, EPI_ISL_2535701, EPI_ISL_2535702, EPI_ISL_2535732, EPI_ISL_2535733, EPI_ISL_2535734, EPI_ISL_2535735, EPI_ISL_2535736, EPI_ISL_2535737, EPI_ISL_2535738, EPI_ISL_2535739, EPI_ISL_2535740, EPI_ISL_2535741, EPI_ISL_2535742, EPI_ISL_2535746, EPI_ISL_2535747, EPI_ISL_2535748, EPI_ISL_2535749, EPI_ISL_2535750, EPI_ISL_2535751, EPI_ISL_2535752, EPI_ISL_2535753, EPI_ISL_2535754, EPI_ISL_2535755, EPI_ISL_2535756, EPI_ISL_2535757, EPI_ISL_2535758, EPI_ISL_2535759, EPI_ISL_2535760, EPI_ISL_2535761, EPI_ISL_2535762, EPI_ISL_2535763, EPI_ISL_2535764, EPI_ISL_2535765, EPI_ISL_2535766, EPI_ISL_2535767, EPI_ISL_2535768, EPI_ISL_2535769, EPI_ISL_2535770, EPI_ISL_2535771, EPI_ISL_2535772, EPI_ISL_2535781			
see above	Borneo Medical Centre	Institute of Health and Community Medicine	Chan Chia Jui; Chua Hock Hin; David Perera; Ooi Mong How; Tonnii Sia Loong Loong; Wong Jyn Shan; Wong Kiing Aik
EPI_ISL_2608406, EPI_ISL_2608407	Clinical Research Centre, Sarawak General Hospital	Institute of Health and Community Medicine	Chan Chia Jui; Chua Hock Hin; David Perera; Ooi Mong How; Tonnii Sia Loong Loong; Wong Jyn Shan; Wong Kiing Aik
EPI_ISL_2342548, EPI_ISL_2342549, EPI_ISL_2342550, EPI_ISL_2342551, EPI_ISL_2342562, EPI_ISL_2342563, EPI_ISL_2342564, EPI_ISL_2342565			
see above	DNA Laboratories Sdn Bhd	Malaysia Genome Institute	Azrin Ahmad; Enizza Kasim; Irni Suhayu Sapian; Mohd Faizal Abu Bakar; Mohd Noor Mat Isa; Nor Azfa Johari.; Nurhezreen Md Iqbal; Shamsidar Sopie; Siti Noraini Othman; Wong Yong Wee; Yusuf Muhammad Noor
EPI_ISL_7972352, EPI_ISL_7972353, EPI_ISL_7972355, EPI_ISL_7972356, EPI_ISL_7972357, EPI_ISL_7972358, EPI_ISL_7972359			
see above	Department of Medical Microbiology and Parasitology, Hospital Universiti Sains Malaysia	Molecular Research Laboratory	Abdul Haris bin Muhammad; Ahmad Sukari Bin Halim; Alexander Chong Shu Chien; Aswini Leela; Azian Harun; Chan Yean Yean; Chua Wei Chuan; Chua Wei Lian; Engku Nur Syafirah bt Engku Abd Rahman; Kamarul Imran Musa; Kirnpal Kaur Banga Singh; Lau Nyok Sean; Lee Lih Huey; Lee Yeong Yeh; Lim Shu Yong; Maizun binti Mohd Zain; Muhammad Fazli bin Khalid; Muhammad Nashrul Farhan Samsudin; Muhammad Zarul Hanifah Bin Md Zoqratt; Najib Majdi Bin Yaacob; Nazmi Liana Binti Azmi; Noor Hafizan binti Mat Salleh; Nurfadhlina Musa; Qasim Ayub; Rosline Hassan; Sadequr Rahman; Syahida binti Omar; Wan Mohd Zahiruddin Wan Mohammad; Wilhelm Eng Wei Han; Zaini bin Hussin; Zakuan Zainy Deris
EPI_ISL_3162218, EPI_ISL_3162219, EPI_ISL_3162220	Department of Medical Microbiology, Hospital Pengajar Universiti Putra Malaysia	Malaysia Genome Institute	Avisha Richards; Azrin Ahmad; Enizza Kasim; Hui-Yee Chee; Irni Suhayu Sapian; Mohd Anuar Jonet; Mohd Faizal Abu Bakar; Mohd Noor Mat Isa; Muhammad MI; Narcisse Joseph; Nor Azfa Johari; Nor Zahrin Hasran; Nurhezreen Md Iqbal; Shamsidar Sopie; Siti Noraini Othman; Syafinaz Amin-Nordin; Yusuf Muhammad Noor
EPI_ISL_3246350, EPI_ISL_3246351, EPI_ISL_3246354, EPI_ISL_3246355, EPI_ISL_3246356, EPI_ISL_3246357, EPI_ISL_3246358, EPI_ISL_3246359, EPI_ISL_3246361, EPI_ISL_3246362, EPI_ISL_3246363, EPI_ISL_3246364, EPI_ISL_3246365, EPI_ISL_3246366, EPI_ISL_3246367, EPI_ISL_3246368, EPI_ISL_3246369, EPI_ISL_3246370, EPI_ISL_3246371, EPI_ISL_3246372, EPI_ISL_3246373, EPI_ISL_3246374, EPI_ISL_3246375, EPI_ISL_3246376, EPI_ISL_3246377, EPI_ISL_3246378, EPI_ISL_3246379, EPI_ISL_3246380, EPI_ISL_3246381, EPI_ISL_3246382, EPI_ISL_3246383, EPI_ISL_3246384, EPI_ISL_3246385, EPI_ISL_3246386, EPI_ISL_3246387, EPI_ISL_3246388, EPI_ISL_3246389, EPI_ISL_3246390, EPI_ISL_3246391, EPI_ISL_3246392, EPI_ISL_3246393, EPI_ISL_3246394, EPI_ISL_3246395, EPI_ISL_3246396, EPI_ISL_3246397, EPI_ISL_3246398, EPI_ISL_3246399, EPI_ISL_3246400, EPI_ISL_3246401, EPI_ISL_3246402, EPI_ISL_3246403, EPI_ISL_3246404, EPI_ISL_3246405, EPI_ISL_3246406, EPI_ISL_3246407, EPI_ISL_3246408, EPI_ISL_3246409, EPI_ISL_3246410, EPI_ISL_3246411, EPI_ISL_3246412, EPI_ISL_3246413, EPI_ISL_3246414, EPI_ISL_3246415, EPI_ISL_3246419, EPI_ISL_3246420, EPI_ISL_3246421, EPI_ISL_3246422, EPI_ISL_3246423, EPI_ISL_3246424, EPI_ISL_3246425, EPI_ISL_3246426, EPI_ISL_3246427, EPI_ISL_3246428, EPI_ISL_3246429, EPI_ISL_3246430, EPI_ISL_3492535			
see above	Department of Medical Microbiology, University Malaya Medical Centre	Department of Medical Microbiology, Faculty of Medicine, University of Malaya	I-Ching SAM; Jolene Yin Ling FU; Yoke Fun Chan; Yoong Min CHONG
EPI_ISL_2621676, EPI_ISL_2621677, EPI_ISL_2622006, EPI_ISL_2622007, EPI_ISL_2622045, EPI_ISL_2622047, EPI_ISL_2622079			
see above	Department of Pathology	Centre for Research in Advanced Tropical Bioscience	Hajar Fauzan Bin Ahmad; Norhidayah Binti Kamarudin; Ummu Afeera Zainulabid
EPI_ISL_2622046, EPI_ISL_2622088	Department of Pathology	Centre for Research in Advanced Tropical Bioscience Universiti Malaysia Pahang, 26300 Gambang, Pahang, Malaysia	Hajar Fauzan Bin Ahmad; Norhidayah Binti Kamarudin; Ummu Afeera Zainulabid
EPI_ISL_1919861	Duchess of Kent Hospital, Sandakan	Institute for Medical Research, Infectious Disease Research Centre, National Institutes of Health, Ministry of Health Malaysia	Kamel K; Mohd Zawawi Z; Suppiah J; Thayan R
EPI_ISL_1673257	Hospital Lahad Datu	Institute for Medical Research, Infectious Disease Research Centre, National Institutes of Health, Ministry of Health Malaysia Jalan Setia Murni U13/52, Bandar Setia Alam, 40170 Selangor, Malaysia	Kamel K; Mohd Zawawi Z; Suppiah J; Thayan R
EPI_ISL_2429132	Hospital Raja Permaisuri Bainun	Institute for Medical Research, Infectious Disease Research Centre, National Institutes of Health, Ministry of Health Malaysia	Azizan MA; Kamel K; Mohd Zawawi Z; Ramly N; Robert F; Suppiah J; Thayan R
EPI_ISL_1672633	Hospital Tuanku Jaafar Seremban	Institute for Medical Research, Infectious Disease Research Centre, National Institutes of Health, Ministry of Health Malaysia	Kamel K; Mohd Zawawi Z; Suppiah J; Thayan R
EPI_ISL_1583006, EPI_ISL_1583007, EPI_ISL_1585242, EPI_ISL_1673287, EPI_ISL_1673293, EPI_ISL_1673296, EPI_ISL_1673299, EPI_ISL_1673304, EPI_ISL_1673340, EPI_ISL_1673342, EPI_ISL_1673345, EPI_ISL_1673420, EPI_ISL_1787254, EPI_ISL_1787317, EPI_ISL_1787318, EPI_ISL_1787319, EPI_ISL_1787320, EPI_ISL_1787321, EPI_ISL_1787322, EPI_ISL_1787323, EPI_ISL_1787324, EPI_ISL_1787689, EPI_ISL_1787983, EPI_ISL_1788079, EPI_ISL_1788080, EPI_ISL_1788081, EPI_ISL_1788082, EPI_ISL_1789203, EPI_ISL_1789204, EPI_ISL_1805505, EPI_ISL_1805508, EPI_ISL_1805509, EPI_ISL_1807185, EPI_ISL_1807186, EPI_ISL_1807188, EPI_ISL_1807206, EPI_ISL_1807207, EPI_ISL_1919650, EPI_ISL_1919651, EPI_ISL_1919652, EPI_ISL_1919653, EPI_ISL_1919654, EPI_ISL_1919860, EPI_ISL_1972534, EPI_ISL_1972535, EPI_ISL_1972536, EPI_ISL_1972537, EPI_ISL_2090891, EPI_ISL_2091000, EPI_ISL_2811884			
see above	Institute for Medical Research, Infectious Disease Research Centre, National Institutes of Health, Ministry of Health Malaysia	Institute for Medical Research, Infectious Disease Research Centre, National Institutes of Health, Ministry of Health Malaysia	Azizan MA; Kamel K; Mohd Zawawi Z; Ramly N; Robert F; Suppiah J; Thayan R
EPI_ISL_1919422, EPI_ISL_1919645, EPI_ISL_1919646	Ipoh Public Health Laboratory (MKAI), Ministry of Health Malaysia	Institute for Medical Research, Infectious Disease Research Centre, National Institutes of Health, Ministry	Kamel K; Mohd Zawawi Z; Suppiah J; Thayan R
EPI_ISL_1821883, EPI_ISL_1821884, EPI_ISL_1919272, EPI_ISL_1919273, EPI_ISL_1919647, EPI_ISL_1919648, EPI_ISL_1919649, EPI_ISL_1919858, EPI_ISL_1919859, EPI_ISL_1919944, EPI_ISL_1919945, EPI_ISL_1919946, EPI_ISL_1919947, EPI_ISL_1919949, EPI_ISL_1919950, EPI_ISL_1919951, EPI_ISL_1919952, EPI_ISL_1919986, EPI_ISL_1919987, EPI_ISL_1921767, EPI_ISL_1921769, EPI_ISL_1921770, EPI_ISL_2620718, EPI_ISL_2620728, EPI_ISL_2620744, EPI_ISL_2620745, EPI_ISL_2620901, EPI_ISL_2621621, EPI_ISL_2621622, EPI_ISL_2621623, EPI_ISL_2621624, EPI_ISL_2621625, EPI_ISL_2621626, EPI_ISL_2621627, EPI_ISL_2621628, EPI_ISL_2621629, EPI_ISL_2621630, EPI_ISL_2621631, EPI_ISL_2815377			
see above	Ipoh Public Health Laboratory (MKAI), Ministry of Health Malaysia	Institute for Medical Research, Infectious Disease Research Centre, National Institutes of Health, Ministry of Health Malaysia	Azizan MA; Kamel K; Mohd Zawawi Z; Ramly N; Robert F; Suppiah J; Thayan R
EPI_ISL_1972549, EPI_ISL_1972550	Klinik Kesihatan Bentong	Institute for Medical Research, Infectious Disease Research Centre, National Institutes of Health, Ministry of Health Malaysia	Azizan MA; Kamel K; Mohd Zawawi Z; Ramly N; Robert F; Suppiah J; Thayan R
EPI_ISL_1972553	Klinik Kesihatan Karak	Institute for Medical Research, Infectious Disease Research Centre, National Institutes of Health, Ministry of Health Malaysia	Azizan MA; Kamel K; Mohd Zawawi Z; Ramly N; Robert F; Suppiah J; Thayan R
EPI_ISL_1972548, EPI_ISL_1972551, EPI_ISL_1972552	Klinik Kesihatan Lurah Bilut (Felda)	Institute for Medical Research, Infectious Disease Research Centre, National Institutes of Health, Ministry of Health Malaysia	Azizan MA; Kamel K; Mohd Zawawi Z; Ramly N; Robert F; Suppiah J; Thayan R
EPI_ISL_2811874	Klinik Kesihatan Pasir Akar	Institute for Medical Research, Infectious Disease Research Centre, National Institutes of Health, Ministry of Health Malaysia	Azizan MA; Kamel K; Mohd Zawawi Z; Ramly N; Robert F; Suppiah J; Thayan R
EPI_ISL_1973324, EPI_ISL_2020075, EPI_ISL_2020076, EPI_ISL_2020077, EPI_ISL_2020078, EPI_ISL_2020165, EPI_ISL_2020166, EPI_ISL_2020167			
see above	Kota Bharu Public Health Laboratory	Institute for Medical Research, Infectious Disease Research Centre, National Institutes of Health, Ministry of Health Malaysia	Azizan MA; Kamel K; Mohd Zawawi Z; Ramly N; Robert F; Suppiah J; Thayan R; uppiah J
EPI_ISL_7972334, EPI_ISL_7972337, EPI_ISL_7972338, EPI_ISL_7972339, EPI_ISL_7972347, EPI_ISL_7972348, EPI_ISL_7972349, EPI_ISL_7972350, EPI_ISL_7972351			
see above	Kota Bharu Public Health Laboratory	Molecular Research Laboratory	Abdul Haris bin Muhammad; Ahmad Sukari Bin Halim; Alexander Chong Shu Chien; Aswini Leela; Azian Harun; Chan Yean Yean; Chua Wei Chuan; Chua Wei Lian; Engku Nur Syafirah bt Engku Abd Rahman; Kamarul Imran Musa; Kirnpal Kaur Banga Singh; Lau Nyok Sean; Lee Lih Huey; Lee Yeong Yeh; Lim Shu Yong; Maizun binti Mohd Zain; Muhammad Fazli bin Khalid; Muhammad Nashrul Farhan Samsudin; Muhammad Zarul Hanifah Bin Md Zoqratt; Najib Majdi Bin Yaacob; Nazmi Liana Binti Azmi; Noor Hafizan binti Mat Salleh; Nurfadhlina Musa; Qasim Ayub; Rosline Hassan; Sadequr Rahman; Syahida binti Omar; Wan Mohd Zahiruddin Wan Mohammad; Wilhelm Eng Wei Han; Zaini bin Hussin; Zakuan Zainy Deris
EPI_ISL_2621664	Kulim Hospital	Institute for Medical Research, Infectious Disease Research Centre, National Institutes of Health, Ministry of Health Malaysia	Azizan MA; Kamel K; Mohd Zawawi Z; Ramly N; Robert F; Suppiah J; Thayan R
EPI_ISL_1585270, EPI_ISL_1585271	Labuan Hospital	Institute for Medical Research, Infectious Disease Research Centre, National Institutes of Health, Ministry of Health Malaysia	Kamel K; Mohd Zawawi Z; Ramly N; Robert F; Suppiah J; Thayan R
EPI_ISL_3356274, EPI_ISL_3356275, EPI_ISL_3356276, EPI_ISL_3356277, EPI_ISL_3356278, EPI_ISL_3356279, EPI_ISL_3356280, EPI_ISL_3356281			
see above	Malaysia Genome Institute	Malaysia Genome Institute	Azrin Ahmad; Enizza Kasim; Irni Suhayu Sapian; Mohd Faizal Abu Bakar; Mohd Noor Mat Isa; Nor Azfa Johari.; Nurhezreen Md Iqbal; Shamsidar Sopie; Siti Noraini Othman; Yusuf Muhammad Noor
EPI_ISL_2811876, EPI_ISL_2811877	Melaka Hospital	Institute for Medical Research, Infectious Disease Research Centre, National Institutes of Health, Ministry of Health Malaysia	Azizan MA; Kamel K; Mohd Zawawi Z; Ramly N; Robert F; Suppiah J; Thayan R
EPI_ISL_2535605, EPI_ISL_2535606, EPI_ISL_2535609, EPI_ISL_2535610, EPI_ISL_2535611, EPI_ISL_2535612, EPI_ISL_2535617, EPI_ISL_2535618, EPI_ISL_2535621, EPI_ISL_2535622, EPI_ISL_2535623, EPI_ISL_2535624, EPI_ISL_2535629, EPI_ISL_2535633, EPI_ISL_2535634, EPI_ISL_2535635, EPI_ISL_2535643, EPI_ISL_2535644, EPI_ISL_2535645, EPI_ISL_2535646, EPI_ISL_2535651, EPI_ISL_2535654, EPI_ISL_2535655, EPI_ISL_2535656, EPI_ISL_2535657, EPI_ISL_2535658, EPI_ISL_2535659, EPI_ISL_2535662, EPI_ISL_2535663, EPI_ISL_2535664, EPI_ISL_2535665, EPI_ISL_2535666, EPI_ISL_2535670, EPI_ISL_2535671, EPI_ISL_2535674, EPI_ISL_2535675, EPI_ISL_2535676, EPI_ISL_2535677, EPI_ISL_2535678, EPI_ISL_2535681, EPI_ISL_2535682, EPI_ISL_2535684, EPI_ISL_2535685, EPI_ISL_2535686, EPI_ISL_2535687, EPI_ISL_2535688, EPI_ISL_2535689, EPI_ISL_2535690, EPI_ISL_2535691, EPI_ISL_2535692, EPI_ISL_2535693, EPI_ISL_2535694, EPI_ISL_2535775, EPI_ISL_2535776, EPI_ISL_2535777			
see above	Ministry of Health Hospitals	Institute of Health and Community Medicine	Chan Chia Jui; Chua Hock Hin; David Perera; Ooi Mong How; Tonnii Sia Loong Loong; Wong Jyn Shan; Wong Kiing Aik
EPI_ISL_5052020, EPI_ISL_5052021, EPI_ISL_5052022, EPI_ISL_5052023, EPI_ISL_5052024, EPI_ISL_5052025, EPI_ISL_5052180, EPI_ISL_6970384, EPI_ISL_8650058, EPI_ISL_8650132, EPI_ISL_8650140, EPI_ISL_8650150			
see above	Sarawak General Hospital (Kuching)	Institute of Health and Community Medicine	Chan Chia Jui; Chua Hock Hin; David Perera; Ooi Mong How; Tonnii Sia Loong Loong; Wong Jyn Shan
EPI_ISL_2828708	Sultan Ahmad Shah Medical Centre	Centre for Research in Advanced Tropical Bioscience (Biotropic	Hajar Fauzan Bin Ahmad

	@IIUM(SASMEC@IIUM)	Centre)	
EPI_ISL_1972365, EPI_ISL_1972366, EPI_ISL_1972533	Sultanah Bahiyah Hospital, Alor Setar	Institute for Medical Research, Infectious Disease Research Centre, National Institutes of Health, Ministry of Health Malaysia	Azizan MA; Kamel K; Mohd Zawawi Z; Ramly N; Robert F; Suppiah J; Thayan R
EPI_ISL_2811878	Sultanah Nora Ismail Hospital, Batu Pahat	Institute for Medical Research, Infectious Disease Research Centre, National Institutes of Health, Ministry of Health Malaysia	Azizan MA; Kamel K; Mohd Zawawi Z; Ramly N; Robert F; Suppiah J; Thayan R
EPI_ISL_1972356, EPI_ISL_2091020, EPI_ISL_2625623	Sungai Buloh Hospital	Institute for Medical Research, Infectious Disease Research Centre, National Institutes of Health, Ministry of Health Malaysia	Azizan MA; Kamel K; Mohd Zawawi Z; Ramly N; Robert F; Suppiah J; Thayan R
EPI_ISL_1972538, EPI_ISL_1972539, EPI_ISL_1972540, EPI_ISL_1972547	Tampin Hospital	Institute for Medical Research, Infectious Disease Research Centre, National Institutes of Health, Ministry of Health Malaysia	Azizan MA; Kamel K; Mohd Zawawi Z; Ramly N; Robert F; Suppiah J; Thayan R
EPI_ISL_1919862, EPI_ISL_1919863	Tawau Hospital	Institute for Medical Research, Infectious Disease Research Centre, National Institutes of Health, Ministry of Health Malaysia	Kamel K; Mohd Zawawi Z; Suppiah J; Thayan R

We gratefully acknowledge the following Authors from the Originating laboratories responsible for obtaining the specimens, as well as the Submitting laboratories where the genome data were generated and shared via GISAID, on which this research is based.

All Submitters of data may be contacted directly via www.gisaid.org

Authors are sorted alphabetically.

Accession ID	Originating Laboratory	Submitting Laboratory	Authors
EPI_ISL_2854069	Bayan Lepas Health Clinic	Institute for Medical Research, Infectious Disease Research Centre, National Institutes of Health, Ministry of Health Malaysia	Anasir MI; Azizan MA; Kamel K; Mohd Zawawi Z; Ramly N; Robert F; Suppiah J; Thayan R
EPI_ISL_2535780, EPI_ISL_2608379, EPI_ISL_2608380, EPI_ISL_2608381, EPI_ISL_2608382, EPI_ISL_2608383, EPI_ISL_2608384, EPI_ISL_2608385, EPI_ISL_2608386, EPI_ISL_2608387, EPI_ISL_2608388, EPI_ISL_2608389, EPI_ISL_2608390, EPI_ISL_2608391, EPI_ISL_2608392, EPI_ISL_2608393, EPI_ISL_2608394, EPI_ISL_2608395, EPI_ISL_2608396, EPI_ISL_2608397, EPI_ISL_2608398, EPI_ISL_2608399, EPI_ISL_2608400, EPI_ISL_2608401, EPI_ISL_2608402, EPI_ISL_2608403, EPI_ISL_2608404, EPI_ISL_2608405, EPI_ISL_4460122			
see above	Borneo Medical Centre	Institute of Health and Community Medicine	Chan Chia Jui; Chua Hock Hin; David Perera; Ooi Mong How; Tonnii Sia Loong Loong; Wong Jyn Shan; Wong Kiing Aik
EPI_ISL_2608408, EPI_ISL_2608409, EPI_ISL_2608410, EPI_ISL_2608411, EPI_ISL_2608412, EPI_ISL_2608413, EPI_ISL_2608414			
see above	Clinical Research Centre, Sarawak General Hospital	Institute of Health and Community Medicine	Chan Chia Jui; Chua Hock Hin; David Perera; Ooi Mong How; Tonnii Sia Loong Loong; Wong Jyn Shan; Wong Kiing Aik
EPI_ISL_13025942, EPI_ISL_13025943, EPI_ISL_13025944, EPI_ISL_13025945, EPI_ISL_13025946, EPI_ISL_13025947, EPI_ISL_13025948, EPI_ISL_13025949, EPI_ISL_13025950, EPI_ISL_13025951, EPI_ISL_13025952, EPI_ISL_13025953, EPI_ISL_13025954, EPI_ISL_13025955, EPI_ISL_13025956, EPI_ISL_13025957, EPI_ISL_13025958, EPI_ISL_13025959, EPI_ISL_13025960, EPI_ISL_13025961, EPI_ISL_13025962, EPI_ISL_13025963, EPI_ISL_13025964, EPI_ISL_13025965, EPI_ISL_13025966, EPI_ISL_13025967, EPI_ISL_13025968, EPI_ISL_13025969, EPI_ISL_13025970, EPI_ISL_13025971, EPI_ISL_13025972, EPI_ISL_13025973, EPI_ISL_13025974, EPI_ISL_13025975, EPI_ISL_13025976, EPI_ISL_13025977, EPI_ISL_13025978, EPI_ISL_13025979, EPI_ISL_13025980, EPI_ISL_13025981, EPI_ISL_13025982, EPI_ISL_13025983, EPI_ISL_13025984, EPI_ISL_13025985, EPI_ISL_13025986, EPI_ISL_13025987, EPI_ISL_13025988, EPI_ISL_13025989, EPI_ISL_13025990, EPI_ISL_13025991, EPI_ISL_13025992, EPI_ISL_13025993, EPI_ISL_13025994, EPI_ISL_13025995, EPI_ISL_13025996, EPI_ISL_13025997, EPI_ISL_13025998, EPI_ISL_13025999, EPI_ISL_13026000, EPI_ISL_13026001, EPI_ISL_13026002, EPI_ISL_13026003, EPI_ISL_13026004, EPI_ISL_13026005, EPI_ISL_13026006, EPI_ISL_13026032, EPI_ISL_13026033, EPI_ISL_13026034, EPI_ISL_13026035, EPI_ISL_13026036, EPI_ISL_13026037, EPI_ISL_13026038, EPI_ISL_13026039, EPI_ISL_13026040, EPI_ISL_13026041, EPI_ISL_13026042, EPI_ISL_13026043, EPI_ISL_13026044, EPI_ISL_13026045, EPI_ISL_13026046, EPI_ISL_13026047, EPI_ISL_13026048, EPI_ISL_13026049, EPI_ISL_13026050			
see above	DNA Laboratories Sdn Bhd	Universiti Putra Malaysia (UPM)	Choo Yee Yu; Hui Yee Chee; Nancy Woan Charn Liew; Narcisse Joseph; Nurulfiza Mat Isa; Sie Yeng Wong; Syafinaz Amin-Nordin; Zunita Zakaria
EPI_ISL_5396015	Department of Forensic Medicine, Hospital Queen Elizabeth	Institute for Medical Research, Infectious Disease Research Centre, National Institutes of Health, Ministry of Health Malaysia	Ahmad FA; Ahmad Fazilah NA; Anasir MI; Azizan MA; Kamel K; Mohd Zawawi Z; Norhisham SN; Ramly N; Robert F; Suppiah J; Thayan R
EPI_ISL_7972360, EPI_ISL_7982613	Department of Medical Microbiology and Parasitology, Hospital Universiti Sains Malaysia	Molecular Research Laboratory	Abdul Haris bin Muhammad; Ahmad Sukari Bin Halim; Alexander Chong Shu Chien; Aswini Leela; Azian Harun; Chan Yean Yean; Chua Wei Chuan; Chua Wei Lian; Engku Nur Syafirah bt Engku Abd Rahman; Kamarul Imran Musa; Kirnpal Kaur Banga Singh; Lau Nyok Sean; Lee Lih Huey; Lee Yeong Yeh; Lim Shu Yong; Maizun binti Mohd Zain; Muhammad Fazli bin Khalid; Muhammad Nashrul Farhan Samsudin; Muhammad Zarul Hanifah Bin Md Zoqratt; Najib Majdi Bin Yaacob; Nazmi Liana Binti Azmi; Noor Hafizan binti Mat Salleh; Nurfadhlina Musa; Qasim Ayub; Rosline Hassan; Sadequr Rahman; Syahida binti Omar; Wan Mohd Zahiruddin Wan Mohammad; Wilhelm Eng Wei Han; Zaini bin Hussin; Zakuan Zainy Deris
EPI_ISL_5782319	Department of Medical Microbiology, Hospital Pengajar Universiti Putra Malaysia	Department of Medical Microbiology, Hospital Pengajar Universiti Putra Malaysia	Afiqah Adzmi; Amiza Azmi; Azmiza Syawani Jasni; Chee Hui Yee; Leslie Than Thian Lung; Muadz Mohtar; Muhammad Mohd Isa; Narcisse MS Joseph; Niazlin Mohd Taib; Noor Hazirah Noor Azhari; Norlaila; Nur Raihana Ithnin; Nurul Huda Mohamed Rashidi; Nurul Nadiah Ismail; Rosni Ibrahim; Sallehhudin; Siti Norbaya Masri; Siti Zulaikha Zakariah; Suppiah J; Syafinaz Amin Nordin; Tengku Zetty Maztura Tengku Jamaluddin; Thayan R; Zamberi Sekawi
EPI_ISL_3162221, EPI_ISL_3162222	Department of Medical Microbiology, Hospital Pengajar Universiti Putra Malaysia	Malaysia Genome Institute	Avisha Richards; Azrin Ahmad; Enizza Kasim; Hui-Yee Chee; Irni Suhayu Sapian; Mohd Anuar Jonet; Mohd Faizal Abu Bakar; Mohd Noor Mat Isa; Muhammad MI; Narcisse Joseph; Nor Azfa Johari; Nor Zahrin Hasran; Nurhezreen Md Iqbal; Shamsidar Sopie; Siti Noraini Othman; Syafinaz Amin-Nordin; Yusuf Muhammad Noor
EPI_ISL_3246345, EPI_ISL_3246346, EPI_ISL_3246347, EPI_ISL_3246348, EPI_ISL_3246349, EPI_ISL_4056144, EPI_ISL_4056145, EPI_ISL_4056146, EPI_ISL_4056163, EPI_ISL_4056167, EPI_ISL_4056173			
see above	Department of Medical Microbiology, University Malaya Medical Centre	Department of Medical Microbiology, Faculty of Medicine, University of Malaya	I-Ching SAM; Izzati Kausar; Jolene Yin Ling FU; Yoke Fun Chan; Yoong Min CHONG
EPI_ISL_2622089	Department of Pathology	Centre for Research in Advanced Tropical Bioscience Universiti Malaysia Pahang, 26300 Gambang, Pahang, Malaysia	Hajar Fauzan Bin Ahmad; Norhidayah Binti Kamarudin; Ummu Afeera Zainulabid
EPI_ISL_2549562	Gribbles Pathology	Institute for Medical Research, Infectious Disease Research Centre, National Institutes of Health, Ministry of Health Malaysia	Azizan MA; Kamel K; Mohd Zawawi Z; Ramly N; Robert F; Suppiah J; Thayan R
EPI_ISL_2233383	Gribbles Pathology Malaysia	Institute for Medical Research, Infectious Disease Research Centre, National Institutes of Health, Ministry of Health Malaysia	Azizan MA; Kamel K; Mohd Zawawi Z; Ramly N; Robert F; Suppiah J; Thayan R
EPI_ISL_8500690	HOSPITAL TUANKU FAUZIAH	iPROMISE, UiTM	Ariza Adnan; Fadzilah Mohd Nor; Lim Wai Feng; Mohd Asif Mohd Sukri; Mohd Nur Fakhruzzaman Noorizhab; Mohd Zaki Salleh; Sazzli Shahlan Kassim; Siti Farah Alwani Mohd Nawi; Siti Hamimah Sheikh Abdul Kadir; Teh Lay Kek; Wang Seok Mui
EPI_ISL_2233382, EPI_ISL_2550733	Hospital Labuan	Institute for Medical Research, Infectious Disease Research Centre, National Institutes of Health, Ministry of Health Malaysia	Azizan MA; Kamel K; Mohd Zawawi Z; Ramly N; Robert F; Suppiah J; Thayan R
EPI_ISL_2379461	Hospital Melaka	Institute for Medical Research, Infectious Disease Research Centre, National Inst	Azizan MA; Kamel K; Mohd Zawawi Z; Ramly N; Robert F; Suppiah J; Thayan R
EPI_ISL_5417631	Hospital Sultanah Nora Ismail	UKM Medical Molecular Biology Institute (UMBI)	Mira Farzana binti Mohamad Mokhtar
EPI_ISL_2650001, EPI_ISL_2650002	Hospital Sultanah Nur Zahirah Kuala Terengganu	Institute for Medical Research, Infectious Disease Research Centre, National Institutes of Health, Ministry of Health Malaysia	Azizan MA; Kamel K; Mohd Zawawi Z; Ramly N; Robert F; Suppiah J; Thayan R
EPI_ISL_8745646	Hospital Sungai Buloh	Malaysia Genome and Vaccine Institute	Azrin Ahmad; Enizza Kasim; Irni Suhayu Sapian; Mohd Faizal Abu Bakar; Mohd Ghows Mohd Azzam.; Mohd Noor Mat Isa; Nor Azfa Johari; Nurhezreen Md Iqbal; Shamsidar Sopie; Siti Noraini Othman; Yusuf Muhammad Noor
EPI_ISL_2549576	Hospital Tuanku Fauziah	Institute for Medical Research, Infectious Disease Research Centre, National Institutes of Health, Ministry of Health Malaysia	Azizan MA; Kamel K; Mohd Zawawi Z; Ramly N; Robert F; Suppiah J; Thayan R
EPI_ISL_2620716	Hospital Tuanku Fauziah, Kangar	Institute for Medical Research, Infectious Disease Research Centre, National Institutes of Health, Ministry of Health	Azizan MA; Kamel K; Mohd Zawawi Z; Ramly N; Robert F; Suppiah J; Thayan R
EPI_ISL_2620715	Hospital Tuanku Fauziah, Kangar	Institute for Medical Research, Infectious Disease Research Centre, National Institutes of Health, Ministry of Health Malaysia	Azizan MA; Kamel K; Mohd Zawawi Z; Ramly N; Robert F; Suppiah J; Thayan R
EPI_ISL_2650003	Hospital Wanita Dan Kanak-Kanak Sabah	Institute for Medical Research, Infectious Disease Research Centre, National Institutes of Health, Ministry of Health Malaysia	40170 Selangor; Bandar Setia Alam; Jalan Setia Murni U13/52; Malaysia
EPI_ISL_2090886, EPI_ISL_2090887, EPI_ISL_2090889, EPI_ISL_2090890, EPI_ISL_2379433, EPI_ISL_2379443, EPI_ISL_2379650, EPI_ISL_2429134, EPI_ISL_2429136, EPI_ISL_2429137, EPI_ISL_2429138, EPI_ISL_2429141, EPI_ISL_2429142, EPI_ISL_2429143, EPI_ISL_2429145, EPI_ISL_2625633, EPI_ISL_2625635, EPI_ISL_2684231, EPI_ISL_2684232, EPI_ISL_2684233, EPI_ISL_2684545, EPI_ISL_2685359, EPI_ISL_2685766, EPI_ISL_2685835, EPI_ISL_2685836, EPI_ISL_2685837, EPI_ISL_2685838, EPI_ISL_2685839, EPI_ISL_2685840, EPI_ISL_2685841, EPI_ISL_2685843, EPI_ISL_2685844, EPI_ISL_2685845, EPI_ISL_2811887			
see above	Institute for Medical Research, Infectious Disease Research Centre, National Institutes of Health, Ministry of Health Malaysia	Institute for Medical Research, Infectious Disease Research Centre, National Institutes of Health, Ministry of Health Malaysia	Azizan MA; Kamel K; Mohd Zawawi Z; Ramly N; Robert F; Suppiah J; Thayan R
EPI_ISL_2650007, EPI_ISL_2683498	Ipoh Public Health Laboratory (MKAI), Ministry of Health Malaysia	Institute for Medical Research, Infectious Disease Research Centre, National Institutes of Health, Ministry of Health Malaysia	Azizan MA; Kamel K; Mohd Zawawi Z; Ramly N; Robert F; Suppiah J; Thayan R
EPI_ISL_2091022, EPI_ISL_2233384, EPI_ISL_2550714, EPI_ISL_2550731, EPI_ISL_2625621, EPI_ISL_2625624, EPI_ISL_2625625, EPI_ISL_2625626, EPI_ISL_2625627, EPI_ISL_2625628, EPI_ISL_2625629, EPI_ISL_2625630, EPI_ISL_2625631, EPI_ISL_2649996, EPI_ISL_2650256			
see above	Johor Bahru Public Health Laboratory	Institute for Medical Research, Infectious Disease Research Centre, National Institutes of Health, Ministry of Health Malaysia	Azizan MA; Kamel K; Mohd Zawawi Z; Ramly N; Robert F; Suppiah J; Thayan R
EPI_ISL_7972332, EPI_ISL_7972333, EPI_ISL_7972335, EPI_ISL_7972336, EPI_ISL_7972341, EPI_ISL_7972342, EPI_ISL_7972343, EPI_ISL_7972345, EPI_ISL_7972346			
see above	Kota Bharu Public Health Laboratory	Molecular Research Laboratory	Abdul Haris bin Muhammad; Ahmad Sukari Bin Halim; Alexander Chong Shu Chien; Aswini Leela; Azian Harun; Chan Yean Yean; Chua Wei Chuan; Chua Wei Lian; Engku Nur Syafirah bt Engku Abd Rahman; Kamarul Imran Musa; Kirnpal Kaur Banga Singh; Lau Nyok Sean; Lee Lih Huey; Lee Yeong Yeh; Lim Shu Yong; Maizun binti Mohd Zain; Muhammad Fazli bin Khalid; Muhammad Nashrul Farhan Samsudin; Muhammad Zarul Hanifah Bin Md Zoqratt; Najib Majdi Bin Yaacob; Nazmi Liana Binti Azmi; Noor Hafizan binti Mat Salleh; Nurfadhlina Musa; Qasim Ayub; Rosline Hassan; Sadequr Rahman; Syahida binti Omar; Wan Mohd Zahiruddin Wan Mohammad; Wilhelm Eng Wei Han; Zaini bin Hussin; Zakuan Zainy Deris
EPI_ISL_2649993	Kuala Lumpur Hospital	Institute for Medical Research, Infectious Disease Research Centre, National Institutes of Health, Ministry of Health Malaysia	Azizan MA; Kamel K; Mohd Zawawi Z; Ramly N; Robert F; Suppiah J; Thayan R
EPI_ISL_2811865, EPI_ISL_2811866, EPI_ISL_2811867, EPI_ISL_2811868, EPI_ISL_2811869, EPI_ISL_2811870	Kuala Lumpur International Healthcare Centre (KLIHC)	Institute for Medical Research, Infectious Disease Research Centre, National Institutes of Health, Ministry of Health Malaysia	Azizan MA; Kamel K; Mohd Zawawi Z; Ramly N; Robert F; Suppiah J; Thaya; Thayan R

EPI_ISL_3120236	Kuala Pilah Health District Office	Institute for Medical Research, Infectious Disease Research Centre, National Institutes of Health, Ministry of Health Malaysia	Anasir MI; Azizan MA; Kamel K; Mohd Zawawi Z; Ramly N; Robert F; Suppiah J; Thayan R
EPI_ISL_2649991	Kudat Hospital	Institute for Medical Research, Infectious Disease Research Centre, National Institutes of Health, Ministry of Health Malaysia	Azizan MA; Kamel K; Mohd Zawawi Z; Ramly N; Robert F; Suppiah J; Thayan R
EPI_ISL_2549741, EPI_ISL_2854048	Labuan Hospital	Institute for Medical Research, Infectious Disease Research Centre, National Institutes of Health, Ministry of Health Malaysia	Anasir MI; Azizan MA; Kamel K; Mohd Zawawi Z; Ramly N; Robert F; Suppiah J; Thayan R
EPI_ISL_2684234, EPI_ISL_2684235, EPI_ISL_2854049, EPI_ISL_2854050, EPI_ISL_2854051	Melaka Hospital	Institute for Medical Research, Infectious Disease Research Centre, National Institutes of Health, Ministry of Health Malaysia	Anasir MI; Azizan MA; Kamel K; Mohd Zawawi Z; Ramly N; Robert F; Suppiah J; Thayan R
EPI_ISL_2535778, EPI_ISL_2535779	Ministry of Health Hospitals	Institute of Health and Community Medicine	Chan Chia Jui; Chua Hock Hin; David Perera; Ooi Mong How; Tonnii Sia Loong Loong; Wong Jyn Shan; Wong Kiing Aik
EPI_ISL_2614740, EPI_ISL_2614741, EPI_ISL_2614742	Miri Hospital	Institute for Medical Research, Infectious Disease Research Centre, National Institutes of Health, Ministry of Health Malaysia	Azizan MA; Kamel K; Mohd Zawawi Z; Ramly N; Robert F; Suppiah J; Thayan R
EPI_ISL_2379748	National Heart Institute	Institute for Medical Research, Infectious Disease Research Centre, National Institutes of Health, Ministry of Health Malaysia	Azizan MA; Kamel K; Mohd Zawawi Z; Ramly N; Robert F; Suppiah J; Thayan R
EPI_ISL_2811871, EPI_ISL_2811873	Pasir Mas Town Health Clinic	Institute for Medical Research, Infectious Disease Research Centre, National Institutes of Health, Ministry of Health Malaysia	Azizan MA; Kamel K; Mohd Zawawi Z; Ramly N; Robert F; Suppiah J; Thayan R
EPI_ISL_2649998, EPI_ISL_2649999	Penang General Hospital	Institute for Medical Research, Infectious Disease Research Centre, National Institutes of Health, Ministry of Health Malaysia	Azizan MA; Kamel K; Mohd Zawawi Z; Ramly N; Robert F; Suppiah J; Thayan R
EPI_ISL_3247358, EPI_ISL_3247403	Perak State Health Department	Institute for Medical Research, Infectious Disease Research Centre, National Institutes of Health, Ministry of Health Malaysia	Anasir MI; Azizan MA; Kamel K; Mohd Zawawi Z; Ramly N; Robert F; Suppiah J; Thayan R
EPI_ISL_2550758, EPI_ISL_2550759	Port Dickson Hospital	Institute for Medical Research, Infectious Disease Research Centre, National Institutes of Health, Ministry of Health Malaysia	Azizan MA; Kamel K; Mohd Zawawi Z; Ramly N; Robert F; Suppiah J; Thayan R
EPI_ISL_2650027, EPI_ISL_2650430, EPI_ISL_2650432, EPI_ISL_2650499, EPI_ISL_2650505	Queen Elizabeth Hospital	Institute for Medical Research, Infectious Disease Research Centre, National Institutes of Health, Ministry of Health Malaysia	Azizan MA; Kamel K; Mohd Zawawi Z; Ramly N; Robert F; Suppiah J; Thayan R
EPI_ISL_2811886	Raja Perempuan Zainab II Hospital, Kota Bharu	Institute for Medical Research, Infectious Disease Research Centre, National Institutes of Health, Ministry of Health Malaysia	Azizan MA; Kamel K; Mohd Zawawi Z; Ramly N; Robert F; Suppiah J; Thayan R
EPI_ISL_2614745	Sarawak General Hospital	Institute for Medical Research, Infectious Disease Research Centre, National Institutes of Health, Ministry of Health Malaysia	Azizan MA; Kamel K; Mohd Zawawi Z; Ramly N; Robert F; Suppiah J; Thayan R
EPI_ISL_5052026, EPI_ISL_5052027, EPI_ISL_5052028, EPI_ISL_6967870, EPI_ISL_6967876, EPI_ISL_6967880, EPI_ISL_6967888, EPI_ISL_6967894, EPI_ISL_6967902, EPI_ISL_6967920, EPI_ISL_6967929, EPI_ISL_6970305, EPI_ISL_8650059			
see above	Sarawak General Hospital (Kuching)	Institute of Health and Community Medicine	Chan Chia Jui; Chua Hock Hin; David Perera; Ooi Mong How; Tonnii Sia Loong Loong; Wong Jyn Shan
EPI_ISL_2649995	Segamat Hospital	Institute for Medical Research, Infectious Disease Research Centre, National Institutes of Health, Ministry of Health Malaysia	Azizan MA; Kamel K; Mohd Zawawi Z; Ramly N; Robert F; Suppiah J; Thayan R
EPI_ISL_3071859	Selayang Hospital	Institute for Medical Research, Infectious Disease Research Centre, National Institutes of Health, Ministry of Health Malaysia	Anasir MI; Kamel K; Mohd Zawawi Z; Ramly N; Robert F; Suppiah J; Thayan R
EPI_ISL_2649997, EPI_ISL_2854107	Sultan Ismail Hospital	Institute for Medical Research, Infectious Disease Research Centre, National Institutes of Health, Ministry of Health Malaysia	Anasir MI; Azizan MA; Kamel K; Mohd Zawawi Z; Ramly N; Robert F; Suppiah J; Thayan R
EPI_ISL_2650028	Sultanah Aminah Hospital	Institute for Medical Research, Infectious Disease Research Centre, National Institutes of Health, Ministry of Health Malaysia	Azizan MA; Kamel K; Mohd Zawawi Z; Ramly N; Robert F; Suppiah J; Thayan R
EPI_ISL_2091024, EPI_ISL_2625637	Sultanah Aminah Hospital, Johor Bahru	Institute for Medical Research, Infectious Disease Research Centre, National Institutes of Health, Ministry of Health Malaysia	Azizan MA; Kamel K; Mohd Zawawi Z; Ramly N; Robert F; Suppiah J; Thayan R
EPI_ISL_2650014, EPI_ISL_2650015	Sultanah Bahiyah Hospital	Institute for Medical Research, Infectious Disease Research Centre, National Institutes of Health, Ministry of Health Malaysia	Azizan MA; Kamel K; Mohd Zawawi Z; Ramly N; Robert F; Suppiah J; Thayan R
EPI_ISL_2614739, EPI_ISL_2683247, EPI_ISL_2683496, EPI_ISL_2684236, EPI_ISL_2811885, EPI_ISL_2812654, EPI_ISL_2812704, EPI_ISL_2815656, EPI_ISL_2815971			
see above	Sultanah Bahiyah Hospital, Alor Setar	Institute for Medical Research, Infectious Disease Research Centre, National Institutes of Health, Ministry of Health Malaysia	Azizan MA; Kamel K; Mohd Zawawi Z; Ramly N; Robert F; Suppiah J; Thayan R
EPI_ISL_2811879, EPI_ISL_2811880, EPI_ISL_2811881, EPI_ISL_2811882, EPI_ISL_2811883	Sultanah Nora Ismail Hospital, Batu Pahat	Institute for Medical Research, Infectious Disease Research Centre, National Institutes of Health, Ministry of Health Malaysia	Azizan MA; Kamel K; Mohd Zawawi Z; Ramly N; Robert F; Suppiah J; Thayan R
EPI_ISL_2614743, EPI_ISL_2650004, EPI_ISL_2650005, EPI_ISL_2650010, EPI_ISL_2683956	Sungai Buloh Hospital	Institute for Medical Research, Infectious Disease Research Centre, National Institutes of Health, Ministry of Health Malaysia	Azizan MA; Kamel K; Mohd Zawawi Z; Ramly N; Robert F; Suppiah J; Thayan R
EPI_ISL_2379749, EPI_ISL_2650506, EPI_ISL_2854070, EPI_ISL_2854103, EPI_ISL_2854104, EPI_ISL_2854105, EPI_ISL_2854108, EPI_ISL_2854174, EPI_ISL_2854186			
see above	Tawau Hospital	Institute for Medical Research, Infectious Disease Research Centre, National Institutes of Health, Ministry of Health Malaysia	40170 Selangor; Anasir MI; Azizan MA; Bandar Setia Alam; Jalan Setia Murni U13/52; Kamel K; Malaysia; Mohd Zawawi Z; Ramly N; Robert F; Suppiah J; Thayan R
EPI_ISL_2811959	Tengku Ampuan Afzan Hospital (HTAA)	Institute for Medical Research, Infectious Disease Research Centre, National Institutes of Health, Ministry of Health Malaysia	Azizan MA; Kamel K; Mohd Zawawi Z; Ramly N; Robert F; Suppiah J; Thayan R
EPI_ISL_2614744, EPI_ISL_2649992	Tuanku Ampuan Najihah Hospital, Kuala Pilah	Institute for Medical Research, Infectious Disease Research Centre, National Institutes of Health, Ministry of Health Malaysia	Azizan MA; Kamel K; Mohd Zawawi Z; Ramly N; Robert F; Suppiah J; Thayan R
EPI_ISL_2649994	Tuanku Fauziah Hopsital	Institute for Medical Research, Infectious Disease Research Centre, National Institutes of Health, Ministry of Health Malaysia	Azizan MA; Kamel K; Mohd Zawawi Z; Ramly N; Robert F; Suppiah J; Thayan R
EPI_ISL_2650006, EPI_ISL_2811872	Tumpat Hospital	Institute for Medical Research, Infectious Disease Research Centre, National Institutes of Health, Ministry of Health Malaysia	Azizan MA; Kamel K; Mohd Zawawi Z; Ramly N; Robert F; Suppiah J; Thayan R
EPI_ISL_6132035, EPI_ISL_8254349	UMMC	Department of Medical Microbiology, Faculty of Medicine, University of Malaya; University of Malaya Medical Centre	I-Ching SAM; Jolene Yin Ling FU; Omar Khalilur Rahman; Yoke Fun Chan
EPI_ISL_2811875	Universiti Sains Malaysia Hospital	Institute for Medical Research, Infectious Disease Research Centre, National Institutes of Health, Ministry of Health Malaysia	Azizan MA; Kamel K; Mohd Zawawi Z; Ramly N; Robert F; Suppiah J; Thayan R

We gratefully acknowledge the following Authors from the Originating laboratories responsible for obtaining the specimens, as well as the Submitting laboratories where the genome data were generated and shared via GISAID, on which this research is based.

All Submitters of data may be contacted directly via www.gisaid.org

Authors are sorted alphabetically.

Acknowledgement EPI_SET Identifier: EPI_SET_20220603dv

Accession ID	Originating Laboratory	Submitting Laboratory	Authors
EPI_ISL_2854052	Ampang Hospital	Institute for Medical Research, Infectious Disease Research Centre, National Institutes of Health, Ministry of Health Malaysia	Anasir MI; Azizan MA; Kamel K; Mohd Zawawi Z; Ramly N; Robert F; Suppiah J; Thayan R
EPI_ISL_3266062	Asia Muar Clinic	Institute for Medical Research, Infectious Disease Research Centre, National Institutes of Health, Ministry of Health Malaysia	Anasir MI; Azizan MA; Kamel K; Mohd Zawawi Z; Ramly N; Robert F; Suppiah J; Thayan R
EPI_ISL_4367711, EPI_ISL_4368189	BIOTROPIK, Universiti Malaysia Pahang, Malaysia	BIOTROPIK, Universiti Malaysia Pahang, Malaysia	Hajar Fauzan Bin Ahmad
EPI_ISL_2811998	Bandar Kuantan Health Clinic	Institute for Medical Research, Infectious Disease Research Centre, National Institutes of Health, Ministry of Health Malaysia	Azizan MA; Kamel K; Mohd Zawawi Z; Ramly N; Robert F; Suppiah J; Thayan R
EPI_ISL_4460123, EPI_ISL_4460124, EPI_ISL_4460125, EPI_ISL_4460126, EPI_ISL_4460127	Borneo Medical Centre	Institute of Health and Community Medicine	Chan Chia Jui; Chua Hock Hin; David Perera; Ooi Mong How; Tonnii Sia Loong Loong; Wong Jyn Shan
EPI_ISL_6713042	COVID WARD HPP	iPROMISE, UiTM	Ariza Adnan; Fadzilah Mohd Nor; Lim Wai Feng; Mohd Asif Mohd Sukri; Mohd Nur Fakhruzzaman Noorizhab; Mohd Zaki Salleh; Sazzli Shahlan Kassim; Siti Farah Alwani Mohd Nawi; Siti Hamimah Sheikh Abdul Kadir; Teh Lay Kek; Wang Seok Mui
EPI_ISL_3374151	Cameron Highlands District Health Office	Institute for Medical Research, Infectious Disease Research Centre, National Institutes of Health, Ministry of Health Malaysia	Anasir MI; Azizan MA; Kamel K; Mohd Zawawi Z; Ramly N; Robert F; Suppiah J; Thayan R
EPI_ISL_3374233	Cheras District Health Office	Institute for Medical Research, Infectious Disease Research Centre, National Institutes of Health, Ministry of Health Malaysia	Anasir MI; Azizan MA; Kamel K; Mohd Zawawi Z; Ramly N; Robert F; Suppiah J; Thayan R
EPI_ISL_5782336, EPI_ISL_5782340, EPI_ISL_5782398, EPI_ISL_5782402, EPI_ISL_5782404	Department of Medical Microbiology, Hospital Pengajar Universiti Putra Malaysia	Department of Medical Microbiology, Hospital Pengajar Universiti Putra Malaysia	Afiqah Adzmi; Amiza Azmi; Azmiza Syawani Jasni; Chee Hui Yee; Leslie Than Thian Lung; Muadz Mohtar; Muhammad Mohd Isa; Narcisse MS Joseph; Niazlin Mohd Taib; Noor Hazirah Noor Azhari; Norlaila; Nur Raihana Ithnin; Nurul Huda Mohamed Rashidi; Nurul Nadiah Ismail; Rosni Ibrahim; Sallehhudin; Siti Norbaya Masri; Siti Zulaikha Zakariah; Suppiah J; Syafinaz Amin Nordin; Tengku Zetty Maztura Tengku Jamaluddin; Thayan R; Zamberi Sekawi
EPI_ISL_3162223	Department of Medical Microbiology, Hospital Pengajar Universiti Putra Malaysia	Malaysia Genome Institute	Avisha Richards; Azrin Ahmad; Enizza Kasim; Hui-Yee Chee; Irni Suhayu Sapian; Mohd Anuar Jonet; Mohd Faizal Abu Bakar; Mohd Noor Mat Isa; Muhammad MI; Narcisse Joseph; Nor Azfa Johari; Nor Zahrin Hasran; Nurhezreen Md Iqbal; Shamsidar Sopie; Siti Noraini Othman; Syafinaz Amin-Nordin; Yusuf Muhammad Noor
EPI_ISL_4056092, EPI_ISL_4056093, EPI_ISL_4056094, EPI_ISL_4056095, EPI_ISL_4056096, EPI_ISL_4056097, EPI_ISL_4056098, EPI_ISL_4056099, EPI_ISL_4056100, EPI_ISL_4056101, EPI_ISL_4056102, EPI_ISL_4056103, EPI_ISL_4056104, EPI_ISL_4056105, EPI_ISL_4056106, EPI_ISL_4056107, EPI_ISL_4056112, EPI_ISL_4056115, EPI_ISL_4056118, EPI_ISL_4056122, EPI_ISL_4056123, EPI_ISL_4056128, EPI_ISL_4056131, EPI_ISL_4056133, EPI_ISL_4056134, EPI_ISL_4056135, EPI_ISL_4056140, EPI_ISL_4056143, EPI_ISL_4056148, EPI_ISL_4056152, EPI_ISL_4056153, EPI_ISL_4056155, EPI_ISL_4056156, EPI_ISL_4056157, EPI_ISL_4056158, EPI_ISL_4056161, EPI_ISL_4056162, EPI_ISL_4056165, EPI_ISL_4056166, EPI_ISL_4056170, EPI_ISL_4056174, EPI_ISL_4056175, EPI_ISL_4056176, EPI_ISL_4056177, EPI_ISL_4071805			
see above	Department of Medical Microbiology, University Malaya Medical Centre	Department of Medical Microbiology, Faculty of Medicine, University of Malaya	I-Ching SAM; Izzati Kausar; Jolene Yin Ling FU; Yoke Fun Chan; Yoong Min CHONG
EPI_ISL_5417146	Department of Medical Microbiology, University Malaya Medical Centre	Department of Medical Microbiology, University Malaya Medical Centre	I-Ching SAM; Jolene Yin Ling FU; Omar Khalilur Rahman; Yoke Fun Chan
EPI_ISL_3221325, EPI_ISL_3239916, EPI_ISL_3241561	Department of Pathology & Laboratory Medicine	Centre for Research in Advanced Tropical Bioscience (Biotropic Centre)	Hajar Fauzan Ahmad; How Soon Hin and Hajar Fauzan Ahmad; Mohd Nazli Kamarulzaman; Norhidayah Kamarudin; Ummu Afeera Zainulabid
EPI_ISL_3221322, EPI_ISL_3221323	Department of Pathology and Laboratory Medicine, Kulliyyah of Medicine, International Islamic University of Malaysia	Centre for Research in Advanced Tropical Bioscience (Biotropic Centre)	Hajar Fauzan Bin Ahmad; How Soon Hin and Hajar Fauzan Ahmad; Norhidayah Kamarudin; Ummu Afeera Zainulabid
EPI_ISL_3334218	Gleneagles Hospital Medini Johor	Institute for Medical Research, Infectious Disease Research Centre, National Institutes of Health, Ministry of Health Malaysia	Anasir MI; Azizan MA; Kamel K; Mohd Zawawi Z; Ramly N; Robert F; Suppiah J; Thayan R
EPI_ISL_3333232	Gribbles Pathology	Institute for Medical Research, Infectious Disease Research Centre, National Institutes of Health, Ministry of Health Malaysia	Anasir MI; Azizan MA; Kamel K; Mohd Zawawi Z; Ramly N; Robert F; Suppiah J; Thayan R
EPI_ISL_3333233	Gribbles Pathology (M) Sdn Bhd	Institute for Medical Research, Infectious Disease Research Centre, National Institutes of Health, Ministry of Health Malaysia	Anasir MI; Azizan MA; Kamel K; Mohd Zawawi Z; Ramly N; Robert F; Suppiah J; Thayan R
EPI_ISL_3980753	HRPB, Ipoh	Tropical Infectious Diseases Research & Education Centre (TIDREC), Universiti Malaya	AsmaAnati CheMatSeri; Che-Norainon Yaacob; Jia-Yi Tan; Jo-Ern Wong; Kim-Kee Tan; Mulya-Mustika-Sari Zulkifli; Noor Syahida Azizan; Nur-Hidayana Mahfodz; Sazaly AbuBakar; Siti-Sarah Nor'e
EPI_ISL_2727836	Hospital Angkatan Tentera Kota Kinabalu	Institute for Medical Research, Infectious Disease Research Centre, National Institutes of Health, Ministry of Health Malaysia	Azizan MA; Kamel K; Mohd Zawawi Z; Ramly N; Robert F; Suppiah J; Thayan R
EPI_ISL_2727837	Hospital Angkatan Tentera Wilayah Kota Kinabalu	Institute for Medical Research, Infectious Disease Research Centre, National Institutes of Health, Ministry of Health Malaysia	Azizan MA; Kamel K; Mohd Zawawi Z; Ramly N; Robert F; Suppiah J; Thayan R
EPI_ISL_5428560, EPI_ISL_5428561, EPI_ISL_5428562, EPI_ISL_6825263, EPI_ISL_6825264, EPI_ISL_6825265, EPI_ISL_6825266, EPI_ISL_6825267, EPI_ISL_6825268, EPI_ISL_6825269, EPI_ISL_6825270, EPI_ISL_6825271, EPI_ISL_6825272, EPI_ISL_6825317, EPI_ISL_7260006			
see above	Hospital Canselor Tuanku Muhriz UKM	Malaysia Genome Institute	Abdul Hamydy A Hamid; Ainihayati Noordin; Anita Sulong; Asiyah Nordin; Azrin Ahmad; Badrul AH Yusoff; Enizza Kasim; Fatin Farahani Ramzah; Habib AH Esa; Haslina Mahbob; Irni Suhayu Sapian; Jauhary Effendy Jumaat; Kon Ken Wong; Marjmin Osman; Mohd Faizal Abu Bakar; Mohd Noor Mat Isa; Muhammad Faiz Mohd Ali; Muttaqillah Najihan A Samat; Najma Kori; Noor Hamiza Ghulam; Nor Maslini Ismail; Nordiana Ismail; Nur Sahairah Awang; Nurhezreen Md Iqbal; Petrick Periasamy; Shamsidar Sopie; Siti Noraini Othman; Siti Norlia Othman; Siti Nurazizah M Asripin; Umi Kalsom Ali; Valerie SM Ting; Wan NH Wan Ghazali; Yusuf Muhammad Noor; Zetti Zainol Rashid
EPI_ISL_8745614, EPI_ISL_8745615, EPI_ISL_8745616, EPI_ISL_8745617, EPI_ISL_8745618, EPI_ISL_8745621	Hospital Canselor Tuanku Muhriz UKM	Malaysia Genome and Vaccine Institute	Apical Scientific Sdn Bhd's Team; Azrin Ahmad; Carey Wee Kai Li; Enizza Kasim; Irni Suhayu Sapian; Jilian Michelle Wong Tzeling; Leong Wai Mun; Mohd Faizal Abu Bakar; Mohd Ghows Mohd Azzam.; Mohd Noor Mat Isa; Nor Azfa Johari; Nurhezreen Md Iqbal; Rose Iszati Ismet Nayan; Shamsidar Sopie; Siti Noraini Othman; Yusuf Muhammad Noor
EPI_ISL_5742659, EPI_ISL_5742665, EPI_ISL_5742675, EPI_ISL_5742693, EPI_ISL_5742697	INSTITUT BIOLOGI MOLEKUL PERUBATAN UKM (UMBI)	UKM Medical Molecular Biology Institute (UMBI)	Mira Farzana binti Mohamad Mokhtar
EPI_ISL_4071977, EPI_ISL_4071980, EPI_ISL_4816832, EPI_ISL_4816833, EPI_ISL_4816834	Institut Biologi Molekul Perubatan UKM	UKM Medical Molecular Biology Institute (UMBI)	Mira Farzana binti Mohamad Mokhtar
EPI_ISL_2815375, EPI_ISL_2819024, EPI_ISL_2819571, EPI_ISL_2819919, EPI_ISL_3247527, EPI_ISL_3247528, EPI_ISL_3247529, EPI_ISL_3247700, EPI_ISL_3247768, EPI_ISL_3247769, EPI_ISL_3247770, EPI_ISL_3247771, EPI_ISL_3247772			
see above	Institute for Medical Research, Infectious Disease Research Centre, National Institutes of Health, Ministry of Health Malaysia	Institute for Medical Research, Infectious Disease Research Centre, National Institutes of Health, Ministry of Health Malaysia	Anasir MI; Azizan MA; Kamel K; Mohd Zawawi Z; Ramly N; Robert F; Suppiah J; Thayan R
EPI_ISL_3071986, EPI_ISL_3071987, EPI_ISL_3100296, EPI_ISL_3100297, EPI_ISL_3100681, EPI_ISL_3100682, EPI_ISL_3100981			
see above	Ipoh Public Health Laboratory	Institute for Medical Research, Infectious Disease Research Centre, National Institutes of Health, Ministry of Health Malaysia	Anasir MI; Azizan MA; Kamel K; Mohd Zawawi Z; Ramly N; Robert F; Suppiah J; Thayan R
EPI_ISL_2815329, EPI_ISL_2815330, EPI_ISL_2921400, EPI_ISL_3334021, EPI_ISL_3334182	Ipoh Public Health Laboratory (MKAI), Ministry of Health Malaysia	Institute for Medical Research, Infectious Disease Research Centre, National Institutes of Health, Ministry of Health Malaysia	Anasir MI; Azizan MA; Kamel K; Mohd Zawawi Z; Ramly N; Robert F; Suppiah J; Thayan R
EPI_ISL_3769363	JKN PAHANG	iPROMISE, UiTM	Ariza Adnan; Fadzilah Mohd Nor; Lim Wai Feng; Mohd Asif Mohd Sukri; Mohd Nur Fakhruzzaman Noorizhab; Mohd Zaki Salleh; Sazzli Shahlan Kassim; Siti Farah Alwani Mohd Nawi; Siti Hamimah Sheikh Abdul Kadir; Teh Lay Kek; Wang Seok Mui
EPI_ISL_3100124, EPI_ISL_3100125, EPI_ISL_3100298, EPI_ISL_3100465, EPI_ISL_3100679, EPI_ISL_3100680, EPI_ISL_3374152, EPI_ISL_5114752, EPI_ISL_5114756, EPI_ISL_5418052			
see above	Johor Bahru Public Health Laboratory	Institute for Medical Research, Infectious	Ahmad FA; Ahmad Fazilah NA; Anasir MI; Azizan MA; Kamel K; Mohd Zawawi Z; Norhisham SN; Ramly N; Robert F; Suppiah J; Thayan R

		Disease Research Centre, National Institutes of Health, Ministry of Health Malaysia	
EPI_ISL_2757930, EPI_ISL_2757951, EPI_ISL_2812981	Johor State Health Department	Institute for Medical Research, Infectious Disease Research Centre, National Institutes of Health, Ministry of Health Malaysia	Azizan MA; Kamel K; Mohd Zawawi Z; Ramly N; Robert F; Suppiah J; Thayan R
EPI_ISL_7086768	KK KUAH	Department of Medical Microbiology, Faculty of Medicine, University of Malaya; University of Malaya Medical Centre	I-Ching SAM; Jolene Yin Ling FU; Omar Khalilur Rahman; Yoke Fun Chan
EPI_ISL_7086707, EPI_ISL_7087061	KLINIK KESIHATAN KUAH	Department of Medical Microbiology, Faculty of Medicine, University of Malaya; University of Malaya Medical Centre	I-Ching SAM; Jolene Yin Ling FU; Omar Khalilur Rahman; Yoke Fun Chan
EPI_ISL_3333234, EPI_ISL_4101561, EPI_ISL_4101562, EPI_ISL_4730419, EPI_ISL_4730421	Kajang Hospital	Institute for Medical Research, Infectious Disease Research Centre, National Institutes of Health, Ministry of Health Malaysia	Ahmad FA; Ahmad Fazilah NA; Anasir MI; Azizan MA; Kamel K; Mohd Zawawi Z; Norhisham SN; Norhisyam SN; Ramly N; Robert F; Suppiah J; Thayan R
EPI_ISL_3050137	Kapit Hospital	Institute for Medical Research, Infectious Disease Research Centre, National Institutes of Health, Ministry of Health Malaysia	Anasir MI; Azizan MA; Kamel K; Mohd Zawawi Z; Ramly N; Robert F; Suppiah J; Thayan R
EPI_ISL_2812576, EPI_ISL_2854053	Kemaman Hospital	Institute for Medical Research, Infectious Disease Research Centre, National Institutes of Health, Ministry of Health Malaysia	Anasir MI; Azizan MA; Kamel K; Mohd Zawawi Z; Ramly N; Robert F; Suppiah J; Thayan R
EPI_ISL_3723542	Kepala Batas Hospital	Institute for Medical Research, Infectious Disease Research Centre, National Institutes of Health, Ministry of Health Malaysia	Anasir MI; Azizan MA; Kamel K; Mohd Zawawi Z; Ramly N; Robert F; Suppiah J; Thayan R
EPI_ISL_3333236, EPI_ISL_3333581, EPI_ISL_3333593	Kota Bharu Public Health Laboratory	Institute for Medical Research, Infectious Disease Research Centre, National Institutes of Health, Ministry of Health Malaysia	Anasir MI; Azizan MA; Kamel K; Mohd Zawawi Z; Ramly N; Robert F; Suppiah J; Thayan R
EPI_ISL_2815331, EPI_ISL_8404630	Kuala Lumpur General Hospital	Institute for Medical Research, Infectious Disease Research Centre, National Institutes of Health, Ministry of Health Malaysia	Ahmad FA; Ahmad Fazilah NA; Anasir MI; Azizan MA; Kamel K; Mohamad Sukri MZ; Mohd Zawawi Z; Norhisham SN; Ramly N; Robert F; Rosli NR; Suppiah J; Thayan R
EPI_ISL_3071860, EPI_ISL_3101006	Kuala Lumpur Hospital	Institute for Medical Research, Infectious Disease Research Centre, National Institutes of Health, Ministry of Health Malaysia	Anasir MI; Azizan MA; Kamel K; Mohd Zawawi Z; Ramly N; Robert F; Suppiah J; Thayan R
EPI_ISL_3071988	Kuala Pilah District Health Office	Institute for Medical Research, Infectious Disease Research Centre, National Institutes of Health, Ministry of Health Malaysia	Anasir MI; Kamel K; Mohd Zawawi Z; Ramly N; Robert F; Suppiah J; Thayan R
EPI_ISL_2854187	Kuala Terengganu District Health Office	Institute for Medical Research, Infectious Disease Research Centre, National Institutes of Health, Ministry of Health Malaysia	Anasir MI; Azizan MA; Kamel K; Mohd Zawawi Z; Ramly N; Robert F; Suppiah J; Thayan R
EPI_ISL_3221324	Kulliyyah of Medicine, International Islamic University Malaysia, Bandar Indera Mahkota Campus, Jalan Sultan Ahmad Shah, 25200 Kuantan, Pahang Darul Makmur	Centre for Research in Advanced Tropical Bioscience (Biotropic Centre)	How Soon Hin and Hajar Fauzan Ahmad; Norhidayah Kamarudin; Ummu Afeera Zainulabid
EPI_ISL_3334843, EPI_ISL_3355465	Lablink (M) Sdn. Bhd	Institute for Medical Research, Infectious Disease Research Centre, National Institutes of Health, Ministry of Health Malaysia	Anasir MI; Azizan MA; Kamel K; Mohd Zawawi Z; Ramly N; Robert F; Suppiah J; Thayan R
EPI_ISL_3355464	Lablink (M) Sdn. Bhd.	Institute for Medical Research, Infectious Disease Research Centre, National Institutes of Health, Ministry of Health Malaysia	Anasir MI; Azizan MA; Kamel K; Mohd Zawawi Z; Ramly N; Robert F; Suppiah J; Thayan R
EPI_ISL_2757931, EPI_ISL_2812651, EPI_ISL_2812971, EPI_ISL_2921610, EPI_ISL_2921611, EPI_ISL_2924057, EPI_ISL_2931921			
see above	Labuan Hospital	Institute for Medical Research, Infectious Disease Research Centre, National Institutes of Health, Ministry of Health Malaysia	Anasir MI; Azizan MA; Kamel K; Mohd Zawawi Z; Ramly N; Robert F; Suppiah J; Thayan R
EPI_ISL_2854068	Lahad Datu Hospital	Institute for Medical Research, Infectious Disease Research Centre, National Institutes of Health, Ministry of Health Malaysia	Anasir MI; Azizan MA; Kamel K; Mohd Zawawi Z; Ramly N; Robert F; Suppiah J; Thayan R
EPI_ISL_3120112, EPI_ISL_3120113	Lembah Pantai Health Office	Institute for Medical Research, Infectious Disease Research Centre, National Institutes of Health, Ministry of Health Malaysia	Anasir MI; Azizan MA; Kamel K; Mohd Zawawi Z; Ramly N; Robert F; Suppiah J; Thayan R
EPI_ISL_2854188	Marang District Health Office	Institute for Medical Research, Infectious Disease Research Centre, National Institutes of Health, Ministry of Health Malaysia	Anasir MI; Azizan MA; Kamel K; Mohd Zawawi Z; Ramly N; Robert F; Suppiah J; Thayan R
EPI_ISL_2814916, EPI_ISL_2815327, EPI_ISL_2815328, EPI_ISL_2921996, EPI_ISL_3071855, EPI_ISL_3071856, EPI_ISL_3266063, EPI_ISL_3266064, EPI_ISL_3266065			
see above	Melaka Hospital	Institute for Medical Research, Infectious Disease Research Centre, National Institutes of Health, Ministry of Health Malaysia	Anasir MI; Azizan MA; Kamel K; Mohd Zawawi Z; Ramly N; Robert F; Suppiah J; Thayan R
EPI_ISL_4460128, EPI_ISL_4460129, EPI_ISL_4460130, EPI_ISL_4460131, EPI_ISL_4460132, EPI_ISL_4460133, EPI_ISL_4460134, EPI_ISL_4460135, EPI_ISL_4460136, EPI_ISL_4460137, EPI_ISL_4460138, EPI_ISL_4460139, EPI_ISL_4460140, EPI_ISL_4460393, EPI_ISL_4460394, EPI_ISL_4460395			
see above	Ministry of Health Hospitals	Institute of Health and Community Medicine	Chan Chia Jui; Chua Hock Hin; David Perera; Ooi Mong How; Tonnii Sia Loong Loong; Wong Jyn Shan; Wong Kiing Aik
EPI_ISL_3266032	Muar Clinic	Institute for Medical Research, Infectious Disease Research Centre, National Institutes of Health, Ministry of Health Malaysia	Anasir MI; Azizan MA; Kamel K; Mohd Zawawi Z; Ramly N; Robert F; Suppiah J; Thayan R
EPI_ISL_3334190, EPI_ISL_3334217	National Public Health Laboratory, Sungai Buloh	Institute for Medical Research, Infectious Disease Research Centre, National Institutes of Health, Ministry of Health Malaysia	Anasir MI; Azizan MA; Kamel K; Mohd Zawawi Z; Ramly N; Robert F; Suppiah J; Thayan R
EPI_ISL_4122383, EPI_ISL_4122394, EPI_ISL_4122479, EPI_ISL_4122486	PEJ KESIHATAN BARAT DAYA	iPROMISE, UiTM	Ariza Adnan; Fadzilah Mohd Nor; Lim Wai Feng; Mohd Asif Mohd Sukri; Mohd Nur Fakhruzzaman Noorizhab; Mohd Zaki Salleh; Sazzli Shahlan Kassim; Siti Farah Alwani Mohd Nawi; Siti Hamimah Sheikh Abdul Kadir; Teh Lay Kek; Wang Seok Mui
EPI_ISL_2921263, EPI_ISL_2921264, EPI_ISL_2921265, EPI_ISL_2921266, EPI_ISL_5852771	Pahang State Health Department	Institute for Medical Research, Infectious Disease Research Centre, National Institutes of Health, Ministry of Health Malaysia	Ahmad FA; Ahmad Fazilah NA; Anasir MI; Azizan MA; Kamel K; Mohd Zawawi Z; Norhisham SN; Ramly N; Robert F; Suppiah J; Thayan R
EPI_ISL_2921262	Pantai Hospital Kuala Lumpur	Institute for Medical Research, Infectious Disease Research Centre, National Institutes of Health, Ministry of Health Malaysia	Anasir MI; Azizan MA; Kamel K; Mohd Zawawi Z; Ramly N; Robert F; Suppiah J; Thayan R
EPI_ISL_8147383, EPI_ISL_8147384, EPI_ISL_8147385, EPI_ISL_8147386, EPI_ISL_8147387, EPI_ISL_8147388, EPI_ISL_8147389, EPI_ISL_8147390			
see above	Pejabat Kesihatan Daerah Lembah Pantai	Tropical Infectious Diseases Research & Education Centre (TIDREC), Universiti Malaya	AsmaAnati CheMatSeri; Che-Norainon Yaacob; Jia-Yi Tan; Jo-Ern Wong; Kim-Kee Tan; Mulya-Mustika-Sari Zulkifli; Noor Syahida Azizan; Nur-Hidayana Mahfodz; Sazaly AbuBakar; Siti-Sarah Nor'e
EPI_ISL_8147381, EPI_ISL_8170432, EPI_ISL_8170433	Pejabat Kesihatan Daerah Melaka Tengah	Tropical Infectious Diseases Research & Education Centre (TIDREC), Universiti Malaya	AsmaAnati CheMatSeri; Che-Norainon Yaacob; Jia-Yi Tan; Jo-Ern Wong; Kim-Kee Tan; Mulya-Mustika-Sari Zulkifli; Noor Syahida Azizan; Nur-Hidayana Mahfodz; Sazaly AbuBakar; Siti-Sarah Nor'e
EPI_ISL_2811984	Pekan Tajau Health Clinic	Institute for Medical Research, Infectious Disease Research Centre, National Institutes of Health, Ministry of Health Malaysia	Azizan MA; Kamel K; Mohd Zawawi Z; Ramly N; Robert F; Suppiah J; Thayan R
EPI_ISL_3723541, EPI_ISL_3723543, EPI_ISL_3723544	Penang General Hospital	Institute for Medical Research, Infectious Disease Research Centre, National Institutes of Health, Ministry of Health Malaysia	Anasir MI; Azizan MA; Kamel K; Mohd Zawawi Z; Ramly N; Robert F; Suppiah J; Thayan R
EPI_ISL_3762773, EPI_ISL_3762789, EPI_ISL_3762790	Penang State Health Department	Institute for Medical Research, Infectious Disease Research Centre, National Institutes of Health, Ministry of Health Malaysia	Anasir MI; Azizan MA; Kamel K; Mohd Zawawi Z; Ramly N; Robert F; Suppiah J; Thayan R

EPI_ISL_3762829	Port Dickson District Health Office	Institute for Medical Research, Infectious Disease Research Centre, National Institutes of Health, Ministry of Health Malaysia	Anasir MI; Azizan MA; Kamel K; Mohd Zawawi Z; Ramly N; Robert F; Suppiah J; Thayan R
EPI_ISL_3374154, EPI_ISL_3374708	Putrajaya Health Office	Institute for Medical Research, Infectious Disease Research Centre, National Institutes of Health, Ministry of Health Malaysia	Anasir MI; Azizan MA; Kamel K; Mohd Zawawi Z; Ramly N; Robert F; Suppiah J; Thayan R
EPI_ISL_2854106	Queen Elizabeth Hospital	Institute for Medical Research, Infectious Disease Research Centre, National Institutes of Health, Ministry of Health Malaysia	Anasir MI; Azizan MA; Kamel K; Mohd Zawawi Z; Ramly N; Robert F; Suppiah J; Thayan R
EPI_ISL_2757941, EPI_ISL_2757942, EPI_ISL_2931924, EPI_ISL_3374470	Queen Elizabeth Hospital, Kota Kinabalu	Institute for Medical Research, Infectious Disease Research Centre, National Institutes of Health, Ministry of Health Malaysia	Anasir MI; Azizan MA; Kamel K; Mohd Zawawi Z; Ramly N; Robert F; Suppiah J; Thayan R
EPI_ISL_3120110	Raja Perempuan Zainab II Hospital	Institute for Medical Research, Infectious Disease Research Centre, National Institutes of Health, Ministry of Health Malaysia	Anasir MI; Azizan MA; Kamel K; Mohd Zawawi Z; Ramly N; Robert F; Suppiah J; Thayan R
EPI_ISL_3762496	Raja Permaisuri Bainun Hospital	Institute for Medical Research, Infectious Disease Research Centre, National Institutes of Health, Ministry of Health Malaysia	Anasir MI; Azizan MA; Kamel K; Mohd Zawawi Z; Ramly N; Robert F; Suppiah J; Thayan R
EPI_ISL_2812026, EPI_ISL_2812027, EPI_ISL_2812028	Rembau Health Clinic	Institute for Medical Research, Infectious Disease Research Centre, National Institutes of Health, Ministry of Health Malaysia	Azizan MA; Kamel K; Mohd Zawawi Z; Ramly N; Robert F; Suppiah J; Thayan R
EPI_ISL_5051747, EPI_ISL_5051750, EPI_ISL_5051751, EPI_ISL_5051752, EPI_ISL_5051755, EPI_ISL_5052001, EPI_ISL_5052002, EPI_ISL_5052015, EPI_ISL_5052086, EPI_ISL_5052087, EPI_ISL_5052088, EPI_ISL_5052089, EPI_ISL_5052090, EPI_ISL_5052091, EPI_ISL_5052092, EPI_ISL_5052179, EPI_ISL_5052181, EPI_ISL_5052185, EPI_ISL_5052186, EPI_ISL_5052187, EPI_ISL_5052188, EPI_ISL_5052189, EPI_ISL_5052190, EPI_ISL_5052191, EPI_ISL_5052192, EPI_ISL_5052193, EPI_ISL_5052194, EPI_ISL_6967907, EPI_ISL_6967914, EPI_ISL_6967934, EPI_ISL_6967940, EPI_ISL_6967942, EPI_ISL_6967946, EPI_ISL_6967957, EPI_ISL_6967968, EPI_ISL_6967970, EPI_ISL_6967980, EPI_ISL_6967988, EPI_ISL_6967999, EPI_ISL_6968005, EPI_ISL_6968021, EPI_ISL_6968032, EPI_ISL_6970293, EPI_ISL_6970298, EPI_ISL_6970313, EPI_ISL_6970323, EPI_ISL_6970332, EPI_ISL_6970337, EPI_ISL_6970341, EPI_ISL_6970429, EPI_ISL_6970438, EPI_ISL_6970444			
see above	Sarawak General Hospital (Kuching)	Institute of Health and Community Medicine	Chan Chia Jui; Chua Hock Hin; David Perera; Ooi Mong How; Tonnii Sia Loong Loong; Wong Jyn Shan; Wong Kiing Aik
EPI_ISL_8650071, EPI_ISL_8650072, EPI_ISL_8650073, EPI_ISL_8650074	Sarawak Heart Centre (SHC), Kota Samarahan	Institute of Health and Community Medicine	Chan Chia Jui; Chua Hock Hin; David Perera; Ooi Mong How; Tonnii Sia Loong Loong; Wong Jyn Shan
EPI_ISL_3120111	Selayang Hospital	Institute for Medical Research, Infectious Disease Research Centre, National Institutes of Health, Ministry of Health Malaysia	Anasir MI; Azizan MA; Kamel K; Mohd Zawawi Z; Ramly N; Robert F; Suppiah J; Thayan R
EPI_ISL_3333235, EPI_ISL_3356282, EPI_ISL_3356283, EPI_ISL_3356352	Serdang Hospital	Institute for Medical Research, Infectious Disease Research Centre, National Institutes of Health, Ministry of Health Malaysia	Anasir MI; Azizan MA; Kamel K; Mohd Zawawi Z; Ramly N; Robert F; Suppiah J; Thayan R
EPI_ISL_3050258, EPI_ISL_3278292	Shah Alam Hospital	Institute for Medical Research, Infectious Disease Research Centre, National Institutes of Health, Ministry of Health Malaysia	Anasir MI; Azizan MA; Kamel K; Mohd Zawawi Z; Ramly N; Robert F; Suppiah J; Thayan R
EPI_ISL_3723540, EPI_ISL_5398318, EPI_ISL_5398926	Sibu Hospital	Institute for Medical Research, Infectious Disease Research Centre, National Institutes of Health, Ministry of Health Malaysia	Ahmad FA; Ahmad Fazilah NA; Anasir MI; Azizan MA; Kamel K; Mohd Zawawi Z; Norhisham SN; Ramly N; Robert F; Suppiah J; Thayan R
EPI_ISL_3333563	Slim River Hospital	Institute for Medical Research, Infectious Disease Research Centre, National Institutes of Health, Ministry of Health Malaysia	Anasir MI; Azizan MA; Kamel K; Mohd Zawawi Z; Ramly N; Robert F; Suppiah J; Thayan R
EPI_ISL_3120116	Sultan Abdul Halim Hospital	Institute for Medical Research, Infectious Disease Research Centre, National Institutes of Health, Ministry of Health Malaysia	Anasir MI; Azizan MA; Kamel K; Mohd Zawawi Z; Ramly N; Robert F; Suppiah J; Thayan R
EPI_ISL_2812706, EPI_ISL_2921612, EPI_ISL_2924137, EPI_ISL_2924139	Sultan Haji Ahmad Shah Hospital	Institute for Medical Research, Infectious Disease Research Centre, National Institutes of Health, Ministry of Health Malaysia	Anasir MI; Azizan MA; Kamel K; Mohd Zawawi Z; Ramly N; Robert F; Suppiah J; Thayan R
EPI_ISL_3278301, EPI_ISL_3278302	Sultanah Bahiyah Hospital	Institute for Medical Research, Infectious Disease Research Centre, National Institutes of Health, Ministry of Health Malaysia	Anasir MI; Azizan MA; Kamel K; Mohd Zawawi Z; Ramly N; Robert F; Suppiah J; Thayan R
EPI_ISL_3100982, EPI_ISL_3120143, EPI_ISL_3120314, EPI_ISL_3333237, EPI_ISL_3333657, EPI_ISL_3333929, EPI_ISL_3374484			
see above	Sultanah Bahiyah Hospital, Alor Setar	Institute for Medical Research, Infectious Disease Research Centre, National Institutes of Health, Ministry of Health Malaysia	Anasir MI; Azizan MA; Kamel K; Mohd Zawawi Z; Ramly N; Robert F; Suppiah J; Thayan R
EPI_ISL_2931920, EPI_ISL_2931923	Sultanah Fatimah Specialist Hospital, Muar	Institute for Medical Research, Infectious Disease Research Centre, National Institutes of Health, Ministry of Health Malaysia	Anasir MI; Azizan MA; Kamel K; Mohd Zawawi Z; Ramly N; Robert F; Suppiah J; Thayan R
EPI_ISL_2650000, EPI_ISL_3278291	Sultanah Nur Zahirah Hospital	Institute for Medical Research, Infectious Disease Research Centre, National Institutes of Health, Ministry of Health Malaysia	Anasir MI; Azizan MA; Kamel K; Mohd Zawawi Z; Ramly N; Robert F; Suppiah J; Thayan R
EPI_ISL_3120237	Sungai Buloh Hospital	Institute for Medical Research, Infectious Disease Research Centre, National Institutes of Health, Ministry of Health Malaysia	Anasir MI; Azizan MA; Kamel K; Mohd Zawawi Z; Ramly N; Robert F; Suppiah J; Thayan R
EPI_ISL_8147382, EPI_ISL_8170431	Svasthom Cosmos Htech Sdn Bhd	Tropical Infectious Diseases Research & Education Centre (TIDREC), Universiti Malaya	AsmaAnati CheMatSeri; Che-Norainon Yaacob; Jia-Yi Tan; Jo-Ern Wong; Kim-Kee Tan; Mulya-Mustika-Sari Zulkifli; Noor Syahida Azizan; Nur-Hidayana Mahfodz; Sazaly AbuBakar; Siti-Sarah Nor'e
EPI_ISL_2854109	Tawau Hospital	Institute for Medical Research, Infectious Disease Research Centre, National Institutes of Health, Ministry of Health Malaysia	Anasir MI; Azizan MA; Kamel K; Mohd Zawawi Z; Ramly N; Robert F; Suppiah J; Thayan R
EPI_ISL_3100126, EPI_ISL_3100172, EPI_ISL_3100173, EPI_ISL_3100174	Tengku Ampuan Afzan Hospital	Institute for Medical Research, Infectious Disease Research Centre, National Institutes of Health, Ministry of Health Malaysia	Anasir MI; Azizan MA; Kamel K; Mohd Zawawi Z; Ramly N; Robert F; Suppiah J; Thayan R
EPI_ISL_2921997, EPI_ISL_3071857, EPI_ISL_3071858, EPI_ISL_3120114, EPI_ISL_3120115, EPI_ISL_3120138, EPI_ISL_3355519			
see above	Tengku Ampuan Rahimah Hospital	Institute for Medical Research, Infectious Disease Research Centre, National Institutes of Health, Ministry of Health Malaysia	Anasir MI; Azizan MA; Kamel K; Mohd Zawawi Z; Ramly N; Robert F; Suppiah J; Thayan R
EPI_ISL_2921260, EPI_ISL_2921261	Terengganu State Health Department	Institute for Medical Research, Infectious Disease Research Centre, National Institutes of Health, Ministry of Health Malaysia	Anasir MI; Azizan MA; Kamel K; Mohd Zawawi Z; Ramly N; Robert F; Suppiah J; Thayan R
EPI_ISL_12300603, EPI_ISL_12300604, EPI_ISL_12300605, EPI_ISL_12300606	Tropical Infectious Diseases Research & Education Centre (TIDREC), Universiti Malaya	Tropical Infectious Diseases Research & Education Centre (TIDREC), Universiti Malaya	AsmaAnati CheMatSeri; Che-Norainon Yaacob; Jia-Yi Tan; Jo-Ern Wong; Kim-Kee Tan; Mulya-Mustika-Sari Zulkifli; Noor Syahida Azizan; Nur-Hidayana Mahfodz; Sazaly AbuBakar; Siti-Sarah Nor'e
EPI_ISL_2931922, EPI_ISL_3333562	Tuanku Fauziah Hospital	Institute for Medical Research, Infectious Disease Research Centre, National Institutes of Health, Ministry of Health Malaysia	Anasir MI; Azizan MA; Kamel K; Mohd Zawawi Z; Ramly N; Robert F; Suppiah J; Thayan R
EPI_ISL_6131996, EPI_ISL_6131997, EPI_ISL_6131998, EPI_ISL_6132001, EPI_ISL_6132002, EPI_ISL_6132029, EPI_ISL_6132032, EPI_ISL_6132048, EPI_ISL_6132053, EPI_ISL_6132056, EPI_ISL_6132059			
see above	UMMC	Department of Medical Microbiology, Faculty of Medicine, University of Malaya; University of Malaya Medical Centre	I-Ching SAM; Jolene Yin Ling FU; Omar Khalilur Rahman; Yoke Fun Chan

We gratefully acknowledge the following Authors from the Originating laboratories responsible for obtaining the specimens, as well as the Submitting laboratories where the genome data were generated and shared via GISAID, on which this research is based.

All Submitters of data may be contacted directly via www.gisaid.org

Authors are sorted alphabetically.

Accession ID	Originating Laboratory	Submitting Laboratory	Authors
EPI_ISL_3425884, EPI_ISL_5113899, EPI_ISL_7380529, EPI_ISL_7380533	Ampang Hospital	Institute for Medical Research, Infectious Disease Research Centre, National Institutes of Health, Ministry of Health Malaysia	Ahmad FA; Ahmad Fazilah NA; Anasir MI; Azizan MA; Kamel K; Mohamad Sukri MZ; Mohd Zawawi Z; Norhisham SN; Ramly N; Robert F; Rosli NR; Suppiah J; Thayan R
EPI_ISL_5400450	Banting Hospital	Institute for Medical Research, Infectious Disease Research Centre, National Institutes of Health, Ministry of Health Malaysia	Ahmad FA; Ahmad Fazilah NA; Anasir MI; Azizan MA; Kamel K; Mohd Zawawi Z; Norhisham SN; Ramly N; Robert F; Suppiah J; Thayan R
EPI_ISL_3446665, EPI_ISL_3446666, EPI_ISL_3446668	Batu Health Clinic	Institute for Medical Research, Infectious Disease Research Centre, National Institutes of Health, Ministry of Health Malaysia	Anasir MI; Azizan MA; Kamel K; Mohd Zawawi Z; Ramly N; Robert F; Suppiah J; Thayan R
EPI_ISL_4101546	Beaufort District Health Office	Institute for Medical Research, Infectious Disease Research Centre, National Institutes of Health, Ministry of Health Malaysia	Ahmad FA; Ahmad Fazilah NA; Anasir MI; Azizan MA; Kamel K; Mohd Zawawi Z; Norhisham SN; Ramly N; Robert F; Suppiah J; Thayan R
EPI_ISL_4051645, EPI_ISL_4051674, EPI_ISL_4051697	Borneo Medical Centre	Institute of Health and Community Medicine	Chan Chia Jui; Chua Hock Hin; David Perera; Ooi Mong How; Tonnii Sia Loong Loong; Wong Jyn Shan
EPI_ISL_3446671, EPI_ISL_3446673	Bukit Changgang Health Clinic	Institute for Medical Research, Infectious Disease Research Centre, National Institutes of Health, Ministry of Health Malaysia	Anasir MI; Azizan MA; Kamel K; Mohd Zawawi Z; Ramly N; Robert F; Suppiah J; Thayan R
EPI_ISL_3446676	Bukit Kuda Health Clinic	Institute for Medical Research, Infectious Disease Research Centre, National Institutes of Health, Ministry of Health Malaysia	Anasir MI; Azizan MA; Kamel K; Mohd Zawawi Z; Ramly N; Robert F; Suppiah J; Thayan R
EPI_ISL_3446674	Bukit Kuda Heath Clinic	Institute for Medical Research, Infectious Disease Research Centre, National Institutes of Health, Ministry of Health Malaysia	Anasir MI; Azizan MA; Kamel K; Mohd Zawawi Z; Ramly N; Robert F; Suppiah J; Thayan R
EPI_ISL_4565023	CPRC HOSPITAL SG BULOH	Tropical Infectious Diseases Research & Education Centre (TIDREC), Universiti Malaya	AsmaAnati CheMatSeri; Che-Norainon Yaacob; Jia-Yi Tan; Jo-Ern Wong; Kim-Kee Tan; Mulya-Mustika-Sari Zulkifli; Noor Syahida Azizan; Nur-Hidayana Mahfodz; Sazaly AbuBakar; Siti-Sarah Nor’e
EPI_ISL_4565033	CPRC IPJKNT	Tropical Infectious Diseases Research & Education Centre (TIDREC), Universiti Malaya	AsmaAnati CheMatSeri; Che-Norainon Yaacob; Jia-Yi Tan; Jo-Ern Wong; Kim-Kee Tan; Mulya-Mustika-Sari Zulkifli; Noor Syahida Azizan; Nur-Hidayana Mahfodz; Sazaly AbuBakar; Siti-Sarah Nor’e
EPI_ISL_3980725, EPI_ISL_4565032, EPI_ISL_4565034, EPI_ISL_4565035, EPI_ISL_4565036, EPI_ISL_4565037, EPI_ISL_4565038, EPI_ISL_4565039, EPI_ISL_4565041, EPI_ISL_4565042			
see above	CPRC JKNT	Tropical Infectious Diseases Research & Education Centre (TIDREC), Universiti Malaya	AsmaAnati CheMatSeri; Che-Norainon Yaacob; Jia-Yi Tan; Jo-Ern Wong; Kim-Kee Tan; Mulya-Mustika-Sari Zulkifli; Noor Syahida Azizan; Nur-Hidayana Mahfodz; Sazaly AbuBakar; Siti-Sarah Nor’e
EPI_ISL_4051646, EPI_ISL_4051647, EPI_ISL_4051648, EPI_ISL_4051649, EPI_ISL_4051650, EPI_ISL_4051651, EPI_ISL_4051652, EPI_ISL_4051664, EPI_ISL_4051665, EPI_ISL_4051666, EPI_ISL_4051676, EPI_ISL_4051703, EPI_ISL_4051704, EPI_ISL_4051772, EPI_ISL_4051773, EPI_ISL_4051774, EPI_ISL_4051775, EPI_ISL_4051776, EPI_ISL_4051777, EPI_ISL_4051778, EPI_ISL_4051833, EPI_ISL_4051834, EPI_ISL_4051835, EPI_ISL_4051836			
see above	CRC, Sibu Hospital	Institute of Health and Community Medicine	Chan Chia Jui; Chua Hock Hin; David Perera; Ooi Mong How; Tonnii Sia Loong Loong; Wong Jyn Shan; Wong Kiing Aik
EPI_ISL_3374709	Cheras District Health Office	Institute for Medical Research, Infectious Disease Research Centre, National Institutes of Health, Ministry of Health Malaysia	Anasir MI; Azizan MA; Kamel K; Mohd Zawawi Z; Ramly N; Robert F; Suppiah J; Thayan R
EPI_ISL_3446678	Dengkil Health Clinic	Institute for Medical Research, Infectious Disease Research Centre, National Institutes of Health, Ministry of Health Malaysia	Anasir MI; Azizan MA; Kamel K; Mohd Zawawi Z; Ramly N; Robert F; Suppiah J; Thayan R
EPI_ISL_5778211	Department of Medical Microbiology, Hospital Pengajar Universiti Putra Malaysia	Department of Medical Microbiology, Hospital Pengajar Universiti Putra Malaysia	Afiqah Adzmi; Amiza Azmi; Azmiza Syawani Jasni; Chee Hui Yee; Leslie Than Thian Lung; Muadz Mohtar; Muhammad Mohd Isa; Narcisse MS Joseph; Niazlin Mohd Taib; Noor Hazirah Noor Azhari; Norlaila; Nur Raihana Ithnin; Nurul Huda Mohamed Rashidi; Nurul Nadiah Ismail; Rosni Ibrahim; Sallehhudin; Siti Norbaya Masri; Siti Zulaikha Zakariah; Suppiah J; Syafinaz Amin Nordin; Tengku Zetty Maztura Tengku Jamaluddin; Thayan R; Zamberi Sekawi
EPI_ISL_4056087, EPI_ISL_4056088, EPI_ISL_4056089, EPI_ISL_4056090, EPI_ISL_4056108, EPI_ISL_4056109, EPI_ISL_4056110, EPI_ISL_4056111, EPI_ISL_4056113, EPI_ISL_4056114, EPI_ISL_4056116, EPI_ISL_4056117, EPI_ISL_4056119, EPI_ISL_4056120, EPI_ISL_4056121, EPI_ISL_4056124, EPI_ISL_4056125, EPI_ISL_4056127, EPI_ISL_4056129, EPI_ISL_4056130, EPI_ISL_4056132, EPI_ISL_4056136, EPI_ISL_4056137, EPI_ISL_4056138, EPI_ISL_4056139, EPI_ISL_4056141, EPI_ISL_4056142, EPI_ISL_4056147, EPI_ISL_4056149, EPI_ISL_4056150, EPI_ISL_4056151, EPI_ISL_4056154, EPI_ISL_4056159, EPI_ISL_4056160, EPI_ISL_4056168, EPI_ISL_4056171, EPI_ISL_4056172			
see above	Department of Medical Microbiology, University Malaya Medical Centre	Department of Medical Microbiology, Faculty of Medicine, University of Malaya	I-Ching SAM; Izzati Kausar; Jolene Yin Ling FU; Yoke Fun Chan; Yoong Min CHONG
EPI_ISL_5417159	Department of Medical Microbiology, University Malaya Medical Centre	Department of Medical Microbiology, University Malaya Medical Centre	I-Ching SAM; Jolene Yin Ling FU; Omar Khalilur Rahman; Yoke Fun Chan
EPI_ISL_3767019	Gribbles Pathology (M) Sdn Bhd	Institute for Medical Research, Infectious Disease Research Centre, National Institutes of Health, Ministry of Health Malaysia	Anasir MI; Azizan MA; Kamel K; Mohd Zawawi Z; Ramly N; Robert F; Suppiah J; Thayan R
EPI_ISL_4565025, EPI_ISL_4565027	H.Banting	Tropical Infectious Diseases Research & Education Centre (TIDREC), Universiti Malaya	AsmaAnati CheMatSeri; Che-Norainon Yaacob; Jia-Yi Tan; Jo-Ern Wong; Kim-Kee Tan; Mulya-Mustika-Sari Zulkifli; Noor Syahida Azizan; Nur-Hidayana Mahfodz; Sazaly AbuBakar; Siti-Sarah Nor’e
EPI_ISL_4565043	HOSPITAL BAHAGIA ULU KINTA	Tropical Infectious Diseases Research & Education Centre (TIDREC), Universiti Malaya	AsmaAnati CheMatSeri; Che-Norainon Yaacob; Jia-Yi Tan; Jo-Ern Wong; Kim-Kee Tan; Mulya-Mustika-Sari Zulkifli; Noor Syahida Azizan; Nur-Hidayana Mahfodz; Sazaly AbuBakar; Siti-Sarah Nor’e
EPI_ISL_7087398	HOSPITAL TUANKU FAUZIAH	Department of Medical Microbiology, Faculty of Medicine, University of Malaya; University of Malaya Medical Centre	I-Ching SAM; Jolene Yin Ling FU; Omar Khalilur Rahman; Yoke Fun Chan
EPI_ISL_3980748, EPI_ISL_3980751	HRPB, Ipoh	Tropical Infectious Diseases Research & Education Centre (TIDREC), Universiti Malaya	AsmaAnati CheMatSeri; Che-Norainon Yaacob; Jia-Yi Tan; Jo-Ern Wong; Kim-Kee Tan; Mulya-Mustika-Sari Zulkifli; Noor Syahida Azizan; Nur-Hidayana Mahfodz; Sazaly AbuBakar; Siti-Sarah Nor’e
EPI_ISL_6825318	Hospital Canselor Tuanku Muhriz UKM	Malaysia Genome Institute	Abdul Hamydy A Hamid; Ainihayati Noordin; Anita Sulong; Azrin Ahmad; Enizza Kasim; Haslina Mahbob; Irni Suhayu Sapian; Kon Ken Wong; Marjmin Osman; Mohd Faizal Abu Bakar; Mohd Noor Mat Isa; Muttaqillah Najihan A Samat; Najma Kori; Noor Hamiza Ghulam; Nor Maslini Ismail; Nordiana Ismail; Nur Sahairah Awang; Nurhezreen Md Iqbal; Petrick Periasamy; Shamsidar Sopie; Siti Noraini Othman; Siti Norlia Othman; Umi Kalsom Ali; Wan Husna M Jamaludin; Yusuf Muhammad Noor; Zetti Zainol Rashid
EPI_ISL_8745619, EPI_ISL_8745620	Hospital Canselor Tuanku Muhriz UKM	Malaysia Genome and Vaccine Institute	Apical Scientific Sdn Bhd's Team; Azrin Ahmad; Carey Wee Kai Li; Enizza Kasim; Irni Suhayu Sapian; Jilian Michelle Wong Tzeling; Leong Wai Mun; Mohd Faizal Abu Bakar; Mohd Ghows Mohd Azzam.; Mohd Noor Mat Isa; Nor Azfa Johari; Nurhezreen Md Iqbal; Rose Iszati Ismet Nayan; Shamsidar Sopie; Siti Noraini Othman; Yusuf Muhammad Noor
EPI_ISL_5428534, EPI_ISL_5428536, EPI_ISL_5428537, EPI_ISL_5428539, EPI_ISL_5428552, EPI_ISL_5428555	Hospital Kajang	Malaysia Genome Institute	Azrin Ahmad; Enizza Kasim; Irni Suhayu Sapian; Mohd Faizal Abu Bakar; Mohd Ghows Mohd Azzam.; Mohd Noor Mat Isa; Nor Azfa Johari; Nurhezreen Md Iqbal; Shamsidar Sopie; Siti Noraini Othman; Yusuf Muhammad Noor
EPI_ISL_3762499	Hulu Perak District Health Office	Institute for Medical Research, Infectious Disease Research Centre, National Institutes of Health, Ministry of Health Malaysia	Anasir MI; Azizan MA; Kamel K; Mohd Zawawi Z; Ramly N; Robert F; Suppiah J; Thayan R
EPI_ISL_5742682, EPI_ISL_5881287	INSTITUT BIOLOGI MOLEKUL PERUBATAN UKM (UMBI)	UKM Medical Molecular Biology Institute (UMBI)	Mira Farzana binti Mohamad Mokhtar
EPI_ISL_4071978, EPI_ISL_4071979, EPI_ISL_4071981, EPI_ISL_4071983	Institut Biologi Molekul Perubatan UKM	UKM Medical Molecular Biology Institute (UMBI)	Mira Farzana binti Mohamad Mokhtar
EPI_ISL_5396597, EPI_ISL_5397601	Ipoh Public Health Laboratory (MKAI), Ministry of Health Malaysia	Institute for Medical Research, Infectious Disease Research Centre, National Institutes of Health, Ministry of Health Malaysia	Ahmad FA; Ahmad Fazilah NA; Anasir MI; Azizan MA; Kamel K; Mohd Zawawi Z; Norhisham SN; Ramly N; Robert F; Suppiah J; Thayan R
EPI_ISL_3769359, EPI_ISL_3769361, EPI_ISL_3769365, EPI_ISL_4122295, EPI_ISL_4122330, EPI_ISL_4435542	JKN PAHANG	iPROMISE, UiTM	Ariza Adnan; Fadzilah Mohd Nor; Lim Wai Feng; Mohd Asif Mohd Sukri; Mohd Nur Fakhruzzaman Noorizhab; Mohd Zaki Salleh; Sazzli Shahlan Kassim; Siti Farah Alwani Mohd Nawi; Siti Hamimah Sheikh Abdul Kadir; Teh Lay Kek; Wang Seok Mui
EPI_ISL_4122527, EPI_ISL_4122530	JKN Pahang	iPROMISE, UiTM	Ariza Adnan; Fadzilah Mohd Nor; Lim Wai Feng; Mohd Asif Mohd Sukri; Mohd Nur Fakhruzzaman Noorizhab; Mohd Zaki Salleh; Sazzli Shahlan Kassim; Siti Farah Alwani Mohd Nawi; Siti Hamimah Sheikh Abdul Kadir; Teh Lay Kek; Wang Seok Mui
EPI_ISL_3281367	JSS Medical Lab SDN BHD	Institute for Medical Research, Infectious Disease Research Centre, National Institutes of Health, Ministry of Health Malaysia	Anasir MI; Azizan MA; Kamel K; Mohd Zawawi Z; Ramly N; Robert F; Suppiah J; Thayan R

EPI_ISL_3980713, EPI_ISL_3980715	Jabatan Forensik Hospital Sultanah Bahiyah	Tropical Infectious Diseases Research & Education Centre (TIDREC), Universiti Malaya	AsmaAnati CheMatSeri; Che-Norainon Yaacob; Jia-Yi Tan; Jo-Ern Wong; Kim-Kee Tan; Mulya-Mustika-Sari Zulkifli; Noor Syahida Azizan; Nur-Hidayana Mahfodz; Sazaly AbuBakar; Siti-Sarah Nor'e
EPI_ISL_3446679	Jenjarom Health Clinic	Institute for Medical Research, Infectious Disease Research Centre, National Institutes of Health, Ministry of Health Malaysia	Anasir MI; Azizan MA; Kamel K; Mohd Zawawi Z; Ramly N; Robert F; Suppiah J; Thayan R
EPI_ISL_3446681, EPI_ISL_3446683	Jeram Health Clinic	Institute for Medical Research, Infectious Disease Research Centre, National Institutes of Health, Ministry of Health Malaysia	Anasir MI; Azizan MA; Kamel K; Mohd Zawawi Z; Ramly N; Robert F; Suppiah J; Thayan R
EPI_ISL_5114684, EPI_ISL_5114688, EPI_ISL_5114742, EPI_ISL_5114746, EPI_ISL_5114747, EPI_ISL_5418034, EPI_ISL_5418038, EPI_ISL_5418040, EPI_ISL_5418042, EPI_ISL_5418047, EPI_ISL_5418055, EPI_ISL_5418058, EPI_ISL_5418063, EPI_ISL_7380254, EPI_ISL_7380466			
see above	Johor Bahru Public Health Laboratory	Institute for Medical Research, Infectious Disease Research Centre, National Institutes of Health, Ministry of Health Malaysia	Ahmad FA; Ahmad Fazilah NA; Anasir MI; Azizan MA; Kamel K; Mohamad Sukri MZ; Mohd Zawawi Z; Norhisham SN; Ramly N; Robert F; Rosli NR; Suppiah J; Thayan R
EPI_ISL_7086638, EPI_ISL_7086694, EPI_ISL_7086720, EPI_ISL_7086734, EPI_ISL_7086744, EPI_ISL_7086779, EPI_ISL_7086792, EPI_ISL_7086826			
see above	KK KUAH	Department of Medical Microbiology, Faculty of Medicine, University of Malaya; University of Malaya Medical Centre	I-Ching SAM; Jolene Yin Ling FU; Omar Khalilur Rahman; Yoke Fun Chan
EPI_ISL_4565050	KK PEROL	Tropical Infectious Diseases Research & Education Centre (TIDREC), Universiti Malaya	AsmaAnati CheMatSeri; Che-Norainon Yaacob; Jia-Yi Tan; Jo-Ern Wong; Kim-Kee Tan; Mulya-Mustika-Sari Zulkifli; Noor Syahida Azizan; Nur-Hidayana Mahfodz; Sazaly AbuBakar; Siti-Sarah Nor'e
EPI_ISL_4565049, EPI_ISL_8090595	KK Pos Brooke	Tropical Infectious Diseases Research & Education Centre (TIDREC), Universiti Malaya	AsmaAnati CheMatSeri; Che-Norainon Yaacob; Jia-Yi Tan; Jo-Ern Wong; Kim-Kee Tan; Mulya-Mustika-Sari Zulkifli; Noor Syahida Azizan; Nur-Hidayana Mahfodz; Sazaly AbuBakar; Siti-Sarah Nor'e
EPI_ISL_4565030	KLINIK KESIHATAN LOJING	Tropical Infectious Diseases Research & Education Centre (TIDREC), Universiti Malaya	AsmaAnati CheMatSeri; Che-Norainon Yaacob; Jia-Yi Tan; Jo-Ern Wong; Kim-Kee Tan; Mulya-Mustika-Sari Zulkifli; Noor Syahida Azizan; Nur-Hidayana Mahfodz; Sazaly AbuBakar; Siti-Sarah Nor'e
EPI_ISL_4101563, EPI_ISL_4101564, EPI_ISL_4101565, EPI_ISL_4101566	Kajang Hospital	Institute for Medical Research, Infectious Disease Research Centre, National Institutes of Health, Ministry of Health Malaysia	Ahmad FA; Ahmad Fazilah NA; Anasir MI; Azizan MA; Kamel K; Mohd Zawawi Z; Norhisham SN; Ramly N; Robert F; Suppiah J; Thayan R
EPI_ISL_6773981, EPI_ISL_6773982, EPI_ISL_6774034	Kampar District Health Office	Institute for Medical Research, Infectious Disease Research Centre, National Institutes of Health, Ministry of Health Malaysia	Ahmad FA; Ahmad Fazilah NA; Anasir MI; Azizan MA; Kamel K; Mohamad Sukri MZ; Mohd Zawawi Z; Norhisham SN; Ramly N; Robert F; Rosli NR; Suppiah J; Thayan R
EPI_ISL_3446663, EPI_ISL_3446664	Kampung Bandar Health Clinic	Institute for Medical Research, Infectious Disease Research Centre, National Institutes of Health, Ministry of Health Malaysia	Anasir MI; Azizan MA; Kamel K; Mohd Zawawi Z; Ramly N; Robert F; Suppiah J; Thayan R
EPI_ISL_3446686, EPI_ISL_3446688	Kebun Baru Health Clinic	Institute for Medical Research, Infectious Disease Research Centre, National Institutes of Health, Ministry of Health Malaysia	Anasir MI; Azizan MA; Kamel K; Mohd Zawawi Z; Ramly N; Robert F; Suppiah J; Thayan R
EPI_ISL_4101548	Kinabatangan Area Health Office	Institute for Medical Research, Infectious Disease Research Centre, National Institutes of Health, Ministry of Health Malaysia	Ahmad FA; Ahmad Fazilah NA; Anasir MI; Azizan MA; Kamel K; Mohd Zawawi Z; Norhisham SN; Ramly N; Robert F; Suppiah J; Thayan R
EPI_ISL_4101558	Kinabatangan Center For Disease Control	Institute for Medical Research, Infectious Disease Research Centre, National Institutes of Health, Ministry of Health Malaysia	Ahmad FA; Ahmad Fazilah NA; Anasir MI; Azizan MA; Kamel K; Mohd Zawawi Z; Norhisham SN; Ramly N; Robert F; Suppiah J; Thayan R
EPI_ISL_4565047	Kk badang	Tropical Infectious Diseases Research & Education Centre (TIDREC), Universiti Malaya	AsmaAnati CheMatSeri; Che-Norainon Yaacob; Jia-Yi Tan; Jo-Ern Wong; Kim-Kee Tan; Mulya-Mustika-Sari Zulkifli; Noor Syahida Azizan; Nur-Hidayana Mahfodz; Sazaly AbuBakar; Siti-Sarah Nor'e
EPI_ISL_3762822, EPI_ISL_4728822, EPI_ISL_7380252	Kluang District Health Office	Institute for Medical Research, Infectious Disease Research Centre, National Institutes of Health, Ministry of Health Malaysia	Ahmad FA; Ahmad Fazilah NA; Anasir MI; Azizan MA; Kamel K; Mohamad Sukri MZ; Mohd Zawawi Z; Norhisham SN; Norhisyam SN; Ramly N; Robert F; Rosli NR; Suppiah J; Thayan R
EPI_ISL_4730428	Kota Kinabalu Area Health Office	Institute for Medical Research, Infectious Disease Research Centre, National Institutes of Health, Ministry of Health Malaysia	Ahmad FA; Ahmad Fazilah NA; Anasir MI; Azizan MA; Kamel K; Mohd Zawawi Z; Norhisham SN; Ramly N; Robert F; Suppiah J; Thayan R
EPI_ISL_4101547	Kota Marudu District Health Office	Institute for Medical Research, Infectious Disease Research Centre, National Institutes of Health, Ministry of Health Malaysia	Ahmad FA; Ahmad Fazilah NA; Anasir MI; Azizan MA; Kamel K; Mohd Zawawi Z; Norhisham SN; Ramly N; Robert F; Suppiah J; Thayan R
EPI_ISL_3446657	Kuala Kubu Bharu Hospital	Institute for Medical Research, Infectious Disease Research Centre, National Institutes of Health, Ministry of Health Malaysia	Anasir MI; Azizan MA; Kamel K; Mohd Zawawi Z; Ramly N; Robert F; Suppiah J; Thayan R
EPI_ISL_3446717, EPI_ISL_3446719, EPI_ISL_3446726, EPI_ISL_3446728	Kuala Langat District Health Office	Institute for Medical Research, Infectious Disease Research Centre, National Institutes of Health, Ministry of Health Malaysia	Anasir MI; Azizan MA; Kamel K; Mohd Zawawi Z; Ramly N; Robert F; Suppiah J; Thayan R
EPI_ISL_5113907	Kuala Lumpur General Hospital	Institute for Medical Research, Infectious Disease Research Centre, National Institutes of Health, Ministry of Health Malaysia	Ahmad FA; Ahmad Fazilah NA; Anasir MI; Azizan MA; Kamel K; Mohd Zawawi Z; Norhisham SN; Ramly N; Robert F; Suppiah J; Thayan R
EPI_ISL_3425312, EPI_ISL_3425462	Kuala Lumpur International Airport (KLIA) Health Office	Institute for Medical Research, Infectious Disease Research Centre, National Institutes of Health, Ministry of Health Malaysia	Anasir MI; Azizan MA; Kamel K; Mohd Zawawi Z; Ramly N; Robert F; Suppiah J; Thayan R
EPI_ISL_3446692, EPI_ISL_3446695	Kuala Selangor Health Clinic	Institute for Medical Research, Infectious Disease Research Centre, National Institutes of Health, Ministry of Health Malaysia	Anasir MI; Azizan MA; Kamel K; Mohd Zawawi Z; Ramly N; Robert F; Suppiah J; Thayan R
EPI_ISL_3446658	Kuala kubu Bharu Hospital	Institute for Medical Research, Infectious Disease Research Centre, National Institutes of Health, Ministry of Health Malaysia	Anasir MI; Azizan MA; Kamel K; Mohd Zawawi Z; Ramly N; Robert F; Suppiah J; Thayan R
EPI_ISL_4096812	Kudat District Health Office	Institute for Medical Research, Infectious Disease Research Centre, National Institutes of Health, Ministry of Health Malaysia	Ahmad FA; Ahmad Fazilah NA; Anasir MI; Azizan MA; Kamel K; Mohd Zawawi Z; Norhisham SN; Ramly N; Robert F; Suppiah J; Thayan R
EPI_ISL_4756037, EPI_ISL_7381176	Kulai District Health Office	Institute for Medical Research, Infectious Disease Research Centre, National Institutes of Health, Ministry of Health Malaysia	Ahmad FA; Ahmad Fazilah NA; Anasir MI; Azizan MA; Kamel K; Mohamad Sukri MZ; Mohd Zawawi Z; Norhisham SN; Ramly N; Robert F; Rosli NR; Suppiah J; Thayan R
EPI_ISL_3374523, EPI_ISL_3374524	Lablink (M) Sdn. Bhd.	Institute for Medical Research, Infectious Disease Research Centre, National Institutes of Health, Ministry of Health Malaysia	Anasir MI; Azizan MA; Kamel K; Mohd Zawawi Z; Ramly N; Robert F; Suppiah J; Thayan R
EPI_ISL_3762823, EPI_ISL_3762824	Lahad Datu Area Health Office	Institute for Medical Research, Infectious Disease Research Centre, National Institutes of Health, Ministry of Health Malaysia	Anasir MI; Azizan MA; Kamel K; Mohd Zawawi Z; Ramly N; Robert F; Suppiah J; Thayan R
EPI_ISL_4096794, EPI_ISL_6773972	Lahad Datu Hospital	Institute for Medical Research, Infectious Disease Research Centre, National Institutes of Health, Ministry of Health Malaysia	Ahmad FA; Ahmad Fazilah NA; Anasir MI; Azizan MA; Kamel K; Mohamad Sukri MZ; Mohd Zawawi Z; Norhisham SN; Ramly N; Robert F; Rosli NR; Suppiah J; Thayan R
EPI_ISL_5114309	Lam Wah Ee Hospital	Institute for Medical Research, Infectious Disease Research Centre, National Institutes of Health, Ministry of Health Malaysia	Ahmad FA; Ahmad Fazilah NA; Anasir MI; Azizan MA; Kamel K; Mohd Zawawi Z; Norhisham SN; Ramly N; Robert F; Suppiah J; Thayan R
EPI_ISL_4101543	Marudu District Health Office	Institute for Medical Research, Infectious Disease Research Centre, National Institutes of Health, Ministry of Health Malaysia	Ahmad FA; Ahmad Fazilah NA; Anasir MI; Azizan MA; Kamel K; Mohd Zawawi Z; Norhisham SN; Ramly N; Robert F; Suppiah J; Thayan R
EPI_ISL_4101544	Marudu District Health Ofiice	Institute for Medical Research, Infectious Disease Research Centre, National Institutes of Health, Ministry of Health Malaysia	Ahmad FA; Ahmad Fazilah NA; Anasir MI; Azizan MA; Kamel K; Mohd Zawawi Z; Norhisham SN; Ramly N; Robert F; Suppiah J; Thayan R
EPI_ISL_3762497, EPI_ISL_3762498	Melaka Hospital	Institute for Medical Research, Infectious Disease Research Centre, National Institutes of Health, Ministry of Health Malaysia	Anasir MI; Azizan MA; Kamel K; Mohd Zawawi Z; Ramly N; Robert F; Suppiah J; Thayan R

EPI_ISL_4730424, EPI_ISL_5114274, EPI_ISL_5852720	Melaka Tengah District Health Office	Institute for Medical Research, Infectious Disease Research Centre, National Institutes of Health, Ministry of Health Malaysia	Ahmad FA; Ahmad Fazilah NA; Anasir MI; Azizan MA; Kamel K; Mohd Zawawi Z; Norhisham SN; Norhisyam SN; Ramly N; Robert F; Suppiah J; Thayan R
EPI_ISL_4051661, EPI_ISL_4051680, EPI_ISL_4051682, EPI_ISL_4051683, EPI_ISL_4051684, EPI_ISL_4051685, EPI_ISL_4051686, EPI_ISL_4051687, EPI_ISL_4051688, EPI_ISL_4051693, EPI_ISL_4051694, EPI_ISL_4051695, EPI_ISL_4051832, EPI_ISL_4051851, EPI_ISL_4051852, EPI_ISL_4051853, EPI_ISL_4051854, EPI_ISL_4051858			
see above	Ministry of Health Hospitals	Institute of Health and Community Medicine	Chan Chia Jui; Chua Hock Hin; David Perera; Ooi Mong How; Tonnii Sia Loong Loong; Wong Jyn Shan; Wong Kiing Aik
EPI_ISL_4101545	Nabawan District Health Ofiice	Institute for Medical Research, Infectious Disease Research Centre, National Institutes of Health, Ministry of Health Malaysia	Ahmad FA; Ahmad Fazilah NA; Anasir MI; Azizan MA; Kamel K; Mohd Zawawi Z; Norhisham SN; Ramly N; Robert F; Suppiah J; Thayan R
EPI_ISL_3281413	National Cancer Institute	Institute for Medical Research, Infectious Disease Research Centre, National Institutes of Health, Ministry of Health Malaysia	Anasir MI; Azizan MA; Kamel K; Mohd Zawawi Z; Ramly N; Robert F; Suppiah J; Thayan R
EPI_ISL_3281014, EPI_ISL_3281015, EPI_ISL_3281016	National Public Health Laboratory	Institute for Medical Research, Infectious Disease Research Centre, National Institutes of Health, Ministry of Health Malaysia	Anasir MI; Azizan MA; Kamel K; Mohd Zawawi Z; Ramly N; Robert F; Suppiah J; Thayan R
EPI_ISL_4565051, EPI_ISL_4565052	PEJABAT KESIHATAN DAERAH MACHANG	Tropical Infectious Diseases Research & Education Centre (TIDREC), Universiti Malaya	AsmaAnati CheMatSeri; Che-Norainon Yaacob; Jia-Yi Tan; Jo-Ern Wong; Kim-Kee Tan; Mulya-Mustika-Sari Zulkifli; Noor Syahida Azizan; Nur-Hidayana Mahfodz; Sazaly AbuBakar; Siti-Sarah Nor'e
EPI_ISL_4565031	PEJABAT KESIHATAN DAERAH TUMPAT	Tropical Infectious Diseases Research & Education Centre (TIDREC), Universiti Malaya	AsmaAnati CheMatSeri; Che-Norainon Yaacob; Jia-Yi Tan; Jo-Ern Wong; Kim-Kee Tan; Mulya-Mustika-Sari Zulkifli; Noor Syahida Azizan; Nur-Hidayana Mahfodz; Sazaly AbuBakar; Siti-Sarah Nor'e
EPI_ISL_8170509	PKD Manjung	Tropical Infectious Diseases Research & Education Centre (TIDREC), Universiti Malaya	AsmaAnati CheMatSeri; Che-Norainon Yaacob; Jia-Yi Tan; Jo-Ern Wong; Kim-Kee Tan; Mulya-Mustika-Sari Zulkifli; Noor Syahida Azizan; Nur-Hidayana Mahfodz; Sazaly AbuBakar; Siti-Sarah Nor'e
EPI_ISL_4730184, EPI_ISL_4730283, EPI_ISL_5114270, EPI_ISL_5852766, EPI_ISL_5852775, EPI_ISL_6773998, EPI_ISL_6774036, EPI_ISL_6774042			
see above	Pahang State Health Department	Institute for Medical Research, Infectious Disease Research Centre, National Institutes of Health, Ministry of Health Malaysia	Ahmad FA; Ahmad Fazilah NA; Anasir MI; Azizan MA; Kamel K; Mohamad Sukri MZ; Mohd Zawawi Z; Norhisham SN; Norhisyam SN; Ramly N; Robert F; Rosli NR; Suppiah J; Thayan R
EPI_ISL_3446696, EPI_ISL_3446697, EPI_ISL_3762835	Pandamaran Health Clinic	Institute for Medical Research, Infectious Disease Research Centre, National Institutes of Health, Ministry of Health Malaysia	Anasir MI; Azizan MA; Kamel K; Mohd Zawawi Z; Ramly N; Robert F; Suppiah J; Thayan R
EPI_ISL_4565048	Pejabat Kesihatan daerah Jeli	Tropical Infectious Diseases Research & Education Centre (TIDREC), Universiti Malaya	AsmaAnati CheMatSeri; Che-Norainon Yaacob; Jia-Yi Tan; Jo-Ern Wong; Kim-Kee Tan; Mulya-Mustika-Sari Zulkifli; Noor Syahida Azizan; Nur-Hidayana Mahfodz; Sazaly AbuBakar; Siti-Sarah Nor'e
EPI_ISL_3723545, EPI_ISL_3762825, EPI_ISL_3762827, EPI_ISL_4348327	Penang General Hospital	Institute for Medical Research, Infectious Disease Research Centre, National Institutes of Health, Ministry of Health Malaysia	Ahmad FA; Ahmad Fazilah NA; Anasir MI; Azizan MA; Kamel K; Mohd Zawawi Z; Norhisyam SN; Ramly N; Robert F; Suppiah J; Thayan R
EPI_ISL_3762818	Penang State Health Department	Institute for Medical Research, Infectious Disease Research Centre, National Institutes of Health, Ministry of Health Malaysia	Anasir MI; Azizan MA; Kamel K; Mohd Zawawi Z; Ramly N; Robert F; Suppiah J; Thayan R
EPI_ISL_5852601	Perlis State Health Department	Institute for Medical Research, Infectious Disease Research Centre, National Institutes of Health, Ministry of Health Malaysia	Ahmad FA; Ahmad Fazilah NA; Anasir MI; Azizan MA; Kamel K; Mohd Zawawi Z; Norhisham SN; Ramly N; Robert F; Suppiah J; Thayan R
EPI_ISL_3769364	Pkd pekan	iPROMISE, UiTM	Ariza Adnan; Fadzilah Mohd Nor; Lim Wai Feng; Mohd Asif Mohd Sukri; Mohd Nur Fakhruzzaman Noorizhab; Mohd Zaki Salleh; Sazzli Shahlan Kassim; Siti Farah Alwani Mohd Nawi; Siti Hamimah Sheikh Abdul Kadir; Teh Lay Kek; Wang Seok Mui
EPI_ISL_3762828	Port Dickson District Health Office	Institute for Medical Research, Infectious Disease Research Centre, National Institutes of Health, Ministry of Health Malaysia	Anasir MI; Azizan MA; Kamel K; Mohd Zawawi Z; Ramly N; Robert F; Suppiah J; Thayan R
EPI_ISL_3446656	Port Klang Health Clinic	Institute for Medical Research, Infectious Disease Research Centre, National Institutes of Health, Ministry of Health Malaysia	Anasir MI; Azizan MA; Kamel K; Mohd Zawawi Z; Ramly N; Robert F; Suppiah J; Thayan R
EPI_ISL_3446702	Pulau Ketam Health Clinic	Institute for Medical Research, Infectious Disease Research Centre, National Institutes of Health, Ministry of Health Malaysia	Anasir MI; Azizan MA; Kamel K; Mohd Zawawi Z; Ramly N; Robert F; Suppiah J; Thayan R
EPI_ISL_3374706, EPI_ISL_3374707	Putrajaya Health Office	Institute for Medical Research, Infectious Disease Research Centre, National Institutes of Health, Ministry of Health Malaysia	Anasir MI; Azizan MA; Kamel K; Mohd Zawawi Z; Ramly N; Robert F; Suppiah J; Thayan R
EPI_ISL_3762495	Queen Elizabeth Hospital	Institute for Medical Research, Infectious Disease Research Centre, National Institutes of Health, Ministry of Health Malaysia	Anasir MI; Azizan MA; Kamel K; Mohd Zawawi Z; Ramly N; Robert F; Suppiah J; Thayan R
EPI_ISL_3762830	Raja Permaisuri Bainun Hospital	Institute for Medical Research, Infectious Disease Research Centre, National Institutes of Health, Ministry of Health Malaysia	Anasir MI; Azizan MA; Kamel K; Mohd Zawawi Z; Ramly N; Robert F; Suppiah J; Thayan R
EPI_ISL_5850347	Ranau District Health Office	Institute for Medical Research, Infectious Disease Research Centre, National Institutes of Health, Ministry of Health Malaysia	Ahmad FA; Ahmad Fazilah NA; Anasir MI; Azizan MA; Kamel K; Mohd Zawawi Z; Norhisham SN; Ramly N; Robert F; Suppiah J; Thayan R
EPI_ISL_4730383	Sabah Women and Children Hospital	Institute for Medical Research, Infectious Disease Research Centre, National Institutes of Health, Ministry of Health Malaysia	Ahmad FA; Ahmad Fazilah NA; Anasir MI; Azizan MA; Kamel K; Mohd Zawawi Z; Norhisyam SN; Ramly N; Robert F; Suppiah J; Thayan R
EPI_ISL_6773979, EPI_ISL_6773980, EPI_ISL_6773995, EPI_ISL_6773996, EPI_ISL_6774021	Sabah Women and Children's Hospital	Institute for Medical Research, Infectious Disease Research Centre, National Institutes of Health, Ministry of Health Malaysia	Ahmad FA; Ahmad Fazilah NA; Anasir MI; Azizan MA; Kamel K; Mohamad Sukri MZ; Mohd Zawawi Z; Norhisham SN; Ramly N; Robert F; Rosli NR; Suppiah J; Thayan R
EPI_ISL_3763022	Salak Health Clinic	Institute for Medical Research, Infectious Disease Research Centre, National Institutes of Health, Ministry of Health Malaysia	Anasir MI; Azizan MA; Kamel K; Mohd Zawawi Z; Ramly N; Robert F; Suppiah J; Thayan R
EPI_ISL_3762831, EPI_ISL_3762832	Seberang Perai Tengah District Health Office	Institute for Medical Research, Infectious Disease Research Centre, National Institutes of Health, Ministry of Health Malaysia	Anasir MI; Azizan MA; Kamel K; Mohd Zawawi Z; Ramly N; Robert F; Suppiah J; Thayan R
EPI_ISL_5401039	Shah Alam Hospital	Institute for Medical Research, Infectious Disease Research Centre, National Institutes of Health, Ministry of Health Malaysia	Ahmad FA; Ahmad Fazilah NA; Anasir MI; Azizan MA; Kamel K; Mohd Zawawi Z; Norhisyam SN; Ramly N; Robert F; Suppiah J; Thayan R
EPI_ISL_3446703	Sijangkang Health Clinic	Institute for Medical Research, Infectious Disease Research Centre, National Institutes of Health, Ministry of Health Malaysia	Anasir MI; Azizan MA; Kamel K; Mohd Zawawi Z; Ramly N; Robert F; Suppiah J; Thayan R
EPI_ISL_4101551, EPI_ISL_4101554, EPI_ISL_4101556, EPI_ISL_4101557, EPI_ISL_4101559	Sipitang District Health Office	Institute for Medical Research, Infectious Disease Research Centre, National Institutes of Health, Ministry of Health Malaysia	Ahmad FA; Ahmad Fazilah NA; Anasir MI; Azizan MA; Kamel K; Mohd Zawawi Z; Norhisham SN; Ramly N; Robert F; Suppiah J; Thayan R
EPI_ISL_6773997	Sultan Abdul Halim Hospital	Institute for Medical Research, Infectious Disease Research Centre, National Institutes of Health, Ministry of Health Malaysia	Ahmad FA; Ahmad Fazilah NA; Anasir MI; Azizan MA; Kamel K; Mohamad Sukri MZ; Mohd Zawawi Z; Norhisham SN; Ramly N; Robert F; Rosli NR; Suppiah J; Thayan R
EPI_ISL_3762737	Sultan Ismail Hospital	Institute for Medical Research, Infectious Disease Research Centre, National Institutes of Health, Ministry of Health Malaysia	Anasir MI; Azizan MA; Kamel K; Mohd Zawawi Z; Ramly N; Robert F; Suppiah J; Thayan R
EPI_ISL_4348321, EPI_ISL_4348322, EPI_ISL_4348323, EPI_ISL_4348325	Sultanah Bahiyah Hospital	Institute for Medical Research, Infectious Disease Research Centre, National Institutes of Health, Ministry of Health Malaysia	Ahmad FA; Ahmad Fazilah NA; Anasir MI; Azizan MA; Kamel K; Mohd Zawawi Z; Norhisyam SN; Ramly N; Robert F; Suppiah J; Thayan R
EPI_ISL_3333656, EPI_ISL_3333930, EPI_ISL_3333931, EPI_ISL_5399439, EPI_ISL_5418064	Sultanah Bahiyah Hospital, Alor Setar	Institute for Medical Research, Infectious Disease Research Centre, National Institutes of Health, Ministry of Health Malaysia	Ahmad FA; Ahmad Fazilah NA; Anasir MI; Azizan MA; Kamel K; Mohd Zawawi Z; Norhisham SN; Ramly N; Robert F; Suppiah J; Thayan R
EPI_ISL_3425706, EPI_ISL_3425707, EPI_ISL_3425789, EPI_ISL_4730121	Sungai Buloh Hospital	Institute for Medical Research, Infectious Disease Research Centre, National Institutes of Health, Ministry of Health	Ahmad FA; Ahmad Fazilah NA; Anasir MI; Azizan MA; Kamel K; Mohd Zawawi Z; Norhisham SN; Ramly N; Robert F; Suppiah J; Thayan R

		Malaysia	
EPI_ISL_3762738, EPI_ISL_3762758, EPI_ISL_5114040, EPI_ISL_5114263, EPI_ISL_5114265, EPI_ISL_5114279	Tampin District Health Office	Institute for Medical Research, Infectious Disease Research Centre, National Institutes of Health, Ministry of Health Malaysia	Ahmad FA; Ahmad Fazilah NA; Anasir MI; Azizan MA; Kamel K; Mohd Zawawi Z; Norhisham SN; Ramly N; Robert F; Suppiah J; Thayan R
EPI_ISL_3446704, EPI_ISL_3446706	Tanjung Sepat Health Clinic	Institute for Medical Research, Infectious Disease Research Centre, National Institutes of Health, Ministry of Health Malaysia	Anasir MI; Azizan MA; Kamel K; Mohd Zawawi Z; Ramly N; Robert F; Suppiah J; Thayan R
EPI_ISL_5852782	Tawau Area Health Center	Institute for Medical Research, Infectious Disease Research Centre, National Institutes of Health, Ministry of Health Malaysia	Ahmad FA; Ahmad Fazilah NA; Anasir MI; Azizan MA; Kamel K; Mohd Zawawi Z; Norhisham SN; Ramly N; Robert F; Suppiah J; Thayan R
EPI_ISL_5852761	Tawau Area Health Centre	Institute for Medical Research, Infectious Disease Research Centre, National Institutes of Health, Ministry of Health Malaysia	Ahmad FA; Ahmad Fazilah NA; Anasir MI; Azizan MA; Kamel K; Mohd Zawawi Z; Norhisham SN; Ramly N; Robert F; Suppiah J; Thayan R
EPI_ISL_5114311, EPI_ISL_5114316, EPI_ISL_6774017	Tawau Health Clinic	Institute for Medical Research, Infectious Disease Research Centre, National Institutes of Health, Ministry of Health Malaysia	Ahmad FA; Ahmad Fazilah NA; Anasir MI; Azizan MA; Kamel K; Mohamad Sukri MZ; Mohd Zawawi Z; Norhisham SN; Ramly N; Robert F; Rosli NR; Suppiah J; Thayan R
EPI_ISL_4348273, EPI_ISL_4348274, EPI_ISL_7380249	Tawau Hospital	Institute for Medical Research, Infectious Disease Research Centre, National Institutes of Health, Ministry of Health Malaysia	Ahmad FA; Ahmad Fazilah NA; Anasir MI; Azizan MA; Kamel K; Mohamad Sukri MZ; Mohd Zawawi Z; Norhisham SN; Norhisyam SN; Ramly N; Robert F; Rosli NR; Suppiah J; Thayan R
EPI_ISL_3446716	Telok Panglima Garang Health Clinic	Institute for Medical Research, Infectious Disease Research Centre, National Institutes of Health, Ministry of Health Malaysia	Anasir MI; Azizan MA; Kamel K; Mohd Zawawi Z; Ramly N; Robert F; Suppiah J; Thayan R
EPI_ISL_3446709, EPI_ISL_3446712	Teluk Datok Health Clinic	Institute for Medical Research, Infectious Disease Research Centre, National Institutes of Health, Ministry of Health Malaysia	Anasir MI; Azizan MA; Kamel K; Mohd Zawawi Z; Ramly N; Robert F; Suppiah J; Thayan R
EPI_ISL_3446714	Teluk Panglima Garang Health Clinic	Institute for Medical Research, Infectious Disease Research Centre, National Institutes of Health, Ministry of Health Malaysia	Anasir MI; Azizan MA; Kamel K; Mohd Zawawi Z; Ramly N; Robert F; Suppiah J; Thayan R
EPI_ISL_5114317, EPI_ISL_5114619	Tengku Ampuan Afzan Hospital	Institute for Medical Research, Infectious Disease Research Centre, National Institutes of Health, Ministry of Health Malaysia	Ahmad FA; Ahmad Fazilah NA; Anasir MI; Azizan MA; Kamel K; Mohd Zawawi Z; Norhisham SN; Ramly N; Robert F; Suppiah J; Thayan R
EPI_ISL_3446659, EPI_ISL_3446660, EPI_ISL_3446661, EPI_ISL_5401551, EPI_ISL_5401783, EPI_ISL_5402232	Tengku Ampuan Rahimah Hospital	Institute for Medical Research, Infectious Disease Research Centre, National Institutes of Health, Ministry of Health Malaysia	Ahmad FA; Ahmad Fazilah NA; Anasir MI; Azizan MA; Kamel K; Mohd Zawawi Z; Norhisham SN; Ramly N; Robert F; Suppiah J; Thayan R
EPI_ISL_4096811, EPI_ISL_4101549, EPI_ISL_4101550	Tenom District Health Office	Institute for Medical Research, Infectious Disease Research Centre, National Institutes of Health, Ministry of Health Malaysia	Ahmad FA; Ahmad Fazilah NA; Anasir MI; Azizan MA; Kamel K; Mohd Zawawi Z; Norhisham SN; Ramly N; Robert F; Suppiah J; Thayan R
EPI_ISL_4347590, EPI_ISL_4348365, EPI_ISL_6774038	Thomson Hospital Kota Damansara	Institute for Medical Research, Infectious Disease Research Centre, National Institutes of Health, Ministry of Health Malaysia	Ahmad FA; Ahmad Fazilah NA; Anasir MI; Azizan MA; Kamel K; Mohamad Sukri MZ; Mohd Zawawi Z; Norhisham SN; Norhisyam SN; Ramly N; Robert F; Rosli NR; Suppiah J; Thayan R
EPI_ISL_8295907	Tropical Infectious Diseases Research & Education Centre (TIDREC), Universiti Malaya	Tropical Infectious Diseases Research & Education Centre (TIDREC), Universiti Malaya	AsmaAnati CheMatSeri; Che-Norainon Yaacob; Jia-Yi Tan; Jo-Ern Wong; Kim-Kee Tan; Mulya-Mustika-Sari Zulkifli; Noor Syahida Azizan; Nur-Hidayana Mahfodz; Sazaly AbuBakar; Siti-Sarah Nor'e
EPI_ISL_6424636, EPI_ISL_6424641	Tuanku Fauziah Hospital	Institute for Medical Research, Infectious Disease Research Centre, National Institutes of Health, Ministry of Health Malaysia	Ahmad FA; Ahmad Fazilah NA; Anasir MI; Azizan MA; Kamel K; Mohd Zawawi Z; Norhisham SN; Ramly N; Robert F; Suppiah J; Thayan R
EPI_ISL_7380502	Tuanku Ja'afar Hospital	Institute for Medical Research, Infectious Disease Research Centre, National Institutes of Health, Ministry of Health Malaysia	Ahmad FA; Ahmad Fazilah NA; Anasir MI; Azizan MA; Kamel K; Mohamad Sukri MZ; Mohd Zawawi Z; Norhisham SN; Ramly N; Robert F; Rosli NR; Suppiah J; Thayan R
EPI_ISL_7189363	UMMC	Department of Medical Microbiology, Faculty of Medicine, University of Malaya; University of Malaya Medical Centre	I-Ching SAM; Jolene Yin Ling FU; Omar Khalilur Rahman; Yoke Fun Chan
EPI_ISL_3980730	UNIT KESIHATAN AWAM (UKA), HRPZ II	Tropical Infectious Diseases Research & Education Centre (TIDREC), Universiti Malaya	AsmaAnati CheMatSeri; Che-Norainon Yaacob; Jia-Yi Tan; Jo-Ern Wong; Kim-Kee Tan; Mulya-Mustika-Sari Zulkifli; Noor Syahida Azizan; Nur-Hidayana Mahfodz; Sazaly AbuBakar; Siti-Sarah Nor'e
EPI_ISL_3980732	UNIT KESIHATAN AWAM , HRPZII	Tropical Infectious Diseases Research & Education Centre (TIDREC), Universiti Malaya	AsmaAnati CheMatSeri; Che-Norainon Yaacob; Jia-Yi Tan; Jo-Ern Wong; Kim-Kee Tan; Mulya-Mustika-Sari Zulkifli; Noor Syahida Azizan; Nur-Hidayana Mahfodz; Sazaly AbuBakar; Siti-Sarah Nor'e
EPI_ISL_3980735	UNIT KESIHATAN AWAM HRPZII	Tropical Infectious Diseases Research & Education Centre (TIDREC), Universiti Malaya	AsmaAnati CheMatSeri; Che-Norainon Yaacob; Jia-Yi Tan; Jo-Ern Wong; Kim-Kee Tan; Mulya-Mustika-Sari Zulkifli; Noor Syahida Azizan; Nur-Hidayana Mahfodz; Sazaly AbuBakar; Siti-Sarah Nor'e
EPI_ISL_3980734	UNIT KESIHATAN AWAM(UKA) HRPZ	Tropical Infectious Diseases Research & Education Centre (TIDREC), Universiti Malaya	AsmaAnati CheMatSeri; Che-Norainon Yaacob; Jia-Yi Tan; Jo-Ern Wong; Kim-Kee Tan; Mulya-Mustika-Sari Zulkifli; Noor Syahida Azizan; Nur-Hidayana Mahfodz; Sazaly AbuBakar; Siti-Sarah Nor'e
EPI_ISL_4565018, EPI_ISL_4565019, EPI_ISL_4565020, EPI_ISL_4565021, EPI_ISL_4565022, EPI_ISL_4565024, EPI_ISL_8090643			
see above	UNIT OSH, HOSPITAL SUNGAI BULOH	Tropical Infectious Diseases Research & Education Centre (TIDREC), Universiti Malaya	AsmaAnati CheMatSeri; Che-Norainon Yaacob; Jia-Yi Tan; Jo-Ern Wong; Kim-Kee Tan; Mulya-Mustika-Sari Zulkifli; Noor Syahida Azizan; Nur-Hidayana Mahfodz; Sazaly AbuBakar; Siti-Sarah Nor'e
EPI_ISL_3374426	UiTM Sungai Buloh Hospital	Institute for Medical Research, Infectious Disease Research Centre, National Institutes of Health, Ministry of Health Malaysia	Anasir MI; Azizan MA; Kamel K; Mohd Zawawi Z; Ramly N; Robert F; Suppiah J; Thayan R
EPI_ISL_4891943	hsajb	UKM Medical Molecular Biology Institute (UMBI)	Mira Farzana binti Mohamad Mokhtar

We gratefully acknowledge the following Authors from the Originating laboratories responsible for obtaining the specimens, as well as the Submitting laboratories where the genome data were generated and shared via GISAID, on which this research is based.

All Submitters of data may be contacted directly via www.gisaid.org

Authors are sorted alphabetically.

Accession ID	Originating Laboratory	Submitting Laboratory	Authors
EPI_ISL_4730409, EPI_ISL_4730413, EPI_ISL_6774020, EPI_ISL_6774023, EPI_ISL_6774024, EPI_ISL_6774031	Ampang Hospital	Institute for Medical Research, Infectious Disease Research Centre, National Institutes of Health, Ministry of Health Malaysia	Ahmad FA; Ahmad Fazilah NA; Anasir MI; Azizan MA; Kamel K; Mohamad Sukri MZ; Mohd Zawawi Z; Norhisham SN; Norhisyam SN; Ramly N; Robert F; Rosli NR; Suppiah J; Thayan R
EPI_ISL_3769373	Bilik Gerakan Hospital Sungai Buloh	iPROMISE, UiTM	Ariza Adnan; Fadzilah Mohd Nor; Lim Wai Feng; Mohd Asif Mohd Sukri; Mohd Nur Fakhruzzaman Noorizhab; Mohd Zaki Salleh; Sazzli Shahlan Kassim; Siti Farah Alwani Mohd Nawi; Siti Hamimah Sheikh Abdul Kadir; Teh Lay Kek; Wang Seok Mui
EPI_ISL_4051657, EPI_ISL_4051658, EPI_ISL_4051659, EPI_ISL_4051660, EPI_ISL_4051726, EPI_ISL_4051727, EPI_ISL_4051728, EPI_ISL_4051729, EPI_ISL_4051819, EPI_ISL_4051820, EPI_ISL_4051821, EPI_ISL_4051822, EPI_ISL_4051823, EPI_ISL_4051824, EPI_ISL_4051825, EPI_ISL_4051826, EPI_ISL_4051846, EPI_ISL_4051848, EPI_ISL_4051860			
see above	Bintulu Hospital PCR Lab	Institute of Health and Community Medicine	Chan Chia Jui; Chua Hock Hin; David Perera; Ooi Mong How; Tan Lee See; Tonnii Sia Loong Loong; Wong Jyn Shan
EPI_ISL_4460067, EPI_ISL_4460068, EPI_ISL_4460069, EPI_ISL_4460070, EPI_ISL_4460071, EPI_ISL_4460072, EPI_ISL_4460073, EPI_ISL_4460074, EPI_ISL_4460075, EPI_ISL_4460076, EPI_ISL_4460077, EPI_ISL_4460078, EPI_ISL_4460079, EPI_ISL_4460080, EPI_ISL_4460081, EPI_ISL_4460082, EPI_ISL_4460083, EPI_ISL_4460084, EPI_ISL_4460085, EPI_ISL_4460086, EPI_ISL_4460087, EPI_ISL_4460088, EPI_ISL_4460089, EPI_ISL_4460091, EPI_ISL_4460092, EPI_ISL_4460093, EPI_ISL_4460094, EPI_ISL_4460170			
see above	Bintulu Hospital PCR Lab, Bintulu	Institute of Health and Community Medicine	Chan Chia Jui; Chua Hock Hin; David Perera; Ooi Mong How; Tan Lee See; Tonnii Sia Loong Loong; Wong Jyn Shan
EPI_ISL_4051675, EPI_ISL_4051696, EPI_ISL_4051698, EPI_ISL_4051699, EPI_ISL_4051700, EPI_ISL_4051701, EPI_ISL_4051702, EPI_ISL_4051827, EPI_ISL_4051855, EPI_ISL_4051856, EPI_ISL_4460102, EPI_ISL_4460103, EPI_ISL_4460109, EPI_ISL_4460110, EPI_ISL_4460111, EPI_ISL_4460112, EPI_ISL_4460231			
see above	Borneo Medical Centre	Institute of Health and Community Medicine	Chan Chia Jui; Chua Hock Hin; David Perera; Ooi Mong How; Tonnii Sia Loong Loong; Wong Jyn Shan
EPI_ISL_3980722, EPI_ISL_3980723, EPI_ISL_3980726	CPRC JKNT	Tropical Infectious Diseases Research & Education Centre (TIDREC), Universiti Malaya	AsmaAnati CheMatSeri; Che-Norainon Yaacob; Jia-Yi Tan; Jo-Ern Wong; Kim-Kee Tan; Mulya-Mustika-Sari Zulkifli; Noor Syahida Azizan; Nur-Hidayana Mahfodz; Sazaly AbuBakar; Siti-Sarah Nor’e
EPI_ISL_3980727	CPRC Jabatan kesihatan Negeri Terengganu	Tropical Infectious Diseases Research & Education Centre (TIDREC), Universiti Malaya	AsmaAnati CheMatSeri; Che-Norainon Yaacob; Jia-Yi Tan; Jo-Ern Wong; Kim-Kee Tan; Mulya-Mustika-Sari Zulkifli; Noor Syahida Azizan; Nur-Hidayana Mahfodz; Sazaly AbuBakar; Siti-Sarah Nor’e
EPI_ISL_4051667, EPI_ISL_4051668, EPI_ISL_4051669, EPI_ISL_4051670, EPI_ISL_4051671, EPI_ISL_4051672, EPI_ISL_4051673, EPI_ISL_4051678, EPI_ISL_4051679, EPI_ISL_4051779, EPI_ISL_4051780, EPI_ISL_4051781, EPI_ISL_4051782, EPI_ISL_4051783, EPI_ISL_4051784, EPI_ISL_4051785, EPI_ISL_4051786, EPI_ISL_4051787, EPI_ISL_4051788, EPI_ISL_4051818, EPI_ISL_4051839, EPI_ISL_4051847, EPI_ISL_4051859, EPI_ISL_4051863			
see above	CRC, Sibu Hospital	Institute of Health and Community Medicine	Chan Chia Jui; Chua Hock Hin; David Perera; Ooi Mong How; Tonnii Sia Loong Loong; Wong Jyn Shan; Wong Kiing Aik
EPI_ISL_4460217, EPI_ISL_5051777, EPI_ISL_5051840, EPI_ISL_5051841, EPI_ISL_5051842, EPI_ISL_5051843, EPI_ISL_5051847, EPI_ISL_5052157, EPI_ISL_5052158, EPI_ISL_5895046, EPI_ISL_5895056, EPI_ISL_5895065			
see above	Clinical Research Centre (CRC), Sibu Hospital, Sibu	Institute of Health and Community Medicine	Chan Chia Jui; Chua Hock Hin; David Perera; Ooi Mong How; Tonnii Sia Loong Loong; Wong Jyn Shan; Wong Kiing Aik
EPI_ISL_5782318, EPI_ISL_5782350, EPI_ISL_5782353	Department of Medical Microbiology, Hospital Pengajar Universiti Putra Malaysia	Department of Medical Microbiology, Hospital Pengajar Universiti Putra Malaysia	Afiqah Adzmi; Amiza Azmi; Azmiza Syawani Jasni; Chee Hui Yee; Leslie Than Thian Lung; Muadz Mohtar; Muhammad Mohd Isa; Narcisse MS Joseph; Niazlin Mohd Taib; Noor Hazirah Noor Azhari; Norlaila; Nur Raihana Ithnin; Nurul Huda Mohamed Rashidi; Nurul Nadiah Ismail; Rosni Ibrahim; Sallehhudin; Siti Norbaya Masri; Siti Zulaikha Zakariah; Suppiah J; Syafinaz Amin Nordin; Tengku Zetty Maztura Tengku Jamaluddin; Thayan R; Zamberi Sekawi
EPI_ISL_5417154	Department of Medical Microbiology, University Malaya Medical Centre	Department of Medical Microbiology, University Malaya Medical Centre	I-Ching SAM; Jolene Yin Ling FU; Omar Khalilur Rahman; Yoke Fun Chan
EPI_ISL_4096801	Duchess of Kent Hospital	Institute for Medical Research, Infectious Disease Research Centre, National Institutes of Health, Ministry of Health Malaysia	Ahmad FA; Ahmad Fazilah NA; Anasir MI; Azizan MA; Kamel K; Mohd Zawawi Z; Norhisham SN; Ramly N; Robert F; Suppiah J; Thayan R
EPI_ISL_6424391	Enche' Besar Hajjah Khalsom Hospital	Institute for Medical Research, Infectious Disease Research Centre, National Institutes of Health, Ministry of Health Malaysia	Ahmad FA; Ahmad Fazilah NA; Anasir MI; Azizan MA; Kamel K; Mohd Zawawi Z; Norhisham SN; Ramly N; Robert F; Suppiah J; Thayan R
EPI_ISL_5396587	Enche' Besar Hajjah Khalsom Hospital (HEBHK)	Institute for Medical Research, Infectious Disease Research Centre, National Institutes of Health, Ministry of Health Malaysia	Ahmad FA; Ahmad Fazilah NA; Anasir MI; Azizan MA; Kamel K; Mohd Zawawi Z; Norhisham SN; Ramly N; Robert F; Suppiah J; Thayan R
EPI_ISL_3858139	GLENEAGLES HOSPITAL MEDINI JOHOR	UKM Medical Molecular Biology Institute (UMBI)	Mira Farzana binti Mohamad Mokhtar
EPI_ISL_3769343	GLENEAGLES HOSPITAL MEDINI JOHOR	iPROMISE, UiTM	Ariza Adnan; Fadzilah Mohd Nor; Lim Wai Feng; Mohd Asif Mohd Sukri; Mohd Nur Fakhruzzaman Noorizhab; Mohd Zaki Salleh; Sazzli Shahlan Kassim; Siti Farah Alwani Mohd Nawi; Siti Hamimah Sheikh Abdul Kadir; Teh Lay Kek; Wang Seok Mui
EPI_ISL_4051705, EPI_ISL_4051770, EPI_ISL_4051771, EPI_ISL_4051796, EPI_ISL_4051840, EPI_ISL_4460141, EPI_ISL_4460142			
see above	Gribbles Pathology, Kuching	Institute of Health and Community Medicine	Chan Chia Jui; Chua Hock Hin; David Perera; Ooi Mong How; Reagan Entigu Linton; Tonnii Sia Loong Loong; Wong Jyn Shan
EPI_ISL_4565029	H.Banting	Tropical Infectious Diseases Research & Education Centre (TIDREC), Universiti Malaya	AsmaAnati CheMatSeri; Che-Norainon Yaacob; Jia-Yi Tan; Jo-Ern Wong; Kim-Kee Tan; Mulya-Mustika-Sari Zulkifli; Noor Syahida Azizan; Nur-Hidayana Mahfodz; Sazaly AbuBakar; Siti-Sarah Nor’e
EPI_ISL_12628154	HCTM	UKM Medical Molecular Biology Institute (UMBI)	Khairun Nur Abd Ghafar; Mira Farzana Mohamad Mokhtar; Muhiddin Ishak; Nor Azila Muhammad Azami; Nur Alyaa Afifah Md Shahri; Nurul Syakima Ab Mutalib; Rahman Jamal; Ryia Illani Mohd Yunos; Siti Nur Hasanah Mohd Yusuf; Zahirrah Begam Mohamed Rasheed
EPI_ISL_4565044, EPI_ISL_4565045	HOSPITAL AMPANG	Tropical Infectious Diseases Research & Education Centre (TIDREC), Universiti Malaya	AsmaAnati CheMatSeri; Che-Norainon Yaacob; Jia-Yi Tan; Jo-Ern Wong; Kim-Kee Tan; Mulya-Mustika-Sari Zulkifli; Noor Syahida Azizan; Nur-Hidayana Mahfodz; Sazaly AbuBakar; Siti-Sarah Nor’e
EPI_ISL_4565040	HOSPITAL ANGKATAN TENTERA TUANKU MIZAN	Tropical Infectious Diseases Research & Education Centre (TIDREC), Universiti Malaya	AsmaAnati CheMatSeri; Che-Norainon Yaacob; Jia-Yi Tan; Jo-Ern Wong; Kim-Kee Tan; Mulya-Mustika-Sari Zulkifli; Noor Syahida Azizan; Nur-Hidayana Mahfodz; Sazaly AbuBakar; Siti-Sarah Nor’e
EPI_ISL_4122282	HOSPITAL PULAU PINANG	iPROMISE, UiTM	Ariza Adnan; Fadzilah Mohd Nor; Lim Wai Feng; Mohd Asif Mohd Sukri; Mohd Nur Fakhruzzaman Noorizhab; Mohd Zaki Salleh; Sazzli Shahlan Kassim; Siti Farah Alwani Mohd Nawi; Siti Hamimah Sheikh Abdul Kadir; Teh Lay Kek; Wang Seok Mui
EPI_ISL_3858163, EPI_ISL_3858184, EPI_ISL_3858185, EPI_ISL_3858186, EPI_ISL_3945538, EPI_ISL_3945539, EPI_ISL_3945541, EPI_ISL_5417615, EPI_ISL_5417617, EPI_ISL_5417618			
see above	HOSPITAL SULTANAH NORA ISMAIL	UKM Medical Molecular Biology Institute (UMBI)	Mira Farzana binti Mohamad Mokhtar; Nur Alyaa Afifah Md Shahri
EPI_ISL_7087421, EPI_ISL_7087442, EPI_ISL_7087509, EPI_ISL_7087556, EPI_ISL_7087637, EPI_ISL_7087650	HRPB, Ipoh	Department of Medical Microbiology, Faculty of Medicine, University of Malaya; University of Malaya Medical Centre	I-Ching SAM; Jolene Yin Ling FU; Omar Khalilur Rahman; Yoke Fun Chan
EPI_ISL_3980747, EPI_ISL_3980752, EPI_ISL_3980754	HRPB, Ipoh	Tropical Infectious Diseases Research & Education Centre (TIDREC), Universiti Malaya	AsmaAnati CheMatSeri; Che-Norainon Yaacob; Jia-Yi Tan; Jo-Ern Wong; Kim-Kee Tan; Mulya-Mustika-Sari Zulkifli; Noor Syahida Azizan; Nur-Hidayana Mahfodz; Sazaly AbuBakar; Siti-Sarah Nor’e
EPI_ISL_5428525, EPI_ISL_5428526, EPI_ISL_5428527, EPI_ISL_5428528, EPI_ISL_5428529, EPI_ISL_5428530, EPI_ISL_5428531, EPI_ISL_5428532, EPI_ISL_5428533, EPI_ISL_5428538, EPI_ISL_5428540, EPI_ISL_5428541, EPI_ISL_5428542, EPI_ISL_5428549, EPI_ISL_5428550, EPI_ISL_5428553, EPI_ISL_5428554, EPI_ISL_5428556, EPI_ISL_5428557, EPI_ISL_5428558, EPI_ISL_5428559, EPI_ISL_5536412, EPI_ISL_5536413, EPI_ISL_5536414, EPI_ISL_5536415, EPI_ISL_5536416, EPI_ISL_5536417, EPI_ISL_5536418, EPI_ISL_5536419, EPI_ISL_5536420, EPI_ISL_5536421, EPI_ISL_5536451, EPI_ISL_5536452, EPI_ISL_5536457			
see above	Hospital Kajang	Malaysia Genome Institute	Azrin Ahmad; Enizza Kasim; Irni Suhayu Sapian; Mohd Faizal Abu Bakar; Mohd Ghows Mohd Azzam.; Mohd Noor Mat Isa; Nor Azfa Johari; Nurhezreen Md Iqbal; Shamsidar Sopie; Siti Noraini Othman; Yusuf Muhammad Noor
EPI_ISL_11900035	Hospital Kajang	Malaysia Genome and Vaccine Institute	Azrin Ahmad; Enizza Kasim; Irni Suhayu Sapian; Mohd Faizal Abu Bakar; Mohd Ghows Mohd Azzam.; Mohd Noor Mat Isa; Nor Azfa Johari; Nurhezreen Md Iqbal; Shamsidar Sopie; Siti Noraini Othman; Yusuf Muhammad Noor
EPI_ISL_4122275	Hospital P. Pinang	iPROMISE, UiTM	Ariza Adnan; Fadzilah Mohd Nor; Lim Wai Feng; Mohd Asif Mohd Sukri; Mohd Nur Fakhruzzaman Noorizhab; Mohd Zaki Salleh; Sazzli Shahlan Kassim; Siti Farah Alwani Mohd Nawi; Siti Hamimah Sheikh Abdul Kadir; Teh Lay Kek; Wang Seok Mui
EPI_ISL_3769378	Hospital Serdang	iPROMISE, UiTM	Ariza Adnan; Fadzilah Mohd Nor; Lim Wai Feng; Mohd Asif Mohd Sukri; Mohd Nur Fakhruzzaman Noorizhab; Mohd Zaki Salleh; Sazzli Shahlan Kassim; Siti Farah Alwani Mohd Nawi; Siti Hamimah Sheikh Abdul Kadir; Teh Lay Kek; Wang Seok Mui
EPI_ISL_4101581	Hospital Sultanah Nora Ismail	UKM Medical Molecular Biology Institute (UMBI)	Nur Alyaa Afifah Md Shahri
EPI_ISL_3945545, EPI_ISL_3945546, EPI_ISL_3945547, EPI_ISL_3945548, EPI_ISL_4071984, EPI_ISL_5417614	Institut Biologi Molekul Perubatan UKM	UKM Medical Molecular Biology Institute (UMBI)	Mira Farzana binti Mohamad Mokhtar; Nur Alyaa Afifah Md Shahri
EPI_ISL_4740399	JABATAN FORENSIK	iPROMISE, UiTM	Ariza Adnan; Fadzilah Mohd Nor; Lim Wai Feng; Mohd Asif Mohd Sukri; Mohd Nur Fakhruzzaman Noorizhab; Mohd Zaki Salleh; Sazzli Shahlan Kassim; Siti Farah Alwani Mohd Nawi; Siti Hamimah Sheikh Abdul Kadir; Teh Lay Kek; Wang Seok Mui
EPI_ISL_4122508	JABATAN KESIHATAN NEGERI PERLIS	iPROMISE, UiTM	Ariza Adnan; Fadzilah Mohd Nor; Lim Wai Feng; Mohd Asif Mohd Sukri; Mohd Nur Fakhruzzaman Noorizhab; Mohd Zaki Salleh; Sazzli Shahlan Kassim; Siti Farah Alwani Mohd Nawi; Siti Hamimah Sheikh Abdul Kadir; Teh Lay Kek; Wang Seok Mui
EPI_ISL_7087466	JABATAN PERUBATAN FORENSIK HOSPITAL SULTANAH BAHIYAH	Department of Medical Microbiology, Faculty of Medicine, University of Malaya; University of Malaya Medical Centre	I-Ching SAM; Jolene Yin Ling FU; Omar Khalilur Rahman; Yoke Fun Chan
EPI_ISL_3980708, EPI_ISL_3980710, EPI_ISL_3980717	JABATAN PERUBATAN FORENSIK HOSPITAL SULTANAH BAHIYAH	Tropical Infectious Diseases Research & Education Centre (TIDREC), Universiti Malaya	AsmaAnati CheMatSeri; Che-Norainon Yaacob; Jia-Yi Tan; Jo-Ern Wong; Kim-Kee Tan; Mulya-Mustika-Sari Zulkifli; Noor Syahida Azizan; Nur-Hidayana Mahfodz; Sazaly AbuBakar; Siti-Sarah Nor’e
EPI_ISL_3769355, EPI_ISL_3769356, EPI_ISL_3769358, EPI_ISL_3769360, EPI_ISL_3769362, EPI_ISL_3769366, EPI_ISL_3769367, EPI_ISL_3769368, EPI_ISL_3769369, EPI_ISL_3769370, EPI_ISL_3769371, EPI_ISL_3769372, EPI_ISL_4122319, EPI_ISL_4435511, EPI_ISL_4435513, EPI_ISL_4435535, EPI_ISL_4435543, EPI_ISL_4435544, EPI_ISL_4435549			
see above	JKN PAHANG	iPROMISE, UiTM	Ariza Adnan; Fadzilah Mohd Nor; Lim Wai Feng; Mohd Asif Mohd Sukri; Mohd Nur Fakhruzzaman Noorizhab; Mohd Zaki Salleh; Sazzli Shahlan Kassim; Siti Farah Alwani Mohd Nawi; Siti Hamimah Sheikh Abdul Kadir; Teh Lay Kek; Wang Seok Mui
EPI_ISL_3769354, EPI_ISL_4122308, EPI_ISL_4435550	JKN Pahang	iPROMISE, UiTM	Ariza Adnan; Fadzilah Mohd Nor; Lim Wai Feng; Mohd Asif Mohd Sukri; Mohd Nur Fakhruzzaman Noorizhab; Mohd Zaki Salleh; Sazzli Shahlan Kassim; Siti Farah Alwani Mohd Nawi; Siti Hamimah Sheikh Abdul Kadir; Teh Lay Kek; Wang Seok Mui
EPI_ISL_3980709, EPI_ISL_3980711, EPI_ISL_3980714,	Jabatan Forensik Hospital Sultanah	Tropical Infectious Diseases Research & Education Centre	AsmaAnati CheMatSeri; Che-Norainon Yaacob; Jia-Yi Tan; Jo-Ern Wong; Kim-Kee Tan; Mulya-Mustika-Sari Zulkifli; Noor Syahida Azizan; Nur-Hidayana Mahfodz; Sazaly AbuBakar; Siti-Sarah Nor’e

EPI_ISL_3980716, EPI_ISL_3980718, EPI_ISL_3980720	Bahiyah	(TIDREC), Universiti Malaya	
EPI_ISL_4565046	Jabatan Perubatan, Hospital Kuala Lumpur	Tropical Infectious Diseases Research & Education Centre (TIDREC), Universiti Malaya	AsmaAnati CheMatSeri; Che-Norainon Yaacob; Jia-Yi Tan; Jo-Ern Wong; Kim-Kee Tan; Mulya-Mustika-Sari Zulkifli; Noor Syahida Azizan; Nur-Hidayana Mahfodz; Sazaly AbuBakar; Siti-Sarah Nor'e
EPI_ISL_7457655	Johor Bahru Public Health Laboratory	Institute for Medical Research, Infectious Disease Research Centre, National Institutes of Health, Ministry of Health Malaysia	Ahmad FA; Ahmad Fazilah NA; Anasir MI; Azizan MA; Kamel K; Mohamad Sukri MZ; Mohd Zawawi Z; Norhisham SN; Ramly N; Robert F; Rosli NR; Suppiah J; Thayan R
EPI_ISL_4101560	Kajang Hospital	Institute for Medical Research, Infectious Disease Research Centre, National Institutes of Health, Ministry of Health Malaysia	Ahmad FA; Ahmad Fazilah NA; Anasir MI; Azizan MA; Kamel K; Mohd Zawawi Z; Norhisham SN; Ramly N; Robert F; Suppiah J; Thayan R
EPI_ISL_7086803	Klinik Kesihatan Kuah	Department of Medical Microbiology, Faculty of Medicine, University of Malaya; University of Malaya Medical Centre	I-Ching SAM; Jolene Yin Ling FU; Omar Khalilur Rahman; Yoke Fun Chan
EPI_ISL_4730455, EPI_ISL_4730459	Kuala Langat District Health Office	Institute for Medical Research, Infectious Disease Research Centre, National Institutes of Health, Ministry of Health Malaysia	Ahmad FA; Ahmad Fazilah NA; Anasir MI; Azizan MA; Kamel K; Mohd Zawawi Z; Norhisham SN; Ramly N; Robert F; Suppiah J; Thayan R
EPI_ISL_3764515, EPI_ISL_3765760, EPI_ISL_4730470	Kuala Lumpur International Airport (KLIA) Health Office	Institute for Medical Research, Infectious Disease Research Centre, National Institutes of Health, Ministry of Health Malaysia	Ahmad FA; Ahmad Fazilah NA; Anasir MI; Azizan MA; Kamel K; Mohd Zawawi Z; Norhisham SN; Ramly N; Robert F; Suppiah J; Thayan R
EPI_ISL_7380070	Labuan Hospital	Institute for Medical Research, Infectious Disease Research Centre, National Institutes of Health, Ministry of Health Malaysia	Ahmad FA; Ahmad Fazilah NA; Anasir MI; Azizan MA; Kamel K; Mohamad Sukri MZ; Mohd Zawawi Z; Norhisham SN; Ramly N; Robert F; Rosli NR; Suppiah J; Thayan R
EPI_ISL_4096793, EPI_ISL_4348240, EPI_ISL_4348270, EPI_ISL_4348271, EPI_ISL_4730425	Lahad Datu Hospital	Institute for Medical Research, Infectious Disease Research Centre, National Institutes of Health, Ministry of Health Malaysia	Ahmad FA; Ahmad Fazilah NA; Anasir MI; Azizan MA; Kamel K; Mohd Zawawi Z; Norhisham SN; Norhisyam SN; Ramly N; Robert F; Suppiah J; Thayan R
EPI_ISL_4730417	Melaka Tengah District Health Office	Institute for Medical Research, Infectious Disease Research Centre, National Institutes of Health, Ministry of Health Malaysia	Ahmad FA; Ahmad Fazilah NA; Anasir MI; Azizan MA; Kamel K; Mohd Zawawi Z; Norhisyam SN; Ramly N; Robert F; Suppiah J; Thayan R
EPI_ISL_4051653, EPI_ISL_4051689, EPI_ISL_4051690, EPI_ISL_4051691, EPI_ISL_4051692, EPI_ISL_4051706, EPI_ISL_4051707, EPI_ISL_4051708, EPI_ISL_4051709, EPI_ISL_4051710, EPI_ISL_4051711, EPI_ISL_4051712, EPI_ISL_4051713, EPI_ISL_4051714, EPI_ISL_4051719, EPI_ISL_4051737, EPI_ISL_4051738, EPI_ISL_4051748, EPI_ISL_4051764, EPI_ISL_4051765, EPI_ISL_4051766, EPI_ISL_4051767, EPI_ISL_4051768, EPI_ISL_4051829, EPI_ISL_4051831, EPI_ISL_4051837, EPI_ISL_4051838, EPI_ISL_4051844, EPI_ISL_4051845, EPI_ISL_4051850, EPI_ISL_4051861			
see above	Ministry of Health Hospitals	Institute of Health and Community Medicine	Chan Chia Jui; Chua Hock Hin; David Perera; Ooi Mong How; Tan Lee See; Tonnii Sia Loong Loong; Wong Jyn Shan; Wong Kiing Aik
EPI_ISL_4051654, EPI_ISL_4051655, EPI_ISL_4051656, EPI_ISL_4051721, EPI_ISL_4051722, EPI_ISL_4051723, EPI_ISL_4051724, EPI_ISL_4051725, EPI_ISL_4051857, EPI_ISL_4051864, EPI_ISL_4460097, EPI_ISL_4460098, EPI_ISL_4460099, EPI_ISL_4460100, EPI_ISL_4460101			
see above	Miri Hospital Molecular Diagnostic Lab	Institute of Health and Community Medicine	Chan Chia Jui; Chua Hock Hin; David Perera; Hanis Syazwani Mohd Hassan; Ooi Mong How; Tonnii Sia Loong Loong; Wong Jyn Shan
EPI_ISL_3769582	National Public Health Laboratory	Institute for Medical Research, Infectious Disease Research Centre, National Institutes of Health, Ministry of Health Malaysia	Anasir MI; Azizan MA; Kamel K; Mohd Zawawi Z; Ramly N; Robert F; Suppiah J; Thayan R
EPI_ISL_5417152	PATHOLOGY HOSPITAL AMPANG	Department of Medical Microbiology, University Malaya Medical Centre	I-Ching SAM; Jolene Yin Ling FU; Omar Khalilur Rahman; Yoke Fun Chan
EPI_ISL_4122406, EPI_ISL_4122415, EPI_ISL_4122427, EPI_ISL_4122551	PEJ KESIHATAN BARAT DAYA	iPROMISE, UiTM	Ariza Adnan; Fadzilah Mohd Nor; Lim Wai Feng; Mohd Asif Mohd Sukri; Mohd Nur Fakhruzzaman Noorizhab; Mohd Zaki Salleh; Sazzli Shahlan Kassim; Siti Farah Alwani Mohd Nawi; Siti Hamimah Sheikh Abdul Kadir; Teh Lay Kek; Wang Seok Mui
EPI_ISL_5417616	PEJABAT KESIHATAN DAERAH BATU PAHAT	UKM Medical Molecular Biology Institute (UMBI)	Mira Farzana binti Mohamad Mokhtar
EPI_ISL_8090608	PEJABAT KESIHATAN DAERAH KUALA KRAI	Tropical Infectious Diseases Research & Education Centre (TIDREC), Universiti Malaya	AsmaAnati CheMatSeri; Che-Norainon Yaacob; Jia-Yi Tan; Jo-Ern Wong; Kim-Kee Tan; Mulya-Mustika-Sari Zulkifli; Noor Syahida Azizan; Nur-Hidayana Mahfodz; Sazaly AbuBakar; Siti-Sarah Nor'e
EPI_ISL_3858171	PKD Alor Gajah	UKM Medical Molecular Biology Institute (UMBI)	Mira Farzana binti Mohamad Mokhtar
EPI_ISL_3945553, EPI_ISL_5159355, EPI_ISL_5159356	PKD KOTA TINGGI	UKM Medical Molecular Biology Institute (UMBI)	Mira Farzana binti Mohamad Mokhtar; Nur Alyaa Afifah Md Shahri
EPI_ISL_5417619, EPI_ISL_5417620	PKD KULAI	UKM Medical Molecular Biology Institute (UMBI)	Mira Farzana binti Mohamad Mokhtar
EPI_ISL_3945554	PKD SEREMBAN	UKM Medical Molecular Biology Institute (UMBI)	Nur Alyaa Afifah Md Shahri
EPI_ISL_3980731	PKD Tanah Merah	Tropical Infectious Diseases Research & Education Centre (TIDREC), Universiti Malaya	AsmaAnati CheMatSeri; Che-Norainon Yaacob; Jia-Yi Tan; Jo-Ern Wong; Kim-Kee Tan; Mulya-Mustika-Sari Zulkifli; Noor Syahida Azizan; Nur-Hidayana Mahfodz; Sazaly AbuBakar; Siti-Sarah Nor'e
EPI_ISL_5852778	Pahang State Health Department	Institute for Medical Research, Infectious Disease Research Centre, National Institutes of Health, Ministry of Health Malaysia	Ahmad FA; Ahmad Fazilah NA; Anasir MI; Azizan MA; Kamel K; Mohd Zawawi Z; Norhisham SN; Ramly N; Robert F; Suppiah J; Thayan R
EPI_ISL_3945550, EPI_ISL_3945551, EPI_ISL_3945552	Pejabat Kesihatan Batu Pahat	UKM Medical Molecular Biology Institute (UMBI)	Nur Alyaa Afifah Md Shahri
EPI_ISL_4557953, EPI_ISL_4558237, EPI_ISL_4558238, EPI_ISL_6825223	Pejabat Kesihatan Daerah Kota Marudu	Malaysia Genome Institute	Azrin Ahmad; Enizza Kasim; Irni Suhayu Sapian; Mohd Faizal Abu Bakar; Mohd Ghows Mohd Azzam.; Mohd Noor Mat Isa; Nor Azfa Johari; Nurhezreen Md Iqbal; Shamsidar Sopie; Siti Noraini Othman; Yusuf Muhammad Noor
EPI_ISL_4557954	Pejabat Kesihatan Daerah Sipitang	Malaysia Genome Institute	Azrin Ahmad; Enizza Kasim; Irni Suhayu Sapian; Mohd Faizal Abu Bakar; Mohd Ghows Mohd Azzam.; Mohd Noor Mat Isa; Nor Azfa Johari; Nurhezreen Md Iqbal; Shamsidar Sopie; Siti Noraini Othman; Yusuf Muhammad Noor
EPI_ISL_3769345	Pejabat Kesihatan Pintu Antarabangsa	iPROMISE, UiTM	Ariza Adnan; Fadzilah Mohd Nor; Lim Wai Feng; Mohd Asif Mohd Sukri; Mohd Nur Fakhruzzaman Noorizhab; Mohd Zaki Salleh; Sazzli Shahlan Kassim; Siti Farah Alwani Mohd Nawi; Siti Hamimah Sheikh Abdul Kadir; Teh Lay Kek; Wang Seok Mui
EPI_ISL_3769339, EPI_ISL_3769349, EPI_ISL_3769350, EPI_ISL_3769351, EPI_ISL_3769352, EPI_ISL_3769353	Pejabat Kesihatan Pintu Masuk Antarabangsa	iPROMISE, UiTM	Ariza Adnan; Fadzilah Mohd Nor; Lim Wai Feng; Mohd Asif Mohd Sukri; Mohd Nur Fakhruzzaman Noorizhab; Mohd Zaki Salleh; Sazzli Shahlan Kassim; Siti Farah Alwani Mohd Nawi; Siti Hamimah Sheikh Abdul Kadir; Teh Lay Kek; Wang Seok Mui
EPI_ISL_3769336, EPI_ISL_3769337, EPI_ISL_3769338, EPI_ISL_3769341, EPI_ISL_3769342, EPI_ISL_3769346	Pejabat Kesihatan Pintu Masuk Antarabangsa Pulau Pinang	iPROMISE, UiTM	Ariza Adnan; Fadzilah Mohd Nor; Lim Wai Feng; Mohd Asif Mohd Sukri; Mohd Nur Fakhruzzaman Noorizhab; Mohd Zaki Salleh; Sazzli Shahlan Kassim; Siti Farah Alwani Mohd Nawi; Siti Hamimah Sheikh Abdul Kadir; Teh Lay Kek; Wang Seok Mui
EPI_ISL_3762826	Penang General Hospital	Institute for Medical Research, Infectious Disease Research Centre, National Institutes of Health, Ministry of Health Malaysia	Anasir MI; Azizan MA; Kamel K; Mohd Zawawi Z; Ramly N; Robert F; Suppiah J; Thayan R
EPI_ISL_5396592, EPI_ISL_5396594	Public Health Unit, Enche' Besar Hajjah Khalsom Hospital (HEBHK)	Institute for Medical Research, Infectious Disease Research Centre, National Institutes of Health, Ministry of Health Malaysia	Ahmad FA; Ahmad Fazilah NA; Anasir MI; Azizan MA; Kamel K; Mohd Zawawi Z; Norhisham SN; Ramly N; Robert F; Suppiah J; Thayan R
EPI_ISL_4346231	Queen Elizabeth Hospital	Institute for Medical Research, Infectious Disease Research Centre, National Institutes of Health, Ministry of Health Malaysia	Ahmad FA; Ahmad Fazilah NA; Anasir MI; Azizan MA; Kamel K; Mohd Zawawi Z; Norhisyam SN; Ramly N; Robert F; Suppiah J; Thayan R
EPI_ISL_4730467	Salak Health Clinic	Institute for Medical Research, Infectious Disease Research Centre, National Institutes of Health, Ministry of Health Malaysia	Ahmad FA; Ahmad Fazilah NA; Anasir MI; Azizan MA; Kamel K; Mohd Zawawi Z; Norhisham SN; Ramly N; Robert F; Suppiah J; Thayan R
EPI_ISL_4051662, EPI_ISL_4051663, EPI_ISL_4051677, EPI_ISL_4051730, EPI_ISL_4051731, EPI_ISL_4051732, EPI_ISL_4051733, EPI_ISL_4051734, EPI_ISL_4051735, EPI_ISL_4051736, EPI_ISL_4051739, EPI_ISL_4051740, EPI_ISL_4051741, EPI_ISL_4051742, EPI_ISL_4051743, EPI_ISL_4051744, EPI_ISL_4051745, EPI_ISL_4051746, EPI_ISL_4051747, EPI_ISL_4051749, EPI_ISL_4051750, EPI_ISL_4051751, EPI_ISL_4051752, EPI_ISL_4051753, EPI_ISL_4051754, EPI_ISL_4051755, EPI_ISL_4051756, EPI_ISL_4051757, EPI_ISL_4051758, EPI_ISL_4051759, EPI_ISL_4051760, EPI_ISL_4051761, EPI_ISL_4051762, EPI_ISL_4051763, EPI_ISL_4051830, EPI_ISL_4051842, EPI_ISL_4051849			
see above	Sarawak Heart Centre (SHC)	Institute of Health and Community Medicine	Chan Chia Jui; Chua Hock Hin; David Perera; Ooi Mong How; Tonnii Sia Loong Loong; Wong Jyn Shan; Wong Kiing Aik
EPI_ISL_6424031	Segamat Hospital	Institute for Medical Research, Infectious Disease Research Centre, National Institutes of Health, Ministry of Health Malaysia	Ahmad FA; Ahmad Fazilah NA; Anasir MI; Azizan MA; Kamel K; Mohd Zawawi Z; Norhisham SN; Ramly N; Robert F; Suppiah J; Thayan R
EPI_ISL_4096802	Selayang Hospital	Institute for Medical Research, Infectious Disease Research Centre, National Institutes of Health, Ministry of Health Malaysia	Ahmad FA; Ahmad Fazilah NA; Anasir MI; Azizan MA; Kamel K; Mohd Zawawi Z; Norhisham SN; Ramly N; Robert F; Suppiah J; Thayan R
EPI_ISL_4730464	Sepang District Health Office	Institute for Medical Research, Infectious Disease Research Centre, National Institutes of Health, Ministry of Health Malaysia	Ahmad FA; Ahmad Fazilah NA; Anasir MI; Azizan MA; Kamel K; Mohd Zawawi Z; Norhisham SN; Ramly N; Robert F; Suppiah J; Thayan R
EPI_ISL_6774055	Sultan Ismail Hospital	Institute for Medical Research, Infectious Disease Research Centre, National Institutes of Health, Ministry of Health Malaysia	Ahmad FA; Ahmad Fazilah NA; Anasir MI; Azizan MA; Kamel K; Mohamad Sukri MZ; Mohd Zawawi Z; Norhisham SN; Ramly N; Robert F; Rosli NR; Suppiah J; Thayan R
EPI_ISL_4730432, EPI_ISL_6424645, EPI_ISL_6424647, EPI_ISL_6424675, EPI_ISL_6774046, EPI_ISL_7379966	Sultanah Bahiyah Hospital	Institute for Medical Research, Infectious Disease Research Centre, National Institutes of Health, Ministry of Health Malaysia	Ahmad FA; Ahmad Fazilah NA; Anasir MI; Azizan MA; Kamel K; Mohamad Sukri MZ; Mohd Zawawi Z; Norhisham SN; Ramly N; Robert F; Rosli NR; Suppiah J; Thayan R
EPI_ISL_5113904	Sultanah Fatimah Specialist Hospital	Institute for Medical Research, Infectious Disease Research Centre, National Institutes of Health, Ministry of Health Malaysia	Ahmad FA; Ahmad Fazilah NA; Anasir MI; Azizan MA; Kamel K; Mohd Zawawi Z; Norhisham SN; Ramly N; Robert F; Suppiah J; Thayan R
EPI_ISL_6774022, EPI_ISL_6774029	Sultanah Fatimah Specialist Hospital, Muar	Institute for Medical Research, Infectious Disease Research Centre, National Institutes of Health, Ministry of Health Malaysia	Ahmad FA; Ahmad Fazilah NA; Anasir MI; Azizan MA; Kamel K; Mohamad Sukri MZ; Mohd Zawawi Z; Norhisham SN; Ramly N; Robert F; Rosli NR; Suppiah J; Thayan R
EPI_ISL_4096796, EPI_ISL_4096797, EPI_ISL_4096798, EPI_ISL_4096799, EPI_ISL_6773978, EPI_ISL_6774018, EPI_ISL_6774019, EPI_ISL_6774050, EPI_ISL_6774051, EPI_ISL_6774052, EPI_ISL_6774053, EPI_ISL_7457550, EPI_ISL_7457646			
see above	Sungai Buloh Hospital	Institute for Medical Research, Infectious Disease Research Centre, National Institutes of Health, Ministry of Health Malaysia	Ahmad FA; Ahmad Fazilah NA; Anasir MI; Azizan MA; Kamel K; Mohamad Sukri MZ; Mohd Zawawi Z; Norhisham SN; Ramly N; Robert F; Rosli NR; Suppiah J; Thayan R
EPI_ISL_3762819	Tampin District Health	Institute for Medical Research, Infectious Disease Research Centre, National Institutes of Health, Ministry of Health Malaysia	Anasir MI; Azizan MA; Kamel K; Mohd Zawawi Z; Ramly N; Robert F; Suppiah J; Thayan R
EPI_ISL_3762820, EPI_ISL_3762821	Tampin District Health Office	Institute for Medical Research, Infectious Disease Research Centre, National Institutes of Health, Ministry of Health Malaysia	Anasir MI; Azizan MA; Kamel K; Mohd Zawawi Z; Ramly N; Robert F; Suppiah J; Thayan R
EPI_ISL_3762833, EPI_ISL_3762834, EPI_ISL_3762836, EPI_ISL_4346611, EPI_ISL_4347144, EPI_ISL_6773977	Tawau Area Health Office	Institute for Medical Research, Infectious Disease Research Centre, National Institutes of Health, Ministry of Health Malaysia	Ahmad FA; Ahmad Fazilah NA; Anasir MI; Azizan MA; Kamel K; Mohamad Sukri MZ; Mohd Zawawi Z; Norhisham SN; Norhisyam SN; Ramly N; Robert F; Rosli NR; Suppiah J; Thayan R
EPI_ISL_4636748	Tengku Ampuan Rahimah Hospital	Institute for Medical Research, Infectious Disease Research Centre, National Institutes of Health, Ministry of Health Malaysia	Ahmad FA; Ahmad Fazilah NA; Anasir MI; Azizan MA; Kamel K; Mohd Zawawi Z; Norhisyam SN; Ramly N; Robert F; Suppiah J; Thayan R

EPI_ISL_4730436, EPI_ISL_7380014	Terengganu State Health Office	Institute for Medical Research, Infectious Disease Research Centre, National Institutes of Health, Ministry of Health Malaysia	Ahmad FA; Ahmad Fazilah NA; Anasir MI; Azizan MA; Kamel K; Mohamad Sukri MZ; Mohd Zawawi Z; Norhisham SN; Ramly N; Robert F; Rosli NR; Suppiah J; Thayan R
EPI_ISL_7457649	Terengganu state health office	Institute for Medical Research, Infectious Disease Research Centre, National Institutes of Health, Ministry of Health Malaysia	Ahmad FA; Ahmad Fazilah NA; Anasir MI; Azizan MA; Kamel K; Mohamad Sukri MZ; Mohd Zawawi Z; Norhisham SN; Ramly N; Robert F; Rosli NR; Suppiah J; Thayan R
EPI_ISL_7380536	Tuanku Ampuan Najihah Hospital	Institute for Medical Research, Infectious Disease Research Centre, National Institutes of Health, Ministry of Health Malaysia	Ahmad FA; Ahmad Fazilah NA; Anasir MI; Azizan MA; Kamel K; Mohamad Sukri MZ; Mohd Zawawi Z; Norhisham SN; Ramly N; Robert F; Rosli NR; Suppiah J; Thayan R
EPI_ISL_4730404	Tuanku Ja'afar Hospital	Institute for Medical Research, Infectious Disease Research Centre, National Institutes of Health, Ministry of Health Malaysia	Ahmad FA; Ahmad Fazilah NA; Anasir MI; Azizan MA; Kamel K; Mohd Zawawi Z; Norhisyam SN; Ramly N; Robert F; Suppiah J; Thayan R
EPI_ISL_4730405	Tuanku Mizan Military's Hospital	Institute for Medical Research, Infectious Disease Research Centre, National Institutes of Health, Ministry of Health Malaysia	Ahmad FA; Ahmad Fazilah NA; Anasir MI; Azizan MA; Kamel K; Mohd Zawawi Z; Norhisyam SN; Ramly N; Robert F; Suppiah J; Thayan R
EPI_ISL_3769374, EPI_ISL_3769376	UKA	iPROMISE, UiTM	Ariza Adnan; Fadzilah Mohd Nor; Lim Wai Feng; Mohd Asif Mohd Sukri; Mohd Nur Fakhruzzaman Noorizhab; Mohd Zaki Salleh; Sazzli Shahlan Kassim; Siti Farah Alwani Mohd Nawi; Siti Hamimah Sheikh Abdul Kadir; Teh Lay Kek; Wang Seok Mui
EPI_ISL_8090597	UKKIP, SW28, Jabatan Perubatan Am, Hospital Kuala Lumpur	Tropical Infectious Diseases Research & Education Centre (TIDREC), Universiti Malaya	AsmaAnati CheMatSeri; Che-Norainon Yaacob; Jia-Yi Tan; Jo-Ern Wong; Kim-Kee Tan; Mulya-Mustika-Sari Zulkifli; Noor Syahida Azizan; Nur-Hidayana Mahfodz; Sazaly AbuBakar; Siti-Sarah Nor'e
EPI_ISL_7189353, EPI_ISL_7189358, EPI_ISL_7189359, EPI_ISL_7189364, EPI_ISL_7189365, EPI_ISL_7189369, EPI_ISL_7189370, EPI_ISL_7189371			
see above	UMMC	Department of Medical Microbiology, Faculty of Medicine, University of Malaya; University of Malaya Medical Centre	I-Ching SAM; Jolene Yin Ling FU; Omar Khalilur Rahman; Yoke Fun Chan
EPI_ISL_3980698	UNIT KESELAMATAN DAN KESIHATAN HOSPITAL SULTANAH BAHIYAH	Tropical Infectious Diseases Research & Education Centre (TIDREC), Universiti Malaya	AsmaAnati CheMatSeri; Che-Norainon Yaacob; Jia-Yi Tan; Jo-Ern Wong; Kim-Kee Tan; Mulya-Mustika-Sari Zulkifli; Noor Syahida Azizan; Nur-Hidayana Mahfodz; Sazaly AbuBakar; Siti-Sarah Nor'e
EPI_ISL_3980696, EPI_ISL_3980697	UNIT KESELAMATAN DAN KESIHATAN PEKERJA HOSPITAL SULTANAH BAHIYAH	Tropical Infectious Diseases Research & Education Centre (TIDREC), Universiti Malaya	AsmaAnati CheMatSeri; Che-Norainon Yaacob; Jia-Yi Tan; Jo-Ern Wong; Kim-Kee Tan; Mulya-Mustika-Sari Zulkifli; Noor Syahida Azizan; Nur-Hidayana Mahfodz; Sazaly AbuBakar; Siti-Sarah Nor'e
EPI_ISL_8090596	UNIT KESIHATAN AWAM	Tropical Infectious Diseases Research & Education Centre (TIDREC), Universiti Malaya	AsmaAnati CheMatSeri; Che-Norainon Yaacob; Jia-Yi Tan; Jo-Ern Wong; Kim-Kee Tan; Mulya-Mustika-Sari Zulkifli; Noor Syahida Azizan; Nur-Hidayana Mahfodz; Sazaly AbuBakar; Siti-Sarah Nor'e
EPI_ISL_3980733	UNIT KESIHATAN AWAM ,UKA . HRPZ	Tropical Infectious Diseases Research & Education Centre (TIDREC), Universiti Malaya	AsmaAnati CheMatSeri; Che-Norainon Yaacob; Jia-Yi Tan; Jo-Ern Wong; Kim-Kee Tan; Mulya-Mustika-Sari Zulkifli; Noor Syahida Azizan; Nur-Hidayana Mahfodz; Sazaly AbuBakar; Siti-Sarah Nor'e
EPI_ISL_3858183	UNIT KESIHATAN AWAM HEBHK	UKM Medical Molecular Biology Institute (UMBI)	Mira Farzana binti Mohamad Mokhtar
EPI_ISL_3980702	UNIT KESIHATAN DAN KESELAMATA PEKERJA HOSPITAL SULTANAH BAHIYAH	Tropical Infectious Diseases Research & Education Centre (TIDREC), Universiti Malaya	AsmaAnati CheMatSeri; Che-Norainon Yaacob; Jia-Yi Tan; Jo-Ern Wong; Kim-Kee Tan; Mulya-Mustika-Sari Zulkifli; Noor Syahida Azizan; Nur-Hidayana Mahfodz; Sazaly AbuBakar; Siti-Sarah Nor'e
EPI_ISL_3980701, EPI_ISL_3980703, EPI_ISL_3980706	UNIT KESIHATAN DAN KESELAMATAN HOSPITAL SULTANAH BAHIYAH	Tropical Infectious Diseases Research & Education Centre (TIDREC), Universiti Malaya	AsmaAnati CheMatSeri; Che-Norainon Yaacob; Jia-Yi Tan; Jo-Ern Wong; Kim-Kee Tan; Mulya-Mustika-Sari Zulkifli; Noor Syahida Azizan; Nur-Hidayana Mahfodz; Sazaly AbuBakar; Siti-Sarah Nor'e
EPI_ISL_3980700, EPI_ISL_3980704, EPI_ISL_3980705, EPI_ISL_3980707	UNIT KESIHATAN DAN KESELAMATAN PEKERJA HOSPITAL SULTANAH BAHIYAH	Tropical Infectious Diseases Research & Education Centre (TIDREC), Universiti Malaya	AsmaAnati CheMatSeri; Che-Norainon Yaacob; Jia-Yi Tan; Jo-Ern Wong; Kim-Kee Tan; Mulya-Mustika-Sari Zulkifli; Noor Syahida Azizan; Nur-Hidayana Mahfodz; Sazaly AbuBakar; Siti-Sarah Nor'e
EPI_ISL_3980699	UNIT KESIHATAN DAN KESIHATAN PEKERJA HOSPITAL SULTANAH BAHIYAH	Tropical Infectious Diseases Research & Education Centre (TIDREC), Universiti Malaya	AsmaAnati CheMatSeri; Che-Norainon Yaacob; Jia-Yi Tan; Jo-Ern Wong; Kim-Kee Tan; Mulya-Mustika-Sari Zulkifli; Noor Syahida Azizan; Nur-Hidayana Mahfodz; Sazaly AbuBakar; Siti-Sarah Nor'e
EPI_ISL_3858150, EPI_ISL_3858152, EPI_ISL_3858161, EPI_ISL_3858165, EPI_ISL_3858167	UNIT KPAS, PKD MELAKA TENGAH	UKM Medical Molecular Biology Institute (UMBI)	Mira Farzana binti Mohamad Mokhtar
EPI_ISL_4565026, EPI_ISL_4565028, EPI_ISL_8090594, EPI_ISL_8090598, EPI_ISL_8090599, EPI_ISL_8090644, EPI_ISL_8090645, EPI_ISL_8090646, EPI_ISL_8090647, EPI_ISL_8090648, EPI_ISL_8090649, EPI_ISL_8090650			
see above	UNIT OSH, HOSPITAL SUNGAI BULOH	Tropical Infectious Diseases Research & Education Centre (TIDREC), Universiti Malaya	AsmaAnati CheMatSeri; Che-Norainon Yaacob; Jia-Yi Tan; Jo-Ern Wong; Kim-Kee Tan; Mulya-Mustika-Sari Zulkifli; Noor Syahida Azizan; Nur-Hidayana Mahfodz; Sazaly AbuBakar; Siti-Sarah Nor'e
EPI_ISL_3769375	UNIT OSH, HOSPITAL SUNGAI BULOH	iPROMISE, UiTM	Ariza Adnan; Fadzilah Mohd Nor; Lim Wai Feng; Mohd Asif Mohd Sukri; Mohd Nur Fakhruzzaman Noorizhab; Mohd Zaki Salleh; Sazzli Shahlan Kassim; Siti Farah Alwani Mohd Nawi; Siti Hamimah Sheikh Abdul Kadir; Teh Lay Kek; Wang Seok Mui
EPI_ISL_4513498	Unit keselamatan dan Kesihatan Pekerjaan	UKM Medical Molecular Biology Institute (UMBI)	Mira Farzana binti Mohamad Mokhtar
EPI_ISL_5261878	jabatan forensik	iPROMISE, UiTM	Ariza Adnan; Fadzilah Mohd Nor; Lim Wai Feng; Mohd Asif Mohd Sukri; Mohd Nur Fakhruzzaman Noorizhab; Mohd Zaki Salleh; Sazzli Shahlan Kassim; Siti Farah Alwani Mohd Nawi; Siti Hamimah Sheikh Abdul Kadir; Teh Lay Kek; Wang Seok Mui
EPI_ISL_3980729	klinik kesihatan perol	Tropical Infectious Diseases Research & Education Centre (TIDREC), Universiti Malaya	AsmaAnati CheMatSeri; Che-Norainon Yaacob; Jia-Yi Tan; Jo-Ern Wong; Kim-Kee Tan; Mulya-Mustika-Sari Zulkifli; Noor Syahida Azizan; Nur-Hidayana Mahfodz; Sazaly AbuBakar; Siti-Sarah Nor'e

We gratefully acknowledge the following Authors from the Originating laboratories responsible for obtaining the specimens, as well as the Submitting laboratories where the genome data were generated and shared via GISAID, on which this research is based.

All Submitters of data may be contacted directly via www.gisaid.org

Authors are sorted alphabetically.

Acknowledgement EPI_SET Identifier: EPI_SET_20220603hr

Accession ID	Originating Laboratory	Submitting Laboratory	Authors
EPI_ISL_4096804, EPI_ISL_4096806, EPI_ISL_4096808, EPI_ISL_4096809, EPI_ISL_4096810	Ampang Hospital	Institute for Medical Research, Infectious Disease Research Centre, National Institutes of Health, Ministry of Health Malaysia	Ahmad FA; Ahmad Fazilah NA; Anasir MI; Azizan MA; Kamel K; Mohd Zawawi Z; Norhisham SN; Ramly N; Robert F; Suppiah J; Thayan R
EPI_ISL_4348280, EPI_ISL_4348281, EPI_ISL_4348298, EPI_ISL_4348299, EPI_ISL_4348300	Banting Hospital	Institute for Medical Research, Infectious Disease Research Centre, National Institutes of Health, Ministry of Health Malaysia	Ahmad FA; Ahmad Fazilah NA; Anasir MI; Azizan MA; Kamel K; Mohd Zawawi Z; Norhisyam SN; Ramly N; Robert F; Suppiah J; Thayan R
EPI_ISL_4460090, EPI_ISL_4460095, EPI_ISL_4460096, EPI_ISL_4460156, EPI_ISL_4460157, EPI_ISL_4460158, EPI_ISL_4460159, EPI_ISL_4460160, EPI_ISL_4460161, EPI_ISL_4460162, EPI_ISL_4460163, EPI_ISL_4460164, EPI_ISL_4460165, EPI_ISL_4460166, EPI_ISL_4460167, EPI_ISL_4460171, EPI_ISL_4460172, EPI_ISL_4460173, EPI_ISL_4460174, EPI_ISL_4460175, EPI_ISL_4460176, EPI_ISL_4460177, EPI_ISL_4460178, EPI_ISL_4460179, EPI_ISL_4460180, EPI_ISL_4460181, EPI_ISL_4460182, EPI_ISL_4460183, EPI_ISL_4460184, EPI_ISL_4460185, EPI_ISL_4460186, EPI_ISL_4460187, EPI_ISL_4460188, EPI_ISL_4460189, EPI_ISL_4460190, EPI_ISL_4460191, EPI_ISL_4460192, EPI_ISL_4460193, EPI_ISL_4460194, EPI_ISL_4460195, EPI_ISL_4460196, EPI_ISL_4460197, EPI_ISL_4460198, EPI_ISL_4460199, EPI_ISL_4460200, EPI_ISL_4460201, EPI_ISL_4460202, EPI_ISL_4460203, EPI_ISL_4460204, EPI_ISL_4460205, EPI_ISL_4460206, EPI_ISL_4460320, EPI_ISL_4460322, EPI_ISL_4460323, EPI_ISL_4460334, EPI_ISL_4460335, EPI_ISL_4460336, EPI_ISL_4460337, EPI_ISL_4460338, EPI_ISL_4460339, EPI_ISL_4460340, EPI_ISL_4460346, EPI_ISL_4460347, EPI_ISL_4460348, EPI_ISL_4460349			
see above	Bintulu Hospital PCR Lab, Bintulu	Institute of Health and Community Medicine	Chan Chia Jui; Chua Hock Hin; David Perera; Ooi Mong How; Tan Lee See; Tonnii Sia Loong Loong; Wong Jyn Shan
EPI_ISL_5051749, EPI_ISL_5051804, EPI_ISL_5051805, EPI_ISL_5051806, EPI_ISL_5051807, EPI_ISL_5051808, EPI_ISL_5051809, EPI_ISL_5051810, EPI_ISL_5051811, EPI_ISL_5051812, EPI_ISL_5051813, EPI_ISL_5051814, EPI_ISL_5051815, EPI_ISL_5051816, EPI_ISL_5051955, EPI_ISL_5051956, EPI_ISL_5051957, EPI_ISL_5051958, EPI_ISL_5051959, EPI_ISL_5052172, EPI_ISL_5896271			
see above	Bintulu Medical Centre (Bintulu)	Institute of Health and Community Medicine	Chan Chia Jui; Chua Hock Hin; David Perera; Ooi Mong How; Tonnii Sia Loong Loong; Wong Jyn Shan
EPI_ISL_4460104, EPI_ISL_4460105, EPI_ISL_4460106, EPI_ISL_4460107, EPI_ISL_4460108, EPI_ISL_4460113, EPI_ISL_4460114, EPI_ISL_4460115, EPI_ISL_4460116, EPI_ISL_4460117, EPI_ISL_4460118, EPI_ISL_4460119, EPI_ISL_4460120, EPI_ISL_4460121, EPI_ISL_4460232, EPI_ISL_4460233, EPI_ISL_4460234, EPI_ISL_4460235, EPI_ISL_4460236, EPI_ISL_4460237, EPI_ISL_4460238, EPI_ISL_4460239, EPI_ISL_4460240, EPI_ISL_4460241, EPI_ISL_4460242, EPI_ISL_4460243, EPI_ISL_4460244, EPI_ISL_4460245, EPI_ISL_4460246, EPI_ISL_4460247, EPI_ISL_4460248, EPI_ISL_4460249, EPI_ISL_4460250, EPI_ISL_4460251, EPI_ISL_4460252, EPI_ISL_4460253, EPI_ISL_4460254, EPI_ISL_4460255, EPI_ISL_4460256, EPI_ISL_4460257, EPI_ISL_4460258, EPI_ISL_4460259, EPI_ISL_4460306, EPI_ISL_4460307, EPI_ISL_4460308, EPI_ISL_4460309, EPI_ISL_4460310, EPI_ISL_4460311, EPI_ISL_4460312, EPI_ISL_4460313, EPI_ISL_4460314			
see above	Borneo Medical Centre	Institute of Health and Community Medicine	Chan Chia Jui; Chua Hock Hin; David Perera; Ooi Mong How; Tonnii Sia Loong Loong; Wong Jyn Shan
EPI_ISL_4460350	Borneo Specialist Hospital, Miri	Institute of Health and Community Medicine	Chan Chia Jui; Chua Hock Hin; David Perera; Ooi Mong How; Tonnii Sia Loong Loong; Wong Jyn Shan
EPI_ISL_7086945	CPRC JKNT	Department of Medical Microbiology, Faculty of Medicine, University of Malaya; University of Malaya Medical Centre	I-Ching SAM; Jolene Yin Ling FU; Omar Khalilur Rahman; Yoke Fun Chan
EPI_ISL_3980728, EPI_ISL_4565000	CPRC JKNT	Tropical Infectious Diseases Research & Education Centre (TIDREC), Universiti Malaya	AsmaAnati CheMatSeri; Che-Norainon Yaacob; Jia-Yi Tan; Jo-Ern Wong; Kim-Kee Tan; Mulya-Mustika-Sari Zulkifli; Noor Syahida Azizan; Nur-Hidayana Mahfodz; Sazaly AbuBakar; Siti-Sarah Nor'e
EPI_ISL_4051789	CRC, Sibu Hospital	Institute of Health and Community Medicine	Chan Chia Jui; Chua Hock Hin; David Perera; Ooi Mong How; Tonnii Sia Loong Loong; Wong Jyn Shan; Wong Kiing Aik
EPI_ISL_4460207, EPI_ISL_4460208, EPI_ISL_4460209, EPI_ISL_4460210, EPI_ISL_4460211, EPI_ISL_4460218, EPI_ISL_4460219, EPI_ISL_4460220, EPI_ISL_4460228, EPI_ISL_4460229, EPI_ISL_5051756, EPI_ISL_5051780, EPI_ISL_5051997, EPI_ISL_5051998, EPI_ISL_5052000, EPI_ISL_5052178			
see above	Clinical Research Centre (CRC), Sibu Hospital, Sibu	Institute of Health and Community Medicine	Chan Chia Jui; Chua Hock Hin; David Perera; Ooi Mong How; Tonnii Sia Loong Loong; Wong Jyn Shan; Wong Kiing Aik
EPI_ISL_5417130, EPI_ISL_5417131, EPI_ISL_5417149, EPI_ISL_5417158, EPI_ISL_5417163, EPI_ISL_5417170, EPI_ISL_5425841			
see above	Department of Medical Microbiology, University Malaya Medical Centre	Department of Medical Microbiology, University Malaya Medical Centre	I-Ching SAM; Jolene Yin Ling FU; Omar Khalilur Rahman; Yoke Fun Chan
EPI_ISL_4051681, EPI_ISL_4051797, EPI_ISL_4051798, EPI_ISL_4051799, EPI_ISL_4051800, EPI_ISL_4051801, EPI_ISL_4051802, EPI_ISL_4051803, EPI_ISL_4051804, EPI_ISL_4051805, EPI_ISL_4051806, EPI_ISL_4051807, EPI_ISL_4051808, EPI_ISL_4051809, EPI_ISL_4051810, EPI_ISL_4051811, EPI_ISL_4051812, EPI_ISL_4051813, EPI_ISL_4051814, EPI_ISL_4051815, EPI_ISL_4051816, EPI_ISL_4051817, EPI_ISL_4051828, EPI_ISL_4051841, EPI_ISL_4051843, EPI_ISL_4460143, EPI_ISL_4460144, EPI_ISL_4460145, EPI_ISL_4460146, EPI_ISL_4460147, EPI_ISL_4460148, EPI_ISL_4460149, EPI_ISL_4460150, EPI_ISL_4460151, EPI_ISL_4460152, EPI_ISL_4460153, EPI_ISL_4460154, EPI_ISL_4460155, EPI_ISL_4460168, EPI_ISL_4460169			
see above	Gribbles Pathology, Kuching	Institute of Health and Community Medicine	Chan Chia Jui; Chua Hock Hin; David Perera; Ooi Mong How; Reagan Entigu Linton; Tonnii Sia Loong Loong; Wong Jyn Shan
EPI_ISL_12628155, EPI_ISL_12628156, EPI_ISL_12628157, EPI_ISL_12628158, EPI_ISL_12628159	HCTM	UKM Medical Molecular Biology Institute (UMBI)	Khairun Nur Abd Ghafar; Mira Farzana Mohamad Mokhtar; Muhiddin Ishak; Nor Azila Muhammad Azami; Nur Alyaa Afifah Md Shahri; Nurul Syakima Ab Mutalib; Rahman Jamal; Ryia Illani Mohd Yunos; Siti Nur Hasanah Mohd Yusuf; Zahirrah Begam Mohamed Rasheed
EPI_ISL_3980741, EPI_ISL_3980743, EPI_ISL_3980745	HOSPITAL AMPANG	Tropical Infectious Diseases Research & Education Centre (TIDREC), Universiti Malaya	AsmaAnati CheMatSeri; Che-Norainon Yaacob; Jia-Yi Tan; Jo-Ern Wong; Kim-Kee Tan; Mulya-Mustika-Sari Zulkifli; Noor Syahida Azizan; Nur-Hidayana Mahfodz; Sazaly AbuBakar; Siti-Sarah Nor'e
EPI_ISL_4740395, EPI_ISL_4740397, EPI_ISL_5261886	HOSPITAL BALIK PULAU	iPROMISE, UiTM	Ariza Adnan; Fadzilah Mohd Nor; Lim Wai Feng; Mohd Asif Mohd Sukri; Mohd Nur Fakhruzzaman Noorizhab; Mohd Zaki Salleh; Sazzli Shahlan Kassim; Siti Farah Alwani Mohd Nawi; Siti Hamimah Sheikh Abdul Kadir; Teh Lay Kek; Wang Seok Mui
EPI_ISL_3945537	HOSPITAL PAKAR SULTANAH FATIMAH, MUAR	UKM Medical Molecular Biology Institute (UMBI)	Nur Alyaa Afifah Md Shahri
EPI_ISL_4122284, EPI_ISL_4122544, EPI_ISL_4435514, EPI_ISL_4435517	HOSPITAL PULAU PINANG	iPROMISE, UiTM	Ariza Adnan; Fadzilah Mohd Nor; Lim Wai Feng; Mohd Asif Mohd Sukri; Mohd Nur Fakhruzzaman Noorizhab; Mohd Zaki Salleh; Sazzli Shahlan Kassim; Siti Farah Alwani Mohd Nawi; Siti Hamimah Sheikh Abdul Kadir; Teh Lay Kek; Wang Seok Mui
EPI_ISL_7086599, EPI_ISL_7086755	HOSPITAL SULTAN ABDUL HALIM	Department of Medical Microbiology, Faculty of Medicine, University of Malaya; University of Malaya Medical Centre	I-Ching SAM; Jolene Yin Ling FU; Omar Khalilur Rahman; Yoke Fun Chan
EPI_ISL_3945540, EPI_ISL_3945542, EPI_ISL_3945543, EPI_ISL_3945544, EPI_ISL_4071989, EPI_ISL_4071990, EPI_ISL_4071991, EPI_ISL_4071992, EPI_ISL_4071994, EPI_ISL_4071995, EPI_ISL_4071997			
see above	HOSPITAL SULTANAH NORA ISMAIL	UKM Medical Molecular Biology Institute (UMBI)	Mira Farzana binti Mohamad Mokhtar; Nur Alyaa Afifah Md Shahri
EPI_ISL_4122540	HPP	iPROMISE, UiTM	Ariza Adnan; Fadzilah Mohd Nor; Lim Wai Feng; Mohd Asif Mohd Sukri; Mohd Nur Fakhruzzaman Noorizhab; Mohd Zaki Salleh; Sazzli Shahlan Kassim; Siti Farah Alwani Mohd Nawi; Siti Hamimah Sheikh Abdul Kadir; Teh Lay Kek; Wang Seok Mui
EPI_ISL_3858142, EPI_ISL_3858144, EPI_ISL_3858146	HPSF MUAR	UKM Medical Molecular Biology Institute (UMBI)	Mira Farzana binti Mohamad Mokhtar
EPI_ISL_4071993, EPI_ISL_5159359, EPI_ISL_5417621	HPSF, MUAR	UKM Medical Molecular Biology Institute (UMBI)	Mira Farzana binti Mohamad Mokhtar
EPI_ISL_8090616, EPI_ISL_8090655	HRPB	Tropical Infectious Diseases Research & Education Centre (TIDREC), Universiti Malaya	AsmaAnati CheMatSeri; Che-Norainon Yaacob; Jia-Yi Tan; Jo-Ern Wong; Kim-Kee Tan; Mulya-Mustika-Sari Zulkifli; Noor Syahida Azizan; Nur-Hidayana Mahfodz; Sazaly AbuBakar; Siti-Sarah Nor'e
EPI_ISL_7087286, EPI_ISL_7087936	HRPB, Ipoh	Department of Medical Microbiology, Faculty of Medicine, University of Malaya; University of Malaya Medical Centre	I-Ching SAM; Jolene Yin Ling FU; Omar Khalilur Rahman; Yoke Fun Chan
EPI_ISL_3980750, EPI_ISL_4565007	HRPB, Ipoh	Tropical Infectious Diseases Research & Education Centre (TIDREC), Universiti Malaya	AsmaAnati CheMatSeri; Che-Norainon Yaacob; Jia-Yi Tan; Jo-Ern Wong; Kim-Kee Tan; Mulya-Mustika-Sari Zulkifli; Noor Syahida Azizan; Nur-Hidayana Mahfodz; Sazaly AbuBakar; Siti-Sarah Nor'e
EPI_ISL_7086876	HRPZ II	Department of Medical Microbiology, Faculty of Medicine, University of Malaya; University of Malaya Medical Centre	I-Ching SAM; Jolene Yin Ling FU; Omar Khalilur Rahman; Yoke Fun Chan
EPI_ISL_4557942, EPI_ISL_4557945, EPI_ISL_4557946, EPI_ISL_4557947, EPI_ISL_4557948, EPI_ISL_4557949, EPI_ISL_4557950, EPI_ISL_4557951			
see above	Hospital Ampang	Malaysia Genome Institute	Azrin Ahmad; Enizza Kasim; Irni Suhayu Sapian; Mohd Faizal Abu Bakar; Mohd Ghows Mohd Azzam.; Mohd Noor Mat Isa; Nor Azfa Johari; Nurhezreen Md Iqbal; Shamsidar Sopie; Siti Noraini Othman; Yusuf Muhammad Noor
EPI_ISL_3980749	Hospital Batu Gajah	Tropical Infectious Diseases Research & Education Centre (TIDREC), Universiti Malaya	AsmaAnati CheMatSeri; Che-Norainon Yaacob; Jia-Yi Tan; Jo-Ern Wong; Kim-Kee Tan; Mulya-Mustika-Sari Zulkifli; Noor Syahida Azizan; Nur-Hidayana Mahfodz; Sazaly AbuBakar; Siti-Sarah Nor'e
EPI_ISL_4513510	Hospital Canselor Tuanku Muhriz (HCTM)	UKM Medical Molecular Biology Institute (UMBI)	Mira Farzana binti Mohamad Mokhtar
EPI_ISL_4463193, EPI_ISL_4463194, EPI_ISL_4463195	Hospital Canselor Tuanku Muhriz UKM	UKM Medical Molecular Biology Institute (UMBI)	Nur Alyaa Afifah Md Shahri
EPI_ISL_5428535, EPI_ISL_5428543, EPI_ISL_5428544, EPI_ISL_5428545, EPI_ISL_5428546, EPI_ISL_5428547, EPI_ISL_5428548, EPI_ISL_5428551			
see above	Hospital Kajang	Malaysia Genome Institute	Azrin Ahmad; Enizza Kasim; Irni Suhayu Sapian; Mohd Faizal Abu Bakar; Mohd Ghows Mohd Azzam.; Mohd Noor Mat Isa; Nor Azfa Johari; Nurhezreen Md Iqbal; Shamsidar Sopie; Siti Noraini Othman; Yusuf Muhammad Noor
EPI_ISL_4122511, EPI_ISL_4435515, EPI_ISL_4435516, EPI_ISL_4435518, EPI_ISL_4435519	Hospital Kepala Batas	iPROMISE, UiTM	Ariza Adnan; Fadzilah Mohd Nor; Lim Wai Feng; Mohd Asif Mohd Sukri; Mohd Nur Fakhruzzaman Noorizhab; Mohd Zaki Salleh; Sazzli Shahlan Kassim; Siti Farah Alwani Mohd Nawi; Siti Hamimah Sheikh Abdul Kadir; Teh Lay Kek; Wang Seok Mui
EPI_ISL_6132042	Hospital Kulim	Department of Medical Microbiology, Faculty of Medicine, University of Malaya; University of Malaya Medical Centre	I-Ching SAM; Jolene Yin Ling FU; Omar Khalilur Rahman; Yoke Fun Chan
EPI_ISL_4853561	Hospital Labuan	Malaysia Genome Institute	Azrin Ahmad; Enizza Kasim; Irni Suhayu Sapian; Mohd Faizal Abu Bakar; Mohd Ghows Mohd Azzam.; Mohd Noor Mat Isa; Nor Azfa Johari; Nurhezreen Md Iqbal; Shamsidar Sopie; Siti Noraini Othman; Yusuf Muhammad Noor
EPI_ISL_4601565, EPI_ISL_4601570, EPI_ISL_4601571, EPI_ISL_4601579	Hospital Lahad Datu	Malaysia Genome Institute	Azrin Ahmad; Enizza Kasim; Irni Suhayu Sapian; Mohd Faizal Abu Bakar; Mohd Ghows Mohd Azzam.; Mohd Noor Mat Isa; Nor Azfa Johari; Nurhezreen Md Iqbal; Shamsidar Sopie; Siti Noraini Othman; Yusuf Muhammad Noor
EPI_ISL_4601560, EPI_ISL_4853596, EPI_ISL_4853613, EPI_ISL_4854010	Hospital Queen Elizabeth 2	Malaysia Genome Institute	Azrin Ahmad; Enizza Kasim; Irni Suhayu Sapian; Mohd Faizal Abu Bakar; Mohd Ghows Mohd Azzam.; Mohd Noor Mat Isa; Nor Azfa Johari; Nurhezreen Md Iqbal; Shamsidar Sopie; Siti Noraini Othman; Yusuf Muhammad Noor
EPI_ISL_5417622	Hospital Sultanah Nora Ismail	UKM Medical Molecular Biology Institute (UMBI)	Mira Farzana binti Mohamad Mokhtar

EPI_ISL_4557936	Hospital Sungai Buloh	Malaysia Genome Institute	Azrin Ahmad; Enizza Kasim; Irni Suhayu Sapian; Mohd Faizal Abu Bakar; Mohd Ghows Mohd Azzam.; Mohd Noor Mat Isa; Nor Azfa Johari; Nurhezreen Md Iqbal; Shamsidar Sopie; Siti Noraini Othman; Yusuf Muhammad Noor
EPI_ISL_6825250	Hospital Tawau	Malaysia Genome Institute	Azrin Ahmad; Enizza Kasim; Irni Suhayu Sapian; Mohd Faizal Abu Bakar; Mohd Ghows Mohd Azzam.; Mohd Noor Mat Isa; Nor Azfa Johari; Nurhezreen Md Iqbal; Shamsidar Sopie; Siti Noraini Othman; Yusuf Muhammad Noor
EPI_ISL_4101584	Hospital Tuanku Jaafar Seremban	UKM Medical Molecular Biology Institute (UMBI)	Nur Alyaa Afifah Md Shahri
EPI_ISL_3945549	Institut Biologi Molekul Perubatan UKM	UKM Medical Molecular Biology Institute (UMBI)	Nur Alyaa Afifah Md Shahri
EPI_ISL_6960054, EPI_ISL_6960055, EPI_ISL_6960056, EPI_ISL_6960057, EPI_ISL_6960058, EPI_ISL_6960059, EPI_ISL_6960060, EPI_ISL_11078709, EPI_ISL_11078712, EPI_ISL_11078726, EPI_ISL_11078729, EPI_ISL_11078733, EPI_ISL_11078734, EPI_ISL_11078738, EPI_ISL_11078745, EPI_ISL_11078753, EPI_ISL_11078755, EPI_ISL_11078756, EPI_ISL_11078765, EPI_ISL_11078766, EPI_ISL_11078775, EPI_ISL_11078783, EPI_ISL_11078786			
see above	Institut Biologi Molekul Perubatan UKM (UMBI)	UKM Medical Molecular Biology Institute (UMBI)	Khairun Nur Abd Ghafar; Mira Farzana Mohamad Mokhtar; Mira Farzana binti Mohamad Mokhtar; Muhiddin Ishak; Nor Azila Muhammad Azami; Nur Alyaa Afifah Md Shahri; Nurul Syakima Ab Mutalib; Rahman Jamal; Ryia Illani Mohd Yunos; Siti Nur Hasanah Mohd Yusuf; Zahirrah Begam Mohamed Rasheed
EPI_ISL_4740396, EPI_ISL_4740407, EPI_ISL_5261879	JABATAN FORENSIK	iPROMISE, UiTM	Ariza Adnan; Fadzilah Mohd Nor; Lim Wai Feng; Mohd Asif Mohd Sukri; Mohd Nur Fakhruzzaman Noorizhab; Mohd Zaki Salleh; Sazzli Shahlan Kassim; Siti Farah Alwani Mohd Nawi; Siti Hamimah Sheikh Abdul Kadir; Teh Lay Kek; Wang Seok Mui
EPI_ISL_5261881	JABATAN FORENSIK HOSPITAL PULAU PINANG	iPROMISE, UiTM	Ariza Adnan; Fadzilah Mohd Nor; Lim Wai Feng; Mohd Asif Mohd Sukri; Mohd Nur Fakhruzzaman Noorizhab; Mohd Zaki Salleh; Sazzli Shahlan Kassim; Siti Farah Alwani Mohd Nawi; Siti Hamimah Sheikh Abdul Kadir; Teh Lay Kek; Wang Seok Mui
EPI_ISL_7087116	JABATAN PATOLOGI, HRPZ II	Department of Medical Microbiology, Faculty of Medicine, University of Malaya; University of Malaya Medical Centre	I-Ching SAM; Jolene Yin Ling FU; Omar Khalilur Rahman; Yoke Fun Chan
EPI_ISL_6132031	JABATAN PERUBATAN FORENSIK HOSPITAL SULTANAH BAHIYAH	Department of Medical Microbiology, Faculty of Medicine, University of Malaya; University of Malaya Medical Centre	I-Ching SAM; Jolene Yin Ling FU; Omar Khalilur Rahman; Yoke Fun Chan
EPI_ISL_4101590	JABATAN PERUBATAN FORENSIK, HOSPITAL TUANKU JA'AFAR SEREMBAN	UKM Medical Molecular Biology Institute (UMBI)	Nur Alyaa Afifah Md Shahri
EPI_ISL_3769357, EPI_ISL_4122298, EPI_ISL_4122301, EPI_ISL_4122304, EPI_ISL_4122341, EPI_ISL_4122352, EPI_ISL_4122363, EPI_ISL_4122505, EPI_ISL_4122533, EPI_ISL_4122537, EPI_ISL_4435529, EPI_ISL_4435531, EPI_ISL_4435533, EPI_ISL_4435534, EPI_ISL_4435536, EPI_ISL_4435537, EPI_ISL_4435547, EPI_ISL_4435548, EPI_ISL_4740391, EPI_ISL_4740392, EPI_ISL_4740393			
see above	JKN PAHANG	iPROMISE, UiTM	Ariza Adnan; Fadzilah Mohd Nor; Lim Wai Feng; Mohd Asif Mohd Sukri; Mohd Nur Fakhruzzaman Noorizhab; Mohd Zaki Salleh; Sazzli Shahlan Kassim; Siti Farah Alwani Mohd Nawi; Siti Hamimah Sheikh Abdul Kadir; Teh Lay Kek; Wang Seok Mui
EPI_ISL_6132027, EPI_ISL_7087037, EPI_ISL_7087144	Jabatan Forensik Hospital Sultanah Bahiyah	Department of Medical Microbiology, Faculty of Medicine, University of Malaya; University of Malaya Medical Centre	I-Ching SAM; Jolene Yin Ling FU; Omar Khalilur Rahman; Yoke Fun Chan
EPI_ISL_4730468	Jenjarom Health Clinic	Institute for Medical Research, Infectious Disease Research Centre, National Institutes of Health, Ministry of Health Malaysia	Ahmad FA; Ahmad Fazilah NA; Anasir MI; Azizan MA; Kamel K; Mohd Zawawi Z; Norhisham SN; Ramly N; Robert F; Suppiah J; Thayan R
EPI_ISL_3419710	Johor Bahru Public Health Laboratory	Institute for Medical Research, Infectious Disease Research Centre, National Institutes of Health, Ministry of Health Malaysia	Anasir MI; Azizan MA; Kamel K; Mohd Zawawi Z; Ramly N; Robert F; Suppiah J; Thayan R
EPI_ISL_7087346, EPI_ISL_7087365, EPI_ISL_7087601, EPI_ISL_7087974	KLINIK KESIHATAN KUALA BETIS	Department of Medical Microbiology, Faculty of Medicine, University of Malaya; University of Malaya Medical Centre	I-Ching SAM; Jolene Yin Ling FU; Omar Khalilur Rahman; Yoke Fun Chan
EPI_ISL_4122374	KLINIK KESIHATAN TRIANG	iPROMISE, UiTM	Ariza Adnan; Fadzilah Mohd Nor; Lim Wai Feng; Mohd Asif Mohd Sukri; Mohd Nur Fakhruzzaman Noorizhab; Mohd Zaki Salleh; Sazzli Shahlan Kassim; Siti Farah Alwani Mohd Nawi; Siti Hamimah Sheikh Abdul Kadir; Teh Lay Kek; Wang Seok Mui
EPI_ISL_3769383	Klinik Kesihatan Kuala Perlis	iPROMISE, UiTM	Ariza Adnan; Fadzilah Mohd Nor; Lim Wai Feng; Mohd Asif Mohd Sukri; Mohd Nur Fakhruzzaman Noorizhab; Mohd Zaki Salleh; Sazzli Shahlan Kassim; Siti Farah Alwani Mohd Nawi; Siti Hamimah Sheikh Abdul Kadir; Teh Lay Kek; Wang Seok Mui
EPI_ISL_3980724	Klinik Kesihatan Padang Rengas	Tropical Infectious Diseases Research & Education Centre (TIDREC), Universiti Malaya	AsmaAnati CheMatSeri; Che-Norainon Yaacob; Jia-Yi Tan; Jo-Ern Wong; Kim-Kee Tan; Mulya-Mustika-Sari Zulkifli; Noor Syahida Azizan; Nur-Hidayana Mahfodz; Sazaly AbuBakar; Siti-Sarah Nor'e
EPI_ISL_4096795	Kuala Kubu Baru Hospital	Institute for Medical Research, Infectious Disease Research Centre, National Institutes of Health, Ministry of Health Malaysia	Ahmad FA; Ahmad Fazilah NA; Anasir MI; Azizan MA; Kamel K; Mohd Zawawi Z; Norhisham SN; Ramly N; Robert F; Suppiah J; Thayan R
EPI_ISL_4348275, EPI_ISL_4348276	Kuala Lumpur General Hospital	Institute for Medical Research, Infectious Disease Research Centre, National Institutes of Health, Ministry of Health Malaysia	Ahmad FA; Ahmad Fazilah NA; Anasir MI; Azizan MA; Kamel K; Mohd Zawawi Z; Norhisyam SN; Ramly N; Robert F; Suppiah J; Thayan R
EPI_ISL_4348272	Lahad Datu Hospital	Institute for Medical Research, Infectious Disease Research Centre, National Institutes of Health, Ministry of Health Malaysia	Ahmad FA; Ahmad Fazilah NA; Anasir MI; Azizan MA; Kamel K; Mohd Zawawi Z; Norhisyam SN; Ramly N; Robert F; Suppiah J; Thayan R
EPI_ISL_4051715, EPI_ISL_4051716, EPI_ISL_4051717, EPI_ISL_4051718, EPI_ISL_4051720, EPI_ISL_4051769, EPI_ISL_4051790, EPI_ISL_4051791, EPI_ISL_4051792, EPI_ISL_4051793, EPI_ISL_4051794, EPI_ISL_4051795, EPI_ISL_4051862			
see above	Ministry of Health Hospitals	Institute of Health and Community Medicine	Chan Chia Jui; Chua Hock Hin; David Perera; Ooi Mong How; Tonnii Sia Loong Loong; Wong Jyn Shan; Wong Kiing Aik
EPI_ISL_4460212, EPI_ISL_4460213, EPI_ISL_4460214, EPI_ISL_4460215, EPI_ISL_4460216	Miri Hospital Molecular Diagnostic Lab	Institute of Health and Community Medicine	Chan Chia Jui; Chua Hock Hin; David Perera; Hanis Syazwani Mohd Hassan; Ooi Mong How; Tonnii Sia Loong Loong; Wong Jyn Shan
EPI_ISL_4347856	National Institute of Medical Forensic	Institute for Medical Research, Infectious Disease Research Centre, National Institutes of Health, Ministry of Health Malaysia	Ahmad FA; Ahmad Fazilah NA; Anasir MI; Azizan MA; Kamel K; Mohd Zawawi Z; Norhisyam SN; Ramly N; Robert F; Suppiah J; Thayan R
EPI_ISL_4347725	National Medical Forensic Institute	Institute for Medical Research, Infectious Disease Research Centre, National Institutes of Health, Ministry of Health Malaysia	Ahmad FA; Ahmad Fazilah NA; Anasir MI; Azizan MA; Kamel K; Mohd Zawawi Z; Norhisyam SN; Ramly N; Robert F; Suppiah J; Thayan R
EPI_ISL_4101588, EPI_ISL_4731191	PEJABAT KESIHATAN DAERAH BATU PAHAT	UKM Medical Molecular Biology Institute (UMBI)	Mira Farzana binti Mohamad Mokhtar; Nur Alyaa Afifah Md Shahri
EPI_ISL_8146831	PEJABAT KESIHATAN DAERAH PASIR MAS	Tropical Infectious Diseases Research & Education Centre (TIDREC), Universiti Malaya	AsmaAnati CheMatSeri; Che-Norainon Yaacob; Jia-Yi Tan; Jo-Ern Wong; Kim-Kee Tan; Mulya-Mustika-Sari Zulkifli; Noor Syahida Azizan; Nur-Hidayana Mahfodz; Sazaly AbuBakar; Siti-Sarah Nor'e
EPI_ISL_7086834	PEJABAT KESIHATAN DAERAH PASIR PUTEH	Department of Medical Microbiology, Faculty of Medicine, University of Malaya; University of Malaya Medical Centre	I-Ching SAM; Jolene Yin Ling FU; Omar Khalilur Rahman; Yoke Fun Chan
EPI_ISL_4565002, EPI_ISL_4565003, EPI_ISL_4565004, EPI_ISL_4565005	PKD BACHOK	Tropical Infectious Diseases Research & Education Centre (TIDREC), Universiti Malaya	AsmaAnati CheMatSeri; Che-Norainon Yaacob; Jia-Yi Tan; Jo-Ern Wong; Kim-Kee Tan; Mulya-Mustika-Sari Zulkifli; Noor Syahida Azizan; Nur-Hidayana Mahfodz; Sazaly AbuBakar; Siti-Sarah Nor'e
EPI_ISL_4071998, EPI_ISL_4101582, EPI_ISL_4101583	PKD JELEBU	UKM Medical Molecular Biology Institute (UMBI)	Mira Farzana binti Mohamad Mokhtar; Nur Alyaa Afifah Md Shahri
EPI_ISL_4071985	PKD JOHOR BAHRU	UKM Medical Molecular Biology Institute (UMBI)	Mira Farzana binti Mohamad Mokhtar
EPI_ISL_5417628	PKD KLUANG	UKM Medical Molecular Biology Institute (UMBI)	Mira Farzana binti Mohamad Mokhtar
EPI_ISL_4101586, EPI_ISL_4101587, EPI_ISL_4101589	PKD MUAR	UKM Medical Molecular Biology Institute (UMBI)	Nur Alyaa Afifah Md Shahri
EPI_ISL_3945555, EPI_ISL_3945556	PKD TAMPIN	UKM Medical Molecular Biology Institute (UMBI)	Nur Alyaa Afifah Md Shahri
EPI_ISL_4557952, EPI_ISL_4601567, EPI_ISL_4601568, EPI_ISL_4601569	Pejabat Kesihatan Kawasan Tawau	Malaysia Genome Institute	Azrin Ahmad; Enizza Kasim; Irni Suhayu Sapian; Mohd Faizal Abu Bakar; Mohd Ghows Mohd Azzam.; Mohd Noor Mat Isa; Nor Azfa Johari; Nurhezreen Md Iqbal; Shamsidar Sopie; Siti Noraini Othman; Yusuf Muhammad Noor
EPI_ISL_3769344	Pejabat Kesihatan Pintu Masuk Antarabangsa	iPROMISE, UiTM	Ariza Adnan; Fadzilah Mohd Nor; Lim Wai Feng; Mohd Asif Mohd Sukri; Mohd Nur Fakhruzzaman Noorizhab; Mohd Zaki Salleh; Sazzli Shahlan Kassim; Siti Farah Alwani Mohd Nawi; Siti Hamimah Sheikh Abdul Kadir; Teh Lay Kek; Wang Seok Mui
EPI_ISL_3769340, EPI_ISL_3769347, EPI_ISL_3769348	Pejabat Kesihatan Pintu Masuk Antarabangsa Pulau Pinang	iPROMISE, UiTM	Ariza Adnan; Fadzilah Mohd Nor; Lim Wai Feng; Mohd Asif Mohd Sukri; Mohd Nur Fakhruzzaman Noorizhab; Mohd Zaki Salleh; Sazzli Shahlan Kassim; Siti Farah Alwani Mohd Nawi; Siti Hamimah Sheikh Abdul Kadir; Teh Lay Kek; Wang Seok Mui
EPI_ISL_4348302	Putrajaya Hospital	Institute for Medical Research, Infectious Disease Research Centre, National Institutes of Health, Ministry of Health Malaysia	Ahmad FA; Ahmad Fazilah NA; Anasir MI; Azizan MA; Kamel K; Mohd Zawawi Z; Norhisyam SN; Ramly N; Robert F; Suppiah J; Thayan R
EPI_ISL_5051949, EPI_ISL_5051950, EPI_ISL_5051953, EPI_ISL_5052165, EPI_ISL_5052166, EPI_ISL_5052167	Rejang Medical Centre (Sibu)	Institute of Health and Community Medicine	Chan Chia Jui; Chua Hock Hin; David Perera; Ooi Mong How; Tonnii Sia Loong Loong; Wong Jyn Shan
EPI_ISL_4347923, EPI_ISL_4347983, EPI_ISL_4348047	Sabah State Health Department	Institute for Medical Research, Infectious Disease Research Centre, National Institutes of Health, Ministry of Health Malaysia	Ahmad FA; Ahmad Fazilah NA; Anasir MI; Azizan MA; Kamel K; Mohd Zawawi Z; Norhisyam SN; Ramly N; Robert F; Suppiah J; Thayan R
EPI_ISL_4638327	Salak Health Clinic	Institute for Medical Research, Infectious Disease Research Centre, National Institutes of Health, Ministry of Health Malaysia	Ahmad FA; Ahmad Fazilah NA; Anasir MI; Azizan MA; Kamel K; Mohd Zawawi Z; Norhisyam SN; Ramly N; Robert F; Suppiah J; Thayan R
EPI_ISL_4460390, EPI_ISL_5051849	Sarawak Heart Centre (SHC), Kota Samarahan	Institute of Health and Community Medicine	Chan Chia Jui; Chua Hock Hin; David Perera; Ooi Mong How; Tonnii Sia Loong Loong; Wong Jyn Shan
EPI_ISL_4348303	Selayang Hospital	Institute for Medical Research, Infectious Disease Research Centre, National Institutes of Health, Ministry of Health Malaysia	Ahmad FA; Ahmad Fazilah NA; Anasir MI; Azizan MA; Kamel K; Mohd Zawawi Z; Norhisyam SN; Ramly N; Robert F; Suppiah J; Thayan R
EPI_ISL_4348277	Serdang Hospital	Institute for Medical Research, Infectious Disease Research Centre, National Institutes of Health, Ministry of Health Malaysia	Ahmad FA; Ahmad Fazilah NA; Anasir MI; Azizan MA; Kamel K; Mohd Zawawi Z; Norhisyam SN; Ramly N; Robert F; Suppiah J; Thayan R
EPI_ISL_6774016, EPI_ISL_6774028, EPI_ISL_6774045	Shell Malaysia	Institute for Medical Research, Infectious Disease Research Centre, National Institutes of Health, Ministry of Health Malaysia	Ahmad FA; Ahmad Fazilah NA; Anasir MI; Azizan MA; Kamel K; Mohamad Sukri MZ; Mohd Zawawi Z; Norhisham SN; Ramly N; Robert F; Rosli NR; Suppiah J; Thayan R
EPI_ISL_4101552	Sipitang District Health Office	Institute for Medical Research, Infectious Disease Research Centre, National Institutes of Health, Ministry of Health Malaysia	Ahmad FA; Ahmad Fazilah NA; Anasir MI; Azizan MA; Kamel K; Mohd Zawawi Z; Norhisham SN; Ramly N; Robert F; Suppiah J; Thayan R
EPI_ISL_7457546	Sultanah Fatimah Specialist Hospital	Institute for Medical Research, Infectious Disease Research Centre, National Institutes of Health, Ministry of Health Malaysia	Ahmad FA; Ahmad Fazilah NA; Anasir MI; Azizan MA; Kamel K; Mohamad Sukri MZ; Mohd Zawawi Z; Norhisham SN; Ramly N; Robert F; Rosli NR; Suppiah J; Thayan R

EPI_ISL_4348278, EPI_ISL_4348279, EPI_ISL_4348301	Sungai Buloh Hospital	Institute for Medical Research, Infectious Disease Research Centre, National Institutes of Health, Ministry of Health Malaysia	Ahmad FA; Ahmad Fazilah NA; Anasir MI; Azizan MA; Kamel K; Mohd Zawawi Z; Norhisyam SN; Ramly N; Robert F; Suppiah J; Thayan R
EPI_ISL_4346360, EPI_ISL_4348149	Tawau Area Health Office	Institute for Medical Research, Infectious Disease Research Centre, National Institutes of Health, Ministry of Health Malaysia	Ahmad FA; Ahmad Fazilah NA; Anasir MI; Azizan MA; Kamel K; Mohd Zawawi Z; Norhisyam SN; Ramly N; Robert F; Suppiah J; Thayan R
EPI_ISL_4601561, EPI_ISL_4601564	Thomson Hospital Kota Damansara	Malaysia Genome Institute	Azrin Ahmad; Enizza Kasim; Irni Suhayu Sapian; Mohd Faizal Abu Bakar; Mohd Ghows Mohd Azzam.; Mohd Noor Mat Isa; Nor Azfa Johari; Nurhezreen Md Iqbal; Shamsidar Sopie; Siti Noraini Othman; Yusuf Muhammad Noor
EPI_ISL_3858148	UKA HPSF	UKM Medical Molecular Biology Institute (UMBI)	Mira Farzana binti Mohamad Mokhtar
EPI_ISL_4236985, EPI_ISL_4236992	UKKP,HOSPITAL PERMAI	UKM Medical Molecular Biology Institute (UMBI)	Mira Farzana binti Mohamad Mokhtar
EPI_ISL_7189350, EPI_ISL_7189352, EPI_ISL_7189354, EPI_ISL_7189355, EPI_ISL_7189357, EPI_ISL_7189361, EPI_ISL_7189362, EPI_ISL_7189366			
see above	UMMC	Department of Medical Microbiology, Faculty of Medicine, University of Malaya; University of Malaya Medical Centre	I-Ching SAM; Jolene Yin Ling FU; Omar Khalilur Rahman; Yoke Fun Chan
EPI_ISL_3980712	UNIT KESELAMATAN DAN KESIHATAN PEKERJA	Tropical Infectious Diseases Research & Education Centre (TIDREC), Universiti Malaya	AsmaAnati CheMatSeri; Che-Norainon Yaacob; Jia-Yi Tan; Jo-Ern Wong; Kim-Kee Tan; Mulya-Mustika-Sari Zulkifli; Noor Syahida Azizan; Nur-Hidayana Mahfodz; Sazaly AbuBakar; Siti-Sarah Nor'e
EPI_ISL_4071988, EPI_ISL_4071996	UNIT KESIHATAN AWAM HEBHK	UKM Medical Molecular Biology Institute (UMBI)	Mira Farzana binti Mohamad Mokhtar
EPI_ISL_7087248	UNIT KESIHATAN DAN KESELAMATAN PEKERJA HOSPITAL SULTANAH BAHIYAH	Department of Medical Microbiology, Faculty of Medicine, University of Malaya; University of Malaya Medical Centre	I-Ching SAM; Jolene Yin Ling FU; Omar Khalilur Rahman; Yoke Fun Chan
EPI_ISL_3858141, EPI_ISL_3858157, EPI_ISL_3858176, EPI_ISL_3858180, EPI_ISL_3858182, EPI_ISL_4101585, EPI_ISL_5159357, EPI_ISL_5159360			
see above	UNIT KPAS, PKD MELAKA TENGAH	UKM Medical Molecular Biology Institute (UMBI)	Mira Farzana binti Mohamad Mokhtar; Nur Alyaa Afifah Md Shahri
EPI_ISL_5159358	UNIT KPAS, PKDMT	UKM Medical Molecular Biology Institute (UMBI)	Mira Farzana binti Mohamad Mokhtar
EPI_ISL_4122502	UNIT MIKROBIOLOGI, JABATAN PATOLOGI, HOSPITAL PULAU PINANG	iPROMISE, UiTM	Ariza Adnan; Fadzilah Mohd Nor; Lim Wai Feng; Mohd Asif Mohd Sukri; Mohd Nur Fakhruzzaman Noorizhab; Mohd Zaki Salleh; Sazzli Shahlan Kassim; Siti Farah Alwani Mohd Nawi; Siti Hamimah Sheikh Abdul Kadir; Teh Lay Kek; Wang Seok Mui
EPI_ISL_3980736, EPI_ISL_3980738, EPI_ISL_3980739	UNIT OSH, HOSPITAL SUNGAI BULOH	Tropical Infectious Diseases Research & Education Centre (TIDREC), Universiti Malaya	AsmaAnati CheMatSeri; Che-Norainon Yaacob; Jia-Yi Tan; Jo-Ern Wong; Kim-Kee Tan; Mulya-Mustika-Sari Zulkifli; Noor Syahida Azizan; Nur-Hidayana Mahfodz; Sazaly AbuBakar; Siti-Sarah Nor'e
EPI_ISL_3769377, EPI_ISL_3769379, EPI_ISL_3769380, EPI_ISL_3769381, EPI_ISL_3769382	UNIT OSH, HOSPITAL SUNGAI BULOH	iPROMISE, UiTM	Ariza Adnan; Fadzilah Mohd Nor; Lim Wai Feng; Mohd Asif Mohd Sukri; Mohd Nur Fakhruzzaman Noorizhab; Mohd Zaki Salleh; Sazzli Shahlan Kassim; Siti Farah Alwani Mohd Nawi; Siti Hamimah Sheikh Abdul Kadir; Teh Lay Kek; Wang Seok Mui
EPI_ISL_3980740	Uka HBTG	Tropical Infectious Diseases Research & Education Centre (TIDREC), Universiti Malaya	AsmaAnati CheMatSeri; Che-Norainon Yaacob; Jia-Yi Tan; Jo-Ern Wong; Kim-Kee Tan; Mulya-Mustika-Sari Zulkifli; Noor Syahida Azizan; Nur-Hidayana Mahfodz; Sazaly AbuBakar; Siti-Sarah Nor'e
EPI_ISL_3980742	Uka Hosp.Banting	Tropical Infectious Diseases Research & Education Centre (TIDREC), Universiti Malaya	AsmaAnati CheMatSeri; Che-Norainon Yaacob; Jia-Yi Tan; Jo-Ern Wong; Kim-Kee Tan; Mulya-Mustika-Sari Zulkifli; Noor Syahida Azizan; Nur-Hidayana Mahfodz; Sazaly AbuBakar; Siti-Sarah Nor'e
EPI_ISL_3980737, EPI_ISL_3980746	Uka Hospital Banting	Tropical Infectious Diseases Research & Education Centre (TIDREC), Universiti Malaya	AsmaAnati CheMatSeri; Che-Norainon Yaacob; Jia-Yi Tan; Jo-Ern Wong; Kim-Kee Tan; Mulya-Mustika-Sari Zulkifli; Noor Syahida Azizan; Nur-Hidayana Mahfodz; Sazaly AbuBakar; Siti-Sarah Nor'e
EPI_ISL_3980744	Uka Hospital Bnating	Tropical Infectious Diseases Research & Education Centre (TIDREC), Universiti Malaya	AsmaAnati CheMatSeri; Che-Norainon Yaacob; Jia-Yi Tan; Jo-Ern Wong; Kim-Kee Tan; Mulya-Mustika-Sari Zulkifli; Noor Syahida Azizan; Nur-Hidayana Mahfodz; Sazaly AbuBakar; Siti-Sarah Nor'e
EPI_ISL_4740394, EPI_ISL_4740398	Unit Forensik Hospital Kepala Batas	iPROMISE, UiTM	Ariza Adnan; Fadzilah Mohd Nor; Lim Wai Feng; Mohd Asif Mohd Sukri; Mohd Nur Fakhruzzaman Noorizhab; Mohd Zaki Salleh; Sazzli Shahlan Kassim; Siti Farah Alwani Mohd Nawi; Siti Hamimah Sheikh Abdul Kadir; Teh Lay Kek; Wang Seok Mui
EPI_ISL_4740406	Unit Forensik, Hospital Bukit Mertajam	iPROMISE, UiTM	Ariza Adnan; Fadzilah Mohd Nor; Lim Wai Feng; Mohd Asif Mohd Sukri; Mohd Nur Fakhruzzaman Noorizhab; Mohd Zaki Salleh; Sazzli Shahlan Kassim; Siti Farah Alwani Mohd Nawi; Siti Hamimah Sheikh Abdul Kadir; Teh Lay Kek; Wang Seok Mui
EPI_ISL_4122458, EPI_ISL_4435551	Unit Kawalan Penyakit Berjangkit, Jabatan Kesihatan Negeri Perlis	iPROMISE, UiTM	Ariza Adnan; Fadzilah Mohd Nor; Lim Wai Feng; Mohd Asif Mohd Sukri; Mohd Nur Fakhruzzaman Noorizhab; Mohd Zaki Salleh; Sazzli Shahlan Kassim; Siti Farah Alwani Mohd Nawi; Siti Hamimah Sheikh Abdul Kadir; Teh Lay Kek; Wang Seok Mui
EPI_ISL_3980719, EPI_ISL_3980721	Unit Keselamatan dan Kesihatan Pekerja	Tropical Infectious Diseases Research & Education Centre (TIDREC), Universiti Malaya	AsmaAnati CheMatSeri; Che-Norainon Yaacob; Jia-Yi Tan; Jo-Ern Wong; Kim-Kee Tan; Mulya-Mustika-Sari Zulkifli; Noor Syahida Azizan; Nur-Hidayana Mahfodz; Sazaly AbuBakar; Siti-Sarah Nor'e
EPI_ISL_5261876	iPROMISE, UiTM	iPROMISE, UiTM	Ariza Adnan; Fadzilah Mohd Nor; Lim Wai Feng; Mohd Asif Mohd Sukri; Mohd Nur Fakhruzzaman Noorizhab; Mohd Zaki Salleh; Sazzli Shahlan Kassim; Siti Farah Alwani Mohd Nawi; Siti Hamimah Sheikh Abdul Kadir; Teh Lay Kek; Wang Seok Mui
EPI_ISL_5261880	jabatan forensik	iPROMISE, UiTM	Ariza Adnan; Fadzilah Mohd Nor; Lim Wai Feng; Mohd Asif Mohd Sukri; Mohd Nur Fakhruzzaman Noorizhab; Mohd Zaki Salleh; Sazzli Shahlan Kassim; Siti Farah Alwani Mohd Nawi; Siti Hamimah Sheikh Abdul Kadir; Teh Lay Kek; Wang Seok Mui
EPI_ISL_6825217	unknown	Malaysia Genome Institute	Azrin Ahmad; Enizza Kasim; Irni Suhayu Sapian; Mohd Faizal Abu Bakar; Mohd Ghows Mohd Azzam.; Mohd Noor Mat Isa; Nor Azfa Johari; Nurhezreen Md Iqbal; Shamsidar Sopie; Siti Noraini Othman; Yusuf Muhammad Noor

We gratefully acknowledge the following Authors from the Originating laboratories responsible for obtaining the specimens, as well as the Submitting laboratories where the genome data were generated and shared via GISAID, on which this research is based.

All Submitters of data may be contacted directly via www.gisaid.org

Authors are sorted alphabetically.

Accession ID	Originating Laboratory	Submitting Laboratory	Authors
EPI_ISL_4601562, EPI_ISL_4601563, EPI_ISL_4601566, EPI_ISL_4601578	Akademi Latihan Ketenteraan	Malaysia Genome Institute	Azrin Ahmad; Enizza Kasim; Irni Suhayu Sapian; Mohd Faizal Abu Bakar; Mohd Ghows Mohd Azzam.; Mohd Noor Mat Isa; Nor Azfa Johari; Nurhezreen Md Iqbal; Shamsidar Sopie; Siti Noraini Othman; Yusuf Muhammad Noor
EPI_ISL_4460315, EPI_ISL_4460316, EPI_ISL_4460317, EPI_ISL_4460318, EPI_ISL_4460319, EPI_ISL_4460321, EPI_ISL_4460324, EPI_ISL_4460325, EPI_ISL_4460326, EPI_ISL_4460327, EPI_ISL_4460328, EPI_ISL_4460329, EPI_ISL_4460330, EPI_ISL_4460331, EPI_ISL_4460332, EPI_ISL_4460333, EPI_ISL_4460341, EPI_ISL_4460342, EPI_ISL_4460343, EPI_ISL_4460344, EPI_ISL_4460345, EPI_ISL_5051793, EPI_ISL_5051855, EPI_ISL_5051856, EPI_ISL_5051857, EPI_ISL_5051863, EPI_ISL_5051864, EPI_ISL_5896156			
see above	Bintulu Hospital PCR Lab, Bintulu	Institute of Health and Community Medicine	Chan Chia Jui; Chua Hock Hin; David Perera; Ooi Mong How; Tan Lee See; Tonnii Sia Loong Loong; Wong Jyn Shan
EPI_ISL_5051759, EPI_ISL_5051817, EPI_ISL_5051818, EPI_ISL_5051819, EPI_ISL_5051820, EPI_ISL_5051960, EPI_ISL_5051961, EPI_ISL_5051962, EPI_ISL_5051963, EPI_ISL_5893968, EPI_ISL_5893974, EPI_ISL_5893980, EPI_ISL_5894649, EPI_ISL_5894659, EPI_ISL_5896075			
see above	Bintulu Medical Centre (Bintulu)	Institute of Health and Community Medicine	Chan Chia Jui; Chua Hock Hin; David Perera; Ooi Mong How; Tonnii Sia Loong Loong; Wong Jyn Shan
EPI_ISL_4460221, EPI_ISL_4460222, EPI_ISL_4460223, EPI_ISL_4460224, EPI_ISL_4460225, EPI_ISL_4460226, EPI_ISL_4460227, EPI_ISL_4460260, EPI_ISL_4460261, EPI_ISL_4460262, EPI_ISL_4460263, EPI_ISL_4460264, EPI_ISL_4460265, EPI_ISL_4460266, EPI_ISL_4460267, EPI_ISL_4460268, EPI_ISL_4460269, EPI_ISL_4460270, EPI_ISL_4460271, EPI_ISL_4460272, EPI_ISL_4460273, EPI_ISL_4460274, EPI_ISL_4460275, EPI_ISL_4460276, EPI_ISL_4460277, EPI_ISL_4460278, EPI_ISL_4460279, EPI_ISL_4460280, EPI_ISL_4460281, EPI_ISL_4460282, EPI_ISL_4460283, EPI_ISL_4460284, EPI_ISL_4460285, EPI_ISL_4460286, EPI_ISL_4460287, EPI_ISL_4460288, EPI_ISL_4460289, EPI_ISL_4460290, EPI_ISL_4460291, EPI_ISL_4460292, EPI_ISL_4460293, EPI_ISL_4460294, EPI_ISL_4460295, EPI_ISL_4460296, EPI_ISL_4460297, EPI_ISL_4460298, EPI_ISL_4460299, EPI_ISL_4460300, EPI_ISL_4460301, EPI_ISL_4460302, EPI_ISL_4460303, EPI_ISL_4460304, EPI_ISL_4460305			
see above	Borneo Medical Centre	Institute of Health and Community Medicine	Chan Chia Jui; Chua Hock Hin; David Perera; Ooi Mong How; Tonnii Sia Loong Loong; Wong Jyn Shan
EPI_ISL_5051839, EPI_ISL_5051914, EPI_ISL_5051915, EPI_ISL_5051916, EPI_ISL_5052098	Borneo Medical Centre (Kuching)	Institute of Health and Community Medicine	Chan Chia Jui; Chua Hock Hin; David Perera; Ooi Mong How; Tonnii Sia Loong Loong; Wong Jyn Shan
EPI_ISL_5896147	Borneo Medical Centre (Miri)	Institute of Health and Community Medicine	Chan Chia Jui; Chua Hock Hin; David Perera; Ooi Mong How; Tonnii Sia Loong Loong; Wong Jyn Shan
EPI_ISL_4460351, EPI_ISL_4460352, EPI_ISL_4460353, EPI_ISL_4460354, EPI_ISL_4460355, EPI_ISL_4460356, EPI_ISL_4460357, EPI_ISL_4460358, EPI_ISL_4460359, EPI_ISL_4460360, EPI_ISL_4460361, EPI_ISL_4460362, EPI_ISL_4460363, EPI_ISL_4460364, EPI_ISL_4460365, EPI_ISL_4460366, EPI_ISL_4460367, EPI_ISL_4460368, EPI_ISL_4460369, EPI_ISL_4460370, EPI_ISL_4460371, EPI_ISL_4460372, EPI_ISL_4460373, EPI_ISL_4460374, EPI_ISL_4460375, EPI_ISL_4460376, EPI_ISL_4460377			
see above	Borneo Specialist Hospital, Miri	Institute of Health and Community Medicine	Chan Chia Jui; Chua Hock Hin; David Perera; Ooi Mong How; Tonnii Sia Loong Loong; Wong Jyn Shan
EPI_ISL_5417156	CDC JKNT	Department of Medical Microbiology, University Malaya Medical Centre	I-Ching SAM; Jolene Yin Ling FU; Omar Khalilur Rahman; Yoke Fun Chan
EPI_ISL_7086649, EPI_ISL_7086814, EPI_ISL_7087225	CPRC JKNT	Department of Medical Microbiology, Faculty of Medicine, University of Malaya; University of Malaya Medical Centre	I-Ching SAM; Jolene Yin Ling FU; Omar Khalilur Rahman; Yoke Fun Chan
EPI_ISL_5417157	CPRC JKNT	Department of Medical Microbiology, University Malaya Medical Centre	I-Ching SAM; Jolene Yin Ling FU; Omar Khalilur Rahman; Yoke Fun Chan
EPI_ISL_4564996, EPI_ISL_4564997, EPI_ISL_4564998, EPI_ISL_4564999, EPI_ISL_4565001, EPI_ISL_4565016, EPI_ISL_8215026			
see above	CPRC JKNT	Tropical Infectious Diseases Research & Education Centre (TIDREC), Universiti Malaya	AsmaAnati CheMatSeri; Che-Norainon Yaacob; Jia-Yi Tan; Jo-Ern Wong; Kim-Kee Tan; Mulya-Mustika-Sari Zulkifli; Noor Syahida Azizan; Nur-Hidayana Mahfodz; Sazaly AbuBakar; Siti-Sarah Nor’e
EPI_ISL_5051999	Clinical Research Centre (CRC), Sibu Hospital, Sibu	Institute of Health and Community Medicine	Chan Chia Jui; Chua Hock Hin; David Perera; Ooi Mong How; Tonnii Sia Loong Loong; Wong Jyn Shan
EPI_ISL_10070822, EPI_ISL_10071205, EPI_ISL_10071912, EPI_ISL_10071928, EPI_ISL_10072005, EPI_ISL_10072161, EPI_ISL_10072186, EPI_ISL_10072374			
see above	Department of Medical Microbiology, Hospital Pengajar Universiti Putra Malaysia	Department of Medical Microbiology, Hospital Pengajar Universiti Putra Malaysia	Afiqah Adzmi; Amiza Azmi; Azmiza Syawani Jasni; Hui Yee Chee; Leslie Thian Lung Than; Muadz Mohtar; Muhammad Mohd Isa; Narcisse MS Joseph; Niazlin Mohd Taib; Noor Hazirah Noor Azhari; Norlaila; Nur Raihana Ithnin; Nurul Huda Mohamed Rashidi; Nurul Nadiah Ismail; Rosni Ibrahim; Rukman Awang Hamat; Sallehhudin; Siti Norbaya Masri; Siti Zulaikha Zakariah; Syafinaz Amin Nordin; Tengku Zetty Maztura Tengku Jamaluddin; Zamberi Sekawi
EPI_ISL_5417168	Department of Medical Microbiology, University Malaya Medical Centre	Department of Medical Microbiology, University Malaya Medical Centre	I-Ching SAM; Jolene Yin Ling FU; Omar Khalilur Rahman; Yoke Fun Chan
EPI_ISL_5051995, EPI_ISL_5051996	Gribbles Pathology, Kuching	Institute of Health and Community Medicine	Chan Chia Jui; Chua Hock Hin; David Perera; Ooi Mong How; Reagan Entigu Linton; Tonnii Sia Loong Loong; Wong Jyn Shan
EPI_ISL_12628160, EPI_ISL_12628161, EPI_ISL_12628162, EPI_ISL_12628163, EPI_ISL_12628164	HCTM	UKM Medical Molecular Biology Institute (UMBI)	Khairun Nur Abd Ghafar; Mira Farzana Mohamad Mokhtar; Muhiddin Ishak; Nor Azila Muhammad Azami; Nur Alyaa Afifah Md Shahri; Nurul Syakima Ab Mutalib; Rahman Jamal; Ryia Illani Mohd Yunos; Siti Nur Hasanah Mohd Yusuf; Zahirrah Begam Mohamed Rasheed
EPI_ISL_4740382, EPI_ISL_4740383, EPI_ISL_4740386, EPI_ISL_4740387, EPI_ISL_5261883, EPI_ISL_5261884, EPI_ISL_5261885			
see above	HOSPITAL BALIK PULAU	iPROMISE, UiTM	Ariza Adnan; Fadzilah Mohd Nor; Lim Wai Feng; Mohd Asif Mohd Sukri; Mohd Nur Fakhruzzaman Noorizhab; Mohd Zaki Salleh; Sazzli Shahlan Kassim; Siti Farah Alwani Mohd Nawi; Siti Hamimah Sheikh Abdul Kadir; Teh Lay Kek; Wang Seok Mui
EPI_ISL_4122279, EPI_ISL_4122288, EPI_ISL_4122516, EPI_ISL_4122563, EPI_ISL_4435520, EPI_ISL_4435521, EPI_ISL_4435523, EPI_ISL_4435525, EPI_ISL_4435526, EPI_ISL_4435527			
see above	HOSPITAL PULAU PINANG	iPROMISE, UiTM	Ariza Adnan; Fadzilah Mohd Nor; Lim Wai Feng; Mohd Asif Mohd Sukri; Mohd Nur Fakhruzzaman Noorizhab; Mohd Zaki Salleh; Sazzli Shahlan Kassim; Siti Farah Alwani Mohd Nawi; Siti Hamimah Sheikh Abdul Kadir; Teh Lay Kek; Wang Seok Mui
EPI_ISL_4122489, EPI_ISL_4435524	HOSPITAL SG BAKAP	iPROMISE, UiTM	Ariza Adnan; Fadzilah Mohd Nor; Lim Wai Feng; Mohd Asif Mohd Sukri; Mohd Nur Fakhruzzaman Noorizhab; Mohd Zaki Salleh; Sazzli Shahlan Kassim; Siti Farah Alwani Mohd Nawi; Siti Hamimah Sheikh Abdul Kadir; Teh Lay Kek; Wang Seok Mui
EPI_ISL_4101591, EPI_ISL_4101599, EPI_ISL_4236993, EPI_ISL_4236996, EPI_ISL_4816829	HOSPITAL SULTANAH NORA ISMAIL	UKM Medical Molecular Biology Institute (UMBI)	Mira Farzana binti Mohamad Mokhtar; Nur Alyaa Afifah Md Shahri
EPI_ISL_4122292	HPP	iPROMISE, UiTM	Ariza Adnan; Fadzilah Mohd Nor; Lim Wai Feng; Mohd Asif Mohd Sukri; Mohd Nur Fakhruzzaman Noorizhab; Mohd Zaki Salleh; Sazzli Shahlan Kassim; Siti Farah Alwani Mohd Nawi; Siti Hamimah Sheikh Abdul Kadir; Teh Lay Kek; Wang Seok Mui
EPI_ISL_4101595, EPI_ISL_4101596, EPI_ISL_4236995, EPI_ISL_4236997, EPI_ISL_5417624	HPSF, MUAR	UKM Medical Molecular Biology Institute (UMBI)	Mira Farzana binti Mohamad Mokhtar; Nur Alyaa Afifah Md Shahri
EPI_ISL_8090615	HRPB	Tropical Infectious Diseases Research & Education Centre (TIDREC), Universiti Malaya	AsmaAnati CheMatSeri; Che-Norainon Yaacob; Jia-Yi Tan; Jo-Ern Wong; Kim-Kee Tan; Mulya-Mustika-Sari Zulkifli; Noor Syahida Azizan; Nur-Hidayana Mahfodz; Sazaly AbuBakar; Siti-Sarah Nor’e
EPI_ISL_4565008, EPI_ISL_4565009, EPI_ISL_4565010, EPI_ISL_4565011, EPI_ISL_4565012	HRPB, Ipoh	Tropical Infectious Diseases Research & Education Centre (TIDREC), Universiti Malaya	AsmaAnati CheMatSeri; Che-Norainon Yaacob; Jia-Yi Tan; Jo-Ern Wong; Kim-Kee Tan; Mulya-Mustika-Sari Zulkifli; Noor Syahida Azizan; Nur-Hidayana Mahfodz; Sazaly AbuBakar; Siti-Sarah Nor’e
EPI_ISL_4557932, EPI_ISL_4557933, EPI_ISL_4557934, EPI_ISL_4557943, EPI_ISL_4557944, EPI_ISL_6825220, EPI_ISL_6825221, EPI_ISL_6825222			
see above	Hospital Ampang	Malaysia Genome Institute	Azrin Ahmad; Enizza Kasim; Irni Suhayu Sapian; Mohd Faizal Abu Bakar; Mohd Ghows Mohd Azzam.; Mohd Noor Mat Isa; Nor Azfa Johari; Nurhezreen Md Iqbal; Shamsidar Sopie; Siti Noraini Othman; Yusuf Muhammad Noor
EPI_ISL_4853513, EPI_ISL_4853516, EPI_ISL_4853523, EPI_ISL_4853533, EPI_ISL_4853537, EPI_ISL_4887469, EPI_ISL_4887477			
see above	Hospital Banting	Malaysia Genome Institute	Azrin Ahmad; Enizza Kasim; Irni Suhayu Sapian; Mohd Faizal Abu Bakar; Mohd Ghows Mohd Azzam.; Mohd Noor Mat Isa; Nor Azfa Johari; Nurhezreen Md Iqbal; Shamsidar Sopie; Siti Noraini Othman; Yusuf Muhammad Noor
EPI_ISL_4557935, EPI_ISL_4601559, EPI_ISL_4853557, EPI_ISL_6825219	Hospital Kuala Lumpur	Malaysia Genome Institute	Azrin Ahmad; Enizza Kasim; Irni Suhayu Sapian; Mohd Faizal Abu Bakar; Mohd Ghows Mohd Azzam.; Mohd Noor Mat Isa; Nor Azfa Johari; Nurhezreen Md Iqbal; Shamsidar Sopie; Siti Noraini Othman; Yusuf Muhammad Noor
EPI_ISL_4853569, EPI_ISL_4853572, EPI_ISL_4853972	Hospital Lahad Datu	Malaysia Genome Institute	Azrin Ahmad; Enizza Kasim; Irni Suhayu Sapian; Mohd Faizal Abu Bakar; Mohd Ghows Mohd Azzam.; Mohd Noor Mat Isa; Nor Azfa Johari; Nurhezreen Md Iqbal; Shamsidar Sopie; Siti Noraini Othman; Yusuf Muhammad Noor
EPI_ISL_4853577, EPI_ISL_4853989	Hospital Putrajaya	Malaysia Genome Institute	Azrin Ahmad; Enizza Kasim; Irni Suhayu Sapian; Mohd Faizal Abu Bakar; Mohd Ghows Mohd Azzam.; Mohd Noor Mat Isa; Nor Azfa Johari; Nurhezreen Md Iqbal; Shamsidar Sopie; Siti Noraini Othman; Yusuf Muhammad Noor
EPI_ISL_5428524	Hospital Queen Elizabeth	Malaysia Genome Institute	Azrin Ahmad; Enizza Kasim; Irni Suhayu Sapian; Mohd Faizal Abu Bakar; Mohd Ghows Mohd Azzam.; Mohd Noor Mat Isa; Nor Azfa Johari; Nurhezreen Md Iqbal; Shamsidar Sopie; Siti Noraini Othman; Yusuf Muhammad Noor
EPI_ISL_4601580, EPI_ISL_4853624, EPI_ISL_4853651	Hospital Selayang	Malaysia Genome Institute	Azrin Ahmad; Enizza Kasim; Irni Suhayu Sapian; Mohd Faizal Abu Bakar; Mohd Ghows Mohd Azzam.; Mohd Noor Mat Isa; Nor Azfa Johari; Nurhezreen Md Iqbal; Shamsidar Sopie; Siti Noraini Othman; Yusuf Muhammad Noor
EPI_ISL_4557928, EPI_ISL_4557929, EPI_ISL_4557930, EPI_ISL_4557931, EPI_ISL_4557937, EPI_ISL_4557938, EPI_ISL_4557939, EPI_ISL_4557940, EPI_ISL_4557941, EPI_ISL_4557955, EPI_ISL_4558235, EPI_ISL_4558236, EPI_ISL_4601572, EPI_ISL_4601573, EPI_ISL_4601574, EPI_ISL_4601575, EPI_ISL_4601576, EPI_ISL_4601577, EPI_ISL_4853678, EPI_ISL_4853689, EPI_ISL_4853699, EPI_ISL_4853713, EPI_ISL_4853721, EPI_ISL_4853740, EPI_ISL_4853751, EPI_ISL_4853774, EPI_ISL_6825218			
see above	Hospital Sungai Buloh	Malaysia Genome Institute	Azrin Ahmad; Enizza Kasim; Irni Suhayu Sapian; Mohd Faizal Abu Bakar; Mohd Ghows Mohd Azzam.; Mohd Noor Mat Isa; Nor Azfa Johari; Nurhezreen Md Iqbal; Shamsidar Sopie; Siti Noraini Othman; Yusuf Muhammad Noor
EPI_ISL_5159362	Hospital tuanku jaafar seremban	UKM Medical Molecular Biology Institute (UMBI)	Mira Farzana binti Mohamad Mokhtar
EPI_ISL_6960061, EPI_ISL_6960062, EPI_ISL_6960063, EPI_ISL_6960064, EPI_ISL_6960065, EPI_ISL_6960066, EPI_ISL_6960067, EPI_ISL_11078684, EPI_ISL_11078690, EPI_ISL_11078698, EPI_ISL_11078718, EPI_ISL_11078724, EPI_ISL_11078736, EPI_ISL_11078739, EPI_ISL_11078740, EPI_ISL_11078744, EPI_ISL_11078749, EPI_ISL_11078750, EPI_ISL_11078761, EPI_ISL_11078764, EPI_ISL_11078769, EPI_ISL_11078782			
see above	Institut Biologi Molekul Perubatan UKM (UMBI)	UKM Medical Molecular Biology Institute (UMBI)	Khairun Nur Abd Ghafar; Mira Farzana Mohamad Mokhtar; Mira Farzana binti Mohamad Mokhtar; Muhiddin Ishak; Nor Azila Muhammad Azami; Nur Alyaa Afifah Md Shahri; Nurul Syakima Ab Mutalib; Rahman Jamal; Ryia Illani Mohd Yunos; Siti Nur Hasanah Mohd Yusuf; Zahirrah Begam Mohamed Rasheed
EPI_ISL_4740385, EPI_ISL_4740389, EPI_ISL_4740404	JABATAN FORENSIK HOSPITAL PULAU PINANG	iPROMISE, UiTM	Ariza Adnan; Fadzilah Mohd Nor; Lim Wai Feng; Mohd Asif Mohd Sukri; Mohd Nur Fakhruzzaman Noorizhab; Mohd Zaki Salleh; Sazzli Shahlan Kassim; Siti Farah Alwani Mohd Nawi; Siti Hamimah Sheikh Abdul Kadir; Teh Lay Kek; Wang Seok Mui
EPI_ISL_6132037, EPI_ISL_8254286	JABATAN PERUBATAN FORENSIK HOSPITAL SULTANAH BAHIYAH	Department of Medical Microbiology, Faculty of Medicine, University of Malaya; University of Malaya Medical Centre	I-Ching SAM; Jolene Yin Ling FU; Omar Khalilur Rahman; Yoke Fun Chan

EPI_ISL_4122483	JKN Komtar	iPROMISE, UiTM	Ariza Adnan; Fadzilah Mohd Nor; Lim Wai Feng; Mohd Asif Mohd Sukri; Mohd Nur Fakhruzzaman Noorizhab; Mohd Zaki Salleh; Sazzli Shahlan Kassim; Siti Farah Alwani Mohd Nawi; Siti Hamimah Sheikh Abdul Kadir; Teh Lay Kek; Wang Seok Mui
EPI_ISL_4435530, EPI_ISL_4435532, EPI_ISL_4435538, EPI_ISL_4435540, EPI_ISL_4435541, EPI_ISL_4435545, EPI_ISL_4435546, EPI_ISL_5450506			
see above	JKN PAHANG	iPROMISE, UiTM	Ariza Adnan; Fadzilah Mohd Nor; Lim Wai Feng; Mohd Asif Mohd Sukri; Mohd Nur Fakhruzzaman Noorizhab; Mohd Zaki Salleh; Sazzli Shahlan Kassim; Siti Farah Alwani Mohd Nawi; Siti Hamimah Sheikh Abdul Kadir; Teh Lay Kek; Wang Seok Mui
EPI_ISL_6132021, EPI_ISL_6132028, EPI_ISL_6132036	Jabatan Forensik Hospital Sultanah Bahiyah	Department of Medical Microbiology, Faculty of Medicine, University of Malaya; University of Malaya Medical Centre	I-Ching SAM; Jolene Yin Ling FU; Omar Khalilur Rahman; Yoke Fun Chan
EPI_ISL_4853926	Jabatan Kesihatan Negeri WP Labuan	Malaysia Genome Institute	Azrin Ahmad; Enizza Kasim; Irni Suhayu Sapian; Mohd Faizal Abu Bakar; Mohd Ghows Mohd Azzam.; Mohd Noor Mat Isa; Nor Azfa Johari; Nurhezreen Md Iqbal; Shamsidar Sopie; Siti Noraini Othman; Yusuf Muhammad Noor
EPI_ISL_4816831	KESIHATAN AWAM	UKM Medical Molecular Biology Institute (UMBI)	Mira Farzana binti Mohamad Mokhtar
EPI_ISL_4435512	KLINIK KESIHATAN TRIANG	iPROMISE, UiTM	Ariza Adnan; Fadzilah Mohd Nor; Lim Wai Feng; Mohd Asif Mohd Sukri; Mohd Nur Fakhruzzaman Noorizhab; Mohd Zaki Salleh; Sazzli Shahlan Kassim; Siti Farah Alwani Mohd Nawi; Siti Hamimah Sheikh Abdul Kadir; Teh Lay Kek; Wang Seok Mui
EPI_ISL_7087797	KLINIK WELLNESS	Department of Medical Microbiology, Faculty of Medicine, University of Malaya; University of Malaya Medical Centre	I-Ching SAM; Jolene Yin Ling FU; Omar Khalilur Rahman; Yoke Fun Chan
EPI_ISL_4730474	Kuala Lumpur International Airport (KLIA) Health Office	Institute for Medical Research, Infectious Disease Research Centre, National Institutes of Health, Ministry of Health Malaysia	Ahmad FA; Ahmad Fazilah NA; Anasir MI; Azizan MA; Kamel K; Mohd Zawawi Z; Norhisham SN; Ramly N; Robert F; Suppiah J; Thayan R
EPI_ISL_4460230	Ministry of Health Hospitals	Institute of Health and Community Medicine	Chan Chia Jui; Chua Hock Hin; David Perera; Ooi Mong How; Tonnii Sia Loong Loong; Wong Jyn Shan; Wong Kiing Aik
EPI_ISL_4460383, EPI_ISL_4460384, EPI_ISL_4460385, EPI_ISL_4460386, EPI_ISL_4460387, EPI_ISL_5052100	Miri Hospital Molecular Diagnostic Lab, Miri	Institute of Health and Community Medicine	Amir Safuan bin Khamshah; Chan Chia Jui; Chua Hock Hin; David Perera; Hanis Syazwani Mohd Hassan; Ooi Mong How; Tonnii Sia Loong Loong; Wong Jyn Shan
EPI_ISL_4071986, EPI_ISL_4071987, EPI_ISL_4816826	PEJABAT KESIHATAN DAERAH BATU PAHAT	UKM Medical Molecular Biology Institute (UMBI)	Mira Farzana binti Mohamad Mokhtar
EPI_ISL_4101592	PEJABAT KESIHATAN DAERAH JELEBU	UKM Medical Molecular Biology Institute (UMBI)	Nur Alyaa Afifah Md Shahri
EPI_ISL_4740381	PEJABAT KESIHATAN DAERAH SEBERANG PERAI SELATAN	iPROMISE, UiTM	Ariza Adnan; Fadzilah Mohd Nor; Lim Wai Feng; Mohd Asif Mohd Sukri; Mohd Nur Fakhruzzaman Noorizhab; Mohd Zaki Salleh; Sazzli Shahlan Kassim; Siti Farah Alwani Mohd Nawi; Siti Hamimah Sheikh Abdul Kadir; Teh Lay Kek; Wang Seok Mui
EPI_ISL_4565006	PEJABAT KESIHATAN DAERAH TUMPAT	Tropical Infectious Diseases Research & Education Centre (TIDREC), Universiti Malaya	AsmaAnati CheMatSeri; Che-Norainon Yaacob; Jia-Yi Tan; Jo-Ern Wong; Kim-Kee Tan; Mulya-Mustika-Sari Zulkifli; Noor Syahida Azizan; Nur-Hidayana Mahfodz; Sazaly AbuBakar; Siti-Sarah Nor'e
EPI_ISL_4236998	PKD ALOR GAJAH	UKM Medical Molecular Biology Institute (UMBI)	Mira Farzana binti Mohamad Mokhtar
EPI_ISL_4731224, EPI_ISL_5365875	PKD JOHOR BAHRU	UKM Medical Molecular Biology Institute (UMBI)	Mira Farzana binti Mohamad Mokhtar
EPI_ISL_4101593	PKD Jempol	UKM Medical Molecular Biology Institute (UMBI)	Nur Alyaa Afifah Md Shahri
EPI_ISL_4513506, EPI_ISL_4731194, EPI_ISL_4731204, EPI_ISL_5417625	PKD KLUANG	UKM Medical Molecular Biology Institute (UMBI)	Mira Farzana binti Mohamad Mokhtar
EPI_ISL_4513488, EPI_ISL_4513489, EPI_ISL_4513490, EPI_ISL_4513491, EPI_ISL_4513492, EPI_ISL_4513493	PKD KUALA PILAH	UKM Medical Molecular Biology Institute (UMBI)	Mira Farzana binti Mohamad Mokhtar
EPI_ISL_4236987	PKD KULAI	UKM Medical Molecular Biology Institute (UMBI)	Mira Farzana binti Mohamad Mokhtar
EPI_ISL_4740384, EPI_ISL_4740390, EPI_ISL_5261873, EPI_ISL_5261874, EPI_ISL_5261875	PKD SPT	iPROMISE, UiTM	Ariza Adnan; Fadzilah Mohd Nor; Lim Wai Feng; Mohd Asif Mohd Sukri; Mohd Nur Fakhruzzaman Noorizhab; Mohd Zaki Salleh; Sazzli Shahlan Kassim; Siti Farah Alwani Mohd Nawi; Siti Hamimah Sheikh Abdul Kadir; Teh Lay Kek; Wang Seok Mui
EPI_ISL_4101597, EPI_ISL_4101598, EPI_ISL_4513494, EPI_ISL_4513495	PKD TAMPIN	UKM Medical Molecular Biology Institute (UMBI)	Mira Farzana binti Mohamad Mokhtar; Nur Alyaa Afifah Md Shahri
EPI_ISL_8745518, EPI_ISL_8745519, EPI_ISL_8745520, EPI_ISL_8745521, EPI_ISL_8745522, EPI_ISL_8745523, EPI_ISL_8745524, EPI_ISL_8745525, EPI_ISL_8745526, EPI_ISL_8745527, EPI_ISL_8745528, EPI_ISL_8745529, EPI_ISL_8745530, EPI_ISL_8745531, EPI_ISL_8745532, EPI_ISL_8745533, EPI_ISL_8745534, EPI_ISL_8745535, EPI_ISL_8745536, EPI_ISL_8745537, EPI_ISL_8745538, EPI_ISL_8745539, EPI_ISL_8745540, EPI_ISL_8745541, EPI_ISL_8745542, EPI_ISL_8745543, EPI_ISL_8745544, EPI_ISL_8745545, EPI_ISL_8745546, EPI_ISL_8745547, EPI_ISL_8745548, EPI_ISL_8745549, EPI_ISL_8745550, EPI_ISL_8745551, EPI_ISL_8745552, EPI_ISL_8745553, EPI_ISL_8745554, EPI_ISL_8745555, EPI_ISL_8745556, EPI_ISL_8745557, EPI_ISL_8745558, EPI_ISL_8745559, EPI_ISL_8745560, EPI_ISL_8745561, EPI_ISL_8745562, EPI_ISL_8745563, EPI_ISL_8745564, EPI_ISL_8745565, EPI_ISL_8745566, EPI_ISL_8745567, EPI_ISL_8745605, EPI_ISL_8745606, EPI_ISL_8745607, EPI_ISL_8745608, EPI_ISL_8745609, EPI_ISL_8745610, EPI_ISL_8745611, EPI_ISL_8745612, EPI_ISL_8745613			
see above	Pejabat Kesihatan Daerah Sepang	Malaysia Genome and Vaccine Institute	Apical Scientific Sdn Bhd's Team; Azrin Ahmad; Carey Wee Kai Li; Enizza Kasim; Irni Suhayu Sapian; Jilian Michelle Wong Tzeling; Leong Wai Mun; Mohd Faizal Abu Bakar; Mohd Ghows Mohd Azzam.; Mohd Noor Mat Isa; Nor Azfa Johari; Nurhezreen Md Iqbal; Rose Iszati Ismet Nayan; Shamsidar Sopie; Siti Noraini Othman; Yusuf Muhammad Noor
EPI_ISL_4853939, EPI_ISL_4853951, EPI_ISL_4853957, EPI_ISL_4887483	Pejabat Kesihatan Kawasan Tawau	Malaysia Genome Institute	Azrin Ahmad; Enizza Kasim; Irni Suhayu Sapian; Mohd Faizal Abu Bakar; Mohd Ghows Mohd Azzam.; Mohd Noor Mat Isa; Nor Azfa Johari; Nurhezreen Md Iqbal; Shamsidar Sopie; Siti Noraini Othman; Yusuf Muhammad Noor
EPI_ISL_4122437, EPI_ISL_4122547	Pejabat Kesihatan Pintu Masuk Antarabangsa	iPROMISE, UiTM	Ariza Adnan; Fadzilah Mohd Nor; Lim Wai Feng; Mohd Asif Mohd Sukri; Mohd Nur Fakhruzzaman Noorizhab; Mohd Zaki Salleh; Sazzli Shahlan Kassim; Siti Farah Alwani Mohd Nawi; Siti Hamimah Sheikh Abdul Kadir; Teh Lay Kek; Wang Seok Mui
EPI_ISL_4122447	Pejabat Kesihatan Pintu Masuk Antarabangsa Pulau Pinang	iPROMISE, UiTM	Ariza Adnan; Fadzilah Mohd Nor; Lim Wai Feng; Mohd Asif Mohd Sukri; Mohd Nur Fakhruzzaman Noorizhab; Mohd Zaki Salleh; Sazzli Shahlan Kassim; Siti Farah Alwani Mohd Nawi; Siti Hamimah Sheikh Abdul Kadir; Teh Lay Kek; Wang Seok Mui
EPI_ISL_6825224	Pusat Kesihatan Kawasan Tawau	Malaysia Genome Institute	Azrin Ahmad; Enizza Kasim; Irni Suhayu Sapian; Mohd Faizal Abu Bakar; Mohd Ghows Mohd Azzam.; Mohd Noor Mat Isa; Nor Azfa Johari; Nurhezreen Md Iqbal; Shamsidar Sopie; Siti Noraini Othman; Yusuf Muhammad Noor
EPI_ISL_4638334	Putrajaya Health Office	Institute for Medical Research, Infectious Disease Research Centre, National Institutes of Health, Ministry of Health Malaysia	Ahmad FA; Ahmad Fazilah NA; Anasir MI; Azizan MA; Kamel K; Mohd Zawawi Z; Norhisyam SN; Ramly N; Robert F; Suppiah J; Thayan R
EPI_ISL_5051803, EPI_ISL_5051926, EPI_ISL_5051927, EPI_ISL_5051928, EPI_ISL_5051929, EPI_ISL_5051930, EPI_ISL_5051931, EPI_ISL_5051932, EPI_ISL_5051954, EPI_ISL_5052134, EPI_ISL_5052168, EPI_ISL_5052169, EPI_ISL_5052170, EPI_ISL_5052171			
see above	Rejang Medical Centre (Sibu)	Institute of Health and Community Medicine	Chan Chia Jui; Chua Hock Hin; David Perera; Ooi Mong How; Tonnii Sia Loong Loong; Wong Jyn Shan
EPI_ISL_4236988, EPI_ISL_5417623	SWAT TEAM, HPSF	UKM Medical Molecular Biology Institute (UMBI)	Mira Farzana binti Mohamad Mokhtar
EPI_ISL_5051993, EPI_ISL_5051994, EPI_ISL_5052156, EPI_ISL_5052177, EPI_ISL_6968292	Sarawak General Hospital (Kuching)	Institute of Health and Community Medicine	Chan Chia Jui; Chua Hock Hin; David Perera; Ooi Mong How; Tonnii Sia Loong Loong; Wong Jyn Shan
EPI_ISL_4460388, EPI_ISL_4460389, EPI_ISL_4460391, EPI_ISL_4460392, EPI_ISL_5051848, EPI_ISL_5895040	Sarawak Heart Centre (SHC), Kota Samarahan	Institute of Health and Community Medicine	Chan Chia Jui; Chua Hock Hin; David Perera; Ooi Mong How; Tonnii Sia Loong Loong; Wong Jyn Shan
EPI_ISL_4730477	Sepang District Health Office	Institute for Medical Research, Infectious Disease Research Centre, National Institutes of Health, Ministry of Health Malaysia	Ahmad FA; Ahmad Fazilah NA; Anasir MI; Azizan MA; Kamel K; Mohd Zawawi Z; Norhisham SN; Ramly N; Robert F; Suppiah J; Thayan R
EPI_ISL_4101567, EPI_ISL_4348304	Serdang Hospital	Institute for Medical Research, Infectious Disease Research Centre, National Institutes of Health, Ministry of Health Malaysia	Ahmad FA; Ahmad Fazilah NA; Anasir MI; Azizan MA; Kamel K; Mohd Zawawi Z; Norhisham SN; Norhisyam SN; Ramly N; Robert F; Suppiah J; Thayan R
EPI_ISL_4236990	UKA HPSF MUAR	UKM Medical Molecular Biology Institute (UMBI)	Mira Farzana binti Mohamad Mokhtar
EPI_ISL_7189349, EPI_ISL_7189351, EPI_ISL_7189356, EPI_ISL_7189360, EPI_ISL_7189367, EPI_ISL_7189368	UMMC	Department of Medical Microbiology, Faculty of Medicine, University of Malaya; University of Malaya Medical Centre	I-Ching SAM; Jolene Yin Ling FU; Omar Khalilur Rahman; Yoke Fun Chan
EPI_ISL_5261882	UNIT FORENSIK HOSPITAL SEBERANG JAYA	iPROMISE, UiTM	Ariza Adnan; Fadzilah Mohd Nor; Lim Wai Feng; Mohd Asif Mohd Sukri; Mohd Nur Fakhruzzaman Noorizhab; Mohd Zaki Salleh; Sazzli Shahlan Kassim; Siti Farah Alwani Mohd Nawi; Siti Hamimah Sheikh Abdul Kadir; Teh Lay Kek; Wang Seok Mui
EPI_ISL_4435522	UNIT FORENSIK SUNGAI BAKAP	iPROMISE, UiTM	Ariza Adnan; Fadzilah Mohd Nor; Lim Wai Feng; Mohd Asif Mohd Sukri; Mohd Nur Fakhruzzaman Noorizhab; Mohd Zaki Salleh; Sazzli Shahlan Kassim; Siti Farah Alwani Mohd Nawi; Siti Hamimah Sheikh Abdul Kadir; Teh Lay Kek; Wang Seok Mui
EPI_ISL_4236986	UNIT KESIHATAN AWAM	UKM Medical Molecular Biology Institute (UMBI)	Mira Farzana binti Mohamad Mokhtar
EPI_ISL_4101594, EPI_ISL_4236989, EPI_ISL_4236991, EPI_ISL_4236994, EPI_ISL_4513505, EPI_ISL_4731199, EPI_ISL_4816828, EPI_ISL_4816830, EPI_ISL_5417627, EPI_ISL_5417630			
see above	UNIT KESIHATAN AWAM HEBHK	UKM Medical Molecular Biology Institute (UMBI)	Mira Farzana binti Mohamad Mokhtar; Nur Alyaa Afifah Md Shahri
EPI_ISL_4513504	UNIT KESIHATAN AWAM UKA	UKM Medical Molecular Biology Institute (UMBI)	Mira Farzana binti Mohamad Mokhtar
EPI_ISL_5159361	UNIT KPAS, PKD MELAKA TENGAH	UKM Medical Molecular Biology Institute (UMBI)	Mira Farzana binti Mohamad Mokhtar
EPI_ISL_4740405	Unit Forensik Hospital Kepala Batas	iPROMISE, UiTM	Ariza Adnan; Fadzilah Mohd Nor; Lim Wai Feng; Mohd Asif Mohd Sukri; Mohd Nur Fakhruzzaman Noorizhab; Mohd Zaki Salleh; Sazzli Shahlan Kassim; Siti Farah Alwani Mohd Nawi; Siti Hamimah Sheikh Abdul Kadir; Teh Lay Kek; Wang Seok Mui
EPI_ISL_4740388	Unit forensik Hospital Kepala Batas	iPROMISE, UiTM	Ariza Adnan; Fadzilah Mohd Nor; Lim Wai Feng; Mohd Asif Mohd Sukri; Mohd Nur Fakhruzzaman Noorizhab; Mohd Zaki Salleh; Sazzli Shahlan Kassim; Siti Farah Alwani Mohd Nawi; Siti Hamimah Sheikh Abdul Kadir; Teh Lay Kek; Wang Seok Mui

We gratefully acknowledge the following Authors from the Originating laboratories responsible for obtaining the specimens, as well as the Submitting laboratories where the genome data were generated and shared via GISAID, on which this research is based.

All Submitters of data may be contacted directly via www.gisaid.org

Authors are sorted alphabetically.

Accession ID	Originating Laboratory	Submitting Laboratory	Authors
EPI_ISL_5051745, EPI_ISL_5051746, EPI_ISL_5051761, EPI_ISL_5051762, EPI_ISL_5051770, EPI_ISL_5051771, EPI_ISL_5051772, EPI_ISL_5051773, EPI_ISL_5051776, EPI_ISL_5051778, EPI_ISL_5051785, EPI_ISL_5051786, EPI_ISL_5051787, EPI_ISL_5051788, EPI_ISL_5051789, EPI_ISL_5051790, EPI_ISL_5051791, EPI_ISL_5051792, EPI_ISL_5051794, EPI_ISL_5051795, EPI_ISL_5051796, EPI_ISL_5051799, EPI_ISL_5051800, EPI_ISL_5051801, EPI_ISL_5051836, EPI_ISL_5051837, EPI_ISL_5051838, EPI_ISL_5051851, EPI_ISL_5051852, EPI_ISL_5051853, EPI_ISL_5051854, EPI_ISL_5051858, EPI_ISL_5051859, EPI_ISL_5051860, EPI_ISL_5051861, EPI_ISL_5051862, EPI_ISL_5051865, EPI_ISL_5051866, EPI_ISL_5051867, EPI_ISL_5051868, EPI_ISL_5051869, EPI_ISL_5051870, EPI_ISL_5051871, EPI_ISL_5051897, EPI_ISL_5051898, EPI_ISL_5051899, EPI_ISL_5051900, EPI_ISL_5051901, EPI_ISL_5051902, EPI_ISL_5051903, EPI_ISL_5051904, EPI_ISL_5051905, EPI_ISL_5051906, EPI_ISL_5051909, EPI_ISL_5051910, EPI_ISL_5051911, EPI_ISL_5051912, EPI_ISL_5052159, EPI_ISL_5052160, EPI_ISL_5894128, EPI_ISL_5894138			
see above	Bintulu Hospital PCR Lab, Bintulu	Institute of Health and Community Medicine	Chan Chia Jui; Chua Hock Hin; David Perera; Ooi Mong How; Tan Lee See; Tonnii Sia Loong Loong; Wong Jyn Shan
EPI_ISL_5893987, EPI_ISL_5894111, EPI_ISL_5894670, EPI_ISL_5894681, EPI_ISL_5894689, EPI_ISL_5894694, EPI_ISL_5894700, EPI_ISL_5894709, EPI_ISL_5894717, EPI_ISL_5896081, EPI_ISL_5896092			
see above	Bintulu Medical Centre (Bintulu)	Institute of Health and Community Medicine	Chan Chia Jui; Chua Hock Hin; David Perera; Ooi Mong How; Tonnii Sia Loong Loong; Wong Jyn Shan
EPI_ISL_5051754, EPI_ISL_5051767, EPI_ISL_5051917, EPI_ISL_5051918, EPI_ISL_5051919, EPI_ISL_5051920, EPI_ISL_5051921, EPI_ISL_5051922, EPI_ISL_5051923, EPI_ISL_5051924, EPI_ISL_5051925, EPI_ISL_5051974, EPI_ISL_5051975, EPI_ISL_5051976, EPI_ISL_5051977, EPI_ISL_5051978, EPI_ISL_5051979, EPI_ISL_5052114, EPI_ISL_5052115, EPI_ISL_5052116, EPI_ISL_5052117, EPI_ISL_5052118, EPI_ISL_5052119, EPI_ISL_5052154, EPI_ISL_5052155, EPI_ISL_5052173, EPI_ISL_5052174, EPI_ISL_5052175, EPI_ISL_8188734, EPI_ISL_8188735			
see above	Borneo Medical Centre (Kuching)	Institute of Health and Community Medicine	Chan Chia Jui; Chua Hock Hin; David Perera; Ooi Mong How; Tonnii Sia Loong Loong; Wong Jyn Shan
EPI_ISL_4460378, EPI_ISL_4460379, EPI_ISL_4460380, EPI_ISL_4460381, EPI_ISL_4460382	Borneo Specialist Hospital, Miri	Institute of Health and Community Medicine	Chan Chia Jui; Chua Hock Hin; David Perera; Ooi Mong How; Tonnii Sia Loong Loong; Wong Jyn Shan
EPI_ISL_4740400, EPI_ISL_5261877	CAC	iPROMISE, UiTM	Ariza Adnan; Fadzilah Mohd Nor; Lim Wai Feng; Mohd Asif Mohd Sukri; Mohd Nur Fakhruzzaman Noorizhab; Mohd Zaki Salleh; Sazzli Shahlan Kassim; Siti Farah Alwani Mohd Nawi; Siti Hamimah Sheikh Abdul Kadir; Teh Lay Kek; Wang Seok Mui
EPI_ISL_6132018	CAC ILKKM	Department of Medical Microbiology, Faculty of Medicine, University of Malaya; University of Malaya Medical Centre	I-Ching SAM; Jolene Yin Ling FU; Omar Khalilur Rahman; Yoke Fun Chan
EPI_ISL_4122269	CAC ILKKM	iPROMISE, UiTM	Ariza Adnan; Fadzilah Mohd Nor; Lim Wai Feng; Mohd Asif Mohd Sukri; Mohd Nur Fakhruzzaman Noorizhab; Mohd Zaki Salleh; Sazzli Shahlan Kassim; Siti Farah Alwani Mohd Nawi; Siti Hamimah Sheikh Abdul Kadir; Teh Lay Kek; Wang Seok Mui
EPI_ISL_4122272	CAC ILKKM Kangar Perlis	iPROMISE, UiTM	Ariza Adnan; Fadzilah Mohd Nor; Lim Wai Feng; Mohd Asif Mohd Sukri; Mohd Nur Fakhruzzaman Noorizhab; Mohd Zaki Salleh; Sazzli Shahlan Kassim; Siti Farah Alwani Mohd Nawi; Siti Hamimah Sheikh Abdul Kadir; Teh Lay Kek; Wang Seok Mui
EPI_ISL_6132025, EPI_ISL_6132039	CPRC JKNT	Department of Medical Microbiology, Faculty of Medicine, University of Malaya; University of Malaya Medical Centre	I-Ching SAM; Jolene Yin Ling FU; Omar Khalilur Rahman; Yoke Fun Chan
EPI_ISL_5417139, EPI_ISL_5417153, EPI_ISL_5417155, EPI_ISL_5417160, EPI_ISL_5425837	CPRC JKNT	Department of Medical Microbiology, University Malaya Medical Centre	I-Ching SAM; Jolene Yin Ling FU; Omar Khalilur Rahman; Yoke Fun Chan
EPI_ISL_4565013, EPI_ISL_4565014, EPI_ISL_4565015, EPI_ISL_4565017, EPI_ISL_8090600, EPI_ISL_8090602, EPI_ISL_8090651			
see above	CPRC JKNT	Tropical Infectious Diseases Research & Education Centre (TIDREC), Universiti Malaya	AsmaAnati CheMatSeri; Che-Norainon Yaacob; Jia-Yi Tan; Jo-Ern Wong; Kim-Kee Tan; Mulya-Mustika-Sari Zulkifli; Noor Syahida Azizan; Nur-Hidayana Mahfodz; Sazaly AbuBakar; Siti-Sarah Nor'e
EPI_ISL_6586433, EPI_ISL_6586434, EPI_ISL_6586435, EPI_ISL_6586436, EPI_ISL_6586437, EPI_ISL_6586438, EPI_ISL_6586439, EPI_ISL_6586440, EPI_ISL_6586441, EPI_ISL_6586442, EPI_ISL_6586443, EPI_ISL_6586444, EPI_ISL_6586454, EPI_ISL_6586455, EPI_ISL_6586458, EPI_ISL_6586459, EPI_ISL_6586461, EPI_ISL_6586463, EPI_ISL_6586469, EPI_ISL_6586470, EPI_ISL_6586474, EPI_ISL_6586475			
see above	Clinical Research Centre (CRC), Sibu Hospital, Sibu	Institute of Health and Community Medicine	Chan Chia Jui; Chua Hock Hin; David Perera; Ooi Mong How; Tonnii Sia Loong Loong; Wong Jyn Shan
EPI_ISL_5782329, EPI_ISL_5782332	Department of Medical Microbiology, Hospital Pengajar Universiti Putra Malaysia	Department of Medical Microbiology, Hospital Pengajar Universiti Putra Malaysia	Afiqah Adzmi; Amiza Azmi; Azmiza Syawani Jasni; Chee Hui Yee; Leslie Than Thian Lung; Muadz Mohtar; Muhammad Mohd Isa; Narcisse MS Joseph; Niazlin Mohd Taib; Noor Hazirah Noor Azhari; Norlaila; Nur Raihana Ithnin; Nurul Huda Mohamed Rashidi; Nurul Nadiah Ismail; Rosni Ibrahim; Sallehhudin; Siti Norbaya Masri; Siti Zulaikha Zakariah; Suppiah J; Syafinaz Amin Nordin; Tengku Zetty Maztura Tengku Jamaluddin; Thayan R; Zamberi Sekawi
EPI_ISL_5051891, EPI_ISL_5051987	Gribbles Pathology, Kuching	Institute of Health and Community Medicine	Chan Chia Jui; Chua Hock Hin; David Perera; Ooi Mong How; Reagan Entigu Linton; Tonnii Sia Loong Loong; Wong Jyn Shan
EPI_ISL_12628165, EPI_ISL_12628166, EPI_ISL_12628167, EPI_ISL_12628168	HCTM	UKM Medical Molecular Biology Institute (UMBI)	Khairun Nur Abd Ghafar; Mira Farzana Mohamad Mokhtar; Muhiddin Ishak; Nor Azila Muhammad Azami; Nur Alyaa Afifah Md Shahri; Nurul Syakima Ab Mutalib; Rahman Jamal; Ryia Illani Mohd Yunos; Siti Nur Hasanah Mohd Yusuf; Zahirrah Begam Mohamed Rasheed
EPI_ISL_4740372, EPI_ISL_4740375, EPI_ISL_4740378, EPI_ISL_4740402	HOSPITAL BALIK PULAU	iPROMISE, UiTM	Ariza Adnan; Fadzilah Mohd Nor; Lim Wai Feng; Mohd Asif Mohd Sukri; Mohd Nur Fakhruzzaman Noorizhab; Mohd Zaki Salleh; Sazzli Shahlan Kassim; Siti Farah Alwani Mohd Nawi; Siti Hamimah Sheikh Abdul Kadir; Teh Lay Kek; Wang Seok Mui
EPI_ISL_5365873, EPI_ISL_5429420, EPI_ISL_5429425	HOSPITAL SEGAMAT	UKM Medical Molecular Biology Institute (UMBI)	Mira Farzana binti Mohamad Mokhtar
EPI_ISL_4513507, EPI_ISL_4513508, EPI_ISL_4731202, EPI_ISL_4731203	HOSPITAL SULTANAH NORA ISMAIL	UKM Medical Molecular Biology Institute (UMBI)	Mira Farzana binti Mohamad Mokhtar
EPI_ISL_8090613, EPI_ISL_8090617, EPI_ISL_8090618, EPI_ISL_8090619, EPI_ISL_8090620, EPI_ISL_8090621, EPI_ISL_8090622, EPI_ISL_8090623, EPI_ISL_8090624, EPI_ISL_8090625, EPI_ISL_8090626, EPI_ISL_8090654, EPI_ISL_8090656, EPI_ISL_8090685			
see above	HRPB	Tropical Infectious Diseases Research & Education Centre (TIDREC), Universiti Malaya	AsmaAnati CheMatSeri; Che-Norainon Yaacob; Jia-Yi Tan; Jo-Ern Wong; Kim-Kee Tan; Mulya-Mustika-Sari Zulkifli; Noor Syahida Azizan; Nur-Hidayana Mahfodz; Sazaly AbuBakar; Siti-Sarah Nor'e
EPI_ISL_8090614	HRPB, Ipoh	Tropical Infectious Diseases Research & Education Centre (TIDREC), Universiti Malaya	AsmaAnati CheMatSeri; Che-Norainon Yaacob; Jia-Yi Tan; Jo-Ern Wong; Kim-Kee Tan; Mulya-Mustika-Sari Zulkifli; Noor Syahida Azizan; Nur-Hidayana Mahfodz; Sazaly AbuBakar; Siti-Sarah Nor'e
EPI_ISL_4853491, EPI_ISL_4853498, EPI_ISL_4853504, EPI_ISL_5428522, EPI_ISL_5428523, EPI_ISL_5536435, EPI_ISL_5536437, EPI_ISL_5536438, EPI_ISL_5536439			
see above	Hospital Ampang	Malaysia Genome Institute	Azrin Ahmad; Enizza Kasim; Irni Suhayu Sapian; Mohd Faizal Abu Bakar; Mohd Ghows Mohd Azzam.; Mohd Noor Mat Isa; Nor Azfa Johari; Nurhezreen Md Iqbal; Shamsidar Sopie; Siti Noraini Othman; Yusuf Muhammad Noor
EPI_ISL_4853544, EPI_ISL_5536411, EPI_ISL_5536433, EPI_ISL_5536434, EPI_ISL_5536440, EPI_ISL_5536453, EPI_ISL_5536454			
see above	Hospital Banting	Malaysia Genome Institute	Azrin Ahmad; Enizza Kasim; Irni Suhayu Sapian; Mohd Faizal Abu Bakar; Mohd Ghows Mohd Azzam.; Mohd Noor Mat Isa; Nor Azfa Johari; Nurhezreen Md Iqbal; Shamsidar Sopie; Siti Noraini Othman; Yusuf Muhammad Noor
EPI_ISL_6017854	Hospital Bukit Mertajam	iPROMISE, UiTM	Ariza Adnan; Fadzilah Mohd Nor; Lim Wai Feng; Mohd Asif Mohd Sukri; Mohd Nur Fakhruzzaman Noorizhab; Mohd Zaki Salleh; Sazzli Shahlan Kassim; Siti Farah Alwani Mohd Nawi; Siti Hamimah Sheikh Abdul Kadir; Teh Lay Kek; Wang Seok Mui
EPI_ISL_4853552	Hospital Kuala Kubu Bharu	Malaysia Genome Institute	Azrin Ahmad; Enizza Kasim; Irni Suhayu Sapian; Mohd Faizal Abu Bakar; Mohd Ghows Mohd Azzam.; Mohd Noor Mat Isa; Nor Azfa Johari; Nurhezreen Md Iqbal; Shamsidar Sopie; Siti Noraini Othman; Yusuf Muhammad Noor
EPI_ISL_4513497	Hospital Melaka	UKM Medical Molecular Biology Institute (UMBI)	Mira Farzana binti Mohamad Mokhtar
EPI_ISL_5536425, EPI_ISL_5536426, EPI_ISL_5536427, EPI_ISL_5536428	Hospital Queen Elizabeth	Malaysia Genome Institute	Azrin Ahmad; Enizza Kasim; Irni Suhayu Sapian; Mohd Faizal Abu Bakar; Mohd Ghows Mohd Azzam.; Mohd Noor Mat Isa; Nor Azfa Johari; Nurhezreen Md Iqbal; Shamsidar Sopie; Siti Noraini Othman; Yusuf Muhammad Noor
EPI_ISL_4853663, EPI_ISL_5428517	Hospital Selayang	Malaysia Genome Institute	Azrin Ahmad; Enizza Kasim; Irni Suhayu Sapian; Mohd Faizal Abu Bakar; Mohd Ghows Mohd Azzam.; Mohd Noor Mat Isa; Nor Azfa Johari; Nurhezreen Md Iqbal; Shamsidar Sopie; Siti Noraini Othman; Yusuf Muhammad Noor
EPI_ISL_4853784, EPI_ISL_4853800, EPI_ISL_4853816, EPI_ISL_4853828, EPI_ISL_4853846, EPI_ISL_4853864, EPI_ISL_4853880, EPI_ISL_4853894, EPI_ISL_5428518, EPI_ISL_5428519, EPI_ISL_5428520, EPI_ISL_5428521			
see above	Hospital Sungai Buloh	Malaysia Genome Institute	Azrin Ahmad; Enizza Kasim; Irni Suhayu Sapian; Mohd Faizal Abu Bakar; Mohd Ghows Mohd Azzam.; Mohd Noor Mat Isa; Nor Azfa Johari; Nurhezreen Md Iqbal; Shamsidar Sopie; Siti Noraini Othman; Yusuf Muhammad Noor
EPI_ISL_5536456, EPI_ISL_6825231, EPI_ISL_6825232, EPI_ISL_6825240, EPI_ISL_6825248	Hospital Tawau	Malaysia Genome Institute	Azrin Ahmad; Enizza Kasim; Irni Suhayu Sapian; Mohd Faizal Abu Bakar; Mohd Ghows Mohd Azzam.; Mohd Noor Mat Isa; Nor Azfa Johari; Nurhezreen Md Iqbal; Shamsidar Sopie; Siti Noraini Othman; Yusuf Muhammad Noor
EPI_ISL_4853905, EPI_ISL_4853913	Hospital Thomson Kota Damansara	Malaysia Genome Institute	Azrin Ahmad; Enizza Kasim; Irni Suhayu Sapian; Mohd Faizal Abu Bakar; Mohd Ghows Mohd Azzam.; Mohd Noor Mat Isa; Nor Azfa Johari; Nurhezreen Md Iqbal; Shamsidar Sopie; Siti Noraini Othman; Yusuf Muhammad Noor
EPI_ISL_6960068, EPI_ISL_6960069, EPI_ISL_6960070, EPI_ISL_6960071, EPI_ISL_6960072, EPI_ISL_6960073, EPI_ISL_11078682, EPI_ISL_11078721, EPI_ISL_11078722, EPI_ISL_11078730, EPI_ISL_11078747, EPI_ISL_11078759, EPI_ISL_11078760, EPI_ISL_11078762, EPI_ISL_11078768, EPI_ISL_11078776, EPI_ISL_11078777			
see above	Institut Biologi Molekul Perubatan UKM (UMBI)	UKM Medical Molecular Biology Institute (UMBI)	Khairun Nur Abd Ghafar; Mira Farzana Mohamad Mokhtar; Mira Farzana binti Mohamad Mokhtar; Muhiddin Ishak; Nor Azila Muhammad Azami; Nur Alyaa Afifah Md Shahri; Nurul Syakima Ab Mutalib; Rahman Jamal; Ryia Illani Mohd Yunos; Siti Nur Hasanah Mohd Yusuf; Zahirrah Begam Mohamed Rasheed
EPI_ISL_4435528	JABATAN PATOLOGI, HOSPITAL PULAU PINANG	iPROMISE, UiTM	Ariza Adnan; Fadzilah Mohd Nor; Lim Wai Feng; Mohd Asif Mohd Sukri; Mohd Nur Fakhruzzaman Noorizhab; Mohd Zaki Salleh; Sazzli Shahlan Kassim; Siti Farah Alwani Mohd Nawi; Siti Hamimah Sheikh Abdul Kadir; Teh Lay Kek; Wang Seok Mui
EPI_ISL_6132023, EPI_ISL_6132030, EPI_ISL_6132038, EPI_ISL_6132040	JABATAN PATOLOGI, HRPZ II	Department of Medical Microbiology, Faculty of Medicine, University of Malaya; University of Malaya Medical Centre	I-Ching SAM; Jolene Yin Ling FU; Omar Khalilur Rahman; Yoke Fun Chan
EPI_ISL_6132022, EPI_ISL_8254283, EPI_ISL_8254285	JABATAN PERUBATAN FORENSIK HOSPITAL SULTANAH BAHIYAH	Department of Medical Microbiology, Faculty of Medicine, University of Malaya; University of Malaya Medical Centre	I-Ching SAM; Jolene Yin Ling FU; Omar Khalilur Rahman; Yoke Fun Chan
EPI_ISL_5417172, EPI_ISL_5417176, EPI_ISL_5425839	JABATAN PERUBATAN FORENSIK HOSPITAL SULTANAH BAHIYAH	Department of Medical Microbiology, University Malaya Medical Centre	I-Ching SAM; Jolene Yin Ling FU; Omar Khalilur Rahman; Yoke Fun Chan
EPI_ISL_4463190, EPI_ISL_4463191, EPI_ISL_4463192, EPI_ISL_4891927	JABATAN PERUBATAN FORENSIK, HOSPITAL TUANKU JA'AFAR SEREMBAN	UKM Medical Molecular Biology Institute (UMBI)	Mira Farzana binti Mohamad Mokhtar; Nur Alyaa Afifah Md Shahri
EPI_ISL_4435539, EPI_ISL_4740368, EPI_ISL_4740370, EPI_ISL_4740373, EPI_ISL_4740374, EPI_ISL_4740379, EPI_ISL_4740380, EPI_ISL_4740401, EPI_ISL_5261871, EPI_ISL_5261872, EPI_ISL_5261895, EPI_ISL_5261896, EPI_ISL_5261897, EPI_ISL_5261898, EPI_ISL_5261899, EPI_ISL_5261900, EPI_ISL_5450478, EPI_ISL_5450503, EPI_ISL_5450504, EPI_ISL_5450511, EPI_ISL_5450515, EPI_ISL_5450516, EPI_ISL_5450518, EPI_ISL_5450519, EPI_ISL_5450520			
see above	JKN PAHANG	iPROMISE, UiTM	Ariza Adnan; Fadzilah Mohd Nor; Lim Wai Feng; Mohd Asif Mohd Sukri; Mohd Nur Fakhruzzaman Noorizhab; Mohd Zaki Salleh; Sazzli Shahlan Kassim; Siti Farah Alwani Mohd Nawi; Siti Hamimah Sheikh Abdul Kadir; Teh Lay Kek; Wang Seok Mui
EPI_ISL_5450286	Jabatan Forensik Hospital Pulau Pinang	iPROMISE, UiTM	Ariza Adnan; Fadzilah Mohd Nor; Lim Wai Feng; Mohd Asif Mohd Sukri; Mohd Nur Fakhruzzaman Noorizhab; Mohd Zaki Salleh; Sazzli Shahlan Kassim; Siti Farah Alwani Mohd Nawi; Siti Hamimah Sheikh Abdul Kadir; Teh Lay Kek; Wang Seok Mui

EPI_ISL_5417116, EPI_ISL_5417117, EPI_ISL_5417118, EPI_ISL_5417119, EPI_ISL_5417165, EPI_ISL_5417180, EPI_ISL_5417181			
see above	Jabatan Forensik Hospital Sultanah Bahiyah	Department of Medical Microbiology, University Malaya Medical Centre	I-Ching SAM; Jolene Yin Ling FU; Omar Khalilur Rahman; Yoke Fun Chan
EPI_ISL_8090603	Jabatan Forensik Hospital Sultanah Bahiyah	Tropical Infectious Diseases Research & Education Centre (TIDREC), Universiti Malaya	AsmaAnati CheMatSeri; Che-Norainon Yaacob; Jia-Yi Tan; Jo-Ern Wong; Kim-Kee Tan; Mulya-Mustika-Sari Zulkifli; Noor Syahida Azizan; Nur-Hidayana Mahfodz; Sazaly AbuBakar; Siti-Sarah Nor'e
EPI_ISL_8090607	Jabatan Kecemasan, Hospital Sultan Ismail Petra, Kuala Krai	Tropical Infectious Diseases Research & Education Centre (TIDREC), Universiti Malaya	AsmaAnati CheMatSeri; Che-Norainon Yaacob; Jia-Yi Tan; Jo-Ern Wong; Kim-Kee Tan; Mulya-Mustika-Sari Zulkifli; Noor Syahida Azizan; Nur-Hidayana Mahfodz; Sazaly AbuBakar; Siti-Sarah Nor'e
EPI_ISL_5536429, EPI_ISL_5536430, EPI_ISL_5536431	Jabatan Kesihatan Negeri WP Labuan	Malaysia Genome Institute	Azrin Ahmad; Enizza Kasim; Irni Suhayu Sapian; Mohd Faizal Abu Bakar; Mohd Ghows Mohd Azzam.; Mohd Noor Mat Isa; Nor Azfa Johari; Nurhezreen Md Iqbal; Shamsidar Sopie; Siti Noraini Othman; Yusuf Muhammad Noor
EPI_ISL_5450264, EPI_ISL_5450272, EPI_ISL_5450280, EPI_ISL_5450296	Jabatan Perubatan Forensik	iPROMISE, UiTM	Ariza Adnan; Fadzilah Mohd Nor; Lim Wai Feng; Mohd Asif Mohd Sukri; Mohd Nur Fakhruzzaman Noorizhab; Mohd Zaki Salleh; Sazzli Shahlan Kassim; Siti Farah Alwani Mohd Nawi; Siti Hamimah Sheikh Abdul Kadir; Teh Lay Kek; Wang Seok Mui
EPI_ISL_5417161, EPI_ISL_5425840	KK KUAH	Department of Medical Microbiology, University Malaya Medical Centre	I-Ching SAM; Jolene Yin Ling FU; Omar Khalilur Rahman; Yoke Fun Chan
EPI_ISL_6825300, EPI_ISL_6825301, EPI_ISL_6825302, EPI_ISL_6825303, EPI_ISL_6825304, EPI_ISL_6825305, EPI_ISL_6825306, EPI_ISL_6825307, EPI_ISL_6825308, EPI_ISL_6825309, EPI_ISL_6825310, EPI_ISL_6825311, EPI_ISL_6825312			
see above	Klinik Kesihatan Mempaga (Felda) (Pk Fasa I)	Malaysia Genome Institute	Azrin Ahmad; Enizza Kasim; Irni Suhayu Sapian; Mohd Faizal Abu Bakar; Mohd Ghows Mohd Azzam.; Mohd Noor Mat Isa; Nor Azfa Johari; Nurhezreen Md Iqbal; Shamsidar Sopie; Siti Noraini Othman; Yusuf Muhammad Noor
EPI_ISL_8745597, EPI_ISL_8745598, EPI_ISL_8745599, EPI_ISL_8745600, EPI_ISL_8745601	Klinik Kesihatan Mempaga (Felda) (Pk Fasa I)	Malaysia Genome and Vaccine Institute	Apical Scientific Sdn Bhd's Team; Azrin Ahmad; Carey Wee Kai Li; Enizza Kasim; Irni Suhayu Sapian; Jilian Michelle Wong Tzeling; Leong Wai Mun; Mohd Faizal Abu Bakar; Mohd Ghows Mohd Azzam.; Mohd Noor Mat Isa; Nor Azfa Johari; Nurhezreen Md Iqbal; Rose Iszati Ismet Nayan; Shamsidar Sopie; Siti Noraini Othman; Yusuf Muhammad Noor
EPI_ISL_5113788	Kota Marudu District Health Office	Institute for Medical Research, Infectious Disease Research Centre, National Institutes of Health, Ministry of Health Malaysia	Ahmad FA; Ahmad Fazilah NA; Anasir MI; Azizan MA; Kamel K; Mohd Zawawi Z; Norhisham SN; Ramly N; Robert F; Suppiah J; Thayan R
EPI_ISL_4730437, EPI_ISL_4730441	Kudat Hospital	Institute for Medical Research, Infectious Disease Research Centre, National Institutes of Health, Ministry of Health Malaysia	Ahmad FA; Ahmad Fazilah NA; Anasir MI; Azizan MA; Kamel K; Mohd Zawawi Z; Norhisham SN; Ramly N; Robert F; Suppiah J; Thayan R
EPI_ISL_4730443, EPI_ISL_5095024, EPI_ISL_5395847, EPI_ISL_5395851, EPI_ISL_5395855, EPI_ISL_5395940, EPI_ISL_5395982, EPI_ISL_6773973, EPI_ISL_6773975, EPI_ISL_6773993, EPI_ISL_6774010, EPI_ISL_6774011, EPI_ISL_6774012			
see above	Lahad Datu Hospital	Institute for Medical Research, Infectious Disease Research Centre, National Institutes of Health, Ministry of Health Malaysia	Ahmad FA; Ahmad Fazilah NA; Anasir MI; Azizan MA; Kamel K; Mohamad Sukri MZ; Mohd Zawawi Z; Norhisham SN; Ramly N; Robert F; Rosli NR; Suppiah J; Thayan R
EPI_ISL_4730447	Langkon Health Clinic	Institute for Medical Research, Infectious Disease Research Centre, National Institutes of Health, Ministry of Health Malaysia	Ahmad FA; Ahmad Fazilah NA; Anasir MI; Azizan MA; Kamel K; Mohd Zawawi Z; Norhisham SN; Ramly N; Robert F; Suppiah J; Thayan R
EPI_ISL_5095026, EPI_ISL_5095030	Miri Hospital	Institute for Medical Research, Infectious Disease Research Centre, National Institutes of Health, Ministry of Health Malaysia	Ahmad FA; Ahmad Fazilah NA; Anasir MI; Azizan MA; Kamel K; Mohd Zawawi Z; Norhisham SN; Ramly N; Robert F; Suppiah J; Thayan R
EPI_ISL_5051850, EPI_ISL_5052101, EPI_ISL_5052102, EPI_ISL_5052103	Miri Hospital Molecular Diagnostic Lab, Miri	Institute of Health and Community Medicine	Amir Safuan bin Khamshah; Chan Chia Jui; Chua Hock Hin; David Perera; Ooi Mong How; Tonnii Sia Loong Loong; Wong Jyn Shan
EPI_ISL_8090609, EPI_ISL_8090610, EPI_ISL_8090611, EPI_ISL_8090612	PEJABAT KESIHATAN DAERAH KUALA KRAI	Tropical Infectious Diseases Research & Education Centre (TIDREC), Universiti Malaya	AsmaAnati CheMatSeri; Che-Norainon Yaacob; Jia-Yi Tan; Jo-Ern Wong; Kim-Kee Tan; Mulya-Mustika-Sari Zulkifli; Noor Syahida Azizan; Nur-Hidayana Mahfodz; Sazaly AbuBakar; Siti-Sarah Nor'e
EPI_ISL_5261889	PEJABAT KESIHATAN DAERAH SEBERANG PERAI SELATAN	iPROMISE, UiTM	Ariza Adnan; Fadzilah Mohd Nor; Lim Wai Feng; Mohd Asif Mohd Sukri; Mohd Nur Fakhruzzaman Noorizhab; Mohd Zaki Salleh; Sazzli Shahlan Kassim; Siti Farah Alwani Mohd Nawi; Siti Hamimah Sheikh Abdul Kadir; Teh Lay Kek; Wang Seok Mui
EPI_ISL_6132011	PEJABAT KESIHATAN PASIR PUTEH	Department of Medical Microbiology, Faculty of Medicine, University of Malaya; University of Malaya Medical Centre	I-Ching SAM; Jolene Yin Ling FU; Omar Khalilur Rahman; Yoke Fun Chan
EPI_ISL_4731196, EPI_ISL_4731208	PKD JELEBU	UKM Medical Molecular Biology Institute (UMBI)	Mira Farzana binti Mohamad Mokhtar
EPI_ISL_4731228, EPI_ISL_4731229, EPI_ISL_5365877	PKD JOHOR BAHRU	UKM Medical Molecular Biology Institute (UMBI)	Mira Farzana binti Mohamad Mokhtar
EPI_ISL_4463183, EPI_ISL_4463184, EPI_ISL_4463189	PKD Jempol	UKM Medical Molecular Biology Institute (UMBI)	Nur Alyaa Afifah Md Shahri
EPI_ISL_4731193	PKD MELAKA TENGAH	UKM Medical Molecular Biology Institute (UMBI)	Mira Farzana binti Mohamad Mokhtar
EPI_ISL_5417611	PKD PD	UKM Medical Molecular Biology Institute (UMBI)	Mira Farzana binti Mohamad Mokhtar
EPI_ISL_4731217, EPI_ISL_4731220, EPI_ISL_4731222, EPI_ISL_4731226	PKD PORT DICKSON	UKM Medical Molecular Biology Institute (UMBI)	Mira Farzana binti Mohamad Mokhtar
EPI_ISL_5450106, EPI_ISL_5450426	PKD SEBERANG PERAI UTARA PENANG	iPROMISE, UiTM	Ariza Adnan; Fadzilah Mohd Nor; Lim Wai Feng; Mohd Asif Mohd Sukri; Mohd Nur Fakhruzzaman Noorizhab; Mohd Zaki Salleh; Sazzli Shahlan Kassim; Siti Farah Alwani Mohd Nawi; Siti Hamimah Sheikh Abdul Kadir; Teh Lay Kek; Wang Seok Mui
EPI_ISL_4463177, EPI_ISL_4463178	PKD SEREMBAN	UKM Medical Molecular Biology Institute (UMBI)	Nur Alyaa Afifah Md Shahri
EPI_ISL_4740369, EPI_ISL_4740371, EPI_ISL_4740377, EPI_ISL_5261891	PKD SPT	iPROMISE, UiTM	Ariza Adnan; Fadzilah Mohd Nor; Lim Wai Feng; Mohd Asif Mohd Sukri; Mohd Nur Fakhruzzaman Noorizhab; Mohd Zaki Salleh; Sazzli Shahlan Kassim; Siti Farah Alwani Mohd Nawi; Siti Hamimah Sheikh Abdul Kadir; Teh Lay Kek; Wang Seok Mui
EPI_ISL_4236999, EPI_ISL_4237000, EPI_ISL_4237001, EPI_ISL_4237002, EPI_ISL_4463176, EPI_ISL_4731215, EPI_ISL_5365862			
see above	PKD TAMPIN	UKM Medical Molecular Biology Institute (UMBI)	Mira Farzana binti Mohamad Mokhtar; Nur Alyaa Afifah Md Shahri
EPI_ISL_5261890, EPI_ISL_5261892	PKDSPT	iPROMISE, UiTM	Ariza Adnan; Fadzilah Mohd Nor; Lim Wai Feng; Mohd Asif Mohd Sukri; Mohd Nur Fakhruzzaman Noorizhab; Mohd Zaki Salleh; Sazzli Shahlan Kassim; Siti Farah Alwani Mohd Nawi; Siti Hamimah Sheikh Abdul Kadir; Teh Lay Kek; Wang Seok Mui
EPI_ISL_4122468	Pejabat Kesihatan Daerah Kangar	iPROMISE, UiTM	Ariza Adnan; Fadzilah Mohd Nor; Lim Wai Feng; Mohd Asif Mohd Sukri; Mohd Nur Fakhruzzaman Noorizhab; Mohd Zaki Salleh; Sazzli Shahlan Kassim; Siti Farah Alwani Mohd Nawi; Siti Hamimah Sheikh Abdul Kadir; Teh Lay Kek; Wang Seok Mui
EPI_ISL_5536422, EPI_ISL_5536423, EPI_ISL_5536424	Pejabat Kesihatan Kawasan Tawau	Malaysia Genome Institute	Azrin Ahmad; Enizza Kasim; Irni Suhayu Sapian; Mohd Faizal Abu Bakar; Mohd Ghows Mohd Azzam.; Mohd Noor Mat Isa; Nor Azfa Johari; Nurhezreen Md Iqbal; Shamsidar Sopie; Siti Noraini Othman; Yusuf Muhammad Noor
EPI_ISL_4730519	Port Klang Health Clinic	Institute for Medical Research, Infectious Disease Research Centre, National Institutes of Health, Ministry of Health Malaysia	Ahmad FA; Ahmad Fazilah NA; Anasir MI; Azizan MA; Kamel K; Mohd Zawawi Z; Norhisham SN; Ramly N; Robert F; Suppiah J; Thayan R
EPI_ISL_6774025	Queen Elizabeth Hospital	Institute for Medical Research, Infectious Disease Research Centre, National Institutes of Health, Ministry of Health Malaysia	Ahmad FA; Ahmad Fazilah NA; Anasir MI; Azizan MA; Kamel K; Mohamad Sukri MZ; Mohd Zawawi Z; Norhisham SN; Ramly N; Robert F; Rosli NR; Suppiah J; Thayan R
EPI_ISL_5051744, EPI_ISL_5051783, EPI_ISL_5051802, EPI_ISL_5051933, EPI_ISL_5051934, EPI_ISL_5051935, EPI_ISL_5051936, EPI_ISL_5051937, EPI_ISL_5051938, EPI_ISL_5051939, EPI_ISL_5051940, EPI_ISL_5051941, EPI_ISL_5051942, EPI_ISL_5051943, EPI_ISL_5051944, EPI_ISL_5051945, EPI_ISL_5051946, EPI_ISL_5051947, EPI_ISL_5051948, EPI_ISL_5052123, EPI_ISL_5052124, EPI_ISL_5052125, EPI_ISL_5052126, EPI_ISL_5052127, EPI_ISL_5052128, EPI_ISL_5052129, EPI_ISL_5052130, EPI_ISL_5052131, EPI_ISL_5052132, EPI_ISL_5052133, EPI_ISL_5052164, EPI_ISL_5052195			
see above	Rejang Medical Centre (Sibu)	Institute of Health and Community Medicine	Chan Chia Jui; Chua Hock Hin; David Perera; Ooi Mong How; Tonnii Sia Loong Loong; Wong Jyn Shan
EPI_ISL_5417626, EPI_ISL_5417629	SWAT TEAM HPSF	UKM Medical Molecular Biology Institute (UMBI)	Mira Farzana binti Mohamad Mokhtar
EPI_ISL_5051760, EPI_ISL_5051892, EPI_ISL_5051893, EPI_ISL_5051894, EPI_ISL_5051896, EPI_ISL_5051913, EPI_ISL_5052003, EPI_ISL_5052105, EPI_ISL_5052106, EPI_ISL_5052182, EPI_ISL_5052183, EPI_ISL_5052184, EPI_ISL_6969015, EPI_ISL_6969029, EPI_ISL_6969037, EPI_ISL_6970028			
see above	Sarawak General Hospital (Kuching)	Institute of Health and Community Medicine	Chan Chia Jui; Chua Hock Hin; David Perera; Ooi Mong How; Tonnii Sia Loong Loong; Wong Jyn Shan; Wong Kiing Aik
EPI_ISL_5051872, EPI_ISL_5051873, EPI_ISL_5051874, EPI_ISL_5051875, EPI_ISL_5051876, EPI_ISL_5051877, EPI_ISL_5051878, EPI_ISL_5051879, EPI_ISL_5051880, EPI_ISL_5051881, EPI_ISL_5051882, EPI_ISL_5051883, EPI_ISL_5051884, EPI_ISL_5051885, EPI_ISL_5051886, EPI_ISL_5051887, EPI_ISL_5051888, EPI_ISL_5051889, EPI_ISL_5051890, EPI_ISL_8188736			
see above	Sarawak Heart Centre (SHC), Kota Samarahan	Institute of Health and Community Medicine	Chan Chia Jui; Chua Hock Hin; David Perera; Ooi Mong How; Tonnii Sia Loong Loong; Wong Jyn Shan
EPI_ISL_4730524	Sepang District Health Office	Institute for Medical Research, Infectious Disease Research Centre, National Institutes of Health, Ministry of Health Malaysia	Ahmad FA; Ahmad Fazilah NA; Anasir MI; Azizan MA; Kamel K; Mohd Zawawi Z; Norhisham SN; Ramly N; Robert F; Suppiah J; Thayan R
EPI_ISL_6773974, EPI_ISL_6774032, EPI_ISL_6774056	Sungai Buloh Hospital	Institute for Medical Research, Infectious Disease Research Centre, National Institutes of Health, Ministry of Health Malaysia	Ahmad FA; Ahmad Fazilah NA; Anasir MI; Azizan MA; Kamel K; Mohamad Sukri MZ; Mohd Zawawi Z; Norhisham SN; Ramly N; Robert F; Rosli NR; Suppiah J; Thayan R
EPI_ISL_8745568	Thomson Hospital Kota Damansara	Malaysia Genome and Vaccine Institute	Apical Scientific Sdn Bhd's Team; Azrin Ahmad; Carey Wee Kai Li; Enizza Kasim; Irni Suhayu Sapian; Jilian Michelle Wong Tzeling; Leong Wai Mun; Mohd Faizal Abu Bakar; Mohd Ghows Mohd Azzam.; Mohd Noor Mat Isa; Nor Azfa Johari; Nurhezreen Md Iqbal; Rose Iszati Ismet Nayan; Shamsidar Sopie; Siti Noraini Othman; Yusuf Muhammad Noor
EPI_ISL_4731223, EPI_ISL_5365871, EPI_ISL_5365876	UKA HEBHK	UKM Medical Molecular Biology Institute (UMBI)	Mira Farzana binti Mohamad Mokhtar
EPI_ISL_8090629	UKKP	Tropical Infectious Diseases Research & Education Centre (TIDREC), Universiti Malaya	AsmaAnati CheMatSeri; Che-Norainon Yaacob; Jia-Yi Tan; Jo-Ern Wong; Kim-Kee Tan; Mulya-Mustika-Sari Zulkifli; Noor Syahida Azizan; Nur-Hidayana Mahfodz; Sazaly AbuBakar; Siti-Sarah Nor'e
EPI_ISL_4740376	UNIT FORENSIK HOSPITAL SEBERANG JAYA	iPROMISE, UiTM	Ariza Adnan; Fadzilah Mohd Nor; Lim Wai Feng; Mohd Asif Mohd Sukri; Mohd Nur Fakhruzzaman Noorizhab; Mohd Zaki Salleh; Sazzli Shahlan Kassim; Siti Farah Alwani Mohd Nawi; Siti Hamimah Sheikh Abdul Kadir; Teh Lay Kek; Wang Seok Mui
EPI_ISL_4513500, EPI_ISL_4513501, EPI_ISL_4513502, EPI_ISL_4513503, EPI_ISL_4513509, EPI_ISL_4731187, EPI_ISL_4731197, EPI_ISL_4731209, EPI_ISL_4731216, EPI_ISL_4816825, EPI_ISL_4816827, EPI_ISL_5365878			
see above	UNIT KESIHATAN AWAM HEBHK	UKM Medical Molecular Biology Institute (UMBI)	Mira Farzana binti Mohamad Mokhtar
EPI_ISL_5417126	UNIT KESIHATAN DAN KESELAMATAN PEKERJA HOSPITAL	Department of Medical Microbiology, University Malaya Medical Centre	I-Ching SAM; Jolene Yin Ling FU; Omar Khalilur Rahman; Yoke Fun Chan
EPI_ISL_4731190	UNIT KPAS	UKM Medical Molecular Biology Institute (UMBI)	Mira Farzana binti Mohamad Mokhtar
EPI_ISL_4463179, EPI_ISL_4463180, EPI_ISL_4463185, EPI_ISL_4463186, EPI_ISL_4463187, EPI_ISL_4463188, EPI_ISL_4513499, EPI_ISL_4731206			

see above	UNIT KPAS, PKD MELAKA TENGAH	UKM Medical Molecular Biology Institute (UMBI)	Mira Farzana binti Mohamad Mokhtar; Nur Alyaa Afifah Md Shahri
EPI_ISL_5261887, EPI_ISL_5261888	Unit Perubatan Forensik, Hospital Seberang Jaya	iPROMISE, UiTM	Ariza Adnan; Fadzilah Mohd Nor; Lim Wai Feng; Mohd Asif Mohd Sukri; Mohd Nur Fakhruzzaman Noorizhab; Mohd Zaki Salleh; Sazzli Shahlan Kassim; Siti Farah Alwani Mohd Nawi; Siti Hamimah Sheikh Abdul Kadir; Teh Lay Kek; Wang Seok Mui
EPI_ISL_4513496	Unit keselamatan dan Kesihatan Pekerjaan Hospital Melaka	UKM Medical Molecular Biology Institute (UMBI)	Mira Farzana binti Mohamad Mokhtar
EPI_ISL_5429414	WAD 6 MEDIKAL HOSPITAL SEGAMAT	UKM Medical Molecular Biology Institute (UMBI)	Mira Farzana binti Mohamad Mokhtar
EPI_ISL_5429421	Wad 6	UKM Medical Molecular Biology Institute (UMBI)	Mira Farzana binti Mohamad Mokhtar
EPI_ISL_5261901, EPI_ISL_5261906, EPI_ISL_5261909	iPROMISE, UiTM	iPROMISE, UiTM	Ariza Adnan; Fadzilah Mohd Nor; Lim Wai Feng; Mohd Asif Mohd Sukri; Mohd Nur Fakhruzzaman Noorizhab; Mohd Zaki Salleh; Sazzli Shahlan Kassim; Siti Farah Alwani Mohd Nawi; Siti Hamimah Sheikh Abdul Kadir; Teh Lay Kek; Wang Seok Mui

We gratefully acknowledge the following Authors from the Originating laboratories responsible for obtaining the specimens, as well as the Submitting laboratories where the genome data were generated and shared via GISAID, on which this research is based.

All Submitters of data may be contacted directly via www.gisaid.org

Authors are sorted alphabetically.

Acknowledgement EPI_SET Identifier: EPI_SET_20220603rd

Accession ID	Originating Laboratory	Submitting Laboratory	Authors
EPI_ISL_5051753, EPI_ISL_5051758, EPI_ISL_5051763, EPI_ISL_5051769, EPI_ISL_5051774, EPI_ISL_5051775, EPI_ISL_5051779, EPI_ISL_5051797, EPI_ISL_5051798, EPI_ISL_5051821, EPI_ISL_5051822, EPI_ISL_5051823, EPI_ISL_5051824, EPI_ISL_5051825, EPI_ISL_5051826, EPI_ISL_5051827, EPI_ISL_5051828, EPI_ISL_5051829, EPI_ISL_5051830, EPI_ISL_5051831, EPI_ISL_5051832, EPI_ISL_5051907, EPI_ISL_5051908, EPI_ISL_5052069, EPI_ISL_5052070, EPI_ISL_5052071, EPI_ISL_5052072, EPI_ISL_5052073, EPI_ISL_5052074, EPI_ISL_5052075, EPI_ISL_5052076, EPI_ISL_5052077, EPI_ISL_5052078, EPI_ISL_5052079, EPI_ISL_5052080, EPI_ISL_5052081, EPI_ISL_5052082, EPI_ISL_5052083, EPI_ISL_5052084, EPI_ISL_5052085, EPI_ISL_5052093, EPI_ISL_5052094, EPI_ISL_5052095, EPI_ISL_5052096, EPI_ISL_5052097, EPI_ISL_5052163, EPI_ISL_5896166			
see above	Bintulu Hospital PCR Lab, Bintulu	Institute of Health and Community Medicine	Chan Chia Jui; Chua Hock Hin; David Perera; Ooi Mong How; Tan Lee See; Tonnii Sia Loong Loong; Wong Jyn Shan
EPI_ISL_5894118, EPI_ISL_5896100, EPI_ISL_6968067, EPI_ISL_6968085, EPI_ISL_6968100, EPI_ISL_6968116, EPI_ISL_6968126, EPI_ISL_6968140, EPI_ISL_6969146, EPI_ISL_6970385, EPI_ISL_6970386			
see above	Bintulu Medical Centre (Bintulu)	Institute of Health and Community Medicine	Chan Chia Jui; Chua Hock Hin; David Perera; Ooi Mong How; Tonnii Sia Loong Loong; Wong Jyn Shan
EPI_ISL_5051964, EPI_ISL_5051965, EPI_ISL_5051966, EPI_ISL_5051967, EPI_ISL_5051968, EPI_ISL_5051969, EPI_ISL_5051970, EPI_ISL_5051971, EPI_ISL_5051972, EPI_ISL_5051973, EPI_ISL_5051980, EPI_ISL_5051981, EPI_ISL_5051982, EPI_ISL_5051983, EPI_ISL_5051984, EPI_ISL_5051985, EPI_ISL_5051986, EPI_ISL_5052120, EPI_ISL_5052121, EPI_ISL_5052122, EPI_ISL_5052153, EPI_ISL_5052176, EPI_ISL_5894966, EPI_ISL_5894972, EPI_ISL_5894982, EPI_ISL_5894986, EPI_ISL_5894996, EPI_ISL_5896134			
see above	Borneo Medical Centre (Kuching)	Institute of Health and Community Medicine	Chan Chia Jui; Chua Hock Hin; David Perera; Ooi Mong How; Tonnii Sia Loong Loong; Wong Jyn Shan
EPI_ISL_5417115, EPI_ISL_5417127, EPI_ISL_5417171, EPI_ISL_5417182	CPRC JKNT	Department of Medical Microbiology, University Malaya Medical Centre	I-Ching SAM; Jolene Yin Ling FU; Omar Khalilur Rahman; Yoke Fun Chan
EPI_ISL_8090601, EPI_ISL_8090606, EPI_ISL_8090652	CPRC JKNT	Tropical Infectious Diseases Research & Education Centre (TIDREC), Universiti Malaya	AsmaAnati CheMatSeri; Che-Norainon Yaacob; Jia-Yi Tan; Jo-Ern Wong; Kim-Kee Tan; Mulya-Mustika-Sari Zulkifli; Noor Syahida Azizan; Nur-Hidayana Mahfodz; Sazaly AbuBakar; Siti-Sarah Nor’e
EPI_ISL_6586445, EPI_ISL_6586446, EPI_ISL_6586447, EPI_ISL_6586448, EPI_ISL_6586449, EPI_ISL_6586450, EPI_ISL_6586451, EPI_ISL_6586452, EPI_ISL_6586453, EPI_ISL_6586456, EPI_ISL_6586477, EPI_ISL_6586479, EPI_ISL_6586482, EPI_ISL_6586483			
see above	Clinical Research Centre (CRC), Sibu Hospital, Sibu	Institute of Health and Community Medicine	Chan Chia Jui; Chua Hock Hin; David Perera; Ooi Mong How; Tonnii Sia Loong Loong; Wong Jyn Shan
EPI_ISL_5395983	Department of Forensic Medicine, Hospital Queen Elizabeth	Institute for Medical Research, Infectious Disease Research Centre, National Institutes of Health, Ministry of Health Malaysia	Ahmad FA; Ahmad Fazilah NA; Anasir MI; Azizan MA; Kamel K; Mohd Zawawi Z; Norhisham SN; Ramly N; Robert F; Suppiah J; Thayan R
EPI_ISL_8564943	Department of Medical Microbiology and Parasitology, Hospital Universiti Sains Malaysia	Molecular Research Laboratory	Abdul Haris bin Muhammad; Ahmad Adebayo Irekeola; Ahmad Sukari Bin Halim; Aswini Leela; Azian Harun; Chan Yean Yean; Chua Wei Chuan; Chua Wei Lian; Engku Nur Syafirah bt Engku Abd Rahman; Farahana binti Mohamed Alexander Chong Shu Chien; Kirnpal Kaur Banga Singh; Lau Nyok Sean; Lee Lih Huey; Lim Shu Yong; Maizun binti Mohd Zain; Muhammad Azamuddeen bin Mohammad Nasir; Muhammad Fazli bin Khalid; Muhammad Nashrul Farhan Samsudin; Muhammad Zarul Hanifah Bin Md Zoqratt; Naveed Ahmed; Nazmi Liana Binti Azmi; Noor Hafizan binti Mat Salleh; Nurfadhlina Musa; Qasim Ayub; Rosline Hassan; Sadequr Rahman; Syahida binti Omar; Wan Mohd Zahiruddin Wan Mohammad; Wardah Yusof; Wilhelm Eng Wei Han; Zaini bin Hussin; Zakuan Zainy Deris
EPI_ISL_5782345	Department of Medical Microbiology, Hospital Pengajar Universiti Putra Malaysia	Department of Medical Microbiology, Hospital Pengajar Universiti Putra Malaysia	Afiqah Adzmi; Amiza Azmi; Azmiza Syawani Jasni; Chee Hui Yee; Leslie Than Thian Lung; Muadz Mohtar; Muhammad Mohd Isa; Narcisse MS Joseph; Niazlin Mohd Taib; Noor Hazirah Noor Azhari; Norlaila; Nur Raihana Ithnin; Nurul Huda Mohamed Rashidi; Nurul Nadiah Ismail; Rosni Ibrahim; Sallehhudin; Siti Norbaya Masri; Siti Zulaikha Zakariah; Suppiah J; Syafinaz Amin Nordin; Tengku Zetty Maztura Tengku Jamaluddin; Thayan R; Zamberi Sekawi
EPI_ISL_4602782, EPI_ISL_4602785	Forensic Department of Queen Elizabeth Hospital	Institute for Medical Research, Infectious Disease Research Centre, National Institutes of Health, Ministry of Health Malaysia	Ahmad FA; Ahmad Fazilah NA; Anasir MI; Azizan MA; Kamel K; Mohd Zawawi Z; Norhisyam SN; Ramly N; Robert F; Suppiah J; Thayan R
EPI_ISL_5396287, EPI_ISL_5396300	Forensic Unit, Keningau Hospital	Institute for Medical Research, Infectious Disease Research Centre, National Institutes of Health, Ministry of Health Malaysia	Ahmad FA; Ahmad Fazilah NA; Anasir MI; Azizan MA; Kamel K; Mohd Zawawi Z; Norhisham SN; Ramly N; Robert F; Suppiah J; Thayan R
EPI_ISL_5052005, EPI_ISL_5052006, EPI_ISL_5052007, EPI_ISL_6970035	Gribbles Pathology, Kuching	Institute of Health and Community Medicine	Chan Chia Jui; Chua Hock Hin; David Perera; Ooi Mong How; Reagan Entigu Linton; Tonnii Sia Loong Loong; Wong Jyn Shan
EPI_ISL_12628169	HCTM	UKM Medical Molecular Biology Institute (UMBI)	Khairun Nur Abd Ghafar; Mira Farzana Mohamad Mokhtar; Muhiddin Ishak; Nor Azila Muhammad Azami; Nur Alyaa Afifah Md Shahri; Nurul Syakima Ab Mutalib; Rahman Jamal; Ryia Illani Mohd Yunos; Siti Nur Hasanah Mohd Yusuf; Zahirrah Begam Mohamed Rasheed
EPI_ISL_4740363, EPI_ISL_4740364, EPI_ISL_4740366, EPI_ISL_4740367, EPI_ISL_5261893, EPI_ISL_5261894, EPI_ISL_5450199, EPI_ISL_5450207, EPI_ISL_5450211			
see above	HOSPITAL BALIK PULAU	iPROMISE, UiTM	Ariza Adnan; Fadzilah Mohd Nor; Lim Wai Feng; Mohd Asif Mohd Sukri; Mohd Nur Fakhruzzaman Noorizhab; Mohd Zaki Salleh; Sazzli Shahlan Kassim; Siti Farah Alwani Mohd Nawi; Siti Hamimah Sheikh Abdul Kadir; Teh Lay Kek; Wang Seok Mui
EPI_ISL_5450410	HOSPITAL BUKIT MERTAJAM	iPROMISE, UiTM	Ariza Adnan; Fadzilah Mohd Nor; Lim Wai Feng; Mohd Asif Mohd Sukri; Mohd Nur Fakhruzzaman Noorizhab; Mohd Zaki Salleh; Sazzli Shahlan Kassim; Siti Farah Alwani Mohd Nawi; Siti Hamimah Sheikh Abdul Kadir; Teh Lay Kek; Wang Seok Mui
EPI_ISL_5429411, EPI_ISL_5429415, EPI_ISL_5429424, EPI_ISL_5429429	HOSPITAL SEGAMAT	UKM Medical Molecular Biology Institute (UMBI)	Mira Farzana binti Mohamad Mokhtar
EPI_ISL_4891931, EPI_ISL_4891934, EPI_ISL_4891947, EPI_ISL_5365874, EPI_ISL_5417634	HOSPITAL SULTANAH NORA ISMAIL	UKM Medical Molecular Biology Institute (UMBI)	Mira Farzana binti Mohamad Mokhtar
EPI_ISL_5365879	HPSF MUAR	UKM Medical Molecular Biology Institute (UMBI)	Mira Farzana binti Mohamad Mokhtar
EPI_ISL_8090632, EPI_ISL_8090633, EPI_ISL_8090634, EPI_ISL_8090638, EPI_ISL_8090639, EPI_ISL_8090641, EPI_ISL_8090667, EPI_ISL_8090671, EPI_ISL_8090673, EPI_ISL_8090675, EPI_ISL_8090676, EPI_ISL_8090680, EPI_ISL_8090682			
see above	HRPB	Tropical Infectious Diseases Research & Education Centre (TIDREC), Universiti Malaya	AsmaAnati CheMatSeri; Che-Norainon Yaacob; Jia-Yi Tan; Jo-Ern Wong; Kim-Kee Tan; Mulya-Mustika-Sari Zulkifli; Noor Syahida Azizan; Nur-Hidayana Mahfodz; Sazaly AbuBakar; Siti-Sarah Nor’e
EPI_ISL_5365872	HSAJB	UKM Medical Molecular Biology Institute (UMBI)	Mira Farzana binti Mohamad Mokhtar
EPI_ISL_5536436	Hospital Ampang	Malaysia Genome Institute	Azrin Ahmad; Enizza Kasim; Irni Suhayu Sapian; Mohd Faizal Abu Bakar; Mohd Ghows Mohd Azzam.; Mohd Noor Mat Isa; Nor Azfa Johari; Nurhezreen Md Iqbal; Shamsidar Sopie; Siti Noraini Othman; Yusuf Muhammad Noor
EPI_ISL_6825225, EPI_ISL_6825226, EPI_ISL_6825227, EPI_ISL_6825228, EPI_ISL_6825229, EPI_ISL_6825242	Hospital Duchess Of Kent	Malaysia Genome Institute	Azrin Ahmad; Enizza Kasim; Irni Suhayu Sapian; Mohd Faizal Abu Bakar; Mohd Ghows Mohd Azzam.; Mohd Noor Mat Isa; Nor Azfa Johari; Nurhezreen Md Iqbal; Shamsidar Sopie; Siti Noraini Othman; Yusuf Muhammad Noor
EPI_ISL_8745574	Hospital Duchess Of Kent	Malaysia Genome and Vaccine Institute	Apical Scientific Sdn Bhd's Team; Azrin Ahmad; Carey Wee Kai Li; Enizza Kasim; Irni Suhayu Sapian; Jilian Michelle Wong Tzeling; Leong Wai Mun; Mohd Faizal Abu Bakar; Mohd Ghows Mohd Azzam.; Mohd Noor Mat Isa; Nor Azfa Johari; Nurhezreen Md Iqbal; Rose Iszati Ismet Nayan; Shamsidar Sopie; Siti Noraini Othman; Yusuf Muhammad Noor
EPI_ISL_5536449, EPI_ISL_5536450	Hospital Duchess of Kent	Malaysia Genome Institute	Azrin Ahmad; Enizza Kasim; Irni Suhayu Sapian; Mohd Faizal Abu Bakar; Mohd Ghows Mohd Azzam.; Mohd Noor Mat Isa; Nor Azfa Johari; Nurhezreen Md Iqbal; Shamsidar Sopie; Siti Noraini Othman; Yusuf Muhammad Noor
EPI_ISL_6825233, EPI_ISL_6825234, EPI_ISL_6825235, EPI_ISL_6825237, EPI_ISL_6825245	Hospital Lahad Datu	Malaysia Genome Institute	Azrin Ahmad; Enizza Kasim; Irni Suhayu Sapian; Mohd Faizal Abu Bakar; Mohd Ghows Mohd Azzam.; Mohd Noor Mat Isa; Nor Azfa Johari; Nurhezreen Md Iqbal; Shamsidar Sopie; Siti Noraini Othman; Yusuf Muhammad Noor
EPI_ISL_5159367, EPI_ISL_5159368	Hospital Sultanah Nora Ismail	UKM Medical Molecular Biology Institute (UMBI)	Mira Farzana binti Mohamad Mokhtar
EPI_ISL_5536432	Hospital Sungai Buloh	Malaysia Genome Institute	Azrin Ahmad; Enizza Kasim; Irni Suhayu Sapian; Mohd Faizal Abu Bakar; Mohd Ghows Mohd Azzam.; Mohd Noor Mat Isa; Nor Azfa Johari; Nurhezreen Md Iqbal; Shamsidar Sopie; Siti Noraini Othman; Yusuf Muhammad Noor
EPI_ISL_5536445, EPI_ISL_6825230, EPI_ISL_6825239, EPI_ISL_6825241, EPI_ISL_6825247, EPI_ISL_6825249	Hospital Tawau	Malaysia Genome Institute	Azrin Ahmad; Enizza Kasim; Irni Suhayu Sapian; Mohd Faizal Abu Bakar; Mohd Ghows Mohd Azzam.; Mohd Noor Mat Isa; Nor Azfa Johari; Nurhezreen Md Iqbal; Shamsidar Sopie; Siti Noraini Othman; Yusuf Muhammad Noor
EPI_ISL_8745570, EPI_ISL_8745572, EPI_ISL_8745575	Hospital Tawau	Malaysia Genome and Vaccine Institute	Apical Scientific Sdn Bhd's Team; Azrin Ahmad; Carey Wee Kai Li; Enizza Kasim; Irni Suhayu Sapian; Jilian Michelle Wong Tzeling; Leong Wai Mun; Mohd Faizal Abu Bakar; Mohd Ghows Mohd Azzam.; Mohd Noor Mat Isa; Nor Azfa Johari; Nurhezreen Md Iqbal; Rose Iszati Ismet Nayan; Shamsidar Sopie; Siti Noraini Othman; Yusuf Muhammad Noor
EPI_ISL_5536442	Hospital Thomson Kota Damansara	Malaysia Genome Institute	Azrin Ahmad; Enizza Kasim; Irni Suhayu Sapian; Mohd Faizal Abu Bakar; Mohd Ghows Mohd Azzam.; Mohd Noor Mat Isa; Nor Azfa Johari; Nurhezreen Md Iqbal; Shamsidar Sopie; Siti Noraini Othman; Yusuf Muhammad Noor
EPI_ISL_5417603	ICU Dahlia, Medical department	UKM Medical Molecular Biology Institute (UMBI)	Mira Farzana binti Mohamad Mokhtar
EPI_ISL_4891929, EPI_ISL_4891935	INSTITUT BIOLOGI MOLEKUL	UKM Medical Molecular Biology Institute	Mira Farzana binti Mohamad Mokhtar

	PERUBATAN UKM (UMBI)	(UMBI)	
EPI_ISL_6960074, EPI_ISL_6960075, EPI_ISL_6960076, EPI_ISL_6960077, EPI_ISL_6960078, EPI_ISL_6960079, EPI_ISL_6960080, EPI_ISL_6960081, EPI_ISL_6960082, EPI_ISL_6960083, EPI_ISL_6960084, EPI_ISL_6960085, EPI_ISL_6960086, EPI_ISL_6960087, EPI_ISL_6960088, EPI_ISL_11078704, EPI_ISL_11078708, EPI_ISL_11078716, EPI_ISL_11078732, EPI_ISL_11078742			
see above	Institut Biologi Molekul Perubatan UKM (UMBI)	UKM Medical Molecular Biology Institute (UMBI)	Khairun Nur Abd Ghafar; Mira Farzana Mohamad Mokhtar; Mira Farzana binti Mohamad Mokhtar; Muhiddin Ishak; Nor Azila Muhammad Azami; Nur Alyaa Afifah Md Shahri; Nurul Syakima Ab Mutalib; Rahman Jamal; Ryia Illani Mohd Yunos; Siti Nur Hasanah Mohd Yusuf; Zahirrah Begam Mohamed Rasheed
EPI_ISL_5450309	JABATAN FORENSIK HOSPITAL PULAU PINANG	iPROMISE, UiTM	Ariza Adnan; Fadzilah Mohd Nor; Lim Wai Feng; Mohd Asif Mohd Sukri; Mohd Nur Fakhruzzaman Noorizhab; Mohd Zaki Salleh; Sazzli Shahlan Kassim; Siti Farah Alwani Mohd Nawi; Siti Hamimah Sheikh Abdul Kadir; Teh Lay Kek; Wang Seok Mui
EPI_ISL_5417140, EPI_ISL_5417162, EPI_ISL_5417179, EPI_ISL_5425838	JABATAN PERUBATAN FORENSIK HOSPITAL SULTANAH BAHIYAH	Department of Medical Microbiology, University Malaya Medical Centre	I-Ching SAM; Jolene Yin Ling FU; Omar Khalilur Rahman; Yoke Fun Chan
EPI_ISL_5261910, EPI_ISL_5261911, EPI_ISL_5261912, EPI_ISL_5450486, EPI_ISL_5450495, EPI_ISL_5450505, EPI_ISL_5450512, EPI_ISL_5450517, EPI_ISL_5450521, EPI_ISL_5450522, EPI_ISL_6017989, EPI_ISL_6018031, EPI_ISL_6712805			
see above	JKN PAHANG	iPROMISE, UiTM	Ariza Adnan; Fadzilah Mohd Nor; Lim Wai Feng; Mohd Asif Mohd Sukri; Mohd Nur Fakhruzzaman Noorizhab; Mohd Zaki Salleh; Sazzli Shahlan Kassim; Siti Farah Alwani Mohd Nawi; Siti Hamimah Sheikh Abdul Kadir; Teh Lay Kek; Wang Seok Mui
EPI_ISL_6132047, EPI_ISL_6132052	Jabatan Forensik Hospital Sultanah Bahiyah	Department of Medical Microbiology, Faculty of Medicine, University of Malaya; University of Malaya Medical Centre	I-Ching SAM; Jolene Yin Ling FU; Omar Khalilur Rahman; Yoke Fun Chan
EPI_ISL_5417150, EPI_ISL_5417151, EPI_ISL_5417164, EPI_ISL_5417177, EPI_ISL_5417185	Jabatan Forensik Hospital Sultanah Bahiyah	Department of Medical Microbiology, University Malaya Medical Centre	I-Ching SAM; Jolene Yin Ling FU; Omar Khalilur Rahman; Yoke Fun Chan
EPI_ISL_8090627, EPI_ISL_8090661, EPI_ISL_8090687, EPI_ISL_8090688	Jabatan Forensik Hospital Sultanah Bahiyah	Tropical Infectious Diseases Research & Education Centre (TIDREC), Universiti Malaya	AsmaAnati CheMatSeri; Che-Norainon Yaacob; Jia-Yi Tan; Jo-Ern Wong; Kim-Kee Tan; Mulya-Mustika-Sari Zulkifli; Noor Syahida Azizan; Nur-Hidayana Mahfodz; Sazaly AbuBakar; Siti-Sarah Nor'e
EPI_ISL_5450299, EPI_ISL_5450304	Jabatan Perubatan Forensik	iPROMISE, UiTM	Ariza Adnan; Fadzilah Mohd Nor; Lim Wai Feng; Mohd Asif Mohd Sukri; Mohd Nur Fakhruzzaman Noorizhab; Mohd Zaki Salleh; Sazzli Shahlan Kassim; Siti Farah Alwani Mohd Nawi; Siti Hamimah Sheikh Abdul Kadir; Teh Lay Kek; Wang Seok Mui
EPI_ISL_9205571, EPI_ISL_9209918	Kajang Hospital	Institute for Medical Research, Infectious Disease Research Centre, National Institutes of Health, Ministry of Health Malaysia	Ahmad FA; Ahmad Fazilah NA; Anasir MI; Azizan MA; Kamel K; Mohamad Sukri MZ; Norhisham SN; Ramly N; Robert F; Rosli NR; Suppiah J; Thayan R
EPI_ISL_6825313, EPI_ISL_6825314, EPI_ISL_6825315	Klinik Kesihatan Pekan Tajau	Malaysia Genome Institute	Azrin Ahmad; Enizza Kasim; Irni Suhayu Sapian; Mohd Faizal Abu Bakar; Mohd Ghows Mohd Azzam.; Mohd Noor Mat Isa; Nor Azfa Johari; Nurhezreen Md Iqbal; Shamsidar Sopie; Siti Noraini Othman; Yusuf Muhammad Noor
EPI_ISL_8745602, EPI_ISL_8745603	Klinik Kesihatan Pekan Tajau	Malaysia Genome and Vaccine Institute	Apical Scientific Sdn Bhd's Team; Azrin Ahmad; Carey Wee Kai Li; Enizza Kasim; Irni Suhayu Sapian; Jilian Michelle Wong Tzeling; Leong Wai Mun; Mohd Faizal Abu Bakar; Mohd Ghows Mohd Azzam.; Mohd Noor Mat Isa; Nor Azfa Johari; Nurhezreen Md Iqbal; Rose Iszati Ismet Nayan; Shamsidar Sopie; Siti Noraini Othman; Yusuf Muhammad Noor
EPI_ISL_5113792	Kota Marudu District Health Office	Institute for Medical Research, Infectious Disease Research Centre, National Institutes of Health, Ministry of Health Malaysia	Ahmad FA; Ahmad Fazilah NA; Anasir MI; Azizan MA; Kamel K; Mohd Zawawi Z; Norhisham SN; Ramly N; Robert F; Suppiah J; Thayan R
EPI_ISL_6774027	Labuan Hospital	Institute for Medical Research, Infectious Disease Research Centre, National Institutes of Health, Ministry of Health Malaysia	Ahmad FA; Ahmad Fazilah NA; Anasir MI; Azizan MA; Kamel K; Mohamad Sukri MZ; Mohd Zawawi Z; Norhisham SN; Ramly N; Robert F; Rosli NR; Suppiah J; Thayan R
EPI_ISL_5395935, EPI_ISL_6774009, EPI_ISL_6774026	Lahad Datu Hospital	Institute for Medical Research, Infectious Disease Research Centre, National Institutes of Health, Ministry of Health Malaysia	Ahmad FA; Ahmad Fazilah NA; Anasir MI; Azizan MA; Kamel K; Mohamad Sukri MZ; Mohd Zawawi Z; Norhisham SN; Ramly N; Robert F; Rosli NR; Suppiah J; Thayan R
EPI_ISL_4602781, EPI_ISL_6774014	Lembah Pantai Health Office	Institute for Medical Research, Infectious Disease Research Centre, National Institutes of Health, Ministry of Health Malaysia	Ahmad FA; Ahmad Fazilah NA; Anasir MI; Azizan MA; Kamel K; Mohamad Sukri MZ; Mohd Zawawi Z; Norhisham SN; Norhisyam SN; Ramly N; Robert F; Rosli NR; Suppiah J; Thayan R
EPI_ISL_5095121	Medical Forensic Queen Elizabeth Hospital	Institute for Medical Research, Infectious Disease Research Centre, National Institutes of Health, Ministry of Health Malaysia	Ahmad FA; Ahmad Fazilah NA; Anasir MI; Azizan MA; Kamel K; Mohd Zawawi Z; Norhisham SN; Ramly N; Robert F; Suppiah J; Thayan R
EPI_ISL_4602788, EPI_ISL_4603809, EPI_ISL_4604200, EPI_ISL_5095415, EPI_ISL_5095533	Medical Forensic Unit, Duchess of Kent Hospital	Institute for Medical Research, Infectious Disease Research Centre, National Institutes of Health, Ministry of Health Malaysia	Ahmad FA; Ahmad Fazilah NA; Anasir MI; Azizan MA; Kamel K; Mohd Zawawi Z; Norhisham SN; Norhisyam SN; Ramly N; Robert F; Suppiah J; Thayan R
EPI_ISL_5095133	Medical Forensic Unit, Queen Elizabeth Hospital.	Institute for Medical Research, Infectious Disease Research Centre, National Institutes of Health, Ministry of Health Malaysia	Ahmad FA; Ahmad Fazilah NA; Anasir MI; Azizan MA; Kamel K; Mohd Zawawi Z; Norhisham SN; Ramly N; Robert F; Suppiah J; Thayan R
EPI_ISL_5052104	Miri Hospital Molecular Diagnostic Lab, Miri	Institute of Health and Community Medicine	Amir Safuan bin Khamshah; Chan Chia Jui; Chua Hock Hin; David Perera; Ooi Mong How; Tonnii Sia Loong Loong; Wong Jyn Shan
EPI_ISL_8090657	PEGAWAI PERUBATAN UD48	Tropical Infectious Diseases Research & Education Centre (TIDREC), Universiti Malaya	AsmaAnati CheMatSeri; Che-Norainon Yaacob; Jia-Yi Tan; Jo-Ern Wong; Kim-Kee Tan; Mulya-Mustika-Sari Zulkifli; Noor Syahida Azizan; Nur-Hidayana Mahfodz; Sazaly AbuBakar; Siti-Sarah Nor'e
EPI_ISL_6132020	PEJABAT KESIHATAN DAERAH	Department of Medical Microbiology, Faculty of Medicine, University of Malaya; University of Malaya Medical Centre	I-Ching SAM; Jolene Yin Ling FU; Omar Khalilur Rahman; Yoke Fun Chan
EPI_ISL_6131984	PEJABAT KESIHATAN DAERAH KUALA KRAI	Department of Medical Microbiology, Faculty of Medicine, University of Malaya; University of Malaya Medical Centre	I-Ching SAM; Jolene Yin Ling FU; Omar Khalilur Rahman; Yoke Fun Chan
EPI_ISL_8090653	PEJABAT KESIHATAN DAERAH KUALA KRAI	Tropical Infectious Diseases Research & Education Centre (TIDREC), Universiti Malaya	AsmaAnati CheMatSeri; Che-Norainon Yaacob; Jia-Yi Tan; Jo-Ern Wong; Kim-Kee Tan; Mulya-Mustika-Sari Zulkifli; Noor Syahida Azizan; Nur-Hidayana Mahfodz; Sazaly AbuBakar; Siti-Sarah Nor'e
EPI_ISL_4731188, EPI_ISL_4731192, EPI_ISL_4731201	PEJABAT KESIHATAN DAERAH MELAKA TENGAH	UKM Medical Molecular Biology Institute (UMBI)	Mira Farzana binti Mohamad Mokhtar
EPI_ISL_5417121	PEJABAT KESIHATAN DAERAH PASIR PUTEH	Department of Medical Microbiology, University Malaya Medical Centre	I-Ching SAM; Jolene Yin Ling FU; Omar Khalilur Rahman; Yoke Fun Chan
EPI_ISL_5450437, EPI_ISL_5450442, EPI_ISL_5450453, EPI_ISL_5450463	PEJABAT KESIHATAN DAERAH TIMUR LAUT	iPROMISE, UiTM	Ariza Adnan; Fadzilah Mohd Nor; Lim Wai Feng; Mohd Asif Mohd Sukri; Mohd Nur Fakhruzzaman Noorizhab; Mohd Zaki Salleh; Sazzli Shahlan Kassim; Siti Farah Alwani Mohd Nawi; Siti Hamimah Sheikh Abdul Kadir; Teh Lay Kek; Wang Seok Mui
EPI_ISL_5417128	PEJABAT KESIHATAN DAERAH TUMPAT	Department of Medical Microbiology, University Malaya Medical Centre	I-Ching SAM; Jolene Yin Ling FU; Omar Khalilur Rahman; Yoke Fun Chan
EPI_ISL_5159364, EPI_ISL_5159365, EPI_ISL_5159366, EPI_ISL_5365867	PKD Alor Gajah	UKM Medical Molecular Biology Institute (UMBI)	Mira Farzana binti Mohamad Mokhtar
EPI_ISL_5417124	PKD GUA MUSANG	Department of Medical Microbiology, University Malaya Medical Centre	I-Ching SAM; Jolene Yin Ling FU; Omar Khalilur Rahman; Yoke Fun Chan
EPI_ISL_4891945	PKD JOHOR BAHRU	UKM Medical Molecular Biology Institute (UMBI)	Mira Farzana binti Mohamad Mokhtar
EPI_ISL_4731218, EPI_ISL_4731225	PKD KUALA PILAH	UKM Medical Molecular Biology Institute (UMBI)	Mira Farzana binti Mohamad Mokhtar
EPI_ISL_5742674	PKD MERSING	UKM Medical Molecular Biology Institute (UMBI)	Mira Farzana binti Mohamad Mokhtar
EPI_ISL_4463181, EPI_ISL_4463182, EPI_ISL_4731211, EPI_ISL_4731219, EPI_ISL_4891928, EPI_ISL_4891930, EPI_ISL_4891932, EPI_ISL_4891936, EPI_ISL_5365861			
see above	PKD TAMPIN	UKM Medical Molecular Biology Institute (UMBI)	Mira Farzana binti Mohamad Mokhtar; Nur Alyaa Afifah Md Shahri
EPI_ISL_5417142	PKD Tanah Merah	Department of Medical Microbiology, University Malaya Medical Centre	I-Ching SAM; Jolene Yin Ling FU; Omar Khalilur Rahman; Yoke Fun Chan
EPI_ISL_5417129	Pejabat Kesihatan Daerah Jeli	Department of Medical Microbiology, University Malaya Medical Centre	I-Ching SAM; Jolene Yin Ling FU; Omar Khalilur Rahman; Yoke Fun Chan
EPI_ISL_4731207	Pejabat Kesihatan Daerah Melaka Tengah	UKM Medical Molecular Biology Institute (UMBI)	Mira Farzana binti Mohamad Mokhtar
EPI_ISL_5417175	Pejabat Kesihatan Daerah Pasir	Department of Medical Microbiology,	I-Ching SAM; Jolene Yin Ling FU; Omar Khalilur Rahman; Yoke Fun Chan

	Mas	University Malaya Medical Centre	
EPI_ISL_5417174	Pejabat Kesihatan Tanah Merah	Department of Medical Microbiology, University Malaya Medical Centre	I-Ching SAM; Jolene Yin Ling FU; Omar Khalilur Rahman; Yoke Fun Chan
EPI_ISL_5113798	Pitas District Health Office	Institute for Medical Research, Infectious Disease Research Centre, National Institutes of Health, Ministry of Health Malaysia	Ahmad FA; Ahmad Fazilah NA; Anasir MI; Azizan MA; Kamel K; Mohd Zawawi Z; Norhisham SN; Ramly N; Robert F; Suppiah J; Thayan R
EPI_ISL_5418733, EPI_ISL_5418995, EPI_ISL_5418999	Pontian District Health Office	Institute for Medical Research, Infectious Disease Research Centre, National Institutes of Health, Ministry of Health Malaysia	Ahmad FA; Ahmad Fazilah NA; Anasir MI; Azizan MA; Kamel K; Mohd Zawawi Z; Norhisham SN; Ramly N; Robert F; Suppiah J; Thayan R
EPI_ISL_5395719	Putrajaya Hospital	Institute for Medical Research, Infectious Disease Research Centre, National Institutes of Health, Ministry of Health Malaysia	Ahmad FA; Ahmad Fazilah NA; Anasir MI; Azizan MA; Kamel K; Mohd Zawawi Z; Norhisham SN; Ramly N; Robert F; Suppiah J; Thayan R
EPI_ISL_6774049	Queen Elizabeth Hospital	Institute for Medical Research, Infectious Disease Research Centre, National Institutes of Health, Ministry of Health Malaysia	Ahmad FA; Ahmad Fazilah NA; Anasir MI; Azizan MA; Kamel K; Mohamad Sukri MZ; Mohd Zawawi Z; Norhisham SN; Ramly N; Robert F; Rosli NR; Suppiah J; Thayan R
EPI_ISL_5113800	Ranau District Health Office	Institute for Medical Research, Infectious Disease Research Centre, National Institutes of Health, Ministry of Health Malaysia	Ahmad FA; Ahmad Fazilah NA; Anasir MI; Azizan MA; Kamel K; Mohd Zawawi Z; Norhisham SN; Ramly N; Robert F; Suppiah J; Thayan R
EPI_ISL_5051764, EPI_ISL_5051765, EPI_ISL_5051784, EPI_ISL_5052135, EPI_ISL_5052136, EPI_ISL_5052137, EPI_ISL_5052138, EPI_ISL_5052139, EPI_ISL_5052140, EPI_ISL_5052141, EPI_ISL_5052142, EPI_ISL_5052143, EPI_ISL_5052144, EPI_ISL_5052145, EPI_ISL_5052146, EPI_ISL_5052147, EPI_ISL_5052148, EPI_ISL_5052149, EPI_ISL_5052150, EPI_ISL_5052151, EPI_ISL_5052152, EPI_ISL_8650113			
see above	Rejang Medical Centre (Sibu)	Institute of Health and Community Medicine	Chan Chia Jui; Chua Hock Hin; David Perera; Ooi Mong How; Tonnii Sia Loong Loong; Wong Jyn Shan
EPI_ISL_4731212, EPI_ISL_4891937	SWAT TEAM HPSF	UKM Medical Molecular Biology Institute (UMBI)	Mira Farzana binti Mohamad Mokhtar
EPI_ISL_5051768, EPI_ISL_5051782, EPI_ISL_5051895, EPI_ISL_5052004, EPI_ISL_5052029, EPI_ISL_5052030, EPI_ISL_5052031, EPI_ISL_5052032, EPI_ISL_5052033, EPI_ISL_5052034, EPI_ISL_5052035, EPI_ISL_5052036, EPI_ISL_5052037, EPI_ISL_5052038, EPI_ISL_5052039, EPI_ISL_5052040, EPI_ISL_5052041, EPI_ISL_5052042, EPI_ISL_5052043, EPI_ISL_5052044, EPI_ISL_5052045, EPI_ISL_5052046, EPI_ISL_5052047, EPI_ISL_5052048, EPI_ISL_5052049, EPI_ISL_5052050, EPI_ISL_5052051, EPI_ISL_5052052, EPI_ISL_5052055, EPI_ISL_5052056, EPI_ISL_5052057, EPI_ISL_5052058, EPI_ISL_5052059, EPI_ISL_5052060, EPI_ISL_5052061, EPI_ISL_5052062, EPI_ISL_5052063, EPI_ISL_5052064, EPI_ISL_5052107, EPI_ISL_5893851, EPI_ISL_5895219, EPI_ISL_5895220, EPI_ISL_5895221, EPI_ISL_5895222, EPI_ISL_5895223			
see above	Sarawak General Hospital (Kuching)	Institute of Health and Community Medicine	Chan Chia Jui; Chua Hock Hin; David Perera; Ooi Mong How; Tonnii Sia Loong Loong; Wong Jyn Shan; Wong Kiing Aik
EPI_ISL_6970044, EPI_ISL_6970059, EPI_ISL_6970071, EPI_ISL_6970077, EPI_ISL_6970091, EPI_ISL_6970097, EPI_ISL_6970103, EPI_ISL_6970111, EPI_ISL_6970116, EPI_ISL_6970119, EPI_ISL_6970125, EPI_ISL_6970128, EPI_ISL_6970132, EPI_ISL_6970138, EPI_ISL_6970140, EPI_ISL_6970149, EPI_ISL_6970155, EPI_ISL_6970161, EPI_ISL_6970169, EPI_ISL_6970174, EPI_ISL_6970177, EPI_ISL_6970183, EPI_ISL_6970191, EPI_ISL_6970194, EPI_ISL_6970201, EPI_ISL_6970202, EPI_ISL_6970208, EPI_ISL_6970216, EPI_ISL_6970222, EPI_ISL_6970227, EPI_ISL_6970234, EPI_ISL_6970419			
see above	Sarawak Heart Centre (SHC)	Institute of Health and Community Medicine	Chan Chia Jui; Chua Hock Hin; David Perera; Ooi Mong How; Tonnii Sia Loong Loong; Wong Jyn Shan
EPI_ISL_5051757, EPI_ISL_5051988, EPI_ISL_5051989, EPI_ISL_5051990, EPI_ISL_5051991, EPI_ISL_5051992, EPI_ISL_5052008, EPI_ISL_5052009, EPI_ISL_5052010, EPI_ISL_5052011, EPI_ISL_5052012			
see above	Sarawak Heart Centre (SHC), Kota Samarahan	Institute of Health and Community Medicine	Chan Chia Jui; Chua Hock Hin; David Perera; Ooi Mong How; Tonnii Sia Loong Loong; Wong Jyn Shan
EPI_ISL_5395714, EPI_ISL_6774015, EPI_ISL_6774043	Sungai Buloh Hospital	Institute for Medical Research, Infectious Disease Research Centre, National Institutes of Health, Ministry of Health Malaysia	Ahmad FA; Ahmad Fazilah NA; Anasir MI; Azizan MA; Kamel K; Mohamad Sukri MZ; Mohd Zawawi Z; Norhisham SN; Ramly N; Robert F; Rosli NR; Suppiah J; Thayan R
EPI_ISL_5395821, EPI_ISL_5395825, EPI_ISL_5395830	Tawau Area Health Office	Institute for Medical Research, Infectious Disease Research Centre, National Institutes of Health, Ministry of Health Malaysia	Ahmad FA; Ahmad Fazilah NA; Anasir MI; Azizan MA; Kamel K; Mohd Zawawi Z; Norhisham SN; Ramly N; Robert F; Suppiah J; Thayan R
EPI_ISL_6774048	Tengku Ampuan Jemaah Hospital	Institute for Medical Research, Infectious Disease Research Centre, National Institutes of Health, Ministry of Health Malaysia	Ahmad FA; Ahmad Fazilah NA; Anasir MI; Azizan MA; Kamel K; Mohamad Sukri MZ; Mohd Zawawi Z; Norhisham SN; Ramly N; Robert F; Rosli NR; Suppiah J; Thayan R
EPI_ISL_8090662	UKKP HOSPITAL KULIM	Tropical Infectious Diseases Research & Education Centre (TIDREC), Universiti Malaya	AsmaAnati CheMatSeri; Che-Norainon Yaacob; Jia-Yi Tan; Jo-Ern Wong; Kim-Kee Tan; Mulya-Mustika-Sari Zulkifli; Noor Syahida Azizan; Nur-Hidayana Mahfodz; Sazaly AbuBakar; Siti-Sarah Nor'e
EPI_ISL_8090660	UKKP HOSPITAL SULTANAH BAHIYAH	Tropical Infectious Diseases Research & Education Centre (TIDREC), Universiti Malaya	AsmaAnati CheMatSeri; Che-Norainon Yaacob; Jia-Yi Tan; Jo-Ern Wong; Kim-Kee Tan; Mulya-Mustika-Sari Zulkifli; Noor Syahida Azizan; Nur-Hidayana Mahfodz; Sazaly AbuBakar; Siti-Sarah Nor'e
EPI_ISL_8090628, EPI_ISL_8090659	UKKP, HOSPITAL SULTANAH BAHIYAH	Tropical Infectious Diseases Research & Education Centre (TIDREC), Universiti Malaya	AsmaAnati CheMatSeri; Che-Norainon Yaacob; Jia-Yi Tan; Jo-Ern Wong; Kim-Kee Tan; Mulya-Mustika-Sari Zulkifli; Noor Syahida Azizan; Nur-Hidayana Mahfodz; Sazaly AbuBakar; Siti-Sarah Nor'e
EPI_ISL_6132000	UMMC	Department of Medical Microbiology, Faculty of Medicine, University of Malaya; University of Malaya Medical Centre	I-Ching SAM; Jolene Yin Ling FU; Omar Khalilur Rahman; Yoke Fun Chan
EPI_ISL_5058548	UNIT KESIHATAN AWAM	UKM Medical Molecular Biology Institute (UMBI)	Mira Farzana binti Mohamad Mokhtar
EPI_ISL_4731210, EPI_ISL_4891933, EPI_ISL_5022674, EPI_ISL_5022678, EPI_ISL_5022680, EPI_ISL_5058545, EPI_ISL_5058551, EPI_ISL_5058553, EPI_ISL_5058554, EPI_ISL_5417598, EPI_ISL_5417599, EPI_ISL_5417600, EPI_ISL_5417601			
see above	UNIT KESIHATAN AWAM HEBHK	UKM Medical Molecular Biology Institute (UMBI)	Mira Farzana binti Mohamad Mokhtar
EPI_ISL_5365869	UNIT KESIHATAN AWAM HPSF	UKM Medical Molecular Biology Institute (UMBI)	Mira Farzana binti Mohamad Mokhtar
EPI_ISL_5365865	UNIT KPAS, PEJABAT KESIHATAN MELAKA TENGAH	UKM Medical Molecular Biology Institute (UMBI)	Mira Farzana binti Mohamad Mokhtar
EPI_ISL_4731189, EPI_ISL_4731198, EPI_ISL_4731200, EPI_ISL_4731213, EPI_ISL_4731214, EPI_ISL_4731221, EPI_ISL_5365864, EPI_ISL_5365866, EPI_ISL_5365868			
see above	UNIT KPAS, PKD MELAKA TENGAH	UKM Medical Molecular Biology Institute (UMBI)	Mira Farzana binti Mohamad Mokhtar
EPI_ISL_5450634	UNIT PERUBATAN FORENSIK, HOSPITAL SEBERANG JAYA	iPROMISE, UiTM	Ariza Adnan; Fadzilah Mohd Nor; Lim Wai Feng; Mohd Asif Mohd Sukri; Mohd Nur Fakhruzzaman Noorizhab; Mohd Zaki Salleh; Sazzli Shahlan Kassim; Siti Farah Alwani Mohd Nawi; Siti Hamimah Sheikh Abdul Kadir; Teh Lay Kek; Wang Seok Mui
EPI_ISL_4740362, EPI_ISL_4740365	Unit Forensik, Hospital Seberang Jaya	iPROMISE, UiTM	Ariza Adnan; Fadzilah Mohd Nor; Lim Wai Feng; Mohd Asif Mohd Sukri; Mohd Nur Fakhruzzaman Noorizhab; Mohd Zaki Salleh; Sazzli Shahlan Kassim; Siti Farah Alwani Mohd Nawi; Siti Hamimah Sheikh Abdul Kadir; Teh Lay Kek; Wang Seok Mui
EPI_ISL_4740403	Unit Perubatan Forensik, Hospital Kepala Batas	iPROMISE, UiTM	Ariza Adnan; Fadzilah Mohd Nor; Lim Wai Feng; Mohd Asif Mohd Sukri; Mohd Nur Fakhruzzaman Noorizhab; Mohd Zaki Salleh; Sazzli Shahlan Kassim; Siti Farah Alwani Mohd Nawi; Siti Hamimah Sheikh Abdul Kadir; Teh Lay Kek; Wang Seok Mui
EPI_ISL_4740408	Unit forensik	iPROMISE, UiTM	Ariza Adnan; Fadzilah Mohd Nor; Lim Wai Feng; Mohd Asif Mohd Sukri; Mohd Nur Fakhruzzaman Noorizhab; Mohd Zaki Salleh; Sazzli Shahlan Kassim; Siti Farah Alwani Mohd Nawi; Siti Hamimah Sheikh Abdul Kadir; Teh Lay Kek; Wang Seok Mui
EPI_ISL_5365870	WARD 6	UKM Medical Molecular Biology Institute (UMBI)	Mira Farzana binti Mohamad Mokhtar
EPI_ISL_8090631	Wad Melati, HRPZ	Tropical Infectious Diseases Research & Education Centre (TIDREC), Universiti Malaya	AsmaAnati CheMatSeri; Che-Norainon Yaacob; Jia-Yi Tan; Jo-Ern Wong; Kim-Kee Tan; Mulya-Mustika-Sari Zulkifli; Noor Syahida Azizan; Nur-Hidayana Mahfodz; Sazaly AbuBakar; Siti-Sarah Nor'e
EPI_ISL_5058546	hospital sultanah nora ismail	UKM Medical Molecular Biology Institute (UMBI)	Mira Farzana binti Mohamad Mokhtar
EPI_ISL_5261902, EPI_ISL_5261903, EPI_ISL_5261904, EPI_ISL_5261905, EPI_ISL_5261907, EPI_ISL_5261908	iPROMISE, UiTM	iPROMISE, UiTM	Ariza Adnan; Fadzilah Mohd Nor; Lim Wai Feng; Mohd Asif Mohd Sukri; Mohd Nur Fakhruzzaman Noorizhab; Mohd Zaki Salleh; Sazzli Shahlan Kassim; Siti Farah Alwani Mohd Nawi; Siti Hamimah Sheikh Abdul Kadir; Teh Lay Kek; Wang Seok Mui
EPI_ISL_5417633	icu hosp tuanku jaafar seremban	UKM Medical Molecular Biology Institute (UMBI)	Mira Farzana binti Mohamad Mokhtar
EPI_ISL_4731195	icu hospita tuanku jaafar	UKM Medical Molecular Biology Institute (UMBI)	Mira Farzana binti Mohamad Mokhtar

We gratefully acknowledge the following Authors from the Originating laboratories responsible for obtaining the specimens, as well as the Submitting laboratories where the genome data were generated and shared via GISAID, on which this research is based.

All Submitters of data may be contacted directly via www.gisaid.org

Authors are sorted alphabetically.

Accession ID	Originating Laboratory	Submitting Laboratory	Authors
EPI_ISL_5395640, EPI_ISL_6773992	Ampang Hospital	Institute for Medical Research, Infectious Disease Research Centre, National Institutes of Health, Ministry of Health Malaysia	Ahmad FA; Ahmad Fazilah NA; Anasir MI; Azizan MA; Kamel K; Mohamad Sukri MZ; Mohd Zawawi Z; Norhisham SN; Ramly N; Robert F; Rosli NR; Suppiah J; Thayan R
EPI_ISL_5396303, EPI_ISL_5396348	Banting Hospital	Institute for Medical Research, Infectious Disease Research Centre, National Institutes of Health, Ministry of Health Malaysia	Ahmad FA; Ahmad Fazilah NA; Anasir MI; Azizan MA; Kamel K; Mohd Zawawi Z; Norhisham SN; Ramly N; Robert F; Suppiah J; Thayan R
EPI_ISL_5893758, EPI_ISL_5893765, EPI_ISL_5893816, EPI_ISL_5893901, EPI_ISL_5893909, EPI_ISL_5893918, EPI_ISL_5893924, EPI_ISL_5893932, EPI_ISL_5893939, EPI_ISL_5893950, EPI_ISL_5893955, EPI_ISL_5894102, EPI_ISL_5894339, EPI_ISL_5894348, EPI_ISL_5894355, EPI_ISL_5894361, EPI_ISL_5894366, EPI_ISL_5894377, EPI_ISL_5894381, EPI_ISL_5894388, EPI_ISL_5894396, EPI_ISL_5894409, EPI_ISL_5894416, EPI_ISL_5894425, EPI_ISL_5894430, EPI_ISL_5894437, EPI_ISL_5894447, EPI_ISL_5894454, EPI_ISL_5894458, EPI_ISL_5894467, EPI_ISL_5894472, EPI_ISL_5894477, EPI_ISL_5894486, EPI_ISL_5894495, EPI_ISL_5896264, EPI_ISL_8782808			
see above	Bintulu Hospital PCR Lab, Bintulu	Institute of Health and Community Medicine	Chan Chia Jui; Chua Hock Hin; David Perera; Ooi Mong How; Tan Lee See; Tonnii Sia Loong Loong; Wong Jyn Shan
EPI_ISL_6586358, EPI_ISL_6586359, EPI_ISL_6968042, EPI_ISL_6968254, EPI_ISL_6968260, EPI_ISL_6969157, EPI_ISL_6970387, EPI_ISL_6970388, EPI_ISL_6970389, EPI_ISL_6970390, EPI_ISL_6970391, EPI_ISL_6970392, EPI_ISL_6970393, EPI_ISL_6970394, EPI_ISL_6970395, EPI_ISL_6970396, EPI_ISL_6970397, EPI_ISL_6970398, EPI_ISL_6970399, EPI_ISL_6970400, EPI_ISL_6970401, EPI_ISL_6970402, EPI_ISL_6970403, EPI_ISL_6970418			
see above	Bintulu Medical Centre (Bintulu)	Institute of Health and Community Medicine	Chan Chia Jui; Chua Hock Hin; David Perera; Ooi Mong How; Tonnii Sia Loong Loong; Wong Jyn Shan
EPI_ISL_5894842, EPI_ISL_5894850, EPI_ISL_5894857, EPI_ISL_5894868, EPI_ISL_5894877, EPI_ISL_5894890, EPI_ISL_5894893, EPI_ISL_5894904, EPI_ISL_5894915, EPI_ISL_5894923, EPI_ISL_5894926, EPI_ISL_5894933, EPI_ISL_5894943, EPI_ISL_5894953, EPI_ISL_5896119, EPI_ISL_5896128			
see above	Borneo Medical Centre (Kuching)	Institute of Health and Community Medicine	Chan Chia Jui; Chua Hock Hin; David Perera; Ooi Mong How; Tonnii Sia Loong Loong; Wong Jyn Shan
EPI_ISL_5450470	CAC ILKKM	iPROMISE, UiTM	Ariza Adnan; Fadzilah Mohd Nor; Lim Wai Feng; Mohd Asif Mohd Sukri; Mohd Nur Fakhruzzaman Noorizhab; Mohd Zaki Salleh; Sazzli Shahlan Kassim; Siti Farah Alwani Mohd Nawi; Siti Hamimah Sheikh Abdul Kadir; Teh Lay Kek; Wang Seok Mui
EPI_ISL_5417143, EPI_ISL_5417169, EPI_ISL_5417186	CPRC JKNT	Department of Medical Microbiology, University Malaya Medical Centre	I-Ching SAM; Jolene Yin Ling FU; Omar Khalilur Rahman; Yoke Fun Chan
EPI_ISL_8090604, EPI_ISL_8090605	CPRC JKNT	Tropical Infectious Diseases Research & Education Centre (TIDREC), Universiti Malaya	AsmaAnati CheMatSeri; Che-Norainon Yaacob; Jia-Yi Tan; Jo-Ern Wong; Kim-Kee Tan; Mulya-Mustika-Sari Zulkifli; Noor Syahida Azizan; Nur-Hidayana Mahfodz; Sazaly AbuBakar; Siti-Sarah Nor’e
EPI_ISL_6132034	CPRC PKD PASIR MAS	Department of Medical Microbiology, Faculty of Medicine, University of Malaya; University of Malaya Medical Centre	I-Ching SAM; Jolene Yin Ling FU; Omar Khalilur Rahman; Yoke Fun Chan
EPI_ISL_6586457, EPI_ISL_6586460, EPI_ISL_6586462, EPI_ISL_6586464, EPI_ISL_6586465, EPI_ISL_6586466, EPI_ISL_6586467, EPI_ISL_6586468, EPI_ISL_6586471, EPI_ISL_6586472, EPI_ISL_6586473, EPI_ISL_6586476, EPI_ISL_6586478			
see above	Clinical Research Centre (CRC), Sibu Hospital, Sibu	Institute of Health and Community Medicine	Chan Chia Jui; Chua Hock Hin; David Perera; Ooi Mong How; Tonnii Sia Loong Loong; Wong Jyn Shan
EPI_ISL_5396281, EPI_ISL_5396283	Department of Forensic Medicine, Hospital Queen Elizabeth	Institute for Medical Research, Infectious Disease Research Centre, National Institutes of Health, Ministry of Health Malaysia	Ahmad FA; Ahmad Fazilah NA; Anasir MI; Azizan MA; Kamel K; Mohd Zawawi Z; Norhisham SN; Ramly N; Robert F; Suppiah J; Thayan R
EPI_ISL_5417132, EPI_ISL_5417133, EPI_ISL_5417134, EPI_ISL_5417135, EPI_ISL_5417145, EPI_ISL_5417147, EPI_ISL_5417166, EPI_ISL_5417173			
see above	Department of Medical Microbiology, University Malaya Medical Centre	Department of Medical Microbiology, University Malaya Medical Centre	I-Ching SAM; Jolene Yin Ling FU; Omar Khalilur Rahman; Yoke Fun Chan
EPI_ISL_5804695, EPI_ISL_5804697	Duchess of Kent Hospital, Sandakan	Institute for Medical Research, Infectious Disease Research Centre, National Institutes of Health, Ministry of Health Malaysia	Ahmad FA; Ahmad Fazilah NA; Anasir MI; Azizan MA; Kamel K; Mohd Zawawi Z; Norhisham SN; Ramly N; Robert F; Suppiah J; Thayan R
EPI_ISL_5396295	Forensic Unit, Keningau Hospital	Institute for Medical Research, Infectious Disease Research Centre, National Institutes of Health, Ministry of Health Malaysia	Ahmad FA; Ahmad Fazilah NA; Anasir MI; Azizan MA; Kamel K; Mohd Zawawi Z; Norhisham SN; Ramly N; Robert F; Suppiah J; Thayan R
EPI_ISL_5052065, EPI_ISL_5052066, EPI_ISL_5052067, EPI_ISL_5052068, EPI_ISL_5893717, EPI_ISL_5894334, EPI_ISL_6970291			
see above	Gribbles Pathology, Kuching	Institute of Health and Community Medicine	Chan Chia Jui; Chua Hock Hin; David Perera; Ooi Mong How; Reagan Entigu Linton; Tonnii Sia Loong Loong; Wong Jyn Shan
EPI_ISL_5450221, EPI_ISL_5450232, EPI_ISL_5450239, EPI_ISL_5450247	HOSPITAL BALIK PULAU	iPROMISE, UiTM	Ariza Adnan; Fadzilah Mohd Nor; Lim Wai Feng; Mohd Asif Mohd Sukri; Mohd Nur Fakhruzzaman Noorizhab; Mohd Zaki Salleh; Sazzli Shahlan Kassim; Siti Farah Alwani Mohd Nawi; Siti Hamimah Sheikh Abdul Kadir; Teh Lay Kek; Wang Seok Mui
EPI_ISL_5450418	HOSPITAL BUKIT MERTAJAM	iPROMISE, UiTM	Ariza Adnan; Fadzilah Mohd Nor; Lim Wai Feng; Mohd Asif Mohd Sukri; Mohd Nur Fakhruzzaman Noorizhab; Mohd Zaki Salleh; Sazzli Shahlan Kassim; Siti Farah Alwani Mohd Nawi; Siti Hamimah Sheikh Abdul Kadir; Teh Lay Kek; Wang Seok Mui
EPI_ISL_5450119	HOSPITAL KEPALA BATAS	iPROMISE, UiTM	Ariza Adnan; Fadzilah Mohd Nor; Lim Wai Feng; Mohd Asif Mohd Sukri; Mohd Nur Fakhruzzaman Noorizhab; Mohd Zaki Salleh; Sazzli Shahlan Kassim; Siti Farah Alwani Mohd Nawi; Siti Hamimah Sheikh Abdul Kadir; Teh Lay Kek; Wang Seok Mui
EPI_ISL_5417632	HOSPITAL SULTANAH NORA ISMAIL	UKM Medical Molecular Biology Institute (UMBI)	Mira Farzana binti Mohamad Mokhtar
EPI_ISL_5058534	HOSPITAL TUANKU AMPUAN NAJIHAH	UKM Medical Molecular Biology Institute (UMBI)	Mira Farzana binti Mohamad Mokhtar
EPI_ISL_6131992, EPI_ISL_6132041	HRPB	Department of Medical Microbiology, Faculty of Medicine, University of Malaya; University of Malaya Medical Centre	I-Ching SAM; Jolene Yin Ling FU; Omar Khalilur Rahman; Yoke Fun Chan
EPI_ISL_8090635, EPI_ISL_8090637, EPI_ISL_8090640, EPI_ISL_8090666, EPI_ISL_8090668, EPI_ISL_8090669, EPI_ISL_8090670, EPI_ISL_8090672, EPI_ISL_8090674, EPI_ISL_8090677, EPI_ISL_8090678, EPI_ISL_8090679, EPI_ISL_8090681, EPI_ISL_8090683, EPI_ISL_8090684			
see above	HRPB	Tropical Infectious Diseases Research & Education Centre (TIDREC), Universiti Malaya	AsmaAnati CheMatSeri; Che-Norainon Yaacob; Jia-Yi Tan; Jo-Ern Wong; Kim-Kee Tan; Mulya-Mustika-Sari Zulkifli; Noor Syahida Azizan; Nur-Hidayana Mahfodz; Sazaly AbuBakar; Siti-Sarah Nor’e
EPI_ISL_6825260	Hospital Ampang	Malaysia Genome Institute	Azrin Ahmad; Enizza Kasim; Irni Suhayu Sapian; Mohd Faizal Abu Bakar; Mohd Ghows Mohd Azzam.; Mohd Noor Mat Isa; Nor Azfa Johari; Nurhezreen Md Iqbal; Shamsidar Sopie; Siti Noraini Othman; Yusuf Muhammad Noor
EPI_ISL_6017872	Hospital Bukit Mertajam	iPROMISE, UiTM	Ariza Adnan; Fadzilah Mohd Nor; Lim Wai Feng; Mohd Asif Mohd Sukri; Mohd Nur Fakhruzzaman Noorizhab; Mohd Zaki Salleh; Sazzli Shahlan Kassim; Siti Farah Alwani Mohd Nawi; Siti Hamimah Sheikh Abdul Kadir; Teh Lay Kek; Wang Seok Mui
EPI_ISL_5159363	Hospital Canselor Tuanku Muhriz UKM	UKM Medical Molecular Biology Institute (UMBI)	Mira Farzana binti Mohamad Mokhtar
EPI_ISL_6825243, EPI_ISL_6825244, EPI_ISL_6825262	Hospital Duchess Of Kent	Malaysia Genome Institute	Azrin Ahmad; Enizza Kasim; Irni Suhayu Sapian; Mohd Faizal Abu Bakar; Mohd Ghows Mohd Azzam.; Mohd Noor Mat Isa; Nor Azfa Johari; Nurhezreen Md Iqbal; Shamsidar Sopie; Siti Noraini Othman; Yusuf Muhammad Noor
EPI_ISL_8745569, EPI_ISL_8745573	Hospital Duchess Of Kent	Malaysia Genome and Vaccine Institute	Apical Scientific Sdn Bhd's Team; Azrin Ahmad; Carey Wee Kai Li; Enizza Kasim; Irni Suhayu Sapian; Jilian Michelle Wong Tzeling; Leong Wai Mun; Mohd Faizal Abu Bakar; Mohd Ghows Mohd Azzam.; Mohd Noor Mat Isa; Nor Azfa Johari; Nurhezreen Md Iqbal; Rose Iszati Ismet Nayan; Shamsidar Sopie; Siti Noraini Othman; Yusuf Muhammad Noor
EPI_ISL_5536446, EPI_ISL_6825252	Hospital Keningau	Malaysia Genome Institute	Azrin Ahmad; Enizza Kasim; Irni Suhayu Sapian; Mohd Faizal Abu Bakar; Mohd Ghows Mohd Azzam.; Mohd Noor Mat Isa; Nor Azfa Johari; Nurhezreen Md Iqbal; Shamsidar Sopie; Siti Noraini Othman; Yusuf Muhammad Noor
EPI_ISL_5536447	Hospital Kota Marudu	Malaysia Genome Institute	Azrin Ahmad; Enizza Kasim; Irni Suhayu Sapian; Mohd Faizal Abu Bakar; Mohd Ghows Mohd Azzam.; Mohd Noor Mat Isa; Nor Azfa Johari; Nurhezreen Md Iqbal; Shamsidar Sopie; Siti Noraini Othman; Yusuf Muhammad Noor
EPI_ISL_5536448, EPI_ISL_5536455, EPI_ISL_5583912	Hospital Kudat	Malaysia Genome Institute	Azrin Ahmad; Enizza Kasim; Irni Suhayu Sapian; Mohd Faizal Abu Bakar; Mohd Ghows Mohd Azzam.; Mohd Noor Mat Isa; Nor Azfa Johari; Nurhezreen Md Iqbal; Shamsidar Sopie; Siti Noraini Othman; Yusuf Muhammad Noor
EPI_ISL_8745580	Hospital Kunak	Malaysia Genome and Vaccine Institute	Apical Scientific Sdn Bhd's Team; Azrin Ahmad; Carey Wee Kai Li; Enizza Kasim; Irni Suhayu Sapian; Jilian Michelle Wong Tzeling; Leong Wai Mun; Mohd Faizal Abu Bakar; Mohd Ghows Mohd Azzam.; Mohd Noor Mat Isa; Nor Azfa Johari; Nurhezreen Md Iqbal; Rose Iszati Ismet Nayan; Shamsidar Sopie; Siti Noraini Othman; Yusuf Muhammad Noor
EPI_ISL_6825236, EPI_ISL_6825238	Hospital Lahad Datu	Malaysia Genome Institute	Azrin Ahmad; Enizza Kasim; Irni Suhayu Sapian; Mohd Faizal Abu Bakar; Mohd Ghows Mohd Azzam.; Mohd Noor Mat Isa; Nor Azfa Johari; Nurhezreen Md Iqbal; Shamsidar Sopie; Siti Noraini Othman; Yusuf Muhammad Noor
EPI_ISL_8745571, EPI_ISL_8745584	Hospital Lahad Datu	Malaysia Genome and Vaccine Institute	Apical Scientific Sdn Bhd's Team; Azrin Ahmad; Carey Wee Kai Li; Enizza Kasim; Irni Suhayu Sapian; Jilian Michelle Wong Tzeling; Leong Wai Mun; Mohd Faizal Abu Bakar; Mohd Ghows Mohd Azzam.; Mohd Noor Mat Isa; Nor Azfa Johari; Nurhezreen Md Iqbal; Rose Iszati Ismet Nayan; Shamsidar Sopie; Siti Noraini Othman; Yusuf Muhammad Noor
EPI_ISL_5450321, EPI_ISL_5450326, EPI_ISL_5450332, EPI_ISL_5450337, EPI_ISL_5450344	Hospital Pulau Pinang	iPROMISE, UiTM	Ariza Adnan; Fadzilah Mohd Nor; Lim Wai Feng; Mohd Asif Mohd Sukri; Mohd Nur Fakhruzzaman Noorizhab; Mohd Zaki Salleh; Sazzli Shahlan Kassim; Siti Farah Alwani Mohd Nawi; Siti Hamimah Sheikh Abdul Kadir; Teh Lay Kek; Wang Seok Mui
EPI_ISL_6825274	Hospital Putrajaya	Malaysia Genome Institute	Azrin Ahmad; Enizza Kasim; Irni Suhayu Sapian; Mohd Faizal Abu Bakar; Mohd Ghows Mohd Azzam.; Mohd Noor Mat Isa; Nor Azfa Johari; Nurhezreen Md Iqbal; Shamsidar Sopie; Siti Noraini Othman; Yusuf Muhammad Noor
EPI_ISL_8745577	Hospital Putrajaya	Malaysia Genome and Vaccine Institute	Apical Scientific Sdn Bhd's Team; Azrin Ahmad; Carey Wee Kai Li; Enizza Kasim; Irni Suhayu Sapian; Jilian Michelle Wong Tzeling; Leong Wai Mun; Mohd Faizal Abu Bakar; Mohd Ghows Mohd Azzam.; Mohd Noor Mat Isa; Nor Azfa Johari; Nurhezreen Md Iqbal; Rose Iszati Ismet Nayan; Shamsidar Sopie; Siti Noraini Othman; Yusuf Muhammad Noor
EPI_ISL_5417123, EPI_ISL_5417125, EPI_ISL_5417148	Hospital Raja Perempuan Zainab II	Department of Medical Microbiology, University Malaya Medical Centre	I-Ching SAM; Jolene Yin Ling FU; Omar Khalilur Rahman; Yoke Fun Chan
EPI_ISL_6825258	Hospital Sungai Buloh	Malaysia Genome Institute	Azrin Ahmad; Enizza Kasim; Irni Suhayu Sapian; Mohd Faizal Abu Bakar; Mohd Ghows Mohd Azzam.; Mohd Noor Mat Isa; Nor Azfa Johari; Nurhezreen Md Iqbal; Shamsidar Sopie; Siti Noraini Othman; Yusuf Muhammad Noor
EPI_ISL_5536441, EPI_ISL_5536443, EPI_ISL_5536444	Hospital Thomson Kota Damansara	Malaysia Genome Institute	Azrin Ahmad; Enizza Kasim; Irni Suhayu Sapian; Mohd Faizal Abu Bakar; Mohd Ghows Mohd Azzam.; Mohd Noor Mat Isa; Nor Azfa Johari; Nurhezreen Md Iqbal; Shamsidar Sopie; Siti Noraini Othman; Yusuf Muhammad Noor

EPI_ISL_5417183	Hospital Tuanku Fauziah	Department of Medical Microbiology, University Malaya Medical Centre	I-Ching SAM; Jolene Yin Ling FU; Omar Khalilur Rahman; Yoke Fun Chan
EPI_ISL_6132009	Hospital tumpat	Department of Medical Microbiology, Faculty of Medicine, University of Malaya; University of Malaya Medical Centre	I-Ching SAM; Jolene Yin Ling FU; Omar Khalilur Rahman; Yoke Fun Chan
EPI_ISL_4891938, EPI_ISL_4891939, EPI_ISL_4891940, EPI_ISL_4891941, EPI_ISL_4891942, EPI_ISL_4891946	INSTITUT BIOLOGI MOLEKUL PERUBATAN UKM (UMBI)	UKM Medical Molecular Biology Institute (UMBI)	Mira Farzana binti Mohamad Mokhtar
EPI_ISL_6960089, EPI_ISL_6960090, EPI_ISL_6960091, EPI_ISL_6960092, EPI_ISL_6960093, EPI_ISL_6960094, EPI_ISL_6960095, EPI_ISL_6960096, EPI_ISL_11078701, EPI_ISL_11078702, EPI_ISL_11078714, EPI_ISL_11078754, EPI_ISL_11078773, EPI_ISL_11078774, EPI_ISL_11078780, EPI_ISL_11078781, EPI_ISL_11078787			
see above	Institut Biologi Molekul Perubatan UKM (UMBI)	UKM Medical Molecular Biology Institute (UMBI)	Khairun Nur Abd Ghafar; Mira Farzana Mohamad Mokhtar; Mira Farzana binti Mohamad Mokhtar; Muhiddin Ishak; Nor Azila Muhammad Azami; Nur Alyaa Afifah Md Shahri; Nurul Syakima Ab Mutalib; Rahman Jamal; Ryia Illani Mohd Yunos; Siti Nur Hasanah Mohd Yusuf; Zahirrah Begam Mohamed Rasheed
EPI_ISL_5450317	JABATAN FORENSIC HOSPITAL PULAU PINANG	iPROMISE, UiTM	Ariza Adnan; Fadzilah Mohd Nor; Lim Wai Feng; Mohd Asif Mohd Sukri; Mohd Nur Fakhruzzaman Noorizhab; Mohd Zaki Salleh; Sazzli Shahlan Kassim; Siti Farah Alwani Mohd Nawi; Siti Hamimah Sheikh Abdul Kadir; Teh Lay Kek; Wang Seok Mui
EPI_ISL_6017886	JABATAN FORENSIK HPP	iPROMISE, UiTM	Ariza Adnan; Fadzilah Mohd Nor; Lim Wai Feng; Mohd Asif Mohd Sukri; Mohd Nur Fakhruzzaman Noorizhab; Mohd Zaki Salleh; Sazzli Shahlan Kassim; Siti Farah Alwani Mohd Nawi; Siti Hamimah Sheikh Abdul Kadir; Teh Lay Kek; Wang Seok Mui
EPI_ISL_5417167	JABATAN PATOLOGI	Department of Medical Microbiology, University Malaya Medical Centre	I-Ching SAM; Jolene Yin Ling FU; Omar Khalilur Rahman; Yoke Fun Chan
EPI_ISL_5417122, EPI_ISL_5417141, EPI_ISL_5417144	JABATAN PATOLOGI, HRPZ II	Department of Medical Microbiology, University Malaya Medical Centre	I-Ching SAM; Jolene Yin Ling FU; Omar Khalilur Rahman; Yoke Fun Chan
EPI_ISL_6132063, EPI_ISL_7087903	JABATAN PERUBATAN FORENSIK HOSPITAL SULTANAH BAHIYAH	Department of Medical Microbiology, Faculty of Medicine, University of Malaya; University of Malaya Medical Centre	I-Ching SAM; Jolene Yin Ling FU; Omar Khalilur Rahman; Yoke Fun Chan
EPI_ISL_5058536, EPI_ISL_5159369	JABATAN PERUBATAN FORENSIK, HOSPITAL TUANKU JA'AFAR SEREMBAN	UKM Medical Molecular Biology Institute (UMBI)	Mira Farzana binti Mohamad Mokhtar
EPI_ISL_5450528, EPI_ISL_5450613, EPI_ISL_5450629, EPI_ISL_6017973, EPI_ISL_6017999, EPI_ISL_6018009, EPI_ISL_6018016, EPI_ISL_6018024, EPI_ISL_6712898			
see above	JKN PAHANG	iPROMISE, UiTM	Ariza Adnan; Fadzilah Mohd Nor; Lim Wai Feng; Mohd Asif Mohd Sukri; Mohd Nur Fakhruzzaman Noorizhab; Mohd Zaki Salleh; Sazzli Shahlan Kassim; Siti Farah Alwani Mohd Nawi; Siti Hamimah Sheikh Abdul Kadir; Teh Lay Kek; Wang Seok Mui
EPI_ISL_8090658	Jabatan Forensik Hospital Sultanah Bahiyah	Tropical Infectious Diseases Research & Education Centre (TIDREC), Universiti Malaya	AsmaAnati CheMatSeri; Che-Norainon Yaacob; Jia-Yi Tan; Jo-Ern Wong; Kim-Kee Tan; Mulya-Mustika-Sari Zulkifli; Noor Syahida Azizan; Nur-Hidayana Mahfodz; Sazaly AbuBakar; Siti-Sarah Nor'e
EPI_ISL_5450577	Jabatan Kesihatan Negeri Perlis	iPROMISE, UiTM	Ariza Adnan; Fadzilah Mohd Nor; Lim Wai Feng; Mohd Asif Mohd Sukri; Mohd Nur Fakhruzzaman Noorizhab; Mohd Zaki Salleh; Sazzli Shahlan Kassim; Siti Farah Alwani Mohd Nawi; Siti Hamimah Sheikh Abdul Kadir; Teh Lay Kek; Wang Seok Mui
EPI_ISL_6825251, EPI_ISL_6825261	Jabatan Kesihatan WP Labuan	Malaysia Genome Institute	Azrin Ahmad; Enizza Kasim; Irni Suhayu Sapian; Mohd Faizal Abu Bakar; Mohd Ghows Mohd Azzam.; Mohd Noor Mat Isa; Nor Azfa Johari; Nurhezreen Md Iqbal; Shamsidar Sopie; Siti Noraini Othman; Yusuf Muhammad Noor
EPI_ISL_8745576	Jabatan Kesihatan WP Labuan	Malaysia Genome and Vaccine Institute	Apical Scientific Sdn Bhd's Team; Azrin Ahmad; Carey Wee Kai Li; Enizza Kasim; Irni Suhayu Sapian; Jilian Michelle Wong Tzeling; Leong Wai Mun; Mohd Faizal Abu Bakar; Mohd Ghows Mohd Azzam.; Mohd Noor Mat Isa; Nor Azfa Johari; Nurhezreen Md Iqbal; Rose Iszati Ismet Nayan; Shamsidar Sopie; Siti Noraini Othman; Yusuf Muhammad Noor
EPI_ISL_5417120	Jabatan Patologi HRPZ II	Department of Medical Microbiology, University Malaya Medical Centre	I-Ching SAM; Jolene Yin Ling FU; Omar Khalilur Rahman; Yoke Fun Chan
EPI_ISL_5417184	Jabatan Patologi, Hospital Raja Perempuan Zainab II	Department of Medical Microbiology, University Malaya Medical Centre	I-Ching SAM; Jolene Yin Ling FU; Omar Khalilur Rahman; Yoke Fun Chan
EPI_ISL_5893827	Kapit Hospital PCR Lab, Kapit	Institute of Health and Community Medicine	Chan Chia Jui; Chua Hock Hin; David Perera; Ooi Mong How; Tonnii Sia Loong Loong; Wong Jyn Shan
EPI_ISL_5804692	Kinabatangan Health Office	Institute for Medical Research, Infectious Disease Research Centre, National Institutes of Health, Ministry of Health Malaysia	Ahmad FA; Ahmad Fazilah NA; Anasir MI; Azizan MA; Kamel K; Mohd Zawawi Z; Norhisham SN; Ramly N; Robert F; Suppiah J; Thayan R
EPI_ISL_5096030	Kuala Lumpur General Hospital	Institute for Medical Research, Infectious Disease Research Centre, National Institutes of Health, Ministry of Health Malaysia	Ahmad FA; Ahmad Fazilah NA; Anasir MI; Azizan MA; Kamel K; Mohd Zawawi Z; Norhisham SN; Ramly N; Robert F; Suppiah J; Thayan R
EPI_ISL_5804669	Kuala Penyu District Health Office	Institute for Medical Research, Infectious Disease Research Centre, National Institutes of Health, Ministry of Health Malaysia	Ahmad FA; Ahmad Fazilah NA; Anasir MI; Azizan MA; Kamel K; Mohd Zawawi Z; Norhisham SN; Ramly N; Robert F; Suppiah J; Thayan R
EPI_ISL_6774013	Labuan Hospital	Institute for Medical Research, Infectious Disease Research Centre, National Institutes of Health, Ministry of Health Malaysia	Ahmad FA; Ahmad Fazilah NA; Anasir MI; Azizan MA; Kamel K; Mohamad Sukri MZ; Mohd Zawawi Z; Norhisham SN; Ramly N; Robert F; Rosli NR; Suppiah J; Thayan R
EPI_ISL_5804690, EPI_ISL_5804703, EPI_ISL_6774037, EPI_ISL_6774054	Lahad Datu Hospital	Institute for Medical Research, Infectious Disease Research Centre, National Institutes of Health, Ministry of Health Malaysia	Ahmad FA; Ahmad Fazilah NA; Anasir MI; Azizan MA; Kamel K; Mohamad Sukri MZ; Mohd Zawawi Z; Norhisham SN; Ramly N; Robert F; Rosli NR; Suppiah J; Thayan R
EPI_ISL_5095032	Lembah Pantai Health Office	Institute for Medical Research, Infectious Disease Research Centre, National Institutes of Health, Ministry of Health Malaysia	Ahmad FA; Ahmad Fazilah NA; Anasir MI; Azizan MA; Kamel K; Mohd Zawawi Z; Norhisham SN; Ramly N; Robert F; Suppiah J; Thayan R
EPI_ISL_5051833, EPI_ISL_5051834, EPI_ISL_5051835, EPI_ISL_5052099, EPI_ISL_5894505, EPI_ISL_5894510, EPI_ISL_5894514, EPI_ISL_5894524, EPI_ISL_5894533			
see above	Miri Hospital Molecular Diagnostic Lab, Miri	Institute of Health and Community Medicine	Amir Safuan bin Khamshah; Chan Chia Jui; Chua Hock Hin; David Perera; Ooi Mong How; Tonnii Sia Loong Loong; Wong Jyn Shan
EPI_ISL_5095553	National Institute of Medical Forensic	Institute for Medical Research, Infectious Disease Research Centre, National Institutes of Health, Ministry of Health Malaysia	Ahmad FA; Ahmad Fazilah NA; Anasir MI; Azizan MA; Kamel K; Mohd Zawawi Z; Norhisham SN; Ramly N; Robert F; Suppiah J; Thayan R
EPI_ISL_5022671, EPI_ISL_5058538, EPI_ISL_5058539, EPI_ISL_5058541, EPI_ISL_5417604, EPI_ISL_5417605	PEJABAT KESIHATAN DAERAH MELAKA TENGAH	UKM Medical Molecular Biology Institute (UMBI)	Mira Farzana binti Mohamad Mokhtar
EPI_ISL_6017912, EPI_ISL_6017919	PEJABAT KESIHATAN DAERAH SEBERANG PERAI UTARA PULAU PINANG	iPROMISE, UiTM	Ariza Adnan; Fadzilah Mohd Nor; Lim Wai Feng; Mohd Asif Mohd Sukri; Mohd Nur Fakhruzzaman Noorizhab; Mohd Zaki Salleh; Sazzli Shahlan Kassim; Siti Farah Alwani Mohd Nawi; Siti Hamimah Sheikh Abdul Kadir; Teh Lay Kek; Wang Seok Mui
EPI_ISL_6018046, EPI_ISL_6018051, EPI_ISL_6018067, EPI_ISL_6018072, EPI_ISL_6018078, EPI_ISL_6018085, EPI_ISL_6018088, EPI_ISL_6018092			
see above	PEJABAT KESIHATAN DAERAH TIMUR LAUT	iPROMISE, UiTM	Ariza Adnan; Fadzilah Mohd Nor; Lim Wai Feng; Mohd Asif Mohd Sukri; Mohd Nur Fakhruzzaman Noorizhab; Mohd Zaki Salleh; Sazzli Shahlan Kassim; Siti Farah Alwani Mohd Nawi; Siti Hamimah Sheikh Abdul Kadir; Teh Lay Kek; Wang Seok Mui
EPI_ISL_5450513, EPI_ISL_5450514	PEJABAT KESIHATAN KUANTAN	iPROMISE, UiTM	Ariza Adnan; Fadzilah Mohd Nor; Lim Wai Feng; Mohd Asif Mohd Sukri; Mohd Nur Fakhruzzaman Noorizhab; Mohd Zaki Salleh; Sazzli Shahlan Kassim; Siti Farah Alwani Mohd Nawi; Siti Hamimah Sheikh Abdul Kadir; Teh Lay Kek; Wang Seok Mui
EPI_ISL_5058542, EPI_ISL_5417612, EPI_ISL_5417613	PKD ALOR GAJAH	UKM Medical Molecular Biology Institute (UMBI)	Mira Farzana binti Mohamad Mokhtar
EPI_ISL_5159370, EPI_ISL_5159371, EPI_ISL_5159372	PKD Alor Gajah	UKM Medical Molecular Biology Institute (UMBI)	Mira Farzana binti Mohamad Mokhtar
EPI_ISL_5417606, EPI_ISL_5417607, EPI_ISL_5417608, EPI_ISL_5417609, EPI_ISL_5417610, EPI_ISL_5417635	PKD JEMPOL	UKM Medical Molecular Biology Institute (UMBI)	Mira Farzana binti Mohamad Mokhtar
EPI_ISL_7087317	PKD Manjung	Department of Medical Microbiology, Faculty of Medicine, University of Malaya; University of Malaya Medical Centre	I-Ching SAM; Jolene Yin Ling FU; Omar Khalilur Rahman; Yoke Fun Chan
EPI_ISL_4891944, EPI_ISL_5022684, EPI_ISL_5022685, EPI_ISL_5058535, EPI_ISL_5058537, EPI_ISL_5365860, EPI_ISL_5365863			
see above	PKD TAMPIN	UKM Medical Molecular Biology Institute (UMBI)	Mira Farzana binti Mohamad Mokhtar
EPI_ISL_8090663	Pejabat Kesihatan Daerah Hulu Perak	Tropical Infectious Diseases Research & Education Centre (TIDREC), Universiti Malaya	AsmaAnati CheMatSeri; Che-Norainon Yaacob; Jia-Yi Tan; Jo-Ern Wong; Kim-Kee Tan; Mulya-Mustika-Sari Zulkifli; Noor Syahida Azizan; Nur-Hidayana Mahfodz; Sazaly AbuBakar; Siti-Sarah Nor'e
EPI_ISL_6825246	Pejabat Kesihatan Kawasan Lahad Datu	Malaysia Genome Institute	Azrin Ahmad; Enizza Kasim; Irni Suhayu Sapian; Mohd Faizal Abu Bakar; Mohd Ghows Mohd Azzam.; Mohd Noor Mat Isa; Nor Azfa Johari; Nurhezreen Md Iqbal; Shamsidar Sopie; Siti Noraini Othman; Yusuf Muhammad Noor
EPI_ISL_5450133, EPI_ISL_5450643	Pejabat Kesihatan Pintu Masuk Antarabangsa Pulau Pinang	iPROMISE, UiTM	Ariza Adnan; Fadzilah Mohd Nor; Lim Wai Feng; Mohd Asif Mohd Sukri; Mohd Nur Fakhruzzaman Noorizhab; Mohd Zaki Salleh; Sazzli Shahlan Kassim; Siti Farah Alwani Mohd Nawi; Siti Hamimah Sheikh Abdul Kadir; Teh Lay Kek; Wang Seok Mui
EPI_ISL_5113895, EPI_ISL_5804674, EPI_ISL_5804676, EPI_ISL_5804681, EPI_ISL_5804685, EPI_ISL_5852492	Pitas District Health Office	Institute for Medical Research, Infectious Disease Research Centre, National Institutes of Health, Ministry of Health Malaysia	Ahmad FA; Ahmad Fazilah NA; Anasir MI; Azizan MA; Kamel K; Mohd Zawawi Z; Norhisham SN; Ramly N; Robert F; Suppiah J; Thayan R
EPI_ISL_5850351, EPI_ISL_5850356	Ranau District Health Office	Institute for Medical Research, Infectious Disease Research Centre, National Institutes of Health, Ministry of Health Malaysia	Ahmad FA; Ahmad Fazilah NA; Anasir MI; Azizan MA; Kamel K; Mohd Zawawi Z; Norhisham SN; Ramly N; Robert F; Suppiah J; Thayan R
EPI_ISL_5893996, EPI_ISL_5894151, EPI_ISL_5894725, EPI_ISL_5894732, EPI_ISL_5894738, EPI_ISL_5894746, EPI_ISL_5894758, EPI_ISL_5894762, EPI_ISL_5894771, EPI_ISL_5894781, EPI_ISL_5894788, EPI_ISL_5894794, EPI_ISL_5894807, EPI_ISL_5894810, EPI_ISL_5894814, EPI_ISL_5894818, EPI_ISL_5894826, EPI_ISL_5894834, EPI_ISL_5895002, EPI_ISL_5895009, EPI_ISL_5895020, EPI_ISL_5895025, EPI_ISL_5895034, EPI_ISL_5896105, EPI_ISL_5896111, EPI_ISL_5896140, EPI_ISL_6969047, EPI_ISL_6969060, EPI_ISL_6969073, EPI_ISL_6969083, EPI_ISL_6969091, EPI_ISL_6969098, EPI_ISL_6969120, EPI_ISL_6969135, EPI_ISL_6970405			
see above	Rejang Medical Centre (Sibu)	Institute of Health and Community Medicine	Chan Chia Jui; Chua Hock Hin; David Perera; Ooi Mong How; Tonnii Sia Loong Loong; Wong Jyn Shan
EPI_ISL_5022679	SWAT TEAM, HPSF	UKM Medical Molecular Biology Institute (UMBI)	Mira Farzana binti Mohamad Mokhtar
EPI_ISL_5052108, EPI_ISL_5052109, EPI_ISL_5052110, EPI_ISL_5052111, EPI_ISL_5052112, EPI_ISL_5052113, EPI_ISL_5893712, EPI_ISL_5893737, EPI_ISL_5893803, EPI_ISL_5893806, EPI_ISL_5893821, EPI_ISL_5893960, EPI_ISL_5894158, EPI_ISL_5894166, EPI_ISL_5894176, EPI_ISL_5894183, EPI_ISL_5894191, EPI_ISL_5894195, EPI_ISL_5894202, EPI_ISL_5894539, EPI_ISL_5894549, EPI_ISL_5894560, EPI_ISL_5894566, EPI_ISL_5894578, EPI_ISL_5894583, EPI_ISL_5895224, EPI_ISL_5895225, EPI_ISL_5895226, EPI_ISL_5895227, EPI_ISL_5895228, EPI_ISL_5895229, EPI_ISL_5895232, EPI_ISL_5895239, EPI_ISL_5895249, EPI_ISL_5895256, EPI_ISL_5895269, EPI_ISL_5895274, EPI_ISL_5895284, EPI_ISL_5895291, EPI_ISL_5895298, EPI_ISL_5895309, EPI_ISL_5895313, EPI_ISL_5895324, EPI_ISL_5895331, EPI_ISL_5895339, EPI_ISL_5895346, EPI_ISL_5895354, EPI_ISL_5895362, EPI_ISL_5895369, EPI_ISL_5895384, EPI_ISL_5895387, EPI_ISL_5895396, EPI_ISL_5895403, EPI_ISL_5895412, EPI_ISL_5895421, EPI_ISL_5895428, EPI_ISL_5895433, EPI_ISL_5895444, EPI_ISL_5895450, EPI_ISL_5895457, EPI_ISL_5895464, EPI_ISL_5895467, EPI_ISL_5895476, EPI_ISL_5895486, EPI_ISL_5895495, EPI_ISL_5895502, EPI_ISL_5895504, EPI_ISL_5895505, EPI_ISL_5895516, EPI_ISL_5895525, EPI_ISL_5895531, EPI_ISL_5895538, EPI_ISL_5896202, EPI_ISL_5896207, EPI_ISL_5896213, EPI_ISL_5896218, EPI_ISL_5896227, EPI_ISL_5896237, EPI_ISL_5896245, EPI_ISL_6586280, EPI_ISL_6586281, EPI_ISL_6586282, EPI_ISL_6586283, EPI_ISL_6586284, EPI_ISL_6586285, EPI_ISL_6586295, EPI_ISL_6968280			
see above	Sarawak General Hospital (Kuching)	Institute of Health and Community Medicine	Chan Chia Jui; Chua Hock Hin; David Perera; Ooi Mong How; Tonnii Sia Loong Loong; Wong Jyn Shan; Wong Kiing Aik
EPI_ISL_6970243, EPI_ISL_6970247, EPI_ISL_6970251, EPI_ISL_6970257, EPI_ISL_6970261, EPI_ISL_6970266, EPI_ISL_6970273, EPI_ISL_6970283, EPI_ISL_6970286			
see above	Sarawak Heart Centre (SHC)	Institute of Health and Community Medicine	Chan Chia Jui; Chua Hock Hin; David Perera; Ooi Mong How; Tonnii Sia Loong Loong; Wong Jyn Shan

EPI_ISL_5051781, EPI_ISL_5052013, EPI_ISL_5052014	Sarawak Heart Centre (SHC), Kota Samarahan	Institute of Health and Community Medicine	Chan Chia Jui; Chua Hock Hin; David Perera; Ooi Mong How; Tonnii Sia Loong Loong; Wong Jyn Shan
EPI_ISL_5395813, EPI_ISL_5395815, EPI_ISL_5395818	State Health Department Federal Territory of Labuan	Institute for Medical Research, Infectious Disease Research Centre, National Institutes of Health, Ministry of Health Malaysia	Ahmad FA; Ahmad Fazilah NA; Anasir MI; Azizan MA; Kamel K; Mohd Zawawi Z; Norhisham SN; Ramly N; Robert F; Suppiah J; Thayan R
EPI_ISL_5395681, EPI_ISL_5395683, EPI_ISL_5395685, EPI_ISL_5395689, EPI_ISL_6774041	Sungai Buloh Hospital	Institute for Medical Research, Infectious Disease Research Centre, National Institutes of Health, Ministry of Health Malaysia	Ahmad FA; Ahmad Fazilah NA; Anasir MI; Azizan MA; Kamel K; Mohamad Sukri MZ; Mohd Zawawi Z; Norhisham SN; Ramly N; Robert F; Rosli NR; Suppiah J; Thayan R
EPI_ISL_5396290	Tuaran Hospital	Institute for Medical Research, Infectious Disease Research Centre, National Institutes of Health, Ministry of Health Malaysia	Ahmad FA; Ahmad Fazilah NA; Anasir MI; Azizan MA; Kamel K; Mohd Zawawi Z; Norhisham SN; Ramly N; Robert F; Suppiah J; Thayan R
EPI_ISL_5022689, EPI_ISL_5058547, EPI_ISL_5058552	UKA HPSF MUAR	UKM Medical Molecular Biology Institute (UMBI)	Mira Farzana binti Mohamad Mokhtar
EPI_ISL_5058540	UKKP HOSPITAL MELAKA	UKM Medical Molecular Biology Institute (UMBI)	Mira Farzana binti Mohamad Mokhtar
EPI_ISL_5058543	UKKP Hospital Melaka	UKM Medical Molecular Biology Institute (UMBI)	Mira Farzana binti Mohamad Mokhtar
EPI_ISL_5058544	UKKP, Hospital Melaka	UKM Medical Molecular Biology Institute (UMBI)	Mira Farzana binti Mohamad Mokhtar
EPI_ISL_6131994	UNIT KESELAMATAN DAN KESIHATAN PEKERJAAN	Department of Medical Microbiology, Faculty of Medicine, University of Malaya; University of Malaya Medical Centre	I-Ching SAM; Jolene Yin Ling FU; Omar Khalilur Rahman; Yoke Fun Chan
EPI_ISL_5417602	UNIT KESIHATAN AWAM	UKM Medical Molecular Biology Institute (UMBI)	Mira Farzana binti Mohamad Mokhtar
EPI_ISL_8090630	UNIT KESIHATAN AWAM ,UKA . HRPZ	Tropical Infectious Diseases Research & Education Centre (TIDREC), Universiti Malaya	AsmaAnati CheMatSeri; Che-Norainon Yaacob; Jia-Yi Tan; Jo-Ern Wong; Kim-Kee Tan; Mulya-Mustika-Sari Zulkifli; Noor Syahida Azizan; Nur-Hidayana Mahfodz; Sazaly AbuBakar; Siti-Sarah Nor'e
EPI_ISL_5022669, EPI_ISL_5022676, EPI_ISL_5022683, EPI_ISL_5022687, EPI_ISL_5429423, EPI_ISL_5429427	UNIT KESIHATAN AWAM HEBHK	UKM Medical Molecular Biology Institute (UMBI)	Mira Farzana binti Mohamad Mokhtar
EPI_ISL_4731205, EPI_ISL_4731227, EPI_ISL_5022672, EPI_ISL_5022677	UNIT KPAS, PKD MELAKA TENGAH	UKM Medical Molecular Biology Institute (UMBI)	Mira Farzana binti Mohamad Mokhtar
EPI_ISL_5450126	UNIT PERUBATAN FORENSIK	iPROMISE, UiTM	Ariza Adnan; Fadzilah Mohd Nor; Lim Wai Feng; Mohd Asif Mohd Sukri; Mohd Nur Fakhruzzaman Noorizhab; Mohd Zaki Salleh; Sazzli Shahlan Kassim; Siti Farah Alwani Mohd Nawi; Siti Hamimah Sheikh Abdul Kadir; Teh Lay Kek; Wang Seok Mui
EPI_ISL_5450100	UNIT PERUBATAN FORENSIK SUNGAI BAKAP	iPROMISE, UiTM	Ariza Adnan; Fadzilah Mohd Nor; Lim Wai Feng; Mohd Asif Mohd Sukri; Mohd Nur Fakhruzzaman Noorizhab; Mohd Zaki Salleh; Sazzli Shahlan Kassim; Siti Farah Alwani Mohd Nawi; Siti Hamimah Sheikh Abdul Kadir; Teh Lay Kek; Wang Seok Mui
EPI_ISL_5022691	hospital sultanah nora ismail	UKM Medical Molecular Biology Institute (UMBI)	Mira Farzana binti Mohamad Mokhtar
EPI_ISL_5450384	pkd spt	iPROMISE, UiTM	Ariza Adnan; Fadzilah Mohd Nor; Lim Wai Feng; Mohd Asif Mohd Sukri; Mohd Nur Fakhruzzaman Noorizhab; Mohd Zaki Salleh; Sazzli Shahlan Kassim; Siti Farah Alwani Mohd Nawi; Siti Hamimah Sheikh Abdul Kadir; Teh Lay Kek; Wang Seok Mui
EPI_ISL_5417178	unit keselamatan dan kesihatan pekerjaan	Department of Medical Microbiology, University Malaya Medical Centre	I-Ching SAM; Jolene Yin Ling FU; Omar Khalilur Rahman; Yoke Fun Chan

We gratefully acknowledge the following Authors from the Originating laboratories responsible for obtaining the specimens, as well as the Submitting laboratories where the genome data were generated and shared via GISAID, on which this research is based.

All Submitters of data may be contacted directly via www.gisaid.org

Authors are sorted alphabetically.

Acknowledgement EPI_SET Identifier: EPI_SET_20220603us

Accession ID	Originating Laboratory	Submitting Laboratory	Authors
EPI_ISL_718167, EPI_ISL_718174, EPI_ISL_718176, EPI_ISL_718178, EPI_ISL_718182, EPI_ISL_718185, EPI_ISL_718191, EPI_ISL_718192, EPI_ISL_718197, EPI_ISL_718198, EPI_ISL_718203, EPI_ISL_718204, EPI_ISL_718205, EPI_ISL_718216, EPI_ISL_718218, EPI_ISL_718222, EPI_ISL_2367474, EPI_ISL_2367476			
see above	Borneo Medical Centre	Institute of Health and Community Medicine	Chan Chia Jui; Chua Hock Hin; David Perera; Ooi Mong How; Tonnii Sia Loong Loong; Wong Jyn Shan; Wong Kiing Aik
EPI_ISL_2827788, EPI_ISL_2827789, EPI_ISL_2827790	DEPARTMENT OF MEDICAL MICROBIOLOGY & PARASITOLOGY, SCHOOL OF MEDICAL SCIENCES	DEPARTMENT OF MEDICAL MICROBIOLOGY & PARASITOLOGY, SCHOOL OF MEDICAL SCIENCES	A.H.; A.S.; Alexander Chong; Ayub, Q.; Azmi; Banga Singh; Chan; Chua; Deris; E.A.R.; Eng; Engku Nur Syafirah; Halim; Harun, A.; Hussin, Z.; K.I.; K.K.; Khalid; L.H.; Lau; Lee; Leela, A.; Lim; M.F.; M.N.F.; M.Z.H.; Mat Salleh; Md Zain, M.; Md Zoqratt; Muhammad; Musa; Musa, N.; N.H.; N.L.; N.M.; N.S.; Omar, S.; Rahman, S.; Rosline, H.; S.C.; S.Y.; Samsudin; W.C.; W.L.; W.M.; W.W.H.; Y.Y.; Yaacob; Z.Z.; Zahiruddin
EPI_ISL_1059902	DNA Laboratories Sdn. Bhd.	Malaysia Genome Institute	Azrin Ahmad; Enizza Kasim; Irni Suhayu Sapian; Mohd Faizal Abu Bakar; Mohd Noor Mat Isa; Nor Azfa Johari.; Nurhezreen Md Iqbal; Shamsidar Sopie; Siti Noraini Othman; Wong Yong Wee; Yusuf Muhammad Noor
EPI_ISL_1435783, EPI_ISL_1435785, EPI_ISL_1435787, EPI_ISL_1435789, EPI_ISL_1435791, EPI_ISL_1435792, EPI_ISL_1435794, EPI_ISL_1435796, EPI_ISL_1435798, EPI_ISL_1435800			
see above	DNALAB Sdn. Bhd.	Malaysia Genome Institute	Azrin Ahmad; Enizza Kasim; Irni Suhayu Sapian; Mohd Faizal Abu Bakar; Mohd Noor Mat Isa; Nor Azfa Johari.; Nurhezreen Md Iqbal; Shamsidar Sopie; Siti Noraini Othman; Wong Yong Wee; Yusuf Muhammad Noor
EPI_ISL_2988859, EPI_ISL_2988860, EPI_ISL_2988861, EPI_ISL_2988862, EPI_ISL_2988863, EPI_ISL_2988864, EPI_ISL_2988865, EPI_ISL_2988866, EPI_ISL_2988867, EPI_ISL_2988868, EPI_ISL_2988869, EPI_ISL_2988875, EPI_ISL_2988876, EPI_ISL_2988877, EPI_ISL_4880398, EPI_ISL_4886618			
see above	Department of Medical Microbiology and Parasitology, Hospital Universiti Sains Malaysia	Molecular Research Laboratory	Abdul Haris bin Muhammad; Ahmad Sukari Bin Halim; Alexander Chong Shu Chien; Aswini Leela; Azian Harun; Chan Yean Yean; Chua Wei Chuan; Chua Wei Lian; Engku Nur Syafirah bt Engku Abd Rahman; Kamarul Imran Musa; Kirnpal Kaur Banga Singh; Lau Nyok Sean; Lee Lih Huey; Lee Yeong Yeh; Lim Shu Yong; Maizun binti Mohd Zain; Muhammad Fazli bin Khalid; Muhammad Nashrul Farhan Samsudin; Muhammad Zarul Hanifah Bin Md Zoqratt; Najib Majdi Bin Yaacob; Nazmi Liana Binti Azmi; Noor Hafizan binti Mat Salleh; Nurfadhlina Musa; Qasim Ayub; Rosline Hassan; Sadequr Rahman; Syahida binti Omar; Wan Mohd Zahiruddin Wan Mohammad; Wilhelm Eng Wei Han; Zaini bin Hussin; Zakuan Zainy Deris
EPI_ISL_2151509, EPI_ISL_2151510, EPI_ISL_2151541	Department of Medical Microbiology, Hospital Pengajar Universiti Putra Malaysia	Department of Medical Microbiology, Hospital Pengajar Universiti Putra Malaysia	Boo Sook Yee; Chin Siang Tean; Hui-Yee Chee; Jia-Yong Lam; Mohd Syis Zulkipli; Narcisse Joseph; Syafinaz Amin-Nordin; Yien-Ping Wong
EPI_ISL_861717, EPI_ISL_861718, EPI_ISL_861719, EPI_ISL_861720, EPI_ISL_861721, EPI_ISL_861722, EPI_ISL_861723, EPI_ISL_861724, EPI_ISL_861725, EPI_ISL_861726, EPI_ISL_861729, EPI_ISL_977585, EPI_ISL_977586, EPI_ISL_977587, EPI_ISL_977588, EPI_ISL_977589, EPI_ISL_977590			
see above	Department of Medical Microbiology, Hospital Pengajar Universiti Putra Malaysia	Malaysia Genome Institute	Avisha Richards; Azrin Ahmad; Enizza Kasim; Hui-Yee Chee; Irni Suhayu Sapian; Mohd Faizal Abu Bakar; Mohd Noor Mat Isa; Muhammad MI; Narcisse Joseph; Nor Azfa Johari; Nor Zahrin Hasran; Nurhezreen Md Iqbal; Shamsidar Sopie; Siti Noraini Othman; Syafinaz Amin-Nordin; Yusuf Muhammad Noor
EPI_ISL_417917	Department of Medical Microbiology, University Malaya Medical Centre	Department of Medical Microbiology	Adeeba KAMARULZAMAN; Chee Kuan WONG; Cindy Shuan Ju TEH; I-Ching SAM; Sasheela PONNAMPALAVANAR; Sharifah Faridah SYED OMAR; University Malaya Medical Centre COVID Team; Vijayan MUNUSAMY; Yoke Fun Chan; Yoong Min CHONG
EPI_ISL_417918, EPI_ISL_417919, EPI_ISL_417920, EPI_ISL_501176, EPI_ISL_501177, EPI_ISL_501178, EPI_ISL_501179, EPI_ISL_501180, EPI_ISL_501181, EPI_ISL_501182, EPI_ISL_501183, EPI_ISL_501184, EPI_ISL_501185, EPI_ISL_501186, EPI_ISL_501187, EPI_ISL_501188, EPI_ISL_501189, EPI_ISL_501190, EPI_ISL_501191, EPI_ISL_501192, EPI_ISL_501193, EPI_ISL_501194, EPI_ISL_501195, EPI_ISL_501196, EPI_ISL_501197, EPI_ISL_501198, EPI_ISL_501199, EPI_ISL_501200, EPI_ISL_501201, EPI_ISL_501202, EPI_ISL_501203, EPI_ISL_501204, EPI_ISL_501205, EPI_ISL_501206, EPI_ISL_501207, EPI_ISL_501208, EPI_ISL_501209, EPI_ISL_501210, EPI_ISL_501211, EPI_ISL_501212, EPI_ISL_501213, EPI_ISL_501214, EPI_ISL_501215, EPI_ISL_501216, EPI_ISL_501217, EPI_ISL_501218, EPI_ISL_501219, EPI_ISL_501220, EPI_ISL_501221, EPI_ISL_501222, EPI_ISL_501223, EPI_ISL_501224, EPI_ISL_501225, EPI_ISL_501226, EPI_ISL_501227, EPI_ISL_501228, EPI_ISL_506996, EPI_ISL_506997, EPI_ISL_506998, EPI_ISL_506999, EPI_ISL_507000, EPI_ISL_2769381, EPI_ISL_2769382, EPI_ISL_2769383, EPI_ISL_2769384, EPI_ISL_2769385, EPI_ISL_2769386, EPI_ISL_2769387, EPI_ISL_2769388, EPI_ISL_2769389, EPI_ISL_2769390, EPI_ISL_2769391, EPI_ISL_2769392, EPI_ISL_2769393, EPI_ISL_2769394, EPI_ISL_2769395, EPI_ISL_2769396, EPI_ISL_2769397, EPI_ISL_2769398, EPI_ISL_2769399			
see above	Department of Medical Microbiology, University Malaya Medical Centre	Department of Medical Microbiology, Faculty of Medicine, University of Malaya	Adeeba KAMARULZAMAN; Chee Kuan WONG; Cindy Shuan Ju TEH; Fadhil Hadi JAMALUDDIN; Han Ming GAN; I-Ching SAM; Jennifer Chong; Sasheela PONNAMPALAVANAR; Sharifah Faridah SYED OMAR; University Malaya Medical Centre COVID Team; Vijayan MUNUSAMY; Yoke Fun CHAN; Yoke Fun Chan; Yoong Min CHONG
EPI_ISL_936494	Hospital Enche Besar Hajjah Khalsom Kluang	Institute for Medical Research, Infectious Disease Research Centre, National Institutes of Health, Ministry of Health Malaysia	Azizan MA; Kamel K; Suppiah J; Thayan R
EPI_ISL_936488	Hospital Queen Elizabeth	Institute for Medical Research, Infectious Disease Research Centre, National Institutes of Health, Ministry of Health Malaysia	Azizan MA; Kamel K; Suppiah J; Thayan R
EPI_ISL_936489	Hospital Raja Perempuan Zainab II	Institute for Medical Research, Infectious Disease Research Centre, National Institutes of Health, Ministry of Health Malaysia	Azizan MA; Kamel K; Suppiah J; Thayan R
EPI_ISL_455790, EPI_ISL_455791, EPI_ISL_455792, EPI_ISL_455793	Institute for Medical Research, Infectious Disease Research Centre, National Institutes of Health, Ministry of Health Malaysia	Malaysia Genome Institute	Azrin Ahmad; Enizza Kasim; Irni Suhayu Sapian; Jeyanthi Suppiah; Mohd Faizal Abu Bakar; Mohd Noor Mat Isa; Norazah Ahmad; Nurhezreen Md Iqbal; Ravindran Thayan; Shahrul Hisham Zainal Ariffin; Shamsidar Sopie; Siti Noraini Othman; Tahir Aris; Yusuf Muhammad Noor; Zarina Mohd Zawawi
EPI_ISL_430439	Institute for Medical Research, Infectious Disease Research Centre, National Institutes of Health, Ministry of Health Malaysia	Institute for Medical Research Infectious Disease Research Centre, National Institutes of Health, Ministry of Health Malaysia	Abdul-Wahid.M-Z; Ahmad.N; Azizan.M-A; Hisham.H-A; Kalyanasundram.J; Mat-Sharani.S; Mohd-Zain.R; Mohd-Zawawi.Z; Suppiah.J; Tan.L-P; Thayan.R
EPI_ISL_459957	Institute for Medical Research, Infectious Disease Research Centre, National Institutes of Health, Minis	Institute for Medical Research, Infectious Disease Research Centre, National Institutes of Health, Minis	Ellan K; Kalyanasundram J; Kamel KA; Mohd-Zain R; Mohd-Zawawi Z; Suppiah J; Thayan R
EPI_ISL_459953	Institute for Medical Research, Infectious Disease Research Centre, National Institutes of Health, Ministry of Health Malaysia	Institute for Medical Research Infectious Disease Research Centre, National Institutes of Health, Ministry of Health Malaysia	Ellan K; Kalyanasundram J; Kamel KA; Mohd-Zain R; Mohd-Zawawi Z; Suppiah J; Thayan R
EPI_ISL_430440, EPI_ISL_430441, EPI_ISL_430442, EPI_ISL_430443, EPI_ISL_430444, EPI_ISL_459954, EPI_ISL_459955, EPI_ISL_459956, EPI_ISL_489992, EPI_ISL_489993, EPI_ISL_489994, EPI_ISL_490014, EPI_ISL_490015, EPI_ISL_490016, EPI_ISL_490047, EPI_ISL_490048, EPI_ISL_490089, EPI_ISL_490090, EPI_ISL_490091, EPI_ISL_490092, EPI_ISL_490093, EPI_ISL_490094, EPI_ISL_490095, EPI_ISL_490096, EPI_ISL_490097, EPI_ISL_490098, EPI_ISL_490099, EPI_ISL_490100, EPI_ISL_490101, EPI_ISL_490102, EPI_ISL_490103, EPI_ISL_596449, EPI_ISL_644671, EPI_ISL_644672, EPI_ISL_718267, EPI_ISL_718268, EPI_ISL_718269, EPI_ISL_718270, EPI_ISL_718271, EPI_ISL_718272, EPI_ISL_718274, EPI_ISL_718275, EPI_ISL_718276, EPI_ISL_718277, EPI_ISL_718278, EPI_ISL_718279, EPI_ISL_718281, EPI_ISL_718282, EPI_ISL_718283, EPI_ISL_718284, EPI_ISL_718285, EPI_ISL_718286, EPI_ISL_718287, EPI_ISL_718288, EPI_ISL_718289, EPI_ISL_718290, EPI_ISL_718291, EPI_ISL_718292, EPI_ISL_718293, EPI_ISL_718294, EPI_ISL_718295, EPI_ISL_718296, EPI_ISL_718297, EPI_ISL_718298, EPI_ISL_718299, EPI_ISL_718300, EPI_ISL_718301, EPI_ISL_718302, EPI_ISL_718303, EPI_ISL_718304, EPI_ISL_718305, EPI_ISL_718306, EPI_ISL_718307, EPI_ISL_718308, EPI_ISL_718309, EPI_ISL_718310, EPI_ISL_718311, EPI_ISL_718312, EPI_ISL_718313, EPI_ISL_718314, EPI_ISL_718315, EPI_ISL_728157, EPI_ISL_728158, EPI_ISL_728160, EPI_ISL_728161, EPI_ISL_728162, EPI_ISL_728163, EPI_ISL_728164, EPI_ISL_728165, EPI_ISL_728166, EPI_ISL_728167, EPI_ISL_728169, EPI_ISL_728170, EPI_ISL_728178, EPI_ISL_728209, EPI_ISL_728210, EPI_ISL_728211, EPI_ISL_728212, EPI_ISL_728241, EPI_ISL_728242, EPI_ISL_728243, EPI_ISL_728244, EPI_ISL_728245, EPI_ISL_728246, EPI_ISL_728247, EPI_ISL_728248, EPI_ISL_728249, EPI_ISL_728250, EPI_ISL_728251, EPI_ISL_728252, EPI_ISL_936496, EPI_ISL_1114726, EPI_ISL_1196930, EPI_ISL_2896287			
see above	Institute for Medical Research, Infectious Disease Research Centre, National Institutes of Health, Ministry of Health Malaysia	Institute for Medical Research, Infectious Disease Research Centre, National Institutes of Health, Ministry of Health Malaysia	Abdul-Wahid.M-Z; Ahmad.N; Azizan MA; Azizan.M-A; Ellan K; Hisham.H-A; Kalyanasundram J; Kalyanasundram.J; Kamel K; Kamel KA; Mat-Sharani.S; Mohd Zawawi Z; Mohd-Zain R; Mohd-Zain.R; Mohd-Zawawi Z; Mohd-Zawawi.Z; Ramly N; Robert F; Suppiah J; Suppiah.J; Tan.L-P; Tengku-Abd-Rashid.T-R; Tengku-Rogayah.TAR; Thayan R; Thayan.R
EPI_ISL_8147365, EPI_ISL_8147392	Klinik Kesihatan Kelana Jaya	Tropical Infectious Diseases Research & Education Centre (TIDREC), Universiti Malaya	AsmaAnati CheMatSeri; Che-Norainon Yaacob; Jia-Yi Tan; Jo-Ern Wong; Kim-Kee Tan; Mulya-Mustika-Sari Zulkifli; Noor Syahida Azizan; Nur-Hidayana Mahfodz; Sazaly AbuBakar; Siti-Sarah Nor’e
EPI_ISL_8147391	Klinik Kesihatan Lahad Datu	Tropical Infectious Diseases Research & Education Centre (TIDREC), Universiti Malaya	AsmaAnati CheMatSeri; Che-Norainon Yaacob; Jia-Yi Tan; Jo-Ern Wong; Kim-Kee Tan; Mulya-Mustika-Sari Zulkifli; Noor Syahida Azizan; Nur-Hidayana Mahfodz; Sazaly AbuBakar; Siti-Sarah Nor’e
EPI_ISL_8147366	Klinik Kesihatan Seri Kembangan	Tropical Infectious Diseases Research & Education Centre (TIDREC), Universiti Malaya	AsmaAnati CheMatSeri; Che-Norainon Yaacob; Jia-Yi Tan; Jo-Ern Wong; Kim-Kee Tan; Mulya-Mustika-Sari Zulkifli; Noor Syahida Azizan; Nur-Hidayana Mahfodz; Sazaly AbuBakar; Siti-Sarah Nor’e
EPI_ISL_2988871, EPI_ISL_2988872, EPI_ISL_2988873, EPI_ISL_2988874, EPI_ISL_4880368, EPI_ISL_4880369, EPI_ISL_4880374, EPI_ISL_4880375, EPI_ISL_4880376, EPI_ISL_4880377, EPI_ISL_4880378, EPI_ISL_4880379, EPI_ISL_4880380, EPI_ISL_4880381, EPI_ISL_4880382, EPI_ISL_4880383, EPI_ISL_4880384, EPI_ISL_4880385, EPI_ISL_4880386, EPI_ISL_4880387, EPI_ISL_4880388, EPI_ISL_4880399, EPI_ISL_4880400, EPI_ISL_7972361, EPI_ISL_7972362, EPI_ISL_7972363, EPI_ISL_7972364, EPI_ISL_7972365, EPI_ISL_7972366, EPI_ISL_7972367, EPI_ISL_7972368, EPI_ISL_7972369, EPI_ISL_7972370, EPI_ISL_7972371, EPI_ISL_7972372, EPI_ISL_7972373, EPI_ISL_7972374, EPI_ISL_7972375, EPI_ISL_7972376, EPI_ISL_7972377, EPI_ISL_7972380, EPI_ISL_7972381, EPI_ISL_7972382, EPI_ISL_7972383, EPI_ISL_7972384, EPI_ISL_7972385, EPI_ISL_7972386			
see above	Kota Bharu Public Health Laboratory	Molecular Research Laboratory	Abdul Haris bin Muhammad; Ahmad Sukari Bin Halim; Alexander Chong Shu Chien; Aswini Leela; Azian Harun; Chan Yean Yean; Chua Wei Chuan; Chua Wei Lian; Engku Nur Syafirah bt Engku Abd Rahman; Kamarul Imran Musa; Kirnpal Kaur Banga Singh; Lau Nyok Sean; Lee Lih Huey; Lee Yeong Yeh; Lim Shu Yong; Maizun binti Mohd Zain; Muhammad Fazli bin Khalid; Muhammad Nashrul Farhan Samsudin; Muhammad Zarul Hanifah Bin Md Zoqratt; Najib Majdi Bin Yaacob; Nazmi Liana Binti Azmi; Noor Hafizan binti Mat Salleh; Nurfadhlina Musa; Qasim Ayub; Rosline Hassan; Sadequr Rahman; Syahida binti Omar; Wan Mohd Zahiruddin Wan Mohammad; Wilhelm Eng Wei Han; Zaini bin Hussin; Zakuan Zainy Deris
EPI_ISL_807151, EPI_ISL_807152, EPI_ISL_807153	Makmal Kesihatan Awam Kebangsaan, Kementerian Kesihatan Malaysia	Integrative Pharmacogenomics Institute (iPROMISE)	Ariza Adnan; Fadzilah Mohd Nor; Hani Mat Hussin; Lim Wai Feng; Mohd Nur Fakhruzzaman Noorizhab; Mohd Zaki Salleh; Nor Zahrin Hasran; Sazzli Shahlan Kassim; Siti Farah Alwani Mohd Nawi; Siti Hamimah Sheikh Abdul Kadir; Teh Lay Kek; Wang Seok Mui
EPI_ISL_807150	Makmal Kesihatan Awam Kebangsaan, Kementerian Kesihatan Malaysia.	Integrative Pharmacogenomics Institute (iPROMISE)	Ariza Adnan; Fadzilah Mohd Nor; Hani Mat Hussin; Lim Wai Feng; Mohd Nur Fakhruzzaman Noorizhab; Mohd Zaki Salleh; Nor Zahrin Hasran; Sazzli Shahlan Kassim; Siti Farah Alwani Mohd Nawi; Siti Hamimah Sheikh Abdul Kadir; Teh Lay Kek; Wang Seok Mui
EPI_ISL_936490	Makmal Kesihatan Awam Kota Bharu	Institute for Medical Research, Infectious Disease Research Centre, National Institutes of Health, Ministry of Health Malaysia	Azizan MA; Kamel K; Suppiah J; Thayan R
EPI_ISL_528738, EPI_ISL_528739, EPI_ISL_528740, EPI_ISL_528741, EPI_ISL_528742, EPI_ISL_528743, EPI_ISL_528744, EPI_ISL_568873, EPI_ISL_568874, EPI_ISL_568875, EPI_ISL_582124, EPI_ISL_977591, EPI_ISL_977592, EPI_ISL_977593, EPI_ISL_2342585, EPI_ISL_2342586, EPI_ISL_2342587			
see above	Malaysia Genome Institute	Malaysia Genome Institute	Azrin Ahmad; Enizza Kasim; Gan Han Ming; Irni Suhayu Sapian; Mohd Faizal Abu Bakar; Mohd Noor Mat Isa; Nor Azfa Johari; Nor Azfa Johari.; Nurhezreen Md Iqbal; Shahrul Hisham Zainal Ariffin; Shamsidar Sopie; Siti Noraini Othman; Tan Ju Lin; Yusuf Muhammad Noor
EPI_ISL_455312, EPI_ISL_455313	Microbiology Unit, Department of Pathology & Laboratory Medicine, IIUM Medical Centre	SEA Microbiome Unit, Faculty of Industrial Sciences & Technology, Universiti Malaysia Pahang	Ahmad Hafiz Bin Zulkifly; Ahmad Hafiz Zulkifly; Ahmad Mahfuz Gazali; Hajar Fauzan Ahmad; IIUM Medical Centre Covid19 Taskforce; Mohd Fazli Farida Asras; Mohd Nazli Bin Kamarulzaman; Muhammad Adam Lee Abdullah; Norhidayah Binti Kamarudin; Norhidayah Kamarudin; UMP Covid19 Team
EPI_ISL_718132, EPI_ISL_718133, EPI_ISL_718134, EPI_ISL_718135, EPI_ISL_718136, EPI_ISL_718137, EPI_ISL_718138, EPI_ISL_718139, EPI_ISL_718140, EPI_ISL_718141, EPI_ISL_718142, EPI_ISL_718143, EPI_ISL_718144, EPI_ISL_718145, EPI_ISL_718146, EPI_ISL_718147, EPI_ISL_718148, EPI_ISL_718149, EPI_ISL_718150, EPI_ISL_718151, EPI_ISL_718152, EPI_ISL_718153, EPI_ISL_718154, EPI_ISL_718155, EPI_ISL_718156, EPI_ISL_718157, EPI_ISL_718158, EPI_ISL_718159, EPI_ISL_718160, EPI_ISL_718161, EPI_ISL_718162, EPI_ISL_718163, EPI_ISL_718164, EPI_ISL_718165, EPI_ISL_718166, EPI_ISL_718168, EPI_ISL_718169, EPI_ISL_718170, EPI_ISL_718171, EPI_ISL_718172, EPI_ISL_718173, EPI_ISL_718175, EPI_ISL_718177, EPI_ISL_718179, EPI_ISL_718180, EPI_ISL_718181, EPI_ISL_718183, EPI_ISL_718184, EPI_ISL_718186, EPI_ISL_718187, EPI_ISL_718188, EPI_ISL_718189, EPI_ISL_718190, EPI_ISL_718193, EPI_ISL_718194, EPI_ISL_718195, EPI_ISL_718196, EPI_ISL_718199, EPI_ISL_718200, EPI_ISL_718201, EPI_ISL_718202, EPI_ISL_718206, EPI_ISL_718207, EPI_ISL_718208, EPI_ISL_718209, EPI_ISL_718210, EPI_ISL_718211, EPI_ISL_718212, EPI_ISL_718213, EPI_ISL_718214, EPI_ISL_718215, EPI_ISL_718217,			

EPI_ISL_718219, EPI_ISL_718220, EPI_ISL_718221, EPI_ISL_2367469, EPI_ISL_2367470, EPI_ISL_2367473, EPI_ISL_2367475, EPI_ISL_2367477, EPI_ISL_2367478, EPI_ISL_2367479, EPI_ISL_2367480

see above	Ministry of Health Hospitals	Institute of Health and Community Medicine	Chan Chia Jui; Chua Hock Hin; David Perera; Ooi Mong How; Tonnii Sia Loong Loong; Wong Jyn Shan; Wong Kiing Aik
EPI_ISL_738086	National Public Health Laboratory	Department of Medical Microbiology, Hospital Pengajar Universiti Putra Malaysia	Hui-Yee Chee; Jia-Yong Lam; Muhammad MI; Narcisse Joseph; Nor Zahrin Hasran; Syafinaz Amin-Nordin; Yien-Ping Wong
EPI_ISL_416829, EPI_ISL_416866, EPI_ISL_416884, EPI_ISL_416885, EPI_ISL_416886, EPI_ISL_416907	National Public Health Laboratory	Malaysia Genome Institute	Azrin Ahmad; Enizza Kasim; Hani Mat Hussin; Irni Suhayu Sapian; Mohd Faizal Abu Bakar; Mohd Noor Mat Isa; Noorliza Mohamad Noordin; Nor Azfa Johari; Norazimah Tajudin; Nurhezreen Md Iqbal; Rehan Shuhada Abu Bakar; Selvanesan Sengol; Shahrul Hisham Zainal Ariffin; Shamsidar Sopie; Siti Noraini Othman; W Afiza W Mohd Arifin; Yu Kie Chem; Yusuf Muhammad Noor
EPI_ISL_410487, EPI_ISL_410488, EPI_ISL_410489	National Public Health Laboratory	National Public Health Laboratory	A.B.; M.H.; M.N. and Hani; Noorliza; Norazimah, T.; Rehan Shuhada; Selvanesan, S.; Yu Kie, C.

EPI_ISL_1972889, EPI_ISL_1972890, EPI_ISL_1972891, EPI_ISL_1972892, EPI_ISL_1972893, EPI_ISL_1972894, EPI_ISL_1972895, EPI_ISL_2001114, EPI_ISL_2001115, EPI_ISL_2001213, EPI_ISL_2001429, EPI_ISL_2001430, EPI_ISL_2001481, EPI_ISL_2001482, EPI_ISL_2001643, EPI_ISL_2001644, EPI_ISL_2001645, EPI_ISL_2001654, EPI_ISL_2001655, EPI_ISL_2001656, EPI_ISL_2001659, EPI_ISL_2009116, EPI_ISL_2009193, EPI_ISL_2009232, EPI_ISL_2009233, EPI_ISL_2009234, EPI_ISL_2009305, EPI_ISL_2009529, EPI_ISL_2009530, EPI_ISL_2009595

see above	National Public Health Laboratory Malaysia	National Public Health Laboratory Malaysia	Aziyati O; Chong CK; Hani MH; Hannah PYP; JL Tan; Kamal HKZ; Mohd AY; Noorliza MN; Norazimah T; Norazimah T Rehan SAB Selvanesan S; Norhayati R; NurulAina MCA; Nurulsyahida I; Rehan SAB; Selvanesan S; Ushananthiny R; W.NurAfiza WMA Zirwatul AA; YF Ngeow; Yukie C
EPI_ISL_8147393	Pejabat Kesihatan Daerah Hulu Langat	Tropical Infectious Diseases Research & Education Centre (TIDREC), Universiti Malaya	AsmaAnati CheMatSeri; Che-Norainon Yaacob; Jia-Yi Tan; Jo-Ern Wong; Kim-Kee Tan; Mulya-Mustika-Sari Zulkifli; Noor Syahida Azizan; Nur-Hidayana Mahfodz; Sazaly AbuBakar; Siti-Sarah Nor’e
EPI_ISL_936495	Sarawak General Hospital	Institute for Medical Research, Infectious Disease Research Centre, National Institutes of Health, Ministry of Health Malaysia	Azizan MA; Kamel K; Suppiah J; Thayan R
EPI_ISL_12300601, EPI_ISL_12300602	Tropical Infectious Diseases Research & Education Centre (TIDREC), Universiti Malaya	Tropical Infectious Diseases Research & Education Centre (TIDREC), Universiti Malaya	AsmaAnati CheMatSeri; Che-Norainon Yaacob; Jia-Yi Tan; Jo-Ern Wong; Kim-Kee Tan; Mulya-Mustika-Sari Zulkifli; Noor Syahida Azizan; Nur-Hidayana Mahfodz; Sazaly AbuBakar; Siti-Sarah Nor’e
EPI_ISL_3429606	Tropical Infectious Diseases Research and Education Centre (TIDREC), Universiti Malaya, Kuala Lumpur, Malaysia	Tropical Infectious Diseases Research and Education Centre (TIDREC), Universiti Malaya, Kuala Lumpur, Malaysia	AsmaAnati CheMatSeri; Boon-Teong Teoh; Che-Norainon Yaacob; Chee-Sieng Khor; Jefree Johari; Jia-Yi Tan; Jo-Ern Wong; Juraina Abd-Jamil; Kim-Kee Tan; Mulya-Mustika-Sari Zulkifli; Noor Syahida Azizan; Nur-Hidayana Mahfodz; Sazaly AbuBakar; Siti-Sarah Nor’e; Vunjia Tiong

We gratefully acknowledge the following Authors from the Originating laboratories responsible for obtaining the specimens, as well as the Submitting laboratories where the genome data were generated and shared via GISAID, on which this research is based.

All Submitters of data may be contacted directly via www.gisaid.org

Authors are sorted alphabetically.

Acknowledgement EPI_SET Identifier: EPI_SET_20220603gq

Accession ID	Originating Laboratory	Submitting Laboratory	Authors
EPI_ISL_5396353, EPI_ISL_5396510	Banting Hospital	Institute for Medical Research, Infectious Disease Research Centre, National Institutes of Health, Ministry of Health Malaysia	Ahmad FA; Ahmad Fazilah NA; Anasir MI; Azizan MA; Kamel K; Mohd Zawawi Z; Norhisham SN; Ramly N; Robert F; Suppiah J; Thayan R
EPI_ISL_5893844, EPI_ISL_5894005, EPI_ISL_5894012, EPI_ISL_5894022, EPI_ISL_5894030, EPI_ISL_5894034, EPI_ISL_5894044, EPI_ISL_5894057, EPI_ISL_5894145, EPI_ISL_5895144, EPI_ISL_5895150, EPI_ISL_5895159, EPI_ISL_5895166, EPI_ISL_5895174, EPI_ISL_5895180, EPI_ISL_5895193, EPI_ISL_5895199, EPI_ISL_5895200, EPI_ISL_5895201, EPI_ISL_5895202, EPI_ISL_5895203, EPI_ISL_5895204, EPI_ISL_5895205, EPI_ISL_5895206, EPI_ISL_5895207, EPI_ISL_5895208, EPI_ISL_5895209, EPI_ISL_5895210, EPI_ISL_5895211, EPI_ISL_5895212, EPI_ISL_5895213, EPI_ISL_5895214, EPI_ISL_5895215, EPI_ISL_5896173, EPI_ISL_5896178, EPI_ISL_5896187, EPI_ISL_5896199			
see above	Bintulu Hospital PCR Lab, Bintulu	Institute of Health and Community Medicine	Chan Chia Jui; Chua Hock Hin; David Perera; Ooi Mong How; Tan Lee See; Tonnii Sia Loong Loong; Wong Jyn Shan
EPI_ISL_6586360, EPI_ISL_6586361, EPI_ISL_6586362, EPI_ISL_6586363, EPI_ISL_6586364, EPI_ISL_6586365, EPI_ISL_6586366, EPI_ISL_6586367, EPI_ISL_6586368, EPI_ISL_6586369, EPI_ISL_6586370, EPI_ISL_6586371, EPI_ISL_6586372, EPI_ISL_6586373, EPI_ISL_6586374, EPI_ISL_6586375, EPI_ISL_6586376, EPI_ISL_6586377, EPI_ISL_6586378, EPI_ISL_6586379, EPI_ISL_6586380, EPI_ISL_6586381, EPI_ISL_6586382, EPI_ISL_6586383, EPI_ISL_6586384, EPI_ISL_6586385, EPI_ISL_6586386, EPI_ISL_6586387, EPI_ISL_6586388, EPI_ISL_6586389, EPI_ISL_6586667, EPI_ISL_6586668, EPI_ISL_6586669, EPI_ISL_6586670, EPI_ISL_6586671, EPI_ISL_6586672, EPI_ISL_6586673, EPI_ISL_6586674, EPI_ISL_6586675, EPI_ISL_6586676, EPI_ISL_6968271, EPI_ISL_6970013, EPI_ISL_6970020, EPI_ISL_7472876, EPI_ISL_7472877, EPI_ISL_7472878, EPI_ISL_7472879, EPI_ISL_7472880, EPI_ISL_7472881, EPI_ISL_7472882, EPI_ISL_7472883, EPI_ISL_7472951, EPI_ISL_7472952, EPI_ISL_7472953, EPI_ISL_7472954, EPI_ISL_7472955, EPI_ISL_7472956, EPI_ISL_7472957, EPI_ISL_7472958, EPI_ISL_7472959, EPI_ISL_7473054, EPI_ISL_7473055, EPI_ISL_7473056, EPI_ISL_7473057, EPI_ISL_7473058, EPI_ISL_7473076, EPI_ISL_7473077			
see above	Bintulu Medical Centre (Bintulu)	Institute of Health and Community Medicine	Chan Chia Jui; Chua Hock Hin; David Perera; Ooi Mong How; Tonnii Sia Loong Loong; Wong Jyn Shan
EPI_ISL_5450524, EPI_ISL_5450525, EPI_ISL_5450526	CAC ILKKM	iPROMISE, UiTM	Ariza Adnan; Fadzilah Mohd Nor; Lim Wai Feng; Mohd Asif Mohd Sukri; Mohd Nur Fakhruzzaman Noorizhab; Mohd Zaki Salleh; Sazzli Shahlan Kassim; Siti Farah Alwani Mohd Nawi; Siti Hamimah Sheikh Abdul Kadir; Teh Lay Kek; Wang Seok Mui
EPI_ISL_6131985, EPI_ISL_6132008, EPI_ISL_6132033, EPI_ISL_6132044, EPI_ISL_6132058, EPI_ISL_6132065	CPRC JKNT	Department of Medical Microbiology, Faculty of Medicine, University of Malaya; University of Malaya Medical Centre	I-Ching SAM; Jolene Yin Ling FU; Omar Khalilur Rahman; Yoke Fun Chan
EPI_ISL_6586480, EPI_ISL_6586481, EPI_ISL_6586484, EPI_ISL_6586485, EPI_ISL_6586486	Clinical Research Centre (CRC), Sibu Hospital, Sibu	Institute of Health and Community Medicine	Chan Chia Jui; Chua Hock Hin; David Perera; Ooi Mong How; Tonnii Sia Loong Loong; Wong Jyn Shan
EPI_ISL_10070740	Department of Medical Microbiology, Hospital Pengajar Universiti Putra Malaysia	Department of Medical Microbiology, Hospital Pengajar Universiti Putra Malaysia	Afiqah Adzmi; Amiza Azmi; Azmiza Syawani Jasni; Hui Yee Chee; Leslie Thian Lung Than; Muadz Mohtar; Muhammad Mohd Isa; Narcisse MS Joseph; Niazlin Mohd Taib; Noor Hazirah Noor Azhari; Norlaila; Nur Raihana Ithnin; Nurul Huda Mohamed Rashidi; Nurul Nadiah Ismail; Rosni Ibrahim; Rukman Awang Hamat; Sallehhudin; Siti Norbaya Masri; Siti Zulaikha Zakariah; Syafinaz Amin Nordin; Tengku Zetty Maztura Tengku Jamaluddin; Zamberi Sekawi
EPI_ISL_5417136, EPI_ISL_5417137, EPI_ISL_5417138	Department of Medical Microbiology, University Malaya Medical Centre	Department of Medical Microbiology, University Malaya Medical Centre	I-Ching SAM; Jolene Yin Ling FU; Omar Khalilur Rahman; Yoke Fun Chan
EPI_ISL_5893721, EPI_ISL_8188769	Gribbles Pathology, Kuching	Institute of Health and Community Medicine	Chan Chia Jui; Chua Hock Hin; David Perera; Josephine Liew Chin Chin; Ooi Mong How; Reagan Entigu Linton; Tonnii Sia Loong Loong; Wong Jyn Shan
EPI_ISL_5450138, EPI_ISL_5450160, EPI_ISL_5450171, EPI_ISL_5450174, EPI_ISL_5450180, EPI_ISL_5450186, EPI_ISL_5450255			
see above	HOSPITAL PULAU PINANG	iPROMISE, UiTM	Ariza Adnan; Fadzilah Mohd Nor; Lim Wai Feng; Mohd Asif Mohd Sukri; Mohd Nur Fakhruzzaman Noorizhab; Mohd Zaki Salleh; Sazzli Shahlan Kassim; Siti Farah Alwani Mohd Nawi; Siti Hamimah Sheikh Abdul Kadir; Teh Lay Kek; Wang Seok Mui
EPI_ISL_6131991	HOSPITAL TUANKU FAUZIAH	Department of Medical Microbiology, Faculty of Medicine, University of Malaya; University of Malaya Medical Centre	I-Ching SAM; Jolene Yin Ling FU; Omar Khalilur Rahman; Yoke Fun Chan
EPI_ISL_5450144, EPI_ISL_5450149, EPI_ISL_5450163	HPP	iPROMISE, UiTM	Ariza Adnan; Fadzilah Mohd Nor; Lim Wai Feng; Mohd Asif Mohd Sukri; Mohd Nur Fakhruzzaman Noorizhab; Mohd Zaki Salleh; Sazzli Shahlan Kassim; Siti Farah Alwani Mohd Nawi; Siti Hamimah Sheikh Abdul Kadir; Teh Lay Kek; Wang Seok Mui
EPI_ISL_6131987, EPI_ISL_6131988, EPI_ISL_6131993, EPI_ISL_6131995, EPI_ISL_6132007, EPI_ISL_6132010, EPI_ISL_6132024, EPI_ISL_6132060, EPI_ISL_6132062, EPI_ISL_6132066, EPI_ISL_6182084			
see above	HRPB	Department of Medical Microbiology, Faculty of Medicine, University of Malaya; University of Malaya Medical Centre	I-Ching SAM; Jolene Yin Ling FU; Omar Khalilur Rahman; Yoke Fun Chan
EPI_ISL_8090713, EPI_ISL_8090714, EPI_ISL_8090715, EPI_ISL_8090716, EPI_ISL_8090722, EPI_ISL_8090723, EPI_ISL_8090724, EPI_ISL_8090726, EPI_ISL_8090728, EPI_ISL_8090729			
see above	HRPB	Tropical Infectious Diseases Research & Education Centre (TIDREC), Universiti Malaya	AsmaAnati CheMatSeri; Che-Norainon Yaacob; Jia-Yi Tan; Jo-Ern Wong; Kim-Kee Tan; Mulya-Mustika-Sari Zulkifli; Noor Syahida Azizan; Nur-Hidayana Mahfodz; Sazaly AbuBakar; Siti-Sarah Nor'e
EPI_ISL_8090706	HRPB IPOH	Tropical Infectious Diseases Research & Education Centre (TIDREC), Universiti Malaya	AsmaAnati CheMatSeri; Che-Norainon Yaacob; Jia-Yi Tan; Jo-Ern Wong; Kim-Kee Tan; Mulya-Mustika-Sari Zulkifli; Noor Syahida Azizan; Nur-Hidayana Mahfodz; Sazaly AbuBakar; Siti-Sarah Nor'e
EPI_ISL_5450527, EPI_ISL_5450569	HTAA	iPROMISE, UiTM	Ariza Adnan; Fadzilah Mohd Nor; Lim Wai Feng; Mohd Asif Mohd Sukri; Mohd Nur Fakhruzzaman Noorizhab; Mohd Zaki Salleh; Sazzli Shahlan Kassim; Siti Farah Alwani Mohd Nawi; Siti Hamimah Sheikh Abdul Kadir; Teh Lay Kek; Wang Seok Mui
EPI_ISL_6825253	Hospital Ampang	Malaysia Genome Institute	Azrin Ahmad; Enizza Kasim; Irni Suhayu Sapian; Mohd Faizal Abu Bakar; Mohd Ghows Mohd Azzam.; Mohd Noor Mat Isa; Nor Azfa Johari; Nurhezreen Md Iqbal; Shamsidar Sopie; Siti Noraini Othman; Yusuf Muhammad Noor
EPI_ISL_6017863	Hospital Bukit Mertajam	iPROMISE, UiTM	Ariza Adnan; Fadzilah Mohd Nor; Lim Wai Feng; Mohd Asif Mohd Sukri; Mohd Nur Fakhruzzaman Noorizhab; Mohd Zaki Salleh; Sazzli Shahlan Kassim; Siti Farah Alwani Mohd Nawi; Siti Hamimah Sheikh Abdul Kadir; Teh Lay Kek; Wang Seok Mui
EPI_ISL_5450398	Hospital Kepala Batas	iPROMISE, UiTM	Ariza Adnan; Fadzilah Mohd Nor; Lim Wai Feng; Mohd Asif Mohd Sukri; Mohd Nur Fakhruzzaman Noorizhab; Mohd Zaki Salleh; Sazzli Shahlan Kassim; Siti Farah Alwani Mohd Nawi; Siti Hamimah Sheikh Abdul Kadir; Teh Lay Kek; Wang Seok Mui
EPI_ISL_8745582, EPI_ISL_8745583, EPI_ISL_8745585, EPI_ISL_8745586, EPI_ISL_8745587	Hospital Lahad Datu	Malaysia Genome and Vaccine Institute	Apical Scientific Sdn Bhd's Team; Azrin Ahmad; Carey Wee Kai Li; Enizza Kasim; Irni Suhayu Sapian; Jilian Michelle Wong Tzeling; Leong Wai Mun; Mohd Faizal Abu Bakar; Mohd Ghows Mohd Azzam.; Mohd Noor Mat Isa; Nor Azfa Johari; Nurhezreen Md Iqbal; Rose Iszati Ismet Nayan; Shamsidar Sopie; Siti Noraini Othman; Yusuf Muhammad Noor
EPI_ISL_5450353, EPI_ISL_5450358, EPI_ISL_5450363, EPI_ISL_5450371, EPI_ISL_5450378, EPI_ISL_6017925, EPI_ISL_6017934			
see above	Hospital Pulau Pinang	iPROMISE, UiTM	Ariza Adnan; Fadzilah Mohd Nor; Lim Wai Feng; Mohd Asif Mohd Sukri; Mohd Nur Fakhruzzaman Noorizhab; Mohd Zaki Salleh; Sazzli Shahlan Kassim; Siti Farah Alwani Mohd Nawi; Siti Hamimah Sheikh Abdul Kadir; Teh Lay Kek; Wang Seok Mui
EPI_ISL_6825276	Hospital Queen Elizabeth	Malaysia Genome Institute	Azrin Ahmad; Enizza Kasim; Irni Suhayu Sapian; Mohd Faizal Abu Bakar; Mohd Ghows Mohd Azzam.; Mohd Noor Mat Isa; Nor Azfa Johari; Nurhezreen Md Iqbal; Shamsidar Sopie; Siti Noraini Othman; Yusuf Muhammad Noor
EPI_ISL_6825254, EPI_ISL_6825255, EPI_ISL_6825256, EPI_ISL_6825257	Hospital Sungai Buloh	Malaysia Genome Institute	Azrin Ahmad; Enizza Kasim; Irni Suhayu Sapian; Mohd Faizal Abu Bakar; Mohd Ghows Mohd Azzam.; Mohd Noor Mat Isa; Nor Azfa Johari; Nurhezreen Md Iqbal; Shamsidar Sopie; Siti Noraini Othman; Yusuf Muhammad Noor
EPI_ISL_8745589	Hospital Sungai Buloh	Malaysia Genome and Vaccine Institute	Apical Scientific Sdn Bhd's Team; Azrin Ahmad; Carey Wee Kai Li; Enizza Kasim; Irni Suhayu Sapian; Jilian Michelle Wong Tzeling; Leong Wai Mun; Mohd Faizal Abu Bakar; Mohd Ghows Mohd Azzam.; Mohd Noor Mat Isa; Nor Azfa Johari; Nurhezreen Md Iqbal; Rose Iszati Ismet Nayan; Shamsidar Sopie; Siti Noraini Othman; Yusuf Muhammad Noor
EPI_ISL_6825259	Hospital Tengku Ampuan Jemaah	Malaysia Genome Institute	Azrin Ahmad; Enizza Kasim; Irni Suhayu Sapian; Mohd Faizal Abu Bakar; Mohd Ghows Mohd Azzam.; Mohd Noor Mat Isa; Nor Azfa Johari; Nurhezreen Md Iqbal; Shamsidar Sopie; Siti Noraini Othman; Yusuf Muhammad Noor
EPI_ISL_5450523	Hospital Tuanku Fauziah	iPROMISE, UiTM	Ariza Adnan; Fadzilah Mohd Nor; Lim Wai Feng; Mohd Asif Mohd Sukri; Mohd Nur Fakhruzzaman Noorizhab; Mohd Zaki Salleh; Sazzli Shahlan Kassim; Siti Farah Alwani Mohd Nawi; Siti Hamimah Sheikh Abdul Kadir; Teh Lay Kek; Wang Seok Mui
EPI_ISL_8090665	Hospital machang	Tropical Infectious Diseases Research & Education Centre (TIDREC), Universiti Malaya	AsmaAnati CheMatSeri; Che-Norainon Yaacob; Jia-Yi Tan; Jo-Ern Wong; Kim-Kee Tan; Mulya-Mustika-Sari Zulkifli; Noor Syahida Azizan; Nur-Hidayana Mahfodz; Sazaly AbuBakar; Siti-Sarah Nor'e
EPI_ISL_5742681	INSTITUT BIOLOGI MOLEKUL PERUBATAN UKM (UMBI)	UKM Medical Molecular Biology Institute (UMBI)	Mira Farzana binti Mohamad Mokhtar
EPI_ISL_6960097, EPI_ISL_6960098, EPI_ISL_7119182, EPI_ISL_7119188, EPI_ISL_7119207, EPI_ISL_7119221, EPI_ISL_7119232, EPI_ISL_7119255, EPI_ISL_7119272, EPI_ISL_7119298			
see above	Institut Biologi Molekul Perubatan UKM (UMBI)	UKM Medical Molecular Biology Institute (UMBI)	Ab Mutalib; Abd Ghafar; Ishak; Jamal; Khairun Nur; Md Shahri; Mira Farzana; Mira Farzana binti Mohamad Mokhtar; Mohamad Mokhtar; Mohamed Rasheed; Mohd Yunos; Mohd Yusuf; Muhammad Azami; Muhiddin; Nor Azi; Nur Alyaa Afifah; Nurul Syakima; Rahman; Ryia Illani; Siti Nur Hasanah; Zahirrah Begam
EPI_ISL_6017938	JABATAN FORENSIK HOSPITAL PULAU PINANG	iPROMISE, UiTM	Ariza Adnan; Fadzilah Mohd Nor; Lim Wai Feng; Mohd Asif Mohd Sukri; Mohd Nur Fakhruzzaman Noorizhab; Mohd Zaki Salleh; Sazzli Shahlan Kassim; Siti Farah Alwani Mohd Nawi; Siti Hamimah Sheikh Abdul Kadir; Teh Lay Kek; Wang Seok Mui
EPI_ISL_6132045	JABATAN PATOLOGI, HRPZ II	Department of Medical Microbiology, Faculty of Medicine, University of Malaya; University of Malaya Medical Centre	I-Ching SAM; Jolene Yin Ling FU; Omar Khalilur Rahman; Yoke Fun Chan
EPI_ISL_7087983	JABATAN PERUBATAN FORENSIK HOSPITAL SULTANAH BAHIYAH	Department of Medical Microbiology, Faculty of Medicine, University of Malaya; University of Malaya Medical Centre	I-Ching SAM; Jolene Yin Ling FU; Omar Khalilur Rahman; Yoke Fun Chan
EPI_ISL_5450529, EPI_ISL_5450535, EPI_ISL_5450544, EPI_ISL_5450550, EPI_ISL_5450593, EPI_ISL_5450601, EPI_ISL_5450608, EPI_ISL_6017979, EPI_ISL_6712944			
see above	JKN PAHANG	iPROMISE, UiTM	Ariza Adnan; Fadzilah Mohd Nor; Lim Wai Feng; Mohd Asif Mohd Sukri; Mohd Nur Fakhruzzaman Noorizhab; Mohd Zaki Salleh; Sazzli Shahlan Kassim; Siti Farah Alwani Mohd Nawi; Siti Hamimah Sheikh Abdul Kadir; Teh Lay Kek; Wang Seok Mui
EPI_ISL_6132003, EPI_ISL_6132019, EPI_ISL_6132049	Jabatan Forensik Hospital Sultanah Bahiyah	Department of Medical Microbiology, Faculty of Medicine, University of Malaya; University of Malaya Medical Centre	I-Ching SAM; Jolene Yin Ling FU; Omar Khalilur Rahman; Yoke Fun Chan
EPI_ISL_6825279	Jabatan Kesihatan WP Labuan	Malaysia Genome Institute	Azrin Ahmad; Enizza Kasim; Irni Suhayu Sapian; Mohd Faizal Abu Bakar; Mohd Ghows Mohd Azzam.; Mohd Noor Mat Isa; Nor Azfa Johari; Nurhezreen Md Iqbal; Shamsidar Sopie; Siti Noraini Othman; Yusuf Muhammad Noor
EPI_ISL_8745588	Jabatan Kesihatan WP Labuan	Malaysia Genome and Vaccine Institute	Apical Scientific Sdn Bhd's Team; Azrin Ahmad; Carey Wee Kai Li; Enizza Kasim; Irni Suhayu Sapian; Jilian Michelle Wong Tzeling; Leong Wai Mun; Mohd Faizal Abu Bakar; Mohd Ghows Mohd Azzam.; Mohd Noor Mat Isa; Nor Azfa Johari; Nurhezreen Md Iqbal; Rose Iszati Ismet Nayan; Shamsidar Sopie; Siti Noraini Othman; Yusuf Muhammad Noor

EPI_ISL_5450587	KLINIK KESIHATAN KARAK	iPROMISE, UiTM	Ariza Adnan; Fadzilah Mohd Nor; Lim Wai Feng; Mohd Asif Mohd Sukri; Mohd Nur Fakhruzzaman Noorizhab; Mohd Zaki Salleh; Sazzli Shahlan Kassim; Siti Farah Alwani Mohd Nawi; Siti Hamimah Sheikh Abdul Kadir; Teh Lay Kek; Wang Seok Mui
EPI_ISL_9205416	Kajang Hospital	Institute for Medical Research, Infectious Disease Research Centre, National Institutes of Health, Ministry of Health Malaysia	Ahmad FA; Ahmad Fazilah NA; Anasir MI; Azizan MA; Kamel K; Mohamad Sukri MZ; Norhisham SN; Ramly N; Robert F; Rosli NR; Suppiah J; Thayan R
EPI_ISL_6131989	Kk selising	Department of Medical Microbiology, Faculty of Medicine, University of Malaya; University of Malaya Medical Centre	I-Ching SAM; Jolene Yin Ling FU; Omar Khalilur Rahman; Yoke Fun Chan
EPI_ISL_5850371, EPI_ISL_5850419, EPI_ISL_5850449	Kota Belud District Health Office	Institute for Medical Research, Infectious Disease Research Centre, National Institutes of Health, Ministry of Health Malaysia	Ahmad FA; Ahmad Fazilah NA; Anasir MI; Azizan MA; Kamel K; Mohd Zawawi Z; Norhisham SN; Ramly N; Robert F; Suppiah J; Thayan R
EPI_ISL_6422904	Kota Kinabalu Area Health Office	Institute for Medical Research, Infectious Disease Research Centre, National Institutes of Health, Ministry of Health Malaysia	Ahmad FA; Ahmad Fazilah NA; Anasir MI; Azizan MA; Kamel K; Mohd Zawawi Z; Norhisham SN; Ramly N; Robert F; Suppiah J; Thayan R
EPI_ISL_6913874	Kuala Langat District Health Office	Institute for Medical Research, Infectious Disease Research Centre, National Institutes of Health, Ministry of Health Malaysia	Ahmad FA; Ahmad Fazilah NA; Anasir MI; Azizan MA; Kamel K; Mohamad Sukri MZ; Mohd Zawawi Z; Norhisham SN; Ramly N; Robert F; Rosli NR; Suppiah J; Thayan R
EPI_ISL_5850457	Kuala Lumpur General Hospital	Institute for Medical Research, Infectious Disease Research Centre, National Institutes of Health, Ministry of Health Malaysia	Ahmad FA; Ahmad Fazilah NA; Anasir MI; Azizan MA; Kamel K; Mohd Zawawi Z; Norhisham SN; Ramly N; Robert F; Suppiah J; Thayan R
EPI_ISL_4730449, EPI_ISL_4730450	Lahad Datu Hospital	Institute for Medical Research, Infectious Disease Research Centre, National Institutes of Health, Ministry of Health Malaysia	Ahmad FA; Ahmad Fazilah NA; Anasir MI; Azizan MA; Kamel K; Mohd Zawawi Z; Norhisham SN; Ramly N; Robert F; Suppiah J; Thayan R
EPI_ISL_5894062, EPI_ISL_5895216, EPI_ISL_5895217	Miri Hospital Molecular Diagnostic Lab, Miri	Institute of Health and Community Medicine	Amir Safuan bin Khamshah; Chan Chia Jui; Chua Hock Hin; David Perera; Ooi Mong How; Tonnii Sia Loong Loong; Wong Jyn Shan
EPI_ISL_6132051	PEGAWAI SAINS MIKROBIOLOGI C41 KONTRAK	Department of Medical Microbiology, Faculty of Medicine, University of Malaya; University of Malaya Medical Centre	I-Ching SAM; Jolene Yin Ling FU; Omar Khalilur Rahman; Yoke Fun Chan
EPI_ISL_5429412, EPI_ISL_5742654	PEJABAT KESIHATAN DAERAH BATU PAHAT	UKM Medical Molecular Biology Institute (UMBI)	Mira Farzana binti Mohamad Mokhtar
EPI_ISL_6567063, EPI_ISL_6567069, EPI_ISL_6567070, EPI_ISL_6567078, EPI_ISL_6567081	PEJABAT KESIHATAN DAERAH KOTA TINGGI	UKM Medical Molecular Biology Institute (UMBI)	Mira Farzana binti Mohamad Mokhtar
EPI_ISL_8090699	PEJABAT KESIHATAN DAERAH PASIR MAS	Tropical Infectious Diseases Research & Education Centre (TIDREC), Universiti Malaya	AsmaAnati CheMatSeri; Che-Norainon Yaacob; Jia-Yi Tan; Jo-Ern Wong; Kim-Kee Tan; Mulya-Mustika-Sari Zulkifli; Noor Syahida Azizan; Nur-Hidayana Mahfodz; Sazaly AbuBakar; Siti-Sarah Nor'e
EPI_ISL_6018054, EPI_ISL_6018059	PEJABAT KESIHATAN DAERAH TIMUR LAUT	iPROMISE, UiTM	Ariza Adnan; Fadzilah Mohd Nor; Lim Wai Feng; Mohd Asif Mohd Sukri; Mohd Nur Fakhruzzaman Noorizhab; Mohd Zaki Salleh; Sazzli Shahlan Kassim; Siti Farah Alwani Mohd Nawi; Siti Hamimah Sheikh Abdul Kadir; Teh Lay Kek; Wang Seok Mui
EPI_ISL_5450560	PEJABAT KESIHATAN KUANTAN	iPROMISE, UiTM	Ariza Adnan; Fadzilah Mohd Nor; Lim Wai Feng; Mohd Asif Mohd Sukri; Mohd Nur Fakhruzzaman Noorizhab; Mohd Zaki Salleh; Sazzli Shahlan Kassim; Siti Farah Alwani Mohd Nawi; Siti Hamimah Sheikh Abdul Kadir; Teh Lay Kek; Wang Seok Mui
EPI_ISL_5428861, EPI_ISL_5428865, EPI_ISL_5428872, EPI_ISL_5429416	PKD MELAKA TENGAH	UKM Medical Molecular Biology Institute (UMBI)	Mira Farzana binti Mohamad Mokhtar
EPI_ISL_5742666, EPI_ISL_5742698, EPI_ISL_5881280, EPI_ISL_5881282	PKD MERSING	UKM Medical Molecular Biology Institute (UMBI)	Mira Farzana binti Mohamad Mokhtar
EPI_ISL_7087489, EPI_ISL_7087534, EPI_ISL_7087746	PKD Manjung	Department of Medical Microbiology, Faculty of Medicine, University of Malaya; University of Malaya Medical Centre	I-Ching SAM; Jolene Yin Ling FU; Omar Khalilur Rahman; Yoke Fun Chan
EPI_ISL_5428879	PKD REMBAU	UKM Medical Molecular Biology Institute (UMBI)	Mira Farzana binti Mohamad Mokhtar
EPI_ISL_6017953, EPI_ISL_6017961, EPI_ISL_6017968	PKD SEBERANG PERAI SELATAN	iPROMISE, UiTM	Ariza Adnan; Fadzilah Mohd Nor; Lim Wai Feng; Mohd Asif Mohd Sukri; Mohd Nur Fakhruzzaman Noorizhab; Mohd Zaki Salleh; Sazzli Shahlan Kassim; Siti Farah Alwani Mohd Nawi; Siti Hamimah Sheikh Abdul Kadir; Teh Lay Kek; Wang Seok Mui
EPI_ISL_5450404, EPI_ISL_6017898, EPI_ISL_6712840, EPI_ISL_6712848	PKD SPT	iPROMISE, UiTM	Ariza Adnan; Fadzilah Mohd Nor; Lim Wai Feng; Mohd Asif Mohd Sukri; Mohd Nur Fakhruzzaman Noorizhab; Mohd Zaki Salleh; Sazzli Shahlan Kassim; Siti Farah Alwani Mohd Nawi; Siti Hamimah Sheikh Abdul Kadir; Teh Lay Kek; Wang Seok Mui
EPI_ISL_5022686, EPI_ISL_5417594, EPI_ISL_5417595, EPI_ISL_5417596, EPI_ISL_5428867, EPI_ISL_5428874	PKD TAMPIN	UKM Medical Molecular Biology Institute (UMBI)	Mira Farzana binti Mohamad Mokhtar
EPI_ISL_6017893	PKS SPT	iPROMISE, UiTM	Ariza Adnan; Fadzilah Mohd Nor; Lim Wai Feng; Mohd Asif Mohd Sukri; Mohd Nur Fakhruzzaman Noorizhab; Mohd Zaki Salleh; Sazzli Shahlan Kassim; Siti Farah Alwani Mohd Nawi; Siti Hamimah Sheikh Abdul Kadir; Teh Lay Kek; Wang Seok Mui
EPI_ISL_8090700	Pejabat Kesihatan Daerah Hulu Perak	Tropical Infectious Diseases Research & Education Centre (TIDREC), Universiti Malaya	AsmaAnati CheMatSeri; Che-Norainon Yaacob; Jia-Yi Tan; Jo-Ern Wong; Kim-Kee Tan; Mulya-Mustika-Sari Zulkifli; Noor Syahida Azizan; Nur-Hidayana Mahfodz; Sazaly AbuBakar; Siti-Sarah Nor'e
EPI_ISL_6825295	Pejabat Kesihatan Daerah Kota Marudu	Malaysia Genome Institute	Azrin Ahmad; Enizza Kasim; Irni Suhayu Sapian; Mohd Faizal Abu Bakar; Mohd Ghows Mohd Azzam.; Mohd Noor Mat Isa; Nor Azfa Johari; Nurhezreen Md Iqbal; Shamsidar Sopie; Siti Noraini Othman; Yusuf Muhammad Noor
EPI_ISL_6825291, EPI_ISL_6825292, EPI_ISL_6825293	Pejabat Kesihatan Daerah Kuala Penyu	Malaysia Genome Institute	Azrin Ahmad; Enizza Kasim; Irni Suhayu Sapian; Mohd Faizal Abu Bakar; Mohd Ghows Mohd Azzam.; Mohd Noor Mat Isa; Nor Azfa Johari; Nurhezreen Md Iqbal; Shamsidar Sopie; Siti Noraini Othman; Yusuf Muhammad Noor
EPI_ISL_8745593, EPI_ISL_8745594	Pejabat Kesihatan Daerah Kuala Penyu	Malaysia Genome and Vaccine Institute	Apical Scientific Sdn Bhd's Team; Azrin Ahmad; Carey Wee Kai Li; Enizza Kasim; Irni Suhayu Sapian; Jilian Michelle Wong Tzeling; Leong Wai Mun; Mohd Faizal Abu Bakar; Mohd Ghows Mohd Azzam.; Mohd Noor Mat Isa; Nor Azfa Johari; Nurhezreen Md Iqbal; Rose Iszati Ismet Nayan; Shamsidar Sopie; Siti Noraini Othman; Yusuf Muhammad Noor
EPI_ISL_6825277	Pejabat Kesihatan Daerah Kudat	Malaysia Genome Institute	Azrin Ahmad; Enizza Kasim; Irni Suhayu Sapian; Mohd Faizal Abu Bakar; Mohd Ghows Mohd Azzam.; Mohd Noor Mat Isa; Nor Azfa Johari; Nurhezreen Md Iqbal; Shamsidar Sopie; Siti Noraini Othman; Yusuf Muhammad Noor
EPI_ISL_6825278	Pejabat Kesihatan Daerah Kunak	Malaysia Genome Institute	Azrin Ahmad; Enizza Kasim; Irni Suhayu Sapian; Mohd Faizal Abu Bakar; Mohd Ghows Mohd Azzam.; Mohd Noor Mat Isa; Nor Azfa Johari; Nurhezreen Md Iqbal; Shamsidar Sopie; Siti Noraini Othman; Yusuf Muhammad Noor
EPI_ISL_6825289, EPI_ISL_6825290	Pejabat Kesihatan Daerah Pitas	Malaysia Genome Institute	Azrin Ahmad; Enizza Kasim; Irni Suhayu Sapian; Mohd Faizal Abu Bakar; Mohd Ghows Mohd Azzam.; Mohd Noor Mat Isa; Nor Azfa Johari; Nurhezreen Md Iqbal; Shamsidar Sopie; Siti Noraini Othman; Yusuf Muhammad Noor
EPI_ISL_8745592	Pejabat Kesihatan Daerah Pitas	Malaysia Genome and Vaccine Institute	Apical Scientific Sdn Bhd's Team; Azrin Ahmad; Carey Wee Kai Li; Enizza Kasim; Irni Suhayu Sapian; Jilian Michelle Wong Tzeling; Leong Wai Mun; Mohd Faizal Abu Bakar; Mohd Ghows Mohd Azzam.; Mohd Noor Mat Isa; Nor Azfa Johari; Nurhezreen Md Iqbal; Rose Iszati Ismet Nayan; Shamsidar Sopie; Siti Noraini Othman; Yusuf Muhammad Noor
EPI_ISL_8745595	Pejabat Kesihatan Daerah Sipitang	Malaysia Genome and Vaccine Institute	Apical Scientific Sdn Bhd's Team; Azrin Ahmad; Carey Wee Kai Li; Enizza Kasim; Irni Suhayu Sapian; Jilian Michelle Wong Tzeling; Leong Wai Mun; Mohd Faizal Abu Bakar; Mohd Ghows Mohd Azzam.; Mohd Noor Mat Isa; Nor Azfa Johari; Nurhezreen Md Iqbal; Rose Iszati Ismet Nayan; Shamsidar Sopie; Siti Noraini Othman; Yusuf Muhammad Noor
EPI_ISL_8745591	Pejabat Kesihatan Kawasan Beaufort	Malaysia Genome and Vaccine Institute	Apical Scientific Sdn Bhd's Team; Azrin Ahmad; Carey Wee Kai Li; Enizza Kasim; Irni Suhayu Sapian; Jilian Michelle Wong Tzeling; Leong Wai Mun; Mohd Faizal Abu Bakar; Mohd Ghows Mohd Azzam.; Mohd Noor Mat Isa; Nor Azfa Johari; Nurhezreen Md Iqbal; Rose Iszati Ismet Nayan; Shamsidar Sopie; Siti Noraini Othman; Yusuf Muhammad Noor
EPI_ISL_6342941	Pejabat Kesihatan Kawasan Kota Kinabalu	Institute for Medical Research, Infectious Disease Research Centre, National Institutes of Health, Ministry of Health Malaysia	Ahmad FA; Ahmad Fazilah NA; Anasir MI; Azizan MA; Kamel K; Mohd Zawawi Z; Norhisham SN; Ramly N; Robert F; Suppiah J; Thayan R
EPI_ISL_6825287, EPI_ISL_6825288	Pejabat Kesihatan Kawasan Tawau	Malaysia Genome Institute	Azrin Ahmad; Enizza Kasim; Irni Suhayu Sapian; Mohd Faizal Abu Bakar; Mohd Ghows Mohd Azzam.; Mohd Noor Mat Isa; Nor Azfa Johari; Nurhezreen Md Iqbal; Shamsidar Sopie; Siti Noraini Othman; Yusuf Muhammad Noor
EPI_ISL_5804668, EPI_ISL_5852400, EPI_ISL_5852487	Pitas District Health Office	Institute for Medical Research, Infectious Disease Research Centre, National Institutes of Health, Ministry of Health Malaysia	Ahmad FA; Ahmad Fazilah NA; Anasir MI; Azizan MA; Kamel K; Mohd Zawawi Z; Norhisham SN; Ramly N; Robert F; Suppiah J; Thayan R
EPI_ISL_7952083	Public Health Laboratory, Ipoh	Public Health Laboratory Ipoh Disease Section	A Nisha; Diyana NM; MH Chew; Norhidayah A; Sabrina R; Syawal A; TAHIR H; Yusof TY
EPI_ISL_5850346	Ranau District Health Office	Institute for Medical Research, Infectious Disease Research Centre, National Institutes of Health, Ministry of Health Malaysia	Ahmad FA; Ahmad Fazilah NA; Anasir MI; Azizan MA; Kamel K; Mohd Zawawi Z; Norhisham SN; Ramly N; Robert F; Suppiah J; Thayan R
EPI_ISL_5893741, EPI_ISL_5895075, EPI_ISL_5895080, EPI_ISL_5895091, EPI_ISL_5895104, EPI_ISL_5895112, EPI_ISL_5895117, EPI_ISL_5895125, EPI_ISL_5895133, EPI_ISL_5895266, EPI_ISL_5896015, EPI_ISL_6969176, EPI_ISL_6969188, EPI_ISL_6969196, EPI_ISL_6969206, EPI_ISL_6969219, EPI_ISL_6969229, EPI_ISL_6969236, EPI_ISL_6969248, EPI_ISL_6969260, EPI_ISL_6969266, EPI_ISL_6970406			
see above	Rejang Medical Centre (Sibu)	Institute of Health and Community Medicine	Chan Chia Jui; Chua Hock Hin; David Perera; Ooi Mong How; Tonnii Sia Loong Loong; Wong Jyn Shan
EPI_ISL_5893725, EPI_ISL_5893746, EPI_ISL_5893773, EPI_ISL_5893792, EPI_ISL_5893839, EPI_ISL_5893862, EPI_ISL_5893867, EPI_ISL_5894205, EPI_ISL_5894211, EPI_ISL_5894216, EPI_ISL_5894224, EPI_ISL_5894231, EPI_ISL_5894240, EPI_ISL_5894246, EPI_ISL_5894253, EPI_ISL_5894260, EPI_ISL_5894265, EPI_ISL_5894272, EPI_ISL_5894280, EPI_ISL_5894286, EPI_ISL_5894293, EPI_ISL_5894303, EPI_ISL_5894306, EPI_ISL_5894312, EPI_ISL_5894321, EPI_ISL_5894328, EPI_ISL_5894593, EPI_ISL_5894602, EPI_ISL_5894612, EPI_ISL_5894616, EPI_ISL_5894622, EPI_ISL_5894636, EPI_ISL_5894643, EPI_ISL_5895541, EPI_ISL_5895548, EPI_ISL_5895555, EPI_ISL_5895568, EPI_ISL_5895580, EPI_ISL_5895590, EPI_ISL_5895597, EPI_ISL_5895600, EPI_ISL_5895604, EPI_ISL_5895611, EPI_ISL_5895617, EPI_ISL_5895624, EPI_ISL_5895631, EPI_ISL_5895639, EPI_ISL_5895647, EPI_ISL_5895659, EPI_ISL_5895669, EPI_ISL_5896050, EPI_ISL_5896058, EPI_ISL_5896065, EPI_ISL_6586286, EPI_ISL_6586287, EPI_ISL_6586288, EPI_ISL_6586289, EPI_ISL_6586290, EPI_ISL_6586291, EPI_ISL_6586292, EPI_ISL_6586293, EPI_ISL_6586294, EPI_ISL_6586296, EPI_ISL_6586297, EPI_ISL_6586298, EPI_ISL_6586299, EPI_ISL_6586300, EPI_ISL_6586301, EPI_ISL_6586302, EPI_ISL_6586303, EPI_ISL_6586304, EPI_ISL_6586305, EPI_ISL_6586306, EPI_ISL_6586307, EPI_ISL_6586308, EPI_ISL_6586309, EPI_ISL_6586310, EPI_ISL_6586311, EPI_ISL_6586312, EPI_ISL_6586313, EPI_ISL_6586314, EPI_ISL_6586315, EPI_ISL_6586316, EPI_ISL_6586317, EPI_ISL_6586318, EPI_ISL_6586319, EPI_ISL_6586320, EPI_ISL_6586321, EPI_ISL_6586322, EPI_ISL_6586323, EPI_ISL_6586324, EPI_ISL_6586325, EPI_ISL_6586326, EPI_ISL_6586327, EPI_ISL_6586329, EPI_ISL_6586330, EPI_ISL_6586332, EPI_ISL_6586333, EPI_ISL_6586335, EPI_ISL_6586336, EPI_ISL_6586337, EPI_ISL_6586339, EPI_ISL_6586340, EPI_ISL_6586341, EPI_ISL_6586342, EPI_ISL_6586343, EPI_ISL_6586344, EPI_ISL_6586346, EPI_ISL_6586348, EPI_ISL_6586350, EPI_ISL_6586351, EPI_ISL_6586352, EPI_ISL_6586353, EPI_ISL_6586354, EPI_ISL_6586420, EPI_ISL_6586421, EPI_ISL_6970443, EPI_ISL_7473122, EPI_ISL_7473123, EPI_ISL_7473124, EPI_ISL_7473125, EPI_ISL_7473126			
see above	Sarawak General Hospital (Kuching)	Institute of Health and Community Medicine	Chan Chia Jui; Chua Hock Hin; David Perera; Ooi Mong How; Tonnii Sia Loong Loong; Wong Jyn Shan
EPI_ISL_6913883, EPI_ISL_6913889, EPI_ISL_6913899, EPI_ISL_6913909, EPI_ISL_6913914, EPI_ISL_6913945, EPI_ISL_6913946, EPI_ISL_6914168			
see above	Selangor State Health Department	Institute for Medical Research, Infectious Disease Research Centre, National Institutes of Health, Ministry of Health Malaysia	Ahmad FA; Ahmad Fazilah NA; Anasir MI; Azizan MA; Kamel K; Mohamad Sukri MZ; Mohd Zawawi Z; Norhisham SN; Ramly N; Robert F; Rosli NR; Suppiah J; Thayan R
EPI_ISL_5850359, EPI_ISL_5850364, EPI_ISL_5852401	Sipitang District Health Office	Institute for Medical Research, Infectious Disease Research Centre, National Institutes of Health, Ministry of Health Malaysia	Ahmad FA; Ahmad Fazilah NA; Anasir MI; Azizan MA; Kamel K; Mohd Zawawi Z; Norhisham SN; Ramly N; Robert F; Suppiah J; Thayan R

EPI_ISL_5852404, EPI_ISL_5852407, EPI_ISL_6773990, EPI_ISL_6773991	Sungai Buloh Hospital	Institute for Medical Research, Infectious Disease Research Centre, National Institutes of Health, Ministry of Health Malaysia	Ahmad FA; Ahmad Fazilah NA; Anasir MI; Azizan MA; Kamel K; Mohamad Sukri MZ; Mohd Zawawi Z; Norhisham SN; Ramly N; Robert F; Rosli NR; Suppiah J; Thayan R
EPI_ISL_6132055, EPI_ISL_6132064, EPI_ISL_8254274	UMMC	Department of Medical Microbiology, Faculty of Medicine, University of Malaya; University of Malaya Medical Centre	I-Ching SAM; Jolene Yin Ling FU; Omar Khalilur Rahman; Yoke Fun Chan
EPI_ISL_8090664	UNIT KESIHATAN AWAM ,UKA . HRPZ	Tropical Infectious Diseases Research & Education Centre (TIDREC), Universiti Malaya	AsmaAnati CheMatSeri; Che-Norainon Yaacob; Jia-Yi Tan; Jo-Ern Wong; Kim-Kee Tan; Mulya-Mustika-Sari Zulkifli; Noor Syahida Azizan; Nur-Hidayana Mahfodz; Sazaly AbuBakar; Siti-Sarah Nor’e
EPI_ISL_5429418, EPI_ISL_5429419, EPI_ISL_5429422, EPI_ISL_5429426, EPI_ISL_5429428, EPI_ISL_5429431	UNIT KESIHATAN AWAM HEBHK	UKM Medical Molecular Biology Institute (UMBI)	Mira Farzana binti Mohamad Mokhtar
EPI_ISL_5429413	UNIT KESIHATAN PERSEKITARAN & KESIHATAN PEKERJAAN, HOSP SULTANAH AMINAH JOHOR BAHRU, JOHOR	UKM Medical Molecular Biology Institute (UMBI)	Mira Farzana binti Mohamad Mokhtar
EPI_ISL_5022690	UNIT KPAS, PKD MELAKA TENGAH	UKM Medical Molecular Biology Institute (UMBI)	Mira Farzana binti Mohamad Mokhtar
EPI_ISL_5450192	Unit Perubatan Forensik, Hospital Kepala Batas	iPROMISE, UiTM	Ariza Adnan; Fadzilah Mohd Nor; Lim Wai Feng; Mohd Asif Mohd Sukri; Mohd Nur Fakhruzzaman Noorizhab; Mohd Zaki Salleh; Sazzli Shahlan Kassim; Siti Farah Alwani Mohd Nawi; Siti Hamimah Sheikh Abdul Kadir; Teh Lay Kek; Wang Seok Mui
EPI_ISL_5450391	Unit Perubatan Forensik, Hospital Seberang Jaya	iPROMISE, UiTM	Ariza Adnan; Fadzilah Mohd Nor; Lim Wai Feng; Mohd Asif Mohd Sukri; Mohd Nur Fakhruzzaman Noorizhab; Mohd Zaki Salleh; Sazzli Shahlan Kassim; Siti Farah Alwani Mohd Nawi; Siti Hamimah Sheikh Abdul Kadir; Teh Lay Kek; Wang Seok Mui
EPI_ISL_5022681	hospital pakar sultanah fatimah muar	UKM Medical Molecular Biology Institute (UMBI)	Mira Farzana binti Mohamad Mokhtar
EPI_ISL_6131990	unit keselamatan dan kesihatan pekerjaan	Department of Medical Microbiology, Faculty of Medicine, University of Malaya; University of Malaya Medical Centre	I-Ching SAM; Jolene Yin Ling FU; Omar Khalilur Rahman; Yoke Fun Chan

We gratefully acknowledge the following Authors from the Originating laboratories responsible for obtaining the specimens, as well as the Submitting laboratories where the genome data were generated and shared via GISAID, on which this research is based.

All Submitters of data may be contacted directly via www.gisaid.org

Authors are sorted alphabetically.

Accession ID	Originating Laboratory	Submitting Laboratory	Authors
EPI_ISL_5850453, EPI_ISL_5850456	Ampang Hospital	Institute for Medical Research, Infectious Disease Research Centre, National Institutes of Health, Ministry of Health Malaysia	Ahmad FA; Ahmad Fazilah NA; Anasir MI; Azizan MA; Kamel K; Mohd Zawawi Z; Norhisham SN; Ramly N; Robert F; Suppiah J; Thayan R
EPI_ISL_5894078, EPI_ISL_5894085, EPI_ISL_5894089, EPI_ISL_5894097, EPI_ISL_5896020, EPI_ISL_5896028, EPI_ISL_5896035, EPI_ISL_5896043, EPI_ISL_6586487, EPI_ISL_6586488, EPI_ISL_6586489, EPI_ISL_6586490, EPI_ISL_6586491, EPI_ISL_6586492, EPI_ISL_6586493, EPI_ISL_6586494, EPI_ISL_6586495, EPI_ISL_6586496, EPI_ISL_6586497, EPI_ISL_6586498, EPI_ISL_6586499, EPI_ISL_6586500, EPI_ISL_6586501, EPI_ISL_6586502, EPI_ISL_6586503, EPI_ISL_6586504, EPI_ISL_6586505, EPI_ISL_6586508			
see above	Bintulu Hospital PCR Lab, Bintulu	Institute of Health and Community Medicine	Chan Chia Jui; Chua Hock Hin; David Perera; Ooi Mong How; Tan Lee See; Tonnii Sia Loong Loong; Wong Jyn Shan
EPI_ISL_7472868, EPI_ISL_7472869, EPI_ISL_7472870, EPI_ISL_7472871, EPI_ISL_7472872, EPI_ISL_7472873, EPI_ISL_7472874, EPI_ISL_7472875, EPI_ISL_7473059, EPI_ISL_7473060, EPI_ISL_7473061, EPI_ISL_7473062, EPI_ISL_7473063, EPI_ISL_7473064, EPI_ISL_7473065, EPI_ISL_7473066			
see above	Bintulu Medical Centre (Bintulu)	Institute of Health and Community Medicine	Chan Chia Jui; Chua Hock Hin; David Perera; Ooi Mong How; Tonnii Sia Loong Loong; Wong Jyn Shan
EPI_ISL_6586411, EPI_ISL_6586412, EPI_ISL_6586413, EPI_ISL_6586414, EPI_ISL_6586415, EPI_ISL_6586416, EPI_ISL_6586417, EPI_ISL_6586591, EPI_ISL_6586592, EPI_ISL_6586593, EPI_ISL_6586594, EPI_ISL_6586595, EPI_ISL_6586596, EPI_ISL_6586597, EPI_ISL_7473051, EPI_ISL_7473052, EPI_ISL_11804907			
see above	Borneo Medical Centre (Kuching)	Institute of Health and Community Medicine	Chan Chia Jui; Chua Hock Hin; David Perera; Ooi Mong How; Tonnii Sia Loong Loong; Wong Jyn Shan
EPI_ISL_6018100, EPI_ISL_6018108	CAC KD Guar Sanji	iPROMISE, UiTM	Ariza Adnan; Fadzilah Mohd Nor; Lim Wai Feng; Mohd Asif Mohd Sukri; Mohd Nur Fakhruzzaman Noorizhab; Mohd Zaki Salleh; Sazzli Shahlan Kassim; Siti Farah Alwani Mohd Nawi; Siti Hamimah Sheikh Abdul Kadir; Teh Lay Kek; Wang Seok Mui
EPI_ISL_10070554, EPI_ISL_10070708, EPI_ISL_10070709	Department of Medical Microbiology, Hospital Pengajar Universiti Putra Malaysia	Department of Medical Microbiology, Hospital Pengajar Universiti Putra Malaysia	Afiqah Adzmi; Amiza Azmi; Azmiza Syawani Jasni; Hui Yee Chee; Leslie Thian Lung Than; Muadz Mohtar; Muhammad Mohd Isa; Narcisse MS Joseph; Niazlin Mohd Taib; Noor Hazirah Noor Azhari; Norlaila; Nur Raihana Ithnin; Nurul Huda Mohamed Rashidi; Nurul Nadiah Ismail; Rosni Ibrahim; Rukman Awang Hamat; Sallehhudin; Siti Norbaya Masri; Siti Zulaikha Zakariah; Syafinaz Amin Nordin; Tengku Zetty Maztura Tengku Jamaluddin; Zamberi Sekawi
EPI_ISL_8090704	HEPB IPOH	Tropical Infectious Diseases Research & Education Centre (TIDREC), Universiti Malaya	AsmaAnati CheMatSeri; Che-Norainon Yaacob; Jia-Yi Tan; Jo-Ern Wong; Kim-Kee Tan; Mulya-Mustika-Sari Zulkifli; Noor Syahida Azizan; Nur-Hidayana Mahfodz; Sazaly AbuBakar; Siti-Sarah Nor'e
EPI_ISL_5881271	HOSPITA SULTANAH NORA ISMAIL BATU PAHAT JOHOR	UKM Medical Molecular Biology Institute (UMBI)	Mira Farzana binti Mohamad Mokhtar
EPI_ISL_6017856	HOSPITAL BUKIT MERTAJAM	iPROMISE, UiTM	Ariza Adnan; Fadzilah Mohd Nor; Lim Wai Feng; Mohd Asif Mohd Sukri; Mohd Nur Fakhruzzaman Noorizhab; Mohd Zaki Salleh; Sazzli Shahlan Kassim; Siti Farah Alwani Mohd Nawi; Siti Hamimah Sheikh Abdul Kadir; Teh Lay Kek; Wang Seok Mui
EPI_ISL_6017904	HOSPITAL SEBERANG JAYA	iPROMISE, UiTM	Ariza Adnan; Fadzilah Mohd Nor; Lim Wai Feng; Mohd Asif Mohd Sukri; Mohd Nur Fakhruzzaman Noorizhab; Mohd Zaki Salleh; Sazzli Shahlan Kassim; Siti Farah Alwani Mohd Nawi; Siti Hamimah Sheikh Abdul Kadir; Teh Lay Kek; Wang Seok Mui
EPI_ISL_8090731	HOSPITAL SULTAN ISMAIL PETRA	Tropical Infectious Diseases Research & Education Centre (TIDREC), Universiti Malaya	AsmaAnati CheMatSeri; Che-Norainon Yaacob; Jia-Yi Tan; Jo-Ern Wong; Kim-Kee Tan; Mulya-Mustika-Sari Zulkifli; Noor Syahida Azizan; Nur-Hidayana Mahfodz; Sazaly AbuBakar; Siti-Sarah Nor'e
EPI_ISL_6452603	HOSPITAL SULTANAH NORA ISMAIL BATU PAHAT JOHOR	UKM Medical Molecular Biology Institute (UMBI)	Mira Farzana binti Mohamad Mokhtar
EPI_ISL_6132061	HOSPITAL TUANKU FAUZIAH	Department of Medical Microbiology, Faculty of Medicine, University of Malaya; University of Malaya Medical Centre	I-Ching SAM; Jolene Yin Ling FU; Omar Khalilur Rahman; Yoke Fun Chan
EPI_ISL_6712800	HOSPITAL TUANKU FAUZIAH, KANGAR	iPROMISE, UiTM	Ariza Adnan; Fadzilah Mohd Nor; Lim Wai Feng; Mohd Asif Mohd Sukri; Mohd Nur Fakhruzzaman Noorizhab; Mohd Zaki Salleh; Sazzli Shahlan Kassim; Siti Farah Alwani Mohd Nawi; Siti Hamimah Sheikh Abdul Kadir; Teh Lay Kek; Wang Seok Mui
EPI_ISL_6131986, EPI_ISL_6132043, EPI_ISL_6132057, EPI_ISL_7086681	HRPB	Department of Medical Microbiology, Faculty of Medicine, University of Malaya; University of Malaya Medical Centre	I-Ching SAM; Jolene Yin Ling FU; Omar Khalilur Rahman; Yoke Fun Chan
EPI_ISL_8090636, EPI_ISL_8090701, EPI_ISL_8090717, EPI_ISL_8090718, EPI_ISL_8090719, EPI_ISL_8090720, EPI_ISL_8090721, EPI_ISL_8090725, EPI_ISL_8090727, EPI_ISL_8090730			
see above	HRPB	Tropical Infectious Diseases Research & Education Centre (TIDREC), Universiti Malaya	AsmaAnati CheMatSeri; Che-Norainon Yaacob; Jia-Yi Tan; Jo-Ern Wong; Kim-Kee Tan; Mulya-Mustika-Sari Zulkifli; Noor Syahida Azizan; Nur-Hidayana Mahfodz; Sazaly AbuBakar; Siti-Sarah Nor'e
EPI_ISL_8090702, EPI_ISL_8090703, EPI_ISL_8090707, EPI_ISL_8090708, EPI_ISL_8090709, EPI_ISL_8090710, EPI_ISL_8090711, EPI_ISL_8090712			
see above	HRPB IPOH	Tropical Infectious Diseases Research & Education Centre (TIDREC), Universiti Malaya	AsmaAnati CheMatSeri; Che-Norainon Yaacob; Jia-Yi Tan; Jo-Ern Wong; Kim-Kee Tan; Mulya-Mustika-Sari Zulkifli; Noor Syahida Azizan; Nur-Hidayana Mahfodz; Sazaly AbuBakar; Siti-Sarah Nor'e
EPI_ISL_8090705	HRPB, IPOH	Tropical Infectious Diseases Research & Education Centre (TIDREC), Universiti Malaya	AsmaAnati CheMatSeri; Che-Norainon Yaacob; Jia-Yi Tan; Jo-Ern Wong; Kim-Kee Tan; Mulya-Mustika-Sari Zulkifli; Noor Syahida Azizan; Nur-Hidayana Mahfodz; Sazaly AbuBakar; Siti-Sarah Nor'e
EPI_ISL_11010500, EPI_ISL_11010501	HRPZ II	Tropical Infectious Diseases Research & Education Centre (TIDREC), Universiti Malaya	AsmaAnati CheMatSeri; Che-Norainon Yaacob; Jia-Yi Tan; Jo-Ern Wong; Kim-Kee Tan; Mulya-Mustika-Sari Zulkifli; Noor Syahida Azizan; Nur-Hidayana Mahfodz; Sazaly AbuBakar; Siti-Sarah Nor'e
EPI_ISL_6825285, EPI_ISL_6825286	Hospital Ampang	Malaysia Genome Institute	Azrin Ahmad; Enizza Kasim; Irni Suhayu Sapian; Mohd Faizal Abu Bakar; Mohd Ghows Mohd Azzam.; Mohd Noor Mat Isa; Nor Azfa Johari; Nurhezreen Md Iqbal; Shamsidar Sopie; Siti Noraini Othman; Yusuf Muhammad Noor
EPI_ISL_5022670, EPI_ISL_5022673, EPI_ISL_5022675, EPI_ISL_5022682, EPI_ISL_5022688, EPI_ISL_5417593, EPI_ISL_5417636, EPI_ISL_5417637, EPI_ISL_5428860, EPI_ISL_5428862, EPI_ISL_5428873, EPI_ISL_5428878			
see above	Hospital Canselor Tuanku Muhriz (HCTM)	UKM Medical Molecular Biology Institute (UMBI)	Mira Farzana binti Mohamad Mokhtar
EPI_ISL_8745581	Hospital Keningau	Malaysia Genome and Vaccine Institute	Apical Scientific Sdn Bhd's Team; Azrin Ahmad; Carey Wee Kai Li; Enizza Kasim; Irni Suhayu Sapian; Jilian Michelle Wong Tzeling; Leong Wai Mun; Mohd Faizal Abu Bakar; Mohd Ghows Mohd Azzam.; Mohd Noor Mat Isa; Nor Azfa Johari; Nurhezreen Md Iqbal; Rose Iszati Ismet Nayan; Shamsidar Sopie; Siti Noraini Othman; Yusuf Muhammad Noor
EPI_ISL_6712877, EPI_ISL_6964622	Hospital Kepala Batas	iPROMISE, UiTM	Ariza Adnan; Fadzilah Mohd Nor; Lim Wai Feng; Mohd Asif Mohd Sukri; Mohd Nur Fakhruzzaman Noorizhab; Mohd Zaki Salleh; Sazzli Shahlan Kassim; Siti Farah Alwani Mohd Nawi; Siti Hamimah Sheikh Abdul Kadir; Teh Lay Kek; Wang Seok Mui
EPI_ISL_7260009	Hospital Lahad Datu	Malaysia Genome Institute	Azrin Ahmad; Enizza Kasim; Irni Suhayu Sapian; Mohd Faizal Abu Bakar; Mohd Ghows Mohd Azzam.; Mohd Noor Mat Isa; Nor Azfa Johari; Nurhezreen Md Iqbal; Shamsidar Sopie; Siti Noraini Othman; Yusuf Muhammad Noor
EPI_ISL_6712882, EPI_ISL_6712887	Hospital Pulau Pinang	iPROMISE, UiTM	Ariza Adnan; Fadzilah Mohd Nor; Lim Wai Feng; Mohd Asif Mohd Sukri; Mohd Nur Fakhruzzaman Noorizhab; Mohd Zaki Salleh; Sazzli Shahlan Kassim; Siti Farah Alwani Mohd Nawi; Siti Hamimah Sheikh Abdul Kadir; Teh Lay Kek; Wang Seok Mui
EPI_ISL_6017949	Hospital Seberang Jaya	iPROMISE, UiTM	Ariza Adnan; Fadzilah Mohd Nor; Lim Wai Feng; Mohd Asif Mohd Sukri; Mohd Nur Fakhruzzaman Noorizhab; Mohd Zaki Salleh; Sazzli Shahlan Kassim; Siti Farah Alwani Mohd Nawi; Siti Hamimah Sheikh Abdul Kadir; Teh Lay Kek; Wang Seok Mui
EPI_ISL_7087671	Hospital Sultanah Bahiyah	Department of Medical Microbiology, Faculty of Medicine, University of Malaya; University of Malaya Medical Centre	I-Ching SAM; Jolene Yin Ling FU; Omar Khalilur Rahman; Yoke Fun Chan
EPI_ISL_8090686	Hospital Sultanah Bahiyah	Tropical Infectious Diseases Research & Education Centre (TIDREC), Universiti Malaya	AsmaAnati CheMatSeri; Che-Norainon Yaacob; Jia-Yi Tan; Jo-Ern Wong; Kim-Kee Tan; Mulya-Mustika-Sari Zulkifli; Noor Syahida Azizan; Nur-Hidayana Mahfodz; Sazaly AbuBakar; Siti-Sarah Nor'e
EPI_ISL_6825280, EPI_ISL_6825281, EPI_ISL_6825282, EPI_ISL_6825283, EPI_ISL_6825284	Hospital Sungai Buloh	Malaysia Genome Institute	Azrin Ahmad; Enizza Kasim; Irni Suhayu Sapian; Mohd Faizal Abu Bakar; Mohd Ghows Mohd Azzam.; Mohd Noor Mat Isa; Nor Azfa Johari; Nurhezreen Md Iqbal; Shamsidar Sopie; Siti Noraini Othman; Yusuf Muhammad Noor
EPI_ISL_6452618	INSTITUT BIOLOGI MOLEKUL PERUBATAN UKM (UMBI)	UKM Medical Molecular Biology Institute (UMBI)	Mira Farzana binti Mohamad Mokhtar
EPI_ISL_7119321, EPI_ISL_7119346, EPI_ISL_7119375, EPI_ISL_7119395, EPI_ISL_7119418, EPI_ISL_7119433, EPI_ISL_7119448, EPI_ISL_7119465, EPI_ISL_7119489, EPI_ISL_7119497, EPI_ISL_7119520, EPI_ISL_7119534, EPI_ISL_11078696, EPI_ISL_11078703, EPI_ISL_11078707, EPI_ISL_11078743, EPI_ISL_11078746, EPI_ISL_11078748, EPI_ISL_11078767, EPI_ISL_11078770, EPI_ISL_11078778, EPI_ISL_11078779, EPI_ISL_11078784, EPI_ISL_11078788			
see above	Institut Biologi Molekul Perubatan UKM (UMBI)	UKM Medical Molecular Biology Institute (UMBI)	Ab Mutalib; Abd Ghafar; Ishak; Jamal; Khairun Nur; Khairun Nur Abd Ghafar; Md Shahri; Mira Farzana; Mira Farzana Mohamad Mokhtar; Mohamad Mokhtar; Mohamed Rasheed; Mohd Yunos; Mohd Yusuf; Muhammad Azami; Muhiddin; Muhiddin Ishak; Nor Azi; Nor Azila Muhammad Azami; Nur Alyaa Afifah; Nur Alyaa Afifah Md Shahri; Nurul Syakima; Nurul Syakima Ab Mutalib; Rahman; Rahman Jamal; Ryia Illani; Ryia Illani Mohd Yunos; Siti Nur Hasanah; Siti Nur Hasanah Mohd Yusuf; Zahirrah Begam; Zahirrah Begam Mohamed Rasheed
EPI_ISL_6017943	JABATAN FORENSIK	iPROMISE, UiTM	Ariza Adnan; Fadzilah Mohd Nor; Lim Wai Feng; Mohd Asif Mohd Sukri; Mohd Nur Fakhruzzaman Noorizhab; Mohd Zaki Salleh; Sazzli Shahlan Kassim; Siti Farah Alwani Mohd Nawi; Siti Hamimah Sheikh Abdul Kadir; Teh Lay Kek; Wang Seok Mui
EPI_ISL_6132006	JABATAN PATOLOGI, HRPZ II	Department of Medical Microbiology, Faculty of Medicine, University of Malaya; University of Malaya Medical Centre	I-Ching SAM; Jolene Yin Ling FU; Omar Khalilur Rahman; Yoke Fun Chan
EPI_ISL_6017983, EPI_ISL_6712901, EPI_ISL_6712910, EPI_ISL_6712914, EPI_ISL_6712922, EPI_ISL_6712930, EPI_ISL_6712936, EPI_ISL_6712949, EPI_ISL_6712967, EPI_ISL_6712972, EPI_ISL_6712990, EPI_ISL_6712995, EPI_ISL_6964435, EPI_ISL_6964442, EPI_ISL_6964450, EPI_ISL_6964457, EPI_ISL_6964612			
see above	JKN PAHANG	iPROMISE, UiTM	Ariza Adnan; Fadzilah Mohd Nor; Lim Wai Feng; Mohd Asif Mohd Sukri; Mohd Nur Fakhruzzaman Noorizhab; Mohd Zaki Salleh; Sazzli Shahlan Kassim; Siti Farah Alwani Mohd Nawi; Siti Hamimah Sheikh Abdul Kadir; Teh Lay Kek; Wang Seok Mui
EPI_ISL_6132005, EPI_ISL_7086613, EPI_ISL_8254290	Jabatan Forensik Hospital Sultanah Bahiyah	Department of Medical Microbiology, Faculty of Medicine, University of Malaya; University of Malaya Medical Centre	I-Ching SAM; Jolene Yin Ling FU; Omar Khalilur Rahman; Yoke Fun Chan
EPI_ISL_6712869	Jabatan Perubatan Forensik	iPROMISE, UiTM	Ariza Adnan; Fadzilah Mohd Nor; Lim Wai Feng; Mohd Asif Mohd Sukri; Mohd Nur Fakhruzzaman Noorizhab; Mohd Zaki Salleh; Sazzli Shahlan Kassim; Siti Farah Alwani Mohd Nawi; Siti Hamimah Sheikh Abdul Kadir; Teh Lay Kek; Wang Seok Mui
EPI_ISL_6017877	KLINIK KESIHATAN MEMPAGA	iPROMISE, UiTM	Ariza Adnan; Fadzilah Mohd Nor; Lim Wai Feng; Mohd Asif Mohd Sukri; Mohd Nur Fakhruzzaman Noorizhab; Mohd Zaki Salleh; Sazzli Shahlan Kassim; Siti Farah Alwani Mohd Nawi; Siti Hamimah Sheikh Abdul Kadir; Teh Lay Kek; Wang Seok Mui
EPI_ISL_6017882	KLINIK KESIHATAN MEMPAGA, PEJABAT KESIHATAN DAERAH BENTONG	iPROMISE, UiTM	Ariza Adnan; Fadzilah Mohd Nor; Lim Wai Feng; Mohd Asif Mohd Sukri; Mohd Nur Fakhruzzaman Noorizhab; Mohd Zaki Salleh; Sazzli Shahlan Kassim; Siti Farah Alwani Mohd Nawi; Siti Hamimah Sheikh Abdul Kadir; Teh Lay Kek; Wang Seok Mui
EPI_ISL_9205286, EPI_ISL_9205488	Kajang Hospital	Institute for Medical Research, Infectious Disease Research Centre, National Institutes of Health, Ministry of Health Malaysia	Ahmad FA; Ahmad Fazilah NA; Anasir MI; Azizan MA; Kamel K; Mohamad Sukri MZ; Norhisham SN; Ramly N; Robert F; Rosli NR; Suppiah J; Thayan R

EPI_ISL_5852386	Kuala Kubu Bharu Hospital	Institute for Medical Research, Infectious Disease Research Centre, National Institutes of Health, Ministry of Health Malaysia	Ahmad FA; Ahmad Fazilah NA; Anasir MI; Azizan MA; Kamel K; Mohd Zawawi Z; Norhisham SN; Ramly N; Robert F; Suppiah J; Thayan R
EPI_ISL_5850459, EPI_ISL_5850461, EPI_ISL_5850481, EPI_ISL_5852155	Kuala Lumpur General Hospital	Institute for Medical Research, Infectious Disease Research Centre, National Institutes of Health, Ministry of Health Malaysia	Ahmad FA; Ahmad Fazilah NA; Anasir MI; Azizan MA; Kamel K; Mohd Zawawi Z; Norhisham SN; Ramly N; Robert F; Suppiah J; Thayan R
EPI_ISL_6422377, EPI_ISL_6422435	Kudat Area Health Office	Institute for Medical Research, Infectious Disease Research Centre, National Institutes of Health, Ministry of Health Malaysia	Ahmad FA; Ahmad Fazilah NA; Anasir MI; Azizan MA; Kamel K; Mohd Zawawi Z; Norhisham SN; Ramly N; Robert F; Suppiah J; Thayan R
EPI_ISL_6774006	Labuan State Health Department	Institute for Medical Research, Infectious Disease Research Centre, National Institutes of Health, Ministry of Health Malaysia	Ahmad FA; Ahmad Fazilah NA; Anasir MI; Azizan MA; Kamel K; Mohamad Sukri MZ; Mohd Zawawi Z; Norhisham SN; Ramly N; Robert F; Rosli NR; Suppiah J; Thayan R
EPI_ISL_6342887, EPI_ISL_6422346, EPI_ISL_6774007	Lahad Datu Hospital	Institute for Medical Research, Infectious Disease Research Centre, National Institutes of Health, Ministry of Health Malaysia	Ahmad FA; Ahmad Fazilah NA; Anasir MI; Azizan MA; Kamel K; Mohamad Sukri MZ; Mohd Zawawi Z; Norhisham SN; Ramly N; Robert F; Rosli NR; Suppiah J; Thayan R
EPI_ISL_6712854, EPI_ISL_6712864	Medical Hospital Pulau Pinang	iPROMISE, UiTM	Ariza Adnan; Fadzilah Mohd Nor; Lim Wai Feng; Mohd Asif Mohd Sukri; Mohd Nur Fakhruzzaman Noorizhab; Mohd Zaki Salleh; Sazzli Shahlan Kassim; Siti Farah Alwani Mohd Nawi; Siti Hamimah Sheikh Abdul Kadir; Teh Lay Kek; Wang Seok Mui
EPI_ISL_5894070, EPI_ISL_5895218, EPI_ISL_7472991	Miri Hospital Molecular Diagnostic Lab, Miri	Institute of Health and Community Medicine	Amir Safuan bin Khamshah; Chan Chia Jui; Chua Hock Hin; David Perera; Ooi Mong How; Tonnii Sia Loong Loong; Wong Jyn Shan
EPI_ISL_8254293, EPI_ISL_8254294, EPI_ISL_8254295, EPI_ISL_8254296, EPI_ISL_8254297, EPI_ISL_8254298, EPI_ISL_8254299, EPI_ISL_8254300, EPI_ISL_8254301, EPI_ISL_8254302, EPI_ISL_8254303, EPI_ISL_8254304, EPI_ISL_8254305, EPI_ISL_8254306, EPI_ISL_8254307			
see above	PEJABAT KESIHATAN DAERAH BATANG PADANG	Department of Medical Microbiology, Faculty of Medicine, University of Malaya; University of Malaya Medical Centre	I-Ching SAM; Jolene Yin Ling FU; Omar Khalilur Rahman; Yoke Fun Chan
EPI_ISL_8090691	PEJABAT KESIHATAN DAERAH KUALA KRAI	Tropical Infectious Diseases Research & Education Centre (TIDREC), Universiti Malaya	AsmaAnati CheMatSeri; Che-Norainon Yaacob; Jia-Yi Tan; Jo-Ern Wong; Kim-Kee Tan; Mulya-Mustika-Sari Zulkifli; Noor Syahida Azizan; Nur-Hidayana Mahfodz; Sazaly AbuBakar; Siti-Sarah Nor'e
EPI_ISL_6452595, EPI_ISL_6452640, EPI_ISL_8344041	PEJABAT KESIHATAN DAERAH MELAKA TENGAH	UKM Medical Molecular Biology Institute (UMBI)	Mira Farzana binti Mohamad Mokhtar
EPI_ISL_8090697, EPI_ISL_8090698, EPI_ISL_8090734, EPI_ISL_8090735	PEJABAT KESIHATAN DAERAH PASIR MAS	Tropical Infectious Diseases Research & Education Centre (TIDREC), Universiti Malaya	AsmaAnati CheMatSeri; Che-Norainon Yaacob; Jia-Yi Tan; Jo-Ern Wong; Kim-Kee Tan; Mulya-Mustika-Sari Zulkifli; Noor Syahida Azizan; Nur-Hidayana Mahfodz; Sazaly AbuBakar; Siti-Sarah Nor'e
EPI_ISL_6018112, EPI_ISL_6018122, EPI_ISL_6712811, EPI_ISL_6712817	PEJABAT KESIHATAN DAERAH SEBERANG PERAI UTARA PULAU PINANG	iPROMISE, UiTM	Ariza Adnan; Fadzilah Mohd Nor; Lim Wai Feng; Mohd Asif Mohd Sukri; Mohd Nur Fakhruzzaman Noorizhab; Mohd Zaki Salleh; Sazzli Shahlan Kassim; Siti Farah Alwani Mohd Nawi; Siti Hamimah Sheikh Abdul Kadir; Teh Lay Kek; Wang Seok Mui
EPI_ISL_5742660, EPI_ISL_5742690	PEJABAT KESIHATAN DAERAH SEREMBAN	UKM Medical Molecular Biology Institute (UMBI)	Mira Farzana binti Mohamad Mokhtar
EPI_ISL_6964386, EPI_ISL_6964391, EPI_ISL_6964396, EPI_ISL_6964403, EPI_ISL_6964409, EPI_ISL_6964418, EPI_ISL_6964424, EPI_ISL_6964652			
see above	PEJABAT KESIHATAN DAERAH TIMUR LAUT	iPROMISE, UiTM	Ariza Adnan; Fadzilah Mohd Nor; Lim Wai Feng; Mohd Asif Mohd Sukri; Mohd Nur Fakhruzzaman Noorizhab; Mohd Zaki Salleh; Sazzli Shahlan Kassim; Siti Farah Alwani Mohd Nawi; Siti Hamimah Sheikh Abdul Kadir; Teh Lay Kek; Wang Seok Mui
EPI_ISL_8090692	PEJABAT KESIHATAN DAREAH KUALA KRAI	Tropical Infectious Diseases Research & Education Centre (TIDREC), Universiti Malaya	AsmaAnati CheMatSeri; Che-Norainon Yaacob; Jia-Yi Tan; Jo-Ern Wong; Kim-Kee Tan; Mulya-Mustika-Sari Zulkifli; Noor Syahida Azizan; Nur-Hidayana Mahfodz; Sazaly AbuBakar; Siti-Sarah Nor'e
EPI_ISL_8090689, EPI_ISL_8090690, EPI_ISL_8090737, EPI_ISL_8090738	PKD BACHOK	Tropical Infectious Diseases Research & Education Centre (TIDREC), Universiti Malaya	AsmaAnati CheMatSeri; Che-Norainon Yaacob; Jia-Yi Tan; Jo-Ern Wong; Kim-Kee Tan; Mulya-Mustika-Sari Zulkifli; Noor Syahida Azizan; Nur-Hidayana Mahfodz; Sazaly AbuBakar; Siti-Sarah Nor'e
EPI_ISL_8095527	PKD HILIR PERAK	Tropical Infectious Diseases Research & Education Centre (TIDREC), Universiti Malaya	AsmaAnati CheMatSeri; Che-Norainon Yaacob; Jia-Yi Tan; Jo-Ern Wong; Kim-Kee Tan; Mulya-Mustika-Sari Zulkifli; Noor Syahida Azizan; Nur-Hidayana Mahfodz; Sazaly AbuBakar; Siti-Sarah Nor'e
EPI_ISL_8095526	PKD Hilir Perak	Tropical Infectious Diseases Research & Education Centre (TIDREC), Universiti Malaya	AsmaAnati CheMatSeri; Che-Norainon Yaacob; Jia-Yi Tan; Jo-Ern Wong; Kim-Kee Tan; Mulya-Mustika-Sari Zulkifli; Noor Syahida Azizan; Nur-Hidayana Mahfodz; Sazaly AbuBakar; Siti-Sarah Nor'e
EPI_ISL_5881265, EPI_ISL_5881268	PKD JEMPOL	UKM Medical Molecular Biology Institute (UMBI)	Mira Farzana binti Mohamad Mokhtar
EPI_ISL_5429417, EPI_ISL_5429430, EPI_ISL_5429432, EPI_ISL_5881285, EPI_ISL_6264275, EPI_ISL_6264284, EPI_ISL_6264287, EPI_ISL_6264294			
see above	PKD KUALA PILAH	UKM Medical Molecular Biology Institute (UMBI)	Mira Farzana binti Mohamad Mokhtar
EPI_ISL_8090693, EPI_ISL_8090694	PKD MACHANG	Tropical Infectious Diseases Research & Education Centre (TIDREC), Universiti Malaya	AsmaAnati CheMatSeri; Che-Norainon Yaacob; Jia-Yi Tan; Jo-Ern Wong; Kim-Kee Tan; Mulya-Mustika-Sari Zulkifli; Noor Syahida Azizan; Nur-Hidayana Mahfodz; Sazaly AbuBakar; Siti-Sarah Nor'e
EPI_ISL_5428869	PKD MELAKA TENGAH	UKM Medical Molecular Biology Institute (UMBI)	Mira Farzana binti Mohamad Mokhtar
EPI_ISL_5742692, EPI_ISL_5881288	PKD MERSING	UKM Medical Molecular Biology Institute (UMBI)	Mira Farzana binti Mohamad Mokhtar
EPI_ISL_7087266, EPI_ISL_7087580, EPI_ISL_7087690, EPI_ISL_7087719, EPI_ISL_7087772, EPI_ISL_7087834, EPI_ISL_7087872			
see above	PKD Manjung	Department of Medical Microbiology, Faculty of Medicine, University of Malaya; University of Malaya Medical Centre	I-Ching SAM; Jolene Yin Ling FU; Omar Khalilur Rahman; Yoke Fun Chan
EPI_ISL_8170455	PKD Manjung	Tropical Infectious Diseases Research & Education Centre (TIDREC), Universiti Malaya	AsmaAnati CheMatSeri; Che-Norainon Yaacob; Jia-Yi Tan; Jo-Ern Wong; Kim-Kee Tan; Mulya-Mustika-Sari Zulkifli; Noor Syahida Azizan; Nur-Hidayana Mahfodz; Sazaly AbuBakar; Siti-Sarah Nor'e
EPI_ISL_8090695, EPI_ISL_8090696	PKD PASIR PUTEH	Tropical Infectious Diseases Research & Education Centre (TIDREC), Universiti Malaya	AsmaAnati CheMatSeri; Che-Norainon Yaacob; Jia-Yi Tan; Jo-Ern Wong; Kim-Kee Tan; Mulya-Mustika-Sari Zulkifli; Noor Syahida Azizan; Nur-Hidayana Mahfodz; Sazaly AbuBakar; Siti-Sarah Nor'e
EPI_ISL_6712893	PKD SPT	iPROMISE, UiTM	Ariza Adnan; Fadzilah Mohd Nor; Lim Wai Feng; Mohd Asif Mohd Sukri; Mohd Nur Fakhruzzaman Noorizhab; Mohd Zaki Salleh; Sazzli Shahlan Kassim; Siti Farah Alwani Mohd Nawi; Siti Hamimah Sheikh Abdul Kadir; Teh Lay Kek; Wang Seok Mui
EPI_ISL_5428863, EPI_ISL_5428864, EPI_ISL_5428866, EPI_ISL_5428868, EPI_ISL_5428870, EPI_ISL_5428871, EPI_ISL_5428875, EPI_ISL_5428876, EPI_ISL_5428877, EPI_ISL_5428880, EPI_ISL_5428881, EPI_ISL_5428882, EPI_ISL_5742645, EPI_ISL_5742653, EPI_ISL_5742667, EPI_ISL_5742704, EPI_ISL_8324839			
see above	PKD TAMPIN	UKM Medical Molecular Biology Institute (UMBI)	Mira Farzana binti Mohamad Mokhtar
EPI_ISL_8090732, EPI_ISL_8090733	PKD Tanah Merah	Tropical Infectious Diseases Research & Education Centre (TIDREC), Universiti Malaya	AsmaAnati CheMatSeri; Che-Norainon Yaacob; Jia-Yi Tan; Jo-Ern Wong; Kim-Kee Tan; Mulya-Mustika-Sari Zulkifli; Noor Syahida Azizan; Nur-Hidayana Mahfodz; Sazaly AbuBakar; Siti-Sarah Nor'e
EPI_ISL_6825275	PKRC Dewan Masyarakat Tambunan	Malaysia Genome Institute	Azrin Ahmad; Enizza Kasim; Irni Suhayu Sapian; Mohd Faizal Abu Bakar; Mohd Ghows Mohd Azzam.; Mohd Noor Mat Isa; Nor Azfa Johari; Nurhezreen Md Iqbal; Shamsidar Sopie; Siti Noraini Othman; Yusuf Muhammad Noor
EPI_ISL_8745578, EPI_ISL_8745579	PKRC Dewan Masyarakat Tambunan	Malaysia Genome and Vaccine Institute	Apical Scientific Sdn Bhd's Team; Azrin Ahmad; Carey Wee Kai Li; Enizza Kasim; Irni Suhayu Sapian; Jilian Michelle Wong Tzeling; Leong Wai Mun; Mohd Faizal Abu Bakar; Mohd Ghows Mohd Azzam.; Mohd Noor Mat Isa; Nor Azfa Johari; Nurhezreen Md Iqbal; Rose Iszati Ismet Nayan; Shamsidar Sopie; Siti Noraini Othman; Yusuf Muhammad Noor
EPI_ISL_5113794	Papar District Health Office	Institute for Medical Research, Infectious Disease Research Centre, National Institutes of Health, Ministry of Health Malaysia	Ahmad FA; Ahmad Fazilah NA; Anasir MI; Azizan MA; Kamel K; Mohd Zawawi Z; Norhisham SN; Ramly N; Robert F; Suppiah J; Thayan R
EPI_ISL_8090736	Pejabat Kesihatan Daerah Jeli	Tropical Infectious Diseases Research & Education Centre (TIDREC), Universiti Malaya	AsmaAnati CheMatSeri; Che-Norainon Yaacob; Jia-Yi Tan; Jo-Ern Wong; Kim-Kee Tan; Mulya-Mustika-Sari Zulkifli; Noor Syahida Azizan; Nur-Hidayana Mahfodz; Sazaly AbuBakar; Siti-Sarah Nor'e
EPI_ISL_6825273, EPI_ISL_6825294, EPI_ISL_6825297, EPI_ISL_6825299	Pejabat Kesihatan Daerah Pitas	Malaysia Genome Institute	Azrin Ahmad; Enizza Kasim; Irni Suhayu Sapian; Mohd Faizal Abu Bakar; Mohd Ghows Mohd Azzam.; Mohd Noor Mat Isa; Nor Azfa Johari; Nurhezreen Md Iqbal; Shamsidar Sopie; Siti Noraini Othman; Yusuf Muhammad Noor
EPI_ISL_8745596	Pejabat Kesihatan Daerah Pitas	Malaysia Genome and Vaccine Institute	Apical Scientific Sdn Bhd's Team; Azrin Ahmad; Carey Wee Kai Li; Enizza Kasim; Irni Suhayu Sapian; Jilian Michelle Wong Tzeling; Leong Wai Mun; Mohd Faizal Abu Bakar; Mohd Ghows Mohd Azzam.; Mohd Noor Mat Isa; Nor Azfa Johari; Nurhezreen Md Iqbal; Rose Iszati Ismet Nayan; Shamsidar Sopie; Siti Noraini Othman; Yusuf Muhammad Noor
EPI_ISL_7260016, EPI_ISL_7260027, EPI_ISL_7260376	Pejabat Kesihatan Daerah Sipitang	Malaysia Genome Institute	Azrin Ahmad; Enizza Kasim; Irni Suhayu Sapian; Mohd Faizal Abu Bakar; Mohd Ghows Mohd Azzam.; Mohd Noor Mat Isa; Nor Azfa Johari; Nurhezreen Md Iqbal; Shamsidar Sopie; Siti Noraini Othman; Yusuf Muhammad Noor
EPI_ISL_6825298	Pejabat Kesihatan Kawasan Beaufort	Malaysia Genome Institute	Azrin Ahmad; Enizza Kasim; Irni Suhayu Sapian; Mohd Faizal Abu Bakar; Mohd Ghows Mohd Azzam.; Mohd Noor Mat Isa; Nor Azfa Johari; Nurhezreen Md Iqbal; Shamsidar Sopie; Siti Noraini Othman; Yusuf Muhammad Noor
EPI_ISL_7260008	Pejabat Kesihatan Kawasan Kota Kinabalu	Malaysia Genome Institute	Azrin Ahmad; Enizza Kasim; Irni Suhayu Sapian; Mohd Faizal Abu Bakar; Mohd Ghows Mohd Azzam.; Mohd Noor Mat Isa; Nor Azfa Johari; Nurhezreen Md Iqbal; Shamsidar Sopie; Siti Noraini Othman; Yusuf Muhammad Noor
EPI_ISL_6825296	Pejabat Kesihatan Kawasan Kudat	Malaysia Genome Institute	Azrin Ahmad; Enizza Kasim; Irni Suhayu Sapian; Mohd Faizal Abu Bakar; Mohd Ghows Mohd Azzam.; Mohd Noor Mat Isa; Nor Azfa Johari; Nurhezreen Md Iqbal; Shamsidar Sopie; Siti Noraini Othman; Yusuf Muhammad Noor
EPI_ISL_8745590	Pejabat Kesihatan Kawasan Tawau	Malaysia Genome and Vaccine Institute	Apical Scientific Sdn Bhd's Team; Azrin Ahmad; Carey Wee Kai Li; Enizza Kasim; Irni Suhayu Sapian; Jilian Michelle Wong Tzeling; Leong Wai Mun; Mohd Faizal Abu Bakar; Mohd Ghows Mohd Azzam.; Mohd Noor Mat Isa; Nor Azfa Johari; Nurhezreen Md Iqbal; Rose Iszati Ismet Nayan; Shamsidar Sopie; Siti Noraini Othman; Yusuf Muhammad Noor
EPI_ISL_6423168, EPI_ISL_6423211, EPI_ISL_6423407	Putrajaya Hospital	Institute for Medical Research, Infectious Disease Research Centre, National Institutes of Health, Ministry of Health Malaysia	Ahmad FA; Ahmad Fazilah NA; Anasir MI; Azizan MA; Kamel K; Mohd Zawawi Z; Norhisham SN; Ramly N; Robert F; Suppiah J; Thayan R
EPI_ISL_6913875, EPI_ISL_6914215	Ranau District Health Office	Institute for Medical Research, Infectious Disease Research Centre, National Institutes of Health, Ministry of Health Malaysia	Ahmad FA; Ahmad Fazilah NA; Anasir MI; Azizan MA; Kamel K; Mohamad Sukri MZ; Mohd Zawawi Z; Norhisham SN; Ramly N; Robert F; Rosli NR; Suppiah J; Thayan R
EPI_ISL_6968153, EPI_ISL_6968165, EPI_ISL_6968177, EPI_ISL_6968193, EPI_ISL_6968206, EPI_ISL_6968221, EPI_ISL_6968237, EPI_ISL_6968244, EPI_ISL_6969111, EPI_ISL_6970432			

see above	Rejang Medical Centre (Sibu)	Institute of Health and Community Medicine	Chan Chia Jui; Chua Hock Hin; David Perera; Ooi Mong How; Tonnii Sia Loong Loong; Wong Jyn Shan
EPI_ISL_5881267, EPI_ISL_5881275, EPI_ISL_5881283, EPI_ISL_8344042	SWAT TEAM HPSF	UKM Medical Molecular Biology Institute (UMBI)	Mira Farzana binti Mohamad Mokhtar
EPI_ISL_5893783, EPI_ISL_5893858, EPI_ISL_5893875, EPI_ISL_5893881, EPI_ISL_5895676, EPI_ISL_5895684, EPI_ISL_5895689, EPI_ISL_5895696, EPI_ISL_5895704, EPI_ISL_5895714, EPI_ISL_5895717, EPI_ISL_5895729, EPI_ISL_5895738, EPI_ISL_5895745, EPI_ISL_5895750, EPI_ISL_5895760, EPI_ISL_5895767, EPI_ISL_5895774, EPI_ISL_5895782, EPI_ISL_5895790, EPI_ISL_5895796, EPI_ISL_5895801, EPI_ISL_5895809, EPI_ISL_5895814, EPI_ISL_5895819, EPI_ISL_5895829, EPI_ISL_5895840, EPI_ISL_5895847, EPI_ISL_5895851, EPI_ISL_5895859, EPI_ISL_5895867, EPI_ISL_5895872, EPI_ISL_5895880, EPI_ISL_5895894, EPI_ISL_5895903, EPI_ISL_5895908, EPI_ISL_5895921, EPI_ISL_5895930, EPI_ISL_5895937, EPI_ISL_5895940, EPI_ISL_5895948, EPI_ISL_5895956, EPI_ISL_5895964, EPI_ISL_5895972, EPI_ISL_5895982, EPI_ISL_5895990, EPI_ISL_5896000, EPI_ISL_5896006, EPI_ISL_5896010, EPI_ISL_5896249, EPI_ISL_5896255, EPI_ISL_6586328, EPI_ISL_6586331, EPI_ISL_6586334, EPI_ISL_6586338, EPI_ISL_6586345, EPI_ISL_6586347, EPI_ISL_6586349, EPI_ISL_6586419, EPI_ISL_6586422, EPI_ISL_6586423, EPI_ISL_6586424, EPI_ISL_6586425, EPI_ISL_6586426, EPI_ISL_6586427, EPI_ISL_6586509, EPI_ISL_6586510, EPI_ISL_6586511, EPI_ISL_6586512, EPI_ISL_6586513, EPI_ISL_6586514, EPI_ISL_6586515, EPI_ISL_6586516, EPI_ISL_6586517, EPI_ISL_6586572, EPI_ISL_6586573, EPI_ISL_6586574, EPI_ISL_6586575, EPI_ISL_6586576, EPI_ISL_6586577, EPI_ISL_6586578, EPI_ISL_6586579, EPI_ISL_6586580, EPI_ISL_6586581, EPI_ISL_6586585, EPI_ISL_6586586, EPI_ISL_6586604, EPI_ISL_6586605, EPI_ISL_6586606, EPI_ISL_6586607, EPI_ISL_6586608, EPI_ISL_6586609, EPI_ISL_6586610, EPI_ISL_6586611, EPI_ISL_6968934, EPI_ISL_6968942, EPI_ISL_6968953, EPI_ISL_6968970, EPI_ISL_6968985, EPI_ISL_6968998, EPI_ISL_6969006, EPI_ISL_6970440, EPI_ISL_7472975, EPI_ISL_7472976, EPI_ISL_7472977, EPI_ISL_7472978, EPI_ISL_7472979, EPI_ISL_7472980, EPI_ISL_7472981, EPI_ISL_7472982, EPI_ISL_7472983, EPI_ISL_7472984, EPI_ISL_7473127, EPI_ISL_7473128			
see above	Sarawak General Hospital (Kuching)	Institute of Health and Community Medicine	Amir Safuan bin Khamshah; Chan Chia Jui; Chua Hock Hin; David Perera; Lim Woon Hook; Ooi Mong How; Tonnii Sia Loong Loong; Wong Jyn Shan
EPI_ISL_6422375	Search Results Kudat Area Health Office	Institute for Medical Research, Infectious Disease Research Centre, National Institutes of Health, Ministry of Health Malaysia	Ahmad FA; Ahmad Fazilah NA; Anasir MI; Azizan MA; Kamel K; Mohd Zawawi Z; Norhisham SN; Ramly N; Robert F; Suppiah J; Thayan R
EPI_ISL_6914192	Selangor State Health Department	Institute for Medical Research, Infectious Disease Research Centre, National Institutes of Health, Ministry of Health Malaysia	Ahmad FA; Ahmad Fazilah NA; Anasir MI; Azizan MA; Kamel K; Mohamad Sukri MZ; Mohd Zawawi Z; Norhisham SN; Ramly N; Robert F; Rosli NR; Suppiah J; Thayan R
EPI_ISL_5852485	Sungai Buloh Hospital	Institute for Medical Research, Infectious Disease Research Centre, National Institutes of Health, Ministry of Health Malaysia	Ahmad FA; Ahmad Fazilah NA; Anasir MI; Azizan MA; Kamel K; Mohd Zawawi Z; Norhisham SN; Ramly N; Robert F; Suppiah J; Thayan R
EPI_ISL_6423042, EPI_ISL_6913957, EPI_ISL_6914613	Tenom District Health Office	Institute for Medical Research, Infectious Disease Research Centre, National Institutes of Health, Ministry of Health Malaysia	Ahmad FA; Ahmad Fazilah NA; Anasir MI; Azizan MA; Kamel K; Mohamad Sukri MZ; Mohd Zawawi Z; Norhisham SN; Ramly N; Robert F; Rosli NR; Suppiah J; Thayan R
EPI_ISL_6422765	Thomson Hospital Kota Damansara	Institute for Medical Research, Infectious Disease Research Centre, National Institutes of Health, Ministry of Health Malaysia	Ahmad FA; Ahmad Fazilah NA; Anasir MI; Azizan MA; Kamel K; Mohd Zawawi Z; Norhisham SN; Ramly N; Robert F; Suppiah J; Thayan R
EPI_ISL_5881278	UKA HOSPITAL ENCHE BESAR HAJJAH KHALSOM, KLUANG	UKM Medical Molecular Biology Institute (UMBI)	Mira Farzana binti Mohamad Mokhtar
EPI_ISL_6132004, EPI_ISL_6132046, EPI_ISL_6132050, EPI_ISL_8254272, EPI_ISL_8254273, EPI_ISL_8254276, EPI_ISL_8254277, EPI_ISL_8254278, EPI_ISL_8254279, EPI_ISL_8254280, EPI_ISL_8254281, EPI_ISL_8254347, EPI_ISL_8254355, EPI_ISL_8254356			
see above	UMMC	Department of Medical Microbiology, Faculty of Medicine, University of Malaya; University of Malaya Medical Centre	I-Ching SAM; Jolene Yin Ling FU; Omar Khalilur Rahman; Yoke Fun Chan
EPI_ISL_7086629	UNIT KESELAMATAN DAN KESIHATAN PEKERJA	Department of Medical Microbiology, Faculty of Medicine, University of Malaya; University of Malaya Medical Centre	I-Ching SAM; Jolene Yin Ling FU; Omar Khalilur Rahman; Yoke Fun Chan
EPI_ISL_6018038	UNIT KESIHATAN AWAM	iPROMISE, UiTM	Ariza Adnan; Fadzilah Mohd Nor; Lim Wai Feng; Mohd Asif Mohd Sukri; Mohd Nur Fakhruzzaman Noorizhab; Mohd Zaki Salleh; Sazzli Shahlan Kassim; Siti Farah Alwani Mohd Nawi; Siti Hamimah Sheikh Abdul Kadir; Teh Lay Kek; Wang Seok Mui
EPI_ISL_5881264, EPI_ISL_5881266, EPI_ISL_5881269, EPI_ISL_5881270, EPI_ISL_5881272, EPI_ISL_5881273, EPI_ISL_5881274, EPI_ISL_5881276, EPI_ISL_5881277, EPI_ISL_5881279, EPI_ISL_5881281, EPI_ISL_5881284, EPI_ISL_5881286, EPI_ISL_6452543, EPI_ISL_6452572, EPI_ISL_6452583, EPI_ISL_6452609, EPI_ISL_6452624, EPI_ISL_6452626, EPI_ISL_6452633, EPI_ISL_6452634, EPI_ISL_6452653, EPI_ISL_6452660			
see above	UNIT KESIHATAN AWAM HEBHK	UKM Medical Molecular Biology Institute (UMBI)	Mira Farzana binti Mohamad Mokhtar
EPI_ISL_6017843	Unit Forensik	iPROMISE, UiTM	Ariza Adnan; Fadzilah Mohd Nor; Lim Wai Feng; Mohd Asif Mohd Sukri; Mohd Nur Fakhruzzaman Noorizhab; Mohd Zaki Salleh; Sazzli Shahlan Kassim; Siti Farah Alwani Mohd Nawi; Siti Hamimah Sheikh Abdul Kadir; Teh Lay Kek; Wang Seok Mui
EPI_ISL_6017835, EPI_ISL_6017849	Unit Forensik Hospital Bukit Mertajam	iPROMISE, UiTM	Ariza Adnan; Fadzilah Mohd Nor; Lim Wai Feng; Mohd Asif Mohd Sukri; Mohd Nur Fakhruzzaman Noorizhab; Mohd Zaki Salleh; Sazzli Shahlan Kassim; Siti Farah Alwani Mohd Nawi; Siti Hamimah Sheikh Abdul Kadir; Teh Lay Kek; Wang Seok Mui
EPI_ISL_7189372	Wad 4 , Hospital Tuanku Fauziah	Department of Medical Microbiology, Faculty of Medicine, University of Malaya; University of Malaya Medical Centre	I-Ching SAM; Jolene Yin Ling FU; Omar Khalilur Rahman; Yoke Fun Chan

We gratefully acknowledge the following Authors from the Originating laboratories responsible for obtaining the specimens, as well as the Submitting laboratories where the genome data were generated and shared via GISAID, on which this research is based.

All Submitters of data may be contacted directly via www.gisaid.org

Authors are sorted alphabetically.

Acknowledgement EPI_SET Identifier: EPI_SET_20220603ov

Accession ID	Originating Laboratory	Submitting Laboratory	Authors
EPI_ISL_6422208	Ampang Hospital	Institute for Medical Research, Infectious Disease Research Centre, National Institutes of Health, Ministry of Health Malaysia	Ahmad FA; Ahmad Fazilah NA; Anasir MI; Azizan MA; Kamel K; Mohd Zawawi Z; Norhisham SN; Ramly N; Robert F; Suppiah J; Thayan R
EPI_ISL_6914268	Beaufort District Health Office	Institute for Medical Research, Infectious Disease Research Centre, National Institutes of Health, Ministry of Health Malaysia	Ahmad FA; Ahmad Fazilah NA; Anasir MI; Azizan MA; Kamel K; Mohamad Sukri MZ; Mohd Zawawi Z; Norhisham SN; Ramly N; Robert F; Rosli NR; Suppiah J; Thayan R
EPI_ISL_6586390, EPI_ISL_6586391, EPI_ISL_6586392, EPI_ISL_6586395, EPI_ISL_6586396, EPI_ISL_6586397, EPI_ISL_6586398, EPI_ISL_6586399, EPI_ISL_6586400, EPI_ISL_6586401, EPI_ISL_6586402, EPI_ISL_6586403, EPI_ISL_6586404, EPI_ISL_6586405, EPI_ISL_6586406, EPI_ISL_6586407, EPI_ISL_6586408, EPI_ISL_6586409, EPI_ISL_6586410, EPI_ISL_6586549, EPI_ISL_6586550, EPI_ISL_6586551, EPI_ISL_6586552, EPI_ISL_6586553, EPI_ISL_6586554, EPI_ISL_6586555, EPI_ISL_6586556, EPI_ISL_6586557, EPI_ISL_6586558, EPI_ISL_6586559, EPI_ISL_6586560, EPI_ISL_6586561, EPI_ISL_6586562, EPI_ISL_6586563, EPI_ISL_6586564, EPI_ISL_6586565, EPI_ISL_6586566, EPI_ISL_6586567, EPI_ISL_6586568, EPI_ISL_6586569, EPI_ISL_6586570, EPI_ISL_6586571, EPI_ISL_7472960, EPI_ISL_7472961, EPI_ISL_7472962, EPI_ISL_7472963, EPI_ISL_7473050, EPI_ISL_7473053			
see above	Bintulu Hospital PCR Lab, Bintulu	Institute of Health and Community Medicine	Chan Chia Jui; Chua Hock Hin; David Perera; Ooi Mong How; Tan Lee See; Tonnii Sia Loong Loong; Wong Jyn Shan
EPI_ISL_6586666, EPI_ISL_6586677, EPI_ISL_6586678, EPI_ISL_6586679, EPI_ISL_6586680, EPI_ISL_6586681, EPI_ISL_6586682, EPI_ISL_6586683, EPI_ISL_6586684, EPI_ISL_6586685, EPI_ISL_6586686, EPI_ISL_6586687, EPI_ISL_6586688, EPI_ISL_6586689, EPI_ISL_6586690, EPI_ISL_6970433, EPI_ISL_7473067, EPI_ISL_7473068, EPI_ISL_7473072, EPI_ISL_7473073, EPI_ISL_7473074, EPI_ISL_7473075			
see above	Bintulu Medical Centre (Bintulu)	Institute of Health and Community Medicine	Chan Chia Jui; Chua Hock Hin; David Perera; Ooi Mong How; Tonnii Sia Loong Loong; Wong Jyn Shan
EPI_ISL_6586418, EPI_ISL_6586428, EPI_ISL_6586429, EPI_ISL_6586430, EPI_ISL_6586431, EPI_ISL_6586432, EPI_ISL_6586598, EPI_ISL_6586599, EPI_ISL_6586612, EPI_ISL_6586613, EPI_ISL_6586614, EPI_ISL_6586615, EPI_ISL_6586616, EPI_ISL_6586617, EPI_ISL_6586618, EPI_ISL_6586619, EPI_ISL_6586620, EPI_ISL_6586621, EPI_ISL_6586623, EPI_ISL_6586624, EPI_ISL_6586625, EPI_ISL_6586649, EPI_ISL_6586650, EPI_ISL_6586651, EPI_ISL_6586652, EPI_ISL_6586653, EPI_ISL_6586654, EPI_ISL_6586655, EPI_ISL_6586656, EPI_ISL_6586657, EPI_ISL_6586658, EPI_ISL_6586659, EPI_ISL_6586660, EPI_ISL_6586661, EPI_ISL_6586662, EPI_ISL_6586663, EPI_ISL_6586664, EPI_ISL_6586665, EPI_ISL_6969440, EPI_ISL_6969449, EPI_ISL_6969465, EPI_ISL_7472884, EPI_ISL_7472917, EPI_ISL_7473078, EPI_ISL_7473079, EPI_ISL_8782823			
see above	Borneo Medical Centre (Kuching)	Institute of Health and Community Medicine	Chan Chia Jui; Chua Hock Hin; David Perera; Ooi Mong How; Tonnii Sia Loong Loong; Wong Jyn Shan
EPI_ISL_10070427	Department of Medical Microbiology, Hospital Pengajar Universiti Putra Malaysia	Department of Medical Microbiology, Hospital Pengajar Universiti Putra Malaysia	Afiqah Adzmi; Amiza Azmi; Azmiza Syawani Jasni; Hui Yee Chee; Leslie Thian Lung Than; Muadz Mohtar; Muhammad Mohd Isa; Narcisse MS Joseph; Niazlin Mohd Taib; Noor Hazirah Noor Azhari; Norlaila; Nur Raihana Ithnin; Nurul Huda Mohamed Rashidi; Nurul Nadiah Ismail; Rosni Ibrahim; Rukman Awang Hamat; Sallehhudin; Siti Norbaya Masri; Siti Zulaikha Zakariah; Syafinaz Amin Nordin; Tengku Zetty Maztura Tengku Jamaluddin; Zamberi Sekawi
EPI_ISL_8188772, EPI_ISL_8188773, EPI_ISL_8188774	Gribbles Pathology, Kuching	Institute of Health and Community Medicine	Chan Chia Jui; Chua Hock Hin; David Perera; Josephine Liew Chin Chin; Ooi Mong How; Tonnii Sia Loong Loong; Wong Jyn Shan
EPI_ISL_12628170	HCTM	UKM Medical Molecular Biology Institute (UMBI)	Khairun Nur Abd Ghafar; Mira Farzana Mohamad Mokhtar; Muhiddin Ishak; Nor Azila Muhammad Azami; Nur Alyaa Afifah Md Shahri; Nurul Syakima Ab Mutalib; Rahman Jamal; Ryia Illani Mohd Yunos; Siti Nur Hasanah Mohd Yusuf; Zahirrah Begam Mohamed Rasheed
EPI_ISL_8083673, EPI_ISL_8083674, EPI_ISL_8083680, EPI_ISL_8083682, EPI_ISL_8083683	HOSPITAL PULAU PINANG	iPROMISE, UiTM	Ariza Adnan; Fadzilah Mohd Nor; Lim Wai Feng; Mohd Asif Mohd Sukri; Mohd Nur Fakhruzzaman Noorizhab; Mohd Zaki Salleh; Sazzli Shahlan Kassim; Siti Farah Alwani Mohd Nawi; Siti Hamimah Sheikh Abdul Kadir; Teh Lay Kek; Wang Seok Mui
EPI_ISL_8254326, EPI_ISL_8254330, EPI_ISL_8254331	HOSPITAL RAJA PERMAISURI BAINUN	Department of Medical Microbiology, Faculty of Medicine, University of Malaya; University of Malaya Medical Centre	I-Ching SAM; Jolene Yin Ling FU; Omar Khalilur Rahman; Yoke Fun Chan
EPI_ISL_8095532, EPI_ISL_8170438, EPI_ISL_8170439, EPI_ISL_8170524, EPI_ISL_8170535	HOSPITAL RAJA PERMAISURI BAINUN	Tropical Infectious Diseases Research & Education Centre (TIDREC), Universiti Malaya	AsmaAnati CheMatSeri; Che-Norainon Yaacob; Jia-Yi Tan; Jo-Ern Wong; Kim-Kee Tan; Mulya-Mustika-Sari Zulkifli; Noor Syahida Azizan; Nur-Hidayana Mahfodz; Sazaly AbuBakar; Siti-Sarah Nor’e
EPI_ISL_6264276	HOSPITAL TUANKU JAAFAR	UKM Medical Molecular Biology Institute (UMBI)	Mira Farzana binti Mohamad Mokhtar
EPI_ISL_6964499, EPI_ISL_6964504, EPI_ISL_6964515, EPI_ISL_6964532	HPP	iPROMISE, UiTM	Ariza Adnan; Fadzilah Mohd Nor; Lim Wai Feng; Mohd Asif Mohd Sukri; Mohd Nur Fakhruzzaman Noorizhab; Mohd Zaki Salleh; Sazzli Shahlan Kassim; Siti Farah Alwani Mohd Nawi; Siti Hamimah Sheikh Abdul Kadir; Teh Lay Kek; Wang Seok Mui
EPI_ISL_6567083	HPSF	UKM Medical Molecular Biology Institute (UMBI)	Mira Farzana binti Mohamad Mokhtar
EPI_ISL_7086867, EPI_ISL_7086901, EPI_ISL_7087001, EPI_ISL_7087031, EPI_ISL_7087088, EPI_ISL_7087167	HRPB	Department of Medical Microbiology, Faculty of Medicine, University of Malaya; University of Malaya Medical Centre	I-Ching SAM; Jolene Yin Ling FU; Omar Khalilur Rahman; Yoke Fun Chan
EPI_ISL_6132017	HRPZ II	Department of Medical Microbiology, Faculty of Medicine, University of Malaya; University of Malaya Medical Centre	I-Ching SAM; Jolene Yin Ling FU; Omar Khalilur Rahman; Yoke Fun Chan
EPI_ISL_7260036, EPI_ISL_7260080, EPI_ISL_7260114, EPI_ISL_7260124	Hospital Ampang	Malaysia Genome Institute	Azrin Ahmad; Enizza Kasim; Irni Suhayu Sapian; Mohd Faizal Abu Bakar; Mohd Ghows Mohd Azzam.; Mohd Noor Mat Isa; Nor Azfa Johari; Nurhezreen Md Iqbal; Shamsidar Sopie; Siti Noraini Othman; Yusuf Muhammad Noor
EPI_ISL_8083667, EPI_ISL_8083668	Hospital Kepala Batas	iPROMISE, UiTM	Ariza Adnan; Fadzilah Mohd Nor; Lim Wai Feng; Mohd Asif Mohd Sukri; Mohd Nur Fakhruzzaman Noorizhab; Mohd Zaki Salleh; Sazzli Shahlan Kassim; Siti Farah Alwani Mohd Nawi; Siti Hamimah Sheikh Abdul Kadir; Teh Lay Kek; Wang Seok Mui
EPI_ISL_7260369	Hospital Kuala Kubu Bharu	Malaysia Genome Institute	Azrin Ahmad; Enizza Kasim; Irni Suhayu Sapian; Mohd Faizal Abu Bakar; Mohd Ghows Mohd Azzam.; Mohd Noor Mat Isa; Nor Azfa Johari; Nurhezreen Md Iqbal; Shamsidar Sopie; Siti Noraini Othman; Yusuf Muhammad Noor
EPI_ISL_7260071, EPI_ISL_7260363	Hospital Kuala Lumpur	Malaysia Genome Institute	Azrin Ahmad; Enizza Kasim; Irni Suhayu Sapian; Mohd Faizal Abu Bakar; Mohd Ghows Mohd Azzam.; Mohd Noor Mat Isa; Nor Azfa Johari; Nurhezreen Md Iqbal; Shamsidar Sopie; Siti Noraini Othman; Yusuf Muhammad Noor
EPI_ISL_7260031	Hospital Lahad Datu	Malaysia Genome Institute	Azrin Ahmad; Enizza Kasim; Irni Suhayu Sapian; Mohd Faizal Abu Bakar; Mohd Ghows Mohd Azzam.; Mohd Noor Mat Isa; Nor Azfa Johari; Nurhezreen Md Iqbal; Shamsidar Sopie; Siti Noraini Othman; Yusuf Muhammad Noor
EPI_ISL_6705555, EPI_ISL_8606779	Hospital Melaka	UKM Medical Molecular Biology Institute (UMBI)	Mira Farzana binti Mohamad Mokhtar
EPI_ISL_6713045, EPI_ISL_6964528, EPI_ISL_6964552, EPI_ISL_8083666, EPI_ISL_8083672, EPI_ISL_8083675, EPI_ISL_8083676, EPI_ISL_8083677, EPI_ISL_8083678, EPI_ISL_8083679, EPI_ISL_8083681, EPI_ISL_8083684, EPI_ISL_8083685			
see above	Hospital Pulau Pinang	iPROMISE, UiTM	Ariza Adnan; Fadzilah Mohd Nor; Lim Wai Feng; Mohd Asif Mohd Sukri; Mohd Nur Fakhruzzaman Noorizhab; Mohd Zaki Salleh; Sazzli Shahlan Kassim; Siti Farah Alwani Mohd Nawi; Siti Hamimah Sheikh Abdul Kadir; Teh Lay Kek; Wang Seok Mui
EPI_ISL_7260108, EPI_ISL_7260171	Hospital Queen Elizabeth	Malaysia Genome Institute	Azrin Ahmad; Enizza Kasim; Irni Suhayu Sapian; Mohd Faizal Abu Bakar; Mohd Ghows Mohd Azzam.; Mohd Noor Mat Isa; Nor Azfa Johari; Nurhezreen Md Iqbal; Shamsidar Sopie; Siti Noraini Othman; Yusuf Muhammad Noor
EPI_ISL_7260193	Hospital Tengku Ampuan Rahimah	Malaysia Genome Institute	Azrin Ahmad; Enizza Kasim; Irni Suhayu Sapian; Mohd Faizal Abu Bakar; Mohd Ghows Mohd Azzam.; Mohd Noor Mat Isa; Nor Azfa Johari; Nurhezreen Md Iqbal; Shamsidar Sopie; Siti Noraini Othman; Yusuf Muhammad Noor
EPI_ISL_6964511	Hospitlal Pulau Pinang	iPROMISE, UiTM	Ariza Adnan; Fadzilah Mohd Nor; Lim Wai Feng; Mohd Asif Mohd Sukri; Mohd Nur Fakhruzzaman Noorizhab; Mohd Zaki Salleh; Sazzli Shahlan Kassim; Siti Farah Alwani Mohd Nawi; Siti Hamimah Sheikh Abdul Kadir; Teh Lay Kek; Wang Seok Mui
EPI_ISL_6264286	ICU Htjs	UKM Medical Molecular Biology Institute (UMBI)	Mira Farzana binti Mohamad Mokhtar
EPI_ISL_6452557, EPI_ISL_6452563, EPI_ISL_6452588, EPI_ISL_6452630, EPI_ISL_6452645, EPI_ISL_6567073, EPI_ISL_8408360, EPI_ISL_8408361			
see above	INSTITUT BIOLOGI MOLEKUL PERUBATAN UKM (UMBI)	UKM Medical Molecular Biology Institute (UMBI)	Mira Farzana binti Mohamad Mokhtar
EPI_ISL_7119553, EPI_ISL_7119574, EPI_ISL_7119597, EPI_ISL_7119615, EPI_ISL_7119629, EPI_ISL_7119644, EPI_ISL_7119656, EPI_ISL_7119667, EPI_ISL_7119676, EPI_ISL_7119683, EPI_ISL_7119692, EPI_ISL_7119703, EPI_ISL_7119712, EPI_ISL_7119720, EPI_ISL_11078687, EPI_ISL_11078688, EPI_ISL_11078692, EPI_ISL_11078710, EPI_ISL_11078763, EPI_ISL_11078771			
see above	Institut Biologi Molekul Perubatan UKM (UMBI)	UKM Medical Molecular Biology Institute (UMBI)	Ab Mutalib; Abd Ghafar; Ishak; Jamal; Khairun Nur; Khairun Nur Abd Ghafar; Md Shahri; Mira Farzana; Mira Farzana Mohamad Mokhtar; Mohamad Mokhtar; Mohamed Rasheed; Mohd Yunos; Mohd Yusuf; Muhammad Azami; Muhiddin; Muhiddin Ishak; Nor Azi; Nor Azila Muhammad Azami; Nur Alyaa Afifah; Nur Alyaa Afifah Md Shahri; Nurul Syakima; Nurul Syakima Ab Mutalib; Rahman; Rahman Jamal; Ryia Illani; Ryia Illani Mohd Yunos; Siti Nur Hasanah; Siti Nur Hasanah Mohd Yusuf; Zahirrah Begam; Zahirrah Begam Mohamed Rasheed
EPI_ISL_6712963, EPI_ISL_6712977, EPI_ISL_6712982, EPI_ISL_6713002, EPI_ISL_6713007, EPI_ISL_6964378	JKN PAHANG	iPROMISE, UiTM	Ariza Adnan; Fadzilah Mohd Nor; Lim Wai Feng; Mohd Asif Mohd Sukri; Mohd Nur Fakhruzzaman Noorizhab; Mohd Zaki Salleh; Sazzli Shahlan Kassim; Siti Farah Alwani Mohd Nawi; Siti Hamimah Sheikh Abdul Kadir; Teh Lay Kek; Wang Seok Mui
EPI_ISL_8254282, EPI_ISL_8254287, EPI_ISL_8254289, EPI_ISL_8254291, EPI_ISL_8254292	Jabatan Forensik Hospital Sultanah Bahiyah	Department of Medical Microbiology, Faculty of Medicine, University of Malaya; University of Malaya Medical Centre	I-Ching SAM; Jolene Yin Ling FU; Omar Khalilur Rahman; Yoke Fun Chan
EPI_ISL_7260131, EPI_ISL_7260146, EPI_ISL_7260151, EPI_ISL_7260160	Jabatan Kesihatan WP Labuan	Malaysia Genome Institute	Azrin Ahmad; Enizza Kasim; Irni Suhayu Sapian; Mohd Faizal Abu Bakar; Mohd Ghows Mohd Azzam.; Mohd Noor Mat Isa; Nor Azfa Johari; Nurhezreen Md Iqbal; Shamsidar Sopie; Siti Noraini Othman; Yusuf Muhammad Noor
EPI_ISL_7086857	KK Tendong	Department of Medical Microbiology, Faculty of Medicine, University of Malaya; University of Malaya Medical Centre	I-Ching SAM; Jolene Yin Ling FU; Omar Khalilur Rahman; Yoke Fun Chan
EPI_ISL_6712822	KPAS JKN PAHANG	iPROMISE, UiTM	Ariza Adnan; Fadzilah Mohd Nor; Lim Wai Feng; Mohd Asif Mohd Sukri; Mohd Nur Fakhruzzaman Noorizhab; Mohd Zaki Salleh; Sazzli Shahlan Kassim; Siti Farah Alwani Mohd Nawi; Siti Hamimah Sheikh Abdul Kadir; Teh Lay Kek; Wang Seok Mui
EPI_ISL_6712955	KPAS JKN Pahang	iPROMISE, UiTM	Ariza Adnan; Fadzilah Mohd Nor; Lim Wai Feng; Mohd Asif Mohd Sukri; Mohd Nur Fakhruzzaman Noorizhab; Mohd Zaki Salleh; Sazzli Shahlan Kassim; Siti Farah Alwani Mohd Nawi; Siti Hamimah Sheikh Abdul Kadir; Teh Lay Kek; Wang Seok Mui
EPI_ISL_9210148, EPI_ISL_9212386	Kajang Hospital	Institute for Medical Research, Infectious Disease Research Centre, National Institutes of Health, Ministry of Health Malaysia	Ahmad FA; Ahmad Fazilah NA; Anasir MI; Azizan MA; Kamel K; Mohamad Sukri MZ; Norhisham SN; Ramly N; Robert F; Rosli NR; Suppiah J; Thayan R
EPI_ISL_6422471	Keningau Area Health Office	Institute for Medical Research, Infectious Disease Research Centre, National Institutes of Health, Ministry of Health Malaysia	Ahmad FA; Ahmad Fazilah NA; Anasir MI; Azizan MA; Kamel K; Mohd Zawawi Z; Norhisham SN; Ramly N; Robert F; Suppiah J; Thayan R

EPI_ISL_8692787, EPI_ISL_8692792, EPI_ISL_8693166, EPI_ISL_8693362	Kinabatangan Area Health Office	Institute for Medical Research, Infectious Disease Research Centre, National Institutes of Health, Ministry of Health Malaysia	Ahmad FA; Ahmad Fazilah NA; Anasir MI; Azizan MA; Kamel K; Mohamad Sukri MZ; Norhisham SN; Ramly N; Robert F; Rosli NR; Suppiah J; Thayan R
EPI_ISL_6423044, EPI_ISL_6423698, EPI_ISL_6913879, EPI_ISL_6913888, EPI_ISL_6913950, EPI_ISL_6914169	Kota Kinabalu Area Health Office	Institute for Medical Research, Infectious Disease Research Centre, National Institutes of Health, Ministry of Health Malaysia	Ahmad FA; Ahmad Fazilah NA; Anasir MI; Azizan MA; Kamel K; Mohamad Sukri MZ; Mohd Zawawi Z; Norhisham SN; Ramly N; Robert F; Rosli NR; Suppiah J; Thayan R
EPI_ISL_6422439, EPI_ISL_6422470	Kuala Kubu Bharu Hospital	Institute for Medical Research, Infectious Disease Research Centre, National Institutes of Health, Ministry of Health Malaysia	Ahmad FA; Ahmad Fazilah NA; Anasir MI; Azizan MA; Kamel K; Mohd Zawawi Z; Norhisham SN; Ramly N; Robert F; Suppiah J; Thayan R
EPI_ISL_8692793	Lahad Datu Hospital	Institute for Medical Research, Infectious Disease Research Centre, National Institutes of Health, Ministry of Health Malaysia	Ahmad FA; Ahmad Fazilah NA; Anasir MI; Azizan MA; Kamel K; Mohamad Sukri MZ; Norhisham SN; Ramly N; Robert F; Rosli NR; Suppiah J; Thayan R
EPI_ISL_6825316	MGI	Malaysia Genome Institute	Azrin Ahmad; Enizza Kasim; Irni Suhayu Sapian; Mohd Faizal Abu Bakar; Mohd Ghows Mohd Azzam.; Mohd Noor Mat Isa; Nor Azfa Johari; Nurhezreen Md Iqbal; Shamsidar Sopie; Siti Noraini Othman; Yusuf Muhammad Noor
EPI_ISL_7775246, EPI_ISL_7775247	MGI	Malaysia Genome and Vaccine Institute	Azrin Ahmad; Enizza Kasim; Irni Suhayu Sapian; Mohd Faizal Abu Bakar; Mohd Ghows Mohd Azzam.; Mohd Noor Mat Isa; Nor Azfa Johari; Nurhezreen Md Iqbal; Shamsidar Sopie; Siti Noraini Othman; Yusuf Muhammad Noor
EPI_ISL_8745604	MGVI	Malaysia Genome and Vaccine Institute	Apical Scientific Sdn Bhd's Team; Azrin Ahmad; Carey Wee Kai Li; Enizza Kasim; Irni Suhayu Sapian; Jilian Michelle Wong Tzeling; Leong Wai Mun; Mohd Faizal Abu Bakar; Mohd Ghows Mohd Azzam.; Mohd Noor Mat Isa; Nor Azfa Johari; Nurhezreen Md Iqbal; Rose Iszati Ismet Nayan; Shamsidar Sopie; Siti Noraini Othman; Yusuf Muhammad Noor
EPI_ISL_8083669, EPI_ISL_8083670, EPI_ISL_8083671	Medical Hospital Pulau Pinang	iPROMISE, UiTM	Ariza Adnan; Fadzilah Mohd Nor; Lim Wai Feng; Mohd Asif Mohd Sukri; Mohd Nur Fakhruzzaman Noorizhab; Mohd Zaki Salleh; Sazzli Shahlan Kassim; Siti Farah Alwani Mohd Nawi; Siti Hamimah Sheikh Abdul Kadir; Teh Lay Kek; Wang Seok Mui
EPI_ISL_6586544, EPI_ISL_6586545, EPI_ISL_6586546, EPI_ISL_6586547, EPI_ISL_6586548, EPI_ISL_7472992, EPI_ISL_7472993, EPI_ISL_7472994, EPI_ISL_7472995			
see above	Miri Hospital Molecular Diagnostic Lab, Miri	Institute of Health and Community Medicine	Amir Safuan bin Khamshah; Chan Chia Jui; Chua Hock Hin; David Perera; Ooi Mong How; Tonnii Sia Loong Loong; Wong Jyn Shan
EPI_ISL_6424688	National Institutes of Health	Institute for Medical Research, Infectious Disease Research Centre, National Institutes of Health, Ministry of Health Malaysia	Ahmad FA; Ahmad Fazilah NA; Anasir MI; Azizan MA; Kamel K; Mohd Zawawi Z; Norhisham SN; Ramly N; Robert F; Suppiah J; Thayan R
EPI_ISL_6264285	OAC COVID MEIDCAL	UKM Medical Molecular Biology Institute (UMBI)	Mira Farzana binti Mohamad Mokhtar
EPI_ISL_7086835, EPI_ISL_7086847, EPI_ISL_7086919	PEJABAT KESIHATAN DAERAH KUALA KRAI	Department of Medical Microbiology, Faculty of Medicine, University of Malaya; University of Malaya Medical Centre	I-Ching SAM; Jolene Yin Ling FU; Omar Khalilur Rahman; Yoke Fun Chan
EPI_ISL_6567077, EPI_ISL_6705551	PEJABAT KESIHATAN DAERAH MELAKA TENGAH	UKM Medical Molecular Biology Institute (UMBI)	Mira Farzana binti Mohamad Mokhtar
EPI_ISL_8083687	PEJABAT KESIHATAN DAERAH SEBERANG PERAI UTARA PULAU PINANG	iPROMISE, UiTM	Ariza Adnan; Fadzilah Mohd Nor; Lim Wai Feng; Mohd Asif Mohd Sukri; Mohd Nur Fakhruzzaman Noorizhab; Mohd Zaki Salleh; Sazzli Shahlan Kassim; Siti Farah Alwani Mohd Nawi; Siti Hamimah Sheikh Abdul Kadir; Teh Lay Kek; Wang Seok Mui
EPI_ISL_6264279, EPI_ISL_6264291	PEJABAT KESIHATAN DAERAH SEREMBAN	UKM Medical Molecular Biology Institute (UMBI)	Mira Farzana binti Mohamad Mokhtar
EPI_ISL_6964663, EPI_ISL_8500652, EPI_ISL_8500653, EPI_ISL_8500654, EPI_ISL_8500655, EPI_ISL_8500656, EPI_ISL_8500657			
see above	PEJABAT KESIHATAN DAERAH TIMUR LAUT	iPROMISE, UiTM	Ariza Adnan; Fadzilah Mohd Nor; Lim Wai Feng; Mohd Asif Mohd Sukri; Mohd Nur Fakhruzzaman Noorizhab; Mohd Zaki Salleh; Sazzli Shahlan Kassim; Siti Farah Alwani Mohd Nawi; Siti Hamimah Sheikh Abdul Kadir; Teh Lay Kek; Wang Seok Mui
EPI_ISL_7086672, EPI_ISL_7086976	PKD BACHOK	Department of Medical Microbiology, Faculty of Medicine, University of Malaya; University of Malaya Medical Centre	I-Ching SAM; Jolene Yin Ling FU; Omar Khalilur Rahman; Yoke Fun Chan
EPI_ISL_8095523, EPI_ISL_8095524, EPI_ISL_8095525	PKD HILIR PERAK	Tropical Infectious Diseases Research & Education Centre (TIDREC), Universiti Malaya	AsmaAnati CheMatSeri; Che-Norainon Yaacob; Jia-Yi Tan; Jo-Ern Wong; Kim-Kee Tan; Mulya-Mustika-Sari Zulkifli; Noor Syahida Azizan; Nur-Hidayana Mahfodz; Sazaly AbuBakar; Siti-Sarah Nor'e
EPI_ISL_6264273	PKD KUALA PILAH	UKM Medical Molecular Biology Institute (UMBI)	Mira Farzana binti Mohamad Mokhtar
EPI_ISL_6264293, EPI_ISL_6567080	PKD MELAKA TENGAH	UKM Medical Molecular Biology Institute (UMBI)	Mira Farzana binti Mohamad Mokhtar
EPI_ISL_6712830, EPI_ISL_6712835, EPI_ISL_6712989, EPI_ISL_6713015	PKD Maran	iPROMISE, UiTM	Ariza Adnan; Fadzilah Mohd Nor; Lim Wai Feng; Mohd Asif Mohd Sukri; Mohd Nur Fakhruzzaman Noorizhab; Mohd Zaki Salleh; Sazzli Shahlan Kassim; Siti Farah Alwani Mohd Nawi; Siti Hamimah Sheikh Abdul Kadir; Teh Lay Kek; Wang Seok Mui
EPI_ISL_7086662	PKD PASIR PUTEH	Department of Medical Microbiology, Faculty of Medicine, University of Malaya; University of Malaya Medical Centre	I-Ching SAM; Jolene Yin Ling FU; Omar Khalilur Rahman; Yoke Fun Chan
EPI_ISL_8095522, EPI_ISL_8170434, EPI_ISL_8170435	PKD PERAK TENGAH	Tropical Infectious Diseases Research & Education Centre (TIDREC), Universiti Malaya	AsmaAnati CheMatSeri; Che-Norainon Yaacob; Jia-Yi Tan; Jo-Ern Wong; Kim-Kee Tan; Mulya-Mustika-Sari Zulkifli; Noor Syahida Azizan; Nur-Hidayana Mahfodz; Sazaly AbuBakar; Siti-Sarah Nor'e
EPI_ISL_6713059	PKD SEBERANG PERAI SELATAN	iPROMISE, UiTM	Ariza Adnan; Fadzilah Mohd Nor; Lim Wai Feng; Mohd Asif Mohd Sukri; Mohd Nur Fakhruzzaman Noorizhab; Mohd Zaki Salleh; Sazzli Shahlan Kassim; Siti Farah Alwani Mohd Nawi; Siti Hamimah Sheikh Abdul Kadir; Teh Lay Kek; Wang Seok Mui
EPI_ISL_6713055	PKD SPS	iPROMISE, UiTM	Ariza Adnan; Fadzilah Mohd Nor; Lim Wai Feng; Mohd Asif Mohd Sukri; Mohd Nur Fakhruzzaman Noorizhab; Mohd Zaki Salleh; Sazzli Shahlan Kassim; Siti Farah Alwani Mohd Nawi; Siti Hamimah Sheikh Abdul Kadir; Teh Lay Kek; Wang Seok Mui
EPI_ISL_6964427, EPI_ISL_6964493, EPI_ISL_6964546, EPI_ISL_6964587, EPI_ISL_6964595, EPI_ISL_8083665	PKD SPT	iPROMISE, UiTM	Ariza Adnan; Fadzilah Mohd Nor; Lim Wai Feng; Mohd Asif Mohd Sukri; Mohd Nur Fakhruzzaman Noorizhab; Mohd Zaki Salleh; Sazzli Shahlan Kassim; Siti Farah Alwani Mohd Nawi; Siti Hamimah Sheikh Abdul Kadir; Teh Lay Kek; Wang Seok Mui
EPI_ISL_6264277, EPI_ISL_6264283, EPI_ISL_6264288, EPI_ISL_8407585	PKD TAMPIN	UKM Medical Molecular Biology Institute (UMBI)	Mira Farzana binti Mohamad Mokhtar
EPI_ISL_6964479, EPI_ISL_6964489	PKDSPT	iPROMISE, UiTM	Ariza Adnan; Fadzilah Mohd Nor; Lim Wai Feng; Mohd Asif Mohd Sukri; Mohd Nur Fakhruzzaman Noorizhab; Mohd Zaki Salleh; Sazzli Shahlan Kassim; Siti Farah Alwani Mohd Nawi; Siti Hamimah Sheikh Abdul Kadir; Teh Lay Kek; Wang Seok Mui
EPI_ISL_7087202	Pejabat Kesihatan Daerah Jeli	Department of Medical Microbiology, Faculty of Medicine, University of Malaya; University of Malaya Medical Centre	I-Ching SAM; Jolene Yin Ling FU; Omar Khalilur Rahman; Yoke Fun Chan
EPI_ISL_7260046, EPI_ISL_7260055, EPI_ISL_7260062, EPI_ISL_7260179, EPI_ISL_7260187, EPI_ISL_7260210, EPI_ISL_7260219, EPI_ISL_7260232, EPI_ISL_7260237			
see above	Pejabat Kesihatan Daerah Sipitang	Malaysia Genome Institute	Azrin Ahmad; Enizza Kasim; Irni Suhayu Sapian; Mohd Faizal Abu Bakar; Mohd Ghows Mohd Azzam.; Mohd Noor Mat Isa; Nor Azfa Johari; Nurhezreen Md Iqbal; Shamsidar Sopie; Siti Noraini Othman; Yusuf Muhammad Noor
EPI_ISL_7260089, EPI_ISL_7260093, EPI_ISL_7260100	Pejabat Kesihatan Daerah Tenom	Malaysia Genome Institute	Azrin Ahmad; Enizza Kasim; Irni Suhayu Sapian; Mohd Faizal Abu Bakar; Mohd Ghows Mohd Azzam.; Mohd Noor Mat Isa; Nor Azfa Johari; Nurhezreen Md Iqbal; Shamsidar Sopie; Siti Noraini Othman; Yusuf Muhammad Noor
EPI_ISL_7260138	Pejabat Kesihatan Kawasan Beaufort	Malaysia Genome Institute	Azrin Ahmad; Enizza Kasim; Irni Suhayu Sapian; Mohd Faizal Abu Bakar; Mohd Ghows Mohd Azzam.; Mohd Noor Mat Isa; Nor Azfa Johari; Nurhezreen Md Iqbal; Shamsidar Sopie; Siti Noraini Othman; Yusuf Muhammad Noor
EPI_ISL_7260201, EPI_ISL_7260354	Pejabat Kesihatan Kawasan Kota Kinabalu	Malaysia Genome Institute	Azrin Ahmad; Enizza Kasim; Irni Suhayu Sapian; Mohd Faizal Abu Bakar; Mohd Ghows Mohd Azzam.; Mohd Noor Mat Isa; Nor Azfa Johari; Nurhezreen Md Iqbal; Shamsidar Sopie; Siti Noraini Othman; Yusuf Muhammad Noor
EPI_ISL_6914426	Pitas District Health Office	Institute for Medical Research, Infectious Disease Research Centre, National Institutes of Health, Ministry of Health Malaysia	Ahmad FA; Ahmad Fazilah NA; Anasir MI; Azizan MA; Kamel K; Mohamad Sukri MZ; Mohd Zawawi Z; Norhisham SN; Ramly N; Robert F; Rosli NR; Suppiah J; Thayan R
EPI_ISL_6422494	Queen Elizabeth Hospital	Institute for Medical Research, Infectious Disease Research Centre, National Institutes of Health, Ministry of Health Malaysia	Ahmad FA; Ahmad Fazilah NA; Anasir MI; Azizan MA; Kamel K; Mohd Zawawi Z; Norhisham SN; Ramly N; Robert F; Suppiah J; Thayan R
EPI_ISL_5850343	Ranau District Health Office	Institute for Medical Research, Infectious Disease Research Centre, National Institutes of Health, Ministry of Health Malaysia	Ahmad FA; Ahmad Fazilah NA; Anasir MI; Azizan MA; Kamel K; Mohd Zawawi Z; Norhisham SN; Ramly N; Robert F; Suppiah J; Thayan R
EPI_ISL_6586355, EPI_ISL_6586356, EPI_ISL_6586357, EPI_ISL_6586626, EPI_ISL_6586627, EPI_ISL_6586628, EPI_ISL_6586629, EPI_ISL_6586630, EPI_ISL_6586631, EPI_ISL_6586632, EPI_ISL_6586633, EPI_ISL_6586634, EPI_ISL_6586635, EPI_ISL_6586636, EPI_ISL_6586637, EPI_ISL_6586638, EPI_ISL_6586639, EPI_ISL_6586640, EPI_ISL_6586641, EPI_ISL_6586642, EPI_ISL_6586643, EPI_ISL_6586644, EPI_ISL_6586645, EPI_ISL_6586646, EPI_ISL_6586647, EPI_ISL_6586648, EPI_ISL_6968497, EPI_ISL_6968510, EPI_ISL_6969432, EPI_ISL_6969997, EPI_ISL_6970009, EPI_ISL_7472885, EPI_ISL_7472886, EPI_ISL_7472887, EPI_ISL_7472888, EPI_ISL_7472889, EPI_ISL_7472890, EPI_ISL_7472891, EPI_ISL_7473080, EPI_ISL_7473081, EPI_ISL_7473082, EPI_ISL_7473083, EPI_ISL_7473084			
see above	Rejang Medical Centre (Sibu)	Institute of Health and Community Medicine	Chan Chia Jui; Chua Hock Hin; David Perera; Ooi Mong How; Tan Lee See; Tonnii Sia Loong Loong; Wong Jyn Shan
EPI_ISL_6264274, EPI_ISL_6264280, EPI_ISL_6264289, EPI_ISL_6264290, EPI_ISL_6264292, EPI_ISL_6567065, EPI_ISL_6567067, EPI_ISL_6567076, EPI_ISL_6567079			
see above	SWAT TEAM HPSF	UKM Medical Molecular Biology Institute (UMBI)	Mira Farzana binti Mohamad Mokhtar
EPI_ISL_6586518, EPI_ISL_6586519, EPI_ISL_6586520, EPI_ISL_6586521, EPI_ISL_6586522, EPI_ISL_6586523, EPI_ISL_6586524, EPI_ISL_6586525, EPI_ISL_6586526, EPI_ISL_6586527, EPI_ISL_6586528, EPI_ISL_6586529, EPI_ISL_6586530, EPI_ISL_6586531, EPI_ISL_6586532, EPI_ISL_6586533, EPI_ISL_6586534, EPI_ISL_6586535, EPI_ISL_6586536, EPI_ISL_6586537, EPI_ISL_6586538, EPI_ISL_6586539, EPI_ISL_6586540, EPI_ISL_6586541, EPI_ISL_6586542, EPI_ISL_6586543, EPI_ISL_6586582, EPI_ISL_6586583, EPI_ISL_6586584, EPI_ISL_6586587, EPI_ISL_6586588, EPI_ISL_6586691, EPI_ISL_6586692, EPI_ISL_6586693, EPI_ISL_6586694, EPI_ISL_6586695, EPI_ISL_6586696, EPI_ISL_6586697, EPI_ISL_7472985, EPI_ISL_7472986, EPI_ISL_7472987, EPI_ISL_7472988, EPI_ISL_7472989, EPI_ISL_7472990			
see above	Sarawak General Hospital (Kuching)	Institute of Health and Community Medicine	Chan Chia Jui; Chua Hock Hin; David Perera; Lim Woon Hook; Ooi Mong How; Tonnii Sia Loong Loong; Wong Jyn Shan
EPI_ISL_6774035	Seberang Perai Tengah District Health Office	Institute for Medical Research, Infectious Disease Research Centre, National Institutes of Health, Ministry of Health Malaysia	Ahmad FA; Ahmad Fazilah NA; Anasir MI; Azizan MA; Kamel K; Mohamad Sukri MZ; Mohd Zawawi Z; Norhisham SN; Ramly N; Robert F; Rosli NR; Suppiah J; Thayan R
EPI_ISL_6914647, EPI_ISL_6914733, EPI_ISL_6915002	Selangor State Health Department	Institute for Medical Research, Infectious Disease Research Centre, National Institutes of Health, Ministry of Health Malaysia	Ahmad FA; Ahmad Fazilah NA; Anasir MI; Azizan MA; Kamel K; Mohamad Sukri MZ; Mohd Zawawi Z; Norhisham SN; Ramly N; Robert F; Rosli NR; Suppiah J; Thayan R

EPI_ISL_9210571	Sipitang District Health Office	Institute for Medical Research, Infectious Disease Research Centre, National Institutes of Health, Ministry of Health Malaysia	Ahmad FA; Ahmad Fazilah NA; Anasir MI; Azizan MA; Kamel K; Mohamad Sukri MZ; Norhisham SN; Ramly N; Robert F; Rosli NR; Suppiah J; Thayan R
EPI_ISL_6423136	Tambunan Hospital	Institute for Medical Research, Infectious Disease Research Centre, National Institutes of Health, Ministry of Health Malaysia	Ahmad FA; Ahmad Fazilah NA; Anasir MI; Azizan MA; Kamel K; Mohd Zawawi Z; Norhisham SN; Ramly N; Robert F; Suppiah J; Thayan R
EPI_ISL_6422721	Tengku Ampuan Jemaah Hospital	Institute for Medical Research, Infectious Disease Research Centre, National Institutes of Health, Ministry of Health Malaysia	Ahmad FA; Ahmad Fazilah NA; Anasir MI; Azizan MA; Kamel K; Mohd Zawawi Z; Norhisham SN; Ramly N; Robert F; Suppiah J; Thayan R
EPI_ISL_6423165, EPI_ISL_7457755	Tenom District Health Office	Institute for Medical Research, Infectious Disease Research Centre, National Institutes of Health, Ministry of Health Malaysia	Ahmad FA; Ahmad Fazilah NA; Anasir MI; Azizan MA; Kamel K; Mohamad Sukri MZ; Mohd Zawawi Z; Norhisham SN; Ramly N; Robert F; Rosli NR; Suppiah J; Thayan R
EPI_ISL_6424684	Terengganu Health Department	Institute for Medical Research, Infectious Disease Research Centre, National Institutes of Health, Ministry of Health Malaysia	Ahmad FA; Ahmad Fazilah NA; Anasir MI; Azizan MA; Kamel K; Mohd Zawawi Z; Norhisham SN; Ramly N; Robert F; Suppiah J; Thayan R
EPI_ISL_6452649	UKA HPSF	UKM Medical Molecular Biology Institute (UMBI)	Mira Farzana binti Mohamad Mokhtar
EPI_ISL_6131999, EPI_ISL_6132012, EPI_ISL_6132026, EPI_ISL_6132054	UMMC	Department of Medical Microbiology, Faculty of Medicine, University of Malaya; University of Malaya Medical Centre	I-Ching SAM; Jolene Yin Ling FU; Omar Khalilur Rahman; Yoke Fun Chan
EPI_ISL_8606778	UNIT KESIHATAN AWAM	UKM Medical Molecular Biology Institute (UMBI)	Mira Farzana binti Mohamad Mokhtar
EPI_ISL_6264278, EPI_ISL_6264281, EPI_ISL_6264282, EPI_ISL_6452551, EPI_ISL_6773823, EPI_ISL_6773830, EPI_ISL_6773832, EPI_ISL_6773835, EPI_ISL_6773836, EPI_ISL_8438471, EPI_ISL_8438472			
see above	UNIT KESIHATAN AWAM HEBHK	UKM Medical Molecular Biology Institute (UMBI)	Mira Farzana binti Mohamad Mokhtar
EPI_ISL_6713022, EPI_ISL_6713027, EPI_ISL_6713034	covid ward HPP	iPROMISE, UiTM	Ariza Adnan; Fadzilah Mohd Nor; Lim Wai Feng; Mohd Asif Mohd Sukri; Mohd Nur Fakhruzzaman Noorizhab; Mohd Zaki Salleh; Sazzli Shahlan Kassim; Siti Farah Alwani Mohd Nawi; Siti Hamimah Sheikh Abdul Kadir; Teh Lay Kek; Wang Seok Mui

We gratefully acknowledge the following Authors from the Originating laboratories responsible for obtaining the specimens, as well as the Submitting laboratories where the genome data were generated and shared via GISAID, on which this research is based.

All Submitters of data may be contacted directly via www.gisaid.org

Authors are sorted alphabetically.

Accession ID	Originating Laboratory	Submitting Laboratory	Authors
EPI_ISL_8693363	Ampang Hospital	Institute for Medical Research, Infectious Disease Research Centre, National Institutes of Health, Ministry of Health Malaysia	Ahmad FA; Ahmad Fazilah NA; Anasir MI; Azizan MA; Kamel K; Mohamad Sukri MZ; Norhisham SN; Ramly N; Robert F; Rosli NR; Suppiah J; Thayan R
EPI_ISL_6969830, EPI_ISL_6969840, EPI_ISL_6969849, EPI_ISL_6969857, EPI_ISL_6969873, EPI_ISL_6969883, EPI_ISL_6969903, EPI_ISL_6969914, EPI_ISL_6969922, EPI_ISL_6969930, EPI_ISL_6969940, EPI_ISL_6969947, EPI_ISL_6969958, EPI_ISL_6969971, EPI_ISL_6969978, EPI_ISL_6969989, EPI_ISL_6970411, EPI_ISL_6970412, EPI_ISL_6970413, EPI_ISL_6970414, EPI_ISL_6970415, EPI_ISL_6970416, EPI_ISL_6970417, EPI_ISL_6970437			
see above	Bintulu Hospital PCR Lab, Bintulu	Institute of Health and Community Medicine	Chan Chia Jui; Chua Hock Hin; David Perera; Ooi Mong How; Tan Lee See; Tonnii Sia Loong Loong; Wong Jyn Shan
EPI_ISL_6969679, EPI_ISL_6969693, EPI_ISL_6969706, EPI_ISL_6969715, EPI_ISL_6969722, EPI_ISL_6969732, EPI_ISL_6969744, EPI_ISL_6969750, EPI_ISL_6969765, EPI_ISL_6969775, EPI_ISL_6969782, EPI_ISL_6969790, EPI_ISL_6969797, EPI_ISL_6970409, EPI_ISL_6970446, EPI_ISL_7472923, EPI_ISL_7472924, EPI_ISL_7472925, EPI_ISL_7472926			
see above	Bintulu Medical Centre (Bintulu)	Institute of Health and Community Medicine	Chan Chia Jui; Chua Hock Hin; David Perera; Ooi Mong How; Tonnii Sia Loong Loong; Wong Jyn Shan
EPI_ISL_6968529, EPI_ISL_6968536, EPI_ISL_6968543, EPI_ISL_6968555, EPI_ISL_6968564, EPI_ISL_6968576, EPI_ISL_6968593, EPI_ISL_6968603, EPI_ISL_6968608, EPI_ISL_6968619, EPI_ISL_6968638, EPI_ISL_6968646, EPI_ISL_6968658, EPI_ISL_6968671, EPI_ISL_6968681, EPI_ISL_6968692, EPI_ISL_6968703, EPI_ISL_6968726, EPI_ISL_6968733, EPI_ISL_6968751, EPI_ISL_6968760, EPI_ISL_6968774, EPI_ISL_6968790, EPI_ISL_6968799, EPI_ISL_6969471, EPI_ISL_6969483, EPI_ISL_6969497, EPI_ISL_6969513, EPI_ISL_6969523, EPI_ISL_6970380, EPI_ISL_6970404, EPI_ISL_6970441, EPI_ISL_7472895, EPI_ISL_7472896, EPI_ISL_7472897, EPI_ISL_7472898, EPI_ISL_7472899, EPI_ISL_7472900, EPI_ISL_7472916, EPI_ISL_7472918, EPI_ISL_7472919, EPI_ISL_7472920, EPI_ISL_7472921			
see above	Borneo Medical Centre (Kuching)	Institute of Health and Community Medicine	Chan Chia Jui; Chua Hock Hin; David Perera; Ooi Mong How; Tonnii Sia Loong Loong; Wong Jyn Shan
EPI_ISL_8405660	Bukit Changgang Health Clinic	Institute for Medical Research, Infectious Disease Research Centre, National Institutes of Health, Ministry of Health Malaysia	Ahmad FA; Ahmad Fazilah NA; Anasir MI; Azizan MA; Kamel K; Mohamad Sukri MZ; Norhisham SN; Ramly N; Robert F; Rosli NR; Suppiah J; Thayan R
EPI_ISL_6964571	COVID WARD HPP	iPROMISE, UiTM	Ariza Adnan; Fadzilah Mohd Nor; Lim Wai Feng; Mohd Asif Mohd Sukri; Mohd Nur Fakhruzzaman Noorizhab; Mohd Zaki Salleh; Sazzli Shahlan Kassim; Siti Farah Alwani Mohd Nawi; Siti Hamimah Sheikh Abdul Kadir; Teh Lay Kek; Wang Seok Mui
EPI_ISL_8095535, EPI_ISL_8095536, EPI_ISL_8170441	CPRC JKNT	Tropical Infectious Diseases Research & Education Centre (TIDREC), Universiti Malaya	AsmaAnati CheMatSeri; Che-Norainon Yaacob; Jia-Yi Tan; Jo-Ern Wong; Kim-Kee Tan; Mulya-Mustika-Sari Zulkifli; Noor Syahida Azizan; Nur-Hidayana Mahfodz; Sazaly AbuBakar; Siti-Sarah Nor'e
EPI_ISL_10070315, EPI_ISL_10070358	Department of Medical Microbiology, Hospital Pengajar Universiti Putra Malaysia	Department of Medical Microbiology, Hospital Pengajar Universiti Putra Malaysia	Afiqah Adzmi; Amiza Azmi; Azmiza Syawani Jasni; Hui Yee Chee; Leslie Thian Lung Than; Muadz Mohtar; Muhammad Mohd Isa; Narcisse MS Joseph; Niazlin Mohd Taib; Noor Hazirah Noor Azhari; Norlaila; Nur Raihana Ithnin; Nurul Huda Mohamed Rashidi; Nurul Nadiah Ismail; Rosni Ibrahim; Rukman Awang Hamat; Sallehhudin; Siti Norbaya Masri; Siti Zulaikha Zakariah; Syafinaz Amin Nordin; Tengku Zetty Maztura Tengku Jamaluddin; Zamberi Sekawi
EPI_ISL_8188771, EPI_ISL_8188775	Gribbles Pathology, Kuching	Institute of Health and Community Medicine	Chan Chia Jui; Chua Hock Hin; David Perera; Josephine Liew Chin Chin; Ooi Mong How; Tonnii Sia Loong Loong; Wong Jyn Shan
EPI_ISL_12628171	HCTM	UKM Medical Molecular Biology Institute (UMBI)	Khairun Nur Abd Ghafar; Mira Farzana Mohamad Mokhtar; Muhiddin Ishak; Nor Azila Muhammad Azami; Nur Alyaa Afifah Md Shahri; Nurul Syakima Ab Mutalib; Rahman Jamal; Ryia Illani Mohd Yunos; Siti Nur Hasanah Mohd Yusuf; Zahirrah Begam Mohamed Rasheed
EPI_ISL_8500646, EPI_ISL_9000486	HOSPITAL PULAU PINANG	iPROMISE, UiTM	Ariza Adnan; Fadzilah Mohd Nor; Lim Wai Feng; Mohd Asif Mohd Sukri; Mohd Nur Fakhruzzaman Noorizhab; Mohd Zaki Salleh; Sazzli Shahlan Kassim; Siti Farah Alwani Mohd Nawi; Siti Hamimah Sheikh Abdul Kadir; Teh Lay Kek; Wang Seok Mui
EPI_ISL_8254325, EPI_ISL_8254327, EPI_ISL_8254328, EPI_ISL_8254332, EPI_ISL_8254341	HOSPITAL RAJA PERMAISURI BAINUN	Department of Medical Microbiology, Faculty of Medicine, University of Malaya; University of Malaya Medical Centre	I-Ching SAM; Jolene Yin Ling FU; Omar Khalilur Rahman; Yoke Fun Chan
EPI_ISL_8095530, EPI_ISL_8095531, EPI_ISL_8095533, EPI_ISL_8095534	HOSPITAL RAJA PERMAISURI BAINUN	Tropical Infectious Diseases Research & Education Centre (TIDREC), Universiti Malaya	AsmaAnati CheMatSeri; Che-Norainon Yaacob; Jia-Yi Tan; Jo-Ern Wong; Kim-Kee Tan; Mulya-Mustika-Sari Zulkifli; Noor Syahida Azizan; Nur-Hidayana Mahfodz; Sazaly AbuBakar; Siti-Sarah Nor'e
EPI_ISL_6773837	HOSPITAL TUANKU JAAFAR SEREMBAN	UKM Medical Molecular Biology Institute (UMBI)	Mira Farzana binti Mohamad Mokhtar
EPI_ISL_10969114	HRPB	Tropical Infectious Diseases Research & Education Centre (TIDREC), Universiti Malaya	AsmaAnati CheMatSeri; Che-Norainon Yaacob; Jia-Yi Tan; Jo-Ern Wong; Kim-Kee Tan; Mulya-Mustika-Sari Zulkifli; Noor Syahida Azizan; Nur-Hidayana Mahfodz; Sazaly AbuBakar; Siti-Sarah Nor'e
EPI_ISL_11010502	HRPZ 11	Tropical Infectious Diseases Research & Education Centre (TIDREC), Universiti Malaya	AsmaAnati CheMatSeri; Che-Norainon Yaacob; Jia-Yi Tan; Jo-Ern Wong; Kim-Kee Tan; Mulya-Mustika-Sari Zulkifli; Noor Syahida Azizan; Nur-Hidayana Mahfodz; Sazaly AbuBakar; Siti-Sarah Nor'e
EPI_ISL_10969111	HRPZ II	Tropical Infectious Diseases Research & Education Centre (TIDREC), Universiti Malaya	AsmaAnati CheMatSeri; Che-Norainon Yaacob; Jia-Yi Tan; Jo-Ern Wong; Kim-Kee Tan; Mulya-Mustika-Sari Zulkifli; Noor Syahida Azizan; Nur-Hidayana Mahfodz; Sazaly AbuBakar; Siti-Sarah Nor'e
EPI_ISL_11900033, EPI_ISL_11900037, EPI_ISL_11900038, EPI_ISL_11900042	Hospital Kajang	Malaysia Genome and Vaccine Institute	Azrin Ahmad; Enizza Kasim; Irni Suhayu Sapian; Mohd Faizal Abu Bakar; Mohd Ghows Mohd Azzam.; Mohd Noor Mat Isa; Nor Azfa Johari; Nurhezreen Md Iqbal; Shamsidar Sopie; Siti Noraini Othman; Yusuf Muhammad Noor
EPI_ISL_7260349	Hospital Keningau	Malaysia Genome Institute	Azrin Ahmad; Enizza Kasim; Irni Suhayu Sapian; Mohd Faizal Abu Bakar; Mohd Ghows Mohd Azzam.; Mohd Noor Mat Isa; Nor Azfa Johari; Nurhezreen Md Iqbal; Shamsidar Sopie; Siti Noraini Othman; Yusuf Muhammad Noor
EPI_ISL_9000489	Hospital Kepala Batas	iPROMISE, UiTM	Ariza Adnan; Fadzilah Mohd Nor; Lim Wai Feng; Mohd Asif Mohd Sukri; Mohd Nur Fakhruzzaman Noorizhab; Mohd Zaki Salleh; Sazzli Shahlan Kassim; Siti Farah Alwani Mohd Nawi; Siti Hamimah Sheikh Abdul Kadir; Teh Lay Kek; Wang Seok Mui
EPI_ISL_7260264	Hospital Kuala Lumpur	Malaysia Genome Institute	Azrin Ahmad; Enizza Kasim; Irni Suhayu Sapian; Mohd Faizal Abu Bakar; Mohd Ghows Mohd Azzam.; Mohd Noor Mat Isa; Nor Azfa Johari; Nurhezreen Md Iqbal; Shamsidar Sopie; Siti Noraini Othman; Yusuf Muhammad Noor
EPI_ISL_7260252	Hospital Kudat	Malaysia Genome Institute	Azrin Ahmad; Enizza Kasim; Irni Suhayu Sapian; Mohd Faizal Abu Bakar; Mohd Ghows Mohd Azzam.; Mohd Noor Mat Isa; Nor Azfa Johari; Nurhezreen Md Iqbal; Shamsidar Sopie; Siti Noraini Othman; Yusuf Muhammad Noor
EPI_ISL_6964578, EPI_ISL_6964606, EPI_ISL_6964641	Hospital Pulau Pinang	iPROMISE, UiTM	Ariza Adnan; Fadzilah Mohd Nor; Lim Wai Feng; Mohd Asif Mohd Sukri; Mohd Nur Fakhruzzaman Noorizhab; Mohd Zaki Salleh; Sazzli Shahlan Kassim; Siti Farah Alwani Mohd Nawi; Siti Hamimah Sheikh Abdul Kadir; Teh Lay Kek; Wang Seok Mui
EPI_ISL_8095537	Hospital Raja Perempuan Zainab 2	Tropical Infectious Diseases Research & Education Centre (TIDREC), Universiti Malaya	AsmaAnati CheMatSeri; Che-Norainon Yaacob; Jia-Yi Tan; Jo-Ern Wong; Kim-Kee Tan; Mulya-Mustika-Sari Zulkifli; Noor Syahida Azizan; Nur-Hidayana Mahfodz; Sazaly AbuBakar; Siti-Sarah Nor'e
EPI_ISL_6773828	Hospital Segamat	UKM Medical Molecular Biology Institute (UMBI)	Mira Farzana binti Mohamad Mokhtar
EPI_ISL_7260274	Hospital Tengku Ampuan Jemaah	Malaysia Genome Institute	Azrin Ahmad; Enizza Kasim; Irni Suhayu Sapian; Mohd Faizal Abu Bakar; Mohd Ghows Mohd Azzam.; Mohd Noor Mat Isa; Nor Azfa Johari; Nurhezreen Md Iqbal; Shamsidar Sopie; Siti Noraini Othman; Yusuf Muhammad Noor
EPI_ISL_8095538	Hospital tanah merah	Tropical Infectious Diseases Research & Education Centre (TIDREC), Universiti Malaya	AsmaAnati CheMatSeri; Che-Norainon Yaacob; Jia-Yi Tan; Jo-Ern Wong; Kim-Kee Tan; Mulya-Mustika-Sari Zulkifli; Noor Syahida Azizan; Nur-Hidayana Mahfodz; Sazaly AbuBakar; Siti-Sarah Nor'e
EPI_ISL_6567066, EPI_ISL_6567071, EPI_ISL_6567072, EPI_ISL_6567074, EPI_ISL_6567075	INSTITUT BIOLOGI MOLEKUL PERUBATAN UKM (UMBI)	UKM Medical Molecular Biology Institute (UMBI)	Mira Farzana binti Mohamad Mokhtar
EPI_ISL_7119730, EPI_ISL_7119740, EPI_ISL_7119744, EPI_ISL_7119747, EPI_ISL_7119753, EPI_ISL_7119763, EPI_ISL_7119770, EPI_ISL_7119782, EPI_ISL_7119792, EPI_ISL_11078681, EPI_ISL_11078694, EPI_ISL_11078723, EPI_ISL_11078735, EPI_ISL_11078737, EPI_ISL_11078741, EPI_ISL_11078758, EPI_ISL_11078772, EPI_ISL_11078785, EPI_ISL_11323771, EPI_ISL_11323788, EPI_ISL_11323817			
see above	Institut Biologi Molekul Perubatan UKM (UMBI)	UKM Medical Molecular Biology Institute (UMBI)	Ab Mutalib; Abd Ghafar; Ishak; Jamal; Khairun Nur; Khairun Nur Abd Ghafar; Md Shahri; Mira Farzana; Mira Farzana Mohamad Mokhtar; Mohamad Mokhtar; Mohamed Rasheed; Mohd Yunos; Mohd Yusuf; Muhammad Azami; Muhiddin; Muhiddin Ishak; Nor Azi; Nor Azila Muhammad Azami; Nur Alyaa Afifah; Nur Alyaa Afifah Md Shahri; Nurul Syakima; Nurul Syakima Ab Mutalib; Rahman; Rahman Jamal; Ryia Illani; Ryia Illani Mohd Yunos; Siti Nur Hasanah; Siti Nur Hasanah Mohd Yusuf; Zahirrah Begam; Zahirrah Begam Mohamed Rasheed
EPI_ISL_8083704, EPI_ISL_8083707, EPI_ISL_8083708, EPI_ISL_8500649, EPI_ISL_9000518, EPI_ISL_11939203	JKN PAHANG	iPROMISE, UiTM	Ariza Adnan; Fadzilah Mohd Nor; Lim Wai Feng; Mohd Asif Mohd Sukri; Mohd Nur Fakhruzzaman Noorizhab; Mohd Zaki Salleh; Sazzli Shahlan Kassim; Siti Farah Alwani Mohd Nawi; Siti Hamimah Sheikh Abdul Kadir; Teh Lay Kek; Wang Seok Mui
EPI_ISL_8254284	Jabatan Forensik Hospital Sultanah Bahiyah	Department of Medical Microbiology, Faculty of Medicine, University of Malaya; University of Malaya Medical Centre	I-Ching SAM; Jolene Yin Ling FU; Omar Khalilur Rahman; Yoke Fun Chan
EPI_ISL_8170534	Jabatan Forensik Hospital Sultanah Bahiyah	Tropical Infectious Diseases Research & Education Centre (TIDREC), Universiti Malaya	AsmaAnati CheMatSeri; Che-Norainon Yaacob; Jia-Yi Tan; Jo-Ern Wong; Kim-Kee Tan; Mulya-Mustika-Sari Zulkifli; Noor Syahida Azizan; Nur-Hidayana Mahfodz; Sazaly AbuBakar; Siti-Sarah Nor'e
EPI_ISL_7260281, EPI_ISL_7260289, EPI_ISL_7260297, EPI_ISL_7260305, EPI_ISL_7260315, EPI_ISL_7260325, EPI_ISL_7260334			
see above	Jabatan Kesihatan WP Labuan	Malaysia Genome Institute	Azrin Ahmad; Enizza Kasim; Irni Suhayu Sapian; Mohd Faizal Abu Bakar; Mohd Ghows Mohd Azzam.; Mohd Noor Mat Isa; Nor Azfa Johari; Nurhezreen Md Iqbal; Shamsidar Sopie; Siti Noraini Othman; Yusuf Muhammad Noor
EPI_ISL_8083686	KK PEKAN AWAH	iPROMISE, UiTM	Ariza Adnan; Fadzilah Mohd Nor; Lim Wai Feng; Mohd Asif Mohd Sukri; Mohd Nur Fakhruzzaman Noorizhab; Mohd Zaki Salleh; Sazzli Shahlan Kassim; Siti Farah Alwani Mohd Nawi; Siti Hamimah Sheikh Abdul Kadir; Teh Lay Kek; Wang Seok Mui
EPI_ISL_8095529	KLINIK KESIHATAN PERINGAT	Tropical Infectious Diseases Research & Education Centre (TIDREC), Universiti Malaya	AsmaAnati CheMatSeri; Che-Norainon Yaacob; Jia-Yi Tan; Jo-Ern Wong; Kim-Kee Tan; Mulya-Mustika-Sari Zulkifli; Noor Syahida Azizan; Nur-Hidayana Mahfodz; Sazaly AbuBakar; Siti-Sarah Nor'e
EPI_ISL_8170442	KOMPLEKS SURVELAN PKD KINTA	Tropical Infectious Diseases Research & Education Centre (TIDREC), Universiti Malaya	AsmaAnati CheMatSeri; Che-Norainon Yaacob; Jia-Yi Tan; Jo-Ern Wong; Kim-Kee Tan; Mulya-Mustika-Sari Zulkifli; Noor Syahida Azizan; Nur-Hidayana Mahfodz; Sazaly AbuBakar; Siti-Sarah Nor'e
EPI_ISL_8405627, EPI_ISL_8692555	Kampung Bandar Health Clinic	Institute for Medical Research, Infectious Disease Research Centre, National Institutes of Health, Ministry of Health Malaysia	Ahmad FA; Ahmad Fazilah NA; Anasir MI; Azizan MA; Kamel K; Mohamad Sukri MZ; Norhisham SN; Ramly N; Robert F; Rosli NR; Suppiah J; Thayan R
EPI_ISL_8170436	Kk perol	Tropical Infectious Diseases Research & Education Centre (TIDREC), Universiti Malaya	AsmaAnati CheMatSeri; Che-Norainon Yaacob; Jia-Yi Tan; Jo-Ern Wong; Kim-Kee Tan; Mulya-Mustika-Sari Zulkifli; Noor Syahida Azizan; Nur-Hidayana Mahfodz; Sazaly AbuBakar; Siti-Sarah Nor'e
EPI_ISL_8692658	Kuala Langat District Health Office	Institute for Medical Research, Infectious Disease Research Centre, National Institutes of Health, Ministry of Health Malaysia	Ahmad FA; Ahmad Fazilah NA; Anasir MI; Azizan MA; Kamel K; Mohamad Sukri MZ; Norhisham SN; Ramly N; Robert F; Rosli NR; Suppiah J; Thayan R

EPI_ISL_7457660, EPI_ISL_7457906, EPI_ISL_7457908, EPI_ISL_7468575, EPI_ISL_7468581, EPI_ISL_7468599, EPI_ISL_7468609, EPI_ISL_7468610, EPI_ISL_7468611, EPI_ISL_7468612, EPI_ISL_7468616, EPI_ISL_7468617, EPI_ISL_7468618, EPI_ISL_7468619, EPI_ISL_7468620, EPI_ISL_7468624, EPI_ISL_7468626, EPI_ISL_7468635, EPI_ISL_7888696, EPI_ISL_7888903			
see above	Kuala Lumpur International Airport (KLIA) Health Office	Institute for Medical Research, Infectious Disease Research Centre, National Institutes of Health, Ministry of Health Malaysia	Ahmad FA; Ahmad Fazilah NA; Anasir MI; Azizan MA; Kamel K; Mohamad Sukri MZ; Mohd Zawawi Z; Norhisham SN; Ramly N; Robert F; Rosli NR; Suppiah J; Thayan R
EPI_ISL_6964560, EPI_ISL_6964567	Medical Hospital Pulau Pinang	iPROMISE, UiTM	Ariza Adnan; Fadzilah Mohd Nor; Lim Wai Feng; Mohd Asif Mohd Sukri; Mohd Nur Fakhruzzaman Noorizhab; Mohd Zaki Salleh; Sazzli Shahlan Kassim; Siti Farah Alwani Mohd Nawi; Siti Hamimah Sheikh Abdul Kadir; Teh Lay Kek; Wang Seok Mui
EPI_ISL_7457916	National Institutes of Health	Institute for Medical Research, Infectious Disease Research Centre, National Institutes of Health, Ministry of Health Malaysia	Ahmad FA; Ahmad Fazilah NA; Anasir MI; Azizan MA; Kamel K; Mohamad Sukri MZ; Mohd Zawawi Z; Norhisham SN; Ramly N; Robert F; Rosli NR; Suppiah J; Thayan R
EPI_ISL_6773833, EPI_ISL_6773839	PEJABAT KESIHATAN DAERAH MELAKA TENGAH	UKM Medical Molecular Biology Institute (UMBI)	Mira Farzana binti Mohamad Mokhtar
EPI_ISL_8083688, EPI_ISL_8083692, EPI_ISL_8083693, EPI_ISL_9000511	PEJABAT KESIHATAN DAERAH SEBERANG PERAI UTARA PULAU PINANG	iPROMISE, UiTM	Ariza Adnan; Fadzilah Mohd Nor; Lim Wai Feng; Mohd Asif Mohd Sukri; Mohd Nur Fakhruzzaman Noorizhab; Mohd Zaki Salleh; Sazzli Shahlan Kassim; Siti Farah Alwani Mohd Nawi; Siti Hamimah Sheikh Abdul Kadir; Teh Lay Kek; Wang Seok Mui
EPI_ISL_6705504	PEJABAT KESIHATAN DAERAH SEREMBAN	UKM Medical Molecular Biology Institute (UMBI)	Mira Farzana binti Mohamad Mokhtar
EPI_ISL_8083694, EPI_ISL_8083695, EPI_ISL_8083696, EPI_ISL_8083697, EPI_ISL_8083698, EPI_ISL_8083699, EPI_ISL_8083700, EPI_ISL_8083701			
see above	PEJABAT KESIHATAN DAERAH TIMUR LAUT	iPROMISE, UiTM	Ariza Adnan; Fadzilah Mohd Nor; Lim Wai Feng; Mohd Asif Mohd Sukri; Mohd Nur Fakhruzzaman Noorizhab; Mohd Zaki Salleh; Sazzli Shahlan Kassim; Siti Farah Alwani Mohd Nawi; Siti Hamimah Sheikh Abdul Kadir; Teh Lay Kek; Wang Seok Mui
EPI_ISL_9000487, EPI_ISL_9000488	PKD BARAT DAYA	iPROMISE, UiTM	Ariza Adnan; Fadzilah Mohd Nor; Lim Wai Feng; Mohd Asif Mohd Sukri; Mohd Nur Fakhruzzaman Noorizhab; Mohd Zaki Salleh; Sazzli Shahlan Kassim; Siti Farah Alwani Mohd Nawi; Siti Hamimah Sheikh Abdul Kadir; Teh Lay Kek; Wang Seok Mui
EPI_ISL_6705559, EPI_ISL_6773819, EPI_ISL_6773826, EPI_ISL_6773827, EPI_ISL_6773829	PKD MELAKA TENGAH	UKM Medical Molecular Biology Institute (UMBI)	Mira Farzana binti Mohamad Mokhtar
EPI_ISL_8254329, EPI_ISL_8254336, EPI_ISL_8254337	PKD Manjung	Department of Medical Microbiology, Faculty of Medicine, University of Malaya; University of Malaya Medical Centre	I-Ching SAM; Jolene Yin Ling FU; Omar Khalilur Rahman; Yoke Fun Chan
EPI_ISL_8170479	PKD Manjung	Tropical Infectious Diseases Research & Education Centre (TIDREC), Universiti Malaya	AsmaAnati CheMatSeri; Che-Norainon Yaacob; Jia-Yi Tan; Jo-Ern Wong; Kim-Kee Tan; Mulya-Mustika-Sari Zulkifli; Noor Syahida Azizan; Nur-Hidayana Mahfodz; Sazaly AbuBakar; Siti-Sarah Nor'e
EPI_ISL_8095528, EPI_ISL_8170437	PKD PASIR PUTEH	Tropical Infectious Diseases Research & Education Centre (TIDREC), Universiti Malaya	AsmaAnati CheMatSeri; Che-Norainon Yaacob; Jia-Yi Tan; Jo-Ern Wong; Kim-Kee Tan; Mulya-Mustika-Sari Zulkifli; Noor Syahida Azizan; Nur-Hidayana Mahfodz; Sazaly AbuBakar; Siti-Sarah Nor'e
EPI_ISL_8170440	PKD PERAK TENGAH	Tropical Infectious Diseases Research & Education Centre (TIDREC), Universiti Malaya	AsmaAnati CheMatSeri; Che-Norainon Yaacob; Jia-Yi Tan; Jo-Ern Wong; Kim-Kee Tan; Mulya-Mustika-Sari Zulkifli; Noor Syahida Azizan; Nur-Hidayana Mahfodz; Sazaly AbuBakar; Siti-Sarah Nor'e
EPI_ISL_8314867, EPI_ISL_8314870, EPI_ISL_8314871	PKD SEGAMAT	UKM Medical Molecular Biology Institute (UMBI)	Khairun Nur Abd Ghafar; Mira Farzana Mohamad Mokhtar; Muhiddin Ishak; Nor Azila Muhammad Azami; Nur Alyaa Afifah Md Shahri; Nurul Syakima Ab Mutalib; Rahman Jamal; Ryia Illani Mohd Yunos; Siti Nur Hasanah Mohd Yusuf; Zahirrah Begam Mohamed Rasheed
EPI_ISL_7464479, EPI_ISL_7464480, EPI_ISL_7464482	PKD SEREMBAN	UKM Medical Molecular Biology Institute (UMBI)	Ab Mutalib; Abd Ghafar; Ishak; Jamal; Khairun Nur; Md Shahri; Mira Farzana; Mohamad Mokhtar; Mohamed Rasheed; Mohd Yunos; Mohd Yusuf; Muhammad Azami; Muhiddin; Nor Azi; Nur Alyaa Afifah; Nurul Syakima; Rahman; Ryia Illani; Siti Nur Hasanah; Zahirrah Begam
EPI_ISL_8083689, EPI_ISL_8083690, EPI_ISL_8083691, EPI_ISL_8083702, EPI_ISL_8083703, EPI_ISL_8500650, EPI_ISL_8500651, EPI_ISL_8500691, EPI_ISL_9000504			
see above	PKD SPT	iPROMISE, UiTM	Ariza Adnan; Fadzilah Mohd Nor; Lim Wai Feng; Mohd Asif Mohd Sukri; Mohd Nur Fakhruzzaman Noorizhab; Mohd Zaki Salleh; Sazzli Shahlan Kassim; Siti Farah Alwani Mohd Nawi; Siti Hamimah Sheikh Abdul Kadir; Teh Lay Kek; Wang Seok Mui
EPI_ISL_7987087, EPI_ISL_7987095, EPI_ISL_7987098, EPI_ISL_7987100, EPI_ISL_7987101, EPI_ISL_7987107, EPI_ISL_7987108			
see above	PKD Segamat	UKM Medical Molecular Biology Institute (UMBI)	Ab Mutalib; Abd Ghafar; Ishak; Jamal; Khairun Nur; Md Shahri; Mira Farzana; Mohamad Mokhtar; Mohamed Rasheed; Mohd Yunos; Mohd Yusuf; Muhammad Azami; Muhiddin; Nor Azila; Nur Alyaa Afifah; Nurul Syakima; Rahman; Ryia Illani; Siti Nur Hasanah; Zahirrah Begam
EPI_ISL_6705470, EPI_ISL_6705476, EPI_ISL_6705512, EPI_ISL_6705522, EPI_ISL_6705536, EPI_ISL_6705544, EPI_ISL_6705565, EPI_ISL_6705572, EPI_ISL_6705602, EPI_ISL_7468518, EPI_ISL_7468521, EPI_ISL_7468523, EPI_ISL_7468526, EPI_ISL_7468527, EPI_ISL_7468530, EPI_ISL_7468533			
see above	PKD TAMPIN	UKM Medical Molecular Biology Institute (UMBI)	Ab Mutalib; Abd Ghafar; Ishak; Jamal; Khairun Nur; Md Shahri; Mira Farzana; Mira Farzana binti Mohamad Mokhtar; Mohamad Mokhtar; Mohamed Rasheed; Mohd Yunos; Mohd Yusuf; Muhammad Azami; Muhiddin; Nor Azila; Nur Alyaa Afifah; Nurul Syakima; Rahman; Ryia Illani; Siti Nur Hasanah; Zahirrah Begam
EPI_ISL_6773821, EPI_ISL_6773824	PKD TANGKAK	UKM Medical Molecular Biology Institute (UMBI)	Mira Farzana binti Mohamad Mokhtar
EPI_ISL_6773838	PKD Tangkak	UKM Medical Molecular Biology Institute (UMBI)	Mira Farzana binti Mohamad Mokhtar
EPI_ISL_6964464, EPI_ISL_6964630, EPI_ISL_6964668	PKPMA	iPROMISE, UiTM	Ariza Adnan; Fadzilah Mohd Nor; Lim Wai Feng; Mohd Asif Mohd Sukri; Mohd Nur Fakhruzzaman Noorizhab; Mohd Zaki Salleh; Sazzli Shahlan Kassim; Siti Farah Alwani Mohd Nawi; Siti Hamimah Sheikh Abdul Kadir; Teh Lay Kek; Wang Seok Mui
EPI_ISL_6964470	PKPMA PULAU PINANG	iPROMISE, UiTM	Ariza Adnan; Fadzilah Mohd Nor; Lim Wai Feng; Mohd Asif Mohd Sukri; Mohd Nur Fakhruzzaman Noorizhab; Mohd Zaki Salleh; Sazzli Shahlan Kassim; Siti Farah Alwani Mohd Nawi; Siti Hamimah Sheikh Abdul Kadir; Teh Lay Kek; Wang Seok Mui
EPI_ISL_6964484, EPI_ISL_6964657	PMA PULAU PINANG	iPROMISE, UiTM	Ariza Adnan; Fadzilah Mohd Nor; Lim Wai Feng; Mohd Asif Mohd Sukri; Mohd Nur Fakhruzzaman Noorizhab; Mohd Zaki Salleh; Sazzli Shahlan Kassim; Siti Farah Alwani Mohd Nawi; Siti Hamimah Sheikh Abdul Kadir; Teh Lay Kek; Wang Seok Mui
EPI_ISL_7260339	Pejabat Kesihatan Daerah Sipitang	Malaysia Genome Institute	Azrin Ahmad; Enizza Kasim; Irni Suhayu Sapian; Mohd Faizal Abu Bakar; Mohd Ghows Mohd Azzam.; Mohd Noor Mat Isa; Nor Azfa Johari; Nurhezreen Md Iqbal; Shamsidar Sopie; Siti Noraini Othman; Yusuf Muhammad Noor
EPI_ISL_8500692	Pejabat Kesihatan Pintu Masuk Antarabangsa	iPROMISE, UiTM	Ariza Adnan; Fadzilah Mohd Nor; Lim Wai Feng; Mohd Asif Mohd Sukri; Mohd Nur Fakhruzzaman Noorizhab; Mohd Zaki Salleh; Sazzli Shahlan Kassim; Siti Farah Alwani Mohd Nawi; Siti Hamimah Sheikh Abdul Kadir; Teh Lay Kek; Wang Seok Mui
EPI_ISL_7468563, EPI_ISL_7468578, EPI_ISL_7889378, EPI_ISL_8405661, EPI_ISL_8405662, EPI_ISL_8692651, EPI_ISL_8692654, EPI_ISL_8692655, EPI_ISL_8692656, EPI_ISL_8692657			
see above	Port Klang Health Clinic	Institute for Medical Research, Infectious Disease Research Centre, National Institutes of Health, Ministry of Health Malaysia	Ahmad FA; Ahmad Fazilah NA; Anasir MI; Azizan MA; Kamel K; Mohamad Sukri MZ; Mohd Zawawi Z; Norhisham SN; Ramly N; Robert F; Rosli NR; Suppiah J; Thayan R
EPI_ISL_9210396	Queen Elizabeth Hospital	Institute for Medical Research, Infectious Disease Research Centre, National Institutes of Health, Ministry of Health Malaysia	Ahmad FA; Ahmad Fazilah NA; Anasir MI; Azizan MA; Kamel K; Mohamad Sukri MZ; Norhisham SN; Ramly N; Robert F; Rosli NR; Suppiah J; Thayan R
EPI_ISL_6968520, EPI_ISL_6968856, EPI_ISL_6968867, EPI_ISL_6968889, EPI_ISL_6968898, EPI_ISL_6968908, EPI_ISL_6968926, EPI_ISL_6969277, EPI_ISL_6969282, EPI_ISL_6969299, EPI_ISL_6969308, EPI_ISL_6969323, EPI_ISL_6969334, EPI_ISL_6969416, EPI_ISL_6970377, EPI_ISL_6970407, EPI_ISL_6970439, EPI_ISL_6970445, EPI_ISL_7472892, EPI_ISL_7472893, EPI_ISL_7472894, EPI_ISL_7472901, EPI_ISL_7472902, EPI_ISL_7472903, EPI_ISL_7472904, EPI_ISL_7472905, EPI_ISL_7472906, EPI_ISL_7472907, EPI_ISL_7472908, EPI_ISL_7472909, EPI_ISL_7472910, EPI_ISL_7472911, EPI_ISL_7472912, EPI_ISL_7472913, EPI_ISL_7472914, EPI_ISL_7472915			
see above	Rejang Medical Centre (Sibu)	Institute of Health and Community Medicine	Chan Chia Jui; Chua Hock Hin; David Perera; Ooi Mong How; Tonnii Sia Loong Loong; Wong Jyn Shan
EPI_ISL_6773825	SWAT TEAM HPSF	UKM Medical Molecular Biology Institute (UMBI)	Mira Farzana binti Mohamad Mokhtar
EPI_ISL_7457712, EPI_ISL_7457717, EPI_ISL_7468580, EPI_ISL_7468583, EPI_ISL_7468621, EPI_ISL_7468628, EPI_ISL_7468633, EPI_ISL_8405421, EPI_ISL_8405425, EPI_ISL_8405426, EPI_ISL_8405427, EPI_ISL_8405428, EPI_ISL_8405489, EPI_ISL_8405628, EPI_ISL_8406364, EPI_ISL_8692653			
see above	Salak Health Clinic	Institute for Medical Research, Infectious Disease Research Centre, National Institutes of Health, Ministry of Health Malaysia	Ahmad FA; Ahmad Fazilah NA; Anasir MI; Azizan MA; Kamel K; Mohamad Sukri MZ; Mohd Zawawi Z; Norhisham SN; Ramly N; Robert F; Rosli NR; Suppiah J; Thayan R
EPI_ISL_6586589, EPI_ISL_6586590, EPI_ISL_6586600, EPI_ISL_6586601, EPI_ISL_6586602, EPI_ISL_6586603, EPI_ISL_6586698, EPI_ISL_6586699, EPI_ISL_6586700, EPI_ISL_6586701, EPI_ISL_6968300, EPI_ISL_6968314, EPI_ISL_6968323, EPI_ISL_6968338, EPI_ISL_6968353, EPI_ISL_6968367, EPI_ISL_6968431, EPI_ISL_7472929, EPI_ISL_7472941, EPI_ISL_7473085, EPI_ISL_7473086, EPI_ISL_7473087, EPI_ISL_7473088, EPI_ISL_7473089, EPI_ISL_7473090, EPI_ISL_7473091, EPI_ISL_7473092, EPI_ISL_7473093, EPI_ISL_7473094, EPI_ISL_7473095, EPI_ISL_7473096, EPI_ISL_7473097, EPI_ISL_7473098, EPI_ISL_7473099, EPI_ISL_7473100, EPI_ISL_7473101, EPI_ISL_7473102, EPI_ISL_7473103, EPI_ISL_7473104, EPI_ISL_7473105, EPI_ISL_7473106, EPI_ISL_7473107, EPI_ISL_7473108, EPI_ISL_7473109, EPI_ISL_7473110, EPI_ISL_7473111, EPI_ISL_7473112, EPI_ISL_7473113, EPI_ISL_7473114, EPI_ISL_7473115, EPI_ISL_7473116, EPI_ISL_7473117, EPI_ISL_7473118, EPI_ISL_7473119, EPI_ISL_7473120, EPI_ISL_7473121, EPI_ISL_8188737, EPI_ISL_8188738, EPI_ISL_8188832			
see above	Sarawak General Hospital (Kuching)	Institute of Health and Community Medicine	Chan Chia Jui; Chua Hock Hin; David Perera; Lim Woon Hook; Ooi Mong How; Tonnii Sia Loong Loong; Wong Jyn Shan
EPI_ISL_6773971, EPI_ISL_6773976, EPI_ISL_6773983, EPI_ISL_6773984, EPI_ISL_6773985, EPI_ISL_6773994, EPI_ISL_6773999, EPI_ISL_6774000, EPI_ISL_6774001, EPI_ISL_6774002, EPI_ISL_6774003, EPI_ISL_6774004, EPI_ISL_6774005, EPI_ISL_6774008, EPI_ISL_6774030, EPI_ISL_6774033, EPI_ISL_6774039, EPI_ISL_6774040, EPI_ISL_6774044, EPI_ISL_6774047, EPI_ISL_6915076, EPI_ISL_6949790, EPI_ISL_6949794, EPI_ISL_6949796, EPI_ISL_6949847, EPI_ISL_6949890, EPI_ISL_6950131, EPI_ISL_6950136, EPI_ISL_6950140, EPI_ISL_6950144, EPI_ISL_7381228, EPI_ISL_7456538, EPI_ISL_7456542, EPI_ISL_7456543, EPI_ISL_7457004, EPI_ISL_7457065, EPI_ISL_7457159, EPI_ISL_7457161, EPI_ISL_7457286, EPI_ISL_7457433, EPI_ISL_7457463, EPI_ISL_7457468, EPI_ISL_7457470, EPI_ISL_7457472, EPI_ISL_7457522, EPI_ISL_7457523, EPI_ISL_7457524, EPI_ISL_7457529, EPI_ISL_7457530, EPI_ISL_7457535, EPI_ISL_7457539, EPI_ISL_7457540, EPI_ISL_7457543, EPI_ISL_7457819			
see above	Selangor State Health Department	Institute for Medical Research, Infectious Disease Research Centre, National Institutes of Health, Ministry of Health Malaysia	Ahmad FA; Ahmad Fazilah NA; Anasir MI; Azizan MA; Kamel K; Mohamad Sukri MZ; Mohd Zawawi Z; Norhisham SN; Ramly N; Robert F; Rosli NR; Suppiah J; Thayan R
EPI_ISL_7468592, EPI_ISL_7468593, EPI_ISL_7889377, EPI_ISL_8405255, EPI_ISL_8405422, EPI_ISL_8405423, EPI_ISL_8405429, EPI_ISL_8405487, EPI_ISL_8405521, EPI_ISL_8405564, EPI_ISL_8405767, EPI_ISL_8406309, EPI_ISL_8406363, EPI_ISL_8406384, EPI_ISL_8692530, EPI_ISL_8692652, EPI_ISL_8692729			
see above	Sepang District Health Office	Institute for Medical Research, Infectious Disease Research Centre, National Institutes of Health, Ministry of Health Malaysia	Ahmad FA; Ahmad Fazilah NA; Anasir MI; Azizan MA; Kamel K; Mohamad Sukri MZ; Mohd Zawawi Z; Norhisham SN; Ramly N; Robert F; Rosli NR; Suppiah J; Thayan R
EPI_ISL_9211160, EPI_ISL_9211296, EPI_ISL_9211369	Sipitang District Health Office	Institute for Medical Research, Infectious Disease Research Centre, National Institutes of Health, Ministry of Health Malaysia	Ahmad FA; Ahmad Fazilah NA; Anasir MI; Azizan MA; Kamel K; Mohamad Sukri MZ; Norhisham SN; Ramly N; Robert F; Rosli NR; Suppiah J; Thayan R
EPI_ISL_6423030	Tenom District Health Office	Institute for Medical Research, Infectious Disease Research Centre, National Institutes of Health, Ministry of Health Malaysia	Ahmad FA; Ahmad Fazilah NA; Anasir MI; Azizan MA; Kamel K; Mohd Zawawi Z; Norhisham SN; Ramly N; Robert F; Suppiah J; Thayan R
EPI_ISL_6773822, EPI_ISL_6773834	UKKP HOSPITAL MELAKA	UKM Medical Molecular Biology Institute (UMBI)	Mira Farzana binti Mohamad Mokhtar
EPI_ISL_6773820, EPI_ISL_6773831,	UNIT KESIHATAN AWAM HEBHK	UKM Medical Molecular Biology Institute (UMBI)	Ab Mutalib; Abd Ghafar; Ishak; Jamal; Khairun Nur; Md Shahri; Mira Farzana; Mira Farzana binti Mohamad Mokhtar; Mohamad Mokhtar; Mohamed Rasheed; Mohd Yunos; Mohd Yusuf; Muhammad Azami; Muhiddin; Nor Azila; Nur Alyaa Afifah; Nurul

EPI_ISL_7649020, EPI_ISL_7649023			Syakima; Rahman; Ryia Illani; Siti Nur Hasanah; Zahirrah Begam
EPI_ISL_6705496	UNIT KPAS, PKD MELAKA TENGAH	UKM Medical Molecular Biology Institute (UMBI)	Mira Farzana binti Mohamad Mokhtar
EPI_ISL_6705593	Ward 8D, HTJS	UKM Medical Molecular Biology Institute (UMBI)	Mira Farzana binti Mohamad Mokhtar

We gratefully acknowledge the following Authors from the Originating laboratories responsible for obtaining the specimens, as well as the Submitting laboratories where the genome data were generated and shared via GISAID, on which this research is based.

All Submitters of data may be contacted directly via www.gisaid.org

Authors are sorted alphabetically.

Acknowledgement EPI_SET Identifier: EPI_SET_20220603pd

Accession ID	Originating Laboratory	Submitting Laboratory	Authors
EPI_ISL_9211853	Ampang Hospital	Institute for Medical Research, Infectious Disease Research Centre, National Institutes of Health, Ministry of Health Malaysia	Ahmad FA; Ahmad Fazilah NA; Anasir MI; Azizan MA; Kamel K; Mohamad Sukri MZ; Norhisham SN; Ramly N; Robert F; Rosli NR; Suppiah J; Thayan R
EPI_ISL_10657470	BP Healthcare Group @ Glenmarie	Institute for Medical Research, Infectious Disease Research Centre, National Institutes of Health, Ministry of Health Malaysia	Ahmad FA; Ahmad Fazilah NA; Anasir MI; Azizan MA; G. Adypatti NM; Jamaluddin MS; Kamel K; Mohamad Sukri MZ; Norhisham SN; Ramly N; Robert F; Rosli NR; Suppiah J; Thayan R
EPI_ISL_7472996, EPI_ISL_7472997, EPI_ISL_7472998, EPI_ISL_7472999, EPI_ISL_7473003, EPI_ISL_7473004, EPI_ISL_7473005, EPI_ISL_7473006, EPI_ISL_7473007, EPI_ISL_7473008, EPI_ISL_7473009, EPI_ISL_7473010, EPI_ISL_7473011, EPI_ISL_7473012, EPI_ISL_7473013, EPI_ISL_7473014, EPI_ISL_7473015, EPI_ISL_7473016, EPI_ISL_7473017, EPI_ISL_7473018, EPI_ISL_7473019, EPI_ISL_7473020, EPI_ISL_7473021			
see above	Bintulu Hospital PCR Lab, Bintulu	Institute of Health and Community Medicine	Chan Chia Jui; Chua Hock Hin; David Perera; Ooi Mong How; Tan Lee See; Tonnii Sia Loong Loong; Wong Jyn Shan
EPI_ISL_6969812, EPI_ISL_6969825, EPI_ISL_6970410, EPI_ISL_7472927, EPI_ISL_7473030, EPI_ISL_7473031, EPI_ISL_7473032, EPI_ISL_7473033, EPI_ISL_7473034, EPI_ISL_7473035, EPI_ISL_7473036, EPI_ISL_7473037, EPI_ISL_7473038, EPI_ISL_7473044, EPI_ISL_7473045, EPI_ISL_7473046, EPI_ISL_7473047, EPI_ISL_7473048, EPI_ISL_7473049, EPI_ISL_8782810, EPI_ISL_8782812, EPI_ISL_8782813, EPI_ISL_8782814, EPI_ISL_8782815, EPI_ISL_8782816, EPI_ISL_8782817, EPI_ISL_8782830, EPI_ISL_8782831, EPI_ISL_8782832, EPI_ISL_8782833, EPI_ISL_8782834			
see above	Bintulu Medical Centre (Bintulu)	Institute of Health and Community Medicine	Chan Chia Jui; Chua Hock Hin; David Perera; Ooi Mong How; Tonnii Sia Loong Loong; Wong Jyn Shan
EPI_ISL_6968810, EPI_ISL_6968817, EPI_ISL_6968837, EPI_ISL_6969538, EPI_ISL_6969550, EPI_ISL_6969564, EPI_ISL_6969570, EPI_ISL_6969587, EPI_ISL_6969595, EPI_ISL_6969608, EPI_ISL_6969614, EPI_ISL_6969623, EPI_ISL_6969630, EPI_ISL_6969646, EPI_ISL_6969657, EPI_ISL_6969669, EPI_ISL_7472922, EPI_ISL_7473022, EPI_ISL_7473023, EPI_ISL_7473024, EPI_ISL_7473025, EPI_ISL_7473026, EPI_ISL_7473027, EPI_ISL_7473028, EPI_ISL_7473029, EPI_ISL_8188805, EPI_ISL_8188806, EPI_ISL_8188818, EPI_ISL_8188855			
see above	Borneo Medical Centre (Kuching)	Institute of Health and Community Medicine	Chan Chia Jui; Chua Hock Hin; David Perera; Ooi Mong How; Tonnii Sia Loong Loong; Wong Jyn Shan
EPI_ISL_8254308, EPI_ISL_8254324, EPI_ISL_8254344, EPI_ISL_8254345, EPI_ISL_8254346	CPRC JKNT	Department of Medical Microbiology, Faculty of Medicine, University of Malaya; University of Malaya Medical Centre	I-Ching SAM; Jolene Yin Ling FU; Omar Khalilur Rahman; Yoke Fun Chan
EPI_ISL_8170464, EPI_ISL_8170532	CPRC JKNT	Tropical Infectious Diseases Research & Education Centre (TIDREC), Universiti Malaya	AsmaAnati CheMatSeri; Che-Norainon Yaacob; Jia-Yi Tan; Jo-Ern Wong; Kim-Kee Tan; Mulya-Mustika-Sari Zulkifli; Noor Syahida Azizan; Nur-Hidayana Mahfodz; Sazaly AbuBakar; Siti-Sarah Nor'e
EPI_ISL_8564937	Department of Medical Microbiology and Parasitology, Hospital Universiti Sains Malaysia	Molecular Research Laboratory	Abdul Haris bin Muhammad; Ahmad Adebayo Irekeola; Ahmad Sukari Bin Halim; Aswini Leela; Azian Harun; Chan Yean Yean; Chua Wei Chuan; Chua Wei Lian; Engku Nur Syafirah bt Engku Abd Rahman; Farahana binti Mohamed Alexander Chong Shu Chien; Kirnpal Kaur Banga Singh; Lau Nyok Sean; Lee Lih Huey; Lim Shu Yong; Maizun binti Mohd Zain; Muhammad Azamuddeen bin Mohammad Nasir; Muhammad Fazli bin Khalid; Muhammad Nashrul Farhan Samsudin; Muhammad Zarul Hanifah Bin Md Zoqratt; Naveed Ahmed; Nazmi Liana Binti Azmi; Noor Hafizan binti Mat Salleh; Nurfadhlina Musa; Qasim Ayub; Rosline Hassan; Sadequr Rahman; Syahida binti Omar; Wan Mohd Zahiruddin Wan Mohammad; Wardah Yusof; Wilhelm Eng Wei Han; Zaini bin Hussin; Zakuan Zainy Deris
EPI_ISL_8646692, EPI_ISL_8646693, EPI_ISL_8646694, EPI_ISL_8646695, EPI_ISL_8646696, EPI_ISL_8646697, EPI_ISL_8646698, EPI_ISL_8646699, EPI_ISL_8646700, EPI_ISL_8646701, EPI_ISL_8646706, EPI_ISL_8646711, EPI_ISL_8646712, EPI_ISL_8646716, EPI_ISL_8646717, EPI_ISL_8646721			
see above	Department of Medical Microbiology and Parasitology, University of Science Malaysia Hospital	Molecular Research Laboratory, Department of Medical Microbiology and Parasitology, University of Science Health Campus	Abdul Haris bin Muhammad; Ahmad Adebayo Irekeola; Ahmad Sukari Bin Halim; Alexander Chong Shu Chien; Aswini Leela; Azian Harun; Chan Yean Yean; Chua Wei Chuan; Chua Wei Lian; Engku Nur Syafirah bt Engku Abd Rahman; Farahana binti Mohamed; Kirnpal Kaur Banga Singh; Lau Nyok Sean; Lee Lih Huey; Lim Shu Yong; Maizun binti Mohd Zain; Muhammad Azamuddeen bin Mohammad Nasir; Muhammad Fazli bin Khalid; Muhammad Nashrul Farhan Samsudin; Muhammad Zarul Hanifah Bin Md Zoqratt; Naveed Ahmed; Nazmi Liana Binti Azmi; Noor Hafizan binti Mat Salleh; Nurfadhlina Musa; Qasim Ayub; Rosline Hassan; Sadequr Rahman; Syahida binti Omar; Wan Mohd Zahiruddin Wan Mohammad; Wardah Yusof; Wilhelm Eng Wei Han; Zaini bin Hussin; Zakuan Zainy Deris
EPI_ISL_10073048	Department of Medical Microbiology, Hospital Pengajar Universiti Putra Malaysia	Department of Medical Microbiology, Hospital Pengajar Universiti Putra Malaysia	Afiqah Adzmi; Amiza Azmi; Azmiza Syawani Jasni; Hui Yee Chee; Leslie Thian Lung Than; Muadz Mohtar; Muhammad Mohd Isa; Narcisse MS Joseph; Niazlin Mohd Taib; Noor Hazirah Noor Azhari; Norlaila; Nur Raihana Ithnin; Nurul Huda Mohamed Rashidi; Nurul Nadiah Ismail; Rosni Ibrahim; Rukman Awang Hamat; Sallehhudin; Siti Norbaya Masri; Siti Zulaikha Zakariah; Syafinaz Amin Nordin; Tengku Zetty Maztura Tengku Jamaluddin; Zamberi Sekawi
EPI_ISL_6567064, EPI_ISL_6567068	HOSPITAL CANSELOR TUANKU MUHRIZ (HCTM)	UKM Medical Molecular Biology Institute (UMBI)	Mira Farzana binti Mohamad Mokhtar
EPI_ISL_9000502, EPI_ISL_9000503, EPI_ISL_9000505, EPI_ISL_9000512	HOSPITAL PULAU PINANG	iPROMISE, UiTM	Ariza Adnan; Fadzilah Mohd Nor; Lim Wai Feng; Mohd Asif Mohd Sukri; Mohd Nur Fakhruzzaman Noorizhab; Mohd Zaki Salleh; Sazzli Shahlan Kassim; Siti Farah Alwani Mohd Nawi; Siti Hamimah Sheikh Abdul Kadir; Teh Lay Kek; Wang Seok Mui
EPI_ISL_8254333, EPI_ISL_8254334, EPI_ISL_8254335, EPI_ISL_8254338, EPI_ISL_8254339, EPI_ISL_8254340, EPI_ISL_8254342			
see above	HOSPITAL RAJA PERMAISURI BAINUN	Department of Medical Microbiology, Faculty of Medicine, University of Malaya; University of Malaya Medical Centre	I-Ching SAM; Jolene Yin Ling FU; Omar Khalilur Rahman; Yoke Fun Chan
EPI_ISL_7464476, EPI_ISL_7464483, EPI_ISL_7468513, EPI_ISL_7649008, EPI_ISL_7649028, EPI_ISL_7649031, EPI_ISL_7649057, EPI_ISL_7649072, EPI_ISL_7649087, EPI_ISL_8438481			
see above	HOSPITAL SULTANAH AMINAH	UKM Medical Molecular Biology Institute (UMBI)	Ab Mutalib; Abd Ghafar; Ishak; Jamal; Khairun Nur; Md Shahri; Mira Farzana; Mohamad Mokhtar; Mohamed Rasheed; Mohd Yunos; Mohd Yusuf; Muhammad Azami; Muhiddin; Nor Azi; Nor Azila; Nur Alyaa Afifah; Nurul Syakima; Rahman; Ryia Illani; Siti Nur Hasanah; Zahirrah Begam
EPI_ISL_7464477, EPI_ISL_7464481, EPI_ISL_7464484, EPI_ISL_8438479	HOSPITAL SULTANAH AMINAH, JB	UKM Medical Molecular Biology Institute (UMBI)	Ab Mutalib; Abd Ghafar; Ishak; Jamal; Khairun Nur; Md Shahri; Mira Farzana; Mohamad Mokhtar; Mohamed Rasheed; Mohd Yunos; Mohd Yusuf; Muhammad Azami; Muhiddin; Nor Azi; Nor Azila; Nur Alyaa Afifah; Nurul Syakima; Rahman; Ryia Illani; Siti Nur Hasanah; Zahirrah Begam
EPI_ISL_9000507	HPP ID department	iPROMISE, UiTM	Ariza Adnan; Fadzilah Mohd Nor; Lim Wai Feng; Mohd Asif Mohd Sukri; Mohd Nur Fakhruzzaman Noorizhab; Mohd Zaki Salleh; Sazzli Shahlan Kassim; Siti Farah Alwani Mohd Nawi; Siti Hamimah Sheikh Abdul Kadir; Teh Lay Kek; Wang Seok Mui
EPI_ISL_8170453, EPI_ISL_8170454, EPI_ISL_8170456, EPI_ISL_8170457, EPI_ISL_8170458, EPI_ISL_8170459, EPI_ISL_8170460, EPI_ISL_8170461, EPI_ISL_8170462, EPI_ISL_8170463, EPI_ISL_8170503			
see above	HRPB	Tropical Infectious Diseases Research & Education Centre (TIDREC), Universiti Malaya	AsmaAnati CheMatSeri; Che-Norainon Yaacob; Jia-Yi Tan; Jo-Ern Wong; Kim-Kee Tan; Mulya-Mustika-Sari Zulkifli; Noor Syahida Azizan; Nur-Hidayana Mahfodz; Sazaly AbuBakar; Siti-Sarah Nor'e
EPI_ISL_8254343	HRPZ II	Department of Medical Microbiology, Faculty of Medicine, University of Malaya; University of Malaya Medical Centre	I-Ching SAM; Jolene Yin Ling FU; Omar Khalilur Rahman; Yoke Fun Chan
EPI_ISL_7649059	HTJ SEREMBAN	UKM Medical Molecular Biology Institute (UMBI)	Ab Mutalib; Abd Ghafar; Ishak; Jamal; Khairun Nur; Md Shahri; Mira Farzana; Mohamad Mokhtar; Mohamed Rasheed; Mohd Yunos; Mohd Yusuf; Muhammad Azami; Muhiddin; Nor Azila; Nur Alyaa Afifah; Nurul Syakima; Rahman; Ryia Illani; Siti Nur Hasanah; Zahirrah Begam
EPI_ISL_7649067, EPI_ISL_7649069	HTJS	UKM Medical Molecular Biology Institute (UMBI)	Ab Mutalib; Abd Ghafar; Ishak; Jamal; Khairun Nur; Md Shahri; Mira Farzana; Mohamad Mokhtar; Mohamed Rasheed; Mohd Yunos; Mohd Yusuf; Muhammad Azami; Muhiddin; Nor Azila; Nur Alyaa Afifah; Nurul Syakima; Rahman; Ryia Illani; Siti Nur Hasanah; Zahirrah Begam
EPI_ISL_7464468, EPI_ISL_7464471, EPI_ISL_7464472, EPI_ISL_7464475, EPI_ISL_7464478, EPI_ISL_7464487, EPI_ISL_7464488, EPI_ISL_8438480			
see above	Hopsital Canselor Tuanku Muhriz (HCTM)	UKM Medical Molecular Biology Institute (UMBI)	Ab Mutalib; Abd Ghafar; Ishak; Jamal; Khairun Nur; Md Shahri; Mira Farzana; Mohamad Mokhtar; Mohamed Rasheed; Mohd Yunos; Mohd Yusuf; Muhammad Azami; Muhiddin; Nor Azi; Nor Azila; Nur Alyaa Afifah; Nurul Syakima; Rahman; Ryia Illani; Siti Nur Hasanah; Zahirrah Begam
EPI_ISL_6705491, EPI_ISL_6705517, EPI_ISL_6705529, EPI_ISL_6705577	Hospital Canselor Tuanku Muhriz (HCTM)	UKM Medical Molecular Biology Institute (UMBI)	Mira Farzana binti Mohamad Mokhtar
EPI_ISL_11900039, EPI_ISL_11900040	Hospital Kajang	Malaysia Genome and Vaccine Institute	Azrin Ahmad; Enizza Kasim; Irni Suhayu Sapian; Mohd Faizal Abu Bakar; Mohd Ghows Mohd Azzam.; Mohd Noor Mat Isa; Nor Azfa Johari; Nurhezreen Md Iqbal; Shamsidar Sopie; Siti Noraini Othman; Yusuf Muhammad Noor
EPI_ISL_8745632	Hospital Lahat Datu	Malaysia Genome and Vaccine Institute	Azrin Ahmad; Enizza Kasim; Irni Suhayu Sapian; Mohd Faizal Abu Bakar; Mohd Ghows Mohd Azzam.; Mohd Noor Mat Isa; Nor Azfa Johari; Nurhezreen Md Iqbal; Shamsidar Sopie; Siti Noraini Othman; Yusuf Muhammad Noor
EPI_ISL_7468516, EPI_ISL_7468519, EPI_ISL_7468520, EPI_ISL_7468528, EPI_ISL_7468534, EPI_ISL_7468535, EPI_ISL_7987104			
see above	Hospital Melaka	UKM Medical Molecular Biology Institute (UMBI)	Ab Mutalib; Abd Ghafar; Ishak; Jamal; Khairun Nur; Md Shahri; Mira Farzana; Mohamad Mokhtar; Mohamed Rasheed; Mohd Yunos; Mohd Yusuf; Muhammad Azami; Muhiddin; Nor Azila; Nur Alyaa Afifah; Nurul Syakima; Rahman; Ryia Illani; Siti Nur Hasanah; Zahirrah Begam
EPI_ISL_6964538, EPI_ISL_9000513	Hospital Pulau Pinang	iPROMISE, UiTM	Ariza Adnan; Fadzilah Mohd Nor; Lim Wai Feng; Mohd Asif Mohd Sukri; Mohd Nur Fakhruzzaman Noorizhab; Mohd Zaki Salleh; Sazzli Shahlan Kassim; Siti Farah Alwani Mohd Nawi; Siti Hamimah Sheikh Abdul Kadir; Teh Lay Kek; Wang Seok Mui
EPI_ISL_8745641, EPI_ISL_8745664	Hospital Queen Elizabeth	Malaysia Genome and Vaccine Institute	Azrin Ahmad; Enizza Kasim; Irni Suhayu Sapian; Mohd Faizal Abu Bakar; Mohd Ghows Mohd Azzam.; Mohd Noor Mat Isa; Nor Azfa Johari; Nurhezreen Md Iqbal; Shamsidar Sopie; Siti Noraini Othman; Yusuf Muhammad Noor
EPI_ISL_10969127	Hospital Sultanah Bahiyah	Tropical Infectious Diseases Research & Education Centre (TIDREC), Universiti Malaya	AsmaAnati CheMatSeri; Che-Norainon Yaacob; Jia-Yi Tan; Jo-Ern Wong; Kim-Kee Tan; Mulya-Mustika-Sari Zulkifli; Noor Syahida Azizan; Nur-Hidayana Mahfodz; Sazaly AbuBakar; Siti-Sarah Nor'e
EPI_ISL_6705484, EPI_ISL_11323772, EPI_ISL_11323782, EPI_ISL_11323789, EPI_ISL_11323790, EPI_ISL_11323799, EPI_ISL_11323808, EPI_ISL_11323818, EPI_ISL_11323824, EPI_ISL_11323829, EPI_ISL_11323830, EPI_ISL_11323831, EPI_ISL_11323832			
see above	Institut Biologi Molekul Perubatan UKM (UMBI)	UKM Medical Molecular Biology Institute (UMBI)	Khairun Nur Abd Ghafar; Mira Farzana Mohamad Mokhtar; Mira Farzana binti Mohamad Mokhtar; Muhiddin Ishak; Nor Azila Muhammad Azami; Nur Alyaa Afifah Md Shahri; Nurul Syakima Ab Mutalib; Rahman Jamal; Ryia Illani Mohd Yunos; Siti Nur Hasanah Mohd Yusuf; Zahirrah Begam Mohamed Rasheed
EPI_ISL_8745626, EPI_ISL_8745627	Institut Perubatan Forensik	Malaysia Genome and Vaccine Institute	Azrin Ahmad; Enizza Kasim; Irni Suhayu Sapian; Mohd Faizal Abu Bakar; Mohd Ghows Mohd Azzam.; Mohd Noor Mat Isa; Nor Azfa Johari; Nurhezreen Md Iqbal; Shamsidar Sopie; Siti Noraini Othman; Yusuf Muhammad Noor

	Negara		
EPI_ISL_8500647, EPI_ISL_8500694	JABATAN KESIHATAN NEGERI	iPROMISE, UiTM	Ariza Adnan; Fadzilah Mohd Nor; Lim Wai Feng; Mohd Asif Mohd Sukri; Mohd Nur Fakhruzzaman Noorizhab; Mohd Zaki Salleh; Sazzli Shahlan Kassim; Siti Farah Alwani Mohd Nawi; Siti Hamimah Sheikh Abdul Kadir; Teh Lay Kek; Wang Seok Mui
EPI_ISL_8170465, EPI_ISL_8170466, EPI_ISL_8170468	JABATAN PATOLOGI HRPZ II	Tropical Infectious Diseases Research & Education Centre (TIDREC), Universiti Malaya	AsmaAnati CheMatSeri; Che-Norainon Yaacob; Jia-Yi Tan; Jo-Ern Wong; Kim-Kee Tan; Mulya-Mustika-Sari Zulkifli; Noor Syahida Azizan; Nur-Hidayana Mahfodz; Sazaly AbuBakar; Siti-Sarah Nor'e
EPI_ISL_8500659	JKN PAHANG	iPROMISE, UiTM	Ariza Adnan; Fadzilah Mohd Nor; Lim Wai Feng; Mohd Asif Mohd Sukri; Mohd Nur Fakhruzzaman Noorizhab; Mohd Zaki Salleh; Sazzli Shahlan Kassim; Siti Farah Alwani Mohd Nawi; Siti Hamimah Sheikh Abdul Kadir; Teh Lay Kek; Wang Seok Mui
EPI_ISL_8500658	Jabatan Kesihatan Negeri	iPROMISE, UiTM	Ariza Adnan; Fadzilah Mohd Nor; Lim Wai Feng; Mohd Asif Mohd Sukri; Mohd Nur Fakhruzzaman Noorizhab; Mohd Zaki Salleh; Sazzli Shahlan Kassim; Siti Farah Alwani Mohd Nawi; Siti Hamimah Sheikh Abdul Kadir; Teh Lay Kek; Wang Seok Mui
EPI_ISL_8745640	Jabatan Kesihatan WP Labuan	Malaysia Genome and Vaccine Institute	Azrin Ahmad; Enizza Kasim; Irni Suhayu Sapian; Mohd Faizal Abu Bakar; Mohd Ghows Mohd Azzam.; Mohd Noor Mat Isa; Nor Azfa Johari; Nurhezreen Md Iqbal; Shamsidar Sopie; Siti Noraini Othman; Yusuf Muhammad Noor
EPI_ISL_8254316, EPI_ISL_8254317	KK KOK LANAS	Department of Medical Microbiology, Faculty of Medicine, University of Malaya; University of Malaya Medical Centre	I-Ching SAM; Jolene Yin Ling FU; Omar Khalilur Rahman; Yoke Fun Chan
EPI_ISL_8254309, EPI_ISL_8254310	KLINIK KESIHATAN KETEREH	Department of Medical Microbiology, Faculty of Medicine, University of Malaya; University of Malaya Medical Centre	I-Ching SAM; Jolene Yin Ling FU; Omar Khalilur Rahman; Yoke Fun Chan
EPI_ISL_8254318	KLINIK KESIHATAN PERINGAT	Department of Medical Microbiology, Faculty of Medicine, University of Malaya; University of Malaya Medical Centre	I-Ching SAM; Jolene Yin Ling FU; Omar Khalilur Rahman; Yoke Fun Chan
EPI_ISL_8170447	KLINIK KESIHATAN PEROL	Tropical Infectious Diseases Research & Education Centre (TIDREC), Universiti Malaya	AsmaAnati CheMatSeri; Che-Norainon Yaacob; Jia-Yi Tan; Jo-Ern Wong; Kim-Kee Tan; Mulya-Mustika-Sari Zulkifli; Noor Syahida Azizan; Nur-Hidayana Mahfodz; Sazaly AbuBakar; Siti-Sarah Nor'e
EPI_ISL_8745622	Klinik Kesihatan Putrajaya Presint 18	Malaysia Genome and Vaccine Institute	Azrin Ahmad; Enizza Kasim; Irni Suhayu Sapian; Mohd Faizal Abu Bakar; Mohd Ghows Mohd Azzam.; Mohd Noor Mat Isa; Nor Azfa Johari; Nurhezreen Md Iqbal; Shamsidar Sopie; Siti Noraini Othman; Yusuf Muhammad Noor
EPI_ISL_8254322	Klinik kesihatan perol	Department of Medical Microbiology, Faculty of Medicine, University of Malaya; University of Malaya Medical Centre	I-Ching SAM; Jolene Yin Ling FU; Omar Khalilur Rahman; Yoke Fun Chan
EPI_ISL_8170445, EPI_ISL_8170446	Klinik kesihatan perol	Tropical Infectious Diseases Research & Education Centre (TIDREC), Universiti Malaya	AsmaAnati CheMatSeri; Che-Norainon Yaacob; Jia-Yi Tan; Jo-Ern Wong; Kim-Kee Tan; Mulya-Mustika-Sari Zulkifli; Noor Syahida Azizan; Nur-Hidayana Mahfodz; Sazaly AbuBakar; Siti-Sarah Nor'e
EPI_ISL_8254320	Klinik kesihatan wakaf che yeh	Department of Medical Microbiology, Faculty of Medicine, University of Malaya; University of Malaya Medical Centre	I-Ching SAM; Jolene Yin Ling FU; Omar Khalilur Rahman; Yoke Fun Chan
EPI_ISL_9212030	Kuala Kubu Bharu Hospital	Institute for Medical Research, Infectious Disease Research Centre, National Institutes of Health, Ministry of Health Malaysia	Ahmad FA; Ahmad Fazilah NA; Anasir MI; Azizan MA; Kamel K; Mohamad Sukri MZ; Norhisham SN; Ramly N; Robert F; Rosli NR; Suppiah J; Thayan R
EPI_ISL_7468564, EPI_ISL_7468565, EPI_ISL_7468566, EPI_ISL_7468567, EPI_ISL_7468568, EPI_ISL_7468569, EPI_ISL_7468570, EPI_ISL_7468571, EPI_ISL_7468572, EPI_ISL_7468573, EPI_ISL_7468574, EPI_ISL_7468576, EPI_ISL_7468577, EPI_ISL_7468579, EPI_ISL_7468582, EPI_ISL_7468584, EPI_ISL_7468585, EPI_ISL_7468586, EPI_ISL_7468587, EPI_ISL_7468588, EPI_ISL_7468589, EPI_ISL_7468590, EPI_ISL_7468591, EPI_ISL_7468594, EPI_ISL_7468595, EPI_ISL_7468596, EPI_ISL_7468597, EPI_ISL_7468598, EPI_ISL_7468600, EPI_ISL_7468601, EPI_ISL_7468602, EPI_ISL_7468603, EPI_ISL_7468604, EPI_ISL_7468605, EPI_ISL_7468606, EPI_ISL_7468607, EPI_ISL_7468608, EPI_ISL_7468622, EPI_ISL_7468623, EPI_ISL_7468625, EPI_ISL_7468627, EPI_ISL_7468629, EPI_ISL_7468630, EPI_ISL_7468631, EPI_ISL_7468632, EPI_ISL_7468634, EPI_ISL_10189543			
see above	Kuala Lumpur International Airport (KLIA) Health Office	Institute for Medical Research, Infectious Disease Research Centre, National Institutes of Health, Ministry of Health Malaysia	Ahmad FA; Ahmad Fazilah NA; Anasir MI; Azizan MA; Kamel K; Mohamad Sukri MZ; Mohd Zawawi Z; Norhisham SN; Ramly N; Robert F; Rosli NR; Suppiah J; Thayan R
EPI_ISL_8254314, EPI_ISL_8254319, EPI_ISL_8254321, EPI_ISL_8254323	MKA KB	Department of Medical Microbiology, Faculty of Medicine, University of Malaya; University of Malaya Medical Centre	I-Ching SAM; Jolene Yin Ling FU; Omar Khalilur Rahman; Yoke Fun Chan
EPI_ISL_8188747, EPI_ISL_8188748, EPI_ISL_8188749, EPI_ISL_8188750, EPI_ISL_8188751	Miri Hospital Molecular Diagnostic Lab, Miri	Institute of Health and Community Medicine	Chan Chia Jui; Chua Hock Hin; David Perera; Noor Amnani binti Limin; Ooi Mong How; Tonnii Sia Loong Loong
EPI_ISL_7889466, EPI_ISL_7890057, EPI_ISL_7890058, EPI_ISL_7890059, EPI_ISL_7890216	National Public Health Laboratory (MKAK), Ministry of Health Malaysia	Institute for Medical Research, Infectious Disease Research Centre, National Institutes of Health, Ministry of Health Malaysia	Ahmad FA; Ahmad Fazilah NA; Anasir MI; Azizan MA; Kamel K; Mohamad Sukri MZ; Mohd Zawawi Z; Norhisham SN; Ramly N; Robert F; Rosli NR; Suppiah J; Thayan R
EPI_ISL_7649090	PEJABAT KESIHATAN DAERAH JOHOR BAHRU	UKM Medical Molecular Biology Institute (UMBI)	Ab Mutalib; Abd Ghafar; Ishak; Jamal; Khairun Nur; Md Shahri; Mira Farzana; Mohamad Mokhtar; Mohamed Rasheed; Mohd Yunos; Mohd Yusuf; Muhammad Azami; Muhiddin; Nor Azila; Nur Alyaa Afifah; Nurul Syakima; Rahman; Ryia Illani; Siti Nur Hasanah; Zahirrah Begam
EPI_ISL_8254313, EPI_ISL_8254315	PEJABAT KESIHATAN DAERAH KUALA KRAI	Department of Medical Microbiology, Faculty of Medicine, University of Malaya; University of Malaya Medical Centre	I-Ching SAM; Jolene Yin Ling FU; Omar Khalilur Rahman; Yoke Fun Chan
EPI_ISL_8170443, EPI_ISL_8170444	PEJABAT KESIHATAN DAERAH KUALA KRAI	Tropical Infectious Diseases Research & Education Centre (TIDREC), Universiti Malaya	AsmaAnati CheMatSeri; Che-Norainon Yaacob; Jia-Yi Tan; Jo-Ern Wong; Kim-Kee Tan; Mulya-Mustika-Sari Zulkifli; Noor Syahida Azizan; Nur-Hidayana Mahfodz; Sazaly AbuBakar; Siti-Sarah Nor'e
EPI_ISL_9000520, EPI_ISL_9000521, EPI_ISL_9000522, EPI_ISL_9578974, EPI_ISL_9578975, EPI_ISL_9578976, EPI_ISL_9578977, EPI_ISL_9578998			
see above	PEJABAT KESIHATAN DAERAH SEBERANG PERAI UTARA PULAU PINANG	iPROMISE, UiTM	Ariza Adnan; Fadzilah Mohd Nor; Lim Wai Feng; Mohd Asif Mohd Sukri; Mohd Nur Fakhruzzaman Noorizhab; Mohd Zaki Salleh; Sazzli Shahlan Kassim; Siti Farah Alwani Mohd Nawi; Siti Hamimah Sheikh Abdul Kadir; Teh Lay Kek; Wang Seok Mui
EPI_ISL_8500672, EPI_ISL_8500673, EPI_ISL_8500674, EPI_ISL_8500675, EPI_ISL_8500676, EPI_ISL_8500677, EPI_ISL_8500678, EPI_ISL_8500680, EPI_ISL_8500681, EPI_ISL_8500682, EPI_ISL_8500683, EPI_ISL_8500684, EPI_ISL_8500685, EPI_ISL_8500686, EPI_ISL_8500687, EPI_ISL_8500688, EPI_ISL_9579011			
see above	PEJABAT KESIHATAN DAERAH TIMUR LAUT	iPROMISE, UiTM	Ariza Adnan; Fadzilah Mohd Nor; Lim Wai Feng; Mohd Asif Mohd Sukri; Mohd Nur Fakhruzzaman Noorizhab; Mohd Zaki Salleh; Sazzli Shahlan Kassim; Siti Farah Alwani Mohd Nawi; Siti Hamimah Sheikh Abdul Kadir; Teh Lay Kek; Wang Seok Mui
EPI_ISL_9000491	PKD BARAT DAYA	iPROMISE, UiTM	Ariza Adnan; Fadzilah Mohd Nor; Lim Wai Feng; Mohd Asif Mohd Sukri; Mohd Nur Fakhruzzaman Noorizhab; Mohd Zaki Salleh; Sazzli Shahlan Kassim; Siti Farah Alwani Mohd Nawi; Siti Hamimah Sheikh Abdul Kadir; Teh Lay Kek; Wang Seok Mui
EPI_ISL_8170448, EPI_ISL_8170449, EPI_ISL_8170450	PKD Bachok	Tropical Infectious Diseases Research & Education Centre (TIDREC), Universiti Malaya	AsmaAnati CheMatSeri; Che-Norainon Yaacob; Jia-Yi Tan; Jo-Ern Wong; Kim-Kee Tan; Mulya-Mustika-Sari Zulkifli; Noor Syahida Azizan; Nur-Hidayana Mahfodz; Sazaly AbuBakar; Siti-Sarah Nor'e
EPI_ISL_8170481	PKD Hilir Perak	Tropical Infectious Diseases Research & Education Centre (TIDREC), Universiti Malaya	AsmaAnati CheMatSeri; Che-Norainon Yaacob; Jia-Yi Tan; Jo-Ern Wong; Kim-Kee Tan; Mulya-Mustika-Sari Zulkifli; Noor Syahida Azizan; Nur-Hidayana Mahfodz; Sazaly AbuBakar; Siti-Sarah Nor'e
EPI_ISL_7468529, EPI_ISL_7468532	PKD Jempol	UKM Medical Molecular Biology Institute (UMBI)	Ab Mutalib; Abd Ghafar; Ishak; Jamal; Khairun Nur; Md Shahri; Mira Farzana; Mohamad Mokhtar; Mohamed Rasheed; Mohd Yunos; Mohd Yusuf; Muhammad Azami; Muhiddin; Nor Azila; Nur Alyaa Afifah; Nurul Syakima; Rahman; Ryia Illani; Siti Nur Hasanah; Zahirrah Begam
EPI_ISL_8170533	PKD Kerian	Tropical Infectious Diseases Research & Education Centre (TIDREC), Universiti Malaya	AsmaAnati CheMatSeri; Che-Norainon Yaacob; Jia-Yi Tan; Jo-Ern Wong; Kim-Kee Tan; Mulya-Mustika-Sari Zulkifli; Noor Syahida Azizan; Nur-Hidayana Mahfodz; Sazaly AbuBakar; Siti-Sarah Nor'e
EPI_ISL_7468515	PKD MELAKA TENGAH	UKM Medical Molecular Biology Institute (UMBI)	Ab Mutalib; Abd Ghafar; Ishak; Jamal; Khairun Nur; Md Shahri; Mira Farzana; Mohamad Mokhtar; Mohamed Rasheed; Mohd Yunos; Mohd Yusuf; Muhammad Azami; Muhiddin; Nor Azila; Nur Alyaa Afifah; Nurul Syakima; Rahman; Ryia Illani; Siti Nur Hasanah; Zahirrah Begam
EPI_ISL_8170525, EPI_ISL_8170529	PKD MUALLIM	Tropical Infectious Diseases Research & Education Centre (TIDREC), Universiti Malaya	AsmaAnati CheMatSeri; Che-Norainon Yaacob; Jia-Yi Tan; Jo-Ern Wong; Kim-Kee Tan; Mulya-Mustika-Sari Zulkifli; Noor Syahida Azizan; Nur-Hidayana Mahfodz; Sazaly AbuBakar; Siti-Sarah Nor'e
EPI_ISL_8170506, EPI_ISL_8170507	PKD Manjung	Tropical Infectious Diseases Research & Education Centre (TIDREC), Universiti Malaya	AsmaAnati CheMatSeri; Che-Norainon Yaacob; Jia-Yi Tan; Jo-Ern Wong; Kim-Kee Tan; Mulya-Mustika-Sari Zulkifli; Noor Syahida Azizan; Nur-Hidayana Mahfodz; Sazaly AbuBakar; Siti-Sarah Nor'e
EPI_ISL_8083705, EPI_ISL_8083706	PKD Maran	iPROMISE, UiTM	Ariza Adnan; Fadzilah Mohd Nor; Lim Wai Feng; Mohd Asif Mohd Sukri; Mohd Nur Fakhruzzaman Noorizhab; Mohd Zaki Salleh; Sazzli Shahlan Kassim; Siti Farah Alwani Mohd Nawi; Siti Hamimah Sheikh Abdul Kadir; Teh Lay Kek; Wang Seok Mui
EPI_ISL_8254311, EPI_ISL_8254312	PKD PASIR PUTEH	Department of Medical Microbiology, Faculty of Medicine, University of Malaya; University of Malaya Medical Centre	I-Ching SAM; Jolene Yin Ling FU; Omar Khalilur Rahman; Yoke Fun Chan
EPI_ISL_8500648	PKD SEBERANG PERAI SELATAN	iPROMISE, UiTM	Ariza Adnan; Fadzilah Mohd Nor; Lim Wai Feng; Mohd Asif Mohd Sukri; Mohd Nur Fakhruzzaman Noorizhab; Mohd Zaki Salleh; Sazzli Shahlan Kassim; Siti Farah Alwani Mohd Nawi; Siti Hamimah Sheikh Abdul Kadir; Teh Lay Kek; Wang Seok Mui
EPI_ISL_8083709, EPI_ISL_8083710, EPI_ISL_8083711, EPI_ISL_8083712, EPI_ISL_8500661, EPI_ISL_8500662, EPI_ISL_8500663, EPI_ISL_8500664, EPI_ISL_8500665, EPI_ISL_8500666, EPI_ISL_8500667, EPI_ISL_8500668, EPI_ISL_8500669, EPI_ISL_8500670, EPI_ISL_8500679, EPI_ISL_8500695, EPI_ISL_9000501, EPI_ISL_9000523, EPI_ISL_9000530			

see above	PKD SPT	iPROMISE, UiTM	Ariza Adnan; Fadzilah Mohd Nor; Lim Wai Feng; Mohd Asif Mohd Sukri; Mohd Nur Fakhruzzaman Noorizhab; Mohd Zaki Salleh; Sazzli Shahlan Kassim; Siti Farah Alwani Mohd Nawi; Siti Hamimah Sheikh Abdul Kadir; Teh Lay Kek; Wang Seok Mui
EPI_ISL_7987091, EPI_ISL_7987096	PKD Segamat	UKM Medical Molecular Biology Institute (UMBI)	Ab Mutalib; Abd Ghafar; Ishak; Jamal; Khairun Nur; Md Shahri; Mira Farzana; Mohamad Mokhtar; Mohamed Rasheed; Mohd Yunos; Mohd Yusuf; Muhammad Azami; Muhiddin; Nor Azila; Nur Alyaa Afifah; Nurul Syakima; Rahman; Ryia Illani; Siti Nur Hasanah; Zahirrah Begam
EPI_ISL_6567062, EPI_ISL_6567082, EPI_ISL_6567084, EPI_ISL_7464469, EPI_ISL_7464470, EPI_ISL_7464473, EPI_ISL_7464474, EPI_ISL_7464485, EPI_ISL_7464486, EPI_ISL_7468514, EPI_ISL_7468517, EPI_ISL_7468522, EPI_ISL_7468531, EPI_ISL_7649011, EPI_ISL_7649025, EPI_ISL_7649046, EPI_ISL_7649048, EPI_ISL_7649051, EPI_ISL_7649054, EPI_ISL_7649062, EPI_ISL_7649064, EPI_ISL_7649080, EPI_ISL_7649085			
see above	PKD TAMPIN	UKM Medical Molecular Biology Institute (UMBI)	Ab Mutalib; Abd Ghafar; Ishak; Jamal; Khairun Nur; Md Shahri; Mira Farzana; Mira Farzana binti Mohamad Mokhtar; Mohamad Mokhtar; Mohamed Rasheed; Mohd Yunos; Mohd Yusuf; Muhammad Azami; Muhiddin; Nor Azi; Nor Azila; Nur Alyaa Afifah; Nurul Syakima; Rahman; Ryia Illani; Siti Nur Hasanah; Zahirrah Begam
EPI_ISL_8314863	PKD TANGKAK	UKM Medical Molecular Biology Institute (UMBI)	Khairun Nur Abd Ghafar; Mira Farzana Mohamad Mokhtar; Muhiddin Ishak; Nor Azila Muhammad Azami; Nur Alyaa Afifah Md Shahri; Nurul Syakima Ab Mutalib; Rahman Jamal; Ryia Illani Mohd Yunos; Siti Nur Hasanah Mohd Yusuf; Zahirrah Begam Mohamed Rasheed
EPI_ISL_6705584, EPI_ISL_7987093, EPI_ISL_7987105	PKD Tampin	UKM Medical Molecular Biology Institute (UMBI)	Ab Mutalib; Abd Ghafar; Ishak; Jamal; Khairun Nur; Md Shahri; Mira Farzana; Mira Farzana binti Mohamad Mokhtar; Mohamad Mokhtar; Mohamed Rasheed; Mohd Yunos; Mohd Yusuf; Muhammad Azami; Muhiddin; Nor Azila; Nur Alyaa Afifah; Nurul Syakima; Rahman; Ryia Illani; Siti Nur Hasanah; Zahirrah Begam
EPI_ISL_8170451, EPI_ISL_8170452	PKD Tanah Merah	Tropical Infectious Diseases Research & Education Centre (TIDREC), Universiti Malaya	AsmaAnati CheMatSeri; Che-Norainon Yaacob; Jia-Yi Tan; Jo-Ern Wong; Kim-Kee Tan; Mulya-Mustika-Sari Zulkifli; Noor Syahida Azizan; Nur-Hidayana Mahfodz; Sazaly AbuBakar; Siti-Sarah Nor'e
EPI_ISL_8500671	PKDSPT	iPROMISE, UiTM	Ariza Adnan; Fadzilah Mohd Nor; Lim Wai Feng; Mohd Asif Mohd Sukri; Mohd Nur Fakhruzzaman Noorizhab; Mohd Zaki Salleh; Sazzli Shahlan Kassim; Siti Farah Alwani Mohd Nawi; Siti Hamimah Sheikh Abdul Kadir; Teh Lay Kek; Wang Seok Mui
EPI_ISL_8745633, EPI_ISL_8745634, EPI_ISL_8745636, EPI_ISL_8745637, EPI_ISL_8745638	Pejabat Kesihatan Daerah Kuala Penyu	Malaysia Genome and Vaccine Institute	Azrin Ahmad; Enizza Kasim; Irni Suhayu Sapian; Mohd Faizal Abu Bakar; Mohd Ghows Mohd Azzam.; Mohd Noor Mat Isa; Nor Azfa Johari; Nurhezreen Md Iqbal; Shamsidar Sopie; Siti Noraini Othman; Yusuf Muhammad Noor
EPI_ISL_8170510	Pejabat Kesihatan Daerah Pasir Mas	Tropical Infectious Diseases Research & Education Centre (TIDREC), Universiti Malaya	AsmaAnati CheMatSeri; Che-Norainon Yaacob; Jia-Yi Tan; Jo-Ern Wong; Kim-Kee Tan; Mulya-Mustika-Sari Zulkifli; Noor Syahida Azizan; Nur-Hidayana Mahfodz; Sazaly AbuBakar; Siti-Sarah Nor'e
EPI_ISL_9000532, EPI_ISL_9000533, EPI_ISL_9000535, EPI_ISL_9578956, EPI_ISL_9578957, EPI_ISL_9578958, EPI_ISL_9578959, EPI_ISL_9578960, EPI_ISL_9578961, EPI_ISL_9578962, EPI_ISL_9578963, EPI_ISL_9578964, EPI_ISL_9578965, EPI_ISL_9578966, EPI_ISL_9578967, EPI_ISL_9578968, EPI_ISL_9579184			
see above	Pejabat Kesihatan Daerah Timur Laut	iPROMISE, UiTM	Ariza Adnan; Fadzilah Mohd Nor; Lim Wai Feng; Mohd Asif Mohd Sukri; Mohd Nur Fakhruzzaman Noorizhab; Mohd Zaki Salleh; Sazzli Shahlan Kassim; Siti Farah Alwani Mohd Nawi; Siti Hamimah Sheikh Abdul Kadir; Teh Lay Kek; Wang Seok Mui
EPI_ISL_8745655, EPI_ISL_8745656, EPI_ISL_8745657	Pejabat Kesihatan Kawasan Kinabatangan	Malaysia Genome and Vaccine Institute	Azrin Ahmad; Enizza Kasim; Irni Suhayu Sapian; Mohd Faizal Abu Bakar; Mohd Ghows Mohd Azzam.; Mohd Noor Mat Isa; Nor Azfa Johari; Nurhezreen Md Iqbal; Shamsidar Sopie; Siti Noraini Othman; Yusuf Muhammad Noor
EPI_ISL_8745623	Pejabat Kesihatan Kawasan Kota Kinabalu	Malaysia Genome and Vaccine Institute	Azrin Ahmad; Enizza Kasim; Irni Suhayu Sapian; Mohd Faizal Abu Bakar; Mohd Ghows Mohd Azzam.; Mohd Noor Mat Isa; Nor Azfa Johari; Nurhezreen Md Iqbal; Shamsidar Sopie; Siti Noraini Othman; Yusuf Muhammad Noor
EPI_ISL_6969345, EPI_ISL_6969356, EPI_ISL_6969366, EPI_ISL_6969382, EPI_ISL_6969396, EPI_ISL_6969407, EPI_ISL_6970382, EPI_ISL_6970383, EPI_ISL_6970447, EPI_ISL_7473039, EPI_ISL_7473040			
see above	Rejang Medical Centre (Sibu)	Institute of Health and Community Medicine	Chan Chia Jui; Chua Hock Hin; David Perera; Ooi Mong How; Tonnii Sia Loong Loong; Wong Jyn Shan
EPI_ISL_11050302	SULTAN AHMAD SHAH MEDICAL CENTRE @IIUM	Microbiology Lab, SULTAN AHMAD SHAH MEDICAL CENTRE @IIUM	Darren Dean Tay; Hajar Fauzan Ahmad; Ummu Afeera Zainulabid
EPI_ISL_6968058, EPI_ISL_6968378, EPI_ISL_6968386, EPI_ISL_6968398, EPI_ISL_6968407, EPI_ISL_6968418, EPI_ISL_6968448, EPI_ISL_6968467, EPI_ISL_6968474, EPI_ISL_6968487, EPI_ISL_6970430, EPI_ISL_6970442, EPI_ISL_7472928, EPI_ISL_7472930, EPI_ISL_7472931, EPI_ISL_7472932, EPI_ISL_7472933, EPI_ISL_7472934, EPI_ISL_7472936, EPI_ISL_7472937, EPI_ISL_7472938, EPI_ISL_7472939, EPI_ISL_7472940, EPI_ISL_7472942, EPI_ISL_7472943, EPI_ISL_7472944, EPI_ISL_7472945, EPI_ISL_7472946, EPI_ISL_7472947, EPI_ISL_7472948, EPI_ISL_7472949, EPI_ISL_7472964, EPI_ISL_7472965, EPI_ISL_7472966, EPI_ISL_7472967, EPI_ISL_7472968, EPI_ISL_7472969, EPI_ISL_7472971, EPI_ISL_7472972, EPI_ISL_7472973, EPI_ISL_7472974, EPI_ISL_8188739, EPI_ISL_8188740, EPI_ISL_8188741			
see above	Sarawak General Hospital (Kuching)	Institute of Health and Community Medicine	Chan Chia Jui; Chua Hock Hin; David Perera; Ooi Mong How; Tonnii Sia Loong Loong; Wong Jyn Shan
EPI_ISL_6914937, EPI_ISL_7015235	Selangor State Health Department	Institute for Medical Research, Infectious Disease Research Centre, National Institutes of Health, Ministry of Health Malaysia	Ahmad FA; Ahmad Fazilah NA; Anasir MI; Azizan MA; Kamel K; Mohamad Sukri MZ; Mohd Zawawi Z; Norhisham SN; Ramly N; Robert F; Rosli NR; Suppiah J; Thayan R
EPI_ISL_9212243	State Health Department Federal Territory of Labuan	Institute for Medical Research, Infectious Disease Research Centre, National Institutes of Health, Ministry of Health Malaysia	Ahmad FA; Ahmad Fazilah NA; Anasir MI; Azizan MA; Kamel K; Mohamad Sukri MZ; Norhisham SN; Ramly N; Robert F; Rosli NR; Suppiah J; Thayan R
EPI_ISL_9211481, EPI_ISL_9211628	Tengku Ampuan Jemaah Hospital	Institute for Medical Research, Infectious Disease Research Centre, National Institutes of Health, Ministry of Health Malaysia	Ahmad FA; Ahmad Fazilah NA; Anasir MI; Azizan MA; Kamel K; Mohamad Sukri MZ; Norhisham SN; Ramly N; Robert F; Rosli NR; Suppiah J; Thayan R
EPI_ISL_8254348, EPI_ISL_8254350, EPI_ISL_8254351, EPI_ISL_8254352, EPI_ISL_8254353, EPI_ISL_8254354	UMMC	Department of Medical Microbiology, Faculty of Medicine, University of Malaya; University of Malaya Medical Centre	I-Ching SAM; Jolene Yin Ling FU; Omar Khalilur Rahman; Yoke Fun Chan
EPI_ISL_7831400	UNIT KESIHATAN AWAM HEBHK	UKM Medical Molecular Biology Institute (UMBI)	Ab Mutalib; Abd Ghafar; Ishak; Jamal; Khairun Nur; Md Shahri; Mira Farzana; Mohamad Mokhtar; Mohamed Rasheed; Mohd Yunos; Mohd Yusuf; Muhammad Azami; Muhiddin; Nor Azila; Nur Alyaa Afifah; Nurul Syakima; Rahman; Ryia Illani; Siti Nur Hasanah; Zahirrah Begam
EPI_ISL_9000509	Unit Perubatan Forensik, Hospital Seberang Jaya	iPROMISE, UiTM	Ariza Adnan; Fadzilah Mohd Nor; Lim Wai Feng; Mohd Asif Mohd Sukri; Mohd Nur Fakhruzzaman Noorizhab; Mohd Zaki Salleh; Sazzli Shahlan Kassim; Siti Farah Alwani Mohd Nawi; Siti Hamimah Sheikh Abdul Kadir; Teh Lay Kek; Wang Seok Mui
EPI_ISL_7987106	Unit kesihatan awam	UKM Medical Molecular Biology Institute (UMBI)	Ab Mutalib; Abd Ghafar; Ishak; Jamal; Khairun Nur; Md Shahri; Mira Farzana; Mohamad Mokhtar; Mohamed Rasheed; Mohd Yunos; Mohd Yusuf; Muhammad Azami; Muhiddin; Nor Azila; Nur Alyaa Afifah; Nurul Syakima; Rahman; Ryia Illani; Siti Nur Hasanah; Zahirrah Begam
EPI_ISL_7987102	Unit kesihatan awam HEBHK	UKM Medical Molecular Biology Institute (UMBI)	Ab Mutalib; Abd Ghafar; Ishak; Jamal; Khairun Nur; Md Shahri; Mira Farzana; Mohamad Mokhtar; Mohamed Rasheed; Mohd Yunos; Mohd Yusuf; Muhammad Azami; Muhiddin; Nor Azila; Nur Alyaa Afifah; Nurul Syakima; Rahman; Ryia Illani; Siti Nur Hasanah; Zahirrah Begam
EPI_ISL_7987097	WARD 3A	UKM Medical Molecular Biology Institute (UMBI)	Ab Mutalib; Abd Ghafar; Ishak; Jamal; Khairun Nur; Md Shahri; Mira Farzana; Mohamad Mokhtar; Mohamed Rasheed; Mohd Yunos; Mohd Yusuf; Muhammad Azami; Muhiddin; Nor Azila; Nur Alyaa Afifah; Nurul Syakima; Rahman; Ryia Illani; Siti Nur Hasanah; Zahirrah Begam
EPI_ISL_8170505, EPI_ISL_8170508, EPI_ISL_9829877, EPI_ISL_9829879, EPI_ISL_9829880, EPI_ISL_9829881, EPI_ISL_10969096, EPI_ISL_10969097, EPI_ISL_10969098, EPI_ISL_11010496			
see above	pkd manjung	Tropical Infectious Diseases Research & Education Centre (TIDREC), Universiti Malaya	AsmaAnati CheMatSeri; Che-Norainon Yaacob; Jia-Yi Tan; Jo-Ern Wong; Kim-Kee Tan; Mulya-Mustika-Sari Zulkifli; Noor Syahida Azizan; Nur-Hidayana Mahfodz; Sazaly AbuBakar; Siti-Sarah Nor'e

We gratefully acknowledge the following Authors from the Originating laboratories responsible for obtaining the specimens, as well as the Submitting laboratories where the genome data were generated and shared via GISAID, on which this research is based.

All Submitters of data may be contacted directly via www.gisaid.org

Authors are sorted alphabetically.

Accession ID	Originating Laboratory	Submitting Laboratory	Authors
EPI_ISL_10657468, EPI_ISL_10657469	BP Healthcare Group @ Glenmarie	Institute for Medical Research, Infectious Disease Research Centre, National Institutes of Health, Ministry of Health Malaysia	Ahmad FA; Ahmad Fazilah NA; Anasir MI; Azizan MA; G. Adypatti NM; Jamaluddin MS; Kamel K; Mohamad Sukri MZ; Norhisham SN; Ramly N; Robert F; Rosli NR; Suppiah J; Thayan R
EPI_ISL_8188790, EPI_ISL_8188791, EPI_ISL_8188792, EPI_ISL_8188793, EPI_ISL_8188794, EPI_ISL_8188795, EPI_ISL_8188796, EPI_ISL_8188797, EPI_ISL_8188798, EPI_ISL_8188799, EPI_ISL_8188800, EPI_ISL_8188801, EPI_ISL_8188802, EPI_ISL_8188803, EPI_ISL_8188804, EPI_ISL_8650107, EPI_ISL_8650126, EPI_ISL_8650127, EPI_ISL_8650128, EPI_ISL_8650152			
see above	Bintulu Hospital PCR Lab, Bintulu	Institute of Health and Community Medicine	Chan Chia Jui; Chua Hock Hin; David Perera; Ooi Mong How; Tan Lee See; Tonnii Sia Loong Loong; Wong Jyn Shan
EPI_ISL_8188819, EPI_ISL_8188820, EPI_ISL_8188821, EPI_ISL_8188822, EPI_ISL_8188823, EPI_ISL_8188824, EPI_ISL_8188825, EPI_ISL_8188836, EPI_ISL_8188837, EPI_ISL_8188838, EPI_ISL_8188839, EPI_ISL_8188840, EPI_ISL_8188841, EPI_ISL_8188842, EPI_ISL_8188873, EPI_ISL_8782818, EPI_ISL_8782819			
see above	Bintulu Medical Centre (Bintulu)	Institute of Health and Community Medicine	Chan Chia Jui; Chua Hock Hin; David Perera; Ooi Mong How; Tonnii Sia Loong Loong; Wong Jyn Shan
EPI_ISL_8188807, EPI_ISL_8188808, EPI_ISL_8188809, EPI_ISL_8188810, EPI_ISL_8188811, EPI_ISL_8188812, EPI_ISL_8188813, EPI_ISL_8188814, EPI_ISL_8188815, EPI_ISL_8188816, EPI_ISL_8188817, EPI_ISL_8188856, EPI_ISL_8782820, EPI_ISL_8782821, EPI_ISL_8782822, EPI_ISL_8782829			
see above	Borneo Medical Centre (Kuching)	Institute of Health and Community Medicine	Chan Chia Jui; Chua Hock Hin; David Perera; Ooi Mong How; Tonnii Sia Loong Loong; Wong Jyn Shan
EPI_ISL_7472950	Borneo Medical Centre (Miri)	Institute of Health and Community Medicine	Chan Chia Jui; Chua Hock Hin; David Perera; Ooi Mong How; Tonnii Sia Loong Loong; Wong Jyn Shan
EPI_ISL_10969147	CPRC JKNT	Tropical Infectious Diseases Research & Education Centre (TIDREC), Universiti Malaya	AsmaAnati CheMatSeri; Che-Norainon Yaacob; Jia-Yi Tan; Jo-Ern Wong; Kim-Kee Tan; Mulya-Mustika-Sari Zulkifli; Noor Syahida Azizan; Nur-Hidayana Mahfodz; Sazaly AbuBakar; Siti-Sarah Nor'e
EPI_ISL_8564938, EPI_ISL_8564939, EPI_ISL_10431136, EPI_ISL_10431143, EPI_ISL_10431144	Department of Medical Microbiology and Parasitology, Hospital Universiti Sains Malaysia	Molecular Research Laboratory	Abdul Haris bin Muhammad; Ahmad Adebayo Irekeola; Ahmad Sukari Bin Halim; Alexander Chong Shu Chien; Aswini Leela; Azian Harun; Chan Yean Yean; Chua Wei Chuan; Chua Wei Lian; Engku Nur Syafirah bt Engku Abd Rahman; Farahana binti Mohamed; Farahana binti Mohamed Alexander Chong Shu Chien; Kirnpal Kaur Banga Singh; Lau Nyok Sean; Lee Lih Huey; Lim Shu Yong; Maizun binti Mohd Zain; Muhammad Azamuddeen bin Mohammad Nasir; Muhammad Fazli bin Khalid; Muhammad Nashrul Farhan Samsudin; Muhammad Zarul Hanifah Bin Md Zoqratt; Naveed Ahmed; Nazmi Liana Binti Azmi; Noor Hafizan binti Mat Salleh; Nurfadhlina Musa; Qasim Ayub; Rosline Hassan; Sadequr Rahman; Syahida binti Omar; Wan Mohd Zahiruddin Wan Mohammad; Wardah Yusof; Wilhelm Eng Wei Han; Zaini bin Hussin; Zakuan Zainy Deris
EPI_ISL_8646690, EPI_ISL_8646691, EPI_ISL_8646707, EPI_ISL_8646718, EPI_ISL_8646719, EPI_ISL_8646722	Department of Medical Microbiology and Parasitology, University of Science Malaysia Hospital	Molecular Research Laboratory, Department of Medical Microbiology and Parasitology, University of Science Health Campus	Abdul Haris bin Muhammad; Ahmad Adebayo Irekeola; Ahmad Sukari Bin Halim; Alexander Chong Shu Chien; Aswini Leela; Azian Harun; Chan Yean Yean; Chua Wei Chuan; Chua Wei Lian; Engku Nur Syafirah bt Engku Abd Rahman; Farahana binti Mohamed; Kirnpal Kaur Banga Singh; Lau Nyok Sean; Lee Lih Huey; Lim Shu Yong; Maizun binti Mohd Zain; Muhammad Azamuddeen bin Mohammad Nasir; Muhammad Fazli bin Khalid; Muhammad Nashrul Farhan Samsudin; Muhammad Zarul Hanifah Bin Md Zoqratt; Naveed Ahmed; Nazmi Liana Binti Azmi; Noor Hafizan binti Mat Salleh; Nurfadhlina Musa; Qasim Ayub; Rosline Hassan; Sadequr Rahman; Syahida binti Omar; Wan Mohd Zahiruddin Wan Mohammad; Wardah Yusof; Wilhelm Eng Wei Han; Zaini bin Hussin; Zakuan Zainy Deris
EPI_ISL_8438517	E2 Covid ward	UKM Medical Molecular Biology Institute (UMBI)	Khairun Nur Abd Ghafar; Mira Farzana Mohamad Mokhtar; Muhiddin Ishak; Nor Azila Muhammad Azami; Nur Alyaa Afifah Md Shahri; Nurul Syakima Ab Mutalib; Rahman Jamal; Ryia Illani Mohd Yunos; Siti Nur Hasanah Mohd Yusuf; Zahirrah Begam Mohamed Rasheed
EPI_ISL_8188754, EPI_ISL_8188755, EPI_ISL_8188756, EPI_ISL_8188757, EPI_ISL_8188758, EPI_ISL_8188759, EPI_ISL_8188760, EPI_ISL_8188761, EPI_ISL_8188762, EPI_ISL_8188763, EPI_ISL_8188764, EPI_ISL_8188765, EPI_ISL_8188766, EPI_ISL_8188770, EPI_ISL_8188776, EPI_ISL_8188777, EPI_ISL_8188778, EPI_ISL_8188779, EPI_ISL_8188780, EPI_ISL_8188781, EPI_ISL_8188782, EPI_ISL_8188783, EPI_ISL_8188784, EPI_ISL_8188785, EPI_ISL_8188786, EPI_ISL_8188787, EPI_ISL_8188788, EPI_ISL_8188789, EPI_ISL_8188833			
see above	Gribbles Pathology, Kuching	Institute of Health and Community Medicine	Chan Chia Jui; Chua Hock Hin; David Perera; Josephine Liew Chin Chin; Ooi Mong How; Tonnii Sia Loong Loong; Wong Jyn Shan
EPI_ISL_8314879	HOSPITAL MELAKA	UKM Medical Molecular Biology Institute (UMBI)	Khairun Nur Abd Ghafar; Mira Farzana Mohamad Mokhtar; Muhiddin Ishak; Nor Azila Muhammad Azami; Nur Alyaa Afifah Md Shahri; Nurul Syakima Ab Mutalib; Rahman Jamal; Ryia Illani Mohd Yunos; Siti Nur Hasanah Mohd Yusuf; Zahirrah Begam Mohamed Rasheed
EPI_ISL_9000495, EPI_ISL_9000496, EPI_ISL_9000497, EPI_ISL_9000500, EPI_ISL_9579001, EPI_ISL_9579002, EPI_ISL_9579004			
see above	HOSPITAL PULAU PINANG	iPROMISE, UiTM	Ariza Adnan; Fadzilah Mohd Nor; Lim Wai Feng; Mohd Asif Mohd Sukri; Mohd Nur Fakhruzzaman Noorizhab; Mohd Zaki Salleh; Sazzli Shahlan Kassim; Siti Farah Alwani Mohd Nawi; Siti Hamimah Sheikh Abdul Kadir; Teh Lay Kek; Wang Seok Mui
EPI_ISL_8380527, EPI_ISL_8380548	HOSPITAL SEGAMAT	UKM Medical Molecular Biology Institute (UMBI)	Khairun Nur Abd Ghafar; Mira Farzana Mohamad Mokhtar; Muhiddin Ishak; Nor Azila Muhammad Azami; Nur Alyaa Afifah Md Shahri; Nurul Syakima Ab Mutalib; Rahman Jamal; Ryia Illani Mohd Yunos; Siti Nur Hasanah Mohd Yusuf; Zahirrah Begam Mohamed Rasheed
EPI_ISL_8380531	HOSPITAL SULTANAH AMINAH JOHOR BAHRU	UKM Medical Molecular Biology Institute (UMBI)	Khairun Nur Abd Ghafar; Mira Farzana Mohamad Mokhtar; Muhiddin Ishak; Nor Azila Muhammad Azami; Nur Alyaa Afifah Md Shahri; Nurul Syakima Ab Mutalib; Rahman Jamal; Ryia Illani Mohd Yunos; Siti Nur Hasanah Mohd Yusuf; Zahirrah Begam Mohamed Rasheed
EPI_ISL_8500689, EPI_ISL_9578972, EPI_ISL_9579006	HOSPITAL TUANKU FAUZIAH	iPROMISE, UiTM	Ariza Adnan; Fadzilah Mohd Nor; Lim Wai Feng; Mohd Asif Mohd Sukri; Mohd Nur Fakhruzzaman Noorizhab; Mohd Zaki Salleh; Sazzli Shahlan Kassim; Siti Farah Alwani Mohd Nawi; Siti Hamimah Sheikh Abdul Kadir; Teh Lay Kek; Wang Seok Mui
EPI_ISL_10969082, EPI_ISL_10969083, EPI_ISL_10969085, EPI_ISL_10969086, EPI_ISL_10969112, EPI_ISL_10969142, EPI_ISL_10969143			
see above	HRPB	Tropical Infectious Diseases Research & Education Centre (TIDREC), Universiti Malaya	AsmaAnati CheMatSeri; Che-Norainon Yaacob; Jia-Yi Tan; Jo-Ern Wong; Kim-Kee Tan; Mulya-Mustika-Sari Zulkifli; Noor Syahida Azizan; Nur-Hidayana Mahfodz; Sazaly AbuBakar; Siti-Sarah Nor'e
EPI_ISL_8170487, EPI_ISL_8170488, EPI_ISL_8170504, EPI_ISL_8170517, EPI_ISL_8170520, EPI_ISL_8170522	HRPB, IPOH	Tropical Infectious Diseases Research & Education Centre (TIDREC), Universiti Malaya	AsmaAnati CheMatSeri; Che-Norainon Yaacob; Jia-Yi Tan; Jo-Ern Wong; Kim-Kee Tan; Mulya-Mustika-Sari Zulkifli; Noor Syahida Azizan; Nur-Hidayana Mahfodz; Sazaly AbuBakar; Siti-Sarah Nor'e
EPI_ISL_9829882, EPI_ISL_9829883, EPI_ISL_9829884, EPI_ISL_11010497, EPI_ISL_11010498, EPI_ISL_11010499	HRPZ II	Tropical Infectious Diseases Research & Education Centre (TIDREC), Universiti Malaya	AsmaAnati CheMatSeri; Che-Norainon Yaacob; Jia-Yi Tan; Jo-Ern Wong; Kim-Kee Tan; Mulya-Mustika-Sari Zulkifli; Noor Syahida Azizan; Nur-Hidayana Mahfodz; Sazaly AbuBakar; Siti-Sarah Nor'e
EPI_ISL_7987109, EPI_ISL_8380526, EPI_ISL_8380538, EPI_ISL_8380540	HSAJB	UKM Medical Molecular Biology Institute (UMBI)	Ab Mutalib; Abd Ghafar; Ishak; Jamal; Khairun Nur; Khairun Nur Abd Ghafar; Md Shahri; Mira Farzana; Mira Farzana Mohamad Mokhtar; Mohamad Mokhtar; Mohamed Rasheed; Mohd Yunos; Mohd Yusuf; Muhammad Azami; Muhiddin; Muhiddin Ishak; Nor Azila; Nor Azila Muhammad Azami; Nur Alyaa Afifah; Nur Alyaa Afifah Md Shahri; Nurul Syakima; Nurul Syakima Ab Mutalib; Rahman; Rahman Jamal; Ryia Illani; Ryia Illani Mohd Yunos; Siti Nur Hasanah; Siti Nur Hasanah Mohd Yusuf; Zahirrah Begam; Zahirrah Begam Mohamed Rasheed
EPI_ISL_7831398, EPI_ISL_8405629, EPI_ISL_8405646, EPI_ISL_8405656, EPI_ISL_8405657	HTJ	UKM Medical Molecular Biology Institute (UMBI)	Ab Mutalib; Abd Ghafar; Ishak; Jamal; Khairun Nur; Khairun Nur Abd Ghafar; Md Shahri; Mira Farzana; Mira Farzana Mohamad Mokhtar; Mohamad Mokhtar; Mohamed Rasheed; Mohd Yunos; Mohd Yusuf; Muhammad Azami; Muhiddin; Muhiddin Ishak; Nor Azila; Nor Azila Muhammad Azami; Nur Alyaa Afifah; Nur Alyaa Afifah Md Shahri; Nurul Syakima; Nurul Syakima Ab Mutalib; Rahman; Rahman Jamal; Ryia Illani; Ryia Illani Mohd Yunos; Siti Nur Hasanah; Siti Nur Hasanah Mohd Yusuf; Zahirrah Begam; Zahirrah Begam Mohamed Rasheed
EPI_ISL_7831383, EPI_ISL_7831388, EPI_ISL_7987088, EPI_ISL_8314875, EPI_ISL_8380529, EPI_ISL_8438516	HTJS	UKM Medical Molecular Biology Institute (UMBI)	Ab Mutalib; Abd Ghafar; Ishak; Jamal; Khairun Nur; Khairun Nur Abd Ghafar; Md Shahri; Mira Farzana; Mira Farzana Mohamad Mokhtar; Mohamad Mokhtar; Mohamed Rasheed; Mohd Yunos; Mohd Yusuf; Muhammad Azami; Muhiddin; Muhiddin Ishak; Nor Azila; Nor Azila Muhammad Azami; Nur Alyaa Afifah; Nur Alyaa Afifah Md Shahri; Nurul Syakima; Nurul Syakima Ab Mutalib; Rahman; Rahman Jamal; Ryia Illani; Ryia Illani Mohd Yunos; Siti Nur Hasanah; Siti Nur Hasanah Mohd Yusuf; Zahirrah Begam; Zahirrah Begam Mohamed Rasheed
EPI_ISL_8745624, EPI_ISL_8745642, EPI_ISL_8745647, EPI_ISL_8745648, EPI_ISL_8745649, EPI_ISL_8745650, EPI_ISL_8745659, EPI_ISL_10590711, EPI_ISL_10590712, EPI_ISL_10590762, EPI_ISL_10590766			
see above	Hospital Ampang	Malaysia Genome and Vaccine Institute	Azrin Ahmad; Enizza Kasim; Irni Suhayu Sapian; Mohd Faizal Abu Bakar; Mohd Ghows Mohd Azzam.; Mohd Noor Mat Isa; Nor Azfa Johari; Nurhezreen Md Iqbal; Shamsidar Sopie; Siti Noraini Othman; Yusuf Muhammad Noor
EPI_ISL_7831385, EPI_ISL_7831387, EPI_ISL_7831390, EPI_ISL_7831392, EPI_ISL_7831394, EPI_ISL_7831401, EPI_ISL_7831402, EPI_ISL_7831404			
see above	Hospital Canselor Tuanku Muhriz (HCTM)	UKM Medical Molecular Biology Institute (UMBI)	Ab Mutalib; Abd Ghafar; Ishak; Jamal; Khairun Nur; Md Shahri; Mira Farzana; Mohamad Mokhtar; Mohamed Rasheed; Mohd Yunos; Mohd Yusuf; Muhammad Azami; Muhiddin; Nor Azila; Nur Alyaa Afifah; Nurul Syakima; Rahman; Ryia Illani; Siti Nur Hasanah; Zahirrah Begam
EPI_ISL_11900034, EPI_ISL_11900036, EPI_ISL_11900041, EPI_ISL_11900043	Hospital Kajang	Malaysia Genome and Vaccine Institute	Azrin Ahmad; Enizza Kasim; Irni Suhayu Sapian; Mohd Faizal Abu Bakar; Mohd Ghows Mohd Azzam.; Mohd Noor Mat Isa; Nor Azfa Johari; Nurhezreen Md Iqbal; Shamsidar Sopie; Siti Noraini Othman; Yusuf Muhammad Noor
EPI_ISL_9579181	Hospital Kepala Batas	iPROMISE, UiTM	Ariza Adnan; Fadzilah Mohd Nor; Lim Wai Feng; Mohd Asif Mohd Sukri; Mohd Nur Fakhruzzaman Noorizhab; Mohd Zaki Salleh; Sazzli Shahlan Kassim; Siti Farah Alwani Mohd Nawi; Siti Hamimah Sheikh Abdul Kadir; Teh Lay Kek; Wang Seok Mui
EPI_ISL_8745645	Hospital Kuala Kubu Bharu	Malaysia Genome and Vaccine Institute	Azrin Ahmad; Enizza Kasim; Irni Suhayu Sapian; Mohd Faizal Abu Bakar; Mohd Ghows Mohd Azzam.; Mohd Noor Mat Isa; Nor Azfa Johari; Nurhezreen Md Iqbal; Shamsidar Sopie; Siti Noraini Othman; Yusuf Muhammad Noor
EPI_ISL_8745660, EPI_ISL_8745661	Hospital Kuala Lumpur	Malaysia Genome and Vaccine Institute	Azrin Ahmad; Enizza Kasim; Irni Suhayu Sapian; Mohd Faizal Abu Bakar; Mohd Ghows Mohd Azzam.; Mohd Noor Mat Isa; Nor Azfa Johari; Nurhezreen Md Iqbal; Shamsidar Sopie; Siti Noraini Othman; Yusuf Muhammad Noor
EPI_ISL_8745644	Hospital Melaka	Malaysia Genome and Vaccine Institute	Azrin Ahmad; Enizza Kasim; Irni Suhayu Sapian; Mohd Faizal Abu Bakar; Mohd Ghows Mohd Azzam.; Mohd Noor Mat Isa; Nor Azfa Johari; Nurhezreen Md Iqbal; Shamsidar Sopie; Siti Noraini Othman; Yusuf Muhammad Noor
EPI_ISL_7468524, EPI_ISL_7468525, EPI_ISL_7987090, EPI_ISL_7987092, EPI_ISL_7987094	Hospital Melaka	UKM Medical Molecular Biology Institute (UMBI)	Ab Mutalib; Abd Ghafar; Ishak; Jamal; Khairun Nur; Md Shahri; Mira Farzana; Mohamad Mokhtar; Mohamed Rasheed; Mohd Yunos; Mohd Yusuf; Muhammad Azami; Muhiddin; Nor Azila; Nur Alyaa Afifah; Nurul Syakima; Rahman; Ryia Illani; Siti Nur Hasanah; Zahirrah Begam
EPI_ISL_9000494, EPI_ISL_9579000, EPI_ISL_9579005	Hospital Pulau Pinang	iPROMISE, UiTM	Ariza Adnan; Fadzilah Mohd Nor; Lim Wai Feng; Mohd Asif Mohd Sukri; Mohd Nur Fakhruzzaman Noorizhab; Mohd Zaki Salleh; Sazzli Shahlan Kassim; Siti Farah Alwani Mohd Nawi; Siti Hamimah Sheikh Abdul Kadir; Teh Lay Kek; Wang Seok Mui
EPI_ISL_10590736	Hospital Selayang	Malaysia Genome and Vaccine Institute	Azrin Ahmad; Enizza Kasim; Irni Suhayu Sapian; Mohd Faizal Abu Bakar; Mohd Ghows Mohd Azzam.; Mohd Noor Mat Isa; Nurhezreen Md Iqbal; Shamsidar Sopie; Siti Noraini Othman; Yusuf Muhammad Noor
EPI_ISL_8380528, EPI_ISL_8380532, EPI_ISL_8380535, EPI_ISL_8380537, EPI_ISL_8380541, EPI_ISL_8380543, EPI_ISL_8380544, EPI_ISL_8405644			
see above	Hospital Sultanah Aminah	UKM Medical Molecular Biology Institute	Khairun Nur Abd Ghafar; Mira Farzana Mohamad Mokhtar; Muhiddin Ishak; Nor Azila Muhammad Azami; Nur Alyaa Afifah Md Shahri; Nurul Syakima Ab Mutalib; Rahman Jamal; Ryia Illani Mohd Yunos; Siti Nur Hasanah Mohd Yusuf; Zahirrah Begam Mohamed Rasheed

		(UMBI)	
EPI_ISL_7831386, EPI_ISL_7831389, EPI_ISL_7831396, EPI_ISL_7831397	Hospital Sultanah Aminah JB	UKM Medical Molecular Biology Institute (UMBI)	Ab Mutalib; Abd Ghafar; Ishak; Jamal; Khairun Nur; Md Shahri; Mira Farzana; Mohamad Mokhtar; Mohamed Rasheed; Mohd Yunos; Mohd Yusuf; Muhammad Azami; Muhiddin; Nor Azila; Nur Alyaa Afifah; Nurul Syakima; Rahman; Ryia Illani; Siti Nur Hasanah; Zahirrah Begam
EPI_ISL_7831399, EPI_ISL_7987089	Hospital Sultanah Aminah Johor Bahru	UKM Medical Molecular Biology Institute (UMBI)	Ab Mutalib; Abd Ghafar; Ishak; Jamal; Khairun Nur; Md Shahri; Mira Farzana; Mohamad Mokhtar; Mohamed Rasheed; Mohd Yunos; Mohd Yusuf; Muhammad Azami; Muhiddin; Nor Azila; Nur Alyaa Afifah; Nurul Syakima; Rahman; Ryia Illani; Siti Nur Hasanah; Zahirrah Begam
EPI_ISL_10969095, EPI_ISL_10969134	Hospital Sultanah Nurzahirah	Tropical Infectious Diseases Research & Education Centre (TIDREC), Universiti Malaya	AsmaAnati CheMatSeri; Che-Norainon Yaacob; Jia-Yi Tan; Jo-Ern Wong; Kim-Kee Tan; Mulya-Mustika-Sari Zulkifli; Noor Syahida Azizan; Nur-Hidayana Mahfodz; Sazaly AbuBakar; Siti-Sarah Nor'e
EPI_ISL_8745625, EPI_ISL_8745628, EPI_ISL_8745629, EPI_ISL_8745630, EPI_ISL_8745631, EPI_ISL_8745643, EPI_ISL_8745662, EPI_ISL_8745663, EPI_ISL_10590738, EPI_ISL_10590739, EPI_ISL_10590740, EPI_ISL_10590746, EPI_ISL_10590770			
see above	Hospital Sungai Buloh	Malaysia Genome and Vaccine Institute	Azrin Ahmad; Enizza Kasim; Irni Suhayu Sapian; Mohd Faizal Abu Bakar; Mohd Ghows Mohd Azzam.; Mohd Noor Mat Isa; Nor Azfa Johari; Nurhezreen Md Iqbal; Shamsidar Sopie; Siti Noraini Othman; Yusuf Muhammad Noor
EPI_ISL_8745653, EPI_ISL_8745654, EPI_ISL_8745658	Hospital Tawau	Malaysia Genome and Vaccine Institute	Azrin Ahmad; Enizza Kasim; Irni Suhayu Sapian; Mohd Faizal Abu Bakar; Mohd Ghows Mohd Azzam.; Mohd Noor Mat Isa; Nor Azfa Johari; Nurhezreen Md Iqbal; Shamsidar Sopie; Siti Noraini Othman; Yusuf Muhammad Noor
EPI_ISL_10590747	Hospital Tengku Ampuan Rahimah	Malaysia Genome and Vaccine Institute	Azrin Ahmad; Enizza Kasim; Irni Suhayu Sapian; Mohd Faizal Abu Bakar; Mohd Ghows Mohd Azzam.; Mohd Noor Mat Isa; Nurhezreen Md Iqbal; Shamsidar Sopie; Siti Noraini Othman; Yusuf Muhammad Noor
EPI_ISL_8170496	Hospital pasir mas	Tropical Infectious Diseases Research & Education Centre (TIDREC), Universiti Malaya	AsmaAnati CheMatSeri; Che-Norainon Yaacob; Jia-Yi Tan; Jo-Ern Wong; Kim-Kee Tan; Mulya-Mustika-Sari Zulkifli; Noor Syahida Azizan; Nur-Hidayana Mahfodz; Sazaly AbuBakar; Siti-Sarah Nor'e
EPI_ISL_9579027	Hospital pulau pinang	iPROMISE, UiTM	Ariza Adnan; Fadzilah Mohd Nor; Lim Wai Feng; Mohd Asif Mohd Sukri; Mohd Nur Fakhruzzaman Noorizhab; Mohd Zaki Salleh; Sazzli Shahlan Kassim; Siti Farah Alwani Mohd Nawi; Siti Hamimah Sheikh Abdul Kadir; Teh Lay Kek; Wang Seok Mui
EPI_ISL_9578973	Hospital tuanku fauziah	iPROMISE, UiTM	Ariza Adnan; Fadzilah Mohd Nor; Lim Wai Feng; Mohd Asif Mohd Sukri; Mohd Nur Fakhruzzaman Noorizhab; Mohd Zaki Salleh; Sazzli Shahlan Kassim; Siti Farah Alwani Mohd Nawi; Siti Hamimah Sheikh Abdul Kadir; Teh Lay Kek; Wang Seok Mui
EPI_ISL_11323754, EPI_ISL_11323757, EPI_ISL_11323762, EPI_ISL_11323775, EPI_ISL_11323781, EPI_ISL_11323792, EPI_ISL_11323800, EPI_ISL_11323802, EPI_ISL_11323807, EPI_ISL_11323811, EPI_ISL_11323814, EPI_ISL_11323822, EPI_ISL_11323836			
see above	Institut Biologi Molekul Perubatan UKM (UMBI)	UKM Medical Molecular Biology Institute (UMBI)	Khairun Nur Abd Ghafar; Mira Farzana Mohamad Mokhtar; Muhiddin Ishak; Nor Azila Muhammad Azami; Nur Alyaa Afifah Md Shahri; Nurul Syakima Ab Mutalib; Rahman Jamal; Ryia Illani Mohd Yunos; Siti Nur Hasanah Mohd Yusuf; Zahirrah Begam Mohamed Rasheed
EPI_ISL_8170469, EPI_ISL_8170470, EPI_ISL_8170471, EPI_ISL_8170472, EPI_ISL_8170473, EPI_ISL_8170474, EPI_ISL_8170476, EPI_ISL_8170477, EPI_ISL_8170478, EPI_ISL_8170484, EPI_ISL_8170485, EPI_ISL_8170486, EPI_ISL_8170489, EPI_ISL_8170490, EPI_ISL_8170491, EPI_ISL_8170492, EPI_ISL_8170493, EPI_ISL_8170494, EPI_ISL_8170495, EPI_ISL_8170511, EPI_ISL_8170512, EPI_ISL_8170513, EPI_ISL_8170514, EPI_ISL_8170521, EPI_ISL_8170526, EPI_ISL_8170527, EPI_ISL_8170528, EPI_ISL_8170530, EPI_ISL_8170531, EPI_ISL_10969115, EPI_ISL_10969116, EPI_ISL_10969117, EPI_ISL_10969118, EPI_ISL_10969119, EPI_ISL_10969120, EPI_ISL_10969121, EPI_ISL_10969122, EPI_ISL_10969123			
see above	JABATAN PATOLOGI HOSPITAL RAJA Female ZAINAB II	Tropical Infectious Diseases Research & Education Centre (TIDREC), Universiti Malaya	AsmaAnati CheMatSeri; Che-Norainon Yaacob; Jia-Yi Tan; Jo-Ern Wong; Kim-Kee Tan; Mulya-Mustika-Sari Zulkifli; Noor Syahida Azizan; Nur-Hidayana Mahfodz; Sazaly AbuBakar; Siti-Sarah Nor'e
EPI_ISL_10969144, EPI_ISL_10969145	JABATAN PATOLOGI HOSPITAL RAJA PEREMPUAN ZAINAB II	Tropical Infectious Diseases Research & Education Centre (TIDREC), Universiti Malaya	AsmaAnati CheMatSeri; Che-Norainon Yaacob; Jia-Yi Tan; Jo-Ern Wong; Kim-Kee Tan; Mulya-Mustika-Sari Zulkifli; Noor Syahida Azizan; Nur-Hidayana Mahfodz; Sazaly AbuBakar; Siti-Sarah Nor'e
EPI_ISL_8170467, EPI_ISL_8170475, EPI_ISL_8170518, EPI_ISL_8170523	JABATAN PATOLOGI HRPZ II	Tropical Infectious Diseases Research & Education Centre (TIDREC), Universiti Malaya	AsmaAnati CheMatSeri; Che-Norainon Yaacob; Jia-Yi Tan; Jo-Ern Wong; Kim-Kee Tan; Mulya-Mustika-Sari Zulkifli; Noor Syahida Azizan; Nur-Hidayana Mahfodz; Sazaly AbuBakar; Siti-Sarah Nor'e
EPI_ISL_9579132, EPI_ISL_9579160, EPI_ISL_9579161, EPI_ISL_11112438	JKN PAHANG	iPROMISE, UiTM	Ariza Adnan; Fadzilah Mohd Nor; Lim Wai Feng; Mohd Asif Mohd Sukri; Mohd Nur Fakhruzzaman Noorizhab; Mohd Zaki Salleh; Sazzli Shahlan Kassim; Siti Farah Alwani Mohd Nawi; Siti Hamimah Sheikh Abdul Kadir; Teh Lay Kek; Wang Seok Mui
EPI_ISL_8170429, EPI_ISL_8170430, EPI_ISL_10969090	Jabatan Forensik Hospital Sultanah Bahiyah	Tropical Infectious Diseases Research & Education Centre (TIDREC), Universiti Malaya	AsmaAnati CheMatSeri; Che-Norainon Yaacob; Jia-Yi Tan; Jo-Ern Wong; Kim-Kee Tan; Mulya-Mustika-Sari Zulkifli; Noor Syahida Azizan; Nur-Hidayana Mahfodz; Sazaly AbuBakar; Siti-Sarah Nor'e
EPI_ISL_9000514, EPI_ISL_9000515, EPI_ISL_9000516, EPI_ISL_9000517, EPI_ISL_9579124, EPI_ISL_9579125, EPI_ISL_9579126, EPI_ISL_9579127, EPI_ISL_9579128, EPI_ISL_9579129, EPI_ISL_9579130, EPI_ISL_9579131			
see above	Jabatan Kesihatan Negeri	iPROMISE, UiTM	Ariza Adnan; Fadzilah Mohd Nor; Lim Wai Feng; Mohd Asif Mohd Sukri; Mohd Nur Fakhruzzaman Noorizhab; Mohd Zaki Salleh; Sazzli Shahlan Kassim; Siti Farah Alwani Mohd Nawi; Siti Hamimah Sheikh Abdul Kadir; Teh Lay Kek; Wang Seok Mui
EPI_ISL_10590764	Jabatan Kesihatan Negeri WP Labuan	Malaysia Genome and Vaccine Institute	Azrin Ahmad; Enizza Kasim; Irni Suhayu Sapian; Mohd Faizal Abu Bakar; Mohd Ghows Mohd Azzam.; Mohd Noor Mat Isa; Nurhezreen Md Iqbal; Shamsidar Sopie; Siti Noraini Othman; Yusuf Muhammad Noor
EPI_ISL_8745665, EPI_ISL_8745666, EPI_ISL_8745674, EPI_ISL_8745676	Jabatan Kesihatan WP Labuan	Malaysia Genome and Vaccine Institute	Azrin Ahmad; Enizza Kasim; Irni Suhayu Sapian; Mohd Faizal Abu Bakar; Mohd Ghows Mohd Azzam.; Mohd Noor Mat Isa; Nor Azfa Johari; Nurhezreen Md Iqbal; Shamsidar Sopie; Siti Noraini Othman; Yusuf Muhammad Noor
EPI_ISL_9000499	Jabatan Pesakit Luar Hospital Pulau Pinang	iPROMISE, UiTM	Ariza Adnan; Fadzilah Mohd Nor; Lim Wai Feng; Mohd Asif Mohd Sukri; Mohd Nur Fakhruzzaman Noorizhab; Mohd Zaki Salleh; Sazzli Shahlan Kassim; Siti Farah Alwani Mohd Nawi; Siti Hamimah Sheikh Abdul Kadir; Teh Lay Kek; Wang Seok Mui
EPI_ISL_8560635, EPI_ISL_8560638, EPI_ISL_8560690	KLIA Health Office	Institute for Medical Research, Infectious Disease Research Centre, National Institutes of Health, Ministry of Health Malaysia	Ahmad FA; Ahmad Fazilah NA; Anasir MI; Azizan MA; Kamel K; Mohamad Sukri MZ; Norhisham SN; Ramly N; Robert F; Rosli NR; Suppiah J; Thayan R
EPI_ISL_11010504	KLINIK KESIHATAN PERINGAT	Tropical Infectious Diseases Research & Education Centre (TIDREC), Universiti Malaya	AsmaAnati CheMatSeri; Che-Norainon Yaacob; Jia-Yi Tan; Jo-Ern Wong; Kim-Kee Tan; Mulya-Mustika-Sari Zulkifli; Noor Syahida Azizan; Nur-Hidayana Mahfodz; Sazaly AbuBakar; Siti-Sarah Nor'e
EPI_ISL_8170519	KOMPLEKS SURVELANS PKD KINTA	Tropical Infectious Diseases Research & Education Centre (TIDREC), Universiti Malaya	AsmaAnati CheMatSeri; Che-Norainon Yaacob; Jia-Yi Tan; Jo-Ern Wong; Kim-Kee Tan; Mulya-Mustika-Sari Zulkifli; Noor Syahida Azizan; Nur-Hidayana Mahfodz; Sazaly AbuBakar; Siti-Sarah Nor'e
EPI_ISL_11010505	Klinik Kesihatan Kok Lanas	Tropical Infectious Diseases Research & Education Centre (TIDREC), Universiti Malaya	AsmaAnati CheMatSeri; Che-Norainon Yaacob; Jia-Yi Tan; Jo-Ern Wong; Kim-Kee Tan; Mulya-Mustika-Sari Zulkifli; Noor Syahida Azizan; Nur-Hidayana Mahfodz; Sazaly AbuBakar; Siti-Sarah Nor'e
EPI_ISL_7715055, EPI_ISL_7951046, EPI_ISL_7951047, EPI_ISL_7951108, EPI_ISL_7951109, EPI_ISL_7951110, EPI_ISL_7951111, EPI_ISL_7951112, EPI_ISL_7951113, EPI_ISL_7951114, EPI_ISL_7951115			
see above	Kuala Lumpur International Airport (KLIA) Health Office	Institute for Medical Research, Infectious Disease Research Centre, National Institutes of Health, Ministry of Health Malaysia	Ahmad FA; Ahmad Fazilah NA; Anasir MI; Azizan MA; Kamel K; Mohamad Sukri MZ; Mohd Zawawi Z; Norhisham SN; Ramly N; Robert F; Rosli NR; Suppiah J; Thayan R
EPI_ISL_8170499, EPI_ISL_8170500, EPI_ISL_8170515	MKA KOTA BHARU	Tropical Infectious Diseases Research & Education Centre (TIDREC), Universiti Malaya	AsmaAnati CheMatSeri; Che-Norainon Yaacob; Jia-Yi Tan; Jo-Ern Wong; Kim-Kee Tan; Mulya-Mustika-Sari Zulkifli; Noor Syahida Azizan; Nur-Hidayana Mahfodz; Sazaly AbuBakar; Siti-Sarah Nor'e
EPI_ISL_9578999	Medical Hospital Pulau Pinang	iPROMISE, UiTM	Ariza Adnan; Fadzilah Mohd Nor; Lim Wai Feng; Mohd Asif Mohd Sukri; Mohd Nur Fakhruzzaman Noorizhab; Mohd Zaki Salleh; Sazzli Shahlan Kassim; Siti Farah Alwani Mohd Nawi; Siti Hamimah Sheikh Abdul Kadir; Teh Lay Kek; Wang Seok Mui
EPI_ISL_8188752, EPI_ISL_8188753	Miri Hospital Molecular Diagnostic Lab, Miri	Institute of Health and Community Medicine	Chan Chia Jui; Chua Hock Hin; David Perera; Noor Amnani binti Limin; Ooi Mong How; Tonnii Sia Loong Loong
EPI_ISL_9578987, EPI_ISL_9578988, EPI_ISL_9578989, EPI_ISL_9578990, EPI_ISL_9578991, EPI_ISL_9578992, EPI_ISL_9578993, EPI_ISL_9578994, EPI_ISL_9578995, EPI_ISL_9578996, EPI_ISL_9578997			
see above	PEJABAT KESIHATAN DAERAH SEBERANG PERAI UTARA PULAU PINANG	iPROMISE, UiTM	Ariza Adnan; Fadzilah Mohd Nor; Lim Wai Feng; Mohd Asif Mohd Sukri; Mohd Nur Fakhruzzaman Noorizhab; Mohd Zaki Salleh; Sazzli Shahlan Kassim; Siti Farah Alwani Mohd Nawi; Siti Hamimah Sheikh Abdul Kadir; Teh Lay Kek; Wang Seok Mui
EPI_ISL_9579008, EPI_ISL_9579009, EPI_ISL_9579010, EPI_ISL_9579012, EPI_ISL_9579013, EPI_ISL_9579014, EPI_ISL_9579015, EPI_ISL_9579016, EPI_ISL_9579017, EPI_ISL_9579018, EPI_ISL_9579019, EPI_ISL_9579020, EPI_ISL_9579021, EPI_ISL_9579022, EPI_ISL_9579023, EPI_ISL_9579024, EPI_ISL_9579025, EPI_ISL_9579026			
see above	PEJABAT KESIHATAN DAERAH TIMUR LAUT	iPROMISE, UiTM	Ariza Adnan; Fadzilah Mohd Nor; Lim Wai Feng; Mohd Asif Mohd Sukri; Mohd Nur Fakhruzzaman Noorizhab; Mohd Zaki Salleh; Sazzli Shahlan Kassim; Siti Farah Alwani Mohd Nawi; Siti Hamimah Sheikh Abdul Kadir; Teh Lay Kek; Wang Seok Mui
EPI_ISL_11010506, EPI_ISL_11010507	PEJABAT KESIHATAN DAERAH TUMPAT	Tropical Infectious Diseases Research & Education Centre (TIDREC), Universiti Malaya	AsmaAnati CheMatSeri; Che-Norainon Yaacob; Jia-Yi Tan; Jo-Ern Wong; Kim-Kee Tan; Mulya-Mustika-Sari Zulkifli; Noor Syahida Azizan; Nur-Hidayana Mahfodz; Sazaly AbuBakar; Siti-Sarah Nor'e
EPI_ISL_8170497, EPI_ISL_8170498	PKD BACHOK	Tropical Infectious Diseases Research & Education Centre (TIDREC), Universiti Malaya	AsmaAnati CheMatSeri; Che-Norainon Yaacob; Jia-Yi Tan; Jo-Ern Wong; Kim-Kee Tan; Mulya-Mustika-Sari Zulkifli; Noor Syahida Azizan; Nur-Hidayana Mahfodz; Sazaly AbuBakar; Siti-Sarah Nor'e
EPI_ISL_9579003, EPI_ISL_9579036, EPI_ISL_9579037	PKD BARAT DAYA	iPROMISE, UiTM	Ariza Adnan; Fadzilah Mohd Nor; Lim Wai Feng; Mohd Asif Mohd Sukri; Mohd Nur Fakhruzzaman Noorizhab; Mohd Zaki Salleh; Sazzli Shahlan Kassim; Siti Farah Alwani Mohd Nawi; Siti Hamimah Sheikh Abdul Kadir; Teh Lay Kek; Wang Seok Mui
EPI_ISL_11010503	PKD Bachok	Tropical Infectious Diseases Research & Education Centre (TIDREC), Universiti Malaya	AsmaAnati CheMatSeri; Che-Norainon Yaacob; Jia-Yi Tan; Jo-Ern Wong; Kim-Kee Tan; Mulya-Mustika-Sari Zulkifli; Noor Syahida Azizan; Nur-Hidayana Mahfodz; Sazaly AbuBakar; Siti-Sarah Nor'e
EPI_ISL_10969036, EPI_ISL_10969037, EPI_ISL_10969079, EPI_ISL_10969080, EPI_ISL_10969081, EPI_ISL_11010509, EPI_ISL_11010510, EPI_ISL_11010511, EPI_ISL_11010512			
see above	PKD HILIR PERAK	Tropical Infectious Diseases Research & Education Centre (TIDREC), Universiti Malaya	AsmaAnati CheMatSeri; Che-Norainon Yaacob; Jia-Yi Tan; Jo-Ern Wong; Kim-Kee Tan; Mulya-Mustika-Sari Zulkifli; Noor Syahida Azizan; Nur-Hidayana Mahfodz; Sazaly AbuBakar; Siti-Sarah Nor'e

EPI_ISL_8170480, EPI_ISL_8170482, EPI_ISL_8170483	PKD Hilir Perak	Tropical Infectious Diseases Research & Education Centre (TIDREC), Universiti Malaya	AsmaAnati CheMatSeri; Che-Norainon Yaacob; Jia-Yi Tan; Jo-Ern Wong; Kim-Kee Tan; Mulya-Mustika-Sari Zulkifli; Noor Syahida Azizan; Nur-Hidayana Mahfodz; Sazaly AbuBakar; Siti-Sarah Nor'e
EPI_ISL_8177264	PKD KERIAN	Tropical Infectious Diseases Research & Education Centre (TIDREC), Universiti Malaya	AsmaAnati CheMatSeri; Che-Norainon Yaacob; Jia-Yi Tan; Jo-Ern Wong; Kim-Kee Tan; Mulya-Mustika-Sari Zulkifli; Noor Syahida Azizan; Nur-Hidayana Mahfodz; Sazaly AbuBakar; Siti-Sarah Nor'e
EPI_ISL_10969128	PKD LMS	Tropical Infectious Diseases Research & Education Centre (TIDREC), Universiti Malaya	AsmaAnati CheMatSeri; Che-Norainon Yaacob; Jia-Yi Tan; Jo-Ern Wong; Kim-Kee Tan; Mulya-Mustika-Sari Zulkifli; Noor Syahida Azizan; Nur-Hidayana Mahfodz; Sazaly AbuBakar; Siti-Sarah Nor'e
EPI_ISL_7831391, EPI_ISL_7831393, EPI_ISL_7831395, EPI_ISL_8314864, EPI_ISL_8314866, EPI_ISL_8314872, EPI_ISL_8314881			
see above	PKD MELAKA TENGAH	UKM Medical Molecular Biology Institute (UMBI)	Ab Mutalib; Abd Ghafar; Ishak; Jamal; Khairun Nur; Khairun Nur Abd Ghafar; Md Shahri; Mira Farzana; Mira Farzana Mohamad Mokhtar; Mohamad Mokhtar; Mohamed Rasheed; Mohd Yunos; Mohd Yusuf; Muhammad Azami; Muhiddin; Muhiddin Ishak; Nor Azila; Nor Azila Muhammad Azami; Nur Alyaa Afifah; Nur Alyaa Afifah Md Shahri; Nurul Syakima; Nurul Syakima Ab Mutalib; Rahman; Rahman Jamal; Ryia Illani; Ryia Illani Mohd Yunos; Siti Nur Hasanah; Siti Nur Hasanah Mohd Yusuf; Zahirrah Begam; Zahirrah Begam Mohamed Rasheed
EPI_ISL_8170501, EPI_ISL_8170502	PKD PASIR PUTEH	Tropical Infectious Diseases Research & Education Centre (TIDREC), Universiti Malaya	AsmaAnati CheMatSeri; Che-Norainon Yaacob; Jia-Yi Tan; Jo-Ern Wong; Kim-Kee Tan; Mulya-Mustika-Sari Zulkifli; Noor Syahida Azizan; Nur-Hidayana Mahfodz; Sazaly AbuBakar; Siti-Sarah Nor'e
EPI_ISL_7831384, EPI_ISL_7831403, EPI_ISL_8314873, EPI_ISL_8380533, EPI_ISL_8380547	PKD SEREMBAN	UKM Medical Molecular Biology Institute (UMBI)	Ab Mutalib; Abd Ghafar; Ishak; Jamal; Khairun Nur; Khairun Nur Abd Ghafar; Md Shahri; Mira Farzana; Mira Farzana Mohamad Mokhtar; Mohamad Mokhtar; Mohamed Rasheed; Mohd Yunos; Mohd Yusuf; Muhammad Azami; Muhiddin; Muhiddin Ishak; Nor Azila; Nor Azila Muhammad Azami; Nur Alyaa Afifah; Nur Alyaa Afifah Md Shahri; Nurul Syakima; Nurul Syakima Ab Mutalib; Rahman; Rahman Jamal; Ryia Illani; Ryia Illani Mohd Yunos; Siti Nur Hasanah; Siti Nur Hasanah Mohd Yusuf; Zahirrah Begam; Zahirrah Begam Mohamed Rasheed
EPI_ISL_9000485, EPI_ISL_9000506, EPI_ISL_9000508, EPI_ISL_9000510, EPI_ISL_9000524, EPI_ISL_9000525, EPI_ISL_9000526, EPI_ISL_9000527, EPI_ISL_9000528, EPI_ISL_9000529, EPI_ISL_9000531, EPI_ISL_9578969, EPI_ISL_9578978, EPI_ISL_9578979, EPI_ISL_9578980, EPI_ISL_9578981, EPI_ISL_9578982, EPI_ISL_9578983, EPI_ISL_9578984, EPI_ISL_9578985, EPI_ISL_9578986			
see above	PKD SPT	iPROMISE, UiTM	Ariza Adnan; Fadzilah Mohd Nor; Lim Wai Feng; Mohd Asif Mohd Sukri; Mohd Nur Fakhruzzaman Noorizhab; Mohd Zaki Salleh; Sazzli Shahlan Kassim; Siti Farah Alwani Mohd Nawi; Siti Hamimah Sheikh Abdul Kadir; Teh Lay Kek; Wang Seok Mui
EPI_ISL_8314865, EPI_ISL_8314868, EPI_ISL_8314869, EPI_ISL_8314877, EPI_ISL_8314878, EPI_ISL_8314880, EPI_ISL_8314882, EPI_ISL_8405634, EPI_ISL_8405638, EPI_ISL_8405642, EPI_ISL_8405643, EPI_ISL_8405651, EPI_ISL_9317723, EPI_ISL_10397089			
see above	PKD TAMPIN	UKM Medical Molecular Biology Institute (UMBI)	Khairun Nur Abd Ghafar; Mira Farzana Mohamad Mokhtar; Muhiddin Ishak; Nor Azila Muhammad Azami; Nur Alyaa Afifah Md Shahri; Nurul Syakima Ab Mutalib; Rahman Jamal; Ryia Illani Mohd Yunos; Siti Nur Hasanah Mohd Yusuf; Zahirrah Begam Mohamed Rasheed
EPI_ISL_8314862, EPI_ISL_8314876	PKD TANGKAK	UKM Medical Molecular Biology Institute (UMBI)	Khairun Nur Abd Ghafar; Mira Farzana Mohamad Mokhtar; Muhiddin Ishak; Nor Azila Muhammad Azami; Nur Alyaa Afifah Md Shahri; Nurul Syakima Ab Mutalib; Rahman Jamal; Ryia Illani Mohd Yunos; Siti Nur Hasanah Mohd Yusuf; Zahirrah Begam Mohamed Rasheed
EPI_ISL_7987099, EPI_ISL_7987103	PKD Tampin	UKM Medical Molecular Biology Institute (UMBI)	Ab Mutalib; Abd Ghafar; Ishak; Jamal; Khairun Nur; Md Shahri; Mira Farzana; Mohamad Mokhtar; Mohamed Rasheed; Mohd Yunos; Mohd Yusuf; Muhammad Azami; Muhiddin; Nor Azila; Nur Alyaa Afifah; Nurul Syakima; Rahman; Ryia Illani; Siti Nur Hasanah; Zahirrah Begam
EPI_ISL_8170516, EPI_ISL_8170536, EPI_ISL_10969141	PKD Tanah Merah	Tropical Infectious Diseases Research & Education Centre (TIDREC), Universiti Malaya	AsmaAnati CheMatSeri; Che-Norainon Yaacob; Jia-Yi Tan; Jo-Ern Wong; Kim-Kee Tan; Mulya-Mustika-Sari Zulkifli; Noor Syahida Azizan; Nur-Hidayana Mahfodz; Sazaly AbuBakar; Siti-Sarah Nor'e
EPI_ISL_11010508	Pejabat Kesihatan Daerah Jeli	Tropical Infectious Diseases Research & Education Centre (TIDREC), Universiti Malaya	AsmaAnati CheMatSeri; Che-Norainon Yaacob; Jia-Yi Tan; Jo-Ern Wong; Kim-Kee Tan; Mulya-Mustika-Sari Zulkifli; Noor Syahida Azizan; Nur-Hidayana Mahfodz; Sazaly AbuBakar; Siti-Sarah Nor'e
EPI_ISL_10969146	Pejabat Kesihatan Daerah Langkawi	Tropical Infectious Diseases Research & Education Centre (TIDREC), Universiti Malaya	AsmaAnati CheMatSeri; Che-Norainon Yaacob; Jia-Yi Tan; Jo-Ern Wong; Kim-Kee Tan; Mulya-Mustika-Sari Zulkifli; Noor Syahida Azizan; Nur-Hidayana Mahfodz; Sazaly AbuBakar; Siti-Sarah Nor'e
EPI_ISL_8745635	Pejabat Kesihatan Kawasan Sandakan	Malaysia Genome and Vaccine Institute	Azrin Ahmad; Enizza Kasim; Irni Suhayu Sapian; Mohd Faizal Abu Bakar; Mohd Ghows Mohd Azzam.; Mohd Noor Mat Isa; Nor Azfa Johari; Nurhezreen Md Iqbal; Shamsidar Sopie; Siti Noraini Othman; Yusuf Muhammad Noor
EPI_ISL_8745670	Pejabat Kesihatan Kawasan Tuaran	Malaysia Genome and Vaccine Institute	Azrin Ahmad; Enizza Kasim; Irni Suhayu Sapian; Mohd Faizal Abu Bakar; Mohd Ghows Mohd Azzam.; Mohd Noor Mat Isa; Nor Azfa Johari; Nurhezreen Md Iqbal; Shamsidar Sopie; Siti Noraini Othman; Yusuf Muhammad Noor
EPI_ISL_8745639	Pejabat Kesihatan Lembah Pantai	Malaysia Genome and Vaccine Institute	Azrin Ahmad; Enizza Kasim; Irni Suhayu Sapian; Mohd Faizal Abu Bakar; Mohd Ghows Mohd Azzam.; Mohd Noor Mat Isa; Nor Azfa Johari; Nurhezreen Md Iqbal; Shamsidar Sopie; Siti Noraini Othman; Yusuf Muhammad Noor
EPI_ISL_10969078	Pejabat kesihatan daerah pasir puteh	Tropical Infectious Diseases Research & Education Centre (TIDREC), Universiti Malaya	AsmaAnati CheMatSeri; Che-Norainon Yaacob; Jia-Yi Tan; Jo-Ern Wong; Kim-Kee Tan; Mulya-Mustika-Sari Zulkifli; Noor Syahida Azizan; Nur-Hidayana Mahfodz; Sazaly AbuBakar; Siti-Sarah Nor'e
EPI_ISL_10495838, EPI_ISL_10495854, EPI_ISL_10495861	QUEEN ELIZABETH HOSPITAL	Institute for Medical Research, Infectious Disease Research Centre, National Institutes of Health, Ministry of Health Malaysia	Ahmad FA; Ahmad Fazilah NA; Anasir MI; Arip MS; Azizan MA; Christopher Hollip K; Jaimin JJ; Kamel K; Mohamad Sukri MZ; Mohammad R; Mohd Yasin R; Norhisham SN; Ramly N; Robert F; Rosli NR; Shamsaddin E; Suppiah J; Tan YC; Thayan R
EPI_ISL_7473041, EPI_ISL_7473042, EPI_ISL_7473043, EPI_ISL_8188860, EPI_ISL_8188861, EPI_ISL_8188862, EPI_ISL_8188863, EPI_ISL_8188864, EPI_ISL_8650114, EPI_ISL_8650115, EPI_ISL_8650116, EPI_ISL_8650117, EPI_ISL_8650118, EPI_ISL_8650119, EPI_ISL_8650138, EPI_ISL_8650139, EPI_ISL_8782811			
see above	Rejang Medical Centre (Sibu)	Institute of Health and Community Medicine	Chan Chia Jui; Chua Hock Hin; David Perera; Ooi Mong How; Tonnii Sia Loong Loong; Wong Jyn Shan
EPI_ISL_8405162	Salak Health Clinic	Institute for Medical Research, Infectious Disease Research Centre, National Institutes of Health, Ministry of Health Malaysia	Ahmad FA; Ahmad Fazilah NA; Anasir MI; Azizan MA; Kamel K; Mohamad Sukri MZ; Norhisham SN; Ramly N; Robert F; Rosli NR; Suppiah J; Thayan R
EPI_ISL_7472970, EPI_ISL_8188726, EPI_ISL_8188727, EPI_ISL_8188728, EPI_ISL_8188729, EPI_ISL_8188730, EPI_ISL_8188731, EPI_ISL_8188732, EPI_ISL_8188733, EPI_ISL_8188742, EPI_ISL_8188743, EPI_ISL_8188744, EPI_ISL_8188745, EPI_ISL_8188767, EPI_ISL_8188768, EPI_ISL_8188828, EPI_ISL_8188829, EPI_ISL_8188830, EPI_ISL_8188831, EPI_ISL_8188843			
see above	Sarawak General Hospital (Kuching)	Institute of Health and Community Medicine	Chan Chia Jui; Chua Hock Hin; David Perera; Ooi Mong How; Tonnii Sia Loong Loong; Wong Jyn Shan
EPI_ISL_8188746	Sarawak Heart Centre (SHC), Kota Samarahan	Institute of Health and Community Medicine	Chan Chia Jui; Chua Hock Hin; David Perera; Ooi Mong How; Tonnii Sia Loong Loong; Wong Jyn Shan
EPI_ISL_8692650	Sepang District Health Office	Institute for Medical Research, Infectious Disease Research Centre, National Institutes of Health, Ministry of Health Malaysia	Ahmad FA; Ahmad Fazilah NA; Anasir MI; Azizan MA; Kamel K; Mohamad Sukri MZ; Norhisham SN; Ramly N; Robert F; Rosli NR; Suppiah J; Thayan R
EPI_ISL_8295901, EPI_ISL_8295906	Tropical Infectious Diseases Research & Education Centre (TIDREC), Universiti Malaya	Tropical Infectious Diseases Research & Education Centre (TIDREC), Universiti Malaya	AsmaAnati CheMatSeri; Che-Norainon Yaacob; Jia-Yi Tan; Jo-Ern Wong; Kim-Kee Tan; Mulya-Mustika-Sari Zulkifli; Noor Syahida Azizan; Nur-Hidayana Mahfodz; Sazaly AbuBakar; Siti-Sarah Nor'e
EPI_ISL_9000493	Unit Forensik Hospital Kepala Batas	iPROMISE, UiTM	Ariza Adnan; Fadzilah Mohd Nor; Lim Wai Feng; Mohd Asif Mohd Sukri; Mohd Nur Fakhruzzaman Noorizhab; Mohd Zaki Salleh; Sazzli Shahlan Kassim; Siti Farah Alwani Mohd Nawi; Siti Hamimah Sheikh Abdul Kadir; Teh Lay Kek; Wang Seok Mui
EPI_ISL_9000492	hospital pulau pinang	iPROMISE, UiTM	Ariza Adnan; Fadzilah Mohd Nor; Lim Wai Feng; Mohd Asif Mohd Sukri; Mohd Nur Fakhruzzaman Noorizhab; Mohd Zaki Salleh; Sazzli Shahlan Kassim; Siti Farah Alwani Mohd Nawi; Siti Hamimah Sheikh Abdul Kadir; Teh Lay Kek; Wang Seok Mui
EPI_ISL_9578970, EPI_ISL_9578971	hospital tuanku fauziah	iPROMISE, UiTM	Ariza Adnan; Fadzilah Mohd Nor; Lim Wai Feng; Mohd Asif Mohd Sukri; Mohd Nur Fakhruzzaman Noorizhab; Mohd Zaki Salleh; Sazzli Shahlan Kassim; Siti Farah Alwani Mohd Nawi; Siti Hamimah Sheikh Abdul Kadir; Teh Lay Kek; Wang Seok Mui
EPI_ISL_10969099	pkd lms	Tropical Infectious Diseases Research & Education Centre (TIDREC), Universiti Malaya	AsmaAnati CheMatSeri; Che-Norainon Yaacob; Jia-Yi Tan; Jo-Ern Wong; Kim-Kee Tan; Mulya-Mustika-Sari Zulkifli; Noor Syahida Azizan; Nur-Hidayana Mahfodz; Sazaly AbuBakar; Siti-Sarah Nor'e
EPI_ISL_9829878	pkd manjung	Tropical Infectious Diseases Research & Education Centre (TIDREC), Universiti Malaya	AsmaAnati CheMatSeri; Che-Norainon Yaacob; Jia-Yi Tan; Jo-Ern Wong; Kim-Kee Tan; Mulya-Mustika-Sari Zulkifli; Noor Syahida Azizan; Nur-Hidayana Mahfodz; Sazaly AbuBakar; Siti-Sarah Nor'e

We gratefully acknowledge the following Authors from the Originating laboratories responsible for obtaining the specimens, as well as the Submitting laboratories where the genome data were generated and shared via GISAID, on which this research is based.

All Submitters of data may be contacted directly via www.gisaid.org

Authors are sorted alphabetically.

Accession ID	Originating Laboratory	Submitting Laboratory	Authors
EPI_ISL_10590707, EPI_ISL_10590708	BP Clinical Lab Sdn Bhd (KLIA)	Malaysia Genome and Vaccine Institute	Azrin Ahmad; Enizza Kasim; Irni Suhayu Sapian; Mohd Faizal Abu Bakar; Mohd Ghows Mohd Azzam.; Mohd Noor Mat Isa; Nurhezreen Md Iqbal; Shamsidar Sopie; Siti Noraini Othman; Yusuf Muhammad Noor
EPI_ISL_8188871, EPI_ISL_8188872	Bintulu Hospital PCR Lab, (sample from KKBTU)	Institute of Health and Community Medicine	Chan Chia Jui; Chua Hock Hin; David Perera; Nur Munirah binti Mazlan; Ooi Mong How; Tonnii Sia Loong Loong; Wong Jyn Shan
EPI_ISL_8650129	Bintulu Hospital PCR Lab, Bintulu	Institute of Health and Community Medicine	Chan Chia Jui; Chua Hock Hin; David Perera; Ooi Mong How; Tonnii Sia Loong Loong; Wong Jyn Shan
EPI_ISL_8188874	Bintulu Medical Centre (Bintulu)	Institute of Health and Community Medicine	Chan Chia Jui; Chua Hock Hin; David Perera; Ooi Mong How; Tonnii Sia Loong Loong; Wong Jyn Shan
EPI_ISL_8188857, EPI_ISL_8188858, EPI_ISL_8188859, EPI_ISL_8782824, EPI_ISL_8782825, EPI_ISL_8782826	Borneo Medical Centre (Kuching)	Institute of Health and Community Medicine	Chan Chia Jui; Chua Hock Hin; David Perera; Ooi Mong How; Tonnii Sia Loong Loong; Wong Jyn Shan
EPI_ISL_8560641, EPI_ISL_8560644	Bukit Changgang Health Clinic	Institute for Medical Research, Infectious Disease Research Centre, National Institutes of Health, Ministry of Health Malaysia	Ahmad FA; Ahmad Fazilah NA; Anasir MI; Azizan MA; Kamel K; Mohamad Sukri MZ; Norhisham SN; Ramly N; Robert F; Rosli NR; Suppiah J; Thayan R
EPI_ISL_8560654, EPI_ISL_8560661, EPI_ISL_8560665, EPI_ISL_8560672, EPI_ISL_8560678	Cheras District Health Office	Institute for Medical Research, Infectious Disease Research Centre, National Institutes of Health, Ministry of Health Malaysia	Ahmad FA; Ahmad Fazilah NA; Anasir MI; Azizan MA; Kamel K; Mohamad Sukri MZ; Norhisham SN; Ramly N; Robert F; Rosli NR; Suppiah J; Thayan R
EPI_ISL_8564944, EPI_ISL_8564945, EPI_ISL_8564946, EPI_ISL_10431130, EPI_ISL_10431140, EPI_ISL_10431142, EPI_ISL_10439972			
see above	Department of Medical Microbiology and Parasitology, Hospital Universiti Sains Malaysia	Molecular Research Laboratory	Abdul Haris bin Muhammad; Ahmad Adebayo Irekeola; Ahmad Sukari Bin Halim; Alexander Chong Shu Chien; Aswini Leela; Azian Harun; Chan Yean Yean; Chua Wei Chuan; Chua Wei Lian; Engku Nur Syafirah bt Engku Abd Rahman; Farahana binti Mohamed; Farahana binti Mohamed Alexander Chong Shu Chien; Kirnpal Kaur Banga Singh; Lau Nyok Sean; Lee Lih Huey; Lim Shu Yong; Maizun binti Mohd Zain; Muhammad Azamuddeen bin Mohammad Nasir; Muhammad Fazli bin Khalid; Muhammad Nashrul Farhan Samsudin; Muhammad Zarul Hanifah Bin Md Zoqratt; Naveed Ahmed; Nazmi Liana Binti Azmi; Noor Hafizan binti Mat Salleh; Nurfadhlina Musa; Qasim Ayub; Rosline Hassan; Sadequr Rahman; Syahida binti Omar; Wan Mohd Zahiruddin Wan Mohammad; Wardah Yusof; Wilhelm Eng Wei Han; Zaini bin Hussin; Zakuan Zainy Deris
EPI_ISL_8646702, EPI_ISL_8646703, EPI_ISL_8646704, EPI_ISL_8646705, EPI_ISL_8646713, EPI_ISL_8646714, EPI_ISL_8646720			
see above	Department of Medical Microbiology and Parasitology, University of Science Malaysia Hospital	Molecular Research Laboratory, Department of Medical Microbiology and Parasitology, University of Science Health Campus	Abdul Haris bin Muhammad; Ahmad Adebayo Irekeola; Ahmad Sukari Bin Halim; Alexander Chong Shu Chien; Aswini Leela; Azian Harun; Chan Yean Yean; Chua Wei Chuan; Chua Wei Lian; Engku Nur Syafirah bt Engku Abd Rahman; Farahana binti Mohamed; Kirnpal Kaur Banga Singh; Lau Nyok Sean; Lee Lih Huey; Lim Shu Yong; Maizun binti Mohd Zain; Muhammad Azamuddeen bin Mohammad Nasir; Muhammad Fazli bin Khalid; Muhammad Nashrul Farhan Samsudin; Muhammad Zarul Hanifah Bin Md Zoqratt; Naveed Ahmed; Nazmi Liana Binti Azmi; Noor Hafizan binti Mat Salleh; Nurfadhlina Musa; Qasim Ayub; Rosline Hassan; Sadequr Rahman; Syahida binti Omar; Wan Mohd Zahiruddin Wan Mohammad; Wardah Yusof; Wilhelm Eng Wei Han; Zaini bin Hussin; Zakuan Zainy Deris
EPI_ISL_8560642	Employee Safety and Health Unit NIH	Institute for Medical Research, Infectious Disease Research Centre, National Institutes of Health, Ministry of Health Malaysia	Ahmad FA; Ahmad Fazilah NA; Anasir MI; Azizan MA; Kamel K; Mohamad Sukri MZ; Norhisham SN; Ramly N; Robert F; Rosli NR; Suppiah J; Thayan R
EPI_ISL_8188834, EPI_ISL_8188835, EPI_ISL_8188870, EPI_ISL_8650089, EPI_ISL_8650090, EPI_ISL_8650091	Gribbles Pathology, Kuching	Institute of Health and Community Medicine	Chan Chia Jui; Chua Hock Hin; David Perera; Josephine Liew Chin Chin; Liew Chin Chin J; Ooi Mong How; Tonnii Sia Loong Loong; Wong Jyn Shan
EPI_ISL_8380534, EPI_ISL_8380536, EPI_ISL_9317731	HOSPITAL MELAKA	UKM Medical Molecular Biology Institute (UMBI)	Khairun Nur Abd Ghafar; Mira Farzana Mohamad Mokhtar; Muhiddin Ishak; Nor Azila Muhammad Azami; Nur Alyaa Afifah Md Shahri; Nurul Syakima Ab Mutalib; Rahman Jamal; Ryia Illani Mohd Yunos; Siti Nur Hasanah Mohd Yusuf; Zahirrah Begam Mohamed Rasheed
EPI_ISL_9579039, EPI_ISL_9579040, EPI_ISL_9579041, EPI_ISL_9579116	HOSPITAL PULAU PINANG	iPROMISE, UiTM	Ariza Adnan; Fadzilah Mohd Nor; Lim Wai Feng; Mohd Asif Mohd Sukri; Mohd Nur Fakhruzzaman Noorizhab; Mohd Zaki Salleh; Sazzli Shahlan Kassim; Siti Farah Alwani Mohd Nawi; Siti Hamimah Sheikh Abdul Kadir; Teh Lay Kek; Wang Seok Mui
EPI_ISL_9829359, EPI_ISL_9829363, EPI_ISL_9829364	HOSPITAL SULTANAH AMINAH	UKM Medical Molecular Biology Institute (UMBI)	Khairun Nur Abd Ghafar; Mira Farzana Mohamad Mokhtar; Muhiddin Ishak; Nor Azila Muhammad Azami; Nur Alyaa Afifah Md Shahri; Nurul Syakima Ab Mutalib; Rahman Jamal; Ryia Illani Mohd Yunos; Siti Nur Hasanah Mohd Yusuf; Zahirrah Begam Mohamed Rasheed
EPI_ISL_10652464, EPI_ISL_10652465	HOSPITAL SULTANAH NUR ZAHIRAH	Department of Medical Microbiology, Faculty of Medicine, University of Malaya; University of Malaya Medical Centre	I-Ching SAM; Jolene Yin Ling FU; Omar Khalilur Rahman; Yoke Fun Chan
EPI_ISL_9579029, EPI_ISL_9579034, EPI_ISL_9579035, EPI_ISL_9579044	HPP	iPROMISE, UiTM	Ariza Adnan; Fadzilah Mohd Nor; Lim Wai Feng; Mohd Asif Mohd Sukri; Mohd Nur Fakhruzzaman Noorizhab; Mohd Zaki Salleh; Sazzli Shahlan Kassim; Siti Farah Alwani Mohd Nawi; Siti Hamimah Sheikh Abdul Kadir; Teh Lay Kek; Wang Seok Mui
EPI_ISL_10969003, EPI_ISL_10969004, EPI_ISL_10969038, EPI_ISL_10969039, EPI_ISL_10969084, EPI_ISL_10969100, EPI_ISL_10969101, EPI_ISL_10969113, EPI_ISL_10969137, EPI_ISL_10969150, EPI_ISL_10969151			
see above	HRPB	Tropical Infectious Diseases Research & Education Centre (TIDREC), Universiti Malaya	AsmaAnati CheMatSeri; Che-Norainon Yaacob; Jia-Yi Tan; Jo-Ern Wong; Kim-Kee Tan; Mulya-Mustika-Sari Zulkifli; Noor Syahida Azizan; Nur-Hidayana Mahfodz; Sazaly AbuBakar; Siti-Sarah Nor'e
EPI_ISL_10969006	HRPZ II	Tropical Infectious Diseases Research & Education Centre (TIDREC), Universiti Malaya	AsmaAnati CheMatSeri; Che-Norainon Yaacob; Jia-Yi Tan; Jo-Ern Wong; Kim-Kee Tan; Mulya-Mustika-Sari Zulkifli; Noor Syahida Azizan; Nur-Hidayana Mahfodz; Sazaly AbuBakar; Siti-Sarah Nor'e
EPI_ISL_9829358, EPI_ISL_9829380, EPI_ISL_9829384	HSAJB	UKM Medical Molecular Biology Institute (UMBI)	Khairun Nur Abd Ghafar; Mira Farzana Mohamad Mokhtar; Muhiddin Ishak; Nor Azila Muhammad Azami; Nur Alyaa Afifah Md Shahri; Nurul Syakima Ab Mutalib; Rahman Jamal; Ryia Illani Mohd Yunos; Siti Nur Hasanah Mohd Yusuf; Zahirrah Begam Mohamed Rasheed
EPI_ISL_10652473	HSOPITAL RAJA PERMAISURI BAINUN	Department of Medical Microbiology, Faculty of Medicine, University of Malaya; University of Malaya Medical Centre	I-Ching SAM; Jolene Yin Ling FU; Omar Khalilur Rahman; Yoke Fun Chan
EPI_ISL_8405654, EPI_ISL_8405655	HTJ	UKM Medical Molecular Biology Institute (UMBI)	Khairun Nur Abd Ghafar; Mira Farzana Mohamad Mokhtar; Muhiddin Ishak; Nor Azila Muhammad Azami; Nur Alyaa Afifah Md Shahri; Nurul Syakima Ab Mutalib; Rahman Jamal; Ryia Illani Mohd Yunos; Siti Nur Hasanah Mohd Yusuf; Zahirrah Begam Mohamed Rasheed
EPI_ISL_8380542, EPI_ISL_8380545, EPI_ISL_8405632, EPI_ISL_8405647, EPI_ISL_8405658, EPI_ISL_8559639	HTJS	UKM Medical Molecular Biology Institute (UMBI)	Khairun Nur Abd Ghafar; Mira Farzana Mohamad Mokhtar; Muhiddin Ishak; Nor Azila Muhammad Azami; Nur Alyaa Afifah Md Shahri; Nurul Syakima Ab Mutalib; Rahman Jamal; Ryia Illani Mohd Yunos; Siti Nur Hasanah Mohd Yusuf; Zahirrah Begam Mohamed Rasheed
EPI_ISL_10590709, EPI_ISL_10590710, EPI_ISL_10590713, EPI_ISL_10590714, EPI_ISL_10590715, EPI_ISL_10590716, EPI_ISL_10590717, EPI_ISL_10590718, EPI_ISL_10590719, EPI_ISL_10590763, EPI_ISL_10590801			
see above	Hospital Ampang	Malaysia Genome and Vaccine Institute	Azrin Ahmad; Enizza Kasim; Irni Suhayu Sapian; Mohd Faizal Abu Bakar; Mohd Ghows Mohd Azzam.; Mohd Noor Mat Isa; Nurhezreen Md Iqbal; Shamsidar Sopie; Siti Noraini Othman; Yusuf Muhammad Noor
EPI_ISL_11899924, EPI_ISL_11899926, EPI_ISL_11899927, EPI_ISL_11899928, EPI_ISL_11899930, EPI_ISL_11899931, EPI_ISL_11899932			
see above	Hospital Kuala Lumpur	Malaysia Genome and Vaccine Institute	Azrin Ahmad; Enizza Kasim; Irni Suhayu Sapian; Mohd Faizal Abu Bakar; Mohd Ghows Mohd Azzam.; Mohd Noor Mat Isa; Nor Azfa Johari; Nurhezreen Md Iqbal; Shamsidar Sopie; Siti Noraini Othman; Yusuf Muhammad Noor
EPI_ISL_8745652	Hospital Kudat	Malaysia Genome and Vaccine Institute	Azrin Ahmad; Enizza Kasim; Irni Suhayu Sapian; Mohd Faizal Abu Bakar; Mohd Ghows Mohd Azzam.; Mohd Noor Mat Isa; Nor Azfa Johari; Nurhezreen Md Iqbal; Shamsidar Sopie; Siti Noraini Othman; Yusuf Muhammad Noor
EPI_ISL_9579042	Hospital Medan	iPROMISE, UiTM	Ariza Adnan; Fadzilah Mohd Nor; Lim Wai Feng; Mohd Asif Mohd Sukri; Mohd Nur Fakhruzzaman Noorizhab; Mohd Zaki Salleh; Sazzli Shahlan Kassim; Siti Farah Alwani Mohd Nawi; Siti Hamimah Sheikh Abdul Kadir; Teh Lay Kek; Wang Seok Mui
EPI_ISL_8405630, EPI_ISL_8405636, EPI_ISL_8405637, EPI_ISL_8405649	Hospital Melaka	UKM Medical Molecular Biology Institute (UMBI)	Khairun Nur Abd Ghafar; Mira Farzana Mohamad Mokhtar; Muhiddin Ishak; Nor Azila Muhammad Azami; Nur Alyaa Afifah Md Shahri; Nurul Syakima Ab Mutalib; Rahman Jamal; Ryia Illani Mohd Yunos; Siti Nur Hasanah Mohd Yusuf; Zahirrah Begam Mohamed Rasheed
EPI_ISL_9579028, EPI_ISL_9579031, EPI_ISL_9579032, EPI_ISL_9579111	Hospital Pulau Pinang	iPROMISE, UiTM	Ariza Adnan; Fadzilah Mohd Nor; Lim Wai Feng; Mohd Asif Mohd Sukri; Mohd Nur Fakhruzzaman Noorizhab; Mohd Zaki Salleh; Sazzli Shahlan Kassim; Siti Farah Alwani Mohd Nawi; Siti Hamimah Sheikh Abdul Kadir; Teh Lay Kek; Wang Seok Mui
EPI_ISL_9829366	Hospital Sultanah A,inah	UKM Medical Molecular Biology Institute (UMBI)	Khairun Nur Abd Ghafar; Mira Farzana Mohamad Mokhtar; Muhiddin Ishak; Nor Azila Muhammad Azami; Nur Alyaa Afifah Md Shahri; Nurul Syakima Ab Mutalib; Rahman Jamal; Ryia Illani Mohd Yunos; Siti Nur Hasanah Mohd Yusuf; Zahirrah Begam Mohamed Rasheed
EPI_ISL_10652466	Hospital Sultanah Nur Zahirah	Department of Medical Microbiology, Faculty of Medicine, University of Malaya; University of Malaya Medical Centre	I-Ching SAM; Jolene Yin Ling FU; Omar Khalilur Rahman; Yoke Fun Chan
EPI_ISL_10969135, EPI_ISL_10969136	Hospital Sultanah Nur Zahirah	Tropical Infectious Diseases Research & Education Centre (TIDREC), Universiti Malaya	AsmaAnati CheMatSeri; Che-Norainon Yaacob; Jia-Yi Tan; Jo-Ern Wong; Kim-Kee Tan; Mulya-Mustika-Sari Zulkifli; Noor Syahida Azizan; Nur-Hidayana Mahfodz; Sazaly AbuBakar; Siti-Sarah Nor'e
EPI_ISL_10590737,	Hospital Sungai Buloh	Malaysia Genome and Vaccine Institute	Azrin Ahmad; Enizza Kasim; Irni Suhayu Sapian; Mohd Faizal Abu Bakar; Mohd Ghows Mohd Azzam.; Mohd Noor Mat Isa; Nurhezreen Md Iqbal; Shamsidar Sopie; Siti Noraini Othman; Yusuf Muhammad Noor

EPI_ISL_10590741, EPI_ISL_10590742, EPI_ISL_10590743			
EPI_ISL_10969109	Hospital Tanah Merah	Tropical Infectious Diseases Research & Education Centre (TIDREC), Universiti Malaya	AsmaAnati CheMatSeri; Che-Norainon Yaacob; Jia-Yi Tan; Jo-Ern Wong; Kim-Kee Tan; Mulya-Mustika-Sari Zulkifli; Noor Syahida Azizan; Nur-Hidayana Mahfodz; Sazaly AbuBakar; Siti-Sarah Nor'e
EPI_ISL_10652516	Hospital Teluk Intan	Department of Medical Microbiology, Faculty of Medicine, University of Malaya; University of Malaya Medical Centre	I-Ching SAM; Jolene Yin Ling FU; Omar Khalilur Rahman; Yoke Fun Chan
EPI_ISL_10590705, EPI_ISL_10590748, EPI_ISL_10590778, EPI_ISL_10590799	Hospital Tengku Ampuan Rahimah	Malaysia Genome and Vaccine Institute	Azrin Ahmad; Enizza Kasim; Irni Suhayu Sapian; Mohd Faizal Abu Bakar; Mohd Ghows Mohd Azzam.; Mohd Noor Mat Isa; Nurhezreen Md Iqbal; Shamsidar Sopie; Siti Noraini Othman; Yusuf Muhammad Noor
EPI_ISL_10590749	Hospital Tuaran	Malaysia Genome and Vaccine Institute	Azrin Ahmad; Enizza Kasim; Irni Suhayu Sapian; Mohd Faizal Abu Bakar; Mohd Ghows Mohd Azzam.; Mohd Noor Mat Isa; Nurhezreen Md Iqbal; Shamsidar Sopie; Siti Noraini Othman; Yusuf Muhammad Noor
EPI_ISL_9317717, EPI_ISL_9317742	Hospital melaka	UKM Medical Molecular Biology Institute (UMBI)	Khairun Nur Abd Ghafar; Mira Farzana Mohamad Mokhtar; Muhiddin Ishak; Nor Azila Muhammad Azami; Nur Alyaa Afifah Md Shahri; Nurul Syakima Ab Mutalib; Rahman Jamal; Ryia Illani Mohd Yunos; Siti Nur Hasanah Mohd Yusuf; Zahirrah Begam Mohamed Rasheed
EPI_ISL_9579043	Hospital pulau pinang	iPROMISE, UiTM	Ariza Adnan; Fadzilah Mohd Nor; Lim Wai Feng; Mohd Asif Mohd Sukri; Mohd Nur Fakhruzzaman Noorizhab; Mohd Zaki Salleh; Sazzli Shahlan Kassim; Siti Farah Alwani Mohd Nawi; Siti Hamimah Sheikh Abdul Kadir; Teh Lay Kek; Wang Seok Mui
EPI_ISL_11323776, EPI_ISL_11323778, EPI_ISL_11323783, EPI_ISL_11323820, EPI_ISL_11323823, EPI_ISL_11323825, EPI_ISL_11323835			
see above	Institut Biologi Molekul Perubatan UKM (UMBI)	UKM Medical Molecular Biology Institute (UMBI)	Khairun Nur Abd Ghafar; Mira Farzana Mohamad Mokhtar; Muhiddin Ishak; Nor Azila Muhammad Azami; Nur Alyaa Afifah Md Shahri; Nurul Syakima Ab Mutalib; Rahman Jamal; Ryia Illani Mohd Yunos; Siti Nur Hasanah Mohd Yusuf; Zahirrah Begam Mohamed Rasheed
EPI_ISL_12534875	JABATAN FORENSIK HOSPITAL SULTANAH BAHIYAH	Tropical Infectious Diseases Research & Education Centre (TIDREC), Universiti Malaya	AsmaAnati CheMatSeri; Che-Norainon Yaacob; Jia-Yi Tan; Jo-Ern Wong; Kim-Kee Tan; Mulya-Mustika-Sari Zulkifli; Noor Syahida Azizan; Nur-Hidayana Mahfodz; Sazaly AbuBakar; Siti-Sarah Nor'e; Wei-Wei Aw
EPI_ISL_10969087, EPI_ISL_10969088, EPI_ISL_10969089, EPI_ISL_10969091, EPI_ISL_10969092, EPI_ISL_10969093, EPI_ISL_10969094, EPI_ISL_10969124, EPI_ISL_10969125, EPI_ISL_10969129, EPI_ISL_10969130, EPI_ISL_10969131, EPI_ISL_10969132			
see above	JABATAN PATOLOGI HOSPITAL RAJA Female ZAINAB II	Tropical Infectious Diseases Research & Education Centre (TIDREC), Universiti Malaya	AsmaAnati CheMatSeri; Che-Norainon Yaacob; Jia-Yi Tan; Jo-Ern Wong; Kim-Kee Tan; Mulya-Mustika-Sari Zulkifli; Noor Syahida Azizan; Nur-Hidayana Mahfodz; Sazaly AbuBakar; Siti-Sarah Nor'e
EPI_ISL_10969149	JABATAN PATOLOGI HOSPITAL RAJA PEREMPUAN ZAINAB II	Tropical Infectious Diseases Research & Education Centre (TIDREC), Universiti Malaya	AsmaAnati CheMatSeri; Che-Norainon Yaacob; Jia-Yi Tan; Jo-Ern Wong; Kim-Kee Tan; Mulya-Mustika-Sari Zulkifli; Noor Syahida Azizan; Nur-Hidayana Mahfodz; Sazaly AbuBakar; Siti-Sarah Nor'e
EPI_ISL_10969133	JABATAN PATOLOGI, HOSPITAL RAJA Female ZAINAB II	Tropical Infectious Diseases Research & Education Centre (TIDREC), Universiti Malaya	AsmaAnati CheMatSeri; Che-Norainon Yaacob; Jia-Yi Tan; Jo-Ern Wong; Kim-Kee Tan; Mulya-Mustika-Sari Zulkifli; Noor Syahida Azizan; Nur-Hidayana Mahfodz; Sazaly AbuBakar; Siti-Sarah Nor'e
EPI_ISL_9829850	JABATAN PATOLOGI, HOSPITAL RAJA PEREMPUAN ZAINAB II	Tropical Infectious Diseases Research & Education Centre (TIDREC), Universiti Malaya	AsmaAnati CheMatSeri; Che-Norainon Yaacob; Jia-Yi Tan; Jo-Ern Wong; Kim-Kee Tan; Mulya-Mustika-Sari Zulkifli; Noor Syahida Azizan; Nur-Hidayana Mahfodz; Sazaly AbuBakar; Siti-Sarah Nor'e
EPI_ISL_10969126	JABATAN PATOLOGI, HRPZ II	Tropical Infectious Diseases Research & Education Centre (TIDREC), Universiti Malaya	AsmaAnati CheMatSeri; Che-Norainon Yaacob; Jia-Yi Tan; Jo-Ern Wong; Kim-Kee Tan; Mulya-Mustika-Sari Zulkifli; Noor Syahida Azizan; Nur-Hidayana Mahfodz; Sazaly AbuBakar; Siti-Sarah Nor'e
EPI_ISL_9579049, EPI_ISL_9579162, EPI_ISL_9579163, EPI_ISL_9579164, EPI_ISL_9579165, EPI_ISL_9579166	JKN PAHANG	iPROMISE, UiTM	Ariza Adnan; Fadzilah Mohd Nor; Lim Wai Feng; Mohd Asif Mohd Sukri; Mohd Nur Fakhruzzaman Noorizhab; Mohd Zaki Salleh; Sazzli Shahlan Kassim; Siti Farah Alwani Mohd Nawi; Siti Hamimah Sheikh Abdul Kadir; Teh Lay Kek; Wang Seok Mui
EPI_ISL_10590750	Jabatan Kesihatan Negeri WP Labuan	Malaysia Genome and Vaccine Institute	Azrin Ahmad; Enizza Kasim; Irni Suhayu Sapian; Mohd Faizal Abu Bakar; Mohd Ghows Mohd Azzam.; Mohd Noor Mat Isa; Nurhezreen Md Iqbal; Shamsidar Sopie; Siti Noraini Othman; Yusuf Muhammad Noor
EPI_ISL_8745667, EPI_ISL_8745677, EPI_ISL_8745678	Jabatan Kesihatan WP Labuan	Malaysia Genome and Vaccine Institute	Azrin Ahmad; Enizza Kasim; Irni Suhayu Sapian; Mohd Faizal Abu Bakar; Mohd Ghows Mohd Azzam.; Mohd Noor Mat Isa; Nor Azfa Johari; Nurhezreen Md Iqbal; Shamsidar Sopie; Siti Noraini Othman; Yusuf Muhammad Noor
EPI_ISL_8560636, EPI_ISL_8560639, EPI_ISL_8560640, EPI_ISL_8560643, EPI_ISL_8560645, EPI_ISL_8560646, EPI_ISL_8560647, EPI_ISL_8560648, EPI_ISL_8560649, EPI_ISL_8560650, EPI_ISL_8560651, EPI_ISL_8560652, EPI_ISL_8560653, EPI_ISL_8560655, EPI_ISL_8560656, EPI_ISL_8560657, EPI_ISL_8560658, EPI_ISL_8560659, EPI_ISL_8560660, EPI_ISL_8560662, EPI_ISL_8560663, EPI_ISL_8560664, EPI_ISL_8560666, EPI_ISL_8560667, EPI_ISL_8560668, EPI_ISL_8560669, EPI_ISL_8560670, EPI_ISL_8560671, EPI_ISL_8560673, EPI_ISL_8560674, EPI_ISL_8560675, EPI_ISL_8560676, EPI_ISL_8560679, EPI_ISL_8560680, EPI_ISL_8560681, EPI_ISL_8560682, EPI_ISL_8560683, EPI_ISL_8560685, EPI_ISL_8560686, EPI_ISL_8560687, EPI_ISL_8560688, EPI_ISL_8560691, EPI_ISL_8560692, EPI_ISL_8560693, EPI_ISL_8560694, EPI_ISL_9832504			
see above	KLIA Health Office	Institute for Medical Research, Infectious Disease Research Centre, National Institutes of Health, Ministry of Health Malaysia	Ahmad FA; Ahmad Fazilah NA; Anasir MI; Azizan MA; Kamel K; Mohamad Sukri MZ; Norhisham SN; Ramly N; Robert F; Rosli NR; Suppiah J; Thayan R
EPI_ISL_10969005	KLINIK KESIHATAN PASIR PUTEH (BILIK GERAKAN PASIR PUTEH)	Tropical Infectious Diseases Research & Education Centre (TIDREC), Universiti Malaya	AsmaAnati CheMatSeri; Che-Norainon Yaacob; Jia-Yi Tan; Jo-Ern Wong; Kim-Kee Tan; Mulya-Mustika-Sari Zulkifli; Noor Syahida Azizan; Nur-Hidayana Mahfodz; Sazaly AbuBakar; Siti-Sarah Nor'e
EPI_ISL_10652471	KOMPLEKS SURVELAN PKD KINTA	Department of Medical Microbiology, Faculty of Medicine, University of Malaya; University of Malaya Medical Centre	I-Ching SAM; Jolene Yin Ling FU; Omar Khalilur Rahman; Yoke Fun Chan
EPI_ISL_10495846	KUALA LUMPUR HOSPITAL	Institute for Medical Research, Infectious Disease Research Centre, National Institutes of Health, Ministry of Health Malaysia	Ahmad FA; Ahmad Fazilah NA; Anasir MI; Arip MS; Azizan MA; Christopher Hollip K; Jaimin JJ; Kamel K; Mohamad Sukri MZ; Mohammad R; Mohd Yasin R; Norhisham SN; Ramly N; Robert F; Rosli NR; Shamsaddin E; Suppiah J; Tan YC; Thayan R
EPI_ISL_8560677, EPI_ISL_8560684	Kampung Bandar Health Clinic	Institute for Medical Research, Infectious Disease Research Centre, National Institutes of Health, Ministry of Health Malaysia	Ahmad FA; Ahmad Fazilah NA; Anasir MI; Azizan MA; Kamel K; Mohamad Sukri MZ; Norhisham SN; Ramly N; Robert F; Rosli NR; Suppiah J; Thayan R
EPI_ISL_8560061, EPI_ISL_8560068, EPI_ISL_8560075, EPI_ISL_8560090, EPI_ISL_8560940, EPI_ISL_8560941, EPI_ISL_8560942, EPI_ISL_8560943, EPI_ISL_8560944, EPI_ISL_8560945, EPI_ISL_8560948, EPI_ISL_8560950, EPI_ISL_8560951, EPI_ISL_8560952, EPI_ISL_8560953, EPI_ISL_8560956, EPI_ISL_8560958, EPI_ISL_8560959, EPI_ISL_8560960, EPI_ISL_8560961, EPI_ISL_8560964, EPI_ISL_8560966, EPI_ISL_8560968, EPI_ISL_8560969, EPI_ISL_8560972, EPI_ISL_8560974, EPI_ISL_8560976, EPI_ISL_8560977, EPI_ISL_8560980, EPI_ISL_8560981, EPI_ISL_8560983, EPI_ISL_8560986, EPI_ISL_8560987, EPI_ISL_8560988, EPI_ISL_8560989, EPI_ISL_8560990, EPI_ISL_8560991, EPI_ISL_8560994, EPI_ISL_8560996, EPI_ISL_8560997, EPI_ISL_8560999, EPI_ISL_8561000, EPI_ISL_8561003, EPI_ISL_8561004, EPI_ISL_8561006, EPI_ISL_8561007, EPI_ISL_8561010, EPI_ISL_8561011, EPI_ISL_8561013, EPI_ISL_8561016, EPI_ISL_8561017, EPI_ISL_8561018, EPI_ISL_8561019, EPI_ISL_8561020, EPI_ISL_8561022, EPI_ISL_8561023, EPI_ISL_8561024, EPI_ISL_8561025, EPI_ISL_8561026, EPI_ISL_8561027, EPI_ISL_8692425, EPI_ISL_8692451, EPI_ISL_8692457, EPI_ISL_8692482, EPI_ISL_9832501, EPI_ISL_9832502			
see above	Kuala Lumpur International Airport (KLIA) Health Office	Institute for Medical Research, Infectious Disease Research Centre, National Institutes of Health, Ministry of Health Malaysia	Ahmad FA; Ahmad Fazilah NA; Anasir MI; Azizan MA; Kamel K; Mohamad Sukri MZ; Norhisham SN; Ramly N; Robert F; Rosli NR; Suppiah J; Thayan R
EPI_ISL_8560637	Lembah Pantai District Health Office	Institute for Medical Research, Infectious Disease Research Centre, National Institutes of Health, Ministry of Health Malaysia	Ahmad FA; Ahmad Fazilah NA; Anasir MI; Azizan MA; Kamel K; Mohamad Sukri MZ; Norhisham SN; Ramly N; Robert F; Rosli NR; Suppiah J; Thayan R
EPI_ISL_10969002, EPI_ISL_10969102, EPI_ISL_10969103, EPI_ISL_10969104, EPI_ISL_10969105, EPI_ISL_10969106, EPI_ISL_10969107, EPI_ISL_10969108, EPI_ISL_10969138, EPI_ISL_10969139, EPI_ISL_10969152, EPI_ISL_11010513, EPI_ISL_11010514			
see above	MKA KB	Tropical Infectious Diseases Research & Education Centre (TIDREC), Universiti Malaya	AsmaAnati CheMatSeri; Che-Norainon Yaacob; Jia-Yi Tan; Jo-Ern Wong; Kim-Kee Tan; Mulya-Mustika-Sari Zulkifli; Noor Syahida Azizan; Nur-Hidayana Mahfodz; Sazaly AbuBakar; Siti-Sarah Nor'e
EPI_ISL_9579033	Medical Hospital Pulau Pinang	iPROMISE, UiTM	Ariza Adnan; Fadzilah Mohd Nor; Lim Wai Feng; Mohd Asif Mohd Sukri; Mohd Nur Fakhruzzaman Noorizhab; Mohd Zaki Salleh; Sazzli Shahlan Kassim; Siti Farah Alwani Mohd Nawi; Siti Hamimah Sheikh Abdul Kadir; Teh Lay Kek; Wang Seok Mui
EPI_ISL_8650063, EPI_ISL_8650130	Miri Hospital Molecular Diagnostic Lab, Miri	Institute of Health and Community Medicine	Chan Chia Jui; Chua Hock Hin; David Perera; Ooi Mong How; Tonnii Sia Loong Loong; Wong Jyn Shan
EPI_ISL_10968984	PEJABAT KESIHATAN DAERAH LANGKAWI	Tropical Infectious Diseases Research & Education Centre (TIDREC), Universiti Malaya	AsmaAnati CheMatSeri; Che-Norainon Yaacob; Jia-Yi Tan; Jo-Ern Wong; Kim-Kee Tan; Mulya-Mustika-Sari Zulkifli; Noor Syahida Azizan; Nur-Hidayana Mahfodz; Sazaly AbuBakar; Siti-Sarah Nor'e
EPI_ISL_10652469	PEJABAT KESIHATAN DAERAH MUALLIM	Department of Medical Microbiology, Faculty of Medicine, University of Malaya; University of Malaya Medical Centre	I-Ching SAM; Jolene Yin Ling FU; Omar Khalilur Rahman; Yoke Fun Chan
EPI_ISL_10652491	PEJABAT KESIHATAN DAERAH PASIR MAS	Department of Medical Microbiology, Faculty of Medicine, University of Malaya; University of Malaya Medical Centre	I-Ching SAM; Jolene Yin Ling FU; Omar Khalilur Rahman; Yoke Fun Chan
EPI_ISL_9579145, EPI_ISL_9579146, EPI_ISL_9579147, EPI_ISL_9579148, EPI_ISL_9579149, EPI_ISL_9579150, EPI_ISL_9579151, EPI_ISL_9579152, EPI_ISL_9579153, EPI_ISL_9579154, EPI_ISL_9579155, EPI_ISL_9579156, EPI_ISL_9579157, EPI_ISL_11112441			

see above	PEJABAT KESIHATAN DAERAH SEBERANG PERAI UTARA PULAU PINANG	iPROMISE, UiTM	Ariza Adnan; Fadzilah Mohd Nor; Lim Wai Feng; Mohd Asif Mohd Sukri; Mohd Nur Fakhruzzaman Noorizhab; Mohd Zaki Salleh; Sazzli Shahlan Kassim; Siti Farah Alwani Mohd Nawi; Siti Hamimah Sheikh Abdul Kadir; Teh Lay Kek; Wang Seok Mui
EPI_ISL_9829851	PKD BARAT DAYA	Tropical Infectious Diseases Research & Education Centre (TIDREC), Universiti Malaya	AsmaAnati CheMatSeri; Che-Norainon Yaacob; Jia-Yi Tan; Jo-Ern Wong; Kim-Kee Tan; Mulya-Mustika-Sari Zulkifli; Noor Syahida Azizan; Nur-Hidayana Mahfodz; Sazaly AbuBakar; Siti-Sarah Nor'e
EPI_ISL_11754744	PKD GUA MUSANG	Department of Medical Microbiology, Faculty of Medicine, University of Malaya; University of Malaya Medical Centre	I-Ching SAM; Jolene Yin Ling FU; Omar Khalilur Rahman; Yoke Fun Chan
EPI_ISL_8380530, EPI_ISL_8380546, EPI_ISL_8405652	PKD JEMPOL	UKM Medical Molecular Biology Institute (UMBI)	Khairun Nur Abd Ghafar; Mira Farzana Mohamad Mokhtar; Muhiddin Ishak; Nor Azila Muhammad Azami; Nur Alyaa Afifah Md Shahri; Nurul Syakima Ab Mutalib; Rahman Jamal; Ryia Illani Mohd Yunos; Siti Nur Hasanah Mohd Yusuf; Zahirrah Begam Mohamed Rasheed
EPI_ISL_8380539, EPI_ISL_9317744, EPI_ISL_9829378, EPI_ISL_9829382	PKD JOHOR BAHRU	UKM Medical Molecular Biology Institute (UMBI)	Khairun Nur Abd Ghafar; Mira Farzana Mohamad Mokhtar; Muhiddin Ishak; Nor Azila Muhammad Azami; Nur Alyaa Afifah Md Shahri; Nurul Syakima Ab Mutalib; Rahman Jamal; Ryia Illani Mohd Yunos; Siti Nur Hasanah Mohd Yusuf; Zahirrah Begam Mohamed Rasheed
EPI_ISL_10969148	PKD KERIAN	Tropical Infectious Diseases Research & Education Centre (TIDREC), Universiti Malaya	AsmaAnati CheMatSeri; Che-Norainon Yaacob; Jia-Yi Tan; Jo-Ern Wong; Kim-Kee Tan; Mulya-Mustika-Sari Zulkifli; Noor Syahida Azizan; Nur-Hidayana Mahfodz; Sazaly AbuBakar; Siti-Sarah Nor'e
EPI_ISL_9317745	PKD KLUANG	UKM Medical Molecular Biology Institute (UMBI)	Khairun Nur Abd Ghafar; Mira Farzana Mohamad Mokhtar; Muhiddin Ishak; Nor Azila Muhammad Azami; Nur Alyaa Afifah Md Shahri; Nurul Syakima Ab Mutalib; Rahman Jamal; Ryia Illani Mohd Yunos; Siti Nur Hasanah Mohd Yusuf; Zahirrah Begam Mohamed Rasheed
EPI_ISL_9829849	PKD KULAI	Tropical Infectious Diseases Research & Education Centre (TIDREC), Universiti Malaya	AsmaAnati CheMatSeri; Che-Norainon Yaacob; Jia-Yi Tan; Jo-Ern Wong; Kim-Kee Tan; Mulya-Mustika-Sari Zulkifli; Noor Syahida Azizan; Nur-Hidayana Mahfodz; Sazaly AbuBakar; Siti-Sarah Nor'e
EPI_ISL_9829848	PKD MELAKA TENGAH	Tropical Infectious Diseases Research & Education Centre (TIDREC), Universiti Malaya	AsmaAnati CheMatSeri; Che-Norainon Yaacob; Jia-Yi Tan; Jo-Ern Wong; Kim-Kee Tan; Mulya-Mustika-Sari Zulkifli; Noor Syahida Azizan; Nur-Hidayana Mahfodz; Sazaly AbuBakar; Siti-Sarah Nor'e
EPI_ISL_8405631, EPI_ISL_8405640, EPI_ISL_8405645, EPI_ISL_8405653, EPI_ISL_9829362	PKD MELAKA TENGAH	UKM Medical Molecular Biology Institute (UMBI)	Khairun Nur Abd Ghafar; Mira Farzana Mohamad Mokhtar; Muhiddin Ishak; Nor Azila Muhammad Azami; Nur Alyaa Afifah Md Shahri; Nurul Syakima Ab Mutalib; Rahman Jamal; Ryia Illani Mohd Yunos; Siti Nur Hasanah Mohd Yusuf; Zahirrah Begam Mohamed Rasheed
EPI_ISL_10652470	PKD PERAK TENGAH	Department of Medical Microbiology, Faculty of Medicine, University of Malaya; University of Malaya Medical Centre	I-Ching SAM; Jolene Yin Ling FU; Omar Khalilur Rahman; Yoke Fun Chan
EPI_ISL_9317741, EPI_ISL_9317750, EPI_ISL_9317755	PKD SEGAMAT	UKM Medical Molecular Biology Institute (UMBI)	Khairun Nur Abd Ghafar; Mira Farzana Mohamad Mokhtar; Muhiddin Ishak; Nor Azila Muhammad Azami; Nur Alyaa Afifah Md Shahri; Nurul Syakima Ab Mutalib; Rahman Jamal; Ryia Illani Mohd Yunos; Siti Nur Hasanah Mohd Yusuf; Zahirrah Begam Mohamed Rasheed
EPI_ISL_9317737	PKD SEREMBAN	UKM Medical Molecular Biology Institute (UMBI)	Khairun Nur Abd Ghafar; Mira Farzana Mohamad Mokhtar; Muhiddin Ishak; Nor Azila Muhammad Azami; Nur Alyaa Afifah Md Shahri; Nurul Syakima Ab Mutalib; Rahman Jamal; Ryia Illani Mohd Yunos; Siti Nur Hasanah Mohd Yusuf; Zahirrah Begam Mohamed Rasheed
EPI_ISL_9579120, EPI_ISL_9579121	PKD SPT	iPROMISE, UiTM	Ariza Adnan; Fadzilah Mohd Nor; Lim Wai Feng; Mohd Asif Mohd Sukri; Mohd Nur Fakhruzzaman Noorizhab; Mohd Zaki Salleh; Sazzli Shahlan Kassim; Siti Farah Alwani Mohd Nawi; Siti Hamimah Sheikh Abdul Kadir; Teh Lay Kek; Wang Seok Mui
EPI_ISL_9317716, EPI_ISL_9317722, EPI_ISL_9317725, EPI_ISL_9317734, EPI_ISL_9317757, EPI_ISL_10397077, EPI_ISL_10397094			
see above	PKD TAMPIN	UKM Medical Molecular Biology Institute (UMBI)	Khairun Nur Abd Ghafar; Mira Farzana Mohamad Mokhtar; Muhiddin Ishak; Nor Azila Muhammad Azami; Nur Alyaa Afifah Md Shahri; Nurul Syakima Ab Mutalib; Rahman Jamal; Ryia Illani Mohd Yunos; Siti Nur Hasanah Mohd Yusuf; Zahirrah Begam Mohamed Rasheed
EPI_ISL_8405635, EPI_ISL_8405639, EPI_ISL_8405650, EPI_ISL_10397105	PKD TANGKAK	UKM Medical Molecular Biology Institute (UMBI)	Khairun Nur Abd Ghafar; Mira Farzana Mohamad Mokhtar; Muhiddin Ishak; Nor Azila Muhammad Azami; Nur Alyaa Afifah Md Shahri; Nurul Syakima Ab Mutalib; Rahman Jamal; Ryia Illani Mohd Yunos; Siti Nur Hasanah Mohd Yusuf; Zahirrah Begam Mohamed Rasheed
EPI_ISL_9579119, EPI_ISL_9579122, EPI_ISL_9579123	PKDSPT	iPROMISE, UiTM	Ariza Adnan; Fadzilah Mohd Nor; Lim Wai Feng; Mohd Asif Mohd Sukri; Mohd Nur Fakhruzzaman Noorizhab; Mohd Zaki Salleh; Sazzli Shahlan Kassim; Siti Farah Alwani Mohd Nawi; Siti Hamimah Sheikh Abdul Kadir; Teh Lay Kek; Wang Seok Mui
EPI_ISL_8745672	Pejabat Kesihatan Daerah Papar	Malaysia Genome and Vaccine Institute	Azrin Ahmad; Enizza Kasim; Irni Suhayu Sapian; Mohd Faizal Abu Bakar; Mohd Ghows Mohd Azzam.; Mohd Noor Mat Isa; Nor Azfa Johari; Nurhezreen Md Iqbal; Shamsidar Sopie; Siti Noraini Othman; Yusuf Muhammad Noor
EPI_ISL_9579133, EPI_ISL_9579134, EPI_ISL_9579135, EPI_ISL_9579136, EPI_ISL_9579137, EPI_ISL_9579138, EPI_ISL_9579139, EPI_ISL_9579140, EPI_ISL_9579141, EPI_ISL_9579142, EPI_ISL_9579143, EPI_ISL_9579144, EPI_ISL_9579158, EPI_ISL_9579200, EPI_ISL_11112410			
see above	Pejabat Kesihatan Daerah Timur Laut	iPROMISE, UiTM	Ariza Adnan; Fadzilah Mohd Nor; Lim Wai Feng; Mohd Asif Mohd Sukri; Mohd Nur Fakhruzzaman Noorizhab; Mohd Zaki Salleh; Sazzli Shahlan Kassim; Siti Farah Alwani Mohd Nawi; Siti Hamimah Sheikh Abdul Kadir; Teh Lay Kek; Wang Seok Mui
EPI_ISL_8745671, EPI_ISL_8745673	Pejabat Kesihatan Kawasan Beaufort	Malaysia Genome and Vaccine Institute	Azrin Ahmad; Enizza Kasim; Irni Suhayu Sapian; Mohd Faizal Abu Bakar; Mohd Ghows Mohd Azzam.; Mohd Noor Mat Isa; Nor Azfa Johari; Nurhezreen Md Iqbal; Shamsidar Sopie; Siti Noraini Othman; Yusuf Muhammad Noor
EPI_ISL_8745668	Pejabat Kesihatan Kawasan Keningau	Malaysia Genome and Vaccine Institute	Azrin Ahmad; Enizza Kasim; Irni Suhayu Sapian; Mohd Faizal Abu Bakar; Mohd Ghows Mohd Azzam.; Mohd Noor Mat Isa; Nor Azfa Johari; Nurhezreen Md Iqbal; Shamsidar Sopie; Siti Noraini Othman; Yusuf Muhammad Noor
EPI_ISL_10590755	Pejabat Kesihatan Kawasan Lahad Datu	Malaysia Genome and Vaccine Institute	Azrin Ahmad; Enizza Kasim; Irni Suhayu Sapian; Mohd Faizal Abu Bakar; Mohd Ghows Mohd Azzam.; Mohd Noor Mat Isa; Nurhezreen Md Iqbal; Shamsidar Sopie; Siti Noraini Othman; Yusuf Muhammad Noor
EPI_ISL_8745669	Pejabat Kesihatan Kawasan Tuaran	Malaysia Genome and Vaccine Institute	Azrin Ahmad; Enizza Kasim; Irni Suhayu Sapian; Mohd Faizal Abu Bakar; Mohd Ghows Mohd Azzam.; Mohd Noor Mat Isa; Nor Azfa Johari; Nurhezreen Md Iqbal; Shamsidar Sopie; Siti Noraini Othman; Yusuf Muhammad Noor
EPI_ISL_10590756, EPI_ISL_10590757, EPI_ISL_10590758, EPI_ISL_10590759, EPI_ISL_10590760	Pejabat Kesihatan Lembah Pantai	Malaysia Genome and Vaccine Institute	Azrin Ahmad; Enizza Kasim; Irni Suhayu Sapian; Mohd Faizal Abu Bakar; Mohd Ghows Mohd Azzam.; Mohd Noor Mat Isa; Nurhezreen Md Iqbal; Shamsidar Sopie; Siti Noraini Othman; Yusuf Muhammad Noor
EPI_ISL_9579007	Pejabat Kesihatan Pintu Masuk Antarabangsa	iPROMISE, UiTM	Ariza Adnan; Fadzilah Mohd Nor; Lim Wai Feng; Mohd Asif Mohd Sukri; Mohd Nur Fakhruzzaman Noorizhab; Mohd Zaki Salleh; Sazzli Shahlan Kassim; Siti Farah Alwani Mohd Nawi; Siti Hamimah Sheikh Abdul Kadir; Teh Lay Kek; Wang Seok Mui
EPI_ISL_8745675	Pejabat Kesihtan Daerah Papar	Malaysia Genome and Vaccine Institute	Azrin Ahmad; Enizza Kasim; Irni Suhayu Sapian; Mohd Faizal Abu Bakar; Mohd Ghows Mohd Azzam.; Mohd Noor Mat Isa; Nor Azfa Johari; Nurhezreen Md Iqbal; Shamsidar Sopie; Siti Noraini Othman; Yusuf Muhammad Noor
EPI_ISL_10495840, EPI_ISL_10495848	QUEEN ELIZABETH HOSPITAL	Institute for Medical Research, Infectious Disease Research Centre, National Institutes of Health, Ministry of Health Malaysia	Ahmad FA; Ahmad Fazilah NA; Anasir MI; Arip MS; Azizan MA; Christopher Hollip K; Jaimin JJ; Kamel K; Mohamad Sukri MZ; Mohammad R; Mohd Yasin R; Norhisham SN; Ramly N; Robert F; Rosli NR; Shamsaddin E; Suppiah J; Tan YC; Thayan R
EPI_ISL_10495839, EPI_ISL_10495845, EPI_ISL_10495847, EPI_ISL_10495853, EPI_ISL_10495855, EPI_ISL_10495860, EPI_ISL_10495862, EPI_ISL_10495868, EPI_ISL_10495869, EPI_ISL_10495870, EPI_ISL_10495876, EPI_ISL_10495877, EPI_ISL_10495878			
see above	RAJA PEREMPUAN ZAINAB II HOSPITAL	Institute for Medical Research, Infectious Disease Research Centre, National Institutes of Health, Ministry of Health Malaysia	Ahmad FA; Ahmad Fazilah NA; Anasir MI; Arip MS; Azizan MA; Christopher Hollip K; Jaimin JJ; Kamel K; Mohamad Sukri MZ; Mohammad R; Mohd Yasin R; Norhisham SN; Ramly N; Robert F; Rosli NR; Shamsaddin E; Suppiah J; Tan YC; Thayan R
EPI_ISL_8188865, EPI_ISL_8188866, EPI_ISL_8188867, EPI_ISL_8188868, EPI_ISL_8188869, EPI_ISL_8650120	Rejang Medical Centre (Sibu)	Institute of Health and Community Medicine	Chan Chia Jui; Chua Hock Hin; David Perera; Ooi Mong How; Tonnii Sia Loong Loong; Wong Jyn Shan
EPI_ISL_9829349	SWAT TEAM	UKM Medical Molecular Biology Institute (UMBI)	Khairun Nur Abd Ghafar; Mira Farzana Mohamad Mokhtar; Muhiddin Ishak; Nor Azila Muhammad Azami; Nur Alyaa Afifah Md Shahri; Nurul Syakima Ab Mutalib; Rahman Jamal; Ryia Illani Mohd Yunos; Siti Nur Hasanah Mohd Yusuf; Zahirrah Begam Mohamed Rasheed
EPI_ISL_10297333	Sabah Women and Children's Hospital	Institute for Medical Research, Infectious Disease Research Centre, National Institutes of Health, Ministry of Health Malaysia	Ahmad FA; Ahmad Fazilah NA; Anasir MI; Azizan MA; Kamel K; Mohamad Sukri MZ; Norhisham SN; Ramly N; Robert F; Rosli NR; Suppiah J; Thayan R
EPI_ISL_8188826, EPI_ISL_8188827, EPI_ISL_8188844, EPI_ISL_8188845, EPI_ISL_8188846, EPI_ISL_8188847, EPI_ISL_8650060, EPI_ISL_8650061, EPI_ISL_8650067, EPI_ISL_8650068, EPI_ISL_8650075, EPI_ISL_8650076, EPI_ISL_8650077, EPI_ISL_8650078, EPI_ISL_8650079, EPI_ISL_8650080, EPI_ISL_8650081, EPI_ISL_8650082, EPI_ISL_8650083, EPI_ISL_8650084, EPI_ISL_8650085, EPI_ISL_8650086			
see above	Sarawak General Hospital (Kuching)	Institute of Health and Community Medicine	Chan Chia Jui; Chua Hock Hin; David Perera; Ooi Mong How; Tonnii Sia Loong Loong; Wong Jyn Shan
EPI_ISL_8560689	Telok Datok Health Clinic	Institute for Medical Research, Infectious Disease Research Centre, National Institutes of Health, Ministry of Health Malaysia	Ahmad FA; Ahmad Fazilah NA; Anasir MI; Azizan MA; Kamel K; Mohamad Sukri MZ; Norhisham SN; Ramly N; Robert F; Rosli NR; Suppiah J; Thayan R
EPI_ISL_8295902, EPI_ISL_8295903, EPI_ISL_8295904, EPI_ISL_8295905	Tropical Infectious Diseases Research & Education Centre (TIDREC), Universiti Malaya	Tropical Infectious Diseases Research & Education Centre (TIDREC), Universiti Malaya	AsmaAnati CheMatSeri; Che-Norainon Yaacob; Jia-Yi Tan; Jo-Ern Wong; Kim-Kee Tan; Mulya-Mustika-Sari Zulkifli; Noor Syahida Azizan; Nur-Hidayana Mahfodz; Sazaly AbuBakar; Siti-Sarah Nor'e
EPI_ISL_8767321, EPI_ISL_8767322, EPI_ISL_8767323, EPI_ISL_8767324	UMMC	Department of Medical Microbiology, Faculty of Medicine, University of Malaya;	I-Ching SAM; Jolene Yin Ling FU; Omar Khalilur Rahman; Yoke Fun Chan

		University of Malaya Medical Centre	
EPI_ISL_9829355, EPI_ISL_9829361, EPI_ISL_9829379	UNIT KESIHATAN AWAM HEBHK	UKM Medical Molecular Biology Institute (UMBI)	Khairun Nur Abd Ghafar; Mira Farzana Mohamad Mokhtar; Muhiddin Ishak; Nor Azila Muhammad Azami; Nur Alyaa Afifah Md Shahri; Nurul Syakima Ab Mutalib; Rahman Jamal; Ryia Illani Mohd Yunos; Siti Nur Hasanah Mohd Yusuf; Zahirrah Begam Mohamed Rasheed
EPI_ISL_9579048	Unit Survelan	iPROMISE, UiTM	Ariza Adnan; Fadzilah Mohd Nor; Lim Wai Feng; Mohd Asif Mohd Sukri; Mohd Nur Fakhruzzaman Noorizhab; Mohd Zaki Salleh; Sazzli Shahlan Kassim; Siti Farah Alwani Mohd Nawi; Siti Hamimah Sheikh Abdul Kadir; Teh Lay Kek; Wang Seok Mui
EPI_ISL_9579045, EPI_ISL_9579046	Unit Survelan JKN	iPROMISE, UiTM	Ariza Adnan; Fadzilah Mohd Nor; Lim Wai Feng; Mohd Asif Mohd Sukri; Mohd Nur Fakhruzzaman Noorizhab; Mohd Zaki Salleh; Sazzli Shahlan Kassim; Siti Farah Alwani Mohd Nawi; Siti Hamimah Sheikh Abdul Kadir; Teh Lay Kek; Wang Seok Mui
EPI_ISL_9579047	Unit Survelan, Jabatan Kesihatan Negeri	iPROMISE, UiTM	Ariza Adnan; Fadzilah Mohd Nor; Lim Wai Feng; Mohd Asif Mohd Sukri; Mohd Nur Fakhruzzaman Noorizhab; Mohd Zaki Salleh; Sazzli Shahlan Kassim; Siti Farah Alwani Mohd Nawi; Siti Hamimah Sheikh Abdul Kadir; Teh Lay Kek; Wang Seok Mui
EPI_ISL_9829365	Unit mikrobiologi, Hospital Sultanah Aminah	UKM Medical Molecular Biology Institute (UMBI)	Khairun Nur Abd Ghafar; Mira Farzana Mohamad Mokhtar; Muhiddin Ishak; Nor Azila Muhammad Azami; Nur Alyaa Afifah Md Shahri; Nurul Syakima Ab Mutalib; Rahman Jamal; Ryia Illani Mohd Yunos; Siti Nur Hasanah Mohd Yusuf; Zahirrah Begam Mohamed Rasheed
EPI_ISL_9579030, EPI_ISL_9579038	Ward C20 HPP	iPROMISE, UiTM	Ariza Adnan; Fadzilah Mohd Nor; Lim Wai Feng; Mohd Asif Mohd Sukri; Mohd Nur Fakhruzzaman Noorizhab; Mohd Zaki Salleh; Sazzli Shahlan Kassim; Siti Farah Alwani Mohd Nawi; Siti Hamimah Sheikh Abdul Kadir; Teh Lay Kek; Wang Seok Mui
EPI_ISL_8560939, EPI_ISL_8560947, EPI_ISL_8560955, EPI_ISL_8560963	Yan District Health Office	Institute for Medical Research, Infectious Disease Research Centre, National Institutes of Health, Ministry of Health Malaysia	Ahmad FA; Ahmad Fazilah NA; Anasir MI; Azizan MA; Kamel K; Mohamad Sukri MZ; Norhisham SN; Ramly N; Robert F; Rosli NR; Suppiah J; Thayan R
EPI_ISL_12534824	hrpz II	Tropical Infectious Diseases Research & Education Centre (TIDREC), Universiti Malaya	AsmaAnati CheMatSeri; Che-Norainon Yaacob; Jia-Yi Tan; Jo-Ern Wong; Kim-Kee Tan; Mulya-Mustika-Sari Zulkifli; Noor Syahida Azizan; Nur-Hidayana Mahfodz; Sazaly AbuBakar; Siti-Sarah Nor'e; Wei-Wei Aw

We gratefully acknowledge the following Authors from the Originating laboratories responsible for obtaining the specimens, as well as the Submitting laboratories where the genome data were generated and shared via GISAID, on which this research is based.

All Submitters of data may be contacted directly via www.gisaid.org

Authors are sorted alphabetically.

Accession ID	Originating Laboratory	Submitting Laboratory	Authors
EPI_ISL_9829373	COVID-Hospital Melaka	UKM Medical Molecular Biology Institute (UMBI)	Khairun Nur Abd Ghafar; Mira Farzana Mohamad Mokhtar; Muhiddin Ishak; Nor Azila Muhammad Azami; Nur Alyaa Afifah Md Shahri; Nurul Syakima Ab Mutalib; Rahman Jamal; Ryia Illani Mohd Yunos; Siti Nur Hasanah Mohd Yusuf; Zahirrah Begam Mohamed Rasheed
EPI_ISL_8564947, EPI_ISL_10431129, EPI_ISL_10431133, EPI_ISL_10431134, EPI_ISL_10431145	Department of Medical Microbiology and Parasitology, Hospital Universiti Sains Malaysia	Molecular Research Laboratory	Abdul Haris bin Muhammad; Ahmad Adebayo Irekeola; Ahmad Sukari Bin Halim; Alexander Chong Shu Chien; Aswini Leela; Azian Harun; Chan Yean Yean; Chua Wei Chuan; Chua Wei Lian; Engku Nur Syafirah bt Engku Abd Rahman; Farahana binti Mohamed; Farahana binti Mohamed Alexander Chong Shu Chien; Kirnpal Kaur Banga Singh; Lau Nyok Sean; Lee Lih Huey; Lim Shu Yong; Maizun binti Mohd Zain; Muhammad Azamuddeen bin Mohammad Nasir; Muhammad Fazli bin Khalid; Muhammad Nashrul Farhan Samsudin; Muhammad Zarul Hanifah Bin Md Zoqratt; Naveed Ahmed; Nazmi Liana Binti Azmi; Noor Hafizan binti Mat Salleh; Nurfadhlina Musa; Qasim Ayub; Rosline Hassan; Sadequr Rahman; Syahida binti Omar; Wan Mohd Zahiruddin Wan Mohammad; Wardah Yusof; Wilhelm Eng Wei Han; Zaini bin Hussin; Zakuan Zainy Deris
EPI_ISL_8646708, EPI_ISL_8646715	Department of Medical Microbiology and Parasitology, University of Science Malaysia Hospital	Molecular Research Laboratory, Department of Medical Microbiology and Parasitology, University of Science Health Campus	Abdul Haris bin Muhammad; Ahmad Adebayo Irekeola; Ahmad Sukari Bin Halim; Alexander Chong Shu Chien; Aswini Leela; Azian Harun; Chan Yean Yean; Chua Wei Chuan; Chua Wei Lian; Engku Nur Syafirah bt Engku Abd Rahman; Farahana binti Mohamed; Kirnpal Kaur Banga Singh; Lau Nyok Sean; Lee Lih Huey; Lim Shu Yong; Maizun binti Mohd Zain; Muhammad Azamuddeen bin Mohammad Nasir; Muhammad Fazli bin Khalid; Muhammad Nashrul Farhan Samsudin; Muhammad Zarul Hanifah Bin Md Zoqratt; Naveed Ahmed; Nazmi Liana Binti Azmi; Noor Hafizan binti Mat Salleh; Nurfadhlina Musa; Qasim Ayub; Rosline Hassan; Sadequr Rahman; Syahida binti Omar; Wan Mohd Zahiruddin Wan Mohammad; Wardah Yusof; Wilhelm Eng Wei Han; Zaini bin Hussin; Zakuan Zainy Deris
EPI_ISL_10073121, EPI_ISL_10073167, EPI_ISL_10073250	Department of Medical Microbiology, Hospital Pengajar Universiti Putra Malaysia	Department of Medical Microbiology, Hospital Pengajar Universiti Putra Malaysia	Afiqah Adzmi; Amiza Azmi; Azmiza Syawani Jasni; Hui Yee Chee; Leslie Thian Lung Than; Muadz Mohtar; Muhammad Mohd Isa; Narcisse MS Joseph; Niazlin Mohd Taib; Noor Hazirah Noor Azhari; Norlaila; Nur Raihana Ithnin; Nurul Huda Mohamed Rashidi; Nurul Nadiah Ismail; Rosni Ibrahim; Rukman Awang Hamat; Sallehhudin; Siti Norbaya Masri; Siti Zulaikha Zakariah; Syafinaz Amin Nordin; Tengku Zetty Maztura Tengku Jamaluddin; Zamberi Sekawi
EPI_ISL_8650062, EPI_ISL_8650092, EPI_ISL_8650098, EPI_ISL_8650099, EPI_ISL_8650100, EPI_ISL_8650101, EPI_ISL_8650102, EPI_ISL_8650141			
see above	Gribbles Pathology, Kuching	Institute of Health and Community Medicine	Chan Chia Jui; Chua Hock Hin; David Perera; Liew Chin Chin J; Ooi Mong How; Tonnii Sia Loong Loong; Wong Jyn Shan
EPI_ISL_9317712	HOSPITAL MELAKA	UKM Medical Molecular Biology Institute (UMBI)	Khairun Nur Abd Ghafar; Mira Farzana Mohamad Mokhtar; Muhiddin Ishak; Nor Azila Muhammad Azami; Nur Alyaa Afifah Md Shahri; Nurul Syakima Ab Mutalib; Rahman Jamal; Ryia Illani Mohd Yunos; Siti Nur Hasanah Mohd Yusuf; Zahirrah Begam Mohamed Rasheed
EPI_ISL_10397067	HOSPITAL PAKAR SULTANAH FATIMAH MUAR	UKM Medical Molecular Biology Institute (UMBI)	Khairun Nur Abd Ghafar; Mira Farzana Mohamad Mokhtar; Muhiddin Ishak; Nor Azila Muhammad Azami; Nur Alyaa Afifah Md Shahri; Nurul Syakima Ab Mutalib; Rahman Jamal; Ryia Illani Mohd Yunos; Siti Nur Hasanah Mohd Yusuf; Zahirrah Begam Mohamed Rasheed
EPI_ISL_9579115, EPI_ISL_9579182	HOSPITAL PULAU PINANG	iPROMISE, UiTM	Ariza Adnan; Fadzilah Mohd Nor; Lim Wai Feng; Mohd Asif Mohd Sukri; Mohd Nur Fakhruzzaman Noorizhab; Mohd Zaki Salleh; Sazzli Shahlan Kassim; Siti Farah Alwani Mohd Nawi; Siti Hamimah Sheikh Abdul Kadir; Teh Lay Kek; Wang Seok Mui
EPI_ISL_10968983	HOSPITAL SULTANAH MALIHA	Tropical Infectious Diseases Research & Education Centre (TIDREC), Universiti Malaya	AsmaAnati CheMatSeri; Che-Norainon Yaacob; Jia-Yi Tan; Jo-Ern Wong; Kim-Kee Tan; Mulya-Mustika-Sari Zulkifli; Noor Syahida Azizan; Nur-Hidayana Mahfodz; Sazaly AbuBakar; Siti-Sarah Nor'e
EPI_ISL_9317739	HOSPITAL SULTANAH NORA ISMAIL	UKM Medical Molecular Biology Institute (UMBI)	Khairun Nur Abd Ghafar; Mira Farzana Mohamad Mokhtar; Muhiddin Ishak; Nor Azila Muhammad Azami; Nur Alyaa Afifah Md Shahri; Nurul Syakima Ab Mutalib; Rahman Jamal; Ryia Illani Mohd Yunos; Siti Nur Hasanah Mohd Yusuf; Zahirrah Begam Mohamed Rasheed
EPI_ISL_10652467, EPI_ISL_10652468	HOSPITAL SULTANAH NUR ZAHIRAH	Department of Medical Microbiology, Faculty of Medicine, University of Malaya; University of Malaya Medical Centre	I-Ching SAM; Jolene Yin Ling FU; Omar Khalilur Rahman; Yoke Fun Chan
EPI_ISL_9317714	HOSPITAL TAMPIN	UKM Medical Molecular Biology Institute (UMBI)	Khairun Nur Abd Ghafar; Mira Farzana Mohamad Mokhtar; Muhiddin Ishak; Nor Azila Muhammad Azami; Nur Alyaa Afifah Md Shahri; Nurul Syakima Ab Mutalib; Rahman Jamal; Ryia Illani Mohd Yunos; Siti Nur Hasanah Mohd Yusuf; Zahirrah Begam Mohamed Rasheed
EPI_ISL_9579171	HOSPITAL TENGKU AMPUAN AFZAN	iPROMISE, UiTM	Ariza Adnan; Fadzilah Mohd Nor; Lim Wai Feng; Mohd Asif Mohd Sukri; Mohd Nur Fakhruzzaman Noorizhab; Mohd Zaki Salleh; Sazzli Shahlan Kassim; Siti Farah Alwani Mohd Nawi; Siti Hamimah Sheikh Abdul Kadir; Teh Lay Kek; Wang Seok Mui
EPI_ISL_9579112	HPP	iPROMISE, UiTM	Ariza Adnan; Fadzilah Mohd Nor; Lim Wai Feng; Mohd Asif Mohd Sukri; Mohd Nur Fakhruzzaman Noorizhab; Mohd Zaki Salleh; Sazzli Shahlan Kassim; Siti Farah Alwani Mohd Nawi; Siti Hamimah Sheikh Abdul Kadir; Teh Lay Kek; Wang Seok Mui
EPI_ISL_10968980, EPI_ISL_10969007, EPI_ISL_10969009, EPI_ISL_10969010, EPI_ISL_10969040, EPI_ISL_12534823	HRPZ II	Tropical Infectious Diseases Research & Education Centre (TIDREC), Universiti Malaya	AsmaAnati CheMatSeri; Che-Norainon Yaacob; Jia-Yi Tan; Jo-Ern Wong; Kim-Kee Tan; Mulya-Mustika-Sari Zulkifli; Noor Syahida Azizan; Nur-Hidayana Mahfodz; Sazaly AbuBakar; Siti-Sarah Nor'e; Wei-Wei Aw
EPI_ISL_9829342, EPI_ISL_9829347, EPI_ISL_9829351, EPI_ISL_9829369, EPI_ISL_9829370	HSAJB	UKM Medical Molecular Biology Institute (UMBI)	Khairun Nur Abd Ghafar; Mira Farzana Mohamad Mokhtar; Muhiddin Ishak; Nor Azila Muhammad Azami; Nur Alyaa Afifah Md Shahri; Nurul Syakima Ab Mutalib; Rahman Jamal; Ryia Illani Mohd Yunos; Siti Nur Hasanah Mohd Yusuf; Zahirrah Begam Mohamed Rasheed
EPI_ISL_8405648, EPI_ISL_9317733, EPI_ISL_9317736, EPI_ISL_9317740, EPI_ISL_9317753	HTJ	UKM Medical Molecular Biology Institute (UMBI)	Khairun Nur Abd Ghafar; Mira Farzana Mohamad Mokhtar; Muhiddin Ishak; Nor Azila Muhammad Azami; Nur Alyaa Afifah Md Shahri; Nurul Syakima Ab Mutalib; Rahman Jamal; Ryia Illani Mohd Yunos; Siti Nur Hasanah Mohd Yusuf; Zahirrah Begam Mohamed Rasheed
EPI_ISL_11899935, EPI_ISL_11899936, EPI_ISL_11899937, EPI_ISL_11899938, EPI_ISL_11899939, EPI_ISL_11899941	Hospital Ampang	Malaysia Genome and Vaccine Institute	Azrin Ahmad; Enizza Kasim; Irni Suhayu Sapian; Mohd Faizal Abu Bakar; Mohd Ghows Mohd Azzam.; Mohd Noor Mat Isa; Nor Azfa Johari; Nurhezreen Md Iqbal; Shamsidar Sopie; Siti Noraini Othman; Yusuf Muhammad Noor
EPI_ISL_10590730, EPI_ISL_10590731, EPI_ISL_11899921, EPI_ISL_11899922, EPI_ISL_11899925, EPI_ISL_11899929, EPI_ISL_11899933, EPI_ISL_11899934, EPI_ISL_11899944			
see above	Hospital Kuala Lumpur	Malaysia Genome and Vaccine Institute	Azrin Ahmad; Enizza Kasim; Irni Suhayu Sapian; Mohd Faizal Abu Bakar; Mohd Ghows Mohd Azzam.; Mohd Noor Mat Isa; Nor Azfa Johari; Nurhezreen Md Iqbal; Shamsidar Sopie; Siti Noraini Othman; Yusuf Muhammad Noor
EPI_ISL_8405641	Hospital Melaka	UKM Medical Molecular Biology Institute (UMBI)	Khairun Nur Abd Ghafar; Mira Farzana Mohamad Mokhtar; Muhiddin Ishak; Nor Azila Muhammad Azami; Nur Alyaa Afifah Md Shahri; Nurul Syakima Ab Mutalib; Rahman Jamal; Ryia Illani Mohd Yunos; Siti Nur Hasanah Mohd Yusuf; Zahirrah Begam Mohamed Rasheed
EPI_ISL_9579114	Hospital Pulau Pinang	iPROMISE, UiTM	Ariza Adnan; Fadzilah Mohd Nor; Lim Wai Feng; Mohd Asif Mohd Sukri; Mohd Nur Fakhruzzaman Noorizhab; Mohd Zaki Salleh; Sazzli Shahlan Kassim; Siti Farah Alwani Mohd Nawi; Siti Hamimah Sheikh Abdul Kadir; Teh Lay Kek; Wang Seok Mui
EPI_ISL_10652515	Hospital Teluk Intan	Department of Medical Microbiology, Faculty of Medicine, University of Malaya; University of Malaya Medical Centre	I-Ching SAM; Jolene Yin Ling FU; Omar Khalilur Rahman; Yoke Fun Chan
EPI_ISL_11899943	Hospital Tengku Ampuan Rahimah	Malaysia Genome and Vaccine Institute	Azrin Ahmad; Enizza Kasim; Irni Suhayu Sapian; Mohd Faizal Abu Bakar; Mohd Ghows Mohd Azzam.; Mohd Noor Mat Isa; Nor Azfa Johari; Nurhezreen Md Iqbal; Shamsidar Sopie; Siti Noraini Othman; Yusuf Muhammad Noor
EPI_ISL_9579159	Hospital Tuanku Fauziah	iPROMISE, UiTM	Ariza Adnan; Fadzilah Mohd Nor; Lim Wai Feng; Mohd Asif Mohd Sukri; Mohd Nur Fakhruzzaman Noorizhab; Mohd Zaki Salleh; Sazzli Shahlan Kassim; Siti Farah Alwani Mohd Nawi; Siti Hamimah Sheikh Abdul Kadir; Teh Lay Kek; Wang Seok Mui
EPI_ISL_9317732	Hospital melaka	UKM Medical Molecular Biology Institute (UMBI)	Khairun Nur Abd Ghafar; Mira Farzana Mohamad Mokhtar; Muhiddin Ishak; Nor Azila Muhammad Azami; Nur Alyaa Afifah Md Shahri; Nurul Syakima Ab Mutalib; Rahman Jamal; Ryia Illani Mohd Yunos; Siti Nur Hasanah Mohd Yusuf; Zahirrah Begam Mohamed Rasheed
EPI_ISL_9579113	Hospital pulau pinang	iPROMISE, UiTM	Ariza Adnan; Fadzilah Mohd Nor; Lim Wai Feng; Mohd Asif Mohd Sukri; Mohd Nur Fakhruzzaman Noorizhab; Mohd Zaki Salleh; Sazzli Shahlan Kassim; Siti Farah Alwani Mohd Nawi; Siti Hamimah Sheikh Abdul Kadir; Teh Lay Kek; Wang Seok Mui
EPI_ISL_10969008	HrpzII	Tropical Infectious Diseases Research & Education Centre (TIDREC), Universiti Malaya	AsmaAnati CheMatSeri; Che-Norainon Yaacob; Jia-Yi Tan; Jo-Ern Wong; Kim-Kee Tan; Mulya-Mustika-Sari Zulkifli; Noor Syahida Azizan; Nur-Hidayana Mahfodz; Sazaly AbuBakar; Siti-Sarah Nor'e
EPI_ISL_9579170	Htaa	iPROMISE, UiTM	Ariza Adnan; Fadzilah Mohd Nor; Lim Wai Feng; Mohd Asif Mohd Sukri; Mohd Nur Fakhruzzaman Noorizhab; Mohd Zaki Salleh; Sazzli Shahlan Kassim; Siti Farah Alwani Mohd Nawi; Siti Hamimah Sheikh Abdul Kadir; Teh Lay Kek; Wang Seok Mui
EPI_ISL_10652503	IMR	Department of Medical Microbiology, Faculty of Medicine, University of Malaya; University of Malaya Medical Centre	I-Ching SAM; Jolene Yin Ling FU; Omar Khalilur Rahman; Yoke Fun Chan
EPI_ISL_11899977	IMR	Malaysia Genome and Vaccine Institute	Azrin Ahmad; Enizza Kasim; Irni Suhayu Sapian; Mohd Faizal Abu Bakar; Mohd Ghows Mohd Azzam.; Mohd Noor Mat Isa; Nor Azfa Johari; Nurhezreen Md Iqbal; Shamsidar Sopie; Siti Noraini Othman; Yusuf Muhammad Noor
EPI_ISL_9829817, EPI_ISL_9829818, EPI_ISL_9829819, EPI_ISL_9829820, EPI_ISL_9829821, EPI_ISL_9829822, EPI_ISL_9829823, EPI_ISL_9829824, EPI_ISL_9829825, EPI_ISL_9829826, EPI_ISL_9829827, EPI_ISL_9829828, EPI_ISL_9829829, EPI_ISL_9829830, EPI_ISL_9829831, EPI_ISL_9829832, EPI_ISL_9829834, EPI_ISL_9829835, EPI_ISL_9829836, EPI_ISL_9829837, EPI_ISL_9829838, EPI_ISL_9829839, EPI_ISL_9829840, EPI_ISL_9829841, EPI_ISL_9829842, EPI_ISL_9829843, EPI_ISL_9829844, EPI_ISL_9829845, EPI_ISL_9829846, EPI_ISL_9829847, EPI_ISL_9829853, EPI_ISL_9829854, EPI_ISL_9829855, EPI_ISL_9829856, EPI_ISL_9829857, EPI_ISL_9829858, EPI_ISL_9829859, EPI_ISL_9829860, EPI_ISL_9829861, EPI_ISL_9829862, EPI_ISL_9829863, EPI_ISL_9829864, EPI_ISL_9829865, EPI_ISL_9829866, EPI_ISL_9829867, EPI_ISL_9829868, EPI_ISL_9829869, EPI_ISL_9829870, EPI_ISL_9829871, EPI_ISL_9829872, EPI_ISL_9829873, EPI_ISL_9829874, EPI_ISL_9829875, EPI_ISL_9829876			
see above	IMR	Tropical Infectious Diseases Research & Education Centre (TIDREC), Universiti Malaya	AsmaAnati CheMatSeri; Che-Norainon Yaacob; Jia-Yi Tan; Jo-Ern Wong; Kim-Kee Tan; Mulya-Mustika-Sari Zulkifli; Noor Syahida Azizan; Nur-Hidayana Mahfodz; Sazaly AbuBakar; Siti-Sarah Nor'e
EPI_ISL_11323779, EPI_ISL_11323796	Institut Biologi Molekul Perubatan UKM (UMBI)	UKM Medical Molecular Biology Institute (UMBI)	Khairun Nur Abd Ghafar; Mira Farzana Mohamad Mokhtar; Muhiddin Ishak; Nor Azila Muhammad Azami; Nur Alyaa Afifah Md Shahri; Nurul Syakima Ab Mutalib; Rahman Jamal; Ryia Illani Mohd Yunos; Siti Nur Hasanah Mohd Yusuf; Zahirrah Begam Mohamed Rasheed
EPI_ISL_10968997, EPI_ISL_10968998, EPI_ISL_10968999, EPI_ISL_10969000, EPI_ISL_12534826, EPI_ISL_12534827, EPI_ISL_12534844, EPI_ISL_12534845			
see above	JABATAN PATOLOGI, HOSPITAL RAJA PEREMPUAN ZAINAB II	Tropical Infectious Diseases Research & Education Centre (TIDREC), Universiti Malaya	AsmaAnati CheMatSeri; Che-Norainon Yaacob; Jia-Yi Tan; Jo-Ern Wong; Kim-Kee Tan; Mulya-Mustika-Sari Zulkifli; Noor Syahida Azizan; Nur-Hidayana Mahfodz; Sazaly AbuBakar; Siti-Sarah Nor'e; Wei-Wei Aw
EPI_ISL_9579167, EPI_ISL_9579172, EPI_ISL_9579173	JKN PAHANG	iPROMISE, UiTM	Ariza Adnan; Fadzilah Mohd Nor; Lim Wai Feng; Mohd Asif Mohd Sukri; Mohd Nur Fakhruzzaman Noorizhab; Mohd Zaki Salleh; Sazzli Shahlan Kassim; Siti Farah Alwani Mohd Nawi; Siti Hamimah Sheikh Abdul Kadir; Teh Lay Kek; Wang Seok Mui

EPI_ISL_9579205, EPI_ISL_11112387	Jabatan Kesihatan Negeri	iPROMISE, UiTM	Ariza Adnan; Fadzilah Mohd Nor; Lim Wai Feng; Mohd Asif Mohd Sukri; Mohd Nur Fakhruzzaman Noorizhab; Mohd Zaki Salleh; Sazzli Shahlan Kassim; Siti Farah Alwani Mohd Nawi; Siti Hamimah Sheikh Abdul Kadir; Teh Lay Kek; Wang Seok Mui
EPI_ISL_11899945, EPI_ISL_11899948, EPI_ISL_11899949	Jabatan Kesihatan Negeri WP Labuan	Malaysia Genome and Vaccine Institute	Azrin Ahmad; Enizza Kasim; Irni Suhayu Sapian; Mohd Faizal Abu Bakar; Mohd Ghows Mohd Azzam.; Mohd Noor Mat Isa; Nor Azfa Johari; Nurhezreen Md Iqbal; Shamsidar Sopie; Siti Noraini Othman; Yusuf Muhammad Noor
EPI_ISL_9317726	KK PORT DICKSON	UKM Medical Molecular Biology Institute (UMBI)	Khairun Nur Abd Ghafar; Mira Farzana Mohamad Mokhtar; Muhiddin Ishak; Nor Azila Muhammad Azami; Nur Alyaa Afifah Md Shahri; Nurul Syakima Ab Mutalib; Rahman Jamal; Ryia Illani Mohd Yunos; Siti Nur Hasanah Mohd Yusuf; Zahirrah Begam Mohamed Rasheed
EPI_ISL_10652484	KLINIK KESIHATAN WAKAF CHE YEH	Department of Medical Microbiology, Faculty of Medicine, University of Malaya; University of Malaya Medical Centre	I-Ching SAM; Jolene Yin Ling FU; Omar Khalilur Rahman; Yoke Fun Chan
EPI_ISL_10652508, EPI_ISL_10652509, EPI_ISL_11754721	KOMPLEKS SURVELAN PKD KINTA	Department of Medical Microbiology, Faculty of Medicine, University of Malaya; University of Malaya Medical Centre	I-Ching SAM; Jolene Yin Ling FU; Omar Khalilur Rahman; Yoke Fun Chan
EPI_ISL_10652490	Kk perol	Department of Medical Microbiology, Faculty of Medicine, University of Malaya; University of Malaya Medical Centre	I-Ching SAM; Jolene Yin Ling FU; Omar Khalilur Rahman; Yoke Fun Chan
EPI_ISL_10968978, EPI_ISL_10968979	Klinik Dr Ramzi Tanah Merah	Tropical Infectious Diseases Research & Education Centre (TIDREC), Universiti Malaya	AsmaAnati CheMatSeri; Che-Norainon Yaacob; Jia-Yi Tan; Jo-Ern Wong; Kim-Kee Tan; Mulya-Mustika-Sari Zulkifli; Noor Syahida Azizan; Nur-Hidayana Mahfodz; Sazaly AbuBakar; Siti-Sarah Nor'e
EPI_ISL_8560032, EPI_ISL_8560033, EPI_ISL_8560034, EPI_ISL_8560036, EPI_ISL_8560037, EPI_ISL_8560038, EPI_ISL_8560039, EPI_ISL_8560040, EPI_ISL_8560041, EPI_ISL_8560043, EPI_ISL_8560044, EPI_ISL_8560045, EPI_ISL_8560046, EPI_ISL_8560047, EPI_ISL_8560048, EPI_ISL_8560050, EPI_ISL_8560051, EPI_ISL_8560052, EPI_ISL_8560053, EPI_ISL_8560054, EPI_ISL_8560055, EPI_ISL_8560057, EPI_ISL_8560058, EPI_ISL_8560059, EPI_ISL_8560060, EPI_ISL_8560063, EPI_ISL_8560064, EPI_ISL_8560065, EPI_ISL_8560066, EPI_ISL_8560067, EPI_ISL_8560070, EPI_ISL_8560071, EPI_ISL_8560072, EPI_ISL_8560073, EPI_ISL_8560074, EPI_ISL_8560077, EPI_ISL_8560078, EPI_ISL_8560079, EPI_ISL_8560080, EPI_ISL_8560081, EPI_ISL_8560082, EPI_ISL_8560085, EPI_ISL_8560086, EPI_ISL_8560087, EPI_ISL_8560088, EPI_ISL_8560089, EPI_ISL_8560094, EPI_ISL_8560095, EPI_ISL_8560097, EPI_ISL_8560098, EPI_ISL_8560099, EPI_ISL_8560102, EPI_ISL_8560103, EPI_ISL_8560104, EPI_ISL_8560105, EPI_ISL_8560108, EPI_ISL_8560109, EPI_ISL_8560110, EPI_ISL_8560111, EPI_ISL_8560114, EPI_ISL_8560115, EPI_ISL_8560946, EPI_ISL_8560949, EPI_ISL_8560954, EPI_ISL_8560957, EPI_ISL_8560962, EPI_ISL_8560965, EPI_ISL_8560967, EPI_ISL_8560970, EPI_ISL_8560973, EPI_ISL_8560975, EPI_ISL_8560978, EPI_ISL_8560979, EPI_ISL_8560982, EPI_ISL_8560984, EPI_ISL_8560985, EPI_ISL_8560992, EPI_ISL_8560993, EPI_ISL_8560998, EPI_ISL_8561001, EPI_ISL_8561002, EPI_ISL_8561005, EPI_ISL_8561008, EPI_ISL_8561009, EPI_ISL_8561012, EPI_ISL_8561014, EPI_ISL_8561015, EPI_ISL_8561021, EPI_ISL_8561028, EPI_ISL_8689977, EPI_ISL_8689978, EPI_ISL_8689979, EPI_ISL_8689980, EPI_ISL_8689981, EPI_ISL_8689982, EPI_ISL_8689983, EPI_ISL_8690070, EPI_ISL_8690073, EPI_ISL_8690314, EPI_ISL_8690549, EPI_ISL_8690644, EPI_ISL_8690988, EPI_ISL_8691336, EPI_ISL_8691874, EPI_ISL_8692048, EPI_ISL_8692139, EPI_ISL_8692421, EPI_ISL_8692422, EPI_ISL_8692423, EPI_ISL_8692424, EPI_ISL_8693690, EPI_ISL_9092706, EPI_ISL_9092707, EPI_ISL_9092708, EPI_ISL_9092709, EPI_ISL_9832500, EPI_ISL_9842677			
see above	Kuala Lumpur International Airport (KLIA) Health Office	Institute for Medical Research, Infectious Disease Research Centre, National Institutes of Health, Ministry of Health Malaysia	Ahmad FA; Ahmad Fazilah NA; Anasir MI; Azizan MA; Kamel K; Mohamad Sukri MZ; Norhisham SN; Ramly N; Robert F; Rosli NR; Suppiah J; Thayan R
EPI_ISL_10495871	LAHAD DATU AREA HEALTH OFFICE	Institute for Medical Research, Infectious Disease Research Centre, National Institutes of Health, Ministry of Health Malaysia	Ahmad FA; Ahmad Fazilah NA; Anasir MI; Arip MS; Azizan MA; Christopher Hollip K; Jaimin JJ; Kamel K; Mohamad Sukri MZ; Mohammad R; Mohd Yasin R; Norhisham SN; Ramly N; Robert F; Rosli NR; Shamsaddin E; Suppiah J; Tan YC; Thayan R
EPI_ISL_12534843	MKA KOTA BHARU	Tropical Infectious Diseases Research & Education Centre (TIDREC), Universiti Malaya	AsmaAnati CheMatSeri; Che-Norainon Yaacob; Jia-Yi Tan; Jo-Ern Wong; Kim-Kee Tan; Mulya-Mustika-Sari Zulkifli; Noor Syahida Azizan; Nur-Hidayana Mahfodz; Sazaly AbuBakar; Siti-Sarah Nor'e; Wei-Wei Aw
EPI_ISL_12534846, EPI_ISL_12534847	MKAKB	Tropical Infectious Diseases Research & Education Centre (TIDREC), Universiti Malaya	AsmaAnati CheMatSeri; Che-Norainon Yaacob; Jia-Yi Tan; Jo-Ern Wong; Kim-Kee Tan; Mulya-Mustika-Sari Zulkifli; Noor Syahida Azizan; Nur-Hidayana Mahfodz; Sazaly AbuBakar; Siti-Sarah Nor'e; Wei-Wei Aw
EPI_ISL_8650093, EPI_ISL_8650094, EPI_ISL_8650095, EPI_ISL_8650096	Normah Medical Specialist Centre, Kuching	Institute of Health and Community Medicine	Chan Chia Jui; Chua Hock Hin; David Perera; Ikwannuddin Y; Ooi Mong How; Tonnii Sia Loong Loong; Wong Jyn Shan
EPI_ISL_9829350, EPI_ISL_9829357	PEJABAT KESIHATAN DAERAH BATU PAHAT	UKM Medical Molecular Biology Institute (UMBI)	Khairun Nur Abd Ghafar; Mira Farzana Mohamad Mokhtar; Muhiddin Ishak; Nor Azila Muhammad Azami; Nur Alyaa Afifah Md Shahri; Nurul Syakima Ab Mutalib; Rahman Jamal; Ryia Illani Mohd Yunos; Siti Nur Hasanah Mohd Yusuf; Zahirrah Begam Mohamed Rasheed
EPI_ISL_12534870	PEJABAT KESIHATAN DAERAH LANGKAWI	Tropical Infectious Diseases Research & Education Centre (TIDREC), Universiti Malaya	AsmaAnati CheMatSeri; Che-Norainon Yaacob; Jia-Yi Tan; Jo-Ern Wong; Kim-Kee Tan; Mulya-Mustika-Sari Zulkifli; Noor Syahida Azizan; Nur-Hidayana Mahfodz; Sazaly AbuBakar; Siti-Sarah Nor'e; Wei-Wei Aw
EPI_ISL_10652489	PEJABAT KESIHATAN DAERAH PASIR MAS	Department of Medical Microbiology, Faculty of Medicine, University of Malaya; University of Malaya Medical Centre	I-Ching SAM; Jolene Yin Ling FU; Omar Khalilur Rahman; Yoke Fun Chan
EPI_ISL_9000519, EPI_ISL_9579186, EPI_ISL_9579187, EPI_ISL_9579188, EPI_ISL_9579189, EPI_ISL_9579190, EPI_ISL_9579191, EPI_ISL_9579192, EPI_ISL_9579193, EPI_ISL_9579194, EPI_ISL_9579195, EPI_ISL_9579196, EPI_ISL_11112439, EPI_ISL_11112442, EPI_ISL_11112443, EPI_ISL_11112445, EPI_ISL_11112446, EPI_ISL_11112447, EPI_ISL_11112448, EPI_ISL_11112450, EPI_ISL_11112451			
see above	PEJABAT KESIHATAN DAERAH SEBERANG PERAI UTARA PULAU PINANG	iPROMISE, UiTM	Ariza Adnan; Fadzilah Mohd Nor; Lim Wai Feng; Mohd Asif Mohd Sukri; Mohd Nur Fakhruzzaman Noorizhab; Mohd Zaki Salleh; Sazzli Shahlan Kassim; Siti Farah Alwani Mohd Nawi; Siti Hamimah Sheikh Abdul Kadir; Teh Lay Kek; Wang Seok Mui
EPI_ISL_9829348, EPI_ISL_9829353, EPI_ISL_9829385	PKD ALOR GAJAH	UKM Medical Molecular Biology Institute (UMBI)	Khairun Nur Abd Ghafar; Mira Farzana Mohamad Mokhtar; Muhiddin Ishak; Nor Azila Muhammad Azami; Nur Alyaa Afifah Md Shahri; Nurul Syakima Ab Mutalib; Rahman Jamal; Ryia Illani Mohd Yunos; Siti Nur Hasanah Mohd Yusuf; Zahirrah Begam Mohamed Rasheed
EPI_ISL_9579199	PKD BARAT DAYA	iPROMISE, UiTM	Ariza Adnan; Fadzilah Mohd Nor; Lim Wai Feng; Mohd Asif Mohd Sukri; Mohd Nur Fakhruzzaman Noorizhab; Mohd Zaki Salleh; Sazzli Shahlan Kassim; Siti Farah Alwani Mohd Nawi; Siti Hamimah Sheikh Abdul Kadir; Teh Lay Kek; Wang Seok Mui
EPI_ISL_10652487, EPI_ISL_10652495	PKD Bachok	Department of Medical Microbiology, Faculty of Medicine, University of Malaya; University of Malaya Medical Centre	I-Ching SAM; Jolene Yin Ling FU; Omar Khalilur Rahman; Yoke Fun Chan
EPI_ISL_9317727	PKD JEMPOL	UKM Medical Molecular Biology Institute (UMBI)	Khairun Nur Abd Ghafar; Mira Farzana Mohamad Mokhtar; Muhiddin Ishak; Nor Azila Muhammad Azami; Nur Alyaa Afifah Md Shahri; Nurul Syakima Ab Mutalib; Rahman Jamal; Ryia Illani Mohd Yunos; Siti Nur Hasanah Mohd Yusuf; Zahirrah Begam Mohamed Rasheed
EPI_ISL_9317724, EPI_ISL_9317749, EPI_ISL_9829341, EPI_ISL_9829368, EPI_ISL_9829371, EPI_ISL_9829372, EPI_ISL_9829374			
see above	PKD JOHOR BAHRU	UKM Medical Molecular Biology Institute (UMBI)	Khairun Nur Abd Ghafar; Mira Farzana Mohamad Mokhtar; Muhiddin Ishak; Nor Azila Muhammad Azami; Nur Alyaa Afifah Md Shahri; Nurul Syakima Ab Mutalib; Rahman Jamal; Ryia Illani Mohd Yunos; Siti Nur Hasanah Mohd Yusuf; Zahirrah Begam Mohamed Rasheed
EPI_ISL_12097308	PKD KLUANG	UKM Medical Molecular Biology Institute (UMBI)	Khairun Nur Abd Ghafar; Mira Farzana Mohamad Mokhtar; Muhiddin Ishak; Nor Azila Muhammad Azami; Nur Alyaa Afifah Md Shahri; Nurul Syakima Ab Mutalib; Rahman Jamal; Ryia Illani Mohd Yunos; Siti Nur Hasanah Mohd Yusuf; Zahirrah Begam Mohamed Rasheed
EPI_ISL_8405633	PKD KOTA TINGGI	UKM Medical Molecular Biology Institute (UMBI)	Khairun Nur Abd Ghafar; Mira Farzana Mohamad Mokhtar; Muhiddin Ishak; Nor Azila Muhammad Azami; Nur Alyaa Afifah Md Shahri; Nurul Syakima Ab Mutalib; Rahman Jamal; Ryia Illani Mohd Yunos; Siti Nur Hasanah Mohd Yusuf; Zahirrah Begam Mohamed Rasheed
EPI_ISL_9317721	PKD KUALA PILAH	UKM Medical Molecular Biology Institute (UMBI)	Khairun Nur Abd Ghafar; Mira Farzana Mohamad Mokhtar; Muhiddin Ishak; Nor Azila Muhammad Azami; Nur Alyaa Afifah Md Shahri; Nurul Syakima Ab Mutalib; Rahman Jamal; Ryia Illani Mohd Yunos; Siti Nur Hasanah Mohd Yusuf; Zahirrah Begam Mohamed Rasheed
EPI_ISL_10652472, EPI_ISL_10652507, EPI_ISL_10652511, EPI_ISL_10652512, EPI_ISL_10652513, EPI_ISL_10652514, EPI_ISL_10652523			
see above	PKD LARUT MATANG DAN SELAMA	Department of Medical Microbiology, Faculty of Medicine, University of Malaya; University of Malaya Medical Centre	I-Ching SAM; Jolene Yin Ling FU; Omar Khalilur Rahman; Yoke Fun Chan
EPI_ISL_9829343, EPI_ISL_9829367, EPI_ISL_9829377, EPI_ISL_9829381	PKD MELAKA TENGAH	UKM Medical Molecular Biology Institute (UMBI)	Khairun Nur Abd Ghafar; Mira Farzana Mohamad Mokhtar; Muhiddin Ishak; Nor Azila Muhammad Azami; Nur Alyaa Afifah Md Shahri; Nurul Syakima Ab Mutalib; Rahman Jamal; Ryia Illani Mohd Yunos; Siti Nur Hasanah Mohd Yusuf; Zahirrah Begam Mohamed Rasheed
EPI_ISL_9317719	PKD Mersing	UKM Medical Molecular Biology Institute (UMBI)	Khairun Nur Abd Ghafar; Mira Farzana Mohamad Mokhtar; Muhiddin Ishak; Nor Azila Muhammad Azami; Nur Alyaa Afifah Md Shahri; Nurul Syakima Ab Mutalib; Rahman Jamal; Ryia Illani Mohd Yunos; Siti Nur Hasanah Mohd Yusuf; Zahirrah Begam Mohamed Rasheed
EPI_ISL_10652510, EPI_ISL_10652524	PKD PERAK TENGAH	Department of Medical Microbiology, Faculty of Medicine, University of Malaya; University of Malaya Medical Centre	I-Ching SAM; Jolene Yin Ling FU; Omar Khalilur Rahman; Yoke Fun Chan
EPI_ISL_9579185	PKD SEBERANG PERAI SELATAN	iPROMISE, UiTM	Ariza Adnan; Fadzilah Mohd Nor; Lim Wai Feng; Mohd Asif Mohd Sukri; Mohd Nur Fakhruzzaman Noorizhab; Mohd Zaki Salleh; Sazzli Shahlan Kassim; Siti Farah Alwani Mohd Nawi; Siti Hamimah Sheikh Abdul Kadir; Teh Lay Kek; Wang Seok Mui
EPI_ISL_9317754	PKD SEGAMAT	UKM Medical Molecular Biology Institute (UMBI)	Khairun Nur Abd Ghafar; Mira Farzana Mohamad Mokhtar; Muhiddin Ishak; Nor Azila Muhammad Azami; Nur Alyaa Afifah Md Shahri; Nurul Syakima Ab Mutalib; Rahman Jamal; Ryia Illani Mohd Yunos; Siti Nur Hasanah Mohd Yusuf; Zahirrah Begam Mohamed Rasheed
EPI_ISL_9317715, EPI_ISL_9317720, EPI_ISL_9317746, EPI_ISL_9317752	PKD SEREMBAN	UKM Medical Molecular Biology Institute (UMBI)	Khairun Nur Abd Ghafar; Mira Farzana Mohamad Mokhtar; Muhiddin Ishak; Nor Azila Muhammad Azami; Nur Alyaa Afifah Md Shahri; Nurul Syakima Ab Mutalib; Rahman Jamal; Ryia Illani Mohd Yunos; Siti Nur Hasanah Mohd Yusuf; Zahirrah Begam Mohamed Rasheed
EPI_ISL_11112412, EPI_ISL_11112413, EPI_ISL_11112415, EPI_ISL_11112416, EPI_ISL_11112419, EPI_ISL_11112423, EPI_ISL_11112453			
see above	PKD SPT	iPROMISE, UiTM	Ariza Adnan; Fadzilah Mohd Nor; Lim Wai Feng; Mohd Asif Mohd Sukri; Mohd Nur Fakhruzzaman Noorizhab; Mohd Zaki Salleh; Sazzli Shahlan Kassim; Siti Farah Alwani Mohd Nawi; Siti Hamimah Sheikh Abdul Kadir; Teh Lay Kek; Wang Seok Mui
EPI_ISL_10397071, EPI_ISL_10397072, EPI_ISL_10397112, EPI_ISL_10397114	PKD TAMPIN	UKM Medical Molecular Biology Institute (UMBI)	Khairun Nur Abd Ghafar; Mira Farzana Mohamad Mokhtar; Muhiddin Ishak; Nor Azila Muhammad Azami; Nur Alyaa Afifah Md Shahri; Nurul Syakima Ab Mutalib; Rahman Jamal; Ryia Illani Mohd Yunos; Siti Nur Hasanah Mohd Yusuf; Zahirrah Begam Mohamed Rasheed
EPI_ISL_9317713, EPI_ISL_9317718, EPI_ISL_9317747, EPI_ISL_9317748, EPI_ISL_9317756	PKD TANGKAK	UKM Medical Molecular Biology Institute (UMBI)	Khairun Nur Abd Ghafar; Mira Farzana Mohamad Mokhtar; Muhiddin Ishak; Nor Azila Muhammad Azami; Nur Alyaa Afifah Md Shahri; Nurul Syakima Ab Mutalib; Rahman Jamal; Ryia Illani Mohd Yunos; Siti Nur Hasanah Mohd Yusuf; Zahirrah Begam Mohamed Rasheed
EPI_ISL_10652483, EPI_ISL_10652485, EPI_ISL_10652486, EPI_ISL_10652488,	PKD Tanah Merah	Department of Medical Microbiology, Faculty of Medicine, University of Malaya;	I-Ching SAM; Jolene Yin Ling FU; Omar Khalilur Rahman; Yoke Fun Chan

EPI_ISL_10652493		University of Malaya Medical Centre	
EPI_ISL_9579197, EPI_ISL_9579198, EPI_ISL_11112414, EPI_ISL_11112417, EPI_ISL_11112418, EPI_ISL_11112420, EPI_ISL_11112421, EPI_ISL_11112422			
see above	PKDSPT	iPROMISE, UiTM	Ariza Adnan; Fadzilah Mohd Nor; Lim Wai Feng; Mohd Asif Mohd Sukri; Mohd Nur Fakhruzzaman Noorizhab; Mohd Zaki Salleh; Sazzli Shahlan Kassim; Siti Farah Alwani Mohd Nawi; Siti Hamimah Sheikh Abdul Kadir; Teh Lay Kek; Wang Seok Mui
EPI_ISL_11112411	Pejabat Kesihatan Daerah Timur Laut	iPROMISE, UiTM	Ariza Adnan; Fadzilah Mohd Nor; Lim Wai Feng; Mohd Asif Mohd Sukri; Mohd Nur Fakhruzzaman Noorizhab; Mohd Zaki Salleh; Sazzli Shahlan Kassim; Siti Farah Alwani Mohd Nawi; Siti Hamimah Sheikh Abdul Kadir; Teh Lay Kek; Wang Seok Mui
EPI_ISL_10590751, EPI_ISL_10590752, EPI_ISL_10590753, EPI_ISL_10590754, EPI_ISL_10590765, EPI_ISL_10590771, EPI_ISL_10590772, EPI_ISL_10590797, EPI_ISL_10590800			
see above	Pejabat Kesihatan Daerah Titiwangsa	Malaysia Genome and Vaccine Institute	Azrin Ahmad; Enizza Kasim; Irni Suhayu Sapian; Mohd Faizal Abu Bakar; Mohd Ghows Mohd Azzam.; Mohd Noor Mat Isa; Nurhezreen Md Iqbal; Shamsidar Sopie; Siti Noraini Othman; Yusuf Muhammad Noor
EPI_ISL_8745679, EPI_ISL_8745680, EPI_ISL_8745681, EPI_ISL_8745682, EPI_ISL_8745683, EPI_ISL_8745684, EPI_ISL_8745685, EPI_ISL_8745686, EPI_ISL_8745687, EPI_ISL_8745688, EPI_ISL_8745689, EPI_ISL_8745690, EPI_ISL_8745691, EPI_ISL_8745692, EPI_ISL_8745693, EPI_ISL_8745694, EPI_ISL_8745695, EPI_ISL_8745696, EPI_ISL_8745697, EPI_ISL_8745698, EPI_ISL_8745699, EPI_ISL_8745700, EPI_ISL_8745701, EPI_ISL_8745702, EPI_ISL_8745703, EPI_ISL_8745704, EPI_ISL_8745705, EPI_ISL_8745706, EPI_ISL_8745707, EPI_ISL_8745708, EPI_ISL_8745709, EPI_ISL_8745710, EPI_ISL_8745711, EPI_ISL_8745712, EPI_ISL_8745713			
see above	Pejabat Kesihatan KLIA	Malaysia Genome and Vaccine Institute	Azrin Ahmad; Enizza Kasim; Irni Suhayu Sapian; Mohd Faizal Abu Bakar; Mohd Ghows Mohd Azzam.; Mohd Noor Mat Isa; Nor Azfa Johari; Nurhezreen Md Iqbal; Shamsidar Sopie; Siti Noraini Othman; Yusuf Muhammad Noor
EPI_ISL_8603729, EPI_ISL_8603730, EPI_ISL_8603731, EPI_ISL_8603732, EPI_ISL_8603733, EPI_ISL_8603734, EPI_ISL_8603735, EPI_ISL_8603736, EPI_ISL_8603737, EPI_ISL_8603738, EPI_ISL_8603739, EPI_ISL_8603755, EPI_ISL_8603756, EPI_ISL_8603757, EPI_ISL_8603758, EPI_ISL_8603759, EPI_ISL_8603760, EPI_ISL_8603761, EPI_ISL_8603762			
see above	Pejabat Kesihatan KLIA	iPROMISE, UiTM	Ariza Adnan; Fadzilah Mohd Nor; Lim Wai Feng; Mohd Asif Mohd Sukri; Mohd Nur Fakhruzzaman Noorizhab; Mohd Zaki Salleh; Sazzli Shahlan Kassim; Siti Farah Alwani Mohd Nawi; Siti Hamimah Sheikh Abdul Kadir; Teh Lay Kek; Wang Seok Mui
EPI_ISL_12646704	Public Health Laboratory, Ipoh	Public Health Laboratory Ipoh Disease Section	A. Nisha; MH. Chew; R. Rajes; S. Humairah
EPI_ISL_8650087, EPI_ISL_8650088	Rejang Medical Centre (Sibu)	Institute of Health and Community Medicine	Chan Chia Jui; Chua Hock Hin; David Perera; Ooi Mong How; Tonnii Sia Loong Loong; Wong Jyn Shan
EPI_ISL_8188848, EPI_ISL_8188849, EPI_ISL_8188850, EPI_ISL_8188851, EPI_ISL_8188852, EPI_ISL_8188853, EPI_ISL_8188854, EPI_ISL_8188875, EPI_ISL_8650103, EPI_ISL_8650104, EPI_ISL_8650105, EPI_ISL_8650145, EPI_ISL_8650148, EPI_ISL_8650151, EPI_ISL_8650153			
see above	Sarawak General Hospital (Kuching)	Institute of Health and Community Medicine	Chan Chia Jui; Chua Hock Hin; David Perera; Ooi Mong How; Tonnii Sia Loong Loong; Wong Jyn Shan
EPI_ISL_8767325	UMMC	Department of Medical Microbiology, Faculty of Medicine, University of Malaya; University of Malaya Medical Centre	I-Ching SAM; Jolene Yin Ling FU; Omar Khalilur Rahman; Yoke Fun Chan
EPI_ISL_9829375	Ward 4-2 (covid), Hospital Melaka	UKM Medical Molecular Biology Institute (UMBI)	Khairun Nur Abd Ghafar; Mira Farzana Mohamad Mokhtar; Muhiddin Ishak; Nor Azila Muhammad Azami; Nur Alyaa Afifah Md Shahri; Nurul Syakima Ab Mutalib; Rahman Jamal; Ryia Illani Mohd Yunos; Siti Nur Hasanah Mohd Yusuf; Zahirrah Begam Mohamed Rasheed
EPI_ISL_9829833	iMR	Tropical Infectious Diseases Research & Education Centre (TIDREC), Universiti Malaya	AsmaAnati CheMatSeri; Che-Norainon Yaacob; Jia-Yi Tan; Jo-Ern Wong; Kim-Kee Tan; Mulya-Mustika-Sari Zulkifli; Noor Syahida Azizan; Nur-Hidayana Mahfodz; Sazaly AbuBakar; Siti-Sarah Nor'e
EPI_ISL_10652530	mka kb	Department of Medical Microbiology, Faculty of Medicine, University of Malaya; University of Malaya Medical Centre	I-Ching SAM; Jolene Yin Ling FU; Omar Khalilur Rahman; Yoke Fun Chan

We gratefully acknowledge the following Authors from the Originating laboratories responsible for obtaining the specimens, as well as the Submitting laboratories where the genome data were generated and shared via GISAID, on which this research is based.

All Submitters of data may be contacted directly via www.gisaid.org

Authors are sorted alphabetically.

Acknowledgement EPI_SET Identifier: EPI_SET_20220603rw

Accession ID	Originating Laboratory	Submitting Laboratory	Authors
EPI_ISL_11899960	BP Clinical Lab Sdn Bhd (KLIA)	Malaysia Genome and Vaccine Institute	Azrin Ahmad; Enizza Kasim; Irni Suhayu Sapian; Mohd Faizal Abu Bakar; Mohd Ghows Mohd Azzam.; Mohd Noor Mat Isa; Nor Azfa Johari; Nurhezreen Md Iqbal; Shamsidar Sopie; Siti Noraini Othman; Yusuf Muhammad Noor
EPI_ISL_10297332	Beaufort Health Clinic	Institute for Medical Research, Infectious Disease Research Centre, National Institutes of Health, Ministry of Health Malaysia	Ahmad FA; Ahmad Fazilah NA; Anasir MI; Azizan MA; Kamel K; Mohamad Sukri MZ; Norhisham SN; Ramly N; Robert F; Rosli NR; Suppiah J; Thayan R
EPI_ISL_8782801, EPI_ISL_8782804, EPI_ISL_8782805, EPI_ISL_8782806, EPI_ISL_8782807	Bintulu Hospital PCR Lab, Bintulu	Institute of Health and Community Medicine	Chan Chia Jui; Chua Hock Hin; David Perera; Ooi Mong How; Tonnii Sia Loong Loong; Wong Jyn Shan
EPI_ISL_10397073	COVID WARD 4-1	UKM Medical Molecular Biology Institute (UMBI)	Khairun Nur Abd Ghafar; Mira Farzana Mohamad Mokhtar; Muhiddin Ishak; Nor Azila Muhammad Azami; Nur Alyaa Afifah Md Shahri; Nurul Syakima Ab Mutalib; Rahman Jamal; Ryia Illani Mohd Yunos; Siti Nur Hasanah Mohd Yusuf; Zahirrah Begam Mohamed Rasheed
EPI_ISL_10652538, EPI_ISL_11754714, EPI_ISL_11754715, EPI_ISL_11754716, EPI_ISL_11754717, EPI_ISL_11754718, EPI_ISL_11754719, EPI_ISL_11754759, EPI_ISL_11754764			
see above	CPRC JKNT	Department of Medical Microbiology, Faculty of Medicine, University of Malaya; University of Malaya Medical Centre	I-Ching SAM; Jolene Yin Ling FU; Omar Khalilur Rahman; Yoke Fun Chan
EPI_ISL_10968982	CPRC JKNT	Tropical Infectious Diseases Research & Education Centre (TIDREC), Universiti Malaya	AsmaAnati CheMatSeri; Che-Norainon Yaacob; Jia-Yi Tan; Jo-Ern Wong; Kim-Kee Tan; Mulya-Mustika-Sari Zulkifli; Noor Syahida Azizan; Nur-Hidayana Mahfodz; Sazaly AbuBakar; Siti-Sarah Nor'e
EPI_ISL_8560083	Dengkil Health Clinic	Institute for Medical Research, Infectious Disease Research Centre, National Institutes of Health, Ministry of Health Malaysia	Ahmad FA; Ahmad Fazilah NA; Anasir MI; Azizan MA; Kamel K; Mohamad Sukri MZ; Norhisham SN; Ramly N; Robert F; Rosli NR; Suppiah J; Thayan R
EPI_ISL_8564948	Department of Medical Microbiology and Parasitology, Hospital Universiti Sains Malaysia	Molecular Research Laboratory	Abdul Haris bin Muhammad; Ahmad Adebayo Irekeola; Ahmad Sukari Bin Halim; Aswini Leela; Azian Harun; Chan Yean Yean; Chua Wei Chuan; Chua Wei Lian; Engku Nur Syafirah bt Engku Abd Rahman; Farahana binti Mohamed Alexander Chong Shu Chien; Kirnpal Kaur Banga Singh; Lau Nyok Sean; Lee Lih Huey; Lim Shu Yong; Maizun binti Mohd Zain; Muhammad Azamuddeen bin Mohammad Nasir; Muhammad Fazli bin Khalid; Muhammad Nashrul Farhan Samsudin; Muhammad Zarul Hanifah Bin Md Zoqratt; Naveed Ahmed; Nazmi Liana Binti Azmi; Noor Hafizan binti Mat Salleh; Nurfadhlina Musa; Qasim Ayub; Rosline Hassan; Sadequr Rahman; Syahida binti Omar; Wan Mohd Zahiruddin Wan Mohammad; Wardah Yusof; Wilhelm Eng Wei Han; Zaini bin Hussin; Zakuan Zainy Deris
EPI_ISL_10073375	Department of Medical Microbiology, Hospital Pengajar Universiti Putra Malaysia	Department of Medical Microbiology, Hospital Pengajar Universiti Putra Malaysia	Afiqah Adzmi; Amiza Azmi; Azmiza Syawani Jasni; Hui Yee Chee; Leslie Thian Lung Than; Muadz Mohtar; Muhammad Mohd Isa; Narcisse MS Joseph; Niazlin Mohd Taib; Noor Hazirah Noor Azhari; Norlaila; Nur Raihana Ithnin; Nurul Huda Mohamed Rashidi; Nurul Nadiah Ismail; Rosni Ibrahim; Rukman Awang Hamat; Sallehhudin; Siti Norbaya Masri; Siti Zulaikha Zakariah; Syafinaz Amin Nordin; Tengku Zetty Maztura Tengku Jamaluddin; Zamberi Sekawi
EPI_ISL_8650066, EPI_ISL_8650070, EPI_ISL_8650149	Gribbles Pathology, Kuching	Institute of Health and Community Medicine	Chan Chia Jui; Chua Hock Hin; David Perera; Liew Chin Chin J; Ooi Mong How; Tonnii Sia Loong Loong; Wong Jyn Shan
EPI_ISL_10652497	HOPSITAL SULTANAH NUR ZAHIRAH	Department of Medical Microbiology, Faculty of Medicine, University of Malaya; University of Malaya Medical Centre	I-Ching SAM; Jolene Yin Ling FU; Omar Khalilur Rahman; Yoke Fun Chan
EPI_ISL_10397083, EPI_ISL_10397108	HOSPITAL MELAKA	UKM Medical Molecular Biology Institute (UMBI)	Khairun Nur Abd Ghafar; Mira Farzana Mohamad Mokhtar; Muhiddin Ishak; Nor Azila Muhammad Azami; Nur Alyaa Afifah Md Shahri; Nurul Syakima Ab Mutalib; Rahman Jamal; Ryia Illani Mohd Yunos; Siti Nur Hasanah Mohd Yusuf; Zahirrah Begam Mohamed Rasheed
EPI_ISL_10652475, EPI_ISL_10652476, EPI_ISL_10652478, EPI_ISL_10652479	HOSPITAL RAJA PERMAISURI BAINUN	Department of Medical Microbiology, Faculty of Medicine, University of Malaya; University of Malaya Medical Centre	I-Ching SAM; Jolene Yin Ling FU; Omar Khalilur Rahman; Yoke Fun Chan
EPI_ISL_12534775	HOSPITAL RAJA PERMAISURI BAINUN, IPOH	Tropical Infectious Diseases Research & Education Centre (TIDREC), Universiti Malaya	AsmaAnati CheMatSeri; Che-Norainon Yaacob; Jia-Yi Tan; Jo-Ern Wong; Kim-Kee Tan; Mulya-Mustika-Sari Zulkifli; Noor Syahida Azizan; Nur-Hidayana Mahfodz; Sazaly AbuBakar; Siti-Sarah Nor'e; Wei-Wei Aw
EPI_ISL_10968989	HOSPITAL SULTANAH MALIHA	Tropical Infectious Diseases Research & Education Centre (TIDREC), Universiti Malaya	AsmaAnati CheMatSeri; Che-Norainon Yaacob; Jia-Yi Tan; Jo-Ern Wong; Kim-Kee Tan; Mulya-Mustika-Sari Zulkifli; Noor Syahida Azizan; Nur-Hidayana Mahfodz; Sazaly AbuBakar; Siti-Sarah Nor'e
EPI_ISL_10397081	HOSPITAL SULTANAH NORA ISMAIL	UKM Medical Molecular Biology Institute (UMBI)	Khairun Nur Abd Ghafar; Mira Farzana Mohamad Mokhtar; Muhiddin Ishak; Nor Azila Muhammad Azami; Nur Alyaa Afifah Md Shahri; Nurul Syakima Ab Mutalib; Rahman Jamal; Ryia Illani Mohd Yunos; Siti Nur Hasanah Mohd Yusuf; Zahirrah Begam Mohamed Rasheed
EPI_ISL_10397084	HOSPITAL SULTANAH NORA ISMAIL, BATU PAHAT	UKM Medical Molecular Biology Institute (UMBI)	Khairun Nur Abd Ghafar; Mira Farzana Mohamad Mokhtar; Muhiddin Ishak; Nor Azila Muhammad Azami; Nur Alyaa Afifah Md Shahri; Nurul Syakima Ab Mutalib; Rahman Jamal; Ryia Illani Mohd Yunos; Siti Nur Hasanah Mohd Yusuf; Zahirrah Begam Mohamed Rasheed
EPI_ISL_10652498, EPI_ISL_10652499, EPI_ISL_10652500	HOSPITAL SULTANAH NUR ZAHIRAH	Department of Medical Microbiology, Faculty of Medicine, University of Malaya; University of Malaya Medical Centre	I-Ching SAM; Jolene Yin Ling FU; Omar Khalilur Rahman; Yoke Fun Chan
EPI_ISL_9829852	HOSPITAL TAMPIN	Tropical Infectious Diseases Research & Education Centre (TIDREC), Universiti Malaya	AsmaAnati CheMatSeri; Che-Norainon Yaacob; Jia-Yi Tan; Jo-Ern Wong; Kim-Kee Tan; Mulya-Mustika-Sari Zulkifli; Noor Syahida Azizan; Nur-Hidayana Mahfodz; Sazaly AbuBakar; Siti-Sarah Nor'e
EPI_ISL_10397093, EPI_ISL_10397111	HOSPITAL TAMPIN	UKM Medical Molecular Biology Institute (UMBI)	Khairun Nur Abd Ghafar; Mira Farzana Mohamad Mokhtar; Muhiddin Ishak; Nor Azila Muhammad Azami; Nur Alyaa Afifah Md Shahri; Nurul Syakima Ab Mutalib; Rahman Jamal; Ryia Illani Mohd Yunos; Siti Nur Hasanah Mohd Yusuf; Zahirrah Begam Mohamed Rasheed
EPI_ISL_10495863	HOSPITAL TAWAU	Institute for Medical Research, Infectious Disease Research Centre, National Institutes of Health, Ministry of Health Malaysia	Ahmad FA; Ahmad Fazilah NA; Anasir MI; Arip MS; Azizan MA; Christopher Hollip K; Jaimin JJ; Kamel K; Mohamad Sukri MZ; Mohammad R; Mohd Yasin R; Norhisham SN; Ramly N; Robert F; Rosli NR; Shamsaddin E; Suppiah J; Tan YC; Thayan R
EPI_ISL_12099541	HPSF MUAR	UKM Medical Molecular Biology Institute (UMBI)	Khairun Nur Abd Ghafar; Mira Farzana Mohamad Mokhtar; Muhiddin Ishak; Nor Azila Muhammad Azami; Nur Alyaa Afifah Md Shahri; Nurul Syakima Ab Mutalib; Rahman Jamal; Ryia Illani Mohd Yunos; Siti Nur Hasanah Mohd Yusuf; Zahirrah Begam Mohamed Rasheed
EPI_ISL_9829352, EPI_ISL_10397052, EPI_ISL_10397064	HSAJB	UKM Medical Molecular Biology Institute (UMBI)	Khairun Nur Abd Ghafar; Mira Farzana Mohamad Mokhtar; Muhiddin Ishak; Nor Azila Muhammad Azami; Nur Alyaa Afifah Md Shahri; Nurul Syakima Ab Mutalib; Rahman Jamal; Ryia Illani Mohd Yunos; Siti Nur Hasanah Mohd Yusuf; Zahirrah Begam Mohamed Rasheed
EPI_ISL_10652474, EPI_ISL_10652477	HSOPITAL RAJA PERMAISURI BAINUN	Department of Medical Microbiology, Faculty of Medicine, University of Malaya; University of Malaya Medical Centre	I-Ching SAM; Jolene Yin Ling FU; Omar Khalilur Rahman; Yoke Fun Chan
EPI_ISL_9829344, EPI_ISL_9829345, EPI_ISL_9829376, EPI_ISL_10397065, EPI_ISL_10397092, EPI_ISL_10397098, EPI_ISL_10397118			
see above	HTJS	UKM Medical Molecular Biology Institute (UMBI)	Khairun Nur Abd Ghafar; Mira Farzana Mohamad Mokhtar; Muhiddin Ishak; Nor Azila Muhammad Azami; Nur Alyaa Afifah Md Shahri; Nurul Syakima Ab Mutalib; Rahman Jamal; Ryia Illani Mohd Yunos; Siti Nur Hasanah Mohd Yusuf; Zahirrah Begam Mohamed Rasheed
EPI_ISL_10590720, EPI_ISL_11899942, EPI_ISL_11899974, EPI_ISL_11899975	Hospital Ampang	Malaysia Genome and Vaccine Institute	Azrin Ahmad; Enizza Kasim; Irni Suhayu Sapian; Mohd Faizal Abu Bakar; Mohd Ghows Mohd Azzam.; Mohd Noor Mat Isa; Nor Azfa Johari; Nurhezreen Md Iqbal; Shamsidar Sopie; Siti Noraini Othman; Yusuf Muhammad Noor
EPI_ISL_8314874	Hospital Canselor Tuanku Muhriz (HCTM)	UKM Medical Molecular Biology Institute (UMBI)	Khairun Nur Abd Ghafar; Mira Farzana Mohamad Mokhtar; Muhiddin Ishak; Nor Azila Muhammad Azami; Nur Alyaa Afifah Md Shahri; Nurul Syakima Ab Mutalib; Rahman Jamal; Ryia Illani Mohd Yunos; Siti Nur Hasanah Mohd Yusuf; Zahirrah Begam Mohamed Rasheed
EPI_ISL_10590732, EPI_ISL_10590733, EPI_ISL_11899923, EPI_ISL_11899946, EPI_ISL_11899947	Hospital Kuala Lumpur	Malaysia Genome and Vaccine Institute	Azrin Ahmad; Enizza Kasim; Irni Suhayu Sapian; Mohd Faizal Abu Bakar; Mohd Ghows Mohd Azzam.; Mohd Noor Mat Isa; Nor Azfa Johari; Nurhezreen Md Iqbal; Shamsidar Sopie; Siti Noraini Othman; Yusuf Muhammad Noor
EPI_ISL_10397082	Hospital Melaka	UKM Medical Molecular Biology Institute (UMBI)	Khairun Nur Abd Ghafar; Mira Farzana Mohamad Mokhtar; Muhiddin Ishak; Nor Azila Muhammad Azami; Nur Alyaa Afifah Md Shahri; Nurul Syakima Ab Mutalib; Rahman Jamal; Ryia Illani Mohd Yunos; Siti Nur Hasanah Mohd Yusuf; Zahirrah Begam Mohamed Rasheed
EPI_ISL_10397104	Hospital Pakar Sultanah Fatimah Muar Johor	UKM Medical Molecular Biology Institute (UMBI)	Khairun Nur Abd Ghafar; Mira Farzana Mohamad Mokhtar; Muhiddin Ishak; Nor Azila Muhammad Azami; Nur Alyaa Afifah Md Shahri; Nurul Syakima Ab Mutalib; Rahman Jamal; Ryia Illani Mohd Yunos; Siti Nur Hasanah Mohd Yusuf; Zahirrah Begam Mohamed Rasheed

EPI_ISL_11112425, EPI_ISL_11112426, EPI_ISL_11112427, EPI_ISL_11112428, EPI_ISL_11112429, EPI_ISL_11112432, EPI_ISL_11112433			
see above	Hospital Pulau Pinang	iPROMISE, UiTM	Ariza Adnan; Fadzilah Mohd Nor; Lim Wai Feng; Mohd Asif Mohd Sukri; Mohd Nur Fakhruzzaman Noorizhab; Mohd Zaki Salleh; Sazzli Shahlan Kassim; Siti Farah Alwani Mohd Nawi; Siti Hamimah Sheikh Abdul Kadir; Teh Lay Kek; Wang Seok Mui
EPI_ISL_11900054	Hospital Putrajaya	Malaysia Genome and Vaccine Institute	Azrin Ahmad; Enizza Kasim; Irni Suhayu Sapian; Mohd Faizal Abu Bakar; Mohd Ghows Mohd Azzam.; Mohd Noor Mat Isa; Nor Azfa Johari; Nurhezreen Md Iqbal; Shamsidar Sopie; Siti Noraini Othman; Yusuf Muhammad Noor
EPI_ISL_10397066	Hospital Sultan Ismail	UKM Medical Molecular Biology Institute (UMBI)	Khairun Nur Abd Ghafar; Mira Farzana Mohamad Mokhtar; Muhiddin Ishak; Nor Azila Muhammad Azami; Nur Alyaa Afifah Md Shahri; Nurul Syakima Ab Mutalib; Rahman Jamal; Ryia Illani Mohd Yunos; Siti Nur Hasanah Mohd Yusuf; Zahirrah Begam Mohamed Rasheed
EPI_ISL_10397053, EPI_ISL_10397117	Hospital Sultanah Aminah Johor Bahru	UKM Medical Molecular Biology Institute (UMBI)	Khairun Nur Abd Ghafar; Mira Farzana Mohamad Mokhtar; Muhiddin Ishak; Nor Azila Muhammad Azami; Nur Alyaa Afifah Md Shahri; Nurul Syakima Ab Mutalib; Rahman Jamal; Ryia Illani Mohd Yunos; Siti Nur Hasanah Mohd Yusuf; Zahirrah Begam Mohamed Rasheed
EPI_ISL_10397068	Hospital Sultanah Nora Ismail	UKM Medical Molecular Biology Institute (UMBI)	Khairun Nur Abd Ghafar; Mira Farzana Mohamad Mokhtar; Muhiddin Ishak; Nor Azila Muhammad Azami; Nur Alyaa Afifah Md Shahri; Nurul Syakima Ab Mutalib; Rahman Jamal; Ryia Illani Mohd Yunos; Siti Nur Hasanah Mohd Yusuf; Zahirrah Begam Mohamed Rasheed
EPI_ISL_10397110	Hospital Sultanah Nora Ismail, Johor	UKM Medical Molecular Biology Institute (UMBI)	Khairun Nur Abd Ghafar; Mira Farzana Mohamad Mokhtar; Muhiddin Ishak; Nor Azila Muhammad Azami; Nur Alyaa Afifah Md Shahri; Nurul Syakima Ab Mutalib; Rahman Jamal; Ryia Illani Mohd Yunos; Siti Nur Hasanah Mohd Yusuf; Zahirrah Begam Mohamed Rasheed
EPI_ISL_11899950, EPI_ISL_11899953, EPI_ISL_11899955, EPI_ISL_11899976, EPI_ISL_11899980, EPI_ISL_11899984	Hospital Sungai Buloh	Malaysia Genome and Vaccine Institute	Azrin Ahmad; Enizza Kasim; Irni Suhayu Sapian; Mohd Faizal Abu Bakar; Mohd Ghows Mohd Azzam.; Mohd Noor Mat Isa; Nor Azfa Johari; Nurhezreen Md Iqbal; Shamsidar Sopie; Siti Noraini Othman; Yusuf Muhammad Noor
EPI_ISL_10652517	Hospital Teluk Intan	Department of Medical Microbiology, Faculty of Medicine, University of Malaya; University of Malaya Medical Centre	I-Ching SAM; Jolene Yin Ling FU; Omar Khalilur Rahman; Yoke Fun Chan
EPI_ISL_9829354	Hospital Tuanku Ja'afar Seremban	UKM Medical Molecular Biology Institute (UMBI)	Khairun Nur Abd Ghafar; Mira Farzana Mohamad Mokhtar; Muhiddin Ishak; Nor Azila Muhammad Azami; Nur Alyaa Afifah Md Shahri; Nurul Syakima Ab Mutalib; Rahman Jamal; Ryia Illani Mohd Yunos; Siti Nur Hasanah Mohd Yusuf; Zahirrah Begam Mohamed Rasheed
EPI_ISL_10397107	Hospital Tuanku Jaafar	UKM Medical Molecular Biology Institute (UMBI)	Khairun Nur Abd Ghafar; Mira Farzana Mohamad Mokhtar; Muhiddin Ishak; Nor Azila Muhammad Azami; Nur Alyaa Afifah Md Shahri; Nurul Syakima Ab Mutalib; Rahman Jamal; Ryia Illani Mohd Yunos; Siti Nur Hasanah Mohd Yusuf; Zahirrah Begam Mohamed Rasheed
EPI_ISL_10397121	Hospital sultanah nora ismail	UKM Medical Molecular Biology Institute (UMBI)	Khairun Nur Abd Ghafar; Mira Farzana Mohamad Mokhtar; Muhiddin Ishak; Nor Azila Muhammad Azami; Nur Alyaa Afifah Md Shahri; Nurul Syakima Ab Mutalib; Rahman Jamal; Ryia Illani Mohd Yunos; Siti Nur Hasanah Mohd Yusuf; Zahirrah Begam Mohamed Rasheed
EPI_ISL_11323753, EPI_ISL_11323766, EPI_ISL_11323774, EPI_ISL_11323787, EPI_ISL_11323804, EPI_ISL_11323809	Institut Biologi Molekul Perubatan UKM (UMBI)	UKM Medical Molecular Biology Institute (UMBI)	Khairun Nur Abd Ghafar; Mira Farzana Mohamad Mokhtar; Muhiddin Ishak; Nor Azila Muhammad Azami; Nur Alyaa Afifah Md Shahri; Nurul Syakima Ab Mutalib; Rahman Jamal; Ryia Illani Mohd Yunos; Siti Nur Hasanah Mohd Yusuf; Zahirrah Begam Mohamed Rasheed
EPI_ISL_10969048, EPI_ISL_10969049, EPI_ISL_10969050, EPI_ISL_10969051	JABATAN PATOLOGI HOSPITAL RAJA PEREMPUAN ZAINAB II	Tropical Infectious Diseases Research & Education Centre (TIDREC), Universiti Malaya	AsmaAnati CheMatSeri; Che-Norainon Yaacob; Jia-Yi Tan; Jo-Ern Wong; Kim-Kee Tan; Mulya-Mustika-Sari Zulkifli; Noor Syahida Azizan; Nur-Hidayana Mahfodz; Sazaly AbuBakar; Siti-Sarah Nor’e
EPI_ISL_10968985, EPI_ISL_10968986, EPI_ISL_10968987, EPI_ISL_10969012, EPI_ISL_10969042, EPI_ISL_10969043, EPI_ISL_10969044, EPI_ISL_10969045, EPI_ISL_10969046, EPI_ISL_10969047, EPI_ISL_12534825, EPI_ISL_12534841, EPI_ISL_12534842			
see above	JABATAN PATOLOGI, HOSPITAL RAJA PEREMPUAN ZAINAB II	Tropical Infectious Diseases Research & Education Centre (TIDREC), Universiti Malaya	AsmaAnati CheMatSeri; Che-Norainon Yaacob; Jia-Yi Tan; Jo-Ern Wong; Kim-Kee Tan; Mulya-Mustika-Sari Zulkifli; Noor Syahida Azizan; Nur-Hidayana Mahfodz; Sazaly AbuBakar; Siti-Sarah Nor’e; Wei-Wei Aw
EPI_ISL_9579168, EPI_ISL_9579169, EPI_ISL_9579174, EPI_ISL_9579175, EPI_ISL_9579176, EPI_ISL_9579177, EPI_ISL_9579178, EPI_ISL_9579179, EPI_ISL_9579180, EPI_ISL_9579201, EPI_ISL_9579204, EPI_ISL_11112397, EPI_ISL_11112399, EPI_ISL_11112400, EPI_ISL_11112402, EPI_ISL_11112403, EPI_ISL_11112404, EPI_ISL_11112407, EPI_ISL_11112457, EPI_ISL_11112458, EPI_ISL_11112459, EPI_ISL_11112460, EPI_ISL_11112461, EPI_ISL_11112462, EPI_ISL_11112463, EPI_ISL_11112464, EPI_ISL_11112482			
see above	JKN PAHANG	iPROMISE, UiTM	Ariza Adnan; Fadzilah Mohd Nor; Lim Wai Feng; Mohd Asif Mohd Sukri; Mohd Nur Fakhruzzaman Noorizhab; Mohd Zaki Salleh; Sazzli Shahlan Kassim; Siti Farah Alwani Mohd Nawi; Siti Hamimah Sheikh Abdul Kadir; Teh Lay Kek; Wang Seok Mui
EPI_ISL_9579203, EPI_ISL_11112388, EPI_ISL_11112389, EPI_ISL_11112390, EPI_ISL_11112391, EPI_ISL_11112398, EPI_ISL_11112401, EPI_ISL_11112405, EPI_ISL_11112406, EPI_ISL_11112437			
see above	Jabatan Kesihatan Negeri	iPROMISE, UiTM	Ariza Adnan; Fadzilah Mohd Nor; Lim Wai Feng; Mohd Asif Mohd Sukri; Mohd Nur Fakhruzzaman Noorizhab; Mohd Zaki Salleh; Sazzli Shahlan Kassim; Siti Farah Alwani Mohd Nawi; Siti Hamimah Sheikh Abdul Kadir; Teh Lay Kek; Wang Seok Mui
EPI_ISL_10810892	KENANGA 3 HSAH	Tropical Infectious Diseases Research & Education Centre (TIDREC), Universiti Malaya	AsmaAnati CheMatSeri; Che-Norainon Yaacob; Jia-Yi Tan; Jo-Ern Wong; Kim-Kee Tan; Mulya-Mustika-Sari Zulkifli; Noor Syahida Azizan; Nur-Hidayana Mahfodz; Sazaly AbuBakar; Siti-Sarah Nor’e
EPI_ISL_10495879	KINABATANGAN AREA HEALTH OFFICE	Institute for Medical Research, Infectious Disease Research Centre, National Institutes of Health, Ministry of Health Malaysia	Ahmad FA; Ahmad Fazilah NA; Anasir MI; Arip MS; Azizan MA; Christopher Hollip K; Jaimin JJ; Kamel K; Mohamad Sukri MZ; Mohammad R; Mohd Yasin R; Norhisham SN; Ramly N; Robert F; Rosli NR; Shamsaddin E; Suppiah J; Tan YC; Thayan R
EPI_ISL_10968981, EPI_ISL_10969011, EPI_ISL_10969041	KLINIK AMARA KOTA BHARU	Tropical Infectious Diseases Research & Education Centre (TIDREC), Universiti Malaya	AsmaAnati CheMatSeri; Che-Norainon Yaacob; Jia-Yi Tan; Jo-Ern Wong; Kim-Kee Tan; Mulya-Mustika-Sari Zulkifli; Noor Syahida Azizan; Nur-Hidayana Mahfodz; Sazaly AbuBakar; Siti-Sarah Nor’e
EPI_ISL_11754720, EPI_ISL_11754722, EPI_ISL_11754723, EPI_ISL_11754724	KOMPLEKS SURVELAN PKD KINTA	Department of Medical Microbiology, Faculty of Medicine, University of Malaya; University of Malaya Medical Centre	I-Ching SAM; Jolene Yin Ling FU; Omar Khalilur Rahman; Yoke Fun Chan
EPI_ISL_10297336, EPI_ISL_10297342, EPI_ISL_10297346, EPI_ISL_10297347	Kota Kinabalu Health Clinic	Institute for Medical Research, Infectious Disease Research Centre, National Institutes of Health, Ministry of Health Malaysia	Ahmad FA; Ahmad Fazilah NA; Anasir MI; Azizan MA; Kamel K; Mohamad Sukri MZ; Norhisham SN; Ramly N; Robert F; Rosli NR; Suppiah J; Thayan R
EPI_ISL_8767248, EPI_ISL_8767249, EPI_ISL_8767250, EPI_ISL_8767251, EPI_ISL_8767252, EPI_ISL_8767253, EPI_ISL_8767254, EPI_ISL_8767255, EPI_ISL_8767256, EPI_ISL_8767257, EPI_ISL_8767258, EPI_ISL_8767259, EPI_ISL_8767260, EPI_ISL_8767261, EPI_ISL_8767262, EPI_ISL_8767263, EPI_ISL_8767264, EPI_ISL_8767265, EPI_ISL_8767266, EPI_ISL_8767267, EPI_ISL_8767268, EPI_ISL_8767269, EPI_ISL_8767270, EPI_ISL_8767271, EPI_ISL_8767272, EPI_ISL_8767273, EPI_ISL_8767274, EPI_ISL_8767275, EPI_ISL_8767276, EPI_ISL_8767277, EPI_ISL_8767278, EPI_ISL_8767279, EPI_ISL_8767280, EPI_ISL_8767281, EPI_ISL_8767282, EPI_ISL_8767283, EPI_ISL_8767284, EPI_ISL_8767285, EPI_ISL_8767286, EPI_ISL_8767287, EPI_ISL_8767288, EPI_ISL_8767289, EPI_ISL_8767290, EPI_ISL_8767291, EPI_ISL_8767292, EPI_ISL_8767293, EPI_ISL_8767294, EPI_ISL_8767295, EPI_ISL_8767296, EPI_ISL_8767297, EPI_ISL_8767298, EPI_ISL_8767299, EPI_ISL_8767300, EPI_ISL_8767301, EPI_ISL_8767302, EPI_ISL_8767303, EPI_ISL_8767304, EPI_ISL_8767305, EPI_ISL_8767306, EPI_ISL_8767307, EPI_ISL_8767308, EPI_ISL_8767309, EPI_ISL_8767310, EPI_ISL_8767311, EPI_ISL_8767312, EPI_ISL_8767313, EPI_ISL_8767314, EPI_ISL_8767315, EPI_ISL_8767316, EPI_ISL_8767317, EPI_ISL_8767318, EPI_ISL_8767319, EPI_ISL_8767320			
see above	Kuala Lumpur Internation Airport (KLIA) Health Office	Department of Medical Microbiology, Faculty of Medicine, University of Malaya; University of Malaya Medical Centre	I-Ching SAM; Jolene Yin Ling FU; Omar Khalilur Rahman; Yoke Fun Chan
EPI_ISL_8560035, EPI_ISL_8560042, EPI_ISL_8560049, EPI_ISL_8560056, EPI_ISL_8560062, EPI_ISL_8560069, EPI_ISL_8560076, EPI_ISL_8560084, EPI_ISL_8560091, EPI_ISL_8560092, EPI_ISL_8560100, EPI_ISL_8560106, EPI_ISL_8560112, EPI_ISL_8560971, EPI_ISL_8690777, EPI_ISL_8690899, EPI_ISL_9092710, EPI_ISL_9092711, EPI_ISL_9095236, EPI_ISL_9095237, EPI_ISL_9095238, EPI_ISL_9095239, EPI_ISL_9095240, EPI_ISL_9095241, EPI_ISL_9095242, EPI_ISL_9095243, EPI_ISL_9095244, EPI_ISL_9095245, EPI_ISL_9095246, EPI_ISL_9095247, EPI_ISL_9095248, EPI_ISL_9095249, EPI_ISL_9095250, EPI_ISL_9095251, EPI_ISL_9095252, EPI_ISL_9095253, EPI_ISL_9095254, EPI_ISL_9095255, EPI_ISL_9095256, EPI_ISL_9095257, EPI_ISL_9095258, EPI_ISL_9095259, EPI_ISL_9095260, EPI_ISL_9095261, EPI_ISL_9095262, EPI_ISL_9095263, EPI_ISL_9832497, EPI_ISL_9832498, EPI_ISL_9842678, EPI_ISL_9842679, EPI_ISL_9842680			
see above	Kuala Lumpur International Airport (KLIA) Health Office	Institute for Medical Research, Infectious Disease Research Centre, National Institutes of Health, Ministry of Health Malaysia	Ahmad FA; Ahmad Fazilah NA; Anasir MI; Azizan MA; Kamel K; Mohamad Sukri MZ; Norhisham SN; Ramly N; Robert F; Rosli NR; Suppiah J; Thayan R
EPI_ISL_10652541, EPI_ISL_10652543, EPI_ISL_10652544, EPI_ISL_11754709, EPI_ISL_11754725, EPI_ISL_11754726, EPI_ISL_11754727, EPI_ISL_11754728, EPI_ISL_11754729, EPI_ISL_11754730, EPI_ISL_11754731, EPI_ISL_11754761, EPI_ISL_11754775			
see above	MAKMAL KESIHATAN AWAM KOTA BHARU	Department of Medical Microbiology, Faculty of Medicine, University of Malaya; University of Malaya Medical Centre	I-Ching SAM; Jolene Yin Ling FU; Omar Khalilur Rahman; Yoke Fun Chan
EPI_ISL_10397059, EPI_ISL_10397116	MEDICAL HOSPITAL SULTANAH NORA ISMAIL	UKM Medical Molecular Biology Institute (UMBI)	Khairun Nur Abd Ghafar; Mira Farzana Mohamad Mokhtar; Muhiddin Ishak; Nor Azila Muhammad Azami; Nur Alyaa Afifah Md Shahri; Nurul Syakima Ab Mutalib; Rahman Jamal; Ryia Illani Mohd Yunos; Siti Nur Hasanah Mohd Yusuf; Zahirrah Begam Mohamed Rasheed
EPI_ISL_11754735, EPI_ISL_11754736	MKA KB	Department of Medical Microbiology, Faculty of Medicine, University of Malaya; University of Malaya Medical Centre	I-Ching SAM; Jolene Yin Ling FU; Omar Khalilur Rahman; Yoke Fun Chan
EPI_ISL_9579183	Medical Hospital Pulau Pinang	iPROMISE, UiTM	Ariza Adnan; Fadzilah Mohd Nor; Lim Wai Feng; Mohd Asif Mohd Sukri; Mohd Nur Fakhruzzaman Noorizhab; Mohd Zaki Salleh; Sazzli Shahlan Kassim; Siti Farah Alwani Mohd Nawi; Siti Hamimah Sheikh Abdul Kadir; Teh Lay Kek; Wang Seok Mui
EPI_ISL_8650097, EPI_ISL_8650108, EPI_ISL_8650109, EPI_ISL_8650110	Normah Medical Specialist Centre, Kuching	Institute of Health and Community Medicine	Chan Chia Jui; Chua Hock Hin; David Perera; Ikwannuddin Y; Ooi Mong How; Tonnii Sia Loong Loong; Wong Jyn Shan
EPI_ISL_10652494	PEJABAT KESIHATAN DAERAH KUALA KRAI	Department of Medical Microbiology, Faculty of Medicine, University of Malaya; University of Malaya Medical Centre	I-Ching SAM; Jolene Yin Ling FU; Omar Khalilur Rahman; Yoke Fun Chan
EPI_ISL_11112440, EPI_ISL_11112444, EPI_ISL_11112449, EPI_ISL_11112452, EPI_ISL_11939094, EPI_ISL_11939099	PEJABAT KESIHATAN DAERAH SEBERANG PERAI UTARA PULAU PINANG	iPROMISE, UiTM	Ariza Adnan; Fadzilah Mohd Nor; Lim Wai Feng; Mohd Asif Mohd Sukri; Mohd Nur Fakhruzzaman Noorizhab; Mohd Zaki Salleh; Sazzli Shahlan Kassim; Siti Farah Alwani Mohd Nawi; Siti Hamimah Sheikh Abdul Kadir; Teh Lay Kek; Wang Seok Mui
EPI_ISL_11112470, EPI_ISL_11112471, EPI_ISL_11112472	PEJABAT KESIHATAN DAERAH TIMUR LAUT	iPROMISE, UiTM	Ariza Adnan; Fadzilah Mohd Nor; Lim Wai Feng; Mohd Asif Mohd Sukri; Mohd Nur Fakhruzzaman Noorizhab; Mohd Zaki Salleh; Sazzli Shahlan Kassim; Siti Farah Alwani Mohd Nawi; Siti Hamimah Sheikh Abdul Kadir; Teh Lay Kek; Wang Seok Mui
EPI_ISL_12534874	PEJABAT KESIHATAN DAERAH YAN	Tropical Infectious Diseases Research & Education Centre (TIDREC), Universiti Malaya	AsmaAnati CheMatSeri; Che-Norainon Yaacob; Jia-Yi Tan; Jo-Ern Wong; Kim-Kee Tan; Mulya-Mustika-Sari Zulkifli; Noor Syahida Azizan; Nur-Hidayana Mahfodz; Sazaly AbuBakar; Siti-Sarah Nor’e; Wei-Wei Aw

EPI_ISL_10397085	PEJABAT KESIHATAN PONTIAN	UKM Medical Molecular Biology Institute (UMBI)	Khairun Nur Abd Ghafar; Mira Farzana Mohamad Mokhtar; Muhiddin Ishak; Nor Azila Muhammad Azami; Nur Alyaa Afifah Md Shahri; Nurul Syakima Ab Mutalib; Rahman Jamal; Ryia Illani Mohd Yunos; Siti Nur Hasanah Mohd Yusuf; Zahirrah Begam Mohamed Rasheed
EPI_ISL_11754743	PKD BACHOK	Department of Medical Microbiology, Faculty of Medicine, University of Malaya; University of Malaya Medical Centre	I-Ching SAM; Jolene Yin Ling FU; Omar Khalilur Rahman; Yoke Fun Chan
EPI_ISL_11112431	PKD BARAT DAYA	iPROMISE, UiTM	Ariza Adnan; Fadzilah Mohd Nor; Lim Wai Feng; Mohd Asif Mohd Sukri; Mohd Nur Fakhruzzaman Noorizhab; Mohd Zaki Salleh; Sazzli Shahlan Kassim; Siti Farah Alwani Mohd Nawi; Siti Hamimah Sheikh Abdul Kadir; Teh Lay Kek; Wang Seok Mui
EPI_ISL_10652492	PKD Bachok	Department of Medical Microbiology, Faculty of Medicine, University of Malaya; University of Malaya Medical Centre	I-Ching SAM; Jolene Yin Ling FU; Omar Khalilur Rahman; Yoke Fun Chan
EPI_ISL_10652501, EPI_ISL_10652502, EPI_ISL_10652518, EPI_ISL_10652519, EPI_ISL_10652520, EPI_ISL_11754762	PKD HILIR PERAK	Department of Medical Microbiology, Faculty of Medicine, University of Malaya; University of Malaya Medical Centre	I-Ching SAM; Jolene Yin Ling FU; Omar Khalilur Rahman; Yoke Fun Chan
EPI_ISL_12534787	PKD HILIR PERAK	Tropical Infectious Diseases Research & Education Centre (TIDREC), Universiti Malaya	AsmaAnati CheMatSeri; Che-Norainon Yaacob; Jia-Yi Tan; Jo-Ern Wong; Kim-Kee Tan; Mulya-Mustika-Sari Zulkifli; Noor Syahida Azizan; Nur-Hidayana Mahfodz; Sazaly AbuBakar; Siti-Sarah Nor'e; Wei-Wei Aw
EPI_ISL_10397102	PKD JEMPOL	UKM Medical Molecular Biology Institute (UMBI)	Khairun Nur Abd Ghafar; Mira Farzana Mohamad Mokhtar; Muhiddin Ishak; Nor Azila Muhammad Azami; Nur Alyaa Afifah Md Shahri; Nurul Syakima Ab Mutalib; Rahman Jamal; Ryia Illani Mohd Yunos; Siti Nur Hasanah Mohd Yusuf; Zahirrah Begam Mohamed Rasheed
EPI_ISL_10397106, EPI_ISL_10397123, EPI_ISL_10549453, EPI_ISL_10549457, EPI_ISL_10549463, EPI_ISL_10549470, EPI_ISL_10549472, EPI_ISL_10549475			
see above	PKD JOHOR BAHRU	UKM Medical Molecular Biology Institute (UMBI)	Khairun Nur Abd Ghafar; Mira Farzana Mohamad Mokhtar; Muhiddin Ishak; Nor Azila Muhammad Azami; Nur Alyaa Afifah Md Shahri; Nurul Syakima Ab Mutalib; Rahman Jamal; Ryia Illani Mohd Yunos; Siti Nur Hasanah Mohd Yusuf; Zahirrah Begam Mohamed Rasheed
EPI_ISL_11754748	PKD KERIAN	Department of Medical Microbiology, Faculty of Medicine, University of Malaya; University of Malaya Medical Centre	I-Ching SAM; Jolene Yin Ling FU; Omar Khalilur Rahman; Yoke Fun Chan
EPI_ISL_12534780, EPI_ISL_12534782, EPI_ISL_12534783, EPI_ISL_12534786	PKD KERIAN	Tropical Infectious Diseases Research & Education Centre (TIDREC), Universiti Malaya	AsmaAnati CheMatSeri; Che-Norainon Yaacob; Jia-Yi Tan; Jo-Ern Wong; Kim-Kee Tan; Mulya-Mustika-Sari Zulkifli; Noor Syahida Azizan; Nur-Hidayana Mahfodz; Sazaly AbuBakar; Siti-Sarah Nor'e; Wei-Wei Aw
EPI_ISL_9317738, EPI_ISL_10397062, EPI_ISL_10397091	PKD KLUANG	UKM Medical Molecular Biology Institute (UMBI)	Khairun Nur Abd Ghafar; Mira Farzana Mohamad Mokhtar; Muhiddin Ishak; Nor Azila Muhammad Azami; Nur Alyaa Afifah Md Shahri; Nurul Syakima Ab Mutalib; Rahman Jamal; Ryia Illani Mohd Yunos; Siti Nur Hasanah Mohd Yusuf; Zahirrah Begam Mohamed Rasheed
EPI_ISL_11754750	PKD KOTA BHARU	Department of Medical Microbiology, Faculty of Medicine, University of Malaya; University of Malaya Medical Centre	I-Ching SAM; Jolene Yin Ling FU; Omar Khalilur Rahman; Yoke Fun Chan
EPI_ISL_10397058, EPI_ISL_10397069, EPI_ISL_10397074, EPI_ISL_10397122	PKD KOTA TINGGI	UKM Medical Molecular Biology Institute (UMBI)	Khairun Nur Abd Ghafar; Mira Farzana Mohamad Mokhtar; Muhiddin Ishak; Nor Azila Muhammad Azami; Nur Alyaa Afifah Md Shahri; Nurul Syakima Ab Mutalib; Rahman Jamal; Ryia Illani Mohd Yunos; Siti Nur Hasanah Mohd Yusuf; Zahirrah Begam Mohamed Rasheed
EPI_ISL_10652506, EPI_ISL_11754752, EPI_ISL_11754766	PKD KUALA KANGSAR	Department of Medical Microbiology, Faculty of Medicine, University of Malaya; University of Malaya Medical Centre	I-Ching SAM; Jolene Yin Ling FU; Omar Khalilur Rahman; Yoke Fun Chan
EPI_ISL_12534785	PKD KUALA KANGSAR	Tropical Infectious Diseases Research & Education Centre (TIDREC), Universiti Malaya	AsmaAnati CheMatSeri; Che-Norainon Yaacob; Jia-Yi Tan; Jo-Ern Wong; Kim-Kee Tan; Mulya-Mustika-Sari Zulkifli; Noor Syahida Azizan; Nur-Hidayana Mahfodz; Sazaly AbuBakar; Siti-Sarah Nor'e; Wei-Wei Aw
EPI_ISL_9829383, EPI_ISL_10397070, EPI_ISL_10397078, EPI_ISL_10397101	PKD KUALA PILAH	UKM Medical Molecular Biology Institute (UMBI)	Khairun Nur Abd Ghafar; Mira Farzana Mohamad Mokhtar; Muhiddin Ishak; Nor Azila Muhammad Azami; Nur Alyaa Afifah Md Shahri; Nurul Syakima Ab Mutalib; Rahman Jamal; Ryia Illani Mohd Yunos; Siti Nur Hasanah Mohd Yusuf; Zahirrah Begam Mohamed Rasheed
EPI_ISL_10397050, EPI_ISL_10397063	PKD KULAI	UKM Medical Molecular Biology Institute (UMBI)	Khairun Nur Abd Ghafar; Mira Farzana Mohamad Mokhtar; Muhiddin Ishak; Nor Azila Muhammad Azami; Nur Alyaa Afifah Md Shahri; Nurul Syakima Ab Mutalib; Rahman Jamal; Ryia Illani Mohd Yunos; Siti Nur Hasanah Mohd Yusuf; Zahirrah Begam Mohamed Rasheed
EPI_ISL_9317728, EPI_ISL_9317743, EPI_ISL_10397056, EPI_ISL_10397090, EPI_ISL_10549454, EPI_ISL_10549460, EPI_ISL_10549461			
see above	PKD MELAKA TENGAH	UKM Medical Molecular Biology Institute (UMBI)	Khairun Nur Abd Ghafar; Mira Farzana Mohamad Mokhtar; Muhiddin Ishak; Nor Azila Muhammad Azami; Nur Alyaa Afifah Md Shahri; Nurul Syakima Ab Mutalib; Rahman Jamal; Ryia Illani Mohd Yunos; Siti Nur Hasanah Mohd Yusuf; Zahirrah Begam Mohamed Rasheed
EPI_ISL_10652480, EPI_ISL_10652482	PKD PASIR PUTEH	Department of Medical Microbiology, Faculty of Medicine, University of Malaya; University of Malaya Medical Centre	I-Ching SAM; Jolene Yin Ling FU; Omar Khalilur Rahman; Yoke Fun Chan
EPI_ISL_10652504, EPI_ISL_10652505, EPI_ISL_10652521, EPI_ISL_10652522	PKD PERAK TENGAH	Department of Medical Microbiology, Faculty of Medicine, University of Malaya; University of Malaya Medical Centre	I-Ching SAM; Jolene Yin Ling FU; Omar Khalilur Rahman; Yoke Fun Chan
EPI_ISL_10397051, EPI_ISL_10397075, EPI_ISL_10397100, EPI_ISL_10397113	PKD REMBAU	UKM Medical Molecular Biology Institute (UMBI)	Khairun Nur Abd Ghafar; Mira Farzana Mohamad Mokhtar; Muhiddin Ishak; Nor Azila Muhammad Azami; Nur Alyaa Afifah Md Shahri; Nurul Syakima Ab Mutalib; Rahman Jamal; Ryia Illani Mohd Yunos; Siti Nur Hasanah Mohd Yusuf; Zahirrah Begam Mohamed Rasheed
EPI_ISL_10397095	PKD Rembau	UKM Medical Molecular Biology Institute (UMBI)	Khairun Nur Abd Ghafar; Mira Farzana Mohamad Mokhtar; Muhiddin Ishak; Nor Azila Muhammad Azami; Nur Alyaa Afifah Md Shahri; Nurul Syakima Ab Mutalib; Rahman Jamal; Ryia Illani Mohd Yunos; Siti Nur Hasanah Mohd Yusuf; Zahirrah Begam Mohamed Rasheed
EPI_ISL_11112409	PKD SEBERANG PERAI SELATAN	iPROMISE, UiTM	Ariza Adnan; Fadzilah Mohd Nor; Lim Wai Feng; Mohd Asif Mohd Sukri; Mohd Nur Fakhruzzaman Noorizhab; Mohd Zaki Salleh; Sazzli Shahlan Kassim; Siti Farah Alwani Mohd Nawi; Siti Hamimah Sheikh Abdul Kadir; Teh Lay Kek; Wang Seok Mui
EPI_ISL_10397079, EPI_ISL_10397080, EPI_ISL_10397096, EPI_ISL_10397109	PKD SEGAMAT	UKM Medical Molecular Biology Institute (UMBI)	Khairun Nur Abd Ghafar; Mira Farzana Mohamad Mokhtar; Muhiddin Ishak; Nor Azila Muhammad Azami; Nur Alyaa Afifah Md Shahri; Nurul Syakima Ab Mutalib; Rahman Jamal; Ryia Illani Mohd Yunos; Siti Nur Hasanah Mohd Yusuf; Zahirrah Begam Mohamed Rasheed
EPI_ISL_9317735, EPI_ISL_9829356, EPI_ISL_9829360, EPI_ISL_12008477, EPI_ISL_12008493, EPI_ISL_12008524	PKD SEREMBAN	UKM Medical Molecular Biology Institute (UMBI)	Khairun Nur Abd Ghafar; Mira Farzana Mohamad Mokhtar; Muhiddin Ishak; Nor Azila Muhammad Azami; Nur Alyaa Afifah Md Shahri; Nurul Syakima Ab Mutalib; Rahman Jamal; Ryia Illani Mohd Yunos; Siti Nur Hasanah Mohd Yusuf; Zahirrah Begam Mohamed Rasheed
EPI_ISL_11112454, EPI_ISL_11112455, EPI_ISL_11112456	PKD SPT	iPROMISE, UiTM	Ariza Adnan; Fadzilah Mohd Nor; Lim Wai Feng; Mohd Asif Mohd Sukri; Mohd Nur Fakhruzzaman Noorizhab; Mohd Zaki Salleh; Sazzli Shahlan Kassim; Siti Farah Alwani Mohd Nawi; Siti Hamimah Sheikh Abdul Kadir; Teh Lay Kek; Wang Seok Mui
EPI_ISL_10397103	PKD TAMPIN	UKM Medical Molecular Biology Institute (UMBI)	Khairun Nur Abd Ghafar; Mira Farzana Mohamad Mokhtar; Muhiddin Ishak; Nor Azila Muhammad Azami; Nur Alyaa Afifah Md Shahri; Nurul Syakima Ab Mutalib; Rahman Jamal; Ryia Illani Mohd Yunos; Siti Nur Hasanah Mohd Yusuf; Zahirrah Begam Mohamed Rasheed
EPI_ISL_10397097	PKD TANGKAK	UKM Medical Molecular Biology Institute (UMBI)	Khairun Nur Abd Ghafar; Mira Farzana Mohamad Mokhtar; Muhiddin Ishak; Nor Azila Muhammad Azami; Nur Alyaa Afifah Md Shahri; Nurul Syakima Ab Mutalib; Rahman Jamal; Ryia Illani Mohd Yunos; Siti Nur Hasanah Mohd Yusuf; Zahirrah Begam Mohamed Rasheed
EPI_ISL_10652539, EPI_ISL_10652540, EPI_ISL_11754753, EPI_ISL_11754754, EPI_ISL_11754755	PKD Tanah Merah	Department of Medical Microbiology, Faculty of Medicine, University of Malaya; University of Malaya Medical Centre	I-Ching SAM; Jolene Yin Ling FU; Omar Khalilur Rahman; Yoke Fun Chan
EPI_ISL_11754740	Pejabat Kesihatan Daerah Tumpat	Department of Medical Microbiology, Faculty of Medicine, University of Malaya; University of Malaya Medical Centre	I-Ching SAM; Jolene Yin Ling FU; Omar Khalilur Rahman; Yoke Fun Chan
EPI_ISL_8603740, EPI_ISL_8603741, EPI_ISL_8603742, EPI_ISL_8603743, EPI_ISL_8603744, EPI_ISL_8603745, EPI_ISL_8603746, EPI_ISL_8603747, EPI_ISL_8603748, EPI_ISL_8603749, EPI_ISL_8603750, EPI_ISL_8603751, EPI_ISL_8603752, EPI_ISL_8603753, EPI_ISL_8603754, EPI_ISL_8603763, EPI_ISL_8603764, EPI_ISL_8603765, EPI_ISL_8603766, EPI_ISL_8603767, EPI_ISL_8603768, EPI_ISL_8603769, EPI_ISL_8603770, EPI_ISL_8603771, EPI_ISL_8603772, EPI_ISL_8603773, EPI_ISL_8603774, EPI_ISL_8603775, EPI_ISL_8603776			
see above	Pejabat Kesihatan KLIA	iPROMISE, UiTM	Ariza Adnan; Fadzilah Mohd Nor; Lim Wai Feng; Mohd Asif Mohd Sukri; Mohd Nur Fakhruzzaman Noorizhab; Mohd Zaki Salleh; Sazzli Shahlan Kassim; Siti Farah Alwani Mohd Nawi; Siti Hamimah Sheikh Abdul Kadir; Teh Lay Kek; Wang Seok Mui
EPI_ISL_11112408, EPI_ISL_11112424	Pejabat Kesihatan Pintu Masuk Antarabangsa Pulau Pinang	iPROMISE, UiTM	Ariza Adnan; Fadzilah Mohd Nor; Lim Wai Feng; Mohd Asif Mohd Sukri; Mohd Nur Fakhruzzaman Noorizhab; Mohd Zaki Salleh; Sazzli Shahlan Kassim; Siti Farah Alwani Mohd Nawi; Siti Hamimah Sheikh Abdul Kadir; Teh Lay Kek; Wang Seok Mui
EPI_ISL_12646708	Public Health Laboratory, Ipoh	Public Health Laboratory Ipoh Disease Section	A.Nisha; MH.Chew; R.Rajes; T.Hafira
EPI_ISL_10495841, EPI_ISL_10495849	QUEEN ELIZABETH HOSPITAL	Institute for Medical Research, Infectious Disease Research Centre, National Institutes of Health, Ministry of Health Malaysia	Ahmad FA; Ahmad Fazilah NA; Anasir MI; Arip MS; Azizan MA; Christopher Hollip K; Jaimin JJ; Kamel K; Mohamad Sukri MZ; Mohammad R; Mohd Yasin R; Norhisham SN; Ramly N; Robert F; Rosli NR; Shamsaddin E; Suppiah J; Tan YC; Thayan R
EPI_ISL_10297334, EPI_ISL_10297335, EPI_ISL_10297350, EPI_ISL_10297351, EPI_ISL_10297354, EPI_ISL_10297356, EPI_ISL_10297359			
see above	Queen Elizabeth Hospital	Institute for Medical Research, Infectious Disease Research Centre, National Institutes of Health, Ministry of Health Malaysia	Ahmad FA; Ahmad Fazilah NA; Anasir MI; Azizan MA; Kamel K; Mohamad Sukri MZ; Norhisham SN; Ramly N; Robert F; Rosli NR; Suppiah J; Thayan R
EPI_ISL_8650146, EPI_ISL_8782835	Rejang Medical Centre (Sibu)	Institute of Health and Community Medicine	Chan Chia Jui; Chua Hock Hin; David Perera; Ooi Mong How; Tonnii Sia Loong Loong; Wong Jyn Shan
EPI_ISL_10297343, EPI_ISL_10297344,	Sandakan Health Clinic	Institute for Medical Research, Infectious	Ahmad FA; Ahmad Fazilah NA; Anasir MI; Azizan MA; Kamel K; Mohamad Sukri MZ; Norhisham SN; Ramly N; Robert F; Rosli NR; Suppiah J; Thayan R

EPI_ISL_10297345, EPI_ISL_10297358		Disease Research Centre, National Institutes of Health, Ministry of Health Malaysia	
EPI_ISL_8650106, EPI_ISL_8650111, EPI_ISL_8650112, EPI_ISL_8650121, EPI_ISL_8650122, EPI_ISL_8650123	Sarawak General Hospital (Kuching)	Institute of Health and Community Medicine	Chan Chia Jui; Chua Hock Hin; David Perera; Ooi Mong How; Tonnii Sia Loong Loong; Wong Jyn Shan
EPI_ISL_8560101, EPI_ISL_8560107, EPI_ISL_8560113	Sg Buloh Hospital	Institute for Medical Research, Infectious Disease Research Centre, National Institutes of Health, Ministry of Health Malaysia	Ahmad FA; Ahmad Fazilah NA; Anasir MI; Azizan MA; Kamel K; Mohamad Sukri MZ; Norhisham SN; Ramly N; Robert F; Rosli NR; Suppiah J; Thayan R
EPI_ISL_11148311	TAWAU AREA HEALTH OFFICE	Institute for Medical Research, Infectious Disease Research Centre, National Institutes of Health, Ministry of Health Malaysia	Ahmad FA; Ahmad Fazilah NA; Anasir MI; Azizan MA; Kamel K; Mohamad Sukri MZ; Norhisham SN; Ramly N; Robert F; Rosli NR; Suppiah J; Thayan R
EPI_ISL_10297338	Tawau Health Clinic	Institute for Medical Research, Infectious Disease Research Centre, National Institutes of Health, Ministry of Health Malaysia	Ahmad FA; Ahmad Fazilah NA; Anasir MI; Azizan MA; Kamel K; Mohamad Sukri MZ; Norhisham SN; Ramly N; Robert F; Rosli NR; Suppiah J; Thayan R
EPI_ISL_10297341	Tenom District Health Office	Institute for Medical Research, Infectious Disease Research Centre, National Institutes of Health, Ministry of Health Malaysia	Ahmad FA; Ahmad Fazilah NA; Anasir MI; Azizan MA; Kamel K; Mohamad Sukri MZ; Norhisham SN; Ramly N; Robert F; Rosli NR; Suppiah J; Thayan R
EPI_ISL_8767326, EPI_ISL_8767327, EPI_ISL_8767328, EPI_ISL_8767329, EPI_ISL_8767330	UMMC	Department of Medical Microbiology, Faculty of Medicine, University of Malaya; University of Malaya Medical Centre	I-Ching SAM; Jolene Yin Ling FU; Omar Khalilur Rahman; Yoke Fun Chan
EPI_ISL_10969013	UNBIT KESIAHTAN DAN KESELAMATAN PEKERJA HOSPITAL SULTANAH BAHIYAH	Tropical Infectious Diseases Research & Education Centre (TIDREC), Universiti Malaya	AsmaAnati CheMatSeri; Che-Norainon Yaacob; Jia-Yi Tan; Jo-Ern Wong; Kim-Kee Tan; Mulya-Mustika-Sari Zulkifli; Noor Syahida Azizan; Nur-Hidayana Mahfodz; Sazaly AbuBakar; Siti-Sarah Nor'e
EPI_ISL_10968988	Unit Keselamatan dan Kesihatan Pekerja Hospital Sultanah Bahiyah	Tropical Infectious Diseases Research & Education Centre (TIDREC), Universiti Malaya	AsmaAnati CheMatSeri; Che-Norainon Yaacob; Jia-Yi Tan; Jo-Ern Wong; Kim-Kee Tan; Mulya-Mustika-Sari Zulkifli; Noor Syahida Azizan; Nur-Hidayana Mahfodz; Sazaly AbuBakar; Siti-Sarah Nor'e
EPI_ISL_10652528, EPI_ISL_10652529, EPI_ISL_10652531, EPI_ISL_10652532, EPI_ISL_10652533, EPI_ISL_10652534, EPI_ISL_10652535, EPI_ISL_10652536, EPI_ISL_10652537			
see above	mka kb	Department of Medical Microbiology, Faculty of Medicine, University of Malaya; University of Malaya Medical Centre	I-Ching SAM; Jolene Yin Ling FU; Omar Khalilur Rahman; Yoke Fun Chan

We gratefully acknowledge the following Authors from the Originating laboratories responsible for obtaining the specimens, as well as the Submitting laboratories where the genome data were generated and shared via GISAID, on which this research is based.

All Submitters of data may be contacted directly via www.gisaid.org

Authors are sorted alphabetically.

Accession ID	Originating Laboratory	Submitting Laboratory	Authors
EPI_ISL_11899956	BP Clinical Lab Sdn Bhd (KLIA)	Malaysia Genome and Vaccine Institute	Azrin Ahmad; Enizza Kasim; Irni Suhayu Sapian; Mohd Faizal Abu Bakar; Mohd Ghows Mohd Azzam.; Mohd Noor Mat Isa; Nor Azfa Johari; Nurhezreen Md Iqbal; Shamsidar Sopie; Siti Noraini Othman; Yusuf Muhammad Noor
EPI_ISL_10297339	Beaufort Health Clinic	Institute for Medical Research, Infectious Disease Research Centre, National Institutes of Health, Ministry of Health Malaysia	Ahmad FA; Ahmad Fazilah NA; Anasir MI; Azizan MA; Kamel K; Mohamad Sukri MZ; Norhisham SN; Ramly N; Robert F; Rosli NR; Suppiah J; Thayan R
EPI_ISL_8782802, EPI_ISL_8782809	Bintulu Hospital PCR Lab, Bintulu	Institute of Health and Community Medicine	Chan Chia Jui; Chua Hock Hin; David Perera; Ooi Mong How; Tonnii Sia Loong Loong; Wong Jyn Shan
EPI_ISL_8782827	Borneo Medical Centre (Kuching)	Institute of Health and Community Medicine	Chan Chia Jui; Chua Hock Hin; David Perera; Ooi Mong How; Tonnii Sia Loong Loong; Wong Jyn Shan
EPI_ISL_9092719, EPI_ISL_9092722, EPI_ISL_9092723	Bukit Changgang Health Clinic	Institute for Medical Research, Infectious Disease Research Centre, National Institutes of Health, Ministry of Health Malaysia	Ahmad FA; Ahmad Fazilah NA; Anasir MI; Azizan MA; Kamel K; Mohamad Sukri MZ; Norhisham SN; Ramly N; Robert F; Rosli NR; Suppiah J; Thayan R
EPI_ISL_9092741, EPI_ISL_9092742, EPI_ISL_9092743, EPI_ISL_9092744	Cheras District Health Office	Institute for Medical Research, Infectious Disease Research Centre, National Institutes of Health, Ministry of Health Malaysia	Ahmad FA; Ahmad Fazilah NA; Anasir MI; Azizan MA; Kamel K; Mohamad Sukri MZ; Norhisham SN; Ramly N; Robert F; Rosli NR; Suppiah J; Thayan R
EPI_ISL_8564949, EPI_ISL_8564950, EPI_ISL_10431132, EPI_ISL_10431135, EPI_ISL_10431138, EPI_ISL_10431139, EPI_ISL_10431141, EPI_ISL_10431146, EPI_ISL_10431149, EPI_ISL_10439970, EPI_ISL_10439971, EPI_ISL_10439973			
see above	Department of Medical Microbiology and Parasitology, Hospital Universiti Sains Malaysia	Molecular Research Laboratory	Abdul Haris bin Muhammad; Ahmad Adebayo Irekeola; Ahmad Sukari Bin Halim; Alexander Chong Shu Chien; Aswini Leela; Azian Harun; Chan Yean Yean; Chua Wei Chuan; Chua Wei Lian; Engku Nur Syafirah bt Engku Abd Rahman; Farahana binti Mohamed; Farahana binti Mohamed Alexander Chong Shu Chien; Kirnpal Kaur Banga Singh; Lau Nyok Sean; Lee Lih Huey; Lim Shu Yong; Maizun binti Mohd Zain; Muhammad Azamuddeen bin Mohammad Nasir; Muhammad Fazli bin Khalid; Muhammad Nashrul Farhan Samsudin; Muhammad Zarul Hanifah Bin Md Zoqratt; Naveed Ahmed; Nazmi Liana Binti Azmi; Noor Hafizan binti Mat Salleh; Nurfadhlina Musa; Qasim Ayub; Rosline Hassan; Sadequr Rahman; Syahida binti Omar; Wan Mohd Zahiruddin Wan Mohammad; Wardah Yusof; Wilhelm Eng Wei Han; Zaini bin Hussin; Zakuan Zainy Deris
EPI_ISL_11344842	Department of Medical Microbiology and Parasitology, School of Medical Sciences, Universiti Sains Malaysia Health Campus	Molecular Research Laboratory, Department of Medical Microbiology and Parasitology, School of Medical Sciences, Universiti Sains Malaysia Health Campus	Abdul Haris bin Muhammad; Ahmad Sukari Bin Halim; Alexander Chong Shu Chien; Asraihan Che Abdul Malik; Azian Harun; Chan Yean Yean; Chua Wei Chuan; Farahana binti Mohamed; Kirnpal Kaur Banga Singh; Lau Nyok Sean; Lee Lih Huey; Lim Shu Yong; Maizun binti Mohd Zain; Mera Edora Binti Abdul Manap; Mohd Iman hafiz Bin Ibrahim; Mohd Nadzri Abu Yazid; Mohd Zulkifli Salleh; Muhamad Khairul Amirin Bin Zulkifli; Muhammad Azamuddeen bin Mohammad Nasir; Muhammad Fazli bin Khalid; Muhammad Nashrul Farhan Samsudin; Muhammad Zarul Hanifah Bin Md Zoqratt; Nik Zuraina binti Nik Mohd Noor; Noor Hafizan binti Mat Salleh; Nor Amizara Binti Azami; Nor Suhana Binti Mohd Satar; Nur Syuhada Binti Abdul Rahim; Nur-Leem Binti Murshid; Nurfadhlina Musa; Qasim Ayub; Rosline Hassan; Sadequr Rahman; Siti Nur Aisyah Binti Mohamad Sham; Syahida binti Omar; Wan Mohd Zahiruddin Wan Mohammad; Wardah Yusof; Zaini bin Hussin; Zakuan Zainy Deris
EPI_ISL_8646709, EPI_ISL_8646710	Department of Medical Microbiology and Parasitology, University of Science Malaysia Hospital	Molecular Research Laboratory, Department of Medical Microbiology and Parasitology, University of Science Health Campus	Abdul Haris bin Muhammad; Ahmad Adebayo Irekeola; Ahmad Sukari Bin Halim; Alexander Chong Shu Chien; Aswini Leela; Azian Harun; Chan Yean Yean; Chua Wei Chuan; Chua Wei Lian; Engku Nur Syafirah bt Engku Abd Rahman; Farahana binti Mohamed; Kirnpal Kaur Banga Singh; Lau Nyok Sean; Lee Lih Huey; Lim Shu Yong; Maizun binti Mohd Zain; Muhammad Azamuddeen bin Mohammad Nasir; Muhammad Fazli bin Khalid; Muhammad Nashrul Farhan Samsudin; Muhammad Zarul Hanifah Bin Md Zoqratt; Naveed Ahmed; Nazmi Liana Binti Azmi; Noor Hafizan binti Mat Salleh; Nurfadhlina Musa; Qasim Ayub; Rosline Hassan; Sadequr Rahman; Syahida binti Omar; Wan Mohd Zahiruddin Wan Mohammad; Wardah Yusof; Wilhelm Eng Wei Han; Zaini bin Hussin; Zakuan Zainy Deris
EPI_ISL_10064500, EPI_ISL_10065077, EPI_ISL_10066376, EPI_ISL_10066577, EPI_ISL_10066836, EPI_ISL_10070307, EPI_ISL_10072708, EPI_ISL_10072709, EPI_ISL_10072710, EPI_ISL_10072805, EPI_ISL_10072889, EPI_ISL_10073320, EPI_ISL_10073418, EPI_ISL_10073419, EPI_ISL_10073513, EPI_ISL_12721581			
see above	Department of Medical Microbiology, Hospital Pengajar Universiti Putra Malaysia	Department of Medical Microbiology, Hospital Pengajar Universiti Putra Malaysia	Afiqah Adzmi; Amiza Azmi; Azmiza Syawani Jasni; Hui Yee Chee; Leslie Thian Lung Than; Muadz Mohtar; Muhammad Mohd Isa; Narcisse MS Joseph; Niazlin Mohd Taib; Noor Hazirah Noor Azhari; Norlaila; Nur Raihana Ithnin; Nurul Huda Mohamed Rashidi; Nurul Nadiah Ismail; Rosni Ibrahim; Rukman Awang Hamat; Sallehhudin; Siti Norbaya Masri; Siti Zulaikha Zakariah; Syafinaz Amin Nordin; Tengku Zetty Maztura Tengku Jamaluddin; Zamberi Sekawi
EPI_ISL_10297362	Dr Justin Surgery Clinic	Institute for Medical Research, Infectious Disease Research Centre, National Institutes of Health, Ministry of Health Malaysia	Ahmad FA; Ahmad Fazilah NA; Anasir MI; Azizan MA; Kamel K; Mohamad Sukri MZ; Norhisham SN; Ramly N; Robert F; Rosli NR; Suppiah J; Thayan R
EPI_ISL_11112474, EPI_ISL_11112475, EPI_ISL_11112476	ED HOSPITAL BALIK PULAU	iPROMISE, UiTM	Ariza Adnan; Fadzilah Mohd Nor; Lim Wai Feng; Mohd Asif Mohd Sukri; Mohd Nur Fakhruzzaman Noorizhab; Mohd Zaki Salleh; Sazzli Shahlan Kassim; Siti Farah Alwani Mohd Nawi; Siti Hamimah Sheikh Abdul Kadir; Teh Lay Kek; Wang Seok Mui
EPI_ISL_11112479, EPI_ISL_11112480	HOSPITAL PULAU PINANG	iPROMISE, UiTM	Ariza Adnan; Fadzilah Mohd Nor; Lim Wai Feng; Mohd Asif Mohd Sukri; Mohd Nur Fakhruzzaman Noorizhab; Mohd Zaki Salleh; Sazzli Shahlan Kassim; Siti Farah Alwani Mohd Nawi; Siti Hamimah Sheikh Abdul Kadir; Teh Lay Kek; Wang Seok Mui
EPI_ISL_10968990, EPI_ISL_10968991, EPI_ISL_10969052, EPI_ISL_10969053, EPI_ISL_12534776, EPI_ISL_12534777, EPI_ISL_12534778			
see above	HOSPITAL RAJA PERMAISURI BAINUN, IPOH	Tropical Infectious Diseases Research & Education Centre (TIDREC), Universiti Malaya	AsmaAnati CheMatSeri; Che-Norainon Yaacob; Jia-Yi Tan; Jo-Ern Wong; Kim-Kee Tan; Mulya-Mustika-Sari Zulkifli; Noor Syahida Azizan; Nur-Hidayana Mahfodz; Sazaly AbuBakar; Siti-Sarah Nor'e; Wei-Wei Aw
EPI_ISL_10968995	HOSPITAL SULTAN ISMAIL PETRA KUALA KRAI, KELANTAN	Tropical Infectious Diseases Research & Education Centre (TIDREC), Universiti Malaya	AsmaAnati CheMatSeri; Che-Norainon Yaacob; Jia-Yi Tan; Jo-Ern Wong; Kim-Kee Tan; Mulya-Mustika-Sari Zulkifli; Noor Syahida Azizan; Nur-Hidayana Mahfodz; Sazaly AbuBakar; Siti-Sarah Nor'e
EPI_ISL_9829346	HOSPITAL SULTAN ISMAIL PETRA, KUALA KRAI KELANTAN	UKM Medical Molecular Biology Institute (UMBI)	Khairun Nur Abd Ghafar; Mira Farzana Mohamad Mokhtar; Muhiddin Ishak; Nor Azila Muhammad Azami; Nur Alyaa Afifah Md Shahri; Nurul Syakima Ab Mutalib; Rahman Jamal; Ryia Illani Mohd Yunos; Siti Nur Hasanah Mohd Yusuf; Zahirrah Begam Mohamed Rasheed
EPI_ISL_10397049	HOSPITAL SULTANAH NORA ISMAIL, BATU PAHAT	UKM Medical Molecular Biology Institute (UMBI)	Khairun Nur Abd Ghafar; Mira Farzana Mohamad Mokhtar; Muhiddin Ishak; Nor Azila Muhammad Azami; Nur Alyaa Afifah Md Shahri; Nurul Syakima Ab Mutalib; Rahman Jamal; Ryia Illani Mohd Yunos; Siti Nur Hasanah Mohd Yusuf; Zahirrah Begam Mohamed Rasheed
EPI_ISL_10810958, EPI_ISL_10969029, EPI_ISL_12534752, EPI_ISL_12534753	HOSPITAL SULTANAH NUR ZAHIRAH	Tropical Infectious Diseases Research & Education Centre (TIDREC), Universiti Malaya	AsmaAnati CheMatSeri; Che-Norainon Yaacob; Jia-Yi Tan; Jo-Ern Wong; Kim-Kee Tan; Mulya-Mustika-Sari Zulkifli; Noor Syahida Azizan; Nur-Hidayana Mahfodz; Sazaly AbuBakar; Siti-Sarah Nor'e; Wei-Wei Aw
EPI_ISL_10969030	HOSPITAL SULTANAH NURZAHIRAH	Tropical Infectious Diseases Research & Education Centre (TIDREC), Universiti Malaya	AsmaAnati CheMatSeri; Che-Norainon Yaacob; Jia-Yi Tan; Jo-Ern Wong; Kim-Kee Tan; Mulya-Mustika-Sari Zulkifli; Noor Syahida Azizan; Nur-Hidayana Mahfodz; Sazaly AbuBakar; Siti-Sarah Nor'e
EPI_ISL_10495864, EPI_ISL_10495872	HOSPITAL TAWAU	Institute for Medical Research, Infectious Disease Research Centre, National Institutes of Health, Ministry of Health Malaysia	Ahmad FA; Ahmad Fazilah NA; Anasir MI; Arip MS; Azizan MA; Christopher Hollip K; Jaimin JJ; Kamel K; Mohamad Sukri MZ; Mohammad R; Mohd Yasin R; Norhisham SN; Ramly N; Robert F; Rosli NR; Shamsaddin E; Suppiah J; Tan YC; Thayan R
EPI_ISL_12534792, EPI_ISL_12534793, EPI_ISL_12534794, EPI_ISL_12534795, EPI_ISL_12534796	HRPB	Tropical Infectious Diseases Research & Education Centre (TIDREC), Universiti Malaya	AsmaAnati CheMatSeri; Che-Norainon Yaacob; Jia-Yi Tan; Jo-Ern Wong; Kim-Kee Tan; Mulya-Mustika-Sari Zulkifli; Noor Syahida Azizan; Nur-Hidayana Mahfodz; Sazaly AbuBakar; Siti-Sarah Nor'e; Wei-Wei Aw
EPI_ISL_10397086, EPI_ISL_10397088, EPI_ISL_10397099, EPI_ISL_10397115, EPI_ISL_10397119	HTJS	UKM Medical Molecular Biology Institute (UMBI)	Khairun Nur Abd Ghafar; Mira Farzana Mohamad Mokhtar; Muhiddin Ishak; Nor Azila Muhammad Azami; Nur Alyaa Afifah Md Shahri; Nurul Syakima Ab Mutalib; Rahman Jamal; Ryia Illani Mohd Yunos; Siti Nur Hasanah Mohd Yusuf; Zahirrah Begam Mohamed Rasheed
EPI_ISL_10590721, EPI_ISL_10590722, EPI_ISL_10590723, EPI_ISL_10590724, EPI_ISL_10590725, EPI_ISL_10590726, EPI_ISL_10590767, EPI_ISL_11899940, EPI_ISL_11899973, EPI_ISL_11899991, EPI_ISL_11899992, EPI_ISL_11899993, EPI_ISL_11899994, EPI_ISL_11899995, EPI_ISL_11899996, EPI_ISL_11899999			
see above	Hospital Ampang	Malaysia Genome and Vaccine Institute	Azrin Ahmad; Enizza Kasim; Irni Suhayu Sapian; Mohd Faizal Abu Bakar; Mohd Ghows Mohd Azzam.; Mohd Noor Mat Isa; Nor Azfa Johari; Nurhezreen Md Iqbal; Shamsidar Sopie; Siti Noraini Othman; Yusuf Muhammad Noor
EPI_ISL_10590734, EPI_ISL_10590735, EPI_ISL_10590761, EPI_ISL_10590769, EPI_ISL_10590773, EPI_ISL_10590781	Hospital Kuala Lumpur	Malaysia Genome and Vaccine Institute	Azrin Ahmad; Enizza Kasim; Irni Suhayu Sapian; Mohd Faizal Abu Bakar; Mohd Ghows Mohd Azzam.; Mohd Noor Mat Isa; Nurhezreen Md Iqbal; Shamsidar Sopie; Siti Noraini Othman; Yusuf Muhammad Noor
EPI_ISL_11112477	Hospital Pulau Pinang	iPROMISE, UiTM	Ariza Adnan; Fadzilah Mohd Nor; Lim Wai Feng; Mohd Asif Mohd Sukri; Mohd Nur Fakhruzzaman Noorizhab; Mohd Zaki Salleh; Sazzli Shahlan Kassim; Siti Farah Alwani Mohd Nawi; Siti Hamimah Sheikh Abdul Kadir; Teh Lay Kek; Wang Seok Mui
EPI_ISL_11900013	Hospital Putrajaya	Malaysia Genome and Vaccine Institute	Azrin Ahmad; Enizza Kasim; Irni Suhayu Sapian; Mohd Faizal Abu Bakar; Mohd Ghows Mohd Azzam.; Mohd Noor Mat Isa; Nor Azfa Johari; Nurhezreen Md Iqbal; Shamsidar Sopie; Siti Noraini Othman; Yusuf Muhammad Noor
EPI_ISL_11899957	Hospital Selayang	Malaysia Genome and Vaccine Institute	Azrin Ahmad; Enizza Kasim; Irni Suhayu Sapian; Mohd Faizal Abu Bakar; Mohd Ghows Mohd Azzam.; Mohd Noor Mat Isa; Nor Azfa Johari; Nurhezreen Md Iqbal; Shamsidar Sopie; Siti Noraini Othman; Yusuf Muhammad Noor
EPI_ISL_10397054, EPI_ISL_10397055	Hospital Sultanah Aminah Johor Bahru	UKM Medical Molecular Biology Institute (UMBI)	Khairun Nur Abd Ghafar; Mira Farzana Mohamad Mokhtar; Muhiddin Ishak; Nor Azila Muhammad Azami; Nur Alyaa Afifah Md Shahri; Nurul Syakima Ab Mutalib; Rahman Jamal; Ryia Illani Mohd Yunos; Siti Nur Hasanah Mohd Yusuf; Zahirrah Begam Mohamed Rasheed
EPI_ISL_12534751	Hospital Sultanah Nur Zahirah	Tropical Infectious Diseases Research & Education Centre (TIDREC), Universiti Malaya	AsmaAnati CheMatSeri; Che-Norainon Yaacob; Jia-Yi Tan; Jo-Ern Wong; Kim-Kee Tan; Mulya-Mustika-Sari Zulkifli; Noor Syahida Azizan; Nur-Hidayana Mahfodz; Sazaly AbuBakar; Siti-Sarah Nor'e; Wei-Wei Aw
EPI_ISL_10590744, EPI_ISL_10590745, EPI_ISL_10590775, EPI_ISL_10590776, EPI_ISL_10590779, EPI_ISL_10590782, EPI_ISL_10590783, EPI_ISL_10590784, EPI_ISL_10590785, EPI_ISL_10590788, EPI_ISL_10590789, EPI_ISL_10590791, EPI_ISL_10590792, EPI_ISL_10590794, EPI_ISL_10590796, EPI_ISL_10590798, EPI_ISL_11899951, EPI_ISL_11899952, EPI_ISL_11899954, EPI_ISL_11899978, EPI_ISL_11899979			

see above	Hospital Sungai Buloh	Malaysia Genome and Vaccine Institute	Azrin Ahmad; Enizza Kasim; Irni Suhayu Sapian; Mohd Faizal Abu Bakar; Mohd Ghows Mohd Azzam.; Mohd Noor Mat Isa; Nor Azfa Johari; Nurhezreen Md Iqbal; Shamsidar Sopie; Siti Noraini Othman; Yusuf Muhammad Noor
EPI_ISL_11899985	Hospital Tengku Ampuan Rahimah	Malaysia Genome and Vaccine Institute	Azrin Ahmad; Enizza Kasim; Irni Suhayu Sapian; Mohd Faizal Abu Bakar; Mohd Ghows Mohd Azzam.; Mohd Noor Mat Isa; Nor Azfa Johari; Nurhezreen Md Iqbal; Shamsidar Sopie; Siti Noraini Othman; Yusuf Muhammad Noor
EPI_ISL_10810915	IMR	Tropical Infectious Diseases Research & Education Centre (TIDREC), Universiti Malaya	AsmaAnati CheMatSeri; Che-Norainon Yaacob; Jia-Yi Tan; Jo-Ern Wong; Kim-Kee Tan; Mulya-Mustika-Sari Zulkifli; Noor Syahida Azizan; Nur-Hidayana Mahfodz; Sazaly AbuBakar; Siti-Sarah Nor'e
EPI_ISL_11939056	IMR	iPROMISE, UiTM	Ariza Adnan; Fadzilah Mohd Nor; Lim Wai Feng; Mohd Asif Mohd Sukri; Mohd Nur Fakhruzzaman Noorizhab; Mohd Zaki Salleh; Sazzli Shahlan Kassim; Siti Farah Alwani Mohd Nawi; Siti Hamimah Sheikh Abdul Kadir; Teh Lay Kek; Wang Seok Mui
EPI_ISL_11323764, EPI_ISL_11323768, EPI_ISL_11323785	Institut Biologi Molekul Perubatan UKM (UMBI)	UKM Medical Molecular Biology Institute (UMBI)	Khairun Nur Abd Ghafar; Mira Farzana Mohamad Mokhtar; Muhiddin Ishak; Nor Azila Muhammad Azami; Nur Alyaa Afifah Md Shahri; Nurul Syakima Ab Mutalib; Rahman Jamal; Ryia Illani Mohd Yunos; Siti Nur Hasanah Mohd Yusuf; Zahirrah Begam Mohamed Rasheed
EPI_ISL_11899958, EPI_ISL_11899959, EPI_ISL_11899962, EPI_ISL_11899963, EPI_ISL_11899964, EPI_ISL_11899965	Institut Kanser Negara	Malaysia Genome and Vaccine Institute	Azrin Ahmad; Enizza Kasim; Irni Suhayu Sapian; Mohd Faizal Abu Bakar; Mohd Ghows Mohd Azzam.; Mohd Noor Mat Isa; Nor Azfa Johari; Nurhezreen Md Iqbal; Shamsidar Sopie; Siti Noraini Othman; Yusuf Muhammad Noor
EPI_ISL_11112473	JABATAN FORENSIC HOSPITAL PULAU PINANG	iPROMISE, UiTM	Ariza Adnan; Fadzilah Mohd Nor; Lim Wai Feng; Mohd Asif Mohd Sukri; Mohd Nur Fakhruzzaman Noorizhab; Mohd Zaki Salleh; Sazzli Shahlan Kassim; Siti Farah Alwani Mohd Nawi; Siti Hamimah Sheikh Abdul Kadir; Teh Lay Kek; Wang Seok Mui
EPI_ISL_10969140	JABATAN PATOLOGI HOSPITAL RAJA Female ZAINAB II	Tropical Infectious Diseases Research & Education Centre (TIDREC), Universiti Malaya	AsmaAnati CheMatSeri; Che-Norainon Yaacob; Jia-Yi Tan; Jo-Ern Wong; Kim-Kee Tan; Mulya-Mustika-Sari Zulkifli; Noor Syahida Azizan; Nur-Hidayana Mahfodz; Sazaly AbuBakar; Siti-Sarah Nor'e
EPI_ISL_10969069, EPI_ISL_10969070, EPI_ISL_10969071, EPI_ISL_12534833, EPI_ISL_12534838	JABATAN PATOLOGI HOSPITAL RAJA PEREMPUAN ZAINAB II	Tropical Infectious Diseases Research & Education Centre (TIDREC), Universiti Malaya	AsmaAnati CheMatSeri; Che-Norainon Yaacob; Jia-Yi Tan; Jo-Ern Wong; Kim-Kee Tan; Mulya-Mustika-Sari Zulkifli; Noor Syahida Azizan; Nur-Hidayana Mahfodz; Sazaly AbuBakar; Siti-Sarah Nor'e; Wei-Wei Aw
EPI_ISL_10969110	JABATAN PATOLOGI, HOSPITAL RAJA Female ZAINAB II	Tropical Infectious Diseases Research & Education Centre (TIDREC), Universiti Malaya	AsmaAnati CheMatSeri; Che-Norainon Yaacob; Jia-Yi Tan; Jo-Ern Wong; Kim-Kee Tan; Mulya-Mustika-Sari Zulkifli; Noor Syahida Azizan; Nur-Hidayana Mahfodz; Sazaly AbuBakar; Siti-Sarah Nor'e
EPI_ISL_10968992, EPI_ISL_10968993, EPI_ISL_10968994, EPI_ISL_10968996, EPI_ISL_10969014, EPI_ISL_10969015, EPI_ISL_10969016, EPI_ISL_10969017, EPI_ISL_10969018, EPI_ISL_10969019, EPI_ISL_10969020, EPI_ISL_10969021, EPI_ISL_10969022, EPI_ISL_10969023, EPI_ISL_10969024, EPI_ISL_10969054, EPI_ISL_10969055, EPI_ISL_10969056, EPI_ISL_10969057, EPI_ISL_10969058, EPI_ISL_10969059, EPI_ISL_10969060, EPI_ISL_10969061, EPI_ISL_10969062, EPI_ISL_10969063, EPI_ISL_10969064, EPI_ISL_10969065, EPI_ISL_10969066, EPI_ISL_10969067, EPI_ISL_10969068, EPI_ISL_10969072, EPI_ISL_10969073, EPI_ISL_10969074, EPI_ISL_10969076, EPI_ISL_10969153, EPI_ISL_10969154, EPI_ISL_12534828, EPI_ISL_12534829, EPI_ISL_12534830, EPI_ISL_12534831, EPI_ISL_12534835, EPI_ISL_12534837, EPI_ISL_12534839, EPI_ISL_12534840, EPI_ISL_12534866			
see above	JABATAN PATOLOGI, HOSPITAL RAJA PEREMPUAN ZAINAB II	Tropical Infectious Diseases Research & Education Centre (TIDREC), Universiti Malaya	AsmaAnati CheMatSeri; Che-Norainon Yaacob; Jia-Yi Tan; Jo-Ern Wong; Kim-Kee Tan; Mulya-Mustika-Sari Zulkifli; Noor Syahida Azizan; Nur-Hidayana Mahfodz; Sazaly AbuBakar; Siti-Sarah Nor'e; Wei-Wei Aw
EPI_ISL_11112394, EPI_ISL_11112395, EPI_ISL_11112396, EPI_ISL_11112434, EPI_ISL_11112435, EPI_ISL_11112436, EPI_ISL_11112465, EPI_ISL_11112466, EPI_ISL_11112467, EPI_ISL_11117197, EPI_ISL_11117198, EPI_ISL_11117199, EPI_ISL_11117200, EPI_ISL_11117201, EPI_ISL_11117202, EPI_ISL_11117203, EPI_ISL_11117205, EPI_ISL_11117207, EPI_ISL_11117211			
see above	JKN PAHANG	iPROMISE, UiTM	Ariza Adnan; Fadzilah Mohd Nor; Lim Wai Feng; Mohd Asif Mohd Sukri; Mohd Nur Fakhruzzaman Noorizhab; Mohd Zaki Salleh; Sazzli Shahlan Kassim; Siti Farah Alwani Mohd Nawi; Siti Hamimah Sheikh Abdul Kadir; Teh Lay Kek; Wang Seok Mui
EPI_ISL_12135870	Jabatan Forensik Hospital Sultanah Bahiyah	Tropical Infectious Diseases Research & Education Centre (TIDREC), Universiti Malaya	AsmaAnati CheMatSeri; Che-Norainon Yaacob; Jia-Yi Tan; Jo-Ern Wong; Kim-Kee Tan; Mulya-Mustika-Sari Zulkifli; Noor Syahida Azizan; Nur-Hidayana Mahfodz; Sazaly AbuBakar; Siti-Sarah Nor'e; Wei-Wei Aw
EPI_ISL_9579202, EPI_ISL_11112392, EPI_ISL_11112393	Jabatan Kesihatan Negeri	iPROMISE, UiTM	Ariza Adnan; Fadzilah Mohd Nor; Lim Wai Feng; Mohd Asif Mohd Sukri; Mohd Nur Fakhruzzaman Noorizhab; Mohd Zaki Salleh; Sazzli Shahlan Kassim; Siti Farah Alwani Mohd Nawi; Siti Hamimah Sheikh Abdul Kadir; Teh Lay Kek; Wang Seok Mui
EPI_ISL_11899967, EPI_ISL_11899968, EPI_ISL_11899969, EPI_ISL_11899970, EPI_ISL_11899971, EPI_ISL_11899972	Jabatan Kesihatan Negeri WP Labuan	Malaysia Genome and Vaccine Institute	Azrin Ahmad; Enizza Kasim; Irni Suhayu Sapian; Mohd Faizal Abu Bakar; Mohd Ghows Mohd Azzam.; Mohd Noor Mat Isa; Nor Azfa Johari; Nurhezreen Md Iqbal; Shamsidar Sopie; Siti Noraini Othman; Yusuf Muhammad Noor
EPI_ISL_9092725	Jenjarom Health Clinic	Institute for Medical Research, Infectious Disease Research Centre, National Institutes of Health, Ministry of Health Malaysia	Ahmad FA; Ahmad Fazilah NA; Anasir MI; Azizan MA; Kamel K; Mohamad Sukri MZ; Norhisham SN; Ramly N; Robert F; Rosli NR; Suppiah J; Thayan R
EPI_ISL_10810917	KEDAH MEDICAL CENTRE	Tropical Infectious Diseases Research & Education Centre (TIDREC), Universiti Malaya	AsmaAnati CheMatSeri; Che-Norainon Yaacob; Jia-Yi Tan; Jo-Ern Wong; Kim-Kee Tan; Mulya-Mustika-Sari Zulkifli; Noor Syahida Azizan; Nur-Hidayana Mahfodz; Sazaly AbuBakar; Siti-Sarah Nor'e
EPI_ISL_11939049, EPI_ISL_11939054, EPI_ISL_11939055	KLINIK MENARA PENANG	iPROMISE, UiTM	Ariza Adnan; Fadzilah Mohd Nor; Lim Wai Feng; Mohd Asif Mohd Sukri; Mohd Nur Fakhruzzaman Noorizhab; Mohd Zaki Salleh; Sazzli Shahlan Kassim; Siti Farah Alwani Mohd Nawi; Siti Hamimah Sheikh Abdul Kadir; Teh Lay Kek; Wang Seok Mui
EPI_ISL_10495881	KOTA KINABALU AREA HEALTH OFFICE	Institute for Medical Research, Infectious Disease Research Centre, National Institutes of Health, Ministry of Health Malaysia	Ahmad FA; Ahmad Fazilah NA; Anasir MI; Arip MS; Azizan MA; Christopher Hollip K; Jaimin JJ; Kamel K; Mohamad Sukri MZ; Mohammad R; Mohd Yasin R; Norhisham SN; Ramly N; Robert F; Rosli NR; Shamsaddin E; Suppiah J; Tan YC; Thayan R
EPI_ISL_9092713	Kampung Bandar Health Clinic	Institute for Medical Research, Infectious Disease Research Centre, National Institutes of Health, Ministry of Health Malaysia	Ahmad FA; Ahmad Fazilah NA; Anasir MI; Azizan MA; Kamel K; Mohamad Sukri MZ; Norhisham SN; Ramly N; Robert F; Rosli NR; Suppiah J; Thayan R
EPI_ISL_9092745, EPI_ISL_9092746	Kepong District Health Office	Institute for Medical Research, Infectious Disease Research Centre, National Institutes of Health, Ministry of Health Malaysia	Ahmad FA; Ahmad Fazilah NA; Anasir MI; Azizan MA; Kamel K; Mohamad Sukri MZ; Norhisham SN; Ramly N; Robert F; Rosli NR; Suppiah J; Thayan R
EPI_ISL_10297366	Kinabatangan Health Clinic	Institute for Medical Research, Infectious Disease Research Centre, National Institutes of Health, Ministry of Health Malaysia	Ahmad FA; Ahmad Fazilah NA; Anasir MI; Azizan MA; Kamel K; Mohamad Sukri MZ; Norhisham SN; Ramly N; Robert F; Rosli NR; Suppiah J; Thayan R
EPI_ISL_12534834	Klinik Dr Izzat	Tropical Infectious Diseases Research & Education Centre (TIDREC), Universiti Malaya	AsmaAnati CheMatSeri; Che-Norainon Yaacob; Jia-Yi Tan; Jo-Ern Wong; Kim-Kee Tan; Mulya-Mustika-Sari Zulkifli; Noor Syahida Azizan; Nur-Hidayana Mahfodz; Sazaly AbuBakar; Siti-Sarah Nor'e; Wei-Wei Aw
EPI_ISL_11332714	Kota Bharu Public Health Laboratory	Tropical Infectious Diseases Research & Education Centre (TIDREC), Universiti Malaya	AsmaAnati CheMatSeri; Che-Norainon Yaacob; Jia-Yi Tan; Jo-Ern Wong; Kim-Kee Tan; Mulya-Mustika-Sari Zulkifli; Noor Syahida Azizan; Nur-Hidayana Mahfodz; Sazaly AbuBakar; Siti-Sarah Nor'e; Wei-Wei Aw
EPI_ISL_10297340, EPI_ISL_10297348	Kota Kinabalu Health Clinic	Institute for Medical Research, Infectious Disease Research Centre, National Institutes of Health, Ministry of Health Malaysia	Ahmad FA; Ahmad Fazilah NA; Anasir MI; Azizan MA; Kamel K; Mohamad Sukri MZ; Norhisham SN; Ramly N; Robert F; Rosli NR; Suppiah J; Thayan R
EPI_ISL_9092752, EPI_ISL_9092753	Kuala Langat District Health Office	Institute for Medical Research, Infectious Disease Research Centre, National Institutes of Health, Ministry of Health Malaysia	Ahmad FA; Ahmad Fazilah NA; Anasir MI; Azizan MA; Kamel K; Mohamad Sukri MZ; Norhisham SN; Ramly N; Robert F; Rosli NR; Suppiah J; Thayan R
EPI_ISL_9092712, EPI_ISL_9095264, EPI_ISL_9095265, EPI_ISL_9095266, EPI_ISL_9095267, EPI_ISL_9095268, EPI_ISL_9095269, EPI_ISL_9095270, EPI_ISL_9095271, EPI_ISL_9095272, EPI_ISL_9095273, EPI_ISL_9095274, EPI_ISL_9095275, EPI_ISL_9095276, EPI_ISL_9095277, EPI_ISL_9095278, EPI_ISL_9095279, EPI_ISL_9095280, EPI_ISL_9095281, EPI_ISL_9095282, EPI_ISL_9095283, EPI_ISL_9095284, EPI_ISL_9095285, EPI_ISL_9095286, EPI_ISL_9095287, EPI_ISL_9095288, EPI_ISL_9095289, EPI_ISL_9095290, EPI_ISL_9095291, EPI_ISL_9095292, EPI_ISL_9095293, EPI_ISL_9095294, EPI_ISL_9095295, EPI_ISL_9095296, EPI_ISL_9095297, EPI_ISL_9095298, EPI_ISL_9095299, EPI_ISL_9095300, EPI_ISL_9095301, EPI_ISL_9095302, EPI_ISL_9095303, EPI_ISL_9095304, EPI_ISL_9095305, EPI_ISL_9095306, EPI_ISL_9095307, EPI_ISL_9095308, EPI_ISL_9095309, EPI_ISL_9095310, EPI_ISL_9095311, EPI_ISL_9095312, EPI_ISL_9095313, EPI_ISL_9095314, EPI_ISL_9095315, EPI_ISL_9212704, EPI_ISL_9212895, EPI_ISL_9212959, EPI_ISL_9213384, EPI_ISL_9213406, EPI_ISL_9213506, EPI_ISL_9213601, EPI_ISL_9213749, EPI_ISL_9213854, EPI_ISL_9213935, EPI_ISL_9214088, EPI_ISL_9214189, EPI_ISL_9214321, EPI_ISL_9214430, EPI_ISL_9214525, EPI_ISL_9214701, EPI_ISL_9215701, EPI_ISL_9215773, EPI_ISL_9216311, EPI_ISL_9216410, EPI_ISL_9216490, EPI_ISL_9216664, EPI_ISL_9216842, EPI_ISL_9217105, EPI_ISL_9217252, EPI_ISL_9217380, EPI_ISL_9217528, EPI_ISL_9217682, EPI_ISL_9217815, EPI_ISL_9832499			
see above	Kuala Lumpur International Airport (KLIA) Health Office	Institute for Medical Research, Infectious Disease Research Centre, National Institutes of Health, Ministry of Health Malaysia	Ahmad FA; Ahmad Fazilah NA; Anasir MI; Azizan MA; Kamel K; Mohamad Sukri MZ; Norhisham SN; Ramly N; Robert F; Rosli NR; Suppiah J; Thayan R
EPI_ISL_11939131	LAM WAH EE HOSPITAL	iPROMISE, UiTM	Ariza Adnan; Fadzilah Mohd Nor; Lim Wai Feng; Mohd Asif Mohd Sukri; Mohd Nur Fakhruzzaman Noorizhab; Mohd Zaki Salleh; Sazzli Shahlan Kassim; Siti Farah Alwani Mohd Nawi; Siti Hamimah Sheikh Abdul Kadir; Teh Lay Kek; Wang Seok Mui
EPI_ISL_9092754, EPI_ISL_9092755, EPI_ISL_9092756	Lembah Pantai District Health Office	Institute for Medical Research, Infectious Disease Research Centre, National Institutes of Health, Ministry of Health Malaysia	Ahmad FA; Ahmad Fazilah NA; Anasir MI; Azizan MA; Kamel K; Mohamad Sukri MZ; Norhisham SN; Ramly N; Robert F; Rosli NR; Suppiah J; Thayan R
EPI_ISL_11112430	Medical Hospital Pulau Pinang	iPROMISE, UiTM	Ariza Adnan; Fadzilah Mohd Nor; Lim Wai Feng; Mohd Asif Mohd Sukri; Mohd Nur Fakhruzzaman Noorizhab; Mohd Zaki Salleh; Sazzli Shahlan Kassim; Siti Farah Alwani Mohd Nawi; Siti Hamimah Sheikh Abdul Kadir; Teh Lay Kek; Wang Seok Mui
EPI_ISL_8650124, EPI_ISL_8650131, EPI_ISL_9608480	Miri Hospital Molecular Diagnostic Lab, Miri	Institute of Health and Community Medicine	Chan Chia Jui; Chua Hock Hin; David Perera; Ooi Mong How; Tonnii Sia Loong Loong; Wong Jyn Shan
EPI_ISL_9092772, EPI_ISL_9092773	National Institutes of Health	Institute for Medical Research, Infectious Disease Research Centre, National Institutes of Health, Ministry of Health Malaysia	Ahmad FA; Ahmad Fazilah NA; Anasir MI; Azizan MA; Kamel K; Mohamad Sukri MZ; Norhisham SN; Ramly N; Robert F; Rosli NR; Suppiah J; Thayan R
EPI_ISL_10297367	National Institutes of Health (NIH)	Institute for Medical Research, Infectious Disease Research Centre, National Institutes of Health, Ministry of Health Malaysia	Ahmad FA; Ahmad Fazilah NA; Anasir MI; Azizan MA; Kamel K; Mohamad Sukri MZ; Norhisham SN; Ramly N; Robert F; Rosli NR; Suppiah J; Thayan R

EPI_ISL_11717937	National Public Health Laboratory	National Public Health Laboratory	Ishma Hana Binti Isharudin; Kamal Hisham Bin Kamarul Zaman; Noriah Binti Mohd Yusof; Rehan Shuhada Binti Abu Bakar; Selvanesan A/L Sengol; Ushananthiny A/P Ravi; W.Nur Afiza Binti W. Ariffin; Yu Kie A/P Chem
EPI_ISL_8650125, EPI_ISL_8650134, EPI_ISL_8650147, EPI_ISL_9608488, EPI_ISL_9608508, EPI_ISL_9608509, EPI_ISL_9608510			
see above	Normah Medical Specialist Centre, Kuching	Institute of Health and Community Medicine	Chan Chia Jui; Chua Hock Hin; David Perera; Ikwannuddin Y; Ooi Mong How; Tonnii Sia Loong Loong; Wong Jyn Shan
EPI_ISL_11754737, EPI_ISL_11754738, EPI_ISL_11754776	PEJABAT KESIHATAN DAERAH PASIR MAS	Department of Medical Microbiology, Faculty of Medicine, University of Malaya; University of Malaya Medical Centre	I-Ching SAM; Jolene Yin Ling FU; Omar Khalilur Rahman; Yoke Fun Chan
EPI_ISL_11939093, EPI_ISL_11939096, EPI_ISL_11939097, EPI_ISL_11939100, EPI_ISL_11939102, EPI_ISL_11939103	PEJABAT KESIHATAN DAERAH SEBERANG PERAI UTARA PULAU PINANG	iPROMISE, UiTM	Ariza Adnan; Fadzilah Mohd Nor; Lim Wai Feng; Mohd Asif Mohd Sukri; Mohd Nur Fakhruzzaman Noorizhab; Mohd Zaki Salleh; Sazzli Shahlan Kassim; Siti Farah Alwani Mohd Nawi; Siti Hamimah Sheikh Abdul Kadir; Teh Lay Kek; Wang Seok Mui
EPI_ISL_11112468, EPI_ISL_11112469	PEJABAT KESIHATAN DAERAH TIMUR LAUT	iPROMISE, UiTM	Ariza Adnan; Fadzilah Mohd Nor; Lim Wai Feng; Mohd Asif Mohd Sukri; Mohd Nur Fakhruzzaman Noorizhab; Mohd Zaki Salleh; Sazzli Shahlan Kassim; Siti Farah Alwani Mohd Nawi; Siti Hamimah Sheikh Abdul Kadir; Teh Lay Kek; Wang Seok Mui
EPI_ISL_12534832	PEJABAT KESIHATAN PASIR MAS	Tropical Infectious Diseases Research & Education Centre (TIDREC), Universiti Malaya	AsmaAnati CheMatSeri; Che-Norainon Yaacob; Jia-Yi Tan; Jo-Ern Wong; Kim-Kee Tan; Mulya-Mustika-Sari Zulkifli; Noor Syahida Azizan; Nur-Hidayana Mahfodz; Sazaly AbuBakar; Siti-Sarah Nor'e; Wei-Wei Aw
EPI_ISL_10549458, EPI_ISL_10549459, EPI_ISL_10549467	PEJABAT KESIHATAN PONTIAN	UKM Medical Molecular Biology Institute (UMBI)	Khairun Nur Abd Ghafar; Mira Farzana Mohamad Mokhtar; Muhiddin Ishak; Nor Azila Muhammad Azami; Nur Alyaa Afifah Md Shahri; Nurul Syakima Ab Mutalib; Rahman Jamal; Ryia Illani Mohd Yunos; Siti Nur Hasanah Mohd Yusuf; Zahirrah Begam Mohamed Rasheed
EPI_ISL_11112478, EPI_ISL_11939040	PKD BARAT DAYA	iPROMISE, UiTM	Ariza Adnan; Fadzilah Mohd Nor; Lim Wai Feng; Mohd Asif Mohd Sukri; Mohd Nur Fakhruzzaman Noorizhab; Mohd Zaki Salleh; Sazzli Shahlan Kassim; Siti Farah Alwani Mohd Nawi; Siti Hamimah Sheikh Abdul Kadir; Teh Lay Kek; Wang Seok Mui
EPI_ISL_11754746, EPI_ISL_11754747	PKD GUA MUSANG	Department of Medical Microbiology, Faculty of Medicine, University of Malaya; University of Malaya Medical Centre	I-Ching SAM; Jolene Yin Ling FU; Omar Khalilur Rahman; Yoke Fun Chan
EPI_ISL_10810893, EPI_ISL_10810896, EPI_ISL_10810944, EPI_ISL_10810945, EPI_ISL_10810946, EPI_ISL_10810947, EPI_ISL_10810948, EPI_ISL_10810949, EPI_ISL_12534781, EPI_ISL_12534789			
see above	PKD HILIR PERAK	Tropical Infectious Diseases Research & Education Centre (TIDREC), Universiti Malaya	AsmaAnati CheMatSeri; Che-Norainon Yaacob; Jia-Yi Tan; Jo-Ern Wong; Kim-Kee Tan; Mulya-Mustika-Sari Zulkifli; Noor Syahida Azizan; Nur-Hidayana Mahfodz; Sazaly AbuBakar; Siti-Sarah Nor'e; Wei-Wei Aw
EPI_ISL_10942298, EPI_ISL_10942307, EPI_ISL_10942323	PKD JOHOR BAHRU	UKM Medical Molecular Biology Institute (UMBI)	Khairun Nur Abd Ghafar; Mira Farzana Mohamad Mokhtar; Muhiddin Ishak; Nor Azila Muhammad Azami; Nur Alyaa Afifah Md Shahri; Nurul Syakima Ab Mutalib; Rahman Jamal; Ryia Illani Mohd Yunos; Siti Nur Hasanah Mohd Yusuf; Zahirrah Begam Mohamed Rasheed
EPI_ISL_10549456, EPI_ISL_10549469, EPI_ISL_10549471, EPI_ISL_10549473, EPI_ISL_10549474, EPI_ISL_10549477, EPI_ISL_10549478			
see above	PKD KLUANG	UKM Medical Molecular Biology Institute (UMBI)	Khairun Nur Abd Ghafar; Mira Farzana Mohamad Mokhtar; Muhiddin Ishak; Nor Azila Muhammad Azami; Nur Alyaa Afifah Md Shahri; Nurul Syakima Ab Mutalib; Rahman Jamal; Ryia Illani Mohd Yunos; Siti Nur Hasanah Mohd Yusuf; Zahirrah Begam Mohamed Rasheed
EPI_ISL_11754749, EPI_ISL_11754751, EPI_ISL_11754767	PKD KOTA BHARU	Department of Medical Microbiology, Faculty of Medicine, University of Malaya; University of Malaya Medical Centre	I-Ching SAM; Jolene Yin Ling FU; Omar Khalilur Rahman; Yoke Fun Chan
EPI_ISL_10397060, EPI_ISL_10397061, EPI_ISL_10397076, EPI_ISL_10397120, EPI_ISL_10397124	PKD KUALA PILAH	UKM Medical Molecular Biology Institute (UMBI)	Khairun Nur Abd Ghafar; Mira Farzana Mohamad Mokhtar; Muhiddin Ishak; Nor Azila Muhammad Azami; Nur Alyaa Afifah Md Shahri; Nurul Syakima Ab Mutalib; Rahman Jamal; Ryia Illani Mohd Yunos; Siti Nur Hasanah Mohd Yusuf; Zahirrah Begam Mohamed Rasheed
EPI_ISL_10549455, EPI_ISL_10549468	PKD KULAI	UKM Medical Molecular Biology Institute (UMBI)	Khairun Nur Abd Ghafar; Mira Farzana Mohamad Mokhtar; Muhiddin Ishak; Nor Azila Muhammad Azami; Nur Alyaa Afifah Md Shahri; Nurul Syakima Ab Mutalib; Rahman Jamal; Ryia Illani Mohd Yunos; Siti Nur Hasanah Mohd Yusuf; Zahirrah Begam Mohamed Rasheed
EPI_ISL_10810894	PKD LARUT MATANG DAN SELAMA	Tropical Infectious Diseases Research & Education Centre (TIDREC), Universiti Malaya	AsmaAnati CheMatSeri; Che-Norainon Yaacob; Jia-Yi Tan; Jo-Ern Wong; Kim-Kee Tan; Mulya-Mustika-Sari Zulkifli; Noor Syahida Azizan; Nur-Hidayana Mahfodz; Sazaly AbuBakar; Siti-Sarah Nor'e
EPI_ISL_10880075, EPI_ISL_10880107	PKD MELAKA TENGAH	UKM Medical Molecular Biology Institute (UMBI)	Khairun Nur Abd Ghafar; Mira Farzana Mohamad Mokhtar; Muhiddin Ishak; Nor Azila Muhammad Azami; Nur Alyaa Afifah Md Shahri; Nurul Syakima Ab Mutalib; Rahman Jamal; Ryia Illani Mohd Yunos; Siti Nur Hasanah Mohd Yusuf; Zahirrah Begam Mohamed Rasheed
EPI_ISL_10810895, EPI_ISL_12136030, EPI_ISL_12534788	PKD PERAK TENGAH	Tropical Infectious Diseases Research & Education Centre (TIDREC), Universiti Malaya	AsmaAnati CheMatSeri; Che-Norainon Yaacob; Jia-Yi Tan; Jo-Ern Wong; Kim-Kee Tan; Mulya-Mustika-Sari Zulkifli; Noor Syahida Azizan; Nur-Hidayana Mahfodz; Sazaly AbuBakar; Siti-Sarah Nor'e; Wei-Wei Aw
EPI_ISL_10397087	PKD SEGAMAT	UKM Medical Molecular Biology Institute (UMBI)	Khairun Nur Abd Ghafar; Mira Farzana Mohamad Mokhtar; Muhiddin Ishak; Nor Azila Muhammad Azami; Nur Alyaa Afifah Md Shahri; Nurul Syakima Ab Mutalib; Rahman Jamal; Ryia Illani Mohd Yunos; Siti Nur Hasanah Mohd Yusuf; Zahirrah Begam Mohamed Rasheed
EPI_ISL_12008492	PKD SEREMBAN	UKM Medical Molecular Biology Institute (UMBI)	Khairun Nur Abd Ghafar; Mira Farzana Mohamad Mokhtar; Muhiddin Ishak; Nor Azila Muhammad Azami; Nur Alyaa Afifah Md Shahri; Nurul Syakima Ab Mutalib; Rahman Jamal; Ryia Illani Mohd Yunos; Siti Nur Hasanah Mohd Yusuf; Zahirrah Begam Mohamed Rasheed
EPI_ISL_11112481, EPI_ISL_11117191, EPI_ISL_11117192, EPI_ISL_11117193, EPI_ISL_11117194, EPI_ISL_11117195, EPI_ISL_11117196, EPI_ISL_11117204, EPI_ISL_11117206, EPI_ISL_11117208, EPI_ISL_11117209, EPI_ISL_11939034, EPI_ISL_11939035, EPI_ISL_11939037, EPI_ISL_11939039, EPI_ISL_11939080, EPI_ISL_11939084			
see above	PKD TIMUR LAUT	iPROMISE, UiTM	Ariza Adnan; Fadzilah Mohd Nor; Lim Wai Feng; Mohd Asif Mohd Sukri; Mohd Nur Fakhruzzaman Noorizhab; Mohd Zaki Salleh; Sazzli Shahlan Kassim; Siti Farah Alwani Mohd Nawi; Siti Hamimah Sheikh Abdul Kadir; Teh Lay Kek; Wang Seok Mui
EPI_ISL_11754756	PKD Tanah Merah	Department of Medical Microbiology, Faculty of Medicine, University of Malaya; University of Malaya Medical Centre	I-Ching SAM; Jolene Yin Ling FU; Omar Khalilur Rahman; Yoke Fun Chan
EPI_ISL_11754760	Pejabat Kesihatan Daerah Jeli	Department of Medical Microbiology, Faculty of Medicine, University of Malaya; University of Malaya Medical Centre	I-Ching SAM; Jolene Yin Ling FU; Omar Khalilur Rahman; Yoke Fun Chan
EPI_ISL_11754713, EPI_ISL_11754739, EPI_ISL_11754741, EPI_ISL_11754742, EPI_ISL_11754769, EPI_ISL_11754777	Pejabat Kesihatan Daerah Tumpat	Department of Medical Microbiology, Faculty of Medicine, University of Malaya; University of Malaya Medical Centre	I-Ching SAM; Jolene Yin Ling FU; Omar Khalilur Rahman; Yoke Fun Chan
EPI_ISL_11939038	Pejabat Kesihatan Pintu Masuk Antarabangsa Pulau Pinang	iPROMISE, UiTM	Ariza Adnan; Fadzilah Mohd Nor; Lim Wai Feng; Mohd Asif Mohd Sukri; Mohd Nur Fakhruzzaman Noorizhab; Mohd Zaki Salleh; Sazzli Shahlan Kassim; Siti Farah Alwani Mohd Nawi; Siti Hamimah Sheikh Abdul Kadir; Teh Lay Kek; Wang Seok Mui
EPI_ISL_9092726	Port Klang Health Clinic	Institute for Medical Research, Infectious Disease Research Centre, National Institutes of Health, Ministry of Health Malaysia	Ahmad FA; Ahmad Fazilah NA; Anasir MI; Azizan MA; Kamel K; Mohamad Sukri MZ; Norhisham SN; Ramly N; Robert F; Rosli NR; Suppiah J; Thayan R
EPI_ISL_9092758, EPI_ISL_9092759, EPI_ISL_9092760	Putrajaya District Health Office	Institute for Medical Research, Infectious Disease Research Centre, National Institutes of Health, Ministry of Health Malaysia	Ahmad FA; Ahmad Fazilah NA; Anasir MI; Azizan MA; Kamel K; Mohamad Sukri MZ; Norhisham SN; Ramly N; Robert F; Rosli NR; Suppiah J; Thayan R
EPI_ISL_10495842, EPI_ISL_10495850, EPI_ISL_10495856, EPI_ISL_10495873	QUEEN ELIZABETH HOSPITAL	Institute for Medical Research, Infectious Disease Research Centre, National Institutes of Health, Ministry of Health Malaysia	Ahmad FA; Ahmad Fazilah NA; Anasir MI; Arip MS; Azizan MA; Christopher Hollip K; Jaimin JJ; Kamel K; Mohamad Sukri MZ; Mohammad R; Mohd Yasin R; Norhisham SN; Ramly N; Robert F; Rosli NR; Shamsaddin E; Suppiah J; Tan YC; Thayan R
EPI_ISL_10297349, EPI_ISL_10297352, EPI_ISL_10297353	Queen Elizabeth Hospital	Institute for Medical Research, Infectious Disease Research Centre, National Institutes of Health, Ministry of Health Malaysia	Ahmad FA; Ahmad Fazilah NA; Anasir MI; Azizan MA; Kamel K; Mohamad Sukri MZ; Norhisham SN; Ramly N; Robert F; Rosli NR; Suppiah J; Thayan R
EPI_ISL_8782836	Rejang Medical Centre (Sibu)	Institute of Health and Community Medicine	Chan Chia Jui; Chua Hock Hin; David Perera; Ooi Mong How; Tonnii Sia Loong Loong; Wong Jyn Shan
EPI_ISL_9092728, EPI_ISL_9092729	Salak Health Clinic	Institute for Medical Research, Infectious Disease Research Centre, National Institutes of Health, Ministry of Health Malaysia	Ahmad FA; Ahmad Fazilah NA; Anasir MI; Azizan MA; Kamel K; Mohamad Sukri MZ; Norhisham SN; Ramly N; Robert F; Rosli NR; Suppiah J; Thayan R
EPI_ISL_10297357	Sandakan Health Clinic	Institute for Medical Research, Infectious Disease Research Centre, National Institutes of Health, Ministry of Health Malaysia	Ahmad FA; Ahmad Fazilah NA; Anasir MI; Azizan MA; Kamel K; Mohamad Sukri MZ; Norhisham SN; Ramly N; Robert F; Rosli NR; Suppiah J; Thayan R
EPI_ISL_8650064, EPI_ISL_8650065, EPI_ISL_8650069, EPI_ISL_8650133, EPI_ISL_8650135, EPI_ISL_8650136, EPI_ISL_8650137, EPI_ISL_8650142, EPI_ISL_8650143, EPI_ISL_8650144, EPI_ISL_8782775, EPI_ISL_8782778, EPI_ISL_8782779, EPI_ISL_8782780, EPI_ISL_8782781, EPI_ISL_8782796, EPI_ISL_8782803			
see above	Sarawak General Hospital (Kuching)	Institute of Health and Community Medicine	Chan Chia Jui; Chua Hock Hin; David Perera; Ooi Mong How; Tonnii Sia Loong Loong; Wong Jyn Shan

EPI_ISL_9092763, EPI_ISL_9092764, EPI_ISL_9842681	Sepang District Health Office	Institute for Medical Research, Infectious Disease Research Centre, National Institutes of Health, Ministry of Health Malaysia	Ahmad FA; Ahmad Fazilah NA; Anasir MI; Azizan MA; Kamel K; Mohamad Sukri MZ; Norhisham SN; Ramly N; Robert F; Rosli NR; Suppiah J; Thayan R
EPI_ISL_8782782, EPI_ISL_8782783, EPI_ISL_8782784, EPI_ISL_8782785, EPI_ISL_8782786, EPI_ISL_8782793, EPI_ISL_8782797, EPI_ISL_8782798, EPI_ISL_9608512, EPI_ISL_9608528, EPI_ISL_9608535			
see above	Sibu Hospital (Sibu, Sarawak)	Institute of Health and Community Medicine	Chan Chia Jui; Chua Hock Hin; David Perera; Ooi Mong How; Tonnii Sia Loong Loong
EPI_ISL_9092733	Sijangkang Health Clinic	Institute for Medical Research, Infectious Disease Research Centre, National Institutes of Health, Ministry of Health Malaysia	Ahmad FA; Ahmad Fazilah NA; Anasir MI; Azizan MA; Kamel K; Mohamad Sukri MZ; Norhisham SN; Ramly N; Robert F; Rosli NR; Suppiah J; Thayan R
EPI_ISL_10495880	TAWAU AREA HEALTH OFFICE	Institute for Medical Research, Infectious Disease Research Centre, National Institutes of Health, Ministry of Health Malaysia	Ahmad FA; Ahmad Fazilah NA; Anasir MI; Arip MS; Azizan MA; Christopher Hollip K; Jaimin JJ; Kamel K; Mohamad Sukri MZ; Mohammad R; Mohd Yasin R; Norhisham SN; Ramly N; Robert F; Rosli NR; Shamsaddin E; Suppiah J; Tan YC; Thayan R
EPI_ISL_9092734, EPI_ISL_9092735, EPI_ISL_9092736, EPI_ISL_9092737, EPI_ISL_9092738	Telok Datok Health Clinic	Institute for Medical Research, Infectious Disease Research Centre, National Institutes of Health, Ministry of Health Malaysia	Ahmad FA; Ahmad Fazilah NA; Anasir MI; Azizan MA; Kamel K; Mohamad Sukri MZ; Norhisham SN; Ramly N; Robert F; Rosli NR; Suppiah J; Thayan R
EPI_ISL_9092739, EPI_ISL_9092740	Telok Panglima Garang Health Clinic	Institute for Medical Research, Infectious Disease Research Centre, National Institutes of Health, Ministry of Health Malaysia	Ahmad FA; Ahmad Fazilah NA; Anasir MI; Azizan MA; Kamel K; Mohamad Sukri MZ; Norhisham SN; Ramly N; Robert F; Rosli NR; Suppiah J; Thayan R
EPI_ISL_9092765, EPI_ISL_9092766, EPI_ISL_9092767	Titiwangsa District Health Office	Institute for Medical Research, Infectious Disease Research Centre, National Institutes of Health, Ministry of Health Malaysia	Ahmad FA; Ahmad Fazilah NA; Anasir MI; Azizan MA; Kamel K; Mohamad Sukri MZ; Norhisham SN; Ramly N; Robert F; Rosli NR; Suppiah J; Thayan R
EPI_ISL_10297337	Tuaran Hospital	Institute for Medical Research, Infectious Disease Research Centre, National Institutes of Health, Ministry of Health Malaysia	Ahmad FA; Ahmad Fazilah NA; Anasir MI; Azizan MA; Kamel K; Mohamad Sukri MZ; Norhisham SN; Ramly N; Robert F; Rosli NR; Suppiah J; Thayan R
EPI_ISL_12534873	UNIT KESELAMATAN DAN KESIHATAN PEKERJA HOSPITAL SULTANAH BAHIYAH	Tropical Infectious Diseases Research & Education Centre (TIDREC), Universiti Malaya	AsmaAnati CheMatSeri; Che-Norainon Yaacob; Jia-Yi Tan; Jo-Ern Wong; Kim-Kee Tan; Mulya-Mustika-Sari Zulkifli; Noor Syahida Azizan; Nur-Hidayana Mahfodz; Sazaly AbuBakar; Siti-Sarah Nor'e; Wei-Wei Aw
EPI_ISL_11939086	UNIT PERUBATAN FORENSIK, HOSPITAL SEBERANG JAYA	iPROMISE, UiTM	Ariza Adnan; Fadzilah Mohd Nor; Lim Wai Feng; Mohd Asif Mohd Sukri; Mohd Nur Fakhruzzaman Noorizhab; Mohd Zaki Salleh; Sazzli Shahlan Kassim; Siti Farah Alwani Mohd Nawi; Siti Hamimah Sheikh Abdul Kadir; Teh Lay Kek; Wang Seok Mui
EPI_ISL_12534779	pkd manjung	Tropical Infectious Diseases Research & Education Centre (TIDREC), Universiti Malaya	AsmaAnati CheMatSeri; Che-Norainon Yaacob; Jia-Yi Tan; Jo-Ern Wong; Kim-Kee Tan; Mulya-Mustika-Sari Zulkifli; Noor Syahida Azizan; Nur-Hidayana Mahfodz; Sazaly AbuBakar; Siti-Sarah Nor'e; Wei-Wei Aw

We gratefully acknowledge the following Authors from the Originating laboratories responsible for obtaining the specimens, as well as the Submitting laboratories where the genome data were generated and shared via GISAID, on which this research is based.

All Submitters of data may be contacted directly via www.gisaid.org

Authors are sorted alphabetically.

Accession ID	Originating Laboratory	Submitting Laboratory	Authors
EPI_ISL_10942315	3A HTJS	UKM Medical Molecular Biology Institute (UMBI)	Khairun Nur Abd Ghafar; Mira Farzana Mohamad Mokhtar; Muhiddin Ishak; Nor Azila Muhammad Azami; Nur Alyaa Afifah Md Shahri; Nurul Syakima Ab Mutalib; Rahman Jamal; Ryia Illani Mohd Yunos; Siti Nur Hasanah Mohd Yusuf; Zahirrah Begam Mohamed Rasheed
EPI_ISL_11900019, EPI_ISL_11900022, EPI_ISL_11900023, EPI_ISL_11900029, EPI_ISL_11900030, EPI_ISL_11900031, EPI_ISL_11900032			
see above	BP Clinical Lab Sdn Bhd (KLIA)	Malaysia Genome and Vaccine Institute	Azrin Ahmad; Enizza Kasim; Irni Suhayu Sapian; Mohd Faizal Abu Bakar; Mohd Ghows Mohd Azzam.; Mohd Noor Mat Isa; Nor Azfa Johari; Nurhezreen Md Iqbal; Shamsidar Sopie; Siti Noraini Othman; Yusuf Muhammad Noor
EPI_ISL_9092720, EPI_ISL_9092721, EPI_ISL_10297304, EPI_ISL_10297305, EPI_ISL_10297306	Bukit Changgang Health Clinic	Institute for Medical Research, Infectious Disease Research Centre, National Institutes of Health, Ministry of Health Malaysia	Ahmad FA; Ahmad Fazilah NA; Anasir MI; Azizan MA; Kamel K; Mohamad Sukri MZ; Norhisham SN; Ramly N; Robert F; Rosli NR; Suppiah J; Thayan R
EPI_ISL_9092724	Bukit Kuda Health Clinic	Institute for Medical Research, Infectious Disease Research Centre, National Institutes of Health, Ministry of Health Malaysia	Ahmad FA; Ahmad Fazilah NA; Anasir MI; Azizan MA; Kamel K; Mohamad Sukri MZ; Norhisham SN; Ramly N; Robert F; Rosli NR; Suppiah J; Thayan R
EPI_ISL_10431128, EPI_ISL_10431131, EPI_ISL_10431137, EPI_ISL_10431147, EPI_ISL_10431148	Department of Medical Microbiology and Parasitology, Hospital Universiti Sains Malaysia	Molecular Research Laboratory	Abdul Haris bin Muhammad; Ahmad Sukari Bin Halim; Alexander Chong Shu Chien; Azian Harun; Chan Yean Yean; Chua Wei Chuan; Farahana binti Mohamed; Kirnpal Kaur Banga Singh; Lau Nyok Sean; Lee Lih Huey; Lim Shu Yong; Maizun binti Mohd Zain; Muhammad Azamuddeen bin Mohammad Nasir; Muhammad Fazli bin Khalid; Muhammad Nashrul Farhan Samsudin; Muhammad Zarul Hanifah Bin Md Zoqratt; Noor Hafizan binti Mat Salleh; Nurfadhlina Musa; Qasim Ayub; Rosline Hassan; Sadequr Rahman; Syahida binti Omar; Wan Mohd Zahiruddin Wan Mohammad; Wardah Yusof; Zaini bin Hussin; Zakuan Zainy Deris
EPI_ISL_11344843, EPI_ISL_11344844, EPI_ISL_11344845, EPI_ISL_11344846, EPI_ISL_11344847, EPI_ISL_11344848, EPI_ISL_11344849, EPI_ISL_11344850, EPI_ISL_11344851, EPI_ISL_12914933			
see above	Department of Medical Microbiology and Parasitology, School of Medical Sciences, Universiti Sains Malaysia Health Campus	Molecular Research Laboratory, Department of Medical Microbiology and Parasitology, School of Medical Sciences, Universiti Sains Malaysia Health Campus	Abdul Haris bin Muhammad; Ahmad Sukari Bin Halim; Alexander Chong Shu Chien; Asraihan Che Abdul Malik; Azian Harun; Chan Yean Yean; Chua Wei Chuan; Farahana binti Mohamed; Kirnpal Kaur Banga Singh; Lau Nyok Sean; Lee Lih Huey; Lim Shu Yong; Maizun binti Mohd Zain; Mera Edora Binti Abdul Manap; Mohd Iman hafiz Bin Ibrahim; Mohd Nadzri Abu Yazid; Mohd Zulkifli Salleh; Muhamad Khairul Amirin Bin Zulkifli; Muhammad Azamuddeen bin Mohammad Nasir; Muhammad Fazli bin Khalid; Muhammad Nashrul Farhan Samsudin; Muhammad Zarul Hanifah Bin Md Zoqratt; Nik Zuraina binti Nik Mohd Noor; Noor Hafizan binti Mat Salleh; Nor Amizara Binti Azami; Nor Suhana Binti Mohd Satar; Nur Syuhada Binti Abdul Rahim; Nur-Leem Binti Murshid; Nurfadhlina Musa; Qasim Ayub; Rosline Hassan; Sadequr Rahman; Siti Nur Aisyah Binti Mohamad Sham; Syahida binti Omar; Wan Mohd Zahiruddin Wan Mohammad; Wardah Yusof; Zaini bin Hussin; Zakuan Zainy Deris
EPI_ISL_12721592, EPI_ISL_12721601, EPI_ISL_12721611	Department of Medical Microbiology, Hospital Pengajar Universiti Putra Malaysia	Department of Medical Microbiology, Hospital Pengajar Universiti Putra Malaysia	Afiqah Adzmi; Amiza Azmi; Azmiza Syawani Jasni; Hui Yee Chee; Leslie Thian Lung Than; Muadz Mohtar; Muhammad Mohd Isa; Narcisse MS Joseph; Niazlin Mohd Taib; Noor Hazirah Noor Azhari; Norlaila; Nur Raihana Ithnin; Nurul Huda Mohamed Rashidi; Nurul Nadiah Ismail; Rosni Ibrahim; Rukman Awang Hamat; Sallehhudin; Siti Norbaya Masri; Siti Zulaikha Zakariah; Syafinaz Amin Nordin; Tengku Zetty Maztura Tengku Jamaluddin; Zamberi Sekawi
EPI_ISL_9608514, EPI_ISL_9608534	Gribbles Pathology, Kuching	Institute of Health and Community Medicine	Chan Chia Jui; Chua Hock Hin; David Perera; Liew Chin Chin J; Ooi Mong How; Tonnii Sia Loong Loong; Wong Jyn Shan
EPI_ISL_11939043, EPI_ISL_11939044, EPI_ISL_11939045, EPI_ISL_11939046, EPI_ISL_11939047, EPI_ISL_11939048	HOSPITAL PULAU PINANG	iPROMISE, UiTM	Ariza Adnan; Fadzilah Mohd Nor; Lim Wai Feng; Mohd Asif Mohd Sukri; Mohd Nur Fakhruzzaman Noorizhab; Mohd Zaki Salleh; Sazzli Shahlan Kassim; Siti Farah Alwani Mohd Nawi; Siti Hamimah Sheikh Abdul Kadir; Teh Lay Kek; Wang Seok Mui
EPI_ISL_10810897, EPI_ISL_10810898, EPI_ISL_10810899, EPI_ISL_10810950, EPI_ISL_10810951, EPI_ISL_10810952, EPI_ISL_10810953			
see above	HOSPITAL RAJA PERMAISURI BAINUN	Tropical Infectious Diseases Research & Education Centre (TIDREC), Universiti Malaya	AsmaAnati CheMatSeri; Che-Norainon Yaacob; Jia-Yi Tan; Jo-Ern Wong; Kim-Kee Tan; Mulya-Mustika-Sari Zulkifli; Noor Syahida Azizan; Nur-Hidayana Mahfodz; Sazaly AbuBakar; Siti-Sarah Nor'e
EPI_ISL_10692529	HOSPITAL SEGAMAT	UKM Medical Molecular Biology Institute (UMBI)	Khairun Nur Abd Ghafar; Mira Farzana Mohamad Mokhtar; Muhiddin Ishak; Nor Azila Muhammad Azami; Nur Alyaa Afifah Md Shahri; Nurul Syakima Ab Mutalib; Rahman Jamal; Ryia Illani Mohd Yunos; Siti Nur Hasanah Mohd Yusuf; Zahirrah Begam Mohamed Rasheed
EPI_ISL_10942327, EPI_ISL_10942356	HOSPITAL SULTANAH AMINAH JOHOR BAHRU	UKM Medical Molecular Biology Institute (UMBI)	Khairun Nur Abd Ghafar; Mira Farzana Mohamad Mokhtar; Muhiddin Ishak; Nor Azila Muhammad Azami; Nur Alyaa Afifah Md Shahri; Nurul Syakima Ab Mutalib; Rahman Jamal; Ryia Illani Mohd Yunos; Siti Nur Hasanah Mohd Yusuf; Zahirrah Begam Mohamed Rasheed
EPI_ISL_10810954, EPI_ISL_10810956, EPI_ISL_10810957, EPI_ISL_10810971, EPI_ISL_10810972, EPI_ISL_10810973, EPI_ISL_10810974			
see above	HOSPITAL SULTANAH NUR ZAHIRAH	Tropical Infectious Diseases Research & Education Centre (TIDREC), Universiti Malaya	AsmaAnati CheMatSeri; Che-Norainon Yaacob; Jia-Yi Tan; Jo-Ern Wong; Kim-Kee Tan; Mulya-Mustika-Sari Zulkifli; Noor Syahida Azizan; Nur-Hidayana Mahfodz; Sazaly AbuBakar; Siti-Sarah Nor'e
EPI_ISL_10810955	HOSPITAL SULTANAH NURZAHIRAH	Tropical Infectious Diseases Research & Education Centre (TIDREC), Universiti Malaya	AsmaAnati CheMatSeri; Che-Norainon Yaacob; Jia-Yi Tan; Jo-Ern Wong; Kim-Kee Tan; Mulya-Mustika-Sari Zulkifli; Noor Syahida Azizan; Nur-Hidayana Mahfodz; Sazaly AbuBakar; Siti-Sarah Nor'e
EPI_ISL_10495865	HOSPITAL TAWAU	Institute for Medical Research, Infectious Disease Research Centre, National Institutes of Health, Ministry of Health Malaysia	Ahmad FA; Ahmad Fazilah NA; Anasir MI; Arip MS; Azizan MA; Christopher Hollip K; Jaimin JJ; Kamel K; Mohamad Sukri MZ; Mohammad R; Mohd Yasin R; Norhisham SN; Ramly N; Robert F; Rosli NR; Shamsaddin E; Suppiah J; Tan YC; Thayan R
EPI_ISL_10810942, EPI_ISL_10969001, EPI_ISL_10969026, EPI_ISL_10969027, EPI_ISL_10969028, EPI_ISL_10969075, EPI_ISL_12534790, EPI_ISL_12534791, EPI_ISL_12534797			
see above	HRPB	Tropical Infectious Diseases Research & Education Centre (TIDREC), Universiti Malaya	AsmaAnati CheMatSeri; Che-Norainon Yaacob; Jia-Yi Tan; Jo-Ern Wong; Kim-Kee Tan; Mulya-Mustika-Sari Zulkifli; Noor Syahida Azizan; Nur-Hidayana Mahfodz; Sazaly AbuBakar; Siti-Sarah Nor'e; Wei-Wei Aw
EPI_ISL_10810901, EPI_ISL_10810902, EPI_ISL_10810903, EPI_ISL_10810904, EPI_ISL_10810905, EPI_ISL_10810906, EPI_ISL_10810907, EPI_ISL_10810908, EPI_ISL_10810909, EPI_ISL_10810910, EPI_ISL_10810911, EPI_ISL_10810912, EPI_ISL_10810961, EPI_ISL_10810962, EPI_ISL_10810963, EPI_ISL_10810964, EPI_ISL_10810965			
see above	HRPZ II	Tropical Infectious Diseases Research & Education Centre (TIDREC), Universiti Malaya	AsmaAnati CheMatSeri; Che-Norainon Yaacob; Jia-Yi Tan; Jo-Ern Wong; Kim-Kee Tan; Mulya-Mustika-Sari Zulkifli; Noor Syahida Azizan; Nur-Hidayana Mahfodz; Sazaly AbuBakar; Siti-Sarah Nor'e
EPI_ISL_10692488, EPI_ISL_10692505, EPI_ISL_10692517, EPI_ISL_10692521, EPI_ISL_10942289	HSAJB	UKM Medical Molecular Biology Institute (UMBI)	Khairun Nur Abd Ghafar; Mira Farzana Mohamad Mokhtar; Muhiddin Ishak; Nor Azila Muhammad Azami; Nur Alyaa Afifah Md Shahri; Nurul Syakima Ab Mutalib; Rahman Jamal; Ryia Illani Mohd Yunos; Siti Nur Hasanah Mohd Yusuf; Zahirrah Begam Mohamed Rasheed
EPI_ISL_10942292, EPI_ISL_10942306, EPI_ISL_10942310, EPI_ISL_10942337, EPI_ISL_10942341	HTJ	UKM Medical Molecular Biology Institute (UMBI)	Khairun Nur Abd Ghafar; Mira Farzana Mohamad Mokhtar; Muhiddin Ishak; Nor Azila Muhammad Azami; Nur Alyaa Afifah Md Shahri; Nurul Syakima Ab Mutalib; Rahman Jamal; Ryia Illani Mohd Yunos; Siti Nur Hasanah Mohd Yusuf; Zahirrah Begam Mohamed Rasheed
EPI_ISL_10942322, EPI_ISL_10942345, EPI_ISL_10942350	HTJS	UKM Medical Molecular Biology Institute (UMBI)	Khairun Nur Abd Ghafar; Mira Farzana Mohamad Mokhtar; Muhiddin Ishak; Nor Azila Muhammad Azami; Nur Alyaa Afifah Md Shahri; Nurul Syakima Ab Mutalib; Rahman Jamal; Ryia Illani Mohd Yunos; Siti Nur Hasanah Mohd Yusuf; Zahirrah Begam Mohamed Rasheed
EPI_ISL_10810943	Hospital Alor Setar	Tropical Infectious Diseases Research & Education Centre (TIDREC), Universiti Malaya	AsmaAnati CheMatSeri; Che-Norainon Yaacob; Jia-Yi Tan; Jo-Ern Wong; Kim-Kee Tan; Mulya-Mustika-Sari Zulkifli; Noor Syahida Azizan; Nur-Hidayana Mahfodz; Sazaly AbuBakar; Siti-Sarah Nor'e
EPI_ISL_10590727, EPI_ISL_10590728, EPI_ISL_10590780, EPI_ISL_11899997, EPI_ISL_11899998, EPI_ISL_11976172	Hospital Ampang	Malaysia Genome and Vaccine Institute	Azrin Ahmad; Enizza Kasim; Irni Suhayu Sapian; Mohd Faizal Abu Bakar; Mohd Ghows Mohd Azzam.; Mohd Noor Mat Isa; Nor Azfa Johari; Nurhezreen Md Iqbal; Shamsidar Sopie; Siti Noraini Othman; Yusuf Muhammad Noor
EPI_ISL_10590729, EPI_ISL_10590774, EPI_ISL_10590787, EPI_ISL_11899981, EPI_ISL_11899982, EPI_ISL_11899983, EPI_ISL_11899987, EPI_ISL_11899988, EPI_ISL_11899989, EPI_ISL_11899990, EPI_ISL_12277129			
see above	Hospital Kuala Lumpur	Malaysia Genome and Vaccine Institute	Azrin Ahmad; Enizza Kasim; Irni Suhayu Sapian; Mohd Faizal Abu Bakar; Mohd Ghows Mohd Azzam.; Mohd Noor Mat Isa; Nor Azfa Johari; Nurhezreen Md Iqbal; Shamsidar Sopie; Siti Noraini Othman; Yusuf Muhammad Noor
EPI_ISL_11939042	Hospital Pulau Pinang	iPROMISE, UiTM	Ariza Adnan; Fadzilah Mohd Nor; Lim Wai Feng; Mohd Asif Mohd Sukri; Mohd Nur Fakhruzzaman Noorizhab; Mohd Zaki Salleh; Sazzli Shahlan Kassim; Siti Farah Alwani Mohd Nawi; Siti Hamimah Sheikh Abdul Kadir; Teh Lay Kek; Wang Seok Mui
EPI_ISL_11899966	Hospital Putrajaya	Malaysia Genome and Vaccine Institute	Azrin Ahmad; Enizza Kasim; Irni Suhayu Sapian; Mohd Faizal Abu Bakar; Mohd Ghows Mohd Azzam.; Mohd Noor Mat Isa; Nor Azfa Johari; Nurhezreen Md Iqbal; Shamsidar Sopie; Siti Noraini Othman; Yusuf Muhammad Noor
EPI_ISL_10810960	Hospital Raja Perempuan Zainab II	Tropical Infectious Diseases Research & Education Centre (TIDREC), Universiti Malaya	AsmaAnati CheMatSeri; Che-Norainon Yaacob; Jia-Yi Tan; Jo-Ern Wong; Kim-Kee Tan; Mulya-Mustika-Sari Zulkifli; Noor Syahida Azizan; Nur-Hidayana Mahfodz; Sazaly AbuBakar; Siti-Sarah Nor'e
EPI_ISL_11900049	Hospital Selayang	Malaysia Genome and Vaccine Institute	Azrin Ahmad; Enizza Kasim; Irni Suhayu Sapian; Mohd Faizal Abu Bakar; Mohd Ghows Mohd Azzam.; Mohd Noor Mat Isa; Nor Azfa Johari; Nurhezreen Md Iqbal; Shamsidar Sopie; Siti Noraini Othman; Yusuf Muhammad Noor
EPI_ISL_10692509,	Hospital Sultanah Aminah Johor	UKM Medical Molecular Biology Institute	Khairun Nur Abd Ghafar; Mira Farzana Mohamad Mokhtar; Muhiddin Ishak; Nor Azila Muhammad Azami; Nur Alyaa Afifah Md Shahri; Nurul Syakima Ab Mutalib; Rahman Jamal; Ryia Illani Mohd Yunos; Siti Nur Hasanah Mohd Yusuf; Zahirrah Begam Mohamed Rasheed

EPI_ISL_10692530	Bahru	(UMBI)	
EPI_ISL_10590704, EPI_ISL_10590706, EPI_ISL_10590768, EPI_ISL_10590777, EPI_ISL_10590786, EPI_ISL_10590790, EPI_ISL_10590793, EPI_ISL_10590795, EPI_ISL_11900000, EPI_ISL_11900001, EPI_ISL_11900002, EPI_ISL_11900003, EPI_ISL_11900004, EPI_ISL_11900005, EPI_ISL_11900006, EPI_ISL_11900007, EPI_ISL_11900008, EPI_ISL_11900009, EPI_ISL_11900010, EPI_ISL_11900011, EPI_ISL_11900012, EPI_ISL_11976176, EPI_ISL_11976177, EPI_ISL_11976178, EPI_ISL_11976179, EPI_ISL_11976180, EPI_ISL_11976181, EPI_ISL_11976188			
see above	Hospital Sungai Buloh	Malaysia Genome and Vaccine Institute	Azrin Ahmad; Enizza Kasim; Irni Suhayu Sapian; Mohd Faizal Abu Bakar; Mohd Ghows Mohd Azzam.; Mohd Noor Mat Isa; Nor Azfa Johari; Nurhezreen Md Iqbal; Shamsidar Sopie; Siti Noraini Othman; Yusuf Muhammad Noor
EPI_ISL_11899986	Hospital Tengku Ampuan Rahimah	Malaysia Genome and Vaccine Institute	Azrin Ahmad; Enizza Kasim; Irni Suhayu Sapian; Mohd Faizal Abu Bakar; Mohd Ghows Mohd Azzam.; Mohd Noor Mat Isa; Nor Azfa Johari; Nurhezreen Md Iqbal; Shamsidar Sopie; Siti Noraini Othman; Yusuf Muhammad Noor
EPI_ISL_10810913, EPI_ISL_10810914, EPI_ISL_10810916	IMR	Tropical Infectious Diseases Research & Education Centre (TIDREC), Universiti Malaya	AsmaAnati CheMatSeri; Che-Norainon Yaacob; Jia-Yi Tan; Jo-Ern Wong; Kim-Kee Tan; Mulya-Mustika-Sari Zulkifli; Noor Syahida Azizan; Nur-Hidayana Mahfodz; Sazaly AbuBakar; Siti-Sarah Nor'e
EPI_ISL_11939057, EPI_ISL_11939058, EPI_ISL_11939059, EPI_ISL_11939060, EPI_ISL_11939061, EPI_ISL_11939062, EPI_ISL_11939063, EPI_ISL_11939064, EPI_ISL_11939065, EPI_ISL_11939066, EPI_ISL_11939067, EPI_ISL_11939068, EPI_ISL_11939069, EPI_ISL_11939129			
see above	IMR	iPROMISE, UiTM	Ariza Adnan; Fadzilah Mohd Nor; Lim Wai Feng; Mohd Asif Mohd Sukri; Mohd Nur Fakhruzzaman Noorizhab; Mohd Zaki Salleh; Sazzli Shahlan Kassim; Siti Farah Alwani Mohd Nawi; Siti Hamimah Sheikh Abdul Kadir; Teh Lay Kek; Wang Seok Mui
EPI_ISL_11323769, EPI_ISL_11323798	Institut Biologi Molekul Perubatan UKM (UMBI)	UKM Medical Molecular Biology Institute (UMBI)	Khairun Nur Abd Ghafar; Mira Farzana Mohamad Mokhtar; Muhiddin Ishak; Nor Azila Muhammad Azami; Nur Alyaa Afifah Md Shahri; Nurul Syakima Ab Mutalib; Rahman Jamal; Ryia Illani Mohd Yunos; Siti Nur Hasanah Mohd Yusuf; Zahirrah Begam Mohamed Rasheed
EPI_ISL_11899961, EPI_ISL_11900025, EPI_ISL_11900026, EPI_ISL_11900027	Institut Kanser Negara	Malaysia Genome and Vaccine Institute	Azrin Ahmad; Enizza Kasim; Irni Suhayu Sapian; Mohd Faizal Abu Bakar; Mohd Ghows Mohd Azzam.; Mohd Noor Mat Isa; Nor Azfa Johari; Nurhezreen Md Iqbal; Shamsidar Sopie; Siti Noraini Othman; Yusuf Muhammad Noor
EPI_ISL_10810900, EPI_ISL_10810959, EPI_ISL_10969025, EPI_ISL_10969031, EPI_ISL_10969032, EPI_ISL_10969033, EPI_ISL_10969034, EPI_ISL_10969035, EPI_ISL_10969077, EPI_ISL_12534836, EPI_ISL_12534848, EPI_ISL_12534849, EPI_ISL_12534850, EPI_ISL_12534851, EPI_ISL_12534852, EPI_ISL_12534853, EPI_ISL_12534854, EPI_ISL_12534855, EPI_ISL_12534856, EPI_ISL_12534857, EPI_ISL_12534858, EPI_ISL_12534859, EPI_ISL_12534860, EPI_ISL_12534861, EPI_ISL_12534862, EPI_ISL_12534863, EPI_ISL_12534864, EPI_ISL_12534865, EPI_ISL_12534868, EPI_ISL_12534869			
see above	JABATAN PATOLOGI, HOSPITAL RAJA PEREMPUAN ZAINAB II	Tropical Infectious Diseases Research & Education Centre (TIDREC), Universiti Malaya	AsmaAnati CheMatSeri; Che-Norainon Yaacob; Jia-Yi Tan; Jo-Ern Wong; Kim-Kee Tan; Mulya-Mustika-Sari Zulkifli; Noor Syahida Azizan; Nur-Hidayana Mahfodz; Sazaly AbuBakar; Siti-Sarah Nor'e; Wei-Wei Aw
EPI_ISL_11117210, EPI_ISL_11939132, EPI_ISL_12771112, EPI_ISL_12771119, EPI_ISL_12771120, EPI_ISL_12771122, EPI_ISL_12771167			
see above	JKN PAHANG	iPROMISE, UiTM	Ariza Adnan; Fadzilah Mohd Nor; Lim Wai Feng; Mohd Asif Mohd Sukri; Mohd Nur Fakhruzzaman Noorizhab; Mohd Zaki Salleh; Sazzli Shahlan Kassim; Siti Farah Alwani Mohd Nawi; Siti Hamimah Sheikh Abdul Kadir; Teh Lay Kek; Wang Seok Mui
EPI_ISL_11976174, EPI_ISL_11976175	Jabatan Kesihatan Negeri WP Labuan	Malaysia Genome and Vaccine Institute	Azrin Ahmad; Enizza Kasim; Irni Suhayu Sapian; Mohd Faizal Abu Bakar; Mohd Ghows Mohd Azzam.; Mohd Noor Mat Isa; Nor Azfa Johari; Nurhezreen Md Iqbal; Shamsidar Sopie; Siti Noraini Othman; Yusuf Muhammad Noor
EPI_ISL_10297303	Jenjarom Health Clinic	Institute for Medical Research, Infectious Disease Research Centre, National Institutes of Health, Ministry of Health Malaysia	Ahmad FA; Ahmad Fazilah NA; Anasir MI; Azizan MA; Kamel K; Mohamad Sukri MZ; Norhisham SN; Ramly N; Robert F; Rosli NR; Suppiah J; Thayan R
EPI_ISL_10495883	KINABATANGAN AREA HEALTH OFFICE	Institute for Medical Research, Infectious Disease Research Centre, National Institutes of Health, Ministry of Health Malaysia	Ahmad FA; Ahmad Fazilah NA; Anasir MI; Arip MS; Azizan MA; Christopher Hollip K; Jaimin JJ; Kamel K; Mohamad Sukri MZ; Mohammad R; Mohd Yasin R; Norhisham SN; Ramly N; Robert F; Rosli NR; Shamsaddin E; Suppiah J; Tan YC; Thayan R
EPI_ISL_9092714, EPI_ISL_9092715, EPI_ISL_9092716, EPI_ISL_9092717, EPI_ISL_9092718	KL International Healthcare Centre Sdn Bhd	Institute for Medical Research, Infectious Disease Research Centre, National Institutes of Health, Ministry of Health Malaysia	Ahmad FA; Ahmad Fazilah NA; Anasir MI; Azizan MA; Kamel K; Mohamad Sukri MZ; Norhisham SN; Ramly N; Robert F; Rosli NR; Suppiah J; Thayan R
EPI_ISL_11939053	KLINIK IHEALTH	iPROMISE, UiTM	Ariza Adnan; Fadzilah Mohd Nor; Lim Wai Feng; Mohd Asif Mohd Sukri; Mohd Nur Fakhruzzaman Noorizhab; Mohd Zaki Salleh; Sazzli Shahlan Kassim; Siti Farah Alwani Mohd Nawi; Siti Hamimah Sheikh Abdul Kadir; Teh Lay Kek; Wang Seok Mui
EPI_ISL_12534867	KLINIK LUNAR	Tropical Infectious Diseases Research & Education Centre (TIDREC), Universiti Malaya	AsmaAnati CheMatSeri; Che-Norainon Yaacob; Jia-Yi Tan; Jo-Ern Wong; Kim-Kee Tan; Mulya-Mustika-Sari Zulkifli; Noor Syahida Azizan; Nur-Hidayana Mahfodz; Sazaly AbuBakar; Siti-Sarah Nor'e; Wei-Wei Aw
EPI_ISL_11939051	KLINIK MEDILIFE	iPROMISE, UiTM	Ariza Adnan; Fadzilah Mohd Nor; Lim Wai Feng; Mohd Asif Mohd Sukri; Mohd Nur Fakhruzzaman Noorizhab; Mohd Zaki Salleh; Sazzli Shahlan Kassim; Siti Farah Alwani Mohd Nawi; Siti Hamimah Sheikh Abdul Kadir; Teh Lay Kek; Wang Seok Mui
EPI_ISL_11939052	KLINIK MENARA PENANG	iPROMISE, UiTM	Ariza Adnan; Fadzilah Mohd Nor; Lim Wai Feng; Mohd Asif Mohd Sukri; Mohd Nur Fakhruzzaman Noorizhab; Mohd Zaki Salleh; Sazzli Shahlan Kassim; Siti Farah Alwani Mohd Nawi; Siti Hamimah Sheikh Abdul Kadir; Teh Lay Kek; Wang Seok Mui
EPI_ISL_11939050	KLINIK PUTRA	iPROMISE, UiTM	Ariza Adnan; Fadzilah Mohd Nor; Lim Wai Feng; Mohd Asif Mohd Sukri; Mohd Nur Fakhruzzaman Noorizhab; Mohd Zaki Salleh; Sazzli Shahlan Kassim; Siti Farah Alwani Mohd Nawi; Siti Hamimah Sheikh Abdul Kadir; Teh Lay Kek; Wang Seok Mui
EPI_ISL_10495843, EPI_ISL_10495851, EPI_ISL_10495858, EPI_ISL_10495866, EPI_ISL_10495874	KOTA KINABALU AREA HEALTH OFFICE	Institute for Medical Research, Infectious Disease Research Centre, National Institutes of Health, Ministry of Health Malaysia	Ahmad FA; Ahmad Fazilah NA; Anasir MI; Arip MS; Azizan MA; Christopher Hollip K; Jaimin JJ; Kamel K; Mohamad Sukri MZ; Mohammad R; Mohd Yasin R; Norhisham SN; Ramly N; Robert F; Rosli NR; Shamsaddin E; Suppiah J; Tan YC; Thayan R
EPI_ISL_9092747, EPI_ISL_9092748, EPI_ISL_9092749, EPI_ISL_9092750, EPI_ISL_9092751	Kepong District Health Office	Institute for Medical Research, Infectious Disease Research Centre, National Institutes of Health, Ministry of Health Malaysia	Ahmad FA; Ahmad Fazilah NA; Anasir MI; Azizan MA; Kamel K; Mohamad Sukri MZ; Norhisham SN; Ramly N; Robert F; Rosli NR; Suppiah J; Thayan R
EPI_ISL_11332692, EPI_ISL_11332693, EPI_ISL_11332694, EPI_ISL_11332695, EPI_ISL_11332696, EPI_ISL_11332713	Kota Bharu Public Health Laboratory	Tropical Infectious Diseases Research & Education Centre (TIDREC), Universiti Malaya	AsmaAnati CheMatSeri; Che-Norainon Yaacob; Jia-Yi Tan; Jo-Ern Wong; Kim-Kee Tan; Mulya-Mustika-Sari Zulkifli; Noor Syahida Azizan; Nur-Hidayana Mahfodz; Sazaly AbuBakar; Siti-Sarah Nor'e; Wei-Wei Aw
EPI_ISL_10297360	Kota Kinabalu Health Clinic	Institute for Medical Research, Infectious Disease Research Centre, National Institutes of Health, Ministry of Health Malaysia	Ahmad FA; Ahmad Fazilah NA; Anasir MI; Azizan MA; Kamel K; Mohamad Sukri MZ; Norhisham SN; Ramly N; Robert F; Rosli NR; Suppiah J; Thayan R
EPI_ISL_9674538, EPI_ISL_9674577, EPI_ISL_9674661, EPI_ISL_9674903, EPI_ISL_9679117, EPI_ISL_9679118, EPI_ISL_9679208, EPI_ISL_9679214, EPI_ISL_9679215, EPI_ISL_9679216, EPI_ISL_9679219, EPI_ISL_9679220, EPI_ISL_9679221, EPI_ISL_9679222, EPI_ISL_9679224, EPI_ISL_9679235, EPI_ISL_9679239, EPI_ISL_9679241, EPI_ISL_9679243, EPI_ISL_9679711, EPI_ISL_9680333, EPI_ISL_9711771, EPI_ISL_9711772, EPI_ISL_9711773, EPI_ISL_9711774, EPI_ISL_9711775, EPI_ISL_9711776, EPI_ISL_9711777, EPI_ISL_9711779, EPI_ISL_9711780, EPI_ISL_9711781, EPI_ISL_9711782, EPI_ISL_9711783, EPI_ISL_9711784, EPI_ISL_9711785, EPI_ISL_9711786, EPI_ISL_9711787, EPI_ISL_10045141, EPI_ISL_10045142, EPI_ISL_10045143, EPI_ISL_10045144, EPI_ISL_10045145, EPI_ISL_10045146, EPI_ISL_10045147, EPI_ISL_10045148, EPI_ISL_10045149, EPI_ISL_10045150, EPI_ISL_10045151, EPI_ISL_10045152, EPI_ISL_10045153, EPI_ISL_10045154, EPI_ISL_10045155, EPI_ISL_10045156, EPI_ISL_10045160, EPI_ISL_10045161, EPI_ISL_10045162, EPI_ISL_10045163, EPI_ISL_10045164, EPI_ISL_10045165, EPI_ISL_10045168, EPI_ISL_10045169, EPI_ISL_10045170, EPI_ISL_10045171, EPI_ISL_10045172, EPI_ISL_10045173, EPI_ISL_10045174, EPI_ISL_10045176, EPI_ISL_10045177, EPI_ISL_10045178, EPI_ISL_10045179, EPI_ISL_10045180, EPI_ISL_10045181, EPI_ISL_10045182, EPI_ISL_10045183, EPI_ISL_10045184, EPI_ISL_10045186, EPI_ISL_10045187, EPI_ISL_10045188, EPI_ISL_10045189, EPI_ISL_10045190, EPI_ISL_10045191, EPI_ISL_10045192, EPI_ISL_10045193, EPI_ISL_10045194, EPI_ISL_10045195, EPI_ISL_10045196, EPI_ISL_10045202, EPI_ISL_10045203, EPI_ISL_10045204, EPI_ISL_10045205, EPI_ISL_10045206, EPI_ISL_10070316, EPI_ISL_10070317			
see above	Kuala Lumpur International Airport (KLIA) Health Office	Institute for Medical Research, Infectious Disease Research Centre, National Institutes of Health, Ministry of Health Malaysia	Ahmad FA; Ahmad Fazilah NA; Anasir MI; Azizan MA; Kamel K; Mohamad Sukri MZ; Norhisham SN; Ramly N; Robert F; Rosli NR; Suppiah J; Thayan R
EPI_ISL_9092757	Lembah Pantai District Health Office	Institute for Medical Research, Infectious Disease Research Centre, National Institutes of Health, Ministry of Health Malaysia	Ahmad FA; Ahmad Fazilah NA; Anasir MI; Azizan MA; Kamel K; Mohamad Sukri MZ; Norhisham SN; Ramly N; Robert F; Rosli NR; Suppiah J; Thayan R
EPI_ISL_9092771	National Institutes of Health	Institute for Medical Research, Infectious Disease Research Centre, National Institutes of Health, Ministry of Health Malaysia	Ahmad FA; Ahmad Fazilah NA; Anasir MI; Azizan MA; Kamel K; Mohamad Sukri MZ; Norhisham SN; Ramly N; Robert F; Rosli NR; Suppiah J; Thayan R
EPI_ISL_11717938, EPI_ISL_11717939, EPI_ISL_11717940, EPI_ISL_11717941	National Public Health Laboratory	National Public Health Laboratory	Aimi Shazana Binti Shahir; Ishma Hana Binti Isharudin; Kamal Hisham Bin Kamarul Zaman; Kiroshika Pillai A/P Veel Pilay; Muhammad Shahir; Noriah Binti Mohd Yusof; Rehan Shuhada Binti Abu Bakar; Selvanesan A/L Sengol; Ushananthiny A/P Ravi; W.Nur Afiza Binti W. Ariffin; Yu Kie A/P Chem
EPI_ISL_9608483, EPI_ISL_9608484, EPI_ISL_9608485, EPI_ISL_9608511, EPI_ISL_9608515, EPI_ISL_9608516	Normah Medical Specialist Centre, Kuching	Institute of Health and Community Medicine	Chan Chia Jui; Chua Hock Hin; David Perera; Ikwannuddin Y; Ooi Mong How; Tonnii Sia Loong Loong; Wong Jyn Shan
EPI_ISL_10810931, EPI_ISL_10810935	PEJABAT KESIHATAN DAERAH MANJUNG	Tropical Infectious Diseases Research & Education Centre (TIDREC), Universiti Malaya	AsmaAnati CheMatSeri; Che-Norainon Yaacob; Jia-Yi Tan; Jo-Ern Wong; Kim-Kee Tan; Mulya-Mustika-Sari Zulkifli; Noor Syahida Azizan; Nur-Hidayana Mahfodz; Sazaly AbuBakar; Siti-Sarah Nor'e
EPI_ISL_11939091, EPI_ISL_11939092, EPI_ISL_11939095, EPI_ISL_11939098, EPI_ISL_11939101, EPI_ISL_11939104, EPI_ISL_11939105			
see above	PEJABAT KESIHATAN DAERAH SEBERANG PERAI UTARA PULAU PINANG	iPROMISE, UiTM	Ariza Adnan; Fadzilah Mohd Nor; Lim Wai Feng; Mohd Asif Mohd Sukri; Mohd Nur Fakhruzzaman Noorizhab; Mohd Zaki Salleh; Sazzli Shahlan Kassim; Siti Farah Alwani Mohd Nawi; Siti Hamimah Sheikh Abdul Kadir; Teh Lay Kek; Wang Seok Mui
EPI_ISL_11939041, EPI_ISL_11939074, EPI_ISL_11939106, EPI_ISL_11939107, EPI_ISL_11939108, EPI_ISL_11939109, EPI_ISL_11939110, EPI_ISL_11939111, EPI_ISL_11939112, EPI_ISL_12771152			
see above	PKD BARAT DAYA	iPROMISE, UiTM	Ariza Adnan; Fadzilah Mohd Nor; Lim Wai Feng; Mohd Asif Mohd Sukri; Mohd Nur Fakhruzzaman Noorizhab; Mohd Zaki Salleh; Sazzli Shahlan Kassim; Siti Farah Alwani Mohd Nawi; Siti Hamimah Sheikh Abdul Kadir; Teh Lay Kek; Wang Seok Mui
EPI_ISL_10942295, EPI_ISL_10942334	PKD JEMPOL	UKM Medical Molecular Biology Institute (UMBI)	Khairun Nur Abd Ghafar; Mira Farzana Mohamad Mokhtar; Muhiddin Ishak; Nor Azila Muhammad Azami; Nur Alyaa Afifah Md Shahri; Nurul Syakima Ab Mutalib; Rahman Jamal; Ryia Illani Mohd Yunos; Siti Nur Hasanah Mohd Yusuf; Zahirrah Begam Mohamed Rasheed

EPI_ISL_10880082, EPI_ISL_10880108, EPI_ISL_10942280, EPI_ISL_10942286, EPI_ISL_10942338, EPI_ISL_10942343, EPI_ISL_10942348			
see above	PKD JOHOR BAHRU	UKM Medical Molecular Biology Institute (UMBI)	Khairun Nur Abd Ghafar; Mira Farzana Mohamad Mokhtar; Muhiddin Ishak; Nor Azila Muhammad Azami; Nur Alyaa Afifah Md Shahri; Nurul Syakima Ab Mutalib; Rahman Jamal; Ryia Illani Mohd Yunos; Siti Nur Hasanah Mohd Yusuf; Zahirrah Begam Mohamed Rasheed
EPI_ISL_10810932, EPI_ISL_10810933, EPI_ISL_10810934	PKD KERIAN	Tropical Infectious Diseases Research & Education Centre (TIDREC), Universiti Malaya	AsmaAnati CheMatSeri; Che-Norainon Yaacob; Jia-Yi Tan; Jo-Ern Wong; Kim-Kee Tan; Mulya-Mustika-Sari Zulkifli; Noor Syahida Azizan; Nur-Hidayana Mahfodz; Sazaly AbuBakar; Siti-Sarah Nor'e
EPI_ISL_10880068, EPI_ISL_10880098, EPI_ISL_10942302, EPI_ISL_10942332	PKD KLUANG	UKM Medical Molecular Biology Institute (UMBI)	Khairun Nur Abd Ghafar; Mira Farzana Mohamad Mokhtar; Muhiddin Ishak; Nor Azila Muhammad Azami; Nur Alyaa Afifah Md Shahri; Nurul Syakima Ab Mutalib; Rahman Jamal; Ryia Illani Mohd Yunos; Siti Nur Hasanah Mohd Yusuf; Zahirrah Begam Mohamed Rasheed
EPI_ISL_10942314, EPI_ISL_10942347	PKD MELAKA TENGAH	UKM Medical Molecular Biology Institute (UMBI)	Khairun Nur Abd Ghafar; Mira Farzana Mohamad Mokhtar; Muhiddin Ishak; Nor Azila Muhammad Azami; Nur Alyaa Afifah Md Shahri; Nurul Syakima Ab Mutalib; Rahman Jamal; Ryia Illani Mohd Yunos; Siti Nur Hasanah Mohd Yusuf; Zahirrah Begam Mohamed Rasheed
EPI_ISL_12771111	PKD SEBERANG PERAI SELATAN	iPROMISE, UiTM	Ariza Adnan; Fadzilah Mohd Nor; Lim Wai Feng; Mohd Asif Mohd Sukri; Mohd Nur Fakhruzzaman Noorizhab; Mohd Zaki Salleh; Sazzli Shahlan Kassim; Siti Farah Alwani Mohd Nawi; Siti Hamimah Sheikh Abdul Kadir; Teh Lay Kek; Wang Seok Mui
EPI_ISL_12213473, EPI_ISL_12213474, EPI_ISL_12628094, EPI_ISL_12628095, EPI_ISL_12628096, EPI_ISL_12628097	PKD SEREMBAN	UKM Medical Molecular Biology Institute (UMBI)	Khairun Nur Abd Ghafar; Mira Farzana Mohamad Mokhtar; Muhiddin Ishak; Nor Azila Muhammad Azami; Nur Alyaa Afifah Md Shahri; Nurul Syakima Ab Mutalib; Rahman Jamal; Ryia Illani Mohd Yunos; Siti Nur Hasanah Mohd Yusuf; Zahirrah Begam Mohamed Rasheed
EPI_ISL_10942290, EPI_ISL_10942293, EPI_ISL_10942309	PKD TANGKAK	UKM Medical Molecular Biology Institute (UMBI)	Khairun Nur Abd Ghafar; Mira Farzana Mohamad Mokhtar; Muhiddin Ishak; Nor Azila Muhammad Azami; Nur Alyaa Afifah Md Shahri; Nurul Syakima Ab Mutalib; Rahman Jamal; Ryia Illani Mohd Yunos; Siti Nur Hasanah Mohd Yusuf; Zahirrah Begam Mohamed Rasheed
EPI_ISL_11939070, EPI_ISL_11939071, EPI_ISL_11939072, EPI_ISL_11939073, EPI_ISL_11939075, EPI_ISL_11939076, EPI_ISL_11939077, EPI_ISL_11939078, EPI_ISL_11939079, EPI_ISL_11939081, EPI_ISL_11939082, EPI_ISL_11939083, EPI_ISL_11939085			
see above	PKD TIMUR LAUT	iPROMISE, UiTM	Ariza Adnan; Fadzilah Mohd Nor; Lim Wai Feng; Mohd Asif Mohd Sukri; Mohd Nur Fakhruzzaman Noorizhab; Mohd Zaki Salleh; Sazzli Shahlan Kassim; Siti Farah Alwani Mohd Nawi; Siti Hamimah Sheikh Abdul Kadir; Teh Lay Kek; Wang Seok Mui
EPI_ISL_10810938	Pejabat Kesihatan Daerah Hilir Perak	Tropical Infectious Diseases Research & Education Centre (TIDREC), Universiti Malaya	AsmaAnati CheMatSeri; Che-Norainon Yaacob; Jia-Yi Tan; Jo-Ern Wong; Kim-Kee Tan; Mulya-Mustika-Sari Zulkifli; Noor Syahida Azizan; Nur-Hidayana Mahfodz; Sazaly AbuBakar; Siti-Sarah Nor'e
EPI_ISL_11332672	Pejabat Kesihatan Daerah Hulu Perak	Tropical Infectious Diseases Research & Education Centre (TIDREC), Universiti Malaya	AsmaAnati CheMatSeri; Che-Norainon Yaacob; Jia-Yi Tan; Jo-Ern Wong; Kim-Kee Tan; Mulya-Mustika-Sari Zulkifli; Noor Syahida Azizan; Nur-Hidayana Mahfodz; Sazaly AbuBakar; Siti-Sarah Nor'e; Wei-Wei Aw
EPI_ISL_12771171, EPI_ISL_12771172, EPI_ISL_12771173, EPI_ISL_12771174, EPI_ISL_12771175, EPI_ISL_12771176, EPI_ISL_12771177			
see above	Pejabat Kesihatan Pintu Masuk Antarabangsa Pulau Pinang	iPROMISE, UiTM	Ariza Adnan; Fadzilah Mohd Nor; Lim Wai Feng; Mohd Asif Mohd Sukri; Mohd Nur Fakhruzzaman Noorizhab; Mohd Zaki Salleh; Sazzli Shahlan Kassim; Siti Farah Alwani Mohd Nawi; Siti Hamimah Sheikh Abdul Kadir; Teh Lay Kek; Wang Seok Mui
EPI_ISL_9092727	Port Klang Health Clinic	Institute for Medical Research, Infectious Disease Research Centre, National Institutes of Health, Ministry of Health Malaysia	Ahmad FA; Ahmad Fazilah NA; Anasir MI; Azizan MA; Kamel K; Mohamad Sukri MZ; Norhisham SN; Ramly N; Robert F; Rosli NR; Suppiah J; Thayan R
EPI_ISL_10297363, EPI_ISL_10297364, EPI_ISL_10297365, EPI_ISL_10657246	Queen Elizabeth Hospital	Institute for Medical Research, Infectious Disease Research Centre, National Institutes of Health, Ministry of Health Malaysia	Ahmad FA; Ahmad Fazilah NA; Anasir MI; Azizan MA; G. Adypatti NM; Jamaluddin MS; Kamel K; Mohamad Sukri MZ; Norhisham SN; Ramly N; Robert F; Rosli NR; Suppiah J; Thayan R
EPI_ISL_12899672	RAJA PERMAISURI BAINUN HOSPITAL	Institute for Medical Research, Infectious Disease Research Centre, National Institutes of Health, Ministry of Health Malaysia	Ahmad FA; Ahmad Fazilah NA; Anasir MI; Azizan MA; Kamel K; Mohamad Sukri MZ; Norhisham SN; Ramly N; Robert F; Rosli NR; Suppiah J; Thayan R
EPI_ISL_11332652	Raja Permaisuri Bainun Hospital, Ipoh	Tropical Infectious Diseases Research & Education Centre (TIDREC), Universiti Malaya	AsmaAnati CheMatSeri; Che-Norainon Yaacob; Jia-Yi Tan; Jo-Ern Wong; Kim-Kee Tan; Mulya-Mustika-Sari Zulkifli; Noor Syahida Azizan; Nur-Hidayana Mahfodz; Sazaly AbuBakar; Siti-Sarah Nor'e; Wei-Wei Aw
EPI_ISL_8782837, EPI_ISL_9608500, EPI_ISL_9608501, EPI_ISL_9608502	Rejang Medical Centre (Sibu)	Institute of Health and Community Medicine	Chan Chia Jui; Chua Hock Hin; David Perera; Ooi Mong How; Tonnii Sia Loong Loong; Wong Jyn Shan
EPI_ISL_9092730, EPI_ISL_9092731, EPI_ISL_9092732, EPI_ISL_10297284, EPI_ISL_10297295, EPI_ISL_10297296, EPI_ISL_10297297, EPI_ISL_10297298			
see above	Salak Health Clinic	Institute for Medical Research, Infectious Disease Research Centre, National Institutes of Health, Ministry of Health Malaysia	Ahmad FA; Ahmad Fazilah NA; Anasir MI; Azizan MA; Kamel K; Mohamad Sukri MZ; Norhisham SN; Ramly N; Robert F; Rosli NR; Suppiah J; Thayan R
EPI_ISL_8782776, EPI_ISL_8782777, EPI_ISL_8782787, EPI_ISL_8782788, EPI_ISL_8782789, EPI_ISL_8782790, EPI_ISL_8782791, EPI_ISL_8782792, EPI_ISL_8782794, EPI_ISL_8782795, EPI_ISL_8782799, EPI_ISL_8782800, EPI_ISL_8782828, EPI_ISL_9608487, EPI_ISL_9608499, EPI_ISL_9608513			
see above	Sarawak General Hospital (Kuching)	Institute of Health and Community Medicine	Chan Chia Jui; Chua Hock Hin; David Perera; Ooi Mong How; Tonnii Sia Loong Loong
EPI_ISL_10512293	Sarawak Heart Center, Samarahan	Institute of Health and Community Medicine	Chan Chia Jui; Chua Hock Hin; David Perera; Ooi Mong How; Tonnii Sia Loong Loong
EPI_ISL_9092761, EPI_ISL_9092762, EPI_ISL_10297285, EPI_ISL_10297286, EPI_ISL_10297287, EPI_ISL_10297288, EPI_ISL_10297289, EPI_ISL_10297290, EPI_ISL_10297291, EPI_ISL_10297292, EPI_ISL_10297293, EPI_ISL_10297294			
see above	Sepang District Health Office	Institute for Medical Research, Infectious Disease Research Centre, National Institutes of Health, Ministry of Health Malaysia	Ahmad FA; Ahmad Fazilah NA; Anasir MI; Azizan MA; Kamel K; Mohamad Sukri MZ; Norhisham SN; Ramly N; Robert F; Rosli NR; Suppiah J; Thayan R
EPI_ISL_9608497, EPI_ISL_9608523, EPI_ISL_9608533	Sibu Hospital (Sibu, Sarawak)	Institute of Health and Community Medicine	Chan Chia Jui; Chua Hock Hin; David Perera; Ooi Mong How; Tonnii Sia Loong Loong
EPI_ISL_10495844	TAMBUNAN DISTRICT HEALTH OFFICE	Institute for Medical Research, Infectious Disease Research Centre, National Institutes of Health, Ministry of Health Malaysia	Ahmad FA; Ahmad Fazilah NA; Anasir MI; Arip MS; Azizan MA; Christopher Hollip K; Jaimin JJ; Kamel K; Mohamad Sukri MZ; Mohammad R; Mohd Yasin R; Norhisham SN; Ramly N; Robert F; Rosli NR; Shamsaddin E; Suppiah J; Tan YC; Thayan R
EPI_ISL_10495857, EPI_ISL_10495882	TAWAU HOSPITAL	Institute for Medical Research, Infectious Disease Research Centre, National Institutes of Health, Ministry of Health Malaysia	Ahmad FA; Ahmad Fazilah NA; Anasir MI; Arip MS; Azizan MA; Christopher Hollip K; Jaimin JJ; Kamel K; Mohamad Sukri MZ; Mohammad R; Mohd Yasin R; Norhisham SN; Ramly N; Robert F; Rosli NR; Shamsaddin E; Suppiah J; Tan YC; Thayan R
EPI_ISL_10297361	Tawau Hospital	Institute for Medical Research, Infectious Disease Research Centre, National Institutes of Health, Ministry of Health Malaysia	Ahmad FA; Ahmad Fazilah NA; Anasir MI; Azizan MA; Kamel K; Mohamad Sukri MZ; Norhisham SN; Ramly N; Robert F; Rosli NR; Suppiah J; Thayan R
EPI_ISL_10297283, EPI_ISL_10297299, EPI_ISL_10297300, EPI_ISL_10297301, EPI_ISL_10297302	Telok Datok Health Clinic	Institute for Medical Research, Infectious Disease Research Centre, National Institutes of Health, Ministry of Health Malaysia	Ahmad FA; Ahmad Fazilah NA; Anasir MI; Azizan MA; Kamel K; Mohamad Sukri MZ; Norhisham SN; Ramly N; Robert F; Rosli NR; Suppiah J; Thayan R
EPI_ISL_11900055, EPI_ISL_11900056	Thomson Hospital Kota Damansara	Malaysia Genome and Vaccine Institute	Azrin Ahmad; Enizza Kasim; Irni Suhayu Sapian; Mohd Faizal Abu Bakar; Mohd Ghows Mohd Azzam.; Mohd Noor Mat Isa; Nor Azfa Johari; Nurhezreen Md Iqbal; Shamsidar Sopie; Siti Noraini Othman; Yusuf Muhammad Noor
EPI_ISL_9092768, EPI_ISL_9092769, EPI_ISL_9092770	Titiwangsa District Health Office	Institute for Medical Research, Infectious Disease Research Centre, National Institutes of Health, Ministry of Health Malaysia	Ahmad FA; Ahmad Fazilah NA; Anasir MI; Azizan MA; Kamel K; Mohamad Sukri MZ; Norhisham SN; Ramly N; Robert F; Rosli NR; Suppiah J; Thayan R
EPI_ISL_10942312	UNIT KESIHATAN AWAM HEBHK	UKM Medical Molecular Biology Institute (UMBI)	Khairun Nur Abd Ghafar; Mira Farzana Mohamad Mokhtar; Muhiddin Ishak; Nor Azila Muhammad Azami; Nur Alyaa Afifah Md Shahri; Nurul Syakima Ab Mutalib; Rahman Jamal; Ryia Illani Mohd Yunos; Siti Nur Hasanah Mohd Yusuf; Zahirrah Begam Mohamed Rasheed
EPI_ISL_10692496	WARD 5 COVID	UKM Medical Molecular Biology Institute (UMBI)	Khairun Nur Abd Ghafar; Mira Farzana Mohamad Mokhtar; Muhiddin Ishak; Nor Azila Muhammad Azami; Nur Alyaa Afifah Md Shahri; Nurul Syakima Ab Mutalib; Rahman Jamal; Ryia Illani Mohd Yunos; Siti Nur Hasanah Mohd Yusuf; Zahirrah Begam Mohamed Rasheed
EPI_ISL_10692493, EPI_ISL_10692524, EPI_ISL_10692525	WARD 5 COVID SEGAMAT	UKM Medical Molecular Biology Institute (UMBI)	Khairun Nur Abd Ghafar; Mira Farzana Mohamad Mokhtar; Muhiddin Ishak; Nor Azila Muhammad Azami; Nur Alyaa Afifah Md Shahri; Nurul Syakima Ab Mutalib; Rahman Jamal; Ryia Illani Mohd Yunos; Siti Nur Hasanah Mohd Yusuf; Zahirrah Begam Mohamed Rasheed

We gratefully acknowledge the following Authors from the Originating laboratories responsible for obtaining the specimens, as well as the Submitting laboratories where the genome data were generated and shared via GISAID, on which this research is based.

All Submitters of data may be contacted directly via www.gisaid.org

Authors are sorted alphabetically.

Acknowledgement EPI_SET Identifier: EPI_SET_20220603uv

Accession ID	Originating Laboratory	Submitting Laboratory	Authors
EPI_ISL_10692489, EPI_ISL_10692492	Air Molek	UKM Medical Molecular Biology Institute (UMBI)	Khairun Nur Abd Ghafar; Mira Farzana Mohamad Mokhtar; Muhiddin Ishak; Nor Azila Muhammad Azami; Nur Alyaa Afifah Md Shahri; Nurul Syakima Ab Mutalib; Rahman Jamal; Ryia Illani Mohd Yunos; Siti Nur Hasanah Mohd Yusuf; Zahirrah Begam Mohamed Rasheed
EPI_ISL_11900017, EPI_ISL_11900018, EPI_ISL_11900020, EPI_ISL_11900021, EPI_ISL_11900024	BP Clinical Lab Sdn Bhd (KLIA)	Malaysia Genome and Vaccine Institute	Azrin Ahmad; Enizza Kasim; Irni Suhayu Sapian; Mohd Faizal Abu Bakar; Mohd Ghows Mohd Azzam.; Mohd Noor Mat Isa; Nor Azfa Johari; Nurhezreen Md Iqbal; Shamsidar Sopie; Siti Noraini Othman; Yusuf Muhammad Noor
EPI_ISL_10692490	Batu Pahat	UKM Medical Molecular Biology Institute (UMBI)	Khairun Nur Abd Ghafar; Mira Farzana Mohamad Mokhtar; Muhiddin Ishak; Nor Azila Muhammad Azami; Nur Alyaa Afifah Md Shahri; Nurul Syakima Ab Mutalib; Rahman Jamal; Ryia Illani Mohd Yunos; Siti Nur Hasanah Mohd Yusuf; Zahirrah Begam Mohamed Rasheed
EPI_ISL_10692531	Bertam Ulu	UKM Medical Molecular Biology Institute (UMBI)	Khairun Nur Abd Ghafar; Mira Farzana Mohamad Mokhtar; Muhiddin Ishak; Nor Azila Muhammad Azami; Nur Alyaa Afifah Md Shahri; Nurul Syakima Ab Mutalib; Rahman Jamal; Ryia Illani Mohd Yunos; Siti Nur Hasanah Mohd Yusuf; Zahirrah Begam Mohamed Rasheed
EPI_ISL_10297310, EPI_ISL_10297324	Bestari Jaya Health Clinic	Institute for Medical Research, Infectious Disease Research Centre, National Institutes of Health, Ministry of Health Malaysia	Ahmad FA; Ahmad Fazilah NA; Anasir MI; Azizan MA; Kamel K; Mohamad Sukri MZ; Norhisham SN; Ramly N; Robert F; Rosli NR; Suppiah J; Thayan R
EPI_ISL_9608481, EPI_ISL_9608505, EPI_ISL_9608536	Borneo Medical Centre (Kuching)	Institute of Health and Community Medicine	Chan Chia Jui; Chua Hock Hin; David Perera; Ooi Mong How; Tonnii Sia Loong Loong; Wong Jyn Shan
EPI_ISL_10297317, EPI_ISL_10297318	Bukit Changgang Health Clinic	Institute for Medical Research, Infectious Disease Research Centre, National Institutes of Health, Ministry of Health Malaysia	Ahmad FA; Ahmad Fazilah NA; Anasir MI; Azizan MA; Kamel K; Mohamad Sukri MZ; Norhisham SN; Ramly N; Robert F; Rosli NR; Suppiah J; Thayan R
EPI_ISL_10692512	Bukit Rambai	UKM Medical Molecular Biology Institute (UMBI)	Khairun Nur Abd Ghafar; Mira Farzana Mohamad Mokhtar; Muhiddin Ishak; Nor Azila Muhammad Azami; Nur Alyaa Afifah Md Shahri; Nurul Syakima Ab Mutalib; Rahman Jamal; Ryia Illani Mohd Yunos; Siti Nur Hasanah Mohd Yusuf; Zahirrah Begam Mohamed Rasheed
EPI_ISL_11344852, EPI_ISL_11344853, EPI_ISL_11344854, EPI_ISL_11344855, EPI_ISL_11344856, EPI_ISL_11344857, EPI_ISL_11344858, EPI_ISL_11344859			
see above	Department of Medical Microbiology and Parasitology, School of Medical Sciences, Universiti Sains Malaysia Health Campus	Molecular Research Laboratory, Department of Medical Microbiology and Parasitology, School of Medical Sciences, Universiti Sains Malaysia Health Campus	Abdul Haris bin Muhammad; Ahmad Sukari Bin Halim; Alexander Chong Shu Chien; Asraihan Che Abdul Malik; Azian Harun; Chan Yean Yean; Chua Wei Chuan; Farahana binti Mohamed; Kirnpal Kaur Banga Singh; Lau Nyok Sean; Lee Lih Huey; Lim Shu Yong; Maizun binti Mohd Zain; Mera Edora Binti Abdul Manap; Mohd Iman hafiz Bin Ibrahim; Mohd Nadzri Abu Yazid; Mohd Zulkifli Salleh; Muhamad Khairul Amirin Bin Zulkifli; Muhammad Azamuddeen bin Mohammad Nasir; Muhammad Fazli bin Khalid; Muhammad Nashrul Farhan Samsudin; Muhammad Zarul Hanifah Bin Md Zoqratt; Nik Zuraina binti Nik Mohd Noor; Noor Hafizan binti Mat Salleh; Nor Amizara Binti Azami; Nor Suhana Binti Mohd Satar; Nur Syuhada Binti Abdul Rahim; Nur-Leem Binti Murshid; Nurfadhlina Musa; Qasim Ayub; Rosline Hassan; Sadequr Rahman; Siti Nur Aisyah Binti Mohamad Sham; Syahida binti Omar; Wan Mohd Zahiruddin Wan Mohammad; Wardah Yusof; Zaini bin Hussin; Zakuan Zainy Deris
EPI_ISL_12721573, EPI_ISL_12721574, EPI_ISL_12721575	Department of Medical Microbiology, Hospital Pengajar Universiti Putra Malaysia	Department of Medical Microbiology, Hospital Pengajar Universiti Putra Malaysia	Afiqah Adzmi; Amiza Azmi; Azmiza Syawani Jasni; Hui Yee Chee; Leslie Thian Lung Than; Muadz Mohtar; Muhammad Mohd Isa; Narcisse MS Joseph; Niazlin Mohd Taib; Noor Hazirah Noor Azhari; Norlaila; Nur Raihana Ithnin; Nurul Huda Mohamed Rashidi; Nurul Nadiah Ismail; Rosni Ibrahim; Rukman Awang Hamat; Sallehhudin; Siti Norbaya Masri; Siti Zulaikha Zakariah; Syafinaz Amin Nordin; Tengku Zetty Maztura Tengku Jamaluddin; Zamberi Sekawi
EPI_ISL_10942291	E4 GHM	UKM Medical Molecular Biology Institute (UMBI)	Khairun Nur Abd Ghafar; Mira Farzana Mohamad Mokhtar; Muhiddin Ishak; Nor Azila Muhammad Azami; Nur Alyaa Afifah Md Shahri; Nurul Syakima Ab Mutalib; Rahman Jamal; Ryia Illani Mohd Yunos; Siti Nur Hasanah Mohd Yusuf; Zahirrah Begam Mohamed Rasheed
EPI_ISL_9608486, EPI_ISL_9608493, EPI_ISL_9804171	Gribbles Pathology, Kuching	Institute of Health and Community Medicine	Chan Chia Jui; Chua Hock Hin; David Perera; Liew Chin Chin J; Ooi Mong How; Tonnii Sia Loong Loong; Wong Jyn Shan
EPI_ISL_11939113, EPI_ISL_11939116, EPI_ISL_11939117, EPI_ISL_11939118	HOSPITAL PULAU PINANG	iPROMISE, UiTM	Ariza Adnan; Fadzilah Mohd Nor; Lim Wai Feng; Mohd Asif Mohd Sukri; Mohd Nur Fakhruzzaman Noorizhab; Mohd Zaki Salleh; Sazzli Shahlan Kassim; Siti Farah Alwani Mohd Nawi; Siti Hamimah Sheikh Abdul Kadir; Teh Lay Kek; Wang Seok Mui
EPI_ISL_11939087	HOSPITAL SEBERANG JAYA	iPROMISE, UiTM	Ariza Adnan; Fadzilah Mohd Nor; Lim Wai Feng; Mohd Asif Mohd Sukri; Mohd Nur Fakhruzzaman Noorizhab; Mohd Zaki Salleh; Sazzli Shahlan Kassim; Siti Farah Alwani Mohd Nawi; Siti Hamimah Sheikh Abdul Kadir; Teh Lay Kek; Wang Seok Mui
EPI_ISL_10728459	HOSPITAL SULTANAH AMINAH	UKM Medical Molecular Biology Institute (UMBI)	Khairun Nur Abd Ghafar; Mira Farzana Mohamad Mokhtar; Muhiddin Ishak; Nor Azila Muhammad Azami; Nur Alyaa Afifah Md Shahri; Nurul Syakima Ab Mutalib; Rahman Jamal; Ryia Illani Mohd Yunos; Siti Nur Hasanah Mohd Yusuf; Zahirrah Begam Mohamed Rasheed
EPI_ISL_10942320	HOSPITAL SULTANAH AMINAH JOHOR BAHRU	UKM Medical Molecular Biology Institute (UMBI)	Khairun Nur Abd Ghafar; Mira Farzana Mohamad Mokhtar; Muhiddin Ishak; Nor Azila Muhammad Azami; Nur Alyaa Afifah Md Shahri; Nurul Syakima Ab Mutalib; Rahman Jamal; Ryia Illani Mohd Yunos; Siti Nur Hasanah Mohd Yusuf; Zahirrah Begam Mohamed Rasheed
EPI_ISL_10728460, EPI_ISL_10728461	HOSPITAL SULTANAH NORA ISMAIL	UKM Medical Molecular Biology Institute (UMBI)	Khairun Nur Abd Ghafar; Mira Farzana Mohamad Mokhtar; Muhiddin Ishak; Nor Azila Muhammad Azami; Nur Alyaa Afifah Md Shahri; Nurul Syakima Ab Mutalib; Rahman Jamal; Ryia Illani Mohd Yunos; Siti Nur Hasanah Mohd Yusuf; Zahirrah Begam Mohamed Rasheed
EPI_ISL_10810975	HOSPITAL SULTANAH NUR ZAHIRAH	Tropical Infectious Diseases Research & Education Centre (TIDREC), Universiti Malaya	AsmaAnati CheMatSeri; Che-Norainon Yaacob; Jia-Yi Tan; Jo-Ern Wong; Kim-Kee Tan; Mulya-Mustika-Sari Zulkifli; Noor Syahida Azizan; Nur-Hidayana Mahfodz; Sazaly AbuBakar; Siti-Sarah Nor’e
EPI_ISL_12771099	HOSPITAL TUANKU FAUZIAH	iPROMISE, UiTM	Ariza Adnan; Fadzilah Mohd Nor; Lim Wai Feng; Mohd Asif Mohd Sukri; Mohd Nur Fakhruzzaman Noorizhab; Mohd Zaki Salleh; Sazzli Shahlan Kassim; Siti Farah Alwani Mohd Nawi; Siti Hamimah Sheikh Abdul Kadir; Teh Lay Kek; Wang Seok Mui
EPI_ISL_11939130	HPP	iPROMISE, UiTM	Ariza Adnan; Fadzilah Mohd Nor; Lim Wai Feng; Mohd Asif Mohd Sukri; Mohd Nur Fakhruzzaman Noorizhab; Mohd Zaki Salleh; Sazzli Shahlan Kassim; Siti Farah Alwani Mohd Nawi; Siti Hamimah Sheikh Abdul Kadir; Teh Lay Kek; Wang Seok Mui
EPI_ISL_12135871	HRPB	Tropical Infectious Diseases Research & Education Centre (TIDREC), Universiti Malaya	AsmaAnati CheMatSeri; Che-Norainon Yaacob; Jia-Yi Tan; Jo-Ern Wong; Kim-Kee Tan; Mulya-Mustika-Sari Zulkifli; Noor Syahida Azizan; Nur-Hidayana Mahfodz; Sazaly AbuBakar; Siti-Sarah Nor’e; Wei-Wei Aw
EPI_ISL_10942316, EPI_ISL_10942321, EPI_ISL_10942325, EPI_ISL_10942333	HSAJB	UKM Medical Molecular Biology Institute (UMBI)	Khairun Nur Abd Ghafar; Mira Farzana Mohamad Mokhtar; Muhiddin Ishak; Nor Azila Muhammad Azami; Nur Alyaa Afifah Md Shahri; Nurul Syakima Ab Mutalib; Rahman Jamal; Ryia Illani Mohd Yunos; Siti Nur Hasanah Mohd Yusuf; Zahirrah Begam Mohamed Rasheed
EPI_ISL_10810936	HSIP	Tropical Infectious Diseases Research & Education Centre (TIDREC), Universiti Malaya	AsmaAnati CheMatSeri; Che-Norainon Yaacob; Jia-Yi Tan; Jo-Ern Wong; Kim-Kee Tan; Mulya-Mustika-Sari Zulkifli; Noor Syahida Azizan; Nur-Hidayana Mahfodz; Sazaly AbuBakar; Siti-Sarah Nor’e
EPI_ISL_12771113	HTAA ward 8b	iPROMISE, UiTM	Ariza Adnan; Fadzilah Mohd Nor; Lim Wai Feng; Mohd Asif Mohd Sukri; Mohd Nur Fakhruzzaman Noorizhab; Mohd Zaki Salleh; Sazzli Shahlan Kassim; Siti Farah Alwani Mohd Nawi; Siti Hamimah Sheikh Abdul Kadir; Teh Lay Kek; Wang Seok Mui
EPI_ISL_10728456, EPI_ISL_10728457, EPI_ISL_10728462, EPI_ISL_10728463, EPI_ISL_10728464, EPI_ISL_10728492, EPI_ISL_10728500, EPI_ISL_10942294, EPI_ISL_10942313, EPI_ISL_10942319, EPI_ISL_10942330, EPI_ISL_10942344, EPI_ISL_10942352, EPI_ISL_10942354, EPI_ISL_12008501, EPI_ISL_12008506			
see above	HTJS	UKM Medical Molecular Biology Institute (UMBI)	Khairun Nur Abd Ghafar; Mira Farzana Mohamad Mokhtar; Muhiddin Ishak; Nor Azila Muhammad Azami; Nur Alyaa Afifah Md Shahri; Nurul Syakima Ab Mutalib; Rahman Jamal; Ryia Illani Mohd Yunos; Siti Nur Hasanah Mohd Yusuf; Zahirrah Begam Mohamed Rasheed
EPI_ISL_10692520	Hang Tuah Jaya	UKM Medical Molecular Biology Institute (UMBI)	Khairun Nur Abd Ghafar; Mira Farzana Mohamad Mokhtar; Muhiddin Ishak; Nor Azila Muhammad Azami; Nur Alyaa Afifah Md Shahri; Nurul Syakima Ab Mutalib; Rahman Jamal; Ryia Illani Mohd Yunos; Siti Nur Hasanah Mohd Yusuf; Zahirrah Begam Mohamed Rasheed
EPI_ISL_11976173, EPI_ISL_11976182, EPI_ISL_11976198, EPI_ISL_11976199, EPI_ISL_11976202, EPI_ISL_11976203, EPI_ISL_11976207, EPI_ISL_11976208, EPI_ISL_12277130, EPI_ISL_12277131, EPI_ISL_12277132, EPI_ISL_12277133, EPI_ISL_12277134, EPI_ISL_12277135, EPI_ISL_12277136, EPI_ISL_12277137, EPI_ISL_12277138, EPI_ISL_12277139, EPI_ISL_12277140, EPI_ISL_12277141, EPI_ISL_12277142, EPI_ISL_12277143, EPI_ISL_12277144, EPI_ISL_12277145, EPI_ISL_12277146, EPI_ISL_12277148, EPI_ISL_12277149, EPI_ISL_12277154, EPI_ISL_12277155			
see above	Hospital Ampang	Malaysia Genome and Vaccine Institute	Azrin Ahmad; Enizza Kasim; Irni Suhayu Sapian; Mohd Faizal Abu Bakar; Mohd Ghows Mohd Azzam.; Mohd Noor Mat Isa; Nor Azfa Johari; Nurhezreen Md Iqbal; Shamsidar Sopie; Siti Noraini Othman; Yusuf Muhammad Noor
EPI_ISL_11976209, EPI_ISL_11976210, EPI_ISL_11976211, EPI_ISL_11976212, EPI_ISL_11976213, EPI_ISL_11976214, EPI_ISL_11976215, EPI_ISL_11976216, EPI_ISL_11976217, EPI_ISL_11976218, EPI_ISL_12277199, EPI_ISL_12277200, EPI_ISL_12277201, EPI_ISL_12277202, EPI_ISL_12277203, EPI_ISL_12277204, EPI_ISL_12277205			
see above	Hospital Kuala Lumpur	Malaysia Genome and Vaccine Institute	Azrin Ahmad; Enizza Kasim; Irni Suhayu Sapian; Mohd Faizal Abu Bakar; Mohd Ghows Mohd Azzam.; Mohd Noor Mat Isa; Nor Azfa Johari; Nurhezreen Md Iqbal; Shamsidar Sopie; Siti Noraini Othman; Yusuf Muhammad Noor
EPI_ISL_10692518	Hospital Melaka	UKM Medical Molecular Biology Institute (UMBI)	Khairun Nur Abd Ghafar; Mira Farzana Mohamad Mokhtar; Muhiddin Ishak; Nor Azila Muhammad Azami; Nur Alyaa Afifah Md Shahri; Nurul Syakima Ab Mutalib; Rahman Jamal; Ryia Illani Mohd Yunos; Siti Nur Hasanah Mohd Yusuf; Zahirrah Begam Mohamed Rasheed
EPI_ISL_10692495, EPI_ISL_10692498, EPI_ISL_10692504, EPI_ISL_10692506, EPI_ISL_10692507, EPI_ISL_10692522, EPI_ISL_10692528			
see above	Hospital Segamat	UKM Medical Molecular Biology Institute (UMBI)	Khairun Nur Abd Ghafar; Mira Farzana Mohamad Mokhtar; Muhiddin Ishak; Nor Azila Muhammad Azami; Nur Alyaa Afifah Md Shahri; Nurul Syakima Ab Mutalib; Rahman Jamal; Ryia Illani Mohd Yunos; Siti Nur Hasanah Mohd Yusuf; Zahirrah Begam Mohamed Rasheed
EPI_ISL_11900058	Hospital Selayang	Malaysia Genome and Vaccine Institute	Azrin Ahmad; Enizza Kasim; Irni Suhayu Sapian; Mohd Faizal Abu Bakar; Mohd Ghows Mohd Azzam.; Mohd Noor Mat Isa; Nor Azfa Johari; Nurhezreen Md Iqbal; Shamsidar Sopie; Siti Noraini Othman; Yusuf Muhammad Noor
EPI_ISL_11332678	Hospital Sultanah Bahiyah	Tropical Infectious Diseases Research & Education Centre (TIDREC), Universiti Malaya	AsmaAnati CheMatSeri; Che-Norainon Yaacob; Jia-Yi Tan; Jo-Ern Wong; Kim-Kee Tan; Mulya-Mustika-Sari Zulkifli; Noor Syahida Azizan; Nur-Hidayana Mahfodz; Sazaly AbuBakar; Siti-Sarah Nor’e; Wei-Wei Aw
EPI_ISL_11976183, EPI_ISL_11976184, EPI_ISL_11976185, EPI_ISL_11976186, EPI_ISL_11976187, EPI_ISL_11976189, EPI_ISL_11976190, EPI_ISL_11976191, EPI_ISL_11976192, EPI_ISL_11976193, EPI_ISL_11976194, EPI_ISL_11976195, EPI_ISL_11976196, EPI_ISL_11976197, EPI_ISL_11976200, EPI_ISL_11976201, EPI_ISL_11976204, EPI_ISL_11976205, EPI_ISL_11976206, EPI_ISL_12277150,			

EPI_ISL_12277151, EPI_ISL_12277152, EPI_ISL_12277153, EPI_ISL_12277158, EPI_ISL_12277177, EPI_ISL_12277178, EPI_ISL_12277179, EPI_ISL_12277180, EPI_ISL_12277181, EPI_ISL_12277182, EPI_ISL_12277191, EPI_ISL_12277192, EPI_ISL_12277193, EPI_ISL_12277194, EPI_ISL_12277195, EPI_ISL_12277196			
see above	Hospital Sungai Buloh	Malaysia Genome and Vaccine Institute	Azrin Ahmad; Enizza Kasim; Irni Suhayu Sapian; Mohd Faizal Abu Bakar; Mohd Ghows Mohd Azzam.; Mohd Noor Mat Isa; Nor Azfa Johari; Nurhezreen Md Iqbal; Shamsidar Sopie; Siti Noraini Othman; Yusuf Muhammad Noor
EPI_ISL_10880072	Hospital Tuanku Jaafar	UKM Medical Molecular Biology Institute (UMBI)	Khairun Nur Abd Ghafar; Mira Farzana Mohamad Mokhtar; Muhiddin Ishak; Nor Azila Muhammad Azami; Nur Alyaa Afifah Md Shahri; Nurul Syakima Ab Mutalib; Rahman Jamal; Ryia Illani Mohd Yunos; Siti Nur Hasanah Mohd Yusuf; Zahirrah Begam Mohamed Rasheed
EPI_ISL_9579117, EPI_ISL_9579118	Hospital UiTM	iPROMISE, UiTM	Ariza Adnan; Fadzilah Mohd Nor; Lim Wai Feng; Mohd Asif Mohd Sukri; Mohd Nur Fakhruzzaman Noorizhab; Mohd Zaki Salleh; Sazzli Shahlan Kassim; Siti Farah Alwani Mohd Nawi; Siti Hamimah Sheikh Abdul Kadir; Teh Lay Kek; Wang Seok Mui
EPI_ISL_11939114	Hospital pulau pinang	iPROMISE, UiTM	Ariza Adnan; Fadzilah Mohd Nor; Lim Wai Feng; Mohd Asif Mohd Sukri; Mohd Nur Fakhruzzaman Noorizhab; Mohd Zaki Salleh; Sazzli Shahlan Kassim; Siti Farah Alwani Mohd Nawi; Siti Hamimah Sheikh Abdul Kadir; Teh Lay Kek; Wang Seok Mui
EPI_ISL_11323763, EPI_ISL_11323773, EPI_ISL_11323794, EPI_ISL_11323821, EPI_ISL_11323827	Institut Biologi Molekul Perubatan UKM (UMBI)	UKM Medical Molecular Biology Institute (UMBI)	Khairun Nur Abd Ghafar; Mira Farzana Mohamad Mokhtar; Muhiddin Ishak; Nor Azila Muhammad Azami; Nur Alyaa Afifah Md Shahri; Nurul Syakima Ab Mutalib; Rahman Jamal; Ryia Illani Mohd Yunos; Siti Nur Hasanah Mohd Yusuf; Zahirrah Begam Mohamed Rasheed
EPI_ISL_11900057	Institut Kanser Negara	Malaysia Genome and Vaccine Institute	Azrin Ahmad; Enizza Kasim; Irni Suhayu Sapian; Mohd Faizal Abu Bakar; Mohd Ghows Mohd Azzam.; Mohd Noor Mat Isa; Nor Azfa Johari; Nurhezreen Md Iqbal; Shamsidar Sopie; Siti Noraini Othman; Yusuf Muhammad Noor
EPI_ISL_10810918, EPI_ISL_10810919, EPI_ISL_10810920, EPI_ISL_10810921, EPI_ISL_10810922, EPI_ISL_10810923, EPI_ISL_10810924, EPI_ISL_10810925, EPI_ISL_10810926, EPI_ISL_10810927, EPI_ISL_10810928, EPI_ISL_10810929, EPI_ISL_10810930, EPI_ISL_10810966, EPI_ISL_10810967, EPI_ISL_10810968, EPI_ISL_10810969, EPI_ISL_10810970			
see above	JABATAN PATOLOGI, HOSPITAL RAJA PEREMPUAN ZAINAB II	Tropical Infectious Diseases Research & Education Centre (TIDREC), Universiti Malaya	AsmaAnati CheMatSeri; Che-Norainon Yaacob; Jia-Yi Tan; Jo-Ern Wong; Kim-Kee Tan; Mulya-Mustika-Sari Zulkifli; Noor Syahida Azizan; Nur-Hidayana Mahfodz; Sazaly AbuBakar; Siti-Sarah Nor'e
EPI_ISL_11939088, EPI_ISL_11939089, EPI_ISL_11939090, EPI_ISL_11939133, EPI_ISL_11939134, EPI_ISL_11939135, EPI_ISL_11939141, EPI_ISL_11939142, EPI_ISL_12771114, EPI_ISL_12771115, EPI_ISL_12771116, EPI_ISL_12771117, EPI_ISL_12771118, EPI_ISL_12771121, EPI_ISL_12771123, EPI_ISL_12771124, EPI_ISL_12771125, EPI_ISL_12771126, EPI_ISL_12771127, EPI_ISL_12771128, EPI_ISL_12771129, EPI_ISL_12771130, EPI_ISL_12771131, EPI_ISL_12771132, EPI_ISL_12771133, EPI_ISL_12771159, EPI_ISL_12771162, EPI_ISL_12771168, EPI_ISL_12771183, EPI_ISL_12771184, EPI_ISL_12771185, EPI_ISL_12771186, EPI_ISL_12771187, EPI_ISL_12771188, EPI_ISL_12771189, EPI_ISL_12771191, EPI_ISL_12771196, EPI_ISL_12771197, EPI_ISL_12771198			
see above	JKN PAHANG	iPROMISE, UiTM	Ariza Adnan; Fadzilah Mohd Nor; Lim Wai Feng; Mohd Asif Mohd Sukri; Mohd Nur Fakhruzzaman Noorizhab; Mohd Zaki Salleh; Sazzli Shahlan Kassim; Siti Farah Alwani Mohd Nawi; Siti Hamimah Sheikh Abdul Kadir; Teh Lay Kek; Wang Seok Mui
EPI_ISL_10297315, EPI_ISL_10297316, EPI_ISL_10297323	Jenjarom Health Clinic	Institute for Medical Research, Infectious Disease Research Centre, National Institutes of Health, Ministry of Health Malaysia	Ahmad FA; Ahmad Fazilah NA; Anasir MI; Azizan MA; Kamel K; Mohamad Sukri MZ; Norhisham SN; Ramly N; Robert F; Rosli NR; Suppiah J; Thayan R
EPI_ISL_10692494	Johor	UKM Medical Molecular Biology Institute (UMBI)	Khairun Nur Abd Ghafar; Mira Farzana Mohamad Mokhtar; Muhiddin Ishak; Nor Azila Muhammad Azami; Nur Alyaa Afifah Md Shahri; Nurul Syakima Ab Mutalib; Rahman Jamal; Ryia Illani Mohd Yunos; Siti Nur Hasanah Mohd Yusuf; Zahirrah Begam Mohamed Rasheed
EPI_ISL_10692508	Johor Bahru	UKM Medical Molecular Biology Institute (UMBI)	Khairun Nur Abd Ghafar; Mira Farzana Mohamad Mokhtar; Muhiddin Ishak; Nor Azila Muhammad Azami; Nur Alyaa Afifah Md Shahri; Nurul Syakima Ab Mutalib; Rahman Jamal; Ryia Illani Mohd Yunos; Siti Nur Hasanah Mohd Yusuf; Zahirrah Begam Mohamed Rasheed
EPI_ISL_10692510, EPI_ISL_10692516	Kluang	UKM Medical Molecular Biology Institute (UMBI)	Khairun Nur Abd Ghafar; Mira Farzana Mohamad Mokhtar; Muhiddin Ishak; Nor Azila Muhammad Azami; Nur Alyaa Afifah Md Shahri; Nurul Syakima Ab Mutalib; Rahman Jamal; Ryia Illani Mohd Yunos; Siti Nur Hasanah Mohd Yusuf; Zahirrah Begam Mohamed Rasheed
EPI_ISL_11332690, EPI_ISL_11332691	Kota Bharu Public Health Laboratory	Tropical Infectious Diseases Research & Education Centre (TIDREC), Universiti Malaya	AsmaAnati CheMatSeri; Che-Norainon Yaacob; Jia-Yi Tan; Jo-Ern Wong; Kim-Kee Tan; Mulya-Mustika-Sari Zulkifli; Noor Syahida Azizan; Nur-Hidayana Mahfodz; Sazaly AbuBakar; Siti-Sarah Nor'e; Wei-Wei Aw
EPI_ISL_10297325, EPI_ISL_10297326	Kuala Langat District Health Office	Institute for Medical Research, Infectious Disease Research Centre, National Institutes of Health, Ministry of Health Malaysia	Ahmad FA; Ahmad Fazilah NA; Anasir MI; Azizan MA; Kamel K; Mohamad Sukri MZ; Norhisham SN; Ramly N; Robert F; Rosli NR; Suppiah J; Thayan R
EPI_ISL_10297321, EPI_ISL_10297330, EPI_ISL_10297331	Kuala Langat Satellite Prison	Institute for Medical Research, Infectious Disease Research Centre, National Institutes of Health, Ministry of Health Malaysia	Ahmad FA; Ahmad Fazilah NA; Anasir MI; Azizan MA; Kamel K; Mohamad Sukri MZ; Norhisham SN; Ramly N; Robert F; Rosli NR; Suppiah J; Thayan R
EPI_ISL_10045157, EPI_ISL_10045158, EPI_ISL_10045159, EPI_ISL_10045166, EPI_ISL_10045167, EPI_ISL_10045175, EPI_ISL_10045185, EPI_ISL_10045197, EPI_ISL_10045198, EPI_ISL_10045199, EPI_ISL_10045200, EPI_ISL_10045201			
see above	Kuala Lumpur International Airport (KLIA) Health Office	Institute for Medical Research, Infectious Disease Research Centre, National Institutes of Health, Ministry of Health Malaysia	Ahmad FA; Ahmad Fazilah NA; Anasir MI; Azizan MA; Kamel K; Mohamad Sukri MZ; Norhisham SN; Ramly N; Robert F; Rosli NR; Suppiah J; Thayan R
EPI_ISL_10297309, EPI_ISL_10297320, EPI_ISL_10297328	Kuala Selangor Health Clinic	Institute for Medical Research, Infectious Disease Research Centre, National Institutes of Health, Ministry of Health Malaysia	Ahmad FA; Ahmad Fazilah NA; Anasir MI; Azizan MA; Kamel K; Mohamad Sukri MZ; Norhisham SN; Ramly N; Robert F; Rosli NR; Suppiah J; Thayan R
EPI_ISL_10495875	LAHAD DATU HOSPITAL	Institute for Medical Research, Infectious Disease Research Centre, National Institutes of Health, Ministry of Health Malaysia	Ahmad FA; Ahmad Fazilah NA; Anasir MI; Arip MS; Azizan MA; Christopher Hollip K; Jaimin JJ; Kamel K; Mohamad Sukri MZ; Mohammad R; Mohd Yasin R; Norhisham SN; Ramly N; Robert F; Rosli NR; Shamsaddin E; Suppiah J; Tan YC; Thayan R
EPI_ISL_10942281, EPI_ISL_10942301, EPI_ISL_10942335	MAKMAL MOLEKULAR HSAJB	UKM Medical Molecular Biology Institute (UMBI)	Khairun Nur Abd Ghafar; Mira Farzana Mohamad Mokhtar; Muhiddin Ishak; Nor Azila Muhammad Azami; Nur Alyaa Afifah Md Shahri; Nurul Syakima Ab Mutalib; Rahman Jamal; Ryia Illani Mohd Yunos; Siti Nur Hasanah Mohd Yusuf; Zahirrah Begam Mohamed Rasheed
EPI_ISL_12135889	MKA KB	Tropical Infectious Diseases Research & Education Centre (TIDREC), Universiti Malaya	AsmaAnati CheMatSeri; Che-Norainon Yaacob; Jia-Yi Tan; Jo-Ern Wong; Kim-Kee Tan; Mulya-Mustika-Sari Zulkifli; Noor Syahida Azizan; Nur-Hidayana Mahfodz; Sazaly AbuBakar; Siti-Sarah Nor'e; Wei-Wei Aw
EPI_ISL_10657372	Marudu District Health Office	Institute for Medical Research, Infectious Disease Research Centre, National Institutes of Health, Ministry of Health Malaysia	Ahmad FA; Ahmad Fazilah NA; Anasir MI; Azizan MA; G. Adypatti NM; Jamaluddin MS; Kamel K; Mohamad Sukri MZ; Norhisham SN; Ramly N; Robert F; Rosli NR; Suppiah J; Thayan R
EPI_ISL_11939115	Medical Hospital Pulau Pinang	iPROMISE, UiTM	Ariza Adnan; Fadzilah Mohd Nor; Lim Wai Feng; Mohd Asif Mohd Sukri; Mohd Nur Fakhruzzaman Noorizhab; Mohd Zaki Salleh; Sazzli Shahlan Kassim; Siti Farah Alwani Mohd Nawi; Siti Hamimah Sheikh Abdul Kadir; Teh Lay Kek; Wang Seok Mui
EPI_ISL_9608495, EPI_ISL_9608498, EPI_ISL_9608503, EPI_ISL_9608518, EPI_ISL_9608519, EPI_ISL_9608527, EPI_ISL_9804137, EPI_ISL_9804170			
see above	Miri Hospital Molecular Diagnostic Lab, Miri	Institute of Health and Community Medicine	Chan Chia Jui; Chua Hock Hin; David Perera; Ooi Mong How; Tonnii Sia Loong Loong
EPI_ISL_10692501	Muar	UKM Medical Molecular Biology Institute (UMBI)	Khairun Nur Abd Ghafar; Mira Farzana Mohamad Mokhtar; Muhiddin Ishak; Nor Azila Muhammad Azami; Nur Alyaa Afifah Md Shahri; Nurul Syakima Ab Mutalib; Rahman Jamal; Ryia Illani Mohd Yunos; Siti Nur Hasanah Mohd Yusuf; Zahirrah Begam Mohamed Rasheed
EPI_ISL_10494644	National Institutes of Health (NIH)	Institute for Medical Research, Infectious Disease Research Centre, National Institutes of Health, Ministry of Health Malaysia	Ahmad FA; Ahmad Fazilah NA; Anasir MI; Azizan MA; Kamel K; Mohamad Sukri MZ; Norhisham SN; Ramly N; Robert F; Rosli NR; Suppiah J; Thayan R
EPI_ISL_11717942, EPI_ISL_11717943	National Public Health Laboratory	National Public Health Laboratory	Aimi Shazana Binti Shahir; Kamal Hisham Bin Kamarul Zaman; Kiroshika Pillai A/P Veel Pilay; Muhammad Shahir; Noriah Binti Mohd Yusof; Rehan Shuhada Binti Abu Bakar; Selvanesan A/L Sengol; Yu Kie A/P Chem
EPI_ISL_9608524, EPI_ISL_9608525, EPI_ISL_9608526	Normah Medical Specialist Centre, Kuching	Institute of Health and Community Medicine	Chan Chia Jui; Chua Hock Hin; David Perera; Ikwannuddin Y; Ooi Mong How; Tonnii Sia Loong Loong; Wong Jyn Shan
EPI_ISL_11939123, EPI_ISL_11939124, EPI_ISL_11939125, EPI_ISL_11939126, EPI_ISL_11939127, EPI_ISL_11939128	PEJABAT KESIHATAN DAERAH TIMUR LAUT	iPROMISE, UiTM	Ariza Adnan; Fadzilah Mohd Nor; Lim Wai Feng; Mohd Asif Mohd Sukri; Mohd Nur Fakhruzzaman Noorizhab; Mohd Zaki Salleh; Sazzli Shahlan Kassim; Siti Farah Alwani Mohd Nawi; Siti Hamimah Sheikh Abdul Kadir; Teh Lay Kek; Wang Seok Mui
EPI_ISL_10728465, EPI_ISL_10728476, EPI_ISL_10728477, EPI_ISL_10728480, EPI_ISL_10728481	PEJABAT KESIHATAN PONTIAN	UKM Medical Molecular Biology Institute (UMBI)	Khairun Nur Abd Ghafar; Mira Farzana Mohamad Mokhtar; Muhiddin Ishak; Nor Azila Muhammad Azami; Nur Alyaa Afifah Md Shahri; Nurul Syakima Ab Mutalib; Rahman Jamal; Ryia Illani Mohd Yunos; Siti Nur Hasanah Mohd Yusuf; Zahirrah Begam Mohamed Rasheed
EPI_ISL_10549462, EPI_ISL_10549464, EPI_ISL_10549465, EPI_ISL_10549466, EPI_ISL_10549476, EPI_ISL_10728466, EPI_ISL_10728467, EPI_ISL_10728482, EPI_ISL_10728485, EPI_ISL_10728486, EPI_ISL_10728490, EPI_ISL_10728491, EPI_ISL_10728493, EPI_ISL_10728494, EPI_ISL_10728496, EPI_ISL_10728498, EPI_ISL_10880074, EPI_ISL_10880079, EPI_ISL_10880080, EPI_ISL_10880085, EPI_ISL_10880097, EPI_ISL_10880101, EPI_ISL_10880102, EPI_ISL_10880104, EPI_ISL_10880109, EPI_ISL_10880112, EPI_ISL_10942285, EPI_ISL_12008430			
see above	PKD JOHOR BAHRU	UKM Medical Molecular Biology Institute (UMBI)	Khairun Nur Abd Ghafar; Mira Farzana Mohamad Mokhtar; Muhiddin Ishak; Nor Azila Muhammad Azami; Nur Alyaa Afifah Md Shahri; Nurul Syakima Ab Mutalib; Rahman Jamal; Ryia Illani Mohd Yunos; Siti Nur Hasanah Mohd Yusuf; Zahirrah Begam Mohamed Rasheed
EPI_ISL_10692499, EPI_ISL_10692500, EPI_ISL_10692503, EPI_ISL_10692511, EPI_ISL_10692514, EPI_ISL_10692515, EPI_ISL_10692526			
see above	PKD Johor Bahru	UKM Medical Molecular Biology Institute (UMBI)	Khairun Nur Abd Ghafar; Mira Farzana Mohamad Mokhtar; Muhiddin Ishak; Nor Azila Muhammad Azami; Nur Alyaa Afifah Md Shahri; Nurul Syakima Ab Mutalib; Rahman Jamal; Ryia Illani Mohd Yunos; Siti Nur Hasanah Mohd Yusuf; Zahirrah Begam Mohamed Rasheed
EPI_ISL_10728458, EPI_ISL_10728468, EPI_ISL_10728469, EPI_ISL_10728478, EPI_ISL_10880077, EPI_ISL_10880100, EPI_ISL_10880105			
see above	PKD KLUANG	UKM Medical Molecular Biology Institute (UMBI)	Khairun Nur Abd Ghafar; Mira Farzana Mohamad Mokhtar; Muhiddin Ishak; Nor Azila Muhammad Azami; Nur Alyaa Afifah Md Shahri; Nurul Syakima Ab Mutalib; Rahman Jamal; Ryia Illani Mohd Yunos; Siti Nur Hasanah Mohd Yusuf; Zahirrah Begam Mohamed Rasheed
EPI_ISL_10880071, EPI_ISL_10880086, EPI_ISL_10880089, EPI_ISL_10880092,	PKD MELAKA TENGAH	UKM Medical Molecular Biology Institute (UMBI)	Khairun Nur Abd Ghafar; Mira Farzana Mohamad Mokhtar; Muhiddin Ishak; Nor Azila Muhammad Azami; Nur Alyaa Afifah Md Shahri; Nurul Syakima Ab Mutalib; Rahman Jamal; Ryia Illani Mohd Yunos; Siti Nur Hasanah Mohd Yusuf; Zahirrah Begam Mohamed Rasheed

EPI_ISL_10880111			
EPI_ISL_10728470, EPI_ISL_10728471, EPI_ISL_10728472, EPI_ISL_10728479, EPI_ISL_10728484, EPI_ISL_10728495	PKD Mersing	UKM Medical Molecular Biology Institute (UMBI)	Khairun Nur Abd Ghafar; Mira Farzana Mohamad Mokhtar; Muhiddin Ishak; Nor Azila Muhammad Azami; Nur Alyaa Afifah Md Shahri; Nurul Syakima Ab Mutalib; Rahman Jamal; Ryia Illani Mohd Yunos; Siti Nur Hasanah Mohd Yusuf; Zahirrah Begam Mohamed Rasheed
EPI_ISL_10810939, EPI_ISL_10810940, EPI_ISL_10810941	PKD PERAK TENGAH	Tropical Infectious Diseases Research & Education Centre (TIDREC), Universiti Malaya	AsmaAnati CheMatSeri; Che-Norainon Yaacob; Jia-Yi Tan; Jo-Ern Wong; Kim-Kee Tan; Mulya-Mustika-Sari Zulkifli; Noor Syahida Azizan; Nur-Hidayana Mahfodz; Sazaly AbuBakar; Siti-Sarah Nor'e
EPI_ISL_10942299, EPI_ISL_10942346	PKD REMBAU	UKM Medical Molecular Biology Institute (UMBI)	Khairun Nur Abd Ghafar; Mira Farzana Mohamad Mokhtar; Muhiddin Ishak; Nor Azila Muhammad Azami; Nur Alyaa Afifah Md Shahri; Nurul Syakima Ab Mutalib; Rahman Jamal; Ryia Illani Mohd Yunos; Siti Nur Hasanah Mohd Yusuf; Zahirrah Begam Mohamed Rasheed
EPI_ISL_10942284, EPI_ISL_12099540, EPI_ISL_12099544, EPI_ISL_12213475, EPI_ISL_12213476, EPI_ISL_12628090	PKD SEREMBAN	UKM Medical Molecular Biology Institute (UMBI)	Khairun Nur Abd Ghafar; Mira Farzana Mohamad Mokhtar; Muhiddin Ishak; Nor Azila Muhammad Azami; Nur Alyaa Afifah Md Shahri; Nurul Syakima Ab Mutalib; Rahman Jamal; Ryia Illani Mohd Yunos; Siti Nur Hasanah Mohd Yusuf; Zahirrah Begam Mohamed Rasheed
EPI_ISL_10728473, EPI_ISL_10728474, EPI_ISL_10728475, EPI_ISL_10728487, EPI_ISL_10728488, EPI_ISL_10728489, EPI_ISL_10728497, EPI_ISL_10728499			
see above	PKD Seremban	UKM Medical Molecular Biology Institute (UMBI)	Khairun Nur Abd Ghafar; Mira Farzana Mohamad Mokhtar; Muhiddin Ishak; Nor Azila Muhammad Azami; Nur Alyaa Afifah Md Shahri; Nurul Syakima Ab Mutalib; Rahman Jamal; Ryia Illani Mohd Yunos; Siti Nur Hasanah Mohd Yusuf; Zahirrah Begam Mohamed Rasheed
EPI_ISL_9317729, EPI_ISL_9317751, EPI_ISL_10397057	PKD TAMPIN	UKM Medical Molecular Biology Institute (UMBI)	Khairun Nur Abd Ghafar; Mira Farzana Mohamad Mokhtar; Muhiddin Ishak; Nor Azila Muhammad Azami; Nur Alyaa Afifah Md Shahri; Nurul Syakima Ab Mutalib; Rahman Jamal; Ryia Illani Mohd Yunos; Siti Nur Hasanah Mohd Yusuf; Zahirrah Begam Mohamed Rasheed
EPI_ISL_12771100, EPI_ISL_12771101, EPI_ISL_12771102, EPI_ISL_12771103, EPI_ISL_12771104, EPI_ISL_12771105, EPI_ISL_12771106, EPI_ISL_12771107, EPI_ISL_12771108, EPI_ISL_12771109, EPI_ISL_12771110			
see above	PKD TIMUR LAUT	iPROMISE, UiTM	Ariza Adnan; Fadzilah Mohd Nor; Lim Wai Feng; Mohd Asif Mohd Sukri; Mohd Nur Fakhruzzaman Noorizhab; Mohd Zaki Salleh; Sazzli Shahlan Kassim; Siti Farah Alwani Mohd Nawi; Siti Hamimah Sheikh Abdul Kadir; Teh Lay Kek; Wang Seok Mui
EPI_ISL_11939147, EPI_ISL_11939148, EPI_ISL_12771264	PKDSPT	iPROMISE, UiTM	Ariza Adnan; Fadzilah Mohd Nor; Lim Wai Feng; Mohd Asif Mohd Sukri; Mohd Nur Fakhruzzaman Noorizhab; Mohd Zaki Salleh; Sazzli Shahlan Kassim; Siti Farah Alwani Mohd Nawi; Siti Hamimah Sheikh Abdul Kadir; Teh Lay Kek; Wang Seok Mui
EPI_ISL_10810937	Pejabat Kesihatan Daerah Hilir Perak	Tropical Infectious Diseases Research & Education Centre (TIDREC), Universiti Malaya	AsmaAnati CheMatSeri; Che-Norainon Yaacob; Jia-Yi Tan; Jo-Ern Wong; Kim-Kee Tan; Mulya-Mustika-Sari Zulkifli; Noor Syahida Azizan; Nur-Hidayana Mahfodz; Sazaly AbuBakar; Siti-Sarah Nor'e
EPI_ISL_10297311, EPI_ISL_10297312, EPI_ISL_10297327, EPI_ISL_10297329	Port Klang Health Clinic	Institute for Medical Research, Infectious Disease Research Centre, National Institutes of Health, Ministry of Health Malaysia	Ahmad FA; Ahmad Fazilah NA; Anasir MI; Azizan MA; Kamel K; Mohamad Sukri MZ; Norhisham SN; Ramly N; Robert F; Rosli NR; Suppiah J; Thayan R
EPI_ISL_10495859	QUEEN ELIZABETH HOSPITAL	Institute for Medical Research, Infectious Disease Research Centre, National Institutes of Health, Ministry of Health Malaysia	Ahmad FA; Ahmad Fazilah NA; Anasir MI; Arip MS; Azizan MA; Christopher Hollip K; Jaimin JJ; Kamel K; Mohamad Sukri MZ; Mohammad R; Mohd Yasin R; Norhisham SN; Ramly N; Robert F; Rosli NR; Shamsaddin E; Suppiah J; Tan YC; Thayan R
EPI_ISL_10657240, EPI_ISL_10657241, EPI_ISL_10657242, EPI_ISL_10657244, EPI_ISL_10657245, EPI_ISL_10657353, EPI_ISL_10657354, EPI_ISL_10657371			
see above	Queen Elizabeth Hospital	Institute for Medical Research, Infectious Disease Research Centre, National Institutes of Health, Ministry of Health Malaysia	Ahmad FA; Ahmad Fazilah NA; Anasir MI; Azizan MA; G. Adypatti NM; Jamaluddin MS; Kamel K; Mohamad Sukri MZ; Norhisham SN; Ramly N; Robert F; Rosli NR; Suppiah J; Thayan R
EPI_ISL_11332656, EPI_ISL_11332657, EPI_ISL_11332658, EPI_ISL_11332659, EPI_ISL_11332660, EPI_ISL_11332661, EPI_ISL_11332662, EPI_ISL_11332663, EPI_ISL_11332665, EPI_ISL_11332685, EPI_ISL_11332686, EPI_ISL_11332687, EPI_ISL_11332688, EPI_ISL_11332689, EPI_ISL_11332697, EPI_ISL_11332698, EPI_ISL_11332699			
see above	Raja Perempuan Zainab II Hospital, Kota Bharu	Tropical Infectious Diseases Research & Education Centre (TIDREC), Universiti Malaya	AsmaAnati CheMatSeri; Che-Norainon Yaacob; Jia-Yi Tan; Jo-Ern Wong; Kim-Kee Tan; Mulya-Mustika-Sari Zulkifli; Noor Syahida Azizan; Nur-Hidayana Mahfodz; Sazaly AbuBakar; Siti-Sarah Nor'e; Wei-Wei Aw
EPI_ISL_11332653, EPI_ISL_11332654, EPI_ISL_11332655, EPI_ISL_11332679, EPI_ISL_11332680, EPI_ISL_11332681, EPI_ISL_11332682, EPI_ISL_11332702, EPI_ISL_11332703, EPI_ISL_11332704, EPI_ISL_11332705, EPI_ISL_11332706, EPI_ISL_11332707, EPI_ISL_11332708, EPI_ISL_11332709, EPI_ISL_11332710, EPI_ISL_11332711, EPI_ISL_11332712			
see above	Raja Permaisuri Bainun Hospital, Ipoh	Tropical Infectious Diseases Research & Education Centre (TIDREC), Universiti Malaya	AsmaAnati CheMatSeri; Che-Norainon Yaacob; Jia-Yi Tan; Jo-Ern Wong; Kim-Kee Tan; Mulya-Mustika-Sari Zulkifli; Noor Syahida Azizan; Nur-Hidayana Mahfodz; Sazaly AbuBakar; Siti-Sarah Nor'e; Wei-Wei Aw
EPI_ISL_10656906, EPI_ISL_10656908, EPI_ISL_10656909, EPI_ISL_10657237, EPI_ISL_10657238, EPI_ISL_10657239	Ranau Hospital	Institute for Medical Research, Infectious Disease Research Centre, National Institutes of Health, Ministry of Health Malaysia	Ahmad FA; Ahmad Fazilah NA; Anasir MI; Azizan MA; G. Adypatti NM; Jamaluddin MS; Kamel K; Mohamad Sukri MZ; Norhisham SN; Ramly N; Robert F; Rosli NR; Suppiah J; Thayan R
EPI_ISL_9608482, EPI_ISL_9608504, EPI_ISL_9608507	Rejang Medical Centre (Sibu)	Institute of Health and Community Medicine	Chan Chia Jui; Chua Hock Hin; David Perera; Ooi Mong How; Tonnii Sia Loong Loong; Wong Jyn Shan
EPI_ISL_10942351	SWAT TEAM	UKM Medical Molecular Biology Institute (UMBI)	Khairun Nur Abd Ghafar; Mira Farzana Mohamad Mokhtar; Muhiddin Ishak; Nor Azila Muhammad Azami; Nur Alyaa Afifah Md Shahri; Nurul Syakima Ab Mutalib; Rahman Jamal; Ryia Illani Mohd Yunos; Siti Nur Hasanah Mohd Yusuf; Zahirrah Begam Mohamed Rasheed
EPI_ISL_9608489, EPI_ISL_9608491, EPI_ISL_9608494, EPI_ISL_9608529	Sarawak General Hospital (Kuching)	Institute of Health and Community Medicine	Chan Chia Jui; Chua Hock Hin; David Perera; Ooi Mong How; Tonnii Sia Loong Loong
EPI_ISL_10512299	Sarawak Heart Center, Samarahan	Institute of Health and Community Medicine	Chan Chia Jui; Chua Hock Hin; David Perera; Ooi Mong How; Tonnii Sia Loong Loong
EPI_ISL_10692502	Segamat	UKM Medical Molecular Biology Institute (UMBI)	Khairun Nur Abd Ghafar; Mira Farzana Mohamad Mokhtar; Muhiddin Ishak; Nor Azila Muhammad Azami; Nur Alyaa Afifah Md Shahri; Nurul Syakima Ab Mutalib; Rahman Jamal; Ryia Illani Mohd Yunos; Siti Nur Hasanah Mohd Yusuf; Zahirrah Begam Mohamed Rasheed
EPI_ISL_10297307, EPI_ISL_10297308, EPI_ISL_10297313, EPI_ISL_10297314	Sepang District Health Office	Institute for Medical Research, Infectious Disease Research Centre, National Institutes of Health, Ministry of Health Malaysia	Ahmad FA; Ahmad Fazilah NA; Anasir MI; Azizan MA; Kamel K; Mohamad Sukri MZ; Norhisham SN; Ramly N; Robert F; Rosli NR; Suppiah J; Thayan R
EPI_ISL_11332683, EPI_ISL_11332684	Seri Iskandar District Health Office	Tropical Infectious Diseases Research & Education Centre (TIDREC), Universiti Malaya	AsmaAnati CheMatSeri; Che-Norainon Yaacob; Jia-Yi Tan; Jo-Ern Wong; Kim-Kee Tan; Mulya-Mustika-Sari Zulkifli; Noor Syahida Azizan; Nur-Hidayana Mahfodz; Sazaly AbuBakar; Siti-Sarah Nor'e; Wei-Wei Aw
EPI_ISL_10692497	Shah Alam	UKM Medical Molecular Biology Institute (UMBI)	Khairun Nur Abd Ghafar; Mira Farzana Mohamad Mokhtar; Muhiddin Ishak; Nor Azila Muhammad Azami; Nur Alyaa Afifah Md Shahri; Nurul Syakima Ab Mutalib; Rahman Jamal; Ryia Illani Mohd Yunos; Siti Nur Hasanah Mohd Yusuf; Zahirrah Begam Mohamed Rasheed
EPI_ISL_9608496, EPI_ISL_9608520, EPI_ISL_9608521, EPI_ISL_9608522, EPI_ISL_9608530, EPI_ISL_9608531, EPI_ISL_9608532, EPI_ISL_9804138, EPI_ISL_9804139, EPI_ISL_9804172			
see above	Sibu Hospital (Sibu, Sarawak)	Institute of Health and Community Medicine	Chan Chia Jui; Chua Hock Hin; David Perera; Ooi Mong How; Tonnii Sia Loong Loong
EPI_ISL_10297319	Sijangkang Health Clinic	Institute for Medical Research, Infectious Disease Research Centre, National Institutes of Health, Ministry of Health Malaysia	Ahmad FA; Ahmad Fazilah NA; Anasir MI; Azizan MA; Kamel K; Mohamad Sukri MZ; Norhisham SN; Ramly N; Robert F; Rosli NR; Suppiah J; Thayan R
EPI_ISL_10692519	Simpang Renggam	UKM Medical Molecular Biology Institute (UMBI)	Khairun Nur Abd Ghafar; Mira Farzana Mohamad Mokhtar; Muhiddin Ishak; Nor Azila Muhammad Azami; Nur Alyaa Afifah Md Shahri; Nurul Syakima Ab Mutalib; Rahman Jamal; Ryia Illani Mohd Yunos; Siti Nur Hasanah Mohd Yusuf; Zahirrah Begam Mohamed Rasheed
EPI_ISL_10692513	Skudai	UKM Medical Molecular Biology Institute (UMBI)	Khairun Nur Abd Ghafar; Mira Farzana Mohamad Mokhtar; Muhiddin Ishak; Nor Azila Muhammad Azami; Nur Alyaa Afifah Md Shahri; Nurul Syakima Ab Mutalib; Rahman Jamal; Ryia Illani Mohd Yunos; Siti Nur Hasanah Mohd Yusuf; Zahirrah Begam Mohamed Rasheed
EPI_ISL_11332675, EPI_ISL_11332718	Sultanah Nur Zahirah Hospital, Kuala Terengganu	Tropical Infectious Diseases Research & Education Centre (TIDREC), Universiti Malaya	AsmaAnati CheMatSeri; Che-Norainon Yaacob; Jia-Yi Tan; Jo-Ern Wong; Kim-Kee Tan; Mulya-Mustika-Sari Zulkifli; Noor Syahida Azizan; Nur-Hidayana Mahfodz; Sazaly AbuBakar; Siti-Sarah Nor'e; Wei-Wei Aw
EPI_ISL_10495867	TAWAU AREA HEALTH OFFICE	Institute for Medical Research, Infectious Disease Research Centre, National Institutes of Health, Ministry of Health Malaysia	Ahmad FA; Ahmad Fazilah NA; Anasir MI; Arip MS; Azizan MA; Christopher Hollip K; Jaimin JJ; Kamel K; Mohamad Sukri MZ; Mohammad R; Mohd Yasin R; Norhisham SN; Ramly N; Robert F; Rosli NR; Shamsaddin E; Suppiah J; Tan YC; Thayan R
EPI_ISL_10495852	TAWAU HOSPITAL	Institute for Medical Research, Infectious Disease Research Centre, National Institutes of Health, Ministry of Health Malaysia	Ahmad FA; Ahmad Fazilah NA; Anasir MI; Arip MS; Azizan MA; Christopher Hollip K; Jaimin JJ; Kamel K; Mohamad Sukri MZ; Mohammad R; Mohd Yasin R; Norhisham SN; Ramly N; Robert F; Rosli NR; Shamsaddin E; Suppiah J; Tan YC; Thayan R
EPI_ISL_10692527	Taman Desa	UKM Medical Molecular Biology Institute (UMBI)	Khairun Nur Abd Ghafar; Mira Farzana Mohamad Mokhtar; Muhiddin Ishak; Nor Azila Muhammad Azami; Nur Alyaa Afifah Md Shahri; Nurul Syakima Ab Mutalib; Rahman Jamal; Ryia Illani Mohd Yunos; Siti Nur Hasanah Mohd Yusuf; Zahirrah Begam Mohamed Rasheed

EPI_ISL_10297322	Telok Datok Health Clinic	Institute for Medical Research, Infectious Disease Research Centre, National Institutes of Health, Ministry of Health Malaysia	Ahmad FA; Ahmad Fazilah NA; Anasir MI; Azizan MA; Kamel K; Mohamad Sukri MZ; Norhisham SN; Ramly N; Robert F; Rosli NR; Suppiah J; Thayan R
EPI_ISL_10657373	Tuaran District Health Office	Institute for Medical Research, Infectious Disease Research Centre, National Institutes of Health, Ministry of Health Malaysia	Ahmad FA; Ahmad Fazilah NA; Anasir MI; Azizan MA; G. Adypatti NM; Jamaluddin MS; Kamel K; Mohamad Sukri MZ; Norhisham SN; Ramly N; Robert F; Rosli NR; Suppiah J; Thayan R
EPI_ISL_9317730	UMBI	UKM Medical Molecular Biology Institute (UMBI)	Khairun Nur Abd Ghafar; Mira Farzana Mohamad Mokhtar; Muhiddin Ishak; Nor Azila Muhammad Azami; Nur Alyaa Afifah Md Shahri; Nurul Syakima Ab Mutalib; Rahman Jamal; Ryia Illani Mohd Yunos; Siti Nur Hasanah Mohd Yusuf; Zahirrah Begam Mohamed Rasheed
EPI_ISL_10942349	UNIT KESIHATAN AWAM	UKM Medical Molecular Biology Institute (UMBI)	Khairun Nur Abd Ghafar; Mira Farzana Mohamad Mokhtar; Muhiddin Ishak; Nor Azila Muhammad Azami; Nur Alyaa Afifah Md Shahri; Nurul Syakima Ab Mutalib; Rahman Jamal; Ryia Illani Mohd Yunos; Siti Nur Hasanah Mohd Yusuf; Zahirrah Begam Mohamed Rasheed
EPI_ISL_10942324, EPI_ISL_10942326	UNIT KESIHATAN AWAM HEBHK	UKM Medical Molecular Biology Institute (UMBI)	Khairun Nur Abd Ghafar; Mira Farzana Mohamad Mokhtar; Muhiddin Ishak; Nor Azila Muhammad Azami; Nur Alyaa Afifah Md Shahri; Nurul Syakima Ab Mutalib; Rahman Jamal; Ryia Illani Mohd Yunos; Siti Nur Hasanah Mohd Yusuf; Zahirrah Begam Mohamed Rasheed
EPI_ISL_10692523	Ulu Tiram	UKM Medical Molecular Biology Institute (UMBI)	Khairun Nur Abd Ghafar; Mira Farzana Mohamad Mokhtar; Muhiddin Ishak; Nor Azila Muhammad Azami; Nur Alyaa Afifah Md Shahri; Nurul Syakima Ab Mutalib; Rahman Jamal; Ryia Illani Mohd Yunos; Siti Nur Hasanah Mohd Yusuf; Zahirrah Begam Mohamed Rasheed
EPI_ISL_10942305	WARD 8D COVID HTJS	UKM Medical Molecular Biology Institute (UMBI)	Khairun Nur Abd Ghafar; Mira Farzana Mohamad Mokhtar; Muhiddin Ishak; Nor Azila Muhammad Azami; Nur Alyaa Afifah Md Shahri; Nurul Syakima Ab Mutalib; Rahman Jamal; Ryia Illani Mohd Yunos; Siti Nur Hasanah Mohd Yusuf; Zahirrah Begam Mohamed Rasheed
EPI_ISL_10942331	Ward 8D HTJS	UKM Medical Molecular Biology Institute (UMBI)	Khairun Nur Abd Ghafar; Mira Farzana Mohamad Mokhtar; Muhiddin Ishak; Nor Azila Muhammad Azami; Nur Alyaa Afifah Md Shahri; Nurul Syakima Ab Mutalib; Rahman Jamal; Ryia Illani Mohd Yunos; Siti Nur Hasanah Mohd Yusuf; Zahirrah Begam Mohamed Rasheed
EPI_ISL_10942318	Ward 8c HTJ	UKM Medical Molecular Biology Institute (UMBI)	Khairun Nur Abd Ghafar; Mira Farzana Mohamad Mokhtar; Muhiddin Ishak; Nor Azila Muhammad Azami; Nur Alyaa Afifah Md Shahri; Nurul Syakima Ab Mutalib; Rahman Jamal; Ryia Illani Mohd Yunos; Siti Nur Hasanah Mohd Yusuf; Zahirrah Begam Mohamed Rasheed

We gratefully acknowledge the following Authors from the Originating laboratories responsible for obtaining the specimens, as well as the Submitting laboratories where the genome data were generated and shared via GISAID, on which this research is based.

All Submitters of data may be contacted directly via www.gisaid.org

Authors are sorted alphabetically.

Accession ID	Originating Laboratory	Submitting Laboratory	Authors
EPI_ISL_11900014, EPI_ISL_11900015, EPI_ISL_11900016	BP Clinical Lab Sdn Bhd (KLIA)	Malaysia Genome and Vaccine Institute	Azrin Ahmad; Enizza Kasim; Irni Suhayu Sapian; Mohd Faizal Abu Bakar; Mohd Ghows Mohd Azzam.; Mohd Noor Mat Isa; Nor Azfa Johari; Nurhezreen Md Iqbal; Shamsidar Sopie; Siti Noraini Othman; Yusuf Muhammad Noor
EPI_ISL_12771306	BP Lab Penang	iPROMISE, UiTM	Ariza Adnan; Fadzilah Mohd Nor; Lim Wai Feng; Mohd Asif Mohd Sukri; Mohd Nur Fakhruzzaman Noorizhab; Mohd Zaki Salleh; Sazzli Shahlan Kassim; Siti Farah Alwani Mohd Nawi; Siti Hamimah Sheikh Abdul Kadir; Teh Lay Kek; Wang Seok Mui
EPI_ISL_10494670, EPI_ISL_10494689	Bestari Jaya Health Clinic	Institute for Medical Research, Infectious Disease Research Centre, National Institutes of Health, Ministry of Health Malaysia	Ahmad FA; Ahmad Fazilah NA; Anasir MI; Azizan MA; Kamel K; Mohamad Sukri MZ; Norhisham SN; Ramly N; Robert F; Rosli NR; Suppiah J; Thayan R
EPI_ISL_9804129	Bintulu Medical Centre (Bintulu)	Institute of Health and Community Medicine	Chan Chia Jui; Chua Hock Hin; David Perera; Ooi Mong How; Tonnii Sia Loong Loong; Wong Jyn Shan
EPI_ISL_9608506, EPI_ISL_9804110, EPI_ISL_9804111, EPI_ISL_9804112, EPI_ISL_9804113, EPI_ISL_9804147, EPI_ISL_9804148, EPI_ISL_9804149, EPI_ISL_9804158, EPI_ISL_9804166			
see above	Borneo Medical Centre (Kuching)	Institute of Health and Community Medicine	Chan Chia Jui; Chua Hock Hin; David Perera; Ooi Mong How; Tonnii Sia Loong Loong; Wong Jyn Shan
EPI_ISL_9804124, EPI_ISL_9804173, EPI_ISL_10512210	Borneo Medical Centre (Miri)	Institute of Health and Community Medicine	Chan Chia Jui; Chua Hock Hin; David Perera; Ooi Mong How; Tonnii Sia Loong Loong; Wong Jyn Shan
EPI_ISL_10494660, EPI_ISL_10494661, EPI_ISL_10494662, EPI_ISL_10494663, EPI_ISL_10494664, EPI_ISL_10494693, EPI_ISL_10494694, EPI_ISL_10494695			
see above	Bukit Changgang Health Clinic	Institute for Medical Research, Infectious Disease Research Centre, National Institutes of Health, Ministry of Health Malaysia	Ahmad FA; Ahmad Fazilah NA; Anasir MI; Azizan MA; Kamel K; Mohamad Sukri MZ; Norhisham SN; Ramly N; Robert F; Rosli NR; Suppiah J; Thayan R
EPI_ISL_10494646	Cheras District Health Office	Institute for Medical Research, Infectious Disease Research Centre, National Institutes of Health, Ministry of Health Malaysia	Ahmad FA; Ahmad Fazilah NA; Anasir MI; Azizan MA; Kamel K; Mohamad Sukri MZ; Norhisham SN; Ramly N; Robert F; Rosli NR; Suppiah J; Thayan R
EPI_ISL_11344860, EPI_ISL_11344861, EPI_ISL_11344862, EPI_ISL_11344863, EPI_ISL_11344864, EPI_ISL_11344865, EPI_ISL_11344866, EPI_ISL_11344867			
see above	Department of Medical Microbiology and Parasitology, School of Medical Sciences, Universiti Sains Malaysia Health Campus	Molecular Research Laboratory, Department of Medical Microbiology and Parasitology, School of Medical Sciences, Universiti Sains Malaysia Health Campus	Abdul Haris bin Muhammad; Ahmad Sukari Bin Halim; Alexander Chong Shu Chien; Asraihan Che Abdul Malik; Azian Harun; Chan Yean Yean; Chua Wei Chuan; Farahana binti Mohamed; Kirnpal Kaur Banga Singh; Lau Nyok Sean; Lee Lih Huey; Lim Shu Yong; Maizun binti Mohd Zain; Mera Edora Binti Abdul Manap; Mohd Iman hafiz Bin Ibrahim; Mohd Nadzri Abu Yazid; Mohd Zulkifli Salleh; Muhamad Khairul Amirin Bin Zulkifli; Muhammad Azamuddeen bin Mohammad Nasir; Muhammad Fazli bin Khalid; Muhammad Nashrul Farhan Samsudin; Muhammad Zarul Hanifah Bin Md Zoqratt; Nik Zuraina binti Nik Mohd Noor; Noor Hafizan binti Mat Salleh; Nor Amizara Binti Azami; Nor Suhana Binti Mohd Satar; Nur Syuhada Binti Abdul Rahim; Nur-Leem Binti Murshid; Nurfadhlina Musa; Qasim Ayub; Rosline Hassan; Sadequr Rahman; Siti Nur Aisyah Binti Mohamad Sham; Syahida binti Omar; Wan Mohd Zahiruddin Wan Mohammad; Wardah Yusof; Zaini bin Hussin; Zakuan Zainy Deris
EPI_ISL_12721576, EPI_ISL_12721577, EPI_ISL_12721578, EPI_ISL_12721579	Department of Medical Microbiology, Hospital Pengajar Universiti Putra Malaysia	Department of Medical Microbiology, Hospital Pengajar Universiti Putra Malaysia	Afiqah Adzmi; Amiza Azmi; Azmiza Syawani Jasni; Hui Yee Chee; Leslie Thian Lung Than; Muadz Mohtar; Muhammad Mohd Isa; Narcisse MS Joseph; Niazlin Mohd Taib; Noor Hazirah Noor Azhari; Norlaila; Nur Raihana Ithnin; Nurul Huda Mohamed Rashidi; Nurul Nadiah Ismail; Rosni Ibrahim; Rukman Awang Hamat; Sallehhudin; Siti Norbaya Masri; Siti Zulaikha Zakariah; Syafinaz Amin Nordin; Tengku Zetty Maztura Tengku Jamaluddin; Zamberi Sekawi
EPI_ISL_10512205, EPI_ISL_10512206, EPI_ISL_10512208	Gribbles Diagnostic Lab @Columbia Asia Hospital, Miri	Institute of Health and Community Medicine	Affif bin Zainal Abidin; Chan Chia Jui; Chua Hock Hin; David Perera; Ooi Mong How; Tonnii Sia Loong Loong
EPI_ISL_9804126, EPI_ISL_9804127, EPI_ISL_9804135, EPI_ISL_9804152, EPI_ISL_9804153, EPI_ISL_10512172, EPI_ISL_10512173, EPI_ISL_10512174, EPI_ISL_10512175, EPI_ISL_10512176, EPI_ISL_10512177, EPI_ISL_10512178, EPI_ISL_10512179, EPI_ISL_10512180, EPI_ISL_10512181, EPI_ISL_10512190			
see above	Gribbles Pathology, Kuching	Institute of Health and Community Medicine	Chan Chia Jui; Chua Hock Hin; David Perera; Liew Chin Chin J; Ooi Mong How; Tonnii Sia Loong Loong; Wong Jyn Shan
EPI_ISL_12771153, EPI_ISL_12771154, EPI_ISL_12771155, EPI_ISL_12771156, EPI_ISL_12771157	HOSPITAL PULAU PINANG	iPROMISE, UiTM	Ariza Adnan; Fadzilah Mohd Nor; Lim Wai Feng; Mohd Asif Mohd Sukri; Mohd Nur Fakhruzzaman Noorizhab; Mohd Zaki Salleh; Sazzli Shahlan Kassim; Siti Farah Alwani Mohd Nawi; Siti Hamimah Sheikh Abdul Kadir; Teh Lay Kek; Wang Seok Mui
EPI_ISL_10652496	HOSPITAL SULTANAH NUR ZAHIRAH	Department of Medical Microbiology, Faculty of Medicine, University of Malaya; University of Malaya Medical Centre	I-Ching SAM; Jolene Yin Ling FU; Omar Khalilur Rahman; Yoke Fun Chan
EPI_ISL_12135898, EPI_ISL_12135899, EPI_ISL_12135900, EPI_ISL_12135901, EPI_ISL_12135902	HOSPITAL SULTANAH NUR ZAHIRAH	Tropical Infectious Diseases Research & Education Centre (TIDREC), Universiti Malaya	AsmaAnati CheMatSeri; Che-Norainon Yaacob; Jia-Yi Tan; Jo-Ern Wong; Kim-Kee Tan; Mulya-Mustika-Sari Zulkifli; Noor Syahida Azizan; Nur-Hidayana Mahfodz; Sazaly AbuBakar; Siti-Sarah Nor'e; Wei-Wei Aw
EPI_ISL_12534871	HOSPITAL SUTANAH MALIHA	Tropical Infectious Diseases Research & Education Centre (TIDREC), Universiti Malaya	AsmaAnati CheMatSeri; Che-Norainon Yaacob; Jia-Yi Tan; Jo-Ern Wong; Kim-Kee Tan; Mulya-Mustika-Sari Zulkifli; Noor Syahida Azizan; Nur-Hidayana Mahfodz; Sazaly AbuBakar; Siti-Sarah Nor'e; Wei-Wei Aw
EPI_ISL_12771271	HPP	iPROMISE, UiTM	Ariza Adnan; Fadzilah Mohd Nor; Lim Wai Feng; Mohd Asif Mohd Sukri; Mohd Nur Fakhruzzaman Noorizhab; Mohd Zaki Salleh; Sazzli Shahlan Kassim; Siti Farah Alwani Mohd Nawi; Siti Hamimah Sheikh Abdul Kadir; Teh Lay Kek; Wang Seok Mui
EPI_ISL_12135872, EPI_ISL_12135873, EPI_ISL_12135874, EPI_ISL_12135875, EPI_ISL_12135876, EPI_ISL_12135877, EPI_ISL_12135878, EPI_ISL_12135879, EPI_ISL_12135880, EPI_ISL_12135881			
see above	HRPB	Tropical Infectious Diseases Research & Education Centre (TIDREC), Universiti Malaya	AsmaAnati CheMatSeri; Che-Norainon Yaacob; Jia-Yi Tan; Jo-Ern Wong; Kim-Kee Tan; Mulya-Mustika-Sari Zulkifli; Noor Syahida Azizan; Nur-Hidayana Mahfodz; Sazaly AbuBakar; Siti-Sarah Nor'e; Wei-Wei Aw
EPI_ISL_10942282, EPI_ISL_10942304, EPI_ISL_10942353, EPI_ISL_12008429, EPI_ISL_12008432, EPI_ISL_12008433, EPI_ISL_12008446, EPI_ISL_12008447, EPI_ISL_12008494, EPI_ISL_12008505			
see above	HSAJB	UKM Medical Molecular Biology Institute (UMBI)	Khairun Nur Abd Ghafar; Mira Farzana Mohamad Mokhtar; Muhiddin Ishak; Nor Azila Muhammad Azami; Nur Alyaa Afifah Md Shahri; Nurul Syakima Ab Mutalib; Rahman Jamal; Ryia Illani Mohd Yunos; Siti Nur Hasanah Mohd Yusuf; Zahirrah Begam Mohamed Rasheed
EPI_ISL_11939139	HTAA, Kuantan	iPROMISE, UiTM	Ariza Adnan; Fadzilah Mohd Nor; Lim Wai Feng; Mohd Asif Mohd Sukri; Mohd Nur Fakhruzzaman Noorizhab; Mohd Zaki Salleh; Sazzli Shahlan Kassim; Siti Farah Alwani Mohd Nawi; Siti Hamimah Sheikh Abdul Kadir; Teh Lay Kek; Wang Seok Mui
EPI_ISL_10942329, EPI_ISL_10942339, EPI_ISL_12008522	HTJS	UKM Medical Molecular Biology Institute (UMBI)	Khairun Nur Abd Ghafar; Mira Farzana Mohamad Mokhtar; Muhiddin Ishak; Nor Azila Muhammad Azami; Nur Alyaa Afifah Md Shahri; Nurul Syakima Ab Mutalib; Rahman Jamal; Ryia Illani Mohd Yunos; Siti Nur Hasanah Mohd Yusuf; Zahirrah Begam Mohamed Rasheed
EPI_ISL_12277147, EPI_ISL_12277156	Hospital Ampang	Malaysia Genome and Vaccine Institute	Azrin Ahmad; Enizza Kasim; Irni Suhayu Sapian; Mohd Faizal Abu Bakar; Mohd Ghows Mohd Azzam.; Mohd Noor Mat Isa; Nor Azfa Johari; Nurhezreen Md Iqbal; Shamsidar Sopie; Siti Noraini Othman; Yusuf Muhammad Noor
EPI_ISL_11332719, EPI_ISL_11332720	Hospital Angkatan Tentera	Tropical Infectious Diseases Research & Education Centre (TIDREC), Universiti Malaya	AsmaAnati CheMatSeri; Che-Norainon Yaacob; Jia-Yi Tan; Jo-Ern Wong; Kim-Kee Tan; Mulya-Mustika-Sari Zulkifli; Noor Syahida Azizan; Nur-Hidayana Mahfodz; Sazaly AbuBakar; Siti-Sarah Nor'e; Wei-Wei Aw
EPI_ISL_12771151	Hospital Kepala Batas	iPROMISE, UiTM	Ariza Adnan; Fadzilah Mohd Nor; Lim Wai Feng; Mohd Asif Mohd Sukri; Mohd Nur Fakhruzzaman Noorizhab; Mohd Zaki Salleh; Sazzli Shahlan Kassim; Siti Farah Alwani Mohd Nawi; Siti Hamimah Sheikh Abdul Kadir; Teh Lay Kek; Wang Seok Mui
EPI_ISL_11900050, EPI_ISL_11900051, EPI_ISL_11900053	Hospital Selayang	Malaysia Genome and Vaccine Institute	Azrin Ahmad; Enizza Kasim; Irni Suhayu Sapian; Mohd Faizal Abu Bakar; Mohd Ghows Mohd Azzam.; Mohd Noor Mat Isa; Nor Azfa Johari; Nurhezreen Md Iqbal; Shamsidar Sopie; Siti Noraini Othman; Yusuf Muhammad Noor
EPI_ISL_11332721	Hospital Sultanah Bahiyah	Tropical Infectious Diseases Research & Education Centre (TIDREC), Universiti Malaya	AsmaAnati CheMatSeri; Che-Norainon Yaacob; Jia-Yi Tan; Jo-Ern Wong; Kim-Kee Tan; Mulya-Mustika-Sari Zulkifli; Noor Syahida Azizan; Nur-Hidayana Mahfodz; Sazaly AbuBakar; Siti-Sarah Nor'e; Wei-Wei Aw
EPI_ISL_12277157, EPI_ISL_12277183, EPI_ISL_12277184, EPI_ISL_12277185, EPI_ISL_12277186, EPI_ISL_12277187, EPI_ISL_12277188, EPI_ISL_12277189, EPI_ISL_12277190, EPI_ISL_12277197, EPI_ISL_12277198			
see above	Hospital Sungai Buloh	Malaysia Genome and Vaccine Institute	Azrin Ahmad; Enizza Kasim; Irni Suhayu Sapian; Mohd Faizal Abu Bakar; Mohd Ghows Mohd Azzam.; Mohd Noor Mat Isa; Nor Azfa Johari; Nurhezreen Md Iqbal; Shamsidar Sopie; Siti Noraini Othman; Yusuf Muhammad Noor
EPI_ISL_11900052	Hospital selayang	Malaysia Genome and Vaccine Institute	Azrin Ahmad; Enizza Kasim; Irni Suhayu Sapian; Mohd Faizal Abu Bakar; Mohd Ghows Mohd Azzam.; Mohd Noor Mat Isa; Nor Azfa Johari; Nurhezreen Md Iqbal; Shamsidar Sopie; Siti Noraini Othman; Yusuf Muhammad Noor
EPI_ISL_11323777, EPI_ISL_11323784, EPI_ISL_11323812, EPI_ISL_11323816	Institut Biologi Molekul Perubatan UKM (UMBI)	UKM Medical Molecular Biology Institute (UMBI)	Khairun Nur Abd Ghafar; Mira Farzana Mohamad Mokhtar; Muhiddin Ishak; Nor Azila Muhammad Azami; Nur Alyaa Afifah Md Shahri; Nurul Syakima Ab Mutalib; Rahman Jamal; Ryia Illani Mohd Yunos; Siti Nur Hasanah Mohd Yusuf; Zahirrah Begam Mohamed Rasheed
EPI_ISL_11939136, EPI_ISL_11939137, EPI_ISL_11939202, EPI_ISL_12771160, EPI_ISL_12771161, EPI_ISL_12771163, EPI_ISL_12771164, EPI_ISL_12771165, EPI_ISL_12771169, EPI_ISL_12771178, EPI_ISL_12771179, EPI_ISL_12771180, EPI_ISL_12771181, EPI_ISL_12771182, EPI_ISL_12771190, EPI_ISL_12771192, EPI_ISL_12771193, EPI_ISL_12771194, EPI_ISL_12771195			
see above	JKN PAHANG	iPROMISE, UiTM	Ariza Adnan; Fadzilah Mohd Nor; Lim Wai Feng; Mohd Asif Mohd Sukri; Mohd Nur Fakhruzzaman Noorizhab; Mohd Zaki Salleh; Sazzli Shahlan Kassim; Siti Farah Alwani Mohd Nawi; Siti Hamimah Sheikh Abdul Kadir; Teh Lay Kek; Wang Seok Mui
EPI_ISL_10494659, EPI_ISL_10494681, EPI_ISL_10494682, EPI_ISL_10494683, EPI_ISL_10494684, EPI_ISL_10494696, EPI_ISL_10494697, EPI_ISL_10494698			
see above	Jenjarom Health Clinic	Institute for Medical Research, Infectious	Ahmad FA; Ahmad Fazilah NA; Anasir MI; Azizan MA; Kamel K; Mohamad Sukri MZ; Norhisham SN; Ramly N; Robert F; Rosli NR; Suppiah J; Thayan R

		Disease Research Centre, National Institutes of Health, Ministry of Health Malaysia	
EPI_ISL_11965306	Kota Kinabalu Area Health Office	Institute for Medical Research, Infectious Disease Research Centre, National Institutes of Health, Ministry of Health Malaysia	Ahmad FA; Ahmad Fazilah NA; Anasir MI; Azizan MA; Kamel K; Mohamad Sukri MZ; Norhisham SN; Ramly N; Robert F; Rosli NR; Suppiah J; Thayan R
EPI_ISL_10494647, EPI_ISL_10494648, EPI_ISL_10494649, EPI_ISL_10494685	Kuala Langat District Health Office	Institute for Medical Research, Infectious Disease Research Centre, National Institutes of Health, Ministry of Health Malaysia	Ahmad FA; Ahmad Fazilah NA; Anasir MI; Azizan MA; Kamel K; Mohamad Sukri MZ; Norhisham SN; Ramly N; Robert F; Rosli NR; Suppiah J; Thayan R
EPI_ISL_10494650, EPI_ISL_10494651, EPI_ISL_10494652, EPI_ISL_10494653, EPI_ISL_10494654, EPI_ISL_10494655, EPI_ISL_10494656, EPI_ISL_10494699			
see above	Kuala Langat Satellite Prison	Institute for Medical Research, Infectious Disease Research Centre, National Institutes of Health, Ministry of Health Malaysia	Ahmad FA; Ahmad Fazilah NA; Anasir MI; Azizan MA; Kamel K; Mohamad Sukri MZ; Norhisham SN; Ramly N; Robert F; Rosli NR; Suppiah J; Thayan R
EPI_ISL_10494736, EPI_ISL_10494737, EPI_ISL_10494738, EPI_ISL_10494739, EPI_ISL_10494740, EPI_ISL_10494741, EPI_ISL_10494742, EPI_ISL_10494743, EPI_ISL_10494744, EPI_ISL_10494745, EPI_ISL_10494746, EPI_ISL_10494747, EPI_ISL_10494748, EPI_ISL_10494749, EPI_ISL_10494750, EPI_ISL_10494751, EPI_ISL_10494752, EPI_ISL_10494753, EPI_ISL_10494754, EPI_ISL_10494755, EPI_ISL_10494756, EPI_ISL_10494757, EPI_ISL_10494758, EPI_ISL_10494759, EPI_ISL_10494760, EPI_ISL_10494761, EPI_ISL_10494762, EPI_ISL_10494763, EPI_ISL_10494764, EPI_ISL_10494765, EPI_ISL_10494766, EPI_ISL_10494767, EPI_ISL_10494768, EPI_ISL_10494769, EPI_ISL_10494770, EPI_ISL_10494771, EPI_ISL_10494772, EPI_ISL_10494773, EPI_ISL_10494774, EPI_ISL_10494775, EPI_ISL_10494776, EPI_ISL_10494777, EPI_ISL_10494778, EPI_ISL_10494779, EPI_ISL_10494780, EPI_ISL_10494781, EPI_ISL_10494782, EPI_ISL_10494783, EPI_ISL_10494784, EPI_ISL_10494785, EPI_ISL_10494786, EPI_ISL_10494787, EPI_ISL_10494788, EPI_ISL_10494789, EPI_ISL_10494790, EPI_ISL_10494791, EPI_ISL_10494792, EPI_ISL_10494793, EPI_ISL_10494794, EPI_ISL_10494795, EPI_ISL_10494796, EPI_ISL_10494797, EPI_ISL_10494799, EPI_ISL_10494800, EPI_ISL_10494801, EPI_ISL_10494802, EPI_ISL_10494803, EPI_ISL_10494804, EPI_ISL_10494805, EPI_ISL_10494806, EPI_ISL_10494807, EPI_ISL_10494808, EPI_ISL_10494809, EPI_ISL_10494810, EPI_ISL_10494811, EPI_ISL_10494812, EPI_ISL_10494813, EPI_ISL_10494814, EPI_ISL_10494815, EPI_ISL_10494816, EPI_ISL_10494817, EPI_ISL_10494818, EPI_ISL_11149357, EPI_ISL_11149358, EPI_ISL_11149359, EPI_ISL_11149360, EPI_ISL_11149361, EPI_ISL_11149362, EPI_ISL_11149363, EPI_ISL_11149364, EPI_ISL_11149365, EPI_ISL_11149366			
see above	Kuala Lumpur International Airport (KLIA) Health Office	Institute for Medical Research, Infectious Disease Research Centre, National Institutes of Health, Ministry of Health Malaysia	Ahmad FA; Ahmad Fazilah NA; Anasir MI; Azizan MA; Kamel K; Mohamad Sukri MZ; Norhisham SN; Ramly N; Robert F; Rosli NR; Suppiah J; Thayan R
EPI_ISL_10494669	Kuala Selangor Health Clinic	Institute for Medical Research, Infectious Disease Research Centre, National Institutes of Health, Ministry of Health Malaysia	Ahmad FA; Ahmad Fazilah NA; Anasir MI; Azizan MA; Kamel K; Mohamad Sukri MZ; Norhisham SN; Ramly N; Robert F; Rosli NR; Suppiah J; Thayan R
EPI_ISL_11754732, EPI_ISL_11754733, EPI_ISL_11754734	MKA KB	Department of Medical Microbiology, Faculty of Medicine, University of Malaya; University of Malaya Medical Centre	I-Ching SAM; Jolene Yin Ling FU; Omar Khalilur Rahman; Yoke Fun Chan
EPI_ISL_12135891, EPI_ISL_12135892, EPI_ISL_12135893, EPI_ISL_12135894, EPI_ISL_12135895, EPI_ISL_12135896, EPI_ISL_12135897			
see above	MKA KB	Tropical Infectious Diseases Research & Education Centre (TIDREC), Universiti Malaya	AsmaAnati CheMatSeri; Che-Norainon Yaacob; Jia-Yi Tan; Jo-Ern Wong; Kim-Kee Tan; Mulya-Mustika-Sari Zulkifli; Noor Syahida Azizan; Nur-Hidayana Mahfodz; Sazaly AbuBakar; Siti-Sarah Nor'e; Wei-Wei Aw
EPI_ISL_11939185, EPI_ISL_12771149	Medical Hospital Pulau Pinang	iPROMISE, UiTM	Ariza Adnan; Fadzilah Mohd Nor; Lim Wai Feng; Mohd Asif Mohd Sukri; Mohd Nur Fakhruzzaman Noorizhab; Mohd Zaki Salleh; Sazzli Shahlan Kassim; Siti Farah Alwani Mohd Nawi; Siti Hamimah Sheikh Abdul Kadir; Teh Lay Kek; Wang Seok Mui
EPI_ISL_9804128, EPI_ISL_9804154, EPI_ISL_9804164, EPI_ISL_9804165, EPI_ISL_9804169	Miri Hospital Molecular Diagnostic Lab, Miri	Institute of Health and Community Medicine	Chan Chia Jui; Chua Hock Hin; David Perera; Ooi Mong How; Tonnii Sia Loong Loong
EPI_ISL_10494645	National Institutes of Health (NIH)	Institute for Medical Research, Infectious Disease Research Centre, National Institutes of Health, Ministry of Health Malaysia	Ahmad FA; Ahmad Fazilah NA; Anasir MI; Azizan MA; Kamel K; Mohamad Sukri MZ; Norhisham SN; Ramly N; Robert F; Rosli NR; Suppiah J; Thayan R
EPI_ISL_11717944, EPI_ISL_11717945, EPI_ISL_11717946, EPI_ISL_11717947, EPI_ISL_11717948, EPI_ISL_11717949, EPI_ISL_11717950, EPI_ISL_11717951, EPI_ISL_11717952, EPI_ISL_11717953, EPI_ISL_11717954			
see above	National Public Health Laboratory	National Public Health Laboratory	Aimi Shazana Binti Shahir; Kamal Hisham Bin Kamarul Zaman; Kiroshika Pillai A/P Veel Pilay; Muhammad Shahir; Noriah Binti Mohd Yusof; Rehan Shuhada Binti Abu Bakar; Selvanesan A/L Sengol; Yu Kie A/P Chem
EPI_ISL_9804115, EPI_ISL_9804116, EPI_ISL_9804118, EPI_ISL_9804119, EPI_ISL_9804120, EPI_ISL_9804121, EPI_ISL_9804122, EPI_ISL_9804123, EPI_ISL_9804151, EPI_ISL_9804155, EPI_ISL_9804156, EPI_ISL_9804157, EPI_ISL_9804160, EPI_ISL_9804162, EPI_ISL_9804163, EPI_ISL_10512211, EPI_ISL_10512212, EPI_ISL_10512213			
see above	Normah Medical Specialist Centre, Kuching	Institute of Health and Community Medicine	Chan Chia Jui; Chua Hock Hin; David Perera; Ikwannuddin Y; Ooi Mong How; Tonnii Sia Loong Loong
EPI_ISL_11939120, EPI_ISL_11939121	PEJABAT KESIHATAN PINTU MASUK ANTARABANGSA PULAU PINANG	iPROMISE, UiTM	Ariza Adnan; Fadzilah Mohd Nor; Lim Wai Feng; Mohd Asif Mohd Sukri; Mohd Nur Fakhruzzaman Noorizhab; Mohd Zaki Salleh; Sazzli Shahlan Kassim; Siti Farah Alwani Mohd Nawi; Siti Hamimah Sheikh Abdul Kadir; Teh Lay Kek; Wang Seok Mui
EPI_ISL_11754745	PKD GUA MUSANG	Department of Medical Microbiology, Faculty of Medicine, University of Malaya; University of Malaya Medical Centre	I-Ching SAM; Jolene Yin Ling FU; Omar Khalilur Rahman; Yoke Fun Chan
EPI_ISL_10880070	PKD JASIN	UKM Medical Molecular Biology Institute (UMBI)	Khairun Nur Abd Ghafar; Mira Farzana Mohamad Mokhtar; Muhiddin Ishak; Nor Azila Muhammad Azami; Nur Alyaa Afifah Md Shahri; Nurul Syakima Ab Mutalib; Rahman Jamal; Ryia Illani Mohd Yunos; Siti Nur Hasanah Mohd Yusuf; Zahirrah Begam Mohamed Rasheed
EPI_ISL_12008488, EPI_ISL_12008491, EPI_ISL_12008511	PKD JEMPOL	UKM Medical Molecular Biology Institute (UMBI)	Khairun Nur Abd Ghafar; Mira Farzana Mohamad Mokhtar; Muhiddin Ishak; Nor Azila Muhammad Azami; Nur Alyaa Afifah Md Shahri; Nurul Syakima Ab Mutalib; Rahman Jamal; Ryia Illani Mohd Yunos; Siti Nur Hasanah Mohd Yusuf; Zahirrah Begam Mohamed Rasheed
EPI_ISL_10880081, EPI_ISL_10880087, EPI_ISL_10880088, EPI_ISL_10880091, EPI_ISL_10880103, EPI_ISL_12008521, EPI_ISL_12008525			
see above	PKD JOHOR BAHRU	UKM Medical Molecular Biology Institute (UMBI)	Khairun Nur Abd Ghafar; Mira Farzana Mohamad Mokhtar; Muhiddin Ishak; Nor Azila Muhammad Azami; Nur Alyaa Afifah Md Shahri; Nurul Syakima Ab Mutalib; Rahman Jamal; Ryia Illani Mohd Yunos; Siti Nur Hasanah Mohd Yusuf; Zahirrah Begam Mohamed Rasheed
EPI_ISL_12135978	PKD KAMPAR	Tropical Infectious Diseases Research & Education Centre (TIDREC), Universiti Malaya	AsmaAnati CheMatSeri; Che-Norainon Yaacob; Jia-Yi Tan; Jo-Ern Wong; Kim-Kee Tan; Mulya-Mustika-Sari Zulkifli; Noor Syahida Azizan; Nur-Hidayana Mahfodz; Sazaly AbuBakar; Siti-Sarah Nor'e; Wei-Wei Aw
EPI_ISL_10880095	PKD MELAKA TENGAH	UKM Medical Molecular Biology Institute (UMBI)	Khairun Nur Abd Ghafar; Mira Farzana Mohamad Mokhtar; Muhiddin Ishak; Nor Azila Muhammad Azami; Nur Alyaa Afifah Md Shahri; Nurul Syakima Ab Mutalib; Rahman Jamal; Ryia Illani Mohd Yunos; Siti Nur Hasanah Mohd Yusuf; Zahirrah Begam Mohamed Rasheed
EPI_ISL_10880090, EPI_ISL_10880113	PKD TAMPIN	UKM Medical Molecular Biology Institute (UMBI)	Khairun Nur Abd Ghafar; Mira Farzana Mohamad Mokhtar; Muhiddin Ishak; Nor Azila Muhammad Azami; Nur Alyaa Afifah Md Shahri; Nurul Syakima Ab Mutalib; Rahman Jamal; Ryia Illani Mohd Yunos; Siti Nur Hasanah Mohd Yusuf; Zahirrah Begam Mohamed Rasheed
EPI_ISL_10880076, EPI_ISL_10880084, EPI_ISL_10880099, EPI_ISL_12008439	PKD TANGKAK	UKM Medical Molecular Biology Institute (UMBI)	Khairun Nur Abd Ghafar; Mira Farzana Mohamad Mokhtar; Muhiddin Ishak; Nor Azila Muhammad Azami; Nur Alyaa Afifah Md Shahri; Nurul Syakima Ab Mutalib; Rahman Jamal; Ryia Illani Mohd Yunos; Siti Nur Hasanah Mohd Yusuf; Zahirrah Begam Mohamed Rasheed
EPI_ISL_12771134, EPI_ISL_12771135, EPI_ISL_12771136, EPI_ISL_12771137, EPI_ISL_12771138, EPI_ISL_12771139, EPI_ISL_12771140, EPI_ISL_12771141, EPI_ISL_12771142, EPI_ISL_12771143, EPI_ISL_12771144, EPI_ISL_12771145, EPI_ISL_12771146, EPI_ISL_12771147, EPI_ISL_12771148, EPI_ISL_12771199, EPI_ISL_12771200			
see above	PKD TIMUR LAUT	iPROMISE, UiTM	Ariza Adnan; Fadzilah Mohd Nor; Lim Wai Feng; Mohd Asif Mohd Sukri; Mohd Nur Fakhruzzaman Noorizhab; Mohd Zaki Salleh; Sazzli Shahlan Kassim; Siti Farah Alwani Mohd Nawi; Siti Hamimah Sheikh Abdul Kadir; Teh Lay Kek; Wang Seok Mui
EPI_ISL_12771267	PKDSPT	iPROMISE, UiTM	Ariza Adnan; Fadzilah Mohd Nor; Lim Wai Feng; Mohd Asif Mohd Sukri; Mohd Nur Fakhruzzaman Noorizhab; Mohd Zaki Salleh; Sazzli Shahlan Kassim; Siti Farah Alwani Mohd Nawi; Siti Hamimah Sheikh Abdul Kadir; Teh Lay Kek; Wang Seok Mui
EPI_ISL_12899688	PUTRAJAYA PUBLIC HEALTH UNIT	Institute for Medical Research, Infectious Disease Research Centre, National Institutes of Health, Ministry of Health Malaysia	Ahmad FA; Ahmad Fazilah NA; Anasir MI; Azizan MA; Kamel K; Mohamad Sukri MZ; Norhisham SN; Ramly N; Robert F; Rosli NR; Suppiah J; Thayan R
EPI_ISL_11939145	Pejabat Kesihatan Daerah Hulu Perak	iPROMISE, UiTM	Ariza Adnan; Fadzilah Mohd Nor; Lim Wai Feng; Mohd Asif Mohd Sukri; Mohd Nur Fakhruzzaman Noorizhab; Mohd Zaki Salleh; Sazzli Shahlan Kassim; Siti Farah Alwani Mohd Nawi; Siti Hamimah Sheikh Abdul Kadir; Teh Lay Kek; Wang Seok Mui
EPI_ISL_10657479	Pejabat Kesihatan Daerah Pitas	Institute for Medical Research, Infectious Disease Research Centre, National Institutes of Health, Ministry of Health Malaysia	Ahmad FA; Ahmad Fazilah NA; Anasir MI; Azizan MA; G. Adypatti NM; Jamaluddin MS; Kamel K; Mohamad Sukri MZ; Norhisham SN; Ramly N; Robert F; Rosli NR; Suppiah J; Thayan R
EPI_ISL_10657477, EPI_ISL_10657478	Pejabat Kesihatan Kawasan Kota Kinabalu	Institute for Medical Research, Infectious Disease Research Centre, National Institutes of Health, Ministry of Health Malaysia	Ahmad FA; Ahmad Fazilah NA; Anasir MI; Azizan MA; G. Adypatti NM; Jamaluddin MS; Kamel K; Mohamad Sukri MZ; Norhisham SN; Ramly N; Robert F; Rosli NR; Suppiah J; Thayan R
EPI_ISL_10657480	Pejabat Kesihatan Kawasan Kota Kinabalu	Institute for Medical Research, Infectious Disease Research Centre, National Institutes of Health, Ministry of Health Malaysia	Ahmad FA; Ahmad Fazilah NA; Anasir MI; Azizan MA; G. Adypatti NM; Jamaluddin MS; Kamel K; Mohamad Sukri MZ; Norhisham SN; Ramly N; Robert F; Rosli NR; Suppiah J; Thayan R
EPI_ISL_11939122, EPI_ISL_12771170	Pejabat Kesihatan Pintu Masuk Antarabangsa Pulau Pinang	iPROMISE, UiTM	Ariza Adnan; Fadzilah Mohd Nor; Lim Wai Feng; Mohd Asif Mohd Sukri; Mohd Nur Fakhruzzaman Noorizhab; Mohd Zaki Salleh; Sazzli Shahlan Kassim; Siti Farah Alwani Mohd Nawi; Siti Hamimah Sheikh Abdul Kadir; Teh Lay Kek; Wang Seok Mui
EPI_ISL_12704549, EPI_ISL_12704550	Pengkalan Chepa Health Clinic	Molecular Research Laboratory	Abdul Haris bin Muhammad; Ahmad Sukari Bin Halim; Alexander Chong Shu Chien; Asraihan Che Abdul Malik; Azian Harun; Chan Yean Yean; Chua Wei Chuan; Farahana binti Mohamed; Kirnpal Kaur Banga Singh; Lau Nyok Sean; Lee Lih Huey; Lim Shu Yong; Maizun binti Mohd Zain; Mera Edora Binti Abdul Manap; Mohd Iman hafiz Bin Ibrahim; Mohd Nadzri Abu Yazid; Mohd Zulkifli Salleh; Muhamad Khairul Amirin Bin Zulkifli; Muhammad Azamuddeen bin Mohammad Nasir; Muhammad Fazli bin Khalid; Muhammad Nashrul Farhan Samsudin; Muhammad Zarul Hanifah Bin Md Zoqratt; Nik Zuraina binti Nik Mohd Noor; Noor Hafizan binti Mat Salleh; Nor Amizara Binti Azami; Nor Suhana Binti Mohd Satar; Nur Syuhada Binti Abdul Rahim; Nur-Leem Binti Murshid; Nurfadhlina Musa; Qasim Ayub; Rosline Hassan; Sadequr Rahman; Siti Nur Aisyah Binti Mohamad Sham; Syahida binti Omar; Wan Mohd Zahiruddin Wan Mohammad; Wardah Yusof; Zaini bin Hussin; Zakuan Zainy Deris
EPI_ISL_10494657, EPI_ISL_10494658, EPI_ISL_10494665, EPI_ISL_10494666, EPI_ISL_10494667, EPI_ISL_10494668, EPI_ISL_10494686, EPI_ISL_10494687, EPI_ISL_10494688			
see above	Port Klang Health Clinic	Institute for Medical Research, Infectious	Ahmad FA; Ahmad Fazilah NA; Anasir MI; Azizan MA; Kamel K; Mohamad Sukri MZ; Norhisham SN; Ramly N; Robert F; Rosli NR; Suppiah J; Thayan R

		Disease Research Centre, National Institutes of Health, Ministry of Health Malaysia	
EPI_ISL_10981868	Putrajaya Health Office	Institute for Medical Research, Infectious Disease Research Centre, National Institutes of Health, Ministry of Health Malaysia	Ahmad FA; Ahmad Fazilah NA; Anasir MI; Azizan MA; Kamel K; Mohamad Sukri MZ; Norhisham SN; Ramly N; Robert F; Rosli NR; Suppiah J; Thayan R
EPI_ISL_10656809, EPI_ISL_10656905, EPI_ISL_10656907, EPI_ISL_10657243, EPI_ISL_10657471, EPI_ISL_10657472, EPI_ISL_10657473, EPI_ISL_10657475, EPI_ISL_10657476			
see above	Queen Elizabeth Hospital	Institute for Medical Research, Infectious Disease Research Centre, National Institutes of Health, Ministry of Health Malaysia	Ahmad FA; Ahmad Fazilah NA; Anasir MI; Azizan MA; G. Adypatti NM; Jamaluddin MS; Kamel K; Mohamad Sukri MZ; Norhisham SN; Ramly N; Robert F; Rosli NR; Suppiah J; Thayan R
EPI_ISL_11332664, EPI_ISL_11332666, EPI_ISL_11332667, EPI_ISL_11332668, EPI_ISL_11332669, EPI_ISL_11332670, EPI_ISL_11332671, EPI_ISL_11332673, EPI_ISL_11332674, EPI_ISL_11332676, EPI_ISL_11332700, EPI_ISL_11332701, EPI_ISL_11332715, EPI_ISL_11332716, EPI_ISL_11332726			
see above	Raja Perempuan Zainab II Hospital, Kota Bharu	Tropical Infectious Diseases Research & Education Centre (TIDREC), Universiti Malaya	AsmaAnati CheMatSeri; Che-Norainon Yaacob; Jia-Yi Tan; Jo-Ern Wong; Kim-Kee Tan; Mulya-Mustika-Sari Zulkifli; Noor Syahida Azizan; Nur-Hidayana Mahfodz; Sazaly AbuBakar; Siti-Sarah Nor'e; Wei-Wei Aw
EPI_ISL_9804104, EPI_ISL_9804105, EPI_ISL_11332474	Rejang Medical Centre (Sibu)	Institute of Health and Community Medicine	Chan Chia Jui; Chua Hock Hin; David Perera; Ooi Mong How; Tonnii Sia Loong Loong; Wong Jyn Shan
EPI_ISL_9608490, EPI_ISL_9608492, EPI_ISL_9608517, EPI_ISL_9804102, EPI_ISL_9804103, EPI_ISL_9804106, EPI_ISL_9804107, EPI_ISL_9804108, EPI_ISL_9804109, EPI_ISL_9804114, EPI_ISL_9804117, EPI_ISL_9804125, EPI_ISL_9804133, EPI_ISL_9804134, EPI_ISL_9804144, EPI_ISL_9804145, EPI_ISL_9804146, EPI_ISL_9804150, EPI_ISL_9804159, EPI_ISL_9804161			
see above	Sarawak General Hospital (Kuching)	Institute of Health and Community Medicine	Chan Chia Jui; Chua Hock Hin; David Perera; Ooi Mong How; Tonnii Sia Loong Loong
EPI_ISL_9804130, EPI_ISL_9804131, EPI_ISL_9804132, EPI_ISL_9804136, EPI_ISL_9804167, EPI_ISL_9804168, EPI_ISL_10512261, EPI_ISL_10512262, EPI_ISL_10512263, EPI_ISL_10512264, EPI_ISL_10512502			
see above	Sibu Hospital (Sibu, Sarawak)	Institute of Health and Community Medicine	Chan Chia Jui; Chua Hock Hin; David Perera; Ooi Mong How; Tonnii Sia Loong Loong
EPI_ISL_10494671	Sijangkang Health Clinic	Institute for Medical Research, Infectious Disease Research Centre, National Institutes of Health, Ministry of Health Malaysia	Ahmad FA; Ahmad Fazilah NA; Anasir MI; Azizan MA; Kamel K; Mohamad Sukri MZ; Norhisham SN; Ramly N; Robert F; Rosli NR; Suppiah J; Thayan R
EPI_ISL_11332722	Sultanah Maliha Hospital, Langkawi	Tropical Infectious Diseases Research & Education Centre (TIDREC), Universiti Malaya	AsmaAnati CheMatSeri; Che-Norainon Yaacob; Jia-Yi Tan; Jo-Ern Wong; Kim-Kee Tan; Mulya-Mustika-Sari Zulkifli; Noor Syahida Azizan; Nur-Hidayana Mahfodz; Sazaly AbuBakar; Siti-Sarah Nor'e; Wei-Wei Aw
EPI_ISL_11332717	Sultanah Nur Zahirah Hospital, Kuala Terengganu	Tropical Infectious Diseases Research & Education Centre (TIDREC), Universiti Malaya	AsmaAnati CheMatSeri; Che-Norainon Yaacob; Jia-Yi Tan; Jo-Ern Wong; Kim-Kee Tan; Mulya-Mustika-Sari Zulkifli; Noor Syahida Azizan; Nur-Hidayana Mahfodz; Sazaly AbuBakar; Siti-Sarah Nor'e; Wei-Wei Aw
EPI_ISL_12322083	THOMSON HOSPITAL	Institute for Medical Research, Infectious Disease Research Centre, National Institutes of Health, Ministry of Health Malaysia	Ahmad FA; Ahmad Fazilah NA; Anasir MI; Azizan MA; Kamel K; Mohamad Sukri MZ; Norhisham SN; Ramly N; Robert F; Rosli NR; Suppiah J; Thayan R
EPI_ISL_10494690	Tanjung Sepat Health Clinic	Institute for Medical Research, Infectious Disease Research Centre, National Institutes of Health, Ministry of Health Malaysia	Ahmad FA; Ahmad Fazilah NA; Anasir MI; Azizan MA; Kamel K; Mohamad Sukri MZ; Norhisham SN; Ramly N; Robert F; Rosli NR; Suppiah J; Thayan R
EPI_ISL_10494674, EPI_ISL_10494675, EPI_ISL_10494676, EPI_ISL_10494677, EPI_ISL_10494678, EPI_ISL_10494679, EPI_ISL_10494680, EPI_ISL_10494691, EPI_ISL_10494692			
see above	Telok Datok Health Clinic	Institute for Medical Research, Infectious Disease Research Centre, National Institutes of Health, Ministry of Health Malaysia	Ahmad FA; Ahmad Fazilah NA; Anasir MI; Azizan MA; Kamel K; Mohamad Sukri MZ; Norhisham SN; Ramly N; Robert F; Rosli NR; Suppiah J; Thayan R
EPI_ISL_10494672, EPI_ISL_10494673	Telok Panglima Garang Health Clinic	Institute for Medical Research, Infectious Disease Research Centre, National Institutes of Health, Ministry of Health Malaysia	Ahmad FA; Ahmad Fazilah NA; Anasir MI; Azizan MA; Kamel K; Mohamad Sukri MZ; Norhisham SN; Ramly N; Robert F; Rosli NR; Suppiah J; Thayan R
EPI_ISL_10494700, EPI_ISL_10494701, EPI_ISL_10494702, EPI_ISL_10494703, EPI_ISL_10494704, EPI_ISL_10494705, EPI_ISL_10494706, EPI_ISL_11148315			
see above	The National Defence University of Malaysia	Institute for Medical Research, Infectious Disease Research Centre, National Institutes of Health, Ministry of Health Malaysia	Ahmad FA; Ahmad Fazilah NA; Anasir MI; Azizan MA; Kamel K; Mohamad Sukri MZ; Norhisham SN; Ramly N; Robert F; Rosli NR; Suppiah J; Thayan R
EPI_ISL_12008481, EPI_ISL_12008510, EPI_ISL_12008512, EPI_ISL_12008518	UKKP HOSPITAL MELAKA	UKM Medical Molecular Biology Institute (UMBI)	Khairun Nur Abd Ghafar; Mira Farzana Mohamad Mokhtar; Muhiddin Ishak; Nor Azila Muhammad Azami; Nur Alyaa Afifah Md Shahri; Nurul Syakima Ab Mutalib; Rahman Jamal; Ryia Illani Mohd Yunos; Siti Nur Hasanah Mohd Yusuf; Zahirrah Begam Mohamed Rasheed
EPI_ISL_11754706	UMMC	Department of Medical Microbiology, Faculty of Medicine, University of Malaya; University of Malaya Medical Centre	I-Ching SAM; Jolene Yin Ling FU; Omar Khalilur Rahman; Yoke Fun Chan
EPI_ISL_12008487	UNIT KESELAMATAN DAN KESIHATAN PEKERJAAN	UKM Medical Molecular Biology Institute (UMBI)	Khairun Nur Abd Ghafar; Mira Farzana Mohamad Mokhtar; Muhiddin Ishak; Nor Azila Muhammad Azami; Nur Alyaa Afifah Md Shahri; Nurul Syakima Ab Mutalib; Rahman Jamal; Ryia Illani Mohd Yunos; Siti Nur Hasanah Mohd Yusuf; Zahirrah Begam Mohamed Rasheed
EPI_ISL_10942296	UNIT KESIHATAN AWAM	UKM Medical Molecular Biology Institute (UMBI)	Khairun Nur Abd Ghafar; Mira Farzana Mohamad Mokhtar; Muhiddin Ishak; Nor Azila Muhammad Azami; Nur Alyaa Afifah Md Shahri; Nurul Syakima Ab Mutalib; Rahman Jamal; Ryia Illani Mohd Yunos; Siti Nur Hasanah Mohd Yusuf; Zahirrah Begam Mohamed Rasheed
EPI_ISL_10942300, EPI_ISL_10942308, EPI_ISL_10942311, EPI_ISL_10942317, EPI_ISL_10942328, EPI_ISL_10942340, EPI_ISL_10942342, EPI_ISL_12008438, EPI_ISL_12008516, EPI_ISL_12097435			
see above	UNIT KESIHATAN AWAM HEBHK	UKM Medical Molecular Biology Institute (UMBI)	Khairun Nur Abd Ghafar; Mira Farzana Mohamad Mokhtar; Muhiddin Ishak; Nor Azila Muhammad Azami; Nur Alyaa Afifah Md Shahri; Nurul Syakima Ab Mutalib; Rahman Jamal; Ryia Illani Mohd Yunos; Siti Nur Hasanah Mohd Yusuf; Zahirrah Begam Mohamed Rasheed
EPI_ISL_10657474	Unit Perubatan Forensik Hospital Tawau	Institute for Medical Research, Infectious Disease Research Centre, National Institutes of Health, Ministry of Health Malaysia	Ahmad FA; Ahmad Fazilah NA; Anasir MI; Azizan MA; G. Adypatti NM; Jamaluddin MS; Kamel K; Mohamad Sukri MZ; Norhisham SN; Ramly N; Robert F; Rosli NR; Suppiah J; Thayan R
EPI_ISL_12771150	jabatan forensik hpp	iPROMISE, UiTM	Ariza Adnan; Fadzilah Mohd Nor; Lim Wai Feng; Mohd Asif Mohd Sukri; Mohd Nur Fakhruzzaman Noorizhab; Mohd Zaki Salleh; Sazzli Shahlan Kassim; Siti Farah Alwani Mohd Nawi; Siti Hamimah Sheikh Abdul Kadir; Teh Lay Kek; Wang Seok Mui
EPI_ISL_10652525, EPI_ISL_10652526, EPI_ISL_10652527	mka kb	Department of Medical Microbiology, Faculty of Medicine, University of Malaya; University of Malaya Medical Centre	I-Ching SAM; Jolene Yin Ling FU; Omar Khalilur Rahman; Yoke Fun Chan

We gratefully acknowledge the following Authors from the Originating laboratories responsible for obtaining the specimens, as well as the Submitting laboratories where the genome data were generated and shared via GISAID, on which this research is based.

All Submitters of data may be contacted directly via www.gisaid.org

Authors are sorted alphabetically.

Accession ID	Originating Laboratory	Submitting Laboratory	Authors
EPI_ISL_10981860	Ampang Hospital	Institute for Medical Research, Infectious Disease Research Centre, National Institutes of Health, Ministry of Health Malaysia	Ahmad FA; Ahmad Fazilah NA; Anasir MI; Azizan MA; Kamel K; Mohamad Sukri MZ; Norhisham SN; Ramly N; Robert F; Rosli NR; Suppiah J; Thayan R
EPI_ISL_12771307, EPI_ISL_12771308	BP Lab Penang	iPROMISE, UiTM	Ariza Adnan; Fadzilah Mohd Nor; Lim Wai Feng; Mohd Asif Mohd Sukri; Mohd Nur Fakhruzzaman Noorizhab; Mohd Zaki Salleh; Sazzli Shahlan Kassim; Siti Farah Alwani Mohd Nawi; Siti Hamimah Sheikh Abdul Kadir; Teh Lay Kek; Wang Seok Mui
EPI_ISL_10512343, EPI_ISL_10512344, EPI_ISL_10512346, EPI_ISL_10512347	Bintulu Hospital PCR Lab, Bintulu	Institute of Health and Community Medicine	Chan Chia Jui; Chua Hock Hin; David Perera; Ooi Mong How; Tan Lee See; Tonnii Sia Loong Loong; Wong Jyn Shan
EPI_ISL_10512324, EPI_ISL_10512415, EPI_ISL_10512416, EPI_ISL_10512417, EPI_ISL_10512418	Bintulu Medical Centre (Bintulu)	Institute of Health and Community Medicine	Chan Chia Jui; Chua Hock Hin; David Perera; Ooi Mong How; Tonnii Sia Loong Loong; Wong Jyn Shan
EPI_ISL_9804097, EPI_ISL_9804098, EPI_ISL_9804099, EPI_ISL_9804100, EPI_ISL_9804101, EPI_ISL_9804141, EPI_ISL_9804142, EPI_ISL_9804143, EPI_ISL_10512328, EPI_ISL_10512329, EPI_ISL_10512335, EPI_ISL_10512339, EPI_ISL_10512411			
see above	Borneo Medical Centre (Kuching)	Institute of Health and Community Medicine	Chan Chia Jui; Chua Hock Hin; David Perera; Ooi Mong How; Tonnii Sia Loong Loong; Wong Jyn Shan
EPI_ISL_10512258, EPI_ISL_10512259, EPI_ISL_10512260	Borneo Medical Centre (Miri)	Institute of Health and Community Medicine	Chan Chia Jui; Chua Hock Hin; David Perera; Ooi Mong How; Tonnii Sia Loong Loong; Wong Jyn Shan
EPI_ISL_11344868, EPI_ISL_11344870, EPI_ISL_11344871, EPI_ISL_11344872, EPI_ISL_11344873	Department of Medical Microbiology and Parasitology, School of Medical Sciences, Universiti Sains Malaysia Health Campus	Molecular Research Laboratory, Department of Medical Microbiology and Parasitology, School of Medical Sciences, Universiti Sains Malaysia Health Campus	Abdul Haris bin Muhammad; Ahmad Sukari Bin Halim; Alexander Chong Shu Chien; Asraihan Che Abdul Malik; Azian Harun; Chan Yean Yean; Chua Wei Chuan; Farahana binti Mohamed; Kirnpal Kaur Banga Singh; Lau Nyok Sean; Lee Lih Huey; Lim Shu Yong; Maizun binti Mohd Zain; Mera Edora Binti Abdul Manap; Mohd Iman hafiz Bin Ibrahim; Mohd Nadzri Abu Yazid; Mohd Zulkifli Salleh; Muhamad Khairul Amirin Bin Zulkifli; Muhammad Azamuddeen bin Mohammad Nasir; Muhammad Fazli bin Khalid; Muhammad Nashrul Farhan Samsudin; Muhammad Zarul Hanifah Bin Md Zoqratt; Nik Zuraina binti Nik Mohd Noor; Noor Hafizan binti Mat Salleh; Nor Amizara Binti Azami; Nor Suhana Binti Mohd Satar; Nur Syuhada Binti Abdul Rahim; Nur-Leem Binti Murshid; Nurfadhlina Musa; Qasim Ayub; Rosline Hassan; Sadequr Rahman; Siti Nur Aisyah Binti Mohamad Sham; Syahida binti Omar; Wan Mohd Zahiruddin Wan Mohammad; Wardah Yusof; Zaini bin Hussin; Zakuan Zainy Deris
EPI_ISL_12721580	Department of Medical Microbiology, Hospital Pengajar Universiti Putra Malaysia	Department of Medical Microbiology, Hospital Pengajar Universiti Putra Malaysia	Afiqah Adzmi; Amiza Azmi; Azmiza Syawani Jasni; Hui Yee Chee; Leslie Thian Lung Than; Muadz Mohtar; Muhammad Mohd Isa; Narcisse MS Joseph; Niazlin Mohd Taib; Noor Hazirah Noor Azhari; Norlaila; Nur Raihana Ithnin; Nurul Huda Mohamed Rashidi; Nurul Nadiah Ismail; Rosni Ibrahim; Rukman Awang Hamat; Sallehhudin; Siti Norbaya Masri; Siti Zulaikha Zakariah; Syafinaz Amin Nordin; Tengku Zetty Maztura Tengku Jamaluddin; Zamberi Sekawi
EPI_ISL_10512207, EPI_ISL_10512267, EPI_ISL_10512270, EPI_ISL_10512273, EPI_ISL_10512279, EPI_ISL_10512287, EPI_ISL_10512334, EPI_ISL_10512336, EPI_ISL_10512337, EPI_ISL_10512338			
see above	Gribbles Diagnostic Lab @Columbia Asia Hospital, Miri	Institute of Health and Community Medicine	Affif bin Zainal Abidin; Chan Chia Jui; Chua Hock Hin; David Perera; Ooi Mong How; Tonnii Sia Loong Loong
EPI_ISL_10512183, EPI_ISL_10512184, EPI_ISL_10512185, EPI_ISL_10512186, EPI_ISL_10512187, EPI_ISL_10512188, EPI_ISL_10512189, EPI_ISL_10512265, EPI_ISL_10512268, EPI_ISL_10512269, EPI_ISL_10512271, EPI_ISL_10512272, EPI_ISL_10512274, EPI_ISL_10512275, EPI_ISL_10512276, EPI_ISL_10512277, EPI_ISL_10512278, EPI_ISL_10512280, EPI_ISL_10512281, EPI_ISL_10512282, EPI_ISL_10512283, EPI_ISL_10512284, EPI_ISL_10512285, EPI_ISL_10512286, EPI_ISL_10512288, EPI_ISL_10512289, EPI_ISL_10512290, EPI_ISL_10512291, EPI_ISL_10512292, EPI_ISL_10512294, EPI_ISL_10512295, EPI_ISL_10512296, EPI_ISL_10512297, EPI_ISL_10512298			
see above	Gribbles Pathology, Kuching	Institute of Health and Community Medicine	Chan Chia Jui; Chua Hock Hin; David Perera; Liew Chin Chin J; Ooi Mong How; Tonnii Sia Loong Loong; Wong Jyn Shan
EPI_ISL_12008469, EPI_ISL_12008502, EPI_ISL_12008515	HOSPITAL MELAKA	UKM Medical Molecular Biology Institute (UMBI)	Khairun Nur Abd Ghafar; Mira Farzana Mohamad Mokhtar; Muhiddin Ishak; Nor Azila Muhammad Azami; Nur Alyaa Afifah Md Shahri; Nurul Syakima Ab Mutalib; Rahman Jamal; Ryia Illani Mohd Yunos; Siti Nur Hasanah Mohd Yusuf; Zahirrah Begam Mohamed Rasheed
EPI_ISL_12771158	HOSPITAL PULAU PINANG	iPROMISE, UiTM	Ariza Adnan; Fadzilah Mohd Nor; Lim Wai Feng; Mohd Asif Mohd Sukri; Mohd Nur Fakhruzzaman Noorizhab; Mohd Zaki Salleh; Sazzli Shahlan Kassim; Siti Farah Alwani Mohd Nawi; Siti Hamimah Sheikh Abdul Kadir; Teh Lay Kek; Wang Seok Mui
EPI_ISL_12135923	HOSPITAL RAJA PERMAISURI BAINUN	Tropical Infectious Diseases Research & Education Centre (TIDREC), Universiti Malaya	AsmaAnati CheMatSeri; Che-Norainon Yaacob; Jia-Yi Tan; Jo-Ern Wong; Kim-Kee Tan; Mulya-Mustika-Sari Zulkifli; Noor Syahida Azizan; Nur-Hidayana Mahfodz; Sazaly AbuBakar; Siti-Sarah Nor'e; Wei-Wei Aw
EPI_ISL_12135918, EPI_ISL_12135919, EPI_ISL_12135920, EPI_ISL_12135921, EPI_ISL_12135922	HOSPITAL RAJA PERMAISURI BAINUN, IPOH	Tropical Infectious Diseases Research & Education Centre (TIDREC), Universiti Malaya	AsmaAnati CheMatSeri; Che-Norainon Yaacob; Jia-Yi Tan; Jo-Ern Wong; Kim-Kee Tan; Mulya-Mustika-Sari Zulkifli; Noor Syahida Azizan; Nur-Hidayana Mahfodz; Sazaly AbuBakar; Siti-Sarah Nor'e; Wei-Wei Aw
EPI_ISL_12135915, EPI_ISL_12135916	HOSPITAL SULTANAH NUR ZAHIRAH	Tropical Infectious Diseases Research & Education Centre (TIDREC), Universiti Malaya	AsmaAnati CheMatSeri; Che-Norainon Yaacob; Jia-Yi Tan; Jo-Ern Wong; Kim-Kee Tan; Mulya-Mustika-Sari Zulkifli; Noor Syahida Azizan; Nur-Hidayana Mahfodz; Sazaly AbuBakar; Siti-Sarah Nor'e; Wei-Wei Aw
EPI_ISL_11939176, EPI_ISL_12771281	HPP	iPROMISE, UiTM	Ariza Adnan; Fadzilah Mohd Nor; Lim Wai Feng; Mohd Asif Mohd Sukri; Mohd Nur Fakhruzzaman Noorizhab; Mohd Zaki Salleh; Sazzli Shahlan Kassim; Siti Farah Alwani Mohd Nawi; Siti Hamimah Sheikh Abdul Kadir; Teh Lay Kek; Wang Seok Mui
EPI_ISL_12008440, EPI_ISL_12008441, EPI_ISL_12008442, EPI_ISL_12008443, EPI_ISL_12008490	HPSF	UKM Medical Molecular Biology Institute (UMBI)	Khairun Nur Abd Ghafar; Mira Farzana Mohamad Mokhtar; Muhiddin Ishak; Nor Azila Muhammad Azami; Nur Alyaa Afifah Md Shahri; Nurul Syakima Ab Mutalib; Rahman Jamal; Ryia Illani Mohd Yunos; Siti Nur Hasanah Mohd Yusuf; Zahirrah Begam Mohamed Rasheed
EPI_ISL_10880073, EPI_ISL_10880078, EPI_ISL_10880096, EPI_ISL_10880114	HPSF MUAR	UKM Medical Molecular Biology Institute (UMBI)	Khairun Nur Abd Ghafar; Mira Farzana Mohamad Mokhtar; Muhiddin Ishak; Nor Azila Muhammad Azami; Nur Alyaa Afifah Md Shahri; Nurul Syakima Ab Mutalib; Rahman Jamal; Ryia Illani Mohd Yunos; Siti Nur Hasanah Mohd Yusuf; Zahirrah Begam Mohamed Rasheed
EPI_ISL_12135882, EPI_ISL_12135883, EPI_ISL_12135884, EPI_ISL_12135885, EPI_ISL_12135886, EPI_ISL_12135887, EPI_ISL_12135888			
see above	HRPB	Tropical Infectious Diseases Research & Education Centre (TIDREC), Universiti Malaya	AsmaAnati CheMatSeri; Che-Norainon Yaacob; Jia-Yi Tan; Jo-Ern Wong; Kim-Kee Tan; Mulya-Mustika-Sari Zulkifli; Noor Syahida Azizan; Nur-Hidayana Mahfodz; Sazaly AbuBakar; Siti-Sarah Nor'e; Wei-Wei Aw
EPI_ISL_12135910, EPI_ISL_12135911, EPI_ISL_12135912, EPI_ISL_12135913	HRPZ II	Tropical Infectious Diseases Research & Education Centre (TIDREC), Universiti Malaya	AsmaAnati CheMatSeri; Che-Norainon Yaacob; Jia-Yi Tan; Jo-Ern Wong; Kim-Kee Tan; Mulya-Mustika-Sari Zulkifli; Noor Syahida Azizan; Nur-Hidayana Mahfodz; Sazaly AbuBakar; Siti-Sarah Nor'e; Wei-Wei Aw
EPI_ISL_12008448, EPI_ISL_12008473, EPI_ISL_12008528	HSAJB	UKM Medical Molecular Biology Institute (UMBI)	Khairun Nur Abd Ghafar; Mira Farzana Mohamad Mokhtar; Muhiddin Ishak; Nor Azila Muhammad Azami; Nur Alyaa Afifah Md Shahri; Nurul Syakima Ab Mutalib; Rahman Jamal; Ryia Illani Mohd Yunos; Siti Nur Hasanah Mohd Yusuf; Zahirrah Begam Mohamed Rasheed
EPI_ISL_10942287, EPI_ISL_10942303, EPI_ISL_10942336	HTJS	UKM Medical Molecular Biology Institute (UMBI)	Khairun Nur Abd Ghafar; Mira Farzana Mohamad Mokhtar; Muhiddin Ishak; Nor Azila Muhammad Azami; Nur Alyaa Afifah Md Shahri; Nurul Syakima Ab Mutalib; Rahman Jamal; Ryia Illani Mohd Yunos; Siti Nur Hasanah Mohd Yusuf; Zahirrah Begam Mohamed Rasheed
EPI_ISL_12277159, EPI_ISL_12277160, EPI_ISL_12277161, EPI_ISL_12277162, EPI_ISL_12277163, EPI_ISL_12277164, EPI_ISL_12277165, EPI_ISL_12277166, EPI_ISL_12277167, EPI_ISL_12277206, EPI_ISL_12277207			
see above	Hospital Ampang	Malaysia Genome and Vaccine Institute	Azrin Ahmad; Enizza Kasim; Irni Suhayu Sapian; Mohd Faizal Abu Bakar; Mohd Ghows Mohd Azzam.; Mohd Noor Mat Isa; Nor Azfa Johari; Nurhezreen Md Iqbal; Shamsidar Sopie; Siti Noraini Othman; Yusuf Muhammad Noor
EPI_ISL_11332723, EPI_ISL_11332724, EPI_ISL_11332725	Hospital Angkatan Tentera	Tropical Infectious Diseases Research & Education Centre (TIDREC), Universiti Malaya	AsmaAnati CheMatSeri; Che-Norainon Yaacob; Jia-Yi Tan; Jo-Ern Wong; Kim-Kee Tan; Mulya-Mustika-Sari Zulkifli; Noor Syahida Azizan; Nur-Hidayana Mahfodz; Sazaly AbuBakar; Siti-Sarah Nor'e; Wei-Wei Aw
EPI_ISL_11900044, EPI_ISL_11900046, EPI_ISL_11900047, EPI_ISL_11900048, EPI_ISL_11900060	Hospital Selayang	Malaysia Genome and Vaccine Institute	Azrin Ahmad; Enizza Kasim; Irni Suhayu Sapian; Mohd Faizal Abu Bakar; Mohd Ghows Mohd Azzam.; Mohd Noor Mat Isa; Nor Azfa Johari; Nurhezreen Md Iqbal; Shamsidar Sopie; Siti Noraini Othman; Yusuf Muhammad Noor
EPI_ISL_12135917	Hospital Sultanah Nur Zahirah	Tropical Infectious Diseases Research & Education Centre (TIDREC), Universiti Malaya	AsmaAnati CheMatSeri; Che-Norainon Yaacob; Jia-Yi Tan; Jo-Ern Wong; Kim-Kee Tan; Mulya-Mustika-Sari Zulkifli; Noor Syahida Azizan; Nur-Hidayana Mahfodz; Sazaly AbuBakar; Siti-Sarah Nor'e; Wei-Wei Aw
EPI_ISL_10981859, EPI_ISL_10981867, EPI_ISL_10981877,	Hospital Sungai Buloh	Institute for Medical Research, Infectious Disease Research Centre, National Institutes of Health, Ministry of Health Malaysia	Ahmad FA; Ahmad Fazilah NA; Anasir MI; Azizan MA; Kamel K; Mohamad Sukri MZ; Norhisham SN; Ramly N; Robert F; Rosli NR; Suppiah J; Thayan R

EPI_ISL_10981884, EPI_ISL_10981892			
EPI_ISL_11976171	Hospital Tengku Ampuan Rahimah	Malaysia Genome and Vaccine Institute	Azrin Ahmad; Enizza Kasim; Irni Suhayu Sapian; Mohd Faizal Abu Bakar; Mohd Ghows Mohd Azzam.; Mohd Noor Mat Isa; Nor Azfa Johari; Nurhezreen Md Iqbal; Shamsidar Sopie; Siti Noraini Othman; Yusuf Muhammad Noor
EPI_ISL_11900045	Hospital selayang	Malaysia Genome and Vaccine Institute	Azrin Ahmad; Enizza Kasim; Irni Suhayu Sapian; Mohd Faizal Abu Bakar; Mohd Ghows Mohd Azzam.; Mohd Noor Mat Isa; Nor Azfa Johari; Nurhezreen Md Iqbal; Shamsidar Sopie; Siti Noraini Othman; Yusuf Muhammad Noor
EPI_ISL_11323761, EPI_ISL_11323810, EPI_ISL_11323834	Institut Biologi Molekul Perubatan UKM (UMBI)	UKM Medical Molecular Biology Institute (UMBI)	Khairun Nur Abd Ghafar; Mira Farzana Mohamad Mokhtar; Muhiddin Ishak; Nor Azila Muhammad Azami; Nur Alyaa Afifah Md Shahri; Nurul Syakima Ab Mutalib; Rahman Jamal; Ryia Illani Mohd Yunos; Siti Nur Hasanah Mohd Yusuf; Zahirrah Begam Mohamed Rasheed
EPI_ISL_10981879	Institute for Medical Research	Institute for Medical Research, Infectious Disease Research Centre, National Institutes of Health, Ministry of Health Malaysia	Ahmad FA; Ahmad Fazilah NA; Anasir MI; Azizan MA; Kamel K; Mohamad Sukri MZ; Norhisham SN; Ramly N; Robert F; Rosli NR; Suppiah J; Thayan R
EPI_ISL_11939140, EPI_ISL_11939193, EPI_ISL_11939194, EPI_ISL_11939196, EPI_ISL_11939197, EPI_ISL_11939199, EPI_ISL_11939200, EPI_ISL_11939201			
see above	JKN PAHANG	iPROMISE, UiTM	Ariza Adnan; Fadzilah Mohd Nor; Lim Wai Feng; Mohd Asif Mohd Sukri; Mohd Nur Fakhruzzaman Noorizhab; Mohd Zaki Salleh; Sazzli Shahlan Kassim; Siti Farah Alwani Mohd Nawi; Siti Hamimah Sheikh Abdul Kadir; Teh Lay Kek; Wang Seok Mui
EPI_ISL_12135903, EPI_ISL_12135904, EPI_ISL_12135905, EPI_ISL_12135944	Jabatan Forensik Hospital Sultanah Bahiyah	Tropical Infectious Diseases Research & Education Centre (TIDREC), Universiti Malaya	AsmaAnati CheMatSeri; Che-Norainon Yaacob; Jia-Yi Tan; Jo-Ern Wong; Kim-Kee Tan; Mulya-Mustika-Sari Zulkifli; Noor Syahida Azizan; Nur-Hidayana Mahfodz; Sazaly AbuBakar; Siti-Sarah Nor'e; Wei-Wei Aw
EPI_ISL_11965256	Jenjarom Health Clinic	Institute for Medical Research, Infectious Disease Research Centre, National Institutes of Health, Ministry of Health Malaysia	Ahmad FA; Ahmad Fazilah NA; Anasir MI; Azizan MA; Kamel K; Mohamad Sukri MZ; Norhisham SN; Ramly N; Robert F; Rosli NR; Suppiah J; Thayan R
EPI_ISL_12055316	KK JELAI	Institut Biologi Molekul Perubatan UKM (UMBI)	Khairun Nur Abd Ghafar; Mira Farzana Mohamad Mokhtar; Muhiddin Ishak; Nor Azila Muhammad Azami; Nur Alyaa Afifah Md Shahri; Nurul Syakima Ab Mutalib; Rahman Jamal; Ryia Illani Mohd Yunos; Siti Nur Hasanah Mohd Yusuf; Zahirrah Begam Mohamed Rasheed
EPI_ISL_11965265, EPI_ISL_12097422	Kampung Bandar Health Clinic	Institute for Medical Research, Infectious Disease Research Centre, National Institutes of Health, Ministry of Health Malaysia	Ahmad FA; Ahmad Fazilah NA; Anasir MI; Azizan MA; Kamel K; Mohamad Sukri MZ; Norhisham SN; Ramly N; Robert F; Rosli NR; Suppiah J; Thayan R
EPI_ISL_11332757, EPI_ISL_11332758, EPI_ISL_11332760, EPI_ISL_11332761, EPI_ISL_11332764, EPI_ISL_11332766, EPI_ISL_11332767, EPI_ISL_11332768			
see above	Kota Bharu Public Health Laboratory	Molecular Research Laboratory	Abdul Haris bin Muhammad; Ahmad Sukari Bin Halim; Alexander Chong Shu Chien; Asraihan Che Abdul Malik; Azian Harun; Chan Yean Yean; Chua Wei Chuan; Farahana binti Mohamed; Kirnpal Kaur Banga Singh; Lau Nyok Sean; Lee Lih Huey; Lim Shu Yong; Maizun binti Mohd Zain; Mera Edora Binti Abdul Manap; Mohd Iman hafiz Bin Ibrahim; Mohd Nadzri Abu Yazid; Mohd Zulkifli Salleh; Muhamad Khairul Amirin Bin Zulkifli; Muhammad Azamuddeen bin Mohammad Nasir; Muhammad Fazli bin Khalid; Muhammad Nashrul Farhan Samsudin; Muhammad Zarul Hanifah Bin Md Zoqratt; Nik Zuraina binti Nik Mohd Noor; Noor Hafizan binti Mat Salleh; Nor Amizara Binti Azami; Nor Suhana Binti Mohd Satar; Nur Syuhada Binti Abdul Rahim; Nur-Leem Binti Murshid; Nurfadhlina Musa; Qasim Ayub; Rosline Hassan; Sadequr Rahman; Siti Nur Aisyah Binti Mohamad Sham; Syahida binti Omar; Wan Mohd Zahiruddin Wan Mohammad; Wardah Yusof; Zaini bin Hussin; Zakuan Zainy Deris
EPI_ISL_11344874, EPI_ISL_11344875, EPI_ISL_11344876, EPI_ISL_11344877, EPI_ISL_11344878, EPI_ISL_11344879, EPI_ISL_11344880, EPI_ISL_11344881, EPI_ISL_11344882, EPI_ISL_11344883			
see above	Kota Bharu Public Health Laboratory	Molecular Research Laboratory, Department of Medical Microbiology and Parasitology, School of Medical Sciences, Universiti Sains Malaysia Health Campus	Abdul Haris bin Muhammad; Ahmad Sukari Bin Halim; Alexander Chong Shu Chien; Asraihan Che Abdul Malik; Azian Harun; Chan Yean Yean; Chua Wei Chuan; Farahana binti Mohamed; Kirnpal Kaur Banga Singh; Lau Nyok Sean; Lee Lih Huey; Lim Shu Yong; Maizun binti Mohd Zain; Mera Edora Binti Abdul Manap; Mohd Iman hafiz Bin Ibrahim; Mohd Nadzri Abu Yazid; Mohd Zulkifli Salleh; Muhamad Khairul Amirin Bin Zulkifli; Muhammad Azamuddeen bin Mohammad Nasir; Muhammad Fazli bin Khalid; Muhammad Nashrul Farhan Samsudin; Muhammad Zarul Hanifah Bin Md Zoqratt; Nik Zuraina binti Nik Mohd Noor; Noor Hafizan binti Mat Salleh; Nor Amizara Binti Azami; Nor Suhana Binti Mohd Satar; Nur Syuhada Binti Abdul Rahim; Nur-Leem Binti Murshid; Nurfadhlina Musa; Qasim Ayub; Rosline Hassan; Sadequr Rahman; Siti Nur Aisyah Binti Mohamad Sham; Syahida binti Omar; Wan Mohd Zahiruddin Wan Mohammad; Wardah Yusof; Zaini bin Hussin; Zakuan Zainy Deris
EPI_ISL_11965305, EPI_ISL_11965307, EPI_ISL_11965309	Kota Kinabalu Area Health Office	Institute for Medical Research, Infectious Disease Research Centre, National Institutes of Health, Ministry of Health Malaysia	Ahmad FA; Ahmad Fazilah NA; Anasir MI; Azizan MA; Kamel K; Mohamad Sukri MZ; Norhisham SN; Ramly N; Robert F; Rosli NR; Suppiah J; Thayan R
EPI_ISL_11965311	Kota Marudu District Health Office	Institute for Medical Research, Infectious Disease Research Centre, National Institutes of Health, Ministry of Health Malaysia	Ahmad FA; Ahmad Fazilah NA; Anasir MI; Azizan MA; Kamel K; Mohamad Sukri MZ; Norhisham SN; Ramly N; Robert F; Rosli NR; Suppiah J; Thayan R
EPI_ISL_10981864	Kuala Kubu Bharu Hospital	Institute for Medical Research, Infectious Disease Research Centre, National Institutes of Health, Ministry of Health Malaysia	Ahmad FA; Ahmad Fazilah NA; Anasir MI; Azizan MA; Kamel K; Mohamad Sukri MZ; Norhisham SN; Ramly N; Robert F; Rosli NR; Suppiah J; Thayan R
EPI_ISL_11966111, EPI_ISL_11966112, EPI_ISL_11966118, EPI_ISL_11966123, EPI_ISL_11966130, EPI_ISL_11966136, EPI_ISL_11966142, EPI_ISL_11966149, EPI_ISL_11966158, EPI_ISL_11966164, EPI_ISL_11966175			
see above	Kuala Lumpur International Airport (KLIA) Health Office	Institute for Medical Research, Infectious Disease Research Centre, National Institutes of Health, Ministry of Health Malaysia	Ahmad FA; Ahmad Fazilah NA; Anasir MI; Azizan MA; Kamel K; Mohamad Sukri MZ; Norhisham SN; Ramly N; Robert F; Rosli NR; Suppiah J; Thayan R
EPI_ISL_11332470	Limbang Hospital Diagnostic Lab, Limbang	Institute of Health and Community Medicine	Chan Chia Jui; Chua Hock Hin; David Perera; Ooi Mong How; Tonnii Sia Loong Loong
EPI_ISL_12135890	MKA KB	Tropical Infectious Diseases Research & Education Centre (TIDREC), Universiti Malaya	AsmaAnati CheMatSeri; Che-Norainon Yaacob; Jia-Yi Tan; Jo-Ern Wong; Kim-Kee Tan; Mulya-Mustika-Sari Zulkifli; Noor Syahida Azizan; Nur-Hidayana Mahfodz; Sazaly AbuBakar; Siti-Sarah Nor'e; Wei-Wei Aw
EPI_ISL_11939157, EPI_ISL_11939158, EPI_ISL_11939159, EPI_ISL_11939160, EPI_ISL_11939161, EPI_ISL_11939169, EPI_ISL_11939170, EPI_ISL_11939171, EPI_ISL_11939172, EPI_ISL_11939174, EPI_ISL_11939175, EPI_ISL_11939179, EPI_ISL_11939180, EPI_ISL_11939182, EPI_ISL_11939183, EPI_ISL_11939184, EPI_ISL_12771268, EPI_ISL_12771276, EPI_ISL_12771277, EPI_ISL_12771278, EPI_ISL_12771279, EPI_ISL_12771280, EPI_ISL_12771285			
see above	Medical Hospital Pulau Pinang	iPROMISE, UiTM	Ariza Adnan; Fadzilah Mohd Nor; Lim Wai Feng; Mohd Asif Mohd Sukri; Mohd Nur Fakhruzzaman Noorizhab; Mohd Zaki Salleh; Sazzli Shahlan Kassim; Siti Farah Alwani Mohd Nawi; Siti Hamimah Sheikh Abdul Kadir; Teh Lay Kek; Wang Seok Mui
EPI_ISL_10759881	Miri Hospital Molecular Diagnostic Lab, Miri	Institute of Health and Community Medicine	Chan Chia Jui; Chua Hock Hin; David Perera; Ooi Mong How; Tonnii Sia Loong Loong
EPI_ISL_11672760, EPI_ISL_11672761, EPI_ISL_11672762, EPI_ISL_11672763, EPI_ISL_11672773, EPI_ISL_11672774, EPI_ISL_11672775, EPI_ISL_11672776, EPI_ISL_11717955			
see above	National Public Health Laboratory	National Public Health Laboratory	Aimi Shazana Binti Shahir; Kamal Hisham Bin Kamarul Zaman; Muhammad Shahir Bin Ali; Noriah Binti Mohd Yusof; Rehan Shuhada Binti Abu Bakar; Selvanesan A/L Sengol; W.Nur Afiza Binti W. Ariffin; Yu Kie A/P Chem
EPI_ISL_10512214, EPI_ISL_10512215, EPI_ISL_10512216, EPI_ISL_10512217, EPI_ISL_10512218, EPI_ISL_10512219, EPI_ISL_10512220, EPI_ISL_10512221, EPI_ISL_10512222, EPI_ISL_10512223, EPI_ISL_10512224, EPI_ISL_10512225, EPI_ISL_10512226, EPI_ISL_10512227, EPI_ISL_10512228, EPI_ISL_10512229, EPI_ISL_10512230, EPI_ISL_10512231, EPI_ISL_10512232, EPI_ISL_10512233, EPI_ISL_10512234, EPI_ISL_10512235, EPI_ISL_10512236, EPI_ISL_10512237, EPI_ISL_10512238, EPI_ISL_10512239, EPI_ISL_10512240			
see above	Normah Medical Specialist Centre, Kuching	Institute of Health and Community Medicine	Chan Chia Jui; Chua Hock Hin; David Perera; Ikwannuddin Y; Ooi Mong How; Tonnii Sia Loong Loong
EPI_ISL_12055326, EPI_ISL_12055344	PKD TAMPIN	Institut Biologi Molekul Perubatan UKM (UMBI)	Khairun Nur Abd Ghafar; Mira Farzana Mohamad Mokhtar; Muhiddin Ishak; Nor Azila Muhammad Azami; Nur Alyaa Afifah Md Shahri; Nurul Syakima Ab Mutalib; Rahman Jamal; Ryia Illani Mohd Yunos; Siti Nur Hasanah Mohd Yusuf; Zahirrah Begam Mohamed Rasheed
EPI_ISL_10880069	PKD TANGKAK	UKM Medical Molecular Biology Institute (UMBI)	Khairun Nur Abd Ghafar; Mira Farzana Mohamad Mokhtar; Muhiddin Ishak; Nor Azila Muhammad Azami; Nur Alyaa Afifah Md Shahri; Nurul Syakima Ab Mutalib; Rahman Jamal; Ryia Illani Mohd Yunos; Siti Nur Hasanah Mohd Yusuf; Zahirrah Begam Mohamed Rasheed
EPI_ISL_12746946, EPI_ISL_12746947	PUBLIC HEALTH UNIT	Institute for Medical Research, Infectious Disease Research Centre, National Institutes of Health, Ministry of Health Malaysia	Ahmad FA; Ahmad Fazilah NA; Anasir MI; Azizan MA; Kamel K; Mohamad Sukri MZ; Norhisham SN; Ramly N; Robert F; Rosli NR; Suppiah J; Thayan R
EPI_ISL_12321874, EPI_ISL_12321875, EPI_ISL_12321876, EPI_ISL_12400590	PUTRAJAYA HOSPITAL	Institute for Medical Research, Infectious Disease Research Centre, National Institutes of Health, Ministry of Health Malaysia	Ahmad FA; Ahmad Fazilah NA; Anasir MI; Azizan MA; Kamel K; Mohamad Sukri MZ; Norhisham SN; Ramly N; Robert F; Rosli NR; Suppiah J; Thayan R
EPI_ISL_11965259, EPI_ISL_11965260	Port Klang Health Clinic	Institute for Medical Research, Infectious Disease Research Centre, National Institutes of Health, Ministry of Health Malaysia	Ahmad FA; Ahmad Fazilah NA; Anasir MI; Azizan MA; Kamel K; Mohamad Sukri MZ; Norhisham SN; Ramly N; Robert F; Rosli NR; Suppiah J; Thayan R
EPI_ISL_11976279, EPI_ISL_12746944, EPI_ISL_12746983, EPI_ISL_12899663	QUEEN ELIZABETH HOSPITAL	Institute for Medical Research, Infectious Disease Research Centre, National Institutes of Health, Ministry of Health Malaysia	Ahmad FA; Ahmad Fazilah NA; Anasir MI; Azizan MA; Kamel K; Mohamad Sukri MZ; Norhisham SN; Ramly N; Robert F; Rosli NR; Suppiah J; Thayan R
EPI_ISL_11685210, EPI_ISL_11685211, EPI_ISL_11685218, EPI_ISL_11685219, EPI_ISL_11965278, EPI_ISL_11965285, EPI_ISL_11965286, EPI_ISL_11965287, EPI_ISL_11965288, EPI_ISL_11965289, EPI_ISL_11965290, EPI_ISL_11965291, EPI_ISL_11965293, EPI_ISL_11965294, EPI_ISL_11965295, EPI_ISL_11965296, EPI_ISL_11965297, EPI_ISL_11965298, EPI_ISL_11965299, EPI_ISL_11965300, EPI_ISL_11965301, EPI_ISL_11965302, EPI_ISL_11965303, EPI_ISL_12097430, EPI_ISL_12097431, EPI_ISL_12097432			
see above	Queen Elizabeth Hospital	Institute for Medical Research, Infectious Disease Research Centre, National Institutes of Health, Ministry of Health Malaysia	Ahmad FA; Ahmad Fazilah NA; Anasir MI; Azizan MA; G. Adypatti NM; Jamaluddin MS; Kamel K; Mohamad Sukri MZ; Norhisham SN; Ramly N; Robert F; Rosli NR; Suppiah J; Thayan R
EPI_ISL_11105875, EPI_ISL_11105876, EPI_ISL_11105877, EPI_ISL_11105883, EPI_ISL_11105884, EPI_ISL_11105885, EPI_ISL_11105886, EPI_ISL_11105887, EPI_ISL_11105888			
see above	Queen Elizabeth Hospital, Kota Kinabalu	Institute for Medical Research, Infectious Disease Research Centre, National Institutes of Health, Ministry of Health Malaysia	Ahmad FA; Ahmad Fazilah NA; Anasir MI; Azizan MA; G. Adypatti NM; Jamaluddin MS; Kamel K; Mohamad Sukri MZ; Norhisham SN; Ramly N; Robert F; Rosli NR; Suppiah J; Thayan R
EPI_ISL_11105874	Queen Elizabeth Hospital, Kota Kinabalu sabah Malaysia	Institute for Medical Research, Infectious Disease Research Centre, National Institutes of Health, Ministry of Health Malaysia	Ahmad FA; Ahmad Fazilah NA; Anasir MI; Azizan MA; G. Adypatti NM; Jamaluddin MS; Kamel K; Mohamad Sukri MZ; Norhisham SN; Ramly N; Robert F; Rosli NR; Suppiah J; Thayan R

EPI_ISL_11332677, EPI_ISL_11332727, EPI_ISL_11332728	Raja Perempuan Zainab II Hospital, Kota Bharu	Tropical Infectious Diseases Research & Education Centre (TIDREC), Universiti Malaya	AsmaAnati CheMatSeri; Che-Norainon Yaacob; Jia-Yi Tan; Jo-Ern Wong; Kim-Kee Tan; Mulya-Mustika-Sari Zulkifli; Noor Syahida Azizan; Nur-Hidayana Mahfodz; Sazaly AbuBakar; Siti-Sarah Nor'e; Wei-Wei Aw
EPI_ISL_10512374, EPI_ISL_10512375, EPI_ISL_10512376, EPI_ISL_10512377, EPI_ISL_10512378, EPI_ISL_10512379, EPI_ISL_10512380, EPI_ISL_10512381, EPI_ISL_10512382, EPI_ISL_10512383, EPI_ISL_10512384, EPI_ISL_10512385, EPI_ISL_10512386, EPI_ISL_10512387, EPI_ISL_10512388, EPI_ISL_10512389, EPI_ISL_10512390, EPI_ISL_10512391, EPI_ISL_11332475, EPI_ISL_12739609, EPI_ISL_12739610, EPI_ISL_12739611, EPI_ISL_12739612, EPI_ISL_12739613, EPI_ISL_12739614, EPI_ISL_12739615, EPI_ISL_12739616			
see above	Rejang Medical Centre (Sibu)	Institute of Health and Community Medicine	Chan Chia Jui; Chua Hock Hin; David Perera; Ooi Mong How; Tonnii Sia Loong Loong; Wong Jyn Shan
EPI_ISL_9804140, EPI_ISL_10512191, EPI_ISL_10512192, EPI_ISL_10512193, EPI_ISL_10512194, EPI_ISL_10512196, EPI_ISL_10512197, EPI_ISL_10512198, EPI_ISL_10512199, EPI_ISL_10512200, EPI_ISL_10512202, EPI_ISL_10512203, EPI_ISL_10512204, EPI_ISL_10512241, EPI_ISL_10512242, EPI_ISL_10512243, EPI_ISL_10512244, EPI_ISL_10512245, EPI_ISL_10512246, EPI_ISL_10512247, EPI_ISL_10512248, EPI_ISL_10512249, EPI_ISL_10512250, EPI_ISL_10512251, EPI_ISL_10512252, EPI_ISL_10512323			
see above	Sarawak General Hospital (Kuching)	Institute of Health and Community Medicine	Chan Chia Jui; Chua Hock Hin; David Perera; Ooi Mong How; Tonnii Sia Loong Loong
EPI_ISL_10512301, EPI_ISL_10512317	Sarawak Heart Center, Samarahan	Institute of Health and Community Medicine	Chan Chia Jui; Chua Hock Hin; David Perera; Ooi Mong How; Tonnii Sia Loong Loong
EPI_ISL_10981891	Selayang Hospital	Institute for Medical Research, Infectious Disease Research Centre, National Institutes of Health, Ministry of Health Malaysia	Ahmad FA; Ahmad Fazilah NA; Anasir MI; Azizan MA; Kamel K; Mohamad Sukri MZ; Norhisham SN; Ramly N; Robert F; Rosli NR; Suppiah J; Thayan R
EPI_ISL_10512182, EPI_ISL_10512195, EPI_ISL_10512201, EPI_ISL_10512209, EPI_ISL_10512253, EPI_ISL_10512254, EPI_ISL_10512266, EPI_ISL_10512499, EPI_ISL_10512500, EPI_ISL_10512501, EPI_ISL_10512503, EPI_ISL_10512504, EPI_ISL_10512506			
see above	Sibu Hospital (Sibu, Sarawak)	Institute of Health and Community Medicine	Chan Chia Jui; Chua Hock Hin; David Perera; Ooi Mong How; Tonnii Sia Loong Loong
EPI_ISL_11965257, EPI_ISL_11965258	Sijangkang Health Clinic	Institute for Medical Research, Infectious Disease Research Centre, National Institutes of Health, Ministry of Health Malaysia	Ahmad FA; Ahmad Fazilah NA; Anasir MI; Azizan MA; Kamel K; Mohamad Sukri MZ; Norhisham SN; Ramly N; Robert F; Rosli NR; Suppiah J; Thayan R
EPI_ISL_11965261, EPI_ISL_11965264, EPI_ISL_12097421	Tanjung Sepat Health Clinic	Institute for Medical Research, Infectious Disease Research Centre, National Institutes of Health, Ministry of Health Malaysia	Ahmad FA; Ahmad Fazilah NA; Anasir MI; Azizan MA; Kamel K; Mohamad Sukri MZ; Norhisham SN; Ramly N; Robert F; Rosli NR; Suppiah J; Thayan R
EPI_ISL_11965292	Tawau Hospital	Institute for Medical Research, Infectious Disease Research Centre, National Institutes of Health, Ministry of Health Malaysia	Ahmad FA; Ahmad Fazilah NA; Anasir MI; Azizan MA; Kamel K; Mohamad Sukri MZ; Norhisham SN; Ramly N; Robert F; Rosli NR; Suppiah J; Thayan R
EPI_ISL_11965263	Telok Datok Health Clinic	Institute for Medical Research, Infectious Disease Research Centre, National Institutes of Health, Ministry of Health Malaysia	Ahmad FA; Ahmad Fazilah NA; Anasir MI; Azizan MA; Kamel K; Mohamad Sukri MZ; Norhisham SN; Ramly N; Robert F; Rosli NR; Suppiah J; Thayan R
EPI_ISL_11965262, EPI_ISL_11965266	Telok Panglima Garang Health Clinic	Institute for Medical Research, Infectious Disease Research Centre, National Institutes of Health, Ministry of Health Malaysia	Ahmad FA; Ahmad Fazilah NA; Anasir MI; Azizan MA; Kamel K; Mohamad Sukri MZ; Norhisham SN; Ramly N; Robert F; Rosli NR; Suppiah J; Thayan R
EPI_ISL_12135909	UKKP HSB	Tropical Infectious Diseases Research & Education Centre (TIDREC), Universiti Malaya	AsmaAnati CheMatSeri; Che-Norainon Yaacob; Jia-Yi Tan; Jo-Ern Wong; Kim-Kee Tan; Mulya-Mustika-Sari Zulkifli; Noor Syahida Azizan; Nur-Hidayana Mahfodz; Sazaly AbuBakar; Siti-Sarah Nor'e; Wei-Wei Aw
EPI_ISL_12135939, EPI_ISL_12135940	UKKP Hospital Sultanah Bahiyah	Tropical Infectious Diseases Research & Education Centre (TIDREC), Universiti Malaya	AsmaAnati CheMatSeri; Che-Norainon Yaacob; Jia-Yi Tan; Jo-Ern Wong; Kim-Kee Tan; Mulya-Mustika-Sari Zulkifli; Noor Syahida Azizan; Nur-Hidayana Mahfodz; Sazaly AbuBakar; Siti-Sarah Nor'e; Wei-Wei Aw
EPI_ISL_12135907	UKKP Hospital sultanah bahiyah	Tropical Infectious Diseases Research & Education Centre (TIDREC), Universiti Malaya	AsmaAnati CheMatSeri; Che-Norainon Yaacob; Jia-Yi Tan; Jo-Ern Wong; Kim-Kee Tan; Mulya-Mustika-Sari Zulkifli; Noor Syahida Azizan; Nur-Hidayana Mahfodz; Sazaly AbuBakar; Siti-Sarah Nor'e; Wei-Wei Aw
EPI_ISL_12135906, EPI_ISL_12135908	UKKP Hospital sutanah Bahiyah	Tropical Infectious Diseases Research & Education Centre (TIDREC), Universiti Malaya	AsmaAnati CheMatSeri; Che-Norainon Yaacob; Jia-Yi Tan; Jo-Ern Wong; Kim-Kee Tan; Mulya-Mustika-Sari Zulkifli; Noor Syahida Azizan; Nur-Hidayana Mahfodz; Sazaly AbuBakar; Siti-Sarah Nor'e; Wei-Wei Aw
EPI_ISL_11754711, EPI_ISL_11754757, EPI_ISL_11754771	UMMC	Department of Medical Microbiology, Faculty of Medicine, University of Malaya; University of Malaya Medical Centre	I-Ching SAM; Jolene Yin Ling FU; Omar Khalilur Rahman; Yoke Fun Chan
EPI_ISL_10942283, EPI_ISL_10942288	UNIT KESIHATAN AWAM	UKM Medical Molecular Biology Institute (UMBI)	Khairun Nur Abd Ghafar; Mira Farzana Mohamad Mokhtar; Muhiddin Ishak; Nor Azila Muhammad Azami; Nur Alyaa Afifah Md Shahri; Nurul Syakima Ab Mutalib; Rahman Jamal; Ryia Illani Mohd Yunos; Siti Nur Hasanah Mohd Yusuf; Zahirrah Begam Mohamed Rasheed
EPI_ISL_12008435, EPI_ISL_12008485, EPI_ISL_12008486	UNIT KESIHATAN AWAM HEBHK	UKM Medical Molecular Biology Institute (UMBI)	Khairun Nur Abd Ghafar; Mira Farzana Mohamad Mokhtar; Muhiddin Ishak; Nor Azila Muhammad Azami; Nur Alyaa Afifah Md Shahri; Nurul Syakima Ab Mutalib; Rahman Jamal; Ryia Illani Mohd Yunos; Siti Nur Hasanah Mohd Yusuf; Zahirrah Begam Mohamed Rasheed

We gratefully acknowledge the following Authors from the Originating laboratories responsible for obtaining the specimens, as well as the Submitting laboratories where the genome data were generated and shared via GISAID, on which this research is based.

All Submitters of data may be contacted directly via www.gisaid.org

Authors are sorted alphabetically.

Acknowledgement EPI_SET Identifier: EPI_SET_20220603mu

Accession ID	Originating Laboratory	Submitting Laboratory	Authors
EPI_ISL_10981863, EPI_ISL_10981870, EPI_ISL_10981875, EPI_ISL_10981882, EPI_ISL_10981890, EPI_ISL_10981896	Ampang Hospital	Institute for Medical Research, Infectious Disease Research Centre, National Institutes of Health, Ministry of Health Malaysia	Ahmad FA; Ahmad Fazilah NA; Anasir MI; Azizan MA; Kamel K; Mohamad Sukri MZ; Norhisham SN; Ramly N; Robert F; Rosli NR; Suppiah J; Thayan R
EPI_ISL_10512302, EPI_ISL_10512345, EPI_ISL_10512349, EPI_ISL_11332472, EPI_ISL_11332473	Bintulu Hospital PCR Lab, Bintulu	Institute of Health and Community Medicine	Chan Chia Jui; Chua Hock Hin; David Perera; Ooi Mong How; Tan Lee See; Tonnii Sia Loong Loong; Wong Jyn Shan
EPI_ISL_10512419, EPI_ISL_10512420, EPI_ISL_10512421, EPI_ISL_10512422, EPI_ISL_10512423, EPI_ISL_10512424, EPI_ISL_10512425, EPI_ISL_10512426, EPI_ISL_10512427, EPI_ISL_10760032, EPI_ISL_10760034			
see above	Bintulu Medical Centre (Bintulu)	Institute of Health and Community Medicine	Chan Chia Jui; Chua Hock Hin; David Perera; Ooi Mong How; Tonnii Sia Loong Loong; Wong Jyn Shan
EPI_ISL_10512340, EPI_ISL_10512412, EPI_ISL_10512413, EPI_ISL_10512414	Borneo Medical Centre (Kuching)	Institute of Health and Community Medicine	Chan Chia Jui; Chua Hock Hin; David Perera; Ooi Mong How; Tonnii Sia Loong Loong; Wong Jyn Shan
EPI_ISL_10759888, EPI_ISL_10760036, EPI_ISL_10760039, EPI_ISL_10760048	Borneo Medical Centre (Miri)	Institute of Health and Community Medicine	Chan Chia Jui; Chua Hock Hin; David Perera; Ooi Mong How; Tonnii Sia Loong Loong; Wong Jyn Shan
EPI_ISL_11965267, EPI_ISL_11965268, EPI_ISL_12097423, EPI_ISL_12097425	Bukit Changgang Health Clinic	Institute for Medical Research, Infectious Disease Research Centre, National Institutes of Health, Ministry of Health Malaysia	Ahmad FA; Ahmad Fazilah NA; Anasir MI; Azizan MA; Kamel K; Mohamad Sukri MZ; Norhisham SN; Ramly N; Robert F; Rosli NR; Suppiah J; Thayan R
EPI_ISL_12097427	Cheras District Health Office	Institute for Medical Research, Infectious Disease Research Centre, National Institutes of Health, Ministry of Health Malaysia	Ahmad FA; Ahmad Fazilah NA; Anasir MI; Azizan MA; Kamel K; Mohamad Sukri MZ; Norhisham SN; Ramly N; Robert F; Rosli NR; Suppiah J; Thayan R
EPI_ISL_13047116	DEPARTMENT OF MEDICAL MICROBIOLOGY AND PARASITOLOGY, HOSPITAL UNIVERSITI SAINS MALAYSIA	MOLECULAR RESEARCH LABORATORY	Abdul Haris bin Muhammad; Ahmad Sukari Bin Halim; Alexander Chong Shu Chien; Azian Harun; Chan Yean Yean; Chua Wei Chuan; Farahana binti Mohamed; Kirnpal Kaur Banga Singh; Lau Nyok Sean; Lee Lih Huey; Lim Shu Yong; Maizun binti Mohd Zain; Mohd Nadzri Abu Yazid; Mohd Zulkifli Salleh; Muhammad Azamuddeen bin Mohammad Nasir; Muhammad Fazli bin Khalid; Muhammad Nashrul Farhan Samsudin; Muhammad Zarul Hanifah Bin Md Zoqratt; Nik Zuraina binti Nik Mohd Noor; Noor Hafizan binti Mat Salleh; Nurfadhlina Musa; Qasim Ayub; Rosline Hassan; Sadequr Rahman; Syahida binti Omar; Wan Mohd Zahiruddin Wan Mohammad; Wardah Yusof; Zaini bin Hussin; Zakuan Zainy Deris
EPI_ISL_12704540, EPI_ISL_12704541	Department of Medical Microbiology and Parasitology, Hospital Universiti Sains Malaysia	Molecular Research Laboratory	Abdul Haris bin Muhammad; Ahmad Sukari Bin Halim; Alexander Chong Shu Chien; Asraihan Che Abdul Malik; Azian Harun; Chan Yean Yean; Chua Wei Chuan; Farahana binti Mohamed; Kirnpal Kaur Banga Singh; Lau Nyok Sean; Lee Lih Huey; Lim Shu Yong; Maizun binti Mohd Zain; Mera Edora Binti Abdul Manap; Mohd Iman hafiz Bin Ibrahim; Mohd Nadzri Abu Yazid; Mohd Zulkifli Salleh; Muhamad Khairul Amirin Bin Zulkifli; Muhammad Azamuddeen bin Mohammad Nasir; Muhammad Fazli bin Khalid; Muhammad Nashrul Farhan Samsudin; Muhammad Zarul Hanifah Bin Md Zoqratt; Nik Zuraina binti Nik Mohd Noor; Noor Hafizan binti Mat Salleh; Nor Amizara Binti Azami; Nor Suhana Binti Mohd Satar; Nur Syuhada Binti Abdul Rahim; Nur-Leem Binti Murshid; Nurfadhlina Musa; Qasim Ayub; Rosline Hassan; Sadequr Rahman; Siti Nur Aisyah Binti Mohamad Sham; Syahida binti Omar; Wan Mohd Zahiruddin Wan Mohammad; Wardah Yusof; Zaini bin Hussin; Zakuan Zainy Deris
EPI_ISL_11344869	Department of Medical Microbiology and Parasitology, School of Medical Sciences, Universiti Sains Malaysia Health Campus	Molecular Research Laboratory, Department of Medical Microbiology and Parasitology, School of Medical Sciences, Universiti Sains Malaysia Health Campus	Abdul Haris bin Muhammad; Ahmad Sukari Bin Halim; Alexander Chong Shu Chien; Asraihan Che Abdul Malik; Azian Harun; Chan Yean Yean; Chua Wei Chuan; Farahana binti Mohamed; Kirnpal Kaur Banga Singh; Lau Nyok Sean; Lee Lih Huey; Lim Shu Yong; Maizun binti Mohd Zain; Mera Edora Binti Abdul Manap; Mohd Iman hafiz Bin Ibrahim; Mohd Nadzri Abu Yazid; Mohd Zulkifli Salleh; Muhamad Khairul Amirin Bin Zulkifli; Muhammad Azamuddeen bin Mohammad Nasir; Muhammad Fazli bin Khalid; Muhammad Nashrul Farhan Samsudin; Muhammad Zarul Hanifah Bin Md Zoqratt; Nik Zuraina binti Nik Mohd Noor; Noor Hafizan binti Mat Salleh; Nor Amizara Binti Azami; Nor Suhana Binti Mohd Satar; Nur Syuhada Binti Abdul Rahim; Nur-Leem Binti Murshid; Nurfadhlina Musa; Qasim Ayub; Rosline Hassan; Sadequr Rahman; Siti Nur Aisyah Binti Mohamad Sham; Syahida binti Omar; Wan Mohd Zahiruddin Wan Mohammad; Wardah Yusof; Zaini bin Hussin; Zakuan Zainy Deris
EPI_ISL_12721582, EPI_ISL_12721583, EPI_ISL_12721584, EPI_ISL_12721585, EPI_ISL_12721586, EPI_ISL_12721587	Department of Medical Microbiology, Hospital Pengajar Universiti Putra Malaysia	Department of Medical Microbiology, Hospital Pengajar Universiti Putra Malaysia	Afiqah Adzmi; Amiza Azmi; Azmiza Syawani Jasni; Hui Yee Chee; Leslie Thian Lung Than; Muadz Mohtar; Muhammad Mohd Isa; Narcisse MS Joseph; Niazlin Mohd Taib; Noor Hazirah Noor Azhari; Norlaila; Nur Raihana Ithnin; Nurul Huda Mohamed Rashidi; Nurul Nadiah Ismail; Rosni Ibrahim; Rukman Awang Hamat; Sallehhudin; Siti Norbaya Masri; Siti Zulaikha Zakariah; Syafinaz Amin Nordin; Tengku Zetty Maztura Tengku Jamaluddin; Zamberi Sekawi
EPI_ISL_12097433	Duchess of Kent Hospital	Institute for Medical Research, Infectious Disease Research Centre, National Institutes of Health, Ministry of Health Malaysia	Ahmad FA; Ahmad Fazilah NA; Anasir MI; Azizan MA; Kamel K; Mohamad Sukri MZ; Norhisham SN; Ramly N; Robert F; Rosli NR; Suppiah J; Thayan R
EPI_ISL_13047119, EPI_ISL_13047120	GUNONG HEALTH CLINIC	MOLECULAR RESEARCH LABORATORY	Abdul Haris bin Muhammad; Ahmad Sukari Bin Halim; Alexander Chong Shu Chien; Azian Harun; Chan Yean Yean; Chua Wei Chuan; Farahana binti Mohamed; Kirnpal Kaur Banga Singh; Lau Nyok Sean; Lee Lih Huey; Lim Shu Yong; Maizun binti Mohd Zain; Mohd Nadzri Abu Yazid; Mohd Zulkifli Salleh; Muhammad Azamuddeen bin Mohammad Nasir; Muhammad Fazli bin Khalid; Muhammad Nashrul Farhan Samsudin; Muhammad Zarul Hanifah Bin Md Zoqratt; Nik Zuraina binti Nik Mohd Noor; Noor Hafizan binti Mat Salleh; Nurfadhlina Musa; Qasim Ayub; Rosline Hassan; Sadequr Rahman; Syahida binti Omar; Wan Mohd Zahiruddin Wan Mohammad; Wardah Yusof; Zaini bin Hussin; Zakuan Zainy Deris
EPI_ISL_10512341, EPI_ISL_10512342, EPI_ISL_10759883, EPI_ISL_10759884, EPI_ISL_10759885, EPI_ISL_10759886, EPI_ISL_10759887			
see above	Gribbles Diagnostic Lab @Columbia Asia Hospital, Miri	Institute of Health and Community Medicine	Affif bin Zainal Abidin; Chan Chia Jui; Chua Hock Hin; David Perera; Ooi Mong How; Tonnii Sia Loong Loong
EPI_ISL_11332461, EPI_ISL_11332462, EPI_ISL_11332463	Gribbles Diagnostic Lab @Timberland Medical Centre, Kuching	Institute of Health and Community Medicine	Chan Chia Jui; Chua Hock Hin; David Perera; Liew Chin Chin J; Ooi Mong How; Tonnii Sia Loong Loong; Wong Jyn Shan
EPI_ISL_10512300, EPI_ISL_10512303, EPI_ISL_10512304, EPI_ISL_10512305, EPI_ISL_10512306, EPI_ISL_10512307, EPI_ISL_10512308, EPI_ISL_10512309, EPI_ISL_10512310, EPI_ISL_10512311, EPI_ISL_10512312, EPI_ISL_10512313, EPI_ISL_10512314, EPI_ISL_10512315			
see above	Gribbles Pathology, Kuching	Institute of Health and Community Medicine	Chan Chia Jui; Chua Hock Hin; David Perera; Liew Chin Chin J; Ooi Mong How; Tonnii Sia Loong Loong
EPI_ISL_12008480	HJTS	UKM Medical Molecular Biology Institute (UMBI)	Khairun Nur Abd Ghafar; Mira Farzana Mohamad Mokhtar; Muhiddin Ishak; Nor Azila Muhammad Azami; Nur Alyaa Afifah Md Shahri; Nurul Syakima Ab Mutalib; Rahman Jamal; Ryia Illani Mohd Yunos; Siti Nur Hasanah Mohd Yusuf; Zahirrah Begam Mohamed Rasheed
EPI_ISL_12771272	HOSPITAL BALIK PULAU	iPROMISE, UiTM	Ariza Adnan; Fadzilah Mohd Nor; Lim Wai Feng; Mohd Asif Mohd Sukri; Mohd Nur Fakhruzzaman Noorizhab; Mohd Zaki Salleh; Sazzli Shahlan Kassim; Siti Farah Alwani Mohd Nawi; Siti Hamimah Sheikh Abdul Kadir; Teh Lay Kek; Wang Seok Mui
EPI_ISL_12135924, EPI_ISL_12135925, EPI_ISL_12135926, EPI_ISL_12135927	HOSPITAL RAJA PERMAISURI BAINUN	Tropical Infectious Diseases Research & Education Centre (TIDREC), Universiti Malaya	AsmaAnati CheMatSeri; Che-Norainon Yaacob; Jia-Yi Tan; Jo-Ern Wong; Kim-Kee Tan; Mulya-Mustika-Sari Zulkifli; Noor Syahida Azizan; Nur-Hidayana Mahfodz; Sazaly AbuBakar; Siti-Sarah Nor'e; Wei-Wei Aw
EPI_ISL_12135928	HOSPITAL RAJA PERMAISURI BAINUN,IPOH	Tropical Infectious Diseases Research & Education Centre (TIDREC), Universiti Malaya	AsmaAnati CheMatSeri; Che-Norainon Yaacob; Jia-Yi Tan; Jo-Ern Wong; Kim-Kee Tan; Mulya-Mustika-Sari Zulkifli; Noor Syahida Azizan; Nur-Hidayana Mahfodz; Sazaly AbuBakar; Siti-Sarah Nor'e; Wei-Wei Aw
EPI_ISL_12135960, EPI_ISL_12135961	HOSPITAL SULTANAH NUR ZAHIRAH	Tropical Infectious Diseases Research & Education Centre (TIDREC), Universiti Malaya	AsmaAnati CheMatSeri; Che-Norainon Yaacob; Jia-Yi Tan; Jo-Ern Wong; Kim-Kee Tan; Mulya-Mustika-Sari Zulkifli; Noor Syahida Azizan; Nur-Hidayana Mahfodz; Sazaly AbuBakar; Siti-Sarah Nor'e; Wei-Wei Aw
EPI_ISL_11939177, EPI_ISL_11939178, EPI_ISL_12771270, EPI_ISL_12771282, EPI_ISL_12771283	HPP	iPROMISE, UiTM	Ariza Adnan; Fadzilah Mohd Nor; Lim Wai Feng; Mohd Asif Mohd Sukri; Mohd Nur Fakhruzzaman Noorizhab; Mohd Zaki Salleh; Sazzli Shahlan Kassim; Siti Farah Alwani Mohd Nawi; Siti Hamimah Sheikh Abdul Kadir; Teh Lay Kek; Wang Seok Mui
EPI_ISL_12008436, EPI_ISL_12008444, EPI_ISL_12008445, EPI_ISL_12008450, EPI_ISL_12008451, EPI_ISL_12008452, EPI_ISL_12008474, EPI_ISL_12008478, EPI_ISL_12008482, EPI_ISL_12008496, EPI_ISL_12008500, EPI_ISL_12008514			
see above	HPSF	UKM Medical Molecular Biology Institute (UMBI)	Khairun Nur Abd Ghafar; Mira Farzana Mohamad Mokhtar; Muhiddin Ishak; Nor Azila Muhammad Azami; Nur Alyaa Afifah Md Shahri; Nurul Syakima Ab Mutalib; Rahman Jamal; Ryia Illani Mohd Yunos; Siti Nur Hasanah Mohd Yusuf; Zahirrah Begam Mohamed Rasheed
EPI_ISL_12055333, EPI_ISL_12055340, EPI_ISL_12055341, EPI_ISL_12055369,	HPSF MUAR	Institut Biologi Molekul Perubatan UKM (UMBI)	Khairun Nur Abd Ghafar; Mira Farzana Mohamad Mokhtar; Muhiddin Ishak; Nor Azila Muhammad Azami; Nur Alyaa Afifah Md Shahri; Nurul Syakima Ab Mutalib; Rahman Jamal; Ryia Illani Mohd Yunos; Siti Nur Hasanah Mohd Yusuf; Zahirrah Begam Mohamed Rasheed

EPI_ISL_12055376, EPI_ISL_12055382			
EPI_ISL_12135914, EPI_ISL_12135930, EPI_ISL_12135931, EPI_ISL_12135932, EPI_ISL_12135933, EPI_ISL_12135934, EPI_ISL_12135935, EPI_ISL_12135936, EPI_ISL_12135937, EPI_ISL_12135938			
see above	HRPZ II	Tropical Infectious Diseases Research & Education Centre (TIDREC), Universiti Malaya	AsmaAnati CheMatSeri; Che-Norainon Yaacob; Jia-Yi Tan; Jo-Ern Wong; Kim-Kee Tan; Mulya-Mustika-Sari Zulkifli; Noor Syahida Azizan; Nur-Hidayana Mahfodz; Sazaly AbuBakar; Siti-Sarah Nor'e; Wei-Wei Aw
EPI_ISL_12008434, EPI_ISL_12008437, EPI_ISL_12008483, EPI_ISL_12008513, EPI_ISL_12008523, EPI_ISL_12099530	HSAJB	UKM Medical Molecular Biology Institute (UMBI)	Khairun Nur Abd Ghafar; Mira Farzana Mohamad Mokhtar; Muhiddin Ishak; Nor Azila Muhammad Azami; Nur Alyaa Afifah Md Shahri; Nurul Syakima Ab Mutalib; Rahman Jamal; Ryia Illani Mohd Yunos; Siti Nur Hasanah Mohd Yusuf; Zahirrah Begam Mohamed Rasheed
EPI_ISL_10880083, EPI_ISL_10880093, EPI_ISL_10880106, EPI_ISL_10880110, EPI_ISL_12008460, EPI_ISL_12008461, EPI_ISL_12008462, EPI_ISL_12008517			
see above	HTJS	UKM Medical Molecular Biology Institute (UMBI)	Khairun Nur Abd Ghafar; Mira Farzana Mohamad Mokhtar; Muhiddin Ishak; Nor Azila Muhammad Azami; Nur Alyaa Afifah Md Shahri; Nurul Syakima Ab Mutalib; Rahman Jamal; Ryia Illani Mohd Yunos; Siti Nur Hasanah Mohd Yusuf; Zahirrah Begam Mohamed Rasheed
EPI_ISL_12277168, EPI_ISL_12277169, EPI_ISL_12277170, EPI_ISL_12277171, EPI_ISL_12277172, EPI_ISL_12277173, EPI_ISL_12277174, EPI_ISL_12277208, EPI_ISL_12277209, EPI_ISL_12277213, EPI_ISL_12277214, EPI_ISL_12277215, EPI_ISL_12277216, EPI_ISL_12277217, EPI_ISL_12277218, EPI_ISL_12277219, EPI_ISL_12277220, EPI_ISL_12277221, EPI_ISL_12277222			
see above	Hospital Ampang	Malaysia Genome and Vaccine Institute	Azrin Ahmad; Enizza Kasim; Irni Suhayu Sapian; Mohd Faizal Abu Bakar; Mohd Ghows Mohd Azzam.; Mohd Noor Mat Isa; Nor Azfa Johari; Nurhezreen Md Iqbal; Shamsidar Sopie; Siti Noraini Othman; Yusuf Muhammad Noor
EPI_ISL_12277210, EPI_ISL_12277211, EPI_ISL_12277212	Hospital Kuala Kubu Bharu	Malaysia Genome and Vaccine Institute	Azrin Ahmad; Enizza Kasim; Irni Suhayu Sapian; Mohd Faizal Abu Bakar; Mohd Ghows Mohd Azzam.; Mohd Noor Mat Isa; Nor Azfa Johari; Nurhezreen Md Iqbal; Shamsidar Sopie; Siti Noraini Othman; Yusuf Muhammad Noor
EPI_ISL_11939166, EPI_ISL_11939168, EPI_ISL_12771273, EPI_ISL_12771274	Hospital Pulau Pinang	iPROMISE, UiTM	Ariza Adnan; Fadzilah Mohd Nor; Lim Wai Feng; Mohd Asif Mohd Sukri; Mohd Nur Fakhruzzaman Noorizhab; Mohd Zaki Salleh; Sazzli Shahlan Kassim; Siti Farah Alwani Mohd Nawi; Siti Hamimah Sheikh Abdul Kadir; Teh Lay Kek; Wang Seok Mui
EPI_ISL_12135929	Hospital Raja Perempuan Zainab II	Tropical Infectious Diseases Research & Education Centre (TIDREC), Universiti Malaya	AsmaAnati CheMatSeri; Che-Norainon Yaacob; Jia-Yi Tan; Jo-Ern Wong; Kim-Kee Tan; Mulya-Mustika-Sari Zulkifli; Noor Syahida Azizan; Nur-Hidayana Mahfodz; Sazaly AbuBakar; Siti-Sarah Nor'e; Wei-Wei Aw
EPI_ISL_11900059	Hospital Selayang	Malaysia Genome and Vaccine Institute	Azrin Ahmad; Enizza Kasim; Irni Suhayu Sapian; Mohd Faizal Abu Bakar; Mohd Ghows Mohd Azzam.; Mohd Noor Mat Isa; Nor Azfa Johari; Nurhezreen Md Iqbal; Shamsidar Sopie; Siti Noraini Othman; Yusuf Muhammad Noor
EPI_ISL_12135962, EPI_ISL_12135963	Hospital Sultanah Nur Zahirah	Tropical Infectious Diseases Research & Education Centre (TIDREC), Universiti Malaya	AsmaAnati CheMatSeri; Che-Norainon Yaacob; Jia-Yi Tan; Jo-Ern Wong; Kim-Kee Tan; Mulya-Mustika-Sari Zulkifli; Noor Syahida Azizan; Nur-Hidayana Mahfodz; Sazaly AbuBakar; Siti-Sarah Nor'e; Wei-Wei Aw
EPI_ISL_11323793, EPI_ISL_11323837	Institut Biologi Molekul Perubatan UKM (UMBI)	UKM Medical Molecular Biology Institute (UMBI)	Khairun Nur Abd Ghafar; Mira Farzana Mohamad Mokhtar; Muhiddin Ishak; Nor Azila Muhammad Azami; Nur Alyaa Afifah Md Shahri; Nurul Syakima Ab Mutalib; Rahman Jamal; Ryia Illani Mohd Yunos; Siti Nur Hasanah Mohd Yusuf; Zahirrah Begam Mohamed Rasheed
EPI_ISL_11900028	Institut Kanser Negara	Malaysia Genome and Vaccine Institute	Azrin Ahmad; Enizza Kasim; Irni Suhayu Sapian; Mohd Faizal Abu Bakar; Mohd Ghows Mohd Azzam.; Mohd Noor Mat Isa; Nor Azfa Johari; Nurhezreen Md Iqbal; Shamsidar Sopie; Siti Noraini Othman; Yusuf Muhammad Noor
EPI_ISL_11939149, EPI_ISL_11939195, EPI_ISL_11939198, EPI_ISL_11939204	JKN PAHANG	iPROMISE, UiTM	Ariza Adnan; Fadzilah Mohd Nor; Lim Wai Feng; Mohd Asif Mohd Sukri; Mohd Nur Fakhruzzaman Noorizhab; Mohd Zaki Salleh; Sazzli Shahlan Kassim; Siti Farah Alwani Mohd Nawi; Siti Hamimah Sheikh Abdul Kadir; Teh Lay Kek; Wang Seok Mui
EPI_ISL_12135941, EPI_ISL_12135942, EPI_ISL_12135943, EPI_ISL_12135945, EPI_ISL_12135946	Jabatan Forensik Hospital Sultanah Bahiyah	Tropical Infectious Diseases Research & Education Centre (TIDREC), Universiti Malaya	AsmaAnati CheMatSeri; Che-Norainon Yaacob; Jia-Yi Tan; Jo-Ern Wong; Kim-Kee Tan; Mulya-Mustika-Sari Zulkifli; Noor Syahida Azizan; Nur-Hidayana Mahfodz; Sazaly AbuBakar; Siti-Sarah Nor'e; Wei-Wei Aw
EPI_ISL_11965269, EPI_ISL_11965270, EPI_ISL_12097424	Jenjarom Health Clinic	Institute for Medical Research, Infectious Disease Research Centre, National Institutes of Health, Ministry of Health Malaysia	Ahmad FA; Ahmad Fazilah NA; Anasir MI; Azizan MA; Kamel K; Mohamad Sukri MZ; Norhisham SN; Ramly N; Robert F; Rosli NR; Suppiah J; Thayan R
EPI_ISL_11939144, EPI_ISL_12771266	KK TRIANG	iPROMISE, UiTM	Ariza Adnan; Fadzilah Mohd Nor; Lim Wai Feng; Mohd Asif Mohd Sukri; Mohd Nur Fakhruzzaman Noorizhab; Mohd Zaki Salleh; Sazzli Shahlan Kassim; Siti Farah Alwani Mohd Nawi; Siti Hamimah Sheikh Abdul Kadir; Teh Lay Kek; Wang Seok Mui
EPI_ISL_11965304, EPI_ISL_11965312	Keningau Area Health Office	Institute for Medical Research, Infectious Disease Research Centre, National Institutes of Health, Ministry of Health Malaysia	Ahmad FA; Ahmad Fazilah NA; Anasir MI; Azizan MA; Kamel K; Mohamad Sukri MZ; Norhisham SN; Ramly N; Robert F; Rosli NR; Suppiah J; Thayan R
EPI_ISL_11965272, EPI_ISL_11965274, EPI_ISL_11965275	Kepong District Health Office	Institute for Medical Research, Infectious Disease Research Centre, National Institutes of Health, Ministry of Health Malaysia	Ahmad FA; Ahmad Fazilah NA; Anasir MI; Azizan MA; Kamel K; Mohamad Sukri MZ; Norhisham SN; Ramly N; Robert F; Rosli NR; Suppiah J; Thayan R
EPI_ISL_11332731, EPI_ISL_11332732, EPI_ISL_11332733, EPI_ISL_11332734, EPI_ISL_11332735, EPI_ISL_11332736, EPI_ISL_11332737, EPI_ISL_11332738, EPI_ISL_11332739, EPI_ISL_11332740, EPI_ISL_11332741, EPI_ISL_11332742, EPI_ISL_11332743, EPI_ISL_11332744, EPI_ISL_11332745, EPI_ISL_11332746, EPI_ISL_11332747, EPI_ISL_11332748, EPI_ISL_11332749, EPI_ISL_11332750, EPI_ISL_11332751, EPI_ISL_11332752, EPI_ISL_11332753, EPI_ISL_11332754, EPI_ISL_11332755, EPI_ISL_11332756, EPI_ISL_11332759, EPI_ISL_11332762, EPI_ISL_11332763, EPI_ISL_11332765			
see above	Kota Bharu Public Health Laboratory	Molecular Research Laboratory	Abdul Haris bin Muhammad; Ahmad Sukari Bin Halim; Alexander Chong Shu Chien; Asraihan Che Abdul Malik; Azian Harun; Chan Yean Yean; Chua Wei Chuan; Farahana binti Mohamed; Kirnpal Kaur Banga Singh; Lau Nyok Sean; Lee Lih Huey; Lim Shu Yong; Maizun binti Mohd Zain; Mera Edora Binti Abdul Manap; Mohd Iman hafiz Bin Ibrahim; Mohd Nadzri Abu Yazid; Mohd Zulkifli Salleh; Muhamad Khairul Amirin Bin Zulkifli; Muhammad Azamuddeen bin Mohammad Nasir; Muhammad Fazli bin Khalid; Muhammad Nashrul Farhan Samsudin; Muhammad Zarul Hanifah Bin Md Zoqratt; Nik Zuraina binti Nik Mohd Noor; Noor Hafizan binti Mat Salleh; Nor Amizara Binti Azami; Nor Suhana Binti Mohd Satar; Nur Syuhada Binti Abdul Rahim; Nur-Leem Binti Murshid; Nurfadhlina Musa; Qasim Ayub; Rosline Hassan; Sadequr Rahman; Siti Nur Aisyah Binti Mohamad Sham; Syahida binti Omar; Wan Mohd Zahiruddin Wan Mohammad; Wardah Yusof; Zaini bin Hussin; Zakuan Zainy Deris
EPI_ISL_11965310, EPI_ISL_11965313, EPI_ISL_11965314	Kota Kinabalu Area Health Office	Institute for Medical Research, Infectious Disease Research Centre, National Institutes of Health, Ministry of Health Malaysia	Ahmad FA; Ahmad Fazilah NA; Anasir MI; Azizan MA; Kamel K; Mohamad Sukri MZ; Norhisham SN; Ramly N; Robert F; Rosli NR; Suppiah J; Thayan R
EPI_ISL_11105872, EPI_ISL_11685212	Kota Marudu Health Office	Institute for Medical Research, Infectious Disease Research Centre, National Institutes of Health, Ministry of Health Malaysia	Ahmad FA; Ahmad Fazilah NA; Anasir MI; Azizan MA; G. Adypatti NM; Jamaluddin MS; Kamel K; Mohamad Sukri MZ; Norhisham SN; Ramly N; Robert F; Rosli NR; Suppiah J; Thayan R
EPI_ISL_11965271, EPI_ISL_11966113, EPI_ISL_11966120, EPI_ISL_11966124, EPI_ISL_11966125, EPI_ISL_11966126, EPI_ISL_11966131, EPI_ISL_11966137, EPI_ISL_11966138, EPI_ISL_11966144, EPI_ISL_11966145, EPI_ISL_11966150, EPI_ISL_11966151, EPI_ISL_11966155, EPI_ISL_11966156, EPI_ISL_11966159, EPI_ISL_11966160, EPI_ISL_11966169, EPI_ISL_11966170, EPI_ISL_11966171, EPI_ISL_12097416, EPI_ISL_12097419			
see above	Kuala Lumpur International Airport (KLIA) Health Office	Institute for Medical Research, Infectious Disease Research Centre, National Institutes of Health, Ministry of Health Malaysia	Ahmad FA; Ahmad Fazilah NA; Anasir MI; Azizan MA; Kamel K; Mohamad Sukri MZ; Norhisham SN; Ramly N; Robert F; Rosli NR; Suppiah J; Thayan R
EPI_ISL_12008471, EPI_ISL_12008499	MKAJB	UKM Medical Molecular Biology Institute (UMBI)	Khairun Nur Abd Ghafar; Mira Farzana Mohamad Mokhtar; Muhiddin Ishak; Nor Azila Muhammad Azami; Nur Alyaa Afifah Md Shahri; Nurul Syakima Ab Mutalib; Rahman Jamal; Ryia Illani Mohd Yunos; Siti Nur Hasanah Mohd Yusuf; Zahirrah Begam Mohamed Rasheed
EPI_ISL_11939181	Medical Hospital Pulau Pinang	iPROMISE, UiTM	Ariza Adnan; Fadzilah Mohd Nor; Lim Wai Feng; Mohd Asif Mohd Sukri; Mohd Nur Fakhruzzaman Noorizhab; Mohd Zaki Salleh; Sazzli Shahlan Kassim; Siti Farah Alwani Mohd Nawi; Siti Hamimah Sheikh Abdul Kadir; Teh Lay Kek; Wang Seok Mui
EPI_ISL_10512490, EPI_ISL_10512491, EPI_ISL_10512492, EPI_ISL_10512493, EPI_ISL_10512494, EPI_ISL_10759882, EPI_ISL_11332471			
see above	Miri Hospital Molecular Diagnostic Lab, Miri	Institute of Health and Community Medicine	Chan Chia Jui; Chua Hock Hin; David Perera; Ooi Mong How; Tonnii Sia Loong Loong
EPI_ISL_11672764, EPI_ISL_11672765, EPI_ISL_11672766, EPI_ISL_11672767, EPI_ISL_11672777, EPI_ISL_11672778	National Public Health Laboratory	National Public Health Laboratory	Aimi Shazana Binti Shahir; Kamal Hisham Bin Kamarul Zaman; Muhammad Shahir Bin Ali; Noriah Binti Mohd Yusof; Rehan Shuhada Binti Abu Bakar; Selvanesan A/L Sengol; W.Nur Afiza Binti W. Ariffin; Yu Kie A/P Chem
EPI_ISL_10512348, EPI_ISL_10512350, EPI_ISL_10512351, EPI_ISL_10512352, EPI_ISL_10512353, EPI_ISL_10512354, EPI_ISL_10512355, EPI_ISL_10512356, EPI_ISL_10512431, EPI_ISL_11332457, EPI_ISL_11332458			
see above	Normah Medical Specialist Centre, Kuching	Institute of Health and Community Medicine	Chan Chia Jui; Chua Hock Hin; David Perera; Ikwannuddin Y; Ooi Mong How; Tonnii Sia Loong Loong
EPI_ISL_11939165	PEJABAT KESIHATAN DAERAH TIMUR LAUT	iPROMISE, UiTM	Ariza Adnan; Fadzilah Mohd Nor; Lim Wai Feng; Mohd Asif Mohd Sukri; Mohd Nur Fakhruzzaman Noorizhab; Mohd Zaki Salleh; Sazzli Shahlan Kassim; Siti Farah Alwani Mohd Nawi; Siti Hamimah Sheikh Abdul Kadir; Teh Lay Kek; Wang Seok Mui
EPI_ISL_12771269	PEJABAT KESIHATAN PINTU MASUK ANTARABANGSA PULAU PINANG	iPROMISE, UiTM	Ariza Adnan; Fadzilah Mohd Nor; Lim Wai Feng; Mohd Asif Mohd Sukri; Mohd Nur Fakhruzzaman Noorizhab; Mohd Zaki Salleh; Sazzli Shahlan Kassim; Siti Farah Alwani Mohd Nawi; Siti Hamimah Sheikh Abdul Kadir; Teh Lay Kek; Wang Seok Mui
EPI_ISL_12628093	PKD JELEBU	UKM Medical Molecular Biology Institute (UMBI)	Khairun Nur Abd Ghafar; Mira Farzana Mohamad Mokhtar; Muhiddin Ishak; Nor Azila Muhammad Azami; Nur Alyaa Afifah Md Shahri; Nurul Syakima Ab Mutalib; Rahman Jamal; Ryia Illani Mohd Yunos; Siti Nur Hasanah Mohd Yusuf; Zahirrah Begam Mohamed Rasheed
EPI_ISL_12135973, EPI_ISL_12135974, EPI_ISL_12135975, EPI_ISL_12135976, EPI_ISL_12135977	PKD KAMPAR	Tropical Infectious Diseases Research & Education Centre (TIDREC), Universiti Malaya	AsmaAnati CheMatSeri; Che-Norainon Yaacob; Jia-Yi Tan; Jo-Ern Wong; Kim-Kee Tan; Mulya-Mustika-Sari Zulkifli; Noor Syahida Azizan; Nur-Hidayana Mahfodz; Sazaly AbuBakar; Siti-Sarah Nor'e; Wei-Wei Aw

EPI_ISL_12628091, EPI_ISL_12628092	PKD KUALA PILAH	UKM Medical Molecular Biology Institute (UMBI)	Khairun Nur Abd Ghafar; Mira Farzana Mohamad Mokhtar; Muhiddin Ishak; Nor Azila Muhammad Azami; Nur Alyaa Afifah Md Shahri; Nurul Syakima Ab Mutalib; Rahman Jamal; Ryia Illani Mohd Yunos; Siti Nur Hasanah Mohd Yusuf; Zahirrah Begam Mohamed Rasheed
EPI_ISL_12008476, EPI_ISL_12008527	PKD MUAR	UKM Medical Molecular Biology Institute (UMBI)	Khairun Nur Abd Ghafar; Mira Farzana Mohamad Mokhtar; Muhiddin Ishak; Nor Azila Muhammad Azami; Nur Alyaa Afifah Md Shahri; Nurul Syakima Ab Mutalib; Rahman Jamal; Ryia Illani Mohd Yunos; Siti Nur Hasanah Mohd Yusuf; Zahirrah Begam Mohamed Rasheed
EPI_ISL_12055307, EPI_ISL_12055332, EPI_ISL_12055361, EPI_ISL_12055388	PKD TAMPIN	Institut Biologi Molekul Perubatan UKM (UMBI)	Khairun Nur Abd Ghafar; Mira Farzana Mohamad Mokhtar; Muhiddin Ishak; Nor Azila Muhammad Azami; Nur Alyaa Afifah Md Shahri; Nurul Syakima Ab Mutalib; Rahman Jamal; Ryia Illani Mohd Yunos; Siti Nur Hasanah Mohd Yusuf; Zahirrah Begam Mohamed Rasheed
EPI_ISL_11939205, EPI_ISL_11939206	PKD TIMUR LAUT	iPROMISE, UiTM	Ariza Adnan; Fadzilah Mohd Nor; Lim Wai Feng; Mohd Asif Mohd Sukri; Mohd Nur Fakhruzzaman Noorizhab; Mohd Zaki Salleh; Sazzli Shahlan Kassim; Siti Farah Alwani Mohd Nawi; Siti Hamimah Sheikh Abdul Kadir; Teh Lay Kek; Wang Seok Mui
EPI_ISL_11939152	Pejabat Kesihatan Pintu Masuk Antarabangsa Pulau PInang	iPROMISE, UiTM	Ariza Adnan; Fadzilah Mohd Nor; Lim Wai Feng; Mohd Asif Mohd Sukri; Mohd Nur Fakhruzzaman Noorizhab; Mohd Zaki Salleh; Sazzli Shahlan Kassim; Siti Farah Alwani Mohd Nawi; Siti Hamimah Sheikh Abdul Kadir; Teh Lay Kek; Wang Seok Mui
EPI_ISL_11939153, EPI_ISL_11939173, EPI_ISL_12771265, EPI_ISL_12771275, EPI_ISL_12771284	Pejabat Kesihatan Pintu Masuk Antarabangsa Pulau Pinang	iPROMISE, UiTM	Ariza Adnan; Fadzilah Mohd Nor; Lim Wai Feng; Mohd Asif Mohd Sukri; Mohd Nur Fakhruzzaman Noorizhab; Mohd Zaki Salleh; Sazzli Shahlan Kassim; Siti Farah Alwani Mohd Nawi; Siti Hamimah Sheikh Abdul Kadir; Teh Lay Kek; Wang Seok Mui
EPI_ISL_11965273, EPI_ISL_12097426	Putrajaya Health Office	Institute for Medical Research, Infectious Disease Research Centre, National Institutes of Health, Ministry of Health Malaysia	Ahmad FA; Ahmad Fazilah NA; Anasir MI; Azizan MA; Kamel K; Mohamad Sukri MZ; Norhisham SN; Ramly N; Robert F; Rosli NR; Suppiah J; Thayan R
EPI_ISL_12289469, EPI_ISL_12289470, EPI_ISL_12289471, EPI_ISL_12289475, EPI_ISL_12289492, EPI_ISL_12665282, EPI_ISL_12665284, EPI_ISL_12665285, EPI_ISL_12665301, EPI_ISL_12746942, EPI_ISL_12746943, EPI_ISL_12746950, EPI_ISL_12746951, EPI_ISL_12746952, EPI_ISL_12746953, EPI_ISL_12746954, EPI_ISL_12746955, EPI_ISL_12746956, EPI_ISL_12746957, EPI_ISL_12746958, EPI_ISL_12746959, EPI_ISL_12746960, EPI_ISL_12746961, EPI_ISL_12746962, EPI_ISL_12746963, EPI_ISL_12746964, EPI_ISL_12746965, EPI_ISL_12746966, EPI_ISL_12746967, EPI_ISL_12746968, EPI_ISL_12746969, EPI_ISL_12746970, EPI_ISL_12746971, EPI_ISL_12746972, EPI_ISL_12746973, EPI_ISL_12746974, EPI_ISL_12746975, EPI_ISL_12746976, EPI_ISL_12746977, EPI_ISL_12746978, EPI_ISL_12746979, EPI_ISL_12746980, EPI_ISL_12746981, EPI_ISL_12746982, EPI_ISL_12746984, EPI_ISL_12749679			
see above	QUEEN ELIZABETH HOSPITAL	Institute for Medical Research, Infectious Disease Research Centre, National Institutes of Health, Ministry of Health Malaysia	Ahmad FA; Ahmad Fazilah NA; Anasir MI; Azizan MA; Kamel K; Mohamad Sukri MZ; Norhisham SN; Ramly N; Robert F; Rosli NR; Suppiah J; Thayan R
EPI_ISL_11685209, EPI_ISL_11685215, EPI_ISL_11685216, EPI_ISL_11685217, EPI_ISL_11685222, EPI_ISL_12084298	Queen Elizabeth Hospital	Institute for Medical Research, Infectious Disease Research Centre, National Institutes of Health, Ministry of Health Malaysia	Ahmad FA; Ahmad Fazilah NA; Anasir MI; Azizan MA; G. Adypatti NM; Jamaluddin MS; Kamel K; Mohamad Sukri MZ; Norhisham SN; Ramly N; Robert F; Rosli NR; Suppiah J; Thayan R
EPI_ISL_11105878, EPI_ISL_11105879, EPI_ISL_11105880, EPI_ISL_11105881, EPI_ISL_11105882, EPI_ISL_11685208	Queen Elizabeth Hospital, Kota Kinabalu	Institute for Medical Research, Infectious Disease Research Centre, National Institutes of Health, Ministry of Health Malaysia	Ahmad FA; Ahmad Fazilah NA; Anasir MI; Azizan MA; G. Adypatti NM; Jamaluddin MS; Kamel K; Mohamad Sukri MZ; Norhisham SN; Ramly N; Robert F; Rosli NR; Suppiah J; Thayan R
EPI_ISL_10512392, EPI_ISL_10512393, EPI_ISL_10512394, EPI_ISL_10512395, EPI_ISL_10512396, EPI_ISL_10512397, EPI_ISL_10512398, EPI_ISL_10512399, EPI_ISL_10512400, EPI_ISL_10512401, EPI_ISL_10512402, EPI_ISL_10512403, EPI_ISL_10512404, EPI_ISL_10512405, EPI_ISL_10512406, EPI_ISL_10512407, EPI_ISL_10512408, EPI_ISL_10512409, EPI_ISL_10512410, EPI_ISL_10759929, EPI_ISL_10759930, EPI_ISL_10759931, EPI_ISL_10759932, EPI_ISL_11332454, EPI_ISL_11332455, EPI_ISL_11332456, EPI_ISL_12739672, EPI_ISL_12739673, EPI_ISL_12739674, EPI_ISL_12739675, EPI_ISL_12739676, EPI_ISL_12739677, EPI_ISL_12739678, EPI_ISL_12739679, EPI_ISL_12739680, EPI_ISL_12739681, EPI_ISL_12739682, EPI_ISL_12739683, EPI_ISL_12739684, EPI_ISL_12739685, EPI_ISL_12739686, EPI_ISL_12739687, EPI_ISL_12739688, EPI_ISL_12739689, EPI_ISL_12739690, EPI_ISL_12739691, EPI_ISL_12739692, EPI_ISL_12739693, EPI_ISL_12739694			
see above	Rejang Medical Centre (Sibu)	Institute of Health and Community Medicine	Chan Chia Jui; Chua Hock Hin; David Perera; Ooi Mong How; Tonnii Sia Loong Loong; Wong Jyn Shan
EPI_ISL_10512255, EPI_ISL_10512256, EPI_ISL_10512257, EPI_ISL_10512316, EPI_ISL_10512318, EPI_ISL_10512319, EPI_ISL_10512320, EPI_ISL_10512321, EPI_ISL_10512322, EPI_ISL_10512325, EPI_ISL_10512326, EPI_ISL_10512327, EPI_ISL_10512330, EPI_ISL_10512331, EPI_ISL_10512332, EPI_ISL_10512333, EPI_ISL_10512428, EPI_ISL_10512429, EPI_ISL_10512430, EPI_ISL_11332466			
see above	Sarawak General Hospital (Kuching)	Institute of Health and Community Medicine	Chan Chia Jui; Chua Hock Hin; David Perera; Ooi Mong How; Tonnii Sia Loong Loong
EPI_ISL_10981858, EPI_ISL_10981869, EPI_ISL_10981873, EPI_ISL_10981876, EPI_ISL_10981880, EPI_ISL_10981883, EPI_ISL_10981888, EPI_ISL_10981897			
see above	Selayang Hospital	Institute for Medical Research, Infectious Disease Research Centre, National Institutes of Health, Ministry of Health Malaysia	Ahmad FA; Ahmad Fazilah NA; Anasir MI; Azizan MA; Kamel K; Mohamad Sukri MZ; Norhisham SN; Ramly N; Robert F; Rosli NR; Suppiah J; Thayan R
EPI_ISL_10512445, EPI_ISL_10512446, EPI_ISL_10512447, EPI_ISL_10512451, EPI_ISL_10512469, EPI_ISL_10512471, EPI_ISL_10512478, EPI_ISL_10512482, EPI_ISL_10512495, EPI_ISL_10512505, EPI_ISL_10512507, EPI_ISL_10512508, EPI_ISL_10512509, EPI_ISL_10512511, EPI_ISL_10512512, EPI_ISL_10512513, EPI_ISL_10512514, EPI_ISL_10512515, EPI_ISL_10512516, EPI_ISL_10512517, EPI_ISL_10512518, EPI_ISL_10512519, EPI_ISL_10512520, EPI_ISL_10512521, EPI_ISL_10512522, EPI_ISL_10512523, EPI_ISL_11332626, EPI_ISL_11332627, EPI_ISL_11332628, EPI_ISL_11332629, EPI_ISL_11332630			
see above	Sibu Hospital (Sibu, Sarawak)	Institute of Health and Community Medicine	Chan Chia Jui; Chua Hock Hin; David Perera; Ooi Mong How; Tonnii Sia Loong Loong
EPI_ISL_10408556, EPI_ISL_10408557, EPI_ISL_10408558, EPI_ISL_10408559, EPI_ISL_10408560, EPI_ISL_10408561, EPI_ISL_10408562, EPI_ISL_10408563, EPI_ISL_10408564, EPI_ISL_10408565, EPI_ISL_13026018, EPI_ISL_13026021, EPI_ISL_13026022, EPI_ISL_13026025, EPI_ISL_13026026, EPI_ISL_13026028, EPI_ISL_13026029, EPI_ISL_13026030, EPI_ISL_13026051			
see above	Synapse Sdn. Bhd.	Universiti Putra Malaysia (UPM)	Choo Yee Yu; Hui Jen Soe; Hui Yee Chee; Nancy Woan Charn Liew; Narcisse Joseph; Nurulfiza Mat Isa; Rachna Kairon; Sie Yeng Wong; Sook Yee Boo; Syafinaz Amin-Nordin; Zunita Zakaria
EPI_ISL_12322082	THOMSON HOSPITAL	Institute for Medical Research, Infectious Disease Research Centre, National Institutes of Health, Ministry of Health Malaysia	Ahmad FA; Ahmad Fazilah NA; Anasir MI; Azizan MA; Kamel K; Mohamad Sukri MZ; Norhisham SN; Ramly N; Robert F; Rosli NR; Suppiah J; Thayan R
EPI_ISL_12097429	Tawau Hospital	Institute for Medical Research, Infectious Disease Research Centre, National Institutes of Health, Ministry of Health Malaysia	Ahmad FA; Ahmad Fazilah NA; Anasir MI; Azizan MA; Kamel K; Mohamad Sukri MZ; Norhisham SN; Ramly N; Robert F; Rosli NR; Suppiah J; Thayan R
EPI_ISL_11685220	Tuaran Hospital	Institute for Medical Research, Infectious Disease Research Centre, National Institutes of Health, Ministry of Health Malaysia	Ahmad FA; Ahmad Fazilah NA; Anasir MI; Azizan MA; G. Adypatti NM; Jamaluddin MS; Kamel K; Mohamad Sukri MZ; Norhisham SN; Ramly N; Robert F; Rosli NR; Suppiah J; Thayan R
EPI_ISL_12055329	UKA	Institut Biologi Molekul Perubatan UKM (UMBI)	Khairun Nur Abd Ghafar; Mira Farzana Mohamad Mokhtar; Muhiddin Ishak; Nor Azila Muhammad Azami; Nur Alyaa Afifah Md Shahri; Nurul Syakima Ab Mutalib; Rahman Jamal; Ryia Illani Mohd Yunos; Siti Nur Hasanah Mohd Yusuf; Zahirrah Begam Mohamed Rasheed
EPI_ISL_12055372, EPI_ISL_12055387, EPI_ISL_12055389	UKA HEBHK	Institut Biologi Molekul Perubatan UKM (UMBI)	Khairun Nur Abd Ghafar; Mira Farzana Mohamad Mokhtar; Muhiddin Ishak; Nor Azila Muhammad Azami; Nur Alyaa Afifah Md Shahri; Nurul Syakima Ab Mutalib; Rahman Jamal; Ryia Illani Mohd Yunos; Siti Nur Hasanah Mohd Yusuf; Zahirrah Begam Mohamed Rasheed
EPI_ISL_11754765	UMMC	Department of Medical Microbiology, Faculty of Medicine, University of Malaya; University of Malaya Medical Centre	I-Ching SAM; Jolene Yin Ling FU; Omar Khalilur Rahman; Yoke Fun Chan
EPI_ISL_12008465, EPI_ISL_12008498, EPI_ISL_12008508	UNIT KESIHATAN AWAM HEBHK	UKM Medical Molecular Biology Institute (UMBI)	Khairun Nur Abd Ghafar; Mira Farzana Mohamad Mokhtar; Muhiddin Ishak; Nor Azila Muhammad Azami; Nur Alyaa Afifah Md Shahri; Nurul Syakima Ab Mutalib; Rahman Jamal; Ryia Illani Mohd Yunos; Siti Nur Hasanah Mohd Yusuf; Zahirrah Begam Mohamed Rasheed
EPI_ISL_11939167	Unit Forensik Hospital Kepala Batas	iPROMISE, UiTM	Ariza Adnan; Fadzilah Mohd Nor; Lim Wai Feng; Mohd Asif Mohd Sukri; Mohd Nur Fakhruzzaman Noorizhab; Mohd Zaki Salleh; Sazzli Shahlan Kassim; Siti Farah Alwani Mohd Nawi; Siti Hamimah Sheikh Abdul Kadir; Teh Lay Kek; Wang Seok Mui
EPI_ISL_12055355	Unit Kesihatan Awam Hebhk	Institut Biologi Molekul Perubatan UKM (UMBI)	Khairun Nur Abd Ghafar; Mira Farzana Mohamad Mokhtar; Muhiddin Ishak; Nor Azila Muhammad Azami; Nur Alyaa Afifah Md Shahri; Nurul Syakima Ab Mutalib; Rahman Jamal; Ryia Illani Mohd Yunos; Siti Nur Hasanah Mohd Yusuf; Zahirrah Begam Mohamed Rasheed
EPI_ISL_12055342, EPI_ISL_12055367, EPI_ISL_12055384	uka hpsf muar	Institut Biologi Molekul Perubatan UKM (UMBI)	Khairun Nur Abd Ghafar; Mira Farzana Mohamad Mokhtar; Muhiddin Ishak; Nor Azila Muhammad Azami; Nur Alyaa Afifah Md Shahri; Nurul Syakima Ab Mutalib; Rahman Jamal; Ryia Illani Mohd Yunos; Siti Nur Hasanah Mohd Yusuf; Zahirrah Begam Mohamed Rasheed
EPI_ISL_12055381	unit kesihatan awam hpsf muar	Institut Biologi Molekul Perubatan UKM (UMBI)	Khairun Nur Abd Ghafar; Mira Farzana Mohamad Mokhtar; Muhiddin Ishak; Nor Azila Muhammad Azami; Nur Alyaa Afifah Md Shahri; Nurul Syakima Ab Mutalib; Rahman Jamal; Ryia Illani Mohd Yunos; Siti Nur Hasanah Mohd Yusuf; Zahirrah Begam Mohamed Rasheed

We gratefully acknowledge the following Authors from the Originating laboratories responsible for obtaining the specimens, as well as the Submitting laboratories where the genome data were generated and shared via GISAID, on which this research is based.

All Submitters of data may be contacted directly via www.gisaid.org

Authors are sorted alphabetically.

Accession ID	Originating Laboratory	Submitting Laboratory	Authors
EPI_ISL_10981874, EPI_ISL_10981878, EPI_ISL_10981885	Ampang Hospital	Institute for Medical Research, Infectious Disease Research Centre, National Institutes of Health, Ministry of Health Malaysia	Ahmad FA; Ahmad Fazilah NA; Anasir MI; Azizan MA; Kamel K; Mohamad Sukri MZ; Norhisham SN; Ramly N; Robert F; Rosli NR; Suppiah J; Thayan R
EPI_ISL_10512444, EPI_ISL_10512448, EPI_ISL_11332607	Bintulu Hospital PCR Lab, Bintulu	Institute of Health and Community Medicine	Chan Chia Jui; Chua Hock Hin; David Perera; Ooi Mong How; Tan Lee See; Tonnii Sia Loong Loong; Wong Jyn Shan
EPI_ISL_10759986, EPI_ISL_10759999, EPI_ISL_10760020, EPI_ISL_10760021, EPI_ISL_10760035, EPI_ISL_10760037, EPI_ISL_10760038, EPI_ISL_10760040, EPI_ISL_10760041, EPI_ISL_10760042, EPI_ISL_10760043, EPI_ISL_10760044, EPI_ISL_10760045, EPI_ISL_10760046, EPI_ISL_10760047, EPI_ISL_10760049, EPI_ISL_10760050, EPI_ISL_11804984, EPI_ISL_11804996, EPI_ISL_11805001			
see above	Bintulu Medical Centre (Bintulu)	Institute of Health and Community Medicine	Chan Chia Jui; Chua Hock Hin; David Perera; Ooi Mong How; Tonnii Sia Loong Loong; Wong Jyn Shan
EPI_ISL_10759893, EPI_ISL_10759894, EPI_ISL_10759895, EPI_ISL_10759896, EPI_ISL_10759897, EPI_ISL_10759901, EPI_ISL_10759903, EPI_ISL_10759904, EPI_ISL_10759905, EPI_ISL_10759906, EPI_ISL_10759907, EPI_ISL_12739638, EPI_ISL_12739639, EPI_ISL_12739640, EPI_ISL_12739641, EPI_ISL_12739642, EPI_ISL_12739643, EPI_ISL_12739644, EPI_ISL_12739645, EPI_ISL_12739646, EPI_ISL_12739647, EPI_ISL_12739648, EPI_ISL_12739649, EPI_ISL_12739650, EPI_ISL_12739654, EPI_ISL_12739656, EPI_ISL_12739657, EPI_ISL_12739658, EPI_ISL_12739659, EPI_ISL_12739660, EPI_ISL_12739661, EPI_ISL_12739662, EPI_ISL_12739663, EPI_ISL_12739664, EPI_ISL_12739665, EPI_ISL_12739666, EPI_ISL_12739667, EPI_ISL_12739668, EPI_ISL_12739669, EPI_ISL_12739670, EPI_ISL_12739671			
see above	Borneo Medical Centre (Kuching)	Institute of Health and Community Medicine	Chan Chia Jui; Chua Hock Hin; David Perera; Ooi Mong How; Tonnii Sia Loong Loong; Wong Jyn Shan
EPI_ISL_10759899, EPI_ISL_10759902, EPI_ISL_10759926, EPI_ISL_10759927, EPI_ISL_10759947, EPI_ISL_10759951, EPI_ISL_10759952, EPI_ISL_10759961, EPI_ISL_10759968, EPI_ISL_10759985			
see above	Borneo Medical Centre (Miri)	Institute of Health and Community Medicine	Chan Chia Jui; Chua Hock Hin; David Perera; Ooi Mong How; Tonnii Sia Loong Loong; Wong Jyn Shan
EPI_ISL_11965282, EPI_ISL_11965283	Cheras District Health Office	Institute for Medical Research, Infectious Disease Research Centre, National Institutes of Health, Ministry of Health Malaysia	Ahmad FA; Ahmad Fazilah NA; Anasir MI; Azizan MA; Kamel K; Mohamad Sukri MZ; Norhisham SN; Ramly N; Robert F; Rosli NR; Suppiah J; Thayan R
EPI_ISL_12665275	DISTRICT HEALTH OFFICE TENOM	Institute for Medical Research, Infectious Disease Research Centre, National Institutes of Health, Ministry of Health Malaysia	Ahmad FA; Ahmad Fazilah NA; Anasir MI; Azizan MA; Kamel K; Mohamad Sukri MZ; Norhisham SN; Ramly N; Robert F; Rosli NR; Suppiah J; Thayan R
EPI_ISL_12721588, EPI_ISL_12721589, EPI_ISL_12721590, EPI_ISL_12721591, EPI_ISL_12721593, EPI_ISL_12721594, EPI_ISL_12721595			
see above	Department of Medical Microbiology, Hospital Pengajar Universiti Putra Malaysia	Department of Medical Microbiology, Hospital Pengajar Universiti Putra Malaysia	Afiqah Adzmi; Amiza Azmi; Azmiza Syawani Jasni; Hui Yee Chee; Leslie Thian Lung Than; Muadz Mohtar; Muhammad Mohd Isa; Narcisse MS Joseph; Niazlin Mohd Taib; Noor Hazirah Noor Azhari; Norlaila; Nur Raihana Ithnin; Nurul Huda Mohamed Rashidi; Nurul Nadiah Ismail; Rosni Ibrahim; Rukman Awang Hamat; Sallehhudin; Siti Norbaya Masri; Siti Zulaikha Zakariah; Syafinaz Amin Nordin; Tengku Zetty Maztura Tengku Jamaluddin; Zamberi Sekawi
EPI_ISL_12097434	Duchess of Kent Hospital	Institute for Medical Research, Infectious Disease Research Centre, National Institutes of Health, Ministry of Health Malaysia	Ahmad FA; Ahmad Fazilah NA; Anasir MI; Azizan MA; Kamel K; Mohamad Sukri MZ; Norhisham SN; Ramly N; Robert F; Rosli NR; Suppiah J; Thayan R
EPI_ISL_10759987, EPI_ISL_10759988, EPI_ISL_10759989, EPI_ISL_10759990, EPI_ISL_10759991, EPI_ISL_10759992, EPI_ISL_10759993, EPI_ISL_10759994, EPI_ISL_10759995, EPI_ISL_10759996, EPI_ISL_10759997, EPI_ISL_10759998			
see above	Gribbles Diagnostic Lab @Columbia Asia Hospital, Miri	Institute of Health and Community Medicine	Chan Chia Jui; Chua Hock Hin; David Perera; Liew Chin Chin J; Ooi Mong How; Tonnii Sia Loong Loong; Wong Jyn Shan
EPI_ISL_11332464, EPI_ISL_11332465, EPI_ISL_11804879, EPI_ISL_12739617, EPI_ISL_12739618, EPI_ISL_12739619, EPI_ISL_12739620, EPI_ISL_12739621, EPI_ISL_12739622, EPI_ISL_12739623, EPI_ISL_12739624, EPI_ISL_12739626, EPI_ISL_12739627, EPI_ISL_12739628, EPI_ISL_12739629, EPI_ISL_12739630, EPI_ISL_12739631, EPI_ISL_12739632, EPI_ISL_12739633, EPI_ISL_12739634, EPI_ISL_12739635, EPI_ISL_12739636, EPI_ISL_12739637			
see above	Gribbles Diagnostic Lab @Timberland Medical Centre, Kuching	Institute of Health and Community Medicine	Chan Chia Jui; Chua Hock Hin; David Perera; Liew Chin Chin J; Ooi Mong How; Tonnii Sia Loong Loong; Wong Jyn Shan
EPI_ISL_10512357, EPI_ISL_10512358, EPI_ISL_10512359, EPI_ISL_10512360, EPI_ISL_10512361, EPI_ISL_10512362, EPI_ISL_10512363, EPI_ISL_10512364, EPI_ISL_10512365, EPI_ISL_10512366, EPI_ISL_10512367, EPI_ISL_10512368, EPI_ISL_10512369, EPI_ISL_10512370, EPI_ISL_10512371, EPI_ISL_10512372, EPI_ISL_10512373, EPI_ISL_10512433, EPI_ISL_10512434, EPI_ISL_10512435, EPI_ISL_10512436, EPI_ISL_10512437, EPI_ISL_10512438, EPI_ISL_10512439, EPI_ISL_10512440, EPI_ISL_10512441, EPI_ISL_10512442, EPI_ISL_10512443, EPI_ISL_10512510			
see above	Gribbles Pathology, Kuching	Institute of Health and Community Medicine	Chan Chia Jui; Chua Hock Hin; David Perera; Liew Chin Chin J; Ooi Mong How; Tonnii Sia Loong Loong
EPI_ISL_12135953, EPI_ISL_12135954, EPI_ISL_12135955, EPI_ISL_12135956, EPI_ISL_12135957, EPI_ISL_12135958	HOSPITAL RAJA PERMAISURI BAINUN	Tropical Infectious Diseases Research & Education Centre (TIDREC), Universiti Malaya	AsmaAnati CheMatSeri; Che-Norainon Yaacob; Jia-Yi Tan; Jo-Ern Wong; Kim-Kee Tan; Mulya-Mustika-Sari Zulkifli; Noor Syahida Azizan; Nur-Hidayana Mahfodz; Sazaly AbuBakar; Siti-Sarah Nor’e; Wei-Wei Aw
EPI_ISL_12136005, EPI_ISL_12136007	HOSPITAL SULTAN ABDUL HALIM	Tropical Infectious Diseases Research & Education Centre (TIDREC), Universiti Malaya	AsmaAnati CheMatSeri; Che-Norainon Yaacob; Jia-Yi Tan; Jo-Ern Wong; Kim-Kee Tan; Mulya-Mustika-Sari Zulkifli; Noor Syahida Azizan; Nur-Hidayana Mahfodz; Sazaly AbuBakar; Siti-Sarah Nor’e; Wei-Wei Aw
EPI_ISL_12135950, EPI_ISL_12136006	HOSPITAL SULTAN ABDUL HALIM, SG PETANI	Tropical Infectious Diseases Research & Education Centre (TIDREC), Universiti Malaya	AsmaAnati CheMatSeri; Che-Norainon Yaacob; Jia-Yi Tan; Jo-Ern Wong; Kim-Kee Tan; Mulya-Mustika-Sari Zulkifli; Noor Syahida Azizan; Nur-Hidayana Mahfodz; Sazaly AbuBakar; Siti-Sarah Nor’e; Wei-Wei Aw
EPI_ISL_11939150	HOSPITAL SULTAN HAJI AHMAD SHAH	iPROMISE, UiTM	Ariza Adnan; Fadzilah Mohd Nor; Lim Wai Feng; Mohd Asif Mohd Sukri; Mohd Nur Fakhruzzaman Noorizhab; Mohd Zaki Salleh; Sazzli Shahlan Kassim; Siti Farah Alwani Mohd Nawi; Siti Hamimah Sheikh Abdul Kadir; Teh Lay Kek; Wang Seok Mui
EPI_ISL_12135964, EPI_ISL_12135993, EPI_ISL_12135994	HOSPITAL SULTANAH NUR ZAHIRAH	Tropical Infectious Diseases Research & Education Centre (TIDREC), Universiti Malaya	AsmaAnati CheMatSeri; Che-Norainon Yaacob; Jia-Yi Tan; Jo-Ern Wong; Kim-Kee Tan; Mulya-Mustika-Sari Zulkifli; Noor Syahida Azizan; Nur-Hidayana Mahfodz; Sazaly AbuBakar; Siti-Sarah Nor’e; Wei-Wei Aw
EPI_ISL_12135966	HOSPITAL TUANKU FAUZIAH KANGAR	Tropical Infectious Diseases Research & Education Centre (TIDREC), Universiti Malaya	AsmaAnati CheMatSeri; Che-Norainon Yaacob; Jia-Yi Tan; Jo-Ern Wong; Kim-Kee Tan; Mulya-Mustika-Sari Zulkifli; Noor Syahida Azizan; Nur-Hidayana Mahfodz; Sazaly AbuBakar; Siti-Sarah Nor’e; Wei-Wei Aw
EPI_ISL_11939187, EPI_ISL_11939188	HOSPITAL TUANKU FAUZIAH KANGAR	iPROMISE, UiTM	Ariza Adnan; Fadzilah Mohd Nor; Lim Wai Feng; Mohd Asif Mohd Sukri; Mohd Nur Fakhruzzaman Noorizhab; Mohd Zaki Salleh; Sazzli Shahlan Kassim; Siti Farah Alwani Mohd Nawi; Siti Hamimah Sheikh Abdul Kadir; Teh Lay Kek; Wang Seok Mui
EPI_ISL_12055327, EPI_ISL_12055328, EPI_ISL_12055336, EPI_ISL_12055383	HSAJB	Institut Biologi Molekul Perubatan UKM (UMBI)	Khairun Nur Abd Ghafar; Mira Farzana Mohamad Mokhtar; Muhiddin Ishak; Nor Azila Muhammad Azami; Nur Alyaa Afifah Md Shahri; Nurul Syakima Ab Mutalib; Rahman Jamal; Ryia Illani Mohd Yunos; Siti Nur Hasanah Mohd Yusuf; Zahirrah Begam Mohamed Rasheed
EPI_ISL_12008472, EPI_ISL_12008495, EPI_ISL_12008509, EPI_ISL_12008530	HSAJB	UKM Medical Molecular Biology Institute (UMBI)	Khairun Nur Abd Ghafar; Mira Farzana Mohamad Mokhtar; Muhiddin Ishak; Nor Azila Muhammad Azami; Nur Alyaa Afifah Md Shahri; Nurul Syakima Ab Mutalib; Rahman Jamal; Ryia Illani Mohd Yunos; Siti Nur Hasanah Mohd Yusuf; Zahirrah Begam Mohamed Rasheed
EPI_ISL_12008449, EPI_ISL_12008484, EPI_ISL_12008489, EPI_ISL_12008507	HTJS	UKM Medical Molecular Biology Institute (UMBI)	Khairun Nur Abd Ghafar; Mira Farzana Mohamad Mokhtar; Muhiddin Ishak; Nor Azila Muhammad Azami; Nur Alyaa Afifah Md Shahri; Nurul Syakima Ab Mutalib; Rahman Jamal; Ryia Illani Mohd Yunos; Siti Nur Hasanah Mohd Yusuf; Zahirrah Begam Mohamed Rasheed
EPI_ISL_11332729, EPI_ISL_11332730, EPI_ISL_12739791	Hospital Raja Perempuan Zainab II, Kota Bharu	Molecular Research Laboratory	Abdul Haris bin Muhammad; Ahmad Sukari Bin Halim; Alexander Chong Shu Chien; Asraihan Che Abdul Malik; Azian Harun; Chan Yean Yean; Chua Wei Chuan; Farahana binti Mohamed; Kirnpal Kaur Banga Singh; Lau Nyok Sean; Lee Lih Huey; Lim Shu Yong; Maizun binti Mohd Zain; Mera Edora Binti Abdul Manap; Mohd Iman hafiz Bin Ibrahim; Mohd Nadzri Abu Yazid; Mohd Zulkifli Salleh; Muhamad Khairul Amirin Bin Zulkifli; Muhammad Azamuddeen bin Mohammad Nasir; Muhammad Fazli bin Khalid; Muhammad Nashrul Farhan Samsudin; Muhammad Zarul Hanifah Bin Md Zoqratt; Nik Zuraina binti Nik Mohd Noor; Noor Hafizan binti Mat Salleh; Nor Amizara Binti Azami; Nor Suhana Binti Mohd Satar; Nur Syuhada Binti Abdul Rahim; Nur-Leem Binti Murshid; Nurfadhlina Musa; Qasim Ayub; Rosline Hassan; Sadequr Rahman; Siti Nur Aisyah Binti Mohamad Sham; Syahida binti Omar; Wan Mohd

			Zahiruddin Wan Mohammad; Wardah Yusof; Zaini bin Hussin; Zakuan Zainy Deris
EPI_ISL_12135965	Hospital Tuanku Fauziah	Tropical Infectious Diseases Research & Education Centre (TIDREC), Universiti Malaya	AsmaAnati CheMatSeri; Che-Norainon Yaacob; Jia-Yi Tan; Jo-Ern Wong; Kim-Kee Tan; Mulya-Mustika-Sari Zulkifli; Noor Syahida Azizan; Nur-Hidayana Mahfodz; Sazaly AbuBakar; Siti-Sarah Nor'e; Wei-Wei Aw
EPI_ISL_11939186	Hospital Tuanku Fauziah	iPROMISE, UiTM	Ariza Adnan; Fadzilah Mohd Nor; Lim Wai Feng; Mohd Asif Mohd Sukri; Mohd Nur Fakhruzzaman Noorizhab; Mohd Zaki Salleh; Sazzli Shahlan Kassim; Siti Farah Alwani Mohd Nawi; Siti Hamimah Sheikh Abdul Kadir; Teh Lay Kek; Wang Seok Mui
EPI_ISL_11939164	ID Department, Hospital Pulau Pinang	iPROMISE, UiTM	Ariza Adnan; Fadzilah Mohd Nor; Lim Wai Feng; Mohd Asif Mohd Sukri; Mohd Nur Fakhruzzaman Noorizhab; Mohd Zaki Salleh; Sazzli Shahlan Kassim; Siti Farah Alwani Mohd Nawi; Siti Hamimah Sheikh Abdul Kadir; Teh Lay Kek; Wang Seok Mui
EPI_ISL_11323755, EPI_ISL_11323770, EPI_ISL_11323795, EPI_ISL_11323805, EPI_ISL_11323806, EPI_ISL_11323826, EPI_ISL_11323833			
see above	Institut Biologi Molekul Perubatan UKM (UMBI)	UKM Medical Molecular Biology Institute (UMBI)	Khairun Nur Abd Ghafar; Mira Farzana Mohamad Mokhtar; Muhiddin Ishak; Nor Azila Muhammad Azami; Nur Alyaa Afifah Md Shahri; Nurul Syakima Ab Mutalib; Rahman Jamal; Ryia Illani Mohd Yunos; Siti Nur Hasanah Mohd Yusuf; Zahirrah Begam Mohamed Rasheed
EPI_ISL_11939151, EPI_ISL_12771233	JKN PAHANG	iPROMISE, UiTM	Ariza Adnan; Fadzilah Mohd Nor; Lim Wai Feng; Mohd Asif Mohd Sukri; Mohd Nur Fakhruzzaman Noorizhab; Mohd Zaki Salleh; Sazzli Shahlan Kassim; Siti Farah Alwani Mohd Nawi; Siti Hamimah Sheikh Abdul Kadir; Teh Lay Kek; Wang Seok Mui
EPI_ISL_12135947, EPI_ISL_12135948, EPI_ISL_12135949, EPI_ISL_12135951, EPI_ISL_12135952, EPI_ISL_12135967, EPI_ISL_12135968, EPI_ISL_12135969			
see above	Jabatan Forensik Hospital Sultanah Bahiyah	Tropical Infectious Diseases Research & Education Centre (TIDREC), Universiti Malaya	AsmaAnati CheMatSeri; Che-Norainon Yaacob; Jia-Yi Tan; Jo-Ern Wong; Kim-Kee Tan; Mulya-Mustika-Sari Zulkifli; Noor Syahida Azizan; Nur-Hidayana Mahfodz; Sazaly AbuBakar; Siti-Sarah Nor'e; Wei-Wei Aw
EPI_ISL_12055359	KK MAHMOODIAH	Institut Biologi Molekul Perubatan UKM (UMBI)	Khairun Nur Abd Ghafar; Mira Farzana Mohamad Mokhtar; Muhiddin Ishak; Nor Azila Muhammad Azami; Nur Alyaa Afifah Md Shahri; Nurul Syakima Ab Mutalib; Rahman Jamal; Ryia Illani Mohd Yunos; Siti Nur Hasanah Mohd Yusuf; Zahirrah Begam Mohamed Rasheed
EPI_ISL_12008453	KK MAHMOODIAH	UKM Medical Molecular Biology Institute (UMBI)	Khairun Nur Abd Ghafar; Mira Farzana Mohamad Mokhtar; Muhiddin Ishak; Nor Azila Muhammad Azami; Nur Alyaa Afifah Md Shahri; Nurul Syakima Ab Mutalib; Rahman Jamal; Ryia Illani Mohd Yunos; Siti Nur Hasanah Mohd Yusuf; Zahirrah Begam Mohamed Rasheed
EPI_ISL_11939143	KLINIK KESIHATAN KUALA KRAU	iPROMISE, UiTM	Ariza Adnan; Fadzilah Mohd Nor; Lim Wai Feng; Mohd Asif Mohd Sukri; Mohd Nur Fakhruzzaman Noorizhab; Mohd Zaki Salleh; Sazzli Shahlan Kassim; Siti Farah Alwani Mohd Nawi; Siti Hamimah Sheikh Abdul Kadir; Teh Lay Kek; Wang Seok Mui
EPI_ISL_12099637, EPI_ISL_12099638	Kapit Hospital, PCR lab (Kapit, Sarawak)	Institute of Health and Community Medicine	Chan Chia Jui; Chua Hock Hin; David Perera; Ooi Mong How; Tonnii Sia Loong Loong
EPI_ISL_12739784, EPI_ISL_12739786	Kota Bharu Public Health Laboratory	Molecular Research Laboratory	Abdul Haris bin Muhammad; Ahmad Sukari Bin Halim; Alexander Chong Shu Chien; Asraihan Che Abdul Malik; Azian Harun; Chan Yean Yean; Chua Wei Chuan; Farahana binti Mohamed; Kirnpal Kaur Banga Singh; Lau Nyok Sean; Lee Lih Huey; Lim Shu Yong; Maizun binti Mohd Zain; Mera Edora Binti Abdul Manap; Mohd Iman hafiz Bin Ibrahim; Mohd Nadzri Abu Yazid; Mohd Zulkifli Salleh; Muhamad Khairul Amirin Bin Zulkifli; Muhammad Azamuddeen bin Mohammad Nasir; Muhammad Fazli bin Khalid; Muhammad Nashrul Farhan Samsudin; Muhammad Zarul Hanifah Bin Md Zoqratt; Nik Zuraina binti Nik Mohd Noor; Noor Hafizan binti Mat Salleh; Nor Amizara Binti Azami; Nor Suhana Binti Mohd Satar; Nur Syuhada Binti Abdul Rahim; Nur-Leem Binti Murshid; Nurfadhlina Musa; Qasim Ayub; Rosline Hassan; Sadequr Rahman; Siti Nur Aisyah Binti Mohamad Sham; Syahida binti Omar; Wan Mohd Zahiruddin Wan Mohammad; Wardah Yusof; Zaini bin Hussin; Zakuan Zainy Deris
EPI_ISL_11966114, EPI_ISL_11966115, EPI_ISL_11966116, EPI_ISL_11966121, EPI_ISL_11966127, EPI_ISL_11966128, EPI_ISL_11966132, EPI_ISL_11966133, EPI_ISL_11966139, EPI_ISL_11966140, EPI_ISL_11966146, EPI_ISL_11966147, EPI_ISL_11966152, EPI_ISL_11966153, EPI_ISL_11966157, EPI_ISL_11966161, EPI_ISL_11966162, EPI_ISL_11966165, EPI_ISL_11966166, EPI_ISL_11966172, EPI_ISL_11966173, EPI_ISL_11966176, EPI_ISL_12097411, EPI_ISL_12097417			
see above	Kuala Lumpur International Airport (KLIA) Health Office	Institute for Medical Research, Infectious Disease Research Centre, National Institutes of Health, Ministry of Health Malaysia	Ahmad FA; Ahmad Fazilah NA; Anasir MI; Azizan MA; Kamel K; Mohamad Sukri MZ; Norhisham SN; Ramly N; Robert F; Rosli NR; Suppiah J; Thayan R
EPI_ISL_11965276, EPI_ISL_11965277	Lahad Datu Hospital	Institute for Medical Research, Infectious Disease Research Centre, National Institutes of Health, Ministry of Health Malaysia	Ahmad FA; Ahmad Fazilah NA; Anasir MI; Azizan MA; Kamel K; Mohamad Sukri MZ; Norhisham SN; Ramly N; Robert F; Rosli NR; Suppiah J; Thayan R
EPI_ISL_10512496, EPI_ISL_10512497, EPI_ISL_10512498	Miri Hospital Molecular Diagnostic Lab, Miri	Institute of Health and Community Medicine	Chan Chia Jui; Chua Hock Hin; David Perera; Ooi Mong How; Tonnii Sia Loong Loong
EPI_ISL_10981871	National Forensic Medical Institute (HKL)	Institute for Medical Research, Infectious Disease Research Centre, National Institutes of Health, Ministry of Health Malaysia	Ahmad FA; Ahmad Fazilah NA; Anasir MI; Azizan MA; Kamel K; Mohamad Sukri MZ; Norhisham SN; Ramly N; Robert F; Rosli NR; Suppiah J; Thayan R
EPI_ISL_10759962, EPI_ISL_10759963, EPI_ISL_10759965, EPI_ISL_10759966, EPI_ISL_10759967, EPI_ISL_10759969, EPI_ISL_10759970, EPI_ISL_10759971, EPI_ISL_10759972, EPI_ISL_10759973, EPI_ISL_10759974, EPI_ISL_10759975, EPI_ISL_10759976, EPI_ISL_10759977, EPI_ISL_10759978, EPI_ISL_10759980, EPI_ISL_10759981, EPI_ISL_10759982, EPI_ISL_10759983, EPI_ISL_10759984, EPI_ISL_10760051, EPI_ISL_11332459, EPI_ISL_11332460			
see above	Normah Medical Specialist Centre, Kuching	Institute of Health and Community Medicine	Chan Chia Jui; Chua Hock Hin; David Perera; Ikwannuddin Y; Ooi Mong How; Tonnii Sia Loong Loong
EPI_ISL_11939190, EPI_ISL_11939191, EPI_ISL_11939192, EPI_ISL_12771286	PEJABAT KESIHATAN DAERAH SEBERANG PERAI SELATAN	iPROMISE, UiTM	Ariza Adnan; Fadzilah Mohd Nor; Lim Wai Feng; Mohd Asif Mohd Sukri; Mohd Nur Fakhruzzaman Noorizhab; Mohd Zaki Salleh; Sazzli Shahlan Kassim; Siti Farah Alwani Mohd Nawi; Siti Hamimah Sheikh Abdul Kadir; Teh Lay Kek; Wang Seok Mui
EPI_ISL_10880094	PKD KLUANG	UKM Medical Molecular Biology Institute (UMBI)	Khairun Nur Abd Ghafar; Mira Farzana Mohamad Mokhtar; Muhiddin Ishak; Nor Azila Muhammad Azami; Nur Alyaa Afifah Md Shahri; Nurul Syakima Ab Mutalib; Rahman Jamal; Ryia Illani Mohd Yunos; Siti Nur Hasanah Mohd Yusuf; Zahirrah Begam Mohamed Rasheed
EPI_ISL_12008463	PKD KULAI	UKM Medical Molecular Biology Institute (UMBI)	Khairun Nur Abd Ghafar; Mira Farzana Mohamad Mokhtar; Muhiddin Ishak; Nor Azila Muhammad Azami; Nur Alyaa Afifah Md Shahri; Nurul Syakima Ab Mutalib; Rahman Jamal; Ryia Illani Mohd Yunos; Siti Nur Hasanah Mohd Yusuf; Zahirrah Begam Mohamed Rasheed
EPI_ISL_12008475	PKD MUAR	UKM Medical Molecular Biology Institute (UMBI)	Khairun Nur Abd Ghafar; Mira Farzana Mohamad Mokhtar; Muhiddin Ishak; Nor Azila Muhammad Azami; Nur Alyaa Afifah Md Shahri; Nurul Syakima Ab Mutalib; Rahman Jamal; Ryia Illani Mohd Yunos; Siti Nur Hasanah Mohd Yusuf; Zahirrah Begam Mohamed Rasheed
EPI_ISL_11939207, EPI_ISL_11939208, EPI_ISL_11939209, EPI_ISL_11939210, EPI_ISL_11939211, EPI_ISL_11939212, EPI_ISL_11939213, EPI_ISL_11939214, EPI_ISL_11939215, EPI_ISL_11939216, EPI_ISL_11939217, EPI_ISL_11939218, EPI_ISL_11939219, EPI_ISL_11939220, EPI_ISL_12771287			
see above	PKD TIMUR LAUT	iPROMISE, UiTM	Ariza Adnan; Fadzilah Mohd Nor; Lim Wai Feng; Mohd Asif Mohd Sukri; Mohd Nur Fakhruzzaman Noorizhab; Mohd Zaki Salleh; Sazzli Shahlan Kassim; Siti Farah Alwani Mohd Nawi; Siti Hamimah Sheikh Abdul Kadir; Teh Lay Kek; Wang Seok Mui
EPI_ISL_12135985, EPI_ISL_12135986, EPI_ISL_12135987	Pejabat Kesihatan Daerah Hulu Perak	Tropical Infectious Diseases Research & Education Centre (TIDREC), Universiti Malaya	AsmaAnati CheMatSeri; Che-Norainon Yaacob; Jia-Yi Tan; Jo-Ern Wong; Kim-Kee Tan; Mulya-Mustika-Sari Zulkifli; Noor Syahida Azizan; Nur-Hidayana Mahfodz; Sazaly AbuBakar; Siti-Sarah Nor'e; Wei-Wei Aw
EPI_ISL_11939154, EPI_ISL_11939155, EPI_ISL_11939156	Pejabat Kesihatan Pintu Masuk Antarabangsa	iPROMISE, UiTM	Ariza Adnan; Fadzilah Mohd Nor; Lim Wai Feng; Mohd Asif Mohd Sukri; Mohd Nur Fakhruzzaman Noorizhab; Mohd Zaki Salleh; Sazzli Shahlan Kassim; Siti Farah Alwani Mohd Nawi; Siti Hamimah Sheikh Abdul Kadir; Teh Lay Kek; Wang Seok Mui
EPI_ISL_11105873	Pitas District Health Office	Institute for Medical Research, Infectious Disease Research Centre, National Institutes of Health, Ministry of Health Malaysia	Ahmad FA; Ahmad Fazilah NA; Anasir MI; Azizan MA; G. Adypatti NM; Jamaluddin MS; Kamel K; Mohamad Sukri MZ; Norhisham SN; Ramly N; Robert F; Rosli NR; Suppiah J; Thayan R
EPI_ISL_11965279, EPI_ISL_11965280, EPI_ISL_12097428	Putrajaya Health Office	Institute for Medical Research, Infectious Disease Research Centre, National Institutes of Health, Ministry of Health Malaysia	Ahmad FA; Ahmad Fazilah NA; Anasir MI; Azizan MA; Kamel K; Mohamad Sukri MZ; Norhisham SN; Ramly N; Robert F; Rosli NR; Suppiah J; Thayan R
EPI_ISL_12289501, EPI_ISL_12289507, EPI_ISL_12289508, EPI_ISL_12289509	QUEEN ELIZABETH HOSPITAL	Institute for Medical Research, Infectious Disease Research Centre, National Institutes of Health, Ministry of Health Malaysia	Ahmad FA; Ahmad Fazilah NA; Anasir MI; Azizan MA; Kamel K; Mohamad Sukri MZ; Norhisham SN; Ramly N; Robert F; Rosli NR; Suppiah J; Thayan R
EPI_ISL_11685213, EPI_ISL_11965315, EPI_ISL_12084288, EPI_ISL_12084291, EPI_ISL_12084293, EPI_ISL_12084295, EPI_ISL_12084296			
see above	Queen Elizabeth Hospital	Institute for Medical Research, Infectious Disease Research Centre, National Institutes of Health, Ministry of Health Malaysia	Ahmad FA; Ahmad Fazilah NA; Anasir MI; Azizan MA; G. Adypatti NM; Jamaluddin MS; Kamel K; Mohamad Sukri MZ; Norhisham SN; Ramly N; Robert F; Rosli NR; Suppiah J; Thayan R
EPI_ISL_10759933, EPI_ISL_10759934, EPI_ISL_10759935, EPI_ISL_10759936, EPI_ISL_10759937, EPI_ISL_10759938, EPI_ISL_10759939			
see above	Rejang Medical Centre (Sibu)	Institute of Health and Community Medicine	Chan Chia Jui; Chua Hock Hin; David Perera; Ooi Mong How; Tonnii Sia Loong Loong; Wong Jyn Shan
EPI_ISL_10512432, EPI_ISL_10512449, EPI_ISL_10512450, EPI_ISL_10512452, EPI_ISL_10512453, EPI_ISL_10512454, EPI_ISL_10512455, EPI_ISL_10512456, EPI_ISL_10512457, EPI_ISL_10512458, EPI_ISL_10512459, EPI_ISL_10512460, EPI_ISL_10512461, EPI_ISL_10512462, EPI_ISL_10512463, EPI_ISL_10512464, EPI_ISL_10512465, EPI_ISL_10512466, EPI_ISL_10512467, EPI_ISL_10512468, EPI_ISL_10512470, EPI_ISL_10512472, EPI_ISL_10512473, EPI_ISL_10512474, EPI_ISL_10512475, EPI_ISL_10512476, EPI_ISL_10512477, EPI_ISL_10512479, EPI_ISL_10512480, EPI_ISL_10512481, EPI_ISL_10512483, EPI_ISL_10512484, EPI_ISL_10512485, EPI_ISL_10512486, EPI_ISL_10512487, EPI_ISL_10512488, EPI_ISL_10512489, EPI_ISL_11332467, EPI_ISL_11332468, EPI_ISL_11332469			
see above	Sarawak General Hospital (Kuching)	Institute of Health and Community Medicine	Chan Chia Jui; Chua Hock Hin; David Perera; Liew Chin Chin J; Ooi Mong How; Tonnii Sia Loong Loong; Wong Jyn Shan
EPI_ISL_10981866	Selayang Hospital	Institute for Medical Research,	Ahmad FA; Ahmad Fazilah NA; Anasir MI; Azizan MA; Kamel K; Mohamad Sukri MZ; Norhisham SN; Ramly N; Robert F; Rosli NR; Suppiah J; Thayan R

		Infectious Disease Research Centre, National Institutes of Health, Ministry of Health Malaysia	
EPI_ISL_11332550, EPI_ISL_11332551, EPI_ISL_11332552, EPI_ISL_11332553, EPI_ISL_11332554, EPI_ISL_11332555, EPI_ISL_11332556, EPI_ISL_11332557, EPI_ISL_11332558, EPI_ISL_11332559, EPI_ISL_11332561, EPI_ISL_11332562, EPI_ISL_11332563, EPI_ISL_11332564, EPI_ISL_11332565, EPI_ISL_11332622, EPI_ISL_11332623, EPI_ISL_11332624, EPI_ISL_11332625, EPI_ISL_11332631, EPI_ISL_11332632, EPI_ISL_11332633, EPI_ISL_11332634, EPI_ISL_11332635, EPI_ISL_11332636			
see above	Sibu Hospital (Sibu, Sarawak)	Institute of Health and Community Medicine	Chan Chia Jui; Chua Hock Hin; David Perera; Ooi Mong How; Tonnii Sia Loong Loong
EPI_ISL_13026007, EPI_ISL_13026008, EPI_ISL_13026009, EPI_ISL_13026010, EPI_ISL_13026011, EPI_ISL_13026012, EPI_ISL_13026013, EPI_ISL_13026014, EPI_ISL_13026015, EPI_ISL_13026016, EPI_ISL_13026017, EPI_ISL_13026019, EPI_ISL_13026020, EPI_ISL_13026023, EPI_ISL_13026024, EPI_ISL_13026027, EPI_ISL_13026031, EPI_ISL_13026052, EPI_ISL_13026053, EPI_ISL_13026054, EPI_ISL_13026055, EPI_ISL_13026056, EPI_ISL_13026057, EPI_ISL_13026058			
see above	Synapse Sdn. Bhd.	Universiti Putra Malaysia (UPM)	Choo Yee Yu; Hui Jen Soe; Hui Yee Chee; Nancy Woan Charn Liew; Narcisse Joseph; Nurulfiza Mat Isa; Rachna Kairon; Sie Yeng Wong; Syafinaz Amin-Nordin; Zunita Zakaria
EPI_ISL_12746985	TAWAU HOSPITAL	Institute for Medical Research, Infectious Disease Research Centre, National Institutes of Health, Ministry of Health Malaysia	Ahmad FA; Ahmad Fazilah NA; Anasir MI; Azizan MA; Kamel K; Mohamad Sukri MZ; Norhisham SN; Ramly N; Robert F; Rosli NR; Suppiah J; Thayan R
EPI_ISL_11105742, EPI_ISL_11105743, EPI_ISL_11105744	Telok Datok Health Clinic	Institute for Medical Research, Infectious Disease Research Centre, National Institutes of Health, Ministry of Health Malaysia	Ahmad FA; Ahmad Fazilah NA; Anasir MI; Azizan MA; G. Adypatti NM; Jamaluddin MS; Kamel K; Mohamad Sukri MZ; Norhisham SN; Ramly N; Robert F; Rosli NR; Suppiah J; Thayan R
EPI_ISL_11965281, EPI_ISL_11965284	Titiwangsa District Health Office	Institute for Medical Research, Infectious Disease Research Centre, National Institutes of Health, Ministry of Health Malaysia	Ahmad FA; Ahmad Fazilah NA; Anasir MI; Azizan MA; Kamel K; Mohamad Sukri MZ; Norhisham SN; Ramly N; Robert F; Rosli NR; Suppiah J; Thayan R
EPI_ISL_12055330, EPI_ISL_12055348, EPI_ISL_12055352, EPI_ISL_12055375, EPI_ISL_12055377	UKA	Institut Biologi Molekul Perubatan UKM (UMBI)	Khairun Nur Abd Ghafar; Mira Farzana Mohamad Mokhtar; Muhiddin Ishak; Nor Azila Muhammad Azami; Nur Alyaa Afifah Md Shahri; Nurul Syakima Ab Mutalib; Rahman Jamal; Ryia Illani Mohd Yunos; Siti Nur Hasanah Mohd Yusuf; Zahirrah Begam Mohamed Rasheed
EPI_ISL_12055320, EPI_ISL_12055335, EPI_ISL_12055337	UKA HEBHK	Institut Biologi Molekul Perubatan UKM (UMBI)	Khairun Nur Abd Ghafar; Mira Farzana Mohamad Mokhtar; Muhiddin Ishak; Nor Azila Muhammad Azami; Nur Alyaa Afifah Md Shahri; Nurul Syakima Ab Mutalib; Rahman Jamal; Ryia Illani Mohd Yunos; Siti Nur Hasanah Mohd Yusuf; Zahirrah Begam Mohamed Rasheed
EPI_ISL_11754707, EPI_ISL_11754708, EPI_ISL_11754710, EPI_ISL_11754763, EPI_ISL_11754770, EPI_ISL_11754774	UMMC	Department of Medical Microbiology, Faculty of Medicine, University of Malaya; University of Malaya Medical Centre	I-Ching SAM; Jolene Yin Ling FU; Omar Khalilur Rahman; Yoke Fun Chan
EPI_ISL_12008467, EPI_ISL_12008468, EPI_ISL_12008470, EPI_ISL_12008479, EPI_ISL_12008497, EPI_ISL_12008503, EPI_ISL_12008504, EPI_ISL_12008519, EPI_ISL_12008520, EPI_ISL_12008526			
see above	UNIT KESIHATAN AWAM HEBHK	UKM Medical Molecular Biology Institute (UMBI)	Khairun Nur Abd Ghafar; Mira Farzana Mohamad Mokhtar; Muhiddin Ishak; Nor Azila Muhammad Azami; Nur Alyaa Afifah Md Shahri; Nurul Syakima Ab Mutalib; Rahman Jamal; Ryia Illani Mohd Yunos; Siti Nur Hasanah Mohd Yusuf; Zahirrah Begam Mohamed Rasheed
EPI_ISL_12055322, EPI_ISL_12055325, EPI_ISL_12055350	Unit Kesihatan Awam Hebhk	Institut Biologi Molekul Perubatan UKM (UMBI)	Khairun Nur Abd Ghafar; Mira Farzana Mohamad Mokhtar; Muhiddin Ishak; Nor Azila Muhammad Azami; Nur Alyaa Afifah Md Shahri; Nurul Syakima Ab Mutalib; Rahman Jamal; Ryia Illani Mohd Yunos; Siti Nur Hasanah Mohd Yusuf; Zahirrah Begam Mohamed Rasheed
EPI_ISL_12055339	Unit Kesihatan Hebhk	Institut Biologi Molekul Perubatan UKM (UMBI)	Khairun Nur Abd Ghafar; Mira Farzana Mohamad Mokhtar; Muhiddin Ishak; Nor Azila Muhammad Azami; Nur Alyaa Afifah Md Shahri; Nurul Syakima Ab Mutalib; Rahman Jamal; Ryia Illani Mohd Yunos; Siti Nur Hasanah Mohd Yusuf; Zahirrah Begam Mohamed Rasheed
EPI_ISL_11939162	jabatan forensik , hospital pulau pinang	iPROMISE, UiTM	Ariza Adnan; Fadzilah Mohd Nor; Lim Wai Feng; Mohd Asif Mohd Sukri; Mohd Nur Fakhruzzaman Noorizhab; Mohd Zaki Salleh; Sazzli Shahlan Kassim; Siti Farah Alwani Mohd Nawi; Siti Hamimah Sheikh Abdul Kadir; Teh Lay Kek; Wang Seok Mui
EPI_ISL_12055318	uka hebhk	Institut Biologi Molekul Perubatan UKM (UMBI)	Khairun Nur Abd Ghafar; Mira Farzana Mohamad Mokhtar; Muhiddin Ishak; Nor Azila Muhammad Azami; Nur Alyaa Afifah Md Shahri; Nurul Syakima Ab Mutalib; Rahman Jamal; Ryia Illani Mohd Yunos; Siti Nur Hasanah Mohd Yusuf; Zahirrah Begam Mohamed Rasheed

We gratefully acknowledge the following Authors from the Originating laboratories responsible for obtaining the specimens, as well as the Submitting laboratories where the genome data were generated and shared via GISAID, on which this research is based.

All Submitters of data may be contacted directly via www.gisaid.org

Authors are sorted alphabetically.

Accession ID	Originating Laboratory	Submitting Laboratory	Authors
EPI_ISL_10981881, EPI_ISL_10981889, EPI_ISL_10981893, EPI_ISL_10981895, EPI_ISL_10981899	Ampang Hospital	Institute for Medical Research, Infectious Disease Research Centre, National Institutes of Health, Ministry of Health Malaysia	Ahmad FA; Ahmad Fazilah NA; Anasir MI; Azizan MA; Kamel K; Mohamad Sukri MZ; Norhisham SN; Ramly N; Robert F; Rosli NR; Suppiah J; Thayan R
EPI_ISL_11332590, EPI_ISL_11332592, EPI_ISL_11332596, EPI_ISL_11332598, EPI_ISL_11332599, EPI_ISL_11332604, EPI_ISL_11332608, EPI_ISL_11332609, EPI_ISL_11332612, EPI_ISL_11332613, EPI_ISL_11332621			
see above	Bintulu Hospital PCR Lab, Bintulu	Institute of Health and Community Medicine	Chan Chia Jui; Chua Hock Hin; David Perera; Ooi Mong How; Tan Lee See; Tonnii Sia Loong Loong; Wong Jyn Shan
EPI_ISL_10760133, EPI_ISL_10760134, EPI_ISL_10760135, EPI_ISL_10760136, EPI_ISL_10760137, EPI_ISL_10760138, EPI_ISL_10760139, EPI_ISL_10760140, EPI_ISL_10760141, EPI_ISL_10760142, EPI_ISL_10760143, EPI_ISL_10760144, EPI_ISL_11332566, EPI_ISL_11332567, EPI_ISL_11332568, EPI_ISL_11332570, EPI_ISL_11332571, EPI_ISL_11332572, EPI_ISL_11332573, EPI_ISL_11332574, EPI_ISL_11332575, EPI_ISL_11332576, EPI_ISL_11332577, EPI_ISL_11332578, EPI_ISL_11332579, EPI_ISL_11332580, EPI_ISL_11332581, EPI_ISL_11332582, EPI_ISL_11332583, EPI_ISL_11804998, EPI_ISL_11805003, EPI_ISL_11805007, EPI_ISL_11805009			
see above	Bintulu Medical Centre (Bintulu)	Institute of Health and Community Medicine	Chan Chia Jui; Chua Hock Hin; David Perera; Ooi Mong How; Tonnii Sia Loong Loong; Wong Jyn Shan
EPI_ISL_10759908, EPI_ISL_10759909, EPI_ISL_10759910, EPI_ISL_10759911, EPI_ISL_10759912, EPI_ISL_10759913, EPI_ISL_10759914, EPI_ISL_10759915, EPI_ISL_10759916, EPI_ISL_10759917, EPI_ISL_10759918, EPI_ISL_10759919, EPI_ISL_10759920, EPI_ISL_10759921, EPI_ISL_10759922, EPI_ISL_10759923, EPI_ISL_10759924, EPI_ISL_10759925, EPI_ISL_10759928, EPI_ISL_11332584, EPI_ISL_11332585, EPI_ISL_11332586, EPI_ISL_11332587, EPI_ISL_11332588, EPI_ISL_11332591, EPI_ISL_11332593, EPI_ISL_11332594, EPI_ISL_11332595, EPI_ISL_11332597, EPI_ISL_11332600, EPI_ISL_11332601, EPI_ISL_11332602, EPI_ISL_11332603, EPI_ISL_11332605, EPI_ISL_11332606, EPI_ISL_11332610, EPI_ISL_11332611, EPI_ISL_11332615, EPI_ISL_11332616, EPI_ISL_11332617, EPI_ISL_11332618			
see above	Borneo Medical Centre (Kuching)	Institute of Health and Community Medicine	Chan Chia Jui; Chua Hock Hin; David Perera; Ooi Mong How; Tonnii Sia Loong Loong; Wong Jyn Shan
EPI_ISL_11105544, EPI_ISL_11105554, EPI_ISL_11105588, EPI_ISL_11105603, EPI_ISL_11105604, EPI_ISL_11105734	Bukit Changgang Health Clinic	Institute for Medical Research, Infectious Disease Research Centre, National Institutes of Health, Ministry of Health Malaysia	Ahmad FA; Ahmad Fazilah NA; Anasir MI; Azizan MA; G. Adypatti NM; Jamaluddin MS; Kamel K; Mohamad Sukri MZ; Norhisham SN; Ramly N; Robert F; Rosli NR; Suppiah J; Thayan R
EPI_ISL_12289490, EPI_ISL_12749677	DISTRICT HEALTH OFFICE TENOM	Institute for Medical Research, Infectious Disease Research Centre, National Institutes of Health, Ministry of Health Malaysia	Ahmad FA; Ahmad Fazilah NA; Anasir MI; Azizan MA; Kamel K; Mohamad Sukri MZ; Norhisham SN; Ramly N; Robert F; Rosli NR; Suppiah J; Thayan R
EPI_ISL_12721596, EPI_ISL_12721597, EPI_ISL_12721598, EPI_ISL_12721599, EPI_ISL_12721600, EPI_ISL_12721602, EPI_ISL_12721603, EPI_ISL_12721604, EPI_ISL_12721605, EPI_ISL_12721606, EPI_ISL_12721607, EPI_ISL_12721608, EPI_ISL_12721609			
see above	Department of Medical Microbiology, Hospital Pengajar Universiti Putra Malaysia	Department of Medical Microbiology, Hospital Pengajar Universiti Putra Malaysia	Afiqah Adzmi; Amiza Azmi; Azmiza Syawani Jasni; Hui Yee Chee; Leslie Thian Lung Than; Muadz Mohtar; Muhammad Mohd Isa; Narcisse MS Joseph; Niazlin Mohd Taib; Noor Hazirah Noor Azhari; Norlaila; Nur Raihana Ithnin; Nurul Huda Mohamed Rashidi; Nurul Nadiah Ismail; Rosni Ibrahim; Rukman Awang Hamat; Sallehhudin; Siti Norbaya Masri; Siti Zulaikha Zakariah; Syafinaz Amin Nordin; Tengku Zetty Maztura Tengku Jamaluddin; Zamberi Sekawi
EPI_ISL_10760000, EPI_ISL_10760001, EPI_ISL_10760002, EPI_ISL_10760003, EPI_ISL_10760004, EPI_ISL_10760005, EPI_ISL_10760006, EPI_ISL_10760007, EPI_ISL_10760008, EPI_ISL_10760009, EPI_ISL_10760010, EPI_ISL_10760011, EPI_ISL_10760012, EPI_ISL_10760013, EPI_ISL_10760014, EPI_ISL_10760015, EPI_ISL_10760016, EPI_ISL_10760017, EPI_ISL_10760018, EPI_ISL_10760019, EPI_ISL_10760022, EPI_ISL_10760023, EPI_ISL_10760024, EPI_ISL_10760025, EPI_ISL_10760026, EPI_ISL_10760027, EPI_ISL_10760028, EPI_ISL_10760029, EPI_ISL_10760030, EPI_ISL_10760052, EPI_ISL_10760053, EPI_ISL_10760054, EPI_ISL_10760055, EPI_ISL_10760057, EPI_ISL_10760059, EPI_ISL_10760060, EPI_ISL_10760061, EPI_ISL_10760062, EPI_ISL_10760063, EPI_ISL_10760064, EPI_ISL_10760065, EPI_ISL_10760067, EPI_ISL_10760068, EPI_ISL_10760069, EPI_ISL_10760070, EPI_ISL_10760071, EPI_ISL_10760072, EPI_ISL_10760073, EPI_ISL_10760074, EPI_ISL_10760075, EPI_ISL_10760076, EPI_ISL_10760077, EPI_ISL_10760078, EPI_ISL_10760079, EPI_ISL_10760080, EPI_ISL_10760081, EPI_ISL_10760082, EPI_ISL_10760083, EPI_ISL_10760084, EPI_ISL_10760085			
see above	Gribbles Diagnostic Lab @Columbia Asia Hospital, Miri	Institute of Health and Community Medicine	Chan Chia Jui; Chua Hock Hin; David Perera; Liew Chin Chin J; Ooi Mong How; Tonnii Sia Loong Loong; Wong Jyn Shan
EPI_ISL_10942297, EPI_ISL_10942355	HCTM	UKM Medical Molecular Biology Institute (UMBI)	Khairun Nur Abd Ghafar; Mira Farzana Mohamad Mokhtar; Muhiddin Ishak; Nor Azila Muhammad Azami; Nur Alyaa Afifah Md Shahri; Nurul Syakima Ab Mutalib; Rahman Jamal; Ryia Illani Mohd Yunos; Siti Nur Hasanah Mohd Yusuf; Zahirrah Begam Mohamed Rasheed
EPI_ISL_12055370	HOSPITAL MELAKA	Institut Biologi Molekul Perubatan UKM (UMBI)	Khairun Nur Abd Ghafar; Mira Farzana Mohamad Mokhtar; Muhiddin Ishak; Nor Azila Muhammad Azami; Nur Alyaa Afifah Md Shahri; Nurul Syakima Ab Mutalib; Rahman Jamal; Ryia Illani Mohd Yunos; Siti Nur Hasanah Mohd Yusuf; Zahirrah Begam Mohamed Rasheed
EPI_ISL_12771289	HOSPITAL PULAU PINANG	iPROMISE, UiTM	Ariza Adnan; Fadzilah Mohd Nor; Lim Wai Feng; Mohd Asif Mohd Sukri; Mohd Nur Fakhruzzaman Noorizhab; Mohd Zaki Salleh; Sazzli Shahlan Kassim; Siti Farah Alwani Mohd Nawi; Siti Hamimah Sheikh Abdul Kadir; Teh Lay Kek; Wang Seok Mui
EPI_ISL_12135959	HOSPITAL RAJA PERMAISURI BAINUN	Tropical Infectious Diseases Research & Education Centre (TIDREC), Universiti Malaya	AsmaAnati CheMatSeri; Che-Norainon Yaacob; Jia-Yi Tan; Jo-Ern Wong; Kim-Kee Tan; Mulya-Mustika-Sari Zulkifli; Noor Syahida Azizan; Nur-Hidayana Mahfodz; Sazaly AbuBakar; Siti-Sarah Nor'e; Wei-Wei Aw
EPI_ISL_12135995, EPI_ISL_12135996, EPI_ISL_12135997, EPI_ISL_12135998, EPI_ISL_12135999, EPI_ISL_12136000	HOSPITAL SULTANAH NUR ZAHIRAH	Tropical Infectious Diseases Research & Education Centre (TIDREC), Universiti Malaya	AsmaAnati CheMatSeri; Che-Norainon Yaacob; Jia-Yi Tan; Jo-Ern Wong; Kim-Kee Tan; Mulya-Mustika-Sari Zulkifli; Noor Syahida Azizan; Nur-Hidayana Mahfodz; Sazaly AbuBakar; Siti-Sarah Nor'e; Wei-Wei Aw
EPI_ISL_11939189	HOSPITAL TUANKU FAUZIAH KANGAR PERLIS	iPROMISE, UiTM	Ariza Adnan; Fadzilah Mohd Nor; Lim Wai Feng; Mohd Asif Mohd Sukri; Mohd Nur Fakhruzzaman Noorizhab; Mohd Zaki Salleh; Sazzli Shahlan Kassim; Siti Farah Alwani Mohd Nawi; Siti Hamimah Sheikh Abdul Kadir; Teh Lay Kek; Wang Seok Mui
EPI_ISL_12135979, EPI_ISL_12135980, EPI_ISL_12135981, EPI_ISL_12135982, EPI_ISL_12135983, EPI_ISL_12135984	HRPB	Tropical Infectious Diseases Research & Education Centre (TIDREC), Universiti Malaya	AsmaAnati CheMatSeri; Che-Norainon Yaacob; Jia-Yi Tan; Jo-Ern Wong; Kim-Kee Tan; Mulya-Mustika-Sari Zulkifli; Noor Syahida Azizan; Nur-Hidayana Mahfodz; Sazaly AbuBakar; Siti-Sarah Nor'e; Wei-Wei Aw
EPI_ISL_12136003	HSAH	Tropical Infectious Diseases Research & Education Centre (TIDREC), Universiti Malaya	AsmaAnati CheMatSeri; Che-Norainon Yaacob; Jia-Yi Tan; Jo-Ern Wong; Kim-Kee Tan; Mulya-Mustika-Sari Zulkifli; Noor Syahida Azizan; Nur-Hidayana Mahfodz; Sazaly AbuBakar; Siti-Sarah Nor'e; Wei-Wei Aw
EPI_ISL_12008455	HSAJB	UKM Medical Molecular Biology Institute (UMBI)	Khairun Nur Abd Ghafar; Mira Farzana Mohamad Mokhtar; Muhiddin Ishak; Nor Azila Muhammad Azami; Nur Alyaa Afifah Md Shahri; Nurul Syakima Ab Mutalib; Rahman Jamal; Ryia Illani Mohd Yunos; Siti Nur Hasanah Mohd Yusuf; Zahirrah Begam Mohamed Rasheed
EPI_ISL_12055374	HTJS	Institut Biologi Molekul Perubatan UKM (UMBI)	Khairun Nur Abd Ghafar; Mira Farzana Mohamad Mokhtar; Muhiddin Ishak; Nor Azila Muhammad Azami; Nur Alyaa Afifah Md Shahri; Nurul Syakima Ab Mutalib; Rahman Jamal; Ryia Illani Mohd Yunos; Siti Nur Hasanah Mohd Yusuf; Zahirrah Begam Mohamed Rasheed
EPI_ISL_12708565	Hospita Sultan Ismail Petra	Molecular Research Laboratory	Abdul Haris bin Muhammad; Ahmad Sukari Bin Halim; Alexander Chong Shu Chien; Asraihan Che Abdul Malik; Azian Harun; Chan Yean Yean; Chua Wei Chuan; Farahana binti Mohamed; Kirnpal Kaur Banga Singh; Lau Nyok Sean; Lee Lih Huey; Lim Shu Yong; Maizun binti Mohd Zain; Mera Edora Binti Abdul Manap; Mohd Iman hafiz Bin Ibrahim; Mohd Nadzri Abu Yazid; Mohd Zulkifli Salleh; Muhamad Khairul Amirin Bin Zulkifli; Muhammad Azamuddeen bin Mohammad Nasir; Muhammad Fazli bin Khalid; Muhammad Nashrul Farhan Samsudin; Muhammad Zarul Hanifah Bin Md Zoqratt; Nik Zuraina binti Nik Mohd Noor; Noor Hafizan binti Mat Salleh; Nor Amizara Binti Azami; Nor Suhana Binti Mohd Satar; Nur Syuhada Binti Abdul Rahim; Nur-Leem Binti Murshid; Nurfadhlina Musa; Qasim Ayub; Rosline Hassan; Sadequr Rahman; Siti Nur Aisyah Binti Mohamad Sham; Syahida binti Omar; Wan Mohd Zahiruddin Wan Mohammad; Wardah Yusof; Zaini bin Hussin; Zakuan Zainy Deris
EPI_ISL_11939163, EPI_ISL_12771202, EPI_ISL_12771288	Hospital Pulau Pinang	iPROMISE, UiTM	Ariza Adnan; Fadzilah Mohd Nor; Lim Wai Feng; Mohd Asif Mohd Sukri; Mohd Nur Fakhruzzaman Noorizhab; Mohd Zaki Salleh; Sazzli Shahlan Kassim; Siti Farah Alwani Mohd Nawi; Siti Hamimah Sheikh Abdul Kadir; Teh Lay Kek; Wang Seok Mui
EPI_ISL_12739785, EPI_ISL_12739789, EPI_ISL_12739790, EPI_ISL_12739792, EPI_ISL_12739794	Hospital Raja Perempuan Zainab II, Kota Bharu	Molecular Research Laboratory	Abdul Haris bin Muhammad; Ahmad Sukari Bin Halim; Alexander Chong Shu Chien; Asraihan Che Abdul Malik; Azian Harun; Chan Yean Yean; Chua Wei Chuan; Farahana binti Mohamed; Kirnpal Kaur Banga Singh; Lau Nyok Sean; Lee Lih Huey; Lim Shu Yong; Maizun binti Mohd Zain; Mera Edora Binti Abdul Manap; Mohd Iman hafiz Bin Ibrahim; Mohd Nadzri Abu Yazid; Mohd Zulkifli Salleh; Muhamad Khairul Amirin Bin Zulkifli; Muhammad Azamuddeen bin Mohammad Nasir; Muhammad Fazli bin Khalid; Muhammad Nashrul Farhan Samsudin; Muhammad Zarul Hanifah Bin Md Zoqratt; Nik Zuraina binti Nik Mohd Noor; Noor Hafizan binti Mat Salleh; Nor Amizara Binti Azami; Nor Suhana Binti Mohd Satar; Nur Syuhada Binti Abdul Rahim; Nur-Leem Binti Murshid; Nurfadhlina Musa; Qasim Ayub; Rosline Hassan; Sadequr Rahman; Siti Nur Aisyah Binti Mohamad Sham; Syahida binti Omar; Wan Mohd Zahiruddin Wan Mohammad; Wardah Yusof; Zaini bin Hussin; Zakuan Zainy Deris
EPI_ISL_10981898	Hospital Sungai Buloh	Institute for Medical Research, Infectious Disease Research Centre, National Institutes of Health, Ministry of Health Malaysia	Ahmad FA; Ahmad Fazilah NA; Anasir MI; Azizan MA; Kamel K; Mohamad Sukri MZ; Norhisham SN; Ramly N; Robert F; Rosli NR; Suppiah J; Thayan R
EPI_ISL_11323756,	Institut Biologi Molekul	UKM Medical Molecular Biology	Khairun Nur Abd Ghafar; Mira Farzana Mohamad Mokhtar; Muhiddin Ishak; Nor Azila Muhammad Azami; Nur Alyaa Afifah Md Shahri; Nurul Syakima Ab Mutalib; Rahman Jamal; Ryia Illani Mohd Yunos; Siti Nur Hasanah Mohd Yusuf; Zahirrah Begam Mohamed Rasheed

EPI_ISL_11323758, EPI_ISL_11323759, EPI_ISL_11323767, EPI_ISL_11323801, EPI_ISL_11323828	Perubatan UKM (UMBI)	Institute (UMBI)	
EPI_ISL_11939224, EPI_ISL_12771166, EPI_ISL_12771235, EPI_ISL_12771249, EPI_ISL_12771296	JKN PAHANG	iPROMISE, UiTM	Ariza Adnan; Fadzilah Mohd Nor; Lim Wai Feng; Mohd Asif Mohd Sukri; Mohd Nur Fakhruzzaman Noorizhab; Mohd Zaki Salleh; Sazzli Shahlan Kassim; Siti Farah Alwani Mohd Nawi; Siti Hamimah Sheikh Abdul Kadir; Teh Lay Kek; Wang Seok Mui
EPI_ISL_12135970, EPI_ISL_12135971, EPI_ISL_12135972, EPI_ISL_12135990, EPI_ISL_12136008, EPI_ISL_12136009, EPI_ISL_12136010, EPI_ISL_12136011, EPI_ISL_12136012, EPI_ISL_12136013, EPI_ISL_12136015, EPI_ISL_12136016, EPI_ISL_12136017			
see above	Jabatan Forensik Hospital Sultanah Bahiyah	Tropical Infectious Diseases Research & Education Centre (TIDREC), Universiti Malaya	AsmaAnati CheMatSeri; Che-Norainon Yaacob; Jia-Yi Tan; Jo-Ern Wong; Kim-Kee Tan; Mulya-Mustika-Sari Zulkifli; Noor Syahida Azizan; Nur-Hidayana Mahfodz; Sazaly AbuBakar; Siti-Sarah Nor'e; Wei-Wei Aw
EPI_ISL_11105735	Jenjarom Health Clinic	Institute for Medical Research, Infectious Disease Research Centre, National Institutes of Health, Ministry of Health Malaysia	Ahmad FA; Ahmad Fazilah NA; Anasir MI; Azizan MA; G. Adypatti NM; Jamaluddin MS; Kamel K; Mohamad Sukri MZ; Norhisham SN; Ramly N; Robert F; Rosli NR; Suppiah J; Thayan R
EPI_ISL_12899690, EPI_ISL_12899691, EPI_ISL_12899692	KUALA LUMPUR GENERAL HOSPITAL	Institute for Medical Research, Infectious Disease Research Centre, National Institutes of Health, Ministry of Health Malaysia	Ahmad FA; Ahmad Fazilah NA; Anasir MI; Azizan MA; Kamel K; Mohamad Sukri MZ; Norhisham SN; Ramly N; Robert F; Rosli NR; Suppiah J; Thayan R
EPI_ISL_12099635, EPI_ISL_12099636, EPI_ISL_12099639, EPI_ISL_12099640, EPI_ISL_12099641, EPI_ISL_12099642, EPI_ISL_12099643			
see above	Kapit Hospital, PCR lab (Kapit, Sarawak)	Institute of Health and Community Medicine	Chan Chia Jui; Chua Hock Hin; David Perera; Ooi Mong How; Tonnii Sia Loong Loong
EPI_ISL_12704545, EPI_ISL_12704546, EPI_ISL_12739787, EPI_ISL_12739788, EPI_ISL_12980330	Kota Bharu Public Health Laboratory	Molecular Research Laboratory	Abdul Haris bin Muhammad; Ahmad Sukari Bin Halim; Alexander Chong Shu Chien; Asraihan Che Abdul Malik; Azian Harun; Chan Yean Yean; Chua Wei Chuan; Farahana binti Mohamed; Kirnpal Kaur Banga Singh; Lau Nyok Sean; Lee Lih Huey; Lim Shu Yong; Maizun binti Mohd Zain; Mera Edora Binti Abdul Manap; Mohd Iman hafiz Bin Ibrahim; Mohd Nadzri Abu Yazid; Mohd Zulkifli Salleh; Muhamad Khairul Amirin Bin Zulkifli; Muhammad Azamuddeen bin Mohammad Nasir; Muhammad Fazli bin Khalid; Muhammad Nashrul Farhan Samsudin; Muhammad Zarul Hanifah Bin Md Zoqratt; Nik Zuraina binti Nik Mohd Noor; Noor Hafizan binti Mat Salleh; Nor Amizara Binti Azami; Nor Suhana Binti Mohd Satar; Nur Syuhada Binti Abdul Rahim; Nur-Leem Binti Murshid; Nurfadhlina Musa; Qasim Ayub; Rosline Hassan; Sadequr Rahman; Siti Nur Aisyah Binti Mohamad Sham; Syahida binti Omar; Wan Mohd Zahiruddin Wan Mohammad; Wardah Yusof; Zaini bin Hussin; Zakuan Zainy Deris
EPI_ISL_11105871	Kuala Langat District Health Office	Institute for Medical Research, Infectious Disease Research Centre, National Institutes of Health, Ministry of Health Malaysia	Ahmad FA; Ahmad Fazilah NA; Anasir MI; Azizan MA; G. Adypatti NM; Jamaluddin MS; Kamel K; Mohamad Sukri MZ; Norhisham SN; Ramly N; Robert F; Rosli NR; Suppiah J; Thayan R
EPI_ISL_10981886, EPI_ISL_11105889, EPI_ISL_11105890, EPI_ISL_11105891, EPI_ISL_11105892, EPI_ISL_11966117, EPI_ISL_11966119, EPI_ISL_11966122, EPI_ISL_11966129, EPI_ISL_11966134, EPI_ISL_11966135, EPI_ISL_11966141, EPI_ISL_11966148, EPI_ISL_11966154, EPI_ISL_11966163, EPI_ISL_11966167, EPI_ISL_11966168, EPI_ISL_11966174, EPI_ISL_11966177, EPI_ISL_11966178, EPI_ISL_12097412, EPI_ISL_12097413, EPI_ISL_12097414, EPI_ISL_12097415, EPI_ISL_12097418, EPI_ISL_12097420			
see above	Kuala Lumpur International Airport (KLIA) Health Office	Institute for Medical Research, Infectious Disease Research Centre, National Institutes of Health, Ministry of Health Malaysia	Ahmad FA; Ahmad Fazilah NA; Anasir MI; Azizan MA; G. Adypatti NM; Jamaluddin MS; Kamel K; Mohamad Sukri MZ; Norhisham SN; Ramly N; Robert F; Rosli NR; Suppiah J; Thayan R
EPI_ISL_12746945	LEMBAH PANTAI DISTRICT HEALTH OFFICE	Institute for Medical Research, Infectious Disease Research Centre, National Institutes of Health, Ministry of Health Malaysia	Ahmad FA; Ahmad Fazilah NA; Anasir MI; Azizan MA; Kamel K; Mohamad Sukri MZ; Norhisham SN; Ramly N; Robert F; Rosli NR; Suppiah J; Thayan R
EPI_ISL_11332541, EPI_ISL_11332542, EPI_ISL_11332543, EPI_ISL_11332544, EPI_ISL_11332545, EPI_ISL_11332546, EPI_ISL_11332547, EPI_ISL_11332548, EPI_ISL_11332549, EPI_ISL_11804987, EPI_ISL_11804988, EPI_ISL_11804989, EPI_ISL_11804990, EPI_ISL_11804991, EPI_ISL_11804992, EPI_ISL_11804993, EPI_ISL_11804994, EPI_ISL_11804995			
see above	Miri Hospital Molecular Diagnostic Lab, Miri	Institute of Health and Community Medicine	Chan Chia Jui; Chua Hock Hin; David Perera; Ooi Mong How; Tonnii Sia Loong Loong
EPI_ISL_11672768, EPI_ISL_11672769, EPI_ISL_11672770, EPI_ISL_11672771, EPI_ISL_11672772, EPI_ISL_11674139, EPI_ISL_11674140, EPI_ISL_11674141, EPI_ISL_11674142, EPI_ISL_11674143, EPI_ISL_11674144, EPI_ISL_11674145, EPI_ISL_11674146, EPI_ISL_11674147, EPI_ISL_11674148, EPI_ISL_11674149, EPI_ISL_11674150, EPI_ISL_11674151, EPI_ISL_11674152, EPI_ISL_11674153, EPI_ISL_11674154, EPI_ISL_11674155, EPI_ISL_11674156, EPI_ISL_11674157, EPI_ISL_11674158, EPI_ISL_11991758, EPI_ISL_11991759, EPI_ISL_11991760, EPI_ISL_11991761, EPI_ISL_11991762, EPI_ISL_11991763			
see above	National Public Health Laboratory	National Public Health Laboratory	Aimi Shazana Binti Shahir; Kamal Hisham Bin Kamarul Zaman; Muhammad Shahir Bin Ali; Noriah Binti Mohd Yusof; Nur Aina Fatini binti Azaman; Rehan Shuhada Binti Abu Bakar; Selvanesan A/L Sengol; Yu Kie A/P Chem
EPI_ISL_12055378	PKD JOHOR BAHRU	Institut Biologi Molekul Perubatan UKM (UMBI)	Khairun Nur Abd Ghafar; Mira Farzana Mohamad Mokhtar; Muhiddin Ishak; Nor Azila Muhammad Azami; Nur Alyaa Afifah Md Shahri; Nurul Syakima Ab Mutalib; Rahman Jamal; Ryia Illani Mohd Yunos; Siti Nur Hasanah Mohd Yusuf; Zahirrah Begam Mohamed Rasheed
EPI_ISL_12099535, EPI_ISL_12099537	PKD KULAI	UKM Medical Molecular Biology Institute (UMBI)	Khairun Nur Abd Ghafar; Mira Farzana Mohamad Mokhtar; Muhiddin Ishak; Nor Azila Muhammad Azami; Nur Alyaa Afifah Md Shahri; Nurul Syakima Ab Mutalib; Rahman Jamal; Ryia Illani Mohd Yunos; Siti Nur Hasanah Mohd Yusuf; Zahirrah Begam Mohamed Rasheed
EPI_ISL_12099543	PKD MUAR	UKM Medical Molecular Biology Institute (UMBI)	Khairun Nur Abd Ghafar; Mira Farzana Mohamad Mokhtar; Muhiddin Ishak; Nor Azila Muhammad Azami; Nur Alyaa Afifah Md Shahri; Nurul Syakima Ab Mutalib; Rahman Jamal; Ryia Illani Mohd Yunos; Siti Nur Hasanah Mohd Yusuf; Zahirrah Begam Mohamed Rasheed
EPI_ISL_12008466	PKD PULAU PINANG	UKM Medical Molecular Biology Institute (UMBI)	Khairun Nur Abd Ghafar; Mira Farzana Mohamad Mokhtar; Muhiddin Ishak; Nor Azila Muhammad Azami; Nur Alyaa Afifah Md Shahri; Nurul Syakima Ab Mutalib; Rahman Jamal; Ryia Illani Mohd Yunos; Siti Nur Hasanah Mohd Yusuf; Zahirrah Begam Mohamed Rasheed
EPI_ISL_11939221, EPI_ISL_11939222, EPI_ISL_11939223, EPI_ISL_12771222, EPI_ISL_12771225, EPI_ISL_12771226, EPI_ISL_12771227, EPI_ISL_12771229, EPI_ISL_12771231, EPI_ISL_12771291			
see above	PKD TIMUR LAUT	iPROMISE, UiTM	Ariza Adnan; Fadzilah Mohd Nor; Lim Wai Feng; Mohd Asif Mohd Sukri; Mohd Nur Fakhruzzaman Noorizhab; Mohd Zaki Salleh; Sazzli Shahlan Kassim; Siti Farah Alwani Mohd Nawi; Siti Hamimah Sheikh Abdul Kadir; Teh Lay Kek; Wang Seok Mui
EPI_ISL_12289479	PULAU PINANG HOSPITAL	Institute for Medical Research, Infectious Disease Research Centre, National Institutes of Health, Ministry of Health Malaysia	Ahmad FA; Ahmad Fazilah NA; Anasir MI; Azizan MA; Kamel K; Mohamad Sukri MZ; Norhisham SN; Ramly N; Robert F; Rosli NR; Suppiah J; Thayan R
EPI_ISL_11629379	Pelabuhan klang health clinic	Institute for Medical Research, Infectious Disease Research Centre, National Institutes of Health, Ministry of Health Malaysia	Ahmad FA; Ahmad Fazilah NA; Anasir MI; Azizan MA; G. Adypatti NM; Jamaluddin MS; Kamel K; Mohamad Sukri MZ; Norhisham SN; Ramly N; Robert F; Rosli NR; Suppiah J; Thayan R
EPI_ISL_12055356, EPI_ISL_12055357	Pkd Johor Bahru	Institut Biologi Molekul Perubatan UKM (UMBI)	Khairun Nur Abd Ghafar; Mira Farzana Mohamad Mokhtar; Muhiddin Ishak; Nor Azila Muhammad Azami; Nur Alyaa Afifah Md Shahri; Nurul Syakima Ab Mutalib; Rahman Jamal; Ryia Illani Mohd Yunos; Siti Nur Hasanah Mohd Yusuf; Zahirrah Begam Mohamed Rasheed
EPI_ISL_11105736, EPI_ISL_11105737, EPI_ISL_11105738, EPI_ISL_11105739, EPI_ISL_11105740, EPI_ISL_11105741	Port Klang Health Clinic	Institute for Medical Research, Infectious Disease Research Centre, National Institutes of Health, Ministry of Health Malaysia	Ahmad FA; Ahmad Fazilah NA; Anasir MI; Azizan MA; G. Adypatti NM; Jamaluddin MS; Kamel K; Mohamad Sukri MZ; Norhisham SN; Ramly N; Robert F; Rosli NR; Suppiah J; Thayan R
EPI_ISL_12289491, EPI_ISL_12289495, EPI_ISL_12289496	QUEEN ELIZABETH HOSPITAL	Institute for Medical Research, Infectious Disease Research Centre, National Institutes of Health, Ministry of Health Malaysia	Ahmad FA; Ahmad Fazilah NA; Anasir MI; Azizan MA; Kamel K; Mohamad Sukri MZ; Norhisham SN; Ramly N; Robert F; Rosli NR; Suppiah J; Thayan R
EPI_ISL_11685237, EPI_ISL_12084287	Queen Elizabeth Hospital	Institute for Medical Research, Infectious Disease Research Centre, National Institutes of Health, Ministry of Health Malaysia	Ahmad FA; Ahmad Fazilah NA; Anasir MI; Azizan MA; G. Adypatti NM; Jamaluddin MS; Kamel K; Mohamad Sukri MZ; Norhisham SN; Ramly N; Robert F; Rosli NR; Suppiah J; Thayan R
EPI_ISL_11332495, EPI_ISL_11332498, EPI_ISL_11332499	Rejang Medical Centre (Sibu)	Institute of Health and Community Medicine	Chan Chia Jui; Chua Hock Hin; David Perera; Ooi Mong How; Tonnii Sia Loong Loong; Wong Jyn Shan
EPI_ISL_10759889, EPI_ISL_10759890, EPI_ISL_10759891, EPI_ISL_10759892, EPI_ISL_10759940, EPI_ISL_10759941, EPI_ISL_10759942, EPI_ISL_10759943, EPI_ISL_10759944, EPI_ISL_10759945, EPI_ISL_10759946, EPI_ISL_10759948, EPI_ISL_10759949, EPI_ISL_10759950, EPI_ISL_10759953, EPI_ISL_10759954, EPI_ISL_10759955, EPI_ISL_10759956, EPI_ISL_10759957, EPI_ISL_10759958, EPI_ISL_10759959, EPI_ISL_10759960, EPI_ISL_10760056, EPI_ISL_10760058, EPI_ISL_10760066, EPI_ISL_10760086, EPI_ISL_10760087, EPI_ISL_10760088, EPI_ISL_10760089, EPI_ISL_10760090, EPI_ISL_10760091, EPI_ISL_10760092, EPI_ISL_10760093, EPI_ISL_10760094, EPI_ISL_10760095, EPI_ISL_10760096, EPI_ISL_10760097, EPI_ISL_10760098, EPI_ISL_10760099, EPI_ISL_10760100, EPI_ISL_10760101, EPI_ISL_10760102, EPI_ISL_10760103, EPI_ISL_10760104, EPI_ISL_10760105, EPI_ISL_10760106, EPI_ISL_10760107, EPI_ISL_10760108, EPI_ISL_10760109, EPI_ISL_10760110, EPI_ISL_10760111, EPI_ISL_10760112, EPI_ISL_10760113, EPI_ISL_10760114, EPI_ISL_10760115, EPI_ISL_10760116, EPI_ISL_10760117, EPI_ISL_10760118, EPI_ISL_10760119, EPI_ISL_10760120, EPI_ISL_10760121, EPI_ISL_10760122, EPI_ISL_10760123, EPI_ISL_10760124, EPI_ISL_10760125, EPI_ISL_10760126, EPI_ISL_10760127, EPI_ISL_10760128, EPI_ISL_10760129, EPI_ISL_10760130, EPI_ISL_10760131, EPI_ISL_10760132, EPI_ISL_11332569, EPI_ISL_11332589, EPI_ISL_11332620			
see above	Sarawak General Hospital (Kuching)	Institute of Health and Community Medicine	Chan Chia Jui; Chua Hock Hin; David Perera; Ooi Mong How; Tonnii Sia Loong Loong
EPI_ISL_10760031	Sarawak Heart Center, Samarahan	Institute of Health and Community Medicine	Chan Chia Jui; Chua Hock Hin; David Perera; Ooi Mong How; Tonnii Sia Loong Loong

EPI_ISL_10981865	Selayang Hospital	Institute for Medical Research, Infectious Disease Research Centre, National Institutes of Health, Ministry of Health Malaysia	Ahmad FA; Ahmad Fazilah NA; Anasir MI; Azizan MA; Kamel K; Mohamad Sukri MZ; Norhisham SN; Ramly N; Robert F; Rosli NR; Suppiah J; Thayan R
EPI_ISL_11332637, EPI_ISL_11332638, EPI_ISL_11332639, EPI_ISL_11332640, EPI_ISL_11332641, EPI_ISL_11332642, EPI_ISL_11332643, EPI_ISL_11332644, EPI_ISL_11332645, EPI_ISL_11332646			
see above	Sibu Hospital (Sibu, Sarawak)	Institute of Health and Community Medicine	Chan Chia Jui; Chua Hock Hin; David Perera; Ooi Mong How; Tonnii Sia Loong Loong
EPI_ISL_13026059, EPI_ISL_13026060, EPI_ISL_13026061, EPI_ISL_13026062, EPI_ISL_13026063, EPI_ISL_13026064, EPI_ISL_13026065, EPI_ISL_13026066, EPI_ISL_13026067, EPI_ISL_13026068, EPI_ISL_13026069, EPI_ISL_13026070			
see above	Synapse Sdn. Bhd.	Universiti Putra Malaysia (UPM)	Choo Yee Yu; Hui Jen Soe; Hui Yee Chee; Nancy Woan Charn Liew; Narcisse Joseph; Nurulfiza Mat Isa; Rachna Kairon; Sie Yeng Wong; Syafinaz Amin-Nordin; Zunita Zakaria
EPI_ISL_11105745, EPI_ISL_11105858, EPI_ISL_11105859, EPI_ISL_11105860, EPI_ISL_11629637, EPI_ISL_11629669, EPI_ISL_11629781			
see above	Telok Datok Health Clinic	Institute for Medical Research, Infectious Disease Research Centre, National Institutes of Health, Ministry of Health Malaysia	Ahmad FA; Ahmad Fazilah NA; Anasir MI; Azizan MA; G. Adypatti NM; Jamaluddin MS; Kamel K; Mohamad Sukri MZ; Norhisham SN; Ramly N; Robert F; Rosli NR; Suppiah J; Thayan R
EPI_ISL_11105862, EPI_ISL_11105863, EPI_ISL_11105864, EPI_ISL_11105865, EPI_ISL_11105869, EPI_ISL_11105870, EPI_ISL_11630066, EPI_ISL_11685206, EPI_ISL_11685207			
see above	Telok Panglima Garang Health Clinic	Institute for Medical Research, Infectious Disease Research Centre, National Institutes of Health, Ministry of Health Malaysia	Ahmad FA; Ahmad Fazilah NA; Anasir MI; Azizan MA; G. Adypatti NM; Jamaluddin MS; Kamel K; Mohamad Sukri MZ; Norhisham SN; Ramly N; Robert F; Rosli NR; Suppiah J; Thayan R
EPI_ISL_12055309, EPI_ISL_12055310, EPI_ISL_12055312, EPI_ISL_12055313, EPI_ISL_12055323, EPI_ISL_12055324, EPI_ISL_12055346, EPI_ISL_12055364, EPI_ISL_12055365, EPI_ISL_12055380, EPI_ISL_12055391			
see above	UKA HEBHK	Institut Biologi Molekul Perubatan UKM (UMBI)	Khairun Nur Abd Ghafar; Mira Farzana Mohamad Mokhtar; Muhiddin Ishak; Nor Azila Muhammad Azami; Nur Alyaa Afifah Md Shahri; Nurul Syakima Ab Mutalib; Rahman Jamal; Ryia Illani Mohd Yunos; Siti Nur Hasanah Mohd Yusuf; Zahirrah Begam Mohamed Rasheed
EPI_ISL_11754712, EPI_ISL_11754758, EPI_ISL_11754772	UMMC	Department of Medical Microbiology, Faculty of Medicine, University of Malaya; University of Malaya Medical Centre	I-Ching SAM; Jolene Yin Ling FU; Omar Khalilur Rahman; Yoke Fun Chan
EPI_ISL_12055368, EPI_ISL_12055379	UNIT KESIHATAN AWAM	Institut Biologi Molekul Perubatan UKM (UMBI)	Khairun Nur Abd Ghafar; Mira Farzana Mohamad Mokhtar; Muhiddin Ishak; Nor Azila Muhammad Azami; Nur Alyaa Afifah Md Shahri; Nurul Syakima Ab Mutalib; Rahman Jamal; Ryia Illani Mohd Yunos; Siti Nur Hasanah Mohd Yusuf; Zahirrah Begam Mohamed Rasheed
EPI_ISL_12055334, EPI_ISL_12055360	UNIT MIKROBIOLOGI, HOSPITAL SULTANAH AMINAH JOHOR BAHRU	Institut Biologi Molekul Perubatan UKM (UMBI)	Khairun Nur Abd Ghafar; Mira Farzana Mohamad Mokhtar; Muhiddin Ishak; Nor Azila Muhammad Azami; Nur Alyaa Afifah Md Shahri; Nurul Syakima Ab Mutalib; Rahman Jamal; Ryia Illani Mohd Yunos; Siti Nur Hasanah Mohd Yusuf; Zahirrah Begam Mohamed Rasheed
EPI_ISL_12055319, EPI_ISL_12055321, EPI_ISL_12055386	Unit Mikrobiologi, Hospital Sultanah Aminah Johor Bahru	Institut Biologi Molekul Perubatan UKM (UMBI)	Khairun Nur Abd Ghafar; Mira Farzana Mohamad Mokhtar; Muhiddin Ishak; Nor Azila Muhammad Azami; Nur Alyaa Afifah Md Shahri; Nurul Syakima Ab Mutalib; Rahman Jamal; Ryia Illani Mohd Yunos; Siti Nur Hasanah Mohd Yusuf; Zahirrah Begam Mohamed Rasheed

We gratefully acknowledge the following Authors from the Originating laboratories responsible for obtaining the specimens, as well as the Submitting laboratories where the genome data were generated and shared via GISAID, on which this research is based.

All Submitters of data may be contacted directly via www.gisaid.org

Authors are sorted alphabetically.

Acknowledgement EPI_SET Identifier: EPI_SET_20220603ug

Accession ID	Originating Laboratory	Submitting Laboratory	Authors
EPI_ISL_12322032, EPI_ISL_12322033, EPI_ISL_12322034, EPI_ISL_12322035, EPI_ISL_12322036, EPI_ISL_12322037, EPI_ISL_12322038, EPI_ISL_12322039, EPI_ISL_12322040, EPI_ISL_12322041, EPI_ISL_12322043, EPI_ISL_12322050, EPI_ISL_12322051			
see above	AMPANG HOSPITAL	Institute for Medical Research, Infectious Disease Research Centre, National Institutes of Health, Ministry of Health Malaysia	Ahmad FA; Ahmad Fazilah NA; Anasir MI; Azizan MA; Kamel K; Mohamad Sukri MZ; Norhisham SN; Ramly N; Robert F; Rosli NR; Suppiah J; Thayan R
EPI_ISL_10981862	Ampang Hospital	Institute for Medical Research, Infectious Disease Research Centre, National Institutes of Health, Ministry of Health Malaysia	Ahmad FA; Ahmad Fazilah NA; Anasir MI; Azizan MA; Kamel K; Mohamad Sukri MZ; Norhisham SN; Ramly N; Robert F; Rosli NR; Suppiah J; Thayan R
EPI_ISL_11805023, EPI_ISL_11805025, EPI_ISL_11805026	Bintulu Hospital PCR Lab, Bintulu	Institute of Health and Community Medicine	Chan Chia Jui; Chien Su Lin; Chua Hock Hin; David Perera; Ooi Mong How; Tonnii Sia Loong Loong
EPI_ISL_11805012, EPI_ISL_11805014, EPI_ISL_11805019, EPI_ISL_11805020, EPI_ISL_11805021, EPI_ISL_11805022, EPI_ISL_11805024, EPI_ISL_11805028, EPI_ISL_11805030, EPI_ISL_12099620			
see above	Bintulu Medical Centre (Bintulu)	Institute of Health and Community Medicine	Chan Chia Jui; Chua Hock Hin; David Perera; Ooi Mong How; Tonnii Sia Loong Loong; Wong Jyn Shan
EPI_ISL_12099728, EPI_ISL_12099729, EPI_ISL_12099730, EPI_ISL_12099731, EPI_ISL_12099732, EPI_ISL_12099733, EPI_ISL_12099734, EPI_ISL_12099735, EPI_ISL_12099736, EPI_ISL_12099737, EPI_ISL_12099738, EPI_ISL_12099739			
see above	Borneo Medical Centre (Miri)	Institute of Health and Community Medicine	Chan Chia Jui; Chua Hock Hin; David Perera; Ooi Mong How; Tonnii Sia Loong Loong; Wong Jyn Shan
EPI_ISL_11967514	Cheras District Health Office	Institute for Medical Research, Infectious Disease Research Centre, National Institutes of Health, Ministry of Health Malaysia	Ahmad FA; Ahmad Fazilah NA; Anasir MI; Azizan MA; Kamel K; Mohamad Sukri MZ; Norhisham SN; Ramly N; Robert F; Rosli NR; Suppiah J; Thayan R
EPI_ISL_12721613, EPI_ISL_12721614, EPI_ISL_12721615, EPI_ISL_12721616, EPI_ISL_12721617, EPI_ISL_12721618	Department of Medical Microbiology, Hospital Pengajar Universiti Putra Malaysia	Department of Medical Microbiology, Hospital Pengajar Universiti Putra Malaysia	Afiqah Adzmi; Amiza Azmi; Azmiza Syawani Jasni; Hui Yee Chee; Leslie Thian Lung Than; Muadz Mohtar; Muhammad Mohd Isa; Narcisse MS Joseph; Niazlin Mohd Taib; Noor Hazirah Noor Azhari; Norlaila; Nur Raihana Ithnin; Nurul Huda Mohamed Rashidi; Nurul Nadiah Ismail; Rosni Ibrahim; Rukman Awang Hamat; Sallehhudin; Siti Norbaya Masri; Siti Zulaikha Zakariah; Syafinaz Amin Nordin; Tengku Zetty Maztura Tengku Jamaluddin; Zamberi Sekawi
EPI_ISL_11332487, EPI_ISL_11332496, EPI_ISL_11332497, EPI_ISL_11332516, EPI_ISL_11332520, EPI_ISL_11332532, EPI_ISL_11332560, EPI_ISL_11332614, EPI_ISL_11332619, EPI_ISL_11332647, EPI_ISL_11332648, EPI_ISL_11332649, EPI_ISL_11332650, EPI_ISL_11332651, EPI_ISL_11804880, EPI_ISL_11804881, EPI_ISL_11804882, EPI_ISL_11804884, EPI_ISL_11804885, EPI_ISL_11804886, EPI_ISL_11804887, EPI_ISL_11804889, EPI_ISL_11804956, EPI_ISL_11804957, EPI_ISL_11804961, EPI_ISL_12099634			
see above	Gribbles Diagnostic Lab @Timberland Medical Centre, Kuching	Institute of Health and Community Medicine	Chan Chia Jui; Chua Hock Hin; David Perera; Liew Chin Chin J; Ooi Mong How; Tonnii Sia Loong Loong; Wong Jyn Shan
EPI_ISL_12135989	HOSPITAL KULIM, KEDAH	Tropical Infectious Diseases Research & Education Centre (TIDREC), Universiti Malaya	AsmaAnati CheMatSeri; Che-Norainon Yaacob; Jia-Yi Tan; Jo-Ern Wong; Kim-Kee Tan; Mulya-Mustika-Sari Zulkifli; Noor Syahida Azizan; Nur-Hidayana Mahfodz; Sazaly AbuBakar; Siti-Sarah Nor'e; Wei-Wei Aw
EPI_ISL_12055353, EPI_ISL_12055363	HOSPITAL MELAKA	Institut Biologi Molekul Perubatan UKM (UMBI)	Khairun Nur Abd Ghafar; Mira Farzana Mohamad Mokhtar; Muhiddin Ishak; Nor Azila Muhammad Azami; Nur Alyaa Afifah Md Shahri; Nurul Syakima Ab Mutalib; Rahman Jamal; Ryia Illani Mohd Yunos; Siti Nur Hasanah Mohd Yusuf; Zahirrah Begam Mohamed Rasheed
EPI_ISL_12008454	HOSPITAL MELAKA	UKM Medical Molecular Biology Institute (UMBI)	Khairun Nur Abd Ghafar; Mira Farzana Mohamad Mokhtar; Muhiddin Ishak; Nor Azila Muhammad Azami; Nur Alyaa Afifah Md Shahri; Nurul Syakima Ab Mutalib; Rahman Jamal; Ryia Illani Mohd Yunos; Siti Nur Hasanah Mohd Yusuf; Zahirrah Begam Mohamed Rasheed
EPI_ISL_12771207, EPI_ISL_12771209, EPI_ISL_12771210, EPI_ISL_12771211, EPI_ISL_12771212, EPI_ISL_12771213, EPI_ISL_12771214, EPI_ISL_12771215, EPI_ISL_12771219, EPI_ISL_12771290			
see above	HOSPITAL PULAU PINANG	iPROMISE, UiTM	Ariza Adnan; Fadzilah Mohd Nor; Lim Wai Feng; Mohd Asif Mohd Sukri; Mohd Nur Fakhruzzaman Noorizhab; Mohd Zaki Salleh; Sazzli Shahlan Kassim; Siti Farah Alwani Mohd Nawi; Siti Hamimah Sheikh Abdul Kadir; Teh Lay Kek; Wang Seok Mui
EPI_ISL_12136004	HOSPITAL SULTAN ABDUL HALIM	Tropical Infectious Diseases Research & Education Centre (TIDREC), Universiti Malaya	AsmaAnati CheMatSeri; Che-Norainon Yaacob; Jia-Yi Tan; Jo-Ern Wong; Kim-Kee Tan; Mulya-Mustika-Sari Zulkifli; Noor Syahida Azizan; Nur-Hidayana Mahfodz; Sazaly AbuBakar; Siti-Sarah Nor'e; Wei-Wei Aw
EPI_ISL_12135992	HOSPITAL SULTAN ABDUL HALIM, SG PETANI KEDAH	Tropical Infectious Diseases Research & Education Centre (TIDREC), Universiti Malaya	AsmaAnati CheMatSeri; Che-Norainon Yaacob; Jia-Yi Tan; Jo-Ern Wong; Kim-Kee Tan; Mulya-Mustika-Sari Zulkifli; Noor Syahida Azizan; Nur-Hidayana Mahfodz; Sazaly AbuBakar; Siti-Sarah Nor'e; Wei-Wei Aw
EPI_ISL_13047106, EPI_ISL_13047107	HOSPITAL SULTAN ISMAIL PETRA, KUALA KRAI, KELANTAN	MOLECULAR RESEARCH LABORATORY	Abdul Haris bin Muhammad; Ahmad Sukari Bin Halim; Alexander Chong Shu Chien; Azian Harun; Chan Yean Yean; Chua Wei Chuan; Farahana binti Mohamed; Kirnpal Kaur Banga Singh; Lau Nyok Sean; Lee Lih Huey; Lim Shu Yong; Maizun binti Mohd Zain; Mohd Nadzri Abu Yazid; Mohd Zulkifli Salleh; Muhammad Azamuddeen bin Mohammad Nasir; Muhammad Fazli bin Khalid; Muhammad Nashrul Farhan Samsudin; Muhammad Zarul Hanifah Bin Md Zoqratt; Nik Zuraina binti Nik Mohd Noor; Noor Hafizan binti Mat Salleh; Nurfadhlina Musa; Qasim Ayub; Rosline Hassan; Sadequr Rahman; Syahida binti Omar; Wan Mohd Zahiruddin Wan Mohammad; Wardah Yusof; Zaini bin Hussin; Zakuan Zainy Deris
EPI_ISL_12534878	HOSPITAL SULTANAH MALIHA	Tropical Infectious Diseases Research & Education Centre (TIDREC), Universiti Malaya	AsmaAnati CheMatSeri; Che-Norainon Yaacob; Jia-Yi Tan; Jo-Ern Wong; Kim-Kee Tan; Mulya-Mustika-Sari Zulkifli; Noor Syahida Azizan; Nur-Hidayana Mahfodz; Sazaly AbuBakar; Siti-Sarah Nor'e; Wei-Wei Aw
EPI_ISL_12534876, EPI_ISL_12534877, EPI_ISL_12534879, EPI_ISL_12534880, EPI_ISL_12534881	HOSPITAL SULTANAH MALIHA, LANGKAWI	Tropical Infectious Diseases Research & Education Centre (TIDREC), Universiti Malaya	AsmaAnati CheMatSeri; Che-Norainon Yaacob; Jia-Yi Tan; Jo-Ern Wong; Kim-Kee Tan; Mulya-Mustika-Sari Zulkifli; Noor Syahida Azizan; Nur-Hidayana Mahfodz; Sazaly AbuBakar; Siti-Sarah Nor'e; Wei-Wei Aw
EPI_ISL_12136001, EPI_ISL_12136002, EPI_ISL_12534754, EPI_ISL_12534755, EPI_ISL_12534756, EPI_ISL_12534757	HOSPITAL SULTANAH NUR ZAHIRAH	Tropical Infectious Diseases Research & Education Centre (TIDREC), Universiti Malaya	AsmaAnati CheMatSeri; Che-Norainon Yaacob; Jia-Yi Tan; Jo-Ern Wong; Kim-Kee Tan; Mulya-Mustika-Sari Zulkifli; Noor Syahida Azizan; Nur-Hidayana Mahfodz; Sazaly AbuBakar; Siti-Sarah Nor'e; Wei-Wei Aw
EPI_ISL_12534758	HOSPTUAL SULTANAH NUR ZAHURAH	Tropical Infectious Diseases Research & Education Centre (TIDREC), Universiti Malaya	AsmaAnati CheMatSeri; Che-Norainon Yaacob; Jia-Yi Tan; Jo-Ern Wong; Kim-Kee Tan; Mulya-Mustika-Sari Zulkifli; Noor Syahida Azizan; Nur-Hidayana Mahfodz; Sazaly AbuBakar; Siti-Sarah Nor'e; Wei-Wei Aw
EPI_ISL_12136023, EPI_ISL_12136024, EPI_ISL_12136025, EPI_ISL_12136026, EPI_ISL_12136027, EPI_ISL_12136028, EPI_ISL_12136029			
see above	HRPB	Tropical Infectious Diseases Research & Education Centre (TIDREC), Universiti Malaya	AsmaAnati CheMatSeri; Che-Norainon Yaacob; Jia-Yi Tan; Jo-Ern Wong; Kim-Kee Tan; Mulya-Mustika-Sari Zulkifli; Noor Syahida Azizan; Nur-Hidayana Mahfodz; Sazaly AbuBakar; Siti-Sarah Nor'e; Wei-Wei Aw
EPI_ISL_12055306, EPI_ISL_12055331, EPI_ISL_12055338, EPI_ISL_12055343, EPI_ISL_12055371	HSAJB	Institut Biologi Molekul Perubatan UKM (UMBI)	Khairun Nur Abd Ghafar; Mira Farzana Mohamad Mokhtar; Muhiddin Ishak; Nor Azila Muhammad Azami; Nur Alyaa Afifah Md Shahri; Nurul Syakima Ab Mutalib; Rahman Jamal; Ryia Illani Mohd Yunos; Siti Nur Hasanah Mohd Yusuf; Zahirrah Begam Mohamed Rasheed
EPI_ISL_12099545, EPI_ISL_12213383, EPI_ISL_12213384, EPI_ISL_12213385	HSAJB	UKM Medical Molecular Biology Institute (UMBI)	Khairun Nur Abd Ghafar; Mira Farzana Mohamad Mokhtar; Muhiddin Ishak; Nor Azila Muhammad Azami; Nur Alyaa Afifah Md Shahri; Nurul Syakima Ab Mutalib; Rahman Jamal; Ryia Illani Mohd Yunos; Siti Nur Hasanah Mohd Yusuf; Zahirrah Begam Mohamed Rasheed
EPI_ISL_12055314	HTJ	Institut Biologi Molekul Perubatan	Khairun Nur Abd Ghafar; Mira Farzana Mohamad Mokhtar; Muhiddin Ishak; Nor Azila Muhammad Azami; Nur Alyaa Afifah Md Shahri; Nurul Syakima Ab Mutalib; Rahman Jamal; Ryia Illani Mohd Yunos; Siti Nur Hasanah Mohd Yusuf; Zahirrah Begam Mohamed Rasheed

		UKM (UMBI)	
EPI_ISL_12055315, EPI_ISL_12055345, EPI_ISL_12055362, EPI_ISL_12055385, EPI_ISL_12055390	HTJS	Institut Biologi Molekul Perubatan UKM (UMBI)	Khairun Nur Abd Ghafar; Mira Farzana Mohamad Mokhtar; Muhiddin Ishak; Nor Azila Muhammad Azami; Nur Alyaa Afifah Md Shahri; Nurul Syakima Ab Mutalib; Rahman Jamal; Ryia Illani Mohd Yunos; Siti Nur Hasanah Mohd Yusuf; Zahirrah Begam Mohamed Rasheed
EPI_ISL_12008456, EPI_ISL_12008457, EPI_ISL_12008458, EPI_ISL_12008529	HTJS	UKM Medical Molecular Biology Institute (UMBI)	Khairun Nur Abd Ghafar; Mira Farzana Mohamad Mokhtar; Muhiddin Ishak; Nor Azila Muhammad Azami; Nur Alyaa Afifah Md Shahri; Nurul Syakima Ab Mutalib; Rahman Jamal; Ryia Illani Mohd Yunos; Siti Nur Hasanah Mohd Yusuf; Zahirrah Begam Mohamed Rasheed
EPI_ISL_12708564, EPI_ISL_12708566, EPI_ISL_12708567	Hospita Sultan Ismail Petra	Molecular Research Laboratory	Abdul Haris bin Muhammad; Ahmad Sukari Bin Halim; Alexander Chong Shu Chien; Asraihan Che Abdul Malik; Azian Harun; Chan Yean Yean; Chua Wei Chuan; Farahana binti Mohamed; Kirnpal Kaur Banga Singh; Lau Nyok Sean; Lee Lih Huey; Lim Shu Yong; Maizun binti Mohd Zain; Mera Edora Binti Abdul Manap; Mohd Iman hafiz Bin Ibrahim; Mohd Nadzri Abu Yazid; Mohd Zulkifli Salleh; Muhamad Khairul Amirin Bin Zulkifli; Muhammad Azamuddeen bin Mohammad Nasir; Muhammad Fazli bin Khalid; Muhammad Nashrul Farhan Samsudin; Muhammad Zarul Hanifah Bin Md Zoqratt; Nik Zuraina binti Nik Mohd Noor; Noor Hafizan binti Mat Salleh; Nor Amizara Binti Azami; Nor Suhana Binti Mohd Satar; Nur Syuhada Binti Abdul Rahim; Nur-Leem Binti Murshid; Nurfadhlina Musa; Qasim Ayub; Rosline Hassan; Sadequr Rahman; Siti Nur Aisyah Binti Mohamad Sham; Syahida binti Omar; Wan Mohd Zahiruddin Wan Mohammad; Wardah Yusof; Zaini bin Hussin; Zakuan Zainy Deris
EPI_ISL_12055311, EPI_ISL_12055392	Hospital Melaka	Institut Biologi Molekul Perubatan UKM (UMBI)	Khairun Nur Abd Ghafar; Mira Farzana Mohamad Mokhtar; Muhiddin Ishak; Nor Azila Muhammad Azami; Nur Alyaa Afifah Md Shahri; Nurul Syakima Ab Mutalib; Rahman Jamal; Ryia Illani Mohd Yunos; Siti Nur Hasanah Mohd Yusuf; Zahirrah Begam Mohamed Rasheed
EPI_ISL_12771204, EPI_ISL_12771208	Hospital Pulau Pinang	iPROMISE, UiTM	Ariza Adnan; Fadzilah Mohd Nor; Lim Wai Feng; Mohd Asif Mohd Sukri; Mohd Nur Fakhruzzaman Noorizhab; Mohd Zaki Salleh; Sazzli Shahlan Kassim; Siti Farah Alwani Mohd Nawi; Siti Hamimah Sheikh Abdul Kadir; Teh Lay Kek; Wang Seok Mui
EPI_ISL_12739793, EPI_ISL_12739795, EPI_ISL_12739796	Hospital Raja Perempuan Zainab II, Kota Bharu	Molecular Research Laboratory	Abdul Haris bin Muhammad; Ahmad Sukari Bin Halim; Alexander Chong Shu Chien; Asraihan Che Abdul Malik; Azian Harun; Chan Yean Yean; Chua Wei Chuan; Farahana binti Mohamed; Kirnpal Kaur Banga Singh; Lau Nyok Sean; Lee Lih Huey; Lim Shu Yong; Maizun binti Mohd Zain; Mera Edora Binti Abdul Manap; Mohd Iman hafiz Bin Ibrahim; Mohd Nadzri Abu Yazid; Mohd Zulkifli Salleh; Muhamad Khairul Amirin Bin Zulkifli; Muhammad Azamuddeen bin Mohammad Nasir; Muhammad Fazli bin Khalid; Muhammad Nashrul Farhan Samsudin; Muhammad Zarul Hanifah Bin Md Zoqratt; Nik Zuraina binti Nik Mohd Noor; Noor Hafizan binti Mat Salleh; Nor Amizara Binti Azami; Nor Suhana Binti Mohd Satar; Nur Syuhada Binti Abdul Rahim; Nur-Leem Binti Murshid; Nurfadhlina Musa; Qasim Ayub; Rosline Hassan; Sadequr Rahman; Siti Nur Aisyah Binti Mohamad Sham; Syahida binti Omar; Wan Mohd Zahiruddin Wan Mohammad; Wardah Yusof; Zaini bin Hussin; Zakuan Zainy Deris
EPI_ISL_10981861	Hospital Sungai Buloh	Institute for Medical Research, Infectious Disease Research Centre, National Institutes of Health, Ministry of Health Malaysia	Ahmad FA; Ahmad Fazilah NA; Anasir MI; Azizan MA; Kamel K; Mohamad Sukri MZ; Norhisham SN; Ramly N; Robert F; Rosli NR; Suppiah J; Thayan R
EPI_ISL_11323752, EPI_ISL_11323760, EPI_ISL_11323765, EPI_ISL_11323780, EPI_ISL_11323786, EPI_ISL_11323791, EPI_ISL_11323797, EPI_ISL_11323803, EPI_ISL_11323813, EPI_ISL_11323815, EPI_ISL_11323819, EPI_ISL_11323838			
see above	Institut Biologi Molekul Perubatan UKM (UMBI)	UKM Medical Molecular Biology Institute (UMBI)	Khairun Nur Abd Ghafar; Mira Farzana Mohamad Mokhtar; Muhiddin Ishak; Nor Azila Muhammad Azami; Nur Alyaa Afifah Md Shahri; Nurul Syakima Ab Mutalib; Rahman Jamal; Ryia Illani Mohd Yunos; Siti Nur Hasanah Mohd Yusuf; Zahirrah Begam Mohamed Rasheed
EPI_ISL_10981887	Institute for Medical Research	Institute for Medical Research, Infectious Disease Research Centre, National Institutes of Health, Ministry of Health Malaysia	Ahmad FA; Ahmad Fazilah NA; Anasir MI; Azizan MA; Kamel K; Mohamad Sukri MZ; Norhisham SN; Ramly N; Robert F; Rosli NR; Suppiah J; Thayan R
EPI_ISL_12771234, EPI_ISL_12771295	JKN PAHANG	iPROMISE, UiTM	Ariza Adnan; Fadzilah Mohd Nor; Lim Wai Feng; Mohd Asif Mohd Sukri; Mohd Nur Fakhruzzaman Noorizhab; Mohd Zaki Salleh; Sazzli Shahlan Kassim; Siti Farah Alwani Mohd Nawi; Siti Hamimah Sheikh Abdul Kadir; Teh Lay Kek; Wang Seok Mui
EPI_ISL_12135988, EPI_ISL_12135991, EPI_ISL_12136014, EPI_ISL_12136018, EPI_ISL_12136019, EPI_ISL_12136020, EPI_ISL_12136021, EPI_ISL_12136022			
see above	Jabatan Forensik Hospital Sultanah Bahiyah	Tropical Infectious Diseases Research & Education Centre (TIDREC), Universiti Malaya	AsmaAnati CheMatSeri; Che-Norainon Yaacob; Jia-Yi Tan; Jo-Ern Wong; Kim-Kee Tan; Mulya-Mustika-Sari Zulkifli; Noor Syahida Azizan; Nur-Hidayana Mahfodz; Sazaly AbuBakar; Siti-Sarah Nor'e; Wei-Wei Aw
EPI_ISL_11967515, EPI_ISL_12097311	Jenjarom Health Clinic	Institute for Medical Research, Infectious Disease Research Centre, National Institutes of Health, Ministry of Health Malaysia	Ahmad FA; Ahmad Fazilah NA; Anasir MI; Azizan MA; Kamel K; Mohamad Sukri MZ; Norhisham SN; Ramly N; Robert F; Rosli NR; Suppiah J; Thayan R
EPI_ISL_12899673	KEPALA BATAS HOSPITAL	Institute for Medical Research, Infectious Disease Research Centre, National Institutes of Health, Ministry of Health Malaysia	Ahmad FA; Ahmad Fazilah NA; Anasir MI; Azizan MA; Kamel K; Mohamad Sukri MZ; Norhisham SN; Ramly N; Robert F; Rosli NR; Suppiah J; Thayan R
EPI_ISL_12322026	KUALA LUMPUR HOSPITAL	Institute for Medical Research, Infectious Disease Research Centre, National Institutes of Health, Ministry of Health Malaysia	Ahmad FA; Ahmad Fazilah NA; Anasir MI; Azizan MA; Kamel K; Mohamad Sukri MZ; Norhisham SN; Ramly N; Robert F; Rosli NR; Suppiah J; Thayan R
EPI_ISL_12289473	KUALA PENYU HOSPITAL	Institute for Medical Research, Infectious Disease Research Centre, National Institutes of Health, Ministry of Health Malaysia	Ahmad FA; Ahmad Fazilah NA; Anasir MI; Azizan MA; Kamel K; Mohamad Sukri MZ; Norhisham SN; Ramly N; Robert F; Rosli NR; Suppiah J; Thayan R
EPI_ISL_12899714	KULIM HOSPITAL	Institute for Medical Research, Infectious Disease Research Centre, National Institutes of Health, Ministry of Health Malaysia	Ahmad FA; Ahmad Fazilah NA; Anasir MI; Azizan MA; Kamel K; Mohamad Sukri MZ; Norhisham SN; Ramly N; Robert F; Rosli NR; Suppiah J; Thayan R
EPI_ISL_11967493, EPI_ISL_11967511	Kampung Bandar Health Clinic	Institute for Medical Research, Infectious Disease Research Centre, National Institutes of Health, Ministry of Health Malaysia	Ahmad FA; Ahmad Fazilah NA; Anasir MI; Azizan MA; Kamel K; Mohamad Sukri MZ; Norhisham SN; Ramly N; Robert F; Rosli NR; Suppiah J; Thayan R
EPI_ISL_12099571, EPI_ISL_12099644, EPI_ISL_12099645, EPI_ISL_12099646, EPI_ISL_12099647, EPI_ISL_12099648, EPI_ISL_12099649, EPI_ISL_12099722			
see above	Kapit Hospital, PCR lab (Kapit, Sarawak)	Institute of Health and Community Medicine	Chan Chia Jui; Chua Hock Hin; David Perera; Ooi Mong How; Tonnii Sia Loong Loong
EPI_ISL_11967499, EPI_ISL_11967512, EPI_ISL_11967513, EPI_ISL_11967516	Kepong District Health Office	Institute for Medical Research, Infectious Disease Research Centre, National Institutes of Health, Ministry of Health Malaysia	Ahmad FA; Ahmad Fazilah NA; Anasir MI; Azizan MA; Kamel K; Mohamad Sukri MZ; Norhisham SN; Ramly N; Robert F; Rosli NR; Suppiah J; Thayan R
EPI_ISL_12704547, EPI_ISL_12704548	Ketereh Health Clinic	Molecular Research Laboratory	Abdul Haris bin Muhammad; Ahmad Sukari Bin Halim; Alexander Chong Shu Chien; Asraihan Che Abdul Malik; Azian Harun; Chan Yean Yean; Chua Wei Chuan; Farahana binti Mohamed; Kirnpal Kaur Banga Singh; Lau Nyok Sean; Lee Lih Huey; Lim Shu Yong; Maizun binti Mohd Zain; Mera Edora Binti Abdul Manap; Mohd Iman hafiz Bin Ibrahim; Mohd Nadzri Abu Yazid; Mohd Zulkifli Salleh; Muhamad Khairul Amirin Bin Zulkifli; Muhammad Azamuddeen bin Mohammad Nasir; Muhammad Fazli bin Khalid; Muhammad Nashrul Farhan Samsudin; Muhammad Zarul Hanifah Bin Md Zoqratt; Nik Zuraina binti Nik Mohd Noor; Noor Hafizan binti Mat Salleh; Nor Amizara Binti Azami; Nor Suhana Binti Mohd Satar; Nur Syuhada Binti Abdul Rahim; Nur-Leem Binti Murshid; Nurfadhlina Musa; Qasim Ayub; Rosline Hassan; Sadequr Rahman; Siti Nur Aisyah Binti Mohamad Sham; Syahida binti Omar; Wan Mohd Zahiruddin Wan Mohammad; Wardah Yusof; Zaini bin Hussin; Zakuan Zainy Deris
EPI_ISL_11967465, EPI_ISL_11967466, EPI_ISL_11967467, EPI_ISL_11967468, EPI_ISL_11967470, EPI_ISL_11967471, EPI_ISL_11967472, EPI_ISL_11967473, EPI_ISL_11967474, EPI_ISL_11967475, EPI_ISL_11967476, EPI_ISL_11967477, EPI_ISL_11967479, EPI_ISL_11967480, EPI_ISL_11967481, EPI_ISL_11967482, EPI_ISL_11967483, EPI_ISL_11967484, EPI_ISL_11967485, EPI_ISL_11967501, EPI_ISL_11967502, EPI_ISL_11967503, EPI_ISL_11967504, EPI_ISL_11967505, EPI_ISL_11967506, EPI_ISL_11967507, EPI_ISL_11967520			
see above	Kuala Langat Satellite Prison	Institute for Medical Research, Infectious Disease Research Centre, National Institutes of Health, Ministry of Health Malaysia	Ahmad FA; Ahmad Fazilah NA; Anasir MI; Azizan MA; Kamel K; Mohamad Sukri MZ; Norhisham SN; Ramly N; Robert F; Rosli NR; Suppiah J; Thayan R
EPI_ISL_12008459	MAKMAL KESIHATAN AWAM JOHOR BAHRU	UKM Medical Molecular Biology Institute (UMBI)	Khairun Nur Abd Ghafar; Mira Farzana Mohamad Mokhtar; Muhiddin Ishak; Nor Azila Muhammad Azami; Nur Alyaa Afifah Md Shahri; Nurul Syakima Ab Mutalib; Rahman Jamal; Ryia Illani Mohd Yunos; Siti Nur Hasanah Mohd Yusuf; Zahirrah Begam Mohamed Rasheed
EPI_ISL_12099549	MKAJB	UKM Medical Molecular Biology Institute (UMBI)	Khairun Nur Abd Ghafar; Mira Farzana Mohamad Mokhtar; Muhiddin Ishak; Nor Azila Muhammad Azami; Nur Alyaa Afifah Md Shahri; Nurul Syakima Ab Mutalib; Rahman Jamal; Ryia Illani Mohd Yunos; Siti Nur Hasanah Mohd Yusuf; Zahirrah Begam Mohamed Rasheed
EPI_ISL_12055349	Makmal Molekular, Hsajb	Institut Biologi Molekul Perubatan UKM (UMBI)	Khairun Nur Abd Ghafar; Mira Farzana Mohamad Mokhtar; Muhiddin Ishak; Nor Azila Muhammad Azami; Nur Alyaa Afifah Md Shahri; Nurul Syakima Ab Mutalib; Rahman Jamal; Ryia Illani Mohd Yunos; Siti Nur Hasanah Mohd Yusuf; Zahirrah Begam Mohamed Rasheed
EPI_ISL_12510013	Miri Hospital Molecular Diagnostic Lab, Miri	Institute of Health and Community Medicine	Chan Chia Jui; Chua Hock Hin; David Perera; Ooi Mong How; Tonnii Sia Loong Loong
EPI_ISL_10981894	National Institutes of Health	Institute for Medical Research, Infectious Disease Research Centre, National Institutes of Health, Ministry of Health Malaysia	Ahmad FA; Ahmad Fazilah NA; Anasir MI; Azizan MA; Kamel K; Mohamad Sukri MZ; Norhisham SN; Ramly N; Robert F; Rosli NR; Suppiah J; Thayan R
EPI_ISL_11967500	National Institutes of Health (NIH)	Institute for Medical Research, Infectious Disease Research Centre, National Institutes of Health, Ministry	Ahmad FA; Ahmad Fazilah NA; Anasir MI; Azizan MA; Kamel K; Mohamad Sukri MZ; Norhisham SN; Ramly N; Robert F; Rosli NR; Suppiah J; Thayan R

		of Health Malaysia	
EPI_ISL_11991764	National Public Health Laboratory	National Public Health Laboratory	Aimi Shazana Binti Shahir; Kamal Hisham Bin Kamarul Zaman; Muhammad Shahir Bin Ali; Noriah Binti Mohd Yusof; Nur Aina Fatini binti Azaman; Rehan Shuhada Binti Abu Bakar; Selvanesan A/L Sengol; Yu Kie A/P Chem
EPI_ISL_13026073	Neogenix Laboratoire Sdn Bhd	Universiti Putra Malaysia (UPM)	Chee Sian Kuan; Choo Yee Yu; Hui Yee Chee; Nancy Woan Charn Liew; Narcisse Joseph; Nurulfiza Mat Isa; Sie Yeng Wong; Su Mei Yew; Syafinaz Amin-Nordin; Zunita Zakaria
EPI_ISL_11804890, EPI_ISL_11804891, EPI_ISL_11804892, EPI_ISL_11804893, EPI_ISL_11804894, EPI_ISL_11804895, EPI_ISL_11804896, EPI_ISL_11804897, EPI_ISL_11804898, EPI_ISL_11804899, EPI_ISL_11804900, EPI_ISL_11804901, EPI_ISL_11804902, EPI_ISL_11804903, EPI_ISL_11804904, EPI_ISL_11804905, EPI_ISL_11804906, EPI_ISL_11804908, EPI_ISL_11804909, EPI_ISL_11804910, EPI_ISL_11804912, EPI_ISL_11804913, EPI_ISL_11804914, EPI_ISL_11804915, EPI_ISL_11804916, EPI_ISL_11804917, EPI_ISL_11804918, EPI_ISL_11804919, EPI_ISL_11804920, EPI_ISL_11804921, EPI_ISL_11804922, EPI_ISL_11804925, EPI_ISL_11804926, EPI_ISL_11804928, EPI_ISL_11804929, EPI_ISL_11804931, EPI_ISL_11804932, EPI_ISL_11804933, EPI_ISL_11804934, EPI_ISL_11804935, EPI_ISL_11804936, EPI_ISL_11804937, EPI_ISL_11804938, EPI_ISL_11804939, EPI_ISL_11804940, EPI_ISL_11804941, EPI_ISL_11804942, EPI_ISL_11804943, EPI_ISL_11804944, EPI_ISL_11804945, EPI_ISL_11804946, EPI_ISL_11804947, EPI_ISL_11804948, EPI_ISL_11804949, EPI_ISL_11804950, EPI_ISL_11804951, EPI_ISL_11804952, EPI_ISL_11804953, EPI_ISL_11804954, EPI_ISL_11804955, EPI_ISL_12099667, EPI_ISL_12099668, EPI_ISL_12099746			
see above	Normah Medical Specialist Centre, Kuching	Institute of Health and Community Medicine	Chan Chia Jui; Chua Hock Hin; David Perera; Ikwannuddin Y; Ooi Mong How; Tonnii Sia Loong Loong
EPI_ISL_12213422	PEJABAT KESIHATAN DAERAH MERSING	UKM Medical Molecular Biology Institute (UMBI)	Khairun Nur Abd Ghafar; Mira Farzana Mohamad Mokhtar; Muhiddin Ishak; Nor Azila Muhammad Azami; Nur Alyaa Afifah Md Shahri; Nurul Syakima Ab Mutalib; Rahman Jamal; Ryia Illani Mohd Yunos; Siti Nur Hasanah Mohd Yusuf; Zahirrah Begam Mohamed Rasheed
EPI_ISL_12771239, EPI_ISL_12771240	PEJABAT KESIHATAN DAERAH SEBERANG PERAI SELATAN	iPROMISE, UiTM	Ariza Adnan; Fadzilah Mohd Nor; Lim Wai Feng; Mohd Asif Mohd Sukri; Mohd Nur Fakhruzzaman Noorizhab; Mohd Zaki Salleh; Sazzli Shahlan Kassim; Siti Farah Alwani Mohd Nawi; Siti Hamimah Sheikh Abdul Kadir; Teh Lay Kek; Wang Seok Mui
EPI_ISL_12534807, EPI_ISL_12534808	PKD KERIAN	Tropical Infectious Diseases Research & Education Centre (TIDREC), Universiti Malaya	AsmaAnati CheMatSeri; Che-Norainon Yaacob; Jia-Yi Tan; Jo-Ern Wong; Kim-Kee Tan; Mulya-Mustika-Sari Zulkifli; Noor Syahida Azizan; Nur-Hidayana Mahfodz; Sazaly AbuBakar; Siti-Sarah Nor'e; Wei-Wei Aw
EPI_ISL_12099536, EPI_ISL_12099546, EPI_ISL_12099547	PKD KULAI	UKM Medical Molecular Biology Institute (UMBI)	Khairun Nur Abd Ghafar; Mira Farzana Mohamad Mokhtar; Muhiddin Ishak; Nor Azila Muhammad Azami; Nur Alyaa Afifah Md Shahri; Nurul Syakima Ab Mutalib; Rahman Jamal; Ryia Illani Mohd Yunos; Siti Nur Hasanah Mohd Yusuf; Zahirrah Begam Mohamed Rasheed
EPI_ISL_12213424, EPI_ISL_12213425	PKD MERSING	UKM Medical Molecular Biology Institute (UMBI)	Khairun Nur Abd Ghafar; Mira Farzana Mohamad Mokhtar; Muhiddin Ishak; Nor Azila Muhammad Azami; Nur Alyaa Afifah Md Shahri; Nurul Syakima Ab Mutalib; Rahman Jamal; Ryia Illani Mohd Yunos; Siti Nur Hasanah Mohd Yusuf; Zahirrah Begam Mohamed Rasheed
EPI_ISL_12534806	PKD MUALLIM, Asia / Malaysia / Perak	Tropical Infectious Diseases Research & Education Centre (TIDREC), Universiti Malaya	AsmaAnati CheMatSeri; Che-Norainon Yaacob; Jia-Yi Tan; Jo-Ern Wong; Kim-Kee Tan; Mulya-Mustika-Sari Zulkifli; Noor Syahida Azizan; Nur-Hidayana Mahfodz; Sazaly AbuBakar; Siti-Sarah Nor'e; Wei-Wei Aw
EPI_ISL_12099538, EPI_ISL_12099539, EPI_ISL_12099542, EPI_ISL_12099550	PKD MUAR	UKM Medical Molecular Biology Institute (UMBI)	Khairun Nur Abd Ghafar; Mira Farzana Mohamad Mokhtar; Muhiddin Ishak; Nor Azila Muhammad Azami; Nur Alyaa Afifah Md Shahri; Nurul Syakima Ab Mutalib; Rahman Jamal; Ryia Illani Mohd Yunos; Siti Nur Hasanah Mohd Yusuf; Zahirrah Begam Mohamed Rasheed
EPI_ISL_12771223, EPI_ISL_12771224, EPI_ISL_12771228, EPI_ISL_12771230, EPI_ISL_12771232, EPI_ISL_12771252, EPI_ISL_12771254, EPI_ISL_12771292, EPI_ISL_12771293, EPI_ISL_12771294, EPI_ISL_12771309, EPI_ISL_12771310, EPI_ISL_12771311, EPI_ISL_12771312, EPI_ISL_12771313			
see above	PKD TIMUR LAUT	iPROMISE, UiTM	Ariza Adnan; Fadzilah Mohd Nor; Lim Wai Feng; Mohd Asif Mohd Sukri; Mohd Nur Fakhruzzaman Noorizhab; Mohd Zaki Salleh; Sazzli Shahlan Kassim; Siti Farah Alwani Mohd Nawi; Siti Hamimah Sheikh Abdul Kadir; Teh Lay Kek; Wang Seok Mui
EPI_ISL_12322081	PUTRAJAYA HEALTH OFFICE	Institute for Medical Research, Infectious Disease Research Centre, National Institutes of Health, Ministry of Health Malaysia	Ahmad FA; Ahmad Fazilah NA; Anasir MI; Azizan MA; Kamel K; Mohamad Sukri MZ; Norhisham SN; Ramly N; Robert F; Rosli NR; Suppiah J; Thayan R
EPI_ISL_12771205, EPI_ISL_12771206, EPI_ISL_12771220, EPI_ISL_12771221	Pejabat Kesihatan PMA	iPROMISE, UiTM	Ariza Adnan; Fadzilah Mohd Nor; Lim Wai Feng; Mohd Asif Mohd Sukri; Mohd Nur Fakhruzzaman Noorizhab; Mohd Zaki Salleh; Sazzli Shahlan Kassim; Siti Farah Alwani Mohd Nawi; Siti Hamimah Sheikh Abdul Kadir; Teh Lay Kek; Wang Seok Mui
EPI_ISL_12771203	Pejabat Kesihatan Pintu Masuk Antarabangsa Pulau Pinang	iPROMISE, UiTM	Ariza Adnan; Fadzilah Mohd Nor; Lim Wai Feng; Mohd Asif Mohd Sukri; Mohd Nur Fakhruzzaman Noorizhab; Mohd Zaki Salleh; Sazzli Shahlan Kassim; Siti Farah Alwani Mohd Nawi; Siti Hamimah Sheikh Abdul Kadir; Teh Lay Kek; Wang Seok Mui
EPI_ISL_11967486, EPI_ISL_11967489, EPI_ISL_11967490, EPI_ISL_11967491, EPI_ISL_11967496, EPI_ISL_11967497, EPI_ISL_11967498, EPI_ISL_11967509			
see above	Port Klang Health Clinic	Institute for Medical Research, Infectious Disease Research Centre, National Institutes of Health, Ministry of Health Malaysia	Ahmad FA; Ahmad Fazilah NA; Anasir MI; Azizan MA; Kamel K; Mohamad Sukri MZ; Norhisham SN; Ramly N; Robert F; Rosli NR; Suppiah J; Thayan R
EPI_ISL_11967508, EPI_ISL_11967517, EPI_ISL_11967518	Putrajaya Health Office	Institute for Medical Research, Infectious Disease Research Centre, National Institutes of Health, Ministry of Health Malaysia	Ahmad FA; Ahmad Fazilah NA; Anasir MI; Azizan MA; Kamel K; Mohamad Sukri MZ; Norhisham SN; Ramly N; Robert F; Rosli NR; Suppiah J; Thayan R
EPI_ISL_12289472, EPI_ISL_12289493, EPI_ISL_12289494, EPI_ISL_12289497, EPI_ISL_12289498, EPI_ISL_12289503, EPI_ISL_12289504			
see above	QUEEN ELIZABETH HOSPITAL	Institute for Medical Research, Infectious Disease Research Centre, National Institutes of Health, Ministry of Health Malaysia	Ahmad FA; Ahmad Fazilah NA; Anasir MI; Azizan MA; Kamel K; Mohamad Sukri MZ; Norhisham SN; Ramly N; Robert F; Rosli NR; Suppiah J; Thayan R
EPI_ISL_11685214, EPI_ISL_11685223	Queen Elizabeth Hospital	Institute for Medical Research, Infectious Disease Research Centre, National Institutes of Health, Ministry of Health Malaysia	Ahmad FA; Ahmad Fazilah NA; Anasir MI; Azizan MA; G. Adypatti NM; Jamaluddin MS; Kamel K; Mohamad Sukri MZ; Norhisham SN; Ramly N; Robert F; Rosli NR; Suppiah J; Thayan R
EPI_ISL_11804883, EPI_ISL_11804888, EPI_ISL_11804911, EPI_ISL_11804923, EPI_ISL_11804924, EPI_ISL_11804927	Rejang Medical Centre (Sibu)	Institute of Health and Community Medicine	Chan Chia Jui; Chua Hock Hin; David Perera; Ooi Mong How; Tonnii Sia Loong Loong; Wong Jyn Shan
EPI_ISL_12289477	SELAYANG HOSPITAL	Institute for Medical Research, Infectious Disease Research Centre, National Institutes of Health, Ministry of Health Malaysia	Ahmad FA; Ahmad Fazilah NA; Anasir MI; Azizan MA; Kamel K; Mohamad Sukri MZ; Norhisham SN; Ramly N; Robert F; Rosli NR; Suppiah J; Thayan R
EPI_ISL_12746991, EPI_ISL_12746992, EPI_ISL_12746993, EPI_ISL_12746994, EPI_ISL_12746995, EPI_ISL_12746996, EPI_ISL_12746997, EPI_ISL_12746998, EPI_ISL_12747000, EPI_ISL_12747001, EPI_ISL_12747002, EPI_ISL_12747003, EPI_ISL_12747004, EPI_ISL_12747006, EPI_ISL_12747007, EPI_ISL_12899710			
see above	SULTANAH BAHIYAH HOSPITAL	Institute for Medical Research, Infectious Disease Research Centre, National Institutes of Health, Ministry of Health Malaysia	Ahmad FA; Ahmad Fazilah NA; Anasir MI; Azizan MA; Kamel K; Mohamad Sukri MZ; Norhisham SN; Ramly N; Robert F; Rosli NR; Suppiah J; Thayan R
EPI_ISL_12322027, EPI_ISL_12322028, EPI_ISL_12322029, EPI_ISL_12322030, EPI_ISL_12322031	SUNGAI BULOH HOSPITAL	Institute for Medical Research, Infectious Disease Research Centre, National Institutes of Health, Ministry of Health Malaysia	Ahmad FA; Ahmad Fazilah NA; Anasir MI; Azizan MA; Kamel K; Mohamad Sukri MZ; Norhisham SN; Ramly N; Robert F; Rosli NR; Suppiah J; Thayan R
EPI_ISL_11332476, EPI_ISL_11332477, EPI_ISL_11332478, EPI_ISL_11332479, EPI_ISL_11332480, EPI_ISL_11332481, EPI_ISL_11332482, EPI_ISL_11332483, EPI_ISL_11332484, EPI_ISL_11332485, EPI_ISL_11332486, EPI_ISL_11332488, EPI_ISL_11332489, EPI_ISL_11332490, EPI_ISL_11332491, EPI_ISL_11332492, EPI_ISL_11332493, EPI_ISL_11332494, EPI_ISL_11332500, EPI_ISL_11332501, EPI_ISL_11332502, EPI_ISL_11332503, EPI_ISL_11332504, EPI_ISL_11332505, EPI_ISL_11332506, EPI_ISL_11332507, EPI_ISL_11332508, EPI_ISL_11332509, EPI_ISL_11332510, EPI_ISL_11332511, EPI_ISL_11332512, EPI_ISL_11332513, EPI_ISL_11332514, EPI_ISL_11332515, EPI_ISL_11332517, EPI_ISL_11332518, EPI_ISL_11332519, EPI_ISL_11332521, EPI_ISL_11332522, EPI_ISL_11332523, EPI_ISL_11332524, EPI_ISL_11332525, EPI_ISL_11332526, EPI_ISL_11332527, EPI_ISL_11332528, EPI_ISL_11332529, EPI_ISL_11332530, EPI_ISL_11332531, EPI_ISL_11332533, EPI_ISL_11332534, EPI_ISL_11332535, EPI_ISL_11332536, EPI_ISL_11332537, EPI_ISL_11332538, EPI_ISL_11332539, EPI_ISL_11332540, EPI_ISL_11804975, EPI_ISL_11804976, EPI_ISL_11804978			
see above	Sarawak General Hospital (Kuching)	Institute of Health and Community Medicine	Chan Chia Jui; Chua Hock Hin; David Perera; Ooi Mong How; Tonnii Sia Loong Loong
EPI_ISL_10981872	Selayang Hospital	Institute for Medical Research, Infectious Disease Research Centre, National Institutes of Health, Ministry of Health Malaysia	Ahmad FA; Ahmad Fazilah NA; Anasir MI; Azizan MA; Kamel K; Mohamad Sukri MZ; Norhisham SN; Ramly N; Robert F; Rosli NR; Suppiah J; Thayan R
EPI_ISL_11069890, EPI_ISL_11069891, EPI_ISL_11069892, EPI_ISL_11069893, EPI_ISL_11069894, EPI_ISL_11069895, EPI_ISL_11069896, EPI_ISL_11069897, EPI_ISL_11069898, EPI_ISL_11069899, EPI_ISL_11069900, EPI_ISL_11069901, EPI_ISL_11069902, EPI_ISL_11069903, EPI_ISL_11069904, EPI_ISL_11069905, EPI_ISL_11069906, EPI_ISL_11069907, EPI_ISL_11069908, EPI_ISL_11069909, EPI_ISL_11069910			
see above	Synapse Sdn. Bhd.	Universiti Putra Malaysia (UPM)	Choo Yee Yu; Hui Jen Soe; Hui Yee Chee; Nancy Woan Charn Liew; Narcisse Joseph; Nurulfiza Mat Isa; Rachna Kairon; Sie Yeng Wong; Syafinaz Amin-Nordin; Zunita Zakaria

EPI_ISL_12289500	TAWAU HOSPITAL	Institute for Medical Research, Infectious Disease Research Centre, National Institutes of Health, Ministry of Health Malaysia	Ahmad FA; Ahmad Fazilah NA; Anasir MI; Azizan MA; Kamel K; Mohamad Sukri MZ; Norhisham SN; Ramly N; Robert F; Rosli NR; Suppiah J; Thayan R
EPI_ISL_11967510	Tanjung Sepat Health Clinic	Institute for Medical Research, Infectious Disease Research Centre, National Institutes of Health, Ministry of Health Malaysia	Ahmad FA; Ahmad Fazilah NA; Anasir MI; Azizan MA; Kamel K; Mohamad Sukri MZ; Norhisham SN; Ramly N; Robert F; Rosli NR; Suppiah J; Thayan R
EPI_ISL_11967487, EPI_ISL_11967492	Telok Datok Health Clinic	Institute for Medical Research, Infectious Disease Research Centre, National Institutes of Health, Ministry of Health Malaysia	Ahmad FA; Ahmad Fazilah NA; Anasir MI; Azizan MA; Kamel K; Mohamad Sukri MZ; Norhisham SN; Ramly N; Robert F; Rosli NR; Suppiah J; Thayan R
EPI_ISL_11967469, EPI_ISL_11967488, EPI_ISL_11967494, EPI_ISL_11967495	Telok Panglima Garang Health Clinic	Institute for Medical Research, Infectious Disease Research Centre, National Institutes of Health, Ministry of Health Malaysia	Ahmad FA; Ahmad Fazilah NA; Anasir MI; Azizan MA; Kamel K; Mohamad Sukri MZ; Norhisham SN; Ramly N; Robert F; Rosli NR; Suppiah J; Thayan R
EPI_ISL_11965255	Tuanku Ja'afar Hospital	Institute for Medical Research, Infectious Disease Research Centre, National Institutes of Health, Ministry of Health Malaysia	Ahmad FA; Ahmad Fazilah NA; Anasir MI; Azizan MA; Kamel K; Mohamad Sukri MZ; Norhisham SN; Ramly N; Robert F; Rosli NR; Suppiah J; Thayan R
EPI_ISL_12055354	UKA HEBHK	Institut Biologi Molekul Perubatan UKM (UMBI)	Khairun Nur Abd Ghafar; Mira Farzana Mohamad Mokhtar; Muhiddin Ishak; Nor Azila Muhammad Azami; Nur Alyaa Afifah Md Shahri; Nurul Syakima Ab Mutalib; Rahman Jamal; Ryia Illani Mohd Yunos; Siti Nur Hasanah Mohd Yusuf; Zahirrah Begam Mohamed Rasheed
EPI_ISL_11754773	UMMC	Department of Medical Microbiology, Faculty of Medicine, University of Malaya; University of Malaya Medical Centre	I-Ching SAM; Jolene Yin Ling FU; Omar Khalilur Rahman; Yoke Fun Chan
EPI_ISL_12213397, EPI_ISL_12213407, EPI_ISL_12213412, EPI_ISL_12213413	UNIT KESIHATAN AWAM	UKM Medical Molecular Biology Institute (UMBI)	Khairun Nur Abd Ghafar; Mira Farzana Mohamad Mokhtar; Muhiddin Ishak; Nor Azila Muhammad Azami; Nur Alyaa Afifah Md Shahri; Nurul Syakima Ab Mutalib; Rahman Jamal; Ryia Illani Mohd Yunos; Siti Nur Hasanah Mohd Yusuf; Zahirrah Begam Mohamed Rasheed
EPI_ISL_12213405, EPI_ISL_12213406, EPI_ISL_12213408, EPI_ISL_12213409, EPI_ISL_12213410, EPI_ISL_12213411	UNIT KESIHATAN AWAM HEBHK	UKM Medical Molecular Biology Institute (UMBI)	Khairun Nur Abd Ghafar; Mira Farzana Mohamad Mokhtar; Muhiddin Ishak; Nor Azila Muhammad Azami; Nur Alyaa Afifah Md Shahri; Nurul Syakima Ab Mutalib; Rahman Jamal; Ryia Illani Mohd Yunos; Siti Nur Hasanah Mohd Yusuf; Zahirrah Begam Mohamed Rasheed

We gratefully acknowledge the following Authors from the Originating laboratories responsible for obtaining the specimens, as well as the Submitting laboratories where the genome data were generated and shared via GISAID, on which this research is based.

All Submitters of data may be contacted directly via www.gisaid.org

Authors are sorted alphabetically.

Accession ID	Originating Laboratory	Submitting Laboratory	Authors
EPI_ISL_12322042, EPI_ISL_12322044, EPI_ISL_12322045, EPI_ISL_12322046, EPI_ISL_12322047, EPI_ISL_12322048, EPI_ISL_12322049, EPI_ISL_12322058			
see above	AMPANG HOSPITAL	Institute for Medical Research, Infectious Disease Research Centre, National Institutes of Health, Ministry of Health Malaysia	Ahmad FA; Ahmad Fazilah NA; Anasir MI; Azizan MA; Kamel K; Mohamad Sukri MZ; Norhisham SN; Ramly N; Robert F; Rosli NR; Suppiah J; Thayan R
EPI_ISL_11805027, EPI_ISL_11805029, EPI_ISL_11805031, EPI_ISL_11805032, EPI_ISL_11805033	Bintulu Hospital PCR Lab, Bintulu	Institute of Health and Community Medicine	Chan Chia Jui; Chien Su Lin; Chua Hock Hin; David Perera; Ooi Mong How; Tonnii Sia Loong Loong
EPI_ISL_12510349, EPI_ISL_12739651, EPI_ISL_12739652, EPI_ISL_12739653	Bintulu Medical Centre (Bintulu)	Institute of Health and Community Medicine	Chan Chia Jui; David Perera; Ooi Mong How; Wong Jyn Shan
EPI_ISL_12099727, EPI_ISL_12099740, EPI_ISL_12099741, EPI_ISL_12099742, EPI_ISL_12099743	Borneo Medical Centre (Miri)	Institute of Health and Community Medicine	Chan Chia Jui; Chua Hock Hin; David Perera; Ooi Mong How; Tonnii Sia Loong Loong; Wong Jyn Shan
EPI_ISL_12704536, EPI_ISL_12704542, EPI_ISL_12704543, EPI_ISL_12704544	Department of Medical Microbiology and Parasitology, Hospital Universiti Sains Malaysia	Molecular Research Laboratory	Abdul Haris bin Muhammad; Ahmad Sukari Bin Halim; Alexander Chong Shu Chien; Asraihan Che Abdul Malik; Azian Harun; Chan Yean Yean; Chua Wei Chuan; Farahana binti Mohamed; Kirnpal Kaur Banga Singh; Lau Nyok Sean; Lee Lih Huey; Lim Shu Yong; Maizun binti Mohd Zain; Mera Edora Binti Abdul Manap; Mohd Iman hafiz Bin Ibrahim; Mohd Nadzri Abu Yazid; Mohd Zulkifli Salleh; Muhamad Khairul Amirin Bin Zulkifli; Muhammad Azamuddeen bin Mohammad Nasir; Muhammad Fazli bin Khalid; Muhammad Nashrul Farhan Samsudin; Muhammad Zarul Hanifah Bin Md Zoqratt; Nik Zuraina binti Nik Mohd Noor; Noor Hafizan binti Mat Salleh; Nor Amizara Binti Azami; Nor Suhana Binti Mohd Satar; Nur Syuhada Binti Abdul Rahim; Nur-Leem Binti Murshid; Nurfadhlina Musa; Qasim Ayub; Rosline Hassan; Sadequr Rahman; Siti Nur Aisyah Binti Mohamad Sham; Syahida binti Omar; Wan Mohd Zahiruddin Wan Mohammad; Wardah Yusof; Zaini bin Hussin; Zakuan Zainy Deris
EPI_ISL_12721619, EPI_ISL_12721620, EPI_ISL_12721621	Department of Medical Microbiology, Hospital Pengajar Universiti Putra Malaysia	Department of Medical Microbiology, Hospital Pengajar Universiti Putra Malaysia	Afiqah Adzmi; Amiza Azmi; Azmiza Syawani Jasni; Hui Yee Chee; Leslie Thian Lung Than; Muadz Mohtar; Muhammad Mohd Isa; Narcisse MS Joseph; Niazlin Mohd Taib; Noor Hazirah Noor Azhari; Norlaila; Nur Raihana Ithnin; Nurul Huda Mohamed Rashidi; Nurul Nadiah Ismail; Rosni Ibrahim; Rukman Awang Hamat; Sallehhudin; Siti Norbaya Masri; Siti Zulaikha Zakariah; Syafinaz Amin Nordin; Tengku Zetty Maztura Tengku Jamaluddin; Zamberi Sekawi
EPI_ISL_11804962, EPI_ISL_11804963, EPI_ISL_11804964, EPI_ISL_11804965, EPI_ISL_11804966, EPI_ISL_11804967, EPI_ISL_11804968, EPI_ISL_11804969, EPI_ISL_11804970, EPI_ISL_11804971, EPI_ISL_11804972, EPI_ISL_11804973, EPI_ISL_11804974			
see above	Gribbles Diagnostic Lab @Timberland Medical Centre, Kuching	Institute of Health and Community Medicine	Chan Chia Jui; Chua Hock Hin; David Perera; Liew Chin Chin J; Ooi Mong How; Tonnii Sia Loong Loong; Wong Jyn Shan
EPI_ISL_12628172, EPI_ISL_12628173, EPI_ISL_12628174, EPI_ISL_12628175, EPI_ISL_12628176, EPI_ISL_12628177, EPI_ISL_12628178, EPI_ISL_12628179, EPI_ISL_12628180, EPI_ISL_12628181			
see above	HCTM	UKM Medical Molecular Biology Institute (UMBI)	Khairun Nur Abd Ghafar; Mira Farzana Mohamad Mokhtar; Muhiddin Ishak; Nor Azila Muhammad Azami; Nur Alyaa Afifah Md Shahri; Nurul Syakima Ab Mutalib; Rahman Jamal; Ryia Illani Mohd Yunos; Siti Nur Hasanah Mohd Yusuf; Zahirrah Begam Mohamed Rasheed
EPI_ISL_12771216, EPI_ISL_12771217, EPI_ISL_12771218, EPI_ISL_12771247	HOSPITAL PULAU PINANG	iPROMISE, UiTM	Ariza Adnan; Fadzilah Mohd Nor; Lim Wai Feng; Mohd Asif Mohd Sukri; Mohd Nur Fakhruzzaman Noorizhab; Mohd Zaki Salleh; Sazzli Shahlan Kassim; Siti Farah Alwani Mohd Nawi; Siti Hamimah Sheikh Abdul Kadir; Teh Lay Kek; Wang Seok Mui
EPI_ISL_12534798, EPI_ISL_12534799, EPI_ISL_12534800, EPI_ISL_12534801, EPI_ISL_12534802, EPI_ISL_12534803, EPI_ISL_12534804, EPI_ISL_12534805			
see above	HOSPITAL RAJA PERMAISURI BAINUN, IPOH	Tropical Infectious Diseases Research & Education Centre (TIDREC), Universiti Malaya	AsmaAnati CheMatSeri; Che-Norainon Yaacob; Jia-Yi Tan; Jo-Ern Wong; Kim-Kee Tan; Mulya-Mustika-Sari Zulkifli; Noor Syahida Azizan; Nur-Hidayana Mahfodz; Sazaly AbuBakar; Siti-Sarah Nor'e; Wei-Wei Aw
EPI_ISL_13047108, EPI_ISL_13047109	HOSPITAL SULTAN ISMAIL PETRA, KUALA KRAI, KELANTAN	MOLECULAR RESEARCH LABORATORY	Abdul Haris bin Muhammad; Ahmad Sukari Bin Halim; Alexander Chong Shu Chien; Azian Harun; Chan Yean Yean; Chua Wei Chuan; Farahana binti Mohamed; Kirnpal Kaur Banga Singh; Lau Nyok Sean; Lee Lih Huey; Lim Shu Yong; Maizun binti Mohd Zain; Mohd Nadzri Abu Yazid; Mohd Zulkifli Salleh; Muhammad Azamuddeen bin Mohammad Nasir; Muhammad Fazli bin Khalid; Muhammad Nashrul Farhan Samsudin; Muhammad Zarul Hanifah Bin Md Zoqratt; Nik Zuraina binti Nik Mohd Noor; Noor Hafizan binti Mat Salleh; Nurfadhlina Musa; Qasim Ayub; Rosline Hassan; Sadequr Rahman; Syahida binti Omar; Wan Mohd Zahiruddin Wan Mohammad; Wardah Yusof; Zaini bin Hussin; Zakuan Zainy Deris
EPI_ISL_12534764, EPI_ISL_12534765	HOSPITAL SULTANAH NUR ZAHIRAH	Tropical Infectious Diseases Research & Education Centre (TIDREC), Universiti Malaya	AsmaAnati CheMatSeri; Che-Norainon Yaacob; Jia-Yi Tan; Jo-Ern Wong; Kim-Kee Tan; Mulya-Mustika-Sari Zulkifli; Noor Syahida Azizan; Nur-Hidayana Mahfodz; Sazaly AbuBakar; Siti-Sarah Nor'e; Wei-Wei Aw
EPI_ISL_12213386, EPI_ISL_12213387, EPI_ISL_12213398	HSAJB	UKM Medical Molecular Biology Institute (UMBI)	Khairun Nur Abd Ghafar; Mira Farzana Mohamad Mokhtar; Muhiddin Ishak; Nor Azila Muhammad Azami; Nur Alyaa Afifah Md Shahri; Nurul Syakima Ab Mutalib; Rahman Jamal; Ryia Illani Mohd Yunos; Siti Nur Hasanah Mohd Yusuf; Zahirrah Begam Mohamed Rasheed
EPI_ISL_12771256	Hospital Balik Pulau	iPROMISE, UiTM	Ariza Adnan; Fadzilah Mohd Nor; Lim Wai Feng; Mohd Asif Mohd Sukri; Mohd Nur Fakhruzzaman Noorizhab; Mohd Zaki Salleh; Sazzli Shahlan Kassim; Siti Farah Alwani Mohd Nawi; Siti Hamimah Sheikh Abdul Kadir; Teh Lay Kek; Wang Seok Mui
EPI_ISL_12771301	Hospital Pulau Pinang	iPROMISE, UiTM	Ariza Adnan; Fadzilah Mohd Nor; Lim Wai Feng; Mohd Asif Mohd Sukri; Mohd Nur Fakhruzzaman Noorizhab; Mohd Zaki Salleh; Sazzli Shahlan Kassim; Siti Farah Alwani Mohd Nawi; Siti Hamimah Sheikh Abdul Kadir; Teh Lay Kek; Wang Seok Mui
EPI_ISL_12739797, EPI_ISL_12739798, EPI_ISL_12739799, EPI_ISL_12739800, EPI_ISL_12739801, EPI_ISL_12739802	Hospital Raja Perempuan Zainab II, Kota Bharu	Molecular Research Laboratory	Abdul Haris bin Muhammad; Ahmad Sukari Bin Halim; Alexander Chong Shu Chien; Asraihan Che Abdul Malik; Azian Harun; Chan Yean Yean; Chua Wei Chuan; Farahana binti Mohamed; Kirnpal Kaur Banga Singh; Lau Nyok Sean; Lee Lih Huey; Lim Shu Yong; Maizun binti Mohd Zain; Mera Edora Binti Abdul Manap; Mohd Iman hafiz Bin Ibrahim; Mohd Nadzri Abu Yazid; Mohd Zulkifli Salleh; Muhamad Khairul Amirin Bin Zulkifli; Muhammad Azamuddeen bin Mohammad Nasir; Muhammad Fazli bin Khalid; Muhammad Nashrul Farhan Samsudin; Muhammad Zarul Hanifah Bin Md Zoqratt; Nik Zuraina binti Nik Mohd Noor; Noor Hafizan binti Mat Salleh; Nor Amizara Binti Azami; Nor Suhana Binti Mohd Satar; Nur Syuhada Binti Abdul Rahim; Nur-Leem Binti Murshid; Nurfadhlina Musa; Qasim Ayub; Rosline Hassan; Sadequr Rahman; Siti Nur Aisyah Binti Mohamad Sham; Syahida binti Omar; Wan Mohd Zahiruddin Wan Mohammad; Wardah Yusof; Zaini bin Hussin; Zakuan Zainy Deris
EPI_ISL_12771241, EPI_ISL_12771298	JKN PAHANG	iPROMISE, UiTM	Ariza Adnan; Fadzilah Mohd Nor; Lim Wai Feng; Mohd Asif Mohd Sukri; Mohd Nur Fakhruzzaman Noorizhab; Mohd Zaki Salleh; Sazzli Shahlan Kassim; Siti Farah Alwani Mohd Nawi; Siti Hamimah Sheikh Abdul Kadir; Teh Lay Kek; Wang Seok Mui
EPI_ISL_12289474	KUALA LUMPUR HOSPITAL	Institute for Medical Research, Infectious Disease Research Centre, National Institutes of Health, Ministry of Health Malaysia	Ahmad FA; Ahmad Fazilah NA; Anasir MI; Azizan MA; Kamel K; Mohamad Sukri MZ; Norhisham SN; Ramly N; Robert F; Rosli NR; Suppiah J; Thayan R
EPI_ISL_12899713	KULIM HOSPITAL	Institute for Medical Research, Infectious Disease Research Centre, National Institutes of Health, Ministry of Health Malaysia	Ahmad FA; Ahmad Fazilah NA; Anasir MI; Azizan MA; Kamel K; Mohamad Sukri MZ; Norhisham SN; Ramly N; Robert F; Rosli NR; Suppiah J; Thayan R
EPI_ISL_12099650, EPI_ISL_12099651	Kapit Hospital, PCR lab (Kapit, Sarawak)	Institute of Health and Community Medicine	Chan Chia Jui; Chua Hock Hin; David Perera; Ooi Mong How; Tonnii Sia Loong Loong
EPI_ISL_12708559	Ketereh Health Clinic	Molecular Research Laboratory	Abdul Haris bin Muhammad; Ahmad Sukari Bin Halim; Alexander Chong Shu Chien; Asraihan Che Abdul Malik; Azian Harun; Chan Yean Yean; Chua Wei Chuan; Farahana binti Mohamed; Kirnpal Kaur Banga Singh; Lau Nyok Sean; Lee Lih Huey; Lim Shu Yong; Maizun binti Mohd Zain; Mera Edora Binti Abdul Manap; Mohd Iman hafiz Bin Ibrahim; Mohd Nadzri Abu Yazid; Mohd Zulkifli Salleh; Muhamad Khairul Amirin Bin Zulkifli; Muhammad Azamuddeen bin Mohammad Nasir; Muhammad Fazli bin Khalid; Muhammad Nashrul Farhan Samsudin; Muhammad Zarul Hanifah Bin Md Zoqratt; Nik Zuraina binti Nik Mohd Noor; Noor Hafizan binti Mat Salleh; Nor Amizara Binti Azami; Nor Suhana Binti Mohd Satar; Nur Syuhada Binti Abdul Rahim; Nur-Leem Binti Murshid; Nurfadhlina Musa; Qasim Ayub; Rosline Hassan; Sadequr Rahman; Siti Nur Aisyah Binti Mohamad Sham; Syahida binti Omar; Wan Mohd Zahiruddin Wan Mohammad; Wardah Yusof; Zaini bin Hussin; Zakuan Zainy Deris
EPI_ISL_12175874, EPI_ISL_12175875, EPI_ISL_12175876, EPI_ISL_12175877, EPI_ISL_12175878, EPI_ISL_12175879, EPI_ISL_12175880, EPI_ISL_12175881, EPI_ISL_12175882, EPI_ISL_12175883, EPI_ISL_12175884, EPI_ISL_12175885, EPI_ISL_12175886, EPI_ISL_12175887, EPI_ISL_12175888, EPI_ISL_12175889, EPI_ISL_12175890, EPI_ISL_12175891, EPI_ISL_12175892, EPI_ISL_12175893, EPI_ISL_12175894, EPI_ISL_12175895, EPI_ISL_12175896, EPI_ISL_12175897, EPI_ISL_12175898, EPI_ISL_12175899, EPI_ISL_12175900, EPI_ISL_12175901, EPI_ISL_12175902, EPI_ISL_12175903, EPI_ISL_12175904, EPI_ISL_12175905, EPI_ISL_12175906, EPI_ISL_12175907, EPI_ISL_12175908, EPI_ISL_12175909, EPI_ISL_12175910, EPI_ISL_12175911, EPI_ISL_12175912, EPI_ISL_12175913, EPI_ISL_12175914, EPI_ISL_12175915, EPI_ISL_12175916, EPI_ISL_12175917, EPI_ISL_12175918, EPI_ISL_12175919, EPI_ISL_12175920, EPI_ISL_12175921, EPI_ISL_12175922, EPI_ISL_12175923, EPI_ISL_12175924, EPI_ISL_12175925, EPI_ISL_12175926, EPI_ISL_12175927, EPI_ISL_12175928, EPI_ISL_12175929, EPI_ISL_12175930, EPI_ISL_12175931, EPI_ISL_12175932, EPI_ISL_12175933, EPI_ISL_12175934, EPI_ISL_12175935, EPI_ISL_12175936, EPI_ISL_12175937, EPI_ISL_12175938, EPI_ISL_12175939, EPI_ISL_12175940, EPI_ISL_12175941, EPI_ISL_12175942, EPI_ISL_12180408, EPI_ISL_12180409, EPI_ISL_12180410, EPI_ISL_12180411, EPI_ISL_12180412, EPI_ISL_12180413, EPI_ISL_12180414, EPI_ISL_12180415, EPI_ISL_12180416, EPI_ISL_12180417, EPI_ISL_12180418, EPI_ISL_12180419, EPI_ISL_12180420, EPI_ISL_12180421, EPI_ISL_12180422, EPI_ISL_12180423, EPI_ISL_12180424, EPI_ISL_12180425, EPI_ISL_12180426, EPI_ISL_12180427, EPI_ISL_12180428, EPI_ISL_12180429, EPI_ISL_12180430, EPI_ISL_12180431, EPI_ISL_12180432, EPI_ISL_12180433, EPI_ISL_12180434, EPI_ISL_12180435, EPI_ISL_12180436, EPI_ISL_12180437, EPI_ISL_12180438, EPI_ISL_12180439			
see above	Kuala Lumpur International Airport	Institute for Medical Research, Infectious Disease Research Centre, National Institutes of Health, Ministry of Health Malaysia	Anasir MI; G.Adypatti NM; Jamaluddin MS; Kalyanasundram J; Kamel K; MatRahim N; Nawi MH; Suib FA; Suppiah J; Thayan R
EPI_ISL_12099531, EPI_ISL_12099532,	MAKMAL KESIHATAN AWAM JB	UKM Medical Molecular Biology Institute (UMBI)	Khairun Nur Abd Ghafar; Mira Farzana Mohamad Mokhtar; Muhiddin Ishak; Nor Azila Muhammad Azami; Nur Alyaa Afifah Md Shahri; Nurul Syakima Ab Mutalib; Rahman Jamal; Ryia Illani Mohd Yunos; Siti Nur Hasanah Mohd Yusuf; Zahirrah Begam Mohamed Rasheed

EPI_ISL_12099533, EPI_ISL_12099548			
EPI_ISL_12534882, EPI_ISL_12534883, EPI_ISL_12534884, EPI_ISL_12534885, EPI_ISL_12534886	MAKMAL SEROLOGI, UNIT MIKROBIOLOGI, HSB	Tropical Infectious Diseases Research & Education Centre (TIDREC), Universiti Malaya	AsmaAnati CheMatSeri; Che-Norainon Yaacob; Jia-Yi Tan; Jo-Ern Wong; Kim-Kee Tan; Mulya-Mustika-Sari Zulkifli; Noor Syahida Azizan; Nur-Hidayana Mahfodz; Sazaly AbuBakar; Siti-Sarah Nor'e; Wei-Wei Aw
EPI_ISL_12099534	MKAJB	UKM Medical Molecular Biology Institute (UMBI)	Khairun Nur Abd Ghafar; Mira Farzana Mohamad Mokhtar; Muhiddin Ishak; Nor Azila Muhammad Azami; Nur Alyaa Afifah Md Shahri; Nurul Syakima Ab Mutalib; Rahman Jamal; Ryia Illani Mohd Yunos; Siti Nur Hasanah Mohd Yusuf; Zahirrah Begam Mohamed Rasheed
EPI_ISL_11804997, EPI_ISL_11804999, EPI_ISL_11805000, EPI_ISL_11805002, EPI_ISL_12510020, EPI_ISL_12510041	Miri Hospital Molecular Diagnostic Lab, Miri	Institute of Health and Community Medicine	Chan Chia Jui; Chua Hock Hin; David Perera; Ooi Mong How; Tonnii Sia Loong Loong
EPI_ISL_13026080	Neogenix Laboratoire Sdn Bhd	Universiti Putra Malaysia (UPM)	Chee Sian Kuan; Choo Yee Yu; Hui Yee Chee; Nancy Woan Charn Liew; Narcisse Joseph; Nurulfiza Mat Isa; Sie Yeng Wong; Su Mei Yew; Syafinaz Amin-Nordin; Zunita Zakaria
EPI_ISL_12899675	PAHANG STATE HEALTH DEPARTMENT	Institute for Medical Research, Infectious Disease Research Centre, National Institutes of Health, Ministry of Health Malaysia	Ahmad FA; Ahmad Fazilah NA; Anasir MI; Azizan MA; Kamel K; Mohamad Sukri MZ; Norhisham SN; Ramly N; Robert F; Rosli NR; Suppiah J; Thayan R
EPI_ISL_12213421, EPI_ISL_12213423, EPI_ISL_12213426	PEJABAT KESIHATAN DAERAH MERSING	UKM Medical Molecular Biology Institute (UMBI)	Khairun Nur Abd Ghafar; Mira Farzana Mohamad Mokhtar; Muhiddin Ishak; Nor Azila Muhammad Azami; Nur Alyaa Afifah Md Shahri; Nurul Syakima Ab Mutalib; Rahman Jamal; Ryia Illani Mohd Yunos; Siti Nur Hasanah Mohd Yusuf; Zahirrah Begam Mohamed Rasheed
EPI_ISL_12628142, EPI_ISL_12628143	PKD MUAR	UKM Medical Molecular Biology Institute (UMBI)	Khairun Nur Abd Ghafar; Mira Farzana Mohamad Mokhtar; Muhiddin Ishak; Nor Azila Muhammad Azami; Nur Alyaa Afifah Md Shahri; Nurul Syakima Ab Mutalib; Rahman Jamal; Ryia Illani Mohd Yunos; Siti Nur Hasanah Mohd Yusuf; Zahirrah Begam Mohamed Rasheed
EPI_ISL_12771250, EPI_ISL_12771253	PKD TIMUR LAUT	iPROMISE, UiTM	Ariza Adnan; Fadzilah Mohd Nor; Lim Wai Feng; Mohd Asif Mohd Sukri; Mohd Nur Fakhruzzaman Noorizhab; Mohd Zaki Salleh; Sazzli Shahlan Kassim; Siti Farah Alwani Mohd Nawi; Siti Hamimah Sheikh Abdul Kadir; Teh Lay Kek; Wang Seok Mui
EPI_ISL_12289502, EPI_ISL_12289505, EPI_ISL_12289506	QUEEN ELIZABETH HOSPITAL	Institute for Medical Research, Infectious Disease Research Centre, National Institutes of Health, Ministry of Health Malaysia	Ahmad FA; Ahmad Fazilah NA; Anasir MI; Azizan MA; Kamel K; Mohamad Sukri MZ; Norhisham SN; Ramly N; Robert F; Rosli NR; Suppiah J; Thayan R
EPI_ISL_11804930, EPI_ISL_11804958, EPI_ISL_11804959, EPI_ISL_11804960, EPI_ISL_11804977, EPI_ISL_11804979, EPI_ISL_11804980, EPI_ISL_11804983, EPI_ISL_12099669, EPI_ISL_12099670, EPI_ISL_12099671, EPI_ISL_12099672, EPI_ISL_12099673, EPI_ISL_12099674, EPI_ISL_12099723, EPI_ISL_12510000, EPI_ISL_12510001, EPI_ISL_12510002, EPI_ISL_12510003, EPI_ISL_12510004, EPI_ISL_12510005			
see above	Rejang Medical Centre (Sibu)	Institute of Health and Community Medicine	Chan Chia Jui; Chua Hock Hin; David Perera; Ooi Mong How; Tonnii Sia Loong Loong; Wong Jyn Shan
EPI_ISL_12746990	SULTAN ABDUL HALIM HOSPITAL	Institute for Medical Research, Infectious Disease Research Centre, National Institutes of Health, Ministry of Health Malaysia	Ahmad FA; Ahmad Fazilah NA; Anasir MI; Azizan MA; Kamel K; Mohamad Sukri MZ; Norhisham SN; Ramly N; Robert F; Rosli NR; Suppiah J; Thayan R
EPI_ISL_12289485, EPI_ISL_12746999, EPI_ISL_12747005, EPI_ISL_12899706, EPI_ISL_12899707, EPI_ISL_12899708, EPI_ISL_12899709			
see above	SULTANAH BAHIYAH HOSPITAL	Institute for Medical Research, Infectious Disease Research Centre, National Institutes of Health, Ministry of Health Malaysia	Ahmad FA; Ahmad Fazilah NA; Anasir MI; Azizan MA; Kamel K; Mohamad Sukri MZ; Norhisham SN; Ramly N; Robert F; Rosli NR; Suppiah J; Thayan R
EPI_ISL_12322052, EPI_ISL_12322053, EPI_ISL_12322054, EPI_ISL_12322055, EPI_ISL_12322056, EPI_ISL_12322057	SUNGAI BULOH HOSPITAL	Institute for Medical Research, Infectious Disease Research Centre, National Institutes of Health, Ministry of Health Malaysia	Ahmad FA; Ahmad Fazilah NA; Anasir MI; Azizan MA; Kamel K; Mohamad Sukri MZ; Norhisham SN; Ramly N; Robert F; Rosli NR; Suppiah J; Thayan R
EPI_ISL_12289499, EPI_ISL_12289510, EPI_ISL_12289511	TAWAU HOSPITAL	Institute for Medical Research, Infectious Disease Research Centre, National Institutes of Health, Ministry of Health Malaysia	Ahmad FA; Ahmad Fazilah NA; Anasir MI; Azizan MA; Kamel K; Mohamad Sukri MZ; Norhisham SN; Ramly N; Robert F; Rosli NR; Suppiah J; Thayan R
EPI_ISL_12213414	UNIT KESIHATAN AWAM HEBHK	UKM Medical Molecular Biology Institute (UMBI)	Khairun Nur Abd Ghafar; Mira Farzana Mohamad Mokhtar; Muhiddin Ishak; Nor Azila Muhammad Azami; Nur Alyaa Afifah Md Shahri; Nurul Syakima Ab Mutalib; Rahman Jamal; Ryia Illani Mohd Yunos; Siti Nur Hasanah Mohd Yusuf; Zahirrah Begam Mohamed Rasheed
EPI_ISL_11804878	home_quarantine	Institute of Health and Community Medicine	Chan Chia Jui; David Perera; Ooi Mong How

We gratefully acknowledge the following Authors from the Originating laboratories responsible for obtaining the specimens, as well as the Submitting laboratories where the genome data were generated and shared via GISAID, on which this research is based.

All Submitters of data may be contacted directly via www.gisaid.org

Authors are sorted alphabetically.

Acknowledgement EPI_SET Identifier: EPI_SET_20220603mf

Accession ID	Originating Laboratory	Submitting Laboratory	Authors
EPI_ISL_11805034, EPI_ISL_11805035, EPI_ISL_11805036	Bintulu Hospital PCR Lab, Bintulu	Institute of Health and Community Medicine	Chan Chia Jui; Chien Su Lin; Chua Hock Hin; David Perera; Ooi Mong How; Tonnii Sia Loong Loong
EPI_ISL_12099606, EPI_ISL_12099607, EPI_ISL_12099608, EPI_ISL_12099609	Bintulu Medical Centre (Bintulu)	Institute of Health and Community Medicine	Chan Chia Jui; Chua Hock Hin; David Perera; Ooi Mong How; Tonnii Sia Loong Loong; Wong Jyn Shan
EPI_ISL_12172463, EPI_ISL_12172464, EPI_ISL_12172465, EPI_ISL_12172466, EPI_ISL_12172467, EPI_ISL_12172468, EPI_ISL_12172469, EPI_ISL_12172470, EPI_ISL_12172471, EPI_ISL_12172472, EPI_ISL_12172473, EPI_ISL_12172474, EPI_ISL_12172475, EPI_ISL_12172476, EPI_ISL_12172477, EPI_ISL_12172478, EPI_ISL_12172479, EPI_ISL_12172480, EPI_ISL_12172481, EPI_ISL_12172482, EPI_ISL_12172483, EPI_ISL_12172484, EPI_ISL_12172485, EPI_ISL_12172486, EPI_ISL_12172487, EPI_ISL_12172488, EPI_ISL_12172489, EPI_ISL_12172490, EPI_ISL_12172491, EPI_ISL_12172492, EPI_ISL_12172493, EPI_ISL_12172494, EPI_ISL_12172495, EPI_ISL_12172496, EPI_ISL_12172497, EPI_ISL_12172498, EPI_ISL_12172499, EPI_ISL_12172500, EPI_ISL_12172501, EPI_ISL_12172502, EPI_ISL_12172503, EPI_ISL_12172504, EPI_ISL_12172505, EPI_ISL_12172506, EPI_ISL_12172507, EPI_ISL_12172508, EPI_ISL_12172509, EPI_ISL_12172510, EPI_ISL_12172511, EPI_ISL_12172512, EPI_ISL_12172513, EPI_ISL_12172514, EPI_ISL_12172515, EPI_ISL_12172516, EPI_ISL_12172517, EPI_ISL_12172518, EPI_ISL_12172519, EPI_ISL_12172520, EPI_ISL_12172521, EPI_ISL_12172522, EPI_ISL_12172523, EPI_ISL_12172524, EPI_ISL_12172525, EPI_ISL_12172526, EPI_ISL_12172527, EPI_ISL_12172528, EPI_ISL_12172529, EPI_ISL_12172530, EPI_ISL_12172531, EPI_ISL_12172532, EPI_ISL_12172533, EPI_ISL_12172534, EPI_ISL_12172535, EPI_ISL_12172536, EPI_ISL_12172537, EPI_ISL_12172538, EPI_ISL_12172539, EPI_ISL_12172540, EPI_ISL_12172541, EPI_ISL_12172542, EPI_ISL_12172543, EPI_ISL_12172544, EPI_ISL_12172545, EPI_ISL_12172546, EPI_ISL_12172547, EPI_ISL_12172548, EPI_ISL_12172549, EPI_ISL_12172550, EPI_ISL_12172551, EPI_ISL_12172552, EPI_ISL_12172553, EPI_ISL_12172554, EPI_ISL_12172555, EPI_ISL_12172556, EPI_ISL_12172557, EPI_ISL_12172558, EPI_ISL_12172559, EPI_ISL_12172560, EPI_ISL_12172561, EPI_ISL_12172562, EPI_ISL_12172563, EPI_ISL_12172564, EPI_ISL_12172565, EPI_ISL_12172566, EPI_ISL_12172567, EPI_ISL_12172568, EPI_ISL_12172569, EPI_ISL_12172570, EPI_ISL_12172571, EPI_ISL_12172572, EPI_ISL_12172573, EPI_ISL_12172574, EPI_ISL_12172575, EPI_ISL_12172576, EPI_ISL_12172577, EPI_ISL_12172578, EPI_ISL_12172579, EPI_ISL_12172580, EPI_ISL_12172581, EPI_ISL_12172582, EPI_ISL_12172583, EPI_ISL_12172584, EPI_ISL_12172585, EPI_ISL_12172586, EPI_ISL_12172587, EPI_ISL_12172588, EPI_ISL_12172589, EPI_ISL_12172590, EPI_ISL_12172591, EPI_ISL_12172592, EPI_ISL_12172593, EPI_ISL_12172594, EPI_ISL_12172595, EPI_ISL_12172596, EPI_ISL_12172597, EPI_ISL_12172598, EPI_ISL_12172599, EPI_ISL_12172600, EPI_ISL_12172601, EPI_ISL_12172602, EPI_ISL_12172603, EPI_ISL_12172604, EPI_ISL_12172605, EPI_ISL_12172606, EPI_ISL_12172607, EPI_ISL_12172608, EPI_ISL_12172609, EPI_ISL_12172610, EPI_ISL_12172611, EPI_ISL_12172612, EPI_ISL_12172613, EPI_ISL_12172614, EPI_ISL_12172615, EPI_ISL_12172616, EPI_ISL_12172617, EPI_ISL_12172618, EPI_ISL_12172619, EPI_ISL_12172620, EPI_ISL_12172621, EPI_ISL_12172743, EPI_ISL_12180440, EPI_ISL_12180441, EPI_ISL_12180442, EPI_ISL_12180443, EPI_ISL_12180444, EPI_ISL_12180445, EPI_ISL_12180446, EPI_ISL_12180447, EPI_ISL_12180448, EPI_ISL_12180449, EPI_ISL_12180450, EPI_ISL_12180451, EPI_ISL_12180452, EPI_ISL_12180453, EPI_ISL_12180454, EPI_ISL_12180455, EPI_ISL_12180456, EPI_ISL_12180457, EPI_ISL_12180458, EPI_ISL_12180459, EPI_ISL_12180460, EPI_ISL_12180461, EPI_ISL_12180462, EPI_ISL_12180463, EPI_ISL_12180464, EPI_ISL_12180465, EPI_ISL_12180466, EPI_ISL_12180467, EPI_ISL_12180468, EPI_ISL_12180469, EPI_ISL_12180470, EPI_ISL_12180471, EPI_ISL_12180472, EPI_ISL_12180473, EPI_ISL_12180474, EPI_ISL_12180475, EPI_ISL_12180476, EPI_ISL_12180477, EPI_ISL_12180478, EPI_ISL_12180479, EPI_ISL_12180480, EPI_ISL_12180481, EPI_ISL_12180482, EPI_ISL_12180483, EPI_ISL_12180484, EPI_ISL_12180485, EPI_ISL_12180486, EPI_ISL_12180487, EPI_ISL_12180488, EPI_ISL_12180489, EPI_ISL_12180490, EPI_ISL_12180491, EPI_ISL_12180492, EPI_ISL_12180493, EPI_ISL_12180494, EPI_ISL_12180495, EPI_ISL_12180496, EPI_ISL_12180497, EPI_ISL_12180498, EPI_ISL_12235800, EPI_ISL_12235801, EPI_ISL_12235802, EPI_ISL_12235803, EPI_ISL_12235804, EPI_ISL_12235805, EPI_ISL_12235806, EPI_ISL_12235807, EPI_ISL_12235808, EPI_ISL_12235809, EPI_ISL_12235810, EPI_ISL_12235811, EPI_ISL_12235812, EPI_ISL_12235813, EPI_ISL_12235814, EPI_ISL_12235815, EPI_ISL_12235816, EPI_ISL_12235817, EPI_ISL_12235818, EPI_ISL_12235819, EPI_ISL_12235820, EPI_ISL_12235821, EPI_ISL_12235822, EPI_ISL_12235823, EPI_ISL_12235824, EPI_ISL_12235825, EPI_ISL_12235826, EPI_ISL_12235827, EPI_ISL_12235828, EPI_ISL_12235829, EPI_ISL_12235830, EPI_ISL_12235831, EPI_ISL_12235832, EPI_ISL_12235833, EPI_ISL_12235834, EPI_ISL_12235835, EPI_ISL_12235836, EPI_ISL_12235837, EPI_ISL_12235838, EPI_ISL_12235839, EPI_ISL_12235840, EPI_ISL_12235841, EPI_ISL_12235842, EPI_ISL_12235843, EPI_ISL_12235844, EPI_ISL_12235845, EPI_ISL_12235846, EPI_ISL_12235847, EPI_ISL_12235848, EPI_ISL_12235849, EPI_ISL_12235850, EPI_ISL_12235851			
see above	Kuala Lumpur International Airport	Institute for Medical Research, Infectious Disease Research Centre, National Institutes of Health, Ministry of Health Malaysia	Anasir MI; G.Adypatti NM; Jamaluddin MS; Kalyanasundram J; Kamel K; MatRahim N; Nawi MH; Suib FA; Suppiah J; Thayan R
EPI_ISL_12213391	MAKMAL KESIHATAN AWAM JOHOR BAHRU	UKM Medical Molecular Biology Institute (UMBI)	Khairun Nur Abd Ghafar; Mira Farzana Mohamad Mokhtar; Muhiddin Ishak; Nor Azila Muhammad Azami; Nur Alyaa Afifah Md Shahri; Nurul Syakima Ab Mutalib; Rahman Jamal; Ryia Illani Mohd Yunos; Siti Nur Hasanah Mohd Yusuf; Zahirrah Begam Mohamed Rasheed
EPI_ISL_12099658	Miri Hospital Molecular Diagnostic Lab, Miri	Institute of Health and Community Medicine	Chan Chia Jui; Chua Hock Hin; David Perera; Ooi Mong How; Tonnii Sia Loong Loong
EPI_ISL_12213431, EPI_ISL_12213432, EPI_ISL_12213433, EPI_ISL_12213434, EPI_ISL_12213435	PKD MUAR	UKM Medical Molecular Biology Institute (UMBI)	Khairun Nur Abd Ghafar; Mira Farzana Mohamad Mokhtar; Muhiddin Ishak; Nor Azila Muhammad Azami; Nur Alyaa Afifah Md Shahri; Nurul Syakima Ab Mutalib; Rahman Jamal; Ryia Illani Mohd Yunos; Siti Nur Hasanah Mohd Yusuf; Zahirrah Begam Mohamed Rasheed
EPI_ISL_12099675, EPI_ISL_12099676, EPI_ISL_12099724, EPI_ISL_12099748	Rejang Medical Centre (Sibu)	Institute of Health and Community Medicine	Chan Chia Jui; Chua Hock Hin; David Perera; Ooi Mong How; Tonnii Sia Loong Loong; Wong Jyn Shan

We gratefully acknowledge the following Authors from the Originating laboratories responsible for obtaining the specimens, as well as the Submitting laboratories where the genome data were generated and shared via GISAID, on which this research is based.

All Submitters of data may be contacted directly via www.gisaid.org

Authors are sorted alphabetically.

Accession ID	Originating Laboratory	Submitting Laboratory	Authors
EPI_ISL_12749678	AMPANG HOSPITAL	Institute for Medical Research, Infectious Disease Research Centre, National Institutes of Health, Ministry of Health Malaysia	Ahmad FA; Ahmad Fazilah NA; Anasir MI; Azizan MA; Kamel K; Mohamad Sukri MZ; Norhisham SN; Ramly N; Robert F; Rosli NR; Suppiah J; Thayan R
EPI_ISL_12510350, EPI_ISL_12510351, EPI_ISL_12739655	Bintulu Medical Centre (Bintulu)	Institute of Health and Community Medicine	Chan Chia Jui; David Perera; Ooi Mong How; Wong Jyn Shan
EPI_ISL_12510111, EPI_ISL_12510112, EPI_ISL_12510113, EPI_ISL_12510114, EPI_ISL_12510115	Borneo Medical Centre (Kuching)	Institute of Health and Community Medicine	Chan Chia Jui; David Perera; Ooi Mong How; Wong Jyn Shan
EPI_ISL_12704539	Department of Medical Microbiology and Parasitology, Hospital Universiti Sains Malaysia	Molecular Research Laboratory	Abdul Haris bin Muhammad; Ahmad Sukari Bin Halim; Alexander Chong Shu Chien; Asraihan Che Abdul Malik; Azian Harun; Chan Yean Yean; Chua Wei Chuan; Farahana binti Mohamed; Kirnpal Kaur Banga Singh; Lau Nyok Sean; Lee Lih Huey; Lim Shu Yong; Maizun binti Mohd Zain; Mera Edora Binti Abdul Manap; Mohd Iman hafiz Bin Ibrahim; Mohd Nadzri Abu Yazid; Mohd Zulkifli Salleh; Muhamad Khairul Amirin Bin Zulkifli; Muhammad Azamuddeen bin Mohammad Nasir; Muhammad Fazli bin Khalid; Muhammad Nashrul Farhan Samsudin; Muhammad Zarul Hanifah Bin Md Zoqratt; Nik Zuraina binti Nik Mohd Noor; Noor Hafizan binti Mat Salleh; Nor Amizara Binti Azami; Nor Suhana Binti Mohd Satar; Nur Syuhada Binti Abdul Rahim; Nur-Leem Binti Murshid; Nurfadhlina Musa; Qasim Ayub; Rosline Hassan; Sadequr Rahman; Siti Nur Aisyah Binti Mohamad Sham; Syahida binti Omar; Wan Mohd Zahiruddin Wan Mohammad; Wardah Yusof; Zaini bin Hussin; Zakuan Zainy Deris
EPI_ISL_12721622	Department of Medical Microbiology, Hospital Pengajar Universiti Putra Malaysia	Department of Medical Microbiology, Hospital Pengajar Universiti Putra Malaysia	Afiqah Adzmi; Amiza Azmi; Azmiza Syawani Jasni; Hui Yee Chee; Leslie Thian Lung Than; Muadz Mohtar; Muhammad Mohd Isa; Narcisse MS Joseph; Niazlin Mohd Taib; Noor Hazirah Noor Azhari; Norlaila; Nur Raihana Ithnin; Nurul Huda Mohamed Rashidi; Nurul Nadiah Ismail; Rosni Ibrahim; Rukman Awang Hamat; Sallehhudin; Siti Norbaya Masri; Siti Zulaikha Zakariah; Syafinaz Amin Nordin; Tengku Zetty Maztura Tengku Jamaluddin; Zamberi Sekawi
EPI_ISL_12771304	HOSPITAL PULAU PINANG	iPROMISE, UiTM	Ariza Adnan; Fadzilah Mohd Nor; Lim Wai Feng; Mohd Asif Mohd Sukri; Mohd Nur Fakhruzzaman Noorizhab; Mohd Zaki Salleh; Sazzli Shahlan Kassim; Siti Farah Alwani Mohd Nawi; Siti Hamimah Sheikh Abdul Kadir; Teh Lay Kek; Wang Seok Mui
EPI_ISL_12534766, EPI_ISL_12534767	HOSPITAL SULTANAH NURZAHIRAH	Tropical Infectious Diseases Research & Education Centre (TIDREC), Universiti Malaya	AsmaAnati CheMatSeri; Che-Norainon Yaacob; Jia-Yi Tan; Jo-Ern Wong; Kim-Kee Tan; Mulya-Mustika-Sari Zulkifli; Noor Syahida Azizan; Nur-Hidayana Mahfodz; Sazaly AbuBakar; Siti-Sarah Nor'e; Wei-Wei Aw
EPI_ISL_12771302, EPI_ISL_12771303	Hospital Pulau Pinang	iPROMISE, UiTM	Ariza Adnan; Fadzilah Mohd Nor; Lim Wai Feng; Mohd Asif Mohd Sukri; Mohd Nur Fakhruzzaman Noorizhab; Mohd Zaki Salleh; Sazzli Shahlan Kassim; Siti Farah Alwani Mohd Nawi; Siti Hamimah Sheikh Abdul Kadir; Teh Lay Kek; Wang Seok Mui
EPI_ISL_12322079, EPI_ISL_12322080	KUALA LUMPUR HOSPITAL	Institute for Medical Research, Infectious Disease Research Centre, National Institutes of Health, Ministry of Health Malaysia	Ahmad FA; Ahmad Fazilah NA; Anasir MI; Azizan MA; Kamel K; Mohamad Sukri MZ; Norhisham SN; Ramly N; Robert F; Rosli NR; Suppiah J; Thayan R
EPI_ISL_12746987	KULIM HOSPITAL	Institute for Medical Research, Infectious Disease Research Centre, National Institutes of Health, Ministry of Health Malaysia	Ahmad FA; Ahmad Fazilah NA; Anasir MI; Azizan MA; Kamel K; Mohamad Sukri MZ; Norhisham SN; Ramly N; Robert F; Rosli NR; Suppiah J; Thayan R
EPI_ISL_12708562	Kota Bharu Public Health Laboratory	Molecular Research Laboratory	Abdul Haris bin Muhammad; Ahmad Sukari Bin Halim; Alexander Chong Shu Chien; Asraihan Che Abdul Malik; Azian Harun; Chan Yean Yean; Chua Wei Chuan; Farahana binti Mohamed; Kirnpal Kaur Banga Singh; Lau Nyok Sean; Lee Lih Huey; Lim Shu Yong; Maizun binti Mohd Zain; Mera Edora Binti Abdul Manap; Mohd Iman hafiz Bin Ibrahim; Mohd Nadzri Abu Yazid; Mohd Zulkifli Salleh; Muhamad Khairul Amirin Bin Zulkifli; Muhammad Azamuddeen bin Mohammad Nasir; Muhammad Fazli bin Khalid; Muhammad Nashrul Farhan Samsudin; Muhammad Zarul Hanifah Bin Md Zoqratt; Nik Zuraina binti Nik Mohd Noor; Noor Hafizan binti Mat Salleh; Nor Amizara Binti Azami; Nor Suhana Binti Mohd Satar; Nur Syuhada Binti Abdul Rahim; Nur-Leem Binti Murshid; Nurfadhlina Musa; Qasim Ayub; Rosline Hassan; Sadequr Rahman; Siti Nur Aisyah Binti Mohamad Sham; Syahida binti Omar; Wan Mohd Zahiruddin Wan Mohammad; Wardah Yusof; Zaini bin Hussin; Zakuan Zainy Deris

EPI_ISL_12299406, EPI_ISL_12299407, EPI_ISL_12299408, EPI_ISL_12299409, EPI_ISL_12299410, EPI_ISL_12299411, EPI_ISL_12299412, EPI_ISL_12299413, EPI_ISL_12299414, EPI_ISL_12299415, EPI_ISL_12299416, EPI_ISL_12299417, EPI_ISL_12299418, EPI_ISL_12299419, EPI_ISL_12299420, EPI_ISL_12299421, EPI_ISL_12299422, EPI_ISL_12299423, EPI_ISL_12299424, EPI_ISL_12299425, EPI_ISL_12299426, EPI_ISL_12299427, EPI_ISL_12299428, EPI_ISL_12299429, EPI_ISL_12299430, EPI_ISL_12299431, EPI_ISL_12299432, EPI_ISL_12299433, EPI_ISL_12299434, EPI_ISL_12299435, EPI_ISL_12299436, EPI_ISL_12299437, EPI_ISL_12299438, EPI_ISL_12299439, EPI_ISL_12299440, EPI_ISL_12299441, EPI_ISL_12299442, EPI_ISL_12299443, EPI_ISL_12299444, EPI_ISL_12299445, EPI_ISL_12299446, EPI_ISL_12299447, EPI_ISL_12299448, EPI_ISL_12299449, EPI_ISL_12299450, EPI_ISL_12299451, EPI_ISL_12299452, EPI_ISL_12299453, EPI_ISL_12299454, EPI_ISL_12299455, EPI_ISL_12299456, EPI_ISL_12299457, EPI_ISL_12299458, EPI_ISL_12299459, EPI_ISL_12299461, EPI_ISL_12299462, EPI_ISL_12299463, EPI_ISL_12299464, EPI_ISL_12299465, EPI_ISL_12299466, EPI_ISL_12299467, EPI_ISL_12299468, EPI_ISL_12299469, EPI_ISL_12299470, EPI_ISL_12299471, EPI_ISL_12299472, EPI_ISL_12299473, EPI_ISL_12299474, EPI_ISL_12299475, EPI_ISL_12299476, EPI_ISL_12299477, EPI_ISL_12299478, EPI_ISL_12299479, EPI_ISL_12299480, EPI_ISL_12299481, EPI_ISL_12299482, EPI_ISL_12299483, EPI_ISL_12299484, EPI_ISL_12299485, EPI_ISL_12299486, EPI_ISL_12299487, EPI_ISL_12299488, EPI_ISL_12299489, EPI_ISL_12299490, EPI_ISL_12299491, EPI_ISL_12299492, EPI_ISL_12299493, EPI_ISL_12299494, EPI_ISL_12299495, EPI_ISL_12299496, EPI_ISL_12299497, EPI_ISL_12299498, EPI_ISL_12299499, EPI_ISL_12299500, EPI_ISL_12299501, EPI_ISL_12299502, EPI_ISL_12299503, EPI_ISL_12299504, EPI_ISL_12299505, EPI_ISL_12299506, EPI_ISL_12299507, EPI_ISL_12299508, EPI_ISL_12299509, EPI_ISL_12299510, EPI_ISL_12299511, EPI_ISL_12299512, EPI_ISL_12299513, EPI_ISL_12299514, EPI_ISL_12299515, EPI_ISL_12299516, EPI_ISL_12299517, EPI_ISL_12299518, EPI_ISL_12299519, EPI_ISL_12299520, EPI_ISL_12299521, EPI_ISL_12299522, EPI_ISL_12299523, EPI_ISL_12299524, EPI_ISL_12299525, EPI_ISL_12299526, EPI_ISL_12299527, EPI_ISL_12299528, EPI_ISL_12299529, EPI_ISL_12299530, EPI_ISL_12299531, EPI_ISL_12299532, EPI_ISL_12299533, EPI_ISL_12299534, EPI_ISL_12299535, EPI_ISL_12299536, EPI_ISL_12299537, EPI_ISL_12299538, EPI_ISL_12299539, EPI_ISL_12299540, EPI_ISL_12299541, EPI_ISL_12299542, EPI_ISL_12299543, EPI_ISL_12299544, EPI_ISL_12299545, EPI_ISL_12299546, EPI_ISL_12299547, EPI_ISL_12299548, EPI_ISL_12299549, EPI_ISL_12299550, EPI_ISL_12299551, EPI_ISL_12299552, EPI_ISL_12299553, EPI_ISL_12299554, EPI_ISL_12299555, EPI_ISL_12299556, EPI_ISL_12299557, EPI_ISL_12299558, EPI_ISL_12299561, EPI_ISL_12309110, EPI_ISL_12309111, EPI_ISL_12309112, EPI_ISL_12309113, EPI_ISL_12309114, EPI_ISL_12309115, EPI_ISL_12309116, EPI_ISL_12309117, EPI_ISL_12309118, EPI_ISL_12322157, EPI_ISL_12322158, EPI_ISL_12322159, EPI_ISL_12322160, EPI_ISL_12322161, EPI_ISL_12322162, EPI_ISL_12322163, EPI_ISL_12322164, EPI_ISL_12322165, EPI_ISL_12322166, EPI_ISL_12322167, EPI_ISL_12322168, EPI_ISL_12322169, EPI_ISL_12322170, EPI_ISL_12322171, EPI_ISL_12322172, EPI_ISL_12322173, EPI_ISL_12322174, EPI_ISL_12322175, EPI_ISL_12322176, EPI_ISL_12322177, EPI_ISL_12322178, EPI_ISL_12322179, EPI_ISL_12322180, EPI_ISL_12322181, EPI_ISL_12322182, EPI_ISL_12322183, EPI_ISL_12322184, EPI_ISL_12322185, EPI_ISL_12322186, EPI_ISL_12322187, EPI_ISL_12322188, EPI_ISL_12322189, EPI_ISL_12322190, EPI_ISL_12322191, EPI_ISL_12322192, EPI_ISL_12322193, EPI_ISL_12322194, EPI_ISL_12322195, EPI_ISL_12322196, EPI_ISL_12322197, EPI_ISL_12322198, EPI_ISL_12322199, EPI_ISL_12322200, EPI_ISL_12322201, EPI_ISL_12322202, EPI_ISL_12322203, EPI_ISL_12322204, EPI_ISL_12322205, EPI_ISL_12322206, EPI_ISL_12322207, EPI_ISL_12322208, EPI_ISL_12322209, EPI_ISL_12322210, EPI_ISL_12322211, EPI_ISL_12322212, EPI_ISL_12322213, EPI_ISL_12322214, EPI_ISL_12322215, EPI_ISL_12322216, EPI_ISL_12322217, EPI_ISL_12322218, EPI_ISL_12322219, EPI_ISL_12322220, EPI_ISL_12322221, EPI_ISL_12322222, EPI_ISL_12322223, EPI_ISL_12322224, EPI_ISL_12322225, EPI_ISL_12322226, EPI_ISL_12322227, EPI_ISL_12322228, EPI_ISL_12322229, EPI_ISL_12322230, EPI_ISL_12322231, EPI_ISL_12322232, EPI_ISL_12322233, EPI_ISL_12322234, EPI_ISL_12322235, EPI_ISL_12322236, EPI_ISL_12322237, EPI_ISL_12322238, EPI_ISL_12322239, EPI_ISL_12322240, EPI_ISL_12322241, EPI_ISL_12322242, EPI_ISL_12322243, EPI_ISL_12322244, EPI_ISL_12322245, EPI_ISL_12322246, EPI_ISL_12322247, EPI_ISL_12322248, EPI_ISL_12322249, EPI_ISL_12322250, EPI_ISL_12322251, EPI_ISL_12322252, EPI_ISL_12322253, EPI_ISL_12322254, EPI_ISL_12322255, EPI_ISL_12322256, EPI_ISL_12322257, EPI_ISL_12322258, EPI_ISL_12322259, EPI_ISL_12322260, EPI_ISL_12322261, EPI_ISL_12322262, EPI_ISL_12322263, EPI_ISL_12322264, EPI_ISL_12322265, EPI_ISL_12322266, EPI_ISL_12322267, EPI_ISL_12322268, EPI_ISL_12322269, EPI_ISL_12322270, EPI_ISL_12322271, EPI_ISL_12322272, EPI_ISL_12322273, EPI_ISL_12322274, EPI_ISL_12322275, EPI_ISL_12322276, EPI_ISL_12322277, EPI_ISL_12322278, EPI_ISL_12322279, EPI_ISL_12322280, EPI_ISL_12322281, EPI_ISL_12322282, EPI_ISL_12322283, EPI_ISL_12322284, EPI_ISL_12322285, EPI_ISL_12322286, EPI_ISL_12322287, EPI_ISL_12322288, EPI_ISL_12322348, EPI_ISL_12322349, EPI_ISL_12322350, EPI_ISL_12322351, EPI_ISL_12322352, EPI_ISL_12322353, EPI_ISL_12322354, EPI_ISL_12322355, EPI_ISL_12322356, EPI_ISL_12322357, EPI_ISL_12322358, EPI_ISL_12322359, EPI_ISL_12322360, EPI_ISL_12322362, EPI_ISL_12322363, EPI_ISL_12322364, EPI_ISL_12322365, EPI_ISL_12322366, EPI_ISL_12322367, EPI_ISL_12322368, EPI_ISL_12322369, EPI_ISL_12322370, EPI_ISL_12322371, EPI_ISL_12322372, EPI_ISL_12322373, EPI_ISL_12322374, EPI_ISL_12322375, EPI_ISL_12322376, EPI_ISL_12322377, EPI_ISL_12322378, EPI_ISL_12322379, EPI_ISL_12322380, EPI_ISL_12322381, EPI_ISL_12322382, EPI_ISL_12322383, EPI_ISL_12322384, EPI_ISL_12322385, EPI_ISL_12322386, EPI_ISL_12332029

see above	Kuala Lumpur International Airport	Institute for Medical Research, Infectious Disease Research Centre, National Institutes of Health, Ministry of Health Malaysia	Anasir MI; G.Adypatti NM; Jamaluddin MS; Kalyanasundram J; Kamel K; MatRahim N; Nawi MH; Suib FA; Suppiah J; Thayan R
EPI_ISL_12534774	MAKMAL SEROLOGI, UNIT MIKROBIOLOGI, HSB	Tropical Infectious Diseases Research & Education Centre (TIDREC), Universiti Malaya	AsmaAnati CheMatSeri; Che-Norainon Yaacob; Jia-Yi Tan; Jo-Ern Wong; Kim-Kee Tan; Mulya-Mustika-Sari Zulkifli; Noor Syahida Azizan; Nur-Hidayana Mahfodz; Sazaly AbuBakar; Siti-Sarah Nor'e; Wei-Wei Aw
EPI_ISL_12289486	SULTANAH BAHIYAH HOSPITAL	Institute for Medical Research, Infectious Disease Research Centre, National Institutes of Health, Ministry of Health Malaysia	Ahmad FA; Ahmad Fazilah NA; Anasir MI; Azizan MA; Kamel K; Mohamad Sukri MZ; Norhisham SN; Ramly N; Robert F; Rosli NR; Suppiah J; Thayan R

We gratefully acknowledge the following Authors from the Originating laboratories responsible for obtaining the specimens, as well as the Submitting laboratories where the genome data were generated and shared via GISAID, on which this research is based.

All Submitters of data may be contacted directly via www.gisaid.org

Authors are sorted alphabetically.

Acknowledgement EPI_SET Identifier: EPI_SET_20220603um

Accession ID	Originating Laboratory	Submitting Laboratory	Authors
EPI_ISL_12322059, EPI_ISL_12322060, EPI_ISL_12322061, EPI_ISL_12899667	AMPANG HOSPITAL	Institute for Medical Research, Infectious Disease Research Centre, National Institutes of Health, Ministry of Health Malaysia	Ahmad FA; Ahmad Fazilah NA; Anasir MI; Azizan MA; Kamel K; Mohamad Sukri MZ; Norhisham SN; Ramly N; Robert F; Rosli NR; Suppiah J; Thayan R
EPI_ISL_11805037, EPI_ISL_11805038	Bintulu Hospital PCR Lab, Bintulu	Institute of Health and Community Medicine	Chan Chia Jui; Chien Su Lin; Chua Hock Hin; David Perera; Ooi Mong How; Tonnii Sia Loong Loong
EPI_ISL_12099610, EPI_ISL_12099611, EPI_ISL_12739625	Bintulu Medical Centre (Bintulu)	Institute of Health and Community Medicine	Chan Chia Jui; Chua Hock Hin; David Perera; Ooi Mong How; Tonnii Sia Loong Loong; Wong Jyn Shan
EPI_ISL_12510116, EPI_ISL_12510117, EPI_ISL_12510118, EPI_ISL_12510119, EPI_ISL_12510120, EPI_ISL_12510121, EPI_ISL_12510122			
see above	Borneo Medical Centre (Kuching)	Institute of Health and Community Medicine	Chan Chia Jui; David Perera; Ooi Mong How; Wong Jyn Shan
EPI_ISL_12099744, EPI_ISL_12099745	Borneo Medical Centre (Miri)	Institute of Health and Community Medicine	Chan Chia Jui; Chua Hock Hin; David Perera; Ooi Mong How; Tonnii Sia Loong Loong; Wong Jyn Shan
EPI_ISL_12704566, EPI_ISL_12704567	Department of Medical Microbiology and Parasitology, Hospital Universiti Sains Malaysia	Molecular Research Laboratory	Abdul Haris bin Muhammad; Ahmad Sukari Bin Halim; Alexander Chong Shu Chien; Asraihan Che Abdul Malik; Azian Harun; Chan Yean Yean; Chua Wei Chuan; Farahana binti Mohamed; Kirnpal Kaur Banga Singh; Lau Nyok Sean; Lee Lih Huey; Lim Shu Yong; Maizun binti Mohd Zain; Mera Edora Binti Abdul Manap; Mohd Iman hafiz Bin Ibrahim; Mohd Nadzri Abu Yazid; Mohd Zulkifli Salleh; Muhamad Khairul Amirin Bin Zulkifli; Muhammad Azamuddeen bin Mohammad Nasir; Muhammad Fazli bin Khalid; Muhammad Nashrul Farhan Samsudin; Muhammad Zarul Hanifah Bin Md Zoqratt; Nik Zuraina binti Nik Mohd Noor; Noor Hafizan binti Mat Salleh; Nor Amizara Binti Azami; Nor Suhana Binti Mohd Satar; Nur Syuhada Binti Abdul Rahim; Nur-Leem Binti Murshid; Nurfadhlina Musa; Qasim Ayub; Rosline Hassan; Sadequr Rahman; Siti Nur Aisyah Binti Mohamad Sham; Syahida binti Omar; Wan Mohd Zahiruddin Wan Mohammad; Wardah Yusof; Zaini bin Hussin; Zakuan Zainy Deris
EPI_ISL_12771305	HOSPITAL PULAU PINANG	iPROMISE, UiTM	Ariza Adnan; Fadzilah Mohd Nor; Lim Wai Feng; Mohd Asif Mohd Sukri; Mohd Nur Fakhruzzaman Noorizhab; Mohd Zaki Salleh; Sazzli Shahlan Kassim; Siti Farah Alwani Mohd Nawi; Siti Hamimah Sheikh Abdul Kadir; Teh Lay Kek; Wang Seok Mui
EPI_ISL_12534768	HOSPITAL SULTANAH NURZAHIRAH	Tropical Infectious Diseases Research & Education Centre (TIDREC), Universiti Malaya	AsmaAnati CheMatSeri; Che-Norainon Yaacob; Jia-Yi Tan; Jo-Ern Wong; Kim-Kee Tan; Mulya-Mustika-Sari Zulkifli; Noor Syahida Azizan; Nur-Hidayana Mahfodz; Sazaly AbuBakar; Siti-Sarah Nor'e; Wei-Wei Aw
EPI_ISL_12213399, EPI_ISL_12213400	HSAJB	UKM Medical Molecular Biology Institute (UMBI)	Khairun Nur Abd Ghafar; Mira Farzana Mohamad Mokhtar; Muhiddin Ishak; Nor Azila Muhammad Azami; Nur Alyaa Afifah Md Shahri; Nurul Syakima Ab Mutalib; Rahman Jamal; Ryia Illani Mohd Yunos; Siti Nur Hasanah Mohd Yusuf; Zahirrah Begam Mohamed Rasheed
EPI_ISL_12708560	Kota Bharu Public Health Laboratory	Molecular Research Laboratory	Abdul Haris bin Muhammad; Ahmad Sukari Bin Halim; Alexander Chong Shu Chien; Asraihan Che Abdul Malik; Azian Harun; Chan Yean Yean; Chua Wei Chuan; Farahana binti Mohamed; Kirnpal Kaur Banga Singh; Lau Nyok Sean; Lee Lih Huey; Lim Shu Yong; Maizun binti Mohd Zain; Mera Edora Binti Abdul Manap; Mohd Iman hafiz Bin Ibrahim; Mohd Nadzri Abu Yazid; Mohd Zulkifli Salleh; Muhamad Khairul Amirin Bin Zulkifli; Muhammad Azamuddeen bin Mohammad Nasir; Muhammad Fazli bin Khalid; Muhammad Nashrul Farhan Samsudin; Muhammad Zarul Hanifah Bin Md Zoqratt; Nik Zuraina binti Nik Mohd Noor; Noor Hafizan binti Mat Salleh; Nor Amizara Binti Azami; Nor Suhana Binti Mohd Satar; Nur Syuhada Binti Abdul Rahim; Nur-Leem Binti Murshid; Nurfadhlina Musa; Qasim Ayub; Rosline Hassan; Sadequr Rahman; Siti Nur Aisyah Binti Mohamad Sham; Syahida binti Omar; Wan Mohd Zahiruddin Wan Mohammad; Wardah Yusof; Zaini bin Hussin; Zakuan Zainy Deris
EPI_ISL_12235852, EPI_ISL_12235853, EPI_ISL_12235854, EPI_ISL_12235855, EPI_ISL_12235856, EPI_ISL_12235857, EPI_ISL_12235858, EPI_ISL_12235859, EPI_ISL_12235860, EPI_ISL_12235861, EPI_ISL_12235862, EPI_ISL_12235863, EPI_ISL_12235864, EPI_ISL_12322289, EPI_ISL_12322290, EPI_ISL_12322291, EPI_ISL_12322292, EPI_ISL_12322293, EPI_ISL_12322294, EPI_ISL_12322295, EPI_ISL_12322296, EPI_ISL_12322297, EPI_ISL_12322298, EPI_ISL_12322299, EPI_ISL_12322300, EPI_ISL_12322301, EPI_ISL_12322302, EPI_ISL_12322303, EPI_ISL_12322304, EPI_ISL_12322305, EPI_ISL_12322306, EPI_ISL_12322307, EPI_ISL_12322308, EPI_ISL_12322309, EPI_ISL_12322310, EPI_ISL_12322311, EPI_ISL_12322312, EPI_ISL_12322313, EPI_ISL_12322314, EPI_ISL_12322315, EPI_ISL_12322316, EPI_ISL_12322317, EPI_ISL_12322318, EPI_ISL_12322319, EPI_ISL_12322320, EPI_ISL_12322321, EPI_ISL_12322322, EPI_ISL_12322323, EPI_ISL_12322324, EPI_ISL_12322325, EPI_ISL_12322326, EPI_ISL_12322327, EPI_ISL_12322328, EPI_ISL_12322329, EPI_ISL_12322330, EPI_ISL_12322331, EPI_ISL_12322332, EPI_ISL_12322333, EPI_ISL_12322334, EPI_ISL_12322335, EPI_ISL_12322336, EPI_ISL_12322337, EPI_ISL_12322338, EPI_ISL_12322339, EPI_ISL_12322340, EPI_ISL_12322341, EPI_ISL_12322342, EPI_ISL_12322343, EPI_ISL_12322344, EPI_ISL_12322345, EPI_ISL_12322346, EPI_ISL_12322347, EPI_ISL_12322361, EPI_ISL_12322387, EPI_ISL_12322388, EPI_ISL_12322389, EPI_ISL_12322390, EPI_ISL_12322391, EPI_ISL_12322392, EPI_ISL_12322393, EPI_ISL_12322394, EPI_ISL_12322395, EPI_ISL_12322396, EPI_ISL_12891125, EPI_ISL_12891126, EPI_ISL_12891127, EPI_ISL_12891128, EPI_ISL_12891129, EPI_ISL_12891130, EPI_ISL_12891131, EPI_ISL_12891132, EPI_ISL_12891133, EPI_ISL_12891134, EPI_ISL_12891135, EPI_ISL_12891136, EPI_ISL_12891137, EPI_ISL_12891138, EPI_ISL_12891139, EPI_ISL_12891140, EPI_ISL_12891141, EPI_ISL_12891142, EPI_ISL_12891143, EPI_ISL_12891144, EPI_ISL_12891145, EPI_ISL_12891146, EPI_ISL_12891147, EPI_ISL_12891148, EPI_ISL_12891149, EPI_ISL_12891150, EPI_ISL_12891151, EPI_ISL_12891152, EPI_ISL_12891153, EPI_ISL_12891154, EPI_ISL_12891155, EPI_ISL_12891156, EPI_ISL_12891157, EPI_ISL_12891158, EPI_ISL_12891159, EPI_ISL_12891160, EPI_ISL_12891161, EPI_ISL_12891162, EPI_ISL_12891163, EPI_ISL_12891164, EPI_ISL_12891165, EPI_ISL_12891166, EPI_ISL_12891167, EPI_ISL_12891168, EPI_ISL_12891169, EPI_ISL_12891170, EPI_ISL_12891171, EPI_ISL_12891172, EPI_ISL_12891173, EPI_ISL_12891174, EPI_ISL_12891175, EPI_ISL_12891176, EPI_ISL_12891177, EPI_ISL_12891178, EPI_ISL_12891179, EPI_ISL_12891180, EPI_ISL_12891181, EPI_ISL_12891182, EPI_ISL_12891183, EPI_ISL_12891184, EPI_ISL_12891185, EPI_ISL_12891186, EPI_ISL_12891187, EPI_ISL_12891188, EPI_ISL_12891189, EPI_ISL_12891190, EPI_ISL_12891191, EPI_ISL_12891192, EPI_ISL_12891193, EPI_ISL_12891194, EPI_ISL_12891195, EPI_ISL_12891196, EPI_ISL_12891197, EPI_ISL_12891198, EPI_ISL_12891199, EPI_ISL_12891200, EPI_ISL_12891201, EPI_ISL_12891202, EPI_ISL_12891203, EPI_ISL_12891204, EPI_ISL_12891205, EPI_ISL_12891206, EPI_ISL_12891207, EPI_ISL_12891208, EPI_ISL_12891209, EPI_ISL_12911692, EPI_ISL_12911693, EPI_ISL_12911694, EPI_ISL_12911695, EPI_ISL_12911696, EPI_ISL_12911697, EPI_ISL_12911698, EPI_ISL_12911699, EPI_ISL_12911700, EPI_ISL_12911701, EPI_ISL_12942470, EPI_ISL_12942471, EPI_ISL_12942472, EPI_ISL_12942473, EPI_ISL_12942474, EPI_ISL_12942475, EPI_ISL_12942476, EPI_ISL_12942477, EPI_ISL_12942478, EPI_ISL_12942479, EPI_ISL_12942480, EPI_ISL_12942481, EPI_ISL_12942482, EPI_ISL_12942483, EPI_ISL_12942484, EPI_ISL_12942485, EPI_ISL_12942486, EPI_ISL_12942487, EPI_ISL_12942488, EPI_ISL_12942489, EPI_ISL_12942490, EPI_ISL_12942491, EPI_ISL_12942492, EPI_ISL_12942493, EPI_ISL_12942494, EPI_ISL_12942495, EPI_ISL_12942496, EPI_ISL_12942497, EPI_ISL_12942498, EPI_ISL_12942499, EPI_ISL_12942500, EPI_ISL_12942501, EPI_ISL_12942502, EPI_ISL_12942503, EPI_ISL_12942504, EPI_ISL_12942505, EPI_ISL_12942506, EPI_ISL_12942507, EPI_ISL_12942508, EPI_ISL_12942509, EPI_ISL_12942510, EPI_ISL_12942511, EPI_ISL_12942512, EPI_ISL_12942513, EPI_ISL_12942514, EPI_ISL_12942515, EPI_ISL_12942516, EPI_ISL_12942517, EPI_ISL_12942518, EPI_ISL_12942519, EPI_ISL_12942520, EPI_ISL_12942521, EPI_ISL_12942522, EPI_ISL_12942523, EPI_ISL_12942524, EPI_ISL_12942525, EPI_ISL_12942526, EPI_ISL_12942527, EPI_ISL_12942528, EPI_ISL_12942529, EPI_ISL_12942530, EPI_ISL_12942531, EPI_ISL_12942532, EPI_ISL_12942533, EPI_ISL_12942534, EPI_ISL_12942535, EPI_ISL_12942536, EPI_ISL_12942537, EPI_ISL_12942538, EPI_ISL_12942539, EPI_ISL_12942540, EPI_ISL_12942541, EPI_ISL_12942542, EPI_ISL_12942543, EPI_ISL_12942544, EPI_ISL_12942545, EPI_ISL_12942546, EPI_ISL_12942547, EPI_ISL_12942548, EPI_ISL_12942549, EPI_ISL_12942550, EPI_ISL_12942551, EPI_ISL_12942552, EPI_ISL_12942553, EPI_ISL_12942554, EPI_ISL_12942555, EPI_ISL_12942556, EPI_ISL_12942557, EPI_ISL_12942558, EPI_ISL_12942559, EPI_ISL_12942560, EPI_ISL_12942562, EPI_ISL_12942563, EPI_ISL_12942564, EPI_ISL_12942565, EPI_ISL_12942566, EPI_ISL_12942567, EPI_ISL_12942568, EPI_ISL_12942569, EPI_ISL_12942570, EPI_ISL_12942571, EPI_ISL_12942572, EPI_ISL_12942573, EPI_ISL_12942574, EPI_ISL_12942575, EPI_ISL_12942576, EPI_ISL_12942577, EPI_ISL_12942578, EPI_ISL_12942579, EPI_ISL_12942580, EPI_ISL_12942581, EPI_ISL_12942582, EPI_ISL_12942583, EPI_ISL_12942584, EPI_ISL_12942585, EPI_ISL_12942586, EPI_ISL_12942587, EPI_ISL_12942588, EPI_ISL_12942589, EPI_ISL_12942590, EPI_ISL_12942591, EPI_ISL_12942592, EPI_ISL_12942593, EPI_ISL_12942594, EPI_ISL_12942595, EPI_ISL_12942596, EPI_ISL_12942597, EPI_ISL_12942598, EPI_ISL_12942599, EPI_ISL_12942600, EPI_ISL_12942601, EPI_ISL_12942602, EPI_ISL_12942603, EPI_ISL_12942604, EPI_ISL_12942605, EPI_ISL_12942606, EPI_ISL_12942607, EPI_ISL_12942608, EPI_ISL_12942609, EPI_ISL_12942610, EPI_ISL_12942611, EPI_ISL_12942612, EPI_ISL_12942613, EPI_ISL_12942614, EPI_ISL_12942615, EPI_ISL_12942616, EPI_ISL_12942617, EPI_ISL_12942618, EPI_ISL_12942619, EPI_ISL_12942620, EPI_ISL_12942621, EPI_ISL_12942622, EPI_ISL_12942623, EPI_ISL_12942624, EPI_ISL_12942625, EPI_ISL_12942626, EPI_ISL_12942627, EPI_ISL_12942628, EPI_ISL_12942629, EPI_ISL_12942630, EPI_ISL_12942631, EPI_ISL_12942632, EPI_ISL_12942633, EPI_ISL_12942634, EPI_ISL_12942635, EPI_ISL_12942636, EPI_ISL_12942637, EPI_ISL_12942638, EPI_ISL_12942639, EPI_ISL_12942640, EPI_ISL_12942642, EPI_ISL_12942643, EPI_ISL_12942644, EPI_ISL_12942645, EPI_ISL_12942646, EPI_ISL_12942647, EPI_ISL_12942648, EPI_ISL_12942649, EPI_ISL_12942650, EPI_ISL_12942651, EPI_ISL_12942652, EPI_ISL_12942653, EPI_ISL_12942654, EPI_ISL_12942655, EPI_ISL_12942656, EPI_ISL_12942657, EPI_ISL_12942658, EPI_ISL_12942659, EPI_ISL_12970789, EPI_ISL_12970790, EPI_ISL_12972584, EPI_ISL_12972585, EPI_ISL_12972586, EPI_ISL_12972587, EPI_ISL_12972588, EPI_ISL_12972589, EPI_ISL_12972590, EPI_ISL_12972591, EPI_ISL_12972592, EPI_ISL_12972593, EPI_ISL_12972594, EPI_ISL_12972595, EPI_ISL_12972596, EPI_ISL_12972597, EPI_ISL_12972598, EPI_ISL_12972599, EPI_ISL_12972600, EPI_ISL_12972601, EPI_ISL_12972602, EPI_ISL_12972603, EPI_ISL_12972604, EPI_ISL_12972605, EPI_ISL_12972606, EPI_ISL_12972607, EPI_ISL_12972608, EPI_ISL_12972609, EPI_ISL_12972610, EPI_ISL_12972611, EPI_ISL_12972612, EPI_ISL_12972613, EPI_ISL_12972614, EPI_ISL_12972615, EPI_ISL_12972616, EPI_ISL_12972617, EPI_ISL_12972618, EPI_ISL_12972619, EPI_ISL_12972620, EPI_ISL_12972621, EPI_ISL_12972622, EPI_ISL_12972623, EPI_ISL_12972624, EPI_ISL_12972625, EPI_ISL_12972626, EPI_ISL_12972627, EPI_ISL_12972628, EPI_ISL_12972629, EPI_ISL_12972630, EPI_ISL_12972631, EPI_ISL_12972632, EPI_ISL_12972633, EPI_ISL_12972634, EPI_ISL_12972635, EPI_ISL_12972636, EPI_ISL_12972637, EPI_ISL_12972638, EPI_ISL_12972639, EPI_ISL_12972640, EPI_ISL_12972641, EPI_ISL_12972642, EPI_ISL_12972643, EPI_ISL_12972644, EPI_ISL_12972645, EPI_ISL_12972646, EPI_ISL_12972647			
see above	Kuala Lumpur International Airport	Institute for Medical Research, Infectious Disease Research Centre, National Institutes of Health, Ministry of Health Malaysia	Anasir MI; G.Adypatti NM; Jamaluddin MS; Kalyanasundram J; Kamel K; MatRahim N; Nawi MH; Suib FA; Suppiah J; Thayan R
EPI_ISL_11685224, EPI_ISL_11685225, EPI_ISL_11685226, EPI_ISL_11685227, EPI_ISL_11685228, EPI_ISL_11685229, EPI_ISL_11685230, EPI_ISL_11685231, EPI_ISL_11685232, EPI_ISL_11685233, EPI_ISL_11685234, EPI_ISL_11685235, EPI_ISL_11685236, EPI_ISL_11685238, EPI_ISL_11685239, EPI_ISL_12084299, EPI_ISL_12084301, EPI_ISL_12084303, EPI_ISL_12084305, EPI_ISL_12084308, EPI_ISL_12084309, EPI_ISL_12084310, EPI_ISL_12084311, EPI_ISL_12084312, EPI_ISL_12084313, EPI_ISL_12084314			
see above	Kuala Lumpur International Airport (KLIA) Health Office	Institute for Medical Research, Infectious Disease Research Centre, National Institutes of Health, Ministry of Health Malaysia	Ahmad FA; Ahmad Fazilah NA; Anasir MI; Azizan MA; G. Adypatti NM; Jamaluddin MS; Kamel K; Mohamad Sukri MZ; Norhisham SN; Ramly N; Robert F; Rosli NR; Suppiah J; Thayan R
EPI_ISL_12213388	MAKMAL KESIHATAN AWAM JOHOR BAHRU	UKM Medical Molecular Biology Institute (UMBI)	Khairun Nur Abd Ghafar; Mira Farzana Mohamad Mokhtar; Muhiddin Ishak; Nor Azila Muhammad Azami; Nur Alyaa Afifah Md Shahri; Nurul Syakima Ab Mutalib; Rahman Jamal; Ryia Illani Mohd Yunos; Siti Nur Hasanah Mohd Yusuf; Zahirrah Begam Mohamed Rasheed
EPI_ISL_12534887, EPI_ISL_12534888	MAKMAL SEROLOGI, UNIT MIKROBIOLOGI, HSB	Tropical Infectious Diseases Research & Education Centre (TIDREC), Universiti Malaya	AsmaAnati CheMatSeri; Che-Norainon Yaacob; Jia-Yi Tan; Jo-Ern Wong; Kim-Kee Tan; Mulya-Mustika-Sari Zulkifli; Noor Syahida Azizan; Nur-Hidayana Mahfodz; Sazaly AbuBakar; Siti-Sarah Nor'e; Wei-Wei Aw
EPI_ISL_12099659, EPI_ISL_12099661	Miri Hospital Molecular Diagnostic Lab, Miri	Institute of Health and Community Medicine	Chan Chia Jui; Chua Hock Hin; David Perera; Ooi Mong How; Tonnii Sia Loong Loong
EPI_ISL_12213427, EPI_ISL_12213428	PEJABAT KESIHATAN DAERAH MERSING	UKM Medical Molecular Biology Institute (UMBI)	Khairun Nur Abd Ghafar; Mira Farzana Mohamad Mokhtar; Muhiddin Ishak; Nor Azila Muhammad Azami; Nur Alyaa Afifah Md Shahri; Nurul Syakima Ab Mutalib; Rahman Jamal; Ryia Illani Mohd Yunos; Siti Nur Hasanah Mohd Yusuf; Zahirrah Begam Mohamed Rasheed
EPI_ISL_12771236, EPI_ISL_12771237, EPI_ISL_12771238, EPI_ISL_12771297	PKD SPT	iPROMISE, UiTM	Ariza Adnan; Fadzilah Mohd Nor; Lim Wai Feng; Mohd Asif Mohd Sukri; Mohd Nur Fakhruzzaman Noorizhab; Mohd Zaki Salleh; Sazzli Shahlan Kassim; Siti Farah Alwani Mohd Nawi; Siti Hamimah Sheikh Abdul Kadir; Teh Lay Kek; Wang Seok Mui
EPI_ISL_12771251	PKD TIMUR LAUT	iPROMISE, UiTM	Ariza Adnan; Fadzilah Mohd Nor; Lim Wai Feng; Mohd Asif Mohd Sukri; Mohd Nur Fakhruzzaman Noorizhab; Mohd Zaki Salleh; Sazzli Shahlan Kassim; Siti Farah Alwani Mohd Nawi; Siti Hamimah Sheikh Abdul Kadir; Teh Lay Kek; Wang Seok Mui
EPI_ISL_12289514	QUEEN ELIZABETH HOSPITAL	Institute for Medical Research, Infectious Disease Research Centre, National Institutes of Health, Ministry of Health Malaysia	Ahmad FA; Ahmad Fazilah NA; Anasir MI; Azizan MA; Kamel K; Mohamad Sukri MZ; Norhisham SN; Ramly N; Robert F; Rosli NR; Suppiah J; Thayan R
EPI_ISL_12099677, EPI_ISL_12099678, EPI_ISL_12099679	Rejang Medical Centre (Sibu)	Institute of Health and Community Medicine	Chan Chia Jui; Chua Hock Hin; David Perera; Ooi Mong How; Tonnii Sia Loong Loong; Wong Jyn Shan

We gratefully acknowledge the following Authors from the Originating laboratories responsible for obtaining the specimens, as well as the Submitting laboratories where the genome data were generated and shared via GISAID, on which this research is based.

All Submitters of data may be contacted directly via www.gisaid.org

Authors are sorted alphabetically.

Accession ID	Originating Laboratory	Submitting Laboratory	Authors
EPI_ISL_12999616, EPI_ISL_12999617, EPI_ISL_12999618, EPI_ISL_12999619, EPI_ISL_12999620, EPI_ISL_12999621, EPI_ISL_12999622, EPI_ISL_12999623, EPI_ISL_12999624, EPI_ISL_12999625, EPI_ISL_12999626, EPI_ISL_12999627, EPI_ISL_12999628, EPI_ISL_12999629, EPI_ISL_12999630, EPI_ISL_12999631, EPI_ISL_12999632, EPI_ISL_12999633, EPI_ISL_12999634, EPI_ISL_12999635, EPI_ISL_12999636, EPI_ISL_12999637, EPI_ISL_12999638, EPI_ISL_12999639, EPI_ISL_12999640, EPI_ISL_12999641, EPI_ISL_12999642, EPI_ISL_12999643, EPI_ISL_12999644, EPI_ISL_12999645, EPI_ISL_12999646, EPI_ISL_12999647, EPI_ISL_12999648, EPI_ISL_12999649, EPI_ISL_12999650, EPI_ISL_12999651, EPI_ISL_12999652, EPI_ISL_12999653, EPI_ISL_12999654, EPI_ISL_12999655, EPI_ISL_12999656, EPI_ISL_12999657, EPI_ISL_12999658, EPI_ISL_12999659, EPI_ISL_12999660, EPI_ISL_12999662, EPI_ISL_12999663, EPI_ISL_12999664, EPI_ISL_12999665, EPI_ISL_12999666, EPI_ISL_12999667, EPI_ISL_12999668, EPI_ISL_12999669, EPI_ISL_12999670, EPI_ISL_12999671, EPI_ISL_12999672, EPI_ISL_12999673, EPI_ISL_12999674, EPI_ISL_12999675, EPI_ISL_12999676, EPI_ISL_12999677, EPI_ISL_12999678, EPI_ISL_12999679, EPI_ISL_12999680, EPI_ISL_12999681, EPI_ISL_12999682, EPI_ISL_12999683, EPI_ISL_12999684, EPI_ISL_12999685, EPI_ISL_12999686, EPI_ISL_12999687, EPI_ISL_12999688, EPI_ISL_12999689, EPI_ISL_12999690, EPI_ISL_12999691, EPI_ISL_12999692, EPI_ISL_12999693, EPI_ISL_12999694, EPI_ISL_12999695, EPI_ISL_12999696, EPI_ISL_12999697, EPI_ISL_12999698, EPI_ISL_12999699, EPI_ISL_12999700, EPI_ISL_12999701, EPI_ISL_12999702, EPI_ISL_12999703, EPI_ISL_12999704, EPI_ISL_12999705, EPI_ISL_12999706, EPI_ISL_12999707, EPI_ISL_12999708, EPI_ISL_12999709, EPI_ISL_12999710, EPI_ISL_12999711, EPI_ISL_12999712, EPI_ISL_12999714, EPI_ISL_13018617, EPI_ISL_13018618, EPI_ISL_13018619, EPI_ISL_13018620, EPI_ISL_13018621, EPI_ISL_13018622, EPI_ISL_13040782, EPI_ISL_13040783, EPI_ISL_13040784, EPI_ISL_13040785, EPI_ISL_13040786, EPI_ISL_13040787, EPI_ISL_13040788, EPI_ISL_13040789, EPI_ISL_13040790, EPI_ISL_13040791, EPI_ISL_13040792, EPI_ISL_13040793, EPI_ISL_13040794, EPI_ISL_13040795, EPI_ISL_13040796, EPI_ISL_13040797, EPI_ISL_13040798, EPI_ISL_13040799, EPI_ISL_13040800, EPI_ISL_13040801, EPI_ISL_13040802, EPI_ISL_13040803, EPI_ISL_13040804, EPI_ISL_13040805, EPI_ISL_13040806, EPI_ISL_13040807, EPI_ISL_13040808, EPI_ISL_13040809, EPI_ISL_13040810, EPI_ISL_13040811, EPI_ISL_13040812, EPI_ISL_13040813, EPI_ISL_13040814, EPI_ISL_13040815, EPI_ISL_13040826			
see above	Kuala Lumpur International Airport	Institute for Medical Research, Infectious Disease Research Centre, National Institutes of Health, Ministry of Health Malaysia	Anasir MI; G.Adypatti NM; Jamaluddin MS; Kalyanasundram J; Kamel K; MatRahim N; Nawi MH; Suib FA; Suppiah J; Thayan R

We gratefully acknowledge the following Authors from the Originating laboratories responsible for obtaining the specimens, as well as the Submitting laboratories where the genome data were generated and shared via GISAID, on which this research is based.

All Submitters of data may be contacted directly via www.gisaid.org

Authors are sorted alphabetically.

Acknowledgement EPI_SET Identifier: EPI_SET_20220603kz

Accession ID	Originating Laboratory	Submitting Laboratory	Authors
EPI_ISL_12322062, EPI_ISL_12322063	AMPANG HOSPITAL	Institute for Medical Research, Infectious Disease Research Centre, National Institutes of Health, Ministry of Health Malaysia	Ahmad FA; Ahmad Fazilah NA; Anasir MI; Azizan MA; Kamel K; Mohamad Sukri MZ; Norhisham SN; Ramly N; Robert F; Rosli NR; Suppiah J; Thayan R
EPI_ISL_12321872	BEAUFORT HOSPITAL	Institute for Medical Research, Infectious Disease Research Centre, National Institutes of Health, Ministry of Health Malaysia	Ahmad FA; Ahmad Fazilah NA; Anasir MI; Azizan MA; Kamel K; Mohamad Sukri MZ; Norhisham SN; Ramly N; Robert F; Rosli NR; Suppiah J; Thayan R
EPI_ISL_11805039	Bintulu Hospital PCR Lab, Bintulu	Institute of Health and Community Medicine	Chan Chia Jui; Chien Su Lin; Chua Hock Hin; David Perera; Ooi Mong How; Tonnii Sia Loong Loong
EPI_ISL_12099612, EPI_ISL_12099613, EPI_ISL_12099614, EPI_ISL_12099615, EPI_ISL_12099616, EPI_ISL_12099617, EPI_ISL_12099618, EPI_ISL_12099619, EPI_ISL_12099621, EPI_ISL_12099622, EPI_ISL_12510352, EPI_ISL_12510353, EPI_ISL_12510354, EPI_ISL_12510355, EPI_ISL_12510356			
see above	Bintulu Medical Centre (Bintulu)	Institute of Health and Community Medicine	Chan Chia Jui; Chua Hock Hin; David Perera; Ooi Mong How; Tonnii Sia Loong Loong; Wong Jyn Shan
EPI_ISL_12704538, EPI_ISL_12704565	Department of Medical Microbiology and Parasitology, Hospital Universiti Sains Malaysia	Molecular Research Laboratory	Abdul Haris bin Muhammad; Ahmad Sukari Bin Halim; Alexander Chong Shu Chien; Asraihan Che Abdul Malik; Azian Harun; Chan Yean Yean; Chua Wei Chuan; Farahana binti Mohamed; Kirnpal Kaur Banga Singh; Lau Nyok Sean; Lee Lih Huey; Lim Shu Yong; Maizun binti Mohd Zain; Mera Edora Binti Abdul Manap; Mohd Iman hafiz Bin Ibrahim; Mohd Nadzri Abu Yazid; Mohd Zulkifli Salleh; Muhamad Khairul Amirin Bin Zulkifli; Muhammad Azamuddeen bin Mohammad Nasir; Muhammad Fazli bin Khalid; Muhammad Nashrul Farhan Samsudin; Muhammad Zarul Hanifah Bin Md Zoqratt; Nik Zuraina binti Nik Mohd Noor; Noor Hafizan binti Mat Salleh; Nor Amizara Binti Azami; Nor Suhana Binti Mohd Satar; Nur Syuhada Binti Abdul Rahim; Nur-Leem Binti Murshid; Nurfadhlina Musa; Qasim Ayub; Rosline Hassan; Sadequr Rahman; Siti Nur Aisyah Binti Mohamad Sham; Syahida binti Omar; Wan Mohd Zahiruddin Wan Mohammad; Wardah Yusof; Zaini bin Hussin; Zakuan Zainy Deris
EPI_ISL_11805004, EPI_ISL_11805005, EPI_ISL_11805006, EPI_ISL_11805008, EPI_ISL_11805010, EPI_ISL_11805011, EPI_ISL_11805013, EPI_ISL_11805015, EPI_ISL_11805016, EPI_ISL_11805017, EPI_ISL_11805018			
see above	Gribbles Diagnostic Lab @Timberland Medical Centre, Kuching	Institute of Health and Community Medicine	Chan Chia Jui; Chua Hock Hin; David Perera; Liew Chin Chin J; Ooi Mong How; Tonnii Sia Loong Loong; Wong Jyn Shan
EPI_ISL_12213450, EPI_ISL_12628100	HOSPITAL MELAKA	UKM Medical Molecular Biology Institute (UMBI)	Khairun Nur Abd Ghafar; Mira Farzana Mohamad Mokhtar; Muhiddin Ishak; Nor Azila Muhammad Azami; Nur Alyaa Afifah Md Shahri; Nurul Syakima Ab Mutalib; Rahman Jamal; Ryia Illani Mohd Yunos; Siti Nur Hasanah Mohd Yusuf; Zahirrah Begam Mohamed Rasheed
EPI_ISL_12771242, EPI_ISL_12771243, EPI_ISL_12771244, EPI_ISL_12771248, EPI_ISL_12771299	HOSPITAL PULAU PINANG	iPROMISE, UiTM	Ariza Adnan; Fadzilah Mohd Nor; Lim Wai Feng; Mohd Asif Mohd Sukri; Mohd Nur Fakhruzzaman Noorizhab; Mohd Zaki Salleh; Sazzli Shahlan Kassim; Siti Farah Alwani Mohd Nawi; Siti Hamimah Sheikh Abdul Kadir; Teh Lay Kek; Wang Seok Mui
EPI_ISL_12534812	HOSPITAL RAJA PERMAISURI BAINUN	Tropical Infectious Diseases Research & Education Centre (TIDREC), Universiti Malaya	AsmaAnati CheMatSeri; Che-Norainon Yaacob; Jia-Yi Tan; Jo-Ern Wong; Kim-Kee Tan; Mulya-Mustika-Sari Zulkifli; Noor Syahida Azizan; Nur-Hidayana Mahfodz; Sazaly AbuBakar; Siti-Sarah Nor'e; Wei-Wei Aw
EPI_ISL_13047110, EPI_ISL_13047111	HOSPITAL SULTAN ISMAIL PETRA, KUALA KRAI, KELANTAN	MOLECULAR RESEARCH LABORATORY	Abdul Haris bin Muhammad; Ahmad Sukari Bin Halim; Alexander Chong Shu Chien; Azian Harun; Chan Yean Yean; Chua Wei Chuan; Farahana binti Mohamed; Kirnpal Kaur Banga Singh; Lau Nyok Sean; Lee Lih Huey; Lim Shu Yong; Maizun binti Mohd Zain; Mohd Nadzri Abu Yazid; Mohd Zulkifli Salleh; Muhammad Azamuddeen bin Mohammad Nasir; Muhammad Fazli bin Khalid; Muhammad Nashrul Farhan Samsudin; Muhammad Zarul Hanifah Bin Md Zoqratt; Nik Zuraina binti Nik Mohd Noor; Noor Hafizan binti Mat Salleh; Nurfadhlina Musa; Qasim Ayub; Rosline Hassan; Sadequr Rahman; Syahida binti Omar; Wan Mohd Zahiruddin Wan Mohammad; Wardah Yusof; Zaini bin Hussin; Zakuan Zainy Deris
EPI_ISL_12534769, EPI_ISL_12534770, EPI_ISL_12534771	HOSPITAL SULTANAH NUR ZAHIRAH	Tropical Infectious Diseases Research & Education Centre (TIDREC), Universiti Malaya	AsmaAnati CheMatSeri; Che-Norainon Yaacob; Jia-Yi Tan; Jo-Ern Wong; Kim-Kee Tan; Mulya-Mustika-Sari Zulkifli; Noor Syahida Azizan; Nur-Hidayana Mahfodz; Sazaly AbuBakar; Siti-Sarah Nor'e; Wei-Wei Aw
EPI_ISL_12534809, EPI_ISL_12534810, EPI_ISL_12534811, EPI_ISL_12534813, EPI_ISL_12534814, EPI_ISL_12534815	HRPB	Tropical Infectious Diseases Research & Education Centre (TIDREC), Universiti Malaya	AsmaAnati CheMatSeri; Che-Norainon Yaacob; Jia-Yi Tan; Jo-Ern Wong; Kim-Kee Tan; Mulya-Mustika-Sari Zulkifli; Noor Syahida Azizan; Nur-Hidayana Mahfodz; Sazaly AbuBakar; Siti-Sarah Nor'e; Wei-Wei Aw
EPI_ISL_12213401, EPI_ISL_12213402, EPI_ISL_12213403, EPI_ISL_12213404, EPI_ISL_12213451	HSAJB	UKM Medical Molecular Biology Institute (UMBI)	Khairun Nur Abd Ghafar; Mira Farzana Mohamad Mokhtar; Muhiddin Ishak; Nor Azila Muhammad Azami; Nur Alyaa Afifah Md Shahri; Nurul Syakima Ab Mutalib; Rahman Jamal; Ryia Illani Mohd Yunos; Siti Nur Hasanah Mohd Yusuf; Zahirrah Begam Mohamed Rasheed
EPI_ISL_12213392, EPI_ISL_12213393, EPI_ISL_12213394, EPI_ISL_12213395, EPI_ISL_12213396, EPI_ISL_12213416, EPI_ISL_12213417, EPI_ISL_12213418, EPI_ISL_12213419, EPI_ISL_12213420			
see above	HTJS	UKM Medical Molecular Biology Institute (UMBI)	Khairun Nur Abd Ghafar; Mira Farzana Mohamad Mokhtar; Muhiddin Ishak; Nor Azila Muhammad Azami; Nur Alyaa Afifah Md Shahri; Nurul Syakima Ab Mutalib; Rahman Jamal; Ryia Illani Mohd Yunos; Siti Nur Hasanah Mohd Yusuf; Zahirrah Begam Mohamed Rasheed
EPI_ISL_12704552, EPI_ISL_12704553, EPI_ISL_12704554	Hospital Raja Perempuan Zainab II, Kota Bharu	Molecular Research Laboratory	Abdul Haris bin Muhammad; Ahmad Sukari Bin Halim; Alexander Chong Shu Chien; Asraihan Che Abdul Malik; Azian Harun; Chan Yean Yean; Chua Wei Chuan; Farahana binti Mohamed; Kirnpal Kaur Banga Singh; Lau Nyok Sean; Lee Lih Huey; Lim Shu Yong; Maizun binti Mohd Zain; Mera Edora Binti Abdul Manap; Mohd Iman hafiz Bin Ibrahim; Mohd Nadzri Abu Yazid; Mohd Zulkifli Salleh; Muhamad Khairul Amirin Bin Zulkifli; Muhammad Azamuddeen bin Mohammad Nasir; Muhammad Fazli bin Khalid; Muhammad Nashrul Farhan Samsudin; Muhammad Zarul Hanifah Bin Md Zoqratt; Nik Zuraina binti Nik Mohd Noor; Noor Hafizan binti Mat Salleh; Nor Amizara Binti Azami; Nor Suhana Binti Mohd Satar; Nur Syuhada Binti Abdul Rahim; Nur-Leem Binti Murshid; Nurfadhlina Musa; Qasim Ayub; Rosline Hassan; Sadequr Rahman; Siti Nur Aisyah Binti Mohamad Sham; Syahida binti Omar; Wan Mohd Zahiruddin Wan Mohammad; Wardah Yusof; Zaini bin Hussin; Zakuan Zainy Deris
EPI_ISL_12899674	KEPALA BATAS HOSPITAL	Institute for Medical Research, Infectious Disease Research Centre, National Institutes of Health, Ministry of Health Malaysia	Ahmad FA; Ahmad Fazilah NA; Anasir MI; Azizan MA; Kamel K; Mohamad Sukri MZ; Norhisham SN; Ramly N; Robert F; Rosli NR; Suppiah J; Thayan R
EPI_ISL_12746948, EPI_ISL_12746949	KOTA KINABALU AREA HEALTH OFFICE	Institute for Medical Research, Infectious Disease Research Centre, National Institutes of Health, Ministry of Health Malaysia	Ahmad FA; Ahmad Fazilah NA; Anasir MI; Azizan MA; Kamel K; Mohamad Sukri MZ; Norhisham SN; Ramly N; Robert F; Rosli NR; Suppiah J; Thayan R
EPI_ISL_12899689	KUALA LUMPUR GENERAL HOSPITAL	Institute for Medical Research, Infectious Disease Research Centre, National Institutes of Health, Ministry of Health Malaysia	Ahmad FA; Ahmad Fazilah NA; Anasir MI; Azizan MA; Kamel K; Mohamad Sukri MZ; Norhisham SN; Ramly N; Robert F; Rosli NR; Suppiah J; Thayan R
EPI_ISL_12665276, EPI_ISL_12665286, EPI_ISL_12665287, EPI_ISL_12665288, EPI_ISL_12665289, EPI_ISL_12665290, EPI_ISL_12665291, EPI_ISL_12665292, EPI_ISL_12665293, EPI_ISL_12665294, EPI_ISL_12665295, EPI_ISL_12665296, EPI_ISL_12665297, EPI_ISL_12665298, EPI_ISL_12665299, EPI_ISL_12665300, EPI_ISL_12665302, EPI_ISL_12749680, EPI_ISL_12749685, EPI_ISL_12749686			
see above	KUALA LUMPUR INTERNATIONAL AIRPORT (KLIA) HEALTH OFFICE	Institute for Medical Research, Infectious Disease Research Centre, National Institutes of Health, Ministry of Health Malaysia	Ahmad FA; Ahmad Fazilah NA; Anasir MI; Azizan MA; Kamel K; Mohamad Sukri MZ; Norhisham SN; Ramly N; Robert F; Rosli NR; Suppiah J; Thayan R
EPI_ISL_12746988, EPI_ISL_12746989	KULIM HOSPITAL	Institute for Medical Research, Infectious Disease Research Centre, National Institutes of Health, Ministry of Health Malaysia	Ahmad FA; Ahmad Fazilah NA; Anasir MI; Azizan MA; Kamel K; Mohamad Sukri MZ; Norhisham SN; Ramly N; Robert F; Rosli NR; Suppiah J; Thayan R
EPI_ISL_12708552, EPI_ISL_12708553, EPI_ISL_12708563	Kota Bharu Public Health Laboratory	Molecular Research Laboratory	Abdul Haris bin Muhammad; Ahmad Sukari Bin Halim; Alexander Chong Shu Chien; Asraihan Che Abdul Malik; Azian Harun; Chan Yean Yean; Chua Wei Chuan; Farahana binti Mohamed; Kirnpal Kaur Banga Singh; Lau Nyok Sean; Lee Lih Huey; Lim Shu Yong; Maizun binti Mohd Zain; Mera Edora Binti Abdul Manap; Mohd Iman hafiz Bin Ibrahim; Mohd Nadzri Abu Yazid; Mohd Zulkifli Salleh; Muhamad Khairul Amirin Bin Zulkifli; Muhammad Azamuddeen bin Mohammad Nasir; Muhammad Fazli bin Khalid; Muhammad Nashrul Farhan Samsudin; Muhammad Zarul Hanifah Bin Md Zoqratt; Nik Zuraina binti Nik Mohd Noor; Noor Hafizan binti Mat Salleh; Nor Amizara Binti Azami; Nor Suhana Binti Mohd Satar; Nur Syuhada Binti Abdul Rahim; Nur-Leem Binti Murshid; Nurfadhlina Musa; Qasim Ayub; Rosline Hassan; Sadequr Rahman; Siti Nur Aisyah Binti Mohamad Sham; Syahida binti Omar; Wan Mohd Zahiruddin Wan Mohammad; Wardah Yusof; Zaini bin Hussin; Zakuan Zainy Deris
EPI_ISL_12004077, EPI_ISL_12004078, EPI_ISL_12004110, EPI_ISL_12891210, EPI_ISL_12891211, EPI_ISL_12891212, EPI_ISL_12891213, EPI_ISL_12891214, EPI_ISL_12891215, EPI_ISL_12891216, EPI_ISL_12911640, EPI_ISL_12911641, EPI_ISL_12911642, EPI_ISL_12911643, EPI_ISL_12911644, EPI_ISL_12911645, EPI_ISL_12911646, EPI_ISL_12911647, EPI_ISL_12911648, EPI_ISL_12911649, EPI_ISL_12911650, EPI_ISL_12911651, EPI_ISL_12911652, EPI_ISL_12911653, EPI_ISL_12911654, EPI_ISL_12911655, EPI_ISL_12911656, EPI_ISL_12911657, EPI_ISL_12911658, EPI_ISL_12911659, EPI_ISL_12911660, EPI_ISL_12911661, EPI_ISL_12911662, EPI_ISL_12911663, EPI_ISL_12911664, EPI_ISL_12911665, EPI_ISL_12911666, EPI_ISL_12911667, EPI_ISL_12911668, EPI_ISL_12911669, EPI_ISL_12911670, EPI_ISL_12911671, EPI_ISL_12911672, EPI_ISL_12911673, EPI_ISL_12911674, EPI_ISL_12911675, EPI_ISL_12911676, EPI_ISL_12911677, EPI_ISL_12911678, EPI_ISL_12911679, EPI_ISL_12911680, EPI_ISL_12911681, EPI_ISL_12911682, EPI_ISL_12911683, EPI_ISL_12911684, EPI_ISL_12911685, EPI_ISL_12911686, EPI_ISL_12911687, EPI_ISL_12911688, EPI_ISL_12911689, EPI_ISL_12911690, EPI_ISL_12911691, EPI_ISL_12911702, EPI_ISL_12911703, EPI_ISL_12911704, EPI_ISL_12911705, EPI_ISL_12911706, EPI_ISL_12911707, EPI_ISL_12911708, EPI_ISL_12911709, EPI_ISL_12911710, EPI_ISL_12911711, EPI_ISL_12911712, EPI_ISL_12911713, EPI_ISL_12911714, EPI_ISL_12911715, EPI_ISL_12911716, EPI_ISL_12911717, EPI_ISL_12911718, EPI_ISL_12911719, EPI_ISL_12911720, EPI_ISL_12911721, EPI_ISL_12911722, EPI_ISL_12911723, EPI_ISL_12911724, EPI_ISL_12911725, EPI_ISL_12911726, EPI_ISL_12911727, EPI_ISL_12911728, EPI_ISL_12911729, EPI_ISL_12911730, EPI_ISL_12911731, EPI_ISL_12911732, EPI_ISL_12911733, EPI_ISL_12911734, EPI_ISL_12911735, EPI_ISL_12911736, EPI_ISL_12911737, EPI_ISL_12911738, EPI_ISL_12911739,			

EPI_ISL_12911740, EPI_ISL_12911741, EPI_ISL_12911742, EPI_ISL_12911743, EPI_ISL_12911744, EPI_ISL_12911745, EPI_ISL_12911746, EPI_ISL_12911747, EPI_ISL_12911748, EPI_ISL_12911749, EPI_ISL_12911750, EPI_ISL_12911751, EPI_ISL_12911752, EPI_ISL_12911753, EPI_ISL_12911754, EPI_ISL_12911755, EPI_ISL_12911756, EPI_ISL_12911757, EPI_ISL_12911758, EPI_ISL_12911759, EPI_ISL_12911760, EPI_ISL_12911761, EPI_ISL_12911762, EPI_ISL_12911763, EPI_ISL_12911764, EPI_ISL_12911765, EPI_ISL_12911766, EPI_ISL_12911767, EPI_ISL_12911768, EPI_ISL_12911769, EPI_ISL_12911770, EPI_ISL_12911771, EPI_ISL_12911772, EPI_ISL_12911773, EPI_ISL_12911774, EPI_ISL_12911775, EPI_ISL_12911776, EPI_ISL_12911777, EPI_ISL_12911778, EPI_ISL_12911779, EPI_ISL_12911780, EPI_ISL_12911781, EPI_ISL_12911782, EPI_ISL_12911783, EPI_ISL_12911784, EPI_ISL_12911785, EPI_ISL_12911786, EPI_ISL_12911787, EPI_ISL_12911788, EPI_ISL_12911789, EPI_ISL_12911790, EPI_ISL_12911791, EPI_ISL_12911792, EPI_ISL_12911793, EPI_ISL_12911794, EPI_ISL_12911795, EPI_ISL_12911796, EPI_ISL_12911797, EPI_ISL_12911798, EPI_ISL_12911799, EPI_ISL_12911800, EPI_ISL_12911801, EPI_ISL_12911802, EPI_ISL_12911803, EPI_ISL_12911804, EPI_ISL_12911805, EPI_ISL_12911806, EPI_ISL_12911807, EPI_ISL_12911808, EPI_ISL_12911809, EPI_ISL_12911810, EPI_ISL_12911811, EPI_ISL_12911812, EPI_ISL_12911813, EPI_ISL_12911814, EPI_ISL_12911815, EPI_ISL_12911816, EPI_ISL_12911817, EPI_ISL_12911818, EPI_ISL_12911819, EPI_ISL_12911820, EPI_ISL_12911821, EPI_ISL_12911822, EPI_ISL_12911823, EPI_ISL_12911824, EPI_ISL_12911825, EPI_ISL_12911826, EPI_ISL_12911827, EPI_ISL_12911828, EPI_ISL_12911829, EPI_ISL_12911830, EPI_ISL_12911831, EPI_ISL_12973047, EPI_ISL_12973048, EPI_ISL_12973049, EPI_ISL_12973050, EPI_ISL_12973051, EPI_ISL_12973052, EPI_ISL_12973053, EPI_ISL_12973054, EPI_ISL_12973055, EPI_ISL_12973056, EPI_ISL_12973057, EPI_ISL_12973058, EPI_ISL_12973059, EPI_ISL_12973060, EPI_ISL_12973061, EPI_ISL_12973062, EPI_ISL_12973063			
see above	Kuala Lumpur International Airport	Institute for Medical Research, Infectious Disease Research Centre, National Institutes of Health, Ministry of Health Malaysia	Anasir MI; G.Adypatti NM; Jamaluddin MS; Kalyanasundram J; Kamel K; MatRahim N; Nawi MH; Suib FA; Suppiah J; Thayan R
EPI_ISL_12321868	LAHAD DATU HOSPITAL	Institute for Medical Research, Infectious Disease Research Centre, National Institutes of Health, Ministry of Health Malaysia	Ahmad FA; Ahmad Fazilah NA; Anasir MI; Azizan MA; Kamel K; Mohamad Sukri MZ; Norhisham SN; Ramly N; Robert F; Rosli NR; Suppiah J; Thayan R
EPI_ISL_12213436	MAKMAL KESIHATAN AWAM JHOR BAHRU	UKM Medical Molecular Biology Institute (UMBI)	Khairun Nur Abd Ghafar; Mira Farzana Mohamad Mokhtar; Muhiddin Ishak; Nor Azila Muhammad Azami; Nur Alyaa Afifah Md Shahri; Nurul Syakima Ab Mutalib; Rahman Jamal; Ryia Illani Mohd Yunos; Siti Nur Hasanah Mohd Yusuf; Zahirrah Begam Mohamed Rasheed
EPI_ISL_12213389, EPI_ISL_12213390, EPI_ISL_12213429, EPI_ISL_12213430	MAKMAL KESIHATAN AWAM JOHOR BAHRU	UKM Medical Molecular Biology Institute (UMBI)	Khairun Nur Abd Ghafar; Mira Farzana Mohamad Mokhtar; Muhiddin Ishak; Nor Azila Muhammad Azami; Nur Alyaa Afifah Md Shahri; Nurul Syakima Ab Mutalib; Rahman Jamal; Ryia Illani Mohd Yunos; Siti Nur Hasanah Mohd Yusuf; Zahirrah Begam Mohamed Rasheed
EPI_ISL_12628144, EPI_ISL_12628145, EPI_ISL_12628146	MAKMAL KESIHATAN JOHOR	UKM Medical Molecular Biology Institute (UMBI)	Khairun Nur Abd Ghafar; Mira Farzana Mohamad Mokhtar; Muhiddin Ishak; Nor Azila Muhammad Azami; Nur Alyaa Afifah Md Shahri; Nurul Syakima Ab Mutalib; Rahman Jamal; Ryia Illani Mohd Yunos; Siti Nur Hasanah Mohd Yusuf; Zahirrah Begam Mohamed Rasheed
EPI_ISL_12534891	MAKMAL MIKROBIOLOGI HOSPITAL SULTAN ABDUL HALIM	Tropical Infectious Diseases Research & Education Centre (TIDREC), Universiti Malaya	AsmaAnati CheMatSeri; Che-Norainon Yaacob; Jia-Yi Tan; Jo-Ern Wong; Kim-Kee Tan; Mulya-Mustika-Sari Zulkifli; Noor Syahida Azizan; Nur-Hidayana Mahfodz; Sazaly AbuBakar; Siti-Sarah Nor'e; Wei-Wei Aw
EPI_ISL_12534889, EPI_ISL_12534890, EPI_ISL_12534892, EPI_ISL_12534893	MAKMAL SEROLOGI, UNIT MIKROBIOLOGI, HSB	Tropical Infectious Diseases Research & Education Centre (TIDREC), Universiti Malaya	AsmaAnati CheMatSeri; Che-Norainon Yaacob; Jia-Yi Tan; Jo-Ern Wong; Kim-Kee Tan; Mulya-Mustika-Sari Zulkifli; Noor Syahida Azizan; Nur-Hidayana Mahfodz; Sazaly AbuBakar; Siti-Sarah Nor'e; Wei-Wei Aw
EPI_ISL_12704551	Medical Clinic Pasir Puteh	Molecular Research Laboratory	Abdul Haris bin Muhammad; Ahmad Sukari Bin Halim; Alexander Chong Shu Chien; Asraihan Che Abdul Malik; Azian Harun; Chan Yean Yean; Chua Wei Chuan; Farahana binti Mohamed; Kirnpal Kaur Banga Singh; Lau Nyok Sean; Lee Lih Huey; Lim Shu Yong; Maizun binti Mohd Zain; Mera Edora Binti Abdul Manap; Mohd Iman hafiz Bin Ibrahim; Mohd Nadzri Abu Yazid; Mohd Zulkifli Salleh; Muhamad Khairul Amirin Bin Zulkifli; Muhammad Azamuddeen bin Mohammad Nasir; Muhammad Fazli bin Khalid; Muhammad Nashrul Farhan Samsudin; Muhammad Zarul Hanifah Bin Md Zoqratt; Nik Zuraina binti Nik Mohd Noor; Noor Hafizan binti Mat Salleh; Nor Amizara Binti Azami; Nor Suhana Binti Mohd Satar; Nur Syuhada Binti Abdul Rahim; Nur-Leem Binti Murshid; Nurfadhlina Musa; Qasim Ayub; Rosline Hassan; Sadequr Rahman; Siti Nur Aisyah Binti Mohamad Sham; Syahida binti Omar; Wan Mohd Zahiruddin Wan Mohammad; Wardah Yusof; Zaini bin Hussin; Zakuan Zainy Deris
EPI_ISL_11804985, EPI_ISL_11804986, EPI_ISL_12099654, EPI_ISL_12099655, EPI_ISL_12099660, EPI_ISL_12099662, EPI_ISL_12099663, EPI_ISL_12099664, EPI_ISL_12099665			
see above	Miri Hospital Molecular Diagnostic Lab, Miri	Institute of Health and Community Medicine	Chan Chia Jui; Chua Hock Hin; David Perera; Ooi Mong How; Tonnii Sia Loong Loong
EPI_ISL_13026071, EPI_ISL_13026072	Neogenix Laboratoire Sdn Bhd	Universiti Putra Malaysia (UPM)	Chee Sian Kuan; Choo Yee Yu; Hui Yee Chee; Nancy Woan Charn Liew; Narcisse Joseph; Nurulfiza Mat Isa; Sie Yeng Wong; Su Mei Yew; Syafinaz Amin-Nordin; Zunita Zakaria
EPI_ISL_12213437, EPI_ISL_12213442	PKD JOHOR BAHRU	UKM Medical Molecular Biology Institute (UMBI)	Khairun Nur Abd Ghafar; Mira Farzana Mohamad Mokhtar; Muhiddin Ishak; Nor Azila Muhammad Azami; Nur Alyaa Afifah Md Shahri; Nurul Syakima Ab Mutalib; Rahman Jamal; Ryia Illani Mohd Yunos; Siti Nur Hasanah Mohd Yusuf; Zahirrah Begam Mohamed Rasheed
EPI_ISL_12289480, EPI_ISL_12289481	PULAU PINANG HOSPITAL	Institute for Medical Research, Infectious Disease Research Centre, National Institutes of Health, Ministry of Health Malaysia	Ahmad FA; Ahmad Fazilah NA; Anasir MI; Azizan MA; Kamel K; Mohamad Sukri MZ; Norhisham SN; Ramly N; Robert F; Rosli NR; Suppiah J; Thayan R
EPI_ISL_12289515, EPI_ISL_12289517, EPI_ISL_12899664	QUEEN ELIZABETH HOSPITAL	Institute for Medical Research, Infectious Disease Research Centre, National Institutes of Health, Ministry of Health Malaysia	Ahmad FA; Ahmad Fazilah NA; Anasir MI; Azizan MA; Kamel K; Mohamad Sukri MZ; Norhisham SN; Ramly N; Robert F; Rosli NR; Suppiah J; Thayan R
EPI_ISL_12510006, EPI_ISL_12510007, EPI_ISL_12510008, EPI_ISL_12510009, EPI_ISL_12510010, EPI_ISL_12510011, EPI_ISL_12510012, EPI_ISL_12510014, EPI_ISL_12510015, EPI_ISL_12510016, EPI_ISL_12510017, EPI_ISL_12510018, EPI_ISL_12510019, EPI_ISL_12510021, EPI_ISL_12510022, EPI_ISL_12510023			
see above	Rejang Medical Centre (Sibu)	Institute of Health and Community Medicine	Chan Chia Jui; David Perera; Ooi Mong How; Wong Jyn Shan
EPI_ISL_12289484, EPI_ISL_12899704, EPI_ISL_12899705	SULTANAH BAHIYAH HOSPITAL	Institute for Medical Research, Infectious Disease Research Centre, National Institutes of Health, Ministry of Health Malaysia	Ahmad FA; Ahmad Fazilah NA; Anasir MI; Azizan MA; Kamel K; Mohamad Sukri MZ; Norhisham SN; Ramly N; Robert F; Rosli NR; Suppiah J; Thayan R
EPI_ISL_11804981, EPI_ISL_11804982, EPI_ISL_12099683, EPI_ISL_12099747, EPI_ISL_12509994	Sarawak General Hospital (Kuching)	Institute of Health and Community Medicine	Chan Chia Jui; Chua Hock Hin; David Perera; Ooi Mong How; Tonnii Sia Loong Loong

We gratefully acknowledge the following Authors from the Originating laboratories responsible for obtaining the specimens, as well as the Submitting laboratories where the genome data were generated and shared via GISAID, on which this research is based.

All Submitters of data may be contacted directly via www.gisaid.org

Authors are sorted alphabetically.

Accession ID	Originating Laboratory	Submitting Laboratory	Authors
EPI_ISL_12510263, EPI_ISL_12510264, EPI_ISL_12510265, EPI_ISL_12510266, EPI_ISL_12510267, EPI_ISL_12510268, EPI_ISL_12510269			
see above	Bintulu Medical Centre (Bintulu)	Institute of Health and Community Medicine	Chan Chia Jui; David Perera; Ooi Mong How; Wong Jyn Shan
EPI_ISL_12510123, EPI_ISL_12510124, EPI_ISL_12510125, EPI_ISL_12510126, EPI_ISL_12510127, EPI_ISL_12510128, EPI_ISL_12510129, EPI_ISL_12510130, EPI_ISL_12510132, EPI_ISL_12510133, EPI_ISL_12510135, EPI_ISL_12510392, EPI_ISL_12510393, EPI_ISL_12510394, EPI_ISL_12510395, EPI_ISL_12510396, EPI_ISL_12510397, EPI_ISL_12510398, EPI_ISL_12510399, EPI_ISL_12510400			
see above	Borneo Medical Centre (Kuching)	Institute of Health and Community Medicine	Chan Chia Jui; David Perera; Ooi Mong How; Wong Jyn Shan
EPI_ISL_12213415	Dr Hazeem bin Ahmayuddin	UKM Medical Molecular Biology Institute (UMBI)	Khairun Nur Abd Ghafar; Mira Farzana Mohamad Mokhtar; Muhiddin Ishak; Nor Azila Muhammad Azami; Nur Alyaa Afifah Md Shahri; Nurul Syakima Ab Mutalib; Rahman Jamal; Ryia Illani Mohd Yunos; Siti Nur Hasanah Mohd Yusuf; Zahirrah Begam Mohamed Rasheed
EPI_ISL_12534822	HOSPITAL RAJA PERMAISURI BAINUN	Tropical Infectious Diseases Research & Education Centre (TIDREC), Universiti Malaya	AsmaAnati CheMatSeri; Che-Norainon Yaacob; Jia-Yi Tan; Jo-Ern Wong; Kim-Kee Tan; Mulya-Mustika-Sari Zulkifli; Noor Syahida Azizan; Nur-Hidayana Mahfodz; Sazaly AbuBakar; Siti-Sarah Nor'e; Wei-Wei Aw
EPI_ISL_12534773	HOSPITAL SULTANAH NUR ZAHIRAH	Tropical Infectious Diseases Research & Education Centre (TIDREC), Universiti Malaya	AsmaAnati CheMatSeri; Che-Norainon Yaacob; Jia-Yi Tan; Jo-Ern Wong; Kim-Kee Tan; Mulya-Mustika-Sari Zulkifli; Noor Syahida Azizan; Nur-Hidayana Mahfodz; Sazaly AbuBakar; Siti-Sarah Nor'e; Wei-Wei Aw
EPI_ISL_12213452	HSAJB	UKM Medical Molecular Biology Institute (UMBI)	Khairun Nur Abd Ghafar; Mira Farzana Mohamad Mokhtar; Muhiddin Ishak; Nor Azila Muhammad Azami; Nur Alyaa Afifah Md Shahri; Nurul Syakima Ab Mutalib; Rahman Jamal; Ryia Illani Mohd Yunos; Siti Nur Hasanah Mohd Yusuf; Zahirrah Begam Mohamed Rasheed
EPI_ISL_12704555	Hospital Raja Perempuan Zainab II, Kota Bharu	Molecular Research Laboratory	Abdul Haris bin Muhammad; Ahmad Sukari Bin Halim; Alexander Chong Shu Chien; Asraihan Che Abdul Malik; Azian Harun; Chan Yean Yean; Chua Wei Chuan; Farahana binti Mohamed; Kirnpal Kaur Banga Singh; Lau Nyok Sean; Lee Lih Huey; Lim Shu Yong; Maizun binti Mohd Zain; Mera Edora Binti Abdul Manap; Mohd Iman hafiz Bin Ibrahim; Mohd Nadzri Abu Yazid; Mohd Zulkifli Salleh; Muhamad Khairul Amirin Bin Zulkifli; Muhammad Azamuddeen bin Mohammad Nasir; Muhammad Fazli bin Khalid; Muhammad Nashrul Farhan Samsudin; Muhammad Zarul Hanifah Bin Md Zoqratt; Nik Zuraina binti Nik Mohd Noor; Noor Hafizan binti Mat Salleh; Nor Amizara Binti Azami; Nor Suhana Binti Mohd Satar; Nur Syuhada Binti Abdul Rahim; Nur-Leem Binti Murshid; Nurfadhlina Musa; Qasim Ayub; Rosline Hassan; Sadequr Rahman; Siti Nur Aisyah Binti Mohamad Sham; Syahida binti Omar; Wan Mohd Zahiruddin Wan Mohammad; Wardah Yusof; Zaini bin Hussin; Zakuan Zainy Deris
EPI_ISL_12899717	KENINGAU HOSPITAL	Institute for Medical Research, Infectious Disease Research Centre, National Institutes of Health, Ministry of Health Malaysia	Ahmad FA; Ahmad Fazilah NA; Anasir MI; Azizan MA; Kamel K; Mohamad Sukri MZ; Norhisham SN; Ramly N; Robert F; Rosli NR; Suppiah J; Thayan R
EPI_ISL_12708561	Kota Bharu Public Health Laboratory	Molecular Research Laboratory	Abdul Haris bin Muhammad; Ahmad Sukari Bin Halim; Alexander Chong Shu Chien; Asraihan Che Abdul Malik; Azian Harun; Chan Yean Yean; Chua Wei Chuan; Farahana binti Mohamed; Kirnpal Kaur Banga Singh; Lau Nyok Sean; Lee Lih Huey; Lim Shu Yong; Maizun binti Mohd Zain; Mera Edora Binti Abdul Manap; Mohd Iman hafiz Bin Ibrahim; Mohd Nadzri Abu Yazid; Mohd Zulkifli Salleh; Muhamad Khairul Amirin Bin Zulkifli; Muhammad Azamuddeen bin Mohammad Nasir; Muhammad Fazli bin Khalid; Muhammad Nashrul Farhan Samsudin; Muhammad Zarul Hanifah Bin Md Zoqratt; Nik Zuraina binti Nik Mohd Noor; Noor Hafizan binti Mat Salleh; Nor Amizara Binti Azami; Nor Suhana Binti Mohd Satar; Nur Syuhada Binti Abdul Rahim; Nur-Leem Binti Murshid; Nurfadhlina Musa; Qasim Ayub; Rosline Hassan; Sadequr Rahman; Siti Nur Aisyah Binti Mohamad Sham; Syahida binti Omar; Wan Mohd Zahiruddin Wan Mohammad; Wardah Yusof; Zaini bin Hussin; Zakuan Zainy Deris
EPI_ISL_12004079, EPI_ISL_12004080, EPI_ISL_12004081, EPI_ISL_12004082, EPI_ISL_12004083, EPI_ISL_12004084, EPI_ISL_12004085, EPI_ISL_12004086, EPI_ISL_12004087, EPI_ISL_12004088, EPI_ISL_12004089, EPI_ISL_12004090, EPI_ISL_12004091, EPI_ISL_12004092, EPI_ISL_12004093, EPI_ISL_12004094, EPI_ISL_12004095, EPI_ISL_12004096, EPI_ISL_12004097, EPI_ISL_12004098, EPI_ISL_12004099, EPI_ISL_12004100, EPI_ISL_12004101, EPI_ISL_12004102, EPI_ISL_12004103, EPI_ISL_12004104, EPI_ISL_12004105, EPI_ISL_12004106, EPI_ISL_12004107, EPI_ISL_12004108, EPI_ISL_12004109, EPI_ISL_12004111, EPI_ISL_12004112, EPI_ISL_12004113, EPI_ISL_12004114, EPI_ISL_12004115, EPI_ISL_12004116, EPI_ISL_12004117, EPI_ISL_12004118, EPI_ISL_12004119, EPI_ISL_12004120, EPI_ISL_12004121, EPI_ISL_12004122, EPI_ISL_12004123, EPI_ISL_12004124, EPI_ISL_12004125, EPI_ISL_12004126, EPI_ISL_12004127, EPI_ISL_12004128, EPI_ISL_12004129, EPI_ISL_12004130, EPI_ISL_12004131, EPI_ISL_12004132, EPI_ISL_12004133, EPI_ISL_12004134, EPI_ISL_12004135, EPI_ISL_12004136, EPI_ISL_12004137, EPI_ISL_12004138, EPI_ISL_12004139, EPI_ISL_12004140, EPI_ISL_12004141, EPI_ISL_12004142, EPI_ISL_12004143, EPI_ISL_12004144, EPI_ISL_12004145, EPI_ISL_12004146, EPI_ISL_12008343, EPI_ISL_12008344, EPI_ISL_12008345, EPI_ISL_12008346, EPI_ISL_12008347, EPI_ISL_12008348, EPI_ISL_12008349, EPI_ISL_12008350, EPI_ISL_12008351, EPI_ISL_12008352, EPI_ISL_12008353, EPI_ISL_12008354, EPI_ISL_12008355, EPI_ISL_12008356, EPI_ISL_12008357, EPI_ISL_12008358, EPI_ISL_12008359, EPI_ISL_12008360, EPI_ISL_12008361, EPI_ISL_12008362, EPI_ISL_12008363, EPI_ISL_12008364, EPI_ISL_12008365, EPI_ISL_12008366, EPI_ISL_12008367, EPI_ISL_12008368, EPI_ISL_12008369, EPI_ISL_12008370, EPI_ISL_12008371, EPI_ISL_12008372, EPI_ISL_12008373, EPI_ISL_12008374, EPI_ISL_12008375, EPI_ISL_12008376, EPI_ISL_12008377, EPI_ISL_12008402, EPI_ISL_12008403, EPI_ISL_12008404, EPI_ISL_12008405, EPI_ISL_12008406, EPI_ISL_12008407, EPI_ISL_12008408, EPI_ISL_12010098, EPI_ISL_12010099, EPI_ISL_12010100, EPI_ISL_12010101, EPI_ISL_12010102, EPI_ISL_12010103, EPI_ISL_12010104, EPI_ISL_12010105, EPI_ISL_12010106, EPI_ISL_12010107, EPI_ISL_12010108, EPI_ISL_12010109, EPI_ISL_12010110, EPI_ISL_12010111, EPI_ISL_12010112, EPI_ISL_12010113, EPI_ISL_12010114, EPI_ISL_12010115			
see above	Kuala Lumpur International Airport	Institute for Medical Research, Infectious Disease Research Centre, National Institutes of Health, Ministry of Health Malaysia	Anasir MI; G.Adypatti NM; Jamaluddin MS; Kalyanasundram J; Kamel K; MatRahim N; Nawi MH; Suib FA; Suppiah J; Thayan R
EPI_ISL_12099656, EPI_ISL_12099666	Miri Hospital Molecular Diagnostic Lab, Miri	Institute of Health and Community Medicine	Chan Chia Jui; Chua Hock Hin; David Perera; Ooi Mong How; Tonnii Sia Loong Loong
EPI_ISL_11991765	National Public Health Laboratory	National Public Health Laboratory	Aimi Shazana Binti Shahir; Kamal Hisham Bin Kamarul Zaman; Muhammad Shahir Bin Ali; Noriah Binti Mohd Yusof; Nur Aina Fatini binti Azaman; Rehan Shuhada Binti Abu Bakar; Selvanesan A/L Sengol; Yu Kie A/P Chem
EPI_ISL_12510034, EPI_ISL_12510035, EPI_ISL_12510036, EPI_ISL_12510037, EPI_ISL_12510038, EPI_ISL_12510039, EPI_ISL_12510040, EPI_ISL_12510042			
see above	Normah Medical Specialist Centre, Kuching	Institute of Health and Community Medicine	Chan Chia Jui; Chua Hock Hin; David Perera; Ikwannuddin Y; Ooi Mong How; Tonnii Sia Loong Loong
EPI_ISL_12213443	PKD JOHOR BAHRU	UKM Medical Molecular Biology Institute (UMBI)	Khairun Nur Abd Ghafar; Mira Farzana Mohamad Mokhtar; Muhiddin Ishak; Nor Azila Muhammad Azami; Nur Alyaa Afifah Md Shahri; Nurul Syakima Ab Mutalib; Rahman Jamal; Ryia Illani Mohd Yunos; Siti Nur Hasanah Mohd Yusuf; Zahirrah Begam Mohamed Rasheed
EPI_ISL_12289516, EPI_ISL_12400592	QUEEN ELIZABETH HOSPITAL	Institute for Medical Research, Infectious Disease Research Centre, National Institutes of Health, Ministry of Health Malaysia	Ahmad FA; Ahmad Fazilah NA; Anasir MI; Azizan MA; Kamel K; Mohamad Sukri MZ; Norhisham SN; Ramly N; Robert F; Rosli NR; Suppiah J; Thayan R
EPI_ISL_12322025	RAJA PERMAISURI BAINUN HOSPITAL	Institute for Medical Research, Infectious Disease Research Centre, National Institutes of Health, Ministry of Health Malaysia	Ahmad FA; Ahmad Fazilah NA; Anasir MI; Azizan MA; Kamel K; Mohamad Sukri MZ; Norhisham SN; Ramly N; Robert F; Rosli NR; Suppiah J; Thayan R
EPI_ISL_12510024, EPI_ISL_12510025, EPI_ISL_12510026	Rejang Medical Centre (Sibu)	Institute of Health and Community Medicine	Chan Chia Jui; David Perera; Ooi Mong How; Wong Jyn Shan
EPI_ISL_12289512	SELAYANG HOSPITAL	Institute for Medical Research, Infectious Disease Research Centre, National Institutes of Health, Ministry of Health Malaysia	Ahmad FA; Ahmad Fazilah NA; Anasir MI; Azizan MA; Kamel K; Mohamad Sukri MZ; Norhisham SN; Ramly N; Robert F; Rosli NR; Suppiah J; Thayan R
EPI_ISL_12322064, EPI_ISL_12322065	SUNGAI BULOH HOSPITAL	Institute for Medical Research, Infectious Disease Research Centre, National Institutes of Health, Ministry of Health Malaysia	Ahmad FA; Ahmad Fazilah NA; Anasir MI; Azizan MA; Kamel K; Mohamad Sukri MZ; Norhisham SN; Ramly N; Robert F; Rosli NR; Suppiah J; Thayan R
EPI_ISL_12510169, EPI_ISL_12510170, EPI_ISL_12510171	Sarawak General Hospital (Kuching)	Institute of Health and Community Medicine	Chan Chia Jui; Chua Hock Hin; David Perera; Ooi Mong How
EPI_ISL_13026081	Synapse Sdn. Bhd.	Universiti Putra Malaysia (UPM)	Choo Yee Yu; Hui Jen Soe; Hui Yee Chee; Nancy Woan Charn Liew; Narcisse Joseph; Nurulfiza Mat Isa; Rachna Kairon; Sie Yeng Wong; Syafinaz Amin-Nordin; Zunita Zakaria
EPI_ISL_12055373	UNIT KESIHATAN AWAM	Institut Biologi Molekul Perubatan UKM (UMBI)	Khairun Nur Abd Ghafar; Mira Farzana Mohamad Mokhtar; Muhiddin Ishak; Nor Azila Muhammad Azami; Nur Alyaa Afifah Md Shahri; Nurul Syakima Ab Mutalib; Rahman Jamal; Ryia Illani Mohd Yunos; Siti Nur Hasanah Mohd Yusuf; Zahirrah Begam Mohamed Rasheed
EPI_ISL_12055317	Unit Kesihatan Awam	Institut Biologi Molekul Perubatan UKM (UMBI)	Khairun Nur Abd Ghafar; Mira Farzana Mohamad Mokhtar; Muhiddin Ishak; Nor Azila Muhammad Azami; Nur Alyaa Afifah Md Shahri; Nurul Syakima Ab Mutalib; Rahman Jamal; Ryia Illani Mohd Yunos; Siti Nur Hasanah Mohd Yusuf; Zahirrah Begam Mohamed Rasheed

We gratefully acknowledge the following Authors from the Originating laboratories responsible for obtaining the specimens, as well as the Submitting laboratories where the genome data were generated and shared via GISAID, on which this research is based.

All Submitters of data may be contacted directly via www.gisaid.org

Authors are sorted alphabetically.

Accession ID	Originating Laboratory	Submitting Laboratory	Authors
EPI_ISL_12322069	AMPANG HOSPITAL	Institute for Medical Research, Infectious Disease Research Centre, National Institutes of Health, Ministry of Health Malaysia	Ahmad FA; Ahmad Fazilah NA; Anasir MI; Azizan MA; Kamel K; Mohamad Sukri MZ; Norhisham SN; Ramly N; Robert F; Rosli NR; Suppiah J; Thayan R
EPI_ISL_12510271, EPI_ISL_12510272, EPI_ISL_12510273, EPI_ISL_12510274, EPI_ISL_12510275, EPI_ISL_12510276	Bintulu Medical Centre (Bintulu)	Institute of Health and Community Medicine	Chan Chia Jui; David Perera; Ooi Mong How; Wong Jyn Shan
EPI_ISL_12510136, EPI_ISL_12510137, EPI_ISL_12510138, EPI_ISL_12510139, EPI_ISL_12510140, EPI_ISL_12510141, EPI_ISL_12510143, EPI_ISL_12510144, EPI_ISL_12510145			
see above	Borneo Medical Centre (Kuching)	Institute of Health and Community Medicine	Chan Chia Jui; David Perera; Ooi Mong How; Wong Jyn Shan
EPI_ISL_12704537	Department of Medical Microbiology and Parasitology, Hospital Universiti Sains Malaysia	Molecular Research Laboratory	Abdul Haris bin Muhammad; Ahmad Sukari Bin Halim; Alexander Chong Shu Chien; Asraihan Che Abdul Malik; Azian Harun; Chan Yean Yean; Chua Wei Chuan; Farahana binti Mohamed; Kirnpal Kaur Banga Singh; Lau Nyok Sean; Lee Lih Huey; Lim Shu Yong; Maizun binti Mohd Zain; Mera Edora Binti Abdul Manap; Mohd Iman hafiz Bin Ibrahim; Mohd Nadzri Abu Yazid; Mohd Zulkifli Salleh; Muhamad Khairul Amirin Bin Zulkifli; Muhammad Azamuddeen bin Mohammad Nasir; Muhammad Fazli bin Khalid; Muhammad Nashrul Farhan Samsudin; Muhammad Zarul Hanifah Bin Md Zoqratt; Nik Zuraina binti Nik Mohd Noor; Noor Hafizan binti Mat Salleh; Nor Amizara Binti Azami; Nor Suhana Binti Mohd Satar; Nur Syuhada Binti Abdul Rahim; Nur-Leem Binti Murshid; Nurfadhlina Musa; Qasim Ayub; Rosline Hassan; Sadequr Rahman; Siti Nur Aisyah Binti Mohamad Sham; Syahida binti Omar; Wan Mohd Zahiruddin Wan Mohammad; Wardah Yusof; Zaini bin Hussin; Zakuan Zainy Deris
EPI_ISL_12534897, EPI_ISL_12534898	HOSPITAL SULTANAH MALIHA	Tropical Infectious Diseases Research & Education Centre (TIDREC), Universiti Malaya	AsmaAnati CheMatSeri; Che-Norainon Yaacob; Jia-Yi Tan; Jo-Ern Wong; Kim-Kee Tan; Mulya-Mustika-Sari Zulkifli; Noor Syahida Azizan; Nur-Hidayana Mahfodz; Sazaly AbuBakar; Siti-Sarah Nor'e; Wei-Wei Aw
EPI_ISL_12534772	HOSPITAL SULTANAH NUR ZAHIRAH	Tropical Infectious Diseases Research & Education Centre (TIDREC), Universiti Malaya	AsmaAnati CheMatSeri; Che-Norainon Yaacob; Jia-Yi Tan; Jo-Ern Wong; Kim-Kee Tan; Mulya-Mustika-Sari Zulkifli; Noor Syahida Azizan; Nur-Hidayana Mahfodz; Sazaly AbuBakar; Siti-Sarah Nor'e; Wei-Wei Aw
EPI_ISL_12213453	HSAJB	UKM Medical Molecular Biology Institute (UMBI)	Khairun Nur Abd Ghafar; Mira Farzana Mohamad Mokhtar; Muhiddin Ishak; Nor Azila Muhammad Azami; Nur Alyaa Afifah Md Shahri; Nurul Syakima Ab Mutalib; Rahman Jamal; Ryia Illani Mohd Yunos; Siti Nur Hasanah Mohd Yusuf; Zahirrah Begam Mohamed Rasheed
EPI_ISL_12704556, EPI_ISL_12704558	Hospital Raja Perempuan Zainab II, Kota Bharu	Molecular Research Laboratory	Abdul Haris bin Muhammad; Ahmad Sukari Bin Halim; Alexander Chong Shu Chien; Asraihan Che Abdul Malik; Azian Harun; Chan Yean Yean; Chua Wei Chuan; Farahana binti Mohamed; Kirnpal Kaur Banga Singh; Lau Nyok Sean; Lee Lih Huey; Lim Shu Yong; Maizun binti Mohd Zain; Mera Edora Binti Abdul Manap; Mohd Iman hafiz Bin Ibrahim; Mohd Nadzri Abu Yazid; Mohd Zulkifli Salleh; Muhamad Khairul Amirin Bin Zulkifli; Muhammad Azamuddeen bin Mohammad Nasir; Muhammad Fazli bin Khalid; Muhammad Nashrul Farhan Samsudin; Muhammad Zarul Hanifah Bin Md Zoqratt; Nik Zuraina binti Nik Mohd Noor; Noor Hafizan binti Mat Salleh; Nor Amizara Binti Azami; Nor Suhana Binti Mohd Satar; Nur Syuhada Binti Abdul Rahim; Nur-Leem Binti Murshid; Nurfadhlina Musa; Qasim Ayub; Rosline Hassan; Sadequr Rahman; Siti Nur Aisyah Binti Mohamad Sham; Syahida binti Omar; Wan Mohd Zahiruddin Wan Mohammad; Wardah Yusof; Zaini bin Hussin; Zakuan Zainy Deris
EPI_ISL_12771255	JKN PAHANG	iPROMISE, UiTM	Ariza Adnan; Fadzilah Mohd Nor; Lim Wai Feng; Mohd Asif Mohd Sukri; Mohd Nur Fakhruzzaman Noorizhab; Mohd Zaki Salleh; Sazzli Shahlan Kassim; Siti Farah Alwani Mohd Nawi; Siti Hamimah Sheikh Abdul Kadir; Teh Lay Kek; Wang Seok Mui
EPI_ISL_12746986	KULIM HOSPITAL	Institute for Medical Research, Infectious Disease Research Centre, National Institutes of Health, Ministry of Health Malaysia	Ahmad FA; Ahmad Fazilah NA; Anasir MI; Azizan MA; Kamel K; Mohamad Sukri MZ; Norhisham SN; Ramly N; Robert F; Rosli NR; Suppiah J; Thayan R
EPI_ISL_12708554	Kota Bharu Public Health Laboratory	Molecular Research Laboratory	Abdul Haris bin Muhammad; Ahmad Sukari Bin Halim; Alexander Chong Shu Chien; Asraihan Che Abdul Malik; Azian Harun; Chan Yean Yean; Chua Wei Chuan; Farahana binti Mohamed; Kirnpal Kaur Banga Singh; Lau Nyok Sean; Lee Lih Huey; Lim Shu Yong; Maizun binti Mohd Zain; Mera Edora Binti Abdul Manap; Mohd Iman hafiz Bin Ibrahim; Mohd Nadzri Abu Yazid; Mohd Zulkifli Salleh; Muhamad Khairul Amirin Bin Zulkifli; Muhammad Azamuddeen bin Mohammad Nasir; Muhammad Fazli bin Khalid; Muhammad Nashrul Farhan Samsudin; Muhammad Zarul Hanifah Bin Md Zoqratt; Nik Zuraina binti Nik Mohd Noor; Noor Hafizan binti Mat Salleh; Nor Amizara Binti Azami; Nor Suhana Binti Mohd Satar; Nur Syuhada Binti Abdul Rahim; Nur-Leem Binti Murshid; Nurfadhlina Musa; Qasim Ayub; Rosline Hassan; Sadequr Rahman; Siti Nur Aisyah Binti Mohamad Sham; Syahida binti Omar; Wan Mohd Zahiruddin Wan Mohammad; Wardah Yusof; Zaini bin Hussin; Zakuan Zainy Deris
EPI_ISL_11809397, EPI_ISL_11809398, EPI_ISL_11809399, EPI_ISL_11809400, EPI_ISL_11809401, EPI_ISL_11809402, EPI_ISL_11809403, EPI_ISL_11809404, EPI_ISL_11809405, EPI_ISL_11809406, EPI_ISL_11809407, EPI_ISL_11809408, EPI_ISL_11809409, EPI_ISL_11809410, EPI_ISL_11809411, EPI_ISL_11809412, EPI_ISL_11809413, EPI_ISL_11809414, EPI_ISL_11809415, EPI_ISL_11809416, EPI_ISL_11809417, EPI_ISL_11809418, EPI_ISL_11809419, EPI_ISL_11809420, EPI_ISL_11809421, EPI_ISL_11809422, EPI_ISL_11809423, EPI_ISL_11809424, EPI_ISL_11809425, EPI_ISL_11809426, EPI_ISL_11809427, EPI_ISL_11809428, EPI_ISL_11809429, EPI_ISL_11809430, EPI_ISL_11809431, EPI_ISL_11809432, EPI_ISL_11809433, EPI_ISL_11809434, EPI_ISL_11809435, EPI_ISL_11809436, EPI_ISL_11809437, EPI_ISL_11809438, EPI_ISL_11809439, EPI_ISL_11809440, EPI_ISL_11809441, EPI_ISL_11809442, EPI_ISL_11809443, EPI_ISL_11809444, EPI_ISL_11809445, EPI_ISL_11809446, EPI_ISL_11809447, EPI_ISL_11809448, EPI_ISL_11809449, EPI_ISL_11809450, EPI_ISL_11809451, EPI_ISL_11809452, EPI_ISL_11809453, EPI_ISL_11809454, EPI_ISL_11809455, EPI_ISL_11809456, EPI_ISL_11809457, EPI_ISL_11809458, EPI_ISL_11809459, EPI_ISL_11809460, EPI_ISL_11809461, EPI_ISL_11809462, EPI_ISL_11809463, EPI_ISL_11809464, EPI_ISL_11809465, EPI_ISL_11809466, EPI_ISL_11809467, EPI_ISL_11809468, EPI_ISL_11809469, EPI_ISL_11809470, EPI_ISL_11809471, EPI_ISL_11809472, EPI_ISL_11809473, EPI_ISL_11809474, EPI_ISL_11809475, EPI_ISL_11809476, EPI_ISL_11809477, EPI_ISL_11809478, EPI_ISL_11809479, EPI_ISL_11809480, EPI_ISL_11809481, EPI_ISL_11809482, EPI_ISL_11809483, EPI_ISL_11809484, EPI_ISL_11809485, EPI_ISL_11809486, EPI_ISL_11809487, EPI_ISL_11825603, EPI_ISL_11882042, EPI_ISL_11882043, EPI_ISL_11882044, EPI_ISL_11882045, EPI_ISL_11882046, EPI_ISL_11882047, EPI_ISL_11882048, EPI_ISL_11882049, EPI_ISL_11882050, EPI_ISL_11882051, EPI_ISL_11882052, EPI_ISL_11882053, EPI_ISL_11882054, EPI_ISL_11882055, EPI_ISL_11882056, EPI_ISL_11882057, EPI_ISL_11882058, EPI_ISL_11882059, EPI_ISL_11882060, EPI_ISL_11882061, EPI_ISL_11882062, EPI_ISL_11882063, EPI_ISL_11882064, EPI_ISL_11882065, EPI_ISL_11882066, EPI_ISL_11882067, EPI_ISL_11882068, EPI_ISL_11882069, EPI_ISL_11882070, EPI_ISL_11882071, EPI_ISL_11882072, EPI_ISL_11882073, EPI_ISL_11882074, EPI_ISL_11882075, EPI_ISL_11882076, EPI_ISL_11882077, EPI_ISL_11882078, EPI_ISL_11882079, EPI_ISL_11882080, EPI_ISL_11882081, EPI_ISL_11882082, EPI_ISL_11882083, EPI_ISL_11882084, EPI_ISL_11882085, EPI_ISL_11882086, EPI_ISL_11882087, EPI_ISL_11882088, EPI_ISL_11882089, EPI_ISL_11882090, EPI_ISL_11882091, EPI_ISL_11882092, EPI_ISL_11882093, EPI_ISL_11882095, EPI_ISL_11882096, EPI_ISL_11882097, EPI_ISL_11882098, EPI_ISL_11882099, EPI_ISL_11882100, EPI_ISL_11882101, EPI_ISL_11882102, EPI_ISL_11882103, EPI_ISL_11882104, EPI_ISL_11882105, EPI_ISL_11882106, EPI_ISL_11882107, EPI_ISL_11882108, EPI_ISL_11882109, EPI_ISL_11882110, EPI_ISL_11882111, EPI_ISL_11882112, EPI_ISL_11882113, EPI_ISL_11882114, EPI_ISL_11882115, EPI_ISL_11882116, EPI_ISL_11882117, EPI_ISL_11882118, EPI_ISL_11882119, EPI_ISL_11882120, EPI_ISL_11882121, EPI_ISL_11882122, EPI_ISL_11882123, EPI_ISL_11882124, EPI_ISL_11882125, EPI_ISL_11882126, EPI_ISL_11882127, EPI_ISL_11882128, EPI_ISL_11882129, EPI_ISL_11882130, EPI_ISL_11882131, EPI_ISL_11882132, EPI_ISL_11882133, EPI_ISL_11882134, EPI_ISL_11882135, EPI_ISL_11900499, EPI_ISL_11900500, EPI_ISL_11900501, EPI_ISL_12008378, EPI_ISL_12008379, EPI_ISL_12008380, EPI_ISL_12008381, EPI_ISL_12008382, EPI_ISL_12008383, EPI_ISL_12008384, EPI_ISL_12008385, EPI_ISL_12008386, EPI_ISL_12008387, EPI_ISL_12008388, EPI_ISL_12008389, EPI_ISL_12008390, EPI_ISL_12008391, EPI_ISL_12008392, EPI_ISL_12008393, EPI_ISL_12008394, EPI_ISL_12008395, EPI_ISL_12008396, EPI_ISL_12008397, EPI_ISL_12008398, EPI_ISL_12008399, EPI_ISL_12008400, EPI_ISL_12008401, EPI_ISL_12008409, EPI_ISL_12008410, EPI_ISL_12008411, EPI_ISL_12008412, EPI_ISL_12008413, EPI_ISL_12008414, EPI_ISL_12008415, EPI_ISL_12008416, EPI_ISL_12008417, EPI_ISL_12008418, EPI_ISL_12008419, EPI_ISL_12008420, EPI_ISL_12008421, EPI_ISL_12008422, EPI_ISL_12008423, EPI_ISL_12008424, EPI_ISL_12061277, EPI_ISL_12061278, EPI_ISL_12061279, EPI_ISL_12061280, EPI_ISL_12061281, EPI_ISL_12061282, EPI_ISL_12061283, EPI_ISL_12061284, EPI_ISL_12061285, EPI_ISL_12061286, EPI_ISL_12061287, EPI_ISL_12061288, EPI_ISL_12061289, EPI_ISL_12061290, EPI_ISL_12061291, EPI_ISL_12061292, EPI_ISL_12061293, EPI_ISL_12061294, EPI_ISL_12061295, EPI_ISL_12061296, EPI_ISL_12061297, EPI_ISL_12061298, EPI_ISL_12061299, EPI_ISL_12061300, EPI_ISL_12061301, EPI_ISL_12061302, EPI_ISL_12061303, EPI_ISL_12061304, EPI_ISL_12061305, EPI_ISL_12061306, EPI_ISL_12061307, EPI_ISL_12061308, EPI_ISL_12061309, EPI_ISL_12061310, EPI_ISL_12061311, EPI_ISL_12061312, EPI_ISL_12061313, EPI_ISL_12061314, EPI_ISL_12061315, EPI_ISL_12061316, EPI_ISL_12061317, EPI_ISL_12061318, EPI_ISL_12061319, EPI_ISL_12061320, EPI_ISL_12061321, EPI_ISL_12061322, EPI_ISL_12061323, EPI_ISL_12061324, EPI_ISL_12061325, EPI_ISL_12061326, EPI_ISL_12061327, EPI_ISL_12061328, EPI_ISL_12061329, EPI_ISL_12061330, EPI_ISL_12061331, EPI_ISL_12061332, EPI_ISL_12061333, EPI_ISL_12061334, EPI_ISL_12061335, EPI_ISL_12061336, EPI_ISL_12061337, EPI_ISL_12061338, EPI_ISL_12061358, EPI_ISL_12061359, EPI_ISL_12061360, EPI_ISL_12061361, EPI_ISL_12061362, EPI_ISL_12061363, EPI_ISL_12061364, EPI_ISL_12061365, EPI_ISL_12061366, EPI_ISL_12061367, EPI_ISL_12061368, EPI_ISL_12085945, EPI_ISL_12085946, EPI_ISL_12085947, EPI_ISL_12085948, EPI_ISL_12085949, EPI_ISL_12085950, EPI_ISL_12085951, EPI_ISL_12085952, EPI_ISL_12085953, EPI_ISL_12085954, EPI_ISL_12085955, EPI_ISL_12085956, EPI_ISL_12085957, EPI_ISL_12085958, EPI_ISL_12085959			
see above	Kuala Lumpur International Airport	Institute for Medical Research, Infectious Disease Research Centre, National Institutes of Health, Ministry of Health Malaysia	Anasir MI; G.Adypatti NM; Jamaluddin MS; Kalyanasundram J; Kamel K; MatRahim N; Nawi MH; Suib FA; Suppiah J; Thayan R
EPI_ISL_12213438, EPI_ISL_12213439	MAKMAL KESIHATAN AWAM JOHOR BAHRU	UKM Medical Molecular Biology Institute (UMBI)	Khairun Nur Abd Ghafar; Mira Farzana Mohamad Mokhtar; Muhiddin Ishak; Nor Azila Muhammad Azami; Nur Alyaa Afifah Md Shahri; Nurul Syakima Ab Mutalib; Rahman Jamal; Ryia Illani Mohd Yunos; Siti Nur Hasanah Mohd Yusuf; Zahirrah Begam Mohamed Rasheed
EPI_ISL_12099652, EPI_ISL_12099657	Miri Hospital Molecular Diagnostic Lab, Miri	Institute of Health and Community Medicine	Chan Chia Jui; Chua Hock Hin; David Perera; Ooi Mong How; Tonnii Sia Loong Loong
EPI_ISL_12289518, EPI_ISL_12289519, EPI_ISL_12289520, EPI_ISL_12289521, EPI_ISL_12289522, EPI_ISL_12289523, EPI_ISL_12289524, EPI_ISL_12289525, EPI_ISL_12289526, EPI_ISL_12289527, EPI_ISL_12289528, EPI_ISL_12289529, EPI_ISL_12289530, EPI_ISL_12289531, EPI_ISL_12289532, EPI_ISL_12289533, EPI_ISL_12289534, EPI_ISL_12289535, EPI_ISL_12665248, EPI_ISL_12665249, EPI_ISL_12665277			
see above	NATIONAL PUBLIC HEALTH LABORATORY	Institute for Medical Research, Infectious Disease Research Centre, National Institutes of Health, Ministry of Health Malaysia	Ahmad FA; Ahmad Fazilah NA; Anasir MI; Azizan MA; Kamel K; Mohamad Sukri MZ; Norhisham SN; Ramly N; Robert F; Rosli NR; Suppiah J; Thayan R
EPI_ISL_12899698	NATIONAL PUBLIC HEALTH LABORATORY (MKAK)	Institute for Medical Research, Infectious Disease Research Centre, National Institutes of Health, Ministry of Health Malaysia	Ahmad FA; Ahmad Fazilah NA; Anasir MI; Azizan MA; Kamel K; Mohamad Sukri MZ; Norhisham SN; Ramly N; Robert F; Rosli NR; Suppiah J; Thayan R
EPI_ISL_11991766, EPI_ISL_12099751, EPI_ISL_12099752, EPI_ISL_12099753, EPI_ISL_12099754, EPI_ISL_12099755, EPI_ISL_12099762, EPI_ISL_12099763			
see above	National Public Health Laboratory	National Public Health Laboratory	Aimi Shazana Binti Shahir; Kamal Hisham Bin Kamarul Zaman; Lim Cheau Ju; Muhammad Shahir Bin Ali; Noriah Binti Mohd Yusof; Nur Aina Fatini binti Azaman; Rehan Shuhada Binti Abu Bakar; Selvanesan A/L Sengol; Syahida Omar; Yu Kie A/P Chem
EPI_ISL_12084315, EPI_ISL_12084316	National Public Health Laboratory (MKAK), Ministry of Health Malaysia	Institute for Medical Research, Infectious Disease Research Centre, National Institutes of Health, Ministry of Health Malaysia	Ahmad FA; Ahmad Fazilah NA; Anasir MI; Azizan MA; G. Adypatti NM; Jamaluddin MS; Kamel K; Mohamad Sukri MZ; Norhisham SN; Ramly N; Robert F; Rosli NR; Suppiah J; Thayan R
EPI_ISL_12510043, EPI_ISL_12510044	Normah Medical Specialist Centre, Kuching	Institute of Health and Community Medicine	Chan Chia Jui; Chua Hock Hin; David Perera; Ikwannuddin Y; Ooi Mong How; Tonnii Sia Loong Loong
EPI_ISL_12213441, EPI_ISL_12213444	PKD JOHOR BAHRU	UKM Medical Molecular Biology Institute (UMBI)	Khairun Nur Abd Ghafar; Mira Farzana Mohamad Mokhtar; Muhiddin Ishak; Nor Azila Muhammad Azami; Nur Alyaa Afifah Md Shahri; Nurul Syakima Ab Mutalib; Rahman Jamal; Ryia Illani Mohd Yunos; Siti Nur Hasanah Mohd Yusuf; Zahirrah Begam Mohamed Rasheed
EPI_ISL_12628126, EPI_ISL_12628127, EPI_ISL_12628128	PKD MUAR	UKM Medical Molecular Biology Institute (UMBI)	Khairun Nur Abd Ghafar; Mira Farzana Mohamad Mokhtar; Muhiddin Ishak; Nor Azila Muhammad Azami; Nur Alyaa Afifah Md Shahri; Nurul Syakima Ab Mutalib; Rahman Jamal; Ryia Illani Mohd Yunos; Siti Nur Hasanah Mohd Yusuf; Zahirrah Begam Mohamed Rasheed
EPI_ISL_12321870	QUEEN ELIZABETH HOSPITAL	Institute for Medical Research, Infectious Disease Research Centre,	Ahmad FA; Ahmad Fazilah NA; Anasir MI; Azizan MA; Kamel K; Mohamad Sukri MZ; Norhisham SN; Ramly N; Robert F; Rosli NR; Suppiah J; Thayan R

		National Institutes of Health, Ministry of Health Malaysia	
EPI_ISL_12510027, EPI_ISL_12510028, EPI_ISL_12510029, EPI_ISL_12510030, EPI_ISL_12510031, EPI_ISL_12510032, EPI_ISL_12510033			
see above	Rejang Medical Centre (Sibu)	Institute of Health and Community Medicine	Chan Chia Jui; David Perera; Ooi Mong How; Wong Jyn Shan
EPI_ISL_12289483, EPI_ISL_12899703	SULTANAH BAHIYAH HOSPITAL	Institute for Medical Research, Infectious Disease Research Centre, National Institutes of Health, Ministry of Health Malaysia	Ahmad FA; Ahmad Fazilah NA; Anasir MI; Azizan MA; Kamel K; Mohamad Sukri MZ; Norhisham SN; Ramly N; Robert F; Rosli NR; Suppiah J; Thayan R
EPI_ISL_12322066	SUNGAI BULOH HOSPITAL	Institute for Medical Research, Infectious Disease Research Centre, National Institutes of Health, Ministry of Health Malaysia	Ahmad FA; Ahmad Fazilah NA; Anasir MI; Azizan MA; Kamel K; Mohamad Sukri MZ; Norhisham SN; Ramly N; Robert F; Rosli NR; Suppiah J; Thayan R
EPI_ISL_12099684, EPI_ISL_12099685, EPI_ISL_12099686	Sibu Hospital, PCR lab (Sibu, Sarawak)	Institute of Health and Community Medicine	Chan Chia Jui; Chua Hock Hin; David Perera; Ooi Mong How; Tonnii Sia Loong Loong
EPI_ISL_13026082, EPI_ISL_13026083, EPI_ISL_13026084	Synapse Sdn. Bhd.	Universiti Putra Malaysia (UPM)	Choo Yee Yu; Hui Jen Soe; Hui Yee Chee; Nancy Woan Charn Liew; Narcisse Joseph; Nurulfiza Mat Isa; Rachna Kairon; Sie Yeng Wong; Syafinaz Amin-Nordin; Zunita Zakaria
EPI_ISL_12289550	TAWAU HOSPITAL	Institute for Medical Research, Infectious Disease Research Centre, National Institutes of Health, Ministry of Health Malaysia	Ahmad FA; Ahmad Fazilah NA; Anasir MI; Azizan MA; Kamel K; Mohamad Sukri MZ; Norhisham SN; Ramly N; Robert F; Rosli NR; Suppiah J; Thayan R
EPI_ISL_12560256, EPI_ISL_12560260	UMMC	Department of Medical Microbiology, Faculty of Medicine, University of Malaya; University of Malaya Medical Centre	I-Ching SAM; Jolene Yin Ling FU; Omar Khalilur Rahman; Yee Von Lee; Yoke Fun Chan

We gratefully acknowledge the following Authors from the Originating laboratories responsible for obtaining the specimens, as well as the Submitting laboratories where the genome data were generated and shared via GISAID, on which this research is based.

All Submitters of data may be contacted directly via www.gisaid.org

Authors are sorted alphabetically.

Acknowledgement EPI_SET Identifier: EPI_SET_20220603va

Accession ID	Originating Laboratory	Submitting Laboratory	Authors
EPI_ISL_12289513, EPI_ISL_12322067, EPI_ISL_12322068, EPI_ISL_12322070, EPI_ISL_12322071, EPI_ISL_12322075, EPI_ISL_12322077, EPI_ISL_12322078, EPI_ISL_12899666			
see above	AMPANG HOSPITAL	Institute for Medical Research, Infectious Disease Research Centre, National Institutes of Health, Ministry of Health Malaysia	Ahmad FA; Ahmad Fazilah NA; Anasir MI; Azizan MA; Kamel K; Mohamad Sukri MZ; Norhisham SN; Ramly N; Robert F; Rosli NR; Suppiah J; Thayan R
EPI_ISL_12099623, EPI_ISL_12099624, EPI_ISL_12099625, EPI_ISL_12509993, EPI_ISL_12510277, EPI_ISL_12510278, EPI_ISL_12510279			
see above	Bintulu Medical Centre (Bintulu)	Institute of Health and Community Medicine	Chan Chia Jui; Chua Hock Hin; David Perera; Ooi Mong How; Tonnii Sia Loong Loong; Wong Jyn Shan
EPI_ISL_12510146, EPI_ISL_12510147, EPI_ISL_12510148, EPI_ISL_12510149	Borneo Medical Centre (Kuching)	Institute of Health and Community Medicine	Chan Chia Jui; David Perera; Ooi Mong How; Wong Jyn Shan
EPI_ISL_12534925	FORENSIC HOSPITAL SULTANAH BAHIYAH	Tropical Infectious Diseases Research & Education Centre (TIDREC), Universiti Malaya	AsmaAnati CheMatSeri; Che-Norainon Yaacob; Jia-Yi Tan; Jo-Ern Wong; Kim-Kee Tan; Mulya-Mustika-Sari Zulkifli; Noor Syahida Azizan; Nur-Hidayana Mahfodz; Sazaly AbuBakar; Siti-Sarah Nor'e; Wei-Wei Aw
EPI_ISL_12628101	HOSPITAL MELAKA	UKM Medical Molecular Biology Institute (UMBI)	Khairun Nur Abd Ghafar; Mira Farzana Mohamad Mokhtar; Muhiddin Ishak; Nor Azila Muhammad Azami; Nur Alyaa Afifah Md Shahri; Nurul Syakima Ab Mutalib; Rahman Jamal; Ryia Illani Mohd Yunos; Siti Nur Hasanah Mohd Yusuf; Zahirrah Begam Mohamed Rasheed
EPI_ISL_12771245, EPI_ISL_12771246	HOSPITAL PULAU PINANG	iPROMISE, UiTM	Ariza Adnan; Fadzilah Mohd Nor; Lim Wai Feng; Mohd Asif Mohd Sukri; Mohd Nur Fakhruzzaman Noorizhab; Mohd Zaki Salleh; Sazzli Shahlan Kassim; Siti Farah Alwani Mohd Nawi; Siti Hamimah Sheikh Abdul Kadir; Teh Lay Kek; Wang Seok Mui
EPI_ISL_12213456	HSAJB	UKM Medical Molecular Biology Institute (UMBI)	Khairun Nur Abd Ghafar; Mira Farzana Mohamad Mokhtar; Muhiddin Ishak; Nor Azila Muhammad Azami; Nur Alyaa Afifah Md Shahri; Nurul Syakima Ab Mutalib; Rahman Jamal; Ryia Illani Mohd Yunos; Siti Nur Hasanah Mohd Yusuf; Zahirrah Begam Mohamed Rasheed
EPI_ISL_12704559, EPI_ISL_12704563	Hospital Raja Perempuan Zainab II, Kota Bharu	Molecular Research Laboratory	Abdul Haris bin Muhammad; Ahmad Sukari Bin Halim; Alexander Chong Shu Chien; Asraihan Che Abdul Malik; Azian Harun; Chan Yean Yean; Chua Wei Chuan; Farahana binti Mohamed; Kirnpal Kaur Banga Singh; Lau Nyok Sean; Lee Lih Huey; Lim Shu Yong; Maizun binti Mohd Zain; Mera Edora Binti Abdul Manap; Mohd Iman hafiz Bin Ibrahim; Mohd Nadzri Abu Yazid; Mohd Zulkifli Salleh; Muhamad Khairul Amirin Bin Zulkifli; Muhammad Azamuddeen bin Mohammad Nasir; Muhammad Fazli bin Khalid; Muhammad Nashrul Farhan Samsudin; Muhammad Zarul Hanifah Bin Md Zoqratt; Nik Zuraina binti Nik Mohd Noor; Noor Hafizan binti Mat Salleh; Nor Amizara Binti Azami; Nor Suhana Binti Mohd Satar; Nur Syuhada Binti Abdul Rahim; Nur-Leem Binti Murshid; Nurfadhlina Musa; Qasim Ayub; Rosline Hassan; Sadequr Rahman; Siti Nur Aisyah Binti Mohamad Sham; Syahida binti Omar; Wan Mohd Zahiruddin Wan Mohammad; Wardah Yusof; Zaini bin Hussin; Zakuan Zainy Deris
EPI_ISL_12213445	Hospital melaka	UKM Medical Molecular Biology Institute (UMBI)	Khairun Nur Abd Ghafar; Mira Farzana Mohamad Mokhtar; Muhiddin Ishak; Nor Azila Muhammad Azami; Nur Alyaa Afifah Md Shahri; Nurul Syakima Ab Mutalib; Rahman Jamal; Ryia Illani Mohd Yunos; Siti Nur Hasanah Mohd Yusuf; Zahirrah Begam Mohamed Rasheed
EPI_ISL_12771314, EPI_ISL_12771315	JKN PAHANG	iPROMISE, UiTM	Ariza Adnan; Fadzilah Mohd Nor; Lim Wai Feng; Mohd Asif Mohd Sukri; Mohd Nur Fakhruzzaman Noorizhab; Mohd Zaki Salleh; Sazzli Shahlan Kassim; Siti Farah Alwani Mohd Nawi; Siti Hamimah Sheikh Abdul Kadir; Teh Lay Kek; Wang Seok Mui
EPI_ISL_12708555, EPI_ISL_12708556	Kota Bharu Public Health Laboratory	Molecular Research Laboratory	Abdul Haris bin Muhammad; Ahmad Sukari Bin Halim; Alexander Chong Shu Chien; Asraihan Che Abdul Malik; Azian Harun; Chan Yean Yean; Chua Wei Chuan; Farahana binti Mohamed; Kirnpal Kaur Banga Singh; Lau Nyok Sean; Lee Lih Huey; Lim Shu Yong; Maizun binti Mohd Zain; Mera Edora Binti Abdul Manap; Mohd Iman hafiz Bin Ibrahim; Mohd Nadzri Abu Yazid; Mohd Zulkifli Salleh; Muhamad Khairul Amirin Bin Zulkifli; Muhammad Azamuddeen bin Mohammad Nasir; Muhammad Fazli bin Khalid; Muhammad Nashrul Farhan Samsudin; Muhammad Zarul Hanifah Bin Md Zoqratt; Nik Zuraina binti Nik Mohd Noor; Noor Hafizan binti Mat Salleh; Nor Amizara Binti Azami; Nor Suhana Binti Mohd Satar; Nur Syuhada Binti Abdul Rahim; Nur-Leem Binti Murshid; Nurfadhlina Musa; Qasim Ayub; Rosline Hassan; Sadequr Rahman; Siti Nur Aisyah Binti Mohamad Sham; Syahida binti Omar; Wan Mohd Zahiruddin Wan Mohammad; Wardah Yusof; Zaini bin Hussin; Zakuan Zainy Deris
EPI_ISL_11900502, EPI_ISL_11902800, EPI_ISL_11902801, EPI_ISL_11902802, EPI_ISL_11902803, EPI_ISL_11902804, EPI_ISL_11902805, EPI_ISL_11902806, EPI_ISL_11902807, EPI_ISL_11902808, EPI_ISL_11902809, EPI_ISL_11902810, EPI_ISL_11902811, EPI_ISL_11902812, EPI_ISL_11902813, EPI_ISL_11902814, EPI_ISL_11902815, EPI_ISL_11902816, EPI_ISL_11902817, EPI_ISL_11902818, EPI_ISL_11902819, EPI_ISL_11902820, EPI_ISL_11902821, EPI_ISL_11902822, EPI_ISL_11902823, EPI_ISL_11902824, EPI_ISL_11902825, EPI_ISL_11902826, EPI_ISL_11902827, EPI_ISL_11902828, EPI_ISL_11902829, EPI_ISL_11902830, EPI_ISL_11902831, EPI_ISL_11902832, EPI_ISL_11902833, EPI_ISL_11902834, EPI_ISL_11902835, EPI_ISL_11902836, EPI_ISL_11902837, EPI_ISL_11902838, EPI_ISL_11902839, EPI_ISL_11902840, EPI_ISL_11902841, EPI_ISL_11902842, EPI_ISL_11902843, EPI_ISL_11902844, EPI_ISL_11902845, EPI_ISL_11902846, EPI_ISL_11902847, EPI_ISL_11902848, EPI_ISL_11902849, EPI_ISL_11902850, EPI_ISL_11902851, EPI_ISL_11902852, EPI_ISL_11902853, EPI_ISL_11902854, EPI_ISL_11902855, EPI_ISL_11902856, EPI_ISL_11902857, EPI_ISL_11902858, EPI_ISL_11902859, EPI_ISL_11902860, EPI_ISL_11902861, EPI_ISL_11902862, EPI_ISL_11902863, EPI_ISL_11902864, EPI_ISL_11902865, EPI_ISL_11902866, EPI_ISL_11902867, EPI_ISL_11902868, EPI_ISL_11902869, EPI_ISL_11902870, EPI_ISL_11902871, EPI_ISL_11902872, EPI_ISL_11902873, EPI_ISL_11902874, EPI_ISL_11902875, EPI_ISL_11902876, EPI_ISL_11902877, EPI_ISL_11902878, EPI_ISL_11902879, EPI_ISL_11902880, EPI_ISL_11902881, EPI_ISL_11902882, EPI_ISL_11902883, EPI_ISL_11902884, EPI_ISL_11902885, EPI_ISL_11902886, EPI_ISL_11902887, EPI_ISL_11902888, EPI_ISL_11902889, EPI_ISL_11902890, EPI_ISL_11902891, EPI_ISL_11902892, EPI_ISL_11902893, EPI_ISL_11902894, EPI_ISL_11902895, EPI_ISL_11902896, EPI_ISL_11902897, EPI_ISL_11902898, EPI_ISL_11902899, EPI_ISL_11902900, EPI_ISL_11902901, EPI_ISL_11902902, EPI_ISL_11902903, EPI_ISL_11902904, EPI_ISL_11902905, EPI_ISL_11902906, EPI_ISL_11902907, EPI_ISL_11902908, EPI_ISL_11902909, EPI_ISL_11902910, EPI_ISL_11902911, EPI_ISL_11902912, EPI_ISL_11902913, EPI_ISL_11902914, EPI_ISL_11902915, EPI_ISL_11902916, EPI_ISL_11902917, EPI_ISL_11902918, EPI_ISL_11902919, EPI_ISL_11902920, EPI_ISL_11902921, EPI_ISL_11902922, EPI_ISL_11902923, EPI_ISL_11902924, EPI_ISL_11902925, EPI_ISL_11902926, EPI_ISL_11902927, EPI_ISL_11902928, EPI_ISL_11902929, EPI_ISL_11902930, EPI_ISL_11902931, EPI_ISL_11902932, EPI_ISL_11902933, EPI_ISL_11902934, EPI_ISL_11902935, EPI_ISL_11902936, EPI_ISL_11902937, EPI_ISL_11902938, EPI_ISL_11902939, EPI_ISL_11902940, EPI_ISL_11902941, EPI_ISL_11902942, EPI_ISL_11902943, EPI_ISL_11902944, EPI_ISL_11902945, EPI_ISL_11902946, EPI_ISL_11902947, EPI_ISL_11902948, EPI_ISL_11902949, EPI_ISL_11902950, EPI_ISL_11902951, EPI_ISL_11902952, EPI_ISL_11902953, EPI_ISL_11903066, EPI_ISL_11903067, EPI_ISL_11903068, EPI_ISL_11903069, EPI_ISL_11903070, EPI_ISL_11903071, EPI_ISL_11903072, EPI_ISL_11903073, EPI_ISL_11903074, EPI_ISL_11903075, EPI_ISL_11903076, EPI_ISL_11903077, EPI_ISL_11903078, EPI_ISL_11903079, EPI_ISL_11903080, EPI_ISL_11903081, EPI_ISL_11903082, EPI_ISL_11903083, EPI_ISL_11903084, EPI_ISL_11903085, EPI_ISL_11903086, EPI_ISL_11903087, EPI_ISL_11903088, EPI_ISL_11903089, EPI_ISL_11903090, EPI_ISL_11903091, EPI_ISL_11903092, EPI_ISL_11903093, EPI_ISL_11903094, EPI_ISL_11903095, EPI_ISL_11903096, EPI_ISL_11903097, EPI_ISL_11929490, EPI_ISL_11929491, EPI_ISL_11929492, EPI_ISL_11929493, EPI_ISL_11929494, EPI_ISL_11929495, EPI_ISL_11929496, EPI_ISL_11929497, EPI_ISL_11929498, EPI_ISL_11929499, EPI_ISL_11929500, EPI_ISL_11929501, EPI_ISL_11929502, EPI_ISL_11929503, EPI_ISL_11929504, EPI_ISL_11929505, EPI_ISL_11929506, EPI_ISL_11929507, EPI_ISL_11929508, EPI_ISL_11929509, EPI_ISL_11929510, EPI_ISL_11929511, EPI_ISL_11929512, EPI_ISL_11929513, EPI_ISL_11929514, EPI_ISL_11929515, EPI_ISL_11929516, EPI_ISL_11929517, EPI_ISL_11929518, EPI_ISL_11929519, EPI_ISL_11929520, EPI_ISL_11929521, EPI_ISL_11929522, EPI_ISL_11929523, EPI_ISL_11929524, EPI_ISL_11929525, EPI_ISL_11929526, EPI_ISL_11929527, EPI_ISL_11929528, EPI_ISL_11929529, EPI_ISL_11929530, EPI_ISL_11929531, EPI_ISL_11929532, EPI_ISL_11929533, EPI_ISL_11929534, EPI_ISL_11929535, EPI_ISL_11929536, EPI_ISL_11929537, EPI_ISL_11929538, EPI_ISL_11929539, EPI_ISL_11929540, EPI_ISL_11929541, EPI_ISL_11929542, EPI_ISL_11929543, EPI_ISL_11929544, EPI_ISL_11929545, EPI_ISL_11929546, EPI_ISL_11929547, EPI_ISL_11929548, EPI_ISL_11929549, EPI_ISL_11929550, EPI_ISL_11929551, EPI_ISL_11929552, EPI_ISL_11929553, EPI_ISL_11929554, EPI_ISL_11929555, EPI_ISL_11929556, EPI_ISL_11929557, EPI_ISL_11929558, EPI_ISL_11929559, EPI_ISL_11929560, EPI_ISL_11929561, EPI_ISL_11929562, EPI_ISL_11929563, EPI_ISL_11929564, EPI_ISL_11929565, EPI_ISL_11929566, EPI_ISL_11929567, EPI_ISL_11929568, EPI_ISL_11929569, EPI_ISL_11929570, EPI_ISL_11929571, EPI_ISL_11929572, EPI_ISL_11929573, EPI_ISL_11929574, EPI_ISL_11929575, EPI_ISL_11929576, EPI_ISL_11929577, EPI_ISL_11929578, EPI_ISL_11929579, EPI_ISL_11929580, EPI_ISL_11929581, EPI_ISL_11929582, EPI_ISL_11929583, EPI_ISL_11929584, EPI_ISL_11929585, EPI_ISL_12061339, EPI_ISL_12061340, EPI_ISL_12061341, EPI_ISL_12061342, EPI_ISL_12061343, EPI_ISL_12061344, EPI_ISL_12061345, EPI_ISL_12061346, EPI_ISL_12061347, EPI_ISL_12061348, EPI_ISL_12061349, EPI_ISL_12061350, EPI_ISL_12061351, EPI_ISL_12061352, EPI_ISL_12061353, EPI_ISL_12061354, EPI_ISL_12061355, EPI_ISL_12061356, EPI_ISL_12061357, EPI_ISL_12085960, EPI_ISL_12085961, EPI_ISL_12085962, EPI_ISL_12085963, EPI_ISL_12085964, EPI_ISL_12085965, EPI_ISL_12085966, EPI_ISL_12085967, EPI_ISL_12085968, EPI_ISL_12085969, EPI_ISL_12085970, EPI_ISL_12085971, EPI_ISL_12085972, EPI_ISL_12085973, EPI_ISL_12085974, EPI_ISL_12085975, EPI_ISL_12085976, EPI_ISL_12085977, EPI_ISL_12085978, EPI_ISL_12085979, EPI_ISL_12085980, EPI_ISL_12085981, EPI_ISL_12085982, EPI_ISL_12085983, EPI_ISL_12085984, EPI_ISL_12085985, EPI_ISL_12085986, EPI_ISL_12085987, EPI_ISL_12085988, EPI_ISL_12085989, EPI_ISL_12085990, EPI_ISL_12085991, EPI_ISL_12085992, EPI_ISL_12085993, EPI_ISL_12085994, EPI_ISL_12085995, EPI_ISL_12085996, EPI_ISL_12085997, EPI_ISL_12085998, EPI_ISL_12085999, EPI_ISL_12086000, EPI_ISL_12086001, EPI_ISL_12086002, EPI_ISL_12086003, EPI_ISL_12086004, EPI_ISL_12086005, EPI_ISL_12086006, EPI_ISL_12086007, EPI_ISL_12086008, EPI_ISL_12086009, EPI_ISL_12086010, EPI_ISL_12086011, EPI_ISL_12086012, EPI_ISL_12086013, EPI_ISL_12086014, EPI_ISL_12086015, EPI_ISL_12086016, EPI_ISL_12086017, EPI_ISL_12086018, EPI_ISL_12086019, EPI_ISL_12086020, EPI_ISL_12086021, EPI_ISL_12086022, EPI_ISL_12086023, EPI_ISL_12086024, EPI_ISL_12086025, EPI_ISL_12086026, EPI_ISL_12086027, EPI_ISL_12086028, EPI_ISL_12086029, EPI_ISL_12086030, EPI_ISL_12086031, EPI_ISL_12086032, EPI_ISL_12086033, EPI_ISL_12086034, EPI_ISL_12086035, EPI_ISL_12148233, EPI_ISL_12148234, EPI_ISL_12148235, EPI_ISL_12148236, EPI_ISL_12148237, EPI_ISL_12148238, EPI_ISL_12148239, EPI_ISL_12148240, EPI_ISL_12148241, EPI_ISL_12148242, EPI_ISL_12148243, EPI_ISL_12148244			
see above	Kuala Lumpur International Airport	Institute for Medical Research, Infectious Disease Research Centre, National Institutes of Health, Ministry of Health Malaysia	Anasir MI; G.Adypatti NM; Jamaluddin MS; Kalyanasundram J; Kamel K; MatRahim N; Nawi MH; Suib FA; Suppiah J; Thayan R
EPI_ISL_12213440	MAKMAL KESIHATAN AWAM JOHOR BAHRU	UKM Medical Molecular Biology Institute (UMBI)	Khairun Nur Abd Ghafar; Mira Farzana Mohamad Mokhtar; Muhiddin Ishak; Nor Azila Muhammad Azami; Nur Alyaa Afifah Md Shahri; Nurul Syakima Ab Mutalib; Rahman Jamal; Ryia Illani Mohd Yunos; Siti Nur Hasanah Mohd Yusuf; Zahirrah Begam Mohamed Rasheed
EPI_ISL_12534894, EPI_ISL_12534895	MAKMAL SEROLOGI, UNIT MIKROBIOLOGI, HSB	Tropical Infectious Diseases Research & Education Centre (TIDREC), Universiti Malaya	AsmaAnati CheMatSeri; Che-Norainon Yaacob; Jia-Yi Tan; Jo-Ern Wong; Kim-Kee Tan; Mulya-Mustika-Sari Zulkifli; Noor Syahida Azizan; Nur-Hidayana Mahfodz; Sazaly AbuBakar; Siti-Sarah Nor'e; Wei-Wei Aw
EPI_ISL_12099653	Miri Hospital Molecular Diagnostic Lab, Miri	Institute of Health and Community Medicine	Chan Chia Jui; Chua Hock Hin; David Perera; Ooi Mong How; Tonnii Sia Loong Loong
EPI_ISL_12289536, EPI_ISL_12289537, EPI_ISL_12289538, EPI_ISL_12289539, EPI_ISL_12289540, EPI_ISL_12665250, EPI_ISL_12665251, EPI_ISL_12665278			
see above	NATIONAL PUBLIC HEALTH LABORATORY	Institute for Medical Research, Infectious Disease Research Centre, National Institutes of Health, Ministry of Health Malaysia	Ahmad FA; Ahmad Fazilah NA; Anasir MI; Azizan MA; Kamel K; Mohamad Sukri MZ; Norhisham SN; Ramly N; Robert F; Rosli NR; Suppiah J; Thayan R
EPI_ISL_12899699, EPI_ISL_12899700, EPI_ISL_12899701	NATIONAL PUBLIC HEALTH LABORATORY (MKAK)	Institute for Medical Research, Infectious Disease Research Centre, National Institutes of Health, Ministry of Health Malaysia	Ahmad FA; Ahmad Fazilah NA; Anasir MI; Azizan MA; Kamel K; Mohamad Sukri MZ; Norhisham SN; Ramly N; Robert F; Rosli NR; Suppiah J; Thayan R
EPI_ISL_12252829, EPI_ISL_12252830	National Public Health Laboratory	National Public Health Laboratory	Kamal Hisham Bin Kamarul Zaman; Muhamad Syamim Bin Roslan; Noriah Binti Mohd Yusof; Nur Hazliza Binti Salleh; Rehan Shuhada Binti Abu Bakar; Selvanesan A/L Sengol; Yu Kie A/P Chem
EPI_ISL_12084318	National Public Health Laboratory (MKAK), Ministry of Health Malaysia	Institute for Medical Research, Infectious Disease Research Centre, National Institutes of Health, Ministry of Health Malaysia	Ahmad FA; Ahmad Fazilah NA; Anasir MI; Azizan MA; G. Adypatti NM; Jamaluddin MS; Kamel K; Mohamad Sukri MZ; Norhisham SN; Ramly N; Robert F; Rosli NR; Suppiah J; Thayan R
EPI_ISL_12628124, EPI_ISL_12628125	PKD MUAR	UKM Medical Molecular Biology Institute (UMBI)	Khairun Nur Abd Ghafar; Mira Farzana Mohamad Mokhtar; Muhiddin Ishak; Nor Azila Muhammad Azami; Nur Alyaa Afifah Md Shahri; Nurul Syakima Ab Mutalib; Rahman Jamal; Ryia Illani Mohd Yunos; Siti Nur Hasanah Mohd Yusuf; Zahirrah Begam Mohamed Rasheed
EPI_ISL_12321871	QUEEN ELIZABETH HOSPITAL	Institute for Medical Research, Infectious Disease Research Centre,	Ahmad FA; Ahmad Fazilah NA; Anasir MI; Azizan MA; Kamel K; Mohamad Sukri MZ; Norhisham SN; Ramly N; Robert F; Rosli NR; Suppiah J; Thayan R

		National Institutes of Health, Ministry of Health Malaysia	
EPI_ISL_12289482	SG BAKAP HOSPITAL	Institute for Medical Research, Infectious Disease Research Centre, National Institutes of Health, Ministry of Health Malaysia	Ahmad FA; Ahmad Fazilah NA; Anasir MI; Azizan MA; Kamel K; Mohamad Sukri MZ; Norhisham SN; Ramly N; Robert F; Rosli NR; Suppiah J; Thayan R
EPI_ISL_12289551	SUNGAI BULOH HOSPITAL	Institute for Medical Research, Infectious Disease Research Centre, National Institutes of Health, Ministry of Health Malaysia	Ahmad FA; Ahmad Fazilah NA; Anasir MI; Azizan MA; Kamel K; Mohamad Sukri MZ; Norhisham SN; Ramly N; Robert F; Rosli NR; Suppiah J; Thayan R
EPI_ISL_12510167	Sarawak General Hospital (Kuching)	Institute of Health and Community Medicine	Chan Chia Jui; Chua Hock Hin; David Perera; Ooi Mong How
EPI_ISL_12099687, EPI_ISL_12099688, EPI_ISL_12099689, EPI_ISL_12099690, EPI_ISL_12099691, EPI_ISL_12099692, EPI_ISL_12099693, EPI_ISL_12099694, EPI_ISL_12099697			
see above	Sibu Hospital, PCR lab (Sibu, Sarawak)	Institute of Health and Community Medicine	Chan Chia Jui; Chua Hock Hin; David Perera; Ooi Mong How; Tonnii Sia Loong Loong
EPI_ISL_13026085, EPI_ISL_13026086, EPI_ISL_13026087	Synapse Sdn. Bhd.	Universiti Putra Malaysia (UPM)	Choo Yee Yu; Hui Jen Soe; Hui Yee Chee; Nancy Woan Charn Liew; Narcisse Joseph; Nurulfiza Mat Isa; Rachna Kairon; Sie Yeng Wong; Syafinaz Amin-Nordin; Zunita Zakaria
EPI_ISL_12560257, EPI_ISL_12560258	UMMC	Department of Medical Microbiology, Faculty of Medicine, University of Malaya; University of Malaya Medical Centre	I-Ching SAM; Jolene Yin Ling FU; Omar Khalilur Rahman; Yee Von Lee; Yoke Fun Chan
EPI_ISL_12213455	hsajb	UKM Medical Molecular Biology Institute (UMBI)	Khairun Nur Abd Ghafar; Mira Farzana Mohamad Mokhtar; Muhiddin Ishak; Nor Azila Muhammad Azami; Nur Alyaa Afifah Md Shahri; Nurul Syakima Ab Mutalib; Rahman Jamal; Ryia Illani Mohd Yunos; Siti Nur Hasanah Mohd Yusuf; Zahirrah Begam Mohamed Rasheed

We gratefully acknowledge the following Authors from the Originating laboratories responsible for obtaining the specimens, as well as the Submitting laboratories where the genome data were generated and shared via GISAID, on which this research is based.

All Submitters of data may be contacted directly via www.gisaid.org

Authors are sorted alphabetically.

Acknowledgement EPI_SET Identifier: EPI_SET_20220603vy

Accession ID	Originating Laboratory	Submitting Laboratory	Authors
EPI_ISL_12322073	AMPANG HOSPITAL	Institute for Medical Research, Infectious Disease Research Centre, National Institutes of Health, Ministry of Health Malaysia	Ahmad FA; Ahmad Fazilah NA; Anasir MI; Azizan MA; Kamel K; Mohamad Sukri MZ; Norhisham SN; Ramly N; Robert F; Rosli NR; Suppiah J; Thayan R
EPI_ISL_12099570, EPI_ISL_12099626, EPI_ISL_12099627, EPI_ISL_12099628, EPI_ISL_12099629	Bintulu Medical Centre (Bintulu)	Institute of Health and Community Medicine	Chan Chia Jui; Chua Hock Hin; David Perera; Ooi Mong How; Tonnii Sia Loong Loong; Wong Jyn Shan
EPI_ISL_12510150, EPI_ISL_12510151, EPI_ISL_12510152, EPI_ISL_12510153, EPI_ISL_12510154, EPI_ISL_12510155, EPI_ISL_12510401, EPI_ISL_12510402, EPI_ISL_12510403, EPI_ISL_12510404, EPI_ISL_12510405, EPI_ISL_12510406, EPI_ISL_12510407, EPI_ISL_12510408, EPI_ISL_12510409, EPI_ISL_12510410, EPI_ISL_12510411			
see above	Borneo Medical Centre (Kuching)	Institute of Health and Community Medicine	Chan Chia Jui; David Perera; Ooi Mong How; Wong Jyn Shan
EPI_ISL_12534905	FORENSIC HOSPITAL SULTANAH BAHIYAH	Tropical Infectious Diseases Research & Education Centre (TIDREC), Universiti Malaya	AsmaAnati CheMatSeri; Che-Norainon Yaacob; Jia-Yi Tan; Jo-Ern Wong; Kim-Kee Tan; Mulya-Mustika-Sari Zulkifli; Noor Syahida Azizan; Nur-Hidayana Mahfodz; Sazaly AbuBakar; Siti-Sarah Nor'e; Wei-Wei Aw
EPI_ISL_13047112	HOSPITAL SULTAN ISMAIL PETRA, KUALA KRAI, KELANTAN	MOLECULAR RESEARCH LABORATORY	Abdul Haris bin Muhammad; Ahmad Sukari Bin Halim; Alexander Chong Shu Chien; Azian Harun; Chan Yean Yean; Chua Wei Chuan; Farahana binti Mohamed; Kirnpal Kaur Banga Singh; Lau Nyok Sean; Lee Lih Huey; Lim Shu Yong; Maizun binti Mohd Zain; Mohd Nadzri Abu Yazid; Mohd Zulkifli Salleh; Muhammad Azamuddeen bin Mohammad Nasir; Muhammad Fazli bin Khalid; Muhammad Nashrul Farhan Samsudin; Muhammad Zarul Hanifah Bin Md Zoqratt; Nik Zuraina binti Nik Mohd Noor; Noor Hafizan binti Mat Salleh; Nurfadhlina Musa; Qasim Ayub; Rosline Hassan; Sadequr Rahman; Syahida binti Omar; Wan Mohd Zahiruddin Wan Mohammad; Wardah Yusof; Zaini bin Hussin; Zakuan Zainy Deris
EPI_ISL_12213454	HSAJB	UKM Medical Molecular Biology Institute (UMBI)	Khairun Nur Abd Ghafar; Mira Farzana Mohamad Mokhtar; Muhiddin Ishak; Nor Azila Muhammad Azami; Nur Alyaa Afifah Md Shahri; Nurul Syakima Ab Mutalib; Rahman Jamal; Ryia Illani Mohd Yunos; Siti Nur Hasanah Mohd Yusuf; Zahirrah Begam Mohamed Rasheed
EPI_ISL_12055308, EPI_ISL_12055358, EPI_ISL_12055366	HTJS	Institut Biologi Molekul Perubatan UKM (UMBI)	Khairun Nur Abd Ghafar; Mira Farzana Mohamad Mokhtar; Muhiddin Ishak; Nor Azila Muhammad Azami; Nur Alyaa Afifah Md Shahri; Nurul Syakima Ab Mutalib; Rahman Jamal; Ryia Illani Mohd Yunos; Siti Nur Hasanah Mohd Yusuf; Zahirrah Begam Mohamed Rasheed
EPI_ISL_12704560, EPI_ISL_12704564, EPI_ISL_12935250	Hospital Raja Perempuan Zainab II, Kota Bharu	Molecular Research Laboratory	Abdul Haris bin Muhammad; Ahmad Sukari Bin Halim; Alexander Chong Shu Chien; Asraihan Che Abdul Malik; Azian Harun; Chan Yean Yean; Chua Wei Chuan; Farahana binti Mohamed; Kirnpal Kaur Banga Singh; Lau Nyok Sean; Lee Lih Huey; Lim Shu Yong; Maizun binti Mohd Zain; Mera Edora Binti Abdul Manap; Mohd Iman hafiz Bin Ibrahim; Mohd Nadzri Abu Yazid; Mohd Zulkifli Salleh; Muhamad Khairul Amirin Bin Zulkifli; Muhammad Azamuddeen bin Mohammad Nasir; Muhammad Fazli bin Khalid; Muhammad Nashrul Farhan Samsudin; Muhammad Zarul Hanifah Bin Md Zoqratt; Nik Zuraina binti Nik Mohd Noor; Noor Hafizan binti Mat Salleh; Nor Amizara Binti Azami; Nor Suhana Binti Mohd Satar; Nur Syuhada Binti Abdul Rahim; Nur-Leem Binti Murshid; Nurfadhlina Musa; Qasim Ayub; Rosline Hassan; Sadequr Rahman; Siti Nur Aisyah Binti Mohamad Sham; Syahida binti Omar; Wan Mohd Zahiruddin Wan Mohammad; Wardah Yusof; Zaini bin Hussin; Zakuan Zainy Deris
EPI_ISL_12771316	JKN PAHANG	iPROMISE, UiTM	Ariza Adnan; Fadzilah Mohd Nor; Lim Wai Feng; Mohd Asif Mohd Sukri; Mohd Nur Fakhruzzaman Noorizhab; Mohd Zaki Salleh; Sazzli Shahlan Kassim; Siti Farah Alwani Mohd Nawi; Siti Hamimah Sheikh Abdul Kadir; Teh Lay Kek; Wang Seok Mui
EPI_ISL_12101259, EPI_ISL_12101260, EPI_ISL_12101261, EPI_ISL_12101262, EPI_ISL_12101263, EPI_ISL_12101264, EPI_ISL_12101265, EPI_ISL_12101266, EPI_ISL_12101267, EPI_ISL_12101268, EPI_ISL_12101269, EPI_ISL_12101270, EPI_ISL_12101271, EPI_ISL_12101272, EPI_ISL_12101273, EPI_ISL_12101274, EPI_ISL_12101275, EPI_ISL_12101276, EPI_ISL_12101277, EPI_ISL_12101278, EPI_ISL_12101279, EPI_ISL_12101280, EPI_ISL_12101281, EPI_ISL_12101282, EPI_ISL_12101283, EPI_ISL_12101284, EPI_ISL_12101285, EPI_ISL_12101286, EPI_ISL_12101287, EPI_ISL_12101288, EPI_ISL_12101289, EPI_ISL_12101290, EPI_ISL_12101291, EPI_ISL_12101292, EPI_ISL_12101293, EPI_ISL_12101294, EPI_ISL_12101295, EPI_ISL_12101296, EPI_ISL_12101297, EPI_ISL_12101298, EPI_ISL_12101299, EPI_ISL_12101300, EPI_ISL_12101301, EPI_ISL_12101302, EPI_ISL_12101303, EPI_ISL_12101304, EPI_ISL_12101305, EPI_ISL_12101306, EPI_ISL_12101307, EPI_ISL_12101308, EPI_ISL_12101309, EPI_ISL_12101310, EPI_ISL_12101311, EPI_ISL_12101312, EPI_ISL_12101313, EPI_ISL_12101314, EPI_ISL_12101315, EPI_ISL_12101316, EPI_ISL_12101317, EPI_ISL_12101318, EPI_ISL_12101319, EPI_ISL_12101320, EPI_ISL_12101321, EPI_ISL_12101322, EPI_ISL_12101323, EPI_ISL_12101324, EPI_ISL_12101325, EPI_ISL_12101326, EPI_ISL_12101327, EPI_ISL_12101328, EPI_ISL_12101329, EPI_ISL_12101330, EPI_ISL_12101331, EPI_ISL_12101332, EPI_ISL_12101333, EPI_ISL_12101334, EPI_ISL_12101335, EPI_ISL_12101336, EPI_ISL_12101337, EPI_ISL_12101338, EPI_ISL_12101339, EPI_ISL_12101340, EPI_ISL_12101341, EPI_ISL_12101342, EPI_ISL_12101343, EPI_ISL_12101344, EPI_ISL_12101345, EPI_ISL_12101346, EPI_ISL_12101347, EPI_ISL_12101348, EPI_ISL_12101349, EPI_ISL_12101350, EPI_ISL_12101351, EPI_ISL_12101352, EPI_ISL_12101353, EPI_ISL_12101354, EPI_ISL_12101355, EPI_ISL_12101356, EPI_ISL_12101357, EPI_ISL_12101358, EPI_ISL_12101359, EPI_ISL_12101360, EPI_ISL_12101361, EPI_ISL_12101362, EPI_ISL_12101363, EPI_ISL_12101364, EPI_ISL_12101365, EPI_ISL_12101366, EPI_ISL_12101367, EPI_ISL_12101368, EPI_ISL_12101369, EPI_ISL_12101370, EPI_ISL_12101371, EPI_ISL_12101372, EPI_ISL_12101373, EPI_ISL_12101374, EPI_ISL_12101375, EPI_ISL_12101376, EPI_ISL_12101377, EPI_ISL_12101378, EPI_ISL_12101379, EPI_ISL_12101380, EPI_ISL_12101381, EPI_ISL_12101382, EPI_ISL_12101383, EPI_ISL_12101384, EPI_ISL_12101385, EPI_ISL_12101386, EPI_ISL_12101387, EPI_ISL_12101388, EPI_ISL_12134339, EPI_ISL_12134340, EPI_ISL_12134341, EPI_ISL_12134342, EPI_ISL_12134343, EPI_ISL_12138816, EPI_ISL_12138817, EPI_ISL_12138818, EPI_ISL_12138819, EPI_ISL_12138820, EPI_ISL_12138821, EPI_ISL_12138822, EPI_ISL_12138823, EPI_ISL_12138824, EPI_ISL_12138825, EPI_ISL_12138826, EPI_ISL_12138827, EPI_ISL_12138828, EPI_ISL_12138829, EPI_ISL_12138830, EPI_ISL_12138832, EPI_ISL_12138833, EPI_ISL_12138834, EPI_ISL_12138835, EPI_ISL_12138836, EPI_ISL_12138837, EPI_ISL_12138838, EPI_ISL_12138839, EPI_ISL_12138840, EPI_ISL_12138841, EPI_ISL_12138842, EPI_ISL_12138843, EPI_ISL_12138844, EPI_ISL_12138845, EPI_ISL_12138846, EPI_ISL_12138847, EPI_ISL_12138848, EPI_ISL_12138849, EPI_ISL_12138850, EPI_ISL_12138851, EPI_ISL_12138852, EPI_ISL_12138853, EPI_ISL_12141450, EPI_ISL_12141451, EPI_ISL_12148245, EPI_ISL_12148246, EPI_ISL_12148247, EPI_ISL_12148248, EPI_ISL_12148249, EPI_ISL_12148250, EPI_ISL_12148251, EPI_ISL_12148252, EPI_ISL_12148253, EPI_ISL_12148254, EPI_ISL_12148255, EPI_ISL_12148256, EPI_ISL_12148257, EPI_ISL_12148258, EPI_ISL_12148259, EPI_ISL_12148260, EPI_ISL_12148261, EPI_ISL_12148262, EPI_ISL_12148263, EPI_ISL_12148264, EPI_ISL_12148265, EPI_ISL_12148266, EPI_ISL_12148267, EPI_ISL_12148268, EPI_ISL_12148269, EPI_ISL_12148270, EPI_ISL_12148271, EPI_ISL_12148272, EPI_ISL_12148273, EPI_ISL_12148274, EPI_ISL_12148275, EPI_ISL_12148276, EPI_ISL_12148277, EPI_ISL_12148278, EPI_ISL_12148279, EPI_ISL_12148280, EPI_ISL_12148281, EPI_ISL_12148282, EPI_ISL_12148283, EPI_ISL_12148284, EPI_ISL_12148285, EPI_ISL_12148286, EPI_ISL_12148287, EPI_ISL_12148288, EPI_ISL_12148289, EPI_ISL_12148290, EPI_ISL_12148291, EPI_ISL_12148292, EPI_ISL_12148293, EPI_ISL_12148294, EPI_ISL_12148295, EPI_ISL_12148296, EPI_ISL_12148297, EPI_ISL_12148298, EPI_ISL_12148299, EPI_ISL_12148300, EPI_ISL_12148301, EPI_ISL_12148302, EPI_ISL_12148303, EPI_ISL_12148304, EPI_ISL_12148305, EPI_ISL_12148306, EPI_ISL_12148307, EPI_ISL_12148308, EPI_ISL_12148309, EPI_ISL_12148310, EPI_ISL_12148311, EPI_ISL_12148312, EPI_ISL_12148313, EPI_ISL_12148314, EPI_ISL_12148315, EPI_ISL_12148316, EPI_ISL_12148317, EPI_ISL_12148318, EPI_ISL_12148319, EPI_ISL_12148320, EPI_ISL_12148321, EPI_ISL_12148322, EPI_ISL_12148323, EPI_ISL_12148324, EPI_ISL_12148325, EPI_ISL_12148326, EPI_ISL_12148327, EPI_ISL_12148328, EPI_ISL_12148329, EPI_ISL_12148330, EPI_ISL_12148331, EPI_ISL_12148332, EPI_ISL_12148333, EPI_ISL_12148334, EPI_ISL_12148335, EPI_ISL_12148336, EPI_ISL_12148337, EPI_ISL_12148338, EPI_ISL_12148339, EPI_ISL_12148340, EPI_ISL_12148341, EPI_ISL_12148342, EPI_ISL_12148343, EPI_ISL_12148344, EPI_ISL_12148345, EPI_ISL_12148346, EPI_ISL_12148347, EPI_ISL_12148348, EPI_ISL_12148349, EPI_ISL_12148350, EPI_ISL_12148351, EPI_ISL_12148352, EPI_ISL_12148353, EPI_ISL_12148354, EPI_ISL_12148355, EPI_ISL_12148356, EPI_ISL_12148357, EPI_ISL_12148358, EPI_ISL_12148359, EPI_ISL_12148360, EPI_ISL_12148361, EPI_ISL_12148362, EPI_ISL_12148363, EPI_ISL_12148364, EPI_ISL_12148365, EPI_ISL_12148366, EPI_ISL_12148367, EPI_ISL_12148368, EPI_ISL_12148369, EPI_ISL_12148370, EPI_ISL_12148371, EPI_ISL_12148372, EPI_ISL_12148373, EPI_ISL_12148374, EPI_ISL_12148375, EPI_ISL_12148376, EPI_ISL_12148377, EPI_ISL_12148378, EPI_ISL_12148379, EPI_ISL_12148380, EPI_ISL_12148381, EPI_ISL_12148382, EPI_ISL_12148383, EPI_ISL_12148384, EPI_ISL_12148385, EPI_ISL_12150067, EPI_ISL_12150068, EPI_ISL_12150069, EPI_ISL_12150070, EPI_ISL_12150071, EPI_ISL_12150072, EPI_ISL_12150073, EPI_ISL_12150074, EPI_ISL_12150620, EPI_ISL_12150621, EPI_ISL_12175735, EPI_ISL_12175736, EPI_ISL_12175737, EPI_ISL_12175738, EPI_ISL_12175739, EPI_ISL_12175740, EPI_ISL_12175741, EPI_ISL_12299559, EPI_ISL_12299560			
see above	Kuala Lumpur International Airport	Institute for Medical Research, Infectious Disease Research Centre, National Institutes of Health, Ministry of Health Malaysia	Anasir MI; G.Adypatti NM; Jamaluddin MS; Kalyanasundram J; Kamel K; MatRahim N; Nawi MH; Suib FA; Suppiah J; Thayan R
EPI_ISL_12213463	MAKMAL KESIHATAN AWAM JOHOR BAHRU	UKM Medical Molecular Biology Institute (UMBI)	Khairun Nur Abd Ghafar; Mira Farzana Mohamad Mokhtar; Muhiddin Ishak; Nor Azila Muhammad Azami; Nur Alyaa Afifah Md Shahri; Nurul Syakima Ab Mutalib; Rahman Jamal; Ryia Illani Mohd Yunos; Siti Nur Hasanah Mohd Yusuf; Zahirrah Begam Mohamed Rasheed
EPI_ISL_12321826, EPI_ISL_12321827, EPI_ISL_12321828, EPI_ISL_12321829, EPI_ISL_12321830, EPI_ISL_12321831, EPI_ISL_12321832, EPI_ISL_12321833, EPI_ISL_12321834, EPI_ISL_12321835, EPI_ISL_12321836, EPI_ISL_12321837, EPI_ISL_12321838, EPI_ISL_12321839			
see above	NATIONAL PUBLIC HEALTH LABORATORY	Institute for Medical Research, Infectious Disease Research Centre, National Institutes of Health, Ministry of Health Malaysia	Ahmad FA; Ahmad Fazilah NA; Anasir MI; Azizan MA; Kamel K; Mohamad Sukri MZ; Norhisham SN; Ramly N; Robert F; Rosli NR; Suppiah J; Thayan R
EPI_ISL_12899702	NATIONAL PUBLIC HEALTH LABORATORY (MKAK)	Institute for Medical Research, Infectious Disease Research Centre, National Institutes of Health, Ministry of Health Malaysia	Ahmad FA; Ahmad Fazilah NA; Anasir MI; Azizan MA; Kamel K; Mohamad Sukri MZ; Norhisham SN; Ramly N; Robert F; Rosli NR; Suppiah J; Thayan R
EPI_ISL_12055347	PKD JOHOR BAHRU	Institut Biologi Molekul Perubatan UKM (UMBI)	Khairun Nur Abd Ghafar; Mira Farzana Mohamad Mokhtar; Muhiddin Ishak; Nor Azila Muhammad Azami; Nur Alyaa Afifah Md Shahri; Nurul Syakima Ab Mutalib; Rahman Jamal; Ryia Illani Mohd Yunos; Siti Nur Hasanah Mohd Yusuf; Zahirrah Begam Mohamed Rasheed
EPI_ISL_12099696, EPI_ISL_12099698, EPI_ISL_12099702	Sibu Hospital, PCR lab (Sibu, Sarawak)	Institute of Health and Community Medicine	Chan Chia Jui; Chua Hock Hin; David Perera; Ooi Mong How; Tonnii Sia Loong Loong
